# A new era for the design of TRPV1 antagonists and agonists with the use of structural information and molecular docking of capsaicin-like compounds

**DOI:** 10.1080/14756366.2022.2110089

**Published:** 2022-08-16

**Authors:** Julio Caballero

**Affiliations:** Centro de Bioinformática, Simulación y Modelado (CBSM), Facultad de Ingeniería, Universidad de Talca, Talca, Chile

**Keywords:** TRPV1 antagonists, TRPV1 agonists, capsaicin receptor, molecular docking

## Abstract

The design of TRPV1 antagonists and agonists has reached a new era since TRPV1 structures at near-atomic resolution are available. Today, the ligand-binding forms of several classical antagonists and agonists are known; therefore, the specific role of key TRPV1’s residues in binding of ligands can be elucidated. It is possible to place the well-defined pharmacophore of TRPV1 ligands, conformed by head, neck, and tail groups, in the right pocket regions of TRPV1. It will allow a more thorough use of molecular modelling methods to conduct more effective rational drug design protocols. In this work, important points about the interactions between TRPV1 and capsaicin-like compounds are spelled out, based on the known pharmacophore of the ligands and the already available TRPV1 structures. These points must be addressed to generate reliable poses of novel candidates and should be considered during the design of novel TRPV1 antagonists and agonists.

## Introduction

Transient receptor potential vanilloid 1 (TRPV1) belongs to the vanilloid family of TRP (transient receptor potential) channels, relatives of voltage-gated potassium channels, and expressed in the central nervous system and the peripheral blood vessels^1^^,^^2^. It is activated by different mechanisms involving voltage, heat, and ligands, resulting in an increase in intracellular Ca^2+^ concentration and blood vessel constrictions^3^. TRPV1 has been identified as a promising therapeutic target to reduce pain perception^4^^,^^5^ and itch sensation under pathological conditions^6^. It is also involved in the regulation of several physiological and pathological processes; therefore, it has been also considered in the development of therapies against schizophrenia^7^, epilepsy^8^, diabetes^9^, ischaemia^10^, chronic cough^11^, etc.

Capsaicin, the primary pungent ingredient of chilli peppers, is the most characteristic exogenous chemical activator of TRPV1. Other exogenous agonists were identified such as olvanil^12^ and resiniferatoxin (RTX)^13^, and endogenous agonists have been also identified, such as anandamide^14^, N-oleoyldopamine^15^, etc. In capsaicin and natural capsaicin‐like compounds, vanillyl was identified as an essential group to modulate TRPV1. Subsequently, a wide number of TRVP1 ligands have been synthesised that have other groups instead of vanillyl, such as pyrimidine^16^, 2-benzothiazolyl acetamide^17^, catechol^18^^,^^19^, chalcone^20^, 2–(3-fluoro-4-methylsulfonylaminophenyl)propanamide^21^, etc.

TRPV1 ligands can be classified to agonists and antagonists^22^. Capsaicin is the classic TRPV1 agonist, while RTX, a natural product found in the cactus-like plants *Euphorbia resinifera* and *Euphorbia poissonii*, is an ultrapotent agonist^23^. The therapeutic role of TRPV1 agonists is based on the desensitisation of pain-conducting nerve fibres, which contributes to analgesic effects^24^. In general, the majority of the potent TRPV1 agonists reported until today contain the vanillyl group; however, several examples show that this group can be replaced by similar chemical groups^25^^,^^26^. For instance, the nonpungent TRPV1 agonist MDR-652 was recently discovered, with a 3-fluoro-4-(hydroxymethyl)phenyl group instead the vanillyl group^27^.

TRPV1 antagonists show analgesic and anti-inflammatory actions in neuropathic pain^28^^,^^29^. Capsazepine was the first capsaicin-like TRPV1 antagonist, discovered in 1991^30^. It contains a 2,3,4,5-Tetrahydro-1*H*-2-benzazepine-7,8-diol instead the vanillyl group. Later on, different series of capsaicin-like TRPV1 antagonists have been discovered, where the vanillyl group was replaced by 4–(3-chloropyridin-2-yl)tetrahydropyrazine^31^, 1,4-Benzodioxane^16^, 3-Amino-1H-quinoxalin-2-one^17^^,^^32^, 2-Aminobenzothiazole^17^^,^^33^, sulfonylaminobenzyl^21^^,^^34^, 3-Hydroxy-3,4-dihydroquinolin-2(1*H*)-one^35^, isoquinoline^35^, etc.

### The pharmacophores of capsaicin-like TRPV1 ligands

**Figure 1.**
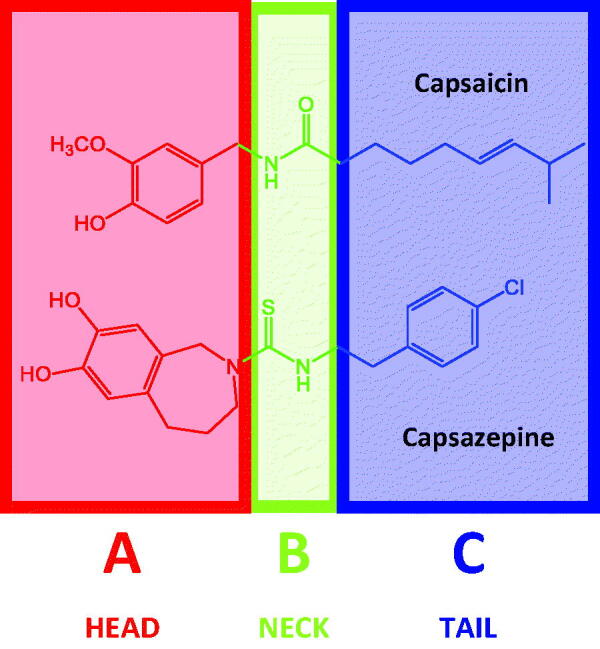
Structures of capsaicin (TRPV1 agonist) and capsazepine (TRPV1 antagonist) and pharmacophoric features.

**Figure 2.**
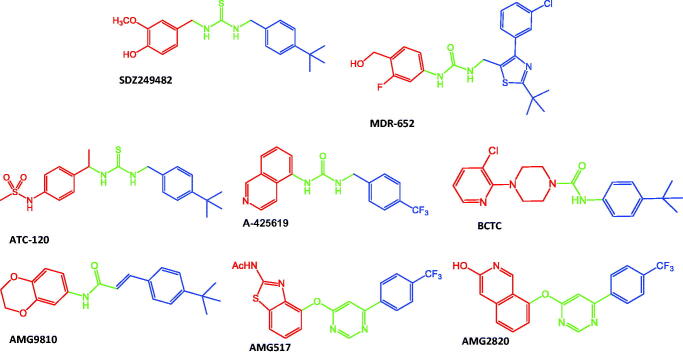
Chemical structures of TRPV1 agonists (SDZ249482, MDR-652) and antagonists (ATC-120, A-425619, BCTC, AMG9810, AMG517, AMG2820) to represent the diversity of chemical groups at the A- (red), B- (green), and C- (blue) regions of the pharmacophore.

Capsaicin-like TRPV1 ligands have a well-defined pharmacophore, composed by a consecutive disposition of three chemical features: head, neck, and tail groups (Figure 1). Capsaicin-like TRPV1 ligands bind to a large pocket formed by transmembrane domains, where they adopt a “tail-up, head-down” configuration, placing the head close to the S4–S5 linker^36^. Head, neck, and tail, also designed as A-, B-, and C-regions, can be easily identified in capsaicin, where the vanilloid (4-hydroxy-3-methoxyphenyl) group is in the A-region, amide is in the B-region, and the lipophilic chain of the 8-methyl-6-nonenoic acid is in the C-region. It is possible to identify these pharmacophoric features in TRPV1 agonists and antagonists. For instance, the agonist MDR-652 contains the 3-fluoro-4-(hydroxymethyl)phenyl in the A-region, urea in the B-region, and 2-(tert-butyl)-4–(3-chlorophenyl)thiazole in the C-region. On the other hand, the antagonist capsazepine contains the 2,3,4,5-Tetrahydro-1*H*-2-benzazepine-7,8-diol in the A-region, thiourea in the B-region, and 4-chlorophenyl)ethyl in the C-region. Figure 2 shows examples of TRPV1 agonists and antagonists, where the pharmacophoric features are represented.

### Structures of TRPV1-ligand complexes

In 2008, Fernández-Ballester and Ferrer-Montiel^37^ modelled a full-length human TRPV1 (the cytosolic N-terminal, C-terminal, and membrane-spanning region) in the closed and open states by using available structural information in the ion channel field. These models were the first attempt to access the structural atomistic model of the TRPV1 channel and they were in agreement with a 19 Å structure of rat TRPV1 determined just that year by single particle electron cryomicroscopy^38^. Despite these models tried to locate the molecular determinants of function in TRPV1, they were not accurate to investigate the interactions between TRPV1 and its ligands.

Previous to the determination of TRPV1 structures, mutagenesis studies allowed to identify residues that specify sensitivity to small vanilloid ligands, such as Y511, S512, and T550^39^. In 2011, Lee et al. performed mutational analysis to evaluate the contribution of TRPV1 residues to ligand binding of capsaicin and RTX, and used this information to construct a tetrameric homology model of rat TRPV1^40^. Then, they performed flexible docking analysis using this model and they found a good agreement with their mutation data. Over the next years, the same group used this model to propose the docked poses of different sulfonylaminobenzyl derivatives^21^^,^^34^^,^^41^^,^^42^. This model used properly the structural information what was known at the time and provided clarification on various issues related to the interactions between TRPV1 and capsaicin-like ligands; however, it had some shortcomings. For instance, this model placed the residue K571 at the vanilloid pocket of the binding site, making a role of a hydrogen bond (HB) donor when ligands are bound^21^^,^^40^, and did not identify the role of R557 and E570 in ligand binding. Previously, site-directed mutagenesis studies had indicated that these residues were involved in the transduction of the capsaicin-binding signal to the opening of the channel pore, with no specific information about their role in the binding of capsaicin^43^.

Only after 2013, when Cao et al.^44^^,^^45^ determined the structures of rat TRPV1 by using single-particle cryo-microscopy, it was possible to prepare more reliable structural models of the complexes between TRPV1 and its ligands. The first TRPV1 structures, available in Protein Data Bank (PDB) with the codes 3J5P, 3J5Q, and 3J5R, allow to understand unique aspects of TRPV1 channel functions, including the interactions with capsaicin-like modulators. These authors reported structures that contain capsaicin and RTX in the binding site; however, they had a low resolution (3.8 and 4.2 Å), and they did not allow to clearly observe the chemical groups of the ligands and the role of several residues in the vanilloid binding site.

**Figure 3.**
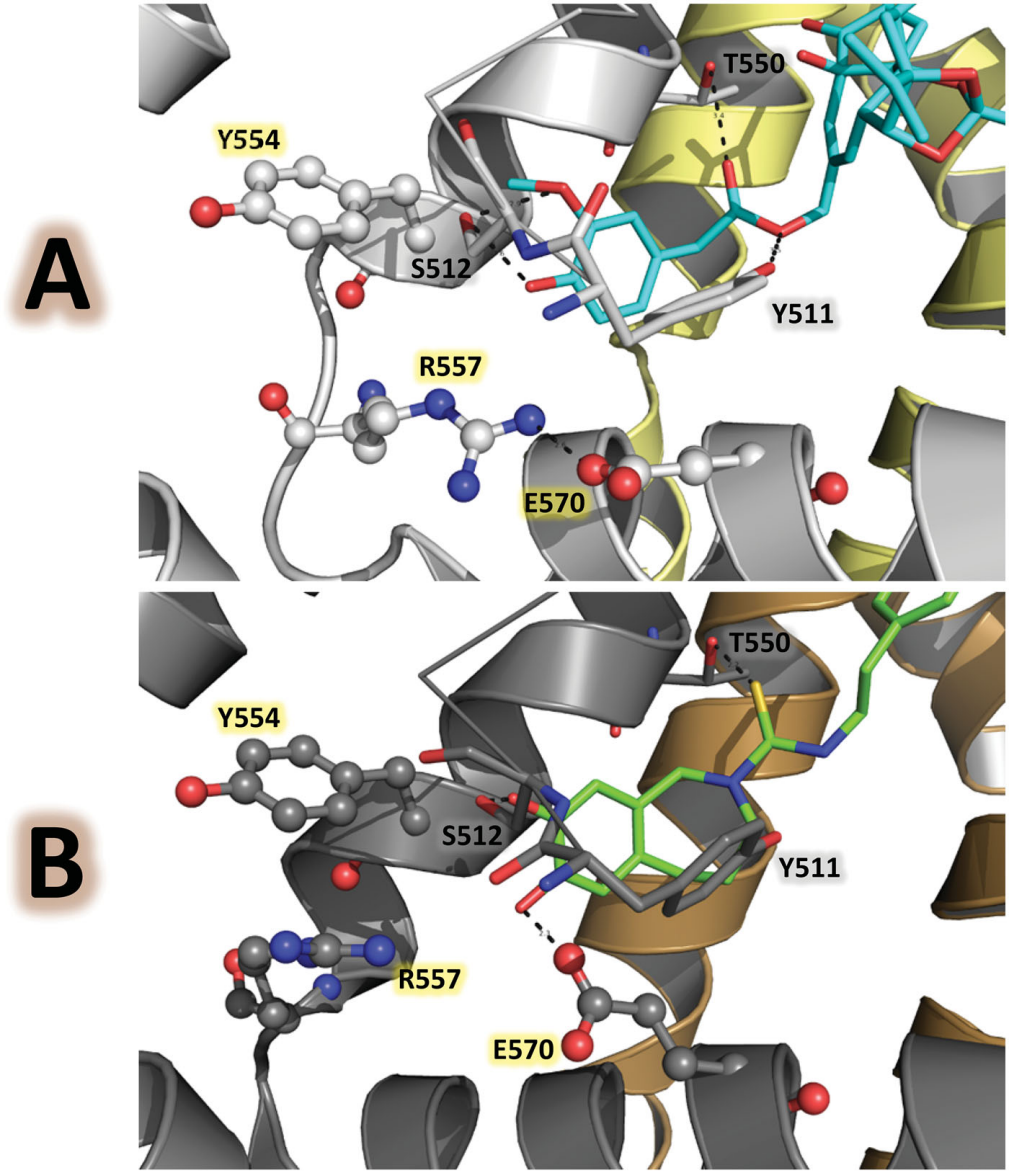
Ligand-protein polar interactions in the TRPV1 vanilloid pocket mediated by agonists and antagonists in PDB structures with codes 5IRX and 5IS0. The residues Y511, S512, T550, Y554, R557, and E570 are in gray stick representation, RTX is in cyan stick representation, and capsazepine is in green stick representation. (A) the vanilloid pocket when the agonist RTX is bound shows the ionic lock between R557 and E570. (B) the vanilloid pocket when the antagonist capsazepine is bound shows the cation-π interaction between R557 and Y554.

A few years later, in 2016, Gao et al. reported rat TRPV1 structures in lipid nanodiscs with single-particle electron cryo-microscopy at near-atomic resolution^46^. They reported the apo state (PDB code 5IRZ) and liganded states forming complexes with RTX and capsazepine (5IRX and 5IS0, respectively) in the native bilayer environment. These structures clearly show the conformational changes induced by antagonist and agonist effects in TRPV1. Furthermore, they revealed the roles of the residues Y511, S512, T550, R557, and E570 proximal to the vanilloid pocket, and revealed details of the conformational changes of these residues when the TRPV1 channel is in the closed and open states (Figure 3). Specifically, these structures show that a cation-π interaction is formed between the residues R557 and Y554 (S4 helix) in the apo and antagonist-bound states (nonconductive form). But this interaction is disrupted in the agonist-bound state and R557 adopted another conformation forming an ionic lock with the residue E570 (S4-S5 linker), leading to the opening of the lower gate of TRPV1 (conductive form). This detailed structural information is extremely useful for the rational design of TRPV1 agonists and antagonists.

### Structural details of the complexes between TRPV1 and capsaicin-like compounds

Over the last decades, the design of capsaicin-like TRPV1 ligands has been conducted by considering the pharmacophoric features head, neck, and tail, derived from the analysis of capsaicin (ligand-based design). However, after the determination of high-resolution structures of TRPV1 and its complexes, it is possible to describe the specific interactions between the pharmacophoric features of the ligands and the residues at the capsaicin binding site. The retrospective analysis of the specific chemical interactions between TRPV1 and its diverse ligands will be an essential step to go forward in a deeper knowledge of the structural aspects of this protein-ligand system. This analysis can be developed by constructing structural models, and by using computational molecular modelling and simulation methods that predict ligand-protein interactions and consider ligand and protein flexibilities. Learning from these models will be a necessary stage to assist the rational drug design of novel TRPV1 agonists and antagonists.

**Figure 4.**
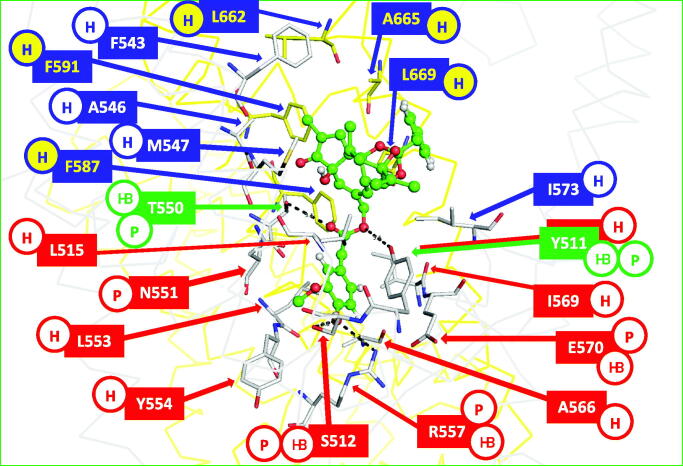
Interactions between ligands and the residues at the capsaicin binding site of rat TRPV1. As an example, the agonist RTX in the structure with PDB code 5IRX is represented as green balls and sticks. The residues of chains A and B of TRPV1 are represented as gray and yellow sticks, respectively. The residues involved in the interactions with the head, neck, and tail groups of the ligand are labelled with red, green, and blue boxes, respectively. The contributions of the residues to interactions with ligands are indicated in circles: hydrophobic H, polar P, hydrogen bond donor or acceptors HB.

Specific structural details of how head, neck, and tail groups interact with the residues at the TRPV1 binding site can be described in detail. The binding site contains residues from two sequence units (chain A and B here). The head is placed inside the vanilloid pocket where the residues Y511, S512, R557, and E570 expose polar groups and can form HBs with ligands (Figure 4). The conformational changes between the position of these residues, when TRPV1 is in closed and open states (Figure 3), have effects in the pharmacophore of ligands with agonistic and antagonistic activities. For instance, in a recent report, a docking protocol by using the antagonistic-bound state of TRPV1 as receptor explains why a set of 5,5-diarylpentadienamides that contain isoquinoline or 3-hydroxy-3,4-dihydroquinolin-2(1*H*)-one as the head groups are antagonists, and not agonists^47^. It was found that the head groups (both isoquinoline and 3-hydroxy-3,4-dihydroquinolin-2(1*H*)-one) have the exact geometry to establish HBs with the residues Y511, S512, and R557 in the closed conformation of TRPV1 and their geometry is not optimal to establish these interactions with these residues in the open TRPV1 conformation.

The neck is formed by polar groups that could form HBs with the side-chain hydroxyl groups of Y511 and T550 (Figure 4). These hydroxyl groups, separated by around 8 Å, define a polar region that connects the vanilloid pocket with a broad hydrophobic pocket. Since Y511 and T550 could establish specific HB interactions, they have important roles in the recognition of TRPV1 modulators.

Finally, the tail is formed by hydrophobic groups that are placed in a hydrophobic pocket formed by residues from the chains A and B of the TRPV1 structure. These residues are represented in Figure 4; it is important to note that rat and human TRPV1 capsaicin binding sites have only one different residue which is located in this pocket: M547 in rat TRPV1 is replaced by L547 in human TRPV1. Residues in this pocket do not form specific interactions, but modulate the potency of compounds from a same congeneric series. For instance, Saku et al.^35^ reported a series of N-(Isoquinolin-5-yl)-2,4-pentadinenamides with IC_50_ values between 0.072 and 950 nM (determined using the human TRPV1 receptor). These compounds have the same head and neck groups, and are differentiated only by their tail groups. Therefore, an accurate description of the interactions with the residues at this hydrophobic pocket should be needed to understand the differential activities of this congeneric series.

## Methods

### Docking of TRPV1 agonists and antagonists guided by available structural information

As discussed above, the recently launched rat TRPV1 structures forming complexes with capsaicin-like antagonists and agonists are reliable sources for generating structural models of TRPV1-ligand complexes by using protein-ligand docking methods. Previous identification of ligand characteristics (head, neck, and tail groups) are also important before applying these methods. Therefore, pharmacophore and geometric filters could be defined during docking calculations to obtain reliable models.

In this section, docking calculations of three series of compounds, named A, B, and C (Tables 1–3)^25^^,^^34^^,^^48^, were carried out. A flexible docking protocol was used to give examples of how molecular docking calculations can be performed to generate reliable models of TRPV1-ligand complexes. This protocol was achieved by using the Induced Fit Docking (IFD) method^49^ from Glide (Schrödinger LLC, New York, NY, USA, 2017).

**Table 1.** Structures of [4-(methylsulfonylamino)benzyl] thioureas (series A) and their rat TRPV1 binding affinities (racemic mixtures) as log(10^3^/Ki) (nM) (reference^34^).

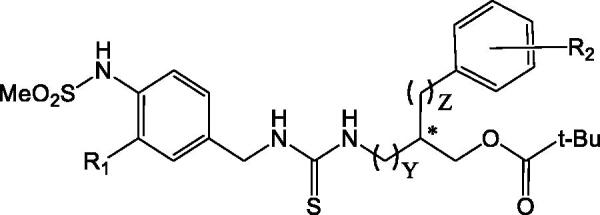					
	Y	Z	R_1_	R_2_	Log(10^3^/Ki)
**A_1**	1	1	H	3,4-Me_2_	1.533
**A_2**	1	1	H	4-t-Bu	1.194
**A_3**	1	1	F	3,4-Me_2_	1.268
**A_4**	1	1	F	4-t-Bu	1.646
**A_5**	0	1	H	3,4-Me_2_	0.237
**A_6**	0	1	H	4-t-Bu	0.381
**A_10**	0	1	F	3,4-Me_2_	0.633
**A_11**	0	1	F	4-t-Bu	0.939
**A_18**	0	2	H	3,4-Me_2_	0.409
**A_19**	0	2	H	4-t-Bu	0.437
**A_20**	0	2	F	3,4-Me_2_	0.921
**A_21**	0	2	F	4-t-Bu	0.721
**A_26**	0	0	H	4-t-Bu	0.706
**A_27**	0	0	F	4-t-Bu	0.613
**A_32**	1	0	H	4-t-Bu	0.845
**A_33**	1	0	F	4-t-Bu	1.161

**Table 2.** Structures of specific stereoisomers of [4-(methylsulfonylamino)benzyl] thioureas (series B) and their rat TRPV1 binding affinities as log(10^3^/Ki) (nM) values (reference^48^).

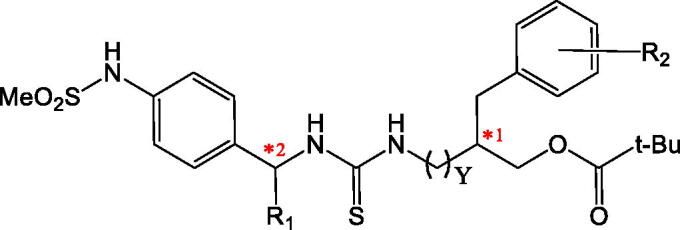						
	R1	R2	Y	Chirality (C1)	Chirality (C2)	Log(10^3^/Ki)
**B_5aR**	H	H	0	R	–	0.599
**B_5aS**	H	H	0	S	–	0.627
**B_19**	Me	H	1	R	R	1.644
**B_20**	Me	H	1	S	R	1.712
**B_21**	Me	H	1	R	S	–0.057
**B_22**	Me	H	1	S	S	0.149
**B_25**	Me	3,4-Me_2_	1	R	R	1.818
**B_26**	Me	3,4-Me_2_	1	S	R	2.674
**B_29**	Me	4-t-Bu	1	R	R	1.747
**B_30**	Me	4-t-Bu	1	S	R	2.171
**B_33**	Me	4-t-Bu	0	R	R	0.377
**B_34**	Me	4-t-Bu	0	S	R	0.565

**Table 3.** Structures of (3-methoxybenzyl)thioureas (series C) and their rat TRPV1 agonist activities as log(1/EC_50_) (μM) values (reference^25^).

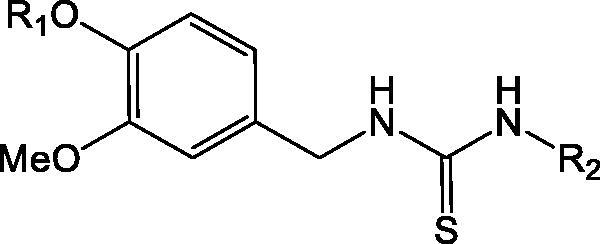			
	R_1_	R_2_	log(1/EC_50_)
**C_1a**	H	4-Cl-benzyl	1.125
**C_1b**	H	4-Cl-phenethyl	1.252
**C_1c**	H	(*E*)- 4-chlorostyryl	0.387
**C_1d**	H	(*Z*)- 4-chlorostyryl	1.387
**C_1e**	H	4-F-phenethyl	0.886
**C_1f**	H	2,4-dichlorophenethyl	0.886
**C_1g**	H	phenethyl	0.319
**C_1h**	H	3-(4-chlorophenyl)propyl	0.319
**C_1i**	H	4-(*tert*-butyl)benzyl	0.292
**C_2a**	‒(CH_2_)_2_-NH_2_	4-Cl-phenethyl	0.022
**C_2b**	‒(CH_2_)_2_-NH_2_	phenethyl	−0.435
**C_2c**	‒(CH_2_)_2_-NH_2_	4-F-phenethyl	−0.362
**C_2d**	‒(CH_2_)_2_-NH_2_	2,4-dichlorophenethyl	0.495
**C_2e**	‒(CH_2_)_2_-NH_2_	4-I-benzyl	−0.127
**C_2g**	‒(CH_2_)_2_-NH_2_	4-(*tert*-butyl)phenyl	−0.220
**C_2h**	‒(CH_2_)_2_-NH_2_	4-(*tert*-butyl)benzyl	0.770
**C_2i**	‒(CH_2_)_2_-NH_2_	3,5-di-*tert*-butylbenzyl	0.658
**C_2j**	‒(CH_2_)_2_-NH_2_	4-(*tert*-butyl)phenethyl	0.149
**C_3**	‒(CH_2_)_3_-NH_2_	4-Cl-phenethyl	−0.739
**C_4**	‒(CH_2_)_2_-NH-CH_3_	4-Cl-phenethyl	−0.847
**C_5**	‒(CH_2_)_2_-N(CH_3_)_2_	4-Cl-phenethyl	−0.547
**C_8**	‒(CH_2_)_2_-NH-COCH_3_	4-Cl-phenethyl	−0.710

The structures of the rat TRPV1 complexes with the agonist resiniferatoxin (5IRX) and with the antagonist capsazepine (5IS0) were imported from the PDB. The coordinates of the protein were extracted from both PDB files and they were modified with the Protein Preparation Wizard using default settings (Protein Preparation Wizard, Schrödinger LLC, New York, NY, USA, 2017). The structures of the ligands were sketched in Maestro’s molecular editor (Maestro 11.1.012, Schrödinger LLC, New York, NY, USA, 2017). Then, their protonation states were defined at physiological pH by using Maestro’s module LigPrep.

A grid box of 30 × 30 × 30 Å^3^ was centred on the centroid of selected residues represented in the Figure 4 (Y511, S512, F543, M547, T550, R557, and I573 from chain A, and L662, A665, and L669 from chain B), to cover the whole binding sites. During IFD (using the OPLS3 force field^50^) the residues within 5 Å of the ligand poses were subjected to refinement and side chain optimisation. Flexibility was induced during a Glide docking procedure with a scaling factor of 0.50 for the van der Waals radii of the protein and ligand. Twenty poses were obtained for each inhibitor by considering IFD scores. These docked top poses were analysed to verify that they adopt a “tail-up, head-down” configuration.

## Results

### Models of [4-(methylsulfonylamino)benzyl]thiourea analogues (series a and B) as TRPV1 antagonists

The TRPV1 antagonists from series A (Table 1) are chiral isomers, but their binding affinities were reported for the racemic mixtures^34^. On the other hand, compounds from series B (Table 2) are also chiral isomers, and their binding affinities were reported for each stereo-isomer^48^. Compounds from both series A and B contain a 4-(methylsulfonylamino)benzyl as a head group. Their neck is the thiourea, and their tail is a series of bulky groups that contains a unique chiral centre with 3-pivaloyloxymethyl and phenyl (or phenylalkyl) substituents. Compounds from series B contain two chiral centres: the first one is at the methylene group of the benzyl fragment of 4-(methylsulfonylamino)benzyl, and the second one is at the bulky tail group.

The 4-(methylsulfonylamino)benzyl group is longer than the 2,3,4,5-Tetrahydro-1*H*-2-benzazepine-7,8-diol group of capsazepine. When docking calculations of compounds from series A and B inside the rigid protein of the PDB structure with code 5IS0 (containing the antagonist capsazepine) were carried out, no solutions were found adopting “tail-up, head-down” configurations. Therefore, a flexible docking protocol was performed, hoping to find adequate solutions.

**Figure 5.**
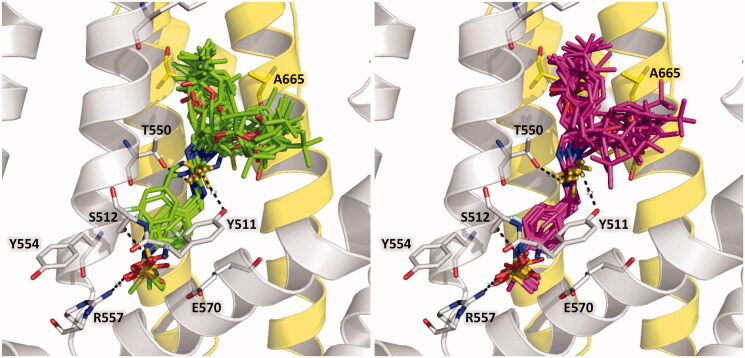
Structural models of the thiourea analogues from series A as TRPV1 antagonists. (Left) R-enantiomer docking poses (represented as green sticks). (Right) S-enantiomer docking poses (represented as purple sticks). The chains A and B of TRPV1 are in gray and yellow cartoon representations, respectively. Relevant residues of TRPV1 in the binding site are in stick representation.

**Figure 6.**
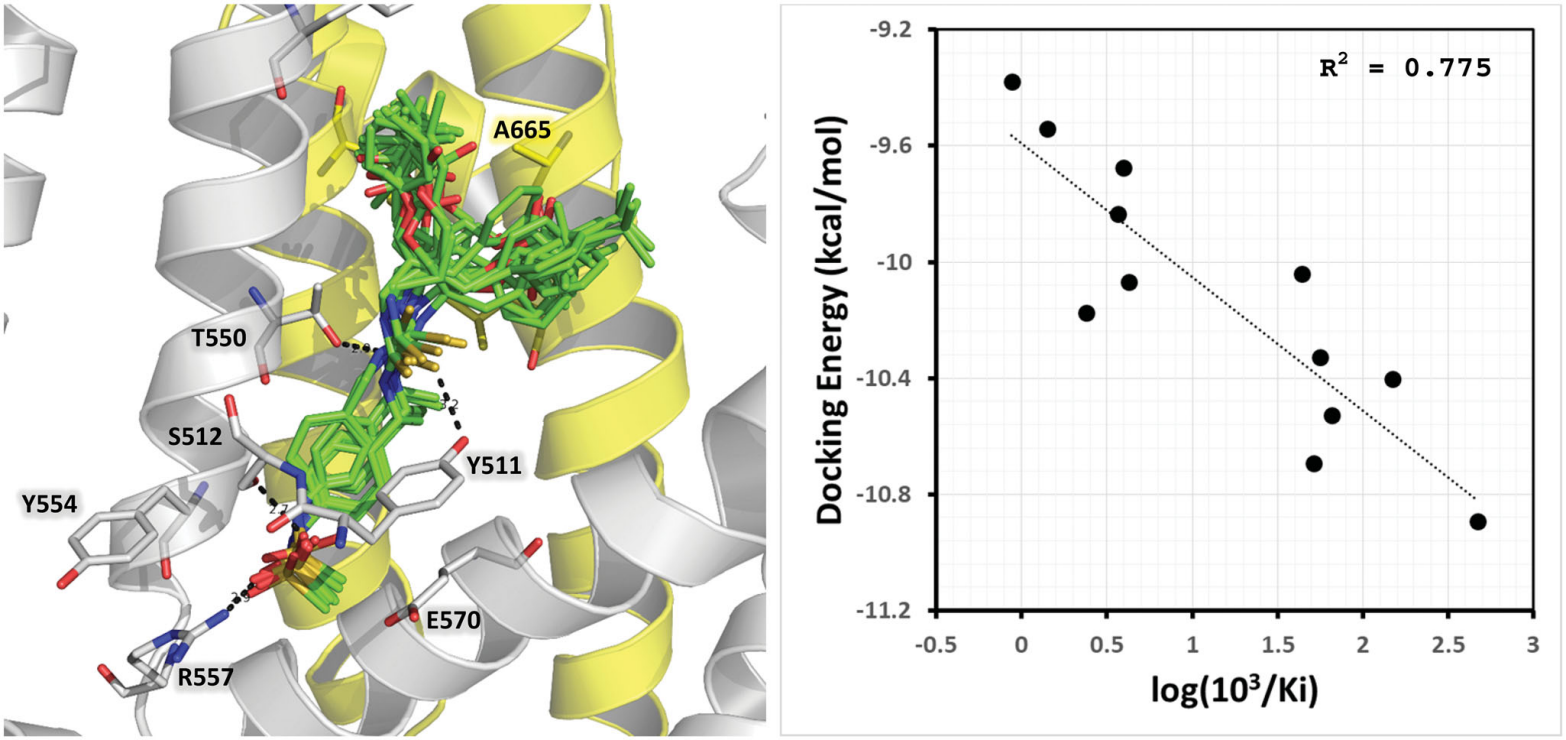
Structural models of thiourea analogues as TRPV1 antagonists. (Left) Docking poses. (Right) Plot of Docking Energy values against biological activity represented as the log (10^3^/Ki) values. The thiourea analogues are represented as green sticks. The chains A and B of TRPV1 are in gray and yellow cartoon representations, respectively. Relevant residues of TRPV1 in the binding site are in stick representation.

The docking poses obtained for compounds from series A inside the binding pocket of rat TRPV1 are shown in Figure 5 and are included in the Sdf format as Supplementary Material (Section S1). These results show that adequate orientations were found for both R and S isomers (with the exception of the R isomer of A_21) by placing the head, neck, and tail groups at the zones of the TRPV1 represented in Figure 4. All the obtained docking poses were similar to the pose of capsazepine in the reference structure. HB interactions between compounds from series A and four amino acids in the TRPV1 binding pocket (S512, R557, Y511, and T550) were observed. The same orientations were observed for compounds from series B, which are represented in Figure 6 (left) and are included in the Sdf format as Supplementary Material (Section S2).

The most probable 4-(methylsulfonylamino)benzyl interactions, as the head group, were elucidated with the docking calculations reported here. The sulphonyl oxygens were hydrogen-bonded to R557 and S512 side-chain groups, respectively. The methyl group was oriented towards the S4-S5 linker and the phenyl formed a π- π stacking with Y511. These interactions were similar but not identical to the ones reported by Lee et al. in their models before the determination of the cryomicroscopy TRPV1 structure. In those models authors reported HBs between the 4-(methylsulfonylamino)benzyl group and G558^34^^,^^42^, I564^41^ or K571^21^; however, these residues are not placed at the TRPV1’s binding site in the cryomicroscopy TRPV1 structure.

Therefore, the poses reported here for compounds from series A and B correct the flaws of earlier models, and explain why compounds that contain a 4-(methylsulfonylamino)benzyl group are antagonists. Previously, it was mentioned that salt bridge between R557 and E570 is not formed when TRPV1 is in a nonconductive TRPV1 conformation. The 4-(methylsulfonylamino)benzyl group contributes to prevent the formation of this salt bridge, since it interacts directly with R557 and places the methyl group at the space between R557 and E570 (Figures 5 and 6). On the other hand, the thiourea, as the neck group, was hydrogen-bonded to Y511 and T550 side-chain groups; meanwhile, the 3-pivaloyloxymethyl and phenyl (or phenylalkyl) substituents, which are the tail groups, occupy the large hydrophobic pocket formed between residues from chain A and B of TRPV1.

The IFD method yielded a scoring-energy value for each compound from series A and B. It was analysed whether a correlation exists between these values and experimentally determined binding affinities. Compounds from series A have two isomers (R and S), but their activities were reported for the racemic mixtures. Possible correlations were explored for R isomers, for S isomers, and for selections that combine both isomers, but low correlations were found (R^2^ < 0.3). This might be due to the fact that experimental Ki values for compounds from series A do not correspond to one isomer, but to the racemic mixture. On the other hand, compounds from series B have the Ki values for each stereo-isomer; therefore, it is possible, in theory, to explore more reliable correlations. A good correlation between the scoring-energy values and the experimental Ki values was found with a correlation coefficient of R^2^ = 0.775 (Figure 6 right). This result confirms that the use of the PDB structure with code 5IS0 as starting coordinates for a flexible docking protocol is a good strategy for studying the structural features of the TRPV1 antagonists.

Ligand-induced fit of the binding pocket could lead to important conformational changes of some residues. During the docking of compounds from series A and B, the residues Y511, S512, L515, R557, and E570 had root mean square deviation (RMSD) values above 1.5 Å (using the structure with PDB code 5IS0 as reference) when interact with the head groups of ligands. Furthermore, the residues F587 and L669 had higher RMSD values to accommodate the bulky tail groups of the ligands, and T550 had an RMSD above 1.9 Å for establishing HB with the thiourea neck group.

### Models of [4-(methylsulfonylamino)benzyl]thiourea analogues (series C) as TRPV1 agonists

Compounds from series C (Table 3) are agonists^25^. According to the head group, the series C can be separated in compounds C_1, which contain the 4-hydroxy-3-methoxybenzyl substituent, and compounds C_2, that contain the 4–(2-aminoethoxy)-3-methoxybenzyl substituent. Besides, compounds that contain modifications to the last substituent are also present (C_3, C_4, C_5, and C_8). Their neck is the thiourea, and their tail is a series of phenylalkyl substituents.

**Figure 7.**
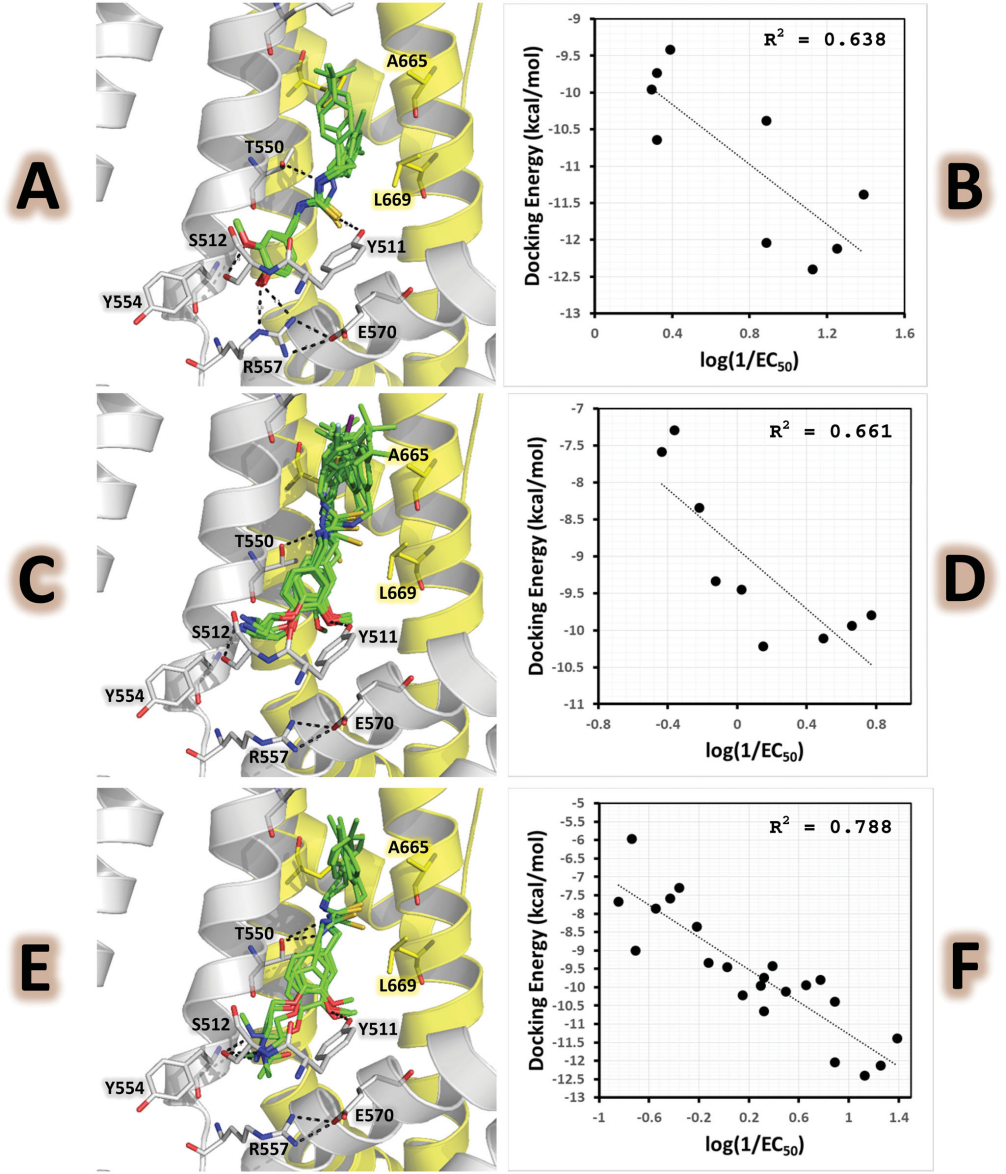
Structural models of vanillyl thioureas as TRPV1 agonists. (Left) Docking poses. (Right) Plot of Docking Energy values against biological activity represented as the log (1/EC_50_) values. (A) poses of 4-Hydroxy-3-methoxybenzyl thioureas **C_1a**–**C_1i**, (B) Plot of Docking Energy values against biological activities for **C_1a**–**C_1i**, (C) poses of 4–(2-Aminoethoxy)-3-methoxybenzyl thioureas **C_2a**–**C_2j**, (D) Plot of Docking Energy values against biological activities for **C_2a**–**C_2j**, (E) poses of compounds **C_3**, **C_4**, **C_5**, and **C_8**, (F) Plot of Docking Energy values against biological activities for **C_1a**–**C_1i**, **C_2a**–**C_2j**, **C_3**, **C_4**, **C_5**, and **C_8**. The vanillyl thioureas are represented as green sticks. The chains A and B of TRPV1 are in gray and yellow cartoon representations, respectively. Relevant residues of TRPV1 in the binding site are in stick representation.

Docking calculations of compounds from series C inside the protein of the PDB structure with code 5IRX (containing the agonist RTX) were carried out by the flexible docking protocol described above. The docking poses obtained for compounds from series C inside the binding pocket of rat TRPV1 are shown in Figure 7 and are included in the Sdf format as Supplementary Material (Section S3). These results show that adequate orientations were found for the whole series by placing the head, neck, and tail groups at the zones of the TRPV1 represented in Figure 4. All the obtained docking poses were similar to the pose of RTX in the reference structure. The head groups of compounds C_1 were hydrogen bonded to the residues S512 and R557, and their neck groups were hydrogen bonded to the residues Y511 and T550 (Figure 7A). Their tail groups were oriented to the pocket near L669 of the chain B, except for compounds **C_1c** (with a rigid (*E*)- 4-chlorostyryl substituent) and **C_1i** (with a bulky 4-(*tert*-butyl)benzyl substituent) that had the tail groups near F543 from chain A.

On the other hand, compounds C_2 (the series), **C_3**, **C_4**, **C_5**, and **C_8** placed their phenyl groups of the head outside the vanilloid pocket, by forming HBs between NH_2_ of the ligand’s aminoethoxy and the side-chain OH of S512, and between the ligand’s methoxy and side-chain OH of Y511 (Figure 7C and 7E). Their neck groups were hydrogen bonded to T550 and their tail groups were oriented to the hydrophobic pocket near the residue F543.

The most probable 4-hydroxy-3-methoxybenzyl interactions, as the head group of compounds C_1, were elucidated with the docking calculations reported here. Docking poses explain why (4-hydroxy-3-methoxybenzyl)thioureas are agonists. Previously, it was mentioned that salt bridge between R557 and E570 is formed when TRPV1 is in a conductive TRPV1 conformation. The 4-hydroxy-3-methoxybenzyl group allows the formation of this salt bridge, since it interacts directly with R557 (Figure 7A). Poses of compounds C_2, **C_3**, **C_4**, **C_5**, and **C_8** show that 4–(2-aminoalkoxy)-3-methoxybenzyl as head group, also allows the formation of the R557–E570 salt bridge (Figures 7D and 7E).

Considering IFD scoring-energy values for compounds from series C, analyses of possible correlations with experimentally determined binding affinities were performed. A good correlation between the scoring-energy values and the experimental log(1/EC_50_) values was found for compounds C_1 (nine compounds) with a correlation coefficient of R^2^ = 0.638 (Figure 7B). A good correlation was also found for compounds C_2 (nine compounds) with a correlation coefficient of R^2^ = 0.661 (Figure 7D). Finally, a good correlation was also found for the whole series C, including compounds C_1, C_2, **C_3**, **C_4**, **C_5**, and **C_8** (twenty-two compounds) with a correlation coefficient of R^2^ = 0.788 (Figure 7F). This result confirms that the use of the PDB structure with code 5IRX as starting coordinates for a flexible docking protocol is a good strategy for studying the structural features of the TRPV1 agonists.

## Conclusions

The design of novel capsaicin-like TRPV1 antagonists and agonists has been moving to a new era in which the structural information should play a preponderant role. Access to structures of antagonist-and agonist- bound states of TRPV1 in PDB will lead to a more rational design. Specific structural features have been provided to medicinal chemists involved in the design of TRPV1 modulators, and they should take full advantage of the new available information with the help of molecular modelling methods, as done in successful studies of other biological targets^51–56^. After the availability of rat TRPV1 structures at near-atomic resolution, several works reported novel TRPV1 agonists or antagonists including docking calculations^28^^,^^57–59^. These works determined the orientations of selected candidates; and there are no recent reports with comparisons between docking poses of congeneric compounds.

Here, several specific structural characteristics, related to capsaicin-like TRPV1 antagonists and agonists, are discussed to draw the attention of medicinal chemists. Capsaicin-like TRPV1 modulators have a pharmacophore composed by head, neck, and tail groups, and these groups are placed at specific subsites at the capsaicin binding site. The available structural information will allow to construct more reliable models between TRPV1 and ligands that inactivate or activate ion conduction. However, it is important to use this information in such a way as to provide more comprehensive details, i.e. more convincing knowledge of the interactions between the pharmacophore of the novel ligands and the key residues at subsites adopting conformations in nonconductive or conductive states.

As a brief conclusion, the intention in this work is not to criticise the lack of the use of structural models and computer-aided molecular design methods in the design of TRPV1 modulators, but to emphasise that nonconductive and conductive models are available since a few years ago, and pharmacophoric characteristics of capsaicin-like antagonists and agonists have been revealed since many years ago, and the use of all this information together will contribute to a deeper understanding of the interactions between novel molecules and TRPV1. Indeed, in this new era, the use of structural information and molecular docking, if used properly, will cause a remarkable leap forward in the design of more potent TRPV1 modulators.

## Supplementary Material

Supplemental Material

## References

[1] Tóth A, Boczán J, Kedei N, et al. Expression and distribution of vanilloid receptor 1 (TRPV1) in the adult rat brain. Brain Res Mol Brain Res 2005;135:162–8. 15857679 10.1016/j.molbrainres.2004.12.003

[2] Lizanecz E, Bagi Z, Pásztor ET, et al. Phosphorylation-dependent desensitization by anandamide of vanilloid receptor-1 (TRPV1) function in rat skeletal muscle arterioles and in Chinese hamster ovary cells expressing TRPV1. Mol Pharmacol 2006;69:1015–23. 16338989 10.1124/mol.105.015644

[3] Cavanaugh DJ, Chesler AT, Jackson AC, et al. Trpv1 reporter mice reveal highly restricted brain distribution and functional expression in arteriolar smooth muscle cells. J Neurosci 2011;31:5067–77. 21451044 10.1523/JNEUROSCI.6451-10.2011 PMC3087977

[4] Duitama M, Vargas-López V, Casas Z, et al. TRP channels role in pain associated with neurodegenerative diseases. Front Neurosci 2020;14:782. 32848557 10.3389/fnins.2020.00782 PMC7417429

[5] Jardín I, López JJ, Diez R, et al. TRPs in pain sensation. Front Physiol 2017;8:392. 28649203 10.3389/fphys.2017.00392 PMC5465271

[6] Xie Z, Hu H. TRP channels as drug targets to relieve itch. Pharmaceuticals. 2018;11:100. 10.3390/ph11040100 PMC6316386 30301231

[7] Chahl LA. TRP channels and psychiatric disorders. Adv Exp Med Biol 2011;704:987–1009. 21290337 10.1007/978-94-007-0265-3_51

[8] Sun F-J, Guo W, Zheng D-H, et al. Increased expression of TRPV1 in the cortex and hippocampus from patients with mesial temporal lobe epilepsy. J Mol Neurosci 2013;49:182–93. 22936245 10.1007/s12031-012-9878-2

[9] Gram DX, Holst JJ, Szallasi A. TRPV1: a potential therapeutic target in type 2 diabetes and comorbidities? Trends Mol Med 2017;23:1002–13. 29137713 10.1016/j.molmed.2017.09.005

[10] Wang M, Ji P, Wang R, et al. TRPV1 agonist capsaicin attenuates lung ischemia-reperfusion injury in rabbits. J Surg Res 2012;173:153–60. 20950828 10.1016/j.jss.2010.08.053

[11] Ternesten-Hasséus E, Johansson K, Löwhagen O, et al. Inhalation method determines outcome of capsaicin inhalation in patients with chronic cough due to sensory hyperreactivity. Pulm Pharmacol Ther 2006;19:172–8. 15990345 10.1016/j.pupt.2005.04.010

[12] Brand L, Berman E, Schwen R, et al. NE-19550: a novel, orally active anti-inflammatory analgesic. Drugs Exp Clin Res 1987;13:259–65. 2960511

[13] Szallasi A, Blumberg PM. Resiniferatoxin, a phorbol-related diterpene, acts as an ultrapotent analog of capsaicin, the irritant constituent in red pepper. Neuroscience 1989;30:515–20. 2747924 10.1016/0306-4522(89)90269-8

[14] Smart D, Gunthorpe MJ, Jerman JC, et al. The endogenous lipid anandamide is a full agonist at the human vanilloid receptor (hVR1). Br J Pharmacol 2000;129:227–30. 10694225 10.1038/sj.bjp.0703050 PMC1571834

[15] Chu CJ, Huang SM, De Petrocellis L, et al. N-oleoyldopamine, a novel endogenous capsaicin-like lipid that produces hyperalgesia. J Biol Chem 2003;278:13633–9. 12569099 10.1074/jbc.M211231200

[16] Norman MH, Zhu J, Fotsch C, et al. Novel vanilloid receptor-1 antagonists: 1. Conformationally restricted analogues of trans-cinnamides. J Med Chem 2007;50:3497–514. 17585749 10.1021/jm070189q

[17] Doherty EM, Fotsch C, Bannon AW, et al. Novel vanilloid receptor-1 antagonists: 2. structure-activity relationships of 4-oxopyrimidines leading to the selection of a clinical candidate. J Med Chem 2007;50:3515–27. 17585750 10.1021/jm070190p

[18] Pretze M, Pallavi P, Roscher M, et al. Radiofluorinated N-Octanoyl Dopamine ([18F]F-NOD) as a tool to study tissue distribution and elimination of NOD *in vitro* and *in vivo*. J Med Chem 2016;59:9855–65. 27731639 10.1021/acs.jmedchem.6b01191

[19] Pallavi P, Pretze M, Caballero J, et al. Analyses of synthetic N-acyl dopamine derivatives revealing different structural requirements for their anti-inflammatory and transient-receptor-potential-channel-of-the-vanilloid-receptor-subfamily-subtype-1 (TRPV1)-activating properties. J Med Chem 2018;61:3126–37. 29543451 10.1021/acs.jmedchem.8b00156

[20] Benso B, Bustos D, Zarraga MO, et al. Chalcone derivatives as non-canonical ligands of TRPV1. Int J Biochem Cell Biol 2019;112:18–23. 31026506 10.1016/j.biocel.2019.04.010

[21] Ha T-H, Ryu H, Kim S-E, et al. TRPV1 antagonist with high analgesic efficacy: 2-Thio pyridine C-region analogues of 2-(3-fluoro-4-methylsulfonylaminophenyl)propanamides. Bioorg Med Chem 2013;21:6657–64. 24035514 10.1016/j.bmc.2013.08.015 PMC6957258

[22] Carnevale V, Rohacs T. TRPV1: a target for rational drug design. Pharmaceuticals. 2016;9:52. 10.3390/ph9030052 PMC5039505 27563913

[23] Raisinghani M, Pabbidi RM, Premkumar LS. Activation of transient receptor potential vanilloid 1 (TRPV1) by resiniferatoxin. J Physiol 2005;567:771–86. 16037081 10.1113/jphysiol.2005.087874 PMC1474234

[24] Touska F, Marsakova L, Teisinger J, et al. A “cute” desensitization of TRPV1. Curr Pharm Biotechnol 2011;12:122–9. 20932251 10.2174/138920111793937826

[25] Wrigglesworth R, Walpole CS, Bevan S, et al. Analogues of capsaicin with agonist activity as novel analgesic agents: structure-activity studies. 4. Potent, orally active analgesics. J Med Chem 1996;39:4942–51. 8960554 10.1021/jm960512h

[26] Cho Y, Kim MS, Kim HS, et al. The SAR analysis of TRPV1 agonists with the α-methylated B-region. Bioorg Med Chem Lett 2012;22:5227–31. 22796184 10.1016/j.bmcl.2012.06.059 PMC3799874

[27] Ann J, Kim HS, Thorat SA, et al. Discovery of nonpungent transient receptor potential vanilloid 1 (TRPV1) agonist as strong topical analgesic. J Med Chem 2020;63:418–24. 31702924 10.1021/acs.jmedchem.9b01046

[28] Li J, Nie C, Qiao Y, et al. Design, synthesis and biological evaluation of novel 2,3,4,9-tetrahydro-1H-pyrido[3,4-b]indole triazole derivatives as potent TRPV1 antagonists. Eur J Med Chem 2019;178:433–45. 31202991 10.1016/j.ejmech.2019.06.007

[29] Ahn S, Kim YS, Kim MS, et al. Discovery of indane propanamides as potent and selective TRPV1 antagonists. Bioorg Med Chem Lett 2020;30:126838. 31864799 10.1016/j.bmcl.2019.126838

[30] Urban L, Dray A. Capsazepine, a novel capsaicin antagonist, selectively antagonises the effects of capsaicin in the mouse spinal cord *in vitro*. Neurosci Lett 1991;134:9–11. 1726117 10.1016/0304-3940(91)90496-g

[31] Valenzano KJ, Grant ER, Wu G, et al. N-(4-tertiarybutylphenyl)-4-(3-chloropyridin-2-yl)tetrahydropyrazine -1(2H)-carbox-amide (BCTC), a novel, orally effective vanilloid receptor 1 antagonist with analgesic properties: I. *in vitro* characterization and pharmacokinetic properties. J Pharmacol Exp Ther 2003;306:377–86. 12721338 10.1124/jpet.102.045674

[32] Tamayo N, Liao H, Stec MM, et al. Design and synthesis of peripherally restricted transient receptor potential vanilloid 1 (TRPV1) antagonists. J Med Chem 2008;51:2744–57. 18386885 10.1021/jm7014638

[33] Wang H-L, Katon J, Balan C, et al. Novel vanilloid receptor-1 antagonists: 3. The identification of a second-generation clinical candidate with improved physicochemical and pharmacokinetic properties. J Med Chem 2007;50:3528–39. 17585751 10.1021/jm070191h

[34] Bhondwe RS, Kang DW, Kim MS, et al. Structure-activity relationships and molecular modeling of the N-(3-pivaloyloxy-2-benzylpropyl)-N’-[4-(methylsulfonylamino)benzyl] thiourea template for TRPV1 antagonism. Bioorg Med Chem Lett 2012;22:3656–60. 22546668 10.1016/j.bmcl.2012.04.034 PMC3799871

[35] Saku O, Ishida H, Atsumi E, et al. Discovery of novel 5,5-diarylpentadienamides as orally available transient receptor potential vanilloid 1 (TRPV1) antagonists. J Med Chem 2012;55:3436–51. 22394104 10.1021/jm300101n

[36] Yang F, Zheng J. Understand spiciness: mechanism of TRPV1 channel activation by capsaicin. Protein Cell 2017;8:169–77. 28044278 10.1007/s13238-016-0353-7 PMC5326624

[37] Fernández-Ballester G, Ferrer-Montiel A. Molecular modeling of the full-length human TRPV1 channel in closed and desensitized states. J Membr Biol 2008;223:161–72. 18791833 10.1007/s00232-008-9123-7

[38] Moiseenkova-Bell VY, Stanciu LA, Serysheva II, et al. Structure of TRPV1 channel revealed by electron cryomicroscopy. Proc Natl Acad Sci USA 2008;105:7451–5. 18490661 10.1073/pnas.0711835105 PMC2396679

[39] Winter Z, Buhala A, Ötvös F, et al. Functionally important amino acid residues in the transient receptor potential vanilloid 1 (TRPV1) ion channel–an overview of the current mutational data. Mol Pain 2013;9:30. 23800232 10.1186/1744-8069-9-30 PMC3707783

[40] Lee JH, Lee Y, Ryu H, et al. Structural insights into transient receptor potential vanilloid type 1 (TRPV1) from homology modeling, flexible docking, and mutational studies. J Comput Aided Mol Des 2011;25:317–27. 21448716 10.1007/s10822-011-9421-5 PMC3420359

[41] Kim MS, Ki Y, Ahn SY, et al. Asymmetric synthesis and receptor activity of chiral simplified resiniferatoxin (sRTX) analogues as transient receptor potential vanilloid 1 (TRPV1) ligands. Bioorg Med Chem Lett 2014;24:382–5. 24321344 10.1016/j.bmcl.2013.10.064 PMC6957263

[42] Kim N-J, Li F-N, Lee JH, et al. Heterocycle-linked phenylbenzyl amides as novel TRPV1 antagonists and their TRPV1 binding modes: constraint-induced enhancement of *in vitro* and *in vivo* activities. Chem Asian J 2013;8:400–9. 23208797 10.1002/asia.201200730

[43] Boukalova S, Marsakova L, Teisinger J, et al. Conserved residues within the putative S4-S5 region serve distinct functions among thermosensitive vanilloid transient receptor potential (TRPV) channels. J Biol Chem 2010;285:41455–62. 21044960 10.1074/jbc.M110.145466 PMC3009871

[44] Liao M, Cao E, Julius D, et al. Structure of the TRPV1 ion channel determined by electron cryo-microscopy. Nature 2013;504:107–12. 24305160 10.1038/nature12822 PMC4078027

[45] Cao E, Liao M, Cheng Y, et al. TRPV1 structures in distinct conformations reveal activation mechanisms. Nature 2013;504:113–8. 24305161 10.1038/nature12823 PMC4023639

[46] Gao Y, Cao E, Julius D, et al. TRPV1 structures in nanodiscs reveal mechanisms of ligand and lipid action. Nature 2016;534:347–51. 27281200 10.1038/nature17964 PMC4911334

[47] Caballero J. Computational modeling to explain why 5,5-diarylpentadienamides are TRPV1 antagonists. Molecules 2021;26:1765. 33801115 10.3390/molecules26061765 PMC8004144

[48] Kim HS, Jin M-K, Kang S-U, et al. α-Methylated simplified resiniferatoxin (sRTX) thiourea analogues as potent and stereospecific TRPV1 antagonists. Bioorg Med Chem Lett 2014;24:2685–8. 24794110 10.1016/j.bmcl.2014.04.054 PMC6957243

[49] Sherman W, Day T, Jacobson MP, et al. Novel procedure for modeling ligand/receptor induced fit effects. J Med Chem 2006;49:534–53. 16420040 10.1021/jm050540c

[50] Harder E, Damm W, Maple J, et al. OPLS3: a force field providing broad coverage of drug-like small molecules and proteins. J Chem Theory Comput 2016;12:281–96. 26584231 10.1021/acs.jctc.5b00864

[51] Lagoutte-Renosi J, Allemand F, Ramseyer C, et al. Molecular modeling in cardiovascular pharmacology: current state of the art and perspectives. Drug Discov Today 2021;2021:S1359-6446(21)00533-X. 10.1016/j.drudis.2021.11.026 34863931

[52] Yalcin-Ozkat G. Molecular modeling strategies of cancer multidrug resistance. Drug Resist Updat 2021;59:100789. 34973929 10.1016/j.drup.2021.100789

[53] Alzate-Morales JH, Vergara-Jaque A, Caballero J. Computational study on the interaction of N1 substituted pyrazole derivatives with B-Raf Kinase: an unusual water wire hydrogen-bond network and novel interactions at the entrance of the active site. J Chem Inf Model 2010;50:1101–12. 20524689 10.1021/ci100049h

[54] Patel H, Kukol A. Integrating molecular modelling methods to advance influenza A virus drug discovery. Drug Discov Today 2021;26:503–10. 33220433 10.1016/j.drudis.2020.11.014

[55] Ramírez D, Concha G, Arévalo B, et al. Discovery of Novel TASK-3 channel blockers using a pharmacophore-based virtual screening. Int J Mol Sci 2019;20:4014. 10.3390/ijms20164014 PMC6720600 31426491

[56] Adelusi TI, Oyedele A-QK, Boyenle ID, et al. Molecular modeling in drug discovery. Informa Med Unlocked 2022;29:100880.

[57] Thorat SA, Lee Y, Jung A, et al. Discovery of benzopyridone-based transient receptor potential vanilloid 1 agonists and antagonists and the structural elucidation of their activity shift. J Med Chem 2021;64:370–84. 33385210 10.1021/acs.jmedchem.0c00982

[58] Kang JM, Kwon SO, Ann J, et al. 2-(Halogenated Phenyl) acetamides and propanamides as potent TRPV1 antagonists. Bioorg Med Chem Lett 2021;48:128266. 34273488 10.1016/j.bmcl.2021.128266

[59] Å Nilsson JL, Mallet C, Shionoya K, et al. Paracetamol analogues conjugated by FAAH induce TRPV1-mediated antinociception without causing acute liver toxicity. Eur J Med Chem 2021;213:113042. 33257173 10.1016/j.ejmech.2020.113042

