# Supplementary material for: A new era for the design of TRPV1 antagonists and agonists with the use of structural information and molecular docking of capsaicin-like compounds
Source: J Enzyme Inhib Med Chem. 2022 Aug 16;37(1):2169–78. doi: 10.1080/14756366.2022.2110089 (PMC9387342; doi:10.1080/14756366.2022.2110089)
Supplement: Supplemental Material [file IENZ_A_2110089_SM3872.pdf]

Supplementary Information

A new era for the design of TRPV1 antagonists and agonists with the use of structural information and molecular docking of capsaicin-like compounds

Julio Caballero

Centro de Bioinformática,, Simulación y Modelado (CBSM), Facultad de Ingeniería, Universidad de Talca, Talca 3460000, Chile; jcaballero@utalca.cl; Tel.: +56-712-418-850

S1. Series A

Coordinates of compounds from Series A in Sdf file.

```
A_1R
                  3D
 Structure written by MMmdl.
 72 73  0  0  1  0            999 V2000
  111.6059   92.9986  110.2248 S   0  0  0  0  0  0
  108.2430   95.8456  103.0331 C   0  0  0  0  0  0
  106.7894   95.7699   97.0473 C   0  0  0  0  0  0
  112.2541   93.3929  108.6367 N   0  0  0  0  0  0
  111.0562   94.2294  110.7927 O   0  0  0  0  0  0
  110.7864   91.7925  110.0583 O   0  0  0  0  0  0
  105.7465   95.4950   95.9228 C   0  0  0  0  0  0
  104.8685  101.2398   99.5247 C   0  0  0  0  0  0
  106.9591   95.3927  104.0166 S   0  0  0  0  0  0
  106.5693   96.9320   97.7119 O   0  0  0  0  0  0
  108.2287   95.7927  101.6663 N   0  0  0  0  0  0
  109.4303   96.3309  103.5049 N   0  0  0  0  0  0
  105.8014  100.2578   99.8770 C   0  0  0  0  0  0
  103.5976  101.2380  100.1243 C   0  0  0  0  0  0
  107.7122   94.9873   97.2873 O   0  0  0  0  0  0
  105.4680   99.2505  100.7881 C   0  0  0  0  0  0
  103.2728  100.2382  101.0455 C   0  0  0  0  0  0
  111.5306   94.2045  107.7560 C   0  0  0  0  0  0
  106.5241   96.9669  100.1711 C   0  0  1  0  0  0
  106.4701   98.1639  101.1623 C   0  0  0  0  0  0
  113.1500   92.5348  111.1855 C   0  0  0  0  0  0
  107.3107   97.3024   98.8734 C   0  0  0  0  0  0
  107.0258   95.6472  100.8191 C   0  0  0  0  0  0
  109.6656   96.8319  104.8714 C   0  0  0  0  0  0
  104.1970   99.2464  101.3683 C   0  0  0  0  0  0
  110.2779   95.8623  105.8833 C   0  0  0  0  0  0
  110.9607   93.6521  106.6081 C   0  0  0  0  0  0
  111.4521   95.5782  107.9773 C   0  0  0  0  0  0
  110.3385   94.4784  105.6710 C   0  0  0  0  0  0
```

```
  110.8245   96.4028  107.0495 C   0  0  0  0  0  0
  104.3241   95.9451   96.3524 C   0  0  0  0  0  0
  106.1663   96.2741   94.6521 C   0  0  0  0  0  0
  105.6891   93.9821   95.5863 C   0  0  0  0  0  0
  105.2366  102.2768   98.4761 C   0  0  0  0  0  0
  102.5592  102.2991   99.7862 C   0  0  0  0  0  0
  112.5535   92.5496  108.1727 H   0  0  0  0  0  0
  109.1206   95.8284  101.1896 H   0  0  0  0  0  0
  110.2148   96.3552  102.8696 H   0  0  0  0  0  0
  106.7845  100.2760   99.4307 H   0  0  0  0  0  0
  102.2983  100.2196  101.5113 H   0  0  0  0  0  0
  105.4906   96.7665   99.8825 H   0  0  0  0  0  0
  107.4561   98.6077  101.2894 H   0  0  0  0  0  0
  106.1989   97.8053  102.1557 H   0  0  0  0  0  0
  112.8594   92.2756  112.2027 H   0  0  0  0  0  0
  113.8197   93.3921  111.2020 H   0  0  0  0  0  0
  113.6345   91.6929  110.6937 H   0  0  0  0  0  0
  107.4832   98.3749   98.7988 H   0  0  0  0  0  0
  108.3085   96.8587   98.8872 H   0  0  0  0  0  0
  106.2063   95.1810  101.3656 H   0  0  0  0  0  0
  107.2512   94.9226  100.0361 H   0  0  0  0  0  0
  110.3624   97.6654  104.7715 H   0  0  0  0  0  0
  108.7635   97.2681  105.3037 H   0  0  0  0  0  0
  103.9221   98.4787  102.0769 H   0  0  0  0  0  0
  111.0182   92.5887  106.4243 H   0  0  0  0  0  0
  111.8962   96.0153  108.8568 H   0  0  0  0  0  0
  109.9178   94.0354  104.7799 H   0  0  0  0  0  0
  110.7851   97.4670  107.2338 H   0  0  0  0  0  0
  103.5799   95.7006   95.5917 H   0  0  0  0  0  0
  104.2640   97.0239   96.5112 H   0  0  0  0  0  0
  104.0067   95.4587   97.2769 H   0  0  0  0  0  0
  105.4543   96.1245   93.8409 H   0  0  0  0  0  0
  107.1396   95.9534   94.2796 H   0  0  0  0  0  0
  106.2217   97.3492   94.8340 H   0  0  0  0  0  0
  104.9751   93.7686   94.7897 H   0  0  0  0  0  0
  105.3912   93.3923   96.4534 H   0  0  0  0  0  0
  106.6553   93.5984   95.2577 H   0  0  0  0  0  0
  106.2409  102.1306   98.0741 H   0  0  0  0  0  0
  105.1761  103.2820   98.8868 H   0  0  0  0  0  0
  104.5315  102.2199   97.6498 H   0  0  0  0  0  0
  102.9489  103.2971   99.9886 H   0  0  0  0  0  0
  101.6483  102.1785  100.3737 H   0  0  0  0  0  0
  102.2844  102.2435   98.7321 H   0  0  0  0  0  0
  1  4  1  0  0  0
  1  5  2  0  0  0
  1  6  2  0  0  0
  1 21  1  0  0  0
  2 12  1  0  0  0
  2  9  2  0  0  0
  2 11  1  0  0  0
  3 10  1  0  0  0
  3  7  1  0  0  0
  3 15  2  0  0  0
  4 18  1  0  0  0
  4 36  1  0  0  0
  7 31  1  0  0  0
  7 32  1  0  0  0
  7 33  1  0  0  0
  8 13  2  0  0  0
  8 34  1  0  0  0
  8 14  1  0  0  0
 10 22  1  0  0  0
 11 23  1  0  0  0
 11 37  1  0  0  0
 12 24  1  0  0  0
 12 38  1  0  0  0
 13 16  1  0  0  0
 13 39  1  0  0  0
 14 17  2  0  0  0
 14 35  1  0  0  0
 16 20  1  0  0  0
```

```
 16 25  2  0  0  0
 17 25  1  0  0  0
 17 40  1  0  0  0
 18 27  2  0  0  0
 18 28  1  0  0  0
 19 23  1  0  0  0
 19 20  1  0  0  0
 19 22  1  0  0  0
 19 41  1  0  0  0
 20 42  1  0  0  0
 20 43  1  0  0  0
 21 44  1  0  0  0
 21 45  1  0  0  0
 21 46  1  0  0  0
 22 47  1  0  0  0
 22 48  1  0  0  0
 23 49  1  0  0  0
 23 50  1  0  0  0
 24 26  1  0  0  0
 24 51  1  0  0  0
 24 52  1  0  0  0
 25 53  1  0  0  0
 26 30  1  0  0  0
 26 29  2  0  0  0
 27 29  1  0  0  0
 27 54  1  0  0  0
 28 30  2  0  0  0
 28 55  1  0  0  0
 29 56  1  0  0  0
 30 57  1  0  0  0
 31 58  1  0  0  0
 31 59  1  0  0  0
 31 60  1  0  0  0
 32 61  1  0  0  0
 32 62  1  0  0  0
 32 63  1  0  0  0
 33 64  1  0  0  0
 33 65  1  0  0  0
 33 66  1  0  0  0
 34 67  1  0  0  0
 34 68  1  0  0  0
 34 69  1  0  0  0
 35 70  1  0  0  0
 35 71  1  0  0  0
 35 72  1  0  0  0
M  END
> <s_m_entry_id>
1

> <s_m_entry_name>
A_1R.1

> <s_m_Source_Path>
C:\Users\julio\OneDrive - Universidad de Talca\Escritorio\Nueva carpeta

> <s_m_Source_File>
A_1R.mol2

> <i_m_Source_File_Index>
1

$$$$
A_1S
                    3D
 Structure written by MMmdl.
 72 73  0  0  1  0            999 V2000
  111.0663   92.7464  109.9592 S   0  0  0  0  0  0
  108.2277   96.2948  102.7432 C   0  0  0  0  0  0
  106.2613   94.9171   96.8310 C   0  0  0  0  0  0
  111.8561   93.3000  108.4851 N   0  0  0  0  0  0
  109.6393   92.5929  109.6574 O   0  0  0  0  0  0
```

111.8487	91.6094	110.4568	O	0	0	0	0	0	0
105.3789	94.8959	95.5458	C	0	0	0	0	0	0
105.0398	101.1214	99.2415	C	0	0	0	0	0	0
106.7519	95.9320	103.4489	S	0	0	0	0	0	0
105.6234	95.3707	97.9385	O	0	0	0	0	0	0
108.3841	96.3271	101.3915	N	0	0	0	0	0	0
109.3804	96.5969	103.4127	N	0	0	0	0	0	0
106.0564	100.2409	98.8661	C	0	0	0	0	0	0
103.7373	100.6349	99.4409	C	0	0	0	0	0	0
107.4503	94.5924	96.7960	O	0	0	0	0	0	0
105.7947	98.8836	98.6724	C	0	0	0	0	0	0
103.4739	99.2765	99.2403	C	0	0	0	0	0	0
111.2474	94.1907	107.5981	C	0	0	0	0	0	0
107.3046	96.7844	99.1452	C	0	0	2	0	0	0
106.9201	97.9661	98.2077	C	0	0	0	0	0	0
111.3219	94.1942	111.1281	C	0	0	0	0	0	0
106.2968	95.6007	99.1768	C	0	0	0	0	0	0
107.6472	97.2935	100.5629	C	0	0	0	0	0	0
109.4657	96.9904	104.8305	C	0	0	0	0	0	0
104.4924	98.4083	98.8506	C	0	0	0	0	0	0
110.0853	95.9739	105.7896	C	0	0	0	0	0	0
111.2636	95.5586	107.8626	C	0	0	0	0	0	0
110.6539	93.7093	106.4311	C	0	0	0	0	0	0
110.6804	96.4469	106.9631	C	0	0	0	0	0	0
110.0761	94.5990	105.5260	C	0	0	0	0	0	0
105.2639	93.4411	95.0348	C	0	0	0	0	0	0
103.9474	95.4514	95.7786	C	0	0	0	0	0	0
106.0608	95.7618	94.4553	C	0	0	0	0	0	0
105.3714	102.5925	99.4111	C	0	0	0	0	0	0
102.6027	101.5600	99.8579	C	0	0	0	0	0	0
112.7989	92.9818	108.3027	H	0	0	0	0	0	0
109.2729	96.0072	101.0269	H	0	0	0	0	0	0
110.2491	96.5603	102.8970	H	0	0	0	0	0	0
107.0548	100.6185	98.7028	H	0	0	0	0	0	0
102.4746	98.8886	99.3762	H	0	0	0	0	0	0
108.2237	96.3775	98.7184	H	0	0	0	0	0	0
106.6554	97.6139	97.2124	H	0	0	0	0	0	0
107.8192	98.5665	98.0567	H	0	0	0	0	0	0
111.0334	93.8862	112.1312	H	0	0	0	0	0	0
110.6914	95.0227	110.8105	H	0	0	0	0	0	0
112.3711	94.4825	111.1178	H	0	0	0	0	0	0
106.7955	94.6859	99.5031	H	0	0	0	0	0	0
105.5262	95.8043	99.9204	H	0	0	0	0	0	0
108.2554	98.1922	100.4753	H	0	0	0	0	0	0
106.7224	97.6133	101.0483	H	0	0	0	0	0	0
110.0852	97.8881	104.8557	H	0	0	0	0	0	0
108.5012	97.3007	105.2331	H	0	0	0	0	0	0
104.2539	97.3705	98.6808	H	0	0	0	0	0	0
111.7286	95.9371	108.7604	H	0	0	0	0	0	0
110.6443	92.6494	106.2217	H	0	0	0	0	0	0
110.6946	97.5043	107.1836	H	0	0	0	0	0	0
109.6160	94.2124	104.6282	H	0	0	0	0	0	0
104.7311	93.3958	94.0843	H	0	0	0	0	0	0
104.7206	92.8036	95.7330	H	0	0	0	0	0	0
106.2442	92.9921	94.8747	H	0	0	0	0	0	0
103.3549	95.4240	94.8627	H	0	0	0	0	0	0
103.9644	96.4895	96.1150	H	0	0	0	0	0	0
103.4028	94.8722	96.5272	H	0	0	0	0	0	0
105.4796	95.7718	93.5315	H	0	0	0	0	0	0
107.0547	95.3938	94.1987	H	0	0	0	0	0	0
106.1721	96.8007	94.7713	H	0	0	0	0	0	0
106.3908	102.8214	99.0929	H	0	0	0	0	0	0
105.2725	102.8819	100.4567	H	0	0	0	0	0	0
104.6943	103.2061	98.8170	H	0	0	0	0	0	0
102.8725	102.1231	100.7522	H	0	0	0	0	0	0
101.6887	101.0087	100.0811	H	0	0	0	0	0	0
102.3783	102.2673	99.0590	H	0	0	0	0	0	0

1	4	1	0	0	0
1	5	2	0	0	0
1	6	2	0	0	0
1	21	1	0	0	0

```
  2 12  1  0  0  0
  2  9  2  0  0  0
  2 11  1  0  0  0
  3 10  1  0  0  0
  3  7  1  0  0  0
  3 15  2  0  0  0
  4 18  1  0  0  0
  4 36  1  0  0  0
  7 31  1  0  0  0
  7 32  1  0  0  0
  7 33  1  0  0  0
  8 13  2  0  0  0
  8 34  1  0  0  0
  8 14  1  0  0  0
 10 22  1  0  0  0
 11 23  1  0  0  0
 11 37  1  0  0  0
 12 24  1  0  0  0
 12 38  1  0  0  0
 13 16  1  0  0  0
 13 39  1  0  0  0
 14 17  2  0  0  0
 14 35  1  0  0  0
 16 20  1  0  0  0
 16 25  2  0  0  0
 17 25  1  0  0  0
 17 40  1  0  0  0
 18 27  2  0  0  0
 18 28  1  0  0  0
 19 23  1  0  0  0
 19 20  1  0  0  0
 19 22  1  0  0  0
 19 41  1  0  0  0
 20 42  1  0  0  0
 20 43  1  0  0  0
 21 44  1  0  0  0
 21 45  1  0  0  0
 21 46  1  0  0  0
 22 47  1  0  0  0
 22 48  1  0  0  0
 23 49  1  0  0  0
 23 50  1  0  0  0
 24 26  1  0  0  0
 24 51  1  0  0  0
 24 52  1  0  0  0
 25 53  1  0  0  0
 26 30  1  0  0  0
 26 29  2  0  0  0
 27 29  1  0  0  0
 27 54  1  0  0  0
 28 30  2  0  0  0
 28 55  1  0  0  0
 29 56  1  0  0  0
 30 57  1  0  0  0
 31 58  1  0  0  0
 31 59  1  0  0  0
 31 60  1  0  0  0
 32 61  1  0  0  0
 32 62  1  0  0  0
 32 63  1  0  0  0
 33 64  1  0  0  0
 33 65  1  0  0  0
 33 66  1  0  0  0
 34 67  1  0  0  0
 34 68  1  0  0  0
 34 69  1  0  0  0
 35 70  1  0  0  0
 35 71  1  0  0  0
 35 72  1  0  0  0
M  END
> <s_m_entry_id>
```

```
2

> <s_m_entry_name>
A_1S.1

> <s_m_Source_Path>
C:\Users\julio\OneDrive - Universidad de Talca\Escritorio\Nueva carpeta

> <s_m_Source_File>
A_1S.mol2

> <i_m_Source_File_Index>
1

$$$$
A_2R
                    3D
 Structure written by MMmdl.
 78 79  0  0  1  0            999 V2000
  111.1539   92.9408  109.8950 S   0  0  0  0  0  0
  108.8906   96.6789  102.7941 C   0  0  0  0  0  0
  103.5101   99.6509  100.2627 C   0  0  0  0  0  0
  112.0585   93.5953  108.5019 N   0  0  0  0  0  0
  109.7466   92.8942  109.4835 O   0  0  0  0  0  0
  111.8306   91.7229  110.3561 O   0  0  0  0  0  0
  102.9291  101.0804  100.0364 C   0  0  0  0  0  0
  107.6661   95.8092  103.5572 S   0  0  0  0  0  0
  104.8356   99.6305  100.5846 O   0  0  0  0  0  0
  108.8068   97.2085  101.5351 N   0  0  0  0  0  0
  110.1094   96.9571  103.3488 N   0  0  0  0  0  0
  103.7317   94.7111   95.6210 C   0  0  0  0  0  0
  104.5701   95.7559   96.4130 C   0  0  0  0  0  0
  102.7994   98.6444  100.1720 O   0  0  0  0  0  0
  105.8824   95.4401   96.8025 C   0  0  0  0  0  0
  104.0635   97.0196   96.7654 C   0  0  0  0  0  0
  111.5795   94.5253  107.5616 C   0  0  0  0  0  0
  106.9453   98.3758  100.2251 C   0  0  1  0  0  0
  111.3359   94.3012  111.1975 C   0  0  0  0  0  0
  105.5112   98.3963  100.8221 C   0  0  0  0  0  0
  107.6864   97.0585  100.5896 C   0  0  0  0  0  0
  106.9789   98.5750   98.6822 C   0  0  0  0  0  0
  110.3367   97.5080  104.6919 C   0  0  0  0  0  0
  106.1426   97.5992   97.8560 C   0  0  0  0  0  0
  110.7846   96.4433  105.6823 C   0  0  0  0  0  0
  106.6594   96.3477   97.5176 C   0  0  0  0  0  0
  104.8405   97.9303   97.4780 C   0  0  0  0  0  0
  112.4397   95.5680  107.2072 C   0  0  0  0  0  0
  110.3135   94.4491  106.9648 C   0  0  0  0  0  0
  112.0443   96.5273  106.2785 C   0  0  0  0  0  0
  109.9168   95.4079  106.0351 C   0  0  0  0  0  0
  102.5194  101.6680  101.4098 C   0  0  0  0  0  0
  104.0006  102.0088   99.3984 C   0  0  0  0  0  0
  101.6919  101.0560   99.0953 C   0  0  0  0  0  0
  103.3227   95.2967   94.2427 C   0  0  0  0  0  0
  102.4207   94.2883   96.3446 C   0  0  0  0  0  0
  104.5681   93.4220   95.4017 C   0  0  0  0  0  0
  113.0108   93.2803  108.3833 H   0  0  0  0  0  0
  109.5950   97.7456  101.2028 H   0  0  0  0  0  0
  110.9207   96.5640  102.8934 H   0  0  0  0  0  0
  106.3167   94.4816   96.5599 H   0  0  0  0  0  0
  103.0639   97.3284   96.5026 H   0  0  0  0  0  0
  107.5130   99.1881  100.6817 H   0  0  0  0  0  0
  110.7845   94.0097  112.0913 H   0  0  0  0  0  0
  110.9272   95.2307  110.8011 H   0  0  0  0  0  0
  112.3879   94.4367  111.4450 H   0  0  0  0  0  0
  104.9242   97.5658  100.4262 H   0  0  0  0  0  0
  105.5622   98.2495  101.9021 H   0  0  0  0  0  0
  106.9749   96.3810  101.0637 H   0  0  0  0  0  0
  108.0078   96.5267   99.6933 H   0  0  0  0  0  0
  108.0118   98.5707   98.3287 H   0  0  0  0  0  0
  106.6093   99.5724   98.4362 H   0  0  0  0  0  0
```

111.0938	98.2936	104.6778	H	0	0	0	0	0	0
109.4142	97.9689	105.0493	H	0	0	0	0	0	0
107.6612	96.0700	97.8127	H	0	0	0	0	0	0
104.4201	98.8906	97.7403	H	0	0	0	0	0	0
113.4210	95.6442	107.6545	H	0	0	0	0	0	0
109.6208	93.6561	107.1964	H	0	0	0	0	0	0
112.7203	97.3308	106.0233	H	0	0	0	0	0	0
108.9358	95.3387	105.5873	H	0	0	0	0	0	0
102.1234	102.6798	101.3094	H	0	0	0	0	0	0
103.3626	101.7262	102.1007	H	0	0	0	0	0	0
101.7444	101.0740	101.8959	H	0	0	0	0	0	0
103.6069	103.0107	99.2193	H	0	0	0	0	0	0
104.3468	101.6301	98.4348	H	0	0	0	0	0	0
104.8785	102.1327	100.0352	H	0	0	0	0	0	0
101.3316	102.0640	98.8834	H	0	0	0	0	0	0
100.8479	100.5149	99.5261	H	0	0	0	0	0	0
101.9207	100.5944	98.1329	H	0	0	0	0	0	0
104.1829	95.5323	93.6154	H	0	0	0	0	0	0
102.7438	96.2151	94.3535	H	0	0	0	0	0	0
102.7079	94.5984	93.6723	H	0	0	0	0	0	0
102.6172	93.8243	97.3126	H	0	0	0	0	0	0
101.8560	93.5578	95.7627	H	0	0	0	0	0	0
101.7514	95.1344	96.5090	H	0	0	0	0	0	0
104.8881	92.9860	96.3495	H	0	0	0	0	0	0
105.4666	93.6013	94.8104	H	0	0	0	0	0	0
103.9993	92.6552	94.8731	H	0	0	0	0	0	0

1	4	1	0	0	0
1	5	2	0	0	0
1	6	2	0	0	0
1	19	1	0	0	0
2	11	1	0	0	0
2	8	2	0	0	0
2	10	1	0	0	0
3	9	1	0	0	0
3	7	1	0	0	0
3	14	2	0	0	0
4	17	1	0	0	0
4	38	1	0	0	0
7	32	1	0	0	0
7	33	1	0	0	0
7	34	1	0	0	0
9	20	1	0	0	0
10	21	1	0	0	0
10	39	1	0	0	0
11	23	1	0	0	0
11	40	1	0	0	0
12	13	1	0	0	0
12	35	1	0	0	0
12	36	1	0	0	0
12	37	1	0	0	0
13	16	2	0	0	0
13	15	1	0	0	0
15	26	2	0	0	0
15	41	1	0	0	0
16	27	1	0	0	0
16	42	1	0	0	0
17	28	2	0	0	0
17	29	1	0	0	0
18	21	1	0	0	0
18	20	1	0	0	0
18	22	1	0	0	0
18	43	1	0	0	0
19	44	1	0	0	0
19	45	1	0	0	0
19	46	1	0	0	0
20	47	1	0	0	0
20	48	1	0	0	0
21	49	1	0	0	0
21	50	1	0	0	0
22	24	1	0	0	0
22	51	1	0	0	0

```
 22 52  1  0  0  0
 23 25  1  0  0  0
 23 53  1  0  0  0
 23 54  1  0  0  0
 24 26  1  0  0  0
 24 27  2  0  0  0
 25 31  1  0  0  0
 25 30  2  0  0  0
 26 55  1  0  0  0
 27 56  1  0  0  0
 28 30  1  0  0  0
 28 57  1  0  0  0
 29 31  2  0  0  0
 29 58  1  0  0  0
 30 59  1  0  0  0
 31 60  1  0  0  0
 32 61  1  0  0  0
 32 62  1  0  0  0
 32 63  1  0  0  0
 33 64  1  0  0  0
 33 65  1  0  0  0
 33 66  1  0  0  0
 34 67  1  0  0  0
 34 68  1  0  0  0
 34 69  1  0  0  0
 35 70  1  0  0  0
 35 71  1  0  0  0
 35 72  1  0  0  0
 36 73  1  0  0  0
 36 74  1  0  0  0
 36 75  1  0  0  0
 37 76  1  0  0  0
 37 77  1  0  0  0
 37 78  1  0  0  0
M  END
> <s_m_entry_id>
3

> <s_m_entry_name>
A_2R.1

> <s_m_Source_Path>
C:\Users\julio\OneDrive - Universidad de Talca\Escritorio\Nueva carpeta

> <s_m_Source_File>
A_2R.mol2

> <i_m_Source_File_Index>
1

$$$$
A_2S
                    3D
 Structure written by MMmdl.
 78 79  0  0  1  0            999 V2000
  111.3777   92.5931  110.3283 S   0  0  0  0  0  0
  108.4266   95.9515  103.2407 C   0  0  0  0  0  0
  105.9053  100.8959  100.6904 C   0  0  0  0  0  0
  112.1496   93.1968  108.8593 N   0  0  0  0  0  0
  109.9761   92.2989  110.0122 O   0  0  0  0  0  0
  112.2555   91.5430  110.8567 O   0  0  0  0  0  0
  104.9963  101.0660   99.4112 C   0  0  0  0  0  0
  107.3343   94.9939  104.0568 S   0  0  0  0  0  0
  105.8316   99.7614  101.4542 O   0  0  0  0  0  0
  108.2560   96.1943  101.9136 N   0  0  0  0  0  0
  109.5355   96.5422  103.7806 N   0  0  0  0  0  0
  105.0604   95.1977   95.3055 C   0  0  0  0  0  0
  105.1256   95.5030   96.8203 C   0  0  0  0  0  0
  106.6563  101.8036  101.0436 O   0  0  0  0  0  0
  104.0288   96.0272   97.5223 C   0  0  0  0  0  0
  106.2972   95.2061   97.5312 C   0  0  0  0  0  0
```

```
111.5123   94.0627  107.9670 C   0  0  0  0  0  0
105.9339   97.3026  101.6528 C   0  0  2  0  0  0
111.4560   94.0768  111.4785 C   0  0  0  0  0  0
105.2178   98.5406  101.0664 C   0  0  0  0  0  0
107.4769   97.3606  101.4602 C   0  0  0  0  0  0
105.2812   96.0014  101.1152 C   0  0  0  0  0  0
109.7016   96.8685  105.2095 C   0  0  0  0  0  0
105.2505   95.8783   99.5964 C   0  0  0  0  0  0
110.3415   95.8375  106.1470 C   0  0  0  0  0  0
104.0907   96.2124   98.8974 C   0  0  0  0  0  0
106.3599   95.3929   98.9074 C   0  0  0  0  0  0
111.0156   93.5874  106.7543 C   0  0  0  0  0  0
111.4339   95.4182  108.2654 C   0  0  0  0  0  0
110.4484   94.4744  105.8372 C   0  0  0  0  0  0
110.8417   96.3006  107.3668 C   0  0  0  0  0  0
104.9224  102.5798   99.0546 C   0  0  0  0  0  0
105.6314  100.3674   98.1773 C   0  0  0  0  0  0
103.5312  100.5892   99.6237 C   0  0  0  0  0  0
106.1761   95.9632   94.5548 C   0  0  0  0  0  0
103.7104   95.6027   94.6567 C   0  0  0  0  0  0
105.2431   93.6748   95.1011 C   0  0  0  0  0  0
113.0975   92.9004  108.6610 H   0  0  0  0  0  0
109.0008   95.8924  101.2995 H   0  0  0  0  0  0
110.2215   96.9223  103.1434 H   0  0  0  0  0  0
103.1025   96.2745   97.0277 H   0  0  0  0  0  0
107.1593   94.7967   97.0264 H   0  0  0  0  0  0
105.7517   97.3294  102.7279 H   0  0  0  0  0  0
111.0693   93.7925  112.4553 H   0  0  0  0  0  0
110.8343   94.8716  111.0706 H   0  0  0  0  0  0
112.4913   94.4037  111.5653 H   0  0  0  0  0  0
105.2181   98.4417   99.9898 H   0  0  0  0  0  0
104.1754   98.5493  101.3896 H   0  0  0  0  0  0
107.6919   97.4907  100.3986 H   0  0  0  0  0  0
107.8569   98.2581  101.9480 H   0  0  0  0  0  0
104.2627   95.9269  101.4976 H   0  0  0  0  0  0
105.7759   95.1157  101.5114 H   0  0  0  0  0  0
110.3368   97.7558  105.2381 H   0  0  0  0  0  0
108.7480   97.1866  105.6371 H   0  0  0  0  0  0
103.2256   96.5925   99.4223 H   0  0  0  0  0  0
107.2554   95.1222   99.4437 H   0  0  0  0  0  0
111.0860   92.5374  106.5102 H   0  0  0  0  0  0
111.8360   95.7928  109.1937 H   0  0  0  0  0  0
110.0883   94.0880  104.8956 H   0  0  0  0  0  0
110.7771   97.3492  107.6239 H   0  0  0  0  0  0
104.3253  102.7649   98.1595 H   0  0  0  0  0  0
105.9138  102.9922   98.8551 H   0  0  0  0  0  0
104.4778  103.1662   99.8602 H   0  0  0  0  0  0
105.0725  100.5967   97.2654 H   0  0  0  0  0  0
105.6634   99.2811   98.2374 H   0  0  0  0  0  0
106.6567  100.7019   98.0150 H   0  0  0  0  0  0
102.8429  101.0855   98.9384 H   0  0  0  0  0  0
103.1731  100.7997  100.6320 H   0  0  0  0  0  0
103.3995   99.5259   99.4323 H   0  0  0  0  0  0
107.1692   95.6829   94.8964 H   0  0  0  0  0  0
106.0821   97.0399   94.6895 H   0  0  0  0  0  0
106.1456   95.7666   93.4830 H   0  0  0  0  0  0
102.8707   95.0581   95.0896 H   0  0  0  0  0  0
103.6999   95.3944   93.5886 H   0  0  0  0  0  0
103.5108   96.6691   94.7678 H   0  0  0  0  0  0
104.5032   93.1016   95.6619 H   0  0  0  0  0  0
106.2257   93.3302   95.4237 H   0  0  0  0  0  0
105.1374   93.3925   94.0566 H   0  0  0  0  0  0
1  4  1  0  0  0
1  5  2  0  0  0
1  6  2  0  0  0
1 19  1  0  0  0
2 11  1  0  0  0
2  8  2  0  0  0
2 10  1  0  0  0
3  9  1  0  0  0
3  7  1  0  0  0
```

```
  3 14  2  0  0  0
  4 17  1  0  0  0
  4 38  1  0  0  0
  7 32  1  0  0  0
  7 33  1  0  0  0
  7 34  1  0  0  0
  9 20  1  0  0  0
 10 21  1  0  0  0
 10 39  1  0  0  0
 11 23  1  0  0  0
 11 40  1  0  0  0
 12 13  1  0  0  0
 12 35  1  0  0  0
 12 36  1  0  0  0
 12 37  1  0  0  0
 13 16  2  0  0  0
 13 15  1  0  0  0
 15 26  2  0  0  0
 15 41  1  0  0  0
 16 27  1  0  0  0
 16 42  1  0  0  0
 17 28  2  0  0  0
 17 29  1  0  0  0
 18 21  1  0  0  0
 18 20  1  0  0  0
 18 22  1  0  0  0
 18 43  1  0  0  0
 19 44  1  0  0  0
 19 45  1  0  0  0
 19 46  1  0  0  0
 20 47  1  0  0  0
 20 48  1  0  0  0
 21 49  1  0  0  0
 21 50  1  0  0  0
 22 24  1  0  0  0
 22 51  1  0  0  0
 22 52  1  0  0  0
 23 25  1  0  0  0
 23 53  1  0  0  0
 23 54  1  0  0  0
 24 26  1  0  0  0
 24 27  2  0  0  0
 25 31  1  0  0  0
 25 30  2  0  0  0
 26 55  1  0  0  0
 27 56  1  0  0  0
 28 30  1  0  0  0
 28 57  1  0  0  0
 29 31  2  0  0  0
 29 58  1  0  0  0
 30 59  1  0  0  0
 31 60  1  0  0  0
 32 61  1  0  0  0
 32 62  1  0  0  0
 32 63  1  0  0  0
 33 64  1  0  0  0
 33 65  1  0  0  0
 33 66  1  0  0  0
 34 67  1  0  0  0
 34 68  1  0  0  0
 34 69  1  0  0  0
 35 70  1  0  0  0
 35 71  1  0  0  0
 35 72  1  0  0  0
 36 73  1  0  0  0
 36 74  1  0  0  0
 36 75  1  0  0  0
 37 76  1  0  0  0
 37 77  1  0  0  0
 37 78  1  0  0  0
M  END
```

```
> <s_m_entry_id>
4

> <s_m_entry_name>
A_2S.1

> <s_m_Source_Path>
C:\Users\julio\OneDrive - Universidad de Talca\Escritorio\Nueva carpeta

> <s_m_Source_File>
A_2S.mol2

> <i_m_Source_File_Index>
1

$$$$
A_3R
                    3D
 Structure written by MMmdl.
 72 73  0  0  1  0            999 V2000
  105.3753   95.3322   96.6388 C   0  0  0  0  0  0
  105.8590   94.2229   96.8714 O   0  0  0  0  0  0
  104.9893   95.7148   95.1778 C   0  0  0  0  0  0
  106.2498   95.6175   94.2883 C   0  0  0  0  0  0
  103.9256   94.7110   94.6624 C   0  0  0  0  0  0
  104.3911   97.1399   95.0565 C   0  0  0  0  0  0
  105.1215   96.3032   97.5653 O   0  0  0  0  0  0
  105.0286   96.0549   98.9717 C   0  0  0  0  0  0
  106.1600   96.5961   99.8967 C   0  0  2  0  0  0
  106.9789   97.7728   99.2939 C   0  0  0  0  0  0
  106.2229   99.0974   99.3088 C   0  0  0  0  0  0
  104.6010  100.6107   98.3107 C   0  0  0  0  0  0
  104.7850  101.5085   99.3753 C   0  0  0  0  0  0
  105.6856  101.1934  100.3976 C   0  0  0  0  0  0
  106.3909   99.9897  100.3704 C   0  0  0  0  0  0
  105.3346   99.4223   98.2813 C   0  0  0  0  0  0
  104.0083  102.8128   99.4390 C   0  0  0  0  0  0
  107.0128   95.4307  100.4622 C   0  0  0  0  0  0
  108.0984   95.8186  101.3891 N   0  0  0  0  0  0
  108.0000   96.0251  102.7389 C   0  0  0  0  0  0
  106.6048   95.7857  103.6410 S   0  0  0  0  0  0
  109.1896   96.4599  103.2594 N   0  0  0  0  0  0
  109.4116   96.9707  104.6281 C   0  0  0  0  0  0
  110.0386   96.0085  105.6432 C   0  0  0  0  0  0
  111.0952   95.6885  107.8075 C   0  0  0  0  0  0
  110.4999   96.5234  106.8594 C   0  0  0  0  0  0
  110.1729   94.6364  105.3955 C   0  0  0  0  0  0
  110.7679   93.7992  106.3406 C   0  0  0  0  0  0
  111.2427   94.3225  107.5515 C   0  0  0  0  0  0
  111.8628   93.4890  108.4934 N   0  0  0  0  0  0
  111.4558   94.3986  111.1678 C   0  0  0  0  0  0
  111.0544   92.9921  109.9853 S   0  0  0  0  0  0
  109.6150   92.9663  109.7108 O   0  0  0  0  0  0
  111.7422   91.7848  110.4568 O   0  0  0  0  0  0
  110.9029   92.4833  106.0566 F   0  0  0  0  0  0
  103.6085  100.9007   97.1916 C   0  0  0  0  0  0
  106.0263   95.8822   93.2547 H   0  0  0  0  0  0
  106.6461   94.6018   94.2859 H   0  0  0  0  0  0
  107.0491   96.2769   94.6303 H   0  0  0  0  0  0
  103.5959   94.9674   93.6545 H   0  0  0  0  0  0
  103.0331   94.7059   95.2912 H   0  0  0  0  0  0
  104.3053   93.6885   94.6314 H   0  0  0  0  0  0
  104.1180   97.3811   94.0264 H   0  0  0  0  0  0
  105.1096   97.8882   95.3766 H   0  0  0  0  0  0
  103.4899   97.2609   95.6637 H   0  0  0  0  0  0
  104.9027   94.9928   99.1419 H   0  0  0  0  0  0
  104.0889   96.5072   99.2893 H   0  0  0  0  0  0
  105.6529   97.0010  100.7749 H   0  0  0  0  0  0
  107.3004   97.5416   98.2787 H   0  0  0  0  0  0
  107.9062   97.9238   99.8476 H   0  0  0  0  0  0
  105.8318  101.8706  101.2272 H   0  0  0  0  0  0
```

```
 107.0704   99.7547  101.1751 H   0  0  0  0  0  0
 105.1982   98.7406   97.4585 H   0  0  0  0  0  0
 102.9362  102.6164   99.4759 H   0  0  0  0  0  0
 104.2189  103.4198   98.5584 H   0  0  0  0  0  0
 104.2665  103.3940  100.3225 H   0  0  0  0  0  0
 106.3737   94.6811  100.9338 H   0  0  0  0  0  0
 107.4552   94.9094   99.6153 H   0  0  0  0  0  0
 109.0117   95.9251  100.9733 H   0  0  0  0  0  0
 110.0083   96.3781  102.6730 H   0  0  0  0  0  0
 110.0986   97.8122  104.5311 H   0  0  0  0  0  0
 108.5046   97.3981  105.0565 H   0  0  0  0  0  0
 111.4554   96.1148  108.7319 H   0  0  0  0  0  0
 110.4007   97.5770  107.0784 H   0  0  0  0  0  0
 109.8193   94.2046  104.4702 H   0  0  0  0  0  0
 112.6856   92.9663  108.2226 H   0  0  0  0  0  0
 111.0790   94.1350  112.1553 H   0  0  0  0  0  0
 110.9566   95.3052  110.8322 H   0  0  0  0  0  0
 112.5350   94.5385  111.2005 H   0  0  0  0  0  0
 103.5944  100.1066   96.4395 H   0  0  0  0  0  0
 103.8556  101.8386   96.6885 H   0  0  0  0  0  0
 102.5959  100.9886   97.5950 H   0  0  0  0  0  0
 1  7  1  0  0  0
 1  3  1  0  0  0
 1  2  2  0  0  0
 3  4  1  0  0  0
 3  5  1  0  0  0
 3  6  1  0  0  0
 4 37  1  0  0  0
 4 38  1  0  0  0
 4 39  1  0  0  0
 5 40  1  0  0  0
 5 41  1  0  0  0
 5 42  1  0  0  0
 6 43  1  0  0  0
 6 44  1  0  0  0
 6 45  1  0  0  0
 7  8  1  0  0  0
 8  9  1  0  0  0
 8 46  1  0  0  0
 8 47  1  0  0  0
 9 18  1  0  0  0
 9 10  1  0  0  0
 9 48  1  0  0  0
10 11  1  0  0  0
10 49  1  0  0  0
10 50  1  0  0  0
11 15  2  0  0  0
11 16  1  0  0  0
12 13  1  0  0  0
12 16  2  0  0  0
12 36  1  0  0  0
13 14  2  0  0  0
13 17  1  0  0  0
14 15  1  0  0  0
14 51  1  0  0  0
15 52  1  0  0  0
16 53  1  0  0  0
17 54  1  0  0  0
17 55  1  0  0  0
17 56  1  0  0  0
18 19  1  0  0  0
18 57  1  0  0  0
18 58  1  0  0  0
19 20  1  0  0  0
19 59  1  0  0  0
20 22  1  0  0  0
20 21  2  0  0  0
22 23  1  0  0  0
22 60  1  0  0  0
23 24  1  0  0  0
23 61  1  0  0  0
```

```
 23 62  1  0  0  0
 24 26  2  0  0  0
 24 27  1  0  0  0
 25 26  1  0  0  0
 25 29  2  0  0  0
 25 63  1  0  0  0
 26 64  1  0  0  0
 27 28  2  0  0  0
 27 65  1  0  0  0
 28 29  1  0  0  0
 28 35  1  0  0  0
 29 30  1  0  0  0
 30 32  1  0  0  0
 30 66  1  0  0  0
 31 32  1  0  0  0
 31 67  1  0  0  0
 31 68  1  0  0  0
 31 69  1  0  0  0
 32 34  2  0  0  0
 32 33  2  0  0  0
 36 70  1  0  0  0
 36 71  1  0  0  0
 36 72  1  0  0  0
M  END
> <s_m_entry_id>
5

> <s_m_entry_name>
A_3R.1

> <s_m_Source_Path>
C:\Users\julio\OneDrive - Universidad de Talca\Escritorio\Nueva carpeta

> <s_m_Source_File>
A_3R.mol2

> <i_m_Source_File_Index>
1

$$$$
A_3S
                    3D
 Structure written by MMmdl.
 72 73  0  0  1  0            999 V2000
  105.6087  100.2380  100.0511 C   0  0  0  0  0  0
  105.9114  100.1853  101.2477 O   0  0  0  0  0  0
  104.5945  101.2860   99.5021 C   0  0  0  0  0  0
  103.6242  101.7621  100.6198 C   0  0  0  0  0  0
  105.3439  102.5285   98.9481 C   0  0  0  0  0  0
  103.7590  100.6349   98.3703 C   0  0  0  0  0  0
  106.1181   99.4176   99.0893 O   0  0  0  0  0  0
  107.0861   98.4213   99.4136 C   0  0  0  0  0  0
  106.4908   97.2318  100.2132 C   0  0  1  0  0  0
  105.1949   96.7441   99.5058 C   0  0  0  0  0  0
  105.3027   96.5655   97.9916 C   0  0  0  0  0  0
  104.4483   96.9837   95.7488 C   0  0  0  0  0  0
  105.4859   96.2006   95.2083 C   0  0  0  0  0  0
  106.4171   95.6067   96.0676 C   0  0  0  0  0  0
  106.3260   95.7853   97.4469 C   0  0  0  0  0  0
  104.3664   97.1565   97.1363 C   0  0  0  0  0  0
  105.6220   95.9795   93.7057 C   0  0  0  0  0  0
  107.5041   96.0677  100.4261 C   0  0  0  0  0  0
  108.6519   96.3666  101.2999 N   0  0  0  0  0  0
  108.6139   96.6038  102.6475 C   0  0  0  0  0  0
  107.3140   96.2390  103.6550 S   0  0  0  0  0  0
  109.7834   97.1767  103.0655 N   0  0  0  0  0  0
  110.0410   97.7282  104.4031 C   0  0  0  0  0  0
  110.5809   96.6796  105.3640 C   0  0  0  0  0  0
  110.2540   94.6791  106.6911 C   0  0  0  0  0  0
  109.7689   95.6234  105.7855 C   0  0  0  0  0  0
  111.8757   96.7936  105.8773 C   0  0  0  0  0  0
```

```
 112.3627   95.8502  106.7840 C   0  0  0  0  0  0
 111.5569   94.7808  107.1997 C   0  0  0  0  0  0
 112.0933   93.8540  108.1114 N   0  0  0  0  0  0
 111.3743   94.5524  110.8113 C   0  0  0  0  0  0
 111.2303   93.1772  109.5190 S   0  0  0  0  0  0
 109.8211   92.9964  109.1509 O   0  0  0  0  0  0
 112.0302   92.0383  109.9829 O   0  0  0  0  0  0
 113.6252   95.9857  107.2567 F   0  0  0  0  0  0
 103.4051   97.6510   94.8587 C   0  0  0  0  0  0
 102.9140  102.5011  100.2452 H   0  0  0  0  0  0
 104.1547  102.2308  101.4509 H   0  0  0  0  0  0
 103.0311  100.9427  101.0303 H   0  0  0  0  0  0
 104.6465  103.2984   98.6141 H   0  0  0  0  0  0
 105.9730  102.2911   98.0887 H   0  0  0  0  0  0
 105.9840  102.9886   99.7033 H   0  0  0  0  0  0
 103.0183  101.3297   97.9710 H   0  0  0  0  0  0
 103.2126   99.7563   98.7185 H   0  0  0  0  0  0
 104.3760  100.3187   97.5280 H   0  0  0  0  0  0
 107.9099   98.8720   99.9701 H   0  0  0  0  0  0
 107.5082   98.0485   98.4783 H   0  0  0  0  0  0
 106.1931   97.6173  101.1898 H   0  0  0  0  0  0
 104.3654   97.4146   99.7390 H   0  0  0  0  0  0
 104.8930   95.7777   99.9144 H   0  0  0  0  0  0
 107.2190   95.0000   95.6721 H   0  0  0  0  0  0
 107.0548   95.3131   98.0900 H   0  0  0  0  0  0
 103.5693   97.7544   97.5553 H   0  0  0  0  0  0
 105.7560   96.9291   93.1866 H   0  0  0  0  0  0
 104.7341   95.4885   93.3060 H   0  0  0  0  0  0
 106.4792   95.3512   93.4599 H   0  0  0  0  0  0
 107.0013   95.1623  100.7644 H   0  0  0  0  0  0
 107.9204   95.7922   99.4563 H   0  0  0  0  0  0
 109.5540   96.3991  100.8469 H   0  0  0  0  0  0
 110.4504   97.4309  102.3509 H   0  0  0  0  0  0
 110.7587   98.5462  104.3197 H   0  0  0  0  0  0
 109.1355   98.1498  104.8422 H   0  0  0  0  0  0
 109.5956   93.8791  106.9902 H   0  0  0  0  0  0
 108.7593   95.5284  105.4122 H   0  0  0  0  0  0
 112.5102   97.6139  105.5727 H   0  0  0  0  0  0
 112.9777   93.4320  107.8668 H   0  0  0  0  0  0
 110.9030   94.2177  111.7354 H   0  0  0  0  0  0
 110.8775   95.4531  110.4548 H   0  0  0  0  0  0
 112.4288   94.7605  110.9930 H   0  0  0  0  0  0
 102.6745   98.2183   95.4371 H   0  0  0  0  0  0
 102.8553   96.9054   94.2833 H   0  0  0  0  0  0
 103.8783   98.3450   94.1634 H   0  0  0  0  0  0
 1  7  1  0  0  0
 1  3  1  0  0  0
 1  2  2  0  0  0
 3  4  1  0  0  0
 3  5  1  0  0  0
 3  6  1  0  0  0
 4 37  1  0  0  0
 4 38  1  0  0  0
 4 39  1  0  0  0
 5 40  1  0  0  0
 5 41  1  0  0  0
 5 42  1  0  0  0
 6 43  1  0  0  0
 6 44  1  0  0  0
 6 45  1  0  0  0
 7  8  1  0  0  0
 8  9  1  0  0  0
 8 46  1  0  0  0
 8 47  1  0  0  0
 9 18  1  0  0  0
 9 10  1  0  0  0
 9 48  1  0  0  0
10 11  1  0  0  0
10 49  1  0  0  0
10 50  1  0  0  0
11 15  2  0  0  0
```

```
 11 16  1  0  0  0
 12 13  1  0  0  0
 12 16  2  0  0  0
 12 36  1  0  0  0
 13 14  2  0  0  0
 13 17  1  0  0  0
 14 15  1  0  0  0
 14 51  1  0  0  0
 15 52  1  0  0  0
 16 53  1  0  0  0
 17 54  1  0  0  0
 17 55  1  0  0  0
 17 56  1  0  0  0
 18 19  1  0  0  0
 18 57  1  0  0  0
 18 58  1  0  0  0
 19 20  1  0  0  0
 19 59  1  0  0  0
 20 22  1  0  0  0
 20 21  2  0  0  0
 22 23  1  0  0  0
 22 60  1  0  0  0
 23 24  1  0  0  0
 23 61  1  0  0  0
 23 62  1  0  0  0
 24 26  2  0  0  0
 24 27  1  0  0  0
 25 26  1  0  0  0
 25 29  2  0  0  0
 25 63  1  0  0  0
 26 64  1  0  0  0
 27 28  2  0  0  0
 27 65  1  0  0  0
 28 29  1  0  0  0
 28 35  1  0  0  0
 29 30  1  0  0  0
 30 32  1  0  0  0
 30 66  1  0  0  0
 31 32  1  0  0  0
 31 67  1  0  0  0
 31 68  1  0  0  0
 31 69  1  0  0  0
 32 34  2  0  0  0
 32 33  2  0  0  0
 36 70  1  0  0  0
 36 71  1  0  0  0
 36 72  1  0  0  0
M  END
> <s_m_entry_id>
6

> <s_m_entry_name>
A_3S.1

> <s_m_Source_Path>
C:\Users\julio\OneDrive - Universidad de Talca\Escritorio\Nueva carpeta

> <s_m_Source_File>
A_3S.mol2

> <i_m_Source_File_Index>
1

$$$$
A_4R
                    3D
 Structure written by MMmdl.
 78 79  0  0  1  0            999 V2000
  106.4063   96.0787   97.3672 C   0  0  0  0  0  0
  107.6336   95.9837   97.4370 O   0  0  0  0  0  0
  105.6026   95.2432   96.3264 C   0  0  0  0  0  0
```

106.1921	93.8137	96.2699	C	0	0	0	0	0	0
104.0899	95.1453	96.6662	C	0	0	0	0	0	0
105.7583	95.9052	94.9344	C	0	0	0	0	0	0
105.6460	96.8711	98.1627	O	0	0	0	0	0	0
106.2156	97.9128	98.9522	C	0	0	0	0	0	0
106.5521	97.5577	100.4273	C	0	0	2	0	0	0
106.7643	98.8808	101.2180	C	0	0	0	0	0	0
105.4716	99.6386	101.4934	C	0	0	0	0	0	0
103.4033	99.8489	102.7190	C	0	0	0	0	0	0
103.1142	101.0558	102.0669	C	0	0	0	0	0	0
104.0154	101.5219	101.0952	C	0	0	0	0	0	0
105.1849	100.8219	100.8137	C	0	0	0	0	0	0
104.5746	99.1503	102.4417	C	0	0	0	0	0	0
101.8263	101.8326	102.4429	C	0	0	0	0	0	0
100.5916	101.0112	102.0083	C	0	0	0	0	0	0
101.7312	103.2261	101.7679	C	0	0	0	0	0	0
101.7697	102.0693	103.9758	C	0	0	0	0	0	0
107.7132	96.5147	100.5350	C	0	0	0	0	0	0
108.6468	96.5942	101.6720	N	0	0	0	0	0	0
108.3720	96.3667	102.9897	C	0	0	0	0	0	0
106.9279	95.7484	103.5864	S	0	0	0	0	0	0
109.4254	96.7585	103.7661	N	0	0	0	0	0	0
109.3764	97.0137	105.2131	C	0	0	0	0	0	0
110.1420	96.0235	106.0781	C	0	0	0	0	0	0
111.4532	95.6113	108.0635	C	0	0	0	0	0	0
110.7528	96.4890	107.2437	C	0	0	0	0	0	0
110.2512	94.6658	105.7440	C	0	0	0	0	0	0
110.9504	93.7869	106.5759	C	0	0	0	0	0	0
111.5485	94.2580	107.7493	C	0	0	0	0	0	0
112.2508	93.3936	108.5975	N	0	0	0	0	0	0
113.1817	92.5391	111.1413	C	0	0	0	0	0	0
111.6265	93.0003	110.2014	S	0	0	0	0	0	0
111.0810	94.2296	110.7770	O	0	0	0	0	0	0
110.8052	91.7926	110.0565	O	0	0	0	0	0	0
111.0553	92.4833	106.2304	F	0	0	0	0	0	0
105.5900	93.1411	95.6564	H	0	0	0	0	0	0
106.2563	93.3923	97.2723	H	0	0	0	0	0	0
107.2016	93.8084	95.8570	H	0	0	0	0	0	0
103.5583	94.5137	95.9524	H	0	0	0	0	0	0
103.6064	96.1233	96.6441	H	0	0	0	0	0	0
103.9233	94.7247	97.6569	H	0	0	0	0	0	0
105.2496	95.3299	94.1592	H	0	0	0	0	0	0
106.8044	95.9842	94.6328	H	0	0	0	0	0	0
105.3364	96.9126	94.9169	H	0	0	0	0	0	0
105.4685	98.7051	98.9507	H	0	0	0	0	0	0
107.0916	98.3407	98.4595	H	0	0	0	0	0	0
105.6772	97.0668	100.8590	H	0	0	0	0	0	0
107.4562	99.5303	100.6830	H	0	0	0	0	0	0
107.2207	98.7139	102.1896	H	0	0	0	0	0	0
102.7300	99.4485	103.4620	H	0	0	0	0	0	0
103.8384	102.4375	100.5541	H	0	0	0	0	0	0
105.8697	101.2082	100.0716	H	0	0	0	0	0	0
104.7848	98.2302	102.9700	H	0	0	0	0	0	0
100.5482	100.0422	102.5083	H	0	0	0	0	0	0
100.5970	100.8249	100.9341	H	0	0	0	0	0	0
99.6614	101.5308	102.2433	H	0	0	0	0	0	0
102.5800	103.8589	102.0282	H	0	0	0	0	0	0
100.8330	103.7617	102.0772	H	0	0	0	0	0	0
101.6897	103.1498	100.6799	H	0	0	0	0	0	0
102.6556	102.5925	104.3340	H	0	0	0	0	0	0
101.6955	101.1379	104.5388	H	0	0	0	0	0	0
100.9047	102.6714	104.2581	H	0	0	0	0	0	0
107.3262	95.4971	100.4418	H	0	0	0	0	0	0
108.3694	96.6510	99.6751	H	0	0	0	0	0	0
109.6189	96.7122	101.4241	H	0	0	0	0	0	0
110.3130	96.9176	103.3119	H	0	0	0	0	0	0
109.8124	98.0023	105.3608	H	0	0	0	0	0	0
108.3598	97.0968	105.5964	H	0	0	0	0	0	0
111.9087	95.9827	108.9627	H	0	0	0	0	0	0
110.6856	97.5319	107.5249	H	0	0	0	0	0	0
109.7877	94.2824	104.8464	H	0	0	0	0	0	0

 112.5215   92.5546 108.1057 H   0 0 0 0 0 0
 112.9059   92.2859 112.1640 H   0 0 0 0 0 0
 113.8546   93.3922 111.1407 H   0 0 0 0 0 0
 113.6556   91.6916 110.6495 H   0 0 0 0 0 0
  1 7 1 0 0 0
  1 3 1 0 0 0
  1 2 2 0 0 0
  3 4 1 0 0 0
  3 5 1 0 0 0
  3 6 1 0 0 0
  4 39 1 0 0 0
  4 40 1 0 0 0
  4 41 1 0 0 0
  5 42 1 0 0 0
  5 43 1 0 0 0
  5 44 1 0 0 0
  6 45 1 0 0 0
  6 46 1 0 0 0
  6 47 1 0 0 0
  7 8 1 0 0 0
  8 9 1 0 0 0
  8 48 1 0 0 0
  8 49 1 0 0 0
  9 21 1 0 0 0
  9 10 1 0 0 0
  9 50 1 0 0 0
 10 11 1 0 0 0
 10 51 1 0 0 0
 10 52 1 0 0 0
 11 15 2 0 0 0
 11 16 1 0 0 0
 12 13 1 0 0 0
 12 16 2 0 0 0
 12 53 1 0 0 0
 13 14 2 0 0 0
 13 17 1 0 0 0
 14 15 1 0 0 0
 14 54 1 0 0 0
 15 55 1 0 0 0
 16 56 1 0 0 0
 17 18 1 0 0 0
 17 19 1 0 0 0
 17 20 1 0 0 0
 18 57 1 0 0 0
 18 58 1 0 0 0
 18 59 1 0 0 0
 19 60 1 0 0 0
 19 61 1 0 0 0
 19 62 1 0 0 0
 20 63 1 0 0 0
 20 64 1 0 0 0
 20 65 1 0 0 0
 21 22 1 0 0 0
 21 66 1 0 0 0
 21 67 1 0 0 0
 22 23 1 0 0 0
 22 68 1 0 0 0
 23 25 1 0 0 0
 23 24 2 0 0 0
 25 26 1 0 0 0
 25 69 1 0 0 0
 26 27 1 0 0 0
 26 70 1 0 0 0
 26 71 1 0 0 0
 27 29 2 0 0 0
 27 30 1 0 0 0
 28 29 1 0 0 0
 28 32 2 0 0 0
 28 72 1 0 0 0
 29 73 1 0 0 0
 30 31 2 0 0 0

```
 30 74  1  0  0  0
 31 32  1  0  0  0
 31 38  1  0  0  0
 32 33  1  0  0  0
 33 35  1  0  0  0
 33 75  1  0  0  0
 34 35  1  0  0  0
 34 76  1  0  0  0
 34 77  1  0  0  0
 34 78  1  0  0  0
 35 37  2  0  0  0
 35 36  2  0  0  0
M  END
> <s_m_entry_id>
7

> <s_m_entry_name>
A_4R.1

> <s_m_Source_Path>
C:\Users\julio\OneDrive - Universidad de Talca\Escritorio\Nueva carpeta

> <s_m_Source_File>
A_4R.mol2

> <i_m_Source_File_Index>
1

$$$$
A_4S
                    3D
 Structure written by MMmdl.
 78 79  0  0  1  0            999 V2000
  105.7981  100.3503   99.9082 C   0  0  0  0  0  0
  106.2563  100.4965  101.0455 O   0  0  0  0  0  0
  104.7392  101.3290   99.3284 C   0  0  0  0  0  0
  103.7111  101.7134  100.4220 C   0  0  0  0  0  0
  105.5005  102.5922   98.8860 C   0  0  0  0  0  0
  103.9716  100.7595   98.1028 C   0  0  0  0  0  0
  106.1500   99.3648   99.0554 O   0  0  0  0  0  0
  107.1614   98.4188   99.3738 C   0  0  0  0  0  0
  106.7608   97.3965  100.4705 C   0  0  1  0  0  0
  105.3354   96.7930  100.3402 C   0  0  0  0  0  0
  104.8864   96.4650   98.9241 C   0  0  0  0  0  0
  105.2319   95.3670   96.8091 C   0  0  0  0  0  0
  104.0802   95.9751   96.2893 C   0  0  0  0  0  0
  103.3240   96.8122   97.1214 C   0  0  0  0  0  0
  103.7228   97.0507   98.4295 C   0  0  0  0  0  0
  105.6309   95.6041   98.1191 C   0  0  0  0  0  0
  103.6867   95.7111   94.8208 C   0  0  0  0  0  0
  102.3867   96.4384   94.3839 C   0  0  0  0  0  0
  103.4535   94.1941   94.6257 C   0  0  0  0  0  0
  104.8272   96.2161   93.9043 C   0  0  0  0  0  0
  107.8457   96.3008  100.6071 C   0  0  0  0  0  0
  108.8686   96.5968  101.6169 N   0  0  0  0  0  0
  108.6792   96.5486  102.9698 C   0  0  0  0  0  0
  107.2836   95.9660  103.7063 S   0  0  0  0  0  0
  109.7604   97.0536  103.6379 N   0  0  0  0  0  0
  109.8313   97.3353  105.0826 C   0  0  0  0  0  0
  110.3400   96.2072  105.9835 C   0  0  0  0  0  0
  111.4032   95.5355  108.0571 C   0  0  0  0  0  0
  110.9068   96.5352  107.2203 C   0  0  0  0  0  0
  110.2776   94.8587  105.6064 C   0  0  0  0  0  0
  110.7584   93.8591  106.4513 C   0  0  0  0  0  0
  111.3309   94.1965  107.6812 C   0  0  0  0  0  0
  111.8560   93.2151  108.5264 N   0  0  0  0  0  0
  111.3192   94.0849  111.1870 C   0  0  0  0  0  0
  111.0248   92.6743  109.9801 S   0  0  0  0  0  0
  109.5967   92.5927  109.6571 O   0  0  0  0  0  0
  111.7571   91.4955  110.4569 O   0  0  0  0  0  0
  110.6841   92.5693  106.0528 F   0  0  0  0  0  0
```

```
 102.9680  102.4157  100.0422 H   0  0  0  0  0  0
 104.1857  102.1913  101.2799 H   0  0  0  0  0  0
 103.1685  100.8409  100.7901 H   0  0  0  0  0  0
 104.8140  103.3768   98.5666 H   0  0  0  0  0  0
 106.1782  102.3886   98.0573 H   0  0  0  0  0  0
 106.1100  102.9924   99.6965 H   0  0  0  0  0  0
 103.2738  101.4924   97.6985 H   0  0  0  0  0  0
 103.3859   99.8812   98.3684 H   0  0  0  0  0  0
 104.6400  100.4804   97.2870 H   0  0  0  0  0  0
 108.0753   98.9426   99.6574 H   0  0  0  0  0  0
 107.3943   97.8878   98.4505 H   0  0  0  0  0  0
 106.7362   97.9512  101.4093 H   0  0  0  0  0  0
 104.6309   97.5047  100.7736 H   0  0  0  0  0  0
 105.2326   95.8989  100.9583 H   0  0  0  0  0  0
 105.8312   94.7092   96.1965 H   0  0  0  0  0  0
 102.4249   97.2969   96.7747 H   0  0  0  0  0  0
 103.1311   97.7044   99.0529 H   0  0  0  0  0  0
 106.5238   95.1272   98.4929 H   0  0  0  0  0  0
 102.4803   97.5225   94.4619 H   0  0  0  0  0  0
 101.5305   96.1304   94.9853 H   0  0  0  0  0  0
 102.1360   96.2187   93.3446 H   0  0  0  0  0  0
 104.3767   93.6173   94.6955 H   0  0  0  0  0  0
 103.0090   93.9791   93.6569 H   0  0  0  0  0  0
 102.7669   93.8003   95.3760 H   0  0  0  0  0  0
 105.7381   95.6252   94.0076 H   0  0  0  0  0  0
 105.0837   97.2530   94.1278 H   0  0  0  0  0  0
 104.5448   96.1908   92.8567 H   0  0  0  0  0  0
 107.4152   95.3206  100.8193 H   0  0  0  0  0  0
 108.3542   96.1926   99.6492 H   0  0  0  0  0  0
 109.8171   96.6816  101.2784 H   0  0  0  0  0  0
 110.5806   97.2813  103.0955 H   0  0  0  0  0  0
 110.5158   98.1781  105.1923 H   0  0  0  0  0  0
 108.8751   97.6950  105.4613 H   0  0  0  0  0  0
 111.8548   95.7930  109.0014 H   0  0  0  0  0  0
 110.9718   97.5661  107.5375 H   0  0  0  0  0  0
 109.8561   94.5647  104.6580 H   0  0  0  0  0  0
 112.7685   92.8249  108.3280 H   0  0  0  0  0  0
 110.9595   93.7850  112.1695 H   0  0  0  0  0  0
 110.7546   94.9562  110.8573 H   0  0  0  0  0  0
 112.3842   94.3038  111.2293 H   0  0  0  0  0  0
  1  7  1  0  0  0
  1  3  1  0  0  0
  1  2  2  0  0  0
  3  4  1  0  0  0
  3  5  1  0  0  0
  3  6  1  0  0  0
  4 39  1  0  0  0
  4 40  1  0  0  0
  4 41  1  0  0  0
  5 42  1  0  0  0
  5 43  1  0  0  0
  5 44  1  0  0  0
  6 45  1  0  0  0
  6 46  1  0  0  0
  6 47  1  0  0  0
  7  8  1  0  0  0
  8  9  1  0  0  0
  8 48  1  0  0  0
  8 49  1  0  0  0
  9 21  1  0  0  0
  9 10  1  0  0  0
  9 50  1  0  0  0
 10 11  1  0  0  0
 10 51  1  0  0  0
 10 52  1  0  0  0
 11 15  2  0  0  0
 11 16  1  0  0  0
 12 13  1  0  0  0
 12 16  2  0  0  0
 12 53  1  0  0  0
 13 14  2  0  0  0
```

```
 13 17  1  0  0  0
 14 15  1  0  0  0
 14 54  1  0  0  0
 15 55  1  0  0  0
 16 56  1  0  0  0
 17 18  1  0  0  0
 17 19  1  0  0  0
 17 20  1  0  0  0
 18 57  1  0  0  0
 18 58  1  0  0  0
 18 59  1  0  0  0
 19 60  1  0  0  0
 19 61  1  0  0  0
 19 62  1  0  0  0
 20 63  1  0  0  0
 20 64  1  0  0  0
 20 65  1  0  0  0
 21 22  1  0  0  0
 21 66  1  0  0  0
 21 67  1  0  0  0
 22 23  1  0  0  0
 22 68  1  0  0  0
 23 25  1  0  0  0
 23 24  2  0  0  0
 25 26  1  0  0  0
 25 69  1  0  0  0
 26 27  1  0  0  0
 26 70  1  0  0  0
 26 71  1  0  0  0
 27 29  2  0  0  0
 27 30  1  0  0  0
 28 29  1  0  0  0
 28 32  2  0  0  0
 28 72  1  0  0  0
 29 73  1  0  0  0
 30 31  2  0  0  0
 30 74  1  0  0  0
 31 32  1  0  0  0
 31 38  1  0  0  0
 32 33  1  0  0  0
 33 35  1  0  0  0
 33 75  1  0  0  0
 34 35  1  0  0  0
 34 76  1  0  0  0
 34 77  1  0  0  0
 34 78  1  0  0  0
 35 37  2  0  0  0
 35 36  2  0  0  0
M  END
> <s_m_entry_id>
8

> <s_m_entry_name>
A_4S.1

> <s_m_Source_Path>
C:\Users\julio\OneDrive - Universidad de Talca\Escritorio\Nueva carpeta

> <s_m_Source_File>
A_4S.mol2

> <i_m_Source_File_Index>
1

$$$$
A_5R
                   3D
 Structure written by MMmdl.
 69 70  0  0  1  0            999 V2000
  109.7449   92.8901  109.7027 O   0  0  0  0  0  0
  111.1627   92.9639  110.0687 S   0  0  0  0  0  0
```

```
111.8570   91.7871  110.6004 O    0   0   0   0   0   0
107.7141   96.3329  101.7692 N    0   0   0   0   0   0
108.1647   95.8842  102.9824 C    0   0   0   0   0   0
109.4452   96.2700  103.2575 N    0   0   0   0   0   0
107.2416   94.9929  104.0573 S    0   0   0   0   0   0
109.8711   96.8362  104.5532 C    0   0   0   0   0   0
110.4215   95.8925  105.6297 C    0   0   0   0   0   0
110.5170   94.5060  105.4482 C    0   0   0   0   0   0
111.0309   93.6982  106.4610 C    0   0   0   0   0   0
111.4761   94.2716  107.6500 C    0   0   0   0   0   0
111.3885   95.6505  107.8360 C    0   0   0   0   0   0
110.8556   96.4561  106.8332 C    0   0   0   0   0   0
112.0473   93.4579  108.6310 N    0   0   0   0   0   0
111.4597   94.4069  111.2360 C    0   0   0   0   0   0
106.5469   99.6345   99.2963 C    0   0   0   0   0   0
106.8308   98.5442  100.0732 O    0   0   0   0   0   0
106.0209   98.0490  101.1329 C    0   0   0   0   0   0
106.2888   96.5648  101.4417 C    0   0   1   0   0   0
105.6966   95.5295  100.4456 C    0   0   0   0   0   0
105.7764   95.8523   98.9550 C    0   0   0   0   0   0
106.7468   95.2602   98.1467 C    0   0   0   0   0   0
106.7874   95.5317   96.7792 C    0   0   0   0   0   0
105.8580   96.4052   96.1991 C    0   0   0   0   0   0
104.8676   96.9924   97.0064 C    0   0   0   0   0   0
104.8313   96.7007   98.3730 C    0   0   0   0   0   0
103.8368   97.9602   96.4439 C    0   0   0   0   0   0
105.9392   96.6963   94.7057 C    0   0   0   0   0   0
107.0598   99.7298   98.1812 O    0   0   0   0   0   0
105.5984  100.7759   99.8244 C    0   0   0   0   0   0
104.1583  100.5058   99.3280 C    0   0   0   0   0   0
105.5860  100.9623  101.3652 C    0   0   0   0   0   0
106.0529  102.1399   99.2211 C    0   0   0   0   0   0
108.3911   96.6609  101.0946 H    0   0   0   0   0   0
110.1009   96.3240  102.4888 H    0   0   0   0   0   0
110.6665   97.5490  104.3341 H    0   0   0   0   0   0
109.0606   97.4264  104.9861 H    0   0   0   0   0   0
110.1934   94.0445  104.5267 H    0   0   0   0   0   0
111.0983   92.6306  106.3087 H    0   0   0   0   0   0
111.7327   96.1029  108.7525 H    0   0   0   0   0   0
110.7928   97.5231  106.9929 H    0   0   0   0   0   0
112.9339   93.0086  108.4459 H    0   0   0   0   0   0
111.0561   94.1515  112.2145 H    0   0   0   0   0   0
110.9469   95.2860  110.8508 H    0   0   0   0   0   0
112.5302   94.5893  111.3124 H    0   0   0   0   0   0
104.9588   98.1935  100.9339 H    0   0   0   0   0   0
106.2633   98.6072  102.0365 H    0   0   0   0   0   0
105.6987   96.4086  102.3447 H    0   0   0   0   0   0
104.6397   95.3935  100.6829 H    0   0   0   0   0   0
106.1316   94.5485  100.6348 H    0   0   0   0   0   0
107.4556   94.5703   98.5765 H    0   0   0   0   0   0
107.5488   95.0600   96.1749 H    0   0   0   0   0   0
104.0671   97.1459   98.9923 H    0   0   0   0   0   0
102.9219   97.9511   97.0356 H    0   0   0   0   0   0
103.5596   97.7181   95.4189 H    0   0   0   0   0   0
104.2396   98.9711   96.4640 H    0   0   0   0   0   0
105.9265   97.7724   94.5162 H    0   0   0   0   0   0
105.0910   96.2512   94.1875 H    0   0   0   0   0   0
106.8467   96.2876   94.2616 H    0   0   0   0   0   0
103.4735  101.2909   99.6508 H    0   0   0   0   0   0
103.7555   99.5639   99.6984 H    0   0   0   0   0   0
104.1064  100.4746   98.2403 H    0   0   0   0   0   0
105.1834  101.9321  101.6408 H    0   0   0   0   0   0
106.5828  100.8961  101.8044 H    0   0   0   0   0   0
104.9303  100.2584  101.8741 H    0   0   0   0   0   0
105.3702  102.9527   99.4859 H    0   0   0   0   0   0
106.0844  102.1211   98.1321 H    0   0   0   0   0   0
107.0456  102.4263   99.5684 H    0   0   0   0   0   0
1  2  2  0  0  0
2  3  2  0  0  0
2 15  1  0  0  0
2 16  1  0  0  0
```

```
  4 20  1  0  0  0
  4  5  1  0  0  0
  4 35  1  0  0  0
  5  6  1  0  0  0
  5  7  2  0  0  0
  6  8  1  0  0  0
  6 36  1  0  0  0
  8  9  1  0  0  0
  8 37  1  0  0  0
  8 38  1  0  0  0
  9 10  2  0  0  0
  9 14  1  0  0  0
 10 11  1  0  0  0
 10 39  1  0  0  0
 11 12  2  0  0  0
 11 40  1  0  0  0
 12 13  1  0  0  0
 12 15  1  0  0  0
 13 14  2  0  0  0
 13 41  1  0  0  0
 14 42  1  0  0  0
 15 43  1  0  0  0
 16 44  1  0  0  0
 16 45  1  0  0  0
 16 46  1  0  0  0
 17 18  1  0  0  0
 17 30  2  0  0  0
 17 31  1  0  0  0
 18 19  1  0  0  0
 19 20  1  0  0  0
 19 47  1  0  0  0
 19 48  1  0  0  0
 20 21  1  0  0  0
 20 49  1  0  0  0
 21 22  1  0  0  0
 21 50  1  0  0  0
 21 51  1  0  0  0
 22 23  2  0  0  0
 22 27  1  0  0  0
 23 24  1  0  0  0
 23 52  1  0  0  0
 24 25  2  0  0  0
 24 53  1  0  0  0
 25 26  1  0  0  0
 25 29  1  0  0  0
 26 27  2  0  0  0
 26 28  1  0  0  0
 27 54  1  0  0  0
 28 55  1  0  0  0
 28 56  1  0  0  0
 28 57  1  0  0  0
 29 58  1  0  0  0
 29 59  1  0  0  0
 29 60  1  0  0  0
 31 32  1  0  0  0
 31 33  1  0  0  0
 31 34  1  0  0  0
 32 61  1  0  0  0
 32 62  1  0  0  0
 32 63  1  0  0  0
 33 64  1  0  0  0
 33 65  1  0  0  0
 33 66  1  0  0  0
 34 67  1  0  0  0
 34 68  1  0  0  0
 34 69  1  0  0  0
M  END
> <s_m_entry_id>
9

> <s_m_entry_name>
```

```
A_5R.1

> <s_m_Source_Path>
C:\Users\julio\OneDrive - Universidad de Talca\Escritorio\Nueva carpeta

> <s_m_Source_File>
A_5R.mol2

> <i_m_Source_File_Index>
1

$$$$
A_5S
                    3D
 Structure written by MMmdl.
 69 70  0  0  1  0            999 V2000
  109.4600   92.9599  109.6715 O   0  0  0  0  0  0
  110.9105   92.9805  109.8907 S   0  0  0  0  0  0
  111.5907   91.7938  110.4267 O   0  0  0  0  0  0
  107.7010   95.9595  101.2755 N   0  0  0  0  0  0
  107.8032   95.8992  102.6374 C   0  0  0  0  0  0
  109.0281   96.3252  103.0728 N   0  0  0  0  0  0
  106.5712   95.3936  103.6554 S   0  0  0  0  0  0
  109.3327   96.8310  104.4249 C   0  0  0  0  0  0
  109.9420   95.8608  105.4470 C   0  0  0  0  0  0
  110.5518   96.4051  106.5819 C   0  0  0  0  0  0
  111.1255   95.5759  107.5443 C   0  0  0  0  0  0
  111.0759   94.1903  107.3875 C   0  0  0  0  0  0
  110.4713   93.6392  106.2568 C   0  0  0  0  0  0
  109.9144   94.4703  105.2835 C   0  0  0  0  0  0
  111.6692   93.3490  108.3354 N   0  0  0  0  0  0
  111.3975   94.4663  110.9418 C   0  0  0  0  0  0
  106.3126   94.9226   96.7505 C   0  0  0  0  0  0
  106.0029   95.1964   98.0444 O   0  0  0  0  0  0
  107.0274   95.2108   99.0318 C   0  0  0  0  0  0
  106.5330   95.5644  100.4553 C   0  0  2  0  0  0
  105.3662   96.5943  100.4858 C   0  0  0  0  0  0
  105.7084   98.0400  100.1426 C   0  0  0  0  0  0
  105.7081   98.4689   98.8123 C   0  0  0  0  0  0
  105.8875   99.8176   98.5101 C   0  0  0  0  0  0
  106.0634  100.7544   99.5299 C   0  0  0  0  0  0
  106.0878  100.3287  100.8681 C   0  0  0  0  0  0
  105.9244   98.9721  101.1635 C   0  0  0  0  0  0
  106.2403  101.3260  102.0031 C   0  0  0  0  0  0
  106.1782  102.2272   99.1644 C   0  0  0  0  0  0
  107.4394   94.6084   96.3580 O   0  0  0  0  0  0
  105.1273   95.0495   95.7498 C   0  0  0  0  0  0
  103.7840   95.4153   96.4388 C   0  0  0  0  0  0
  105.4704   96.1558   94.7195 C   0  0  0  0  0  0
  104.9420   93.7045   94.9993 C   0  0  0  0  0  0
  108.5130   96.2615  100.7628 H   0  0  0  0  0  0
  109.7982   96.2859  102.4182 H   0  0  0  0  0  0
  110.0598   97.6344  104.2840 H   0  0  0  0  0  0
  108.4602   97.3125  104.8700 H   0  0  0  0  0  0
  110.5885   97.4735  106.7245 H   0  0  0  0  0  0
  111.6093   96.0147  108.4045 H   0  0  0  0  0  0
  110.4367   92.5675  106.1265 H   0  0  0  0  0  0
  109.4557   94.0231  104.4124 H   0  0  0  0  0  0
  112.5612   92.9241  108.1267 H   0  0  0  0  0  0
  111.0560   94.2945  111.9628 H   0  0  0  0  0  0
  110.9224   95.3670  110.5473 H   0  0  0  0  0  0
  112.4826   94.5714  110.9277 H   0  0  0  0  0  0
  107.8069   95.9062   98.7174 H   0  0  0  0  0  0
  107.4559   94.2093   99.0557 H   0  0  0  0  0  0
  106.1456   94.6218  100.8505 H   0  0  0  0  0  0
  104.9092   96.5900  101.4754 H   0  0  0  0  0  0
  104.5548   96.2630   99.8359 H   0  0  0  0  0  0
  105.5269   97.7680   98.0121 H   0  0  0  0  0  0
  105.8501  100.1439   97.4837 H   0  0  0  0  0  0
  105.9118   98.6446  102.1938 H   0  0  0  0  0  0
  106.1621  100.8508  102.9793 H   0  0  0  0  0  0
```

```
 105.4484  102.0656  101.9243 H   0  0  0  0  0  0
 107.1960  101.8447  101.9463 H   0  0  0  0  0  0
 107.0540  102.6765   99.6292 H   0  0  0  0  0  0
 105.2933  102.7637   99.5064 H   0  0  0  0  0  0
 106.2546  102.3766   98.0858 H   0  0  0  0  0  0
 102.9734   95.4970   95.7134 H   0  0  0  0  0  0
 103.8365   96.3726   96.9575 H   0  0  0  0  0  0
 103.4816   94.6626   97.1689 H   0  0  0  0  0  0
 104.6785   96.2652   93.9753 H   0  0  0  0  0  0
 106.3889   95.9427   94.1692 H   0  0  0  0  0  0
 105.5971   97.1282   95.1973 H   0  0  0  0  0  0
 104.1552   93.7747   94.2485 H   0  0  0  0  0  0
 104.6645   92.8937   95.6792 H   0  0  0  0  0  0
 105.8511   93.3982   94.4763 H   0  0  0  0  0  0
 1  2  2  0  0  0
 2  3  2  0  0  0
 2 15  1  0  0  0
 2 16  1  0  0  0
 4 20  1  0  0  0
 4  5  1  0  0  0
 4 35  1  0  0  0
 5  6  1  0  0  0
 5  7  2  0  0  0
 6  8  1  0  0  0
 6 36  1  0  0  0
 8  9  1  0  0  0
 8 37  1  0  0  0
 8 38  1  0  0  0
 9 10  2  0  0  0
 9 14  1  0  0  0
10 11  1  0  0  0
10 39  1  0  0  0
11 12  2  0  0  0
11 40  1  0  0  0
12 13  1  0  0  0
12 15  1  0  0  0
13 14  2  0  0  0
13 41  1  0  0  0
14 42  1  0  0  0
15 43  1  0  0  0
16 44  1  0  0  0
16 45  1  0  0  0
16 46  1  0  0  0
17 18  1  0  0  0
17 30  2  0  0  0
17 31  1  0  0  0
18 19  1  0  0  0
19 20  1  0  0  0
19 47  1  0  0  0
19 48  1  0  0  0
20 21  1  0  0  0
20 49  1  0  0  0
21 22  1  0  0  0
21 50  1  0  0  0
21 51  1  0  0  0
22 23  2  0  0  0
22 27  1  0  0  0
23 24  1  0  0  0
23 52  1  0  0  0
24 25  2  0  0  0
24 53  1  0  0  0
25 26  1  0  0  0
25 29  1  0  0  0
26 27  2  0  0  0
26 28  1  0  0  0
27 54  1  0  0  0
28 55  1  0  0  0
28 56  1  0  0  0
28 57  1  0  0  0
29 58  1  0  0  0
29 59  1  0  0  0
```

```
 29 60  1  0  0  0
 31 32  1  0  0  0
 31 33  1  0  0  0
 31 34  1  0  0  0
 32 61  1  0  0  0
 32 62  1  0  0  0
 32 63  1  0  0  0
 33 64  1  0  0  0
 33 65  1  0  0  0
 33 66  1  0  0  0
 34 67  1  0  0  0
 34 68  1  0  0  0
 34 69  1  0  0  0
M  END
> <s_m_entry_id>
10

> <s_m_entry_name>
A_5S.1

> <s_m_Source_Path>
C:\Users\julio\OneDrive - Universidad de Talca\Escritorio\Nueva carpeta

> <s_m_Source_File>
A_5S.mol2

> <i_m_Source_File_Index>
1

$$$$
A_6R
                    3D
 Structure written by MMmdl.
 75 76  0  0  1  0            999 V2000
  109.6752   92.8579  109.7137 O   0  0  0  0  0  0
  111.1106   92.9527  109.9956 S   0  0  0  0  0  0
  111.8534   91.7783  110.4637 O   0  0  0  0  0  0
  107.4556   97.5788  102.4327 N   0  0  0  0  0  0
  108.0247   96.4110  102.8576 C   0  0  0  0  0  0
  109.2338   96.5565  103.4555 N   0  0  0  0  0  0
  107.4537   94.8719  102.5666 S   0  0  0  0  0  0
  109.3631   97.0600  104.8271 C   0  0  0  0  0  0
  110.0226   96.0854  105.8081 C   0  0  0  0  0  0
  110.6727   96.5991  106.9343 C   0  0  0  0  0  0
  111.3004   95.7416  107.8354 C   0  0  0  0  0  0
  111.2759   94.3639  107.6191 C   0  0  0  0  0  0
  110.6377   93.8427  106.4948 C   0  0  0  0  0  0
  110.0119   94.6995  105.5921 C   0  0  0  0  0  0
  111.9021   93.4966  108.5162 N   0  0  0  0  0  0
  111.4435   94.3698  111.1838 C   0  0  0  0  0  0
  103.5324   97.7247  102.3735 C   0  0  0  0  0  0
  104.5940   98.5612  102.5445 O   0  0  0  0  0  0
  105.5079   98.8082  101.4748 C   0  0  0  0  0  0
  106.5508   97.6861  101.2720 C   0  0  1  0  0  0
  107.3540   97.8851   99.9560 C   0  0  0  0  0  0
  106.6928   97.2587   98.7310 C   0  0  0  0  0  0
  105.7484   97.9684   97.9911 C   0  0  0  0  0  0
  105.1755   97.3939   96.8581 C   0  0  0  0  0  0
  105.5313   96.1026   96.4385 C   0  0  0  0  0  0
  106.4797   95.3985   97.1964 C   0  0  0  0  0  0
  107.0505   95.9719   98.3294 C   0  0  0  0  0  0
  104.9113   95.5156   95.1418 C   0  0  0  0  0  0
  103.3548   97.0174  101.3765 O   0  0  0  0  0  0
  102.5124   97.7646  103.5507 C   0  0  0  0  0  0
  101.3456   96.7696  103.3059 C   0  0  0  0  0  0
  103.2019   97.3867  104.8840 C   0  0  0  0  0  0
  101.9268   99.1952  103.6713 C   0  0  0  0  0  0
  105.4959   96.2706   93.9236 C   0  0  0  0  0  0
  103.3661   95.6745   95.1449 C   0  0  0  0  0  0
  105.1948   93.9997   94.9642 C   0  0  0  0  0  0
  107.8130   98.4440  102.8108 H   0  0  0  0  0  0
```

```
 110.0401   96.1210  103.0268 H   0  0  0  0  0  0
 109.9662   97.9683  104.7817 H   0  0  0  0  0  0
 108.4000   97.3682  105.2398 H   0  0  0  0  0  0
 110.6927   97.6645  107.1164 H   0  0  0  0  0  0
 111.8049   96.1500  108.6983 H   0  0  0  0  0  0
 110.6226   92.7762  106.3225 H   0  0  0  0  0  0
 109.5149   94.2746  104.7327 H   0  0  0  0  0  0
 112.8107   93.1151  108.2919 H   0  0  0  0  0  0
 111.0574   94.0942  112.1639 H   0  0  0  0  0  0
 110.9251   95.2608  110.8339 H   0  0  0  0  0  0
 112.5162   94.5438  111.2399 H   0  0  0  0  0  0
 106.0219   99.7446  101.6821 H   0  0  0  0  0  0
 104.9417   98.9715  100.5579 H   0  0  0  0  0  0
 105.9788   96.7632  101.1658 H   0  0  0  0  0  0
 108.3463   97.4392  100.0455 H   0  0  0  0  0  0
 107.5493   98.9430   99.7688 H   0  0  0  0  0  0
 105.4661   98.9688   98.2831 H   0  0  0  0  0  0
 104.4623   97.9763   96.2958 H   0  0  0  0  0  0
 106.8006   94.4042   96.9259 H   0  0  0  0  0  0
 107.7890   95.4196   98.8873 H   0  0  0  0  0  0
 100.6059   96.8142  104.1074 H   0  0  0  0  0  0
 101.7000   95.7380  103.2468 H   0  0  0  0  0  0
 100.8152   96.9832  102.3765 H   0  0  0  0  0  0
 102.4703   97.2374  105.6812 H   0  0  0  0  0  0
 103.8610   98.1834  105.2287 H   0  0  0  0  0  0
 103.7879   96.4667  104.8089 H   0  0  0  0  0  0
 101.1591   99.2475  104.4483 H   0  0  0  0  0  0
 101.4625   99.5229  102.7387 H   0  0  0  0  0  0
 102.6935   99.9295  103.9318 H   0  0  0  0  0  0
 106.5793   96.1586   93.8668 H   0  0  0  0  0  0
 105.2812   97.3389   93.9669 H   0  0  0  0  0  0
 105.0841   95.9042   92.9827 H   0  0  0  0  0  0
 102.9170   95.1992   96.0201 H   0  0  0  0  0  0
 102.9135   95.2180   94.2616 H   0  0  0  0  0  0
 103.0511   96.7155   95.1409 H   0  0  0  0  0  0
 104.8424   93.4174   95.8137 H   0  0  0  0  0  0
 106.2550   93.7968   94.8477 H   0  0  0  0  0  0
 104.7020   93.5988   94.0776 H   0  0  0  0  0  0
  1  2  2  0  0  0
  2 15  1  0  0  0
  2 16  1  0  0  0
  2  3  2  0  0  0
  4 20  1  0  0  0
  4  5  1  0  0  0
  4 37  1  0  0  0
  5  6  1  0  0  0
  5  7  2  0  0  0
  6  8  1  0  0  0
  6 38  1  0  0  0
  8  9  1  0  0  0
  8 39  1  0  0  0
  8 40  1  0  0  0
  9 10  2  0  0  0
  9 14  1  0  0  0
 10 11  1  0  0  0
 10 41  1  0  0  0
 11 12  2  0  0  0
 11 42  1  0  0  0
 12 13  1  0  0  0
 12 15  1  0  0  0
 13 14  2  0  0  0
 13 43  1  0  0  0
 14 44  1  0  0  0
 15 45  1  0  0  0
 16 46  1  0  0  0
 16 47  1  0  0  0
 16 48  1  0  0  0
 17 29  2  0  0  0
 17 30  1  0  0  0
 17 18  1  0  0  0
 18 19  1  0  0  0
```

```
 19 20  1  0  0  0
 19 49  1  0  0  0
 19 50  1  0  0  0
 20 21  1  0  0  0
 20 51  1  0  0  0
 21 22  1  0  0  0
 21 52  1  0  0  0
 21 53  1  0  0  0
 22 23  2  0  0  0
 22 27  1  0  0  0
 23 24  1  0  0  0
 23 54  1  0  0  0
 24 25  2  0  0  0
 24 55  1  0  0  0
 25 26  1  0  0  0
 25 28  1  0  0  0
 26 27  2  0  0  0
 26 56  1  0  0  0
 27 57  1  0  0  0
 28 34  1  0  0  0
 28 35  1  0  0  0
 28 36  1  0  0  0
 30 31  1  0  0  0
 30 32  1  0  0  0
 30 33  1  0  0  0
 31 58  1  0  0  0
 31 59  1  0  0  0
 31 60  1  0  0  0
 32 61  1  0  0  0
 32 62  1  0  0  0
 32 63  1  0  0  0
 33 64  1  0  0  0
 33 65  1  0  0  0
 33 66  1  0  0  0
 34 67  1  0  0  0
 34 68  1  0  0  0
 34 69  1  0  0  0
 35 70  1  0  0  0
 35 71  1  0  0  0
 35 72  1  0  0  0
 36 73  1  0  0  0
 36 74  1  0  0  0
 36 75  1  0  0  0
M  END
> <s_m_entry_id>
11

> <s_m_entry_name>
A_6R.1

> <s_m_Source_Path>
C:\Users\julio\OneDrive - Universidad de Talca\Escritorio\Nueva carpeta

> <s_m_Source_File>
A_6R.mol2

> <i_m_Source_File_Index>
1

$$$$
A_6S
                    3D
 Structure written by MMmdl.
 75 76  0  0  1  0            999 V2000
  109.7322   92.8868  109.6885 O   0  0  0  0  0  0
  111.1522   92.9539  110.0422 S   0  0  0  0  0  0
  111.8561   91.7644  110.5300 O   0  0  0  0  0  0
  107.9161   96.4448  101.6168 N   0  0  0  0  0  0
  108.2986   95.8954  102.8142 C   0  0  0  0  0  0
  109.4588   96.4338  103.2924 N   0  0  0  0  0  0
  107.4559   94.7152  103.6411 S   0  0  0  0  0  0
```

109.6286	96.9060	104.6877	C	0	0	0	0	0	0
110.2859	95.9650	105.7046	C	0	0	0	0	0	0
110.4618	94.5961	105.4736	C	0	0	0	0	0	0
111.0111	93.7783	106.4567	C	0	0	0	0	0	0
111.4359	94.3301	107.6614	C	0	0	0	0	0	0
111.3064	95.6983	107.8893	C	0	0	0	0	0	0
110.7255	96.5119	106.9164	C	0	0	0	0	0	0
112.0241	93.4982	108.6129	N	0	0	0	0	0	0
111.4555	94.3674	111.2385	C	0	0	0	0	0	0
105.9418	95.3929	97.2933	C	0	0	0	0	0	0
105.8666	95.8967	98.5477	O	0	0	0	0	0	0
107.0006	95.8877	99.4052	C	0	0	0	0	0	0
106.6566	96.1887	100.8820	C	0	0	2	0	0	0
105.6606	97.3707	101.0694	C	0	0	0	0	0	0
104.5454	97.0485	102.0561	C	0	0	0	0	0	0
103.3918	96.3973	101.6149	C	0	0	0	0	0	0
102.3896	96.0615	102.5245	C	0	0	0	0	0	0
102.5316	96.3477	103.8895	C	0	0	0	0	0	0
103.6894	97.0214	104.3109	C	0	0	0	0	0	0
104.6840	97.3742	103.4016	C	0	0	0	0	0	0
101.4170	95.9234	104.8812	C	0	0	0	0	0	0
106.9063	94.7628	96.8474	O	0	0	0	0	0	0
104.6748	95.6799	96.4331	C	0	0	0	0	0	0
103.3712	95.5859	97.2698	C	0	0	0	0	0	0
104.7944	97.1167	95.8677	C	0	0	0	0	0	0
104.5783	94.6854	95.2458	C	0	0	0	0	0	0
101.8163	96.1051	106.3711	C	0	0	0	0	0	0
101.0722	94.4226	104.6981	C	0	0	0	0	0	0
100.1499	96.7719	104.6214	C	0	0	0	0	0	0
108.5306	97.1316	101.2030	H	0	0	0	0	0	0
110.2104	96.6098	102.6413	H	0	0	0	0	0	0
110.2614	97.7908	104.6280	H	0	0	0	0	0	0
108.6740	97.2559	105.0889	H	0	0	0	0	0	0
110.1687	94.1420	104.5325	H	0	0	0	0	0	0
111.1239	92.7188	106.2712	H	0	0	0	0	0	0
111.6568	96.1314	108.8136	H	0	0	0	0	0	0
110.6260	97.5679	107.1149	H	0	0	0	0	0	0
112.8901	93.0285	108.3856	H	0	0	0	0	0	0
111.0499	94.0979	112.2147	H	0	0	0	0	0	0
110.9455	95.2590	110.8681	H	0	0	0	0	0	0
112.5290	94.5379	111.3197	H	0	0	0	0	0	0
107.7371	96.5964	99.0246	H	0	0	0	0	0	0
107.4556	94.8894	99.3652	H	0	0	0	0	0	0
106.1759	95.2737	101.2334	H	0	0	0	0	0	0
105.1804	97.6604	100.1320	H	0	0	0	0	0	0
106.1731	98.2839	101.3814	H	0	0	0	0	0	0
103.2751	96.1443	100.5712	H	0	0	0	0	0	0
101.5087	95.5665	102.1516	H	0	0	0	0	0	0
103.8545	97.2779	105.3489	H	0	0	0	0	0	0
105.5718	97.8789	103.7543	H	0	0	0	0	0	0
102.4958	95.7915	96.6554	H	0	0	0	0	0	0
103.3536	96.3080	98.0872	H	0	0	0	0	0	0
103.2332	94.5931	97.7044	H	0	0	0	0	0	0
103.9607	97.3519	95.2087	H	0	0	0	0	0	0
105.7110	97.2588	95.2876	H	0	0	0	0	0	0
104.7954	97.8648	96.6636	H	0	0	0	0	0	0
103.6998	94.8773	94.6333	H	0	0	0	0	0	0
104.5131	93.6507	95.5890	H	0	0	0	0	0	0
105.4458	94.7529	94.5853	H	0	0	0	0	0	0
102.0091	97.1487	106.6260	H	0	0	0	0	0	0
102.7098	95.5283	106.6160	H	0	0	0	0	0	0
101.0282	95.7622	107.0441	H	0	0	0	0	0	0
100.6800	94.2025	103.7061	H	0	0	0	0	0	0
100.3124	94.0934	105.4116	H	0	0	0	0	0	0
101.9514	93.7978	104.8531	H	0	0	0	0	0	0
99.7617	96.6273	103.6128	H	0	0	0	0	0	0
100.3504	97.8381	104.7403	H	0	0	0	0	0	0
99.3438	96.5162	105.3119	H	0	0	0	0	0	0

1	2	2	0	0	0
2	15	1	0	0	0
2	16	1	0	0	0

2	3	2	0	0	0
4	20	1	0	0	0
4	5	1	0	0	0
4	37	1	0	0	0
5	6	1	0	0	0
5	7	2	0	0	0
6	8	1	0	0	0
6	38	1	0	0	0
8	9	1	0	0	0
8	39	1	0	0	0
8	40	1	0	0	0
9	10	2	0	0	0
9	14	1	0	0	0
10	11	1	0	0	0
10	41	1	0	0	0
11	12	2	0	0	0
11	42	1	0	0	0
12	13	1	0	0	0
12	15	1	0	0	0
13	14	2	0	0	0
13	43	1	0	0	0
14	44	1	0	0	0
15	45	1	0	0	0
16	46	1	0	0	0
16	47	1	0	0	0
16	48	1	0	0	0
17	29	2	0	0	0
17	30	1	0	0	0
17	18	1	0	0	0
18	19	1	0	0	0
19	20	1	0	0	0
19	49	1	0	0	0
19	50	1	0	0	0
20	21	1	0	0	0
20	51	1	0	0	0
21	22	1	0	0	0
21	52	1	0	0	0
21	53	1	0	0	0
22	23	2	0	0	0
22	27	1	0	0	0
23	24	1	0	0	0
23	54	1	0	0	0
24	25	2	0	0	0
24	55	1	0	0	0
25	26	1	0	0	0
25	28	1	0	0	0
26	27	2	0	0	0
26	56	1	0	0	0
27	57	1	0	0	0
28	34	1	0	0	0
28	35	1	0	0	0
28	36	1	0	0	0
30	31	1	0	0	0
30	32	1	0	0	0
30	33	1	0	0	0
31	58	1	0	0	0
31	59	1	0	0	0
31	60	1	0	0	0
32	61	1	0	0	0
32	62	1	0	0	0
32	63	1	0	0	0
33	64	1	0	0	0
33	65	1	0	0	0
33	66	1	0	0	0
34	67	1	0	0	0
34	68	1	0	0	0
34	69	1	0	0	0
35	70	1	0	0	0
35	71	1	0	0	0
35	72	1	0	0	0
36	73	1	0	0	0

```
 36 74  1  0  0  0
 36 75  1  0  0  0
M  END
> <s_m_entry_id>
12

> <s_m_entry_name>
A_6S.1

> <s_m_Source_Path>
C:\Users\julio\OneDrive - Universidad de Talca\Escritorio\Nueva carpeta

> <s_m_Source_File>
A_6S.mol2

> <i_m_Source_File_Index>
1

$$$$
A_10R
                    3D
 Structure written by MMmdl.
 69 70  0  0  1  0            999 V2000
  109.5430   92.6627  109.6576 O   0  0  0  0  0  0
  110.9686   92.7951  109.9813 S   0  0  0  0  0  0
  111.7319   91.6370  110.4573 O   0  0  0  0  0  0
  108.1060   96.4781  101.4421 N   0  0  0  0  0  0
  108.2229   96.1733  102.7743 C   0  0  0  0  0  0
  109.4093   96.6100  103.2937 N   0  0  0  0  0  0
  107.0477   95.3922  103.6913 S   0  0  0  0  0  0
  109.6055   97.0415  104.6937 C   0  0  0  0  0  0
  110.1423   96.0248  105.7062 C   0  0  0  0  0  0
  110.2548   94.6566  105.4255 C   0  0  0  0  0  0
  110.7825   93.7779  106.3737 C   0  0  0  0  0  0
  111.2147   94.2554  107.6171 C   0  0  0  0  0  0
  111.0848   95.6155  107.9085 C   0  0  0  0  0  0
  110.5516   96.4910  106.9603 C   0  0  0  0  0  0
  111.8094   93.3891  108.5442 N   0  0  0  0  0  0
  111.2131   94.1937  111.2033 C   0  0  0  0  0  0
  105.2105   99.6510  100.8480 C   0  0  0  0  0  0
  106.0230   98.6136  100.5486 O   0  0  0  0  0  0
  105.7691   97.3071  101.0434 C   0  0  0  0  0  0
  106.8889   96.3190  100.6129 C   0  0  1  0  0  0
  107.2522   96.4190   99.0977 C   0  0  0  0  0  0
  106.1676   96.0306   98.0887 C   0  0  0  0  0  0
  105.0314   96.8254   97.9143 C   0  0  0  0  0  0
  104.0792   96.5054   96.9484 C   0  0  0  0  0  0
  104.2539   95.3940   96.1222 C   0  0  0  0  0  0
  105.3991   94.5963   96.2739 C   0  0  0  0  0  0
  106.3397   94.9130   97.2626 C   0  0  0  0  0  0
  105.6403   93.3979   95.3719 C   0  0  0  0  0  0
  103.2027   95.0786   95.0648 C   0  0  0  0  0  0
  104.3626   99.6450  101.7469 O   0  0  0  0  0  0
  105.4091  100.8817   99.9108 C   0  0  0  0  0  0
  106.4304  101.8415  100.5639 C   0  0  0  0  0  0
  105.9331  100.4654   98.5055 C   0  0  0  0  0  0
  104.0723  101.6460   99.7218 C   0  0  0  0  0  0
  110.9048   92.4674  106.0590 F   0  0  0  0  0  0
  108.9318   96.8119  100.9626 H   0  0  0  0  0  0
  110.1858   96.7405  102.6600 H   0  0  0  0  0  0
  110.3226   97.8616  104.6620 H   0  0  0  0  0  0
  108.6865   97.4742  105.0966 H   0  0  0  0  0  0
  109.9445   94.2636  104.4697 H   0  0  0  0  0  0
  111.4198   96.0062  108.8587 H   0  0  0  0  0  0
  110.4696   97.5408  107.2025 H   0  0  0  0  0  0
  112.6646   92.9170  108.2888 H   0  0  0  0  0  0
  110.8132   93.8766  112.1634 H   0  0  0  0  0  0
  110.6705   95.0701  110.8554 H   0  0  0  0  0  0
  112.2761   94.4091  111.2932 H   0  0  0  0  0  0
  104.8033   96.9545  100.6853 H   0  0  0  0  0  0
  105.6843   97.3552  102.1280 H   0  0  0  0  0  0
```

```
 106.5297   95.3025  100.7838 H   0  0  0  0  0  0
 108.1203   95.7955   98.8861 H   0  0  0  0  0  0
 107.5837   97.4314   98.8620 H   0  0  0  0  0  0
 104.8749   97.7079   98.5126 H   0  0  0  0  0  0
 103.2097   97.1383   96.8370 H   0  0  0  0  0  0
 107.2226   94.2998   97.3676 H   0  0  0  0  0  0
 106.6367   92.9887   95.5203 H   0  0  0  0  0  0
 105.5534   93.6776   94.3217 H   0  0  0  0  0  0
 104.9137   92.6134   95.5778 H   0  0  0  0  0  0
 103.6298   95.1803   94.0643 H   0  0  0  0  0  0
 102.3480   95.7540   95.1235 H   0  0  0  0  0  0
 102.8323   94.0596   95.1768 H   0  0  0  0  0  0
 106.5806  102.7363   99.9558 H   0  0  0  0  0  0
 107.4048  101.3675  100.6654 H   0  0  0  0  0  0
 106.1219  102.1645  101.5591 H   0  0  0  0  0  0
 106.2183  101.3393   97.9226 H   0  0  0  0  0  0
 105.1763   99.9172   97.9369 H   0  0  0  0  0  0
 106.8154   99.8198   98.5454 H   0  0  0  0  0  0
 104.1895  102.4932   99.0468 H   0  0  0  0  0  0
 103.6894  102.0429  100.6638 H   0  0  0  0  0  0
 103.2952  101.0049   99.2996 H   0  0  0  0  0  0
  1  2  2  0  0  0
  2 15  1  0  0  0
  2 16  1  0  0  0
  2  3  2  0  0  0
  4 20  1  0  0  0
  4  5  1  0  0  0
  4 36  1  0  0  0
  5  6  1  0  0  0
  5  7  2  0  0  0
  6  8  1  0  0  0
  6 37  1  0  0  0
  8  9  1  0  0  0
  8 38  1  0  0  0
  8 39  1  0  0  0
  9 10  2  0  0  0
  9 14  1  0  0  0
 10 11  1  0  0  0
 10 40  1  0  0  0
 11 12  2  0  0  0
 11 35  1  0  0  0
 12 13  1  0  0  0
 12 15  1  0  0  0
 13 14  2  0  0  0
 13 41  1  0  0  0
 14 42  1  0  0  0
 15 43  1  0  0  0
 16 44  1  0  0  0
 16 45  1  0  0  0
 16 46  1  0  0  0
 17 18  1  0  0  0
 17 30  2  0  0  0
 17 31  1  0  0  0
 18 19  1  0  0  0
 19 20  1  0  0  0
 19 47  1  0  0  0
 19 48  1  0  0  0
 20 21  1  0  0  0
 20 49  1  0  0  0
 21 22  1  0  0  0
 21 50  1  0  0  0
 21 51  1  0  0  0
 22 23  2  0  0  0
 22 27  1  0  0  0
 23 24  1  0  0  0
 23 52  1  0  0  0
 24 25  2  0  0  0
 24 53  1  0  0  0
 25 26  1  0  0  0
 25 29  1  0  0  0
 26 27  2  0  0  0
```

```
 26 28  1  0  0  0
 27 54  1  0  0  0
 28 55  1  0  0  0
 28 56  1  0  0  0
 28 57  1  0  0  0
 29 58  1  0  0  0
 29 59  1  0  0  0
 29 60  1  0  0  0
 31 32  1  0  0  0
 31 33  1  0  0  0
 31 34  1  0  0  0
 32 61  1  0  0  0
 32 62  1  0  0  0
 32 63  1  0  0  0
 33 64  1  0  0  0
 33 65  1  0  0  0
 33 66  1  0  0  0
 34 67  1  0  0  0
 34 68  1  0  0  0
 34 69  1  0  0  0
M  END
> <s_m_entry_id>
13

> <s_m_entry_name>
A_10R.1

> <s_m_Source_Path>
C:\Users\julio\OneDrive - Universidad de Talca\Escritorio\Nueva carpeta

> <s_m_Source_File>
A_10R.mol2

> <i_m_Source_File_Index>
1

$$$$
A_10S
                    3D
 Structure written by MMmdl.
 69 70  0  0  1  0            999 V2000
  109.6559   92.8460  109.7296 O   0  0  0  0  0  0
  111.0975   92.9444  109.9791 S   0  0  0  0  0  0
  111.8485   91.7784  110.4570 O   0  0  0  0  0  0
  107.9903   95.8227  101.3497 N   0  0  0  0  0  0
  107.8213   96.0013  102.6901 C   0  0  0  0  0  0
  109.0078   96.3044  103.2958 N   0  0  0  0  0  0
  106.3400   95.8765  103.4646 S   0  0  0  0  0  0
  109.1739   96.8193  104.6669 C   0  0  0  0  0  0
  109.8592   95.8874  105.6632 C   0  0  0  0  0  0
  110.0328   94.5197  105.4177 C   0  0  0  0  0  0
  110.6909   93.7105  106.3442 C   0  0  0  0  0  0
  111.1881   94.2582  107.5344 C   0  0  0  0  0  0
  111.0141   95.6217  107.7835 C   0  0  0  0  0  0
  110.3540   96.4290  106.8550 C   0  0  0  0  0  0
  111.8565   93.4542  108.4676 N   0  0  0  0  0  0
  111.4510   94.3842  111.1370 C   0  0  0  0  0  0
  106.3602   94.9440   97.0871 C   0  0  0  0  0  0
  105.8488   95.1647   98.3309 O   0  0  0  0  0  0
  106.6065   94.9020   99.5133 C   0  0  0  0  0  0
  107.0179   96.1648  100.2947 C   0  0  2  0  0  0
  107.6059   97.3025   99.4159 C   0  0  0  0  0  0
  106.8824   98.6284   99.5860 C   0  0  0  0  0  0
  106.4451   99.3323   98.4643 C   0  0  0  0  0  0
  105.8009  100.5583   98.6192 C   0  0  0  0  0  0
  105.5830  101.0897   99.8950 C   0  0  0  0  0  0
  106.0032  100.3747  101.0287 C   0  0  0  0  0  0
  106.6564   99.1541  100.8619 C   0  0  0  0  0  0
  105.7749  100.8955  102.4408 C   0  0  0  0  0  0
  104.8984  102.4353  100.0259 C   0  0  0  0  0  0
  107.5209   94.5934   96.8608 O   0  0  0  0  0  0
```

105.3641	95.1978	95.9154	C	0	0	0	0	0	0
106.0495	96.0695	94.8299	C	0	0	0	0	0	0
104.9830	93.8315	95.2905	C	0	0	0	0	0	0
104.0676	95.9185	96.3730	C	0	0	0	0	0	0
110.8697	92.4002	106.0570	F	0	0	0	0	0	0
108.9118	95.5570	101.0299	H	0	0	0	0	0	0
109.8523	96.1987	102.7495	H	0	0	0	0	0	0
109.7902	97.7166	104.5842	H	0	0	0	0	0	0
108.2400	97.1662	105.1063	H	0	0	0	0	0	0
109.6626	94.0691	104.5079	H	0	0	0	0	0	0
111.4008	96.0673	108.6886	H	0	0	0	0	0	0
110.2361	97.4827	107.0645	H	0	0	0	0	0	0
112.6974	92.9653	108.1870	H	0	0	0	0	0	0
111.0976	94.1158	112.1315	H	0	0	0	0	0	0
110.9046	95.2615	110.7964	H	0	0	0	0	0	0
112.5212	94.5745	111.1554	H	0	0	0	0	0	0
107.4559	94.2599	99.2996	H	0	0	0	0	0	0
105.9763	94.3153	100.1795	H	0	0	0	0	0	0
106.0912	96.5299	100.7386	H	0	0	0	0	0	0
107.6151	97.0289	98.3621	H	0	0	0	0	0	0
108.6560	97.4781	99.6432	H	0	0	0	0	0	0
106.6076	98.9265	97.4789	H	0	0	0	0	0	0
105.4740	101.0963	97.7420	H	0	0	0	0	0	0
106.9993	98.6140	101.7321	H	0	0	0	0	0	0
105.8541	100.0991	103.1805	H	0	0	0	0	0	0
104.7792	101.3286	102.5432	H	0	0	0	0	0	0
106.5151	101.6586	102.6822	H	0	0	0	0	0	0
103.8336	102.3059	100.2196	H	0	0	0	0	0	0
105.0174	103.0283	99.1195	H	0	0	0	0	0	0
105.3361	102.9924	100.8544	H	0	0	0	0	0	0
105.3895	96.2375	93.9779	H	0	0	0	0	0	0
106.9592	95.6071	94.4392	H	0	0	0	0	0	0
106.3265	97.0508	95.2170	H	0	0	0	0	0	0
104.3047	93.9536	94.4455	H	0	0	0	0	0	0
104.4804	93.1811	96.0082	H	0	0	0	0	0	0
105.8554	93.2902	94.9195	H	0	0	0	0	0	0
103.3971	96.1074	95.5338	H	0	0	0	0	0	0
104.2828	96.8845	96.8331	H	0	0	0	0	0	0
103.5071	95.3283	97.1011	H	0	0	0	0	0	0

1	2	2	0	0	0
2	15	1	0	0	0
2	16	1	0	0	0
2	3	2	0	0	0
4	20	1	0	0	0
4	5	1	0	0	0
4	36	1	0	0	0
5	6	1	0	0	0
5	7	2	0	0	0
6	8	1	0	0	0
6	37	1	0	0	0
8	9	1	0	0	0
8	38	1	0	0	0
8	39	1	0	0	0
9	10	2	0	0	0
9	14	1	0	0	0
10	11	1	0	0	0
10	40	1	0	0	0
11	12	2	0	0	0
11	35	1	0	0	0
12	13	1	0	0	0
12	15	1	0	0	0
13	14	2	0	0	0
13	41	1	0	0	0
14	42	1	0	0	0
15	43	1	0	0	0
16	44	1	0	0	0
16	45	1	0	0	0
16	46	1	0	0	0
17	18	1	0	0	0
17	30	2	0	0	0
17	31	1	0	0	0

```
 18 19  1  0  0  0
 19 20  1  0  0  0
 19 47  1  0  0  0
 19 48  1  0  0  0
 20 21  1  0  0  0
 20 49  1  0  0  0
 21 22  1  0  0  0
 21 50  1  0  0  0
 21 51  1  0  0  0
 22 23  2  0  0  0
 22 27  1  0  0  0
 23 24  1  0  0  0
 23 52  1  0  0  0
 24 25  2  0  0  0
 24 53  1  0  0  0
 25 26  1  0  0  0
 25 29  1  0  0  0
 26 27  2  0  0  0
 26 28  1  0  0  0
 27 54  1  0  0  0
 28 55  1  0  0  0
 28 56  1  0  0  0
 28 57  1  0  0  0
 29 58  1  0  0  0
 29 59  1  0  0  0
 29 60  1  0  0  0
 31 32  1  0  0  0
 31 33  1  0  0  0
 31 34  1  0  0  0
 32 61  1  0  0  0
 32 62  1  0  0  0
 32 63  1  0  0  0
 33 64  1  0  0  0
 33 65  1  0  0  0
 33 66  1  0  0  0
 34 67  1  0  0  0
 34 68  1  0  0  0
 34 69  1  0  0  0
M  END
> <s_m_entry_id>
14

> <s_m_entry_name>
A_10S.1

> <s_m_Source_Path>
C:\Users\julio\OneDrive - Universidad de Talca\Escritorio\Nueva carpeta

> <s_m_Source_File>
A_10S.mol2

> <i_m_Source_File_Index>
1

$$$$
A_11R
                    3D
 Structure written by MMmdl.
 75 76  0  0  1  0            999 V2000
  109.9223   94.1802  109.8662 O   0  0  0  0  0  0
  111.0176   93.2085  109.7756 S   0  0  0  0  0  0
  110.7607   91.7768  109.9714 O   0  0  0  0  0  0
  107.7490   97.0808  101.7007 N   0  0  0  0  0  0
  107.5674   96.1065  102.6458 C   0  0  0  0  0  0
  108.7325   95.4195  102.8498 N   0  0  0  0  0  0
  106.1193   95.7925  103.4478 S   0  0  0  0  0  0
  108.8411   94.0090  103.2470 C   0  0  0  0  0  0
  109.5891   93.8373  104.5605 C   0  0  0  0  0  0
  109.8997   94.9509  105.3456 C   0  0  0  0  0  0
  110.6136   94.8001  106.5360 C   0  0  0  0  0  0
  111.0373   93.5312  106.9556 C   0  0  0  0  0  0
```

```
110.7339   92.4192  106.1569 C   0  0  0  0  0  0
110.0208   92.5721  104.9673 C   0  0  0  0  0  0
111.7502   93.4296  108.1637 N   0  0  0  0  0  0
112.3906   93.7705  110.9508 C   0  0  0  0  0  0
104.3529  100.5580  101.3537 C   0  0  0  0  0  0
105.4633   99.9090  100.9102 O   0  0  0  0  0  0
106.1664   98.9929  101.7466 C   0  0  0  0  0  0
106.7041   97.7899  100.9348 C   0  0  1  0  0  0
107.2272   98.2234   99.5369 C   0  0  0  0  0  0
106.5712   97.5082   98.3556 C   0  0  0  0  0  0
105.3942   98.0121   97.8009 C   0  0  0  0  0  0
104.8003   97.3686   96.7182 C   0  0  0  0  0  0
105.3670   96.2097   96.1616 C   0  0  0  0  0  0
106.5552   95.7175   96.7304 C   0  0  0  0  0  0
107.1507   96.3598   97.8136 C   0  0  0  0  0  0
104.6723   95.5203   94.9518 C   0  0  0  0  0  0
103.8904  100.4567  102.4947 O   0  0  0  0  0  0
103.6965  101.4634  100.2692 C   0  0  0  0  0  0
102.6708  102.4181  100.9363 C   0  0  0  0  0  0
104.7640  102.3241   99.5367 C   0  0  0  0  0  0
102.9753  100.5780   99.2186 C   0  0  0  0  0  0
105.0761   96.2350   93.6343 C   0  0  0  0  0  0
103.1185   95.5476   95.0286 C   0  0  0  0  0  0
105.0992   94.0297   94.8778 C   0  0  0  0  0  0
110.8917   95.8993  107.2776 F   0  0  0  0  0  0
108.6973   97.3794  101.5229 H   0  0  0  0  0  0
109.5810   95.8431  102.5022 H   0  0  0  0  0  0
107.8578   93.5492  103.3583 H   0  0  0  0  0  0
109.3588   93.4557  102.4616 H   0  0  0  0  0  0
109.5859   95.9378  105.0363 H   0  0  0  0  0  0
111.0480   91.4258  106.4352 H   0  0  0  0  0  0
109.8044   91.7016  104.3647 H   0  0  0  0  0  0
112.7365   93.6444  108.1315 H   0  0  0  0  0  0
112.0245   93.7077  111.9755 H   0  0  0  0  0  0
112.6562   94.8021  110.7198 H   0  0  0  0  0  0
113.2644   93.1311  110.8366 H   0  0  0  0  0  0
105.4826   98.6389  102.5201 H   0  0  0  0  0  0
106.9882   99.5019  102.2540 H   0  0  0  0  0  0
105.8668   97.1182  100.7445 H   0  0  0  0  0  0
108.3089   98.0842   99.4844 H   0  0  0  0  0  0
107.0731   99.2940   99.3936 H   0  0  0  0  0  0
104.9351   98.9029   98.2052 H   0  0  0  0  0  0
103.8895   97.7875   96.3168 H   0  0  0  0  0  0
107.0463   94.8340   96.3539 H   0  0  0  0  0  0
108.0645   95.9585   98.2279 H   0  0  0  0  0  0
102.2235  103.0951  100.2067 H   0  0  0  0  0  0
103.1329  103.0417  101.7039 H   0  0  0  0  0  0
101.8449  101.8821  101.4073 H   0  0  0  0  0  0
104.3107  102.9614   98.7757 H   0  0  0  0  0  0
105.5085  101.7103   99.0260 H   0  0  0  0  0  0
105.2985  102.9866  100.2202 H   0  0  0  0  0  0
102.5389  101.1814   98.4210 H   0  0  0  0  0  0
102.1576   99.9985   99.6505 H   0  0  0  0  0  0
103.6578   99.8713   98.7425 H   0  0  0  0  0  0
106.1476   96.1801   93.4393 H   0  0  0  0  0  0
104.8060   97.2921   93.6529 H   0  0  0  0  0  0
104.5824   95.7942   92.7666 H   0  0  0  0  0  0
102.7418   95.0286   95.9116 H   0  0  0  0  0  0
102.6630   95.0574   94.1664 H   0  0  0  0  0  0
102.7261   96.5657   95.0463 H   0  0  0  0  0  0
104.8455   93.4944   95.7943 H   0  0  0  0  0  0
106.1714   93.9083   94.7207 H   0  0  0  0  0  0
104.6064   93.5076   94.0559 H   0  0  0  0  0  0
1  2  2  0  0  0
2 15  1  0  0  0
2 16  1  0  0  0
2  3  2  0  0  0
4 20  1  0  0  0
4  5  1  0  0  0
4 38  1  0  0  0
5  6  1  0  0  0
```

```
  5  7  2  0  0  0
  6  8  1  0  0  0
  6 39  1  0  0  0
  8  9  1  0  0  0
  8 40  1  0  0  0
  8 41  1  0  0  0
  9 14  2  0  0  0
  9 10  1  0  0  0
 10 11  2  0  0  0
 10 42  1  0  0  0
 11 12  1  0  0  0
 11 37  1  0  0  0
 12 13  2  0  0  0
 12 15  1  0  0  0
 13 14  1  0  0  0
 13 43  1  0  0  0
 14 44  1  0  0  0
 15 45  1  0  0  0
 16 46  1  0  0  0
 16 47  1  0  0  0
 16 48  1  0  0  0
 17 29  2  0  0  0
 17 30  1  0  0  0
 17 18  1  0  0  0
 18 19  1  0  0  0
 19 20  1  0  0  0
 19 49  1  0  0  0
 19 50  1  0  0  0
 20 21  1  0  0  0
 20 51  1  0  0  0
 21 22  1  0  0  0
 21 52  1  0  0  0
 21 53  1  0  0  0
 22 23  2  0  0  0
 22 27  1  0  0  0
 23 24  1  0  0  0
 23 54  1  0  0  0
 24 25  2  0  0  0
 24 55  1  0  0  0
 25 26  1  0  0  0
 25 28  1  0  0  0
 26 27  2  0  0  0
 26 56  1  0  0  0
 27 57  1  0  0  0
 28 34  1  0  0  0
 28 35  1  0  0  0
 28 36  1  0  0  0
 30 31  1  0  0  0
 30 32  1  0  0  0
 30 33  1  0  0  0
 31 58  1  0  0  0
 31 59  1  0  0  0
 31 60  1  0  0  0
 32 61  1  0  0  0
 32 62  1  0  0  0
 32 63  1  0  0  0
 33 64  1  0  0  0
 33 65  1  0  0  0
 33 66  1  0  0  0
 34 67  1  0  0  0
 34 68  1  0  0  0
 34 69  1  0  0  0
 35 70  1  0  0  0
 35 71  1  0  0  0
 35 72  1  0  0  0
 36 73  1  0  0  0
 36 74  1  0  0  0
 36 75  1  0  0  0
M  END
> <s_m_entry_id>
15
```

```
> <s_m_entry_name>
A_11R.1

> <s_m_Source_Path>
C:\Users\julio\OneDrive - Universidad de Talca\Escritorio\Nueva carpeta

> <s_m_Source_File>
A_11R.mol2

> <i_m_Source_File_Index>
1

$$$$
A_11S
                    3D
 Structure written by MMmdl.
 75 76  0  0  1  0            999 V2000
  109.6400   92.5904  109.6597 O   0  0  0  0  0  0
  111.0864   92.7069  109.8781 S   0  0  0  0  0  0
  111.8520   91.5956  110.4567 O   0  0  0  0  0  0
  107.6088   95.8061  101.2058 N   0  0  0  0  0  0
  107.5242   96.1289  102.5320 C   0  0  0  0  0  0
  108.6864   95.8193  103.1844 N   0  0  0  0  0  0
  106.1805   96.8343  103.2568 S   0  0  0  0  0  0
  109.1113   96.3835  104.4783 C   0  0  0  0  0  0
  109.8549   95.4484  105.4307 C   0  0  0  0  0  0
  110.5401   96.0260  106.5007 C   0  0  0  0  0  0
  111.1897   95.2293  107.4388 C   0  0  0  0  0  0
  111.1583   93.8377  107.3350 C   0  0  0  0  0  0
  110.5092   93.2532  106.2441 C   0  0  0  0  0  0
  109.8602   94.0520  105.2953 C   0  0  0  0  0  0
  111.8070   93.0472  108.2955 N   0  0  0  0  0  0
  111.4267   94.2499  110.8967 C   0  0  0  0  0  0
  106.4115   95.2187   96.7597 C   0  0  0  0  0  0
  105.7940   95.2752   97.9727 O   0  0  0  0  0  0
  106.4886   94.9924   99.1879 C   0  0  0  0  0  0
  106.6692   96.2004  100.1341 C   0  0  2  0  0  0
  107.1668   97.4909   99.4180 C   0  0  0  0  0  0
  106.1059   98.5650   99.1637 C   0  0  0  0  0  0
  104.7971   98.2488   98.7898 C   0  0  0  0  0  0
  103.8836   99.2601   98.5038 C   0  0  0  0  0  0
  104.2527  100.6119   98.5865 C   0  0  0  0  0  0
  105.5632  100.9181   98.9850 C   0  0  0  0  0  0
  106.4678   99.9065   99.2793 C   0  0  0  0  0  0
  103.2148  101.7131   98.2565 C   0  0  0  0  0  0
  107.6159   95.0032   96.5992 O   0  0  0  0  0  0
  105.4577   95.4584   95.5520 C   0  0  0  0  0  0
  104.3387   96.4758   95.8932 C   0  0  0  0  0  0
  106.2328   96.0093   94.3281 C   0  0  0  0  0  0
  104.8180   94.1010   95.1672 C   0  0  0  0  0  0
  103.8138  103.1447   98.3052 C   0  0  0  0  0  0
  102.0581  101.6564   99.2864 C   0  0  0  0  0  0
  102.6494  101.4997   96.8292 C   0  0  0  0  0  0
  111.8563   95.8215  108.4457 F   0  0  0  0  0  0
  108.4535   95.3474  100.8925 H   0  0  0  0  0  0
  109.4128   95.3733  102.6424 H   0  0  0  0  0  0
  109.7571   97.2330  104.2512 H   0  0  0  0  0  0
  108.2799   96.8011  105.0482 H   0  0  0  0  0  0
  110.5618   97.0988  106.6177 H   0  0  0  0  0  0
  110.4936   92.1784  106.1311 H   0  0  0  0  0  0
  109.3405   93.5755  104.4752 H   0  0  0  0  0  0
  112.7316   92.6806  108.1081 H   0  0  0  0  0  0
  111.1572   94.0492  111.9322 H   0  0  0  0  0  0
  110.8105   95.0642  110.5251 H   0  0  0  0  0  0
  112.4813   94.5022  110.8272 H   0  0  0  0  0  0
  107.4468   94.5154   98.9893 H   0  0  0  0  0  0
  105.8936   94.2564   99.7277 H   0  0  0  0  0  0
  105.6833   96.3915  100.5618 H   0  0  0  0  0  0
  107.6395   97.2496   98.4640 H   0  0  0  0  0  0
  107.9614   97.9509  100.0132 H   0  0  0  0  0  0
```

```
 104.4714   97.2246   98.7035 H   0  0  0  0  0  0
 102.8830   98.9844   98.2127 H   0  0  0  0  0  0
 105.9088  101.9365   99.0798 H   0  0  0  0  0  0
 107.4574  100.1829   99.6006 H   0  0  0  0  0  0
 103.7214   96.6942   95.0230 H   0  0  0  0  0  0
 104.7531   97.4247   96.2299 H   0  0  0  0  0  0
 103.6667   96.1112   96.6718 H   0  0  0  0  0  0
 105.5832   96.1227   93.4549 H   0  0  0  0  0  0
 107.0555   95.3599   94.0267 H   0  0  0  0  0  0
 106.6552   96.9912   94.5388 H   0  0  0  0  0  0
 104.1411   94.2037   94.3169 H   0  0  0  0  0  0
 104.2366   93.6806   95.9887 H   0  0  0  0  0  0
 105.5690   93.3579   94.8887 H   0  0  0  0  0  0
 104.6329  103.2641   97.5923 H   0  0  0  0  0  0
 104.1915  103.3866   99.3006 H   0  0  0  0  0  0
 103.0691  103.9014   98.0587 H   0  0  0  0  0  0
 101.5310  100.7014   99.2560 H   0  0  0  0  0  0
 101.3196  102.4365   99.1004 H   0  0  0  0  0  0
 102.4267  101.7934  100.3048 H   0  0  0  0  0  0
 102.1117  100.5534   96.7329 H   0  0  0  0  0  0
 103.4453  101.5000   96.0811 H   0  0  0  0  0  0
 101.9498  102.2904   96.5550 H   0  0  0  0  0  0
  1  2  2  0  0  0
  2 15  1  0  0  0
  2 16  1  0  0  0
  2  3  2  0  0  0
  4 20  1  0  0  0
  4  5  1  0  0  0
  4 38  1  0  0  0
  5  6  1  0  0  0
  5  7  2  0  0  0
  6  8  1  0  0  0
  6 39  1  0  0  0
  8  9  1  0  0  0
  8 40  1  0  0  0
  8 41  1  0  0  0
  9 14  2  0  0  0
  9 10  1  0  0  0
 10 11  2  0  0  0
 10 42  1  0  0  0
 11 12  1  0  0  0
 11 37  1  0  0  0
 12 13  2  0  0  0
 12 15  1  0  0  0
 13 14  1  0  0  0
 13 43  1  0  0  0
 14 44  1  0  0  0
 15 45  1  0  0  0
 16 46  1  0  0  0
 16 47  1  0  0  0
 16 48  1  0  0  0
 17 29  2  0  0  0
 17 30  1  0  0  0
 17 18  1  0  0  0
 18 19  1  0  0  0
 19 20  1  0  0  0
 19 49  1  0  0  0
 19 50  1  0  0  0
 20 21  1  0  0  0
 20 51  1  0  0  0
 21 22  1  0  0  0
 21 52  1  0  0  0
 21 53  1  0  0  0
 22 23  2  0  0  0
 22 27  1  0  0  0
 23 24  1  0  0  0
 23 54  1  0  0  0
 24 25  2  0  0  0
 24 55  1  0  0  0
 25 26  1  0  0  0
 25 28  1  0  0  0
```

```
 26 27  2  0  0  0
 26 56  1  0  0  0
 27 57  1  0  0  0
 28 34  1  0  0  0
 28 35  1  0  0  0
 28 36  1  0  0  0
 30 31  1  0  0  0
 30 32  1  0  0  0
 30 33  1  0  0  0
 31 58  1  0  0  0
 31 59  1  0  0  0
 31 60  1  0  0  0
 32 61  1  0  0  0
 32 62  1  0  0  0
 32 63  1  0  0  0
 33 64  1  0  0  0
 33 65  1  0  0  0
 33 66  1  0  0  0
 34 67  1  0  0  0
 34 68  1  0  0  0
 34 69  1  0  0  0
 35 70  1  0  0  0
 35 71  1  0  0  0
 35 72  1  0  0  0
 36 73  1  0  0  0
 36 74  1  0  0  0
 36 75  1  0  0  0
M  END
> <s_m_entry_id>
16

> <s_m_entry_name>
A_11S.1

> <s_m_Source_Path>
C:\Users\julio\OneDrive - Universidad de Talca\Escritorio\Nueva carpeta

> <s_m_Source_File>
A_11S.mol2

> <i_m_Source_File_Index>
1

$$$$
A_18R
                    3D
 Structure written by MMmdl.
 72 73  0  0  1  0            999 V2000
  109.6586   92.6877  109.6575 O   0  0  0  0  0  0
  111.0810   92.8377  109.9827 S   0  0  0  0  0  0
  111.8565   91.6874  110.4569 O   0  0  0  0  0  0
  108.0204   95.6265  101.6847 N   0  0  0  0  0  0
  108.1137   95.7963  103.0295 C   0  0  0  0  0  0
  109.1665   96.5817  103.4050 N   0  0  0  0  0  0
  107.0560   95.0122  104.0560 S   0  0  0  0  0  0
  109.4660   97.0156  104.7849 C   0  0  0  0  0  0
  110.1207   96.0289  105.7577 C   0  0  0  0  0  0
  110.2892   94.6670  105.4653 C   0  0  0  0  0  0
  110.8464   93.8087  106.4097 C   0  0  0  0  0  0
  111.2805   94.3093  107.6343 C   0  0  0  0  0  0
  111.1433   95.6693  107.9227 C   0  0  0  0  0  0
  110.5564   96.5233  106.9909 C   0  0  0  0  0  0
  111.8949   93.4419  108.5417 N   0  0  0  0  0  0
  111.3189   94.2471  111.1971 C   0  0  0  0  0  0
  106.2831   95.8183   97.1663 C   0  0  0  0  0  0
  106.3465   96.3456   98.4180 O   0  0  0  0  0  0
  107.2401   95.7936   99.3755 C   0  0  0  0  0  0
  107.1166   96.3955  100.7947 C   0  0  1  0  0  0
  105.6236   96.4885  101.2538 C   0  0  0  0  0  0
  107.0537   94.9574   96.7294 O   0  0  0  0  0  0
  105.1279   96.3783   96.2866 C   0  0  0  0  0  0
```

105.6539	96.6556	94.8539	C	0	0	0	0	0	0
104.0083	95.3086	96.2107	C	0	0	0	0	0	0
104.5227	97.6910	96.8599	C	0	0	0	0	0	0
105.3038	97.6600	102.2175	C	0	0	0	0	0	0
104.9466	98.9969	101.5637	C	0	0	0	0	0	0
104.0332	99.8409	102.2017	C	0	0	0	0	0	0
103.6706	101.0555	101.6247	C	0	0	0	0	0	0
104.2222	101.4553	100.4046	C	0	0	0	0	0	0
105.1583	100.6242	99.7639	C	0	0	0	0	0	0
105.5068	99.4027	100.3466	C	0	0	0	0	0	0
105.7940	101.0238	98.4387	C	0	0	0	0	0	0
103.7902	102.7766	99.7895	C	0	0	0	0	0	0
108.8511	95.2675	101.2365	H	0	0	0	0	0	0
109.7245	96.9953	102.6680	H	0	0	0	0	0	0
110.1414	97.8693	104.6945	H	0	0	0	0	0	0
108.5628	97.4090	105.2519	H	0	0	0	0	0	0
109.9801	94.2608	104.5154	H	0	0	0	0	0	0
110.9614	92.7581	106.1768	H	0	0	0	0	0	0
111.4935	96.0749	108.8578	H	0	0	0	0	0	0
110.4421	97.5708	107.2334	H	0	0	0	0	0	0
112.8181	93.0799	108.3323	H	0	0	0	0	0	0
110.9606	93.9280	112.1743	H	0	0	0	0	0	0
110.7405	95.1053	110.8587	H	0	0	0	0	0	0
112.3746	94.4993	111.2495	H	0	0	0	0	0	0
108.2561	95.9454	99.0052	H	0	0	0	0	0	0
107.0651	94.7168	99.4298	H	0	0	0	0	0	0
107.5296	97.4028	100.7490	H	0	0	0	0	0	0
105.3256	95.5386	101.7025	H	0	0	0	0	0	0
104.9440	96.5782	100.4083	H	0	0	0	0	0	0
104.8678	97.0437	94.2030	H	0	0	0	0	0	0
106.0434	95.7552	94.3758	H	0	0	0	0	0	0
106.4566	97.3914	94.8631	H	0	0	0	0	0	0
103.1917	95.6303	95.5607	H	0	0	0	0	0	0
103.5764	95.1033	97.1909	H	0	0	0	0	0	0
104.3764	94.3632	95.8112	H	0	0	0	0	0	0
103.7339	98.0864	96.2153	H	0	0	0	0	0	0
105.2778	98.4736	96.9463	H	0	0	0	0	0	0
104.0835	97.5435	97.8498	H	0	0	0	0	0	0
106.1137	97.8319	102.9251	H	0	0	0	0	0	0
104.4547	97.3527	102.8272	H	0	0	0	0	0	0
103.5904	99.5508	103.1440	H	0	0	0	0	0	0
102.9592	101.6871	102.1368	H	0	0	0	0	0	0
106.2069	98.7680	99.8287	H	0	0	0	0	0	0
106.5656	100.3178	98.1268	H	0	0	0	0	0	0
106.2618	102.0083	98.5074	H	0	0	0	0	0	0
105.0333	101.0550	97.6576	H	0	0	0	0	0	0
104.6565	103.3928	99.5703	H	0	0	0	0	0	0
103.1432	103.3518	100.4538	H	0	0	0	0	0	0
103.2503	102.6009	98.8597	H	0	0	0	0	0	0

1	2	2	0	0	0
2	15	1	0	0	0
2	16	1	0	0	0
2	3	2	0	0	0
4	20	1	0	0	0
4	5	1	0	0	0
4	36	1	0	0	0
5	6	1	0	0	0
5	7	2	0	0	0
6	8	1	0	0	0
6	37	1	0	0	0
8	9	1	0	0	0
8	38	1	0	0	0
8	39	1	0	0	0
9	10	2	0	0	0
9	14	1	0	0	0
10	11	1	0	0	0
10	40	1	0	0	0
11	12	2	0	0	0
11	41	1	0	0	0
12	13	1	0	0	0
12	15	1	0	0	0

```
 13 14  2  0  0  0
 13 42  1  0  0  0
 14 43  1  0  0  0
 15 44  1  0  0  0
 16 45  1  0  0  0
 16 46  1  0  0  0
 16 47  1  0  0  0
 17 18  1  0  0  0
 17 22  2  0  0  0
 17 23  1  0  0  0
 18 19  1  0  0  0
 19 20  1  0  0  0
 19 48  1  0  0  0
 19 49  1  0  0  0
 20 21  1  0  0  0
 20 50  1  0  0  0
 21 27  1  0  0  0
 21 51  1  0  0  0
 21 52  1  0  0  0
 23 24  1  0  0  0
 23 25  1  0  0  0
 23 26  1  0  0  0
 24 53  1  0  0  0
 24 54  1  0  0  0
 24 55  1  0  0  0
 25 56  1  0  0  0
 25 57  1  0  0  0
 25 58  1  0  0  0
 26 59  1  0  0  0
 26 60  1  0  0  0
 26 61  1  0  0  0
 27 28  1  0  0  0
 27 62  1  0  0  0
 27 63  1  0  0  0
 28 29  2  0  0  0
 28 33  1  0  0  0
 29 30  1  0  0  0
 29 64  1  0  0  0
 30 31  2  0  0  0
 30 65  1  0  0  0
 31 32  1  0  0  0
 31 35  1  0  0  0
 32 33  2  0  0  0
 32 34  1  0  0  0
 33 66  1  0  0  0
 34 67  1  0  0  0
 34 68  1  0  0  0
 34 69  1  0  0  0
 35 70  1  0  0  0
 35 71  1  0  0  0
 35 72  1  0  0  0
M  END
> <s_m_entry_id>
17

> <s_m_entry_name>
A_18R.1

> <s_m_Source_Path>
C:\Users\julio\OneDrive - Universidad de Talca\Escritorio\Nueva carpeta

> <s_m_Source_File>
A_18R.mol2

> <i_m_Source_File_Index>
1

$$$$
A_18S
                    3D
 Structure written by MMmdl.
```

```
72 73  0  0  1  0            999 V2000
 109.6462   92.5960  109.6567 O   0  0  0  0  0  0
 111.0770   92.7548  109.9316 S   0  0  0  0  0  0
 111.8561   91.6295  110.4567 O   0  0  0  0  0  0
 107.8599   96.3390  101.3280 N   0  0  0  0  0  0
 107.7983   96.1116  102.6752 C   0  0  0  0  0  0
 109.0338   96.1865  103.2587 N   0  0  0  0  0  0
 106.3852   95.7975  103.5233 S   0  0  0  0  0  0
 109.2839   96.6847  104.6270 C   0  0  0  0  0  0
 109.9608   95.7325  105.6147 C   0  0  0  0  0  0
 109.9591   94.3420  105.4408 C   0  0  0  0  0  0
 110.5822   93.5203  106.3826 C   0  0  0  0  0  0
 111.2158   94.0812  107.4930 C   0  0  0  0  0  0
 111.2315   95.4659  107.6601 C   0  0  0  0  0  0
 110.6004   96.2854  106.7274 C   0  0  0  0  0  0
 111.8542   93.2560  108.4256 N   0  0  0  0  0  0
 111.3546   94.2453  111.0428 C   0  0  0  0  0  0
 105.7956   95.0591   97.1960 C   0  0  0  0  0  0
 105.6400   95.6949   98.3903 O   0  0  0  0  0  0
 106.4973   95.4045   99.4935 C   0  0  0  0  0  0
 106.7278   96.6216  100.4182 C   0  0  2  0  0  0
 107.0051   97.9308   99.6357 C   0  0  0  0  0  0
 106.6401   94.1925   96.9659 O   0  0  0  0  0  0
 104.7821   95.5267   96.1084 C   0  0  0  0  0  0
 104.7761   94.5507   94.8990 C   0  0  0  0  0  0
 103.3459   95.5964   96.6904 C   0  0  0  0  0  0
 105.2031   96.9311   95.6098 C   0  0  0  0  0  0
 107.0564   99.2061  100.5077 C   0  0  0  0  0  0
 105.7261   99.9193  100.6925 C   0  0  0  0  0  0
 104.8956   99.5980  101.7686 C   0  0  0  0  0  0
 103.7080  100.3004  101.9680 C   0  0  0  0  0  0
 103.3375  101.3317  101.0986 C   0  0  0  0  0  0
 104.1698  101.6565  100.0124 C   0  0  0  0  0  0
 105.3564  100.9437   99.8176 C   0  0  0  0  0  0
 103.8051  102.7719   99.0412 C   0  0  0  0  0  0
 102.0403  102.0859  101.3561 C   0  0  0  0  0  0
 108.7751   96.3375  100.8996 H   0  0  0  0  0  0
 109.8435   96.0121  102.6785 H   0  0  0  0  0  0
 109.9288   97.5589  104.5217 H   0  0  0  0  0  0
 108.3794   97.0644  105.1054 H   0  0  0  0  0  0
 109.4692   93.8919  104.5880 H   0  0  0  0  0  0
 110.5736   92.4479  106.2459 H   0  0  0  0  0  0
 111.7278   95.9108  108.5105 H   0  0  0  0  0  0
 110.6104   97.3557  106.8768 H   0  0  0  0  0  0
 112.7945   92.9309  108.2417 H   0  0  0  0  0  0
 111.0466   93.9905  112.0562 H   0  0  0  0  0  0
 110.7576   95.0803  110.6793 H   0  0  0  0  0  0
 112.4126   94.5006  111.0333 H   0  0  0  0  0  0
 107.4561   95.0430   99.1308 H   0  0  0  0  0  0
 106.0571   94.5922  100.0728 H   0  0  0  0  0  0
 105.8016   96.7827  100.9729 H   0  0  0  0  0  0
 106.2719   98.0801   98.8417 H   0  0  0  0  0  0
 107.9616   97.8248   99.1196 H   0  0  0  0  0  0
 104.0645   94.8658   94.1356 H   0  0  0  0  0  0
 104.4982   93.5371   95.1958 H   0  0  0  0  0  0
 105.7542   94.4901   94.4178 H   0  0  0  0  0  0
 102.6243   95.8843   95.9261 H   0  0  0  0  0  0
 103.2620   96.3295   97.4956 H   0  0  0  0  0  0
 103.0233   94.6319   97.0902 H   0  0  0  0  0  0
 104.5367   97.2920   94.8261 H   0  0  0  0  0  0
 106.2149   96.9293   95.1930 H   0  0  0  0  0  0
 105.1841   97.6731   96.4115 H   0  0  0  0  0  0
 107.7558   99.9101  100.0571 H   0  0  0  0  0  0
 107.4561   98.9935  101.4988 H   0  0  0  0  0  0
 105.1728   98.8149  102.4593 H   0  0  0  0  0  0
 103.0804  100.0404  102.8090 H   0  0  0  0  0  0
 106.0040  101.1958   98.9896 H   0  0  0  0  0  0
 104.6228  102.9942   98.3549 H   0  0  0  0  0  0
 103.5670  103.6904   99.5768 H   0  0  0  0  0  0
 102.9390  102.4843   98.4454 H   0  0  0  0  0  0
 102.2500  103.1284  101.6004 H   0  0  0  0  0  0
```

```
 101.4761  101.6580  102.1863 H   0  0  0  0  0  0
 101.4001  102.0587  100.4738 H   0  0  0  0  0  0
 1  2  2  0  0  0
 2 15  1  0  0  0
 2 16  1  0  0  0
 2  3  2  0  0  0
 4 20  1  0  0  0
 4  5  1  0  0  0
 4 36  1  0  0  0
 5  6  1  0  0  0
 5  7  2  0  0  0
 6  8  1  0  0  0
 6 37  1  0  0  0
 8  9  1  0  0  0
 8 38  1  0  0  0
 8 39  1  0  0  0
 9 10  2  0  0  0
 9 14  1  0  0  0
10 11  1  0  0  0
10 40  1  0  0  0
11 12  2  0  0  0
11 41  1  0  0  0
12 13  1  0  0  0
12 15  1  0  0  0
13 14  2  0  0  0
13 42  1  0  0  0
14 43  1  0  0  0
15 44  1  0  0  0
16 45  1  0  0  0
16 46  1  0  0  0
16 47  1  0  0  0
17 18  1  0  0  0
17 22  2  0  0  0
17 23  1  0  0  0
18 19  1  0  0  0
19 20  1  0  0  0
19 48  1  0  0  0
19 49  1  0  0  0
20 21  1  0  0  0
20 50  1  0  0  0
21 27  1  0  0  0
21 51  1  0  0  0
21 52  1  0  0  0
23 24  1  0  0  0
23 25  1  0  0  0
23 26  1  0  0  0
24 53  1  0  0  0
24 54  1  0  0  0
24 55  1  0  0  0
25 56  1  0  0  0
25 57  1  0  0  0
25 58  1  0  0  0
26 59  1  0  0  0
26 60  1  0  0  0
26 61  1  0  0  0
27 28  1  0  0  0
27 62  1  0  0  0
27 63  1  0  0  0
28 29  2  0  0  0
28 33  1  0  0  0
29 30  1  0  0  0
29 64  1  0  0  0
30 31  2  0  0  0
30 65  1  0  0  0
31 32  1  0  0  0
31 35  1  0  0  0
32 33  2  0  0  0
32 34  1  0  0  0
33 66  1  0  0  0
34 67  1  0  0  0
34 68  1  0  0  0
```

```
 34 69  1  0  0  0
 35 70  1  0  0  0
 35 71  1  0  0  0
 35 72  1  0  0  0
M  END
> <s_m_entry_id>
18

> <s_m_entry_name>
A_18S.1

> <s_m_Source_Path>
C:\Users\julio\OneDrive - Universidad de Talca\Escritorio\Nueva carpeta

> <s_m_Source_File>
A_18S.mol2

> <i_m_Source_File_Index>
1

$$$$
A_19R
                   3D
 Structure written by MMmdl.
 78 79  0  0  1  0            999 V2000
  109.6607   92.8748  109.5437 O   0  0  0  0  0  0
  111.0970   92.9882  109.7871 S   0  0  0  0  0  0
  111.8396   91.8544  110.3531 O   0  0  0  0  0  0
  107.4539   95.0363  101.5707 N   0  0  0  0  0  0
  107.7946   95.5442  102.7845 C   0  0  0  0  0  0
  109.0560   96.0641  102.8578 N   0  0  0  0  0  0
  106.7418   95.3913  104.0565 S   0  0  0  0  0  0
  109.6673   96.6706  104.0619 C   0  0  0  0  0  0
  110.1919   95.7542  105.1833 C   0  0  0  0  0  0
  110.1424   94.3552  105.1105 C   0  0  0  0  0  0
  110.6559   93.5757  106.1493 C   0  0  0  0  0  0
  111.2438   94.1838  107.2609 C   0  0  0  0  0  0
  111.2871   95.5761  107.3437 C   0  0  0  0  0  0
  110.7587   96.3540  106.3135 C   0  0  0  0  0  0
  111.8562   93.3932  108.2423 N   0  0  0  0  0  0
  111.4572   94.5049  110.8351 C   0  0  0  0  0  0
  106.3521   95.2691   96.8725 C   0  0  0  0  0  0
  106.1713   95.7488   98.1331 O   0  0  0  0  0  0
  107.0563   95.3616   99.1820 C   0  0  0  0  0  0
  106.5958   95.7589  100.6075 C   0  0  1  0  0  0
  105.0788   95.4570  100.8264 C   0  0  0  0  0  0
  107.3208   94.5929   96.5148 O   0  0  0  0  0  0
  105.2065   95.6504   95.8890 C   0  0  0  0  0  0
  104.8073   97.1368   96.0621 C   0  0  0  0  0  0
  105.6386   95.4292   94.4139 C   0  0  0  0  0  0
  103.9804   94.7539   96.1842 C   0  0  0  0  0  0
  104.2545   96.4877  101.6291 C   0  0  0  0  0  0
  104.2330   97.8571  100.9768 C   0  0  0  0  0  0
  104.9788   98.9001  101.5216 C   0  0  0  0  0  0
  104.9964  100.1376  100.8905 C   0  0  0  0  0  0
  104.2808  100.3481   99.7020 C   0  0  0  0  0  0
  103.5121   99.2954   99.1825 C   0  0  0  0  0  0
  103.4872   98.0586   99.8164 C   0  0  0  0  0  0
  104.3734  101.7215   99.0004 C   0  0  0  0  0  0
  105.8559  102.0374   98.7057 C   0  0  0  0  0  0
  103.7794  102.8130   99.9190 C   0  0  0  0  0  0
  103.6143  101.7811   97.6482 C   0  0  0  0  0  0
  107.4738   94.0301  101.4735 H   0  0  0  0  0  0
  109.6637   95.9407  102.0554 H   0  0  0  0  0  0
  110.5233   97.2497  103.7159 H   0  0  0  0  0  0
  108.9784   97.3999  104.4906 H   0  0  0  0  0  0
  109.7128   93.8583  104.2536 H   0  0  0  0  0  0
  110.6206   92.4981  106.0745 H   0  0  0  0  0  0
  111.7471   96.0590  108.1937 H   0  0  0  0  0  0
  110.8086   97.4313  106.3878 H   0  0  0  0  0  0
  112.7776   93.0150  108.0619 H   0  0  0  0  0  0
```

```
  111.0563   94.3425  111.8345 H   0  0  0  0  0  0
  110.9800   95.3753  110.3882 H   0  0  0  0  0  0
  112.5364   94.6356  110.8879 H   0  0  0  0  0  0
  108.0333   95.7990   98.9776 H   0  0  0  0  0  0
  107.1767   94.2769   99.1423 H   0  0  0  0  0  0
  106.7732   96.8315  100.7074 H   0  0  0  0  0  0
  104.9722   94.4684  101.2688 H   0  0  0  0  0  0
  104.5658   95.3928   99.8690 H   0  0  0  0  0  0
  104.0555   97.4371   95.3315 H   0  0  0  0  0  0
  105.6667   97.7916   95.9248 H   0  0  0  0  0  0
  104.3923   97.3431   97.0501 H   0  0  0  0  0  0
  104.8510   95.7242   93.7192 H   0  0  0  0  0  0
  105.8623   94.3797   94.2101 H   0  0  0  0  0  0
  106.5256   96.0120   94.1586 H   0  0  0  0  0  0
  103.1496   94.9887   95.5168 H   0  0  0  0  0  0
  103.6136   94.8874   97.2039 H   0  0  0  0  0  0
  104.2096   93.6943   96.0509 H   0  0  0  0  0  0
  104.5931   96.5808  102.6566 H   0  0  0  0  0  0
  103.2252   96.1357  101.7102 H   0  0  0  0  0  0
  105.5584   98.7502  102.4219 H   0  0  0  0  0  0
  105.5837  100.9328  101.3258 H   0  0  0  0  0  0
  102.9347   99.4074   98.2778 H   0  0  0  0  0  0
  102.9065   97.2535   99.3905 H   0  0  0  0  0  0
  106.2923  101.2748   98.0592 H   0  0  0  0  0  0
  106.4675  102.0966   99.6061 H   0  0  0  0  0  0
  105.9626  102.9922   98.2008 H   0  0  0  0  0  0
  102.7406  102.5995  100.1698 H   0  0  0  0  0  0
  103.8013  103.7924   99.4427 H   0  0  0  0  0  0
  104.3282  102.8987  100.8558 H   0  0  0  0  0  0
  102.5432  101.6256   97.7761 H   0  0  0  0  0  0
  103.9753  101.0272   96.9478 H   0  0  0  0  0  0
  103.7353  102.7517   97.1629 H   0  0  0  0  0  0
  1  2  2  0  0  0
  2 15  1  0  0  0
  2 16  1  0  0  0
  2  3  2  0  0  0
  4 20  1  0  0  0
  4  5  1  0  0  0
  4 38  1  0  0  0
  5  6  1  0  0  0
  5  7  2  0  0  0
  6  8  1  0  0  0
  6 39  1  0  0  0
  8  9  1  0  0  0
  8 40  1  0  0  0
  8 41  1  0  0  0
  9 14  2  0  0  0
  9 10  1  0  0  0
 10 11  2  0  0  0
 10 42  1  0  0  0
 11 12  1  0  0  0
 11 43  1  0  0  0
 12 13  2  0  0  0
 12 15  1  0  0  0
 13 14  1  0  0  0
 13 44  1  0  0  0
 14 45  1  0  0  0
 15 46  1  0  0  0
 16 47  1  0  0  0
 16 48  1  0  0  0
 16 49  1  0  0  0
 17 22  2  0  0  0
 17 23  1  0  0  0
 17 18  1  0  0  0
 18 19  1  0  0  0
 19 20  1  0  0  0
 19 50  1  0  0  0
 19 51  1  0  0  0
 20 21  1  0  0  0
 20 52  1  0  0  0
 21 27  1  0  0  0
```

```
 21 53  1  0  0  0
 21 54  1  0  0  0
 23 24  1  0  0  0
 23 25  1  0  0  0
 23 26  1  0  0  0
 24 55  1  0  0  0
 24 56  1  0  0  0
 24 57  1  0  0  0
 25 58  1  0  0  0
 25 59  1  0  0  0
 25 60  1  0  0  0
 26 61  1  0  0  0
 26 62  1  0  0  0
 26 63  1  0  0  0
 27 28  1  0  0  0
 27 64  1  0  0  0
 27 65  1  0  0  0
 28 29  2  0  0  0
 28 33  1  0  0  0
 29 30  1  0  0  0
 29 66  1  0  0  0
 30 31  2  0  0  0
 30 67  1  0  0  0
 31 32  1  0  0  0
 31 34  1  0  0  0
 32 33  2  0  0  0
 32 68  1  0  0  0
 33 69  1  0  0  0
 34 35  1  0  0  0
 34 36  1  0  0  0
 34 37  1  0  0  0
 35 70  1  0  0  0
 35 71  1  0  0  0
 35 72  1  0  0  0
 36 73  1  0  0  0
 36 74  1  0  0  0
 36 75  1  0  0  0
 37 76  1  0  0  0
 37 77  1  0  0  0
 37 78  1  0  0  0
M  END
> <s_m_entry_id>
19

> <s_m_entry_name>
A_19R.1

> <s_m_Source_Path>
C:\Users\julio\OneDrive - Universidad de Talca\Escritorio\Nueva carpeta

> <s_m_Source_File>
A_19R.mol2

> <i_m_Source_File_Index>
1

$$$$
A_19S
                   3D
 Structure written by MMmdl.
 78 79  0  0  1  0            999 V2000
  109.5544   94.1887  109.4948 O   0  0  0  0  0  0
  110.7051   93.2858  109.6064 S   0  0  0  0  0  0
  110.5153   91.8945  110.0330 O   0  0  0  0  0  0
  107.6506   96.9246  102.2173 N   0  0  0  0  0  0
  108.4689   96.6744  103.2863 C   0  0  0  0  0  0
  109.7802   96.8135  102.9232 N   0  0  0  0  0  0
  107.9439   96.2530  104.8308 S   0  0  0  0  0  0
  110.8913   97.1496  103.8244 C   0  0  0  0  0  0
  111.0369   96.1451  104.9579 C   0  0  0  0  0  0
  110.6179   94.8254  104.7798 C   0  0  0  0  0  0
```

```
110.7793   93.8996  105.8074 C   0  0  0  0  0  0
111.3724   94.2756  107.0157 C   0  0  0  0  0  0
111.7970   95.6003  107.1871 C   0  0  0  0  0  0
111.6356   96.5277  106.1600 C   0  0  0  0  0  0
111.4918   93.2815  108.0037 N   0  0  0  0  0  0
111.9954   94.1192  110.7113 C   0  0  0  0  0  0
105.6226   98.7843  101.3283 C   0  0  0  0  0  0
106.1604   98.3405  100.1589 O   0  0  0  0  0  0
107.5618   98.1077  100.0340 C   0  0  0  0  0  0
107.9918   96.8212  100.7851 C   0  0  2  0  0  0
107.3709   95.5245  100.2009 C   0  0  0  0  0  0
106.2514   99.3875  102.2040 O   0  0  0  0  0  0
104.1050   98.4618  101.4706 C   0  0  0  0  0  0
103.6097   98.8860  102.8789 C   0  0  0  0  0  0
103.3044   99.2291  100.3856 C   0  0  0  0  0  0
103.8369   96.9405  101.2941 C   0  0  0  0  0  0
105.8271   95.4731  100.1748 C   0  0  0  0  0  0
105.2478   95.6358   98.7733 C   0  0  0  0  0  0
104.0531   96.3316   98.5841 C   0  0  0  0  0  0
103.5247   96.4822   97.3041 C   0  0  0  0  0  0
104.1789   95.9404   96.1832 C   0  0  0  0  0  0
105.3804   95.2433   96.3940 C   0  0  0  0  0  0
105.9103   95.0916   97.6727 C   0  0  0  0  0  0
103.6201   96.0877   94.7383 C   0  0  0  0  0  0
102.5626   97.2233   94.6951 C   0  0  0  0  0  0
102.9606   94.7560   94.2896 C   0  0  0  0  0  0
104.7090   96.4367   93.6830 C   0  0  0  0  0  0
106.7137   97.2432  102.4192 H   0  0  0  0  0  0
109.9755   96.9003  101.9361 H   0  0  0  0  0  0
111.8187   97.1834  103.2501 H   0  0  0  0  0  0
110.7608   98.1385  104.2666 H   0  0  0  0  0  0
110.1646   94.5138  103.8497 H   0  0  0  0  0  0
110.4435   92.8833  105.6554 H   0  0  0  0  0  0
112.2476   95.9402  108.1057 H   0  0  0  0  0  0
111.9726   97.5441  106.3052 H   0  0  0  0  0  0
112.0925   92.4985  107.7886 H   0  0  0  0  0  0
111.5948   94.2006  111.7217 H   0  0  0  0  0  0
112.2098   95.1140  110.3205 H   0  0  0  0  0  0
112.9077   93.5244  110.7267 H   0  0  0  0  0  0
108.1276   98.9690  100.3936 H   0  0  0  0  0  0
107.7864   98.0058   98.9703 H   0  0  0  0  0  0
109.0702   96.7015  100.6799 H   0  0  0  0  0  0
107.7566   94.6587  100.7427 H   0  0  0  0  0  0
107.7255   95.4075   99.1747 H   0  0  0  0  0  0
102.5582   98.6332  103.0244 H   0  0  0  0  0  0
103.6949   99.9610  103.0463 H   0  0  0  0  0  0
104.1672   98.3883  103.6747 H   0  0  0  0  0  0
102.2414   98.9862  100.4281 H   0  0  0  0  0  0
103.6442   98.9845   99.3774 H   0  0  0  0  0  0
103.3783  100.3118  100.4999 H   0  0  0  0  0  0
102.7758   96.7068  101.3946 H   0  0  0  0  0  0
104.3603   96.3408  102.0412 H   0  0  0  0  0  0
104.1436   96.5795  100.3105 H   0  0  0  0  0  0
105.4148   96.2494  100.8220 H   0  0  0  0  0  0
105.4694   94.5297  100.5881 H   0  0  0  0  0  0
103.5283   96.7582   99.4268 H   0  0  0  0  0  0
102.5990   97.0290   97.2172 H   0  0  0  0  0  0
105.9209   94.8083   95.5664 H   0  0  0  0  0  0
106.8361   94.5495   97.8020 H   0  0  0  0  0  0
102.9845   98.1761   95.0191 H   0  0  0  0  0  0
101.7021   97.0189   95.3329 H   0  0  0  0  0  0
102.1705   97.3734   93.6878 H   0  0  0  0  0  0
103.6699   93.9273   94.3210 H   0  0  0  0  0  0
102.5832   94.8152   93.2674 H   0  0  0  0  0  0
102.1121   94.4770   94.9151 H   0  0  0  0  0  0
105.4794   95.6667   93.6180 H   0  0  0  0  0  0
105.2020   97.3857   93.9003 H   0  0  0  0  0  0
104.2849   96.5327   92.6820 H   0  0  0  0  0  0
1  2  2  0  0  0
2 15  1  0  0  0
2 16  1  0  0  0
```

2	3	2	0	0	0
4	20	1	0	0	0
4	5	1	0	0	0
4	38	1	0	0	0
5	6	1	0	0	0
5	7	2	0	0	0
6	8	1	0	0	0
6	39	1	0	0	0
8	9	1	0	0	0
8	40	1	0	0	0
8	41	1	0	0	0
9	14	2	0	0	0
9	10	1	0	0	0
10	11	2	0	0	0
10	42	1	0	0	0
11	12	1	0	0	0
11	43	1	0	0	0
12	13	2	0	0	0
12	15	1	0	0	0
13	14	1	0	0	0
13	44	1	0	0	0
14	45	1	0	0	0
15	46	1	0	0	0
16	47	1	0	0	0
16	48	1	0	0	0
16	49	1	0	0	0
17	22	2	0	0	0
17	23	1	0	0	0
17	18	1	0	0	0
18	19	1	0	0	0
19	20	1	0	0	0
19	50	1	0	0	0
19	51	1	0	0	0
20	21	1	0	0	0
20	52	1	0	0	0
21	27	1	0	0	0
21	53	1	0	0	0
21	54	1	0	0	0
23	24	1	0	0	0
23	25	1	0	0	0
23	26	1	0	0	0
24	55	1	0	0	0
24	56	1	0	0	0
24	57	1	0	0	0
25	58	1	0	0	0
25	59	1	0	0	0
25	60	1	0	0	0
26	61	1	0	0	0
26	62	1	0	0	0
26	63	1	0	0	0
27	28	1	0	0	0
27	64	1	0	0	0
27	65	1	0	0	0
28	29	2	0	0	0
28	33	1	0	0	0
29	30	1	0	0	0
29	66	1	0	0	0
30	31	2	0	0	0
30	67	1	0	0	0
31	32	1	0	0	0
31	34	1	0	0	0
32	33	2	0	0	0
32	68	1	0	0	0
33	69	1	0	0	0
34	35	1	0	0	0
34	36	1	0	0	0
34	37	1	0	0	0
35	70	1	0	0	0
35	71	1	0	0	0
35	72	1	0	0	0
36	73	1	0	0	0

```
 36 74  1  0  0  0
 36 75  1  0  0  0
 37 76  1  0  0  0
 37 77  1  0  0  0
 37 78  1  0  0  0
M  END
> <s_m_entry_id>
20

> <s_m_entry_name>
A_19S.1

> <s_m_Source_Path>
C:\Users\julio\OneDrive - Universidad de Talca\Escritorio\Nueva carpeta

> <s_m_Source_File>
A_19S.mol2

> <i_m_Source_File_Index>
1

$$$$
A_20R
                    3D
 Structure written by MMmdl.
 72 73  0  0  1  0            999 V2000
  109.5958   93.9808  109.2271 O   0  0  0  0  0  0
  110.8410   93.2052  109.2355 S   0  0  0  0  0  0
  110.8945   91.8902  109.8851 O   0  0  0  0  0  0
  107.6415   97.0095  101.2094 N   0  0  0  0  0  0
  108.2499   96.4697  102.3110 C   0  0  0  0  0  0
  108.5008   95.1401  102.1110 N   0  0  0  0  0  0
  108.6389   97.3154  103.7152 S   0  0  0  0  0  0
  108.0097   94.0367  102.9481 C   0  0  0  0  0  0
  108.9042   93.7868  104.1529 C   0  0  0  0  0  0
  109.5393   92.5526  104.3155 C   0  0  0  0  0  0
  110.3357   92.3092  105.4362 C   0  0  0  0  0  0
  110.5018   93.2955  106.4187 C   0  0  0  0  0  0
  109.8516   94.5272  106.2563 C   0  0  0  0  0  0
  109.0556   94.7684  105.1359 C   0  0  0  0  0  0
  111.3167   93.0046  107.5274 N   0  0  0  0  0  0
  112.2089   94.3111  109.9340 C   0  0  0  0  0  0
  103.0938   96.8830  101.4948 C   0  0  0  0  0  0
  104.0777   95.9450  101.5715 O   0  0  0  0  0  0
  105.3952   96.2922  101.9928 C   0  0  0  0  0  0
  106.2190   96.9044  100.8305 C   0  0  1  0  0  0
  105.7053   98.2946  100.3705 C   0  0  0  0  0  0
  103.2009   98.0452  101.8994 O   0  0  0  0  0  0
  101.7844   96.3507  100.8392 C   0  0  0  0  0  0
  101.3807   95.0036  101.4959 C   0  0  0  0  0  0
  100.6148   97.3577  101.0269 C   0  0  0  0  0  0
  102.0101   96.1401   99.3188 C   0  0  0  0  0  0
  106.1434   98.7370   98.9567 C   0  0  0  0  0  0
  105.7925   97.7184   97.8770 C   0  0  0  0  0  0
  104.7097   97.9407   97.0228 C   0  0  0  0  0  0
  104.3886   97.0084   96.0377 C   0  0  0  0  0  0
  105.1430   95.8389   95.8892 C   0  0  0  0  0  0
  106.2359   95.6079   96.7462 C   0  0  0  0  0  0
  106.5496   96.5515   97.7326 C   0  0  0  0  0  0
  107.0914   94.3510   96.6281 C   0  0  0  0  0  0
  104.7575   94.8453   94.7987 C   0  0  0  0  0  0
  110.9434   91.1046  105.5597 F   0  0  0  0  0  0
  108.2154   97.5677  100.5937 H   0  0  0  0  0  0
  108.8723   94.8744  101.2102 H   0  0  0  0  0  0
  107.0006   94.2324  103.3142 H   0  0  0  0  0  0
  107.9539   93.1287  102.3452 H   0  0  0  0  0  0
  109.4192   91.7781  103.5714 H   0  0  0  0  0  0
  109.9428   95.3112  106.9911 H   0  0  0  0  0  0
  108.5589   95.7227  105.0354 H   0  0  0  0  0  0
  112.2896   92.8081  107.3403 H   0  0  0  0  0  0
  111.9881   94.5261  110.9795 H   0  0  0  0  0  0
```

```
 112.2377   95.2418  109.3672 H   0  0  0  0  0  0
 113.1706   93.8060  109.8614 H   0  0  0  0  0  0
 105.8798   95.3804  102.3475 H   0  0  0  0  0  0
 105.3588   96.9790  102.8404 H   0  0  0  0  0  0
 106.1257   96.2558   99.9593 H   0  0  0  0  0  0
 106.0636   99.0436  101.0798 H   0  0  0  0  0  0
 104.6163   98.3235  100.4418 H   0  0  0  0  0  0
 100.4423   94.6261  101.0866 H   0  0  0  0  0  0
 101.2384   95.1015  102.5738 H   0  0  0  0  0  0
 102.1243   94.2208  101.3364 H   0  0  0  0  0  0
  99.6935   96.9903  100.5719 H   0  0  0  0  0  0
 100.8257   98.3246  100.5662 H   0  0  0  0  0  0
 100.3919   97.5382  102.0802 H   0  0  0  0  0  0
 101.0956   95.8130   98.8216 H   0  0  0  0  0  0
 102.7639   95.3793   99.1101 H   0  0  0  0  0  0
 102.3287   97.0585   98.8219 H   0  0  0  0  0  0
 107.2190   98.9126   98.9277 H   0  0  0  0  0  0
 105.6848   99.6946   98.7039 H   0  0  0  0  0  0
 104.1126   98.8363   97.1177 H   0  0  0  0  0  0
 103.5464   97.2035   95.3895 H   0  0  0  0  0  0
 107.3880   96.3794   98.3929 H   0  0  0  0  0  0
 107.8941   94.3300   97.3666 H   0  0  0  0  0  0
 107.5551   94.2912   95.6429 H   0  0  0  0  0  0
 106.4863   93.4568   96.7809 H   0  0  0  0  0  0
 104.5087   93.8755   95.2308 H   0  0  0  0  0  0
 105.5774   94.7099   94.0927 H   0  0  0  0  0  0
 103.8895   95.1791   94.2284 H   0  0  0  0  0  0
  1  2  2  0  0  0
  2 15  1  0  0  0
  2 16  1  0  0  0
  2  3  2  0  0  0
  4 20  1  0  0  0
  4  5  1  0  0  0
  4 37  1  0  0  0
  5  6  1  0  0  0
  5  7  2  0  0  0
  6  8  1  0  0  0
  6 38  1  0  0  0
  8  9  1  0  0  0
  8 39  1  0  0  0
  8 40  1  0  0  0
  9 10  2  0  0  0
  9 14  1  0  0  0
 10 11  1  0  0  0
 10 41  1  0  0  0
 11 12  2  0  0  0
 11 36  1  0  0  0
 12 13  1  0  0  0
 12 15  1  0  0  0
 13 14  2  0  0  0
 13 42  1  0  0  0
 14 43  1  0  0  0
 15 44  1  0  0  0
 16 45  1  0  0  0
 16 46  1  0  0  0
 16 47  1  0  0  0
 17 22  2  0  0  0
 17 23  1  0  0  0
 17 18  1  0  0  0
 18 19  1  0  0  0
 19 20  1  0  0  0
 19 48  1  0  0  0
 19 49  1  0  0  0
 20 21  1  0  0  0
 20 50  1  0  0  0
 21 27  1  0  0  0
 21 51  1  0  0  0
 21 52  1  0  0  0
 23 24  1  0  0  0
 23 25  1  0  0  0
 23 26  1  0  0  0
```

```
 24 53  1  0  0  0
 24 54  1  0  0  0
 24 55  1  0  0  0
 25 56  1  0  0  0
 25 57  1  0  0  0
 25 58  1  0  0  0
 26 59  1  0  0  0
 26 60  1  0  0  0
 26 61  1  0  0  0
 27 28  1  0  0  0
 27 62  1  0  0  0
 27 63  1  0  0  0
 28 29  2  0  0  0
 28 33  1  0  0  0
 29 30  1  0  0  0
 29 64  1  0  0  0
 30 31  2  0  0  0
 30 65  1  0  0  0
 31 32  1  0  0  0
 31 35  1  0  0  0
 32 33  2  0  0  0
 32 34  1  0  0  0
 33 66  1  0  0  0
 34 67  1  0  0  0
 34 68  1  0  0  0
 34 69  1  0  0  0
 35 70  1  0  0  0
 35 71  1  0  0  0
 35 72  1  0  0  0
M  END
> <s_m_entry_id>
21

> <s_m_entry_name>
A_20R.1

> <s_m_Source_Path>
C:\Users\julio\OneDrive - Universidad de Talca\Escritorio\Nueva carpeta

> <s_m_Source_File>
A_20R.mol2

> <i_m_Source_File_Index>
1

$$$$
A_20S
                   3D
 Structure written by MMmdl.
 72 73  0  0  1  0            999 V2000
  109.8248   92.5935  109.4127 O   0  0  0  0  0  0
  111.2052   92.9601  109.7425 S   0  0  0  0  0  0
  112.1161   91.9710  110.3269 O   0  0  0  0  0  0
  108.2528   96.5919  101.0482 N   0  0  0  0  0  0
  107.7800   96.2805  102.2895 C   0  0  0  0  0  0
  108.8006   95.9473  103.1207 N   0  0  0  0  0  0
  106.1814   96.2599  102.7864 S   0  0  0  0  0  0
  109.0229   96.6165  104.4061 C   0  0  0  0  0  0
  109.8375   95.7803  105.3835 C   0  0  0  0  0  0
  110.8881   96.3733  106.0895 C   0  0  0  0  0  0
  111.6164   95.6387  107.0253 C   0  0  0  0  0  0
  111.2811   94.3031  107.2909 C   0  0  0  0  0  0
  110.2578   93.7004  106.5545 C   0  0  0  0  0  0
  109.5475   94.4276  105.5994 C   0  0  0  0  0  0
  111.9694   93.5632  108.2618 N   0  0  0  0  0  0
  111.2087   94.4718  110.8463 C   0  0  0  0  0  0
  104.7535   99.2275   99.9963 C   0  0  0  0  0  0
  105.6004   98.1644  100.1197 O   0  0  0  0  0  0
  106.8641   98.0381   99.4861 C   0  0  0  0  0  0
  107.5285   96.6658   99.7642 C   0  0  2  0  0  0
  106.7052   95.3926   99.4028 C   0  0  0  0  0  0
```

103.5397	99.0631	100.1303	O	0	0	0	0	0	0
105.3489	100.6648	99.7699	C	0	0	0	0	0	0
106.4487	101.0034	100.8224	C	0	0	0	0	0	0
105.8739	100.8320	98.3198	C	0	0	0	0	0	0
104.2149	101.7202	99.9462	C	0	0	0	0	0	0
106.8886	94.8778	97.9498	C	0	0	0	0	0	0
105.7657	95.1726	96.9567	C	0	0	0	0	0	0
105.6161	94.3427	95.8437	C	0	0	0	0	0	0
104.5964	94.5720	94.9214	C	0	0	0	0	0	0
103.7115	95.6411	95.0956	C	0	0	0	0	0	0
103.8609	96.4905	96.2043	C	0	0	0	0	0	0
104.8830	96.2454	97.1236	C	0	0	0	0	0	0
102.9315	97.6756	96.4277	C	0	0	0	0	0	0
102.5951	95.8606	94.0832	C	0	0	0	0	0	0
112.6504	96.2444	107.6526	F	0	0	0	0	0	0
109.2450	96.7578	100.9838	H	0	0	0	0	0	0
109.6196	95.5349	102.6888	H	0	0	0	0	0	0
109.5421	97.5565	104.2035	H	0	0	0	0	0	0
108.0842	96.8824	104.8962	H	0	0	0	0	0	0
111.1455	97.4097	105.9208	H	0	0	0	0	0	0
110.0129	92.6613	106.7150	H	0	0	0	0	0	0
108.7494	93.9471	105.0501	H	0	0	0	0	0	0
112.9734	93.4886	108.1962	H	0	0	0	0	0	0
110.7259	94.1977	111.7818	H	0	0	0	0	0	0
110.6576	95.2755	110.3613	H	0	0	0	0	0	0
112.2372	94.7736	111.0334	H	0	0	0	0	0	0
107.5467	98.8204	99.8058	H	0	0	0	0	0	0
106.7334	98.1514	98.4085	H	0	0	0	0	0	0
108.3763	96.6648	99.0796	H	0	0	0	0	0	0
105.6535	95.5006	99.6725	H	0	0	0	0	0	0
107.0469	94.5802	100.0501	H	0	0	0	0	0	0
106.4670	102.0640	101.0692	H	0	0	0	0	0	0
107.4555	100.7785	100.4758	H	0	0	0	0	0	0
106.3093	100.4741	101.7656	H	0	0	0	0	0	0
106.0524	101.8783	98.0661	H	0	0	0	0	0	0
105.1641	100.4483	97.5859	H	0	0	0	0	0	0
106.8231	100.3280	98.1473	H	0	0	0	0	0	0
104.5820	102.7389	99.8058	H	0	0	0	0	0	0
103.7712	101.6773	100.9430	H	0	0	0	0	0	0
103.4127	101.5776	99.2220	H	0	0	0	0	0	0
106.9825	93.7942	97.9994	H	0	0	0	0	0	0
107.8303	95.2208	97.5190	H	0	0	0	0	0	0
106.2913	93.5152	95.6873	H	0	0	0	0	0	0
104.4945	93.9116	94.0701	H	0	0	0	0	0	0
104.9720	96.8943	97.9765	H	0	0	0	0	0	0
103.1868	98.2219	97.3349	H	0	0	0	0	0	0
101.8989	97.3372	96.5204	H	0	0	0	0	0	0
103.0026	98.3744	95.5918	H	0	0	0	0	0	0
102.6622	96.8601	93.6580	H	0	0	0	0	0	0
101.6222	95.7508	94.5607	H	0	0	0	0	0	0
102.6401	95.1450	93.2629	H	0	0	0	0	0	0

1	2	2	0	0	0
2	15	1	0	0	0
2	16	1	0	0	0
2	3	2	0	0	0
4	20	1	0	0	0
4	5	1	0	0	0
4	37	1	0	0	0
5	6	1	0	0	0
5	7	2	0	0	0
6	8	1	0	0	0
6	38	1	0	0	0
8	9	1	0	0	0
8	39	1	0	0	0
8	40	1	0	0	0
9	10	2	0	0	0
9	14	1	0	0	0
10	11	1	0	0	0
10	41	1	0	0	0
11	12	2	0	0	0
11	36	1	0	0	0

```
 12 13  1  0  0  0
 12 15  1  0  0  0
 13 14  2  0  0  0
 13 42  1  0  0  0
 14 43  1  0  0  0
 15 44  1  0  0  0
 16 45  1  0  0  0
 16 46  1  0  0  0
 16 47  1  0  0  0
 17 22  2  0  0  0
 17 23  1  0  0  0
 17 18  1  0  0  0
 18 19  1  0  0  0
 19 20  1  0  0  0
 19 48  1  0  0  0
 19 49  1  0  0  0
 20 21  1  0  0  0
 20 50  1  0  0  0
 21 27  1  0  0  0
 21 51  1  0  0  0
 21 52  1  0  0  0
 23 24  1  0  0  0
 23 25  1  0  0  0
 23 26  1  0  0  0
 24 53  1  0  0  0
 24 54  1  0  0  0
 24 55  1  0  0  0
 25 56  1  0  0  0
 25 57  1  0  0  0
 25 58  1  0  0  0
 26 59  1  0  0  0
 26 60  1  0  0  0
 26 61  1  0  0  0
 27 28  1  0  0  0
 27 62  1  0  0  0
 27 63  1  0  0  0
 28 29  2  0  0  0
 28 33  1  0  0  0
 29 30  1  0  0  0
 29 64  1  0  0  0
 30 31  2  0  0  0
 30 65  1  0  0  0
 31 32  1  0  0  0
 31 35  1  0  0  0
 32 33  2  0  0  0
 32 34  1  0  0  0
 33 66  1  0  0  0
 34 67  1  0  0  0
 34 68  1  0  0  0
 34 69  1  0  0  0
 35 70  1  0  0  0
 35 71  1  0  0  0
 35 72  1  0  0  0
M  END
> <s_m_entry_id>
22

> <s_m_entry_name>
A_20S.1

> <s_m_Source_Path>
C:\Users\julio\OneDrive - Universidad de Talca\Escritorio\Nueva carpeta

> <s_m_Source_File>
A_20S.mol2

> <i_m_Source_File_Index>
1

$$$$
A_21S
```

```
                    3D
Structure written by MMmdl.
78 79  0  0  1  0            999 V2000
 110.6561   94.2855  110.4578 O   0  0  0  0  0  0
 111.3742   93.1108  109.9694 S   0  0  0  0  0  0
 110.7121   91.8087  109.8209 O   0  0  0  0  0  0
 107.8829   96.5983  101.2852 N   0  0  0  0  0  0
 107.8045   96.3197  102.6244 C   0  0  0  0  0  0
 109.0262   96.3283  103.2400 N   0  0  0  0  0  0
 106.3654   96.0343  103.4419 S   0  0  0  0  0  0
 109.2604   96.8504  104.6052 C   0  0  0  0  0  0
 109.9549   95.9296  105.6118 C   0  0  0  0  0  0
 110.1580   94.5625  105.3785 C   0  0  0  0  0  0
 110.8315   93.7766  106.3182 C   0  0  0  0  0  0
 111.3152   94.3436  107.5027 C   0  0  0  0  0  0
 111.0932   95.7033  107.7474 C   0  0  0  0  0  0
 110.4201   96.4867  106.8090 C   0  0  0  0  0  0
 112.0483   93.5530  108.4006 N   0  0  0  0  0  0
 112.9032   92.8588  111.0242 C   0  0  0  0  0  0
 105.1829   95.3981   97.6580 C   0  0  0  0  0  0
 106.1657   95.6763   98.5725 O   0  0  0  0  0  0
 105.8246   96.1289   99.8814 C   0  0  0  0  0  0
 106.7954   97.1904  100.4707 C   0  0  2  0  0  0
 107.3947   98.2089   99.4582 C   0  0  0  0  0  0
 103.9722   95.4015   97.9047 O   0  0  0  0  0  0
 105.7012   95.0761   96.2266 C   0  0  0  0  0  0
 104.8429   93.9615   95.5681 C   0  0  0  0  0  0
 105.5888   96.3530   95.3547 C   0  0  0  0  0  0
 107.1742   94.6168   96.2420 C   0  0  0  0  0  0
 106.4705   98.8410   98.3894 C   0  0  0  0  0  0
 105.4014   99.7925   98.9023 C   0  0  0  0  0  0
 104.1204   99.3224   99.1913 C   0  0  0  0  0  0
 103.1203  100.2110   99.5737 C   0  0  0  0  0  0
 103.3716  101.5884   99.6510 C   0  0  0  0  0  0
 104.6713  102.0443   99.3773 C   0  0  0  0  0  0
 105.6761  101.1555   99.0081 C   0  0  0  0  0  0
 102.2186  102.5571  100.0227 C   0  0  0  0  0  0
 101.8244  102.3487  101.5027 C   0  0  0  0  0  0
 100.9823  102.2912   99.1250 C   0  0  0  0  0  0
 102.5928  104.0495   99.8290 C   0  0  0  0  0  0
 111.0560   92.4680  106.0543 F   0  0  0  0  0  0
 108.7716   96.4672  100.8250 H   0  0  0  0  0  0
 109.8440   96.1279  102.6765 H   0  0  0  0  0  0
 109.8850   97.7384  104.4899 H   0  0  0  0  0  0
 108.3454   97.2173  105.0732 H   0  0  0  0  0  0
 109.8088   94.1007  104.4668 H   0  0  0  0  0  0
 111.4527   96.1750  108.6527 H   0  0  0  0  0  0
 110.2679   97.5360  107.0153 H   0  0  0  0  0  0
 112.4338   92.7432  107.9356 H   0  0  0  0  0  0
 112.5892   92.5890  112.0326 H   0  0  0  0  0  0
 113.4561   93.7917  111.0509 H   0  0  0  0  0  0
 113.5101   92.0696  110.5864 H   0  0  0  0  0  0
 105.7557   95.2603  100.5374 H   0  0  0  0  0  0
 104.8274   96.5679   99.8800 H   0  0  0  0  0  0
 106.1930   97.7962  101.1515 H   0  0  0  0  0  0
 108.2133   97.7188   98.9262 H   0  0  0  0  0  0
 107.8563   99.0233  100.0171 H   0  0  0  0  0  0
 105.1368   93.7945   94.5306 H   0  0  0  0  0  0
 103.7797   94.2114   95.5552 H   0  0  0  0  0  0
 104.9464   93.0007   96.0724 H   0  0  0  0  0  0
 105.8678   96.1513   94.3188 H   0  0  0  0  0  0
 106.2499   97.1482   95.7041 H   0  0  0  0  0  0
 104.5735   96.7534   95.3386 H   0  0  0  0  0  0
 107.4562   94.2674   95.2569 H   0  0  0  0  0  0
 107.3358   93.7927   96.9342 H   0  0  0  0  0  0
 107.8617   95.4191   96.5142 H   0  0  0  0  0  0
 105.9923   98.0826   97.7780 H   0  0  0  0  0  0
 107.0941   99.3931   97.6834 H   0  0  0  0  0  0
 103.8810   98.2749   99.0923 H   0  0  0  0  0  0
 102.1383   99.8174   99.7891 H   0  0  0  0  0  0
 104.9260  103.0899   99.4224 H   0  0  0  0  0  0
```

```
 106.6565  101.5381   98.7671 H   0  0  0  0  0  0
 102.6599  102.5535  102.1727 H   0  0  0  0  0  0
 101.4953  101.3267  101.6942 H   0  0  0  0  0  0
 101.0052  103.0077  101.7965 H   0  0  0  0  0  0
 101.2312  102.3843   98.0670 H   0  0  0  0  0  0
 100.1786  103.0007   99.3285 H   0  0  0  0  0  0
 100.5614  101.2979   99.2735 H   0  0  0  0  0  0
 102.8688  104.2599   98.7938 H   0  0  0  0  0  0
 103.4292  104.3461  100.4607 H   0  0  0  0  0  0
 101.7607  104.7087  100.0771 H   0  0  0  0  0  0
  1  2  2  0  0  0
  2 15  1  0  0  0
  2 16  1  0  0  0
  2  3  2  0  0  0
  4 20  1  0  0  0
  4  5  1  0  0  0
  4 39  1  0  0  0
  5  6  1  0  0  0
  5  7  2  0  0  0
  6  8  1  0  0  0
  6 40  1  0  0  0
  8  9  1  0  0  0
  8 41  1  0  0  0
  8 42  1  0  0  0
  9 14  2  0  0  0
  9 10  1  0  0  0
 10 11  2  0  0  0
 10 43  1  0  0  0
 11 12  1  0  0  0
 11 38  1  0  0  0
 12 13  2  0  0  0
 12 15  1  0  0  0
 13 14  1  0  0  0
 13 44  1  0  0  0
 14 45  1  0  0  0
 15 46  1  0  0  0
 16 47  1  0  0  0
 16 48  1  0  0  0
 16 49  1  0  0  0
 17 22  2  0  0  0
 17 23  1  0  0  0
 17 18  1  0  0  0
 18 19  1  0  0  0
 19 20  1  0  0  0
 19 50  1  0  0  0
 19 51  1  0  0  0
 20 21  1  0  0  0
 20 52  1  0  0  0
 21 27  1  0  0  0
 21 53  1  0  0  0
 21 54  1  0  0  0
 23 24  1  0  0  0
 23 25  1  0  0  0
 23 26  1  0  0  0
 24 55  1  0  0  0
 24 56  1  0  0  0
 24 57  1  0  0  0
 25 58  1  0  0  0
 25 59  1  0  0  0
 25 60  1  0  0  0
 26 61  1  0  0  0
 26 62  1  0  0  0
 26 63  1  0  0  0
 27 28  1  0  0  0
 27 64  1  0  0  0
 27 65  1  0  0  0
 28 29  2  0  0  0
 28 33  1  0  0  0
 29 30  1  0  0  0
 29 66  1  0  0  0
 30 31  2  0  0  0
```

```
 30 67  1  0  0  0
 31 32  1  0  0  0
 31 34  1  0  0  0
 32 33  2  0  0  0
 32 68  1  0  0  0
 33 69  1  0  0  0
 34 35  1  0  0  0
 34 36  1  0  0  0
 34 37  1  0  0  0
 35 70  1  0  0  0
 35 71  1  0  0  0
 35 72  1  0  0  0
 36 73  1  0  0  0
 36 74  1  0  0  0
 36 75  1  0  0  0
 37 76  1  0  0  0
 37 77  1  0  0  0
 37 78  1  0  0  0
M  END
> <s_m_entry_id>
23

> <s_m_entry_name>
A_21S.1

> <s_m_Source_Path>
C:\Users\julio\OneDrive - Universidad de Talca\Escritorio\Nueva carpeta

> <s_m_Source_File>
A_21S.mol2

> <i_m_Source_File_Index>
1

$$$$
A_26R
                    3D
 Structure written by MMmdl.
 72 73  0  0  1  0            999 V2000
  109.6258   92.7973  109.6091 O   0  0  0  0  0  0
  111.0641   92.8265  109.8962 S   0  0  0  0  0  0
  111.7335   91.6411  110.4442 O   0  0  0  0  0  0
  108.2570   96.1210  101.6055 N   0  0  0  0  0  0
  108.4961   95.9956  102.9481 C   0  0  0  0  0  0
  109.7018   96.5554  103.2699 N   0  0  0  0  0  0
  107.4584   95.2597  104.0528 S   0  0  0  0  0  0
  110.0549   97.1519  104.5657 C   0  0  0  0  0  0
  110.5027   96.1070  105.5770 C   0  0  0  0  0  0
  111.2617   96.4879  106.6851 C   0  0  0  0  0  0
  111.6972   95.5267  107.5937 C   0  0  0  0  0  0
  111.3853   94.1776  107.4048 C   0  0  0  0  0  0
  110.6400   93.7981  106.2800 C   0  0  0  0  0  0
  110.2040   94.7588  105.3700 C   0  0  0  0  0  0
  111.8684   93.2708  108.3655 N   0  0  0  0  0  0
  111.4070   94.3202  111.0060 C   0  0  0  0  0  0
  104.5638   99.3533  101.7995 C   0  0  0  0  0  0
  105.7359   98.8720  101.3032 O   0  0  0  0  0  0
  106.3386   97.6978  101.8432 C   0  0  0  0  0  0
  107.4755   97.1775  100.9297 C   0  0  2  0  0  0
  106.9387   96.7058   99.5699 C   0  0  0  0  0  0
  103.9773   98.8917  102.7838 O   0  0  0  0  0  0
  104.0111  100.5705  100.9996 C   0  0  0  0  0  0
  103.4518  100.0902   99.6339 C   0  0  0  0  0  0
  102.8808  101.2559  101.8121 C   0  0  0  0  0  0
  105.1279  101.6183  100.7311 C   0  0  0  0  0  0
  106.5439   97.6219   98.5889 C   0  0  0  0  0  0
  106.0471   97.1773   97.3659 C   0  0  0  0  0  0
  105.9276   95.8042   97.0897 C   0  0  0  0  0  0
  106.3245   94.8961   98.0847 C   0  0  0  0  0  0
  106.8219   95.3388   99.3076 C   0  0  0  0  0  0
  105.3770   95.2690   95.7363 C   0  0  0  0  0  0
```

```
 105.5655   93.7356   95.5511 C   0  0  0  0  0  0
 106.1092   95.9642   94.5572 C   0  0  0  0  0  0
 103.8549   95.5567   95.6353 C   0  0  0  0  0  0
 108.5716   95.3673  101.0113 H   0  0  0  0  0  0
 110.4765   96.3617  102.6515 H   0  0  0  0  0  0
 110.8568   97.8846  104.4621 H   0  0  0  0  0  0
 109.1897   97.6883  104.9591 H   0  0  0  0  0  0
 111.5147   97.5259  106.8471 H   0  0  0  0  0  0
 112.2805   95.8391  108.4486 H   0  0  0  0  0  0
 110.3944   92.7675  106.0802 H   0  0  0  0  0  0
 109.6345   94.4503  104.5050 H   0  0  0  0  0  0
 112.7637   92.8435  108.1757 H   0  0  0  0  0  0
 110.9245   94.1612  111.9704 H   0  0  0  0  0  0
 110.9994   95.2148  110.5351 H   0  0  0  0  0  0
 112.4806   94.4351  111.1490 H   0  0  0  0  0  0
 105.5846   96.9215  101.9868 H   0  0  0  0  0  0
 106.7389   97.9347  102.8306 H   0  0  0  0  0  0
 108.1395   98.0123  100.7041 H   0  0  0  0  0  0
 103.0963  100.9280   99.0319 H   0  0  0  0  0  0
 102.6066   99.4085   99.7439 H   0  0  0  0  0  0
 104.2097   99.5746   99.0411 H   0  0  0  0  0  0
 102.4979  102.1381  101.2965 H   0  0  0  0  0  0
 103.2266  101.5884  102.7927 H   0  0  0  0  0  0
 102.0257  100.5978  101.9757 H   0  0  0  0  0  0
 104.7476  102.4735  100.1700 H   0  0  0  0  0  0
 105.9505  101.2024  100.1463 H   0  0  0  0  0  0
 105.5512  102.0156  101.6556 H   0  0  0  0  0  0
 106.6244   98.6852   98.7635 H   0  0  0  0  0  0
 105.7621   97.9315   96.6493 H   0  0  0  0  0  0
 106.2517   93.8309   97.9229 H   0  0  0  0  0  0
 107.1154   94.6097  100.0492 H   0  0  0  0  0  0
 105.0470   93.1622   96.3213 H   0  0  0  0  0  0
 106.6174   93.4461   95.5698 H   0  0  0  0  0  0
 105.1692   93.3929   94.5936 H   0  0  0  0  0  0
 105.9505   97.0429   94.5432 H   0  0  0  0  0  0
 105.7670   95.5900   93.5909 H   0  0  0  0  0  0
 107.1868   95.7978   94.6010 H   0  0  0  0  0  0
 103.6280   96.6231   95.6538 H   0  0  0  0  0  0
 103.3048   95.0962   96.4576 H   0  0  0  0  0  0
 103.4297   95.1675   94.7087 H   0  0  0  0  0  0
  1  2  2  0  0  0
  2 15  1  0  0  0
  2 16  1  0  0  0
  2  3  2  0  0  0
  4 20  1  0  0  0
  4  5  1  0  0  0
  4 36  1  0  0  0
  5  6  1  0  0  0
  5  7  2  0  0  0
  6  8  1  0  0  0
  6 37  1  0  0  0
  8  9  1  0  0  0
  8 38  1  0  0  0
  8 39  1  0  0  0
  9 10  2  0  0  0
  9 14  1  0  0  0
 10 11  1  0  0  0
 10 40  1  0  0  0
 11 12  2  0  0  0
 11 41  1  0  0  0
 12 13  1  0  0  0
 12 15  1  0  0  0
 13 14  2  0  0  0
 13 42  1  0  0  0
 14 43  1  0  0  0
 15 44  1  0  0  0
 16 45  1  0  0  0
 16 46  1  0  0  0
 16 47  1  0  0  0
 17 18  1  0  0  0
 17 22  2  0  0  0
```

```
 17 23  1  0  0  0
 18 19  1  0  0  0
 19 20  1  0  0  0
 19 48  1  0  0  0
 19 49  1  0  0  0
 20 21  1  0  0  0
 20 50  1  0  0  0
 21 27  2  0  0  0
 21 31  1  0  0  0
 23 24  1  0  0  0
 23 25  1  0  0  0
 23 26  1  0  0  0
 24 51  1  0  0  0
 24 52  1  0  0  0
 24 53  1  0  0  0
 25 54  1  0  0  0
 25 55  1  0  0  0
 25 56  1  0  0  0
 26 57  1  0  0  0
 26 58  1  0  0  0
 26 59  1  0  0  0
 27 28  1  0  0  0
 27 60  1  0  0  0
 28 29  2  0  0  0
 28 61  1  0  0  0
 29 30  1  0  0  0
 29 32  1  0  0  0
 30 31  2  0  0  0
 30 62  1  0  0  0
 31 63  1  0  0  0
 32 33  1  0  0  0
 32 34  1  0  0  0
 32 35  1  0  0  0
 33 64  1  0  0  0
 33 65  1  0  0  0
 33 66  1  0  0  0
 34 67  1  0  0  0
 34 68  1  0  0  0
 34 69  1  0  0  0
 35 70  1  0  0  0
 35 71  1  0  0  0
 35 72  1  0  0  0
M  END
> <s_m_entry_id>
24

> <s_m_entry_name>
A_26R.1

> <s_m_Source_Path>
C:\Users\julio\OneDrive - Universidad de Talca\Escritorio\Nueva carpeta

> <s_m_Source_File>
A_26R.mol2

> <i_m_Source_File_Index>
1

$$$$
A_26S
                    3D
 Structure written by MMmdl.
 72 73  0  0  1  0            999 V2000
  109.6475   92.6969  109.6339 O   0  0  0  0  0  0
  111.0803   92.8573  109.9012 S   0  0  0  0  0  0
  111.8576   91.7414  110.4551 O   0  0  0  0  0  0
  107.3523   96.3320  101.6050 N   0  0  0  0  0  0
  107.7351   95.9817  102.8756 C   0  0  0  0  0  0
  109.0644   96.1937  103.1110 N   0  0  0  0  0  0
  106.6965   95.3922  104.0498 S   0  0  0  0  0  0
  109.6013   96.7422  104.3750 C   0  0  0  0  0  0
```

```
110.1555   95.7790  105.4354 C   0  0  0  0  0  0
110.1171   94.3852  105.2987 C   0  0  0  0  0  0
110.6559   93.5682  106.2955 C   0  0  0  0  0  0
111.2459   94.1372  107.4259 C   0  0  0  0  0  0
111.2822   95.5228  107.5686 C   0  0  0  0  0  0
110.7345   96.3376  106.5800 C   0  0  0  0  0  0
111.8576   93.3178  108.3821 N   0  0  0  0  0  0
111.3616   94.3679  110.9806 C   0  0  0  0  0  0
105.3731  100.2314  100.7436 C   0  0  0  0  0  0
105.5440   98.9112  100.5258 O   0  0  0  0  0  0
105.4744   97.9621  101.5850 C   0  0  0  0  0  0
105.9494   96.5406  101.1611 C   0  0  1  0  0  0
105.6832   96.1717   99.6809 C   0  0  0  0  0  0
105.4875  100.7833  101.8425 O   0  0  0  0  0  0
104.9835  101.0195   99.4574 C   0  0  0  0  0  0
105.4229  102.5034   99.5515 C   0  0  0  0  0  0
105.5878  100.4009   98.1657 C   0  0  0  0  0  0
103.4373  100.9741   99.3405 C   0  0  0  0  0  0
104.5218   96.6013   99.0275 C   0  0  0  0  0  0
104.3020   96.2999   97.6837 C   0  0  0  0  0  0
105.2329   95.5389   96.9560 C   0  0  0  0  0  0
106.3791   95.0843   97.6218 C   0  0  0  0  0  0
106.5908   95.3909   98.9620 C   0  0  0  0  0  0
105.0376   95.1991   95.4542 C   0  0  0  0  0  0
103.6711   95.6678   94.8884 C   0  0  0  0  0  0
105.1198   93.6669   95.2378 C   0  0  0  0  0  0
106.1469   95.8901   94.6249 C   0  0  0  0  0  0
108.0692   96.6597  100.9705 H   0  0  0  0  0  0
109.7159   96.0842  102.3430 H   0  0  0  0  0  0
110.4237   97.4019  104.0971 H   0  0  0  0  0  0
108.8623   97.3921  104.8481 H   0  0  0  0  0  0
109.6764   93.9289  104.4243 H   0  0  0  0  0  0
110.6321   92.4944  106.1766 H   0  0  0  0  0  0
111.7454   95.9738  108.4349 H   0  0  0  0  0  0
110.7715   97.4101  106.7051 H   0  0  0  0  0  0
112.7944   92.9768  108.2134 H   0  0  0  0  0  0
111.0559   94.1285  111.9985 H   0  0  0  0  0  0
110.7508   95.1864  110.6053 H   0  0  0  0  0  0
112.4169   94.6279  110.9596 H   0  0  0  0  0  0
104.4325   97.9051  101.9049 H   0  0  0  0  0  0
106.0354   98.3137  102.4548 H   0  0  0  0  0  0
105.3176   95.8206  101.6816 H   0  0  0  0  0  0
105.0691  103.0680   98.6881 H   0  0  0  0  0  0
106.5096  102.6129   99.5845 H   0  0  0  0  0  0
105.0194  102.9909  100.4395 H   0  0  0  0  0  0
105.1244  100.8287   97.2773 H   0  0  0  0  0  0
105.4293   99.3253   98.0883 H   0  0  0  0  0  0
106.6625  100.5809   98.0974 H   0  0  0  0  0  0
103.0845  101.5551   98.4865 H   0  0  0  0  0  0
102.9452  101.3855  100.2246 H   0  0  0  0  0  0
103.0697   99.9549   99.2050 H   0  0  0  0  0  0
103.7842   97.1909   99.5529 H   0  0  0  0  0  0
103.4016   96.6770   97.2248 H   0  0  0  0  0  0
107.1153   94.4858   97.1079 H   0  0  0  0  0  0
107.4560   94.9914   99.4514 H   0  0  0  0  0  0
103.5574   96.7506   94.9448 H   0  0  0  0  0  0
102.8350   95.2163   95.4227 H   0  0  0  0  0  0
103.5566   95.3993   93.8364 H   0  0  0  0  0  0
106.0998   93.2659   95.4946 H   0  0  0  0  0  0
104.9376   93.3936   94.1984 H   0  0  0  0  0  0
104.3820   93.1387   95.8425 H   0  0  0  0  0  0
107.1446   95.5627   94.9202 H   0  0  0  0  0  0
106.1162   96.9756   94.7444 H   0  0  0  0  0  0
106.0456   95.6764   93.5595 H   0  0  0  0  0  0
1  2  2  0  0  0
2 15  1  0  0  0
2 16  1  0  0  0
2  3  2  0  0  0
4 20  1  0  0  0
4  5  1  0  0  0
4 36  1  0  0  0
```

```
  5  6  1  0  0  0
  5  7  2  0  0  0
  6  8  1  0  0  0
  6 37  1  0  0  0
  8  9  1  0  0  0
  8 38  1  0  0  0
  8 39  1  0  0  0
  9 10  2  0  0  0
  9 14  1  0  0  0
 10 11  1  0  0  0
 10 40  1  0  0  0
 11 12  2  0  0  0
 11 41  1  0  0  0
 12 13  1  0  0  0
 12 15  1  0  0  0
 13 14  2  0  0  0
 13 42  1  0  0  0
 14 43  1  0  0  0
 15 44  1  0  0  0
 16 45  1  0  0  0
 16 46  1  0  0  0
 16 47  1  0  0  0
 17 18  1  0  0  0
 17 22  2  0  0  0
 17 23  1  0  0  0
 18 19  1  0  0  0
 19 20  1  0  0  0
 19 48  1  0  0  0
 19 49  1  0  0  0
 20 21  1  0  0  0
 20 50  1  0  0  0
 21 27  2  0  0  0
 21 31  1  0  0  0
 23 24  1  0  0  0
 23 25  1  0  0  0
 23 26  1  0  0  0
 24 51  1  0  0  0
 24 52  1  0  0  0
 24 53  1  0  0  0
 25 54  1  0  0  0
 25 55  1  0  0  0
 25 56  1  0  0  0
 26 57  1  0  0  0
 26 58  1  0  0  0
 26 59  1  0  0  0
 27 28  1  0  0  0
 27 60  1  0  0  0
 28 29  2  0  0  0
 28 61  1  0  0  0
 29 30  1  0  0  0
 29 32  1  0  0  0
 30 31  2  0  0  0
 30 62  1  0  0  0
 31 63  1  0  0  0
 32 33  1  0  0  0
 32 34  1  0  0  0
 32 35  1  0  0  0
 33 64  1  0  0  0
 33 65  1  0  0  0
 33 66  1  0  0  0
 34 67  1  0  0  0
 34 68  1  0  0  0
 34 69  1  0  0  0
 35 70  1  0  0  0
 35 71  1  0  0  0
 35 72  1  0  0  0
M  END
> <s_m_entry_id>
25

> <s_m_entry_name>
```

```
A_26S.1

> <s_m_Source_Path>
C:\Users\julio\OneDrive - Universidad de Talca\Escritorio\Nueva carpeta

> <s_m_Source_File>
A_26S.mol2

> <i_m_Source_File_Index>
1

$$$$
A_27R
                    3D
 Structure written by MMmdl.
 72 73  0  0  1  0            999 V2000
  109.4576   92.9825  109.6646 O   0  0  0  0  0  0
  110.9017   92.9912  109.9122 S   0  0  0  0  0  0
  111.5690   91.7921  110.4286 O   0  0  0  0  0  0
  107.7056   95.7936  101.1611 N   0  0  0  0  0  0
  107.6074   96.0768  102.4971 C   0  0  0  0  0  0
  108.8389   96.1215  103.0960 N   0  0  0  0  0  0
  106.1500   96.3419  103.2842 S   0  0  0  0  0  0
  109.1404   96.7040  104.4211 C   0  0  0  0  0  0
  109.8300   95.7942  105.4431 C   0  0  0  0  0  0
  110.0419   94.4265  105.2129 C   0  0  0  0  0  0
  110.6561   93.6308  106.1839 C   0  0  0  0  0  0
  111.0784   94.1900  107.3998 C   0  0  0  0  0  0
  110.8716   95.5540  107.6250 C   0  0  0  0  0  0
  110.2573   96.3486  106.6553 C   0  0  0  0  0  0
  111.6937   93.3956  108.3783 N   0  0  0  0  0  0
  111.3549   94.4381  111.0200 C   0  0  0  0  0  0
  106.2595   94.9945   96.5649 C   0  0  0  0  0  0
  105.9964   95.1300   97.8875 O   0  0  0  0  0  0
  107.0561   95.1855   98.8406 C   0  0  0  0  0  0
  106.6071   95.8388  100.1660 C   0  0  2  0  0  0
  106.0395   97.2445   99.9083 C   0  0  0  0  0  0
  107.3925   94.9279   96.0847 O   0  0  0  0  0  0
  104.9949   94.9566   95.6532 C   0  0  0  0  0  0
  104.7699   93.4953   95.1976 C   0  0  0  0  0  0
  103.7214   95.4667   96.3842 C   0  0  0  0  0  0
  105.2236   95.8433   94.4015 C   0  0  0  0  0  0
  110.8549   92.3163  105.9216 F   0  0  0  0  0  0
  106.8930   98.3426   99.7825 C   0  0  0  0  0  0
  106.3690   99.6141   99.5790 C   0  0  0  0  0  0
  104.9844   99.8197   99.4998 C   0  0  0  0  0  0
  104.1375   98.7080   99.6318 C   0  0  0  0  0  0
  104.6599   97.4342   99.8294 C   0  0  0  0  0  0
  104.3804  101.2361   99.2973 C   0  0  0  0  0  0
  103.3740  101.2296   98.1181 C   0  0  0  0  0  0
  105.4468  102.3157   98.9703 C   0  0  0  0  0  0
  103.6582  101.6737  100.5932 C   0  0  0  0  0  0
  108.5987   95.4556  100.8282 H   0  0  0  0  0  0
  109.6356   95.8050  102.5576 H   0  0  0  0  0  0
  109.8012   97.5550  104.2393 H   0  0  0  0  0  0
  108.2652   97.1354  104.9073 H   0  0  0  0  0  0
  109.7207   93.9623  104.2922 H   0  0  0  0  0  0
  111.1944   96.0117  108.5503 H   0  0  0  0  0  0
  110.1053   97.3999  106.8565 H   0  0  0  0  0  0
  112.4882   92.8293  108.1112 H   0  0  0  0  0  0
  111.0594   94.1915  112.0410 H   0  0  0  0  0  0
  110.8163   95.3287  110.6983 H   0  0  0  0  0  0
  112.4307   94.5941  110.9767 H   0  0  0  0  0  0
  107.8918   95.7549   98.4357 H   0  0  0  0  0  0
  107.4105   94.1714   99.0155 H   0  0  0  0  0  0
  105.8047   95.2110  100.5562 H   0  0  0  0  0  0
  103.8690   93.3944   94.5884 H   0  0  0  0  0  0
  104.6565   92.8127   96.0412 H   0  0  0  0  0  0
  105.6005   93.1226   94.5998 H   0  0  0  0  0  0
  102.8566   95.4645   95.7207 H   0  0  0  0  0  0
  103.8452   96.4913   96.7428 H   0  0  0  0  0  0
```

103.4615	94.8481	97.2441	H	0	0	0	0	0	0
104.3589	95.8234	93.7351	H	0	0	0	0	0	0
106.0815	95.5131	93.8142	H	0	0	0	0	0	0
105.3979	96.8858	94.6705	H	0	0	0	0	0	0
107.9647	98.2137	99.8412	H	0	0	0	0	0	0
107.0567	100.4367	99.4944	H	0	0	0	0	0	0
103.0659	98.8282	99.5913	H	0	0	0	0	0	0
103.9862	96.5945	99.9277	H	0	0	0	0	0	0
102.5060	100.5965	98.3122	H	0	0	0	0	0	0
103.8386	100.8705	97.1986	H	0	0	0	0	0	0
102.9870	102.2280	97.9150	H	0	0	0	0	0	0
106.1703	102.4359	99.7763	H	0	0	0	0	0	0
104.9863	103.2927	98.8274	H	0	0	0	0	0	0
105.9938	102.0827	98.0569	H	0	0	0	0	0	0
104.3445	101.6919	101.4412	H	0	0	0	0	0	0
102.8383	101.0019	100.8519	H	0	0	0	0	0	0
103.2340	102.6748	100.4973	H	0	0	0	0	0	0

1	2	2	0	0	0
2	15	1	0	0	0
2	16	1	0	0	0
2	3	2	0	0	0
4	20	1	0	0	0
4	5	1	0	0	0
4	37	1	0	0	0
5	6	1	0	0	0
5	7	2	0	0	0
6	8	1	0	0	0
6	38	1	0	0	0
8	9	1	0	0	0
8	39	1	0	0	0
8	40	1	0	0	0
9	10	2	0	0	0
9	14	1	0	0	0
10	11	1	0	0	0
10	41	1	0	0	0
11	12	2	0	0	0
11	27	1	0	0	0
12	13	1	0	0	0
12	15	1	0	0	0
13	14	2	0	0	0
13	42	1	0	0	0
14	43	1	0	0	0
15	44	1	0	0	0
16	45	1	0	0	0
16	46	1	0	0	0
16	47	1	0	0	0
17	22	2	0	0	0
17	23	1	0	0	0
17	18	1	0	0	0
18	19	1	0	0	0
19	20	1	0	0	0
19	48	1	0	0	0
19	49	1	0	0	0
20	21	1	0	0	0
20	50	1	0	0	0
21	28	2	0	0	0
21	32	1	0	0	0
23	24	1	0	0	0
23	25	1	0	0	0
23	26	1	0	0	0
24	51	1	0	0	0
24	52	1	0	0	0
24	53	1	0	0	0
25	54	1	0	0	0
25	55	1	0	0	0
25	56	1	0	0	0
26	57	1	0	0	0
26	58	1	0	0	0
26	59	1	0	0	0
28	29	1	0	0	0
28	60	1	0	0	0

```
 29 30  2  0  0  0
 29 61  1  0  0  0
 30 31  1  0  0  0
 30 33  1  0  0  0
 31 32  2  0  0  0
 31 62  1  0  0  0
 32 63  1  0  0  0
 33 34  1  0  0  0
 33 35  1  0  0  0
 33 36  1  0  0  0
 34 64  1  0  0  0
 34 65  1  0  0  0
 34 66  1  0  0  0
 35 67  1  0  0  0
 35 68  1  0  0  0
 35 69  1  0  0  0
 36 70  1  0  0  0
 36 71  1  0  0  0
 36 72  1  0  0  0
M  END
> <s_m_entry_id>
26

> <s_m_entry_name>
A_27R.1

> <s_m_Source_Path>
C:\Users\julio\OneDrive - Universidad de Talca\Escritorio\Nueva carpeta

> <s_m_Source_File>
A_27R.mol2

> <i_m_Source_File_Index>
1

$$$$
A_27S
                    3D
 Structure written by MMmdl.
 72 73  0  0  1  0            999 V2000
  109.6051   92.9927  109.1281 O   0  0  0  0  0  0
  111.0355   93.1112  109.4202 S   0  0  0  0  0  0
  111.7570   92.0023  110.0567 O   0  0  0  0  0  0
  107.7018   96.9788  101.2023 N   0  0  0  0  0  0
  107.8722   96.6884  102.5262 C   0  0  0  0  0  0
  109.1918   96.6462  102.8609 N   0  0  0  0  0  0
  106.6346   96.4425  103.6355 S   0  0  0  0  0  0
  109.7256   97.2080  104.1076 C   0  0  0  0  0  0
  110.2812   96.1922  105.0932 C   0  0  0  0  0  0
  111.4345   96.5180  105.8040 C   0  0  0  0  0  0
  111.9797   95.6092  106.7033 C   0  0  0  0  0  0
  111.3421   94.3851  106.9619 C   0  0  0  0  0  0
  110.1893   94.0589  106.2453 C   0  0  0  0  0  0
  109.6760   94.9456  105.2998 C   0  0  0  0  0  0
  111.8561   93.4768  107.8983 N   0  0  0  0  0  0
  111.3460   94.6638  110.4151 C   0  0  0  0  0  0
  103.9389   99.4728  100.9351 C   0  0  0  0  0  0
  105.1633   99.3660  101.5162 O   0  0  0  0  0  0
  106.3259   99.0157  100.7705 C   0  0  0  0  0  0
  106.4527   97.4717  100.5909 C   0  0  1  0  0  0
  106.2536   96.9180   99.1710 C   0  0  0  0  0  0
  103.7098   99.3450   99.7315 O   0  0  0  0  0  0
  102.8083   99.7789  101.9576 C   0  0  0  0  0  0
  102.8875   98.7884  103.1485 C   0  0  0  0  0  0
  102.9929  101.2264  102.4724 C   0  0  0  0  0  0
  101.4041   99.6601  101.3078 C   0  0  0  0  0  0
  113.1668   95.9198  107.2572 F   0  0  0  0  0  0
  105.8255   97.7155   98.1006 C   0  0  0  0  0  0
  105.5808   97.1499   96.8558 C   0  0  0  0  0  0
  105.7696   95.7816   96.6371 C   0  0  0  0  0  0
  106.2416   94.9938   97.6947 C   0  0  0  0  0  0
```

```
 106.4677   95.5536   98.9463 C   0  0  0  0  0  0
 105.4602   95.1942   95.2418 C   0  0  0  0  0  0
 103.9925   95.5012   94.8427 C   0  0  0  0  0  0
 105.6324   93.6564   95.1717 C   0  0  0  0  0  0
 106.4151   95.8365   94.2112 C   0  0  0  0  0  0
 108.5232   97.0291  100.6208 H   0  0  0  0  0  0
 109.8598   96.2890  102.1875 H   0  0  0  0  0  0
 110.5227   97.8973  103.8234 H   0  0  0  0  0  0
 108.9864   97.8122  104.6357 H   0  0  0  0  0  0
 111.9390   97.4568  105.6345 H   0  0  0  0  0  0
 109.7012   93.1060  106.4032 H   0  0  0  0  0  0
 108.7910   94.6627  104.7508 H   0  0  0  0  0  0
 112.8470   93.2754  107.8811 H   0  0  0  0  0  0
 110.7404   94.6065  111.3182 H   0  0  0  0  0  0
 111.0562   95.5276  109.8206 H   0  0  0  0  0  0
 112.4003   94.7107  110.6719 H   0  0  0  0  0  0
 107.1654   99.3730  101.3675 H   0  0  0  0  0  0
 106.4032   99.5859   99.8478 H   0  0  0  0  0  0
 105.6116   97.0117  101.1158 H   0  0  0  0  0  0
 102.1199   98.9962  103.8942 H   0  0  0  0  0  0
 103.8485   98.8350  103.6637 H   0  0  0  0  0  0
 102.7465   97.7557  102.8238 H   0  0  0  0  0  0
 102.2985  101.4578  103.2807 H   0  0  0  0  0  0
 102.8236  101.9608  101.6850 H   0  0  0  0  0  0
 104.0001  101.3904  102.8578 H   0  0  0  0  0  0
 100.6136   99.8706  102.0265 H   0  0  0  0  0  0
 101.2220   98.6573  100.9187 H   0  0  0  0  0  0
 101.2772  100.3599  100.4811 H   0  0  0  0  0  0
 105.6356   98.7708   98.2019 H   0  0  0  0  0  0
 105.2305   97.7913   96.0610 H   0  0  0  0  0  0
 106.4159   93.9338   97.5800 H   0  0  0  0  0  0
 106.7954   94.9050   99.7477 H   0  0  0  0  0  0
 103.8000   96.5699   94.7562 H   0  0  0  0  0  0
 103.2882   95.1051   95.5741 H   0  0  0  0  0  0
 103.7358   95.0610   93.8793 H   0  0  0  0  0  0
 106.6542   93.3516   95.3942 H   0  0  0  0  0  0
 105.4007   93.2831   94.1757 H   0  0  0  0  0  0
 104.9680   93.1370   95.8678 H   0  0  0  0  0  0
 107.4560   95.7851   94.5351 H   0  0  0  0  0  0
 106.1869   96.8874   94.0396 H   0  0  0  0  0  0
 106.3476   95.3311   93.2522 H   0  0  0  0  0  0
  1  2  2  0  0  0
  2 15  1  0  0  0
  2 16  1  0  0  0
  2  3  2  0  0  0
  4 20  1  0  0  0
  4  5  1  0  0  0
  4 37  1  0  0  0
  5  6  1  0  0  0
  5  7  2  0  0  0
  6  8  1  0  0  0
  6 38  1  0  0  0
  8  9  1  0  0  0
  8 39  1  0  0  0
  8 40  1  0  0  0
  9 10  2  0  0  0
  9 14  1  0  0  0
 10 11  1  0  0  0
 10 41  1  0  0  0
 11 12  2  0  0  0
 11 27  1  0  0  0
 12 13  1  0  0  0
 12 15  1  0  0  0
 13 14  2  0  0  0
 13 42  1  0  0  0
 14 43  1  0  0  0
 15 44  1  0  0  0
 16 45  1  0  0  0
 16 46  1  0  0  0
 16 47  1  0  0  0
 17 22  2  0  0  0
```

```
 17 23  1  0  0  0
 17 18  1  0  0  0
 18 19  1  0  0  0
 19 20  1  0  0  0
 19 48  1  0  0  0
 19 49  1  0  0  0
 20 21  1  0  0  0
 20 50  1  0  0  0
 21 28  2  0  0  0
 21 32  1  0  0  0
 23 24  1  0  0  0
 23 25  1  0  0  0
 23 26  1  0  0  0
 24 51  1  0  0  0
 24 52  1  0  0  0
 24 53  1  0  0  0
 25 54  1  0  0  0
 25 55  1  0  0  0
 25 56  1  0  0  0
 26 57  1  0  0  0
 26 58  1  0  0  0
 26 59  1  0  0  0
 28 29  1  0  0  0
 28 60  1  0  0  0
 29 30  2  0  0  0
 29 61  1  0  0  0
 30 31  1  0  0  0
 30 33  1  0  0  0
 31 32  2  0  0  0
 31 62  1  0  0  0
 32 63  1  0  0  0
 33 34  1  0  0  0
 33 35  1  0  0  0
 33 36  1  0  0  0
 34 64  1  0  0  0
 34 65  1  0  0  0
 34 66  1  0  0  0
 35 67  1  0  0  0
 35 68  1  0  0  0
 35 69  1  0  0  0
 36 70  1  0  0  0
 36 71  1  0  0  0
 36 72  1  0  0  0
M  END
> <s_m_entry_id>
27

> <s_m_entry_name>
A_27S.1

> <s_m_Source_Path>
C:\Users\julio\OneDrive - Universidad de Talca\Escritorio\Nueva carpeta

> <s_m_Source_File>
A_27S.mol2

> <i_m_Source_File_Index>
1

$$$$
A_32R
                    3D
 Structure written by MMmdl.
 75 76  0  0  1  0            999 V2000
  109.6261   92.6327  109.6018 O   0  0  0  0  0  0
  111.0595   92.7228  109.9005 S   0  0  0  0  0  0
  111.7736   91.5673  110.4559 O   0  0  0  0  0  0
  105.4031   99.8215  101.5513 C   0  0  0  0  0  0
  105.3676   98.4621  101.5056 O   0  0  0  0  0  0
  104.1292  100.4898  100.9535 C   0  0  0  0  0  0
  104.5064  101.8543  100.3183 C   0  0  0  0  0  0
```

103.4909	99.5955	99.8535	C	0	0	0	0	0	0
103.0797	100.7111	102.0752	C	0	0	0	0	0	0
106.3382	100.4832	102.0135	O	0	0	0	0	0	0
106.4858	97.6796	101.9215	C	0	0	0	0	0	0
107.5490	97.5722	100.7964	C	0	0	1	0	0	0
108.8284	96.8108	101.2391	C	0	0	0	0	0	0
106.9825	96.9820	99.4960	C	0	0	0	0	0	0
108.6426	95.8875	102.3721	N	0	0	0	0	0	0
107.8177	96.0693	103.4487	C	0	0	0	0	0	0
108.4292	96.8481	104.3922	N	0	0	0	0	0	0
106.2664	95.4284	103.5936	S	0	0	0	0	0	0
109.8795	96.9974	104.5747	C	0	0	0	0	0	0
110.4379	96.0041	105.5829	C	0	0	0	0	0	0
110.2116	94.6367	105.4150	C	0	0	0	0	0	0
110.6966	93.7308	106.3548	C	0	0	0	0	0	0
111.4069	94.1781	107.4721	C	0	0	0	0	0	0
111.6141	95.5534	107.6463	C	0	0	0	0	0	0
111.1289	96.4608	106.7071	C	0	0	0	0	0	0
111.8570	93.1985	108.3757	N	0	0	0	0	0	0
111.3301	94.2311	111.0106	C	0	0	0	0	0	0
106.9875	95.6015	99.2752	C	0	0	0	0	0	0
106.4772	95.0769	98.0906	C	0	0	0	0	0	0
105.9497	95.9131	97.0932	C	0	0	0	0	0	0
105.9534	97.2994	97.3262	C	0	0	0	0	0	0
106.4636	97.8260	98.5105	C	0	0	0	0	0	0
105.3890	95.2875	95.7834	C	0	0	0	0	0	0
103.8469	95.4588	95.7354	C	0	0	0	0	0	0
105.6845	93.7661	95.6448	C	0	0	0	0	0	0
106.0210	95.9887	94.5511	C	0	0	0	0	0	0
103.6413	102.3306	99.8541	H	0	0	0	0	0	0
105.2635	101.7467	99.5392	H	0	0	0	0	0	0
104.8938	102.5640	101.0513	H	0	0	0	0	0	0
102.5973	100.0577	99.4308	H	0	0	0	0	0	0
103.1849	98.6218	100.2408	H	0	0	0	0	0	0
104.1733	99.4170	99.0204	H	0	0	0	0	0	0
102.1579	101.1404	101.6794	H	0	0	0	0	0	0
103.4319	101.3956	102.8488	H	0	0	0	0	0	0
102.8064	99.7764	102.5686	H	0	0	0	0	0	0
106.1499	96.6856	102.2218	H	0	0	0	0	0	0
106.9262	98.1434	102.8056	H	0	0	0	0	0	0
107.8564	98.5957	100.5739	H	0	0	0	0	0	0
109.1779	96.1954	100.4094	H	0	0	0	0	0	0
109.6452	97.5070	101.4343	H	0	0	0	0	0	0
109.1717	95.0281	102.3313	H	0	0	0	0	0	0
107.8555	97.5334	104.8626	H	0	0	0	0	0	0
110.4160	96.8594	103.6345	H	0	0	0	0	0	0
110.0940	98.0132	104.9111	H	0	0	0	0	0	0
109.6628	94.2718	104.5586	H	0	0	0	0	0	0
110.5162	92.6751	106.2074	H	0	0	0	0	0	0
112.1361	95.9470	108.5035	H	0	0	0	0	0	0
111.2922	97.5184	106.8576	H	0	0	0	0	0	0
112.7226	92.7323	108.1448	H	0	0	0	0	0	0
110.8468	94.0532	111.9712	H	0	0	0	0	0	0
110.8891	95.1071	110.5348	H	0	0	0	0	0	0
112.3967	94.3913	111.1621	H	0	0	0	0	0	0
107.3877	94.9263	100.0175	H	0	0	0	0	0	0
106.4994	94.0052	97.9599	H	0	0	0	0	0	0
105.5661	98.0014	96.6045	H	0	0	0	0	0	0
106.4512	98.8970	98.6536	H	0	0	0	0	0	0
103.5418	96.5057	95.7257	H	0	0	0	0	0	0
103.3649	94.9909	96.5954	H	0	0	0	0	0	0
103.4165	95.0044	94.8414	H	0	0	0	0	0	0
106.7551	93.5555	95.6312	H	0	0	0	0	0	0
105.2792	93.3591	94.7168	H	0	0	0	0	0	0
105.2397	93.1860	96.4550	H	0	0	0	0	0	0
107.1090	95.9038	94.5562	H	0	0	0	0	0	0
105.7815	97.0516	94.5053	H	0	0	0	0	0	0
105.6708	95.5536	93.6136	H	0	0	0	0	0	0

1	2	2	0	0	0
2	26	1	0	0	0
2	27	1	0	0	0

2	3	2	0	0	0
4	6	1	0	0	0
4	5	1	0	0	0
4	10	2	0	0	0
5	11	1	0	0	0
6	7	1	0	0	0
6	8	1	0	0	0
6	9	1	0	0	0
7	37	1	0	0	0
7	38	1	0	0	0
7	39	1	0	0	0
8	40	1	0	0	0
8	41	1	0	0	0
8	42	1	0	0	0
9	43	1	0	0	0
9	44	1	0	0	0
9	45	1	0	0	0
11	12	1	0	0	0
11	46	1	0	0	0
11	47	1	0	0	0
12	13	1	0	0	0
12	14	1	0	0	0
12	48	1	0	0	0
13	15	1	0	0	0
13	49	1	0	0	0
13	50	1	0	0	0
14	28	2	0	0	0
14	32	1	0	0	0
15	16	1	0	0	0
15	51	1	0	0	0
16	17	1	0	0	0
16	18	2	0	0	0
17	19	1	0	0	0
17	52	1	0	0	0
19	20	1	0	0	0
19	53	1	0	0	0
19	54	1	0	0	0
20	21	2	0	0	0
20	25	1	0	0	0
21	22	1	0	0	0
21	55	1	0	0	0
22	23	2	0	0	0
22	56	1	0	0	0
23	24	1	0	0	0
23	26	1	0	0	0
24	25	2	0	0	0
24	57	1	0	0	0
25	58	1	0	0	0
26	59	1	0	0	0
27	60	1	0	0	0
27	61	1	0	0	0
27	62	1	0	0	0
28	29	1	0	0	0
28	63	1	0	0	0
29	30	2	0	0	0
29	64	1	0	0	0
30	31	1	0	0	0
30	33	1	0	0	0
31	32	2	0	0	0
31	65	1	0	0	0
32	66	1	0	0	0
33	34	1	0	0	0
33	35	1	0	0	0
33	36	1	0	0	0
34	67	1	0	0	0
34	68	1	0	0	0
34	69	1	0	0	0
35	70	1	0	0	0
35	71	1	0	0	0
35	72	1	0	0	0
36	73	1	0	0	0

```
 36 74  1  0  0  0
 36 75  1  0  0  0
M  END
> <s_m_entry_id>
28

> <s_m_entry_name>
A_32R.1

> <s_m_Source_Path>
C:\Users\julio\OneDrive - Universidad de Talca\Escritorio\Nueva carpeta

> <s_m_Source_File>
A_32R.mol2

> <i_m_Source_File_Index>
1

$$$$
A_32S
                    3D
 Structure written by MMmdl.
 75 76  0  0  1  0            999 V2000
  109.7456   92.7153  109.6725 O   0  0  0  0  0  0
  111.1641   92.7565  110.0441 S   0  0  0  0  0  0
  111.8566   91.5333  110.4591 O   0  0  0  0  0  0
  106.2904   99.7072   99.2552 C   0  0  0  0  0  0
  106.8196   99.0298  100.3032 O   0  0  0  0  0  0
  105.3710  100.8879   99.6896 C   0  0  0  0  0  0
  104.6722  100.6052  101.0461 C   0  0  0  0  0  0
  106.2328  102.1622   99.8244 C   0  0  0  0  0  0
  104.2715  101.1416   98.6290 C   0  0  0  0  0  0
  106.4867   99.4299   98.0712 O   0  0  0  0  0  0
  107.5188   97.7991  100.1368 C   0  0  0  0  0  0
  106.5381   96.5909  100.2398 C   0  0  2  0  0  0
  107.1095   95.2853  100.8631 C   0  0  0  0  0  0
  105.8949   96.3053   98.8844 C   0  0  0  0  0  0
  108.2557   95.4768  101.7673 N   0  0  0  0  0  0
  108.1764   95.7504  103.0987 C   0  0  0  0  0  0
  109.3191   96.3580  103.5413 N   0  0  0  0  0  0
  106.8427   95.3930  104.0480 S   0  0  0  0  0  0
  109.5535   96.8749  104.9020 C   0  0  0  0  0  0
  110.2103   95.9147  105.8905 C   0  0  0  0  0  0
  110.7967   96.4489  107.0406 C   0  0  0  0  0  0
  111.4106   95.6121  107.9662 C   0  0  0  0  0  0
  111.4412   94.2370  107.7435 C   0  0  0  0  0  0
  110.8644   93.6943  106.6001 C   0  0  0  0  0  0
  110.2581   94.5319  105.6687 C   0  0  0  0  0  0
  112.0513   93.3912  108.6690 N   0  0  0  0  0  0
  111.4530   94.0902  111.3362 C   0  0  0  0  0  0
  106.6806   95.8534   97.8205 C   0  0  0  0  0  0
  106.1124   95.6376   96.5707 C   0  0  0  0  0  0
  104.7484   95.8806   96.3493 C   0  0  0  0  0  0
  103.9651   96.3202   97.4305 C   0  0  0  0  0  0
  104.5285   96.5284   98.6834 C   0  0  0  0  0  0
  104.1466   95.6349   94.9451 C   0  0  0  0  0  0
  104.0389   94.1119   94.7190 C   0  0  0  0  0  0
  105.0386   96.2687   93.8422 C   0  0  0  0  0  0
  102.7299   96.2482   94.7756 C   0  0  0  0  0  0
  103.9760  101.4029  101.3114 H   0  0  0  0  0  0
  105.3870  100.5267  101.8698 H   0  0  0  0  0  0
  104.1006   99.6742  101.0204 H   0  0  0  0  0  0
  105.6288  102.9906  100.1928 H   0  0  0  0  0  0
  106.6579  102.4744   98.8666 H   0  0  0  0  0  0
  107.0578  102.0320  100.5286 H   0  0  0  0  0  0
  103.6316  101.9787   98.9076 H   0  0  0  0  0  0
  103.6262  100.2713   98.4931 H   0  0  0  0  0  0
  104.6977  101.3849   97.6571 H   0  0  0  0  0  0
  108.2420   97.7862  100.9497 H   0  0  0  0  0  0
  108.1054   97.7749   99.2171 H   0  0  0  0  0  0
  105.7522   96.8979  100.9343 H   0  0  0  0  0  0
```

```
  106.3116   94.7099  101.3338 H   0  0  0  0  0  0
  107.4544   94.6188  100.0758 H   0  0  0  0  0  0
  109.1786   95.4200  101.3527 H   0  0  0  0  0  0
  110.0982   96.4152  102.9001 H   0  0  0  0  0  0
  110.2193   97.7355  104.7948 H   0  0  0  0  0  0
  108.6390   97.2726  105.3449 H   0  0  0  0  0  0
  110.7750   97.5133  107.2251 H   0  0  0  0  0  0
  111.8555   96.0327  108.8544 H   0  0  0  0  0  0
  110.8903   92.6278  106.4257 H   0  0  0  0  0  0
  109.8182   94.0895  104.7866 H   0  0  0  0  0  0
  112.9514   92.9815  108.4554 H   0  0  0  0  0  0
  110.9722   93.7898  112.2652 H   0  0  0  0  0  0
  111.0119   95.0248  110.9878 H   0  0  0  0  0  0
  112.5248   94.2100  111.4924 H   0  0  0  0  0  0
  107.7380   95.6804   97.9559 H   0  0  0  0  0  0
  106.7466   95.2777   95.7748 H   0  0  0  0  0  0
  102.9094   96.5100   97.3208 H   0  0  0  0  0  0
  103.9100   96.8830   99.4940 H   0  0  0  0  0  0
  103.4561   93.6316   95.5071 H   0  0  0  0  0  0
  105.0180   93.6296   94.6999 H   0  0  0  0  0  0
  103.5431   93.8841   93.7781 H   0  0  0  0  0  0
  105.2156   97.3300   94.0281 H   0  0  0  0  0  0
  104.5815   96.1933   92.8569 H   0  0  0  0  0  0
  106.0142   95.7854   93.7599 H   0  0  0  0  0  0
  102.7274   97.3203   94.9752 H   0  0  0  0  0  0
  102.0010   95.7813   95.4391 H   0  0  0  0  0  0
  102.3507   96.1104   93.7602 H   0  0  0  0  0  0
  1  2  2  0  0  0
  2 26  1  0  0  0
  2 27  1  0  0  0
  2  3  2  0  0  0
  4  6  1  0  0  0
  4  5  1  0  0  0
  4 10  2  0  0  0
  5 11  1  0  0  0
  6  7  1  0  0  0
  6  8  1  0  0  0
  6  9  1  0  0  0
  7 37  1  0  0  0
  7 38  1  0  0  0
  7 39  1  0  0  0
  8 40  1  0  0  0
  8 41  1  0  0  0
  8 42  1  0  0  0
  9 43  1  0  0  0
  9 44  1  0  0  0
  9 45  1  0  0  0
 11 12  1  0  0  0
 11 46  1  0  0  0
 11 47  1  0  0  0
 12 13  1  0  0  0
 12 14  1  0  0  0
 12 48  1  0  0  0
 13 15  1  0  0  0
 13 49  1  0  0  0
 13 50  1  0  0  0
 14 28  2  0  0  0
 14 32  1  0  0  0
 15 16  1  0  0  0
 15 51  1  0  0  0
 16 17  1  0  0  0
 16 18  2  0  0  0
 17 19  1  0  0  0
 17 52  1  0  0  0
 19 20  1  0  0  0
 19 53  1  0  0  0
 19 54  1  0  0  0
 20 21  2  0  0  0
 20 25  1  0  0  0
 21 22  1  0  0  0
 21 55  1  0  0  0
```

```
 22 23  2  0  0  0
 22 56  1  0  0  0
 23 24  1  0  0  0
 23 26  1  0  0  0
 24 25  2  0  0  0
 24 57  1  0  0  0
 25 58  1  0  0  0
 26 59  1  0  0  0
 27 60  1  0  0  0
 27 61  1  0  0  0
 27 62  1  0  0  0
 28 29  1  0  0  0
 28 63  1  0  0  0
 29 30  2  0  0  0
 29 64  1  0  0  0
 30 31  1  0  0  0
 30 33  1  0  0  0
 31 32  2  0  0  0
 31 65  1  0  0  0
 32 66  1  0  0  0
 33 34  1  0  0  0
 33 35  1  0  0  0
 33 36  1  0  0  0
 34 67  1  0  0  0
 34 68  1  0  0  0
 34 69  1  0  0  0
 35 70  1  0  0  0
 35 71  1  0  0  0
 35 72  1  0  0  0
 36 73  1  0  0  0
 36 74  1  0  0  0
 36 75  1  0  0  0
M  END
> <s_m_entry_id>
29

> <s_m_entry_name>
A_32S.1

> <s_m_Source_Path>
C:\Users\julio\OneDrive - Universidad de Talca\Escritorio\Nueva carpeta

> <s_m_Source_File>
A_32S.mol2

> <i_m_Source_File_Index>
1

$$$$
A_33R
                    3D
 Structure written by MMmdl.
 75 76  0  0  1  0            999 V2000
  109.4578   92.9757  109.6983 O   0  0  0  0  0  0
  110.9040   92.9930  109.9402 S   0  0  0  0  0  0
  111.5856   91.7924  110.4378 O   0  0  0  0  0  0
  105.1097  100.2285  100.3112 C   0  0  0  0  0  0
  106.2771   99.6501   99.9452 O   0  0  0  0  0  0
  104.7935  100.3071  101.8452 C   0  0  0  0  0  0
  106.0297  100.8751  102.5852 C   0  0  0  0  0  0
  103.5935  101.2664  102.0736 C   0  0  0  0  0  0
  104.3914   98.9337  102.4496 C   0  0  0  0  0  0
  104.3858  100.7643   99.4716 O   0  0  0  0  0  0
  106.7071   98.4063  100.4785 C   0  0  0  0  0  0
  107.6311   97.6519   99.5018 C   0  0  1  0  0  0
  108.5812   96.7416  100.3317 C   0  0  0  0  0  0
  106.8360   96.9641   98.3704 C   0  0  0  0  0  0
  107.8899   95.8186  101.2573 N   0  0  0  0  0  0
  107.9241   95.9463  102.6163 C   0  0  0  0  0  0
  109.0720   96.4599  103.1610 N   0  0  0  0  0  0
  106.5948   95.4335  103.4770 S   0  0  0  0  0  0
```

109.2295	96.9237	104.5535	C	0	0	0	0	0	0
109.8633	95.9552	105.5569	C	0	0	0	0	0	0
110.0585	94.5950	105.2835	C	0	0	0	0	0	0
110.6557	93.7591	106.2325	C	0	0	0	0	0	0
111.0775	94.2715	107.4671	C	0	0	0	0	0	0
110.8838	95.6280	107.7370	C	0	0	0	0	0	0
110.2794	96.4606	106.7936	C	0	0	0	0	0	0
111.6894	93.4368	108.4156	N	0	0	0	0	0	0
111.3495	94.4232	111.0738	C	0	0	0	0	0	0
107.0454	95.6285	98.0011	C	0	0	0	0	0	0
106.2694	95.0349	97.0102	C	0	0	0	0	0	0
105.2638	95.7625	96.3563	C	0	0	0	0	0	0
105.0867	97.1050	96.6965	C	0	0	0	0	0	0
105.8774	97.7013	97.6654	C	0	0	0	0	0	0
104.3353	95.1269	95.2983	C	0	0	0	0	0	0
102.9359	94.9114	95.9185	C	0	0	0	0	0	0
104.8578	93.7616	94.8007	C	0	0	0	0	0	0
104.2031	96.0471	94.0540	C	0	0	0	0	0	0
110.8345	92.4511	105.9315	F	0	0	0	0	0	0
105.8562	100.9923	103.6524	H	0	0	0	0	0	0
106.3198	101.8375	102.1828	H	0	0	0	0	0	0
106.9114	100.2510	102.4890	H	0	0	0	0	0	0
103.3732	101.3932	103.1336	H	0	0	0	0	0	0
102.6802	100.8899	101.6085	H	0	0	0	0	0	0
103.7773	102.2576	101.6562	H	0	0	0	0	0	0
103.8343	99.0509	103.3792	H	0	0	0	0	0	0
105.2547	98.3233	102.7091	H	0	0	0	0	0	0
103.7585	98.3522	101.7768	H	0	0	0	0	0	0
105.8608	97.7581	100.7048	H	0	0	0	0	0	0
107.2411	98.5805	101.4083	H	0	0	0	0	0	0
108.2563	98.3897	98.9987	H	0	0	0	0	0	0
109.2467	96.1926	99.6725	H	0	0	0	0	0	0
109.2586	97.3921	100.8847	H	0	0	0	0	0	0
107.3046	95.0974	100.8585	H	0	0	0	0	0	0
109.9002	96.4675	102.5795	H	0	0	0	0	0	0
109.8746	97.8020	104.5093	H	0	0	0	0	0	0
108.2834	97.2846	104.9600	H	0	0	0	0	0	0
109.7460	94.1699	104.3416	H	0	0	0	0	0	0
111.2147	96.0529	108.6736	H	0	0	0	0	0	0
110.1415	97.5065	107.0279	H	0	0	0	0	0	0
112.4844	92.8822	108.1334	H	0	0	0	0	0	0
111.0558	94.1624	112.0887	H	0	0	0	0	0	0
110.8013	95.3120	110.7693	H	0	0	0	0	0	0
112.4228	94.5918	111.0274	H	0	0	0	0	0	0
107.8082	95.0247	98.4575	H	0	0	0	0	0	0
106.4619	93.9981	96.7785	H	0	0	0	0	0	0
104.3300	97.7091	96.2220	H	0	0	0	0	0	0
105.7085	98.7449	97.8579	H	0	0	0	0	0	0
102.4854	95.8533	96.2386	H	0	0	0	0	0	0
102.9856	94.2621	96.7954	H	0	0	0	0	0	0
102.2488	94.4484	95.2087	H	0	0	0	0	0	0
105.8638	93.8366	94.3869	H	0	0	0	0	0	0
104.2168	93.3925	94.0113	H	0	0	0	0	0	0
104.8614	93.0024	95.5839	H	0	0	0	0	0	0
105.1745	96.2580	93.6020	H	0	0	0	0	0	0
103.7360	97.0057	94.2789	H	0	0	0	0	0	0
103.5759	95.5955	93.2857	H	0	0	0	0	0	0

1	2	2	0	0	0
2	26	1	0	0	0
2	27	1	0	0	0
2	3	2	0	0	0
4	6	1	0	0	0
4	5	1	0	0	0
4	10	2	0	0	0
5	11	1	0	0	0
6	7	1	0	0	0
6	8	1	0	0	0
6	9	1	0	0	0
7	38	1	0	0	0
7	39	1	0	0	0
7	40	1	0	0	0

```
  8 41  1  0  0  0
  8 42  1  0  0  0
  8 43  1  0  0  0
  9 44  1  0  0  0
  9 45  1  0  0  0
  9 46  1  0  0  0
 11 12  1  0  0  0
 11 47  1  0  0  0
 11 48  1  0  0  0
 12 13  1  0  0  0
 12 14  1  0  0  0
 12 49  1  0  0  0
 13 15  1  0  0  0
 13 50  1  0  0  0
 13 51  1  0  0  0
 14 28  2  0  0  0
 14 32  1  0  0  0
 15 16  1  0  0  0
 15 52  1  0  0  0
 16 17  1  0  0  0
 16 18  2  0  0  0
 17 19  1  0  0  0
 17 53  1  0  0  0
 19 20  1  0  0  0
 19 54  1  0  0  0
 19 55  1  0  0  0
 20 21  2  0  0  0
 20 25  1  0  0  0
 21 22  1  0  0  0
 21 56  1  0  0  0
 22 23  2  0  0  0
 22 37  1  0  0  0
 23 24  1  0  0  0
 23 26  1  0  0  0
 24 25  2  0  0  0
 24 57  1  0  0  0
 25 58  1  0  0  0
 26 59  1  0  0  0
 27 60  1  0  0  0
 27 61  1  0  0  0
 27 62  1  0  0  0
 28 29  1  0  0  0
 28 63  1  0  0  0
 29 30  2  0  0  0
 29 64  1  0  0  0
 30 31  1  0  0  0
 30 33  1  0  0  0
 31 32  2  0  0  0
 31 65  1  0  0  0
 32 66  1  0  0  0
 33 34  1  0  0  0
 33 35  1  0  0  0
 33 36  1  0  0  0
 34 67  1  0  0  0
 34 68  1  0  0  0
 34 69  1  0  0  0
 35 70  1  0  0  0
 35 71  1  0  0  0
 35 72  1  0  0  0
 36 73  1  0  0  0
 36 74  1  0  0  0
 36 75  1  0  0  0
M  END
> <s_m_entry_id>
30

> <s_m_entry_name>
A_33R.1

> <s_m_Source_Path>
C:\Users\julio\OneDrive - Universidad de Talca\Escritorio\Nueva carpeta
```

```
> <s_m_Source_File>
A_33R.mol2

> <i_m_Source_File_Index>
1

$$$$
A_33S
                    3D
 Structure written by MMmdl.
 75 76  0  0  1  0            999 V2000
  109.6691   92.7161  109.8283 O   0  0  0  0  0  0
  111.0910   92.8951  110.1270 S   0  0  0  0  0  0
  111.8778   91.7893  110.6817 O   0  0  0  0  0  0
  106.6312   99.6603   99.2836 C   0  0  0  0  0  0
  106.9686   98.8571  100.3171 O   0  0  0  0  0  0
  105.7572  100.8700   99.7308 C   0  0  0  0  0  0
  105.3791  101.7699   98.5259 C   0  0  0  0  0  0
  104.4487  100.3425  100.3724 C   0  0  0  0  0  0
  106.5152  101.7333  100.7717 C   0  0  0  0  0  0
  106.9532   99.4639   98.1065 O   0  0  0  0  0  0
  107.5386   97.5674  100.1157 C   0  0  0  0  0  0
  106.4270   96.4783  100.0814 C   0  0  2  0  0  0
  106.8163   95.0745  100.6248 C   0  0  0  0  0  0
  105.8052   96.3630   98.6920 C   0  0  0  0  0  0
  107.8577   95.0808  101.6569 N   0  0  0  0  0  0
  107.7191   95.6064  102.9049 C   0  0  0  0  0  0
  108.9296   95.9657  103.4212 N   0  0  0  0  0  0
  106.2314   95.7920  103.6570 S   0  0  0  0  0  0
  109.1715   96.5928  104.7267 C   0  0  0  0  0  0
  109.8848   95.7101  105.7496 C   0  0  0  0  0  0
  110.1393   94.3497  105.5163 C   0  0  0  0  0  0
  110.8070   93.5791  106.4694 C   0  0  0  0  0  0
  111.2322   94.1608  107.6695 C   0  0  0  0  0  0
  110.9893   95.5147  107.8994 C   0  0  0  0  0  0
  110.3172   96.2857  106.9497 C   0  0  0  0  0  0
  111.8870   93.3866  108.6341 N   0  0  0  0  0  0
  111.3276   94.4008  111.2194 C   0  0  0  0  0  0
  104.5168   96.8378   98.4623 C   0  0  0  0  0  0
  103.9496   96.7279   97.1972 C   0  0  0  0  0  0
  104.6628   96.1328   96.1450 C   0  0  0  0  0  0
  105.9562   95.6563   96.3913 C   0  0  0  0  0  0
  106.5309   95.7822   97.6514 C   0  0  0  0  0  0
  104.0485   95.9561   94.7391 C   0  0  0  0  0  0
  105.0523   96.4423   93.6584 C   0  0  0  0  0  0
  102.7365   96.7572   94.5323 C   0  0  0  0  0  0
  103.7148   94.4587   94.5438 C   0  0  0  0  0  0
  111.0511   92.2749  106.2060 F   0  0  0  0  0  0
  104.7374  102.6002   98.8274 H   0  0  0  0  0  0
  104.8463  101.2163   97.7502 H   0  0  0  0  0  0
  106.2562  102.2135   98.0594 H   0  0  0  0  0  0
  103.8069  101.1589  100.7002 H   0  0  0  0  0  0
  104.6418   99.7284  101.2530 H   0  0  0  0  0  0
  103.8651   99.7366   99.6775 H   0  0  0  0  0  0
  105.9191  102.5931  101.0808 H   0  0  0  0  0  0
  107.4509  102.1255  100.3719 H   0  0  0  0  0  0
  106.7512  101.1705  101.6733 H   0  0  0  0  0  0
  108.2001   97.4101  100.9658 H   0  0  0  0  0  0
  108.1795   97.5200   99.2314 H   0  0  0  0  0  0
  105.6522   96.8300  100.7702 H   0  0  0  0  0  0
  105.9293   94.5570  100.9831 H   0  0  0  0  0  0
  107.1923   94.4449   99.8215 H   0  0  0  0  0  0
  108.7861   94.8046  101.3600 H   0  0  0  0  0  0
  109.7458   95.8045  102.8441 H   0  0  0  0  0  0
  109.8107   97.4594  104.5498 H   0  0  0  0  0  0
  108.2616   96.9945  105.1838 H   0  0  0  0  0  0
  109.8186   93.8695  104.6006 H   0  0  0  0  0  0
  111.3247   95.9859  108.8144 H   0  0  0  0  0  0
  110.1363   97.3332  107.1506 H   0  0  0  0  0  0
  112.7450   92.9061  108.3872 H   0  0  0  0  0  0
```

```
 111.0570   94.1360  112.2404 H   0  0  0  0  0  0
 110.6681   95.1920  110.8608 H   0  0  0  0  0  0
 112.3685   94.7110  111.1795 H   0  0  0  0  0  0
 103.9481   97.2921   99.2616 H   0  0  0  0  0  0
 102.9519   97.1074   97.0601 H   0  0  0  0  0  0
 106.5324   95.1795   95.6150 H   0  0  0  0  0  0
 107.5357   95.4157   97.8150 H   0  0  0  0  0  0
 105.9536   95.8291   93.6132 H   0  0  0  0  0  0
 105.3702   97.4694   93.8455 H   0  0  0  0  0  0
 104.6231   96.4243   92.6606 H   0  0  0  0  0  0
 101.9432   96.4307   95.2074 H   0  0  0  0  0  0
 102.3479   96.6230   93.5220 H   0  0  0  0  0  0
 102.8837   97.8272   94.6835 H   0  0  0  0  0  0
 103.1276   94.0681   95.3785 H   0  0  0  0  0  0
 104.6102   93.8399   94.4600 H   0  0  0  0  0  0
 103.1117   94.2905   93.6560 H   0  0  0  0  0  0
  1  2  2  0  0  0
  2 26  1  0  0  0
  2 27  1  0  0  0
  2  3  2  0  0  0
  4  6  1  0  0  0
  4  5  1  0  0  0
  4 10  2  0  0  0
  5 11  1  0  0  0
  6  7  1  0  0  0
  6  8  1  0  0  0
  6  9  1  0  0  0
  7 38  1  0  0  0
  7 39  1  0  0  0
  7 40  1  0  0  0
  8 41  1  0  0  0
  8 42  1  0  0  0
  8 43  1  0  0  0
  9 44  1  0  0  0
  9 45  1  0  0  0
  9 46  1  0  0  0
 11 12  1  0  0  0
 11 47  1  0  0  0
 11 48  1  0  0  0
 12 13  1  0  0  0
 12 14  1  0  0  0
 12 49  1  0  0  0
 13 15  1  0  0  0
 13 50  1  0  0  0
 13 51  1  0  0  0
 14 28  2  0  0  0
 14 32  1  0  0  0
 15 16  1  0  0  0
 15 52  1  0  0  0
 16 17  1  0  0  0
 16 18  2  0  0  0
 17 19  1  0  0  0
 17 53  1  0  0  0
 19 20  1  0  0  0
 19 54  1  0  0  0
 19 55  1  0  0  0
 20 21  2  0  0  0
 20 25  1  0  0  0
 21 22  1  0  0  0
 21 56  1  0  0  0
 22 23  2  0  0  0
 22 37  1  0  0  0
 23 24  1  0  0  0
 23 26  1  0  0  0
 24 25  2  0  0  0
 24 57  1  0  0  0
 25 58  1  0  0  0
 26 59  1  0  0  0
 27 60  1  0  0  0
 27 61  1  0  0  0
 27 62  1  0  0  0
```

```
 28 29  1  0  0  0
 28 63  1  0  0  0
 29 30  2  0  0  0
 29 64  1  0  0  0
 30 31  1  0  0  0
 30 33  1  0  0  0
 31 32  2  0  0  0
 31 65  1  0  0  0
 32 66  1  0  0  0
 33 34  1  0  0  0
 33 35  1  0  0  0
 33 36  1  0  0  0
 34 67  1  0  0  0
 34 68  1  0  0  0
 34 69  1  0  0  0
 35 70  1  0  0  0
 35 71  1  0  0  0
 35 72  1  0  0  0
 36 73  1  0  0  0
 36 74  1  0  0  0
 36 75  1  0  0  0
M  END
> <s_m_entry_id>
31

> <s_m_entry_name>
A_33S.1

> <s_m_Source_Path>
C:\Users\julio\OneDrive - Universidad de Talca\Escritorio\Nueva carpeta

> <s_m_Source_File>
A_33S.mol2

> <i_m_Source_File_Index>
1

$$$$
```

Coordinates of TRPV1 in PDB format in conformation for docking of compounds from series A.

```
ATOM      1  N   LYS B 571     132.999 108.143 111.393  1.00  0.00           N1+
ATOM      2  CA  LYS B 571     132.237 107.198 112.281  1.00  0.00           C
ATOM      3  C   LYS B 571     130.841 106.931 111.662  1.00  0.00           C
ATOM      4  O   LYS B 571     130.319 105.841 111.899  1.00  0.00           O
ATOM      5  CB  LYS B 571     132.181 107.669 113.753  1.00 20.00           C
ATOM      6  CG  LYS B 571     133.553 107.804 114.450  1.00 20.00           C
ATOM      7  CD  LYS B 571     134.373 106.500 114.501  1.00 20.00           C
ATOM      8  CE  LYS B 571     135.658 106.640 115.331  1.00 20.00           C
ATOM      9  NZ  LYS B 571     136.437 105.389 115.338  1.00 20.00           N1+
ATOM     10  H   LYS B 571     132.562 109.054 111.412  1.00  0.00           H
ATOM     11  HA  LYS B 571     132.804 106.270 112.199  1.00  0.00           H
ATOM     12  HB2 LYS B 571     131.668 108.631 113.809  1.00  0.00           H
ATOM     13  HB3 LYS B 571     131.569 106.981 114.336  1.00  0.00           H
ATOM     14  HG2 LYS B 571     134.138 108.583 113.960  1.00  0.00           H
ATOM     15  HG3 LYS B 571     133.386 108.159 115.468  1.00  0.00           H
ATOM     16  HD2 LYS B 571     133.757 105.697 114.908  1.00  0.00           H
ATOM     17  HD3 LYS B 571     134.647 106.196 113.491  1.00  0.00           H
ATOM     18  HE2 LYS B 571     136.283 107.438 114.929  1.00  0.00           H
ATOM     19  HE3 LYS B 571     135.417 106.909 116.361  1.00  0.00           H
ATOM     20  HZ1 LYS B 571     135.885 104.644 115.737  1.00  0.00           H
ATOM     21  HZ2 LYS B 571     136.702 105.146 114.395  1.00  0.00           H
ATOM     22  HZ3 LYS B 571     137.272 105.517 115.894  1.00  0.00           H
ATOM     23  H1  LYS B 571     133.028 107.779 110.451  1.00  0.00           H
ATOM     24  H2  LYS B 571     133.942 108.224 111.746  1.00  0.00           H
ATOM     25  N   MET B 572     130.260 107.871 110.887  1.00  0.00           N
ATOM     26  CA  MET B 572     128.999 107.686 110.156  1.00  0.00           C
ATOM     27  C   MET B 572     129.123 106.755 108.934  1.00  0.00           C
ATOM     28  O   MET B 572     128.113 106.195 108.522  1.00  0.00           O
ATOM     29  CB  MET B 572     128.429 109.054 109.725  1.00 20.00           C
ATOM     30  CG  MET B 572     128.109 110.008 110.884  1.00 20.00           C
ATOM     31  SD  MET B 572     126.807 109.437 112.008  1.00 20.00           S
ATOM     32  CE  MET B 572     126.837 110.808 113.186  1.00 20.00           C
ATOM     33  H   MET B 572     130.704 108.767 110.752  1.00  0.00           H
ATOM     34  HA  MET B 572     128.282 107.215 110.832  1.00  0.00           H
ATOM     35  HB2 MET B 572     129.126 109.545 109.044  1.00  0.00           H
ATOM     36  HB3 MET B 572     127.513 108.902 109.150  1.00  0.00           H
ATOM     37  HG2 MET B 572     129.006 110.210 111.470  1.00  0.00           H
ATOM     38  HG3 MET B 572     127.793 110.968 110.475  1.00  0.00           H
ATOM     39  HE1 MET B 572     126.007 110.729 113.887  1.00  0.00           H
ATOM     40  HE2 MET B 572     127.771 110.807 113.748  1.00  0.00           H
ATOM     41  HE3 MET B 572     126.761 111.755 112.654  1.00  0.00           H
ATOM     42  N   ILE B 573     130.340 106.575 108.393  1.00  0.00           N
ATOM     43  CA  ILE B 573     130.639 105.586 107.350  1.00  0.00           C
ATOM     44  C   ILE B 573     130.785 104.165 107.943  1.00  0.00           C
ATOM     45  O   ILE B 573     130.444 103.191 107.277  1.00  0.00           O
ATOM     46  CB  ILE B 573     131.929 105.975 106.561  1.00 20.00           C
ATOM     47  CG1 ILE B 573     131.698 107.282 105.764  1.00 20.00           C
ATOM     48  CG2 ILE B 573     132.475 104.876 105.617  1.00 20.00           C
ATOM     49  CD1 ILE B 573     132.983 107.922 105.216  1.00 20.00           C
ATOM     50  H   ILE B 573     131.126 107.096 108.750  1.00  0.00           H
ATOM     51  HA  ILE B 573     129.807 105.561 106.643  1.00  0.00           H
ATOM     52  HB  ILE B 573     132.710 106.178 107.296  1.00  0.00           H
ATOM     53 HG12 ILE B 573     131.016 107.083 104.936  1.00  0.00           H
ATOM     54 HG13 ILE B 573     131.195 108.024 106.383  1.00  0.00           H
ATOM     55 HG21 ILE B 573     133.337 105.227 105.049  1.00  0.00           H
ATOM     56 HG22 ILE B 573     132.814 103.993 106.157  1.00  0.00           H
ATOM     57 HG23 ILE B 573     131.717 104.560 104.899  1.00  0.00           H
ATOM     58 HD11 ILE B 573     132.870 109.003 105.124  1.00  0.00           H
ATOM     59 HD12 ILE B 573     133.838 107.736 105.866  1.00  0.00           H
ATOM     60 HD13 ILE B 573     133.224 107.534 104.226  1.00  0.00           H
ATOM     61  N   LEU B 574     131.249 104.070 109.199  1.00  0.00           N
```

```
ATOM     62  CA  LEU B 574     131.428 102.807 109.920  1.00  0.00           C
ATOM     63  C   LEU B 574     130.137 102.301 110.599  1.00  0.00           C
ATOM     64  O   LEU B 574     130.175 101.233 111.207  1.00  0.00           O
ATOM     65  CB  LEU B 574     132.584 102.975 110.934  1.00 20.00           C
ATOM     66  CG  LEU B 574     133.954 103.309 110.293  1.00 20.00           C
ATOM     67  CD1 LEU B 574     135.016 103.605 111.372  1.00 20.00           C
ATOM     68  CD2 LEU B 574     134.423 102.223 109.299  1.00 20.00           C
ATOM     69  H   LEU B 574     131.484 104.909 109.707  1.00  0.00           H
ATOM     70  HA  LEU B 574     131.707 102.030 109.207  1.00  0.00           H
ATOM     71  HB2 LEU B 574     132.316 103.758 111.645  1.00  0.00           H
ATOM     72  HB3 LEU B 574     132.693 102.063 111.524  1.00  0.00           H
ATOM     73  HG  LEU B 574     133.840 104.233 109.725  1.00  0.00           H
ATOM     74 HD11 LEU B 574     135.497 104.565 111.187  1.00  0.00           H
ATOM     75 HD12 LEU B 574     134.581 103.651 112.371  1.00  0.00           H
ATOM     76 HD13 LEU B 574     135.800 102.849 111.406  1.00  0.00           H
ATOM     77 HD21 LEU B 574     135.494 102.029 109.363  1.00  0.00           H
ATOM     78 HD22 LEU B 574     133.916 101.272 109.463  1.00  0.00           H
ATOM     79 HD23 LEU B 574     134.219 102.529 108.272  1.00  0.00           H
ATOM     80  N   ARG B 575     129.028 103.055 110.485  1.00  0.00           N
ATOM     81  CA  ARG B 575     127.718 102.719 111.042  1.00  0.00           C
ATOM     82  C   ARG B 575     126.643 102.862 109.954  1.00  0.00           C
ATOM     83  O   ARG B 575     126.082 101.854 109.524  1.00  0.00           O
ATOM     84  CB  ARG B 575     127.413 103.615 112.267  1.00 20.00           C
ATOM     85  CG  ARG B 575     128.326 103.359 113.477  1.00 20.00           C
ATOM     86  CD  ARG B 575     128.060 104.343 114.628  1.00 20.00           C
ATOM     87  NE  ARG B 575     128.754 103.946 115.863  1.00 20.00           N
ATOM     88  CZ  ARG B 575     130.081 103.956 116.082  1.00 20.00           C
ATOM     89  NH1 ARG B 575     130.943 104.387 115.151  1.00 20.00           N
ATOM     90  NH2 ARG B 575     130.551 103.527 117.260  1.00 20.00           N1+
ATOM     91  H   ARG B 575     129.075 103.916 109.958  1.00  0.00           H
ATOM     92  HA  ARG B 575     127.701 101.677 111.369  1.00  0.00           H
ATOM     93  HB2 ARG B 575     127.477 104.668 111.985  1.00  0.00           H
ATOM     94  HB3 ARG B 575     126.381 103.448 112.581  1.00  0.00           H
ATOM     95  HG2 ARG B 575     128.283 102.324 113.818  1.00  0.00           H
ATOM     96  HG3 ARG B 575     129.347 103.523 113.137  1.00  0.00           H
ATOM     97  HD2 ARG B 575     128.467 105.321 114.368  1.00  0.00           H
ATOM     98  HD3 ARG B 575     126.991 104.491 114.787  1.00  0.00           H
ATOM     99  HE  ARG B 575     128.155 103.585 116.593  1.00  0.00           H
ATOM    100 HH11 ARG B 575     131.937 104.377 115.326  1.00  0.00           H
ATOM    101 HH12 ARG B 575     130.595 104.705 114.257  1.00  0.00           H
ATOM    102 HH21 ARG B 575     131.542 103.524 117.449  1.00  0.00           H
ATOM    103 HH22 ARG B 575     129.916 103.196 117.973  1.00  0.00           H
ATOM    104  N   ASP B 576     126.375 104.113 109.543  1.00 30.00           N
ATOM    105  CA  ASP B 576     125.278 104.532 108.659  1.00 30.00           C
ATOM    106  C   ASP B 576     125.623 104.421 107.156  1.00 30.00           C
ATOM    107  O   ASP B 576     125.101 105.195 106.353  1.00 30.00           O
ATOM    108  CB  ASP B 576     124.713 105.942 109.007  1.00 20.00           C
ATOM    109  CG  ASP B 576     124.424 106.238 110.487  1.00 20.00           C
ATOM    110  OD1 ASP B 576     124.500 105.310 111.322  1.00 20.00           O
ATOM    111  OD2 ASP B 576     124.086 107.410 110.757  1.00 20.00           O1-
ATOM    112  H   ASP B 576     126.910 104.877 109.932  1.00  0.00           H
ATOM    113  HA  ASP B 576     124.455 103.831 108.813  1.00  0.00           H
ATOM    114  HB2 ASP B 576     125.417 106.699 108.658  1.00  0.00           H
ATOM    115  HB3 ASP B 576     123.777 106.085 108.469  1.00  0.00           H
ATOM    116  N   LEU B 577     126.461 103.438 106.795  1.00  0.00           N
ATOM    117  CA  LEU B 577     126.728 103.021 105.418  1.00  0.00           C
ATOM    118  C   LEU B 577     126.727 101.489 105.356  1.00  0.00           C
ATOM    119  O   LEU B 577     126.145 100.945 104.423  1.00  0.00           O
ATOM    120  CB  LEU B 577     128.017 103.690 104.886  1.00 20.00           C
ATOM    121  CG  LEU B 577     128.351 103.482 103.386  1.00 20.00           C
ATOM    122  CD1 LEU B 577     129.297 104.593 102.874  1.00 20.00           C
ATOM    123  CD2 LEU B 577     128.915 102.077 103.070  1.00 20.00           C
ATOM    124  H   LEU B 577     126.857 102.858 107.519  1.00  0.00           H
ATOM    125  HA  LEU B 577     125.902 103.351 104.784  1.00  0.00           H
ATOM    126  HB2 LEU B 577     127.901 104.760 105.066  1.00  0.00           H
ATOM    127  HB3 LEU B 577     128.870 103.384 105.480  1.00  0.00           H
ATOM    128  HG  LEU B 577     127.420 103.599 102.831  1.00  0.00           H
ATOM    129 HD11 LEU B 577     128.856 105.111 102.021  1.00  0.00           H
ATOM    130 HD12 LEU B 577     129.503 105.348 103.633  1.00  0.00           H
ATOM    131 HD13 LEU B 577     130.267 104.213 102.553  1.00  0.00           H
ATOM    132 HD21 LEU B 577     128.157 101.451 102.599  1.00  0.00           H
```

```
ATOM    133 HD22 LEU B 577     129.763 102.102 102.385  1.00  0.00           H
ATOM    134 HD23 LEU B 577     129.252 101.560 103.969  1.00  0.00           H
ATOM    135  N   CYS B 578     127.300 100.813 106.369  1.00  0.00           N
ATOM    136  CA  CYS B 578     127.218  99.359 106.547  1.00  0.00           C
ATOM    137  C   CYS B 578     125.758  98.882 106.715  1.00  0.00           C
ATOM    138  O   CYS B 578     125.352  97.921 106.060  1.00  0.00           O
ATOM    139  CB  CYS B 578     128.122  98.898 107.714  1.00 20.00           C
ATOM    140  SG  CYS B 578     128.085  97.100 107.988  1.00 20.00           S
ATOM    141  H   CYS B 578     127.786 101.319 107.094  1.00  0.00           H
ATOM    142  HA  CYS B 578     127.603  98.904 105.633  1.00  0.00           H
ATOM    143  HB2 CYS B 578     129.155  99.189 107.520  1.00  0.00           H
ATOM    144  HB3 CYS B 578     127.828  99.392 108.641  1.00  0.00           H
ATOM    145  HG  CYS B 578     128.690  96.768 106.843  1.00  0.00           H
ATOM    146  N   ARG B 579     125.003  99.593 107.567  1.00  0.00           N
ATOM    147  CA  ARG B 579     123.612  99.292 107.899  1.00  0.00           C
ATOM    148  C   ARG B 579     122.611  99.923 106.914  1.00  0.00           C
ATOM    149  O   ARG B 579     121.562  99.321 106.685  1.00  0.00           O
ATOM    150  CB  ARG B 579     123.343  99.719 109.359  1.00 20.00           C
ATOM    151  CG  ARG B 579     124.300  99.046 110.370  1.00 20.00           C
ATOM    152  CD  ARG B 579     124.058  99.420 111.839  1.00 20.00           C
ATOM    153  NE  ARG B 579     124.096 100.871 112.070  1.00 20.00           N
ATOM    154  CZ  ARG B 579     123.022 101.667 112.191  1.00 20.00           C
ATOM    155  NH1 ARG B 579     121.780 101.168 112.163  1.00 20.00           N
ATOM    156  NH2 ARG B 579     123.182 102.987 112.337  1.00 20.00           N1+
ATOM    157  H   ARG B 579     125.415 100.373 108.061  1.00  0.00           H
ATOM    158  HA  ARG B 579     123.471  98.213 107.839  1.00  0.00           H
ATOM    159  HB2 ARG B 579     123.439 100.803 109.431  1.00  0.00           H
ATOM    160  HB3 ARG B 579     122.312  99.484 109.629  1.00  0.00           H
ATOM    161  HG2 ARG B 579     124.103  97.978 110.284  1.00  0.00           H
ATOM    162  HG3 ARG B 579     125.356  99.169 110.126  1.00  0.00           H
ATOM    163  HD2 ARG B 579     123.224  98.895 112.304  1.00  0.00           H
ATOM    164  HD3 ARG B 579     124.944  99.081 112.376  1.00  0.00           H
ATOM    165  HE  ARG B 579     125.011 101.297 112.056  1.00  0.00           H
ATOM    166 HH11 ARG B 579     120.979 101.779 112.237  1.00  0.00           H
ATOM    167 HH12 ARG B 579     121.637 100.174 112.056  1.00  0.00           H
ATOM    168 HH21 ARG B 579     124.092 103.426 112.264  1.00  0.00           H
ATOM    169 HH22 ARG B 579     122.380 103.592 112.431  1.00  0.00           H
ATOM    170  N   PHE B 580     122.948 101.091 106.332  1.00  0.00           N
ATOM    171  CA  PHE B 580     122.114 101.774 105.337  1.00  0.00           C
ATOM    172  C   PHE B 580     122.156 101.117 103.947  1.00  0.00           C
ATOM    173  O   PHE B 580     121.092 100.855 103.387  1.00  0.00           O
ATOM    174  CB  PHE B 580     122.425 103.290 105.295  1.00 20.00           C
ATOM    175  CG  PHE B 580     121.755 104.076 104.173  1.00 20.00           C
ATOM    176  CD1 PHE B 580     120.384 104.403 104.248  1.00 20.00           C
ATOM    177  CD2 PHE B 580     122.448 104.337 102.971  1.00 20.00           C
ATOM    178  CE1 PHE B 580     119.747 105.009 103.173  1.00 20.00           C
ATOM    179  CE2 PHE B 580     121.795 104.943 101.906  1.00 20.00           C
ATOM    180  CZ  PHE B 580     120.451 105.279 102.007  1.00 20.00           C
ATOM    181  H   PHE B 580     123.829 101.527 106.558  1.00  0.00           H
ATOM    182  HA  PHE B 580     121.078 101.694 105.674  1.00  0.00           H
ATOM    183  HB2 PHE B 580     122.118 103.737 106.241  1.00  0.00           H
ATOM    184  HB3 PHE B 580     123.501 103.438 105.225  1.00  0.00           H
ATOM    185  HD1 PHE B 580     119.820 104.182 105.138  1.00  0.00           H
ATOM    186  HD2 PHE B 580     123.487 104.061 102.874  1.00  0.00           H
ATOM    187  HE1 PHE B 580     118.700 105.265 103.242  1.00  0.00           H
ATOM    188  HE2 PHE B 580     122.331 105.146 100.991  1.00  0.00           H
ATOM    189  HZ  PHE B 580     119.949 105.746 101.173  1.00  0.00           H
ATOM    190  N   MET B 581     123.369 100.875 103.412  1.00  0.00           N
ATOM    191  CA  MET B 581     123.558 100.331 102.062  1.00  0.00           C
ATOM    192  C   MET B 581     123.152  98.862 101.915  1.00  0.00           C
ATOM    193  O   MET B 581     122.818  98.485 100.797  1.00  0.00           O
ATOM    194  CB  MET B 581     124.987 100.557 101.526  1.00 20.00           C
ATOM    195  CG  MET B 581     125.370 102.034 101.338  1.00 20.00           C
ATOM    196  SD  MET B 581     124.383 102.961 100.124  1.00 20.00           S
ATOM    197  CE  MET B 581     124.843 102.121  98.585  1.00 20.00           C
ATOM    198  H   MET B 581     124.211 101.092 103.929  1.00  0.00           H
ATOM    199  HA  MET B 581     122.889 100.886 101.407  1.00  0.00           H
ATOM    200  HB2 MET B 581     125.714 100.059 102.165  1.00  0.00           H
ATOM    201  HB3 MET B 581     125.094 100.058 100.563  1.00  0.00           H
ATOM    202  HG2 MET B 581     125.299 102.555 102.292  1.00  0.00           H
ATOM    203  HG3 MET B 581     126.414 102.100 101.034  1.00  0.00           H
```

```
ATOM    204  HE1 MET B 581     124.396 102.634  97.733  1.00  0.00           H
ATOM    205  HE2 MET B 581     125.925 102.119  98.457  1.00  0.00           H
ATOM    206  HE3 MET B 581     124.488 101.091  98.583  1.00  0.00           H
ATOM    207  N   PHE B 582     123.120  98.073 103.007  1.00  0.00           N
ATOM    208  CA  PHE B 582     122.515  96.736 102.990  1.00  0.00           C
ATOM    209  C   PHE B 582     121.020  96.783 102.626  1.00  0.00           C
ATOM    210  O   PHE B 582     120.595  96.077 101.712  1.00  0.00           O
ATOM    211  CB  PHE B 582     122.785  95.966 104.306  1.00 20.00           C
ATOM    212  CG  PHE B 582     122.015  94.655 104.436  1.00 20.00           C
ATOM    213  CD1 PHE B 582     122.435  93.519 103.711  1.00 20.00           C
ATOM    214  CD2 PHE B 582     120.759  94.622 105.083  1.00 20.00           C
ATOM    215  CE1 PHE B 582     121.640  92.380 103.676  1.00 20.00           C
ATOM    216  CE2 PHE B 582     119.973  93.479 105.024  1.00 20.00           C
ATOM    217  CZ  PHE B 582     120.413  92.361 104.328  1.00 20.00           C
ATOM    218  H   PHE B 582     123.418  98.431 103.903  1.00  0.00           H
ATOM    219  HA  PHE B 582     123.006  96.173 102.193  1.00  0.00           H
ATOM    220  HB2 PHE B 582     123.850  95.738 104.368  1.00  0.00           H
ATOM    221  HB3 PHE B 582     122.563  96.597 105.169  1.00  0.00           H
ATOM    222  HD1 PHE B 582     123.366  93.538 103.164  1.00  0.00           H
ATOM    223  HD2 PHE B 582     120.390  95.497 105.598  1.00  0.00           H
ATOM    224  HE1 PHE B 582     121.967  91.513 103.122  1.00  0.00           H
ATOM    225  HE2 PHE B 582     119.006  93.466 105.503  1.00  0.00           H
ATOM    226  HZ  PHE B 582     119.793  91.478 104.283  1.00  0.00           H
ATOM    227  N   VAL B 583     120.266  97.651 103.319  1.00  0.00           N
ATOM    228  CA  VAL B 583     118.828  97.803 103.113  1.00  0.00           C
ATOM    229  C   VAL B 583     118.527  98.488 101.766  1.00  0.00           C
ATOM    230  O   VAL B 583     117.678  97.998 101.020  1.00  0.00           O
ATOM    231  CB  VAL B 583     118.159  98.606 104.266  1.00 20.00           C
ATOM    232  CG1 VAL B 583     116.642  98.818 104.077  1.00 20.00           C
ATOM    233  CG2 VAL B 583     118.403  97.938 105.633  1.00 20.00           C
ATOM    234  H   VAL B 583     120.685  98.251 104.015  1.00  0.00           H
ATOM    235  HA  VAL B 583     118.385  96.806 103.081  1.00  0.00           H
ATOM    236  HB  VAL B 583     118.615  99.598 104.306  1.00  0.00           H
ATOM    237 HG11 VAL B 583     116.209  99.342 104.925  1.00  0.00           H
ATOM    238 HG12 VAL B 583     116.407  99.408 103.193  1.00  0.00           H
ATOM    239 HG13 VAL B 583     116.119  97.868 103.978  1.00  0.00           H
ATOM    240 HG21 VAL B 583     117.921  98.495 106.437  1.00  0.00           H
ATOM    241 HG22 VAL B 583     118.010  96.921 105.654  1.00  0.00           H
ATOM    242 HG23 VAL B 583     119.464  97.888 105.877  1.00  0.00           H
ATOM    243  N   TYR B 584     119.267  99.562 101.443  1.00  0.00           N
ATOM    244  CA  TYR B 584     119.113 100.265 100.165  1.00  0.00           C
ATOM    245  C   TYR B 584     119.370  99.368  98.939  1.00  0.00           C
ATOM    246  O   TYR B 584     118.585  99.437  97.995  1.00  0.00           O
ATOM    247  CB  TYR B 584     119.956 101.564 100.125  1.00 20.00           C
ATOM    248  CG  TYR B 584     119.938 102.252  98.766  1.00 20.00           C
ATOM    249  CD1 TYR B 584     118.744 102.842  98.305  1.00 20.00           C
ATOM    250  CD2 TYR B 584     121.060 102.189  97.913  1.00 20.00           C
ATOM    251  CE1 TYR B 584     118.647 103.303  96.978  1.00 20.00           C
ATOM    252  CE2 TYR B 584     120.969 102.664  96.590  1.00 20.00           C
ATOM    253  CZ  TYR B 584     119.753 103.193  96.114  1.00 20.00           C
ATOM    254  OH  TYR B 584     119.637 103.575  94.810  1.00 20.00           O
ATOM    255  H   TYR B 584     119.992  99.902 102.062  1.00  0.00           H
ATOM    256  HA  TYR B 584     118.065 100.569 100.115  1.00  0.00           H
ATOM    257  HB2 TYR B 584     119.606 102.264 100.884  1.00  0.00           H
ATOM    258  HB3 TYR B 584     120.990 101.336 100.377  1.00  0.00           H
ATOM    259  HD1 TYR B 584     117.880 102.882  98.951  1.00  0.00           H
ATOM    260  HD2 TYR B 584     121.976 101.728  98.253  1.00  0.00           H
ATOM    261  HE1 TYR B 584     117.713 103.708  96.618  1.00  0.00           H
ATOM    262  HE2 TYR B 584     121.821 102.583  95.930  1.00  0.00           H
ATOM    263  HH  TYR B 584     118.730 103.768  94.557  1.00  0.00           H
ATOM    264  N   LEU B 585     120.429  98.537  98.975  1.00  0.00           N
ATOM    265  CA  LEU B 585     120.756  97.598  97.898  1.00  0.00           C
ATOM    266  C   LEU B 585     119.734  96.459  97.752  1.00  0.00           C
ATOM    267  O   LEU B 585     119.585  95.960  96.640  1.00  0.00           O
ATOM    268  CB  LEU B 585     122.186  97.040  98.059  1.00 20.00           C
ATOM    269  CG  LEU B 585     123.300  98.063  97.729  1.00 20.00           C
ATOM    270  CD1 LEU B 585     124.679  97.560  98.210  1.00 20.00           C
ATOM    271  CD2 LEU B 585     123.308  98.469  96.239  1.00 20.00           C
ATOM    272  H   LEU B 585     121.045  98.531  99.778  1.00  0.00           H
ATOM    273  HA  LEU B 585     120.721  98.158  96.966  1.00  0.00           H
ATOM    274  HB2 LEU B 585     122.298  96.658  99.074  1.00  0.00           H
```

ATOM	275	HB3	LEU	B	585	122.326	96.173	97.410	1.00	0.00	H
ATOM	276	HG	LEU	B	585	123.089	98.970	98.295	1.00	0.00	H
ATOM	277	HD11	LEU	B	585	125.160	98.304	98.844	1.00	0.00	H
ATOM	278	HD12	LEU	B	585	124.599	96.646	98.799	1.00	0.00	H
ATOM	279	HD13	LEU	B	585	125.362	97.345	97.389	1.00	0.00	H
ATOM	280	HD21	LEU	B	585	124.293	98.387	95.780	1.00	0.00	H
ATOM	281	HD22	LEU	B	585	122.630	97.860	95.641	1.00	0.00	H
ATOM	282	HD23	LEU	B	585	122.994	99.508	96.127	1.00	0.00	H
ATOM	283	N	VAL	B	586	119.039	96.075	98.833	1.00	0.00	N
ATOM	284	CA	VAL	B	586	117.922	95.131	98.764	1.00	0.00	C
ATOM	285	C	VAL	B	586	116.719	95.743	98.011	1.00	0.00	C
ATOM	286	O	VAL	B	586	116.168	95.080	97.130	1.00	0.00	O
ATOM	287	CB	VAL	B	586	117.510	94.597	100.170	1.00	20.00	C
ATOM	288	CG1	VAL	B	586	116.120	93.934	100.240	1.00	20.00	C
ATOM	289	CG2	VAL	B	586	118.563	93.607	100.700	1.00	20.00	C
ATOM	290	H	VAL	B	586	119.226	96.495	99.735	1.00	0.00	H
ATOM	291	HA	VAL	B	586	118.254	94.274	98.173	1.00	0.00	H
ATOM	292	HB	VAL	B	586	117.493	95.437	100.865	1.00	0.00	H
ATOM	293	HG11	VAL	B	586	115.980	93.405	101.181	1.00	0.00	H
ATOM	294	HG12	VAL	B	586	115.316	94.666	100.160	1.00	0.00	H
ATOM	295	HG13	VAL	B	586	115.995	93.205	99.439	1.00	0.00	H
ATOM	296	HG21	VAL	B	586	118.373	93.339	101.740	1.00	0.00	H
ATOM	297	HG22	VAL	B	586	118.570	92.686	100.116	1.00	0.00	H
ATOM	298	HG23	VAL	B	586	119.569	94.023	100.656	1.00	0.00	H
ATOM	299	N	PHE	B	587	116.384	97.010	98.304	1.00	0.00	N
ATOM	300	CA	PHE	B	587	115.358	97.760	97.571	1.00	0.00	C
ATOM	301	C	PHE	B	587	115.733	97.942	96.090	1.00	0.00	C
ATOM	302	O	PHE	B	587	114.948	97.596	95.208	1.00	0.00	O
ATOM	303	CB	PHE	B	587	115.094	99.139	98.221	1.00	20.00	C
ATOM	304	CG	PHE	B	587	114.597	99.169	99.659	1.00	20.00	C
ATOM	305	CD1	PHE	B	587	113.864	98.097	100.218	1.00	20.00	C
ATOM	306	CD2	PHE	B	587	114.695	100.374	100.389	1.00	20.00	C
ATOM	307	CE1	PHE	B	587	113.309	98.219	101.483	1.00	20.00	C
ATOM	308	CE2	PHE	B	587	114.128	100.475	101.652	1.00	20.00	C
ATOM	309	CZ	PHE	B	587	113.447	99.398	102.203	1.00	20.00	C
ATOM	310	H	PHE	B	587	116.859	97.494	99.060	1.00	0.00	H
ATOM	311	HA	PHE	B	587	114.434	97.180	97.586	1.00	0.00	H
ATOM	312	HB2	PHE	B	587	115.996	99.751	98.171	1.00	0.00	H
ATOM	313	HB3	PHE	B	587	114.351	99.670	97.623	1.00	0.00	H
ATOM	314	HD1	PHE	B	587	113.714	97.175	99.679	1.00	0.00	H
ATOM	315	HD2	PHE	B	587	115.197	101.232	99.963	1.00	0.00	H
ATOM	316	HE1	PHE	B	587	112.747	97.394	101.890	1.00	0.00	H
ATOM	317	HE2	PHE	B	587	114.200	101.401	102.202	1.00	0.00	H
ATOM	318	HZ	PHE	B	587	113.001	99.487	103.183	1.00	0.00	H
ATOM	319	N	LEU	B	588	116.953	98.433	95.846	1.00	0.00	N
ATOM	320	CA	LEU	B	588	117.494	98.709	94.514	1.00	0.00	C
ATOM	321	C	LEU	B	588	117.531	97.453	93.633	1.00	0.00	C
ATOM	322	O	LEU	B	588	116.917	97.474	92.572	1.00	0.00	O
ATOM	323	CB	LEU	B	588	118.862	99.423	94.628	1.00	20.00	C
ATOM	324	CG	LEU	B	588	119.641	99.664	93.307	1.00	20.00	C
ATOM	325	CD1	LEU	B	588	118.834	100.458	92.256	1.00	20.00	C
ATOM	326	CD2	LEU	B	588	121.018	100.300	93.592	1.00	20.00	C
ATOM	327	H	LEU	B	588	117.582	98.588	96.624	1.00	0.00	H
ATOM	328	HA	LEU	B	588	116.804	99.404	94.035	1.00	0.00	H
ATOM	329	HB2	LEU	B	588	118.718	100.378	95.136	1.00	0.00	H
ATOM	330	HB3	LEU	B	588	119.497	98.831	95.283	1.00	0.00	H
ATOM	331	HG	LEU	B	588	119.855	98.689	92.868	1.00	0.00	H
ATOM	332	HD11	LEU	B	588	119.383	101.305	91.846	1.00	0.00	H
ATOM	333	HD12	LEU	B	588	118.569	99.818	91.414	1.00	0.00	H
ATOM	334	HD13	LEU	B	588	117.906	100.855	92.662	1.00	0.00	H
ATOM	335	HD21	LEU	B	588	121.113	101.304	93.183	1.00	0.00	H
ATOM	336	HD22	LEU	B	588	121.215	100.378	94.661	1.00	0.00	H
ATOM	337	HD23	LEU	B	588	121.819	99.697	93.162	1.00	0.00	H
ATOM	338	N	PHE	B	589	118.204	96.384	94.096	1.00	0.00	N
ATOM	339	CA	PHE	B	589	118.348	95.121	93.365	1.00	0.00	C
ATOM	340	C	PHE	B	589	117.020	94.356	93.205	1.00	0.00	C
ATOM	341	O	PHE	B	589	116.833	93.705	92.177	1.00	0.00	O
ATOM	342	CB	PHE	B	589	119.432	94.254	94.042	1.00	20.00	C
ATOM	343	CG	PHE	B	589	119.855	93.015	93.272	1.00	20.00	C
ATOM	344	CD1	PHE	B	589	120.761	93.138	92.197	1.00	20.00	C
ATOM	345	CD2	PHE	B	589	119.240	91.766	93.511	1.00	20.00	C

ATOM	346	CE1	PHE	B	589	121.086	92.028	91.432	1.00	20.00	C
ATOM	347	CE2	PHE	B	589	119.574	90.670	92.726	1.00	20.00	C
ATOM	348	CZ	PHE	B	589	120.497	90.798	91.696	1.00	20.00	C
ATOM	349	H	PHE	B	589	118.666	96.432	94.995	1.00	0.00	H
ATOM	350	HA	PHE	B	589	118.707	95.373	92.366	1.00	0.00	H
ATOM	351	HB2	PHE	B	589	120.328	94.859	94.190	1.00	0.00	H
ATOM	352	HB3	PHE	B	589	119.104	93.951	95.038	1.00	0.00	H
ATOM	353	HD1	PHE	B	589	121.208	94.095	91.970	1.00	0.00	H
ATOM	354	HD2	PHE	B	589	118.505	91.659	94.296	1.00	0.00	H
ATOM	355	HE1	PHE	B	589	121.796	92.123	90.625	1.00	0.00	H
ATOM	356	HE2	PHE	B	589	119.107	89.715	92.905	1.00	0.00	H
ATOM	357	HZ	PHE	B	589	120.748	89.940	91.090	1.00	0.00	H
ATOM	358	N	GLY	B	590	116.123	94.464	94.202	1.00	0.00	N
ATOM	359	CA	GLY	B	590	114.825	93.793	94.224	1.00	0.00	C
ATOM	360	C	GLY	B	590	113.881	94.360	93.160	1.00	0.00	C
ATOM	361	O	GLY	B	590	113.339	93.595	92.361	1.00	0.00	O
ATOM	362	H	GLY	B	590	116.357	95.019	95.015	1.00	0.00	H
ATOM	363	HA2	GLY	B	590	114.965	92.726	94.061	1.00	0.00	H
ATOM	364	HA3	GLY	B	590	114.374	93.923	95.207	1.00	0.00	H
ATOM	365	N	PHE	B	591	113.745	95.692	93.119	1.00	0.00	N
ATOM	366	CA	PHE	B	591	112.952	96.392	92.110	1.00	0.00	C
ATOM	367	C	PHE	B	591	113.619	96.406	90.724	1.00	0.00	C
ATOM	368	O	PHE	B	591	112.911	96.345	89.719	1.00	0.00	O
ATOM	369	CB	PHE	B	591	112.586	97.811	92.590	1.00	20.00	C
ATOM	370	CG	PHE	B	591	111.429	97.870	93.576	1.00	20.00	C
ATOM	371	CD1	PHE	B	591	110.108	98.006	93.100	1.00	20.00	C
ATOM	372	CD2	PHE	B	591	111.631	97.657	94.956	1.00	20.00	C
ATOM	373	CE1	PHE	B	591	109.049	98.044	93.996	1.00	20.00	C
ATOM	374	CE2	PHE	B	591	110.559	97.689	95.835	1.00	20.00	C
ATOM	375	CZ	PHE	B	591	109.275	97.908	95.359	1.00	20.00	C
ATOM	376	H	PHE	B	591	114.226	96.272	93.801	1.00	0.00	H
ATOM	377	HA	PHE	B	591	112.014	95.848	91.983	1.00	0.00	H
ATOM	378	HB2	PHE	B	591	113.457	98.296	93.032	1.00	0.00	H
ATOM	379	HB3	PHE	B	591	112.312	98.431	91.735	1.00	0.00	H
ATOM	380	HD1	PHE	B	591	109.918	98.125	92.043	1.00	0.00	H
ATOM	381	HD2	PHE	B	591	112.614	97.478	95.351	1.00	0.00	H
ATOM	382	HE1	PHE	B	591	108.045	98.185	93.626	1.00	0.00	H
ATOM	383	HE2	PHE	B	591	110.727	97.539	96.891	1.00	0.00	H
ATOM	384	HZ	PHE	B	591	108.451	97.950	96.055	1.00	0.00	H
ATOM	385	N	SER	B	592	114.960	96.431	90.677	1.00	0.00	N
ATOM	386	CA	SER	B	592	115.734	96.388	89.428	1.00	0.00	C
ATOM	387	C	SER	B	592	115.557	95.062	88.682	1.00	0.00	C
ATOM	388	O	SER	B	592	115.362	95.092	87.470	1.00	0.00	O
ATOM	389	CB	SER	B	592	117.224	96.709	89.671	1.00	20.00	C
ATOM	390	OG	SER	B	592	117.962	96.695	88.466	1.00	20.00	O
ATOM	391	H	SER	B	592	115.497	96.416	91.534	1.00	0.00	H
ATOM	392	HA	SER	B	592	115.355	97.170	88.773	1.00	0.00	H
ATOM	393	HB2	SER	B	592	117.337	97.703	90.101	1.00	0.00	H
ATOM	394	HB3	SER	B	592	117.669	95.995	90.363	1.00	0.00	H
ATOM	395	HG	SER	B	592	117.992	95.796	88.130	1.00	0.00	H
ATOM	396	N	THR	B	593	115.587	93.933	89.407	1.00	30.00	N
ATOM	397	CA	THR	B	593	115.307	92.621	88.828	1.00	30.00	C
ATOM	398	C	THR	B	593	113.830	92.493	88.398	1.00	30.00	C
ATOM	399	O	THR	B	593	113.569	91.947	87.330	1.00	30.00	O
ATOM	400	CB	THR	B	593	115.643	91.461	89.798	1.00	20.00	C
ATOM	401	CG2	THR	B	593	115.477	90.058	89.189	1.00	20.00	C
ATOM	402	OG1	THR	B	593	116.989	91.562	90.220	1.00	20.00	O
ATOM	403	H	THR	B	593	115.755	93.968	90.404	1.00	0.00	H
ATOM	404	HA	THR	B	593	115.928	92.507	87.936	1.00	0.00	H
ATOM	405	HB	THR	B	593	115.022	91.544	90.690	1.00	0.00	H
ATOM	406	HG1	THR	B	593	117.072	92.317	90.811	1.00	0.00	H
ATOM	407	HG21	THR	B	593	115.673	89.294	89.938	1.00	0.00	H
ATOM	408	HG22	THR	B	593	114.476	89.872	88.806	1.00	0.00	H
ATOM	409	HG23	THR	B	593	116.167	89.909	88.358	1.00	0.00	H
ATOM	410	N	ALA	B	594	112.901	93.043	89.201	1.00	30.00	N
ATOM	411	CA	ALA	B	594	111.465	93.033	88.919	1.00	30.00	C
ATOM	412	C	ALA	B	594	111.071	93.805	87.646	1.00	30.00	C
ATOM	413	O	ALA	B	594	110.216	93.319	86.910	1.00	30.00	O
ATOM	414	CB	ALA	B	594	110.700	93.556	90.141	1.00	30.00	C
ATOM	415	H	ALA	B	594	113.183	93.487	90.064	1.00	0.00	H
ATOM	416	HA	ALA	B	594	111.175	91.990	88.774	1.00	0.00	H

ATOM	417	HB1	ALA	B	594	109.627	93.423	90.014	1.00	0.00	H
ATOM	418	HB2	ALA	B	594	110.984	93.018	91.045	1.00	0.00	H
ATOM	419	HB3	ALA	B	594	110.884	94.617	90.309	1.00	0.00	H
ATOM	420	N	VAL	B	595	111.709	94.960	87.378	1.00	30.00	N
ATOM	421	CA	VAL	B	595	111.429	95.763	86.183	1.00	30.00	C
ATOM	422	C	VAL	B	595	112.113	95.219	84.903	1.00	30.00	C
ATOM	423	O	VAL	B	595	111.562	95.430	83.823	1.00	30.00	O
ATOM	424	CB	VAL	B	595	111.753	97.272	86.401	1.00	20.00	C
ATOM	425	CG1	VAL	B	595	113.250	97.615	86.424	1.00	20.00	C
ATOM	426	CG2	VAL	B	595	111.028	98.187	85.396	1.00	20.00	C
ATOM	427	H	VAL	B	595	112.399	95.323	88.022	1.00	0.00	H
ATOM	428	HA	VAL	B	595	110.354	95.704	86.000	1.00	0.00	H
ATOM	429	HB	VAL	B	595	111.361	97.534	87.385	1.00	0.00	H
ATOM	430	HG11	VAL	B	595	113.407	98.668	86.662	1.00	0.00	H
ATOM	431	HG12	VAL	B	595	113.764	97.034	87.182	1.00	0.00	H
ATOM	432	HG13	VAL	B	595	113.731	97.422	85.466	1.00	0.00	H
ATOM	433	HG21	VAL	B	595	111.185	99.239	85.639	1.00	0.00	H
ATOM	434	HG22	VAL	B	595	111.380	98.035	84.376	1.00	0.00	H
ATOM	435	HG23	VAL	B	595	109.952	98.009	85.409	1.00	0.00	H
ATOM	436	N	VAL	B	596	113.234	94.476	85.030	1.00	30.00	N
ATOM	437	CA	VAL	B	596	113.863	93.745	83.916	1.00	30.00	C
ATOM	438	C	VAL	B	596	112.946	92.658	83.337	1.00	30.00	C
ATOM	439	O	VAL	B	596	112.827	92.570	82.118	1.00	30.00	O
ATOM	440	CB	VAL	B	596	115.201	93.045	84.309	1.00	20.00	C
ATOM	441	CG1	VAL	B	596	115.690	91.945	83.340	1.00	20.00	C
ATOM	442	CG2	VAL	B	596	116.335	94.049	84.484	1.00	20.00	C
ATOM	443	H	VAL	B	596	113.646	94.342	85.943	1.00	0.00	H
ATOM	444	HA	VAL	B	596	114.071	94.468	83.123	1.00	0.00	H
ATOM	445	HB	VAL	B	596	115.064	92.573	85.282	1.00	0.00	H
ATOM	446	HG11	VAL	B	596	116.739	91.723	83.503	1.00	0.00	H
ATOM	447	HG12	VAL	B	596	115.143	91.010	83.463	1.00	0.00	H
ATOM	448	HG13	VAL	B	596	115.591	92.261	82.302	1.00	0.00	H
ATOM	449	HG21	VAL	B	596	117.201	93.543	84.898	1.00	0.00	H
ATOM	450	HG22	VAL	B	596	116.620	94.509	83.536	1.00	0.00	H
ATOM	451	HG23	VAL	B	596	116.062	94.836	85.176	1.00	0.00	H
ATOM	452	N	THR	B	597	112.348	91.842	84.220	1.00	30.00	N
ATOM	453	CA	THR	B	597	111.501	90.711	83.838	1.00	30.00	C
ATOM	454	C	THR	B	597	110.136	91.143	83.263	1.00	30.00	C
ATOM	455	O	THR	B	597	109.525	90.346	82.556	1.00	30.00	O
ATOM	456	CB	THR	B	597	111.279	89.742	85.029	1.00	20.00	C
ATOM	457	CG2	THR	B	597	112.595	89.216	85.618	1.00	20.00	C
ATOM	458	OG1	THR	B	597	110.497	90.307	86.063	1.00	20.00	O
ATOM	459	H	THR	B	597	112.516	91.969	85.208	1.00	0.00	H
ATOM	460	HA	THR	B	597	112.020	90.158	83.053	1.00	0.00	H
ATOM	461	HB	THR	B	597	110.721	88.877	84.668	1.00	0.00	H
ATOM	462	HG1	THR	B	597	110.987	91.028	86.467	1.00	0.00	H
ATOM	463	HG21	THR	B	597	112.424	88.571	86.478	1.00	0.00	H
ATOM	464	HG22	THR	B	597	113.136	88.648	84.868	1.00	0.00	H
ATOM	465	HG23	THR	B	597	113.263	90.010	85.934	1.00	0.00	H
ATOM	466	N	LEU	B	598	109.712	92.396	83.507	1.00	30.00	N
ATOM	467	CA	LEU	B	598	108.554	92.999	82.852	1.00	30.00	C
ATOM	468	C	LEU	B	598	108.902	93.548	81.453	1.00	30.00	C
ATOM	469	O	LEU	B	598	108.083	93.410	80.544	1.00	30.00	O
ATOM	470	CB	LEU	B	598	107.970	94.086	83.771	1.00	20.00	C
ATOM	471	CG	LEU	B	598	106.600	94.633	83.321	1.00	20.00	C
ATOM	472	CD1	LEU	B	598	105.481	93.570	83.330	1.00	20.00	C
ATOM	473	CD2	LEU	B	598	106.227	95.831	84.188	1.00	20.00	C
ATOM	474	H	LEU	B	598	110.258	93.000	84.105	1.00	0.00	H
ATOM	475	HA	LEU	B	598	107.799	92.223	82.720	1.00	0.00	H
ATOM	476	HB2	LEU	B	598	107.877	93.697	84.785	1.00	0.00	H
ATOM	477	HB3	LEU	B	598	108.686	94.907	83.843	1.00	0.00	H
ATOM	478	HG	LEU	B	598	106.686	95.013	82.302	1.00	0.00	H
ATOM	479	HD11	LEU	B	598	104.597	93.909	83.869	1.00	0.00	H
ATOM	480	HD12	LEU	B	598	105.166	93.332	82.313	1.00	0.00	H
ATOM	481	HD13	LEU	B	598	105.797	92.638	83.797	1.00	0.00	H
ATOM	482	HD21	LEU	B	598	105.252	96.224	83.905	1.00	0.00	H
ATOM	483	HD22	LEU	B	598	106.198	95.563	85.242	1.00	0.00	H
ATOM	484	HD23	LEU	B	598	106.965	96.623	84.072	1.00	0.00	H
ATOM	485	N	ILE	B	599	110.112	94.119	81.296	1.00	30.00	N
ATOM	486	CA	ILE	B	599	110.682	94.528	80.008	1.00	30.00	C
ATOM	487	C	ILE	B	599	110.956	93.293	79.125	1.00	30.00	C

```
ATOM    488  O   ILE B 599     111.536  92.317  79.600  1.00 30.00           O
ATOM    489  CB  ILE B 599     111.976  95.384  80.191  1.00 20.00           C
ATOM    490  CG1 ILE B 599     111.628  96.769  80.789  1.00 20.00           C
ATOM    491  CG2 ILE B 599     112.821  95.565  78.910  1.00 20.00           C
ATOM    492  CD1 ILE B 599     112.823  97.491  81.432  1.00 20.00           C
ATOM    493  H   ILE B 599     110.729  94.202  82.093  1.00  0.00           H
ATOM    494  HA  ILE B 599     109.944  95.146  79.499  1.00  0.00           H
ATOM    495  HB  ILE B 599     112.608  94.871  80.916  1.00  0.00           H
ATOM    496 HG12 ILE B 599     111.196  97.403  80.013  1.00  0.00           H
ATOM    497 HG13 ILE B 599     110.850  96.672  81.546  1.00  0.00           H
ATOM    498 HG21 ILE B 599     113.658  96.244  79.070  1.00  0.00           H
ATOM    499 HG22 ILE B 599     113.248  94.628  78.558  1.00  0.00           H
ATOM    500 HG23 ILE B 599     112.216  95.969  78.101  1.00  0.00           H
ATOM    501 HD11 ILE B 599     112.485  98.243  82.144  1.00  0.00           H
ATOM    502 HD12 ILE B 599     113.474  96.800  81.969  1.00  0.00           H
ATOM    503 HD13 ILE B 599     113.423  98.000  80.679  1.00  0.00           H
ATOM    504  N   GLU B 600     110.487  93.345  77.872  1.00 30.00           N
ATOM    505  CA  GLU B 600     110.508  92.203  76.958  1.00 30.00           C
ATOM    506  C   GLU B 600     111.806  92.117  76.142  1.00 30.00           C
ATOM    507  O   GLU B 600     112.338  91.019  75.981  1.00 30.00           O
ATOM    508  CB  GLU B 600     109.256  92.261  76.063  1.00 20.00           C
ATOM    509  CG  GLU B 600     107.966  92.038  76.880  1.00 20.00           C
ATOM    510  CD  GLU B 600     106.695  92.046  76.034  1.00 20.00           C
ATOM    511  OE1 GLU B 600     106.585  92.935  75.163  1.00 20.00           O
ATOM    512  OE2 GLU B 600     105.849  91.160  76.292  1.00 20.00           O1-
ATOM    513  H   GLU B 600     110.016  94.177  77.542  1.00  0.00           H
ATOM    514  HA  GLU B 600     110.446  91.278  77.535  1.00  0.00           H
ATOM    515  HB2 GLU B 600     109.214  93.228  75.558  1.00  0.00           H
ATOM    516  HB3 GLU B 600     109.323  91.513  75.272  1.00  0.00           H
ATOM    517  HG2 GLU B 600     108.030  91.081  77.396  1.00  0.00           H
ATOM    518  HG3 GLU B 600     107.858  92.799  77.652  1.00  0.00           H
ATOM    519  N   ASP B 601     112.299  93.270  75.668  1.00 30.00           N
ATOM    520  CA  ASP B 601     113.493  93.399  74.828  1.00 30.00           C
ATOM    521  C   ASP B 601     113.926  94.877  74.771  1.00 30.00           C
ATOM    522  O   ASP B 601     113.244  95.734  75.331  1.00 30.00           O
ATOM    523  CB  ASP B 601     113.362  92.751  73.414  1.00 20.00           C
ATOM    524  CG  ASP B 601     112.035  92.967  72.671  1.00  0.00           C
ATOM    525  OD1 ASP B 601     111.376  94.002  72.916  1.00  0.00           O
ATOM    526  OD2 ASP B 601     111.743  92.119  71.801  1.00  0.00           O1-
ATOM    527  H   ASP B 601     111.805  94.133  75.845  1.00  0.00           H
ATOM    528  HA  ASP B 601     114.298  92.879  75.348  1.00  0.00           H
ATOM    529  HB2 ASP B 601     114.170  93.070  72.756  1.00  0.00           H
ATOM    530  HB3 ASP B 601     113.484  91.675  73.544  1.00  0.00           H
ATOM    531  N   GLY B 602     115.059  95.147  74.105  1.00 30.00           N
ATOM    532  CA  GLY B 602     115.595  96.495  73.916  1.00 30.00           C
ATOM    533  C   GLY B 602     116.732  96.781  74.907  1.00 30.00           C
ATOM    534  O   GLY B 602     117.286  95.871  75.526  1.00 30.00           O
ATOM    535  H   GLY B 602     115.578  94.388  73.690  1.00  0.00           H
ATOM    536  HA2 GLY B 602     115.988  96.568  72.902  1.00  0.00           H
ATOM    537  HA3 GLY B 602     114.817  97.256  74.007  1.00  0.00           H
ATOM    538  N   LYS B 603     117.097  98.071  75.003  1.00 30.00           N
ATOM    539  CA  LYS B 603     118.228  98.621  75.762  1.00 30.00           C
ATOM    540  C   LYS B 603     118.246  98.254  77.258  1.00 30.00           C
ATOM    541  O   LYS B 603     119.293  97.859  77.773  1.00 30.00           O
ATOM    542  CB  LYS B 603     118.240 100.154  75.541  1.00 20.00           C
ATOM    543  CG  LYS B 603     119.310 100.944  76.321  1.00 20.00           C
ATOM    544  CD  LYS B 603     119.243 102.450  76.035  1.00 20.00           C
ATOM    545  CE  LYS B 603     120.261 103.252  76.857  1.00 20.00           C
ATOM    546  NZ  LYS B 603     120.083 104.700  76.660  1.00 20.00           N1+
ATOM    547  H   LYS B 603     116.585  98.749  74.457  1.00  0.00           H
ATOM    548  HA  LYS B 603     119.135  98.209  75.316  1.00  0.00           H
ATOM    549  HB2 LYS B 603     118.364 100.354  74.476  1.00  0.00           H
ATOM    550  HB3 LYS B 603     117.261 100.557  75.808  1.00  0.00           H
ATOM    551  HG2 LYS B 603     119.183 100.797  77.394  1.00  0.00           H
ATOM    552  HG3 LYS B 603     120.301 100.562  76.072  1.00  0.00           H
ATOM    553  HD2 LYS B 603     119.416 102.624  74.972  1.00  0.00           H
ATOM    554  HD3 LYS B 603     118.236 102.813  76.243  1.00  0.00           H
ATOM    555  HE2 LYS B 603     120.146 103.034  77.919  1.00  0.00           H
ATOM    556  HE3 LYS B 603     121.278 102.973  76.578  1.00  0.00           H
ATOM    557  HZ1 LYS B 603     119.158 104.966  76.972  1.00  0.00           H
ATOM    558  HZ2 LYS B 603     120.773 105.202  77.201  1.00  0.00           H
```

ATOM	559	HZ3	LYS	B	603	120.189	104.928	75.682	1.00	0.00	H
ATOM	560	N	TYR	B	627	117.091	98.413	77.921	1.00	30.00	N
ATOM	561	CA	TYR	B	627	116.931	98.285	79.372	1.00	30.00	C
ATOM	562	C	TYR	B	627	116.590	96.858	79.845	1.00	30.00	C
ATOM	563	O	TYR	B	627	116.304	96.679	81.029	1.00	30.00	O
ATOM	564	CB	TYR	B	627	115.902	99.337	79.832	1.00	20.00	C
ATOM	565	CG	TYR	B	627	116.376	100.765	79.619	1.00	20.00	C
ATOM	566	CD1	TYR	B	627	117.422	101.273	80.415	1.00	20.00	C
ATOM	567	CD2	TYR	B	627	115.805	101.577	78.617	1.00	20.00	C
ATOM	568	CE1	TYR	B	627	117.895	102.581	80.213	1.00	20.00	C
ATOM	569	CE2	TYR	B	627	116.264	102.897	78.429	1.00	20.00	C
ATOM	570	CZ	TYR	B	627	117.305	103.402	79.234	1.00	20.00	C
ATOM	571	OH	TYR	B	627	117.741	104.686	79.079	1.00	20.00	O
ATOM	572	H	TYR	B	627	116.276	98.726	77.415	1.00	0.00	H
ATOM	573	HA	TYR	B	627	117.881	98.526	79.849	1.00	0.00	H
ATOM	574	HB2	TYR	B	627	114.952	99.187	79.316	1.00	0.00	H
ATOM	575	HB3	TYR	B	627	115.687	99.225	80.895	1.00	0.00	H
ATOM	576	HD1	TYR	B	627	117.865	100.657	81.183	1.00	0.00	H
ATOM	577	HD2	TYR	B	627	115.011	101.195	77.993	1.00	0.00	H
ATOM	578	HE1	TYR	B	627	118.701	102.955	80.827	1.00	0.00	H
ATOM	579	HE2	TYR	B	627	115.810	103.522	77.675	1.00	0.00	H
ATOM	580	HH	TYR	B	627	118.210	105.002	79.860	1.00	0.00	H
ATOM	581	N	ASN	B	628	116.658	95.870	78.936	1.00	30.00	N
ATOM	582	CA	ASN	B	628	116.437	94.449	79.218	1.00	30.00	C
ATOM	583	C	ASN	B	628	117.611	93.780	79.973	1.00	30.00	C
ATOM	584	O	ASN	B	628	117.397	92.725	80.568	1.00	30.00	O
ATOM	585	CB	ASN	B	628	116.121	93.735	77.880	1.00	20.00	C
ATOM	586	CG	ASN	B	628	115.512	92.331	78.002	1.00	20.00	C
ATOM	587	ND2	ASN	B	628	114.502	92.148	78.854	1.00	20.00	N
ATOM	588	OD1	ASN	B	628	115.945	91.415	77.308	1.00	20.00	O
ATOM	589	H	ASN	B	628	116.905	96.096	77.982	1.00	0.00	H
ATOM	590	HA	ASN	B	628	115.577	94.357	79.884	1.00	0.00	H
ATOM	591	HB2	ASN	B	628	115.396	94.329	77.322	1.00	0.00	H
ATOM	592	HB3	ASN	B	628	117.014	93.690	77.256	1.00	0.00	H
ATOM	593	HD21	ASN	B	628	114.071	91.238	78.934	1.00	0.00	H
ATOM	594	HD22	ASN	B	628	114.143	92.906	79.416	1.00	0.00	H
ATOM	595	N	SER	B	629	118.807	94.397	79.978	1.00	30.00	N
ATOM	596	CA	SER	B	629	119.927	93.977	80.827	1.00	30.00	C
ATOM	597	C	SER	B	629	119.764	94.527	82.258	1.00	30.00	C
ATOM	598	O	SER	B	629	119.200	95.610	82.437	1.00	30.00	O
ATOM	599	CB	SER	B	629	121.269	94.377	80.170	1.00	20.00	C
ATOM	600	OG	SER	B	629	121.638	95.725	80.385	1.00	20.00	O
ATOM	601	H	SER	B	629	118.922	95.266	79.477	1.00	0.00	H
ATOM	602	HA	SER	B	629	119.917	92.886	80.880	1.00	0.00	H
ATOM	603	HB2	SER	B	629	121.249	94.176	79.098	1.00	0.00	H
ATOM	604	HB3	SER	B	629	122.067	93.758	80.580	1.00	0.00	H
ATOM	605	HG	SER	B	629	121.140	96.288	79.786	1.00	0.00	H
ATOM	606	N	LEU	B	630	120.282	93.772	83.243	1.00	30.00	N
ATOM	607	CA	LEU	B	630	120.223	94.102	84.671	1.00	30.00	C
ATOM	608	C	LEU	B	630	120.923	95.423	85.031	1.00	30.00	C
ATOM	609	O	LEU	B	630	120.346	96.214	85.774	1.00	30.00	O
ATOM	610	CB	LEU	B	630	120.754	92.905	85.495	1.00	20.00	C
ATOM	611	CG	LEU	B	630	120.620	93.023	87.033	1.00	20.00	C
ATOM	612	CD1	LEU	B	630	119.153	93.193	87.482	1.00	20.00	C
ATOM	613	CD2	LEU	B	630	121.296	91.819	87.724	1.00	20.00	C
ATOM	614	H	LEU	B	630	120.723	92.893	83.012	1.00	0.00	H
ATOM	615	HA	LEU	B	630	119.176	94.248	84.915	1.00	0.00	H
ATOM	616	HB2	LEU	B	630	120.240	91.998	85.181	1.00	0.00	H
ATOM	617	HB3	LEU	B	630	121.804	92.749	85.243	1.00	0.00	H
ATOM	618	HG	LEU	B	630	121.164	93.909	87.363	1.00	0.00	H
ATOM	619	HD11	LEU	B	630	118.955	92.738	88.453	1.00	0.00	H
ATOM	620	HD12	LEU	B	630	118.901	94.249	87.572	1.00	0.00	H
ATOM	621	HD13	LEU	B	630	118.457	92.750	86.770	1.00	0.00	H
ATOM	622	HD21	LEU	B	630	120.617	91.271	88.378	1.00	0.00	H
ATOM	623	HD22	LEU	B	630	121.685	91.099	87.004	1.00	0.00	H
ATOM	624	HD23	LEU	B	630	122.139	92.148	88.329	1.00	0.00	H
ATOM	625	N	TYR	B	631	122.126	95.640	84.476	1.00	30.00	N
ATOM	626	CA	TYR	B	631	122.945	96.833	84.696	1.00	30.00	C
ATOM	627	C	TYR	B	631	122.297	98.136	84.193	1.00	30.00	C
ATOM	628	O	TYR	B	631	122.347	99.134	84.912	1.00	30.00	O
ATOM	629	CB	TYR	B	631	124.347	96.598	84.088	1.00	20.00	C

ATOM	630	CG	TYR	B	631	125.269	97.807	84.067	1.00	20.00	C
ATOM	631	CD1	TYR	B	631	125.781	98.327	85.274	1.00	20.00	C
ATOM	632	CD2	TYR	B	631	125.595	98.430	82.843	1.00	20.00	C
ATOM	633	CE1	TYR	B	631	126.609	99.467	85.259	1.00	20.00	C
ATOM	634	CE2	TYR	B	631	126.427	99.566	82.828	1.00	20.00	C
ATOM	635	CZ	TYR	B	631	126.932	100.087	84.035	1.00	20.00	C
ATOM	636	OH	TYR	B	631	127.726	101.195	84.018	1.00	20.00	O
ATOM	637	H	TYR	B	631	122.515	94.943	83.859	1.00	0.00	H
ATOM	638	HA	TYR	B	631	123.070	96.933	85.776	1.00	0.00	H
ATOM	639	HB2	TYR	B	631	124.849	95.793	84.627	1.00	0.00	H
ATOM	640	HB3	TYR	B	631	124.236	96.241	83.062	1.00	0.00	H
ATOM	641	HD1	TYR	B	631	125.531	97.861	86.215	1.00	0.00	H
ATOM	642	HD2	TYR	B	631	125.202	98.046	81.913	1.00	0.00	H
ATOM	643	HE1	TYR	B	631	126.990	99.863	86.188	1.00	0.00	H
ATOM	644	HE2	TYR	B	631	126.670	100.042	81.889	1.00	0.00	H
ATOM	645	HH	TYR	B	631	128.022	101.463	84.891	1.00	0.00	H
ATOM	646	N	SER	B	632	121.706	98.103	82.984	1.00	30.00	N
ATOM	647	CA	SER	B	632	121.145	99.276	82.310	1.00	30.00	C
ATOM	648	C	SER	B	632	119.989	99.949	83.067	1.00	30.00	C
ATOM	649	O	SER	B	632	119.954	101.177	83.105	1.00	30.00	O
ATOM	650	CB	SER	B	632	120.711	98.909	80.878	1.00	20.00	C
ATOM	651	OG	SER	B	632	121.837	98.816	80.030	1.00	20.00	O
ATOM	652	H	SER	B	632	121.699	97.242	82.454	1.00	0.00	H
ATOM	653	HA	SER	B	632	121.940	100.022	82.244	1.00	0.00	H
ATOM	654	HB2	SER	B	632	120.144	97.978	80.858	1.00	0.00	H
ATOM	655	HB3	SER	B	632	120.062	99.679	80.458	1.00	0.00	H
ATOM	656	HG	SER	B	632	122.193	99.697	79.893	1.00	0.00	H
ATOM	657	N	THR	B	633	119.088	99.147	83.662	1.00	30.00	N
ATOM	658	CA	THR	B	633	117.945	99.648	84.430	1.00	30.00	C
ATOM	659	C	THR	B	633	118.217	99.726	85.953	1.00	30.00	C
ATOM	660	O	THR	B	633	117.457	100.394	86.653	1.00	30.00	O
ATOM	661	CB	THR	B	633	116.667	98.822	84.138	1.00	20.00	C
ATOM	662	CG2	THR	B	633	116.710	97.372	84.633	1.00	20.00	C
ATOM	663	OG1	THR	B	633	115.527	99.421	84.724	1.00	20.00	O
ATOM	664	H	THR	B	633	119.181	98.143	83.591	1.00	0.00	H
ATOM	665	HA	THR	B	633	117.729	100.667	84.105	1.00	0.00	H
ATOM	666	HB	THR	B	633	116.504	98.811	83.061	1.00	0.00	H
ATOM	667	HG1	THR	B	633	115.655	99.451	85.676	1.00	0.00	H
ATOM	668	HG21	THR	B	633	115.814	96.852	84.296	1.00	0.00	H
ATOM	669	HG22	THR	B	633	117.571	96.839	84.231	1.00	0.00	H
ATOM	670	HG23	THR	B	633	116.748	97.301	85.720	1.00	0.00	H
ATOM	671	N	CYS	B	634	119.321	99.120	86.436	1.00	30.00	N
ATOM	672	CA	CYS	B	634	119.848	99.343	87.790	1.00	30.00	C
ATOM	673	C	CYS	B	634	120.369	100.785	87.948	1.00	30.00	C
ATOM	674	O	CYS	B	634	120.166	101.383	89.004	1.00	30.00	O
ATOM	675	CB	CYS	B	634	120.919	98.299	88.182	1.00	20.00	C
ATOM	676	SG	CYS	B	634	121.434	98.445	89.921	1.00	20.00	S
ATOM	677	H	CYS	B	634	119.895	98.559	85.822	1.00	0.00	H
ATOM	678	HA	CYS	B	634	119.013	99.228	88.484	1.00	0.00	H
ATOM	679	HB2	CYS	B	634	120.529	97.292	88.055	1.00	0.00	H
ATOM	680	HB3	CYS	B	634	121.797	98.382	87.541	1.00	0.00	H
ATOM	681	HG	CYS	B	634	122.014	99.642	89.797	1.00	0.00	H
ATOM	682	N	LEU	B	635	120.966	101.330	86.872	1.00	30.00	N
ATOM	683	CA	LEU	B	635	121.337	102.739	86.746	1.00	30.00	C
ATOM	684	C	LEU	B	635	120.112	103.667	86.748	1.00	30.00	C
ATOM	685	O	LEU	B	635	120.160	104.678	87.440	1.00	30.00	O
ATOM	686	CB	LEU	B	635	122.168	102.959	85.464	1.00	20.00	C
ATOM	687	CG	LEU	B	635	123.586	102.352	85.502	1.00	20.00	C
ATOM	688	CD1	LEU	B	635	124.197	102.316	84.086	1.00	20.00	C
ATOM	689	CD2	LEU	B	635	124.496	103.064	86.528	1.00	20.00	C
ATOM	690	H	LEU	B	635	121.110	100.761	86.048	1.00	0.00	H
ATOM	691	HA	LEU	B	635	121.945	102.999	87.613	1.00	0.00	H
ATOM	692	HB2	LEU	B	635	121.618	102.540	84.621	1.00	0.00	H
ATOM	693	HB3	LEU	B	635	122.256	104.027	85.253	1.00	0.00	H
ATOM	694	HG	LEU	B	635	123.499	101.315	85.826	1.00	0.00	H
ATOM	695	HD11	LEU	B	635	125.209	102.717	84.044	1.00	0.00	H
ATOM	696	HD12	LEU	B	635	124.243	101.289	83.722	1.00	0.00	H
ATOM	697	HD13	LEU	B	635	123.601	102.879	83.367	1.00	0.00	H
ATOM	698	HD21	LEU	B	635	124.741	102.393	87.352	1.00	0.00	H
ATOM	699	HD22	LEU	B	635	125.441	103.398	86.101	1.00	0.00	H
ATOM	700	HD23	LEU	B	635	124.018	103.944	86.960	1.00	0.00	H

```
ATOM    701  N   GLU B 636     119.041 103.305  86.016  1.00 30.00           N
ATOM    702  CA  GLU B 636     117.795 104.079  85.944  1.00 30.00           C
ATOM    703  C   GLU B 636     117.027 104.148  87.274  1.00 30.00           C
ATOM    704  O   GLU B 636     116.416 105.181  87.543  1.00 30.00           O
ATOM    705  CB  GLU B 636     116.884 103.544  84.820  1.00 20.00           C
ATOM    706  CG  GLU B 636     117.468 103.683  83.399  1.00 20.00           C
ATOM    707  CD  GLU B 636     117.632 105.122  82.897  1.00 20.00           C
ATOM    708  OE1 GLU B 636     116.876 106.000  83.366  1.00 20.00           O
ATOM    709  OE2 GLU B 636     118.514 105.320  82.030  1.00 20.00           O1-
ATOM    710  H   GLU B 636     119.071 102.457  85.470  1.00  0.00           H
ATOM    711  HA  GLU B 636     118.070 105.106  85.702  1.00  0.00           H
ATOM    712  HB2 GLU B 636     116.660 102.494  85.007  1.00  0.00           H
ATOM    713  HB3 GLU B 636     115.914 104.044  84.856  1.00  0.00           H
ATOM    714  HG2 GLU B 636     118.435 103.187  83.345  1.00  0.00           H
ATOM    715  HG3 GLU B 636     116.819 103.159  82.701  1.00  0.00           H
ATOM    716  N   LEU B 637     117.102 103.086  88.097  1.00 30.00           N
ATOM    717  CA  LEU B 637     116.566 103.096  89.458  1.00 30.00           C
ATOM    718  C   LEU B 637     117.478 103.838  90.451  1.00 30.00           C
ATOM    719  O   LEU B 637     116.960 104.465  91.374  1.00 30.00           O
ATOM    720  CB  LEU B 637     116.207 101.674  89.930  1.00 20.00           C
ATOM    721  CG  LEU B 637     114.954 101.076  89.248  1.00 20.00           C
ATOM    722  CD1 LEU B 637     114.629  99.704  89.847  1.00 20.00           C
ATOM    723  CD2 LEU B 637     113.704 101.981  89.293  1.00 20.00           C
ATOM    724  H   LEU B 637     117.592 102.251  87.803  1.00  0.00           H
ATOM    725  HA  LEU B 637     115.643 103.660  89.435  1.00  0.00           H
ATOM    726  HB2 LEU B 637     117.064 101.013  89.784  1.00  0.00           H
ATOM    727  HB3 LEU B 637     116.025 101.690  91.005  1.00  0.00           H
ATOM    728  HG  LEU B 637     115.202 100.916  88.197  1.00  0.00           H
ATOM    729 HD11 LEU B 637     114.207  99.049  89.087  1.00  0.00           H
ATOM    730 HD12 LEU B 637     115.514  99.223  90.262  1.00  0.00           H
ATOM    731 HD13 LEU B 637     113.901  99.778  90.656  1.00  0.00           H
ATOM    732 HD21 LEU B 637     112.789 101.410  89.454  1.00  0.00           H
ATOM    733 HD22 LEU B 637     113.756 102.721  90.090  1.00  0.00           H
ATOM    734 HD23 LEU B 637     113.584 102.517  88.351  1.00  0.00           H
ATOM    735  N   PHE B 638     118.803 103.819  90.227  1.00 30.00           N
ATOM    736  CA  PHE B 638     119.769 104.600  91.004  1.00 30.00           C
ATOM    737  C   PHE B 638     119.672 106.122  90.746  1.00 30.00           C
ATOM    738  O   PHE B 638     120.019 106.897  91.637  1.00 30.00           O
ATOM    739  CB  PHE B 638     121.186 104.025  90.785  1.00 20.00           C
ATOM    740  CG  PHE B 638     122.290 104.673  91.604  1.00 20.00           C
ATOM    741  CD1 PHE B 638     122.445 104.334  92.964  1.00 20.00           C
ATOM    742  CD2 PHE B 638     123.052 105.736  91.073  1.00 20.00           C
ATOM    743  CE1 PHE B 638     123.369 105.008  93.751  1.00 20.00           C
ATOM    744  CE2 PHE B 638     123.972 106.398  91.876  1.00 20.00           C
ATOM    745  CZ  PHE B 638     124.131 106.033  93.207  1.00 20.00           C
ATOM    746  H   PHE B 638     119.176 103.277  89.459  1.00  0.00           H
ATOM    747  HA  PHE B 638     119.529 104.451  92.059  1.00  0.00           H
ATOM    748  HB2 PHE B 638     121.178 102.966  91.046  1.00  0.00           H
ATOM    749  HB3 PHE B 638     121.454 104.069  89.729  1.00  0.00           H
ATOM    750  HD1 PHE B 638     121.842 103.552  93.400  1.00  0.00           H
ATOM    751  HD2 PHE B 638     122.915 106.043  90.047  1.00  0.00           H
ATOM    752  HE1 PHE B 638     123.487 104.739  94.790  1.00  0.00           H
ATOM    753  HE2 PHE B 638     124.558 107.208  91.466  1.00  0.00           H
ATOM    754  HZ  PHE B 638     124.845 106.557  93.825  1.00  0.00           H
ATOM    755  N   LYS B 639     119.159 106.531  89.569  1.00 30.00           N
ATOM    756  CA  LYS B 639     118.895 107.929  89.219  1.00 30.00           C
ATOM    757  C   LYS B 639     117.814 108.598  90.086  1.00 30.00           C
ATOM    758  O   LYS B 639     117.934 109.797  90.315  1.00 30.00           O
ATOM    759  CB  LYS B 639     118.531 108.065  87.727  1.00 20.00           C
ATOM    760  CG  LYS B 639     119.712 107.953  86.754  1.00 20.00           C
ATOM    761  CD  LYS B 639     119.238 107.978  85.290  1.00 20.00           C
ATOM    762  CE  LYS B 639     120.300 107.522  84.280  1.00 20.00           C
ATOM    763  NZ  LYS B 639     121.466 108.422  84.267  1.00 20.00           N1+
ATOM    764  H   LYS B 639     118.913 105.841  88.872  1.00  0.00           H
ATOM    765  HA  LYS B 639     119.822 108.480  89.388  1.00  0.00           H
ATOM    766  HB2 LYS B 639     117.754 107.346  87.472  1.00  0.00           H
ATOM    767  HB3 LYS B 639     118.095 109.047  87.551  1.00  0.00           H
ATOM    768  HG2 LYS B 639     120.401 108.779  86.930  1.00  0.00           H
ATOM    769  HG3 LYS B 639     120.281 107.049  86.949  1.00  0.00           H
ATOM    770  HD2 LYS B 639     118.354 107.351  85.178  1.00  0.00           H
ATOM    771  HD3 LYS B 639     118.909 108.987  85.037  1.00  0.00           H
```

```
ATOM    772  HE2 LYS B 639     120.628 106.507  84.507  1.00  0.00           H
ATOM    773  HE3 LYS B 639     119.870 107.500  83.278  1.00  0.00           H
ATOM    774  HZ1 LYS B 639     121.166 109.355  84.023  1.00  0.00           H
ATOM    775  HZ2 LYS B 639     121.892 108.433  85.183  1.00  0.00           H
ATOM    776  HZ3 LYS B 639     122.136 108.095  83.586  1.00  0.00           H
ATOM    777  N   PHE B 640     116.801 107.850  90.569  1.00 30.00           N
ATOM    778  CA  PHE B 640     115.768 108.385  91.473  1.00 30.00           C
ATOM    779  C   PHE B 640     116.324 108.877  92.817  1.00 30.00           C
ATOM    780  O   PHE B 640     115.839 109.890  93.316  1.00 30.00           O
ATOM    781  CB  PHE B 640     114.640 107.366  91.723  1.00 20.00           C
ATOM    782  CG  PHE B 640     113.708 107.120  90.553  1.00 20.00           C
ATOM    783  CD1 PHE B 640     112.617 107.979  90.304  1.00 20.00           C
ATOM    784  CD2 PHE B 640     114.009 106.116  89.616  1.00 20.00           C
ATOM    785  CE1 PHE B 640     111.799 107.755  89.205  1.00 20.00           C
ATOM    786  CE2 PHE B 640     113.181 105.901  88.524  1.00 20.00           C
ATOM    787  CZ  PHE B 640     112.068 106.707  88.332  1.00 20.00           C
ATOM    788  H   PHE B 640     116.750 106.865  90.350  1.00  0.00           H
ATOM    789  HA  PHE B 640     115.324 109.256  90.985  1.00  0.00           H
ATOM    790  HB2 PHE B 640     115.073 106.414  92.032  1.00  0.00           H
ATOM    791  HB3 PHE B 640     114.023 107.693  92.561  1.00  0.00           H
ATOM    792  HD1 PHE B 640     112.403 108.798  90.975  1.00  0.00           H
ATOM    793  HD2 PHE B 640     114.889 105.520  89.764  1.00  0.00           H
ATOM    794  HE1 PHE B 640     110.957 108.405  89.023  1.00  0.00           H
ATOM    795  HE2 PHE B 640     113.407 105.113  87.821  1.00  0.00           H
ATOM    796  HZ  PHE B 640     111.421 106.532  87.487  1.00  0.00           H
ATOM    797  N   THR B 641     117.348 108.180  93.342  1.00 30.00           N
ATOM    798  CA  THR B 641     118.090 108.540  94.555  1.00 30.00           C
ATOM    799  C   THR B 641     118.886 109.856  94.407  1.00 30.00           C
ATOM    800  O   THR B 641     119.002 110.596  95.384  1.00 30.00           O
ATOM    801  CB  THR B 641     119.062 107.401  94.963  1.00 20.00           C
ATOM    802  CG2 THR B 641     119.930 107.640  96.212  1.00 20.00           C
ATOM    803  OG1 THR B 641     118.309 106.226  95.186  1.00 20.00           O
ATOM    804  H   THR B 641     117.679 107.355  92.863  1.00  0.00           H
ATOM    805  HA  THR B 641     117.368 108.672  95.359  1.00  0.00           H
ATOM    806  HB  THR B 641     119.736 107.190  94.134  1.00  0.00           H
ATOM    807  HG1 THR B 641     118.894 105.568  95.570  1.00  0.00           H
ATOM    808 HG21 THR B 641     120.523 106.755  96.449  1.00  0.00           H
ATOM    809 HG22 THR B 641     120.634 108.461  96.073  1.00  0.00           H
ATOM    810 HG23 THR B 641     119.318 107.866  97.084  1.00  0.00           H
ATOM    811  N   ILE B 642     119.371 110.138  93.185  1.00 30.00           N
ATOM    812  CA  ILE B 642     120.036 111.390  92.805  1.00 30.00           C
ATOM    813  C   ILE B 642     119.019 112.540  92.596  1.00 30.00           C
ATOM    814  O   ILE B 642     119.372 113.699  92.814  1.00 30.00           O
ATOM    815  CB  ILE B 642     120.867 111.209  91.491  1.00 20.00           C
ATOM    816  CG1 ILE B 642     121.913 110.073  91.623  1.00 20.00           C
ATOM    817  CG2 ILE B 642     121.563 112.497  90.991  1.00 20.00           C
ATOM    818  CD1 ILE B 642     122.507 109.617  90.280  1.00 20.00           C
ATOM    819  H   ILE B 642     119.228 109.475  92.437  1.00  0.00           H
ATOM    820  HA  ILE B 642     120.716 111.681  93.608  1.00  0.00           H
ATOM    821  HB  ILE B 642     120.174 110.910  90.706  1.00  0.00           H
ATOM    822 HG12 ILE B 642     122.716 110.396  92.286  1.00  0.00           H
ATOM    823 HG13 ILE B 642     121.478 109.196  92.100  1.00  0.00           H
ATOM    824 HG21 ILE B 642     122.166 112.309  90.103  1.00  0.00           H
ATOM    825 HG22 ILE B 642     120.853 113.274  90.711  1.00  0.00           H
ATOM    826 HG23 ILE B 642     122.223 112.909  91.755  1.00  0.00           H
ATOM    827 HD11 ILE B 642     122.590 108.531  90.242  1.00  0.00           H
ATOM    828 HD12 ILE B 642     121.893 109.922  89.433  1.00  0.00           H
ATOM    829 HD13 ILE B 642     123.505 110.030  90.134  1.00  0.00           H
ATOM    830  N   GLY B 643     117.782 112.198  92.195  1.00 30.00           N
ATOM    831  CA  GLY B 643     116.723 113.144  91.844  1.00 30.00           C
ATOM    832  C   GLY B 643     116.665 113.383  90.325  1.00 30.00           C
ATOM    833  O   GLY B 643     116.121 114.400  89.901  1.00 30.00           O
ATOM    834  H   GLY B 643     117.565 111.218  92.080  1.00  0.00           H
ATOM    835  HA2 GLY B 643     115.774 112.715  92.163  1.00  0.00           H
ATOM    836  HA3 GLY B 643     116.836 114.093  92.372  1.00  0.00           H
ATOM    837  N   MET B 644     117.228 112.463  89.521  1.00 30.00           N
ATOM    838  CA  MET B 644     117.288 112.491  88.057  1.00 30.00           C
ATOM    839  C   MET B 644     116.469 111.362  87.395  1.00 30.00           C
ATOM    840  O   MET B 644     116.533 111.225  86.173  1.00 30.00           O
ATOM    841  CB  MET B 644     118.773 112.415  87.624  1.00 20.00           C
ATOM    842  CG  MET B 644     119.541 113.736  87.799  1.00 20.00           C
```

```
ATOM    843  SD  MET B 644     118.916 115.165  86.862  1.00 20.00           S
ATOM    844  CE  MET B 644     118.915 114.513  85.169  1.00 20.00           C
ATOM    845  H   MET B 644     117.652 111.650  89.949  1.00  0.00           H
ATOM    846  HA  MET B 644     116.860 113.419  87.688  1.00  0.00           H
ATOM    847  HB2 MET B 644     119.278 111.630  88.189  1.00  0.00           H
ATOM    848  HB3 MET B 644     118.865 112.110  86.583  1.00  0.00           H
ATOM    849  HG2 MET B 644     119.551 114.014  88.852  1.00  0.00           H
ATOM    850  HG3 MET B 644     120.583 113.583  87.517  1.00  0.00           H
ATOM    851  HE1 MET B 644     118.694 115.313  84.463  1.00  0.00           H
ATOM    852  HE2 MET B 644     119.888 114.090  84.921  1.00  0.00           H
ATOM    853  HE3 MET B 644     118.154 113.741  85.054  1.00  0.00           H
ATOM    854  N   GLY B 645     115.736 110.560  88.188  1.00 30.00           N
ATOM    855  CA  GLY B 645     115.050 109.359  87.715  1.00 30.00           C
ATOM    856  C   GLY B 645     113.774 109.724  86.953  1.00 30.00           C
ATOM    857  O   GLY B 645     112.900 110.406  87.490  1.00 30.00           O
ATOM    858  H   GLY B 645     115.709 110.743  89.180  1.00  0.00           H
ATOM    859  HA2 GLY B 645     115.714 108.755  87.095  1.00  0.00           H
ATOM    860  HA3 GLY B 645     114.785 108.749  88.575  1.00  0.00           H
ATOM    861  N   ASP B 646     113.669 109.215  85.716  1.00 30.00           N
ATOM    862  CA  ASP B 646     112.513 109.375  84.838  1.00 30.00           C
ATOM    863  C   ASP B 646     111.579 108.166  85.022  1.00 30.00           C
ATOM    864  O   ASP B 646     112.031 107.023  84.956  1.00 30.00           O
ATOM    865  CB  ASP B 646     112.967 109.540  83.365  1.00 20.00           C
ATOM    866  CG  ASP B 646     111.850 109.814  82.348  1.00 20.00           C
ATOM    867  OD1 ASP B 646     110.737 110.207  82.768  1.00 20.00           O
ATOM    868  OD2 ASP B 646     112.150 109.669  81.144  1.00 20.00           O1-
ATOM    869  H   ASP B 646     114.418 108.645  85.349  1.00  0.00           H
ATOM    870  HA  ASP B 646     111.977 110.284  85.123  1.00  0.00           H
ATOM    871  HB2 ASP B 646     113.678 110.364  83.305  1.00  0.00           H
ATOM    872  HB3 ASP B 646     113.492 108.635  83.057  1.00  0.00           H
ATOM    873  N   LEU B 647     110.286 108.459  85.227  1.00 30.00           N
ATOM    874  CA  LEU B 647     109.191 107.491  85.331  1.00 30.00           C
ATOM    875  C   LEU B 647     108.862 106.800  83.996  1.00 30.00           C
ATOM    876  O   LEU B 647     108.408 105.659  84.017  1.00 30.00           O
ATOM    877  CB  LEU B 647     107.937 108.229  85.846  1.00 20.00           C
ATOM    878  CG  LEU B 647     108.045 108.725  87.304  1.00 20.00           C
ATOM    879  CD1 LEU B 647     106.949 109.763  87.615  1.00 20.00           C
ATOM    880  CD2 LEU B 647     108.028 107.557  88.310  1.00 20.00           C
ATOM    881  H   LEU B 647     110.016 109.430  85.256  1.00  0.00           H
ATOM    882  HA  LEU B 647     109.475 106.715  86.043  1.00  0.00           H
ATOM    883  HB2 LEU B 647     107.746 109.074  85.182  1.00  0.00           H
ATOM    884  HB3 LEU B 647     107.057 107.588  85.759  1.00  0.00           H
ATOM    885  HG  LEU B 647     108.996 109.246  87.421  1.00  0.00           H
ATOM    886 HD11 LEU B 647     107.368 110.633  88.119  1.00  0.00           H
ATOM    887 HD12 LEU B 647     106.452 110.127  86.715  1.00  0.00           H
ATOM    888 HD13 LEU B 647     106.171 109.348  88.254  1.00  0.00           H
ATOM    889 HD21 LEU B 647     107.577 107.844  89.260  1.00  0.00           H
ATOM    890 HD22 LEU B 647     107.475 106.697  87.928  1.00  0.00           H
ATOM    891 HD23 LEU B 647     109.038 107.215  88.532  1.00  0.00           H
ATOM    892  N   GLU B 648     109.090 107.499  82.873  1.00 30.00           N
ATOM    893  CA  GLU B 648     108.762 107.051  81.520  1.00 30.00           C
ATOM    894  C   GLU B 648     110.057 106.826  80.713  1.00 30.00           C
ATOM    895  O   GLU B 648     110.120 107.203  79.543  1.00 30.00           O
ATOM    896  CB  GLU B 648     107.819 108.096  80.870  1.00 20.00           C
ATOM    897  CG  GLU B 648     106.475 108.339  81.599  1.00  0.00           C
ATOM    898  CD  GLU B 648     105.495 107.167  81.498  1.00  0.00           C
ATOM    899  OE1 GLU B 648     105.662 106.196  82.266  1.00  0.00           O
ATOM    900  OE2 GLU B 648     104.577 107.271  80.654  1.00  0.00           O1-
ATOM    901  H   GLU B 648     109.509 108.418  82.937  1.00  0.00           H
ATOM    902  HA  GLU B 648     108.251 106.087  81.542  1.00  0.00           H
ATOM    903  HB2 GLU B 648     108.344 109.050  80.796  1.00  0.00           H
ATOM    904  HB3 GLU B 648     107.601 107.804  79.841  1.00  0.00           H
ATOM    905  HG2 GLU B 648     106.636 108.578  82.650  1.00  0.00           H
ATOM    906  HG3 GLU B 648     105.995 109.219  81.168  1.00  0.00           H
ATOM    907  N   PHE B 649     111.070 106.209  81.353  1.00 30.00           N
ATOM    908  CA  PHE B 649     112.399 105.953  80.779  1.00 30.00           C
ATOM    909  C   PHE B 649     112.414 104.883  79.666  1.00 30.00           C
ATOM    910  O   PHE B 649     113.427 104.764  78.977  1.00 30.00           O
ATOM    911  CB  PHE B 649     113.393 105.615  81.919  1.00 20.00           C
ATOM    912  CG  PHE B 649     113.326 104.225  82.541  1.00 20.00           C
ATOM    913  CD1 PHE B 649     112.378 103.930  83.545  1.00 20.00           C
```

```
ATOM    914  CD2 PHE B 649     114.135 103.182  82.041  1.00 20.00           C
ATOM    915  CE1 PHE B 649     112.310 102.650  84.081  1.00 20.00           C
ATOM    916  CE2 PHE B 649     114.052 101.909  82.591  1.00 20.00           C
ATOM    917  CZ  PHE B 649     113.147 101.647  83.612  1.00 20.00           C
ATOM    918  H   PHE B 649     110.946 105.933  82.317  1.00  0.00           H
ATOM    919  HA  PHE B 649     112.730 106.890  80.327  1.00  0.00           H
ATOM    920  HB2 PHE B 649     113.297 106.352  82.715  1.00  0.00           H
ATOM    921  HB3 PHE B 649     114.408 105.749  81.540  1.00  0.00           H
ATOM    922  HD1 PHE B 649     111.710 104.695  83.910  1.00  0.00           H
ATOM    923  HD2 PHE B 649     114.836 103.373  81.242  1.00  0.00           H
ATOM    924  HE1 PHE B 649     111.594 102.431  84.860  1.00  0.00           H
ATOM    925  HE2 PHE B 649     114.690 101.120  82.222  1.00  0.00           H
ATOM    926  HZ  PHE B 649     113.084 100.654  84.033  1.00  0.00           H
ATOM    927  N   THR B 650     111.316 104.126  79.512  1.00 30.00           N
ATOM    928  CA  THR B 650     111.171 103.055  78.531  1.00 30.00           C
ATOM    929  C   THR B 650     109.675 102.724  78.324  1.00 30.00           C
ATOM    930  O   THR B 650     108.856 103.059  79.184  1.00 30.00           O
ATOM    931  CB  THR B 650     111.984 101.797  78.965  1.00 20.00           C
ATOM    932  CG2 THR B 650     111.500 101.110  80.253  1.00 20.00           C
ATOM    933  OG1 THR B 650     111.973 100.815  77.953  1.00 20.00           O
ATOM    934  H   THR B 650     110.511 104.290  80.100  1.00  0.00           H
ATOM    935  HA  THR B 650     111.556 103.419  77.577  1.00  0.00           H
ATOM    936  HB  THR B 650     113.024 102.085  79.111  1.00  0.00           H
ATOM    937  HG1 THR B 650     112.436 101.159  77.185  1.00  0.00           H
ATOM    938 HG21 THR B 650     112.188 100.318  80.549  1.00  0.00           H
ATOM    939 HG22 THR B 650     111.446 101.818  81.079  1.00  0.00           H
ATOM    940 HG23 THR B 650     110.519 100.656  80.133  1.00  0.00           H
ATOM    941  N   GLU B 651     109.348 102.106  77.174  1.00 30.00           N
ATOM    942  CA  GLU B 651     107.983 101.751  76.762  1.00 30.00           C
ATOM    943  C   GLU B 651     107.856 100.302  76.243  1.00 30.00           C
ATOM    944  O   GLU B 651     106.728  99.859  76.031  1.00 30.00           O
ATOM    945  CB  GLU B 651     107.481 102.752  75.696  1.00 20.00           C
ATOM    946  CG  GLU B 651     107.339 104.203  76.206  1.00  0.00           C
ATOM    947  CD  GLU B 651     106.743 105.163  75.172  1.00  0.00           C
ATOM    948  OE1 GLU B 651     106.907 104.901  73.959  1.00  0.00           O
ATOM    949  OE2 GLU B 651     106.132 106.159  75.617  1.00  0.00           O1-
ATOM    950  H   GLU B 651     110.077 101.887  76.512  1.00  0.00           H
ATOM    951  HA  GLU B 651     107.310 101.814  77.617  1.00  0.00           H
ATOM    952  HB2 GLU B 651     108.162 102.734  74.844  1.00  0.00           H
ATOM    953  HB3 GLU B 651     106.513 102.424  75.311  1.00  0.00           H
ATOM    954  HG2 GLU B 651     106.711 104.217  77.098  1.00  0.00           H
ATOM    955  HG3 GLU B 651     108.310 104.600  76.502  1.00  0.00           H
ATOM    956  N   ASN B 652     108.984  99.595  76.036  1.00 30.00           N
ATOM    957  CA  ASN B 652     109.061  98.237  75.468  1.00 30.00           C
ATOM    958  C   ASN B 652     108.699  97.144  76.496  1.00 30.00           C
ATOM    959  O   ASN B 652     109.583  96.496  77.061  1.00 30.00           O
ATOM    960  CB  ASN B 652     110.427  98.006  74.756  1.00 20.00           C
ATOM    961  CG  ASN B 652     111.651  98.650  75.420  1.00 20.00           C
ATOM    962  ND2 ASN B 652     112.036  98.151  76.593  1.00 20.00           N
ATOM    963  OD1 ASN B 652     112.232  99.595  74.890  1.00 20.00           O
ATOM    964  H   ASN B 652     109.871 100.015  76.270  1.00  0.00           H
ATOM    965  HA  ASN B 652     108.310  98.236  74.675  1.00  0.00           H
ATOM    966  HB2 ASN B 652     110.360  98.452  73.763  1.00  0.00           H
ATOM    967  HB3 ASN B 652     110.609  96.944  74.576  1.00  0.00           H
ATOM    968 HD21 ASN B 652     112.815  98.554  77.091  1.00  0.00           H
ATOM    969 HD22 ASN B 652     111.530  97.375  76.998  1.00  0.00           H
ATOM    970  N   TYR B 653     107.387  96.975  76.709  1.00 30.00           N
ATOM    971  CA  TYR B 653     106.773  96.030  77.642  1.00 30.00           C
ATOM    972  C   TYR B 653     105.267  95.929  77.361  1.00 30.00           C
ATOM    973  O   TYR B 653     104.687  96.840  76.766  1.00 30.00           O
ATOM    974  CB  TYR B 653     107.039  96.458  79.115  1.00 20.00           C
ATOM    975  CG  TYR B 653     106.724  97.907  79.470  1.00 20.00           C
ATOM    976  CD1 TYR B 653     107.777  98.811  79.718  1.00 20.00           C
ATOM    977  CD2 TYR B 653     105.391  98.368  79.530  1.00 20.00           C
ATOM    978  CE1 TYR B 653     107.498 100.166  79.975  1.00 20.00           C
ATOM    979  CE2 TYR B 653     105.118  99.731  79.745  1.00 20.00           C
ATOM    980  CZ  TYR B 653     106.172 100.636  79.958  1.00 20.00           C
ATOM    981  OH  TYR B 653     105.908 101.962  80.139  1.00 20.00           O
ATOM    982  H   TYR B 653     106.730  97.563  76.214  1.00  0.00           H
ATOM    983  HA  TYR B 653     107.215  95.048  77.466  1.00  0.00           H
ATOM    984  HB2 TYR B 653     108.088  96.288  79.345  1.00  0.00           H
```

ATOM	985	HB3	TYR	B	653	106.499	95.807	79.804	1.00	0.00	H
ATOM	986	HD1	TYR	B	653	108.804	98.476	79.694	1.00	0.00	H
ATOM	987	HD2	TYR	B	653	104.566	97.691	79.370	1.00	0.00	H
ATOM	988	HE1	TYR	B	653	108.308	100.846	80.170	1.00	0.00	H
ATOM	989	HE2	TYR	B	653	104.101	100.083	79.730	1.00	0.00	H
ATOM	990	HH	TYR	B	653	106.704	102.490	80.245	1.00	0.00	H
ATOM	991	N	ASP	B	654	104.654	94.864	77.898	1.00	30.00	N
ATOM	992	CA	ASP	B	654	103.224	94.822	78.218	1.00	30.00	C
ATOM	993	C	ASP	B	654	103.016	95.215	79.692	1.00	30.00	C
ATOM	994	O	ASP	B	654	103.935	95.095	80.504	1.00	30.00	O
ATOM	995	CB	ASP	B	654	102.569	93.446	77.938	1.00	20.00	C
ATOM	996	CG	ASP	B	654	102.626	92.966	76.484	1.00	0.00	C
ATOM	997	OD1	ASP	B	654	102.849	93.810	75.589	1.00	0.00	O
ATOM	998	OD2	ASP	B	654	102.324	91.769	76.289	1.00	0.00	O1-
ATOM	999	H	ASP	B	654	105.212	94.143	78.332	1.00	0.00	H
ATOM	1000	HA	ASP	B	654	102.695	95.563	77.615	1.00	0.00	H
ATOM	1001	HB2	ASP	B	654	103.070	92.696	78.552	1.00	0.00	H
ATOM	1002	HB3	ASP	B	654	101.518	93.471	78.231	1.00	0.00	H
ATOM	1003	N	PHE	B	655	101.779	95.637	80.003	1.00	30.00	N
ATOM	1004	CA	PHE	B	655	101.259	95.950	81.340	1.00	30.00	C
ATOM	1005	C	PHE	B	655	101.935	97.203	81.934	1.00	30.00	C
ATOM	1006	O	PHE	B	655	102.765	97.088	82.837	1.00	30.00	O
ATOM	1007	CB	PHE	B	655	101.339	94.715	82.282	1.00	20.00	C
ATOM	1008	CG	PHE	B	655	100.761	93.436	81.698	1.00	20.00	C
ATOM	1009	CD1	PHE	B	655	99.368	93.301	81.518	1.00	20.00	C
ATOM	1010	CD2	PHE	B	655	101.620	92.448	81.169	1.00	20.00	C
ATOM	1011	CE1	PHE	B	655	98.861	92.204	80.833	1.00	20.00	C
ATOM	1012	CE2	PHE	B	655	101.094	91.362	80.484	1.00	20.00	C
ATOM	1013	CZ	PHE	B	655	99.721	91.248	80.308	1.00	20.00	C
ATOM	1014	H	PHE	B	655	101.097	95.689	79.260	1.00	0.00	H
ATOM	1015	HA	PHE	B	655	100.204	96.190	81.204	1.00	0.00	H
ATOM	1016	HB2	PHE	B	655	100.789	94.941	83.194	1.00	0.00	H
ATOM	1017	HB3	PHE	B	655	102.366	94.518	82.595	1.00	0.00	H
ATOM	1018	HD1	PHE	B	655	98.694	94.058	81.891	1.00	0.00	H
ATOM	1019	HD2	PHE	B	655	102.692	92.547	81.267	1.00	0.00	H
ATOM	1020	HE1	PHE	B	655	97.795	92.102	80.697	1.00	0.00	H
ATOM	1021	HE2	PHE	B	655	101.755	90.613	80.073	1.00	0.00	H
ATOM	1022	HZ	PHE	B	655	99.321	90.405	79.764	1.00	0.00	H
ATOM	1023	N	LYS	B	656	101.559	98.385	81.409	1.00	30.00	N
ATOM	1024	CA	LYS	B	656	102.062	99.688	81.865	1.00	30.00	C
ATOM	1025	C	LYS	B	656	101.674	100.029	83.316	1.00	30.00	C
ATOM	1026	O	LYS	B	656	102.452	100.695	83.999	1.00	30.00	O
ATOM	1027	CB	LYS	B	656	101.629	100.806	80.889	1.00	20.00	C
ATOM	1028	CG	LYS	B	656	102.288	102.164	81.206	1.00	20.00	C
ATOM	1029	CD	LYS	B	656	102.082	103.234	80.128	1.00	20.00	C
ATOM	1030	CE	LYS	B	656	102.870	104.508	80.463	1.00	20.00	C
ATOM	1031	NZ	LYS	B	656	102.697	105.548	79.439	1.00	20.00	N1+
ATOM	1032	H	LYS	B	656	100.889	98.405	80.653	1.00	0.00	H
ATOM	1033	HA	LYS	B	656	103.150	99.625	81.836	1.00	0.00	H
ATOM	1034	HB2	LYS	B	656	101.903	100.514	79.875	1.00	0.00	H
ATOM	1035	HB3	LYS	B	656	100.543	100.910	80.887	1.00	0.00	H
ATOM	1036	HG2	LYS	B	656	101.900	102.555	82.147	1.00	0.00	H
ATOM	1037	HG3	LYS	B	656	103.357	102.014	81.360	1.00	0.00	H
ATOM	1038	HD2	LYS	B	656	102.399	102.846	79.159	1.00	0.00	H
ATOM	1039	HD3	LYS	B	656	101.020	103.465	80.043	1.00	0.00	H
ATOM	1040	HE2	LYS	B	656	102.559	104.912	81.427	1.00	0.00	H
ATOM	1041	HE3	LYS	B	656	103.935	104.283	80.543	1.00	0.00	H
ATOM	1042	HZ1	LYS	B	656	103.269	106.342	79.711	1.00	0.00	H
ATOM	1043	HZ2	LYS	B	656	103.012	105.204	78.543	1.00	0.00	H
ATOM	1044	HZ3	LYS	B	656	101.729	105.827	79.385	1.00	0.00	H
ATOM	1045	N	ALA	B	657	100.504	99.539	83.766	1.00	30.00	N
ATOM	1046	CA	ALA	B	657	100.031	99.651	85.145	1.00	30.00	C
ATOM	1047	C	ALA	B	657	100.982	98.984	86.151	1.00	30.00	C
ATOM	1048	O	ALA	B	657	101.282	99.594	87.171	1.00	30.00	O
ATOM	1049	CB	ALA	B	657	98.618	99.059	85.250	1.00	30.00	C
ATOM	1050	H	ALA	B	657	99.920	99.007	83.138	1.00	0.00	H
ATOM	1051	HA	ALA	B	657	99.973	100.713	85.390	1.00	0.00	H
ATOM	1052	HB1	ALA	B	657	98.231	99.138	86.267	1.00	0.00	H
ATOM	1053	HB2	ALA	B	657	97.923	99.587	84.596	1.00	0.00	H
ATOM	1054	HB3	ALA	B	657	98.601	98.005	84.970	1.00	0.00	H
ATOM	1055	N	VAL	B	658	101.483	97.782	85.815	1.00	30.00	N

```
ATOM   1056  CA  VAL B 658     102.462  97.038  86.611  1.00 30.00           C
ATOM   1057  C   VAL B 658     103.830  97.749  86.641  1.00 30.00           C
ATOM   1058  O   VAL B 658     104.412  97.859  87.718  1.00 30.00           O
ATOM   1059  CB  VAL B 658     102.660  95.593  86.066  1.00 20.00           C
ATOM   1060  CG1 VAL B 658     103.700  94.749  86.837  1.00 20.00           C
ATOM   1061  CG2 VAL B 658     101.329  94.824  86.029  1.00 20.00           C
ATOM   1062  H   VAL B 658     101.212  97.359  84.940  1.00  0.00           H
ATOM   1063  HA  VAL B 658     102.087  96.976  87.634  1.00  0.00           H
ATOM   1064  HB  VAL B 658     103.012  95.667  85.037  1.00  0.00           H
ATOM   1065 HG11 VAL B 658     103.807  93.761  86.391  1.00  0.00           H
ATOM   1066 HG12 VAL B 658     104.691  95.197  86.843  1.00  0.00           H
ATOM   1067 HG13 VAL B 658     103.405  94.607  87.875  1.00  0.00           H
ATOM   1068 HG21 VAL B 658     101.459  93.874  85.516  1.00  0.00           H
ATOM   1069 HG22 VAL B 658     100.957  94.622  87.034  1.00  0.00           H
ATOM   1070 HG23 VAL B 658     100.550  95.365  85.492  1.00  0.00           H
ATOM   1071  N   PHE B 659     104.284  98.251  85.476  1.00 30.00           N
ATOM   1072  CA  PHE B 659     105.542  98.983  85.290  1.00 30.00           C
ATOM   1073  C   PHE B 659     105.676 100.223  86.182  1.00 30.00           C
ATOM   1074  O   PHE B 659     106.705 100.394  86.834  1.00 30.00           O
ATOM   1075  CB  PHE B 659     105.733  99.323  83.790  1.00 20.00           C
ATOM   1076  CG  PHE B 659     106.892 100.242  83.438  1.00 20.00           C
ATOM   1077  CD1 PHE B 659     108.216  99.753  83.425  1.00 20.00           C
ATOM   1078  CD2 PHE B 659     106.668 101.621  83.236  1.00 20.00           C
ATOM   1079  CE1 PHE B 659     109.270 100.615  83.148  1.00 20.00           C
ATOM   1080  CE2 PHE B 659     107.732 102.463  82.944  1.00 20.00           C
ATOM   1081  CZ  PHE B 659     109.026 101.961  82.898  1.00 20.00           C
ATOM   1082  H   PHE B 659     103.729  98.117  84.641  1.00  0.00           H
ATOM   1083  HA  PHE B 659     106.350  98.312  85.586  1.00  0.00           H
ATOM   1084  HB2 PHE B 659     105.861  98.409  83.213  1.00  0.00           H
ATOM   1085  HB3 PHE B 659     104.823  99.774  83.401  1.00  0.00           H
ATOM   1086  HD1 PHE B 659     108.412  98.709  83.615  1.00  0.00           H
ATOM   1087  HD2 PHE B 659     105.666 102.024  83.283  1.00  0.00           H
ATOM   1088  HE1 PHE B 659     110.281 100.239  83.120  1.00  0.00           H
ATOM   1089  HE2 PHE B 659     107.552 103.511  82.760  1.00  0.00           H
ATOM   1090  HZ  PHE B 659     109.845 102.628  82.673  1.00  0.00           H
ATOM   1091  N   ILE B 660     104.626 101.056  86.190  1.00 30.00           N
ATOM   1092  CA  ILE B 660     104.617 102.317  86.919  1.00 30.00           C
ATOM   1093  C   ILE B 660     104.237 102.143  88.404  1.00 30.00           C
ATOM   1094  O   ILE B 660     104.624 102.993  89.203  1.00 30.00           O
ATOM   1095  CB  ILE B 660     103.690 103.346  86.217  1.00 20.00           C
ATOM   1096  CG1 ILE B 660     104.111 104.802  86.517  1.00 20.00           C
ATOM   1097  CG2 ILE B 660     102.179 103.143  86.455  1.00 20.00           C
ATOM   1098  CD1 ILE B 660     103.808 105.765  85.362  1.00 20.00           C
ATOM   1099  H   ILE B 660     103.813 100.852  85.623  1.00  0.00           H
ATOM   1100  HA  ILE B 660     105.634 102.712  86.898  1.00  0.00           H
ATOM   1101  HB  ILE B 660     103.848 103.182  85.150  1.00  0.00           H
ATOM   1102 HG12 ILE B 660     103.634 105.151  87.433  1.00  0.00           H
ATOM   1103 HG13 ILE B 660     105.183 104.857  86.709  1.00  0.00           H
ATOM   1104 HG21 ILE B 660     101.590 103.791  85.806  1.00  0.00           H
ATOM   1105 HG22 ILE B 660     101.881 102.117  86.244  1.00  0.00           H
ATOM   1106 HG23 ILE B 660     101.891 103.367  87.483  1.00  0.00           H
ATOM   1107 HD11 ILE B 660     104.649 106.436  85.192  1.00  0.00           H
ATOM   1108 HD12 ILE B 660     103.633 105.250  84.417  1.00  0.00           H
ATOM   1109 HD13 ILE B 660     102.926 106.366  85.580  1.00  0.00           H
ATOM   1110  N   ILE B 661     103.564 101.035  88.765  1.00 30.00           N
ATOM   1111  CA  ILE B 661     103.384 100.616  90.157  1.00 30.00           C
ATOM   1112  C   ILE B 661     104.731 100.273  90.817  1.00 30.00           C
ATOM   1113  O   ILE B 661     104.975 100.753  91.923  1.00 30.00           O
ATOM   1114  CB  ILE B 661     102.372  99.434  90.321  1.00 20.00           C
ATOM   1115  CG1 ILE B 661     100.923  99.961  90.200  1.00 20.00           C
ATOM   1116  CG2 ILE B 661     102.493  98.601  91.622  1.00 20.00           C
ATOM   1117  CD1 ILE B 661      99.879  98.862  89.936  1.00 20.00           C
ATOM   1118  H   ILE B 661     103.225 100.398  88.055  1.00  0.00           H
ATOM   1119  HA  ILE B 661     102.984 101.475  90.699  1.00  0.00           H
ATOM   1120  HB  ILE B 661     102.546  98.747  89.491  1.00  0.00           H
ATOM   1121 HG12 ILE B 661     100.656 100.498  91.110  1.00  0.00           H
ATOM   1122 HG13 ILE B 661     100.858 100.704  89.404  1.00  0.00           H
ATOM   1123 HG21 ILE B 661     101.731  97.823  91.673  1.00  0.00           H
ATOM   1124 HG22 ILE B 661     103.451  98.088  91.703  1.00  0.00           H
ATOM   1125 HG23 ILE B 661     102.378  99.229  92.506  1.00  0.00           H
ATOM   1126 HD11 ILE B 661      99.036  99.261  89.372  1.00  0.00           H
```

```
ATOM   1127 HD12 ILE B 661     100.296  98.039  89.354  1.00  0.00           H
ATOM   1128 HD13 ILE B 661      99.490  98.452  90.868  1.00  0.00           H
ATOM   1129  N   LEU B 662     105.595  99.512  90.120  1.00 30.00           N
ATOM   1130  CA  LEU B 662     106.955  99.201  90.580  1.00 30.00           C
ATOM   1131  C   LEU B 662     107.772 100.473  90.856  1.00 30.00           C
ATOM   1132  O   LEU B 662     108.427 100.556  91.894  1.00 30.00           O
ATOM   1133  CB  LEU B 662     107.715  98.339  89.548  1.00 20.00           C
ATOM   1134  CG  LEU B 662     107.236  96.882  89.393  1.00 20.00           C
ATOM   1135  CD1 LEU B 662     107.893  96.253  88.144  1.00 20.00           C
ATOM   1136  CD2 LEU B 662     107.457  96.043  90.671  1.00 20.00           C
ATOM   1137  H   LEU B 662     105.320  99.142  89.215  1.00  0.00           H
ATOM   1138  HA  LEU B 662     106.873  98.655  91.521  1.00  0.00           H
ATOM   1139  HB2 LEU B 662     107.667  98.841  88.582  1.00  0.00           H
ATOM   1140  HB3 LEU B 662     108.777  98.317  89.802  1.00  0.00           H
ATOM   1141  HG  LEU B 662     106.163  96.899  89.209  1.00  0.00           H
ATOM   1142 HD11 LEU B 662     108.539  95.410  88.381  1.00  0.00           H
ATOM   1143 HD12 LEU B 662     107.133  95.905  87.446  1.00  0.00           H
ATOM   1144 HD13 LEU B 662     108.512  96.963  87.595  1.00  0.00           H
ATOM   1145 HD21 LEU B 662     107.879  95.061  90.464  1.00  0.00           H
ATOM   1146 HD22 LEU B 662     108.126  96.535  91.377  1.00  0.00           H
ATOM   1147 HD23 LEU B 662     106.512  95.875  91.187  1.00  0.00           H
ATOM   1148  N   LEU B 663     107.687 101.450  89.943  1.00 30.00           N
ATOM   1149  CA  LEU B 663     108.409 102.718  90.063  1.00 30.00           C
ATOM   1150  C   LEU B 663     107.910 103.573  91.236  1.00 30.00           C
ATOM   1151  O   LEU B 663     108.740 104.110  91.965  1.00 30.00           O
ATOM   1152  CB  LEU B 663     108.314 103.509  88.743  1.00 20.00           C
ATOM   1153  CG  LEU B 663     109.095 102.879  87.570  1.00 20.00           C
ATOM   1154  CD1 LEU B 663     108.612 103.446  86.225  1.00 20.00           C
ATOM   1155  CD2 LEU B 663     110.623 103.010  87.750  1.00 20.00           C
ATOM   1156  H   LEU B 663     107.095 101.347  89.131  1.00  0.00           H
ATOM   1157  HA  LEU B 663     109.457 102.486  90.260  1.00  0.00           H
ATOM   1158  HB2 LEU B 663     107.261 103.601  88.473  1.00  0.00           H
ATOM   1159  HB3 LEU B 663     108.669 104.531  88.886  1.00  0.00           H
ATOM   1160  HG  LEU B 663     108.870 101.812  87.550  1.00  0.00           H
ATOM   1161 HD11 LEU B 663     109.432 103.703  85.554  1.00  0.00           H
ATOM   1162 HD12 LEU B 663     107.995 102.712  85.708  1.00  0.00           H
ATOM   1163 HD13 LEU B 663     108.003 104.342  86.353  1.00  0.00           H
ATOM   1164 HD21 LEU B 663     111.089 103.630  86.985  1.00  0.00           H
ATOM   1165 HD22 LEU B 663     110.891 103.457  88.708  1.00  0.00           H
ATOM   1166 HD23 LEU B 663     111.099 102.030  87.705  1.00  0.00           H
ATOM   1167  N   LEU B 664     106.585 103.714  91.372  1.00 30.00           N
ATOM   1168  CA  LEU B 664     105.974 104.505  92.439  1.00 30.00           C
ATOM   1169  C   LEU B 664     106.207 103.846  93.807  1.00 30.00           C
ATOM   1170  O   LEU B 664     106.597 104.545  94.739  1.00 30.00           O
ATOM   1171  CB  LEU B 664     104.474 104.751  92.146  1.00 20.00           C
ATOM   1172  CG  LEU B 664     104.165 106.085  91.419  1.00 20.00           C
ATOM   1173  CD1 LEU B 664     104.944 106.302  90.104  1.00 20.00           C
ATOM   1174  CD2 LEU B 664     102.647 106.269  91.221  1.00 20.00           C
ATOM   1175  H   LEU B 664     105.956 103.281  90.703  1.00  0.00           H
ATOM   1176  HA  LEU B 664     106.477 105.474  92.474  1.00  0.00           H
ATOM   1177  HB2 LEU B 664     104.051 103.907  91.600  1.00  0.00           H
ATOM   1178  HB3 LEU B 664     103.931 104.774  93.093  1.00  0.00           H
ATOM   1179  HG  LEU B 664     104.479 106.880  92.098  1.00  0.00           H
ATOM   1180 HD11 LEU B 664     104.321 106.704  89.305  1.00  0.00           H
ATOM   1181 HD12 LEU B 664     105.746 107.020  90.263  1.00  0.00           H
ATOM   1182 HD13 LEU B 664     105.397 105.390  89.722  1.00  0.00           H
ATOM   1183 HD21 LEU B 664     102.327 107.251  91.572  1.00  0.00           H
ATOM   1184 HD22 LEU B 664     102.347 106.182  90.177  1.00  0.00           H
ATOM   1185 HD23 LEU B 664     102.069 105.528  91.774  1.00  0.00           H
ATOM   1186  N   ALA B 665     106.051 102.515  93.885  1.00 30.00           N
ATOM   1187  CA  ALA B 665     106.377 101.711  95.063  1.00 30.00           C
ATOM   1188  C   ALA B 665     107.833 101.898  95.508  1.00 30.00           C
ATOM   1189  O   ALA B 665     108.066 102.236  96.665  1.00 30.00           O
ATOM   1190  CB  ALA B 665     106.088 100.230  94.782  1.00 20.00           C
ATOM   1191  H   ALA B 665     105.738 101.998  93.066  1.00  0.00           H
ATOM   1192  HA  ALA B 665     105.741 102.045  95.884  1.00  0.00           H
ATOM   1193  HB1 ALA B 665     106.437  99.602  95.602  1.00  0.00           H
ATOM   1194  HB2 ALA B 665     105.021 100.053  94.655  1.00  0.00           H
ATOM   1195  HB3 ALA B 665     106.589  99.894  93.876  1.00  0.00           H
ATOM   1196  N   TYR B 666     108.770 101.748  94.562  1.00 30.00           N
ATOM   1197  CA  TYR B 666     110.195 101.988  94.769  1.00 30.00           C
```

```
ATOM   1198  C   TYR B 666     110.484 103.413  95.276  1.00 30.00           C
ATOM   1199  O   TYR B 666     111.074 103.552  96.346  1.00 30.00           O
ATOM   1200  CB  TYR B 666     110.978 101.637  93.486  1.00 20.00           C
ATOM   1201  CG  TYR B 666     112.459 101.972  93.505  1.00 20.00           C
ATOM   1202  CD1 TYR B 666     113.360 101.217  94.288  1.00 20.00           C
ATOM   1203  CD2 TYR B 666     112.932 103.068  92.758  1.00 20.00           C
ATOM   1204  CE1 TYR B 666     114.725 101.567  94.330  1.00 20.00           C
ATOM   1205  CE2 TYR B 666     114.294 103.410  92.796  1.00 20.00           C
ATOM   1206  CZ  TYR B 666     115.194 102.664  93.582  1.00 20.00           C
ATOM   1207  OH  TYR B 666     116.512 103.020  93.622  1.00 20.00           O
ATOM   1208  H   TYR B 666     108.496 101.459  93.627  1.00  0.00           H
ATOM   1209  HA  TYR B 666     110.519 101.296  95.550  1.00  0.00           H
ATOM   1210  HB2 TYR B 666     110.873 100.577  93.271  1.00  0.00           H
ATOM   1211  HB3 TYR B 666     110.528 102.151  92.637  1.00  0.00           H
ATOM   1212  HD1 TYR B 666     113.006 100.377  94.869  1.00  0.00           H
ATOM   1213  HD2 TYR B 666     112.254 103.659  92.157  1.00  0.00           H
ATOM   1214  HE1 TYR B 666     115.408 101.002  94.947  1.00  0.00           H
ATOM   1215  HE2 TYR B 666     114.628 104.253  92.216  1.00  0.00           H
ATOM   1216  HH  TYR B 666     116.707 103.659  92.917  1.00  0.00           H
ATOM   1217  N   VAL B 667     110.024 104.441  94.543  1.00 30.00           N
ATOM   1218  CA  VAL B 667     110.223 105.852  94.885  1.00 30.00           C
ATOM   1219  C   VAL B 667     109.691 106.214  96.287  1.00 30.00           C
ATOM   1220  O   VAL B 667     110.423 106.848  97.053  1.00 30.00           O
ATOM   1221  CB  VAL B 667     109.617 106.803  93.806  1.00 20.00           C
ATOM   1222  CG1 VAL B 667     109.423 108.280  94.222  1.00 20.00           C
ATOM   1223  CG2 VAL B 667     110.475 106.766  92.530  1.00 20.00           C
ATOM   1224  H   VAL B 667     109.507 104.254  93.687  1.00  0.00           H
ATOM   1225  HA  VAL B 667     111.303 106.013  94.911  1.00  0.00           H
ATOM   1226  HB  VAL B 667     108.630 106.418  93.544  1.00  0.00           H
ATOM   1227 HG11 VAL B 667     109.075 108.881  93.382  1.00  0.00           H
ATOM   1228 HG12 VAL B 667     108.677 108.395  95.010  1.00  0.00           H
ATOM   1229 HG13 VAL B 667     110.355 108.722  94.575  1.00  0.00           H
ATOM   1230 HG21 VAL B 667     110.004 107.322  91.719  1.00  0.00           H
ATOM   1231 HG22 VAL B 667     111.455 107.204  92.714  1.00  0.00           H
ATOM   1232 HG23 VAL B 667     110.643 105.752  92.170  1.00  0.00           H
ATOM   1233  N   ILE B 668     108.472 105.763  96.615  1.00  0.00           N
ATOM   1234  CA  ILE B 668     107.878 105.981  97.931  1.00  0.00           C
ATOM   1235  C   ILE B 668     108.627 105.218  99.051  1.00  0.00           C
ATOM   1236  O   ILE B 668     108.808 105.769 100.134  1.00  0.00           O
ATOM   1237  CB  ILE B 668     106.347 105.689  97.961  1.00 20.00           C
ATOM   1238  CG1 ILE B 668     105.557 106.629  97.010  1.00 20.00           C
ATOM   1239  CG2 ILE B 668     105.715 105.721  99.371  1.00 20.00           C
ATOM   1240  CD1 ILE B 668     105.611 108.128  97.347  1.00 20.00           C
ATOM   1241  H   ILE B 668     107.925 105.221  95.950  1.00  0.00           H
ATOM   1242  HA  ILE B 668     107.994 107.039  98.139  1.00  0.00           H
ATOM   1243  HB  ILE B 668     106.204 104.675  97.580  1.00  0.00           H
ATOM   1244 HG12 ILE B 668     104.516 106.307  96.966  1.00  0.00           H
ATOM   1245 HG13 ILE B 668     105.928 106.523  95.993  1.00  0.00           H
ATOM   1246 HG21 ILE B 668     104.629 105.666  99.324  1.00  0.00           H
ATOM   1247 HG22 ILE B 668     106.056 104.888  99.984  1.00  0.00           H
ATOM   1248 HG23 ILE B 668     105.969 106.635  99.906  1.00  0.00           H
ATOM   1249 HD11 ILE B 668     104.790 108.658  96.866  1.00  0.00           H
ATOM   1250 HD12 ILE B 668     105.532 108.320  98.416  1.00  0.00           H
ATOM   1251 HD13 ILE B 668     106.536 108.578  96.986  1.00  0.00           H
ATOM   1252  N   LEU B 669     109.100 104.000  98.760  1.00  0.00           N
ATOM   1253  CA  LEU B 669     109.857 103.159  99.687  1.00  0.00           C
ATOM   1254  C   LEU B 669     111.271 103.695 100.001  1.00  0.00           C
ATOM   1255  O   LEU B 669     111.751 103.506 101.120  1.00  0.00           O
ATOM   1256  CB  LEU B 669     109.889 101.722  99.112  1.00 20.00           C
ATOM   1257  CG  LEU B 669     110.614 100.633  99.932  1.00 20.00           C
ATOM   1258  CD1 LEU B 669     109.936 100.384 101.295  1.00 20.00           C
ATOM   1259  CD2 LEU B 669     110.752  99.343  99.100  1.00 20.00           C
ATOM   1260  H   LEU B 669     108.891 103.585  97.856  1.00  0.00           H
ATOM   1261  HA  LEU B 669     109.306 103.148 100.627  1.00  0.00           H
ATOM   1262  HB2 LEU B 669     108.865 101.392  98.941  1.00  0.00           H
ATOM   1263  HB3 LEU B 669     110.355 101.763  98.128  1.00  0.00           H
ATOM   1264  HG  LEU B 669     111.631 100.970 100.135  1.00  0.00           H
ATOM   1265 HD11 LEU B 669     110.662 100.464 102.104  1.00  0.00           H
ATOM   1266 HD12 LEU B 669     109.146 101.101 101.511  1.00  0.00           H
ATOM   1267 HD13 LEU B 669     109.476  99.399 101.353  1.00  0.00           H
ATOM   1268 HD21 LEU B 669     111.761  99.257  98.696  1.00  0.00           H
```

```
ATOM   1269 HD22 LEU B 669     110.568  98.439  99.680  1.00  0.00           H
ATOM   1270 HD23 LEU B 669     110.070  99.335  98.250  1.00  0.00           H
ATOM   1271  N   THR B 670     111.926 104.344  99.031  1.00  0.00           N
ATOM   1272  CA  THR B 670     113.358 104.652  99.118  1.00  0.00           C
ATOM   1273  C   THR B 670     113.672 106.161  99.135  1.00  0.00           C
ATOM   1274  O   THR B 670     114.486 106.576  99.958  1.00  0.00           O
ATOM   1275  CB  THR B 670     114.201 103.948  98.012  1.00 20.00           C
ATOM   1276  CG2 THR B 670     114.147 104.562  96.599  1.00 20.00           C
ATOM   1277  OG1 THR B 670     115.567 103.981  98.377  1.00 20.00           O
ATOM   1278  H   THR B 670     111.480 104.473  98.133  1.00  0.00           H
ATOM   1279  HA  THR B 670     113.744 104.265 100.059  1.00  0.00           H
ATOM   1280  HB  THR B 670     113.896 102.902  97.952  1.00  0.00           H
ATOM   1281  HG1 THR B 670     115.873 104.890  98.341  1.00  0.00           H
ATOM   1282 HG21 THR B 670     114.632 103.902  95.885  1.00  0.00           H
ATOM   1283 HG22 THR B 670     113.129 104.726  96.258  1.00  0.00           H
ATOM   1284 HG23 THR B 670     114.668 105.519  96.540  1.00  0.00           H
ATOM   1285  N   TYR B 671     113.063 106.952  98.235  1.00  0.00           N
ATOM   1286  CA  TYR B 671     113.467 108.343  98.016  1.00  0.00           C
ATOM   1287  C   TYR B 671     112.851 109.320  99.028  1.00  0.00           C
ATOM   1288  O   TYR B 671     113.551 110.223  99.481  1.00  0.00           O
ATOM   1289  CB  TYR B 671     113.181 108.771  96.561  1.00 20.00           C
ATOM   1290  CG  TYR B 671     113.685 110.168  96.242  1.00 20.00           C
ATOM   1291  CD1 TYR B 671     115.071 110.418  96.247  1.00 20.00           C
ATOM   1292  CD2 TYR B 671     112.787 111.231  96.014  1.00 20.00           C
ATOM   1293  CE1 TYR B 671     115.568 111.714  96.019  1.00 20.00           C
ATOM   1294  CE2 TYR B 671     113.286 112.530  95.802  1.00 20.00           C
ATOM   1295  CZ  TYR B 671     114.672 112.773  95.795  1.00 20.00           C
ATOM   1296  OH  TYR B 671     115.143 114.033  95.571  1.00 20.00           O
ATOM   1297  H   TYR B 671     112.366 106.576  97.606  1.00  0.00           H
ATOM   1298  HA  TYR B 671     114.550 108.395  98.151  1.00  0.00           H
ATOM   1299  HB2 TYR B 671     113.661 108.077  95.869  1.00  0.00           H
ATOM   1300  HB3 TYR B 671     112.113 108.718  96.353  1.00  0.00           H
ATOM   1301  HD1 TYR B 671     115.756 109.610  96.440  1.00  0.00           H
ATOM   1302  HD2 TYR B 671     111.721 111.061  96.023  1.00  0.00           H
ATOM   1303  HE1 TYR B 671     116.634 111.893  96.020  1.00  0.00           H
ATOM   1304  HE2 TYR B 671     112.607 113.348  95.644  1.00  0.00           H
ATOM   1305  HH  TYR B 671     114.450 114.645  95.313  1.00  0.00           H
ATOM   1306  N   ILE B 672     111.567 109.123  99.361  1.00  0.00           N
ATOM   1307  CA  ILE B 672     110.850 109.957 100.329  1.00  0.00           C
ATOM   1308  C   ILE B 672     110.715 109.267 101.701  1.00  0.00           C
ATOM   1309  O   ILE B 672     110.135 109.879 102.595  1.00  0.00           O
ATOM   1310  CB  ILE B 672     109.438 110.371  99.810  1.00 20.00           C
ATOM   1311  CG1 ILE B 672     108.409 109.216  99.725  1.00 20.00           C
ATOM   1312  CG2 ILE B 672     109.551 111.100  98.457  1.00 20.00           C
ATOM   1313  CD1 ILE B 672     107.487 109.059 100.948  1.00 20.00           C
ATOM   1314  H   ILE B 672     111.043 108.370  98.935  1.00  0.00           H
ATOM   1315  HA  ILE B 672     111.403 110.880 100.514  1.00  0.00           H
ATOM   1316  HB  ILE B 672     109.048 111.120 100.500  1.00  0.00           H
ATOM   1317 HG12 ILE B 672     108.942 108.284  99.551  1.00  0.00           H
ATOM   1318 HG13 ILE B 672     107.773 109.357  98.852  1.00  0.00           H
ATOM   1319 HG21 ILE B 672     108.591 111.525  98.165  1.00  0.00           H
ATOM   1320 HG22 ILE B 672     110.269 111.920  98.508  1.00  0.00           H
ATOM   1321 HG23 ILE B 672     109.869 110.426  97.660  1.00  0.00           H
ATOM   1322 HD11 ILE B 672     106.474 108.798 100.641  1.00  0.00           H
ATOM   1323 HD12 ILE B 672     107.840 108.265 101.606  1.00  0.00           H
ATOM   1324 HD13 ILE B 672     107.420 109.972 101.540  1.00  0.00           H
ATOM   1325  N   LEU B 673     111.222 108.035 101.850  1.00 30.00           N
ATOM   1326  CA  LEU B 673     111.184 107.276 103.098  1.00 30.00           C
ATOM   1327  C   LEU B 673     112.623 107.032 103.560  1.00 30.00           C
ATOM   1328  O   LEU B 673     113.073 107.736 104.458  1.00 30.00           O
ATOM   1329  CB  LEU B 673     110.315 106.006 102.931  1.00 20.00           C
ATOM   1330  CG  LEU B 673     110.368 104.955 104.071  1.00 20.00           C
ATOM   1331  CD1 LEU B 673     109.926 105.546 105.425  1.00 20.00           C
ATOM   1332  CD2 LEU B 673     109.547 103.704 103.697  1.00 20.00           C
ATOM   1333  H   LEU B 673     111.692 107.611 101.066  1.00  0.00           H
ATOM   1334  HA  LEU B 673     110.718 107.872 103.885  1.00  0.00           H
ATOM   1335  HB2 LEU B 673     109.280 106.316 102.778  1.00  0.00           H
ATOM   1336  HB3 LEU B 673     110.604 105.523 102.004  1.00  0.00           H
ATOM   1337  HG  LEU B 673     111.394 104.607 104.190  1.00  0.00           H
ATOM   1338 HD11 LEU B 673     110.781 105.953 105.965  1.00  0.00           H
ATOM   1339 HD12 LEU B 673     109.206 106.354 105.289  1.00  0.00           H
```

```
ATOM   1340 HD13 LEU B 673     109.459 104.808 106.077  1.00  0.00           H
ATOM   1341 HD21 LEU B 673     110.202 102.858 103.485  1.00  0.00           H
ATOM   1342 HD22 LEU B 673     108.868 103.393 104.487  1.00  0.00           H
ATOM   1343 HD23 LEU B 673     108.938 103.869 102.808  1.00  0.00           H
ATOM   1344  N   LEU B 674     113.341 106.071 102.956  1.00 30.00           N
ATOM   1345  CA  LEU B 674     114.665 105.637 103.429  1.00 30.00           C
ATOM   1346  C   LEU B 674     115.727 106.762 103.485  1.00 30.00           C
ATOM   1347  O   LEU B 674     116.496 106.822 104.443  1.00 30.00           O
ATOM   1348  CB  LEU B 674     115.154 104.426 102.600  1.00 20.00           C
ATOM   1349  CG  LEU B 674     116.163 103.516 103.342  1.00 20.00           C
ATOM   1350  CD1 LEU B 674     115.492 102.706 104.473  1.00 20.00           C
ATOM   1351  CD2 LEU B 674     116.963 102.629 102.363  1.00 20.00           C
ATOM   1352  H   LEU B 674     112.903 105.495 102.248  1.00  0.00           H
ATOM   1353  HA  LEU B 674     114.511 105.304 104.456  1.00  0.00           H
ATOM   1354  HB2 LEU B 674     114.307 103.811 102.293  1.00  0.00           H
ATOM   1355  HB3 LEU B 674     115.597 104.798 101.675  1.00  0.00           H
ATOM   1356  HG  LEU B 674     116.894 104.165 103.824  1.00  0.00           H
ATOM   1357 HD11 LEU B 674     115.645 101.632 104.371  1.00  0.00           H
ATOM   1358 HD12 LEU B 674     115.897 102.991 105.445  1.00  0.00           H
ATOM   1359 HD13 LEU B 674     114.414 102.865 104.514  1.00  0.00           H
ATOM   1360 HD21 LEU B 674     116.744 101.568 102.470  1.00  0.00           H
ATOM   1361 HD22 LEU B 674     116.772 102.881 101.320  1.00  0.00           H
ATOM   1362 HD23 LEU B 674     118.033 102.745 102.532  1.00  0.00           H
ATOM   1363  N   LEU B 675     115.714 107.673 102.504  1.00  0.00           N
ATOM   1364  CA  LEU B 675     116.612 108.830 102.425  1.00  0.00           C
ATOM   1365  C   LEU B 675     116.282 109.881 103.504  1.00  0.00           C
ATOM   1366  O   LEU B 675     117.206 110.502 104.027  1.00  0.00           O
ATOM   1367  CB  LEU B 675     116.560 109.435 101.002  1.00 20.00           C
ATOM   1368  CG  LEU B 675     117.666 108.967 100.024  1.00 20.00           C
ATOM   1369  CD1 LEU B 675     117.639 107.455  99.715  1.00 20.00           C
ATOM   1370  CD2 LEU B 675     117.657 109.813  98.733  1.00 20.00           C
ATOM   1371  H   LEU B 675     115.038 107.576 101.759  1.00  0.00           H
ATOM   1372  HA  LEU B 675     117.631 108.487 102.620  1.00  0.00           H
ATOM   1373  HB2 LEU B 675     115.584 109.251 100.559  1.00  0.00           H
ATOM   1374  HB3 LEU B 675     116.641 110.522 101.076  1.00  0.00           H
ATOM   1375  HG  LEU B 675     118.616 109.166 100.522  1.00  0.00           H
ATOM   1376 HD11 LEU B 675     118.652 107.068  99.611  1.00  0.00           H
ATOM   1377 HD12 LEU B 675     117.160 106.870 100.498  1.00  0.00           H
ATOM   1378 HD13 LEU B 675     117.109 107.233  98.788  1.00  0.00           H
ATOM   1379 HD21 LEU B 675     118.589 110.369  98.626  1.00  0.00           H
ATOM   1380 HD22 LEU B 675     117.546 109.206  97.837  1.00  0.00           H
ATOM   1381 HD23 LEU B 675     116.845 110.541  98.724  1.00  0.00           H
ATOM   1382  N   ASN B 676     114.991 110.019 103.856  1.00  0.00           N
ATOM   1383  CA  ASN B 676     114.512 110.861 104.959  1.00  0.00           C
ATOM   1384  C   ASN B 676     114.772 110.205 106.331  1.00  0.00           C
ATOM   1385  O   ASN B 676     115.010 110.929 107.297  1.00  0.00           O
ATOM   1386  CB  ASN B 676     113.007 111.174 104.765  1.00 20.00           C
ATOM   1387  CG  ASN B 676     112.682 112.089 103.574  1.00 20.00           C
ATOM   1388  ND2 ASN B 676     111.419 112.507 103.471  1.00 20.00           N
ATOM   1389  OD1 ASN B 676     113.543 112.434 102.767  1.00 20.00           O
ATOM   1390  H   ASN B 676     114.289 109.472 103.379  1.00  0.00           H
ATOM   1391  HA  ASN B 676     115.069 111.798 104.913  1.00  0.00           H
ATOM   1392  HB2 ASN B 676     112.631 111.680 105.656  1.00  0.00           H
ATOM   1393  HB3 ASN B 676     112.432 110.251 104.671  1.00  0.00           H
ATOM   1394 HD21 ASN B 676     111.151 113.125 102.720  1.00  0.00           H
ATOM   1395 HD22 ASN B 676     110.722 112.201 104.135  1.00  0.00           H
ATOM   1396  N   MET B 677     114.759 108.860 106.385  1.00  0.00           N
ATOM   1397  CA  MET B 677     115.076 108.050 107.563  1.00  0.00           C
ATOM   1398  C   MET B 677     116.571 108.085 107.912  1.00  0.00           C
ATOM   1399  O   MET B 677     116.907 108.055 109.094  1.00  0.00           O
ATOM   1400  CB  MET B 677     114.612 106.591 107.341  1.00 20.00           C
ATOM   1401  CG  MET B 677     114.410 105.774 108.627  1.00 20.00           C
ATOM   1402  SD  MET B 677     113.224 106.462 109.820  1.00 20.00           S
ATOM   1403  CE  MET B 677     111.770 106.663 108.754  1.00 20.00           C
ATOM   1404  H   MET B 677     114.508 108.342 105.553  1.00  0.00           H
ATOM   1405  HA  MET B 677     114.520 108.486 108.394  1.00  0.00           H
ATOM   1406  HB2 MET B 677     113.678 106.565 106.789  1.00  0.00           H
ATOM   1407  HB3 MET B 677     115.323 106.067 106.703  1.00  0.00           H
ATOM   1408  HG2 MET B 677     115.366 105.647 109.131  1.00  0.00           H
ATOM   1409  HG3 MET B 677     114.073 104.770 108.364  1.00  0.00           H
ATOM   1410  HE1 MET B 677     110.939 107.066 109.329  1.00  0.00           H
```

```
ATOM   1411  HE2 MET B 677     111.972 107.362 107.946  1.00  0.00           H
ATOM   1412  HE3 MET B 677     111.469 105.707 108.326  1.00  0.00           H
ATOM   1413  N   LEU B 678     117.429 108.166 106.879  1.00  0.00           N
ATOM   1414  CA  LEU B 678     118.876 108.307 107.004  1.00  0.00           C
ATOM   1415  C   LEU B 678     119.265 109.655 107.632  1.00  0.00           C
ATOM   1416  O   LEU B 678     120.141 109.667 108.489  1.00  0.00           O
ATOM   1417  CB  LEU B 678     119.538 108.085 105.624  1.00 20.00           C
ATOM   1418  CG  LEU B 678     121.085 108.084 105.626  1.00 20.00           C
ATOM   1419  CD1 LEU B 678     121.661 106.964 106.518  1.00 20.00           C
ATOM   1420  CD2 LEU B 678     121.645 108.037 104.189  1.00 20.00           C
ATOM   1421  H   LEU B 678     117.066 108.148 105.936  1.00  0.00           H
ATOM   1422  HA  LEU B 678     119.212 107.519 107.681  1.00  0.00           H
ATOM   1423  HB2 LEU B 678     119.190 107.138 105.210  1.00  0.00           H
ATOM   1424  HB3 LEU B 678     119.183 108.855 104.938  1.00  0.00           H
ATOM   1425  HG  LEU B 678     121.426 109.033 106.041  1.00  0.00           H
ATOM   1426 HD11 LEU B 678     122.549 106.499 106.091  1.00  0.00           H
ATOM   1427 HD12 LEU B 678     121.946 107.362 107.492  1.00  0.00           H
ATOM   1428 HD13 LEU B 678     120.936 106.169 106.694  1.00  0.00           H
ATOM   1429 HD21 LEU B 678     122.275 108.905 103.992  1.00  0.00           H
ATOM   1430 HD22 LEU B 678     122.251 107.151 104.002  1.00  0.00           H
ATOM   1431 HD23 LEU B 678     120.852 108.040 103.440  1.00  0.00           H
ATOM   1432  N   ILE B 679     118.576 110.743 107.240  1.00  0.00           N
ATOM   1433  CA  ILE B 679     118.743 112.083 107.812  1.00  0.00           C
ATOM   1434  C   ILE B 679     118.275 112.149 109.283  1.00  0.00           C
ATOM   1435  O   ILE B 679     118.959 112.763 110.101  1.00  0.00           O
ATOM   1436  CB  ILE B 679     117.984 113.160 106.975  1.00 20.00           C
ATOM   1437  CG1 ILE B 679     118.554 113.257 105.540  1.00 20.00           C
ATOM   1438  CG2 ILE B 679     117.955 114.570 107.612  1.00 20.00           C
ATOM   1439  CD1 ILE B 679     117.597 113.921 104.537  1.00 20.00           C
ATOM   1440  H   ILE B 679     117.868 110.649 106.525  1.00  0.00           H
ATOM   1441  HA  ILE B 679     119.809 112.322 107.792  1.00  0.00           H
ATOM   1442  HB  ILE B 679     116.949 112.827 106.889  1.00  0.00           H
ATOM   1443 HG12 ILE B 679     119.497 113.806 105.560  1.00  0.00           H
ATOM   1444 HG13 ILE B 679     118.808 112.270 105.156  1.00  0.00           H
ATOM   1445 HG21 ILE B 679     117.488 115.296 106.947  1.00  0.00           H
ATOM   1446 HG22 ILE B 679     117.388 114.596 108.542  1.00  0.00           H
ATOM   1447 HG23 ILE B 679     118.963 114.925 107.825  1.00  0.00           H
ATOM   1448 HD11 ILE B 679     117.765 113.536 103.530  1.00  0.00           H
ATOM   1449 HD12 ILE B 679     116.553 113.732 104.786  1.00  0.00           H
ATOM   1450 HD13 ILE B 679     117.744 115.000 104.506  1.00  0.00           H
ATOM   1451  N   ALA B 680     117.145 111.485 109.588  1.00  0.00           N
ATOM   1452  CA  ALA B 680     116.564 111.378 110.928  1.00  0.00           C
ATOM   1453  C   ALA B 680     117.451 110.605 111.921  1.00  0.00           C
ATOM   1454  O   ALA B 680     117.622 111.063 113.050  1.00  0.00           O
ATOM   1455  CB  ALA B 680     115.171 110.738 110.818  1.00 20.00           C
ATOM   1456  H   ALA B 680     116.638 111.013 108.852  1.00  0.00           H
ATOM   1457  HA  ALA B 680     116.438 112.391 111.317  1.00  0.00           H
ATOM   1458  HB1 ALA B 680     114.710 110.610 111.797  1.00  0.00           H
ATOM   1459  HB2 ALA B 680     114.500 111.357 110.220  1.00  0.00           H
ATOM   1460  HB3 ALA B 680     115.214 109.754 110.353  1.00  0.00           H
ATOM   1461  N   LEU B 681     118.011 109.466 111.475  1.00  0.00           N
ATOM   1462  CA  LEU B 681     118.867 108.595 112.283  1.00  0.00           C
ATOM   1463  C   LEU B 681     120.331 109.061 112.351  1.00  0.00           C
ATOM   1464  O   LEU B 681     120.983 108.774 113.354  1.00  0.00           O
ATOM   1465  CB  LEU B 681     118.748 107.138 111.785  1.00 20.00           C
ATOM   1466  CG  LEU B 681     117.422 106.458 112.200  1.00 20.00           C
ATOM   1467  CD1 LEU B 681     117.122 105.203 111.365  1.00 20.00           C
ATOM   1468  CD2 LEU B 681     117.368 106.155 113.711  1.00 20.00           C
ATOM   1469  H   LEU B 681     117.807 109.145 110.537  1.00  0.00           H
ATOM   1470  HA  LEU B 681     118.503 108.640 113.308  1.00  0.00           H
ATOM   1471  HB2 LEU B 681     118.863 107.130 110.700  1.00  0.00           H
ATOM   1472  HB3 LEU B 681     119.573 106.536 112.169  1.00  0.00           H
ATOM   1473  HG  LEU B 681     116.622 107.164 111.977  1.00  0.00           H
ATOM   1474 HD11 LEU B 681     116.047 105.033 111.306  1.00  0.00           H
ATOM   1475 HD12 LEU B 681     117.491 105.301 110.345  1.00  0.00           H
ATOM   1476 HD13 LEU B 681     117.567 104.308 111.798  1.00  0.00           H
ATOM   1477 HD21 LEU B 681     116.377 106.358 114.115  1.00  0.00           H
ATOM   1478 HD22 LEU B 681     117.602 105.114 113.925  1.00  0.00           H
ATOM   1479 HD23 LEU B 681     118.079 106.743 114.286  1.00  0.00           H
ATOM   1480  N   MET B 682     120.811 109.823 111.351  1.00  0.00           N
ATOM   1481  CA  MET B 682     122.071 110.568 111.446  1.00  0.00           C
```

```
ATOM   1482  C   MET B 682     121.984 111.684 112.498  1.00  0.00           C
ATOM   1483  O   MET B 682     122.941 111.851 113.246  1.00  0.00           O
ATOM   1484  CB  MET B 682     122.489 111.147 110.078  1.00 20.00           C
ATOM   1485  CG  MET B 682     123.210 110.127 109.186  1.00 20.00           C
ATOM   1486  SD  MET B 682     123.536 110.718 107.504  1.00 20.00           S
ATOM   1487  CE  MET B 682     124.605 109.375 106.923  1.00 20.00           C
ATOM   1488  H   MET B 682     120.248 109.995 110.528  1.00  0.00           H
ATOM   1489  HA  MET B 682     122.853 109.880 111.774  1.00  0.00           H
ATOM   1490  HB2 MET B 682     121.626 111.569 109.561  1.00  0.00           H
ATOM   1491  HB3 MET B 682     123.174 111.983 110.226  1.00  0.00           H
ATOM   1492  HG2 MET B 682     124.166 109.870 109.643  1.00  0.00           H
ATOM   1493  HG3 MET B 682     122.640 109.201 109.123  1.00  0.00           H
ATOM   1494  HE1 MET B 682     124.880 109.534 105.881  1.00  0.00           H
ATOM   1495  HE2 MET B 682     125.517 109.329 107.519  1.00  0.00           H
ATOM   1496  HE3 MET B 682     124.097 108.415 107.004  1.00  0.00           H
ATOM   1497  N   GLY B 683     120.837 112.384 112.570  1.00  0.00           N
ATOM   1498  CA  GLY B 683     120.581 113.456 113.533  1.00  0.00           C
ATOM   1499  C   GLY B 683     120.491 112.935 114.977  1.00  0.00           C
ATOM   1500  O   GLY B 683     120.967 113.611 115.887  1.00  0.00           O
ATOM   1501  H   GLY B 683     120.096 112.179 111.913  1.00  0.00           H
ATOM   1502  HA2 GLY B 683     121.361 114.215 113.456  1.00  0.00           H
ATOM   1503  HA3 GLY B 683     119.639 113.939 113.275  1.00  0.00           H
ATOM   1504  N   GLU B 684     119.923 111.732 115.179  1.00  0.00           N
ATOM   1505  CA  GLU B 684     119.814 111.065 116.479  1.00  0.00           C
ATOM   1506  C   GLU B 684     121.173 110.581 117.018  1.00  0.00           C
ATOM   1507  O   GLU B 684     121.482 110.842 118.182  1.00  0.00           O
ATOM   1508  CB  GLU B 684     118.706 109.986 116.396  1.00 20.00           C
ATOM   1509  CG  GLU B 684     118.536 109.022 117.597  1.00  0.00           C
ATOM   1510  CD  GLU B 684     119.460 107.799 117.569  1.00  0.00           C
ATOM   1511  OE1 GLU B 684     119.451 107.096 116.534  1.00  0.00           O
ATOM   1512  OE2 GLU B 684     120.155 107.583 118.585  1.00  0.00           O1-
ATOM   1513  H   GLU B 684     119.538 111.231 114.390  1.00  0.00           H
ATOM   1514  HA  GLU B 684     119.460 111.811 117.194  1.00  0.00           H
ATOM   1515  HB2 GLU B 684     117.763 110.515 116.266  1.00  0.00           H
ATOM   1516  HB3 GLU B 684     118.828 109.409 115.479  1.00  0.00           H
ATOM   1517  HG2 GLU B 684     118.659 109.564 118.535  1.00  0.00           H
ATOM   1518  HG3 GLU B 684     117.517 108.637 117.604  1.00  0.00           H
ATOM   1519  N   THR B 685     121.971 109.926 116.157  1.00  0.00           N
ATOM   1520  CA  THR B 685     123.313 109.445 116.496  1.00  0.00           C
ATOM   1521  C   THR B 685     124.377 110.568 116.547  1.00  0.00           C
ATOM   1522  O   THR B 685     125.403 110.353 117.189  1.00  0.00           O
ATOM   1523  CB  THR B 685     123.803 108.342 115.521  1.00 20.00           C
ATOM   1524  CG2 THR B 685     122.907 107.093 115.525  1.00 20.00           C
ATOM   1525  OG1 THR B 685     123.936 108.818 114.195  1.00 20.00           O
ATOM   1526  H   THR B 685     121.648 109.737 115.218  1.00  0.00           H
ATOM   1527  HA  THR B 685     123.267 109.003 117.493  1.00  0.00           H
ATOM   1528  HB  THR B 685     124.799 108.021 115.829  1.00  0.00           H
ATOM   1529  HG1 THR B 685     123.071 108.802 113.774  1.00  0.00           H
ATOM   1530 HG21 THR B 685     123.275 106.342 114.825  1.00  0.00           H
ATOM   1531 HG22 THR B 685     122.883 106.637 116.515  1.00  0.00           H
ATOM   1532 HG23 THR B 685     121.878 107.321 115.253  1.00  0.00           H
ATOM   1533  N   VAL B 686     124.112 111.747 115.947  1.00  0.00           N
ATOM   1534  CA  VAL B 686     124.929 112.964 116.078  1.00  0.00           C
ATOM   1535  C   VAL B 686     125.006 113.474 117.531  1.00  0.00           C
ATOM   1536  O   VAL B 686     126.081 113.897 117.954  1.00  0.00           O
ATOM   1537  CB  VAL B 686     124.438 114.109 115.123  1.00 20.00           C
ATOM   1538  CG1 VAL B 686     124.463 115.564 115.652  1.00 20.00           C
ATOM   1539  CG2 VAL B 686     125.190 114.066 113.782  1.00 20.00           C
ATOM   1540  H   VAL B 686     123.266 111.841 115.402  1.00  0.00           H
ATOM   1541  HA  VAL B 686     125.946 112.686 115.792  1.00  0.00           H
ATOM   1542  HB  VAL B 686     123.394 113.910 114.889  1.00  0.00           H
ATOM   1543 HG11 VAL B 686     124.138 116.263 114.881  1.00  0.00           H
ATOM   1544 HG12 VAL B 686     123.785 115.703 116.495  1.00  0.00           H
ATOM   1545 HG13 VAL B 686     125.464 115.864 115.961  1.00  0.00           H
ATOM   1546 HG21 VAL B 686     124.793 114.799 113.080  1.00  0.00           H
ATOM   1547 HG22 VAL B 686     126.251 114.274 113.916  1.00  0.00           H
ATOM   1548 HG23 VAL B 686     125.102 113.092 113.310  1.00  0.00           H
ATOM   1549  N   ASN B 687     123.880 113.400 118.263  1.00  0.00           N
ATOM   1550  CA  ASN B 687     123.779 113.814 119.667  1.00  0.00           C
ATOM   1551  C   ASN B 687     124.539 112.872 120.619  1.00  0.00           C
ATOM   1552  O   ASN B 687     125.044 113.341 121.638  1.00  0.00           O
```

```
ATOM   1553  CB  ASN B 687     122.296 113.923 120.096  1.00 20.00           C
ATOM   1554  CG  ASN B 687     121.453 114.882 119.247  1.00 20.00           C
ATOM   1555  ND2 ASN B 687     121.903 116.129 119.085  1.00 20.00           N
ATOM   1556  OD1 ASN B 687     120.394 114.503 118.754  1.00 20.00           O
ATOM   1557  H   ASN B 687     123.036 113.035 117.842  1.00  0.00           H
ATOM   1558  HA  ASN B 687     124.243 114.798 119.764  1.00  0.00           H
ATOM   1559  HB2 ASN B 687     121.830 112.937 120.066  1.00  0.00           H
ATOM   1560  HB3 ASN B 687     122.233 114.257 121.133  1.00  0.00           H
ATOM   1561 HD21 ASN B 687     121.369 116.785 118.534  1.00  0.00           H
ATOM   1562 HD22 ASN B 687     122.772 116.424 119.505  1.00  0.00           H
ATOM   1563  N   LYS B 688     124.625 111.579 120.259  1.00  0.00           N
ATOM   1564  CA  LYS B 688     125.386 110.561 120.982  1.00  0.00           C
ATOM   1565  C   LYS B 688     126.902 110.648 120.708  1.00  0.00           C
ATOM   1566  O   LYS B 688     127.688 110.404 121.622  1.00  0.00           O
ATOM   1567  CB  LYS B 688     124.805 109.175 120.625  1.00 20.00           C
ATOM   1568  CG  LYS B 688     125.485 107.988 121.332  1.00 20.00           C
ATOM   1569  CD  LYS B 688     124.785 106.653 121.048  1.00 20.00           C
ATOM   1570  CE  LYS B 688     125.467 105.478 121.761  1.00 20.00           C
ATOM   1571  NZ  LYS B 688     124.759 104.214 121.504  1.00 20.00           N1+
ATOM   1572  H   LYS B 688     124.172 111.276 119.408  1.00  0.00           H
ATOM   1573  HA  LYS B 688     125.235 110.719 122.052  1.00  0.00           H
ATOM   1574  HB2 LYS B 688     123.743 109.170 120.878  1.00  0.00           H
ATOM   1575  HB3 LYS B 688     124.856 109.020 119.546  1.00  0.00           H
ATOM   1576  HG2 LYS B 688     126.525 107.910 121.014  1.00  0.00           H
ATOM   1577  HG3 LYS B 688     125.503 108.170 122.407  1.00  0.00           H
ATOM   1578  HD2 LYS B 688     123.741 106.718 121.361  1.00  0.00           H
ATOM   1579  HD3 LYS B 688     124.771 106.474 119.972  1.00  0.00           H
ATOM   1580  HE2 LYS B 688     126.500 105.379 121.426  1.00  0.00           H
ATOM   1581  HE3 LYS B 688     125.491 105.651 122.837  1.00  0.00           H
ATOM   1582  HZ1 LYS B 688     124.750 104.025 120.512  1.00  0.00           H
ATOM   1583  HZ2 LYS B 688     125.226 103.459 121.987  1.00  0.00           H
ATOM   1584  HZ3 LYS B 688     123.808 104.287 121.839  1.00  0.00           H
ATOM   1585  N   ILE B 689     127.277 110.990 119.462  1.00  0.00           N
ATOM   1586  CA  ILE B 689     128.657 111.057 118.958  1.00  0.00           C
ATOM   1587  C   ILE B 689     129.076 112.506 119.142  1.00  0.00           C
ATOM   1588  O   ILE B 689     130.257 112.850 119.191  1.00  0.00           O
ATOM   1589  CB  ILE B 689     128.738 110.541 117.481  1.00 20.00           C
ATOM   1590  CG1 ILE B 689     128.417 109.026 117.401  1.00 20.00           C
ATOM   1591  CG2 ILE B 689     130.074 110.840 116.760  1.00 20.00           C
ATOM   1592  CD1 ILE B 689     128.065 108.518 115.994  1.00 20.00           C
ATOM   1593  H   ILE B 689     126.558 111.159 118.771  1.00  0.00           H
ATOM   1594  HA  ILE B 689     129.312 110.431 119.569  1.00  0.00           H
ATOM   1595  HB  ILE B 689     127.969 111.067 116.912  1.00  0.00           H
ATOM   1596 HG12 ILE B 689     129.261 108.455 117.790  1.00  0.00           H
ATOM   1597 HG13 ILE B 689     127.578 108.784 118.054  1.00  0.00           H
ATOM   1598 HG21 ILE B 689     130.074 110.456 115.740  1.00  0.00           H
ATOM   1599 HG22 ILE B 689     130.265 111.910 116.683  1.00  0.00           H
ATOM   1600 HG23 ILE B 689     130.915 110.387 117.285  1.00  0.00           H
ATOM   1601 HD11 ILE B 689     127.620 107.525 116.044  1.00  0.00           H
ATOM   1602 HD12 ILE B 689     127.342 109.171 115.506  1.00  0.00           H
ATOM   1603 HD13 ILE B 689     128.946 108.448 115.356  1.00  0.00           H
ATOM   1604  HXT ILE B 689     128.272 113.239 119.222  1.00  0.00           H
TER    1605      ILE B 689
ATOM   1606  N   THR C 335      97.045  79.138 134.838  1.00 36.89           N1+
ATOM   1607  CA  THR C 335      97.667  79.340 136.195  1.00 36.74           C
ATOM   1608  C   THR C 335      97.713  80.877 136.459  1.00 36.68           C
ATOM   1609  O   THR C 335      97.592  81.665 135.519  1.00 36.82           O
ATOM   1610  CB  THR C 335      99.108  78.746 136.295  1.00 36.74           C
ATOM   1611  CG2 THR C 335      99.147  77.225 136.083  1.00 36.34           C
ATOM   1612  OG1 THR C 335      99.998  79.334 135.360  1.00 37.07           O
ATOM   1613  HA  THR C 335      96.975  78.839 136.870  1.00  0.00           H
ATOM   1614  HB  THR C 335      99.522  78.939 137.285  1.00  0.00           H
ATOM   1615  HG1 THR C 335     100.802  78.808 135.330  1.00  0.00           H
ATOM   1616 HG21 THR C 335     100.158  76.835 136.209  1.00  0.00           H
ATOM   1617 HG22 THR C 335      98.510  76.713 136.805  1.00  0.00           H
ATOM   1618 HG23 THR C 335      98.810  76.945 135.085  1.00  0.00           H
ATOM   1619  H1  THR C 335      96.981  78.148 134.648  1.00  0.00           H
ATOM   1620  H2  THR C 335      96.123  79.551 134.826  1.00  0.00           H
ATOM   1621  H   THR C 335      97.636  79.577 134.146  1.00  0.00           H
ATOM   1622  N   PRO C 336      97.917  81.293 137.735  1.00 36.46           N
ATOM   1623  CA  PRO C 336      98.170  82.708 138.088  1.00 35.71           C
```

```
ATOM   1624  C   PRO C 336      99.431  83.333 137.457  1.00 35.26           C
ATOM   1625  O   PRO C 336      99.430  84.536 137.199  1.00 35.27           O
ATOM   1626  CB  PRO C 336      98.250  82.706 139.625  1.00 36.20           C
ATOM   1627  CG  PRO C 336      97.483  81.466 140.046  1.00 36.12           C
ATOM   1628  CD  PRO C 336      97.815  80.472 138.942  1.00 36.23           C
ATOM   1629  HA  PRO C 336      97.293  83.281 137.783  1.00  0.00           H
ATOM   1630  HB2 PRO C 336      99.285  82.617 139.960  1.00  0.00           H
ATOM   1631  HB3 PRO C 336      97.840  83.614 140.068  1.00  0.00           H
ATOM   1632  HG2 PRO C 336      97.744  81.110 141.044  1.00  0.00           H
ATOM   1633  HG3 PRO C 336      96.413  81.679 140.034  1.00  0.00           H
ATOM   1634  HD2 PRO C 336      97.053  79.695 138.891  1.00  0.00           H
ATOM   1635  HD3 PRO C 336      98.779  80.002 139.142  1.00  0.00           H
ATOM   1636  N   LEU C 337     100.463  82.508 137.199  1.00 35.26           N
ATOM   1637  CA  LEU C 337     101.693  82.890 136.502  1.00 34.53           C
ATOM   1638  C   LEU C 337     101.451  83.182 135.011  1.00 34.37           C
ATOM   1639  O   LEU C 337     101.933  84.200 134.515  1.00 34.02           O
ATOM   1640  CB  LEU C 337     102.758  81.789 136.734  1.00 34.61           C
ATOM   1641  CG  LEU C 337     104.091  81.918 135.953  1.00 34.07           C
ATOM   1642  CD1 LEU C 337     104.796  83.280 136.159  1.00 34.18           C
ATOM   1643  CD2 LEU C 337     105.016  80.717 136.252  1.00 32.60           C
ATOM   1644  H   LEU C 337     100.388  81.535 137.454  1.00  0.00           H
ATOM   1645  HA  LEU C 337     102.047  83.816 136.959  1.00  0.00           H
ATOM   1646  HB2 LEU C 337     102.969  81.736 137.802  1.00  0.00           H
ATOM   1647  HB3 LEU C 337     102.316  80.825 136.478  1.00  0.00           H
ATOM   1648  HG  LEU C 337     103.852  81.850 134.893  1.00  0.00           H
ATOM   1649 HD11 LEU C 337     104.799  83.855 135.232  1.00  0.00           H
ATOM   1650 HD12 LEU C 337     104.304  83.893 136.912  1.00  0.00           H
ATOM   1651 HD13 LEU C 337     105.833  83.182 136.472  1.00  0.00           H
ATOM   1652 HD21 LEU C 337     105.992  81.016 136.632  1.00  0.00           H
ATOM   1653 HD22 LEU C 337     104.582  80.042 136.990  1.00  0.00           H
ATOM   1654 HD23 LEU C 337     105.192  80.128 135.352  1.00  0.00           H
ATOM   1655  N   ALA C 338     100.679  82.307 134.341  1.00 33.95           N
ATOM   1656  CA  ALA C 338     100.247  82.480 132.953  1.00 34.02           C
ATOM   1657  C   ALA C 338      99.228  83.620 132.776  1.00 34.07           C
ATOM   1658  O   ALA C 338      99.200  84.229 131.708  1.00 34.62           O
ATOM   1659  CB  ALA C 338      99.678  81.153 132.431  1.00 34.42           C
ATOM   1660  H   ALA C 338     100.329  81.487 134.816  1.00  0.00           H
ATOM   1661  HA  ALA C 338     101.128  82.722 132.356  1.00  0.00           H
ATOM   1662  HB1 ALA C 338      99.381  81.236 131.384  1.00  0.00           H
ATOM   1663  HB2 ALA C 338     100.420  80.357 132.494  1.00  0.00           H
ATOM   1664  HB3 ALA C 338      98.803  80.841 133.001  1.00  0.00           H
ATOM   1665  N   LEU C 339      98.434  83.909 133.823  1.00 33.85           N
ATOM   1666  CA  LEU C 339      97.490  85.024 133.872  1.00 33.53           C
ATOM   1667  C   LEU C 339      98.217  86.372 133.999  1.00 33.66           C
ATOM   1668  O   LEU C 339      97.858  87.299 133.278  1.00 34.18           O
ATOM   1669  CB  LEU C 339      96.442  84.770 134.979  1.00 33.69           C
ATOM   1670  CG  LEU C 339      95.251  85.765 135.023  1.00 33.84           C
ATOM   1671  CD1 LEU C 339      93.985  85.106 135.617  1.00 31.31           C
ATOM   1672  CD2 LEU C 339      95.590  87.093 135.738  1.00 33.49           C
ATOM   1673  H   LEU C 339      98.495  83.343 134.658  1.00  0.00           H
ATOM   1674  HA  LEU C 339      96.951  85.028 132.924  1.00  0.00           H
ATOM   1675  HB2 LEU C 339      96.045  83.773 134.789  1.00  0.00           H
ATOM   1676  HB3 LEU C 339      96.917  84.712 135.958  1.00  0.00           H
ATOM   1677  HG  LEU C 339      95.002  86.007 133.988  1.00  0.00           H
ATOM   1678 HD11 LEU C 339      93.125  85.271 134.967  1.00  0.00           H
ATOM   1679 HD12 LEU C 339      94.088  84.028 135.738  1.00  0.00           H
ATOM   1680 HD13 LEU C 339      93.726  85.501 136.599  1.00  0.00           H
ATOM   1681 HD21 LEU C 339      94.835  87.388 136.466  1.00  0.00           H
ATOM   1682 HD22 LEU C 339      96.539  87.040 136.273  1.00  0.00           H
ATOM   1683 HD23 LEU C 339      95.667  87.909 135.019  1.00  0.00           H
ATOM   1684  N   ALA C 340      99.253  86.443 134.857  1.00 33.60           N
ATOM   1685  CA  ALA C 340     100.150  87.596 134.986  1.00 33.36           C
ATOM   1686  C   ALA C 340     100.957  87.879 133.705  1.00 33.23           C
ATOM   1687  O   ALA C 340     101.195  89.045 133.394  1.00 33.09           O
ATOM   1688  CB  ALA C 340     101.089  87.381 136.184  1.00 33.41           C
ATOM   1689  H   ALA C 340      99.476  85.642 135.433  1.00  0.00           H
ATOM   1690  HA  ALA C 340      99.537  88.474 135.196  1.00  0.00           H
ATOM   1691  HB1 ALA C 340     101.754  88.234 136.324  1.00  0.00           H
ATOM   1692  HB2 ALA C 340     100.520  87.255 137.105  1.00  0.00           H
ATOM   1693  HB3 ALA C 340     101.711  86.494 136.057  1.00  0.00           H
ATOM   1694  N   ALA C 341     101.318  86.809 132.975  1.00 33.42           N
```

```
ATOM   1695  CA  ALA C 341     101.999  86.858 131.684  1.00 33.00           C
ATOM   1696  C   ALA C 341     101.091  87.296 130.520  1.00 33.03           C
ATOM   1697  O   ALA C 341     101.591  87.934 129.598  1.00 33.45           O
ATOM   1698  CB  ALA C 341     102.626  85.485 131.402  1.00 32.81           C
ATOM   1699  H   ALA C 341     101.104  85.884 133.324  1.00  0.00           H
ATOM   1700  HA  ALA C 341     102.806  87.584 131.760  1.00  0.00           H
ATOM   1701  HB1 ALA C 341     103.213  85.500 130.486  1.00  0.00           H
ATOM   1702  HB2 ALA C 341     103.292  85.181 132.209  1.00  0.00           H
ATOM   1703  HB3 ALA C 341     101.868  84.711 131.291  1.00  0.00           H
ATOM   1704  N   SER C 342      99.792  86.952 130.566  1.00 32.96           N
ATOM   1705  CA  SER C 342      98.812  87.316 129.536  1.00 32.56           C
ATOM   1706  C   SER C 342      98.229  88.729 129.729  1.00 32.96           C
ATOM   1707  O   SER C 342      97.857  89.354 128.738  1.00 33.30           O
ATOM   1708  CB  SER C 342      97.715  86.231 129.447  1.00 32.35           C
ATOM   1709  OG  SER C 342      96.806  86.267 130.529  1.00 30.21           O
ATOM   1710  H   SER C 342      99.451  86.395 131.337  1.00  0.00           H
ATOM   1711  HA  SER C 342      99.324  87.314 128.571  1.00  0.00           H
ATOM   1712  HB2 SER C 342      98.153  85.235 129.386  1.00  0.00           H
ATOM   1713  HB3 SER C 342      97.141  86.367 128.530  1.00  0.00           H
ATOM   1714  HG  SER C 342      97.268  85.991 131.325  1.00  0.00           H
ATOM   1715  N   SER C 343      98.170  89.205 130.986  1.00 32.75           N
ATOM   1716  CA  SER C 343      97.669  90.529 131.366  1.00 32.98           C
ATOM   1717  C   SER C 343      98.763  91.617 131.405  1.00 33.17           C
ATOM   1718  O   SER C 343      98.417  92.789 131.553  1.00 32.85           O
ATOM   1719  CB  SER C 343      96.896  90.412 132.697  1.00 32.58           C
ATOM   1720  OG  SER C 343      97.738  90.101 133.789  1.00 32.92           O
ATOM   1721  H   SER C 343      98.473  88.613 131.747  1.00  0.00           H
ATOM   1722  HA  SER C 343      96.946  90.850 130.614  1.00  0.00           H
ATOM   1723  HB2 SER C 343      96.121  89.648 132.621  1.00  0.00           H
ATOM   1724  HB3 SER C 343      96.386  91.350 132.919  1.00  0.00           H
ATOM   1725  HG  SER C 343      97.916  89.155 133.766  1.00  0.00           H
ATOM   1726  N   GLY C 344     100.042  91.232 131.245  1.00 33.56           N
ATOM   1727  CA  GLY C 344     101.168  92.157 131.121  1.00 34.45           C
ATOM   1728  C   GLY C 344     101.663  92.726 132.460  1.00 35.18           C
ATOM   1729  O   GLY C 344     102.356  93.743 132.449  1.00 35.31           O
ATOM   1730  H   GLY C 344     100.248  90.249 131.141  1.00  0.00           H
ATOM   1731  HA2 GLY C 344     100.927  92.968 130.436  1.00  0.00           H
ATOM   1732  HA3 GLY C 344     101.990  91.615 130.670  1.00  0.00           H
ATOM   1733  N   LYS C 345     101.315  92.094 133.595  1.00 35.75           N
ATOM   1734  CA  LYS C 345     101.703  92.494 134.950  1.00 36.22           C
ATOM   1735  C   LYS C 345     103.168  92.115 135.241  1.00 36.63           C
ATOM   1736  O   LYS C 345     103.438  91.036 135.770  1.00 36.92           O
ATOM   1737  CB  LYS C 345     100.712  91.870 135.960  1.00 36.19           C
ATOM   1738  CG  LYS C 345      99.250  92.344 135.800  1.00 36.14           C
ATOM   1739  CD  LYS C 345      98.982  93.830 136.115  1.00 36.58           C
ATOM   1740  CE  LYS C 345      99.157  94.222 137.591  1.00 37.13           C
ATOM   1741  NZ  LYS C 345      98.170  93.551 138.455  1.00 37.20           N1+
ATOM   1742  H   LYS C 345     100.763  91.250 133.528  1.00  0.00           H
ATOM   1743  HA  LYS C 345     101.629  93.580 135.025  1.00  0.00           H
ATOM   1744  HB2 LYS C 345     100.734  90.784 135.858  1.00  0.00           H
ATOM   1745  HB3 LYS C 345     101.046  92.075 136.977  1.00  0.00           H
ATOM   1746  HG2 LYS C 345      98.932  92.165 134.775  1.00  0.00           H
ATOM   1747  HG3 LYS C 345      98.604  91.715 136.412  1.00  0.00           H
ATOM   1748  HD2 LYS C 345      99.622  94.461 135.499  1.00  0.00           H
ATOM   1749  HD3 LYS C 345      97.966  94.072 135.801  1.00  0.00           H
ATOM   1750  HE2 LYS C 345     100.163  93.991 137.942  1.00  0.00           H
ATOM   1751  HE3 LYS C 345      99.027  95.300 137.700  1.00  0.00           H
ATOM   1752  HZ1 LYS C 345      98.288  92.550 138.389  1.00  0.00           H
ATOM   1753  HZ2 LYS C 345      98.302  93.844 139.413  1.00  0.00           H
ATOM   1754  HZ3 LYS C 345      97.237  93.799 138.156  1.00  0.00           H
ATOM   1755  N   ILE C 346     104.080  93.024 134.857  1.00 36.53           N
ATOM   1756  CA  ILE C 346     105.538  92.886 134.914  1.00 36.02           C
ATOM   1757  C   ILE C 346     106.140  92.659 136.317  1.00 36.09           C
ATOM   1758  O   ILE C 346     107.079  91.871 136.430  1.00 36.19           O
ATOM   1759  CB  ILE C 346     106.256  94.111 134.267  1.00 35.75           C
ATOM   1760  CG1 ILE C 346     105.707  95.483 134.739  1.00 35.02           C
ATOM   1761  CG2 ILE C 346     106.248  94.004 132.733  1.00 35.66           C
ATOM   1762  CD1 ILE C 346     106.559  96.683 134.299  1.00 35.16           C
ATOM   1763  H   ILE C 346     103.751  93.862 134.399  1.00  0.00           H
ATOM   1764  HA  ILE C 346     105.780  91.997 134.328  1.00  0.00           H
ATOM   1765  HB  ILE C 346     107.308  94.081 134.558  1.00  0.00           H
```

```
ATOM   1766 HG12 ILE C 346     104.693  95.622 134.359  1.00  0.00           H
ATOM   1767 HG13 ILE C 346     105.625  95.509 135.824  1.00  0.00           H
ATOM   1768 HG21 ILE C 346     106.735  94.857 132.262  1.00  0.00           H
ATOM   1769 HG22 ILE C 346     106.797  93.121 132.411  1.00  0.00           H
ATOM   1770 HG23 ILE C 346     105.234  93.935 132.339  1.00  0.00           H
ATOM   1771 HD11 ILE C 346     106.457  97.511 135.001  1.00  0.00           H
ATOM   1772 HD12 ILE C 346     107.618  96.429 134.241  1.00  0.00           H
ATOM   1773 HD13 ILE C 346     106.250  97.045 133.317  1.00  0.00           H
ATOM   1774  N   GLY C 347     105.592  93.321 137.352  1.00 35.51           N
ATOM   1775  CA  GLY C 347     106.097  93.238 138.725  1.00 35.19           C
ATOM   1776  C   GLY C 347     105.624  91.957 139.429  1.00 34.92           C
ATOM   1777  O   GLY C 347     106.344  91.434 140.280  1.00 34.78           O
ATOM   1778  H   GLY C 347     104.818  93.948 137.181  1.00  0.00           H
ATOM   1779  HA2 GLY C 347     107.187  93.290 138.735  1.00  0.00           H
ATOM   1780  HA3 GLY C 347     105.734  94.100 139.285  1.00  0.00           H
ATOM   1781  N   VAL C 348     104.440  91.441 139.056  1.00 34.20           N
ATOM   1782  CA  VAL C 348     103.880  90.181 139.551  1.00 33.89           C
ATOM   1783  C   VAL C 348     104.641  88.971 138.974  1.00 34.13           C
ATOM   1784  O   VAL C 348     104.955  88.045 139.721  1.00 34.36           O
ATOM   1785  CB  VAL C 348     102.370  90.055 139.189  1.00 33.36           C
ATOM   1786  CG1 VAL C 348     101.734  88.697 139.558  1.00 33.30           C
ATOM   1787  CG2 VAL C 348     101.548  91.192 139.823  1.00 34.16           C
ATOM   1788  H   VAL C 348     103.904  91.910 138.341  1.00  0.00           H
ATOM   1789  HA  VAL C 348     103.981  90.161 140.638  1.00  0.00           H
ATOM   1790  HB  VAL C 348     102.270  90.162 138.108  1.00  0.00           H
ATOM   1791 HG11 VAL C 348     100.660  88.702 139.370  1.00  0.00           H
ATOM   1792 HG12 VAL C 348     102.146  87.879 138.969  1.00  0.00           H
ATOM   1793 HG13 VAL C 348     101.885  88.462 140.611  1.00  0.00           H
ATOM   1794 HG21 VAL C 348     100.493  91.111 139.559  1.00  0.00           H
ATOM   1795 HG22 VAL C 348     101.620  91.172 140.911  1.00  0.00           H
ATOM   1796 HG23 VAL C 348     101.886  92.171 139.485  1.00  0.00           H
ATOM   1797  N   LEU C 349     104.957  89.031 137.669  1.00 34.10           N
ATOM   1798  CA  LEU C 349     105.756  88.039 136.953  1.00 33.94           C
ATOM   1799  C   LEU C 349     107.220  87.998 137.435  1.00 33.11           C
ATOM   1800  O   LEU C 349     107.768  86.905 137.557  1.00 32.65           O
ATOM   1801  CB  LEU C 349     105.571  88.270 135.434  1.00 34.24           C
ATOM   1802  CG  LEU C 349     106.361  87.315 134.500  1.00 34.70           C
ATOM   1803  CD1 LEU C 349     105.563  86.951 133.229  1.00 35.42           C
ATOM   1804  CD2 LEU C 349     107.757  87.868 134.140  1.00 37.49           C
ATOM   1805  H   LEU C 349     104.652  89.827 137.124  1.00  0.00           H
ATOM   1806  HA  LEU C 349     105.327  87.061 137.181  1.00  0.00           H
ATOM   1807  HB2 LEU C 349     104.502  88.150 135.250  1.00  0.00           H
ATOM   1808  HB3 LEU C 349     105.779  89.309 135.175  1.00  0.00           H
ATOM   1809  HG  LEU C 349     106.498  86.375 135.036  1.00  0.00           H
ATOM   1810 HD11 LEU C 349     105.579  85.874 133.060  1.00  0.00           H
ATOM   1811 HD12 LEU C 349     104.517  87.246 133.293  1.00  0.00           H
ATOM   1812 HD13 LEU C 349     105.964  87.425 132.333  1.00  0.00           H
ATOM   1813 HD21 LEU C 349     108.031  87.681 133.102  1.00  0.00           H
ATOM   1814 HD22 LEU C 349     107.812  88.945 134.293  1.00  0.00           H
ATOM   1815 HD23 LEU C 349     108.530  87.412 134.757  1.00  0.00           H
ATOM   1816  N   ALA C 350     107.805  89.163 137.768  1.00 32.35           N
ATOM   1817  CA  ALA C 350     109.138  89.271 138.368  1.00 31.91           C
ATOM   1818  C   ALA C 350     109.234  88.660 139.779  1.00 31.54           C
ATOM   1819  O   ALA C 350     110.260  88.058 140.091  1.00 31.60           O
ATOM   1820  CB  ALA C 350     109.585  90.739 138.371  1.00 31.68           C
ATOM   1821  H   ALA C 350     107.306  90.032 137.630  1.00  0.00           H
ATOM   1822  HA  ALA C 350     109.830  88.716 137.734  1.00  0.00           H
ATOM   1823  HB1 ALA C 350     110.575  90.851 138.814  1.00  0.00           H
ATOM   1824  HB2 ALA C 350     109.638  91.132 137.356  1.00  0.00           H
ATOM   1825  HB3 ALA C 350     108.894  91.367 138.936  1.00  0.00           H
ATOM   1826  N   TYR C 351     108.164  88.792 140.584  1.00 30.99           N
ATOM   1827  CA  TYR C 351     108.050  88.209 141.924  1.00 30.59           C
ATOM   1828  C   TYR C 351     107.980  86.669 141.911  1.00 31.05           C
ATOM   1829  O   TYR C 351     108.686  86.032 142.693  1.00 31.61           O
ATOM   1830  CB  TYR C 351     106.854  88.853 142.665  1.00 29.56           C
ATOM   1831  CG  TYR C 351     106.474  88.208 143.990  1.00 28.61           C
ATOM   1832  CD1 TYR C 351     107.258  88.441 145.140  1.00 29.08           C
ATOM   1833  CD2 TYR C 351     105.354  87.353 144.070  1.00 26.21           C
ATOM   1834  CE1 TYR C 351     106.929  87.816 146.359  1.00 28.61           C
ATOM   1835  CE2 TYR C 351     105.027  86.727 145.288  1.00 23.37           C
ATOM   1836  CZ  TYR C 351     105.814  86.957 146.433  1.00 27.49           C
```

```
ATOM   1837  OH  TYR C 351     105.496  86.345 147.610  1.00 29.18           O
ATOM   1838  H   TYR C 351     107.362  89.313 140.256  1.00  0.00           H
ATOM   1839  HA  TYR C 351     108.955  88.484 142.470  1.00  0.00           H
ATOM   1840  HB2 TYR C 351     107.056  89.910 142.837  1.00  0.00           H
ATOM   1841  HB3 TYR C 351     105.973  88.829 142.023  1.00  0.00           H
ATOM   1842  HD1 TYR C 351     108.119  89.091 145.087  1.00  0.00           H
ATOM   1843  HD2 TYR C 351     104.749  87.167 143.195  1.00  0.00           H
ATOM   1844  HE1 TYR C 351     107.537  87.996 147.233  1.00  0.00           H
ATOM   1845  HE2 TYR C 351     104.170  86.072 145.344  1.00  0.00           H
ATOM   1846  HH  TYR C 351     106.087  86.577 148.330  1.00  0.00           H
ATOM   1847  N   ILE C 352     107.135  86.105 141.031  1.00 30.80           N
ATOM   1848  CA  ILE C 352     106.905  84.660 140.910  1.00 30.99           C
ATOM   1849  C   ILE C 352     108.143  83.891 140.401  1.00 31.33           C
ATOM   1850  O   ILE C 352     108.374  82.765 140.846  1.00 32.20           O
ATOM   1851  CB  ILE C 352     105.682  84.352 139.987  1.00 31.03           C
ATOM   1852  CG1 ILE C 352     104.367  84.883 140.608  1.00 29.77           C
ATOM   1853  CG2 ILE C 352     105.505  82.859 139.622  1.00 30.83           C
ATOM   1854  CD1 ILE C 352     103.227  85.069 139.594  1.00 28.73           C
ATOM   1855  H   ILE C 352     106.586  86.696 140.420  1.00  0.00           H
ATOM   1856  HA  ILE C 352     106.677  84.282 141.908  1.00  0.00           H
ATOM   1857  HB  ILE C 352     105.841  84.895 139.053  1.00  0.00           H
ATOM   1858 HG12 ILE C 352     104.038  84.206 141.395  1.00  0.00           H
ATOM   1859 HG13 ILE C 352     104.532  85.839 141.102  1.00  0.00           H
ATOM   1860 HG21 ILE C 352     104.590  82.693 139.061  1.00  0.00           H
ATOM   1861 HG22 ILE C 352     106.314  82.485 138.996  1.00  0.00           H
ATOM   1862 HG23 ILE C 352     105.455  82.238 140.517  1.00  0.00           H
ATOM   1863 HD11 ILE C 352     102.440  85.693 140.015  1.00  0.00           H
ATOM   1864 HD12 ILE C 352     103.575  85.552 138.681  1.00  0.00           H
ATOM   1865 HD13 ILE C 352     102.773  84.116 139.323  1.00  0.00           H
ATOM   1866  N   LEU C 353     108.912  84.517 139.495  1.00 30.90           N
ATOM   1867  CA  LEU C 353     110.085  83.921 138.858  1.00 30.99           C
ATOM   1868  C   LEU C 353     111.399  84.120 139.642  1.00 31.13           C
ATOM   1869  O   LEU C 353     112.407  83.558 139.216  1.00 31.12           O
ATOM   1870  CB  LEU C 353     110.197  84.436 137.404  1.00 30.86           C
ATOM   1871  CG  LEU C 353     109.013  84.047 136.486  1.00 29.88           C
ATOM   1872  CD1 LEU C 353     109.264  84.503 135.036  1.00 29.83           C
ATOM   1873  CD2 LEU C 353     108.665  82.552 136.546  1.00 30.44           C
ATOM   1874  H   LEU C 353     108.657  85.443 139.178  1.00  0.00           H
ATOM   1875  HA  LEU C 353     109.941  82.844 138.806  1.00  0.00           H
ATOM   1876  HB2 LEU C 353     110.321  85.521 137.406  1.00  0.00           H
ATOM   1877  HB3 LEU C 353     111.103  84.032 136.954  1.00  0.00           H
ATOM   1878  HG  LEU C 353     108.126  84.571 136.834  1.00  0.00           H
ATOM   1879 HD11 LEU C 353     108.378  84.981 134.619  1.00  0.00           H
ATOM   1880 HD12 LEU C 353     110.083  85.220 134.967  1.00  0.00           H
ATOM   1881 HD13 LEU C 353     109.514  83.667 134.382  1.00  0.00           H
ATOM   1882 HD21 LEU C 353     108.082  82.251 135.677  1.00  0.00           H
ATOM   1883 HD22 LEU C 353     109.561  81.931 136.593  1.00  0.00           H
ATOM   1884 HD23 LEU C 353     108.058  82.325 137.422  1.00  0.00           H
ATOM   1885  N   GLN C 354     111.388  84.873 140.760  1.00 31.81           N
ATOM   1886  CA  GLN C 354     112.600  85.205 141.524  1.00 32.41           C
ATOM   1887  C   GLN C 354     112.455  85.033 143.052  1.00 33.15           C
ATOM   1888  O   GLN C 354     113.385  85.412 143.765  1.00 33.51           O
ATOM   1889  CB  GLN C 354     113.089  86.625 141.149  1.00 32.04           C
ATOM   1890  CG  GLN C 354     113.447  86.797 139.652  1.00 30.84           C
ATOM   1891  CD  GLN C 354     113.957  88.186 139.253  1.00 31.08           C
ATOM   1892  NE2 GLN C 354     113.970  89.157 140.169  1.00 31.66           N
ATOM   1893  OE1 GLN C 354     114.334  88.384 138.101  1.00 32.26           O
ATOM   1894  H   GLN C 354     110.530  85.315 141.060  1.00  0.00           H
ATOM   1895  HA  GLN C 354     113.398  84.512 141.257  1.00  0.00           H
ATOM   1896  HB2 GLN C 354     112.328  87.350 141.439  1.00  0.00           H
ATOM   1897  HB3 GLN C 354     113.974  86.868 141.740  1.00  0.00           H
ATOM   1898  HG2 GLN C 354     114.200  86.061 139.367  1.00  0.00           H
ATOM   1899  HG3 GLN C 354     112.573  86.602 139.031  1.00  0.00           H
ATOM   1900 HE21 GLN C 354     114.297  90.080 139.919  1.00  0.00           H
ATOM   1901 HE22 GLN C 354     113.643  88.981 141.107  1.00  0.00           H
ATOM   1902  N   ARG C 355     111.334  84.466 143.543  1.00 34.49           N
ATOM   1903  CA  ARG C 355     111.086  84.225 144.976  1.00 35.92           C
ATOM   1904  C   ARG C 355     112.108  83.249 145.600  1.00 36.56           C
ATOM   1905  O   ARG C 355     112.457  82.254 144.967  1.00 37.15           O
ATOM   1906  CB  ARG C 355     109.631  83.741 145.196  1.00 36.12           C
ATOM   1907  CG  ARG C 355     109.251  82.440 144.461  1.00 37.70           C
```

```
ATOM   1908  CD  ARG C 355     107.875  81.875 144.832  1.00 41.27           C
ATOM   1909  NE  ARG C 355     106.777  82.726 144.356  1.00 44.15           N
ATOM   1910  CZ  ARG C 355     105.471  82.431 144.474  1.00 43.72           C
ATOM   1911  NH1 ARG C 355     105.065  81.283 145.038  1.00 44.30           N
ATOM   1912  NH2 ARG C 355     104.560  83.300 144.021  1.00 41.81           N1+
ATOM   1913  H   ARG C 355     110.602  84.181 142.909  1.00  0.00           H
ATOM   1914  HA  ARG C 355     111.189  85.189 145.477  1.00  0.00           H
ATOM   1915  HB2 ARG C 355     108.939  84.532 144.907  1.00  0.00           H
ATOM   1916  HB3 ARG C 355     109.477  83.599 146.268  1.00  0.00           H
ATOM   1917  HG2 ARG C 355     109.272  82.642 143.391  1.00  0.00           H
ATOM   1918  HG3 ARG C 355     109.980  81.654 144.636  1.00  0.00           H
ATOM   1919  HD2 ARG C 355     107.784  81.832 145.918  1.00  0.00           H
ATOM   1920  HD3 ARG C 355     107.777  80.853 144.466  1.00  0.00           H
ATOM   1921  HE  ARG C 355     107.045  83.613 143.954  1.00  0.00           H
ATOM   1922 HH11 ARG C 355     104.083  81.070 145.130  1.00  0.00           H
ATOM   1923 HH12 ARG C 355     105.749  80.623 145.382  1.00  0.00           H
ATOM   1924 HH21 ARG C 355     103.574  83.099 144.107  1.00  0.00           H
ATOM   1925 HH22 ARG C 355     104.850  84.176 143.610  1.00  0.00           H
ATOM   1926  N   GLU C 356     112.549  83.559 146.829  1.00 30.00           N
ATOM   1927  CA  GLU C 356     113.541  82.791 147.584  1.00 30.00           C
ATOM   1928  C   GLU C 356     113.012  82.516 149.000  1.00 30.00           C
ATOM   1929  O   GLU C 356     112.529  83.440 149.655  1.00 30.00           O
ATOM   1930  CB  GLU C 356     114.887  83.560 147.583  1.00 20.00           C
ATOM   1931  CG  GLU C 356     116.029  82.997 148.466  1.00  0.00           C
ATOM   1932  CD  GLU C 356     116.518  81.607 148.049  1.00  0.00           C
ATOM   1933  OE1 GLU C 356     115.829  80.623 148.398  1.00  0.00           O
ATOM   1934  OE2 GLU C 356     117.587  81.544 147.404  1.00  0.00           O1-
ATOM   1935  H   GLU C 356     112.199  84.384 147.294  1.00  0.00           H
ATOM   1936  HA  GLU C 356     113.714  81.834 147.096  1.00  0.00           H
ATOM   1937  HB2 GLU C 356     115.248  83.620 146.555  1.00  0.00           H
ATOM   1938  HB3 GLU C 356     114.705  84.593 147.884  1.00  0.00           H
ATOM   1939  HG2 GLU C 356     116.878  83.681 148.423  1.00  0.00           H
ATOM   1940  HG3 GLU C 356     115.731  82.966 149.514  1.00  0.00           H
ATOM   1941  N   ILE C 357     113.150  81.257 149.445  1.00 30.00           N
ATOM   1942  CA  ILE C 357     112.882  80.803 150.807  1.00 30.00           C
ATOM   1943  C   ILE C 357     113.900  79.695 151.149  1.00 30.00           C
ATOM   1944  O   ILE C 357     114.071  78.758 150.366  1.00 30.00           O
ATOM   1945  CB  ILE C 357     111.460  80.181 151.003  1.00 20.00           C
ATOM   1946  CG1 ILE C 357     110.285  80.999 150.415  1.00  0.00           C
ATOM   1947  CG2 ILE C 357     111.189  79.833 152.478  1.00  0.00           C
ATOM   1948  CD1 ILE C 357     109.941  82.283 151.184  1.00  0.00           C
ATOM   1949  H   ILE C 357     113.610  80.583 148.849  1.00  0.00           H
ATOM   1950  HA  ILE C 357     113.020  81.635 151.500  1.00  0.00           H
ATOM   1951  HB  ILE C 357     111.433  79.226 150.478  1.00  0.00           H
ATOM   1952 HG12 ILE C 357     110.465  81.235 149.366  1.00  0.00           H
ATOM   1953 HG13 ILE C 357     109.399  80.365 150.402  1.00  0.00           H
ATOM   1954 HG21 ILE C 357     110.174  79.479 152.628  1.00  0.00           H
ATOM   1955 HG22 ILE C 357     111.853  79.042 152.816  1.00  0.00           H
ATOM   1956 HG23 ILE C 357     111.333  80.694 153.132  1.00  0.00           H
ATOM   1957 HD11 ILE C 357     109.528  83.038 150.514  1.00  0.00           H
ATOM   1958 HD12 ILE C 357     109.199  82.086 151.958  1.00  0.00           H
ATOM   1959 HD13 ILE C 357     110.816  82.717 151.667  1.00  0.00           H
ATOM   1960  N   HIS C 358     114.507  79.804 152.342  1.00 30.00           N
ATOM   1961  CA  HIS C 358     115.315  78.764 152.979  1.00 30.00           C
ATOM   1962  C   HIS C 358     114.647  78.428 154.321  1.00 30.00           C
ATOM   1963  O   HIS C 358     114.921  79.086 155.325  1.00 30.00           O
ATOM   1964  CB  HIS C 358     116.772  79.252 153.159  1.00 20.00           C
ATOM   1965  CG  HIS C 358     117.686  79.040 151.973  1.00  0.00           C
ATOM   1966  CD2 HIS C 358     118.927  78.446 151.887  1.00  0.00           C
ATOM   1967  ND1 HIS C 358     117.382  79.435 150.681  1.00  0.00           N
ATOM   1968  CE1 HIS C 358     118.415  79.096 149.906  1.00  0.00           C
ATOM   1969  NE2 HIS C 358     119.388  78.484 150.569  1.00  0.00           N
ATOM   1970  H   HIS C 358     114.308  80.607 152.921  1.00  0.00           H
ATOM   1971  HA  HIS C 358     115.329  77.846 152.386  1.00  0.00           H
ATOM   1972  HB2 HIS C 358     116.791  80.310 153.424  1.00  0.00           H
ATOM   1973  HB3 HIS C 358     117.237  78.730 153.996  1.00  0.00           H
ATOM   1974  HD1 HIS C 358     116.540  79.897 150.357  1.00  0.00           H
ATOM   1975  HD2 HIS C 358     119.524  77.999 152.669  1.00  0.00           H
ATOM   1976  HE1 HIS C 358     118.450  79.290 148.843  1.00  0.00           H
ATOM   1977  N   GLU C 359     113.745  77.434 154.291  1.00 30.00           N
ATOM   1978  CA  GLU C 359     112.890  77.033 155.410  1.00 30.00           C
```

ATOM	1979	C	GLU	C	359	112.370	75.608	155.121	1.00	30.00	C
ATOM	1980	O	GLU	C	359	112.055	75.325	153.961	1.00	30.00	O
ATOM	1981	CB	GLU	C	359	111.718	78.041	155.506	1.00	20.00	C
ATOM	1982	CG	GLU	C	359	110.722	77.826	156.666	1.00	20.00	C
ATOM	1983	CD	GLU	C	359	109.545	78.808	156.657	1.00	20.00	C
ATOM	1984	OE1	GLU	C	359	109.357	79.504	155.634	1.00	20.00	O
ATOM	1985	OE2	GLU	C	359	108.839	78.837	157.688	1.00	20.00	O1-
ATOM	1986	H	GLU	C	359	113.571	76.946	153.424	1.00	0.00	H
ATOM	1987	HA	GLU	C	359	113.492	77.063	156.317	1.00	0.00	H
ATOM	1988	HB2	GLU	C	359	112.107	79.059	155.564	1.00	0.00	H
ATOM	1989	HB3	GLU	C	359	111.158	77.986	154.577	1.00	0.00	H
ATOM	1990	HG2	GLU	C	359	110.303	76.821	156.635	1.00	0.00	H
ATOM	1991	HG3	GLU	C	359	111.241	77.926	157.619	1.00	0.00	H
ATOM	1992	N	PRO	C	360	112.260	74.736	156.154	1.00	30.00	N
ATOM	1993	CA	PRO	C	360	111.634	73.409	155.995	1.00	30.00	C
ATOM	1994	C	PRO	C	360	110.152	73.500	155.583	1.00	30.00	C
ATOM	1995	O	PRO	C	360	109.408	74.304	156.144	1.00	30.00	O
ATOM	1996	CB	PRO	C	360	111.816	72.746	157.370	1.00	20.00	C
ATOM	1997	CG	PRO	C	360	111.914	73.904	158.349	1.00	0.00	C
ATOM	1998	CD	PRO	C	360	112.655	74.966	157.546	1.00	0.00	C
ATOM	1999	HA	PRO	C	360	112.181	72.837	155.244	1.00	0.00	H
ATOM	2000	HB2	PRO	C	360	111.019	72.049	157.632	1.00	0.00	H
ATOM	2001	HB3	PRO	C	360	112.751	72.184	157.375	1.00	0.00	H
ATOM	2002	HG2	PRO	C	360	110.912	74.262	158.587	1.00	0.00	H
ATOM	2003	HG3	PRO	C	360	112.410	73.644	159.284	1.00	0.00	H
ATOM	2004	HD2	PRO	C	360	112.411	75.958	157.923	1.00	0.00	H
ATOM	2005	HD3	PRO	C	360	113.734	74.827	157.627	1.00	0.00	H
ATOM	2006	N	GLU	C	361	109.787	72.687	154.578	1.00	30.00	N
ATOM	2007	CA	GLU	C	361	108.467	72.574	153.943	1.00	30.00	C
ATOM	2008	C	GLU	C	361	108.116	73.729	152.980	1.00	30.00	C
ATOM	2009	O	GLU	C	361	107.035	73.690	152.390	1.00	30.00	O
ATOM	2010	CB	GLU	C	361	107.336	72.316	154.973	1.00	20.00	C
ATOM	2011	CG	GLU	C	361	107.570	71.079	155.864	1.00	0.00	C
ATOM	2012	CD	GLU	C	361	106.386	70.813	156.793	1.00	0.00	C
ATOM	2013	OE1	GLU	C	361	105.316	70.442	156.261	1.00	0.00	O
ATOM	2014	OE2	GLU	C	361	106.570	70.981	158.018	1.00	0.00	O1-
ATOM	2015	H	GLU	C	361	110.490	72.072	154.195	1.00	0.00	H
ATOM	2016	HA	GLU	C	361	108.538	71.685	153.317	1.00	0.00	H
ATOM	2017	HB2	GLU	C	361	107.188	73.193	155.603	1.00	0.00	H
ATOM	2018	HB3	GLU	C	361	106.391	72.197	154.443	1.00	0.00	H
ATOM	2019	HG2	GLU	C	361	107.734	70.195	155.248	1.00	0.00	H
ATOM	2020	HG3	GLU	C	361	108.470	71.205	156.467	1.00	0.00	H
ATOM	2021	N	CYS	C	362	109.019	74.713	152.812	1.00	30.00	N
ATOM	2022	CA	CYS	C	362	108.780	75.935	152.038	1.00	30.00	C
ATOM	2023	C	CYS	C	362	109.927	76.251	151.048	1.00	30.00	C
ATOM	2024	O	CYS	C	362	109.762	77.137	150.210	1.00	30.00	O
ATOM	2025	CB	CYS	C	362	108.409	77.099	152.984	1.00	20.00	C
ATOM	2026	SG	CYS	C	362	107.607	78.484	152.124	1.00	20.00	S
ATOM	2027	H	CYS	C	362	109.879	74.686	153.343	1.00	0.00	H
ATOM	2028	HA	CYS	C	362	107.915	75.769	151.394	1.00	0.00	H
ATOM	2029	HB2	CYS	C	362	107.705	76.752	153.741	1.00	0.00	H
ATOM	2030	HB3	CYS	C	362	109.284	77.455	153.523	1.00	0.00	H
ATOM	2031	HG	CYS	C	362	108.676	78.829	151.400	1.00	0.00	H
ATOM	2032	N	ARG	C	363	111.029	75.474	151.078	1.00	30.00	N
ATOM	2033	CA	ARG	C	363	112.047	75.408	150.016	1.00	30.00	C
ATOM	2034	C	ARG	C	363	111.476	74.817	148.704	1.00	30.00	C
ATOM	2035	O	ARG	C	363	111.946	75.180	147.628	1.00	30.00	O
ATOM	2036	CB	ARG	C	363	113.285	74.639	150.555	1.00	20.00	C
ATOM	2037	CG	ARG	C	363	114.399	74.270	149.545	1.00	0.00	C
ATOM	2038	CD	ARG	C	363	115.094	75.461	148.861	1.00	0.00	C
ATOM	2039	NE	ARG	C	363	116.172	75.001	147.970	1.00	0.00	N
ATOM	2040	CZ	ARG	C	363	117.499	75.108	148.163	1.00	0.00	C
ATOM	2041	NH1	ARG	C	363	118.005	75.755	149.221	1.00	0.00	N
ATOM	2042	NH2	ARG	C	363	118.336	74.557	147.274	1.00	0.00	N1+
ATOM	2043	H	ARG	C	363	111.123	74.789	151.815	1.00	0.00	H
ATOM	2044	HA	ARG	C	363	112.360	76.432	149.803	1.00	0.00	H
ATOM	2045	HB2	ARG	C	363	113.724	75.204	151.378	1.00	0.00	H
ATOM	2046	HB3	ARG	C	363	112.935	73.704	150.995	1.00	0.00	H
ATOM	2047	HG2	ARG	C	363	115.149	73.629	150.010	1.00	0.00	H
ATOM	2048	HG3	ARG	C	363	113.949	73.649	148.770	1.00	0.00	H
ATOM	2049	HD2	ARG	C	363	114.376	75.928	148.190	1.00	0.00	H

```
ATOM   2050  HD3 ARG C 363     115.392  76.246 149.557  1.00  0.00           H
ATOM   2051  HE  ARG C 363     115.860  74.519 147.138  1.00  0.00           H
ATOM   2052 HH11 ARG C 363     119.006  75.834 149.342  1.00  0.00           H
ATOM   2053 HH12 ARG C 363     117.391  76.195 149.890  1.00  0.00           H
ATOM   2054 HH21 ARG C 363     119.346  74.632 147.383  1.00  0.00           H
ATOM   2055 HH22 ARG C 363     117.980  74.010 146.500  1.00  0.00           H
ATOM   2056  N   HIS C 364     110.439  73.968 148.811  1.00 30.00           N
ATOM   2057  CA  HIS C 364     109.631  73.475 147.692  1.00 30.00           C
ATOM   2058  C   HIS C 364     108.822  74.590 146.999  1.00 30.00           C
ATOM   2059  O   HIS C 364     108.677  74.561 145.777  1.00 30.00           O
ATOM   2060  CB  HIS C 364     108.695  72.374 148.227  1.00 20.00           C
ATOM   2061  CG  HIS C 364     107.869  71.647 147.192  1.00  0.00           C
ATOM   2062  CD2 HIS C 364     108.144  71.279 145.894  1.00  0.00           C
ATOM   2063  ND1 HIS C 364     106.592  71.177 147.447  1.00  0.00           N
ATOM   2064  CE1 HIS C 364     106.167  70.566 146.338  1.00  0.00           C
ATOM   2065  NE2 HIS C 364     107.058  70.588 145.352  1.00  0.00           N
ATOM   2066  H   HIS C 364     110.115  73.708 149.731  1.00  0.00           H
ATOM   2067  HA  HIS C 364     110.307  73.038 146.955  1.00  0.00           H
ATOM   2068  HB2 HIS C 364     109.298  71.623 148.732  1.00  0.00           H
ATOM   2069  HB3 HIS C 364     108.024  72.784 148.985  1.00  0.00           H
ATOM   2070  HD1 HIS C 364     106.077  71.265 148.311  1.00  0.00           H
ATOM   2071  HD2 HIS C 364     109.044  71.448 145.321  1.00  0.00           H
ATOM   2072  HE1 HIS C 364     105.200  70.094 146.252  1.00  0.00           H
ATOM   2073  N   LEU C 365     108.319  75.547 147.797  1.00 30.00           N
ATOM   2074  CA  LEU C 365     107.483  76.666 147.355  1.00 30.00           C
ATOM   2075  C   LEU C 365     108.301  77.846 146.789  1.00 30.00           C
ATOM   2076  O   LEU C 365     107.722  78.704 146.121  1.00 30.00           O
ATOM   2077  CB  LEU C 365     106.590  77.121 148.534  1.00 20.00           C
ATOM   2078  CG  LEU C 365     105.739  76.001 149.185  1.00  0.00           C
ATOM   2079  CD1 LEU C 365     104.962  76.536 150.408  1.00  0.00           C
ATOM   2080  CD2 LEU C 365     104.819  75.287 148.172  1.00  0.00           C
ATOM   2081  H   LEU C 365     108.504  75.507 148.789  1.00  0.00           H
ATOM   2082  HA  LEU C 365     106.841  76.317 146.546  1.00  0.00           H
ATOM   2083  HB2 LEU C 365     107.224  77.569 149.299  1.00  0.00           H
ATOM   2084  HB3 LEU C 365     105.927  77.918 148.194  1.00  0.00           H
ATOM   2085  HG  LEU C 365     106.419  75.243 149.577  1.00  0.00           H
ATOM   2086 HD11 LEU C 365     105.266  76.015 151.317  1.00  0.00           H
ATOM   2087 HD12 LEU C 365     105.142  77.598 150.571  1.00  0.00           H
ATOM   2088 HD13 LEU C 365     103.884  76.414 150.312  1.00  0.00           H
ATOM   2089 HD21 LEU C 365     103.838  75.052 148.585  1.00  0.00           H
ATOM   2090 HD22 LEU C 365     104.654  75.888 147.278  1.00  0.00           H
ATOM   2091 HD23 LEU C 365     105.262  74.343 147.853  1.00  0.00           H
ATOM   2092  N   SER C 366     109.622  77.857 147.042  1.00 30.00           N
ATOM   2093  CA  SER C 366     110.603  78.772 146.459  1.00 30.00           C
ATOM   2094  C   SER C 366     110.903  78.427 144.983  1.00 30.00           C
ATOM   2095  O   SER C 366     110.690  77.290 144.558  1.00 30.00           O
ATOM   2096  CB  SER C 366     111.854  78.747 147.366  1.00 20.00           C
ATOM   2097  OG  SER C 366     112.956  79.446 146.828  1.00 20.00           O
ATOM   2098  H   SER C 366     110.007  77.111 147.604  1.00  0.00           H
ATOM   2099  HA  SER C 366     110.190  79.783 146.484  1.00  0.00           H
ATOM   2100  HB2 SER C 366     111.604  79.205 148.317  1.00  0.00           H
ATOM   2101  HB3 SER C 366     112.164  77.731 147.596  1.00  0.00           H
ATOM   2102  HG  SER C 366     113.736  79.271 147.362  1.00  0.00           H
ATOM   2103  N   ARG C 367     111.397  79.432 144.243  1.00 30.00           N
ATOM   2104  CA  ARG C 367     111.723  79.367 142.821  1.00 30.00           C
ATOM   2105  C   ARG C 367     113.241  79.468 142.623  1.00 30.00           C
ATOM   2106  O   ARG C 367     113.861  78.495 142.197  1.00 30.00           O
ATOM   2107  CB  ARG C 367     110.955  80.485 142.084  1.00 20.00           C
ATOM   2108  CG  ARG C 367     111.265  80.616 140.582  1.00 20.00           C
ATOM   2109  CD  ARG C 367     110.576  79.588 139.679  1.00 20.00           C
ATOM   2110  NE  ARG C 367     109.134  79.845 139.560  1.00 20.00           N
ATOM   2111  CZ  ARG C 367     108.262  79.108 138.851  1.00 20.00           C
ATOM   2112  NH1 ARG C 367     108.659  78.048 138.134  1.00 20.00           N
ATOM   2113  NH2 ARG C 367     106.967  79.442 138.856  1.00 20.00           N1+
ATOM   2114  H   ARG C 367     111.605  80.313 144.696  1.00  0.00           H
ATOM   2115  HA  ARG C 367     111.398  78.416 142.409  1.00  0.00           H
ATOM   2116  HB2 ARG C 367     109.882  80.354 142.231  1.00  0.00           H
ATOM   2117  HB3 ARG C 367     111.202  81.441 142.547  1.00  0.00           H
ATOM   2118  HG2 ARG C 367     112.323  80.728 140.346  1.00  0.00           H
ATOM   2119  HG3 ARG C 367     110.821  81.570 140.327  1.00  0.00           H
ATOM   2120  HD2 ARG C 367     110.625  78.626 140.182  1.00  0.00           H
```

ATOM	2121	HD3	ARG	C	367	111.085	79.474	138.722	1.00	0.00	H
ATOM	2122	HE	ARG	C	367	108.787	80.649	140.068	1.00	0.00	H
ATOM	2123	HH11	ARG	C	367	107.983	77.519	137.592	1.00	0.00	H
ATOM	2124	HH12	ARG	C	367	109.630	77.774	138.127	1.00	0.00	H
ATOM	2125	HH21	ARG	C	367	106.302	78.894	138.328	1.00	0.00	H
ATOM	2126	HH22	ARG	C	367	106.645	80.235	139.391	1.00	0.00	H
ATOM	2127	N	LYS	C	368	113.794	80.655	142.921	1.00	30.00	N
ATOM	2128	CA	LYS	C	368	115.217	80.960	142.864	1.00	30.00	C
ATOM	2129	C	LYS	C	368	115.917	80.354	144.084	1.00	30.00	C
ATOM	2130	O	LYS	C	368	115.556	80.707	145.203	1.00	30.00	O
ATOM	2131	CB	LYS	C	368	115.395	82.492	142.816	1.00	20.00	C
ATOM	2132	CG	LYS	C	368	116.862	82.955	142.727	1.00	20.00	C
ATOM	2133	CD	LYS	C	368	116.994	84.479	142.603	1.00	20.00	C
ATOM	2134	CE	LYS	C	368	118.452	84.919	142.410	1.00	20.00	C
ATOM	2135	NZ	LYS	C	368	118.555	86.376	142.238	1.00	20.00	N1+
ATOM	2136	H	LYS	C	368	113.212	81.384	143.311	1.00	0.00	H
ATOM	2137	HA	LYS	C	368	115.629	80.541	141.946	1.00	0.00	H
ATOM	2138	HB2	LYS	C	368	114.851	82.874	141.954	1.00	0.00	H
ATOM	2139	HB3	LYS	C	368	114.931	82.947	143.693	1.00	0.00	H
ATOM	2140	HG2	LYS	C	368	117.416	82.629	143.608	1.00	0.00	H
ATOM	2141	HG3	LYS	C	368	117.339	82.474	141.873	1.00	0.00	H
ATOM	2142	HD2	LYS	C	368	116.386	84.840	141.773	1.00	0.00	H
ATOM	2143	HD3	LYS	C	368	116.588	84.947	143.501	1.00	0.00	H
ATOM	2144	HE2	LYS	C	368	119.055	84.621	143.267	1.00	0.00	H
ATOM	2145	HE3	LYS	C	368	118.879	84.436	141.530	1.00	0.00	H
ATOM	2146	HZ1	LYS	C	368	118.028	86.657	141.423	1.00	0.00	H
ATOM	2147	HZ2	LYS	C	368	118.185	86.840	143.055	1.00	0.00	H
ATOM	2148	HZ3	LYS	C	368	119.524	86.633	142.115	1.00	0.00	H
ATOM	2149	N	PHE	C	369	116.900	79.477	143.837	1.00	30.00	N
ATOM	2150	CA	PHE	C	369	117.755	78.885	144.861	1.00	30.00	C
ATOM	2151	C	PHE	C	369	119.180	79.374	144.589	1.00	30.00	C
ATOM	2152	O	PHE	C	369	119.754	79.020	143.559	1.00	30.00	O
ATOM	2153	CB	PHE	C	369	117.726	77.343	144.777	1.00	20.00	C
ATOM	2154	CG	PHE	C	369	116.378	76.648	144.789	1.00	20.00	C
ATOM	2155	CD1	PHE	C	369	115.235	77.196	145.411	1.00	20.00	C
ATOM	2156	CD2	PHE	C	369	116.313	75.330	144.303	1.00	20.00	C
ATOM	2157	CE1	PHE	C	369	114.046	76.480	145.424	1.00	20.00	C
ATOM	2158	CE2	PHE	C	369	115.130	74.621	144.371	1.00	20.00	C
ATOM	2159	CZ	PHE	C	369	113.989	75.197	144.905	1.00	20.00	C
ATOM	2160	H	PHE	C	369	117.099	79.206	142.882	1.00	0.00	H
ATOM	2161	HA	PHE	C	369	117.447	79.190	145.864	1.00	0.00	H
ATOM	2162	HB2	PHE	C	369	118.244	77.004	143.878	1.00	0.00	H
ATOM	2163	HB3	PHE	C	369	118.296	76.942	145.616	1.00	0.00	H
ATOM	2164	HD1	PHE	C	369	115.264	78.176	145.862	1.00	0.00	H
ATOM	2165	HD2	PHE	C	369	117.187	74.867	143.876	1.00	0.00	H
ATOM	2166	HE1	PHE	C	369	113.160	76.908	145.855	1.00	0.00	H
ATOM	2167	HE2	PHE	C	369	115.115	73.615	143.996	1.00	0.00	H
ATOM	2168	HZ	PHE	C	369	113.060	74.647	144.934	1.00	0.00	H
ATOM	2169	N	THR	C	370	119.735	80.155	145.525	1.00	30.00	N
ATOM	2170	CA	THR	C	370	121.110	80.657	145.482	1.00	30.00	C
ATOM	2171	C	THR	C	370	122.085	79.620	146.098	1.00	30.00	C
ATOM	2172	O	THR	C	370	122.752	79.898	147.096	1.00	30.00	O
ATOM	2173	CB	THR	C	370	121.201	82.026	146.217	1.00	20.00	C
ATOM	2174	CG2	THR	C	370	120.292	83.103	145.606	1.00	20.00	C
ATOM	2175	OG1	THR	C	370	120.904	81.927	147.599	1.00	20.00	O
ATOM	2176	H	THR	C	370	119.189	80.439	146.330	1.00	0.00	H
ATOM	2177	HA	THR	C	370	121.411	80.816	144.444	1.00	0.00	H
ATOM	2178	HB	THR	C	370	122.227	82.389	146.138	1.00	0.00	H
ATOM	2179	HG1	THR	C	370	121.534	81.319	147.997	1.00	0.00	H
ATOM	2180	HG21	THR	C	370	120.413	84.056	146.121	1.00	0.00	H
ATOM	2181	HG22	THR	C	370	120.539	83.265	144.558	1.00	0.00	H
ATOM	2182	HG23	THR	C	370	119.238	82.831	145.661	1.00	0.00	H
ATOM	2183	N	GLU	C	371	122.139	78.421	145.485	1.00	30.00	N
ATOM	2184	CA	GLU	C	371	122.908	77.265	145.959	1.00	30.00	C
ATOM	2185	C	GLU	C	371	124.425	77.521	146.026	1.00	30.00	C
ATOM	2186	O	GLU	C	371	125.060	77.133	147.005	1.00	30.00	O
ATOM	2187	CB	GLU	C	371	122.601	76.041	145.066	1.00	20.00	C
ATOM	2188	CG	GLU	C	371	123.146	74.703	145.616	1.00	0.00	C
ATOM	2189	CD	GLU	C	371	122.399	74.174	146.849	1.00	0.00	C
ATOM	2190	OE1	GLU	C	371	121.236	74.588	147.064	1.00	0.00	O
ATOM	2191	OE2	GLU	C	371	123.001	73.331	147.546	1.00	0.00	O1-

```
ATOM   2192  H   GLU C 371     121.565  78.265 144.668  1.00  0.00           H
ATOM   2193  HA  GLU C 371     122.559  77.057 146.972  1.00  0.00           H
ATOM   2194  HB2 GLU C 371     121.526  75.961 144.899  1.00  0.00           H
ATOM   2195  HB3 GLU C 371     123.024  76.204 144.073  1.00  0.00           H
ATOM   2196  HG2 GLU C 371     123.105  73.949 144.835  1.00  0.00           H
ATOM   2197  HG3 GLU C 371     124.206  74.785 145.859  1.00  0.00           H
ATOM   2198  N   TRP C 372     124.958  78.193 144.993  1.00 30.00           N
ATOM   2199  CA  TRP C 372     126.353  78.604 144.890  1.00 30.00           C
ATOM   2200  C   TRP C 372     126.469  80.127 145.054  1.00 30.00           C
ATOM   2201  O   TRP C 372     126.973  80.820 144.169  1.00 30.00           O
ATOM   2202  CB  TRP C 372     126.967  78.044 143.590  1.00 20.00           C
ATOM   2203  CG  TRP C 372     127.479  76.640 143.692  1.00  0.00           C
ATOM   2204  CD1 TRP C 372     126.744  75.510 143.583  1.00  0.00           C
ATOM   2205  CD2 TRP C 372     128.838  76.206 144.008  1.00  0.00           C
ATOM   2206  CE2 TRP C 372     128.859  74.780 144.058  1.00  0.00           C
ATOM   2207  CE3 TRP C 372     130.060  76.876 144.259  1.00  0.00           C
ATOM   2208  NE1 TRP C 372     127.555  74.412 143.789  1.00  0.00           N
ATOM   2209  CZ2 TRP C 372     130.032  74.056 144.340  1.00  0.00           C
ATOM   2210  CZ3 TRP C 372     131.241  76.162 144.548  1.00  0.00           C
ATOM   2211  CH2 TRP C 372     131.228  74.754 144.587  1.00  0.00           C
ATOM   2212  H   TRP C 372     124.357  78.491 144.237  1.00  0.00           H
ATOM   2213  HA  TRP C 372     126.944  78.205 145.717  1.00  0.00           H
ATOM   2214  HB2 TRP C 372     126.243  78.098 142.776  1.00  0.00           H
ATOM   2215  HB3 TRP C 372     127.825  78.640 143.281  1.00  0.00           H
ATOM   2216  HD1 TRP C 372     125.684  75.489 143.376  1.00  0.00           H
ATOM   2217  HE1 TRP C 372     127.206  73.465 143.752  1.00  0.00           H
ATOM   2218  HE3 TRP C 372     130.089  77.954 144.222  1.00  0.00           H
ATOM   2219  HZ2 TRP C 372     130.013  72.977 144.371  1.00  0.00           H
ATOM   2220  HZ3 TRP C 372     132.159  76.699 144.737  1.00  0.00           H
ATOM   2221  HH2 TRP C 372     132.136  74.212 144.808  1.00  0.00           H
ATOM   2222  N   ALA C 373     126.058  80.599 146.243  1.00 30.00           N
ATOM   2223  CA  ALA C 373     126.381  81.917 146.788  1.00 30.00           C
ATOM   2224  C   ALA C 373     127.774  81.862 147.448  1.00 30.00           C
ATOM   2225  O   ALA C 373     127.886  81.930 148.673  1.00 30.00           O
ATOM   2226  CB  ALA C 373     125.271  82.326 147.773  1.00 30.00           C
ATOM   2227  H   ALA C 373     125.627  79.958 146.894  1.00  0.00           H
ATOM   2228  HA  ALA C 373     126.414  82.656 145.985  1.00  0.00           H
ATOM   2229  HB1 ALA C 373     125.493  83.284 148.244  1.00  0.00           H
ATOM   2230  HB2 ALA C 373     124.316  82.430 147.259  1.00  0.00           H
ATOM   2231  HB3 ALA C 373     125.141  81.587 148.565  1.00  0.00           H
ATOM   2232  N   TYR C 374     128.800  81.674 146.604  1.00 30.00           N
ATOM   2233  CA  TYR C 374     130.190  81.410 146.972  1.00 30.00           C
ATOM   2234  C   TYR C 374     130.942  82.745 147.132  1.00 30.00           C
ATOM   2235  O   TYR C 374     130.595  83.524 148.020  1.00 30.00           O
ATOM   2236  CB  TYR C 374     130.753  80.433 145.908  1.00 20.00           C
ATOM   2237  CG  TYR C 374     132.181  79.950 146.104  1.00  0.00           C
ATOM   2238  CD1 TYR C 374     132.511  79.169 147.231  1.00  0.00           C
ATOM   2239  CD2 TYR C 374     133.180  80.268 145.158  1.00  0.00           C
ATOM   2240  CE1 TYR C 374     133.832  78.720 147.419  1.00  0.00           C
ATOM   2241  CE2 TYR C 374     134.502  79.824 145.350  1.00  0.00           C
ATOM   2242  CZ  TYR C 374     134.829  79.051 146.481  1.00  0.00           C
ATOM   2243  OH  TYR C 374     136.111  78.621 146.663  1.00  0.00           O
ATOM   2244  H   TYR C 374     128.603  81.646 145.613  1.00  0.00           H
ATOM   2245  HA  TYR C 374     130.215  80.909 147.942  1.00  0.00           H
ATOM   2246  HB2 TYR C 374     130.123  79.543 145.893  1.00  0.00           H
ATOM   2247  HB3 TYR C 374     130.658  80.872 144.913  1.00  0.00           H
ATOM   2248  HD1 TYR C 374     131.751  78.912 147.955  1.00  0.00           H
ATOM   2249  HD2 TYR C 374     132.939  80.855 144.285  1.00  0.00           H
ATOM   2250  HE1 TYR C 374     134.075  78.124 148.286  1.00  0.00           H
ATOM   2251  HE2 TYR C 374     135.264  80.073 144.625  1.00  0.00           H
ATOM   2252  HH  TYR C 374     136.223  78.093 147.456  1.00  0.00           H
ATOM   2253  N   GLY C 375     131.910  83.022 146.241  1.00 30.00           N
ATOM   2254  CA  GLY C 375     132.497  84.346 146.042  1.00 30.00           C
ATOM   2255  C   GLY C 375     131.487  85.268 145.325  1.00 30.00           C
ATOM   2256  O   GLY C 375     130.327  84.888 145.152  1.00 30.00           O
ATOM   2257  H   GLY C 375     132.152  82.318 145.559  1.00  0.00           H
ATOM   2258  HA2 GLY C 375     133.400  84.239 145.439  1.00  0.00           H
ATOM   2259  HA3 GLY C 375     132.785  84.779 147.001  1.00  0.00           H
ATOM   2260  N   PRO C 376     131.900  86.488 144.914  1.00 30.00           N
ATOM   2261  CA  PRO C 376     131.000  87.520 144.355  1.00 30.00           C
ATOM   2262  C   PRO C 376     130.030  87.114 143.223  1.00 30.00           C
```

```
ATOM   2263  O   PRO C 376     128.941  87.683 143.166  1.00 30.00           O
ATOM   2264  CB  PRO C 376     131.960  88.637 143.935  1.00 20.00           C
ATOM   2265  CG  PRO C 376     133.121  88.518 144.906  1.00 20.00           C
ATOM   2266  CD  PRO C 376     133.256  87.013 145.094  1.00 20.00           C
ATOM   2267  HA  PRO C 376     130.398  87.878 145.192  1.00  0.00           H
ATOM   2268  HB2 PRO C 376     132.316  88.461 142.920  1.00  0.00           H
ATOM   2269  HB3 PRO C 376     131.502  89.626 143.973  1.00  0.00           H
ATOM   2270  HG2 PRO C 376     134.036  88.995 144.553  1.00  0.00           H
ATOM   2271  HG3 PRO C 376     132.844  88.981 145.854  1.00  0.00           H
ATOM   2272  HD2 PRO C 376     133.901  86.586 144.325  1.00  0.00           H
ATOM   2273  HD3 PRO C 376     133.686  86.784 146.070  1.00  0.00           H
ATOM   2274  N   VAL C 377     130.390  86.112 142.399  1.00 30.00           N
ATOM   2275  CA  VAL C 377     129.479  85.477 141.444  1.00 30.00           C
ATOM   2276  C   VAL C 377     128.501  84.532 142.175  1.00 30.00           C
ATOM   2277  O   VAL C 377     128.924  83.497 142.694  1.00 30.00           O
ATOM   2278  CB  VAL C 377     130.256  84.695 140.341  1.00 20.00           C
ATOM   2279  CG1 VAL C 377     129.390  83.773 139.448  1.00 20.00           C
ATOM   2280  CG2 VAL C 377     131.054  85.666 139.452  1.00 20.00           C
ATOM   2281  H   VAL C 377     131.293  85.676 142.508  1.00  0.00           H
ATOM   2282  HA  VAL C 377     128.908  86.270 140.965  1.00  0.00           H
ATOM   2283  HB  VAL C 377     130.986  84.052 140.836  1.00  0.00           H
ATOM   2284 HG11 VAL C 377     129.980  83.347 138.636  1.00  0.00           H
ATOM   2285 HG12 VAL C 377     128.972  82.931 139.999  1.00  0.00           H
ATOM   2286 HG13 VAL C 377     128.561  84.315 138.994  1.00  0.00           H
ATOM   2287 HG21 VAL C 377     131.626  85.131 138.693  1.00  0.00           H
ATOM   2288 HG22 VAL C 377     130.393  86.364 138.936  1.00  0.00           H
ATOM   2289 HG23 VAL C 377     131.763  86.254 140.035  1.00  0.00           H
ATOM   2290  N   HIS C 378     127.216  84.923 142.206  1.00 30.00           N
ATOM   2291  CA  HIS C 378     126.140  84.200 142.887  1.00 30.00           C
ATOM   2292  C   HIS C 378     125.356  83.341 141.884  1.00 30.00           C
ATOM   2293  O   HIS C 378     124.303  83.761 141.403  1.00 30.00           O
ATOM   2294  CB  HIS C 378     125.227  85.201 143.630  1.00 20.00           C
ATOM   2295  CG  HIS C 378     125.791  85.717 144.930  1.00  0.00           C
ATOM   2296  CD2 HIS C 378     125.341  85.590 146.226  1.00  0.00           C
ATOM   2297  ND1 HIS C 378     126.960  86.452 145.017  1.00  0.00           N
ATOM   2298  CE1 HIS C 378     127.169  86.717 146.308  1.00  0.00           C
ATOM   2299  NE2 HIS C 378     126.225  86.225 147.101  1.00  0.00           N
ATOM   2300  H   HIS C 378     126.953  85.788 141.756  1.00  0.00           H
ATOM   2301  HA  HIS C 378     126.550  83.518 143.635  1.00  0.00           H
ATOM   2302  HB2 HIS C 378     124.989  86.054 142.992  1.00  0.00           H
ATOM   2303  HB3 HIS C 378     124.272  84.730 143.872  1.00  0.00           H
ATOM   2304  HD1 HIS C 378     127.557  86.737 144.249  1.00  0.00           H
ATOM   2305  HD2 HIS C 378     124.460  85.085 146.596  1.00  0.00           H
ATOM   2306  HE1 HIS C 378     128.020  87.275 146.670  1.00  0.00           H
ATOM   2307  N   SER C 379     125.887  82.142 141.591  1.00 30.00           N
ATOM   2308  CA  SER C 379     125.248  81.145 140.732  1.00 30.00           C
ATOM   2309  C   SER C 379     123.931  80.628 141.344  1.00 30.00           C
ATOM   2310  O   SER C 379     123.931  80.105 142.460  1.00 30.00           O
ATOM   2311  CB  SER C 379     126.267  80.042 140.385  1.00 20.00           C
ATOM   2312  OG  SER C 379     125.688  78.980 139.651  1.00 20.00           O
ATOM   2313  H   SER C 379     126.746  81.858 142.042  1.00  0.00           H
ATOM   2314  HA  SER C 379     124.998  81.651 139.800  1.00  0.00           H
ATOM   2315  HB2 SER C 379     127.078  80.464 139.789  1.00  0.00           H
ATOM   2316  HB3 SER C 379     126.726  79.645 141.286  1.00  0.00           H
ATOM   2317  HG  SER C 379     125.375  79.325 138.808  1.00  0.00           H
ATOM   2318  N   SER C 380     122.840  80.844 140.593  1.00 30.00           N
ATOM   2319  CA  SER C 380     121.457  80.632 141.016  1.00 30.00           C
ATOM   2320  C   SER C 380     120.754  79.657 140.063  1.00 30.00           C
ATOM   2321  O   SER C 380     121.147  79.553 138.900  1.00 30.00           O
ATOM   2322  CB  SER C 380     120.734  81.994 140.998  1.00 20.00           C
ATOM   2323  OG  SER C 380     121.227  82.830 142.024  1.00 20.00           O
ATOM   2324  H   SER C 380     122.952  81.239 139.670  1.00  0.00           H
ATOM   2325  HA  SER C 380     121.425  80.213 142.022  1.00  0.00           H
ATOM   2326  HB2 SER C 380     119.664  81.862 141.162  1.00  0.00           H
ATOM   2327  HB3 SER C 380     120.849  82.493 140.034  1.00  0.00           H
ATOM   2328  HG  SER C 380     122.146  83.038 141.831  1.00  0.00           H
ATOM   2329  N   LEU C 381     119.717  78.973 140.574  1.00 30.00           N
ATOM   2330  CA  LEU C 381     118.928  78.002 139.818  1.00 30.00           C
ATOM   2331  C   LEU C 381     117.425  78.249 140.015  1.00 30.00           C
ATOM   2332  O   LEU C 381     117.007  78.553 141.128  1.00 30.00           O
ATOM   2333  CB  LEU C 381     119.304  76.559 140.222  1.00 20.00           C
```

ATOM	2334	CG	LEU	C	381	120.766	76.144	139.935	1.00	20.00	C
ATOM	2335	CD1	LEU	C	381	121.723	76.493	141.098	1.00	20.00	C
ATOM	2336	CD2	LEU	C	381	120.850	74.655	139.548	1.00	20.00	C
ATOM	2337	H	LEU	C	381	119.459	79.098	141.544	1.00	0.00	H
ATOM	2338	HA	LEU	C	381	119.129	78.140	138.761	1.00	0.00	H
ATOM	2339	HB2	LEU	C	381	119.037	76.360	141.260	1.00	0.00	H
ATOM	2340	HB3	LEU	C	381	118.672	75.905	139.626	1.00	0.00	H
ATOM	2341	HG	LEU	C	381	121.106	76.702	139.064	1.00	0.00	H
ATOM	2342	HD11	LEU	C	381	122.204	75.612	141.523	1.00	0.00	H
ATOM	2343	HD12	LEU	C	381	122.515	77.162	140.761	1.00	0.00	H
ATOM	2344	HD13	LEU	C	381	121.208	76.993	141.919	1.00	0.00	H
ATOM	2345	HD21	LEU	C	381	121.813	74.212	139.802	1.00	0.00	H
ATOM	2346	HD22	LEU	C	381	120.078	74.062	140.038	1.00	0.00	H
ATOM	2347	HD23	LEU	C	381	120.711	74.532	138.474	1.00	0.00	H
ATOM	2348	N	TYR	C	382	116.648	78.097	138.928	1.00	30.00	N
ATOM	2349	CA	TYR	C	382	115.211	78.400	138.863	1.00	30.00	C
ATOM	2350	C	TYR	C	382	114.420	77.148	138.448	1.00	30.00	C
ATOM	2351	O	TYR	C	382	114.881	76.428	137.561	1.00	30.00	O
ATOM	2352	CB	TYR	C	382	114.990	79.534	137.837	1.00	20.00	C
ATOM	2353	CG	TYR	C	382	115.815	80.784	138.094	1.00	20.00	C
ATOM	2354	CD1	TYR	C	382	115.355	81.768	138.993	1.00	20.00	C
ATOM	2355	CD2	TYR	C	382	117.059	80.955	137.450	1.00	20.00	C
ATOM	2356	CE1	TYR	C	382	116.130	82.918	139.239	1.00	20.00	C
ATOM	2357	CE2	TYR	C	382	117.837	82.098	137.707	1.00	20.00	C
ATOM	2358	CZ	TYR	C	382	117.373	83.084	138.599	1.00	20.00	C
ATOM	2359	OH	TYR	C	382	118.125	84.199	138.832	1.00	20.00	O
ATOM	2360	H	TYR	C	382	117.074	77.807	138.058	1.00	0.00	H
ATOM	2361	HA	TYR	C	382	114.847	78.739	139.832	1.00	0.00	H
ATOM	2362	HB2	TYR	C	382	115.225	79.178	136.832	1.00	0.00	H
ATOM	2363	HB3	TYR	C	382	113.936	79.813	137.815	1.00	0.00	H
ATOM	2364	HD1	TYR	C	382	114.406	81.646	139.495	1.00	0.00	H
ATOM	2365	HD2	TYR	C	382	117.428	80.203	136.767	1.00	0.00	H
ATOM	2366	HE1	TYR	C	382	115.761	83.677	139.911	1.00	0.00	H
ATOM	2367	HE2	TYR	C	382	118.789	82.216	137.217	1.00	0.00	H
ATOM	2368	HH	TYR	C	382	118.908	84.237	138.276	1.00	0.00	H
ATOM	2369	N	ASP	C	383	113.244	76.923	139.074	1.00	30.00	N
ATOM	2370	CA	ASP	C	383	112.400	75.719	138.907	1.00	30.00	C
ATOM	2371	C	ASP	C	383	111.903	75.428	137.473	1.00	30.00	C
ATOM	2372	O	ASP	C	383	111.523	74.289	137.207	1.00	30.00	O
ATOM	2373	CB	ASP	C	383	111.163	75.737	139.839	1.00	20.00	C
ATOM	2374	CG	ASP	C	383	111.411	75.856	141.343	1.00	0.00	C
ATOM	2375	OD1	ASP	C	383	112.510	75.805	141.884	1.00	0.00	O
ATOM	2376	OD2	ASP	C	383	110.243	76.025	142.025	1.00	0.00	O
ATOM	2377	H	ASP	C	383	112.961	77.553	139.811	1.00	0.00	H
ATOM	2378	HA	ASP	C	383	113.019	74.868	139.193	1.00	0.00	H
ATOM	2379	HB2	ASP	C	383	110.486	76.533	139.539	1.00	0.00	H
ATOM	2380	HB3	ASP	C	383	110.606	74.816	139.705	1.00	0.00	H
ATOM	2381	HD2	ASP	C	383	110.383	76.132	142.973	1.00	0.00	H
ATOM	2382	N	LEU	C	384	111.905	76.439	136.586	1.00	30.00	N
ATOM	2383	CA	LEU	C	384	111.706	76.326	135.136	1.00	30.00	C
ATOM	2384	C	LEU	C	384	110.413	75.588	134.712	1.00	30.00	C
ATOM	2385	O	LEU	C	384	110.469	74.585	134.003	1.00	30.00	O
ATOM	2386	CB	LEU	C	384	113.009	75.768	134.497	1.00	20.00	C
ATOM	2387	CG	LEU	C	384	113.089	75.796	132.951	1.00	20.00	C
ATOM	2388	CD1	LEU	C	384	112.778	77.190	132.380	1.00	20.00	C
ATOM	2389	CD2	LEU	C	384	114.439	75.246	132.445	1.00	20.00	C
ATOM	2390	H	LEU	C	384	112.225	77.342	136.903	1.00	0.00	H
ATOM	2391	HA	LEU	C	384	111.581	77.355	134.800	1.00	0.00	H
ATOM	2392	HB2	LEU	C	384	113.855	76.337	134.886	1.00	0.00	H
ATOM	2393	HB3	LEU	C	384	113.161	74.746	134.842	1.00	0.00	H
ATOM	2394	HG	LEU	C	384	112.340	75.118	132.547	1.00	0.00	H
ATOM	2395	HD11	LEU	C	384	113.292	77.355	131.438	1.00	0.00	H
ATOM	2396	HD12	LEU	C	384	111.711	77.297	132.179	1.00	0.00	H
ATOM	2397	HD13	LEU	C	384	113.074	77.989	133.061	1.00	0.00	H
ATOM	2398	HD21	LEU	C	384	114.288	74.389	131.788	1.00	0.00	H
ATOM	2399	HD22	LEU	C	384	115.009	75.985	131.881	1.00	0.00	H
ATOM	2400	HD23	LEU	C	384	115.077	74.908	133.263	1.00	0.00	H
ATOM	2401	N	SER	C	385	109.260	76.132	135.131	1.00	30.00	N
ATOM	2402	CA	SER	C	385	107.939	75.784	134.591	1.00	30.00	C
ATOM	2403	C	SER	C	385	107.520	76.760	133.466	1.00	30.00	C
ATOM	2404	O	SER	C	385	106.336	76.839	133.141	1.00	30.00	O

ATOM	2405	CB	SER	C	385	106.926	75.709	135.757	1.00	20.00	C
ATOM	2406	OG	SER	C	385	106.590	76.986	136.270	1.00	0.00	O
ATOM	2407	H	SER	C	385	109.284	76.942	135.732	1.00	0.00	H
ATOM	2408	HA	SER	C	385	107.976	74.791	134.141	1.00	0.00	H
ATOM	2409	HB2	SER	C	385	106.007	75.229	135.416	1.00	0.00	H
ATOM	2410	HB3	SER	C	385	107.315	75.086	136.564	1.00	0.00	H
ATOM	2411	HG	SER	C	385	106.019	77.418	135.626	1.00	0.00	H
ATOM	2412	N	CYS	C	386	108.496	77.518	132.934	1.00	30.00	N
ATOM	2413	CA	CYS	C	386	108.294	78.670	132.060	1.00	30.00	C
ATOM	2414	C	CYS	C	386	108.363	78.292	130.578	1.00	30.00	C
ATOM	2415	O	CYS	C	386	107.613	78.862	129.788	1.00	30.00	O
ATOM	2416	CB	CYS	C	386	109.347	79.763	132.324	1.00	20.00	C
ATOM	2417	SG	CYS	C	386	109.314	80.269	134.060	1.00	0.00	S
ATOM	2418	H	CYS	C	386	109.441	77.378	133.258	1.00	0.00	H
ATOM	2419	HA	CYS	C	386	107.308	79.091	132.257	1.00	0.00	H
ATOM	2420	HB2	CYS	C	386	110.352	79.413	132.085	1.00	0.00	H
ATOM	2421	HB3	CYS	C	386	109.152	80.638	131.705	1.00	0.00	H
ATOM	2422	HG	CYS	C	386	110.243	81.224	133.946	1.00	0.00	H
ATOM	2423	N	ILE	C	387	109.275	77.372	130.225	1.00	30.00	N
ATOM	2424	CA	ILE	C	387	109.504	76.917	128.854	1.00	30.00	C
ATOM	2425	C	ILE	C	387	108.451	75.901	128.382	1.00	30.00	C
ATOM	2426	O	ILE	C	387	107.912	75.136	129.184	1.00	30.00	O
ATOM	2427	CB	ILE	C	387	110.928	76.321	128.659	1.00	20.00	C
ATOM	2428	CG1	ILE	C	387	111.339	75.240	129.694	1.00	0.00	C
ATOM	2429	CG2	ILE	C	387	111.959	77.459	128.612	1.00	0.00	C
ATOM	2430	CD1	ILE	C	387	111.351	73.822	129.124	1.00	0.00	C
ATOM	2431	H	ILE	C	387	109.843	76.936	130.936	1.00	0.00	H
ATOM	2432	HA	ILE	C	387	109.415	77.786	128.200	1.00	0.00	H
ATOM	2433	HB	ILE	C	387	110.967	75.859	127.671	1.00	0.00	H
ATOM	2434	HG12	ILE	C	387	112.344	75.445	130.061	1.00	0.00	H
ATOM	2435	HG13	ILE	C	387	110.702	75.259	130.579	1.00	0.00	H
ATOM	2436	HG21	ILE	C	387	112.961	77.077	128.420	1.00	0.00	H
ATOM	2437	HG22	ILE	C	387	111.724	78.169	127.818	1.00	0.00	H
ATOM	2438	HG23	ILE	C	387	111.978	78.012	129.550	1.00	0.00	H
ATOM	2439	HD11	ILE	C	387	111.571	73.088	129.901	1.00	0.00	H
ATOM	2440	HD12	ILE	C	387	110.383	73.576	128.697	1.00	0.00	H
ATOM	2441	HD13	ILE	C	387	112.104	73.717	128.342	1.00	0.00	H
ATOM	2442	N	ASP	C	388	108.175	75.958	127.070	1.00	30.00	N
ATOM	2443	CA	ASP	C	388	107.108	75.237	126.365	1.00	30.00	C
ATOM	2444	C	ASP	C	388	107.295	73.707	126.323	1.00	30.00	C
ATOM	2445	O	ASP	C	388	106.324	72.998	126.067	1.00	30.00	O
ATOM	2446	CB	ASP	C	388	106.854	75.782	124.933	1.00	20.00	C
ATOM	2447	CG	ASP	C	388	106.831	77.311	124.755	1.00	0.00	C
ATOM	2448	OD1	ASP	C	388	106.722	78.049	125.760	1.00	0.00	O
ATOM	2449	OD2	ASP	C	388	106.876	77.722	123.576	1.00	0.00	O1-
ATOM	2450	H	ASP	C	388	108.652	76.649	126.509	1.00	0.00	H
ATOM	2451	HA	ASP	C	388	106.194	75.399	126.935	1.00	0.00	H
ATOM	2452	HB2	ASP	C	388	107.634	75.393	124.275	1.00	0.00	H
ATOM	2453	HB3	ASP	C	388	105.898	75.398	124.573	1.00	0.00	H
ATOM	2454	N	THR	C	389	108.520	73.223	126.588	1.00	30.00	N
ATOM	2455	CA	THR	C	389	108.853	71.799	126.689	1.00	30.00	C
ATOM	2456	C	THR	C	389	108.437	71.168	128.042	1.00	30.00	C
ATOM	2457	O	THR	C	389	108.350	69.944	128.131	1.00	30.00	O
ATOM	2458	CB	THR	C	389	110.379	71.549	126.491	1.00	20.00	C
ATOM	2459	CG2	THR	C	389	110.718	70.145	125.963	1.00	0.00	C
ATOM	2460	OG1	THR	C	389	110.920	72.472	125.563	1.00	0.00	O
ATOM	2461	H	THR	C	389	109.283	73.864	126.744	1.00	0.00	H
ATOM	2462	HA	THR	C	389	108.313	71.268	125.902	1.00	0.00	H
ATOM	2463	HB	THR	C	389	110.913	71.688	127.431	1.00	0.00	H
ATOM	2464	HG1	THR	C	389	111.865	72.316	125.480	1.00	0.00	H
ATOM	2465	HG21	THR	C	389	111.795	70.018	125.846	1.00	0.00	H
ATOM	2466	HG22	THR	C	389	110.372	69.361	126.634	1.00	0.00	H
ATOM	2467	HG23	THR	C	389	110.258	69.968	124.990	1.00	0.00	H
ATOM	2468	N	CYS	C	390	108.185	72.012	129.063	1.00	30.00	N
ATOM	2469	CA	CYS	C	390	107.804	71.614	130.419	1.00	30.00	C
ATOM	2470	C	CYS	C	390	106.274	71.560	130.579	1.00	30.00	C
ATOM	2471	O	CYS	C	390	105.764	70.545	131.054	1.00	30.00	O
ATOM	2472	CB	CYS	C	390	108.460	72.532	131.478	1.00	20.00	C
ATOM	2473	SG	CYS	C	390	108.174	71.950	133.176	1.00	0.00	S
ATOM	2474	H	CYS	C	390	108.247	73.008	128.901	1.00	0.00	H
ATOM	2475	HA	CYS	C	390	108.184	70.606	130.600	1.00	0.00	H

ATOM	2476	HB2	CYS	C	390	109.538	72.571	131.321	1.00	0.00	H
ATOM	2477	HB3	CYS	C	390	108.096	73.556	131.392	1.00	0.00	H
ATOM	2478	HG	CYS	C	390	106.859	72.198	133.212	1.00	0.00	H
ATOM	2479	N	GLU	C	391	105.583	72.645	130.188	1.00	30.00	N
ATOM	2480	CA	GLU	C	391	104.135	72.817	130.350	1.00	30.00	C
ATOM	2481	C	GLU	C	391	103.460	73.038	128.987	1.00	30.00	C
ATOM	2482	O	GLU	C	391	104.109	73.489	128.044	1.00	30.00	O
ATOM	2483	CB	GLU	C	391	103.859	74.043	131.256	1.00	20.00	C
ATOM	2484	CG	GLU	C	391	104.549	74.048	132.638	1.00	0.00	C
ATOM	2485	CD	GLU	C	391	104.165	72.885	133.554	1.00	0.00	C
ATOM	2486	OE1	GLU	C	391	103.019	72.398	133.437	1.00	0.00	O
ATOM	2487	OE2	GLU	C	391	105.030	72.519	134.379	1.00	0.00	O1-
ATOM	2488	H	GLU	C	391	106.072	73.426	129.775	1.00	0.00	H
ATOM	2489	HA	GLU	C	391	103.691	71.929	130.803	1.00	0.00	H
ATOM	2490	HB2	GLU	C	391	104.155	74.951	130.733	1.00	0.00	H
ATOM	2491	HB3	GLU	C	391	102.783	74.146	131.404	1.00	0.00	H
ATOM	2492	HG2	GLU	C	391	105.633	74.062	132.515	1.00	0.00	H
ATOM	2493	HG3	GLU	C	391	104.295	74.971	133.159	1.00	0.00	H
ATOM	2494	N	LYS	C	392	102.146	72.766	128.937	1.00	30.00	N
ATOM	2495	CA	LYS	C	392	101.267	73.127	127.823	1.00	30.00	C
ATOM	2496	C	LYS	C	392	100.640	74.505	128.104	1.00	30.00	C
ATOM	2497	O	LYS	C	392	100.184	74.737	129.224	1.00	30.00	O
ATOM	2498	CB	LYS	C	392	100.203	72.020	127.655	1.00	20.00	C
ATOM	2499	CG	LYS	C	392	99.231	72.242	126.481	1.00	0.00	C
ATOM	2500	CD	LYS	C	392	98.287	71.050	126.264	1.00	0.00	C
ATOM	2501	CE	LYS	C	392	97.315	71.277	125.099	1.00	0.00	C
ATOM	2502	NZ	LYS	C	392	96.428	70.117	124.906	1.00	0.00	N1+
ATOM	2503	H	LYS	C	392	101.685	72.413	129.763	1.00	0.00	H
ATOM	2504	HA	LYS	C	392	101.847	73.183	126.899	1.00	0.00	H
ATOM	2505	HB2	LYS	C	392	100.717	71.070	127.504	1.00	0.00	H
ATOM	2506	HB3	LYS	C	392	99.632	71.911	128.578	1.00	0.00	H
ATOM	2507	HG2	LYS	C	392	98.635	73.137	126.657	1.00	0.00	H
ATOM	2508	HG3	LYS	C	392	99.800	72.426	125.569	1.00	0.00	H
ATOM	2509	HD2	LYS	C	392	98.877	70.151	126.079	1.00	0.00	H
ATOM	2510	HD3	LYS	C	392	97.725	70.865	127.181	1.00	0.00	H
ATOM	2511	HE2	LYS	C	392	96.702	72.161	125.284	1.00	0.00	H
ATOM	2512	HE3	LYS	C	392	97.866	71.454	124.174	1.00	0.00	H
ATOM	2513	HZ1	LYS	C	392	95.801	70.296	124.135	1.00	0.00	H
ATOM	2514	HZ2	LYS	C	392	96.983	69.298	124.704	1.00	0.00	H
ATOM	2515	HZ3	LYS	C	392	95.891	69.960	125.747	1.00	0.00	H
ATOM	2516	N	ASN	C	393	100.632	75.384	127.081	1.00	30.00	N
ATOM	2517	CA	ASN	C	393	100.188	76.790	127.141	1.00	30.00	C
ATOM	2518	C	ASN	C	393	101.076	77.606	128.104	1.00	30.00	C
ATOM	2519	O	ASN	C	393	100.571	78.249	129.026	1.00	30.00	O
ATOM	2520	CB	ASN	C	393	98.670	76.922	127.457	1.00	20.00	C
ATOM	2521	CG	ASN	C	393	97.750	76.152	126.500	1.00	0.00	C
ATOM	2522	ND2	ASN	C	393	97.922	76.338	125.189	1.00	0.00	N
ATOM	2523	OD1	ASN	C	393	96.883	75.402	126.942	1.00	0.00	O
ATOM	2524	H	ASN	C	393	101.034	75.105	126.197	1.00	0.00	H
ATOM	2525	HA	ASN	C	393	100.356	77.226	126.157	1.00	0.00	H
ATOM	2526	HB2	ASN	C	393	98.461	76.591	128.475	1.00	0.00	H
ATOM	2527	HB3	ASN	C	393	98.377	77.972	127.417	1.00	0.00	H
ATOM	2528	HD21	ASN	C	393	97.329	75.857	124.529	1.00	0.00	H
ATOM	2529	HD22	ASN	C	393	98.644	76.956	124.846	1.00	0.00	H
ATOM	2530	N	SER	C	394	102.398	77.502	127.892	1.00	30.00	N
ATOM	2531	CA	SER	C	394	103.432	77.937	128.833	1.00	30.00	C
ATOM	2532	C	SER	C	394	103.773	79.438	128.712	1.00	30.00	C
ATOM	2533	O	SER	C	394	103.253	80.121	127.831	1.00	30.00	O
ATOM	2534	CB	SER	C	394	104.653	77.023	128.630	1.00	20.00	C
ATOM	2535	OG	SER	C	394	105.455	76.986	129.789	1.00	20.00	O
ATOM	2536	H	SER	C	394	102.724	76.994	127.081	1.00	0.00	H
ATOM	2537	HA	SER	C	394	103.048	77.766	129.841	1.00	0.00	H
ATOM	2538	HB2	SER	C	394	104.339	76.003	128.419	1.00	0.00	H
ATOM	2539	HB3	SER	C	394	105.250	77.356	127.782	1.00	0.00	H
ATOM	2540	HG	SER	C	394	106.213	76.419	129.618	1.00	0.00	H
ATOM	2541	N	VAL	C	395	104.613	79.930	129.640	1.00	30.00	N
ATOM	2542	CA	VAL	C	395	104.894	81.351	129.879	1.00	30.00	C
ATOM	2543	C	VAL	C	395	105.492	82.111	128.676	1.00	30.00	C
ATOM	2544	O	VAL	C	395	104.996	83.197	128.377	1.00	30.00	O
ATOM	2545	CB	VAL	C	395	105.806	81.558	131.126	1.00	20.00	C
ATOM	2546	CG1	VAL	C	395	106.234	83.018	131.397	1.00	20.00	C

```
ATOM   2547  CG2 VAL C 395     105.146  80.983 132.391  1.00 20.00           C
ATOM   2548  H   VAL C 395     105.035  79.283 130.294  1.00  0.00           H
ATOM   2549  HA  VAL C 395     103.930  81.817 130.095  1.00  0.00           H
ATOM   2550  HB  VAL C 395     106.726  81.001 130.964  1.00  0.00           H
ATOM   2551 HG11 VAL C 395     106.775  83.101 132.340  1.00  0.00           H
ATOM   2552 HG12 VAL C 395     106.902  83.400 130.625  1.00  0.00           H
ATOM   2553 HG13 VAL C 395     105.369  83.679 131.454  1.00  0.00           H
ATOM   2554 HG21 VAL C 395     105.783  81.130 133.263  1.00  0.00           H
ATOM   2555 HG22 VAL C 395     104.189  81.468 132.588  1.00  0.00           H
ATOM   2556 HG23 VAL C 395     104.964  79.912 132.305  1.00  0.00           H
ATOM   2557  N   LEU C 396     106.512  81.547 127.998  1.00 30.00           N
ATOM   2558  CA  LEU C 396     107.120  82.178 126.816  1.00 30.00           C
ATOM   2559  C   LEU C 396     106.158  82.219 125.617  1.00 30.00           C
ATOM   2560  O   LEU C 396     106.114  83.241 124.933  1.00 30.00           O
ATOM   2561  CB  LEU C 396     108.450  81.504 126.407  1.00 20.00           C
ATOM   2562  CG  LEU C 396     109.663  81.856 127.298  1.00 20.00           C
ATOM   2563  CD1 LEU C 396     109.664  81.075 128.616  1.00 20.00           C
ATOM   2564  CD2 LEU C 396     110.993  81.645 126.549  1.00 20.00           C
ATOM   2565  H   LEU C 396     106.873  80.644 128.277  1.00  0.00           H
ATOM   2566  HA  LEU C 396     107.338  83.217 127.072  1.00  0.00           H
ATOM   2567  HB2 LEU C 396     108.329  80.424 126.317  1.00  0.00           H
ATOM   2568  HB3 LEU C 396     108.683  81.854 125.401  1.00  0.00           H
ATOM   2569  HG  LEU C 396     109.611  82.920 127.536  1.00  0.00           H
ATOM   2570 HD11 LEU C 396     110.666  80.966 129.032  1.00  0.00           H
ATOM   2571 HD12 LEU C 396     109.042  81.561 129.368  1.00  0.00           H
ATOM   2572 HD13 LEU C 396     109.282  80.073 128.450  1.00  0.00           H
ATOM   2573 HD21 LEU C 396     111.676  82.472 126.744  1.00  0.00           H
ATOM   2574 HD22 LEU C 396     111.491  80.726 126.857  1.00  0.00           H
ATOM   2575 HD23 LEU C 396     110.862  81.579 125.469  1.00  0.00           H
ATOM   2576  N   GLU C 397     105.394  81.132 125.409  1.00 30.00           N
ATOM   2577  CA  GLU C 397     104.335  81.012 124.404  1.00 30.00           C
ATOM   2578  C   GLU C 397     103.225  82.073 124.551  1.00 30.00           C
ATOM   2579  O   GLU C 397     102.797  82.636 123.545  1.00 30.00           O
ATOM   2580  CB  GLU C 397     103.832  79.548 124.400  1.00 20.00           C
ATOM   2581  CG  GLU C 397     102.493  79.265 123.691  1.00  0.00           C
ATOM   2582  CD  GLU C 397     102.205  77.766 123.570  1.00  0.00           C
ATOM   2583  OE1 GLU C 397     102.365  77.056 124.588  1.00  0.00           O
ATOM   2584  OE2 GLU C 397     101.821  77.353 122.453  1.00  0.00           O1-
ATOM   2585  H   GLU C 397     105.522  80.324 126.003  1.00  0.00           H
ATOM   2586  HA  GLU C 397     104.796  81.193 123.431  1.00  0.00           H
ATOM   2587  HB2 GLU C 397     104.596  78.943 123.915  1.00  0.00           H
ATOM   2588  HB3 GLU C 397     103.775  79.176 125.424  1.00  0.00           H
ATOM   2589  HG2 GLU C 397     101.667  79.726 124.232  1.00  0.00           H
ATOM   2590  HG3 GLU C 397     102.506  79.703 122.696  1.00  0.00           H
ATOM   2591  N   VAL C 398     102.826  82.360 125.800  1.00 30.00           N
ATOM   2592  CA  VAL C 398     101.851  83.391 126.157  1.00 30.00           C
ATOM   2593  C   VAL C 398     102.346  84.830 125.874  1.00 30.00           C
ATOM   2594  O   VAL C 398     101.581  85.618 125.317  1.00 30.00           O
ATOM   2595  CB  VAL C 398     101.416  83.236 127.647  1.00 20.00           C
ATOM   2596  CG1 VAL C 398     100.763  84.473 128.294  1.00 20.00           C
ATOM   2597  CG2 VAL C 398     100.477  82.027 127.817  1.00 20.00           C
ATOM   2598  H   VAL C 398     103.220  81.838 126.572  1.00  0.00           H
ATOM   2599  HA  VAL C 398     100.971  83.230 125.530  1.00  0.00           H
ATOM   2600  HB  VAL C 398     102.309  83.019 128.233  1.00  0.00           H
ATOM   2601 HG11 VAL C 398     100.406  84.243 129.296  1.00  0.00           H
ATOM   2602 HG12 VAL C 398     101.467  85.298 128.397  1.00  0.00           H
ATOM   2603 HG13 VAL C 398      99.913  84.824 127.708  1.00  0.00           H
ATOM   2604 HG21 VAL C 398     100.267  81.835 128.869  1.00  0.00           H
ATOM   2605 HG22 VAL C 398      99.525  82.195 127.314  1.00  0.00           H
ATOM   2606 HG23 VAL C 398     100.906  81.116 127.401  1.00  0.00           H
ATOM   2607  N   ILE C 399     103.601  85.144 126.245  1.00 30.00           N
ATOM   2608  CA  ILE C 399     104.199  86.477 126.081  1.00 30.00           C
ATOM   2609  C   ILE C 399     104.516  86.807 124.605  1.00 30.00           C
ATOM   2610  O   ILE C 399     104.263  87.932 124.169  1.00 30.00           O
ATOM   2611  CB  ILE C 399     105.497  86.645 126.937  1.00 20.00           C
ATOM   2612  CG1 ILE C 399     105.188  86.532 128.448  1.00 20.00           C
ATOM   2613  CG2 ILE C 399     106.282  87.952 126.668  1.00 20.00           C
ATOM   2614  CD1 ILE C 399     106.407  86.167 129.309  1.00 20.00           C
ATOM   2615  H   ILE C 399     104.176  84.446 126.698  1.00  0.00           H
ATOM   2616  HA  ILE C 399     103.471  87.212 126.432  1.00  0.00           H
ATOM   2617  HB  ILE C 399     106.158  85.814 126.684  1.00  0.00           H
```

```
ATOM   2618 HG12 ILE C 399     104.746  87.461 128.810  1.00  0.00           H
ATOM   2619 HG13 ILE C 399     104.430  85.770 128.618  1.00  0.00           H
ATOM   2620 HG21 ILE C 399     107.101  88.082 127.373  1.00  0.00           H
ATOM   2621 HG22 ILE C 399     106.728  87.973 125.673  1.00  0.00           H
ATOM   2622 HG23 ILE C 399     105.636  88.826 126.762  1.00  0.00           H
ATOM   2623 HD11 ILE C 399     106.097  85.908 130.321  1.00  0.00           H
ATOM   2624 HD12 ILE C 399     106.941  85.309 128.899  1.00  0.00           H
ATOM   2625 HD13 ILE C 399     107.113  86.993 129.386  1.00  0.00           H
ATOM   2626  N   ALA C 400     105.053  85.819 123.869  1.00 30.00           N
ATOM   2627  CA  ALA C 400     105.460  85.947 122.470  1.00 30.00           C
ATOM   2628  C   ALA C 400     104.286  86.035 121.482  1.00 30.00           C
ATOM   2629  O   ALA C 400     104.448  86.676 120.444  1.00 30.00           O
ATOM   2630  CB  ALA C 400     106.374  84.770 122.102  1.00 20.00           C
ATOM   2631  H   ALA C 400     105.230  84.920 124.299  1.00  0.00           H
ATOM   2632  HA  ALA C 400     106.043  86.866 122.372  1.00  0.00           H
ATOM   2633  HB1 ALA C 400     106.727  84.843 121.073  1.00  0.00           H
ATOM   2634  HB2 ALA C 400     107.254  84.741 122.745  1.00  0.00           H
ATOM   2635  HB3 ALA C 400     105.853  83.816 122.205  1.00  0.00           H
ATOM   2636  N   TYR C 401     103.145  85.398 121.806  1.00 30.00           N
ATOM   2637  CA  TYR C 401     101.958  85.332 120.942  1.00 30.00           C
ATOM   2638  C   TYR C 401     100.803  86.213 121.472  1.00 30.00           C
ATOM   2639  O   TYR C 401      99.649  85.963 121.120  1.00 30.00           O
ATOM   2640  CB  TYR C 401     101.520  83.855 120.741  1.00 20.00           C
ATOM   2641  CG  TYR C 401     102.559  82.838 120.263  1.00 20.00           C
ATOM   2642  CD1 TYR C 401     103.726  83.219 119.562  1.00 20.00           C
ATOM   2643  CD2 TYR C 401     102.338  81.468 120.519  1.00 20.00           C
ATOM   2644  CE1 TYR C 401     104.668  82.254 119.158  1.00 20.00           C
ATOM   2645  CE2 TYR C 401     103.272  80.498 120.102  1.00 20.00           C
ATOM   2646  CZ  TYR C 401     104.440  80.890 119.422  1.00 20.00           C
ATOM   2647  OH  TYR C 401     105.337  79.949 119.003  1.00 20.00           O
ATOM   2648  H   TYR C 401     103.093  84.876 122.671  1.00  0.00           H
ATOM   2649  HA  TYR C 401     102.189  85.733 119.954  1.00  0.00           H
ATOM   2650  HB2 TYR C 401     101.111  83.484 121.682  1.00  0.00           H
ATOM   2651  HB3 TYR C 401     100.696  83.815 120.027  1.00  0.00           H
ATOM   2652  HD1 TYR C 401     103.914  84.254 119.322  1.00  0.00           H
ATOM   2653  HD2 TYR C 401     101.448  81.154 121.045  1.00  0.00           H
ATOM   2654  HE1 TYR C 401     105.559  82.562 118.630  1.00  0.00           H
ATOM   2655  HE2 TYR C 401     103.088  79.453 120.306  1.00  0.00           H
ATOM   2656  HH  TYR C 401     105.995  80.298 118.387  1.00  0.00           H
ATOM   2657  N   SER C 402     101.121  87.236 122.288  1.00 30.00           N
ATOM   2658  CA  SER C 402     100.180  88.232 122.817  1.00 30.00           C
ATOM   2659  C   SER C 402      99.560  89.137 121.730  1.00 30.00           C
ATOM   2660  O   SER C 402     100.138  89.296 120.655  1.00 30.00           O
ATOM   2661  CB  SER C 402     100.897  89.068 123.901  1.00 20.00           C
ATOM   2662  OG  SER C 402     100.731  88.477 125.173  1.00 20.00           O
ATOM   2663  H   SER C 402     102.089  87.370 122.541  1.00  0.00           H
ATOM   2664  HA  SER C 402      99.350  87.692 123.278  1.00  0.00           H
ATOM   2665  HB2 SER C 402     101.957  89.185 123.685  1.00  0.00           H
ATOM   2666  HB3 SER C 402     100.484  90.077 123.968  1.00  0.00           H
ATOM   2667  HG  SER C 402     101.133  87.602 125.164  1.00  0.00           H
ATOM   2668  N   SER C 403      98.389  89.718 122.047  1.00 30.00           N
ATOM   2669  CA  SER C 403      97.570  90.552 121.155  1.00 30.00           C
ATOM   2670  C   SER C 403      98.154  91.936 120.791  1.00 30.00           C
ATOM   2671  O   SER C 403      97.613  92.581 119.894  1.00 30.00           O
ATOM   2672  CB  SER C 403      96.166  90.690 121.782  1.00 20.00           C
ATOM   2673  OG  SER C 403      96.181  91.501 122.944  1.00  0.00           O
ATOM   2674  H   SER C 403      97.990  89.551 122.959  1.00  0.00           H
ATOM   2675  HA  SER C 403      97.461  90.002 120.218  1.00  0.00           H
ATOM   2676  HB2 SER C 403      95.476  91.138 121.066  1.00  0.00           H
ATOM   2677  HB3 SER C 403      95.757  89.710 122.033  1.00  0.00           H
ATOM   2678  HG  SER C 403      95.281  91.580 123.272  1.00  0.00           H
ATOM   2679  N   SER C 404      99.211  92.370 121.502  1.00 30.00           N
ATOM   2680  CA  SER C 404      99.872  93.681 121.420  1.00 30.00           C
ATOM   2681  C   SER C 404      99.122  94.814 122.159  1.00 30.00           C
ATOM   2682  O   SER C 404      99.667  95.913 122.264  1.00 30.00           O
ATOM   2683  CB  SER C 404     100.251  94.046 119.962  1.00 20.00           C
ATOM   2684  OG  SER C 404     101.249  95.046 119.927  1.00  0.00           O
ATOM   2685  H   SER C 404      99.580  91.752 122.211  1.00  0.00           H
ATOM   2686  HA  SER C 404     100.801  93.549 121.970  1.00  0.00           H
ATOM   2687  HB2 SER C 404     100.630  93.175 119.429  1.00  0.00           H
ATOM   2688  HB3 SER C 404      99.386  94.406 119.405  1.00  0.00           H
```

```
ATOM   2689  HG  SER C 404     100.893  95.843 120.331  1.00  0.00           H
ATOM   2690  N   GLU C 405      97.919  94.528 122.686  1.00 30.00           N
ATOM   2691  CA  GLU C 405      97.107  95.444 123.496  1.00 30.00           C
ATOM   2692  C   GLU C 405      97.464  95.381 124.997  1.00 30.00           C
ATOM   2693  O   GLU C 405      97.037  96.252 125.753  1.00 30.00           O
ATOM   2694  CB  GLU C 405      95.615  95.131 123.257  1.00 20.00           C
ATOM   2695  CG  GLU C 405      95.199  95.273 121.776  1.00  0.00           C
ATOM   2696  CD  GLU C 405      93.727  94.945 121.528  1.00  0.00           C
ATOM   2697  OE1 GLU C 405      92.875  95.444 122.294  1.00  0.00           O
ATOM   2698  OE2 GLU C 405      93.467  94.217 120.545  1.00  0.00           O1-
ATOM   2699  H   GLU C 405      97.528  93.607 122.545  1.00  0.00           H
ATOM   2700  HA  GLU C 405      97.289  96.468 123.167  1.00  0.00           H
ATOM   2701  HB2 GLU C 405      95.388  94.123 123.606  1.00  0.00           H
ATOM   2702  HB3 GLU C 405      95.006  95.802 123.867  1.00  0.00           H
ATOM   2703  HG2 GLU C 405      95.384  96.291 121.434  1.00  0.00           H
ATOM   2704  HG3 GLU C 405      95.803  94.619 121.146  1.00  0.00           H
ATOM   2705  N   THR C 406      98.263  94.373 125.390  1.00 30.00           N
ATOM   2706  CA  THR C 406      98.872  94.201 126.710  1.00 30.00           C
ATOM   2707  C   THR C 406      99.923  95.313 126.985  1.00 30.00           C
ATOM   2708  O   THR C 406     100.617  95.716 126.049  1.00 30.00           O
ATOM   2709  CB  THR C 406      99.610  92.835 126.748  1.00 20.00           C
ATOM   2710  CG2 THR C 406      98.670  91.636 126.562  1.00 20.00           C
ATOM   2711  OG1 THR C 406     100.608  92.756 125.748  1.00 20.00           O
ATOM   2712  H   THR C 406      98.570  93.709 124.694  1.00  0.00           H
ATOM   2713  HA  THR C 406      98.065  94.211 127.441  1.00  0.00           H
ATOM   2714  HB  THR C 406     100.109  92.723 127.712  1.00  0.00           H
ATOM   2715  HG1 THR C 406     101.195  93.511 125.845  1.00  0.00           H
ATOM   2716 HG21 THR C 406      99.211  90.695 126.664  1.00  0.00           H
ATOM   2717 HG22 THR C 406      97.879  91.646 127.311  1.00  0.00           H
ATOM   2718 HG23 THR C 406      98.197  91.637 125.579  1.00  0.00           H
ATOM   2719  N   PRO C 407     100.003  95.807 128.243  1.00 30.00           N
ATOM   2720  CA  PRO C 407     100.771  97.020 128.587  1.00 30.00           C
ATOM   2721  C   PRO C 407     102.289  96.972 128.319  1.00 30.00           C
ATOM   2722  O   PRO C 407     102.795  97.912 127.707  1.00 30.00           O
ATOM   2723  CB  PRO C 407     100.435  97.269 130.071  1.00 20.00           C
ATOM   2724  CG  PRO C 407      99.997  95.915 130.605  1.00  0.00           C
ATOM   2725  CD  PRO C 407      99.279  95.303 129.410  1.00  0.00           C
ATOM   2726  HA  PRO C 407     100.369  97.851 128.003  1.00  0.00           H
ATOM   2727  HB2 PRO C 407     101.258  97.693 130.649  1.00  0.00           H
ATOM   2728  HB3 PRO C 407      99.604  97.971 130.138  1.00  0.00           H
ATOM   2729  HG2 PRO C 407     100.882  95.327 130.846  1.00  0.00           H
ATOM   2730  HG3 PRO C 407      99.379  95.974 131.501  1.00  0.00           H
ATOM   2731  HD2 PRO C 407      99.265  94.216 129.466  1.00  0.00           H
ATOM   2732  HD3 PRO C 407      98.248  95.656 129.365  1.00  0.00           H
ATOM   2733  N   ASN C 408     102.989  95.918 128.780  1.00 30.00           N
ATOM   2734  CA  ASN C 408     104.458  95.823 128.735  1.00 30.00           C
ATOM   2735  C   ASN C 408     104.899  94.384 128.396  1.00 30.00           C
ATOM   2736  O   ASN C 408     105.601  93.758 129.191  1.00 30.00           O
ATOM   2737  CB  ASN C 408     105.076  96.327 130.072  1.00 20.00           C
ATOM   2738  CG  ASN C 408     104.905  97.825 130.345  1.00  0.00           C
ATOM   2739  ND2 ASN C 408     105.894  98.636 129.965  1.00  0.00           N
ATOM   2740  OD1 ASN C 408     103.899  98.246 130.913  1.00  0.00           O
ATOM   2741  H   ASN C 408     102.511  95.181 129.278  1.00  0.00           H
ATOM   2742  HA  ASN C 408     104.831  96.457 127.928  1.00  0.00           H
ATOM   2743  HB2 ASN C 408     104.658  95.770 130.911  1.00  0.00           H
ATOM   2744  HB3 ASN C 408     106.148  96.129 130.079  1.00  0.00           H
ATOM   2745 HD21 ASN C 408     105.821  99.630 130.120  1.00  0.00           H
ATOM   2746 HD22 ASN C 408     106.745  98.262 129.554  1.00  0.00           H
ATOM   2747  N   ARG C 409     104.512  93.886 127.206  1.00 30.00           N
ATOM   2748  CA  ARG C 409     104.942  92.575 126.688  1.00 30.00           C
ATOM   2749  C   ARG C 409     106.425  92.509 126.296  1.00 30.00           C
ATOM   2750  O   ARG C 409     107.042  91.454 126.445  1.00 30.00           O
ATOM   2751  CB  ARG C 409     104.038  92.125 125.526  1.00 20.00           C
ATOM   2752  CG  ARG C 409     104.096  93.015 124.268  1.00 20.00           C
ATOM   2753  CD  ARG C 409     103.157  92.551 123.154  1.00 20.00           C
ATOM   2754  NE  ARG C 409     103.521  91.221 122.650  1.00 20.00           N
ATOM   2755  CZ  ARG C 409     103.173  90.721 121.454  1.00 20.00           C
ATOM   2756  NH1 ARG C 409     102.489  91.451 120.569  1.00 20.00           N
ATOM   2757  NH2 ARG C 409     103.517  89.471 121.132  1.00 20.00           N1+
ATOM   2758  H   ARG C 409     103.930  94.444 126.599  1.00  0.00           H
ATOM   2759  HA  ARG C 409     104.822  91.846 127.480  1.00  0.00           H
```

```
ATOM   2760  HB2 ARG C 409     103.016  92.070 125.891  1.00  0.00           H
ATOM   2761  HB3 ARG C 409     104.304  91.101 125.259  1.00  0.00           H
ATOM   2762  HG2 ARG C 409     103.852  94.047 124.522  1.00  0.00           H
ATOM   2763  HG3 ARG C 409     105.109  93.039 123.870  1.00  0.00           H
ATOM   2764  HD2 ARG C 409     103.203  93.283 122.347  1.00  0.00           H
ATOM   2765  HD3 ARG C 409     102.125  92.515 123.501  1.00  0.00           H
ATOM   2766  HE  ARG C 409     104.007  90.616 123.297  1.00  0.00           H
ATOM   2767 HH11 ARG C 409     102.261  91.052 119.659  1.00  0.00           H
ATOM   2768 HH12 ARG C 409     102.251  92.412 120.762  1.00  0.00           H
ATOM   2769 HH21 ARG C 409     103.257  89.098 120.228  1.00  0.00           H
ATOM   2770 HH22 ARG C 409     104.050  88.897 121.769  1.00  0.00           H
ATOM   2771  N   HIS C 410     106.963  93.641 125.819  1.00 30.00           N
ATOM   2772  CA  HIS C 410     108.346  93.794 125.379  1.00 30.00           C
ATOM   2773  C   HIS C 410     109.353  93.913 126.543  1.00 30.00           C
ATOM   2774  O   HIS C 410     110.554  93.926 126.276  1.00 30.00           O
ATOM   2775  CB  HIS C 410     108.413  95.002 124.423  1.00 20.00           C
ATOM   2776  CG  HIS C 410     107.461  94.953 123.251  1.00 20.00           C
ATOM   2777  CD2 HIS C 410     106.389  95.747 122.905  1.00 20.00           C
ATOM   2778  ND1 HIS C 410     107.543  93.991 122.260  1.00 20.00           N
ATOM   2779  CE1 HIS C 410     106.553  94.225 121.395  1.00 20.00           C
ATOM   2780  NE2 HIS C 410     105.810  95.274 121.725  1.00 20.00           N
ATOM   2781  H   HIS C 410     106.383  94.464 125.736  1.00  0.00           H
ATOM   2782  HA  HIS C 410     108.628  92.905 124.813  1.00  0.00           H
ATOM   2783  HB2 HIS C 410     109.407  95.060 123.995  1.00  0.00           H
ATOM   2784  HB3 HIS C 410     108.250  95.932 124.970  1.00  0.00           H
ATOM   2785  HD1 HIS C 410     108.223  93.246 122.202  1.00  0.00           H
ATOM   2786  HD2 HIS C 410     105.987  96.612 123.413  1.00  0.00           H
ATOM   2787  HE1 HIS C 410     106.373  93.621 120.518  1.00  0.00           H
ATOM   2788  N   ASP C 411     108.851  93.983 127.792  1.00 30.00           N
ATOM   2789  CA  ASP C 411     109.623  94.138 129.032  1.00 30.00           C
ATOM   2790  C   ASP C 411     109.486  92.931 129.983  1.00 30.00           C
ATOM   2791  O   ASP C 411     110.271  92.850 130.927  1.00 30.00           O
ATOM   2792  CB  ASP C 411     109.283  95.445 129.791  1.00 20.00           C
ATOM   2793  CG  ASP C 411     109.285  96.716 128.932  1.00 20.00           C
ATOM   2794  OD1 ASP C 411     110.138  96.808 128.021  1.00 20.00           O
ATOM   2795  OD2 ASP C 411     108.486  97.618 129.264  1.00 20.00           O1-
ATOM   2796  H   ASP C 411     107.848  93.973 127.908  1.00  0.00           H
ATOM   2797  HA  ASP C 411     110.684  94.189 128.782  1.00  0.00           H
ATOM   2798  HB2 ASP C 411     108.292  95.335 130.231  1.00  0.00           H
ATOM   2799  HB3 ASP C 411     109.980  95.598 130.616  1.00  0.00           H
ATOM   2800  N   MET C 412     108.546  91.995 129.738  1.00 30.00           N
ATOM   2801  CA  MET C 412     108.457  90.732 130.492  1.00 30.00           C
ATOM   2802  C   MET C 412     109.579  89.737 130.141  1.00 30.00           C
ATOM   2803  O   MET C 412     109.933  88.919 130.990  1.00 30.00           O
ATOM   2804  CB  MET C 412     107.077  90.070 130.303  1.00 20.00           C
ATOM   2805  CG  MET C 412     105.974  90.724 131.145  1.00 20.00           C
ATOM   2806  SD  MET C 412     104.376  89.879 131.088  1.00 20.00           S
ATOM   2807  CE  MET C 412     103.877  90.330 129.412  1.00 20.00           C
ATOM   2808  H   MET C 412     107.911  92.107 128.960  1.00  0.00           H
ATOM   2809  HA  MET C 412     108.575  90.957 131.554  1.00  0.00           H
ATOM   2810  HB2 MET C 412     106.807  90.076 129.247  1.00  0.00           H
ATOM   2811  HB3 MET C 412     107.126  89.020 130.594  1.00  0.00           H
ATOM   2812  HG2 MET C 412     106.283  90.749 132.191  1.00  0.00           H
ATOM   2813  HG3 MET C 412     105.827  91.757 130.836  1.00  0.00           H
ATOM   2814  HE1 MET C 412     102.876  89.976 129.185  1.00  0.00           H
ATOM   2815  HE2 MET C 412     103.864  91.416 129.332  1.00  0.00           H
ATOM   2816  HE3 MET C 412     104.562  89.914 128.676  1.00  0.00           H
ATOM   2817  N   LEU C 413     110.135  89.843 128.922  1.00 30.00           N
ATOM   2818  CA  LEU C 413     111.276  89.054 128.450  1.00 30.00           C
ATOM   2819  C   LEU C 413     112.623  89.520 129.041  1.00 30.00           C
ATOM   2820  O   LEU C 413     113.597  88.777 128.931  1.00 30.00           O
ATOM   2821  CB  LEU C 413     111.316  89.104 126.904  1.00 20.00           C
ATOM   2822  CG  LEU C 413     110.079  88.490 126.207  1.00 20.00           C
ATOM   2823  CD1 LEU C 413     110.057  88.848 124.708  1.00 20.00           C
ATOM   2824  CD2 LEU C 413     109.960  86.968 126.457  1.00 20.00           C
ATOM   2825  H   LEU C 413     109.780  90.537 128.280  1.00  0.00           H
ATOM   2826  HA  LEU C 413     111.136  88.020 128.770  1.00  0.00           H
ATOM   2827  HB2 LEU C 413     111.418  90.147 126.596  1.00  0.00           H
ATOM   2828  HB3 LEU C 413     112.209  88.597 126.537  1.00  0.00           H
ATOM   2829  HG  LEU C 413     109.189  88.955 126.633  1.00  0.00           H
ATOM   2830 HD11 LEU C 413     109.859  87.990 124.067  1.00  0.00           H
```

ATOM	2831	HD12	LEU	C	413	109.276	89.580	124.504	1.00	0.00	H
ATOM	2832	HD13	LEU	C	413	111.001	89.285	124.378	1.00	0.00	H
ATOM	2833	HD21	LEU	C	413	109.874	86.389	125.538	1.00	0.00	H
ATOM	2834	HD22	LEU	C	413	110.822	86.573	126.993	1.00	0.00	H
ATOM	2835	HD23	LEU	C	413	109.080	86.741	127.059	1.00	0.00	H
ATOM	2836	N	LEU	C	414	112.657	90.719	129.651	1.00	30.00	N
ATOM	2837	CA	LEU	C	414	113.834	91.314	130.292	1.00	30.00	C
ATOM	2838	C	LEU	C	414	114.061	90.802	131.729	1.00	30.00	C
ATOM	2839	O	LEU	C	414	115.141	91.043	132.267	1.00	30.00	O
ATOM	2840	CB	LEU	C	414	113.733	92.860	130.254	1.00	20.00	C
ATOM	2841	CG	LEU	C	414	114.164	93.518	128.922	1.00	20.00	C
ATOM	2842	CD1	LEU	C	414	113.366	93.043	127.692	1.00	20.00	C
ATOM	2843	CD2	LEU	C	414	114.135	95.055	129.044	1.00	20.00	C
ATOM	2844	H	LEU	C	414	111.809	91.265	129.706	1.00	0.00	H
ATOM	2845	HA	LEU	C	414	114.720	91.031	129.728	1.00	0.00	H
ATOM	2846	HB2	LEU	C	414	112.728	93.181	130.522	1.00	0.00	H
ATOM	2847	HB3	LEU	C	414	114.379	93.273	131.031	1.00	0.00	H
ATOM	2848	HG	LEU	C	414	115.205	93.239	128.755	1.00	0.00	H
ATOM	2849	HD11	LEU	C	414	113.865	92.204	127.206	1.00	0.00	H
ATOM	2850	HD12	LEU	C	414	112.361	92.719	127.957	1.00	0.00	H
ATOM	2851	HD13	LEU	C	414	113.264	93.828	126.942	1.00	0.00	H
ATOM	2852	HD21	LEU	C	414	115.033	95.499	128.613	1.00	0.00	H
ATOM	2853	HD22	LEU	C	414	113.273	95.488	128.535	1.00	0.00	H
ATOM	2854	HD23	LEU	C	414	114.079	95.385	130.083	1.00	0.00	H
ATOM	2855	N	VAL	C	415	113.078	90.095	132.318	1.00	30.00	N
ATOM	2856	CA	VAL	C	415	113.170	89.483	133.647	1.00	30.00	C
ATOM	2857	C	VAL	C	415	114.180	88.312	133.632	1.00	30.00	C
ATOM	2858	O	VAL	C	415	114.153	87.502	132.704	1.00	30.00	O
ATOM	2859	CB	VAL	C	415	111.771	89.001	134.131	1.00	20.00	C
ATOM	2860	CG1	VAL	C	415	111.769	88.307	135.508	1.00	20.00	C
ATOM	2861	CG2	VAL	C	415	110.780	90.181	134.178	1.00	20.00	C
ATOM	2862	H	VAL	C	415	112.221	89.924	131.811	1.00	0.00	H
ATOM	2863	HA	VAL	C	415	113.530	90.245	134.341	1.00	0.00	H
ATOM	2864	HB	VAL	C	415	111.387	88.278	133.408	1.00	0.00	H
ATOM	2865	HG11	VAL	C	415	110.762	87.990	135.776	1.00	0.00	H
ATOM	2866	HG12	VAL	C	415	112.384	87.415	135.530	1.00	0.00	H
ATOM	2867	HG13	VAL	C	415	112.128	88.976	136.290	1.00	0.00	H
ATOM	2868	HG21	VAL	C	415	109.822	89.882	134.597	1.00	0.00	H
ATOM	2869	HG22	VAL	C	415	111.163	90.992	134.799	1.00	0.00	H
ATOM	2870	HG23	VAL	C	415	110.586	90.589	133.186	1.00	0.00	H
ATOM	2871	N	GLU	C	416	115.080	88.301	134.633	1.00	30.00	N
ATOM	2872	CA	GLU	C	416	116.375	87.603	134.652	1.00	30.00	C
ATOM	2873	C	GLU	C	416	116.443	86.125	134.195	1.00	30.00	C
ATOM	2874	O	GLU	C	416	117.363	85.818	133.433	1.00	30.00	O
ATOM	2875	CB	GLU	C	416	117.049	87.768	136.039	1.00	20.00	C
ATOM	2876	CG	GLU	C	416	117.290	89.222	136.496	1.00	0.00	C
ATOM	2877	CD	GLU	C	416	118.308	89.958	135.626	1.00	0.00	C
ATOM	2878	OE1	GLU	C	416	119.515	89.694	135.824	1.00	0.00	O
ATOM	2879	OE2	GLU	C	416	117.865	90.754	134.773	1.00	0.00	O1-
ATOM	2880	H	GLU	C	416	114.979	88.993	135.361	1.00	0.00	H
ATOM	2881	HA	GLU	C	416	116.984	88.146	133.927	1.00	0.00	H
ATOM	2882	HB2	GLU	C	416	116.453	87.274	136.804	1.00	0.00	H
ATOM	2883	HB3	GLU	C	416	118.002	87.237	136.047	1.00	0.00	H
ATOM	2884	HG2	GLU	C	416	116.356	89.783	136.519	1.00	0.00	H
ATOM	2885	HG3	GLU	C	416	117.661	89.220	137.522	1.00	0.00	H
ATOM	2886	N	PRO	C	417	115.502	85.237	134.608	1.00	30.00	N
ATOM	2887	CA	PRO	C	417	115.531	83.824	134.185	1.00	30.00	C
ATOM	2888	C	PRO	C	417	115.182	83.613	132.704	1.00	30.00	C
ATOM	2889	O	PRO	C	417	115.754	82.713	132.096	1.00	30.00	O
ATOM	2890	CB	PRO	C	417	114.522	83.117	135.113	1.00	20.00	C
ATOM	2891	CG	PRO	C	417	114.296	84.091	136.255	1.00	20.00	C
ATOM	2892	CD	PRO	C	417	114.425	85.439	135.572	1.00	20.00	C
ATOM	2893	HA	PRO	C	417	116.530	83.430	134.385	1.00	0.00	H
ATOM	2894	HB2	PRO	C	417	113.573	82.942	134.603	1.00	0.00	H
ATOM	2895	HB3	PRO	C	417	114.884	82.151	135.467	1.00	0.00	H
ATOM	2896	HG2	PRO	C	417	113.346	83.948	136.765	1.00	0.00	H
ATOM	2897	HG3	PRO	C	417	115.093	83.991	136.989	1.00	0.00	H
ATOM	2898	HD2	PRO	C	417	114.588	86.233	136.298	1.00	0.00	H
ATOM	2899	HD3	PRO	C	417	113.516	85.654	135.013	1.00	0.00	H
ATOM	2900	N	LEU	C	418	114.270	84.440	132.162	1.00	30.00	N
ATOM	2901	CA	LEU	C	418	113.819	84.393	130.769	1.00	30.00	C

```
ATOM   2902  C   LEU C 418     114.826  85.040 129.805  1.00 30.00           C
ATOM   2903  O   LEU C 418     114.994  84.537 128.694  1.00 30.00           O
ATOM   2904  CB  LEU C 418     112.447  85.097 130.629  1.00 20.00           C
ATOM   2905  CG  LEU C 418     111.311  84.546 131.522  1.00 20.00           C
ATOM   2906  CD1 LEU C 418     110.018  85.369 131.328  1.00 20.00           C
ATOM   2907  CD2 LEU C 418     111.074  83.035 131.322  1.00 20.00           C
ATOM   2908  H   LEU C 418     113.864  85.171 132.730  1.00  0.00           H
ATOM   2909  HA  LEU C 418     113.711  83.347 130.477  1.00  0.00           H
ATOM   2910  HB2 LEU C 418     112.127  85.041 129.586  1.00  0.00           H
ATOM   2911  HB3 LEU C 418     112.568  86.161 130.842  1.00  0.00           H
ATOM   2912  HG  LEU C 418     111.610  84.688 132.562  1.00  0.00           H
ATOM   2913 HD11 LEU C 418     109.676  85.784 132.277  1.00  0.00           H
ATOM   2914 HD12 LEU C 418     110.168  86.209 130.650  1.00  0.00           H
ATOM   2915 HD13 LEU C 418     109.196  84.782 130.917  1.00  0.00           H
ATOM   2916 HD21 LEU C 418     110.015  82.781 131.285  1.00  0.00           H
ATOM   2917 HD22 LEU C 418     111.523  82.672 130.398  1.00  0.00           H
ATOM   2918 HD23 LEU C 418     111.508  82.464 132.144  1.00  0.00           H
ATOM   2919  N   ASN C 419     115.464  86.140 130.242  1.00 30.00           N
ATOM   2920  CA  ASN C 419     116.347  86.977 129.429  1.00 30.00           C
ATOM   2921  C   ASN C 419     117.629  86.254 128.977  1.00 30.00           C
ATOM   2922  O   ASN C 419     117.970  86.325 127.796  1.00 30.00           O
ATOM   2923  CB  ASN C 419     116.662  88.282 130.201  1.00 20.00           C
ATOM   2924  CG  ASN C 419     117.198  89.423 129.328  1.00 20.00           C
ATOM   2925  ND2 ASN C 419     116.500  89.757 128.240  1.00 20.00           N
ATOM   2926  OD1 ASN C 419     118.229  90.011 129.640  1.00 20.00           O
ATOM   2927  H   ASN C 419     115.257  86.488 131.169  1.00  0.00           H
ATOM   2928  HA  ASN C 419     115.817  87.223 128.507  1.00  0.00           H
ATOM   2929  HB2 ASN C 419     115.746  88.657 130.657  1.00  0.00           H
ATOM   2930  HB3 ASN C 419     117.345  88.089 131.031  1.00  0.00           H
ATOM   2931 HD21 ASN C 419     116.820  90.505 127.646  1.00  0.00           H
ATOM   2932 HD22 ASN C 419     115.632  89.284 128.028  1.00  0.00           H
ATOM   2933  N   ARG C 420     118.284  85.543 129.910  1.00 30.00           N
ATOM   2934  CA  ARG C 420     119.475  84.737 129.629  1.00 30.00           C
ATOM   2935  C   ARG C 420     119.145  83.337 129.081  1.00 30.00           C
ATOM   2936  O   ARG C 420     120.048  82.689 128.556  1.00 30.00           O
ATOM   2937  CB  ARG C 420     120.356  84.650 130.891  1.00 20.00           C
ATOM   2938  CG  ARG C 420     120.899  86.015 131.342  1.00 20.00           C
ATOM   2939  CD  ARG C 420     121.961  85.899 132.443  1.00 20.00           C
ATOM   2940  NE  ARG C 420     122.308  87.215 132.991  1.00 20.00           N
ATOM   2941  CZ  ARG C 420     121.576  87.878 133.901  1.00 20.00           C
ATOM   2942  NH1 ARG C 420     121.934  89.112 134.271  1.00 20.00           N
ATOM   2943  NH2 ARG C 420     120.481  87.335 134.449  1.00 20.00           N1+
ATOM   2944  H   ARG C 420     117.945  85.535 130.862  1.00  0.00           H
ATOM   2945  HA  ARG C 420     120.061  85.244 128.860  1.00  0.00           H
ATOM   2946  HB2 ARG C 420     119.802  84.189 131.710  1.00  0.00           H
ATOM   2947  HB3 ARG C 420     121.208  83.999 130.691  1.00  0.00           H
ATOM   2948  HG2 ARG C 420     121.395  86.441 130.468  1.00  0.00           H
ATOM   2949  HG3 ARG C 420     120.111  86.720 131.613  1.00  0.00           H
ATOM   2950  HD2 ARG C 420     121.725  85.157 133.203  1.00  0.00           H
ATOM   2951  HD3 ARG C 420     122.887  85.571 131.969  1.00  0.00           H
ATOM   2952  HE  ARG C 420     123.117  87.668 132.591  1.00  0.00           H
ATOM   2953 HH11 ARG C 420     121.339  89.616 134.922  1.00  0.00           H
ATOM   2954 HH12 ARG C 420     122.757  89.557 133.894  1.00  0.00           H
ATOM   2955 HH21 ARG C 420     119.938  87.888 135.104  1.00  0.00           H
ATOM   2956 HH22 ARG C 420     120.201  86.389 134.226  1.00  0.00           H
ATOM   2957  N   LEU C 421     117.884  82.885 129.208  1.00 30.00           N
ATOM   2958  CA  LEU C 421     117.404  81.605 128.677  1.00 30.00           C
ATOM   2959  C   LEU C 421     117.197  81.656 127.158  1.00 30.00           C
ATOM   2960  O   LEU C 421     117.575  80.707 126.474  1.00 30.00           O
ATOM   2961  CB  LEU C 421     116.146  81.175 129.460  1.00 20.00           C
ATOM   2962  CG  LEU C 421     115.349  79.974 128.907  1.00 20.00           C
ATOM   2963  CD1 LEU C 421     114.730  79.144 130.045  1.00 20.00           C
ATOM   2964  CD2 LEU C 421     114.286  80.382 127.866  1.00 20.00           C
ATOM   2965  H   LEU C 421     117.186  83.476 129.637  1.00  0.00           H
ATOM   2966  HA  LEU C 421     118.171  80.850 128.866  1.00  0.00           H
ATOM   2967  HB2 LEU C 421     116.494  80.929 130.461  1.00  0.00           H
ATOM   2968  HB3 LEU C 421     115.469  82.019 129.587  1.00  0.00           H
ATOM   2969  HG  LEU C 421     116.068  79.322 128.415  1.00  0.00           H
ATOM   2970 HD11 LEU C 421     114.683  78.093 129.761  1.00  0.00           H
ATOM   2971 HD12 LEU C 421     115.305  79.192 130.970  1.00  0.00           H
ATOM   2972 HD13 LEU C 421     113.720  79.475 130.284  1.00  0.00           H
```

```
ATOM   2973 HD21 LEU C 421     114.545  80.002 126.879  1.00  0.00           H
ATOM   2974 HD22 LEU C 421     113.300  79.994 128.113  1.00  0.00           H
ATOM   2975 HD23 LEU C 421     114.187  81.464 127.777  1.00  0.00           H
ATOM   2976  N   LEU C 422     116.634  82.772 126.665  1.00 30.00           N
ATOM   2977  CA  LEU C 422     116.482  83.064 125.240  1.00 30.00           C
ATOM   2978  C   LEU C 422     117.840  83.309 124.557  1.00 30.00           C
ATOM   2979  O   LEU C 422     118.003  82.910 123.407  1.00 30.00           O
ATOM   2980  CB  LEU C 422     115.537  84.273 125.065  1.00 20.00           C
ATOM   2981  CG  LEU C 422     114.064  83.983 125.439  1.00 20.00           C
ATOM   2982  CD1 LEU C 422     113.262  85.292 125.594  1.00 20.00           C
ATOM   2983  CD2 LEU C 422     113.393  83.002 124.455  1.00 20.00           C
ATOM   2984  H   LEU C 422     116.325  83.495 127.301  1.00  0.00           H
ATOM   2985  HA  LEU C 422     116.030  82.194 124.760  1.00  0.00           H
ATOM   2986  HB2 LEU C 422     115.913  85.093 125.681  1.00  0.00           H
ATOM   2987  HB3 LEU C 422     115.574  84.638 124.037  1.00  0.00           H
ATOM   2988  HG  LEU C 422     114.049  83.505 126.419  1.00  0.00           H
ATOM   2989 HD11 LEU C 422     112.354  85.309 124.992  1.00  0.00           H
ATOM   2990 HD12 LEU C 422     112.961  85.434 126.632  1.00  0.00           H
ATOM   2991 HD13 LEU C 422     113.845  86.168 125.308  1.00  0.00           H
ATOM   2992 HD21 LEU C 422     112.343  83.232 124.276  1.00  0.00           H
ATOM   2993 HD22 LEU C 422     113.880  83.008 123.483  1.00  0.00           H
ATOM   2994 HD23 LEU C 422     113.436  81.981 124.836  1.00  0.00           H
ATOM   2995  N   GLN C 423     118.802  83.909 125.283  1.00 30.00           N
ATOM   2996  CA  GLN C 423     120.170  84.125 124.808  1.00 30.00           C
ATOM   2997  C   GLN C 423     120.976  82.813 124.717  1.00 30.00           C
ATOM   2998  O   GLN C 423     121.722  82.644 123.753  1.00 30.00           O
ATOM   2999  CB  GLN C 423     120.858  85.174 125.711  1.00 20.00           C
ATOM   3000  CG  GLN C 423     122.251  85.648 125.239  1.00 20.00           C
ATOM   3001  CD  GLN C 423     122.235  86.310 123.855  1.00 20.00           C
ATOM   3002  NE2 GLN C 423     123.192  85.956 122.997  1.00 20.00           N
ATOM   3003  OE1 GLN C 423     121.378  87.143 123.566  1.00 20.00           O
ATOM   3004  H   GLN C 423     118.600  84.215 126.224  1.00  0.00           H
ATOM   3005  HA  GLN C 423     120.094  84.532 123.797  1.00  0.00           H
ATOM   3006  HB2 GLN C 423     120.207  86.046 125.802  1.00  0.00           H
ATOM   3007  HB3 GLN C 423     120.950  84.774 126.720  1.00  0.00           H
ATOM   3008  HG2 GLN C 423     122.955  84.815 125.242  1.00  0.00           H
ATOM   3009  HG3 GLN C 423     122.643  86.375 125.951  1.00  0.00           H
ATOM   3010 HE21 GLN C 423     123.239  86.371 122.080  1.00  0.00           H
ATOM   3011 HE22 GLN C 423     123.868  85.236 123.236  1.00  0.00           H
ATOM   3012  N   ASP C 424     120.775  81.902 125.690  1.00 30.00           N
ATOM   3013  CA  ASP C 424     121.335  80.545 125.720  1.00 30.00           C
ATOM   3014  C   ASP C 424     120.770  79.712 124.555  1.00 30.00           C
ATOM   3015  O   ASP C 424     121.566  79.207 123.766  1.00 30.00           O
ATOM   3016  CB  ASP C 424     121.148  79.887 127.115  1.00 20.00           C
ATOM   3017  CG  ASP C 424     121.777  78.500 127.357  1.00 20.00           C
ATOM   3018  OD1 ASP C 424     121.897  78.155 128.552  1.00 20.00           O
ATOM   3019  OD2 ASP C 424     122.051  77.757 126.389  1.00 20.00           O1-
ATOM   3020  H   ASP C 424     120.162  82.135 126.459  1.00  0.00           H
ATOM   3021  HA  ASP C 424     122.410  80.645 125.555  1.00  0.00           H
ATOM   3022  HB2 ASP C 424     121.589  80.561 127.849  1.00  0.00           H
ATOM   3023  HB3 ASP C 424     120.085  79.819 127.341  1.00  0.00           H
ATOM   3024  N   LYS C 425     119.429  79.659 124.417  1.00  0.00           N
ATOM   3025  CA  LYS C 425     118.726  79.030 123.290  1.00  0.00           C
ATOM   3026  C   LYS C 425     119.184  79.523 121.907  1.00  0.00           C
ATOM   3027  O   LYS C 425     119.235  78.711 120.985  1.00  0.00           O
ATOM   3028  CB  LYS C 425     117.195  79.200 123.428  1.00 20.00           C
ATOM   3029  CG  LYS C 425     116.540  78.190 124.382  1.00 20.00           C
ATOM   3030  CD  LYS C 425     115.004  78.232 124.350  1.00 20.00           C
ATOM   3031  CE  LYS C 425     114.360  77.221 125.314  1.00 20.00           C
ATOM   3032  NZ  LYS C 425     112.902  77.140 125.119  1.00 20.00           N1+
ATOM   3033  H   LYS C 425     118.843  80.115 125.104  1.00  0.00           H
ATOM   3034  HA  LYS C 425     118.963  77.965 123.336  1.00  0.00           H
ATOM   3035  HB2 LYS C 425     116.941  80.224 123.701  1.00  0.00           H
ATOM   3036  HB3 LYS C 425     116.738  79.034 122.451  1.00  0.00           H
ATOM   3037  HG2 LYS C 425     116.853  77.193 124.079  1.00  0.00           H
ATOM   3038  HG3 LYS C 425     116.904  78.345 125.398  1.00  0.00           H
ATOM   3039  HD2 LYS C 425     114.659  79.240 124.583  1.00  0.00           H
ATOM   3040  HD3 LYS C 425     114.665  78.022 123.335  1.00  0.00           H
ATOM   3041  HE2 LYS C 425     114.777  76.226 125.156  1.00  0.00           H
ATOM   3042  HE3 LYS C 425     114.567  77.498 126.347  1.00  0.00           H
ATOM   3043  HZ1 LYS C 425     112.706  76.809 124.178  1.00  0.00           H
```

ATOM	3044	HZ2	LYS	C	425	112.507	76.482	125.774	1.00	0.00	H
ATOM	3045	HZ3	LYS	C	425	112.480	78.047	125.246	1.00	0.00	H
ATOM	3046	N	TRP	C	426	119.530	80.819	121.798	1.00	0.00	N
ATOM	3047	CA	TRP	C	426	120.048	81.417	120.575	1.00	0.00	C
ATOM	3048	C	TRP	C	426	121.467	80.912	120.255	1.00	0.00	C
ATOM	3049	O	TRP	C	426	121.592	80.103	119.338	1.00	0.00	O
ATOM	3050	CB	TRP	C	426	119.870	82.949	120.588	1.00	20.00	C
ATOM	3051	CG	TRP	C	426	119.999	83.593	119.240	1.00	20.00	C
ATOM	3052	CD1	TRP	C	426	119.092	83.488	118.241	1.00	20.00	C
ATOM	3053	CD2	TRP	C	426	121.101	84.373	118.692	1.00	20.00	C
ATOM	3054	CE2	TRP	C	426	120.830	84.633	117.315	1.00	20.00	C
ATOM	3055	CE3	TRP	C	426	122.314	84.874	119.214	1.00	20.00	C
ATOM	3056	NE1	TRP	C	426	119.586	84.082	117.099	1.00	20.00	N
ATOM	3057	CZ2	TRP	C	426	121.739	85.312	116.488	1.00	20.00	C
ATOM	3058	CZ3	TRP	C	426	123.222	85.584	118.402	1.00	20.00	C
ATOM	3059	CH2	TRP	C	426	122.941	85.794	117.039	1.00	20.00	C
ATOM	3060	H	TRP	C	426	119.453	81.428	122.601	1.00	0.00	H
ATOM	3061	HA	TRP	C	426	119.411	81.059	119.773	1.00	0.00	H
ATOM	3062	HB2	TRP	C	426	118.867	83.189	120.940	1.00	0.00	H
ATOM	3063	HB3	TRP	C	426	120.556	83.420	121.294	1.00	0.00	H
ATOM	3064	HD1	TRP	C	426	118.144	82.980	118.329	1.00	0.00	H
ATOM	3065	HE1	TRP	C	426	119.081	84.083	116.223	1.00	0.00	H
ATOM	3066	HE3	TRP	C	426	122.540	84.709	120.255	1.00	0.00	H
ATOM	3067	HZ2	TRP	C	426	121.514	85.468	115.443	1.00	0.00	H
ATOM	3068	HZ3	TRP	C	426	124.144	85.950	118.828	1.00	0.00	H
ATOM	3069	HH2	TRP	C	426	123.645	86.327	116.417	1.00	0.00	H
ATOM	3070	N	ASP	C	427	122.483	81.338	121.031	1.00	30.00	N
ATOM	3071	CA	ASP	C	427	123.903	80.977	120.855	1.00	30.00	C
ATOM	3072	C	ASP	C	427	124.213	79.471	120.755	1.00	30.00	C
ATOM	3073	O	ASP	C	427	125.064	79.100	119.945	1.00	30.00	O
ATOM	3074	CB	ASP	C	427	124.833	81.610	121.921	1.00	20.00	C
ATOM	3075	CG	ASP	C	427	124.815	83.139	121.974	1.00	0.00	C
ATOM	3076	OD1	ASP	C	427	124.748	83.754	120.887	1.00	0.00	O
ATOM	3077	OD2	ASP	C	427	124.990	83.673	123.091	1.00	0.00	O1-
ATOM	3078	H	ASP	C	427	122.286	81.971	121.795	1.00	0.00	H
ATOM	3079	HA	ASP	C	427	124.183	81.395	119.887	1.00	0.00	H
ATOM	3080	HB2	ASP	C	427	124.522	81.241	122.900	1.00	0.00	H
ATOM	3081	HB3	ASP	C	427	125.866	81.293	121.776	1.00	0.00	H
ATOM	3082	N	ARG	C	428	123.535	78.638	121.563	1.00	30.00	N
ATOM	3083	CA	ARG	C	428	123.757	77.191	121.617	1.00	30.00	C
ATOM	3084	C	ARG	C	428	123.052	76.414	120.489	1.00	30.00	C
ATOM	3085	O	ARG	C	428	123.608	75.410	120.042	1.00	30.00	O
ATOM	3086	CB	ARG	C	428	123.342	76.675	123.013	1.00	20.00	C
ATOM	3087	CG	ARG	C	428	123.460	75.156	123.235	1.00	20.00	C
ATOM	3088	CD	ARG	C	428	123.270	74.778	124.708	1.00	20.00	C
ATOM	3089	NE	ARG	C	428	122.988	73.344	124.867	1.00	20.00	N
ATOM	3090	CZ	ARG	C	428	122.846	72.700	126.038	1.00	20.00	C
ATOM	3091	NH1	ARG	C	428	122.980	73.343	127.207	1.00	20.00	N
ATOM	3092	NH2	ARG	C	428	122.558	71.392	126.035	1.00	20.00	N1+
ATOM	3093	H	ARG	C	428	122.851	79.013	122.209	1.00	0.00	H
ATOM	3094	HA	ARG	C	428	124.829	77.011	121.513	1.00	0.00	H
ATOM	3095	HB2	ARG	C	428	123.926	77.200	123.770	1.00	0.00	H
ATOM	3096	HB3	ARG	C	428	122.300	76.933	123.194	1.00	0.00	H
ATOM	3097	HG2	ARG	C	428	122.800	74.577	122.587	1.00	0.00	H
ATOM	3098	HG3	ARG	C	428	124.480	74.886	122.959	1.00	0.00	H
ATOM	3099	HD2	ARG	C	428	124.228	74.918	125.210	1.00	0.00	H
ATOM	3100	HD3	ARG	C	428	122.559	75.425	125.220	1.00	0.00	H
ATOM	3101	HE	ARG	C	428	122.890	72.818	124.012	1.00	0.00	H
ATOM	3102	HH11	ARG	C	428	122.894	72.845	128.081	1.00	0.00	H
ATOM	3103	HH12	ARG	C	428	123.232	74.322	127.220	1.00	0.00	H
ATOM	3104	HH21	ARG	C	428	122.454	70.889	126.904	1.00	0.00	H
ATOM	3105	HH22	ARG	C	428	122.455	70.890	125.165	1.00	0.00	H
ATOM	3106	N	PHE	C	429	121.845	76.852	120.084	1.00	30.00	N
ATOM	3107	CA	PHE	C	429	120.940	76.057	119.254	1.00	30.00	C
ATOM	3108	C	PHE	C	429	120.467	76.795	117.990	1.00	30.00	C
ATOM	3109	O	PHE	C	429	120.991	76.499	116.915	1.00	30.00	O
ATOM	3110	CB	PHE	C	429	119.832	75.423	120.135	1.00	20.00	C
ATOM	3111	CG	PHE	C	429	118.709	74.698	119.411	1.00	20.00	C
ATOM	3112	CD1	PHE	C	429	118.955	73.459	118.782	1.00	20.00	C
ATOM	3113	CD2	PHE	C	429	117.444	75.305	119.259	1.00	20.00	C
ATOM	3114	CE1	PHE	C	429	117.944	72.829	118.069	1.00	20.00	C

```
ATOM   3115  CE2 PHE C 429     116.447  74.661 118.538  1.00 20.00           C
ATOM   3116  CZ  PHE C 429     116.695  73.427 117.950  1.00 20.00           C
ATOM   3117  H   PHE C 429     121.470  77.711 120.463  1.00  0.00           H
ATOM   3118  HA  PHE C 429     121.487  75.203 118.850  1.00  0.00           H
ATOM   3119  HB2 PHE C 429     120.303  74.690 120.792  1.00  0.00           H
ATOM   3120  HB3 PHE C 429     119.399  76.167 120.802  1.00  0.00           H
ATOM   3121  HD1 PHE C 429     119.930  73.000 118.853  1.00  0.00           H
ATOM   3122  HD2 PHE C 429     117.245  76.270 119.703  1.00  0.00           H
ATOM   3123  HE1 PHE C 429     118.131  71.877 117.595  1.00  0.00           H
ATOM   3124  HE2 PHE C 429     115.477  75.125 118.429  1.00  0.00           H
ATOM   3125  HZ  PHE C 429     115.913  72.938 117.389  1.00  0.00           H
ATOM   3126  N   VAL C 430     119.469  77.690 118.113  1.00 30.00           N
ATOM   3127  CA  VAL C 430     118.740  78.229 116.956  1.00 30.00           C
ATOM   3128  C   VAL C 430     119.542  79.193 116.046  1.00 30.00           C
ATOM   3129  O   VAL C 430     119.184  79.320 114.878  1.00 30.00           O
ATOM   3130  CB  VAL C 430     117.361  78.849 117.337  1.00 20.00           C
ATOM   3131  CG1 VAL C 430     117.458  80.090 118.224  1.00 20.00           C
ATOM   3132  CG2 VAL C 430     116.462  79.183 116.132  1.00 20.00           C
ATOM   3133  H   VAL C 430     119.104  77.923 119.027  1.00  0.00           H
ATOM   3134  HA  VAL C 430     118.511  77.356 116.347  1.00  0.00           H
ATOM   3135  HB  VAL C 430     116.817  78.093 117.906  1.00  0.00           H
ATOM   3136 HG11 VAL C 430     116.511  80.623 118.239  1.00  0.00           H
ATOM   3137 HG12 VAL C 430     117.692  79.812 119.248  1.00  0.00           H
ATOM   3138 HG13 VAL C 430     118.210  80.787 117.861  1.00  0.00           H
ATOM   3139 HG21 VAL C 430     115.469  79.485 116.460  1.00  0.00           H
ATOM   3140 HG22 VAL C 430     116.847  80.011 115.537  1.00  0.00           H
ATOM   3141 HG23 VAL C 430     116.347  78.317 115.482  1.00  0.00           H
ATOM   3142  N   LYS C 431     120.640  79.797 116.538  1.00  0.00           N
ATOM   3143  CA  LYS C 431     121.570  80.622 115.747  1.00  0.00           C
ATOM   3144  C   LYS C 431     122.168  79.882 114.530  1.00  0.00           C
ATOM   3145  O   LYS C 431     122.387  80.513 113.497  1.00  0.00           O
ATOM   3146  CB  LYS C 431     122.657  81.175 116.694  1.00 20.00           C
ATOM   3147  CG  LYS C 431     123.749  82.064 116.072  1.00 20.00           C
ATOM   3148  CD  LYS C 431     124.741  82.543 117.147  1.00 20.00           C
ATOM   3149  CE  LYS C 431     125.783  83.551 116.644  1.00 20.00           C
ATOM   3150  NZ  LYS C 431     126.694  82.953 115.656  1.00 20.00           N1+
ATOM   3151  H   LYS C 431     120.890  79.657 117.509  1.00  0.00           H
ATOM   3152  HA  LYS C 431     120.997  81.469 115.364  1.00  0.00           H
ATOM   3153  HB2 LYS C 431     122.162  81.763 117.467  1.00  0.00           H
ATOM   3154  HB3 LYS C 431     123.144  80.341 117.202  1.00  0.00           H
ATOM   3155  HG2 LYS C 431     124.291  81.519 115.299  1.00  0.00           H
ATOM   3156  HG3 LYS C 431     123.285  82.921 115.582  1.00  0.00           H
ATOM   3157  HD2 LYS C 431     124.190  82.990 117.974  1.00  0.00           H
ATOM   3158  HD3 LYS C 431     125.253  81.680 117.575  1.00  0.00           H
ATOM   3159  HE2 LYS C 431     125.296  84.418 116.200  1.00  0.00           H
ATOM   3160  HE3 LYS C 431     126.376  83.916 117.484  1.00  0.00           H
ATOM   3161  HZ1 LYS C 431     126.159  82.632 114.862  1.00  0.00           H
ATOM   3162  HZ2 LYS C 431     127.364  83.646 115.353  1.00  0.00           H
ATOM   3163  HZ3 LYS C 431     127.181  82.173 116.074  1.00  0.00           H
ATOM   3164  N   ARG C 432     122.371  78.559 114.665  1.00  0.00           N
ATOM   3165  CA  ARG C 432     122.825  77.661 113.605  1.00  0.00           C
ATOM   3166  C   ARG C 432     121.773  77.468 112.498  1.00  0.00           C
ATOM   3167  O   ARG C 432     122.090  77.711 111.333  1.00  0.00           O
ATOM   3168  CB  ARG C 432     123.231  76.301 114.211  1.00 20.00           C
ATOM   3169  CG  ARG C 432     124.406  76.384 115.204  1.00 20.00           C
ATOM   3170  CD  ARG C 432     124.813  75.015 115.774  1.00 20.00           C
ATOM   3171  NE  ARG C 432     125.397  74.133 114.751  1.00 20.00           N
ATOM   3172  CZ  ARG C 432     126.666  74.158 114.309  1.00 20.00           C
ATOM   3173  NH1 ARG C 432     127.567  75.012 114.817  1.00 20.00           N
ATOM   3174  NH2 ARG C 432     127.038  73.311 113.341  1.00 20.00           N1+
ATOM   3175  H   ARG C 432     122.139  78.119 115.546  1.00  0.00           H
ATOM   3176  HA  ARG C 432     123.711  78.107 113.149  1.00  0.00           H
ATOM   3177  HB2 ARG C 432     122.371  75.852 114.709  1.00  0.00           H
ATOM   3178  HB3 ARG C 432     123.501  75.619 113.404  1.00  0.00           H
ATOM   3179  HG2 ARG C 432     125.250  76.784 114.641  1.00  0.00           H
ATOM   3180  HG3 ARG C 432     124.228  77.088 116.018  1.00  0.00           H
ATOM   3181  HD2 ARG C 432     123.893  74.501 116.054  1.00  0.00           H
ATOM   3182  HD3 ARG C 432     125.400  75.085 116.690  1.00  0.00           H
ATOM   3183  HE  ARG C 432     124.750  73.500 114.303  1.00  0.00           H
ATOM   3184 HH11 ARG C 432     128.517  75.022 114.478  1.00  0.00           H
ATOM   3185 HH12 ARG C 432     127.295  75.651 115.550  1.00  0.00           H
```

```
ATOM   3186 HH21 ARG C 432     127.987  73.314 112.994  1.00  0.00           H
ATOM   3187 HH22 ARG C 432     126.375  72.661 112.945  1.00  0.00           H
ATOM   3188  N   ILE C 433     120.553  77.035 112.873  1.00  0.00           N
ATOM   3189  CA  ILE C 433     119.479  76.725 111.920  1.00  0.00           C
ATOM   3190  C   ILE C 433     118.847  77.987 111.287  1.00  0.00           C
ATOM   3191  O   ILE C 433     118.380  77.906 110.154  1.00  0.00           O
ATOM   3192  CB  ILE C 433     118.371  75.824 112.548  1.00 20.00           C
ATOM   3193  CG1 ILE C 433     117.524  75.094 111.478  1.00 20.00           C
ATOM   3194  CG2 ILE C 433     117.450  76.550 113.547  1.00 20.00           C
ATOM   3195  CD1 ILE C 433     116.669  73.947 112.041  1.00 20.00           C
ATOM   3196  H   ILE C 433     120.352  76.873 113.849  1.00  0.00           H
ATOM   3197  HA  ILE C 433     119.939  76.151 111.115  1.00  0.00           H
ATOM   3198  HB  ILE C 433     118.898  75.053 113.113  1.00  0.00           H
ATOM   3199 HG12 ILE C 433     116.870  75.803 110.970  1.00  0.00           H
ATOM   3200 HG13 ILE C 433     118.177  74.687 110.705  1.00  0.00           H
ATOM   3201 HG21 ILE C 433     116.839  75.849 114.114  1.00  0.00           H
ATOM   3202 HG22 ILE C 433     118.042  77.111 114.260  1.00  0.00           H
ATOM   3203 HG23 ILE C 433     116.774  77.249 113.054  1.00  0.00           H
ATOM   3204 HD11 ILE C 433     116.392  73.248 111.252  1.00  0.00           H
ATOM   3205 HD12 ILE C 433     117.203  73.383 112.806  1.00  0.00           H
ATOM   3206 HD13 ILE C 433     115.747  74.323 112.484  1.00  0.00           H
ATOM   3207  N   PHE C 434     118.883  79.129 111.996  1.00  0.00           N
ATOM   3208  CA  PHE C 434     118.412  80.427 111.510  1.00  0.00           C
ATOM   3209  C   PHE C 434     119.304  80.999 110.392  1.00  0.00           C
ATOM   3210  O   PHE C 434     118.770  81.527 109.416  1.00  0.00           O
ATOM   3211  CB  PHE C 434     118.248  81.397 112.700  1.00 20.00           C
ATOM   3212  CG  PHE C 434     117.706  82.771 112.350  1.00 20.00           C
ATOM   3213  CD1 PHE C 434     116.316  82.960 112.188  1.00 20.00           C
ATOM   3214  CD2 PHE C 434     118.585  83.819 112.006  1.00 20.00           C
ATOM   3215  CE1 PHE C 434     115.828  84.186 111.756  1.00 20.00           C
ATOM   3216  CE2 PHE C 434     118.080  85.028 111.552  1.00 20.00           C
ATOM   3217  CZ  PHE C 434     116.707  85.214 111.444  1.00 20.00           C
ATOM   3218  H   PHE C 434     119.265  79.119 112.934  1.00  0.00           H
ATOM   3219  HA  PHE C 434     117.421  80.271 111.080  1.00  0.00           H
ATOM   3220  HB2 PHE C 434     117.561  80.955 113.422  1.00  0.00           H
ATOM   3221  HB3 PHE C 434     119.200  81.515 113.221  1.00  0.00           H
ATOM   3222  HD1 PHE C 434     115.629  82.155 112.403  1.00  0.00           H
ATOM   3223  HD2 PHE C 434     119.652  83.679 112.084  1.00  0.00           H
ATOM   3224  HE1 PHE C 434     114.764  84.338 111.651  1.00  0.00           H
ATOM   3225  HE2 PHE C 434     118.757  85.825 111.285  1.00  0.00           H
ATOM   3226  HZ  PHE C 434     116.320  86.165 111.113  1.00  0.00           H
ATOM   3227  N   TYR C 435     120.635  80.847 110.532  1.00  0.00           N
ATOM   3228  CA  TYR C 435     121.616  81.191 109.495  1.00  0.00           C
ATOM   3229  C   TYR C 435     121.545  80.247 108.281  1.00  0.00           C
ATOM   3230  O   TYR C 435     121.795  80.703 107.166  1.00  0.00           O
ATOM   3231  CB  TYR C 435     123.043  81.208 110.087  1.00 20.00           C
ATOM   3232  CG  TYR C 435     123.415  82.322 111.061  1.00 20.00           C
ATOM   3233  CD1 TYR C 435     122.528  83.373 111.393  1.00 20.00           C
ATOM   3234  CD2 TYR C 435     124.703  82.308 111.635  1.00 20.00           C
ATOM   3235  CE1 TYR C 435     122.920  84.381 112.294  1.00 20.00           C
ATOM   3236  CE2 TYR C 435     125.103  83.325 112.522  1.00 20.00           C
ATOM   3237  CZ  TYR C 435     124.211  84.362 112.856  1.00 20.00           C
ATOM   3238  OH  TYR C 435     124.600  85.349 113.713  1.00 20.00           O
ATOM   3239  H   TYR C 435     121.005  80.414 111.367  1.00  0.00           H
ATOM   3240  HA  TYR C 435     121.383  82.189 109.121  1.00  0.00           H
ATOM   3241  HB2 TYR C 435     123.249  80.251 110.569  1.00  0.00           H
ATOM   3242  HB3 TYR C 435     123.761  81.287 109.268  1.00  0.00           H
ATOM   3243  HD1 TYR C 435     121.542  83.425 110.962  1.00  0.00           H
ATOM   3244  HD2 TYR C 435     125.396  81.517 111.386  1.00  0.00           H
ATOM   3245  HE1 TYR C 435     122.230  85.175 112.540  1.00  0.00           H
ATOM   3246  HE2 TYR C 435     126.100  83.312 112.936  1.00  0.00           H
ATOM   3247  HH  TYR C 435     123.941  86.041 113.812  1.00  0.00           H
ATOM   3248  N   PHE C 436     121.163  78.976 108.505  1.00  0.00           N
ATOM   3249  CA  PHE C 436     120.867  78.006 107.451  1.00  0.00           C
ATOM   3250  C   PHE C 436     119.617  78.370 106.628  1.00  0.00           C
ATOM   3251  O   PHE C 436     119.674  78.293 105.404  1.00  0.00           O
ATOM   3252  CB  PHE C 436     120.838  76.576 108.037  1.00 20.00           C
ATOM   3253  CG  PHE C 436     120.299  75.483 107.129  1.00 20.00           C
ATOM   3254  CD1 PHE C 436     121.113  74.945 106.111  1.00 20.00           C
ATOM   3255  CD2 PHE C 436     118.936  75.118 107.185  1.00 20.00           C
ATOM   3256  CE1 PHE C 436     120.587  74.020 105.219  1.00 20.00           C
```

```
ATOM   3257  CE2 PHE C 436     118.429  74.193 106.282  1.00 20.00           C
ATOM   3258  CZ  PHE C 436     119.253  73.642 105.309  1.00 20.00           C
ATOM   3259  H   PHE C 436     120.995  78.669 109.453  1.00  0.00           H
ATOM   3260  HA  PHE C 436     121.705  78.030 106.750  1.00  0.00           H
ATOM   3261  HB2 PHE C 436     121.852  76.297 108.327  1.00  0.00           H
ATOM   3262  HB3 PHE C 436     120.257  76.565 108.955  1.00  0.00           H
ATOM   3263  HD1 PHE C 436     122.145  75.251 106.020  1.00  0.00           H
ATOM   3264  HD2 PHE C 436     118.283  75.559 107.925  1.00  0.00           H
ATOM   3265  HE1 PHE C 436     121.214  73.602 104.446  1.00  0.00           H
ATOM   3266  HE2 PHE C 436     117.388  73.910 106.332  1.00  0.00           H
ATOM   3267  HZ  PHE C 436     118.849  72.927 104.607  1.00  0.00           H
ATOM   3268  N   ASN C 437     118.534  78.796 107.304  1.00 30.00           N
ATOM   3269  CA  ASN C 437     117.289  79.258 106.676  1.00 30.00           C
ATOM   3270  C   ASN C 437     117.469  80.574 105.898  1.00 30.00           C
ATOM   3271  O   ASN C 437     116.810  80.742 104.874  1.00 30.00           O
ATOM   3272  CB  ASN C 437     116.179  79.408 107.744  1.00 20.00           C
ATOM   3273  CG  ASN C 437     115.694  78.082 108.346  1.00 20.00           C
ATOM   3274  ND2 ASN C 437     115.222  78.124 109.593  1.00 20.00           N
ATOM   3275  OD1 ASN C 437     115.720  77.039 107.696  1.00 20.00           O
ATOM   3276  H   ASN C 437     118.555  78.806 108.315  1.00  0.00           H
ATOM   3277  HA  ASN C 437     116.999  78.479 105.968  1.00  0.00           H
ATOM   3278  HB2 ASN C 437     116.510  80.078 108.539  1.00  0.00           H
ATOM   3279  HB3 ASN C 437     115.302  79.877 107.294  1.00  0.00           H
ATOM   3280 HD21 ASN C 437     114.891  77.278 110.033  1.00  0.00           H
ATOM   3281 HD22 ASN C 437     115.213  78.990 110.111  1.00  0.00           H
ATOM   3282  N   PHE C 438     118.372  81.456 106.365  1.00  0.00           N
ATOM   3283  CA  PHE C 438     118.775  82.677 105.666  1.00  0.00           C
ATOM   3284  C   PHE C 438     119.590  82.384 104.392  1.00  0.00           C
ATOM   3285  O   PHE C 438     119.334  83.004 103.362  1.00  0.00           O
ATOM   3286  CB  PHE C 438     119.527  83.606 106.646  1.00 20.00           C
ATOM   3287  CG  PHE C 438     120.078  84.913 106.090  1.00 20.00           C
ATOM   3288  CD1 PHE C 438     119.329  85.694 105.180  1.00 20.00           C
ATOM   3289  CD2 PHE C 438     121.270  85.450 106.622  1.00 20.00           C
ATOM   3290  CE1 PHE C 438     119.798  86.936 104.775  1.00 20.00           C
ATOM   3291  CE2 PHE C 438     121.724  86.693 106.201  1.00 20.00           C
ATOM   3292  CZ  PHE C 438     120.995  87.430 105.277  1.00 20.00           C
ATOM   3293  H   PHE C 438     118.852  81.259 107.233  1.00  0.00           H
ATOM   3294  HA  PHE C 438     117.860  83.186 105.355  1.00  0.00           H
ATOM   3295  HB2 PHE C 438     118.876  83.859 107.481  1.00  0.00           H
ATOM   3296  HB3 PHE C 438     120.356  83.049 107.083  1.00  0.00           H
ATOM   3297  HD1 PHE C 438     118.384  85.341 104.795  1.00  0.00           H
ATOM   3298  HD2 PHE C 438     121.834  84.898 107.359  1.00  0.00           H
ATOM   3299  HE1 PHE C 438     119.221  87.526 104.080  1.00  0.00           H
ATOM   3300  HE2 PHE C 438     122.643  87.094 106.604  1.00  0.00           H
ATOM   3301  HZ  PHE C 438     121.351  88.399 104.960  1.00  0.00           H
ATOM   3302  N   PHE C 439     120.526  81.422 104.483  1.00  0.00           N
ATOM   3303  CA  PHE C 439     121.339  80.925 103.371  1.00  0.00           C
ATOM   3304  C   PHE C 439     120.507  80.237 102.274  1.00  0.00           C
ATOM   3305  O   PHE C 439     120.721  80.511 101.094  1.00  0.00           O
ATOM   3306  CB  PHE C 439     122.472  80.035 103.933  1.00 20.00           C
ATOM   3307  CG  PHE C 439     123.343  79.321 102.914  1.00 20.00           C
ATOM   3308  CD1 PHE C 439     124.375  80.020 102.252  1.00 20.00           C
ATOM   3309  CD2 PHE C 439     123.036  78.004 102.507  1.00 20.00           C
ATOM   3310  CE1 PHE C 439     125.117  79.389 101.262  1.00 20.00           C
ATOM   3311  CE2 PHE C 439     123.788  77.393 101.513  1.00 20.00           C
ATOM   3312  CZ  PHE C 439     124.828  78.080 100.899  1.00 20.00           C
ATOM   3313  H   PHE C 439     120.684  80.974 105.376  1.00  0.00           H
ATOM   3314  HA  PHE C 439     121.811  81.793 102.906  1.00  0.00           H
ATOM   3315  HB2 PHE C 439     123.128  80.655 104.546  1.00  0.00           H
ATOM   3316  HB3 PHE C 439     122.057  79.289 104.611  1.00  0.00           H
ATOM   3317  HD1 PHE C 439     124.593  81.045 102.515  1.00  0.00           H
ATOM   3318  HD2 PHE C 439     122.218  77.470 102.967  1.00  0.00           H
ATOM   3319  HE1 PHE C 439     125.914  79.922 100.766  1.00  0.00           H
ATOM   3320  HE2 PHE C 439     123.558  76.382 101.210  1.00  0.00           H
ATOM   3321  HZ  PHE C 439     125.405  77.598 100.124  1.00  0.00           H
ATOM   3322  N   VAL C 440     119.545  79.396 102.691  1.00  0.00           N
ATOM   3323  CA  VAL C 440     118.566  78.737 101.826  1.00  0.00           C
ATOM   3324  C   VAL C 440     117.618  79.738 101.134  1.00  0.00           C
ATOM   3325  O   VAL C 440     117.317  79.540  99.957  1.00  0.00           O
ATOM   3326  CB  VAL C 440     117.749  77.668 102.616  1.00 20.00           C
ATOM   3327  CG1 VAL C 440     116.416  77.213 101.981  1.00 20.00           C
```

```
ATOM   3328  CG2 VAL C 440     118.617  76.428 102.897  1.00 20.00           C
ATOM   3329  H   VAL C 440     119.454  79.205 103.681  1.00  0.00           H
ATOM   3330  HA  VAL C 440     119.124  78.227 101.038  1.00  0.00           H
ATOM   3331  HB  VAL C 440     117.495  78.100 103.586  1.00  0.00           H
ATOM   3332 HG11 VAL C 440     115.971  76.396 102.550  1.00  0.00           H
ATOM   3333 HG12 VAL C 440     115.675  78.013 101.960  1.00  0.00           H
ATOM   3334 HG13 VAL C 440     116.563  76.859 100.960  1.00  0.00           H
ATOM   3335 HG21 VAL C 440     118.098  75.726 103.549  1.00  0.00           H
ATOM   3336 HG22 VAL C 440     118.864  75.903 101.973  1.00  0.00           H
ATOM   3337 HG23 VAL C 440     119.557  76.687 103.382  1.00  0.00           H
ATOM   3338  N   TYR C 441     117.197  80.800 101.848  1.00  0.00           N
ATOM   3339  CA  TYR C 441     116.347  81.855 101.292  1.00  0.00           C
ATOM   3340  C   TYR C 441     117.086  82.760 100.290  1.00  0.00           C
ATOM   3341  O   TYR C 441     116.482  83.171  99.300  1.00  0.00           O
ATOM   3342  CB  TYR C 441     115.686  82.682 102.411  1.00 20.00           C
ATOM   3343  CG  TYR C 441     114.607  83.611 101.886  1.00 20.00           C
ATOM   3344  CD1 TYR C 441     113.391  83.057 101.439  1.00 20.00           C
ATOM   3345  CD2 TYR C 441     114.827  85.002 101.778  1.00 20.00           C
ATOM   3346  CE1 TYR C 441     112.397  83.881 100.886  1.00 20.00           C
ATOM   3347  CE2 TYR C 441     113.829  85.829 101.225  1.00 20.00           C
ATOM   3348  CZ  TYR C 441     112.617  85.265 100.780  1.00 20.00           C
ATOM   3349  OH  TYR C 441     111.648  86.046 100.234  1.00 20.00           O
ATOM   3350  H   TYR C 441     117.467  80.897 102.818  1.00  0.00           H
ATOM   3351  HA  TYR C 441     115.545  81.354 100.751  1.00  0.00           H
ATOM   3352  HB2 TYR C 441     115.211  82.011 103.128  1.00  0.00           H
ATOM   3353  HB3 TYR C 441     116.433  83.246 102.973  1.00  0.00           H
ATOM   3354  HD1 TYR C 441     113.231  81.991 101.499  1.00  0.00           H
ATOM   3355  HD2 TYR C 441     115.761  85.433 102.104  1.00  0.00           H
ATOM   3356  HE1 TYR C 441     111.470  83.454 100.537  1.00  0.00           H
ATOM   3357  HE2 TYR C 441     114.000  86.892 101.136  1.00  0.00           H
ATOM   3358  HH  TYR C 441     111.941  86.951 100.078  1.00  0.00           H
ATOM   3359  N   CYS C 442     118.379  83.023 100.546  1.00  0.00           N
ATOM   3360  CA  CYS C 442     119.284  83.748  99.655  1.00  0.00           C
ATOM   3361  C   CYS C 442     119.445  83.020  98.306  1.00  0.00           C
ATOM   3362  O   CYS C 442     119.307  83.653  97.261  1.00  0.00           O
ATOM   3363  CB  CYS C 442     120.638  84.020 100.347  1.00 20.00           C
ATOM   3364  SG  CYS C 442     121.781  84.983  99.313  1.00 20.00           S
ATOM   3365  H   CYS C 442     118.791  82.671 101.401  1.00  0.00           H
ATOM   3366  HA  CYS C 442     118.820  84.715  99.451  1.00  0.00           H
ATOM   3367  HB2 CYS C 442     120.480  84.568 101.276  1.00  0.00           H
ATOM   3368  HB3 CYS C 442     121.128  83.085 100.619  1.00  0.00           H
ATOM   3369  HG  CYS C 442     121.965  84.038  98.386  1.00  0.00           H
ATOM   3370  N   LEU C 443     119.674  81.696  98.368  1.00  0.00           N
ATOM   3371  CA  LEU C 443     119.781  80.806  97.211  1.00  0.00           C
ATOM   3372  C   LEU C 443     118.446  80.650  96.458  1.00  0.00           C
ATOM   3373  O   LEU C 443     118.473  80.596  95.230  1.00  0.00           O
ATOM   3374  CB  LEU C 443     120.342  79.434  97.656  1.00 20.00           C
ATOM   3375  CG  LEU C 443     121.880  79.286  97.567  1.00 20.00           C
ATOM   3376  CD1 LEU C 443     122.664  80.241  98.489  1.00 20.00           C
ATOM   3377  CD2 LEU C 443     122.314  77.821  97.780  1.00 20.00           C
ATOM   3378  H   LEU C 443     119.772  81.255  99.272  1.00  0.00           H
ATOM   3379  HA  LEU C 443     120.486  81.257  96.509  1.00  0.00           H
ATOM   3380  HB2 LEU C 443     119.994  79.188  98.661  1.00  0.00           H
ATOM   3381  HB3 LEU C 443     119.919  78.659  97.013  1.00  0.00           H
ATOM   3382  HG  LEU C 443     122.155  79.548  96.545  1.00  0.00           H
ATOM   3383 HD11 LEU C 443     123.651  80.450  98.075  1.00  0.00           H
ATOM   3384 HD12 LEU C 443     122.176  81.202  98.628  1.00  0.00           H
ATOM   3385 HD13 LEU C 443     122.810  79.812  99.480  1.00  0.00           H
ATOM   3386 HD21 LEU C 443     123.067  77.720  98.561  1.00  0.00           H
ATOM   3387 HD22 LEU C 443     121.477  77.181  98.061  1.00  0.00           H
ATOM   3388 HD23 LEU C 443     122.744  77.409  96.867  1.00  0.00           H
ATOM   3389  N   TYR C 444     117.314  80.615  97.185  1.00  0.00           N
ATOM   3390  CA  TYR C 444     115.960  80.573  96.624  1.00  0.00           C
ATOM   3391  C   TYR C 444     115.631  81.804  95.761  1.00  0.00           C
ATOM   3392  O   TYR C 444     115.136  81.633  94.647  1.00  0.00           O
ATOM   3393  CB  TYR C 444     114.926  80.351  97.753  1.00 20.00           C
ATOM   3394  CG  TYR C 444     113.473  80.569  97.360  1.00 20.00           C
ATOM   3395  CD1 TYR C 444     112.800  79.601  96.588  1.00 20.00           C
ATOM   3396  CD2 TYR C 444     112.809  81.761  97.721  1.00 20.00           C
ATOM   3397  CE1 TYR C 444     111.479  79.835  96.164  1.00 20.00           C
ATOM   3398  CE2 TYR C 444     111.489  81.993  97.291  1.00 20.00           C
```

```
ATOM   3399  CZ  TYR C 444     110.824  81.034  96.504  1.00 20.00           C
ATOM   3400  OH  TYR C 444     109.549  81.267  96.076  1.00 20.00           O
ATOM   3401  H   TYR C 444     117.375  80.635  98.195  1.00  0.00           H
ATOM   3402  HA  TYR C 444     115.919  79.700  95.969  1.00  0.00           H
ATOM   3403  HB2 TYR C 444     115.037  79.349  98.169  1.00  0.00           H
ATOM   3404  HB3 TYR C 444     115.140  81.036  98.570  1.00  0.00           H
ATOM   3405  HD1 TYR C 444     113.298  78.684  96.308  1.00  0.00           H
ATOM   3406  HD2 TYR C 444     113.318  82.514  98.306  1.00  0.00           H
ATOM   3407  HE1 TYR C 444     110.966  79.081  95.589  1.00  0.00           H
ATOM   3408  HE2 TYR C 444     110.993  82.913  97.559  1.00  0.00           H
ATOM   3409  HH  TYR C 444     109.231  80.609  95.449  1.00  0.00           H
ATOM   3410  N   MET C 445     115.929  83.007  96.282  1.00  0.00           N
ATOM   3411  CA  MET C 445     115.715  84.272  95.578  1.00  0.00           C
ATOM   3412  C   MET C 445     116.654  84.476  94.378  1.00  0.00           C
ATOM   3413  O   MET C 445     116.227  85.106  93.414  1.00  0.00           O
ATOM   3414  CB  MET C 445     115.789  85.459  96.561  1.00 20.00           C
ATOM   3415  CG  MET C 445     114.600  85.560  97.536  1.00 20.00           C
ATOM   3416  SD  MET C 445     112.935  85.580  96.799  1.00 20.00           S
ATOM   3417  CE  MET C 445     113.124  86.869  95.537  1.00 20.00           C
ATOM   3418  H   MET C 445     116.324  83.069  97.211  1.00  0.00           H
ATOM   3419  HA  MET C 445     114.711  84.237  95.154  1.00  0.00           H
ATOM   3420  HB2 MET C 445     116.716  85.400  97.134  1.00  0.00           H
ATOM   3421  HB3 MET C 445     115.854  86.396  96.010  1.00  0.00           H
ATOM   3422  HG2 MET C 445     114.707  86.466  98.132  1.00  0.00           H
ATOM   3423  HG3 MET C 445     114.630  84.732  98.243  1.00  0.00           H
ATOM   3424  HE1 MET C 445     112.152  87.143  95.127  1.00  0.00           H
ATOM   3425  HE2 MET C 445     113.582  87.758  95.967  1.00  0.00           H
ATOM   3426  HE3 MET C 445     113.752  86.521  94.717  1.00  0.00           H
ATOM   3427  N   ILE C 446     117.876  83.915  94.429  1.00  0.00           N
ATOM   3428  CA  ILE C 446     118.830  83.894  93.315  1.00  0.00           C
ATOM   3429  C   ILE C 446     118.380  82.966  92.165  1.00  0.00           C
ATOM   3430  O   ILE C 446     118.518  83.347  91.002  1.00  0.00           O
ATOM   3431  CB  ILE C 446     120.264  83.515  93.804  1.00 20.00           C
ATOM   3432  CG1 ILE C 446     120.877  84.691  94.601  1.00 20.00           C
ATOM   3433  CG2 ILE C 446     121.253  83.054  92.706  1.00 20.00           C
ATOM   3434  CD1 ILE C 446     122.080  84.304  95.473  1.00 20.00           C
ATOM   3435  H   ILE C 446     118.162  83.428  95.268  1.00  0.00           H
ATOM   3436  HA  ILE C 446     118.877  84.906  92.908  1.00  0.00           H
ATOM   3437  HB  ILE C 446     120.155  82.679  94.496  1.00  0.00           H
ATOM   3438 HG12 ILE C 446     121.170  85.483  93.911  1.00  0.00           H
ATOM   3439 HG13 ILE C 446     120.124  85.136  95.249  1.00  0.00           H
ATOM   3440 HG21 ILE C 446     122.242  82.855  93.118  1.00  0.00           H
ATOM   3441 HG22 ILE C 446     120.937  82.127  92.227  1.00  0.00           H
ATOM   3442 HG23 ILE C 446     121.365  83.813  91.932  1.00  0.00           H
ATOM   3443 HD11 ILE C 446     122.238  85.045  96.256  1.00  0.00           H
ATOM   3444 HD12 ILE C 446     121.933  83.339  95.959  1.00  0.00           H
ATOM   3445 HD13 ILE C 446     122.996  84.251  94.886  1.00  0.00           H
ATOM   3446  N   ILE C 447     117.824  81.790  92.505  1.00  0.00           N
ATOM   3447  CA  ILE C 447     117.258  80.828  91.554  1.00  0.00           C
ATOM   3448  C   ILE C 447     115.955  81.331  90.898  1.00  0.00           C
ATOM   3449  O   ILE C 447     115.779  81.134  89.695  1.00  0.00           O
ATOM   3450  CB  ILE C 447     117.041  79.435  92.228  1.00 20.00           C
ATOM   3451  CG1 ILE C 447     118.389  78.711  92.465  1.00 20.00           C
ATOM   3452  CG2 ILE C 447     116.032  78.490  91.538  1.00 20.00           C
ATOM   3453  CD1 ILE C 447     119.162  78.311  91.197  1.00 20.00           C
ATOM   3454  H   ILE C 447     117.764  81.535  93.482  1.00  0.00           H
ATOM   3455  HA  ILE C 447     117.980  80.712  90.745  1.00  0.00           H
ATOM   3456  HB  ILE C 447     116.630  79.624  93.221  1.00  0.00           H
ATOM   3457 HG12 ILE C 447     118.221  77.825  93.079  1.00  0.00           H
ATOM   3458 HG13 ILE C 447     119.041  79.351  93.059  1.00  0.00           H
ATOM   3459 HG21 ILE C 447     116.048  77.498  91.989  1.00  0.00           H
ATOM   3460 HG22 ILE C 447     115.008  78.855  91.624  1.00  0.00           H
ATOM   3461 HG23 ILE C 447     116.257  78.378  90.479  1.00  0.00           H
ATOM   3462 HD11 ILE C 447     119.804  77.453  91.394  1.00  0.00           H
ATOM   3463 HD12 ILE C 447     118.506  78.043  90.371  1.00  0.00           H
ATOM   3464 HD13 ILE C 447     119.804  79.125  90.859  1.00  0.00           H
ATOM   3465  N   PHE C 448     115.097  82.002  91.684  1.00  0.00           N
ATOM   3466  CA  PHE C 448     113.875  82.657  91.214  1.00  0.00           C
ATOM   3467  C   PHE C 448     114.161  83.868  90.302  1.00  0.00           C
ATOM   3468  O   PHE C 448     113.472  84.035  89.295  1.00  0.00           O
ATOM   3469  CB  PHE C 448     112.989  82.999  92.433  1.00 20.00           C
```

ATOM	3470	CG	PHE	C	448	111.693	83.760	92.185	1.00	20.00	C
ATOM	3471	CD1	PHE	C	448	110.912	83.539	91.027	1.00	20.00	C
ATOM	3472	CD2	PHE	C	448	111.157	84.556	93.219	1.00	20.00	C
ATOM	3473	CE1	PHE	C	448	109.698	84.188	90.870	1.00	20.00	C
ATOM	3474	CE2	PHE	C	448	109.927	85.176	93.054	1.00	20.00	C
ATOM	3475	CZ	PHE	C	448	109.213	85.012	91.875	1.00	20.00	C
ATOM	3476	H	PHE	C	448	115.300	82.096	92.671	1.00	0.00	H
ATOM	3477	HA	PHE	C	448	113.338	81.926	90.607	1.00	0.00	H
ATOM	3478	HB2	PHE	C	448	112.725	82.076	92.951	1.00	0.00	H
ATOM	3479	HB3	PHE	C	448	113.583	83.574	93.144	1.00	0.00	H
ATOM	3480	HD1	PHE	C	448	111.252	82.874	90.248	1.00	0.00	H
ATOM	3481	HD2	PHE	C	448	111.697	84.680	94.146	1.00	0.00	H
ATOM	3482	HE1	PHE	C	448	109.117	84.031	89.974	1.00	0.00	H
ATOM	3483	HE2	PHE	C	448	109.520	85.781	93.850	1.00	0.00	H
ATOM	3484	HZ	PHE	C	448	108.262	85.502	91.747	1.00	0.00	H
ATOM	3485	N	THR	C	449	115.211	84.641	90.632	1.00	30.00	N
ATOM	3486	CA	THR	C	449	115.742	85.730	89.810	1.00	30.00	C
ATOM	3487	C	THR	C	449	116.243	85.227	88.446	1.00	30.00	C
ATOM	3488	O	THR	C	449	115.878	85.827	87.439	1.00	30.00	O
ATOM	3489	CB	THR	C	449	116.899	86.471	90.538	1.00	20.00	C
ATOM	3490	CG2	THR	C	449	117.856	87.334	89.690	1.00	20.00	C
ATOM	3491	OG1	THR	C	449	116.322	87.279	91.539	1.00	20.00	O
ATOM	3492	H	THR	C	449	115.721	84.442	91.482	1.00	0.00	H
ATOM	3493	HA	THR	C	449	114.930	86.436	89.626	1.00	0.00	H
ATOM	3494	HB	THR	C	449	117.513	85.742	91.064	1.00	0.00	H
ATOM	3495	HG1	THR	C	449	115.978	86.695	92.223	1.00	0.00	H
ATOM	3496	HG21	THR	C	449	118.475	87.973	90.320	1.00	0.00	H
ATOM	3497	HG22	THR	C	449	118.535	86.714	89.103	1.00	0.00	H
ATOM	3498	HG23	THR	C	449	117.317	87.973	88.993	1.00	0.00	H
ATOM	3499	N	ALA	C	450	117.022	84.131	88.433	1.00	30.00	N
ATOM	3500	CA	ALA	C	450	117.559	83.510	87.221	1.00	30.00	C
ATOM	3501	C	ALA	C	450	116.473	82.939	86.291	1.00	30.00	C
ATOM	3502	O	ALA	C	450	116.539	83.176	85.086	1.00	30.00	O
ATOM	3503	CB	ALA	C	450	118.575	82.428	87.615	1.00	30.00	C
ATOM	3504	H	ALA	C	450	117.280	83.693	89.307	1.00	0.00	H
ATOM	3505	HA	ALA	C	450	118.094	84.283	86.666	1.00	0.00	H
ATOM	3506	HB1	ALA	C	450	119.011	81.955	86.735	1.00	0.00	H
ATOM	3507	HB2	ALA	C	450	119.393	82.855	88.196	1.00	0.00	H
ATOM	3508	HB3	ALA	C	450	118.115	81.645	88.220	1.00	0.00	H
ATOM	3509	N	ALA	C	451	115.482	82.236	86.867	1.00	30.00	N
ATOM	3510	CA	ALA	C	451	114.351	81.643	86.148	1.00	30.00	C
ATOM	3511	C	ALA	C	451	113.426	82.680	85.485	1.00	30.00	C
ATOM	3512	O	ALA	C	451	112.943	82.430	84.380	1.00	30.00	O
ATOM	3513	CB	ALA	C	451	113.561	80.745	87.113	1.00	30.00	C
ATOM	3514	H	ALA	C	451	115.502	82.080	87.867	1.00	0.00	H
ATOM	3515	HA	ALA	C	451	114.757	81.010	85.357	1.00	0.00	H
ATOM	3516	HB1	ALA	C	451	112.730	80.251	86.607	1.00	0.00	H
ATOM	3517	HB2	ALA	C	451	114.201	79.963	87.523	1.00	0.00	H
ATOM	3518	HB3	ALA	C	451	113.154	81.312	87.951	1.00	0.00	H
ATOM	3519	N	ALA	C	452	113.223	83.826	86.158	1.00	30.00	N
ATOM	3520	CA	ALA	C	452	112.423	84.946	85.666	1.00	30.00	C
ATOM	3521	C	ALA	C	452	113.183	85.830	84.660	1.00	30.00	C
ATOM	3522	O	ALA	C	452	112.559	86.314	83.717	1.00	30.00	O
ATOM	3523	CB	ALA	C	452	111.932	85.769	86.866	1.00	30.00	C
ATOM	3524	H	ALA	C	452	113.645	83.948	87.069	1.00	0.00	H
ATOM	3525	HA	ALA	C	452	111.543	84.545	85.159	1.00	0.00	H
ATOM	3526	HB1	ALA	C	452	111.251	86.559	86.549	1.00	0.00	H
ATOM	3527	HB2	ALA	C	452	111.387	85.143	87.574	1.00	0.00	H
ATOM	3528	HB3	ALA	C	452	112.762	86.230	87.404	1.00	0.00	H
ATOM	3529	N	TYR	C	453	114.501	86.020	84.865	1.00	30.00	N
ATOM	3530	CA	TYR	C	453	115.397	86.800	83.999	1.00	30.00	C
ATOM	3531	C	TYR	C	453	115.594	86.153	82.617	1.00	30.00	C
ATOM	3532	O	TYR	C	453	115.616	86.873	81.620	1.00	30.00	O
ATOM	3533	CB	TYR	C	453	116.738	87.038	84.735	1.00	20.00	C
ATOM	3534	CG	TYR	C	453	117.789	87.868	84.011	1.00	20.00	C
ATOM	3535	CD1	TYR	C	453	117.846	89.263	84.206	1.00	20.00	C
ATOM	3536	CD2	TYR	C	453	118.730	87.250	83.162	1.00	20.00	C
ATOM	3537	CE1	TYR	C	453	118.825	90.031	83.546	1.00	20.00	C
ATOM	3538	CE2	TYR	C	453	119.708	88.018	82.500	1.00	20.00	C
ATOM	3539	CZ	TYR	C	453	119.755	89.413	82.691	1.00	20.00	C
ATOM	3540	OH	TYR	C	453	120.697	90.168	82.053	1.00	20.00	O

ATOM	3541	H	TYR	C	453	114.933	85.609	85.683	1.00	0.00	H
ATOM	3542	HA	TYR	C	453	114.938	87.775	83.833	1.00	0.00	H
ATOM	3543	HB2	TYR	C	453	116.536	87.543	85.680	1.00	0.00	H
ATOM	3544	HB3	TYR	C	453	117.188	86.080	84.998	1.00	0.00	H
ATOM	3545	HD1	TYR	C	453	117.138	89.747	84.862	1.00	0.00	H
ATOM	3546	HD2	TYR	C	453	118.696	86.182	83.013	1.00	0.00	H
ATOM	3547	HE1	TYR	C	453	118.860	91.100	83.689	1.00	0.00	H
ATOM	3548	HE2	TYR	C	453	120.420	87.535	81.848	1.00	0.00	H
ATOM	3549	HH	TYR	C	453	121.268	89.647	81.484	1.00	0.00	H
ATOM	3550	N	TYR	C	454	115.696	84.813	82.590	1.00	30.00	N
ATOM	3551	CA	TYR	C	454	115.828	84.007	81.377	1.00	30.00	C
ATOM	3552	C	TYR	C	454	114.485	83.380	80.955	1.00	30.00	C
ATOM	3553	O	TYR	C	454	114.478	82.257	80.450	1.00	30.00	O
ATOM	3554	CB	TYR	C	454	116.945	82.953	81.575	1.00	20.00	C
ATOM	3555	CG	TYR	C	454	118.344	83.521	81.745	1.00	20.00	C
ATOM	3556	CD1	TYR	C	454	118.931	84.272	80.704	1.00	20.00	C
ATOM	3557	CD2	TYR	C	454	119.076	83.286	82.928	1.00	20.00	C
ATOM	3558	CE1	TYR	C	454	120.231	84.791	80.848	1.00	20.00	C
ATOM	3559	CE2	TYR	C	454	120.375	83.810	83.075	1.00	20.00	C
ATOM	3560	CZ	TYR	C	454	120.953	84.565	82.036	1.00	20.00	C
ATOM	3561	OH	TYR	C	454	122.207	85.083	82.178	1.00	20.00	O
ATOM	3562	H	TYR	C	454	115.677	84.299	83.461	1.00	0.00	H
ATOM	3563	HA	TYR	C	454	116.121	84.645	80.543	1.00	0.00	H
ATOM	3564	HB2	TYR	C	454	116.702	82.308	82.421	1.00	0.00	H
ATOM	3565	HB3	TYR	C	454	116.998	82.296	80.706	1.00	0.00	H
ATOM	3566	HD1	TYR	C	454	118.385	84.451	79.789	1.00	0.00	H
ATOM	3567	HD2	TYR	C	454	118.650	82.699	83.728	1.00	0.00	H
ATOM	3568	HE1	TYR	C	454	120.671	85.368	80.048	1.00	0.00	H
ATOM	3569	HE2	TYR	C	454	120.924	83.628	83.988	1.00	0.00	H
ATOM	3570	HH	TYR	C	454	122.611	84.868	83.022	1.00	0.00	H
ATOM	3571	N	ARG	C	455	113.368	84.114	81.123	1.00	30.00	N
ATOM	3572	CA	ARG	C	455	112.067	83.728	80.567	1.00	30.00	C
ATOM	3573	C	ARG	C	455	112.036	83.958	79.035	1.00	30.00	C
ATOM	3574	O	ARG	C	455	112.663	84.911	78.567	1.00	30.00	O
ATOM	3575	CB	ARG	C	455	110.929	84.483	81.297	1.00	20.00	C
ATOM	3576	CG	ARG	C	455	110.744	85.969	80.931	1.00	20.00	C
ATOM	3577	CD	ARG	C	455	109.547	86.597	81.657	1.00	20.00	C
ATOM	3578	NE	ARG	C	455	109.241	87.935	81.142	1.00	20.00	N
ATOM	3579	CZ	ARG	C	455	108.632	88.238	79.984	1.00	20.00	C
ATOM	3580	NH1	ARG	C	455	108.211	87.285	79.139	1.00	20.00	N
ATOM	3581	NH2	ARG	C	455	108.444	89.527	79.673	1.00	20.00	N1+
ATOM	3582	H	ARG	C	455	113.428	85.031	81.543	1.00	0.00	H
ATOM	3583	HA	ARG	C	455	111.945	82.669	80.790	1.00	0.00	H
ATOM	3584	HB2	ARG	C	455	111.050	84.376	82.376	1.00	0.00	H
ATOM	3585	HB3	ARG	C	455	109.994	83.974	81.060	1.00	0.00	H
ATOM	3586	HG2	ARG	C	455	111.651	86.485	81.245	1.00	0.00	H
ATOM	3587	HG3	ARG	C	455	110.655	86.146	79.859	1.00	0.00	H
ATOM	3588	HD2	ARG	C	455	108.677	85.942	81.721	1.00	0.00	H
ATOM	3589	HD3	ARG	C	455	109.859	86.789	82.685	1.00	0.00	H
ATOM	3590	HE	ARG	C	455	109.530	88.710	81.730	1.00	0.00	H
ATOM	3591	HH11	ARG	C	455	107.777	87.533	78.262	1.00	0.00	H
ATOM	3592	HH12	ARG	C	455	108.359	86.311	79.358	1.00	0.00	H
ATOM	3593	HH21	ARG	C	455	107.983	89.792	78.814	1.00	0.00	H
ATOM	3594	HH22	ARG	C	455	108.760	90.250	80.305	1.00	0.00	H
ATOM	3595	N	PRO	C	456	111.293	83.115	78.281	1.00	30.00	N
ATOM	3596	CA	PRO	C	456	111.098	83.311	76.833	1.00	30.00	C
ATOM	3597	C	PRO	C	456	110.264	84.566	76.514	1.00	30.00	C
ATOM	3598	O	PRO	C	456	109.195	84.761	77.095	1.00	30.00	O
ATOM	3599	CB	PRO	C	456	110.398	82.018	76.381	1.00	20.00	C
ATOM	3600	CG	PRO	C	456	109.670	81.522	77.614	1.00	20.00	C
ATOM	3601	CD	PRO	C	456	110.598	81.916	78.749	1.00	20.00	C
ATOM	3602	HA	PRO	C	456	112.065	83.386	76.333	1.00	0.00	H
ATOM	3603	HB2	PRO	C	456	109.721	82.156	75.536	1.00	0.00	H
ATOM	3604	HB3	PRO	C	456	111.150	81.288	76.077	1.00	0.00	H
ATOM	3605	HG2	PRO	C	456	108.730	82.065	77.715	1.00	0.00	H
ATOM	3606	HG3	PRO	C	456	109.452	80.455	77.589	1.00	0.00	H
ATOM	3607	HD2	PRO	C	456	110.035	82.066	79.669	1.00	0.00	H
ATOM	3608	HD3	PRO	C	456	111.341	81.135	78.909	1.00	0.00	H
ATOM	3609	N	VAL	C	457	110.788	85.384	75.587	1.00	30.00	N
ATOM	3610	CA	VAL	C	457	110.128	86.571	75.037	1.00	30.00	C
ATOM	3611	C	VAL	C	457	108.946	86.227	74.096	1.00	30.00	C

ATOM	3612	O	VAL	C	457	108.070	87.071	73.908	1.00	30.00	O
ATOM	3613	CB	VAL	C	457	111.162	87.468	74.291	1.00	20.00	C
ATOM	3614	CG1	VAL	C	457	111.746	86.834	73.011	1.00	20.00	C
ATOM	3615	CG2	VAL	C	457	110.634	88.883	73.987	1.00	20.00	C
ATOM	3616	H	VAL	C	457	111.666	85.134	75.155	1.00	0.00	H
ATOM	3617	HA	VAL	C	457	109.722	87.141	75.875	1.00	0.00	H
ATOM	3618	HB	VAL	C	457	111.999	87.605	74.978	1.00	0.00	H
ATOM	3619	HG11	VAL	C	457	112.534	87.461	72.594	1.00	0.00	H
ATOM	3620	HG12	VAL	C	457	112.184	85.857	73.213	1.00	0.00	H
ATOM	3621	HG13	VAL	C	457	110.992	86.706	72.234	1.00	0.00	H
ATOM	3622	HG21	VAL	C	457	111.433	89.530	73.622	1.00	0.00	H
ATOM	3623	HG22	VAL	C	457	109.851	88.880	73.229	1.00	0.00	H
ATOM	3624	HG23	VAL	C	457	110.228	89.348	74.885	1.00	0.00	H
ATOM	3625	N	GLU	C	458	108.930	84.987	73.566	1.00	30.00	N
ATOM	3626	CA	GLU	C	458	107.911	84.398	72.692	1.00	30.00	C
ATOM	3627	C	GLU	C	458	106.468	84.539	73.224	1.00	30.00	C
ATOM	3628	O	GLU	C	458	106.253	84.640	74.433	1.00	30.00	O
ATOM	3629	CB	GLU	C	458	108.235	82.901	72.442	1.00	20.00	C
ATOM	3630	CG	GLU	C	458	109.550	82.601	71.680	1.00	0.00	C
ATOM	3631	CD	GLU	C	458	110.761	82.291	72.570	1.00	0.00	C
ATOM	3632	OE1	GLU	C	458	111.138	83.174	73.370	1.00	0.00	O
ATOM	3633	OE2	GLU	C	458	111.304	81.170	72.430	1.00	0.00	O1-
ATOM	3634	H	GLU	C	458	109.715	84.375	73.762	1.00	0.00	H
ATOM	3635	HA	GLU	C	458	107.963	84.931	71.742	1.00	0.00	H
ATOM	3636	HB2	GLU	C	458	108.238	82.370	73.395	1.00	0.00	H
ATOM	3637	HB3	GLU	C	458	107.417	82.452	71.876	1.00	0.00	H
ATOM	3638	HG2	GLU	C	458	109.387	81.738	71.034	1.00	0.00	H
ATOM	3639	HG3	GLU	C	458	109.802	83.426	71.013	1.00	0.00	H
ATOM	3640	N	GLY	C	459	105.499	84.526	72.296	1.00	30.00	N
ATOM	3641	CA	GLY	C	459	104.074	84.596	72.609	1.00	30.00	C
ATOM	3642	C	GLY	C	459	103.541	83.225	73.054	1.00	30.00	C
ATOM	3643	O	GLY	C	459	104.108	82.186	72.713	1.00	30.00	O
ATOM	3644	H	GLY	C	459	105.748	84.427	71.323	1.00	0.00	H
ATOM	3645	HA2	GLY	C	459	103.885	85.348	73.376	1.00	0.00	H
ATOM	3646	HA3	GLY	C	459	103.535	84.909	71.715	1.00	0.00	H
ATOM	3647	N	LEU	C	460	102.405	83.256	73.772	1.00	30.00	N
ATOM	3648	CA	LEU	C	460	101.577	82.119	74.190	1.00	30.00	C
ATOM	3649	C	LEU	C	460	102.286	81.143	75.163	1.00	30.00	C
ATOM	3650	O	LEU	C	460	102.878	80.164	74.707	1.00	30.00	O
ATOM	3651	CB	LEU	C	460	100.990	81.403	72.940	1.00	20.00	C
ATOM	3652	CG	LEU	C	460	99.909	80.336	73.238	1.00	0.00	C
ATOM	3653	CD1	LEU	C	460	98.703	80.937	73.980	1.00	0.00	C
ATOM	3654	CD2	LEU	C	460	99.477	79.602	71.952	1.00	0.00	C
ATOM	3655	H	LEU	C	460	102.014	84.159	73.997	1.00	0.00	H
ATOM	3656	HA	LEU	C	460	100.733	82.569	74.711	1.00	0.00	H
ATOM	3657	HB2	LEU	C	460	100.559	82.151	72.272	1.00	0.00	H
ATOM	3658	HB3	LEU	C	460	101.796	80.935	72.374	1.00	0.00	H
ATOM	3659	HG	LEU	C	460	100.342	79.576	73.889	1.00	0.00	H
ATOM	3660	HD11	LEU	C	460	97.803	80.334	73.859	1.00	0.00	H
ATOM	3661	HD12	LEU	C	460	98.915	80.996	75.046	1.00	0.00	H
ATOM	3662	HD13	LEU	C	460	98.478	81.946	73.636	1.00	0.00	H
ATOM	3663	HD21	LEU	C	460	99.618	78.526	72.055	1.00	0.00	H
ATOM	3664	HD22	LEU	C	460	98.426	79.769	71.713	1.00	0.00	H
ATOM	3665	HD23	LEU	C	460	100.054	79.921	71.084	1.00	0.00	H
ATOM	3666	N	PRO	C	461	102.157	81.378	76.491	1.00	30.00	N
ATOM	3667	CA	PRO	C	461	102.401	80.334	77.512	1.00	30.00	C
ATOM	3668	C	PRO	C	461	101.423	79.133	77.404	1.00	30.00	C
ATOM	3669	O	PRO	C	461	100.373	79.274	76.775	1.00	30.00	O
ATOM	3670	CB	PRO	C	461	102.186	81.084	78.845	1.00	20.00	C
ATOM	3671	CG	PRO	C	461	102.293	82.562	78.518	1.00	20.00	C
ATOM	3672	CD	PRO	C	461	101.740	82.638	77.104	1.00	20.00	C
ATOM	3673	HA	PRO	C	461	103.440	80.019	77.413	1.00	0.00	H
ATOM	3674	HB2	PRO	C	461	101.185	80.900	79.237	1.00	0.00	H
ATOM	3675	HB3	PRO	C	461	102.896	80.783	79.616	1.00	0.00	H
ATOM	3676	HG2	PRO	C	461	101.761	83.197	79.226	1.00	0.00	H
ATOM	3677	HG3	PRO	C	461	103.340	82.860	78.521	1.00	0.00	H
ATOM	3678	HD2	PRO	C	461	102.118	83.518	76.583	1.00	0.00	H
ATOM	3679	HD3	PRO	C	461	100.651	82.684	77.120	1.00	0.00	H
ATOM	3680	N	PRO	C	462	101.723	78.000	78.080	1.00	30.00	N
ATOM	3681	CA	PRO	C	462	103.050	77.599	78.581	1.00	30.00	C
ATOM	3682	C	PRO	C	462	103.985	77.173	77.429	1.00	30.00	C

```
ATOM   3683  O   PRO C 462     103.510  76.814  76.350  1.00 30.00           O
ATOM   3684  CB  PRO C 462     102.713  76.418  79.505  1.00 20.00           C
ATOM   3685  CG  PRO C 462     101.520  75.750  78.842  1.00 20.00           C
ATOM   3686  CD  PRO C 462     100.739  76.932  78.278  1.00 20.00           C
ATOM   3687  HA  PRO C 462     103.518  78.397  79.158  1.00  0.00           H
ATOM   3688  HB2 PRO C 462     103.545  75.730  79.663  1.00  0.00           H
ATOM   3689  HB3 PRO C 462     102.415  76.800  80.483  1.00  0.00           H
ATOM   3690  HG2 PRO C 462     101.865  75.117  78.024  1.00  0.00           H
ATOM   3691  HG3 PRO C 462     100.932  75.130  79.518  1.00  0.00           H
ATOM   3692  HD2 PRO C 462     100.224  76.658  77.356  1.00  0.00           H
ATOM   3693  HD3 PRO C 462      99.996  77.274  78.999  1.00  0.00           H
ATOM   3694  N   TYR C 463     105.300  77.236  77.679  1.00 30.00           N
ATOM   3695  CA  TYR C 463     106.333  77.015  76.664  1.00 30.00           C
ATOM   3696  C   TYR C 463     106.925  75.604  76.771  1.00 30.00           C
ATOM   3697  O   TYR C 463     107.202  75.139  77.878  1.00 30.00           O
ATOM   3698  CB  TYR C 463     107.430  78.084  76.831  1.00 20.00           C
ATOM   3699  CG  TYR C 463     106.893  79.503  76.798  1.00 20.00           C
ATOM   3700  CD1 TYR C 463     106.687  80.163  75.570  1.00 20.00           C
ATOM   3701  CD2 TYR C 463     106.558  80.151  78.004  1.00 20.00           C
ATOM   3702  CE1 TYR C 463     106.152  81.464  75.555  1.00 20.00           C
ATOM   3703  CE2 TYR C 463     106.011  81.448  77.989  1.00 20.00           C
ATOM   3704  CZ  TYR C 463     105.813  82.107  76.762  1.00 20.00           C
ATOM   3705  OH  TYR C 463     105.302  83.370  76.744  1.00 20.00           O
ATOM   3706  H   TYR C 463     105.627  77.516  78.594  1.00  0.00           H
ATOM   3707  HA  TYR C 463     105.908  77.139  75.665  1.00  0.00           H
ATOM   3708  HB2 TYR C 463     108.183  77.975  76.048  1.00  0.00           H
ATOM   3709  HB3 TYR C 463     107.957  77.938  77.776  1.00  0.00           H
ATOM   3710  HD1 TYR C 463     106.933  79.674  74.639  1.00  0.00           H
ATOM   3711  HD2 TYR C 463     106.721  79.642  78.939  1.00  0.00           H
ATOM   3712  HE1 TYR C 463     105.997  81.960  74.609  1.00  0.00           H
ATOM   3713  HE2 TYR C 463     105.754  81.937  78.917  1.00  0.00           H
ATOM   3714  HH  TYR C 463     105.400  83.780  75.878  1.00  0.00           H
ATOM   3715  N   LYS C 464     107.141  74.971  75.605  1.00 30.00           N
ATOM   3716  CA  LYS C 464     107.827  73.686  75.465  1.00 30.00           C
ATOM   3717  C   LYS C 464     109.342  73.878  75.642  1.00 30.00           C
ATOM   3718  O   LYS C 464     109.927  74.719  74.957  1.00 30.00           O
ATOM   3719  CB  LYS C 464     107.446  73.066  74.097  1.00  0.00           C
ATOM   3720  CG  LYS C 464     108.049  71.676  73.791  1.00  0.00           C
ATOM   3721  CD  LYS C 464     109.365  71.712  72.994  1.00  0.00           C
ATOM   3722  CE  LYS C 464     109.996  70.320  72.831  1.00  0.00           C
ATOM   3723  NZ  LYS C 464     111.355  70.414  72.270  1.00  0.00           N1+
ATOM   3724  H   LYS C 464     106.885  75.428  74.742  1.00  0.00           H
ATOM   3725  HA  LYS C 464     107.462  73.019  76.248  1.00  0.00           H
ATOM   3726  HB2 LYS C 464     106.360  72.960  74.083  1.00  0.00           H
ATOM   3727  HB3 LYS C 464     107.679  73.754  73.283  1.00  0.00           H
ATOM   3728  HG2 LYS C 464     108.198  71.133  74.725  1.00  0.00           H
ATOM   3729  HG3 LYS C 464     107.319  71.094  73.227  1.00  0.00           H
ATOM   3730  HD2 LYS C 464     109.167  72.129  72.006  1.00  0.00           H
ATOM   3731  HD3 LYS C 464     110.078  72.389  73.458  1.00  0.00           H
ATOM   3732  HE2 LYS C 464     110.066  69.821  73.798  1.00  0.00           H
ATOM   3733  HE3 LYS C 464     109.378  69.693  72.188  1.00  0.00           H
ATOM   3734  HZ1 LYS C 464     111.930  70.952  72.904  1.00  0.00           H
ATOM   3735  HZ2 LYS C 464     111.755  69.491  72.170  1.00  0.00           H
ATOM   3736  HZ3 LYS C 464     111.325  70.868  71.369  1.00  0.00           H
ATOM   3737  N   LEU C 465     109.943  73.085  76.547  1.00 30.00           N
ATOM   3738  CA  LEU C 465     111.379  73.095  76.831  1.00 30.00           C
ATOM   3739  C   LEU C 465     112.191  72.530  75.654  1.00 30.00           C
ATOM   3740  O   LEU C 465     111.879  71.453  75.144  1.00 30.00           O
ATOM   3741  CB  LEU C 465     111.668  72.406  78.189  1.00  0.00           C
ATOM   3742  CG  LEU C 465     111.659  70.856  78.254  1.00  0.00           C
ATOM   3743  CD1 LEU C 465     112.172  70.366  79.624  1.00  0.00           C
ATOM   3744  CD2 LEU C 465     110.284  70.240  77.923  1.00  0.00           C
ATOM   3745  H   LEU C 465     109.394  72.419  77.070  1.00  0.00           H
ATOM   3746  HA  LEU C 465     111.658  74.144  76.957  1.00  0.00           H
ATOM   3747  HB2 LEU C 465     112.658  72.743  78.501  1.00  0.00           H
ATOM   3748  HB3 LEU C 465     110.976  72.800  78.933  1.00  0.00           H
ATOM   3749  HG  LEU C 465     112.377  70.479  77.526  1.00  0.00           H
ATOM   3750 HD11 LEU C 465     112.844  69.517  79.501  1.00  0.00           H
ATOM   3751 HD12 LEU C 465     112.728  71.137  80.159  1.00  0.00           H
ATOM   3752 HD13 LEU C 465     111.360  70.048  80.278  1.00  0.00           H
ATOM   3753 HD21 LEU C 465     110.091  69.336  78.502  1.00  0.00           H
```

```
ATOM   3754 HD22 LEU C 465     109.467  70.931  78.129  1.00  0.00           H
ATOM   3755 HD23 LEU C 465     110.228  69.961  76.871  1.00  0.00           H
ATOM   3756  N   LYS C 466     113.207  73.293  75.234  1.00 30.00           N
ATOM   3757  CA  LYS C 466     114.062  72.988  74.090  1.00 30.00           C
ATOM   3758  C   LYS C 466     115.135  71.960  74.492  1.00 30.00           C
ATOM   3759  O   LYS C 466     115.491  71.862  75.667  1.00 30.00           O
ATOM   3760  CB  LYS C 466     114.656  74.311  73.553  1.00  0.00           C
ATOM   3761  CG  LYS C 466     113.572  75.323  73.111  1.00  0.00           C
ATOM   3762  CD  LYS C 466     114.096  76.749  72.872  1.00  0.00           C
ATOM   3763  CE  LYS C 466     112.948  77.747  72.625  1.00  0.00           C
ATOM   3764  NZ  LYS C 466     113.432  79.132  72.492  1.00  0.00           N1+
ATOM   3765  H   LYS C 466     113.439  74.146  75.734  1.00  0.00           H
ATOM   3766  HA  LYS C 466     113.448  72.549  73.302  1.00  0.00           H
ATOM   3767  HB2 LYS C 466     115.278  74.764  74.323  1.00  0.00           H
ATOM   3768  HB3 LYS C 466     115.323  74.111  72.713  1.00  0.00           H
ATOM   3769  HG2 LYS C 466     113.088  74.957  72.205  1.00  0.00           H
ATOM   3770  HG3 LYS C 466     112.784  75.383  73.861  1.00  0.00           H
ATOM   3771  HD2 LYS C 466     114.681  77.069  73.736  1.00  0.00           H
ATOM   3772  HD3 LYS C 466     114.776  76.749  72.020  1.00  0.00           H
ATOM   3773  HE2 LYS C 466     112.394  77.476  71.725  1.00  0.00           H
ATOM   3774  HE3 LYS C 466     112.242  77.717  73.456  1.00  0.00           H
ATOM   3775  HZ1 LYS C 466     112.643  79.762  72.376  1.00  0.00           H
ATOM   3776  HZ2 LYS C 466     113.935  79.396  73.326  1.00  0.00           H
ATOM   3777  HZ3 LYS C 466     114.041  79.210  71.690  1.00  0.00           H
ATOM   3778  N   ASN C 467     115.608  71.183  73.505  1.00 30.00           N
ATOM   3779  CA  ASN C 467     116.524  70.050  73.713  1.00 30.00           C
ATOM   3780  C   ASN C 467     117.980  70.461  74.016  1.00 30.00           C
ATOM   3781  O   ASN C 467     118.765  69.587  74.384  1.00 30.00           O
ATOM   3782  CB  ASN C 467     116.433  69.088  72.506  1.00  0.00           C
ATOM   3783  CG  ASN C 467     115.064  68.404  72.398  1.00  0.00           C
ATOM   3784  ND2 ASN C 467     114.971  67.152  72.853  1.00  0.00           N
ATOM   3785  OD1 ASN C 467     114.102  68.997  71.915  1.00  0.00           O
ATOM   3786  H   ASN C 467     115.272  71.312  72.562  1.00  0.00           H
ATOM   3787  HA  ASN C 467     116.148  69.518  74.589  1.00  0.00           H
ATOM   3788  HB2 ASN C 467     116.646  69.619  71.576  1.00  0.00           H
ATOM   3789  HB3 ASN C 467     117.193  68.309  72.589  1.00  0.00           H
ATOM   3790 HD21 ASN C 467     114.092  66.658  72.803  1.00  0.00           H
ATOM   3791 HD22 ASN C 467     115.777  66.687  73.245  1.00  0.00           H
ATOM   3792  N   THR C 468     118.309  71.761  73.913  1.00 30.00           N
ATOM   3793  CA  THR C 468     119.561  72.345  74.400  1.00 30.00           C
ATOM   3794  C   THR C 468     119.628  72.316  75.943  1.00 30.00           C
ATOM   3795  O   THR C 468     118.608  72.524  76.596  1.00 30.00           O
ATOM   3796  CB  THR C 468     119.729  73.808  73.895  1.00  0.00           C
ATOM   3797  CG2 THR C 468     118.620  74.788  74.328  1.00  0.00           C
ATOM   3798  OG1 THR C 468     120.955  74.365  74.337  1.00  0.00           O
ATOM   3799  H   THR C 468     117.601  72.421  73.626  1.00  0.00           H
ATOM   3800  HA  THR C 468     120.385  71.747  74.003  1.00  0.00           H
ATOM   3801  HB  THR C 468     119.761  73.789  72.806  1.00  0.00           H
ATOM   3802  HG1 THR C 468     120.987  75.286  74.066  1.00  0.00           H
ATOM   3803 HG21 THR C 468     118.753  75.762  73.857  1.00  0.00           H
ATOM   3804 HG22 THR C 468     117.635  74.423  74.043  1.00  0.00           H
ATOM   3805 HG23 THR C 468     118.612  74.950  75.407  1.00  0.00           H
ATOM   3806  N   VAL C 469     120.830  72.064  76.488  1.00 30.00           N
ATOM   3807  CA  VAL C 469     121.104  71.926  77.925  1.00 30.00           C
ATOM   3808  C   VAL C 469     120.846  73.193  78.781  1.00 30.00           C
ATOM   3809  O   VAL C 469     120.782  73.075  80.005  1.00 30.00           O
ATOM   3810  CB  VAL C 469     122.573  71.471  78.166  1.00  0.00           C
ATOM   3811  CG1 VAL C 469     122.837  70.076  77.572  1.00  0.00           C
ATOM   3812  CG2 VAL C 469     123.641  72.481  77.697  1.00  0.00           C
ATOM   3813  H   VAL C 469     121.623  71.923  75.879  1.00  0.00           H
ATOM   3814  HA  VAL C 469     120.448  71.143  78.305  1.00  0.00           H
ATOM   3815  HB  VAL C 469     122.710  71.356  79.243  1.00  0.00           H
ATOM   3816 HG11 VAL C 469     123.839  69.727  77.822  1.00  0.00           H
ATOM   3817 HG12 VAL C 469     122.130  69.343  77.960  1.00  0.00           H
ATOM   3818 HG13 VAL C 469     122.752  70.078  76.485  1.00  0.00           H
ATOM   3819 HG21 VAL C 469     124.646  72.093  77.871  1.00  0.00           H
ATOM   3820 HG22 VAL C 469     123.553  72.691  76.631  1.00  0.00           H
ATOM   3821 HG23 VAL C 469     123.572  73.429  78.230  1.00  0.00           H
ATOM   3822  N   GLY C 470     120.709  74.365  78.135  1.00 30.00           N
ATOM   3823  CA  GLY C 470     120.592  75.670  78.783  1.00 30.00           C
ATOM   3824  C   GLY C 470     119.262  75.852  79.530  1.00 30.00           C
```

```
ATOM   3825  O   GLY C 470     119.288  76.238  80.698  1.00 30.00           O
ATOM   3826  H   GLY C 470     120.748  74.363  77.125  1.00  0.00           H
ATOM   3827  HA2 GLY C 470     121.427  75.813  79.470  1.00  0.00           H
ATOM   3828  HA3 GLY C 470     120.673  76.445  78.021  1.00  0.00           H
ATOM   3829  N   ASP C 471     118.113  75.580  78.880  1.00 30.00           N
ATOM   3830  CA  ASP C 471     116.780  75.808  79.462  1.00 30.00           C
ATOM   3831  C   ASP C 471     116.287  74.662  80.370  1.00 30.00           C
ATOM   3832  O   ASP C 471     115.216  74.803  80.956  1.00 30.00           O
ATOM   3833  CB  ASP C 471     115.684  76.264  78.460  1.00 20.00           C
ATOM   3834  CG  ASP C 471     115.438  75.389  77.228  1.00 20.00           C
ATOM   3835  OD1 ASP C 471     116.327  74.588  76.870  1.00 20.00           O
ATOM   3836  OD2 ASP C 471     114.363  75.593  76.622  1.00 20.00           O1-
ATOM   3837  H   ASP C 471     118.123  75.222  77.933  1.00  0.00           H
ATOM   3838  HA  ASP C 471     116.880  76.657  80.140  1.00  0.00           H
ATOM   3839  HB2 ASP C 471     115.950  77.257  78.097  1.00  0.00           H
ATOM   3840  HB3 ASP C 471     114.735  76.375  78.987  1.00  0.00           H
ATOM   3841  N   TYR C 472     117.084  73.594  80.560  1.00  0.00           N
ATOM   3842  CA  TYR C 472     116.875  72.642  81.658  1.00  0.00           C
ATOM   3843  C   TYR C 472     117.199  73.271  83.024  1.00  0.00           C
ATOM   3844  O   TYR C 472     116.505  72.958  83.988  1.00  0.00           O
ATOM   3845  CB  TYR C 472     117.667  71.342  81.428  1.00 20.00           C
ATOM   3846  CG  TYR C 472     117.109  70.481  80.310  1.00 20.00           C
ATOM   3847  CD1 TYR C 472     116.133  69.497  80.577  1.00 20.00           C
ATOM   3848  CD2 TYR C 472     117.550  70.680  78.989  1.00 20.00           C
ATOM   3849  CE1 TYR C 472     115.608  68.720  79.525  1.00 20.00           C
ATOM   3850  CE2 TYR C 472     117.026  69.907  77.937  1.00 20.00           C
ATOM   3851  CZ  TYR C 472     116.050  68.928  78.203  1.00 20.00           C
ATOM   3852  OH  TYR C 472     115.531  68.185  77.183  1.00 20.00           O
ATOM   3853  H   TYR C 472     117.947  73.511  80.041  1.00  0.00           H
ATOM   3854  HA  TYR C 472     115.815  72.376  81.672  1.00  0.00           H
ATOM   3855  HB2 TYR C 472     118.718  71.563  81.236  1.00  0.00           H
ATOM   3856  HB3 TYR C 472     117.652  70.738  82.337  1.00  0.00           H
ATOM   3857  HD1 TYR C 472     115.782  69.339  81.586  1.00  0.00           H
ATOM   3858  HD2 TYR C 472     118.282  71.443  78.789  1.00  0.00           H
ATOM   3859  HE1 TYR C 472     114.860  67.969  79.734  1.00  0.00           H
ATOM   3860  HE2 TYR C 472     117.368  70.077  76.927  1.00  0.00           H
ATOM   3861  HH  TYR C 472     115.896  68.427  76.329  1.00  0.00           H
ATOM   3862  N   PHE C 473     118.175  74.197  83.076  1.00  0.00           N
ATOM   3863  CA  PHE C 473     118.452  75.024  84.256  1.00  0.00           C
ATOM   3864  C   PHE C 473     117.372  76.090  84.517  1.00  0.00           C
ATOM   3865  O   PHE C 473     117.174  76.452  85.676  1.00  0.00           O
ATOM   3866  CB  PHE C 473     119.848  75.671  84.146  1.00 20.00           C
ATOM   3867  CG  PHE C 473     121.030  74.714  84.098  1.00 20.00           C
ATOM   3868  CD1 PHE C 473     121.122  73.617  84.986  1.00 20.00           C
ATOM   3869  CD2 PHE C 473     122.132  75.008  83.267  1.00 20.00           C
ATOM   3870  CE1 PHE C 473     122.253  72.812  84.984  1.00 20.00           C
ATOM   3871  CE2 PHE C 473     123.257  74.194  83.285  1.00 20.00           C
ATOM   3872  CZ  PHE C 473     123.314  73.098  84.136  1.00 20.00           C
ATOM   3873  H   PHE C 473     118.708  74.414  82.245  1.00  0.00           H
ATOM   3874  HA  PHE C 473     118.447  74.376  85.131  1.00  0.00           H
ATOM   3875  HB2 PHE C 473     119.875  76.295  83.253  1.00  0.00           H
ATOM   3876  HB3 PHE C 473     120.013  76.350  84.984  1.00  0.00           H
ATOM   3877  HD1 PHE C 473     120.323  73.392  85.676  1.00  0.00           H
ATOM   3878  HD2 PHE C 473     122.106  75.865  82.610  1.00  0.00           H
ATOM   3879  HE1 PHE C 473     122.312  71.967  85.654  1.00  0.00           H
ATOM   3880  HE2 PHE C 473     124.092  74.417  82.637  1.00  0.00           H
ATOM   3881  HZ  PHE C 473     124.194  72.470  84.145  1.00  0.00           H
ATOM   3882  N   ARG C 474     116.670  76.541  83.460  1.00 30.00           N
ATOM   3883  CA  ARG C 474     115.500  77.412  83.576  1.00 30.00           C
ATOM   3884  C   ARG C 474     114.289  76.654  84.149  1.00 30.00           C
ATOM   3885  O   ARG C 474     113.639  77.186  85.042  1.00 30.00           O
ATOM   3886  CB  ARG C 474     115.156  78.049  82.211  1.00 20.00           C
ATOM   3887  CG  ARG C 474     114.001  79.069  82.274  1.00 20.00           C
ATOM   3888  CD  ARG C 474     113.205  79.165  80.967  1.00 20.00           C
ATOM   3889  NE  ARG C 474     111.952  79.897  81.184  1.00 20.00           N
ATOM   3890  CZ  ARG C 474     110.799  79.359  81.621  1.00 20.00           C
ATOM   3891  NH1 ARG C 474     109.742  80.151  81.838  1.00 20.00           N
ATOM   3892  NH2 ARG C 474     110.686  78.041  81.849  1.00 20.00           N1+
ATOM   3893  H   ARG C 474     116.892  76.204  82.534  1.00  0.00           H
ATOM   3894  HA  ARG C 474     115.748  78.222  84.266  1.00  0.00           H
ATOM   3895  HB2 ARG C 474     116.033  78.516  81.761  1.00  0.00           H
```

ATOM	3896	HB3	ARG	C	474	114.872	77.246	81.533	1.00	0.00	H
ATOM	3897	HG2	ARG	C	474	113.294	78.882	83.082	1.00	0.00	H
ATOM	3898	HG3	ARG	C	474	114.450	80.035	82.508	1.00	0.00	H
ATOM	3899	HD2	ARG	C	474	113.769	79.807	80.293	1.00	0.00	H
ATOM	3900	HD3	ARG	C	474	113.083	78.218	80.439	1.00	0.00	H
ATOM	3901	HE	ARG	C	474	112.016	80.900	81.089	1.00	0.00	H
ATOM	3902	HH11	ARG	C	474	108.869	79.758	82.162	1.00	0.00	H
ATOM	3903	HH12	ARG	C	474	109.810	81.146	81.690	1.00	0.00	H
ATOM	3904	HH21	ARG	C	474	109.817	77.648	82.183	1.00	0.00	H
ATOM	3905	HH22	ARG	C	474	111.476	77.434	81.691	1.00	0.00	H
ATOM	3906	N	VAL	C	475	114.002	75.450	83.622	1.00	0.00	N
ATOM	3907	CA	VAL	C	475	112.855	74.619	84.001	1.00	0.00	C
ATOM	3908	C	VAL	C	475	112.974	74.033	85.422	1.00	0.00	C
ATOM	3909	O	VAL	C	475	111.992	74.075	86.163	1.00	0.00	O
ATOM	3910	CB	VAL	C	475	112.615	73.490	82.956	1.00	20.00	C
ATOM	3911	CG1	VAL	C	475	111.719	72.316	83.410	1.00	20.00	C
ATOM	3912	CG2	VAL	C	475	112.038	74.087	81.661	1.00	20.00	C
ATOM	3913	H	VAL	C	475	114.578	75.090	82.873	1.00	0.00	H
ATOM	3914	HA	VAL	C	475	111.975	75.266	84.000	1.00	0.00	H
ATOM	3915	HB	VAL	C	475	113.587	73.062	82.702	1.00	0.00	H
ATOM	3916	HG11	VAL	C	475	111.522	71.632	82.583	1.00	0.00	H
ATOM	3917	HG12	VAL	C	475	112.186	71.723	84.197	1.00	0.00	H
ATOM	3918	HG13	VAL	C	475	110.757	72.671	83.779	1.00	0.00	H
ATOM	3919	HG21	VAL	C	475	111.986	73.330	80.883	1.00	0.00	H
ATOM	3920	HG22	VAL	C	475	111.031	74.473	81.817	1.00	0.00	H
ATOM	3921	HG23	VAL	C	475	112.647	74.905	81.277	1.00	0.00	H
ATOM	3922	N	THR	C	476	114.176	73.558	85.795	1.00	0.00	N
ATOM	3923	CA	THR	C	476	114.485	73.135	87.165	1.00	0.00	C
ATOM	3924	C	THR	C	476	114.588	74.326	88.144	1.00	0.00	C
ATOM	3925	O	THR	C	476	114.348	74.119	89.329	1.00	0.00	O
ATOM	3926	CB	THR	C	476	115.794	72.311	87.268	1.00	20.00	C
ATOM	3927	CG2	THR	C	476	115.737	70.990	86.485	1.00	20.00	C
ATOM	3928	OG1	THR	C	476	116.929	73.054	86.866	1.00	20.00	O
ATOM	3929	H	THR	C	476	114.941	73.536	85.133	1.00	0.00	H
ATOM	3930	HA	THR	C	476	113.676	72.486	87.499	1.00	0.00	H
ATOM	3931	HB	THR	C	476	115.961	72.051	88.315	1.00	0.00	H
ATOM	3932	HG1	THR	C	476	116.935	73.095	85.905	1.00	0.00	H
ATOM	3933	HG21	THR	C	476	116.687	70.458	86.540	1.00	0.00	H
ATOM	3934	HG22	THR	C	476	114.971	70.333	86.898	1.00	0.00	H
ATOM	3935	HG23	THR	C	476	115.498	71.137	85.433	1.00	0.00	H
ATOM	3936	N	GLY	C	477	114.879	75.542	87.647	1.00	0.00	N
ATOM	3937	CA	GLY	C	477	114.877	76.764	88.452	1.00	0.00	C
ATOM	3938	C	GLY	C	477	113.451	77.307	88.643	1.00	0.00	C
ATOM	3939	O	GLY	C	477	113.166	77.906	89.678	1.00	0.00	O
ATOM	3940	H	GLY	C	477	115.084	75.642	86.663	1.00	0.00	H
ATOM	3941	HA2	GLY	C	477	115.340	76.575	89.420	1.00	0.00	H
ATOM	3942	HA3	GLY	C	477	115.479	77.520	87.948	1.00	0.00	H
ATOM	3943	N	GLU	C	478	112.561	77.084	87.659	1.00	0.00	N
ATOM	3944	CA	GLU	C	478	111.158	77.494	87.660	1.00	0.00	C
ATOM	3945	C	GLU	C	478	110.337	76.669	88.659	1.00	0.00	C
ATOM	3946	O	GLU	C	478	109.635	77.262	89.473	1.00	0.00	O
ATOM	3947	CB	GLU	C	478	110.619	77.495	86.206	1.00	20.00	C
ATOM	3948	CG	GLU	C	478	109.141	77.907	85.985	1.00	20.00	C
ATOM	3949	CD	GLU	C	478	108.087	76.832	86.276	1.00	20.00	C
ATOM	3950	OE1	GLU	C	478	108.390	75.640	86.052	1.00	20.00	O
ATOM	3951	OE2	GLU	C	478	106.982	77.230	86.705	1.00	20.00	O1-
ATOM	3952	H	GLU	C	478	112.873	76.612	86.821	1.00	0.00	H
ATOM	3953	HA	GLU	C	478	111.127	78.530	88.004	1.00	0.00	H
ATOM	3954	HB2	GLU	C	478	111.229	78.192	85.630	1.00	0.00	H
ATOM	3955	HB3	GLU	C	478	110.802	76.523	85.747	1.00	0.00	H
ATOM	3956	HG2	GLU	C	478	108.919	78.801	86.569	1.00	0.00	H
ATOM	3957	HG3	GLU	C	478	109.011	78.191	84.940	1.00	0.00	H
ATOM	3958	N	ILE	C	479	110.476	75.332	88.626	1.00	0.00	N
ATOM	3959	CA	ILE	C	479	109.789	74.433	89.559	1.00	0.00	C
ATOM	3960	C	ILE	C	479	110.320	74.546	91.010	1.00	0.00	C
ATOM	3961	O	ILE	C	479	109.546	74.308	91.935	1.00	0.00	O
ATOM	3962	CB	ILE	C	479	109.837	72.955	89.074	1.00	20.00	C
ATOM	3963	CG1	ILE	C	479	108.771	72.070	89.762	1.00	20.00	C
ATOM	3964	CG2	ILE	C	479	111.230	72.309	89.181	1.00	20.00	C
ATOM	3965	CD1	ILE	C	479	108.537	70.727	89.058	1.00	20.00	C
ATOM	3966	H	ILE	C	479	111.053	74.900	87.917	1.00	0.00	H

```
ATOM   3967  HA  ILE C 479     108.742  74.741  89.571  1.00  0.00           H
ATOM   3968  HB  ILE C 479     109.587  72.985  88.012  1.00  0.00           H
ATOM   3969 HG12 ILE C 479     109.052  71.882  90.799  1.00  0.00           H
ATOM   3970 HG13 ILE C 479     107.821  72.605  89.803  1.00  0.00           H
ATOM   3971 HG21 ILE C 479     111.288  71.388  88.601  1.00  0.00           H
ATOM   3972 HG22 ILE C 479     111.988  72.986  88.798  1.00  0.00           H
ATOM   3973 HG23 ILE C 479     111.490  72.067  90.211  1.00  0.00           H
ATOM   3974 HD11 ILE C 479     107.589  70.288  89.369  1.00  0.00           H
ATOM   3975 HD12 ILE C 479     108.508  70.841  87.974  1.00  0.00           H
ATOM   3976 HD13 ILE C 479     109.324  70.012  89.300  1.00  0.00           H
ATOM   3977  N   LEU C 480     111.587  74.971  91.190  1.00  0.00           N
ATOM   3978  CA  LEU C 480     112.178  75.307  92.492  1.00  0.00           C
ATOM   3979  C   LEU C 480     111.619  76.623  93.062  1.00  0.00           C
ATOM   3980  O   LEU C 480     111.391  76.692  94.270  1.00  0.00           O
ATOM   3981  CB  LEU C 480     113.724  75.367  92.390  1.00 20.00           C
ATOM   3982  CG  LEU C 480     114.466  74.117  92.922  1.00 20.00           C
ATOM   3983  CD1 LEU C 480     114.340  73.991  94.456  1.00 20.00           C
ATOM   3984  CD2 LEU C 480     114.106  72.809  92.186  1.00 20.00           C
ATOM   3985  H   LEU C 480     112.170  75.139  90.381  1.00  0.00           H
ATOM   3986  HA  LEU C 480     111.905  74.516  93.192  1.00  0.00           H
ATOM   3987  HB2 LEU C 480     114.023  75.574  91.364  1.00  0.00           H
ATOM   3988  HB3 LEU C 480     114.106  76.223  92.950  1.00  0.00           H
ATOM   3989  HG  LEU C 480     115.523  74.302  92.721  1.00  0.00           H
ATOM   3990 HD11 LEU C 480     115.321  73.868  94.916  1.00  0.00           H
ATOM   3991 HD12 LEU C 480     113.888  74.877  94.903  1.00  0.00           H
ATOM   3992 HD13 LEU C 480     113.730  73.140  94.760  1.00  0.00           H
ATOM   3993 HD21 LEU C 480     113.737  72.033  92.856  1.00  0.00           H
ATOM   3994 HD22 LEU C 480     113.339  72.957  91.429  1.00  0.00           H
ATOM   3995 HD23 LEU C 480     114.980  72.401  91.677  1.00  0.00           H
ATOM   3996  N   SER C 481     111.386  77.627  92.195  1.00  0.00           N
ATOM   3997  CA  SER C 481     110.759  78.898  92.571  1.00  0.00           C
ATOM   3998  C   SER C 481     109.273  78.737  92.956  1.00  0.00           C
ATOM   3999  O   SER C 481     108.817  79.402  93.888  1.00  0.00           O
ATOM   4000  CB  SER C 481     111.018  79.969  91.492  1.00 20.00           C
ATOM   4001  OG  SER C 481     110.159  79.898  90.373  1.00 20.00           O
ATOM   4002  H   SER C 481     111.622  77.513  91.219  1.00  0.00           H
ATOM   4003  HA  SER C 481     111.293  79.247  93.452  1.00  0.00           H
ATOM   4004  HB2 SER C 481     112.056  79.944  91.158  1.00  0.00           H
ATOM   4005  HB3 SER C 481     110.861  80.947  91.942  1.00  0.00           H
ATOM   4006  HG  SER C 481     110.239  79.028  89.970  1.00  0.00           H
ATOM   4007  N   VAL C 482     108.572  77.816  92.271  1.00  0.00           N
ATOM   4008  CA  VAL C 482     107.197  77.409  92.558  1.00  0.00           C
ATOM   4009  C   VAL C 482     107.086  76.561  93.841  1.00  0.00           C
ATOM   4010  O   VAL C 482     106.082  76.691  94.537  1.00  0.00           O
ATOM   4011  CB  VAL C 482     106.586  76.629  91.354  1.00 20.00           C
ATOM   4012  CG1 VAL C 482     105.271  75.868  91.638  1.00 20.00           C
ATOM   4013  CG2 VAL C 482     106.373  77.571  90.158  1.00 20.00           C
ATOM   4014  H   VAL C 482     109.019  77.336  91.501  1.00  0.00           H
ATOM   4015  HA  VAL C 482     106.607  78.314  92.715  1.00  0.00           H
ATOM   4016  HB  VAL C 482     107.313  75.881  91.038  1.00  0.00           H
ATOM   4017 HG11 VAL C 482     104.845  75.465  90.719  1.00  0.00           H
ATOM   4018 HG12 VAL C 482     105.423  75.020  92.306  1.00  0.00           H
ATOM   4019 HG13 VAL C 482     104.524  76.522  92.089  1.00  0.00           H
ATOM   4020 HG21 VAL C 482     105.981  77.035  89.294  1.00  0.00           H
ATOM   4021 HG22 VAL C 482     105.671  78.364  90.412  1.00  0.00           H
ATOM   4022 HG23 VAL C 482     107.294  78.055  89.843  1.00  0.00           H
ATOM   4023  N   SER C 483     108.112  75.748  94.156  1.00  0.00           N
ATOM   4024  CA  SER C 483     108.166  74.912  95.361  1.00  0.00           C
ATOM   4025  C   SER C 483     108.255  75.722  96.668  1.00  0.00           C
ATOM   4026  O   SER C 483     107.697  75.284  97.674  1.00  0.00           O
ATOM   4027  CB  SER C 483     109.332  73.908  95.263  1.00 20.00           C
ATOM   4028  OG  SER C 483     109.001  72.852  94.385  1.00 20.00           O
ATOM   4029  H   SER C 483     108.901  75.679  93.529  1.00  0.00           H
ATOM   4030  HA  SER C 483     107.234  74.345  95.416  1.00  0.00           H
ATOM   4031  HB2 SER C 483     109.543  73.462  96.236  1.00  0.00           H
ATOM   4032  HB3 SER C 483     110.251  74.391  94.933  1.00  0.00           H
ATOM   4033  HG  SER C 483     109.027  73.189  93.484  1.00  0.00           H
ATOM   4034  N   GLY C 484     108.895  76.904  96.620  1.00  0.00           N
ATOM   4035  CA  GLY C 484     108.899  77.860  97.729  1.00  0.00           C
ATOM   4036  C   GLY C 484     107.574  78.635  97.788  1.00  0.00           C
ATOM   4037  O   GLY C 484     107.136  78.990  98.879  1.00  0.00           O
```

ATOM	4038	H	GLY	C	484	109.342	77.190	95.760	1.00	0.00	H
ATOM	4039	HA2	GLY	C	484	109.087	77.348	98.672	1.00	0.00	H
ATOM	4040	HA3	GLY	C	484	109.713	78.567	97.604	1.00	0.00	H
ATOM	4041	N	GLY	C	485	106.908	78.844	96.636	1.00	0.00	N
ATOM	4042	CA	GLY	C	485	105.571	79.437	96.535	1.00	0.00	C
ATOM	4043	C	GLY	C	485	104.469	78.500	97.062	1.00	0.00	C
ATOM	4044	O	GLY	C	485	103.384	78.975	97.386	1.00	0.00	O
ATOM	4045	H	GLY	C	485	107.318	78.518	95.772	1.00	0.00	H
ATOM	4046	HA2	GLY	C	485	105.543	80.387	97.070	1.00	0.00	H
ATOM	4047	HA3	GLY	C	485	105.364	79.650	95.487	1.00	0.00	H
ATOM	4048	N	VAL	C	486	104.747	77.191	97.172	1.00	0.00	N
ATOM	4049	CA	VAL	C	486	103.890	76.191	97.806	1.00	0.00	C
ATOM	4050	C	VAL	C	486	104.214	76.067	99.309	1.00	0.00	C
ATOM	4051	O	VAL	C	486	103.285	75.955	100.110	1.00	0.00	O
ATOM	4052	CB	VAL	C	486	104.049	74.808	97.103	1.00	20.00	C
ATOM	4053	CG1	VAL	C	486	103.497	73.595	97.880	1.00	20.00	C
ATOM	4054	CG2	VAL	C	486	103.406	74.838	95.704	1.00	20.00	C
ATOM	4055	H	VAL	C	486	105.639	76.856	96.836	1.00	0.00	H
ATOM	4056	HA	VAL	C	486	102.845	76.498	97.716	1.00	0.00	H
ATOM	4057	HB	VAL	C	486	105.115	74.628	96.955	1.00	0.00	H
ATOM	4058	HG11	VAL	C	486	103.551	72.686	97.281	1.00	0.00	H
ATOM	4059	HG12	VAL	C	486	104.056	73.397	98.796	1.00	0.00	H
ATOM	4060	HG13	VAL	C	486	102.453	73.753	98.146	1.00	0.00	H
ATOM	4061	HG21	VAL	C	486	103.598	73.913	95.161	1.00	0.00	H
ATOM	4062	HG22	VAL	C	486	102.325	74.964	95.773	1.00	0.00	H
ATOM	4063	HG23	VAL	C	486	103.789	75.655	95.096	1.00	0.00	H
ATOM	4064	N	TYR	C	487	105.511	76.117	99.668	1.00	0.00	N
ATOM	4065	CA	TYR	C	487	105.998	76.026	101.047	1.00	0.00	C
ATOM	4066	C	TYR	C	487	105.543	77.201	101.927	1.00	0.00	C
ATOM	4067	O	TYR	C	487	104.972	76.953	102.986	1.00	0.00	O
ATOM	4068	CB	TYR	C	487	107.532	75.852	101.057	1.00	20.00	C
ATOM	4069	CG	TYR	C	487	108.180	75.847	102.435	1.00	20.00	C
ATOM	4070	CD1	TYR	C	487	107.920	74.790	103.331	1.00	20.00	C
ATOM	4071	CD2	TYR	C	487	109.022	76.908	102.832	1.00	20.00	C
ATOM	4072	CE1	TYR	C	487	108.493	74.795	104.618	1.00	20.00	C
ATOM	4073	CE2	TYR	C	487	109.600	76.910	104.116	1.00	20.00	C
ATOM	4074	CZ	TYR	C	487	109.334	75.854	105.011	1.00	20.00	C
ATOM	4075	OH	TYR	C	487	109.883	75.857	106.260	1.00	20.00	O
ATOM	4076	H	TYR	C	487	106.220	76.197	98.952	1.00	0.00	H
ATOM	4077	HA	TYR	C	487	105.565	75.118	101.470	1.00	0.00	H
ATOM	4078	HB2	TYR	C	487	107.802	74.926	100.548	1.00	0.00	H
ATOM	4079	HB3	TYR	C	487	107.983	76.656	100.477	1.00	0.00	H
ATOM	4080	HD1	TYR	C	487	107.273	73.976	103.038	1.00	0.00	H
ATOM	4081	HD2	TYR	C	487	109.223	77.728	102.156	1.00	0.00	H
ATOM	4082	HE1	TYR	C	487	108.285	73.986	105.302	1.00	0.00	H
ATOM	4083	HE2	TYR	C	487	110.244	77.726	104.409	1.00	0.00	H
ATOM	4084	HH	TYR	C	487	110.475	76.596	106.416	1.00	0.00	H
ATOM	4085	N	PHE	C	488	105.778	78.443	101.468	1.00	0.00	N
ATOM	4086	CA	PHE	C	488	105.384	79.672	102.166	1.00	0.00	C
ATOM	4087	C	PHE	C	488	103.864	79.918	102.199	1.00	0.00	C
ATOM	4088	O	PHE	C	488	103.400	80.650	103.073	1.00	0.00	O
ATOM	4089	CB	PHE	C	488	106.142	80.880	101.576	1.00	20.00	C
ATOM	4090	CG	PHE	C	488	107.648	80.872	101.789	1.00	20.00	C
ATOM	4091	CD1	PHE	C	488	108.194	80.702	103.081	1.00	20.00	C
ATOM	4092	CD2	PHE	C	488	108.520	81.123	100.707	1.00	20.00	C
ATOM	4093	CE1	PHE	C	488	109.570	80.708	103.258	1.00	20.00	C
ATOM	4094	CE2	PHE	C	488	109.894	81.113	100.904	1.00	20.00	C
ATOM	4095	CZ	PHE	C	488	110.415	80.897	102.173	1.00	20.00	C
ATOM	4096	H	PHE	C	488	106.255	78.568	100.583	1.00	0.00	H
ATOM	4097	HA	PHE	C	488	105.681	79.563	103.207	1.00	0.00	H
ATOM	4098	HB2	PHE	C	488	105.959	80.907	100.502	1.00	0.00	H
ATOM	4099	HB3	PHE	C	488	105.752	81.816	101.978	1.00	0.00	H
ATOM	4100	HD1	PHE	C	488	107.551	80.560	103.937	1.00	0.00	H
ATOM	4101	HD2	PHE	C	488	108.128	81.301	99.717	1.00	0.00	H
ATOM	4102	HE1	PHE	C	488	109.986	80.564	104.244	1.00	0.00	H
ATOM	4103	HE2	PHE	C	488	110.558	81.274	100.068	1.00	0.00	H
ATOM	4104	HZ	PHE	C	488	111.483	80.886	102.324	1.00	0.00	H
ATOM	4105	N	PHE	C	489	103.120	79.275	101.282	1.00	0.00	N
ATOM	4106	CA	PHE	C	489	101.659	79.255	101.258	1.00	0.00	C
ATOM	4107	C	PHE	C	489	101.084	78.397	102.394	1.00	0.00	C
ATOM	4108	O	PHE	C	489	100.261	78.897	103.159	1.00	0.00	O

ATOM	4109	CB	PHE	C	489	101.184	78.837	99.852	1.00	20.00	C
ATOM	4110	CG	PHE	C	489	99.688	78.708	99.636	1.00	20.00	C
ATOM	4111	CD1	PHE	C	489	98.901	79.863	99.441	1.00	20.00	C
ATOM	4112	CD2	PHE	C	489	99.055	77.449	99.724	1.00	20.00	C
ATOM	4113	CE1	PHE	C	489	97.530	79.745	99.262	1.00	20.00	C
ATOM	4114	CE2	PHE	C	489	97.682	77.353	99.539	1.00	20.00	C
ATOM	4115	CZ	PHE	C	489	96.925	78.494	99.299	1.00	20.00	C
ATOM	4116	H	PHE	C	489	103.580	78.696	100.595	1.00	0.00	H
ATOM	4117	HA	PHE	C	489	101.306	80.273	101.424	1.00	0.00	H
ATOM	4118	HB2	PHE	C	489	101.539	79.584	99.144	1.00	0.00	H
ATOM	4119	HB3	PHE	C	489	101.657	77.903	99.553	1.00	0.00	H
ATOM	4120	HD1	PHE	C	489	99.366	80.838	99.411	1.00	0.00	H
ATOM	4121	HD2	PHE	C	489	99.636	76.559	99.917	1.00	0.00	H
ATOM	4122	HE1	PHE	C	489	96.932	80.628	99.094	1.00	0.00	H
ATOM	4123	HE2	PHE	C	489	97.201	76.387	99.587	1.00	0.00	H
ATOM	4124	HZ	PHE	C	489	95.858	78.410	99.159	1.00	0.00	H
ATOM	4125	N	PHE	C	490	101.573	77.150	102.520	1.00	0.00	N
ATOM	4126	CA	PHE	C	490	101.225	76.239	103.614	1.00	0.00	C
ATOM	4127	C	PHE	C	490	101.758	76.669	104.991	1.00	0.00	C
ATOM	4128	O	PHE	C	490	101.075	76.428	105.985	1.00	0.00	O
ATOM	4129	CB	PHE	C	490	101.666	74.799	103.277	1.00	20.00	C
ATOM	4130	CG	PHE	C	490	100.822	74.086	102.233	1.00	20.00	C
ATOM	4131	CD1	PHE	C	490	99.424	73.966	102.398	1.00	20.00	C
ATOM	4132	CD2	PHE	C	490	101.436	73.445	101.135	1.00	20.00	C
ATOM	4133	CE1	PHE	C	490	98.670	73.265	101.467	1.00	20.00	C
ATOM	4134	CE2	PHE	C	490	100.663	72.743	100.219	1.00	20.00	C
ATOM	4135	CZ	PHE	C	490	99.287	72.659	100.381	1.00	20.00	C
ATOM	4136	H	PHE	C	490	102.248	76.806	101.850	1.00	0.00	H
ATOM	4137	HA	PHE	C	490	100.139	76.253	103.707	1.00	0.00	H
ATOM	4138	HB2	PHE	C	490	102.704	74.818	102.941	1.00	0.00	H
ATOM	4139	HB3	PHE	C	490	101.657	74.177	104.174	1.00	0.00	H
ATOM	4140	HD1	PHE	C	490	98.930	74.412	103.249	1.00	0.00	H
ATOM	4141	HD2	PHE	C	490	102.507	73.493	101.003	1.00	0.00	H
ATOM	4142	HE1	PHE	C	490	97.600	73.184	101.593	1.00	0.00	H
ATOM	4143	HE2	PHE	C	490	101.134	72.253	99.381	1.00	0.00	H
ATOM	4144	HZ	PHE	C	490	98.694	72.111	99.664	1.00	0.00	H
ATOM	4145	N	ARG	C	491	102.940	77.311	105.029	1.00	0.00	N
ATOM	4146	CA	ARG	C	491	103.564	77.820	106.252	1.00	0.00	C
ATOM	4147	C	ARG	C	491	102.830	79.046	106.825	1.00	0.00	C
ATOM	4148	O	ARG	C	491	102.699	79.142	108.044	1.00	0.00	O
ATOM	4149	CB	ARG	C	491	105.064	78.082	106.011	1.00	20.00	C
ATOM	4150	CG	ARG	C	491	105.868	78.383	107.289	1.00	20.00	C
ATOM	4151	CD	ARG	C	491	107.365	78.564	107.006	1.00	20.00	C
ATOM	4152	NE	ARG	C	491	108.153	78.552	108.246	1.00	20.00	N
ATOM	4153	CZ	ARG	C	491	109.467	78.811	108.345	1.00	20.00	C
ATOM	4154	NH1	ARG	C	491	110.061	78.738	109.543	1.00	20.00	N
ATOM	4155	NH2	ARG	C	491	110.195	79.138	107.267	1.00	20.00	N1+
ATOM	4156	H	ARG	C	491	103.462	77.444	104.172	1.00	0.00	H
ATOM	4157	HA	ARG	C	491	103.506	77.024	106.992	1.00	0.00	H
ATOM	4158	HB2	ARG	C	491	105.496	77.193	105.551	1.00	0.00	H
ATOM	4159	HB3	ARG	C	491	105.193	78.894	105.297	1.00	0.00	H
ATOM	4160	HG2	ARG	C	491	105.492	79.324	107.693	1.00	0.00	H
ATOM	4161	HG3	ARG	C	491	105.707	77.630	108.061	1.00	0.00	H
ATOM	4162	HD2	ARG	C	491	107.736	77.875	106.249	1.00	0.00	H
ATOM	4163	HD3	ARG	C	491	107.503	79.570	106.607	1.00	0.00	H
ATOM	4164	HE	ARG	C	491	107.647	78.303	109.084	1.00	0.00	H
ATOM	4165	HH11	ARG	C	491	111.048	78.930	109.639	1.00	0.00	H
ATOM	4166	HH12	ARG	C	491	109.525	78.497	110.364	1.00	0.00	H
ATOM	4167	HH21	ARG	C	491	111.180	79.346	107.352	1.00	0.00	H
ATOM	4168	HH22	ARG	C	491	109.744	79.221	106.368	1.00	0.00	H
ATOM	4169	N	GLY	C	492	102.331	79.927	105.939	1.00	0.00	N
ATOM	4170	CA	GLY	C	492	101.521	81.088	106.310	1.00	0.00	C
ATOM	4171	C	GLY	C	492	100.094	80.675	106.708	1.00	0.00	C
ATOM	4172	O	GLY	C	492	99.490	81.338	107.552	1.00	0.00	O
ATOM	4173	H	GLY	C	492	102.494	79.785	104.952	1.00	0.00	H
ATOM	4174	HA2	GLY	C	492	101.996	81.639	107.123	1.00	0.00	H
ATOM	4175	HA3	GLY	C	492	101.468	81.763	105.455	1.00	0.00	H
ATOM	4176	N	ILE	C	493	99.571	79.573	106.137	1.00	30.00	N
ATOM	4177	CA	ILE	C	493	98.273	78.991	106.486	1.00	30.00	C
ATOM	4178	C	ILE	C	493	98.279	78.306	107.861	1.00	30.00	C
ATOM	4179	O	ILE	C	493	97.381	78.590	108.648	1.00	30.00	O

```
ATOM   4180  CB  ILE C 493      97.742  78.023 105.384  1.00 20.00           C
ATOM   4181  CG1 ILE C 493      97.185  78.852 104.208  1.00 20.00           C
ATOM   4182  CG2 ILE C 493      96.688  76.978 105.828  1.00 20.00           C
ATOM   4183  CD1 ILE C 493      97.047  78.070 102.897  1.00 20.00           C
ATOM   4184  H   ILE C 493     100.101  79.082 105.431  1.00  0.00           H
ATOM   4185  HA  ILE C 493      97.563  79.819 106.559  1.00  0.00           H
ATOM   4186  HB  ILE C 493      98.599  77.462 105.012  1.00  0.00           H
ATOM   4187 HG12 ILE C 493      96.216  79.271 104.482  1.00  0.00           H
ATOM   4188 HG13 ILE C 493      97.830  79.710 104.026  1.00  0.00           H
ATOM   4189 HG21 ILE C 493      96.336  76.386 104.984  1.00  0.00           H
ATOM   4190 HG22 ILE C 493      97.090  76.265 106.548  1.00  0.00           H
ATOM   4191 HG23 ILE C 493      95.818  77.458 106.277  1.00  0.00           H
ATOM   4192 HD11 ILE C 493      97.061  78.757 102.053  1.00  0.00           H
ATOM   4193 HD12 ILE C 493      97.863  77.360 102.757  1.00  0.00           H
ATOM   4194 HD13 ILE C 493      96.107  77.519 102.857  1.00  0.00           H
ATOM   4195  N   GLN C 494      99.278  77.447 108.141  1.00 30.00           N
ATOM   4196  CA  GLN C 494      99.381  76.727 109.417  1.00 30.00           C
ATOM   4197  C   GLN C 494      99.756  77.648 110.597  1.00 30.00           C
ATOM   4198  O   GLN C 494      99.402  77.324 111.729  1.00 30.00           O
ATOM   4199  CB  GLN C 494     100.309  75.500 109.276  1.00 20.00           C
ATOM   4200  CG  GLN C 494     101.821  75.799 109.252  1.00 20.00           C
ATOM   4201  CD  GLN C 494     102.680  74.584 108.883  1.00 20.00           C
ATOM   4202  NE2 GLN C 494     103.974  74.812 108.655  1.00 20.00           N
ATOM   4203  OE1 GLN C 494     102.199  73.454 108.816  1.00 20.00           O
ATOM   4204  H   GLN C 494      99.987  77.246 107.446  1.00  0.00           H
ATOM   4205  HA  GLN C 494      98.388  76.330 109.633  1.00  0.00           H
ATOM   4206  HB2 GLN C 494     100.020  74.953 108.377  1.00  0.00           H
ATOM   4207  HB3 GLN C 494     100.110  74.820 110.107  1.00  0.00           H
ATOM   4208  HG2 GLN C 494     102.011  76.592 108.534  1.00  0.00           H
ATOM   4209  HG3 GLN C 494     102.152  76.170 110.222  1.00  0.00           H
ATOM   4210 HE21 GLN C 494     104.585  74.045 108.415  1.00  0.00           H
ATOM   4211 HE22 GLN C 494     104.351  75.745 108.729  1.00  0.00           H
ATOM   4212  N   TYR C 495     100.400  78.795 110.310  1.00 30.00           N
ATOM   4213  CA  TYR C 495     100.604  79.901 111.248  1.00 30.00           C
ATOM   4214  C   TYR C 495      99.266  80.522 111.699  1.00 30.00           C
ATOM   4215  O   TYR C 495      99.035  80.666 112.900  1.00 30.00           O
ATOM   4216  CB  TYR C 495     101.543  80.948 110.600  1.00 20.00           C
ATOM   4217  CG  TYR C 495     101.792  82.203 111.421  1.00 20.00           C
ATOM   4218  CD1 TYR C 495     102.889  82.252 112.304  1.00 20.00           C
ATOM   4219  CD2 TYR C 495     100.924  83.313 111.323  1.00 20.00           C
ATOM   4220  CE1 TYR C 495     103.098  83.389 113.105  1.00 20.00           C
ATOM   4221  CE2 TYR C 495     101.117  84.439 112.147  1.00 20.00           C
ATOM   4222  CZ  TYR C 495     102.197  84.469 113.050  1.00 20.00           C
ATOM   4223  OH  TYR C 495     102.369  85.538 113.879  1.00 20.00           O
ATOM   4224  H   TYR C 495     100.683  78.972 109.356  1.00  0.00           H
ATOM   4225  HA  TYR C 495     101.102  79.498 112.133  1.00  0.00           H
ATOM   4226  HB2 TYR C 495     102.507  80.486 110.382  1.00  0.00           H
ATOM   4227  HB3 TYR C 495     101.145  81.259 109.635  1.00  0.00           H
ATOM   4228  HD1 TYR C 495     103.572  81.417 112.373  1.00  0.00           H
ATOM   4229  HD2 TYR C 495     100.089  83.291 110.638  1.00  0.00           H
ATOM   4230  HE1 TYR C 495     103.947  83.419 113.771  1.00  0.00           H
ATOM   4231  HE2 TYR C 495     100.431  85.271 112.086  1.00  0.00           H
ATOM   4232  HH  TYR C 495     101.697  86.212 113.761  1.00  0.00           H
ATOM   4233  N   PHE C 496      98.419  80.870 110.714  1.00 30.00           N
ATOM   4234  CA  PHE C 496      97.109  81.490 110.915  1.00 30.00           C
ATOM   4235  C   PHE C 496      96.066  80.535 111.527  1.00 30.00           C
ATOM   4236  O   PHE C 496      95.244  80.981 112.326  1.00 30.00           O
ATOM   4237  CB  PHE C 496      96.640  82.112 109.580  1.00 20.00           C
ATOM   4238  CG  PHE C 496      95.280  82.792 109.614  1.00 20.00           C
ATOM   4239  CD1 PHE C 496      95.166  84.105 110.118  1.00 20.00           C
ATOM   4240  CD2 PHE C 496      94.105  82.068 109.317  1.00 20.00           C
ATOM   4241  CE1 PHE C 496      93.914  84.687 110.269  1.00 20.00           C
ATOM   4242  CE2 PHE C 496      92.862  82.667 109.475  1.00 20.00           C
ATOM   4243  CZ  PHE C 496      92.767  83.971 109.946  1.00 20.00           C
ATOM   4244  H   PHE C 496      98.689  80.713 109.753  1.00  0.00           H
ATOM   4245  HA  PHE C 496      97.243  82.308 111.626  1.00  0.00           H
ATOM   4246  HB2 PHE C 496      97.371  82.857 109.264  1.00  0.00           H
ATOM   4247  HB3 PHE C 496      96.634  81.352 108.796  1.00  0.00           H
ATOM   4248  HD1 PHE C 496      96.053  84.659 110.387  1.00  0.00           H
ATOM   4249  HD2 PHE C 496      94.168  81.045 108.976  1.00  0.00           H
ATOM   4250  HE1 PHE C 496      93.831  85.696 110.643  1.00  0.00           H
```

ATOM	4251	HE2	PHE	C	496	91.965	82.114	109.241	1.00	0.00	H
ATOM	4252	HZ	PHE	C	496	91.797	84.429	110.069	1.00	0.00	H
ATOM	4253	N	LEU	C	497	96.125	79.247	111.150	1.00	30.00	N
ATOM	4254	CA	LEU	C	497	95.242	78.178	111.621	1.00	30.00	C
ATOM	4255	C	LEU	C	497	95.427	77.865	113.119	1.00	30.00	C
ATOM	4256	O	LEU	C	497	94.449	77.510	113.776	1.00	30.00	O
ATOM	4257	CB	LEU	C	497	95.466	76.937	110.725	1.00	20.00	C
ATOM	4258	CG	LEU	C	497	94.495	75.752	110.930	1.00	0.00	C
ATOM	4259	CD1	LEU	C	497	93.025	76.147	110.675	1.00	0.00	C
ATOM	4260	CD2	LEU	C	497	94.929	74.542	110.074	1.00	0.00	C
ATOM	4261	H	LEU	C	497	96.814	78.973	110.460	1.00	0.00	H
ATOM	4262	HA	LEU	C	497	94.219	78.532	111.484	1.00	0.00	H
ATOM	4263	HB2	LEU	C	497	95.405	77.238	109.679	1.00	0.00	H
ATOM	4264	HB3	LEU	C	497	96.487	76.587	110.880	1.00	0.00	H
ATOM	4265	HG	LEU	C	497	94.571	75.429	111.969	1.00	0.00	H
ATOM	4266	HD11	LEU	C	497	92.477	75.393	110.110	1.00	0.00	H
ATOM	4267	HD12	LEU	C	497	92.496	76.278	111.620	1.00	0.00	H
ATOM	4268	HD13	LEU	C	497	92.942	77.083	110.123	1.00	0.00	H
ATOM	4269	HD21	LEU	C	497	95.034	73.649	110.691	1.00	0.00	H
ATOM	4270	HD22	LEU	C	497	94.217	74.307	109.283	1.00	0.00	H
ATOM	4271	HD23	LEU	C	497	95.892	74.707	109.588	1.00	0.00	H
ATOM	4272	N	GLN	C	498	96.657	78.047	113.631	1.00	30.00	N
ATOM	4273	CA	GLN	C	498	96.993	77.971	115.051	1.00	30.00	C
ATOM	4274	C	GLN	C	498	96.463	79.177	115.846	1.00	30.00	C
ATOM	4275	O	GLN	C	498	95.638	78.997	116.740	1.00	30.00	O
ATOM	4276	CB	GLN	C	498	98.523	77.844	115.221	1.00	20.00	C
ATOM	4277	CG	GLN	C	498	99.081	76.447	114.896	1.00	20.00	C
ATOM	4278	CD	GLN	C	498	100.614	76.408	114.895	1.00	20.00	C
ATOM	4279	NE2	GLN	C	498	101.185	75.328	114.358	1.00	20.00	N
ATOM	4280	OE1	GLN	C	498	101.280	77.324	115.376	1.00	20.00	O
ATOM	4281	H	GLN	C	498	97.408	78.328	113.016	1.00	0.00	H
ATOM	4282	HA	GLN	C	498	96.530	77.074	115.467	1.00	0.00	H
ATOM	4283	HB2	GLN	C	498	99.016	78.587	114.592	1.00	0.00	H
ATOM	4284	HB3	GLN	C	498	98.802	78.090	116.247	1.00	0.00	H
ATOM	4285	HG2	GLN	C	498	98.713	75.724	115.625	1.00	0.00	H
ATOM	4286	HG3	GLN	C	498	98.720	76.116	113.923	1.00	0.00	H
ATOM	4287	HE21	GLN	C	498	102.191	75.248	114.338	1.00	0.00	H
ATOM	4288	HE22	GLN	C	498	100.618	74.588	113.970	1.00	0.00	H
ATOM	4289	N	ARG	C	499	96.998	80.369	115.531	1.00	30.00	N
ATOM	4290	CA	ARG	C	499	96.881	81.568	116.363	1.00	30.00	C
ATOM	4291	C	ARG	C	499	95.579	82.348	116.127	1.00	30.00	C
ATOM	4292	O	ARG	C	499	94.940	82.742	117.101	1.00	30.00	O
ATOM	4293	CB	ARG	C	499	98.112	82.476	116.139	1.00	20.00	C
ATOM	4294	CG	ARG	C	499	99.455	81.748	116.324	1.00	20.00	C
ATOM	4295	CD	ARG	C	499	100.673	82.671	116.195	1.00	20.00	C
ATOM	4296	NE	ARG	C	499	101.904	81.880	116.080	1.00	20.00	N
ATOM	4297	CZ	ARG	C	499	103.163	82.341	116.142	1.00	20.00	C
ATOM	4298	NH1	ARG	C	499	103.432	83.636	116.363	1.00	20.00	N
ATOM	4299	NH2	ARG	C	499	104.166	81.473	115.970	1.00	20.00	N1+
ATOM	4300	H	ARG	C	499	97.649	80.427	114.760	1.00	0.00	H
ATOM	4301	HA	ARG	C	499	96.890	81.260	117.411	1.00	0.00	H
ATOM	4302	HB2	ARG	C	499	98.065	83.327	116.819	1.00	0.00	H
ATOM	4303	HB3	ARG	C	499	98.081	82.890	115.129	1.00	0.00	H
ATOM	4304	HG2	ARG	C	499	99.447	81.356	117.343	1.00	0.00	H
ATOM	4305	HG3	ARG	C	499	99.564	80.883	115.669	1.00	0.00	H
ATOM	4306	HD2	ARG	C	499	100.560	83.425	115.416	1.00	0.00	H
ATOM	4307	HD3	ARG	C	499	100.785	83.203	117.140	1.00	0.00	H
ATOM	4308	HE	ARG	C	499	101.770	80.898	115.887	1.00	0.00	H
ATOM	4309	HH11	ARG	C	499	104.388	83.959	116.398	1.00	0.00	H
ATOM	4310	HH12	ARG	C	499	102.678	84.300	116.462	1.00	0.00	H
ATOM	4311	HH21	ARG	C	499	105.137	81.763	116.023	1.00	0.00	H
ATOM	4312	HH22	ARG	C	499	103.973	80.494	115.818	1.00	0.00	H
ATOM	4313	N	ARG	C	500	95.234	82.560	114.845	1.00	30.00	N
ATOM	4314	CA	ARG	C	500	94.108	83.355	114.343	1.00	30.00	C
ATOM	4315	C	ARG	C	500	94.070	84.792	114.927	1.00	30.00	C
ATOM	4316	O	ARG	C	500	93.256	85.049	115.815	1.00	30.00	O
ATOM	4317	CB	ARG	C	500	92.782	82.574	114.527	1.00	20.00	C
ATOM	4318	CG	ARG	C	500	91.565	83.220	113.830	1.00	20.00	C
ATOM	4319	CD	ARG	C	500	90.264	82.421	114.000	1.00	20.00	C
ATOM	4320	NE	ARG	C	500	90.277	81.166	113.233	1.00	20.00	N
ATOM	4321	CZ	ARG	C	500	89.345	80.200	113.300	1.00	20.00	C

```
ATOM   4322  NH1 ARG C 500      89.486  79.101 112.549  1.00 20.00           N
ATOM   4323  NH2 ARG C 500      88.276  80.315 114.101  1.00 20.00           N1+
ATOM   4324  H   ARG C 500      95.805  82.141 114.124  1.00  0.00           H
ATOM   4325  HA  ARG C 500      94.261  83.450 113.267  1.00  0.00           H
ATOM   4326  HB2 ARG C 500      92.913  81.563 114.140  1.00  0.00           H
ATOM   4327  HB3 ARG C 500      92.570  82.460 115.592  1.00  0.00           H
ATOM   4328  HG2 ARG C 500      91.395  84.255 114.128  1.00  0.00           H
ATOM   4329  HG3 ARG C 500      91.805  83.255 112.767  1.00  0.00           H
ATOM   4330  HD2 ARG C 500      90.206  82.100 115.041  1.00  0.00           H
ATOM   4331  HD3 ARG C 500      89.379  83.030 113.816  1.00  0.00           H
ATOM   4332  HE  ARG C 500      91.059  81.042 112.605  1.00  0.00           H
ATOM   4333 HH11 ARG C 500      88.798  78.363 112.585  1.00  0.00           H
ATOM   4334 HH12 ARG C 500      90.289  78.994 111.945  1.00  0.00           H
ATOM   4335 HH21 ARG C 500      87.582  79.583 114.146  1.00  0.00           H
ATOM   4336 HH22 ARG C 500      88.162  81.140 114.672  1.00  0.00           H
ATOM   4337  N   PRO C 501      94.946  85.708 114.440  1.00 30.00           N
ATOM   4338  CA  PRO C 501      94.978  87.121 114.888  1.00 30.00           C
ATOM   4339  C   PRO C 501      93.712  87.977 114.634  1.00 30.00           C
ATOM   4340  O   PRO C 501      93.685  89.129 115.065  1.00 30.00           O
ATOM   4341  CB  PRO C 501      96.212  87.706 114.178  1.00 20.00           C
ATOM   4342  CG  PRO C 501      97.078  86.506 113.839  1.00  0.00           C
ATOM   4343  CD  PRO C 501      96.041  85.445 113.505  1.00  0.00           C
ATOM   4344  HA  PRO C 501      95.168  87.109 115.963  1.00  0.00           H
ATOM   4345  HB2 PRO C 501      95.915  88.200 113.253  1.00  0.00           H
ATOM   4346  HB3 PRO C 501      96.745  88.436 114.788  1.00  0.00           H
ATOM   4347  HG2 PRO C 501      97.790  86.692 113.034  1.00  0.00           H
ATOM   4348  HG3 PRO C 501      97.642  86.200 114.721  1.00  0.00           H
ATOM   4349  HD2 PRO C 501      95.668  85.587 112.492  1.00  0.00           H
ATOM   4350  HD3 PRO C 501      96.480  84.451 113.574  1.00  0.00           H
ATOM   4351  N   SER C 502      92.689  87.409 113.968  1.00 30.00           N
ATOM   4352  CA  SER C 502      91.336  87.953 113.798  1.00 30.00           C
ATOM   4353  C   SER C 502      91.286  89.276 112.998  1.00 30.00           C
ATOM   4354  O   SER C 502      90.419  90.114 113.247  1.00 30.00           O
ATOM   4355  CB  SER C 502      90.620  88.005 115.172  1.00 20.00           C
ATOM   4356  OG  SER C 502      89.230  88.231 115.041  1.00  0.00           O
ATOM   4357  H   SER C 502      92.804  86.459 113.646  1.00  0.00           H
ATOM   4358  HA  SER C 502      90.811  87.219 113.185  1.00  0.00           H
ATOM   4359  HB2 SER C 502      90.752  87.060 115.700  1.00  0.00           H
ATOM   4360  HB3 SER C 502      91.044  88.784 115.807  1.00  0.00           H
ATOM   4361  HG  SER C 502      89.103  89.106 114.663  1.00  0.00           H
ATOM   4362  N   LEU C 503      92.223  89.424 112.044  1.00 30.00           N
ATOM   4363  CA  LEU C 503      92.372  90.513 111.069  1.00 30.00           C
ATOM   4364  C   LEU C 503      92.836  91.870 111.641  1.00 30.00           C
ATOM   4365  O   LEU C 503      93.423  92.641 110.882  1.00 30.00           O
ATOM   4366  CB  LEU C 503      91.104  90.677 110.188  1.00 20.00           C
ATOM   4367  CG  LEU C 503      90.661  89.399 109.434  1.00  0.00           C
ATOM   4368  CD1 LEU C 503      89.311  89.619 108.718  1.00  0.00           C
ATOM   4369  CD2 LEU C 503      91.748  88.873 108.473  1.00  0.00           C
ATOM   4370  H   LEU C 503      92.899  88.681 111.942  1.00  0.00           H
ATOM   4371  HA  LEU C 503      93.184  90.189 110.420  1.00  0.00           H
ATOM   4372  HB2 LEU C 503      90.280  91.034 110.806  1.00  0.00           H
ATOM   4373  HB3 LEU C 503      91.276  91.467 109.455  1.00  0.00           H
ATOM   4374  HG  LEU C 503      90.481  88.618 110.173  1.00  0.00           H
ATOM   4375 HD11 LEU C 503      88.587  88.858 109.012  1.00  0.00           H
ATOM   4376 HD12 LEU C 503      88.872  90.587 108.961  1.00  0.00           H
ATOM   4377 HD13 LEU C 503      89.399  89.580 107.632  1.00  0.00           H
ATOM   4378 HD21 LEU C 503      91.336  88.503 107.534  1.00  0.00           H
ATOM   4379 HD22 LEU C 503      92.473  89.646 108.219  1.00  0.00           H
ATOM   4380 HD23 LEU C 503      92.296  88.046 108.927  1.00  0.00           H
ATOM   4381  N   LYS C 504      92.601  92.146 112.938  1.00 30.00           N
ATOM   4382  CA  LYS C 504      92.995  93.394 113.595  1.00 30.00           C
ATOM   4383  C   LYS C 504      94.444  93.316 114.107  1.00 30.00           C
ATOM   4384  O   LYS C 504      95.252  94.166 113.736  1.00 30.00           O
ATOM   4385  CB  LYS C 504      92.001  93.737 114.726  1.00 20.00           C
ATOM   4386  CG  LYS C 504      92.148  95.185 115.240  1.00  0.00           C
ATOM   4387  CD  LYS C 504      91.350  95.486 116.518  1.00  0.00           C
ATOM   4388  CE  LYS C 504      92.011  94.908 117.779  1.00  0.00           C
ATOM   4389  NZ  LYS C 504      91.291  95.292 119.004  1.00  0.00           N1+
ATOM   4390  H   LYS C 504      92.095  91.476 113.500  1.00  0.00           H
ATOM   4391  HA  LYS C 504      92.945  94.204 112.864  1.00  0.00           H
ATOM   4392  HB2 LYS C 504      90.981  93.616 114.358  1.00  0.00           H
```

```
ATOM   4393  HB3 LYS C 504      92.109  93.019 115.540  1.00  0.00           H
ATOM   4394  HG2 LYS C 504      93.196  95.430 115.415  1.00  0.00           H
ATOM   4395  HG3 LYS C 504      91.818  95.864 114.454  1.00  0.00           H
ATOM   4396  HD2 LYS C 504      91.242  96.567 116.623  1.00  0.00           H
ATOM   4397  HD3 LYS C 504      90.338  95.089 116.420  1.00  0.00           H
ATOM   4398  HE2 LYS C 504      92.062  93.820 117.732  1.00  0.00           H
ATOM   4399  HE3 LYS C 504      93.036  95.271 117.863  1.00  0.00           H
ATOM   4400  HZ1 LYS C 504      91.803  94.918 119.797  1.00  0.00           H
ATOM   4401  HZ2 LYS C 504      91.256  96.298 119.083  1.00  0.00           H
ATOM   4402  HZ3 LYS C 504      90.356  94.913 118.994  1.00  0.00           H
ATOM   4403  N   SER C 505      94.753  92.285 114.917  1.00 30.00           N
ATOM   4404  CA  SER C 505      96.102  92.012 115.432  1.00 30.00           C
ATOM   4405  C   SER C 505      97.062  91.474 114.343  1.00 30.00           C
ATOM   4406  O   SER C 505      98.274  91.474 114.559  1.00 30.00           O
ATOM   4407  CB  SER C 505      95.995  91.071 116.654  1.00 20.00           C
ATOM   4408  OG  SER C 505      97.229  90.900 117.323  1.00  0.00           O
ATOM   4409  H   SER C 505      94.040  91.614 115.167  1.00  0.00           H
ATOM   4410  HA  SER C 505      96.512  92.957 115.789  1.00  0.00           H
ATOM   4411  HB2 SER C 505      95.278  91.468 117.374  1.00  0.00           H
ATOM   4412  HB3 SER C 505      95.637  90.087 116.360  1.00  0.00           H
ATOM   4413  HG  SER C 505      97.408  91.683 117.852  1.00  0.00           H
ATOM   4414  N   LEU C 506      96.508  91.064 113.190  1.00 30.00           N
ATOM   4415  CA  LEU C 506      97.242  90.581 112.026  1.00 30.00           C
ATOM   4416  C   LEU C 506      98.265  91.628 111.554  1.00 30.00           C
ATOM   4417  O   LEU C 506      99.454  91.463 111.801  1.00 30.00           O
ATOM   4418  CB  LEU C 506      96.229  90.152 110.932  1.00 20.00           C
ATOM   4419  CG  LEU C 506      96.835  89.447 109.697  1.00  0.00           C
ATOM   4420  CD1 LEU C 506      97.607  88.161 110.056  1.00  0.00           C
ATOM   4421  CD2 LEU C 506      95.758  89.176 108.630  1.00  0.00           C
ATOM   4422  H   LEU C 506      95.510  91.163 113.097  1.00  0.00           H
ATOM   4423  HA  LEU C 506      97.805  89.705 112.351  1.00  0.00           H
ATOM   4424  HB2 LEU C 506      95.486  89.487 111.370  1.00  0.00           H
ATOM   4425  HB3 LEU C 506      95.675  91.031 110.597  1.00  0.00           H
ATOM   4426  HG  LEU C 506      97.541  90.139 109.239  1.00  0.00           H
ATOM   4427 HD11 LEU C 506      97.406  87.345 109.361  1.00  0.00           H
ATOM   4428 HD12 LEU C 506      98.683  88.335 110.029  1.00  0.00           H
ATOM   4429 HD13 LEU C 506      97.358  87.797 111.051  1.00  0.00           H
ATOM   4430 HD21 LEU C 506      96.143  89.394 107.633  1.00  0.00           H
ATOM   4431 HD22 LEU C 506      95.419  88.140 108.630  1.00  0.00           H
ATOM   4432 HD23 LEU C 506      94.876  89.801 108.769  1.00  0.00           H
ATOM   4433  N   PHE C 507      97.787  92.722 110.959  1.00 30.00           N
ATOM   4434  CA  PHE C 507      98.646  93.761 110.385  1.00 30.00           C
ATOM   4435  C   PHE C 507      99.388  94.606 111.440  1.00 30.00           C
ATOM   4436  O   PHE C 507     100.368  95.262 111.092  1.00 30.00           O
ATOM   4437  CB  PHE C 507      97.802  94.650 109.451  1.00 20.00           C
ATOM   4438  CG  PHE C 507      97.017  93.866 108.411  1.00  0.00           C
ATOM   4439  CD1 PHE C 507      95.674  93.510 108.659  1.00  0.00           C
ATOM   4440  CD2 PHE C 507      97.671  93.323 107.285  1.00  0.00           C
ATOM   4441  CE1 PHE C 507      95.002  92.670 107.783  1.00  0.00           C
ATOM   4442  CE2 PHE C 507      96.981  92.484 106.420  1.00  0.00           C
ATOM   4443  CZ  PHE C 507      95.652  92.161 106.666  1.00  0.00           C
ATOM   4444  H   PHE C 507      96.798  92.808 110.776  1.00  0.00           H
ATOM   4445  HA  PHE C 507      99.412  93.270 109.782  1.00  0.00           H
ATOM   4446  HB2 PHE C 507      97.109  95.260 110.033  1.00  0.00           H
ATOM   4447  HB3 PHE C 507      98.459  95.353 108.935  1.00  0.00           H
ATOM   4448  HD1 PHE C 507      95.161  93.888 109.532  1.00  0.00           H
ATOM   4449  HD2 PHE C 507      98.712  93.544 107.098  1.00  0.00           H
ATOM   4450  HE1 PHE C 507      93.972  92.407 107.979  1.00  0.00           H
ATOM   4451  HE2 PHE C 507      97.482  92.071 105.556  1.00  0.00           H
ATOM   4452  HZ  PHE C 507      95.124  91.503 105.991  1.00  0.00           H
ATOM   4453  N   VAL C 508      98.917  94.585 112.695  1.00 30.00           N
ATOM   4454  CA  VAL C 508      99.532  95.303 113.814  1.00 30.00           C
ATOM   4455  C   VAL C 508     100.836  94.640 114.303  1.00 30.00           C
ATOM   4456  O   VAL C 508     101.824  95.354 114.487  1.00 30.00           O
ATOM   4457  CB  VAL C 508      98.526  95.447 114.998  1.00 20.00           C
ATOM   4458  CG1 VAL C 508      99.130  95.820 116.371  1.00  0.00           C
ATOM   4459  CG2 VAL C 508      97.419  96.454 114.642  1.00  0.00           C
ATOM   4460  H   VAL C 508      98.103  94.030 112.917  1.00  0.00           H
ATOM   4461  HA  VAL C 508      99.788  96.306 113.467  1.00  0.00           H
ATOM   4462  HB  VAL C 508      98.040  94.481 115.128  1.00  0.00           H
ATOM   4463 HG11 VAL C 508      98.346  95.997 117.108  1.00  0.00           H
```

ATOM	4464	HG12	VAL	C	508	99.759	95.026	116.777	1.00	0.00	H
ATOM	4465	HG13	VAL	C	508	99.731	96.727	116.306	1.00	0.00	H
ATOM	4466	HG21	VAL	C	508	96.642	96.475	115.408	1.00	0.00	H
ATOM	4467	HG22	VAL	C	508	97.820	97.464	114.548	1.00	0.00	H
ATOM	4468	HG23	VAL	C	508	96.936	96.207	113.697	1.00	0.00	H
ATOM	4469	N	ASP	C	509	100.805	93.312	114.515	1.00	30.00	N
ATOM	4470	CA	ASP	C	509	101.885	92.559	115.157	1.00	30.00	C
ATOM	4471	C	ASP	C	509	102.561	91.603	114.158	1.00	30.00	C
ATOM	4472	O	ASP	C	509	103.782	91.663	114.015	1.00	30.00	O
ATOM	4473	CB	ASP	C	509	101.363	91.803	116.405	1.00	20.00	C
ATOM	4474	CG	ASP	C	509	102.450	91.183	117.294	1.00	0.00	C
ATOM	4475	OD1	ASP	C	509	103.585	91.708	117.305	1.00	0.00	O
ATOM	4476	OD2	ASP	C	509	102.095	90.247	118.043	1.00	0.00	O1-
ATOM	4477	H	ASP	C	509	99.955	92.796	114.326	1.00	0.00	H
ATOM	4478	HA	ASP	C	509	102.655	93.256	115.493	1.00	0.00	H
ATOM	4479	HB2	ASP	C	509	100.775	92.483	117.022	1.00	0.00	H
ATOM	4480	HB3	ASP	C	509	100.691	91.006	116.084	1.00	0.00	H
ATOM	4481	N	SER	C	510	101.779	90.831	113.396	1.00	30.00	N
ATOM	4482	CA	SER	C	510	102.232	89.737	112.531	1.00	30.00	C
ATOM	4483	C	SER	C	510	102.928	90.179	111.211	1.00	30.00	C
ATOM	4484	O	SER	C	510	102.705	89.564	110.163	1.00	30.00	O
ATOM	4485	CB	SER	C	510	101.023	88.799	112.308	1.00	20.00	C
ATOM	4486	OG	SER	C	510	101.410	87.524	111.856	1.00	20.00	O
ATOM	4487	H	SER	C	510	100.779	90.993	113.398	1.00	0.00	H
ATOM	4488	HA	SER	C	510	102.981	89.179	113.097	1.00	0.00	H
ATOM	4489	HB2	SER	C	510	100.339	89.209	111.569	1.00	0.00	H
ATOM	4490	HB3	SER	C	510	100.456	88.669	113.231	1.00	0.00	H
ATOM	4491	HG	SER	C	510	101.857	87.651	111.008	1.00	0.00	H
ATOM	4492	N	TYR	C	511	103.777	91.214	111.278	1.00	30.00	N
ATOM	4493	CA	TYR	C	511	104.563	91.760	110.167	1.00	30.00	C
ATOM	4494	C	TYR	C	511	105.570	90.724	109.617	1.00	30.00	C
ATOM	4495	O	TYR	C	511	105.908	89.755	110.301	1.00	30.00	O
ATOM	4496	CB	TYR	C	511	105.276	93.054	110.627	1.00	20.00	C
ATOM	4497	CG	TYR	C	511	105.449	94.113	109.545	1.00	0.00	C
ATOM	4498	CD1	TYR	C	511	106.556	94.088	108.668	1.00	0.00	C
ATOM	4499	CD2	TYR	C	511	104.503	95.155	109.433	1.00	0.00	C
ATOM	4500	CE1	TYR	C	511	106.705	95.081	107.679	1.00	0.00	C
ATOM	4501	CE2	TYR	C	511	104.668	96.163	108.465	1.00	0.00	C
ATOM	4502	CZ	TYR	C	511	105.763	96.124	107.583	1.00	0.00	C
ATOM	4503	OH	TYR	C	511	105.892	97.093	106.635	1.00	0.00	O
ATOM	4504	H	TYR	C	511	103.958	91.591	112.206	1.00	0.00	H
ATOM	4505	HA	TYR	C	511	103.860	92.005	109.368	1.00	0.00	H
ATOM	4506	HB2	TYR	C	511	104.692	93.527	111.420	1.00	0.00	H
ATOM	4507	HB3	TYR	C	511	106.239	92.832	111.090	1.00	0.00	H
ATOM	4508	HD1	TYR	C	511	107.306	93.318	108.752	1.00	0.00	H
ATOM	4509	HD2	TYR	C	511	103.654	95.202	110.102	1.00	0.00	H
ATOM	4510	HE1	TYR	C	511	107.556	95.047	107.013	1.00	0.00	H
ATOM	4511	HE2	TYR	C	511	103.949	96.965	108.393	1.00	0.00	H
ATOM	4512	HH	TYR	C	511	106.243	96.748	105.803	1.00	0.00	H
ATOM	4513	N	SER	C	512	106.015	90.922	108.370	1.00	30.00	N
ATOM	4514	CA	SER	C	512	106.838	90.010	107.565	1.00	30.00	C
ATOM	4515	C	SER	C	512	106.099	88.717	107.151	1.00	30.00	C
ATOM	4516	O	SER	C	512	106.029	88.435	105.958	1.00	30.00	O
ATOM	4517	CB	SER	C	512	108.226	89.724	108.198	1.00	20.00	C
ATOM	4518	OG	SER	C	512	109.231	90.563	107.653	1.00	0.00	O
ATOM	4519	H	SER	C	512	105.729	91.765	107.894	1.00	0.00	H
ATOM	4520	HA	SER	C	512	107.015	90.537	106.625	1.00	0.00	H
ATOM	4521	HB2	SER	C	512	108.230	89.831	109.284	1.00	0.00	H
ATOM	4522	HB3	SER	C	512	108.527	88.692	107.997	1.00	0.00	H
ATOM	4523	HG	SER	C	512	109.251	91.398	108.143	1.00	0.00	H
ATOM	4524	N	GLU	C	513	105.505	87.979	108.101	1.00	30.00	N
ATOM	4525	CA	GLU	C	513	104.836	86.691	107.868	1.00	30.00	C
ATOM	4526	C	GLU	C	513	103.684	86.786	106.844	1.00	30.00	C
ATOM	4527	O	GLU	C	513	103.588	85.957	105.937	1.00	30.00	O
ATOM	4528	CB	GLU	C	513	104.387	86.103	109.228	1.00	20.00	C
ATOM	4529	CG	GLU	C	513	103.741	84.697	109.187	1.00	20.00	C
ATOM	4530	CD	GLU	C	513	104.649	83.587	108.648	1.00	20.00	C
ATOM	4531	OE1	GLU	C	513	105.886	83.763	108.686	1.00	20.00	O
ATOM	4532	OE2	GLU	C	513	104.088	82.563	108.206	1.00	20.00	O1-
ATOM	4533	H	GLU	C	513	105.556	88.310	109.062	1.00	0.00	H
ATOM	4534	HA	GLU	C	513	105.589	86.032	107.433	1.00	0.00	H

```
ATOM   4535  HB2 GLU C 513     105.244  86.070 109.903  1.00  0.00           H
ATOM   4536  HB3 GLU C 513     103.681  86.789 109.698  1.00  0.00           H
ATOM   4537  HG2 GLU C 513     102.818  84.713 108.606  1.00  0.00           H
ATOM   4538  HG3 GLU C 513     103.453  84.414 110.199  1.00  0.00           H
ATOM   4539  N   ILE C 514     102.894  87.864 106.949  1.00  0.00           N
ATOM   4540  CA  ILE C 514     101.875  88.251 105.969  1.00  0.00           C
ATOM   4541  C   ILE C 514     102.467  88.414 104.552  1.00  0.00           C
ATOM   4542  O   ILE C 514     101.903  87.901 103.589  1.00  0.00           O
ATOM   4543  CB  ILE C 514     101.195  89.594 106.383  1.00 20.00           C
ATOM   4544  CG1 ILE C 514     100.423  89.448 107.712  1.00 20.00           C
ATOM   4545  CG2 ILE C 514     100.268  90.235 105.322  1.00 20.00           C
ATOM   4546  CD1 ILE C 514     100.211  90.785 108.433  1.00 20.00           C
ATOM   4547  H   ILE C 514     103.022  88.480 107.741  1.00  0.00           H
ATOM   4548  HA  ILE C 514     101.122  87.461 105.933  1.00  0.00           H
ATOM   4549  HB  ILE C 514     102.003  90.307 106.556  1.00  0.00           H
ATOM   4550 HG12 ILE C 514      99.457  88.975 107.526  1.00  0.00           H
ATOM   4551 HG13 ILE C 514     100.940  88.775 108.395  1.00  0.00           H
ATOM   4552 HG21 ILE C 514      99.803  91.144 105.702  1.00  0.00           H
ATOM   4553 HG22 ILE C 514     100.802  90.525 104.416  1.00  0.00           H
ATOM   4554 HG23 ILE C 514      99.468  89.553 105.033  1.00  0.00           H
ATOM   4555 HD11 ILE C 514     100.188  90.630 109.509  1.00  0.00           H
ATOM   4556 HD12 ILE C 514     101.009  91.499 108.233  1.00  0.00           H
ATOM   4557 HD13 ILE C 514      99.268  91.244 108.143  1.00  0.00           H
ATOM   4558  N   LEU C 515     103.618  89.085 104.460  1.00  0.00           N
ATOM   4559  CA  LEU C 515     104.297  89.406 103.212  1.00  0.00           C
ATOM   4560  C   LEU C 515     104.910  88.158 102.548  1.00  0.00           C
ATOM   4561  O   LEU C 515     104.891  88.067 101.320  1.00  0.00           O
ATOM   4562  CB  LEU C 515     105.337  90.527 103.438  1.00 20.00           C
ATOM   4563  CG  LEU C 515     104.799  91.899 103.921  1.00 20.00           C
ATOM   4564  CD1 LEU C 515     103.676  92.462 103.027  1.00 20.00           C
ATOM   4565  CD2 LEU C 515     104.444  91.960 105.422  1.00 20.00           C
ATOM   4566  H   LEU C 515     104.116  89.336 105.301  1.00  0.00           H
ATOM   4567  HA  LEU C 515     103.544  89.781 102.522  1.00  0.00           H
ATOM   4568  HB2 LEU C 515     106.138  90.189 104.095  1.00  0.00           H
ATOM   4569  HB3 LEU C 515     105.829  90.700 102.482  1.00  0.00           H
ATOM   4570  HG  LEU C 515     105.646  92.577 103.793  1.00  0.00           H
ATOM   4571 HD11 LEU C 515     103.819  93.530 102.859  1.00  0.00           H
ATOM   4572 HD12 LEU C 515     103.663  91.993 102.044  1.00  0.00           H
ATOM   4573 HD13 LEU C 515     102.686  92.327 103.463  1.00  0.00           H
ATOM   4574 HD21 LEU C 515     103.389  92.174 105.594  1.00  0.00           H
ATOM   4575 HD22 LEU C 515     104.673  91.031 105.935  1.00  0.00           H
ATOM   4576 HD23 LEU C 515     105.012  92.751 105.915  1.00  0.00           H
ATOM   4577  N   PHE C 516     105.376  87.185 103.347  1.00  0.00           N
ATOM   4578  CA  PHE C 516     105.745  85.837 102.884  1.00  0.00           C
ATOM   4579  C   PHE C 516     104.552  85.074 102.274  1.00  0.00           C
ATOM   4580  O   PHE C 516     104.690  84.444 101.222  1.00  0.00           O
ATOM   4581  CB  PHE C 516     106.423  85.018 104.008  1.00 20.00           C
ATOM   4582  CG  PHE C 516     107.930  85.190 104.141  1.00 20.00           C
ATOM   4583  CD1 PHE C 516     108.804  84.335 103.436  1.00 20.00           C
ATOM   4584  CD2 PHE C 516     108.480  86.269 104.865  1.00 20.00           C
ATOM   4585  CE1 PHE C 516     110.178  84.506 103.538  1.00 20.00           C
ATOM   4586  CE2 PHE C 516     109.856  86.432 104.941  1.00 20.00           C
ATOM   4587  CZ  PHE C 516     110.704  85.548 104.289  1.00 20.00           C
ATOM   4588  H   PHE C 516     105.438  87.367 104.344  1.00  0.00           H
ATOM   4589  HA  PHE C 516     106.471  85.942 102.078  1.00  0.00           H
ATOM   4590  HB2 PHE C 516     105.963  85.247 104.969  1.00  0.00           H
ATOM   4591  HB3 PHE C 516     106.240  83.953 103.853  1.00  0.00           H
ATOM   4592  HD1 PHE C 516     108.415  83.535 102.824  1.00  0.00           H
ATOM   4593  HD2 PHE C 516     107.840  86.969 105.372  1.00  0.00           H
ATOM   4594  HE1 PHE C 516     110.834  83.827 103.017  1.00  0.00           H
ATOM   4595  HE2 PHE C 516     110.267  87.259 105.498  1.00  0.00           H
ATOM   4596  HZ  PHE C 516     111.773  85.690 104.347  1.00  0.00           H
ATOM   4597  N   PHE C 517     103.373  85.189 102.900  1.00  0.00           N
ATOM   4598  CA  PHE C 517     102.138  84.608 102.382  1.00  0.00           C
ATOM   4599  C   PHE C 517     101.624  85.332 101.121  1.00  0.00           C
ATOM   4600  O   PHE C 517     101.142  84.661 100.212  1.00  0.00           O
ATOM   4601  CB  PHE C 517     101.085  84.502 103.505  1.00 20.00           C
ATOM   4602  CG  PHE C 517      99.774  83.868 103.074  1.00 20.00           C
ATOM   4603  CD1 PHE C 517      99.709  82.475 102.861  1.00 20.00           C
ATOM   4604  CD2 PHE C 517      98.677  84.672 102.695  1.00 20.00           C
ATOM   4605  CE1 PHE C 517      98.563  81.908 102.323  1.00 20.00           C
```

```
ATOM   4606  CE2 PHE C 517      97.535  84.082 102.168  1.00 20.00           C
ATOM   4607  CZ  PHE C 517      97.478  82.706 101.984  1.00 20.00           C
ATOM   4608  H   PHE C 517     103.289  85.769 103.724  1.00  0.00           H
ATOM   4609  HA  PHE C 517     102.368  83.584 102.086  1.00  0.00           H
ATOM   4610  HB2 PHE C 517     101.495  83.888 104.309  1.00  0.00           H
ATOM   4611  HB3 PHE C 517     100.888  85.479 103.948  1.00  0.00           H
ATOM   4612  HD1 PHE C 517     100.557  81.848 103.097  1.00  0.00           H
ATOM   4613  HD2 PHE C 517      98.726  85.746 102.802  1.00  0.00           H
ATOM   4614  HE1 PHE C 517      98.521  80.843 102.153  1.00  0.00           H
ATOM   4615  HE2 PHE C 517      96.694  84.698 101.885  1.00  0.00           H
ATOM   4616  HZ  PHE C 517      96.592  82.256 101.562  1.00  0.00           H
ATOM   4617  N   VAL C 518     101.791  86.660 101.034  1.00  0.00           N
ATOM   4618  CA  VAL C 518     101.478  87.452  99.839  1.00  0.00           C
ATOM   4619  C   VAL C 518     102.420  87.127  98.656  1.00  0.00           C
ATOM   4620  O   VAL C 518     101.933  86.889  97.550  1.00  0.00           O
ATOM   4621  CB  VAL C 518     101.481  88.980 100.140  1.00 20.00           C
ATOM   4622  CG1 VAL C 518     101.491  89.902  98.904  1.00 20.00           C
ATOM   4623  CG2 VAL C 518     100.286  89.361 101.035  1.00 20.00           C
ATOM   4624  H   VAL C 518     102.101  87.168 101.857  1.00  0.00           H
ATOM   4625  HA  VAL C 518     100.468  87.180  99.526  1.00  0.00           H
ATOM   4626  HB  VAL C 518     102.387  89.204 100.700  1.00  0.00           H
ATOM   4627 HG11 VAL C 518     101.333  90.944  99.182  1.00  0.00           H
ATOM   4628 HG12 VAL C 518     102.445  89.859  98.378  1.00  0.00           H
ATOM   4629 HG13 VAL C 518     100.705  89.631  98.196  1.00  0.00           H
ATOM   4630 HG21 VAL C 518     100.353  90.399 101.363  1.00  0.00           H
ATOM   4631 HG22 VAL C 518      99.342  89.242 100.501  1.00  0.00           H
ATOM   4632 HG23 VAL C 518     100.226  88.744 101.929  1.00  0.00           H
ATOM   4633  N   GLN C 519     103.736  87.040  98.919  1.00  0.00           N
ATOM   4634  CA  GLN C 519     104.764  86.559  97.985  1.00  0.00           C
ATOM   4635  C   GLN C 519     104.384  85.218  97.340  1.00  0.00           C
ATOM   4636  O   GLN C 519     104.470  85.068  96.120  1.00  0.00           O
ATOM   4637  CB  GLN C 519     106.122  86.473  98.725  1.00 20.00           C
ATOM   4638  CG  GLN C 519     107.259  85.651  98.060  1.00 20.00           C
ATOM   4639  CD  GLN C 519     108.459  85.463  98.988  1.00 20.00           C
ATOM   4640  NE2 GLN C 519     109.619  85.127  98.447  1.00 20.00           N
ATOM   4641  OE1 GLN C 519     108.359  85.636 100.198  1.00 20.00           O
ATOM   4642  H   GLN C 519     104.061  87.284  99.852  1.00  0.00           H
ATOM   4643  HA  GLN C 519     104.853  87.294  97.183  1.00  0.00           H
ATOM   4644  HB2 GLN C 519     106.489  87.482  98.909  1.00  0.00           H
ATOM   4645  HB3 GLN C 519     105.935  86.049  99.709  1.00  0.00           H
ATOM   4646  HG2 GLN C 519     106.933  84.645  97.796  1.00  0.00           H
ATOM   4647  HG3 GLN C 519     107.578  86.136  97.135  1.00  0.00           H
ATOM   4648 HE21 GLN C 519     110.436  85.138  99.057  1.00  0.00           H
ATOM   4649 HE22 GLN C 519     109.730  84.960  97.458  1.00  0.00           H
ATOM   4650  N   SER C 520     103.963  84.281  98.195  1.00  0.00           N
ATOM   4651  CA  SER C 520     103.580  82.927  97.802  1.00  0.00           C
ATOM   4652  C   SER C 520     102.244  82.873  97.037  1.00  0.00           C
ATOM   4653  O   SER C 520     102.111  82.056  96.129  1.00  0.00           O
ATOM   4654  CB  SER C 520     103.600  82.038  99.063  1.00 20.00           C
ATOM   4655  OG  SER C 520     102.409  82.101  99.820  1.00 20.00           O
ATOM   4656  H   SER C 520     103.969  84.464  99.189  1.00  0.00           H
ATOM   4657  HA  SER C 520     104.360  82.564  97.132  1.00  0.00           H
ATOM   4658  HB2 SER C 520     104.437  82.307  99.702  1.00  0.00           H
ATOM   4659  HB3 SER C 520     103.756  80.999  98.788  1.00  0.00           H
ATOM   4660  HG  SER C 520     102.257  83.009 100.097  1.00  0.00           H
ATOM   4661  N   LEU C 521     101.304  83.767  97.391  1.00  0.00           N
ATOM   4662  CA  LEU C 521     100.012  83.943  96.733  1.00  0.00           C
ATOM   4663  C   LEU C 521     100.180  84.494  95.308  1.00  0.00           C
ATOM   4664  O   LEU C 521      99.581  83.939  94.389  1.00  0.00           O
ATOM   4665  CB  LEU C 521      99.102  84.798  97.647  1.00 20.00           C
ATOM   4666  CG  LEU C 521      97.660  85.050  97.144  1.00 20.00           C
ATOM   4667  CD1 LEU C 521      96.695  85.279  98.323  1.00 20.00           C
ATOM   4668  CD2 LEU C 521      97.560  86.204  96.123  1.00 20.00           C
ATOM   4669  H   LEU C 521     101.486  84.388  98.168  1.00  0.00           H
ATOM   4670  HA  LEU C 521      99.556  82.954  96.648  1.00  0.00           H
ATOM   4671  HB2 LEU C 521      99.046  84.264  98.596  1.00  0.00           H
ATOM   4672  HB3 LEU C 521      99.579  85.749  97.882  1.00  0.00           H
ATOM   4673  HG  LEU C 521      97.326  84.135  96.652  1.00  0.00           H
ATOM   4674 HD11 LEU C 521      95.672  85.036  98.036  1.00  0.00           H
ATOM   4675 HD12 LEU C 521      96.940  84.656  99.184  1.00  0.00           H
ATOM   4676 HD13 LEU C 521      96.708  86.317  98.658  1.00  0.00           H
```

```
ATOM   4677 HD21 LEU C 521      96.774  86.915  96.380  1.00  0.00           H
ATOM   4678 HD22 LEU C 521      98.488  86.771  96.048  1.00  0.00           H
ATOM   4679 HD23 LEU C 521      97.331  85.821  95.129  1.00  0.00           H
ATOM   4680  N   PHE C 522     101.049  85.502  95.147  1.00  0.00           N
ATOM   4681  CA  PHE C 522     101.486  86.008  93.847  1.00  0.00           C
ATOM   4682  C   PHE C 522     102.159  84.919  92.984  1.00  0.00           C
ATOM   4683  O   PHE C 522     101.876  84.834  91.787  1.00  0.00           O
ATOM   4684  CB  PHE C 522     102.451  87.200  94.029  1.00 20.00           C
ATOM   4685  CG  PHE C 522     101.914  88.536  94.513  1.00 20.00           C
ATOM   4686  CD1 PHE C 522     100.740  89.095  93.964  1.00 20.00           C
ATOM   4687  CD2 PHE C 522     102.724  89.341  95.345  1.00 20.00           C
ATOM   4688  CE1 PHE C 522     100.373  90.395  94.288  1.00 20.00           C
ATOM   4689  CE2 PHE C 522     102.342  90.638  95.653  1.00 20.00           C
ATOM   4690  CZ  PHE C 522     101.168  91.161  95.131  1.00 20.00           C
ATOM   4691  H   PHE C 522     101.455  85.940  95.972  1.00  0.00           H
ATOM   4692  HA  PHE C 522     100.602  86.335  93.299  1.00  0.00           H
ATOM   4693  HB2 PHE C 522     103.244  86.896  94.712  1.00  0.00           H
ATOM   4694  HB3 PHE C 522     102.931  87.422  93.079  1.00  0.00           H
ATOM   4695  HD1 PHE C 522     100.126  88.534  93.276  1.00  0.00           H
ATOM   4696  HD2 PHE C 522     103.657  88.962  95.737  1.00  0.00           H
ATOM   4697  HE1 PHE C 522      99.472  90.820  93.869  1.00  0.00           H
ATOM   4698  HE2 PHE C 522     102.968  91.246  96.290  1.00  0.00           H
ATOM   4699  HZ  PHE C 522     100.878  92.175  95.368  1.00  0.00           H
ATOM   4700  N   MET C 523     102.997  84.074  93.607  1.00  0.00           N
ATOM   4701  CA  MET C 523     103.709  82.990  92.924  1.00  0.00           C
ATOM   4702  C   MET C 523     102.763  81.911  92.365  1.00  0.00           C
ATOM   4703  O   MET C 523     103.004  81.419  91.265  1.00  0.00           O
ATOM   4704  CB  MET C 523     104.777  82.377  93.860  1.00 20.00           C
ATOM   4705  CG  MET C 523     105.801  81.481  93.140  1.00 20.00           C
ATOM   4706  SD  MET C 523     106.822  82.363  91.926  1.00 20.00           S
ATOM   4707  CE  MET C 523     106.989  81.095  90.647  1.00 20.00           C
ATOM   4708  H   MET C 523     103.106  84.124  94.611  1.00  0.00           H
ATOM   4709  HA  MET C 523     104.219  83.449  92.076  1.00  0.00           H
ATOM   4710  HB2 MET C 523     105.316  83.169  94.376  1.00  0.00           H
ATOM   4711  HB3 MET C 523     104.293  81.798  94.647  1.00  0.00           H
ATOM   4712  HG2 MET C 523     105.299  80.646  92.652  1.00  0.00           H
ATOM   4713  HG3 MET C 523     106.477  81.038  93.871  1.00  0.00           H
ATOM   4714  HE1 MET C 523     107.627  81.456  89.840  1.00  0.00           H
ATOM   4715  HE2 MET C 523     107.437  80.196  91.066  1.00  0.00           H
ATOM   4716  HE3 MET C 523     106.014  80.842  90.232  1.00  0.00           H
ATOM   4717  N   LEU C 524     101.700  81.578  93.118  1.00  0.00           N
ATOM   4718  CA  LEU C 524     100.701  80.582  92.730  1.00  0.00           C
ATOM   4719  C   LEU C 524      99.690  81.114  91.701  1.00  0.00           C
ATOM   4720  O   LEU C 524      99.237  80.328  90.871  1.00  0.00           O
ATOM   4721  CB  LEU C 524      99.992  80.042  93.988  1.00 20.00           C
ATOM   4722  CG  LEU C 524     100.901  79.188  94.903  1.00 20.00           C
ATOM   4723  CD1 LEU C 524     100.247  78.991  96.282  1.00 20.00           C
ATOM   4724  CD2 LEU C 524     101.314  77.850  94.250  1.00 20.00           C
ATOM   4725  H   LEU C 524     101.574  82.013  94.022  1.00  0.00           H
ATOM   4726  HA  LEU C 524     101.222  79.753  92.248  1.00  0.00           H
ATOM   4727  HB2 LEU C 524      99.599  80.889  94.555  1.00  0.00           H
ATOM   4728  HB3 LEU C 524      99.122  79.446  93.705  1.00  0.00           H
ATOM   4729  HG  LEU C 524     101.825  79.738  95.078  1.00  0.00           H
ATOM   4730 HD11 LEU C 524     100.438  78.006  96.708  1.00  0.00           H
ATOM   4731 HD12 LEU C 524     100.641  79.724  96.985  1.00  0.00           H
ATOM   4732 HD13 LEU C 524      99.166  79.129  96.248  1.00  0.00           H
ATOM   4733 HD21 LEU C 524     101.046  76.985  94.855  1.00  0.00           H
ATOM   4734 HD22 LEU C 524     100.847  77.705  93.276  1.00  0.00           H
ATOM   4735 HD23 LEU C 524     102.394  77.813  94.100  1.00  0.00           H
ATOM   4736  N   VAL C 525      99.389  82.426  91.720  1.00  0.00           N
ATOM   4737  CA  VAL C 525      98.596  83.088  90.676  1.00  0.00           C
ATOM   4738  C   VAL C 525      99.377  83.191  89.346  1.00  0.00           C
ATOM   4739  O   VAL C 525      98.762  83.071  88.288  1.00  0.00           O
ATOM   4740  CB  VAL C 525      98.097  84.494  91.125  1.00 20.00           C
ATOM   4741  CG1 VAL C 525      97.473  85.359  90.006  1.00 20.00           C
ATOM   4742  CG2 VAL C 525      97.063  84.364  92.259  1.00 20.00           C
ATOM   4743  H   VAL C 525      99.765  83.019  92.448  1.00  0.00           H
ATOM   4744  HA  VAL C 525      97.716  82.471  90.486  1.00  0.00           H
ATOM   4745  HB  VAL C 525      98.953  85.042  91.522  1.00  0.00           H
ATOM   4746 HG11 VAL C 525      97.104  86.305  90.403  1.00  0.00           H
ATOM   4747 HG12 VAL C 525      98.183  85.606  89.219  1.00  0.00           H
```

```
ATOM   4748 HG13 VAL C 525      96.631  84.847  89.538  1.00  0.00           H
ATOM   4749 HG21 VAL C 525      96.797  85.341  92.662  1.00  0.00           H
ATOM   4750 HG22 VAL C 525      96.147  83.892  91.902  1.00  0.00           H
ATOM   4751 HG23 VAL C 525      97.431  83.760  93.086  1.00  0.00           H
ATOM   4752  N   SER C 526     100.713  83.338  89.420  1.00  0.00           N
ATOM   4753  CA  SER C 526     101.612  83.266  88.266  1.00  0.00           C
ATOM   4754  C   SER C 526     101.632  81.866  87.616  1.00  0.00           C
ATOM   4755  O   SER C 526     101.654  81.789  86.392  1.00  0.00           O
ATOM   4756  CB  SER C 526     103.019  83.742  88.677  1.00 20.00           C
ATOM   4757  OG  SER C 526     103.890  83.790  87.568  1.00 20.00           O
ATOM   4758  H   SER C 526     101.154  83.438  90.324  1.00  0.00           H
ATOM   4759  HA  SER C 526     101.231  83.966  87.519  1.00  0.00           H
ATOM   4760  HB2 SER C 526     102.970  84.736  89.118  1.00  0.00           H
ATOM   4761  HB3 SER C 526     103.458  83.088  89.426  1.00  0.00           H
ATOM   4762  HG  SER C 526     103.630  84.531  87.010  1.00  0.00           H
ATOM   4763  N   VAL C 527     101.562  80.794  88.427  1.00 30.00           N
ATOM   4764  CA  VAL C 527     101.429  79.412  87.952  1.00 30.00           C
ATOM   4765  C   VAL C 527     100.081  79.160  87.245  1.00 30.00           C
ATOM   4766  O   VAL C 527     100.078  78.562  86.170  1.00 30.00           O
ATOM   4767  CB  VAL C 527     101.622  78.388  89.111  1.00 20.00           C
ATOM   4768  CG1 VAL C 527     101.165  76.941  88.823  1.00 20.00           C
ATOM   4769  CG2 VAL C 527     103.089  78.363  89.559  1.00 20.00           C
ATOM   4770  H   VAL C 527     101.559  80.926  89.429  1.00  0.00           H
ATOM   4771  HA  VAL C 527     102.218  79.242  87.216  1.00  0.00           H
ATOM   4772  HB  VAL C 527     101.043  78.732  89.966  1.00  0.00           H
ATOM   4773 HG11 VAL C 527     101.431  76.276  89.646  1.00  0.00           H
ATOM   4774 HG12 VAL C 527     100.085  76.863  88.700  1.00  0.00           H
ATOM   4775 HG13 VAL C 527     101.639  76.552  87.922  1.00  0.00           H
ATOM   4776 HG21 VAL C 527     103.219  77.735  90.440  1.00  0.00           H
ATOM   4777 HG22 VAL C 527     103.731  77.970  88.769  1.00  0.00           H
ATOM   4778 HG23 VAL C 527     103.455  79.357  89.811  1.00  0.00           H
ATOM   4779  N   VAL C 528      98.978  79.651  87.838  1.00  0.00           N
ATOM   4780  CA  VAL C 528      97.622  79.541  87.292  1.00  0.00           C
ATOM   4781  C   VAL C 528      97.469  80.224  85.919  1.00  0.00           C
ATOM   4782  O   VAL C 528      96.938  79.594  85.007  1.00  0.00           O
ATOM   4783  CB  VAL C 528      96.556  80.086  88.294  1.00 20.00           C
ATOM   4784  CG1 VAL C 528      95.162  80.394  87.703  1.00 20.00           C
ATOM   4785  CG2 VAL C 528      96.390  79.119  89.479  1.00 20.00           C
ATOM   4786  H   VAL C 528      99.063  80.126  88.726  1.00  0.00           H
ATOM   4787  HA  VAL C 528      97.429  78.477  87.142  1.00  0.00           H
ATOM   4788  HB  VAL C 528      96.936  81.023  88.702  1.00  0.00           H
ATOM   4789 HG11 VAL C 528      94.458  80.673  88.488  1.00  0.00           H
ATOM   4790 HG12 VAL C 528      95.183  81.226  86.999  1.00  0.00           H
ATOM   4791 HG13 VAL C 528      94.750  79.526  87.186  1.00  0.00           H
ATOM   4792 HG21 VAL C 528      95.751  79.548  90.252  1.00  0.00           H
ATOM   4793 HG22 VAL C 528      95.939  78.179  89.159  1.00  0.00           H
ATOM   4794 HG23 VAL C 528      97.344  78.876  89.944  1.00  0.00           H
ATOM   4795  N   LEU C 529      97.972  81.465  85.787  1.00  0.00           N
ATOM   4796  CA  LEU C 529      97.914  82.243  84.546  1.00  0.00           C
ATOM   4797  C   LEU C 529      98.857  81.707  83.451  1.00  0.00           C
ATOM   4798  O   LEU C 529      98.524  81.844  82.274  1.00  0.00           O
ATOM   4799  CB  LEU C 529      98.147  83.745  84.835  1.00 20.00           C
ATOM   4800  CG  LEU C 529      96.853  84.560  85.081  1.00 20.00           C
ATOM   4801  CD1 LEU C 529      96.065  84.114  86.333  1.00 20.00           C
ATOM   4802  CD2 LEU C 529      97.149  86.073  85.092  1.00 20.00           C
ATOM   4803  H   LEU C 529      98.399  81.919  86.583  1.00  0.00           H
ATOM   4804  HA  LEU C 529      96.906  82.145  84.151  1.00  0.00           H
ATOM   4805  HB2 LEU C 529      98.853  83.876  85.656  1.00  0.00           H
ATOM   4806  HB3 LEU C 529      98.627  84.197  83.965  1.00  0.00           H
ATOM   4807  HG  LEU C 529      96.200  84.385  84.224  1.00  0.00           H
ATOM   4808 HD11 LEU C 529      95.099  83.696  86.050  1.00  0.00           H
ATOM   4809 HD12 LEU C 529      96.585  83.353  86.908  1.00  0.00           H
ATOM   4810 HD13 LEU C 529      95.867  84.932  87.025  1.00  0.00           H
ATOM   4811 HD21 LEU C 529      96.616  86.612  85.875  1.00  0.00           H
ATOM   4812 HD22 LEU C 529      98.209  86.276  85.239  1.00  0.00           H
ATOM   4813 HD23 LEU C 529      96.855  86.526  84.145  1.00  0.00           H
ATOM   4814  N   TYR C 530      99.984  81.086  83.845  1.00  0.00           N
ATOM   4815  CA  TYR C 530     100.946  80.448  82.943  1.00  0.00           C
ATOM   4816  C   TYR C 530     100.360  79.222  82.219  1.00  0.00           C
ATOM   4817  O   TYR C 530     100.569  79.088  81.015  1.00  0.00           O
ATOM   4818  CB  TYR C 530     102.228  80.104  83.732  1.00 20.00           C
```

```
ATOM   4819  CG  TYR C 530     103.342  79.437  82.946  1.00 20.00           C
ATOM   4820  CD1 TYR C 530     104.181  80.219  82.130  1.00 20.00           C
ATOM   4821  CD2 TYR C 530     103.559  78.046  83.041  1.00 20.00           C
ATOM   4822  CE1 TYR C 530     105.238  79.620  81.422  1.00 20.00           C
ATOM   4823  CE2 TYR C 530     104.608  77.442  82.320  1.00 20.00           C
ATOM   4824  CZ  TYR C 530     105.448  78.229  81.506  1.00 20.00           C
ATOM   4825  OH  TYR C 530     106.459  77.647  80.798  1.00 20.00           O
ATOM   4826  H   TYR C 530     100.192  81.020  84.832  1.00  0.00           H
ATOM   4827  HA  TYR C 530     101.212  81.185  82.182  1.00  0.00           H
ATOM   4828  HB2 TYR C 530     102.643  81.019  84.154  1.00  0.00           H
ATOM   4829  HB3 TYR C 530     101.984  79.470  84.584  1.00  0.00           H
ATOM   4830  HD1 TYR C 530     104.018  81.282  82.049  1.00  0.00           H
ATOM   4831  HD2 TYR C 530     102.919  77.439  83.664  1.00  0.00           H
ATOM   4832  HE1 TYR C 530     105.882  80.241  80.821  1.00  0.00           H
ATOM   4833  HE2 TYR C 530     104.760  76.376  82.394  1.00  0.00           H
ATOM   4834  HH  TYR C 530     106.511  76.697  80.932  1.00  0.00           H
ATOM   4835  N   PHE C 531      99.620  78.375  82.956  1.00  0.00           N
ATOM   4836  CA  PHE C 531      98.958  77.188  82.409  1.00  0.00           C
ATOM   4837  C   PHE C 531      97.573  77.466  81.794  1.00  0.00           C
ATOM   4838  O   PHE C 531      97.161  76.691  80.932  1.00  0.00           O
ATOM   4839  CB  PHE C 531      98.923  76.059  83.460  1.00 20.00           C
ATOM   4840  CG  PHE C 531     100.286  75.445  83.746  1.00 20.00           C
ATOM   4841  CD1 PHE C 531     100.986  74.765  82.726  1.00 20.00           C
ATOM   4842  CD2 PHE C 531     100.896  75.594  85.010  1.00 20.00           C
ATOM   4843  CE1 PHE C 531     102.257  74.262  82.969  1.00 20.00           C
ATOM   4844  CE2 PHE C 531     102.166  75.079  85.234  1.00 20.00           C
ATOM   4845  CZ  PHE C 531     102.844  74.419  84.217  1.00 20.00           C
ATOM   4846  H   PHE C 531      99.499  78.544  83.946  1.00  0.00           H
ATOM   4847  HA  PHE C 531      99.558  76.823  81.574  1.00  0.00           H
ATOM   4848  HB2 PHE C 531      98.489  76.429  84.390  1.00  0.00           H
ATOM   4849  HB3 PHE C 531      98.268  75.255  83.121  1.00  0.00           H
ATOM   4850  HD1 PHE C 531     100.539  74.641  81.752  1.00  0.00           H
ATOM   4851  HD2 PHE C 531     100.378  76.102  85.809  1.00  0.00           H
ATOM   4852  HE1 PHE C 531     102.791  73.748  82.183  1.00  0.00           H
ATOM   4853  HE2 PHE C 531     102.632  75.195  86.201  1.00  0.00           H
ATOM   4854  HZ  PHE C 531     103.834  74.027  84.400  1.00  0.00           H
ATOM   4855  N   SER C 532      96.911  78.577  82.177  1.00 30.00           N
ATOM   4856  CA  SER C 532      95.698  79.084  81.515  1.00 30.00           C
ATOM   4857  C   SER C 532      96.000  79.931  80.255  1.00 30.00           C
ATOM   4858  O   SER C 532      95.075  80.524  79.700  1.00 30.00           O
ATOM   4859  CB  SER C 532      94.774  79.792  82.533  1.00 20.00           C
ATOM   4860  OG  SER C 532      95.104  81.150  82.743  1.00 20.00           O
ATOM   4861  H   SER C 532      97.289  79.160  82.912  1.00  0.00           H
ATOM   4862  HA  SER C 532      95.137  78.218  81.161  1.00  0.00           H
ATOM   4863  HB2 SER C 532      94.762  79.265  83.487  1.00  0.00           H
ATOM   4864  HB3 SER C 532      93.749  79.769  82.163  1.00  0.00           H
ATOM   4865  HG  SER C 532      94.907  81.635  81.937  1.00  0.00           H
ATOM   4866  N   GLN C 533      97.271  79.932  79.814  1.00 30.00           N
ATOM   4867  CA  GLN C 533      97.770  80.408  78.522  1.00 30.00           C
ATOM   4868  C   GLN C 533      97.802  81.939  78.340  1.00 30.00           C
ATOM   4869  O   GLN C 533      97.931  82.402  77.206  1.00 30.00           O
ATOM   4870  CB  GLN C 533      97.051  79.685  77.352  1.00 20.00           C
ATOM   4871  CG  GLN C 533      97.155  78.147  77.406  1.00 20.00           C
ATOM   4872  CD  GLN C 533      96.587  77.453  76.166  1.00 20.00           C
ATOM   4873  NE2 GLN C 533      96.827  76.145  76.055  1.00 20.00           N
ATOM   4874  OE1 GLN C 533      95.932  78.068  75.327  1.00 20.00           O
ATOM   4875  H   GLN C 533      97.959  79.443  80.370  1.00  0.00           H
ATOM   4876  HA  GLN C 533      98.814  80.108  78.518  1.00  0.00           H
ATOM   4877  HB2 GLN C 533      96.001  79.976  77.317  1.00  0.00           H
ATOM   4878  HB3 GLN C 533      97.476  80.019  76.408  1.00  0.00           H
ATOM   4879  HG2 GLN C 533      98.198  77.859  77.510  1.00  0.00           H
ATOM   4880  HG3 GLN C 533      96.634  77.759  78.282  1.00  0.00           H
ATOM   4881 HE21 GLN C 533      96.473  75.635  75.259  1.00  0.00           H
ATOM   4882 HE22 GLN C 533      97.362  75.660  76.760  1.00  0.00           H
ATOM   4883  N   ARG C 534      97.708  82.701  79.441  1.00 30.00           N
ATOM   4884  CA  ARG C 534      97.674  84.162  79.423  1.00 30.00           C
ATOM   4885  C   ARG C 534      99.057  84.734  79.776  1.00 30.00           C
ATOM   4886  O   ARG C 534      99.642  84.330  80.781  1.00 30.00           O
ATOM   4887  CB  ARG C 534      96.572  84.635  80.394  1.00 20.00           C
ATOM   4888  CG  ARG C 534      96.225  86.130  80.259  1.00 20.00           C
ATOM   4889  CD  ARG C 534      94.981  86.551  81.059  1.00 20.00           C
```

ATOM	4890	NE	ARG	C	534	93.742	85.998	80.489	1.00	20.00	N
ATOM	4891	CZ	ARG	C	534	93.045	86.519	79.463	1.00	20.00	C
ATOM	4892	NH1	ARG	C	534	93.413	87.670	78.882	1.00	20.00	N
ATOM	4893	NH2	ARG	C	534	91.961	85.874	79.012	1.00	20.00	N1+
ATOM	4894	H	ARG	C	534	97.660	82.262	80.351	1.00	0.00	H
ATOM	4895	HA	ARG	C	534	97.403	84.513	78.424	1.00	0.00	H
ATOM	4896	HB2	ARG	C	534	95.670	84.054	80.197	1.00	0.00	H
ATOM	4897	HB3	ARG	C	534	96.855	84.409	81.423	1.00	0.00	H
ATOM	4898	HG2	ARG	C	534	97.068	86.785	80.470	1.00	0.00	H
ATOM	4899	HG3	ARG	C	534	95.993	86.285	79.204	1.00	0.00	H
ATOM	4900	HD2	ARG	C	534	95.047	86.059	82.030	1.00	0.00	H
ATOM	4901	HD3	ARG	C	534	94.941	87.619	81.273	1.00	0.00	H
ATOM	4902	HE	ARG	C	534	93.439	85.116	80.876	1.00	0.00	H
ATOM	4903	HH11	ARG	C	534	92.884	88.055	78.114	1.00	0.00	H
ATOM	4904	HH12	ARG	C	534	94.216	88.173	79.233	1.00	0.00	H
ATOM	4905	HH21	ARG	C	534	91.425	86.249	78.244	1.00	0.00	H
ATOM	4906	HH22	ARG	C	534	91.668	85.007	79.439	1.00	0.00	H
ATOM	4907	N	LYS	C	535	99.537	85.688	78.956	1.00	30.00	N
ATOM	4908	CA	LYS	C	535	100.822	86.387	79.120	1.00	30.00	C
ATOM	4909	C	LYS	C	535	100.913	87.260	80.394	1.00	30.00	C
ATOM	4910	O	LYS	C	535	102.023	87.537	80.845	1.00	30.00	O
ATOM	4911	CB	LYS	C	535	101.134	87.165	77.818	1.00	20.00	C
ATOM	4912	CG	LYS	C	535	102.467	87.946	77.811	1.00	20.00	C
ATOM	4913	CD	LYS	C	535	102.837	88.537	76.437	1.00	20.00	C
ATOM	4914	CE	LYS	C	535	103.449	87.536	75.442	1.00	20.00	C
ATOM	4915	NZ	LYS	C	535	104.836	87.190	75.801	1.00	20.00	N1+
ATOM	4916	H	LYS	C	535	98.993	85.960	78.150	1.00	0.00	H
ATOM	4917	HA	LYS	C	535	101.587	85.616	79.216	1.00	0.00	H
ATOM	4918	HB2	LYS	C	535	101.143	86.451	76.994	1.00	0.00	H
ATOM	4919	HB3	LYS	C	535	100.320	87.860	77.604	1.00	0.00	H
ATOM	4920	HG2	LYS	C	535	102.397	88.777	78.513	1.00	0.00	H
ATOM	4921	HG3	LYS	C	535	103.275	87.312	78.177	1.00	0.00	H
ATOM	4922	HD2	LYS	C	535	101.945	88.976	75.988	1.00	0.00	H
ATOM	4923	HD3	LYS	C	535	103.527	89.370	76.581	1.00	0.00	H
ATOM	4924	HE2	LYS	C	535	102.848	86.629	75.372	1.00	0.00	H
ATOM	4925	HE3	LYS	C	535	103.469	87.981	74.446	1.00	0.00	H
ATOM	4926	HZ1	LYS	C	535	105.225	86.551	75.119	1.00	0.00	H
ATOM	4927	HZ2	LYS	C	535	105.392	88.034	75.816	1.00	0.00	H
ATOM	4928	HZ3	LYS	C	535	104.855	86.759	76.713	1.00	0.00	H
ATOM	4929	N	GLU	C	536	99.756	87.615	80.983	1.00	30.00	N
ATOM	4930	CA	GLU	C	536	99.600	88.364	82.236	1.00	30.00	C
ATOM	4931	C	GLU	C	536	100.116	87.636	83.499	1.00	30.00	C
ATOM	4932	O	GLU	C	536	100.164	88.253	84.562	1.00	30.00	O
ATOM	4933	CB	GLU	C	536	98.128	88.825	82.328	1.00	20.00	C
ATOM	4934	CG	GLU	C	536	97.795	89.811	83.468	1.00	0.00	C
ATOM	4935	CD	GLU	C	536	96.391	90.407	83.339	1.00	0.00	C
ATOM	4936	OE1	GLU	C	536	95.475	89.655	82.938	1.00	0.00	O
ATOM	4937	OE2	GLU	C	536	96.260	91.612	83.643	1.00	0.00	O1-
ATOM	4938	H	GLU	C	536	98.893	87.324	80.549	1.00	0.00	H
ATOM	4939	HA	GLU	C	536	100.221	89.255	82.149	1.00	0.00	H
ATOM	4940	HB2	GLU	C	536	97.840	89.279	81.378	1.00	0.00	H
ATOM	4941	HB3	GLU	C	536	97.493	87.946	82.432	1.00	0.00	H
ATOM	4942	HG2	GLU	C	536	97.849	89.316	84.437	1.00	0.00	H
ATOM	4943	HG3	GLU	C	536	98.525	90.621	83.483	1.00	0.00	H
ATOM	4944	N	TYR	C	537	100.570	86.374	83.362	1.00	30.00	N
ATOM	4945	CA	TYR	C	537	101.319	85.624	84.378	1.00	30.00	C
ATOM	4946	C	TYR	C	537	102.593	86.357	84.856	1.00	30.00	C
ATOM	4947	O	TYR	C	537	102.979	86.190	86.013	1.00	30.00	O
ATOM	4948	CB	TYR	C	537	101.644	84.211	83.826	1.00	20.00	C
ATOM	4949	CG	TYR	C	537	102.982	84.028	83.120	1.00	20.00	C
ATOM	4950	CD1	TYR	C	537	103.111	84.353	81.755	1.00	20.00	C
ATOM	4951	CD2	TYR	C	537	104.113	83.582	83.839	1.00	20.00	C
ATOM	4952	CE1	TYR	C	537	104.364	84.262	81.118	1.00	20.00	C
ATOM	4953	CE2	TYR	C	537	105.367	83.495	83.205	1.00	20.00	C
ATOM	4954	CZ	TYR	C	537	105.495	83.841	81.845	1.00	20.00	C
ATOM	4955	OH	TYR	C	537	106.712	83.765	81.234	1.00	20.00	O
ATOM	4956	H	TYR	C	537	100.492	85.927	82.460	1.00	0.00	H
ATOM	4957	HA	TYR	C	537	100.665	85.513	85.245	1.00	0.00	H
ATOM	4958	HB2	TYR	C	537	100.844	83.849	83.182	1.00	0.00	H
ATOM	4959	HB3	TYR	C	537	101.651	83.529	84.671	1.00	0.00	H
ATOM	4960	HD1	TYR	C	537	102.251	84.690	81.201	1.00	0.00	H

```
ATOM   4961  HD2 TYR C 537     104.025  83.325  84.884  1.00  0.00           H
ATOM   4962  HE1 TYR C 537     104.457  84.523  80.074  1.00  0.00           H
ATOM   4963  HE2 TYR C 537     106.227  83.165  83.768  1.00  0.00           H
ATOM   4964  HH  TYR C 537     107.406  83.474  81.829  1.00  0.00           H
ATOM   4965  N   VAL C 538     103.208  87.144  83.951  1.00 30.00           N
ATOM   4966  CA  VAL C 538     104.413  87.933  84.189  1.00 30.00           C
ATOM   4967  C   VAL C 538     104.204  89.058  85.223  1.00 30.00           C
ATOM   4968  O   VAL C 538     105.133  89.334  85.974  1.00 30.00           O
ATOM   4969  CB  VAL C 538     104.973  88.533  82.863  1.00 20.00           C
ATOM   4970  CG1 VAL C 538     104.140  89.692  82.273  1.00 20.00           C
ATOM   4971  CG2 VAL C 538     106.443  88.970  82.997  1.00 20.00           C
ATOM   4972  H   VAL C 538     102.819  87.212  83.020  1.00  0.00           H
ATOM   4973  HA  VAL C 538     105.160  87.246  84.588  1.00  0.00           H
ATOM   4974  HB  VAL C 538     104.951  87.728  82.127  1.00  0.00           H
ATOM   4975 HG11 VAL C 538     104.453  89.915  81.252  1.00  0.00           H
ATOM   4976 HG12 VAL C 538     103.078  89.452  82.243  1.00  0.00           H
ATOM   4977 HG13 VAL C 538     104.251  90.611  82.850  1.00  0.00           H
ATOM   4978 HG21 VAL C 538     106.834  89.308  82.037  1.00  0.00           H
ATOM   4979 HG22 VAL C 538     106.566  89.795  83.700  1.00  0.00           H
ATOM   4980 HG23 VAL C 538     107.073  88.147  83.336  1.00  0.00           H
ATOM   4981  N   ALA C 539     102.990  89.636  85.293  1.00  0.00           N
ATOM   4982  CA  ALA C 539     102.607  90.643  86.286  1.00  0.00           C
ATOM   4983  C   ALA C 539     102.659  90.108  87.726  1.00  0.00           C
ATOM   4984  O   ALA C 539     103.165  90.795  88.612  1.00  0.00           O
ATOM   4985  CB  ALA C 539     101.197  91.161  85.962  1.00  0.00           C
ATOM   4986  H   ALA C 539     102.261  89.337  84.661  1.00  0.00           H
ATOM   4987  HA  ALA C 539     103.309  91.474  86.204  1.00  0.00           H
ATOM   4988  HB1 ALA C 539     100.899  91.944  86.659  1.00  0.00           H
ATOM   4989  HB2 ALA C 539     101.149  91.572  84.953  1.00  0.00           H
ATOM   4990  HB3 ALA C 539     100.449  90.371  86.033  1.00  0.00           H
ATOM   4991  N   SER C 540     102.170  88.873  87.909  1.00  0.00           N
ATOM   4992  CA  SER C 540     102.158  88.159  89.181  1.00  0.00           C
ATOM   4993  C   SER C 540     103.538  87.589  89.564  1.00  0.00           C
ATOM   4994  O   SER C 540     103.837  87.522  90.754  1.00  0.00           O
ATOM   4995  CB  SER C 540     101.076  87.069  89.108  1.00 20.00           C
ATOM   4996  OG  SER C 540      99.796  87.668  89.117  1.00 20.00           O
ATOM   4997  H   SER C 540     101.768  88.381  87.123  1.00  0.00           H
ATOM   4998  HA  SER C 540     101.882  88.861  89.972  1.00  0.00           H
ATOM   4999  HB2 SER C 540     101.186  86.451  88.216  1.00  0.00           H
ATOM   5000  HB3 SER C 540     101.127  86.405  89.967  1.00  0.00           H
ATOM   5001  HG  SER C 540      99.637  88.044  89.986  1.00  0.00           H
ATOM   5002  N   MET C 541     104.375  87.241  88.566  1.00  0.00           N
ATOM   5003  CA  MET C 541     105.782  86.860  88.748  1.00  0.00           C
ATOM   5004  C   MET C 541     106.632  88.035  89.262  1.00  0.00           C
ATOM   5005  O   MET C 541     107.419  87.855  90.188  1.00  0.00           O
ATOM   5006  CB  MET C 541     106.342  86.292  87.423  1.00 20.00           C
ATOM   5007  CG  MET C 541     107.832  85.902  87.449  1.00 20.00           C
ATOM   5008  SD  MET C 541     108.396  84.994  85.982  1.00 20.00           S
ATOM   5009  CE  MET C 541     107.724  83.350  86.347  1.00 20.00           C
ATOM   5010  H   MET C 541     104.055  87.310  87.609  1.00  0.00           H
ATOM   5011  HA  MET C 541     105.819  86.066  89.498  1.00  0.00           H
ATOM   5012  HB2 MET C 541     105.752  85.436  87.111  1.00  0.00           H
ATOM   5013  HB3 MET C 541     106.211  87.032  86.635  1.00  0.00           H
ATOM   5014  HG2 MET C 541     108.451  86.796  87.528  1.00  0.00           H
ATOM   5015  HG3 MET C 541     108.045  85.300  88.332  1.00  0.00           H
ATOM   5016  HE1 MET C 541     108.024  82.643  85.573  1.00  0.00           H
ATOM   5017  HE2 MET C 541     108.097  82.988  87.305  1.00  0.00           H
ATOM   5018  HE3 MET C 541     106.635  83.374  86.385  1.00  0.00           H
ATOM   5019  N   VAL C 542     106.425  89.211  88.649  1.00  0.00           N
ATOM   5020  CA  VAL C 542     107.057  90.490  88.964  1.00  0.00           C
ATOM   5021  C   VAL C 542     106.761  90.973  90.397  1.00  0.00           C
ATOM   5022  O   VAL C 542     107.693  91.391  91.084  1.00  0.00           O
ATOM   5023  CB  VAL C 542     106.613  91.558  87.919  1.00 20.00           C
ATOM   5024  CG1 VAL C 542     106.744  93.034  88.334  1.00 20.00           C
ATOM   5025  CG2 VAL C 542     107.348  91.344  86.586  1.00 20.00           C
ATOM   5026  H   VAL C 542     105.770  89.242  87.878  1.00  0.00           H
ATOM   5027  HA  VAL C 542     108.137  90.349  88.883  1.00  0.00           H
ATOM   5028  HB  VAL C 542     105.556  91.395  87.714  1.00  0.00           H
ATOM   5029 HG11 VAL C 542     106.512  93.684  87.492  1.00  0.00           H
ATOM   5030 HG12 VAL C 542     106.058  93.305  89.138  1.00  0.00           H
ATOM   5031 HG13 VAL C 542     107.759  93.255  88.657  1.00  0.00           H
```

ATOM	5032	HG21	VAL	C	542	106.845	91.866	85.774	1.00	0.00	H
ATOM	5033	HG22	VAL	C	542	108.365	91.717	86.640	1.00	0.00	H
ATOM	5034	HG23	VAL	C	542	107.417	90.293	86.308	1.00	0.00	H
ATOM	5035	N	PHE	C	543	105.499	90.878	90.836	1.00	0.00	N
ATOM	5036	CA	PHE	C	543	105.123	91.264	92.195	1.00	0.00	C
ATOM	5037	C	PHE	C	543	105.554	90.244	93.262	1.00	0.00	C
ATOM	5038	O	PHE	C	543	105.885	90.661	94.372	1.00	0.00	O
ATOM	5039	CB	PHE	C	543	103.622	91.595	92.276	1.00	20.00	C
ATOM	5040	CG	PHE	C	543	103.159	92.812	91.484	1.00	20.00	C
ATOM	5041	CD1	PHE	C	543	103.916	94.008	91.447	1.00	20.00	C
ATOM	5042	CD2	PHE	C	543	101.866	92.817	90.919	1.00	20.00	C
ATOM	5043	CE1	PHE	C	543	103.417	95.132	90.806	1.00	20.00	C
ATOM	5044	CE2	PHE	C	543	101.379	93.956	90.292	1.00	20.00	C
ATOM	5045	CZ	PHE	C	543	102.151	95.108	90.237	1.00	20.00	C
ATOM	5046	H	PHE	C	543	104.756	90.643	90.187	1.00	0.00	H
ATOM	5047	HA	PHE	C	543	105.676	92.166	92.455	1.00	0.00	H
ATOM	5048	HB2	PHE	C	543	103.051	90.725	91.949	1.00	0.00	H
ATOM	5049	HB3	PHE	C	543	103.341	91.763	93.315	1.00	0.00	H
ATOM	5050	HD1	PHE	C	543	104.890	94.073	91.907	1.00	0.00	H
ATOM	5051	HD2	PHE	C	543	101.247	91.932	90.968	1.00	0.00	H
ATOM	5052	HE1	PHE	C	543	104.011	96.032	90.754	1.00	0.00	H
ATOM	5053	HE2	PHE	C	543	100.393	93.946	89.851	1.00	0.00	H
ATOM	5054	HZ	PHE	C	543	101.766	95.992	89.751	1.00	0.00	H
ATOM	5055	N	SER	C	544	105.621	88.947	92.918	1.00	0.00	N
ATOM	5056	CA	SER	C	544	106.206	87.930	93.797	1.00	0.00	C
ATOM	5057	C	SER	C	544	107.716	88.159	94.013	1.00	0.00	C
ATOM	5058	O	SER	C	544	108.197	87.989	95.131	1.00	0.00	O
ATOM	5059	CB	SER	C	544	105.917	86.528	93.222	1.00	20.00	C
ATOM	5060	OG	SER	C	544	106.454	85.503	94.032	1.00	20.00	O
ATOM	5061	H	SER	C	544	105.409	88.655	91.973	1.00	0.00	H
ATOM	5062	HA	SER	C	544	105.716	88.001	94.771	1.00	0.00	H
ATOM	5063	HB2	SER	C	544	104.847	86.357	93.145	1.00	0.00	H
ATOM	5064	HB3	SER	C	544	106.311	86.431	92.212	1.00	0.00	H
ATOM	5065	HG	SER	C	544	105.879	85.385	94.795	1.00	0.00	H
ATOM	5066	N	LEU	C	545	108.427	88.514	92.932	1.00	0.00	N
ATOM	5067	CA	LEU	C	545	109.874	88.704	92.915	1.00	0.00	C
ATOM	5068	C	LEU	C	545	110.286	89.970	93.677	1.00	0.00	C
ATOM	5069	O	LEU	C	545	111.178	89.897	94.524	1.00	0.00	O
ATOM	5070	CB	LEU	C	545	110.355	88.644	91.447	1.00	20.00	C
ATOM	5071	CG	LEU	C	545	111.884	88.559	91.234	1.00	20.00	C
ATOM	5072	CD1	LEU	C	545	112.234	87.684	90.012	1.00	20.00	C
ATOM	5073	CD2	LEU	C	545	112.527	89.952	91.120	1.00	20.00	C
ATOM	5074	H	LEU	C	545	107.970	88.553	92.028	1.00	0.00	H
ATOM	5075	HA	LEU	C	545	110.327	87.866	93.445	1.00	0.00	H
ATOM	5076	HB2	LEU	C	545	109.921	87.734	91.032	1.00	0.00	H
ATOM	5077	HB3	LEU	C	545	109.930	89.459	90.858	1.00	0.00	H
ATOM	5078	HG	LEU	C	545	112.317	88.067	92.107	1.00	0.00	H
ATOM	5079	HD11	LEU	C	545	113.071	88.081	89.439	1.00	0.00	H
ATOM	5080	HD12	LEU	C	545	112.513	86.677	90.328	1.00	0.00	H
ATOM	5081	HD13	LEU	C	545	111.396	87.581	89.322	1.00	0.00	H
ATOM	5082	HD21	LEU	C	545	113.522	89.954	91.561	1.00	0.00	H
ATOM	5083	HD22	LEU	C	545	112.622	90.260	90.080	1.00	0.00	H
ATOM	5084	HD23	LEU	C	545	111.951	90.732	91.616	1.00	0.00	H
ATOM	5085	N	ALA	C	546	109.581	91.084	93.418	1.00	0.00	N
ATOM	5086	CA	ALA	C	546	109.756	92.351	94.131	1.00	0.00	C
ATOM	5087	C	ALA	C	546	109.529	92.198	95.646	1.00	0.00	C
ATOM	5088	O	ALA	C	546	110.392	92.602	96.428	1.00	0.00	O
ATOM	5089	CB	ALA	C	546	108.822	93.418	93.538	1.00	20.00	C
ATOM	5090	H	ALA	C	546	108.878	91.066	92.685	1.00	0.00	H
ATOM	5091	HA	ALA	C	546	110.788	92.675	93.980	1.00	0.00	H
ATOM	5092	HB1	ALA	C	546	108.954	94.375	94.045	1.00	0.00	H
ATOM	5093	HB2	ALA	C	546	109.028	93.582	92.482	1.00	0.00	H
ATOM	5094	HB3	ALA	C	546	107.774	93.133	93.628	1.00	0.00	H
ATOM	5095	N	MET	C	547	108.412	91.548	96.019	1.00	0.00	N
ATOM	5096	CA	MET	C	547	108.071	91.231	97.408	1.00	0.00	C
ATOM	5097	C	MET	C	547	109.111	90.293	98.052	1.00	0.00	C
ATOM	5098	O	MET	C	547	109.540	90.529	99.178	1.00	0.00	O
ATOM	5099	CB	MET	C	547	106.639	90.649	97.486	1.00	20.00	C
ATOM	5100	CG	MET	C	547	106.113	90.405	98.915	1.00	20.00	C
ATOM	5101	SD	MET	C	547	105.912	91.893	99.931	1.00	20.00	S
ATOM	5102	CE	MET	C	547	104.347	92.540	99.291	1.00	20.00	C

```
ATOM   5103  H   MET C 547     107.759  91.233  95.312  1.00  0.00           H
ATOM   5104  HA  MET C 547     108.073  92.172  97.960  1.00  0.00           H
ATOM   5105  HB2 MET C 547     105.951  91.323  96.979  1.00  0.00           H
ATOM   5106  HB3 MET C 547     106.596  89.710  96.931  1.00  0.00           H
ATOM   5107  HG2 MET C 547     106.785  89.740  99.454  1.00  0.00           H
ATOM   5108  HG3 MET C 547     105.156  89.886  98.874  1.00  0.00           H
ATOM   5109  HE1 MET C 547     104.116  93.492  99.768  1.00  0.00           H
ATOM   5110  HE2 MET C 547     104.404  92.700  98.217  1.00  0.00           H
ATOM   5111  HE3 MET C 547     103.537  91.844  99.501  1.00  0.00           H
ATOM   5112  N   GLY C 548     109.536  89.260  97.316  1.00  0.00           N
ATOM   5113  CA  GLY C 548     110.454  88.239  97.810  1.00  0.00           C
ATOM   5114  C   GLY C 548     111.829  88.806  98.179  1.00  0.00           C
ATOM   5115  O   GLY C 548     112.395  88.402  99.199  1.00  0.00           O
ATOM   5116  H   GLY C 548     109.142  89.105  96.392  1.00  0.00           H
ATOM   5117  HA2 GLY C 548     110.017  87.783  98.696  1.00  0.00           H
ATOM   5118  HA3 GLY C 548     110.567  87.462  97.056  1.00  0.00           H
ATOM   5119  N   TRP C 549     112.354  89.761  97.400  1.00  0.00           N
ATOM   5120  CA  TRP C 549     113.619  90.419  97.724  1.00  0.00           C
ATOM   5121  C   TRP C 549     113.534  91.284  98.993  1.00  0.00           C
ATOM   5122  O   TRP C 549     114.400  91.143  99.854  1.00  0.00           O
ATOM   5123  CB  TRP C 549     114.207  91.170  96.516  1.00 20.00           C
ATOM   5124  CG  TRP C 549     114.973  90.306  95.554  1.00 20.00           C
ATOM   5125  CD1 TRP C 549     114.599  89.960  94.300  1.00 20.00           C
ATOM   5126  CD2 TRP C 549     116.249  89.634  95.788  1.00 20.00           C
ATOM   5127  CE2 TRP C 549     116.597  88.896  94.618  1.00 20.00           C
ATOM   5128  CE3 TRP C 549     117.151  89.570  96.877  1.00 20.00           C
ATOM   5129  NE1 TRP C 549     115.569  89.147  93.738  1.00 20.00           N
ATOM   5130  CZ2 TRP C 549     117.768  88.123  94.535  1.00 20.00           C
ATOM   5131  CZ3 TRP C 549     118.336  88.809  96.801  1.00 20.00           C
ATOM   5132  CH2 TRP C 549     118.642  88.080  95.635  1.00 20.00           C
ATOM   5133  H   TRP C 549     111.861  90.058  96.562  1.00  0.00           H
ATOM   5134  HA  TRP C 549     114.339  89.638  97.982  1.00  0.00           H
ATOM   5135  HB2 TRP C 549     113.426  91.720  95.989  1.00  0.00           H
ATOM   5136  HB3 TRP C 549     114.920  91.920  96.865  1.00  0.00           H
ATOM   5137  HD1 TRP C 549     113.688  90.298  93.827  1.00  0.00           H
ATOM   5138  HE1 TRP C 549     115.556  88.801  92.778  1.00  0.00           H
ATOM   5139  HE3 TRP C 549     116.930  90.118  97.782  1.00  0.00           H
ATOM   5140  HZ2 TRP C 549     117.998  87.574  93.634  1.00  0.00           H
ATOM   5141  HZ3 TRP C 549     119.011  88.783  97.645  1.00  0.00           H
ATOM   5142  HH2 TRP C 549     119.550  87.497  95.584  1.00  0.00           H
ATOM   5143  N   THR C 550     112.480  92.097  99.152  1.00  0.00           N
ATOM   5144  CA  THR C 550     112.300  92.888 100.376  1.00  0.00           C
ATOM   5145  C   THR C 550     111.938  92.024 101.609  1.00  0.00           C
ATOM   5146  O   THR C 550     112.305  92.406 102.718  1.00  0.00           O
ATOM   5147  CB  THR C 550     111.289  94.052 100.200  1.00 20.00           C
ATOM   5148  OG1 THR C 550     111.165  94.849 101.370  1.00  0.00           O
ATOM   5149  CG2 THR C 550     109.890  93.588  99.799  1.00  0.00           C
ATOM   5150  H   THR C 550     111.749  92.141  98.451  1.00  0.00           H
ATOM   5151  HA  THR C 550     113.263  93.351 100.602  1.00  0.00           H
ATOM   5152  HB1 THR C 550     111.666  94.708  99.414  1.00  0.00           H
ATOM   5153  HG1 THR C 550     111.365  94.291 102.136  1.00  0.00           H
ATOM   5154 HG21 THR C 550     109.251  94.428  99.567  1.00  0.00           H
ATOM   5155 HG22 THR C 550     109.939  93.005  98.888  1.00  0.00           H
ATOM   5156 HG23 THR C 550     109.398  92.987 100.565  1.00  0.00           H
ATOM   5157  N   ASN C 551     111.313  90.851 101.418  1.00  0.00           N
ATOM   5158  CA  ASN C 551     111.084  89.852 102.477  1.00  0.00           C
ATOM   5159  C   ASN C 551     112.390  89.296 103.066  1.00  0.00           C
ATOM   5160  O   ASN C 551     112.372  88.807 104.194  1.00  0.00           O
ATOM   5161  CB  ASN C 551     110.208  88.694 101.949  1.00 20.00           C
ATOM   5162  CG  ASN C 551     108.721  89.016 101.868  1.00 20.00           C
ATOM   5163  ND2 ASN C 551     107.929  88.110 101.315  1.00 20.00           N
ATOM   5164  OD1 ASN C 551     108.270  90.065 102.309  1.00 20.00           O
ATOM   5165  H   ASN C 551     110.903  90.647 100.509  1.00  0.00           H
ATOM   5166  HA  ASN C 551     110.550  90.388 103.266  1.00  0.00           H
ATOM   5167  HB2 ASN C 551     110.561  88.347 100.982  1.00  0.00           H
ATOM   5168  HB3 ASN C 551     110.282  87.839 102.621  1.00  0.00           H
ATOM   5169 HD21 ASN C 551     106.935  88.284 101.275  1.00  0.00           H
ATOM   5170 HD22 ASN C 551     108.282  87.203 100.997  1.00  0.00           H
ATOM   5171  N   MET C 552     113.523  89.431 102.358  1.00 30.00           N
ATOM   5172  CA  MET C 552     114.846  89.094 102.888  1.00 30.00           C
ATOM   5173  C   MET C 552     115.254  89.960 104.100  1.00 30.00           C
```

```
ATOM   5174  O   MET C 552     116.043  89.498 104.924  1.00 30.00           O
ATOM   5175  CB  MET C 552     115.889  89.136 101.755  1.00 20.00           C
ATOM   5176  CG  MET C 552     117.188  88.383 102.087  1.00 20.00           C
ATOM   5177  SD  MET C 552     118.319  88.131 100.690  1.00 20.00           S
ATOM   5178  CE  MET C 552     117.318  87.084  99.596  1.00 20.00           C
ATOM   5179  H   MET C 552     113.490  89.881 101.450  1.00  0.00           H
ATOM   5180  HA  MET C 552     114.774  88.064 103.240  1.00  0.00           H
ATOM   5181  HB2 MET C 552     115.451  88.730 100.846  1.00  0.00           H
ATOM   5182  HB3 MET C 552     116.137  90.173 101.524  1.00  0.00           H
ATOM   5183  HG2 MET C 552     117.737  88.930 102.854  1.00  0.00           H
ATOM   5184  HG3 MET C 552     116.962  87.406 102.515  1.00  0.00           H
ATOM   5185  HE1 MET C 552     117.924  86.744  98.757  1.00  0.00           H
ATOM   5186  HE2 MET C 552     116.941  86.208 100.123  1.00  0.00           H
ATOM   5187  HE3 MET C 552     116.477  87.641  99.187  1.00  0.00           H
ATOM   5188  N   LEU C 553     114.647  91.150 104.244  1.00 30.00           N
ATOM   5189  CA  LEU C 553     114.801  92.036 105.401  1.00 30.00           C
ATOM   5190  C   LEU C 553     114.227  91.450 106.705  1.00 30.00           C
ATOM   5191  O   LEU C 553     114.521  91.991 107.770  1.00 30.00           O
ATOM   5192  CB  LEU C 553     114.172  93.417 105.117  1.00 20.00           C
ATOM   5193  CG  LEU C 553     114.788  94.168 103.918  1.00 20.00           C
ATOM   5194  CD1 LEU C 553     113.929  95.385 103.527  1.00 20.00           C
ATOM   5195  CD2 LEU C 553     116.266  94.543 104.150  1.00 20.00           C
ATOM   5196  H   LEU C 553     113.960  91.448 103.558  1.00  0.00           H
ATOM   5197  HA  LEU C 553     115.871  92.170 105.564  1.00  0.00           H
ATOM   5198  HB2 LEU C 553     113.102  93.282 104.960  1.00  0.00           H
ATOM   5199  HB3 LEU C 553     114.249  94.050 106.004  1.00  0.00           H
ATOM   5200  HG  LEU C 553     114.753  93.498 103.061  1.00  0.00           H
ATOM   5201 HD11 LEU C 553     114.408  96.335 103.759  1.00  0.00           H
ATOM   5202 HD12 LEU C 553     113.729  95.376 102.455  1.00  0.00           H
ATOM   5203 HD13 LEU C 553     112.964  95.388 104.034  1.00  0.00           H
ATOM   5204 HD21 LEU C 553     116.507  95.533 103.767  1.00  0.00           H
ATOM   5205 HD22 LEU C 553     116.533  94.532 105.207  1.00  0.00           H
ATOM   5206 HD23 LEU C 553     116.927  93.842 103.639  1.00  0.00           H
ATOM   5207  N   TYR C 554     113.466  90.345 106.634  1.00 30.00           N
ATOM   5208  CA  TYR C 554     113.080  89.533 107.791  1.00 30.00           C
ATOM   5209  C   TYR C 554     114.304  89.059 108.598  1.00 30.00           C
ATOM   5210  O   TYR C 554     114.321  89.178 109.823  1.00 30.00           O
ATOM   5211  CB  TYR C 554     112.207  88.346 107.327  1.00 20.00           C
ATOM   5212  CG  TYR C 554     111.929  87.271 108.366  1.00 20.00           C
ATOM   5213  CD1 TYR C 554     110.942  87.472 109.351  1.00 20.00           C
ATOM   5214  CD2 TYR C 554     112.677  86.075 108.366  1.00 20.00           C
ATOM   5215  CE1 TYR C 554     110.691  86.477 110.314  1.00 20.00           C
ATOM   5216  CE2 TYR C 554     112.443  85.089 109.340  1.00 20.00           C
ATOM   5217  CZ  TYR C 554     111.443  85.286 110.309  1.00 20.00           C
ATOM   5218  OH  TYR C 554     111.207  84.320 111.239  1.00 20.00           O
ATOM   5219  H   TYR C 554     113.217  89.971 105.725  1.00  0.00           H
ATOM   5220  HA  TYR C 554     112.470  90.159 108.439  1.00  0.00           H
ATOM   5221  HB2 TYR C 554     111.259  88.725 106.943  1.00  0.00           H
ATOM   5222  HB3 TYR C 554     112.695  87.846 106.491  1.00  0.00           H
ATOM   5223  HD1 TYR C 554     110.382  88.395 109.374  1.00  0.00           H
ATOM   5224  HD2 TYR C 554     113.442  85.911 107.624  1.00  0.00           H
ATOM   5225  HE1 TYR C 554     109.927  86.645 111.060  1.00  0.00           H
ATOM   5226  HE2 TYR C 554     113.023  84.177 109.332  1.00  0.00           H
ATOM   5227  HH  TYR C 554     110.379  84.454 111.710  1.00  0.00           H
ATOM   5228  N   TYR C 555     115.337  88.593 107.882  1.00 30.00           N
ATOM   5229  CA  TYR C 555     116.546  87.979 108.434  1.00 30.00           C
ATOM   5230  C   TYR C 555     117.561  88.990 109.002  1.00 30.00           C
ATOM   5231  O   TYR C 555     118.702  88.619 109.263  1.00 30.00           O
ATOM   5232  CB  TYR C 555     117.184  87.059 107.375  1.00 20.00           C
ATOM   5233  CG  TYR C 555     116.287  85.927 106.899  1.00 20.00           C
ATOM   5234  CD1 TYR C 555     116.191  84.738 107.652  1.00 20.00           C
ATOM   5235  CD2 TYR C 555     115.547  86.057 105.705  1.00 20.00           C
ATOM   5236  CE1 TYR C 555     115.371  83.684 107.207  1.00 20.00           C
ATOM   5237  CE2 TYR C 555     114.712  85.011 105.272  1.00 20.00           C
ATOM   5238  CZ  TYR C 555     114.629  83.820 106.018  1.00 20.00           C
ATOM   5239  OH  TYR C 555     113.829  82.799 105.599  1.00 20.00           O
ATOM   5240  H   TYR C 555     115.298  88.646 106.872  1.00  0.00           H
ATOM   5241  HA  TYR C 555     116.245  87.353 109.273  1.00  0.00           H
ATOM   5242  HB2 TYR C 555     117.504  87.652 106.517  1.00  0.00           H
ATOM   5243  HB3 TYR C 555     118.095  86.609 107.774  1.00  0.00           H
ATOM   5244  HD1 TYR C 555     116.748  84.624 108.572  1.00  0.00           H
```

```
ATOM   5245  HD2 TYR C 555     115.612  86.963 105.124  1.00  0.00           H
ATOM   5246  HE1 TYR C 555     115.307  82.772 107.783  1.00  0.00           H
ATOM   5247  HE2 TYR C 555     114.134  85.133 104.369  1.00  0.00           H
ATOM   5248  HH  TYR C 555     113.311  83.019 104.820  1.00  0.00           H
ATOM   5249  N   THR C 556     117.138  90.242 109.206  1.00 30.00           N
ATOM   5250  CA  THR C 556     117.893  91.239 109.955  1.00 30.00           C
ATOM   5251  C   THR C 556     117.785  90.987 111.479  1.00 30.00           C
ATOM   5252  O   THR C 556     118.704  91.345 112.217  1.00 30.00           O
ATOM   5253  CB  THR C 556     117.354  92.654 109.636  1.00 20.00           C
ATOM   5254  CG2 THR C 556     117.594  93.065 108.176  1.00 20.00           C
ATOM   5255  OG1 THR C 556     115.968  92.754 109.921  1.00 20.00           O
ATOM   5256  H   THR C 556     116.175  90.476 109.009  1.00  0.00           H
ATOM   5257  HA  THR C 556     118.948  91.186 109.677  1.00  0.00           H
ATOM   5258  HB  THR C 556     117.865  93.377 110.267  1.00  0.00           H
ATOM   5259  HG1 THR C 556     115.446  92.527 109.130  1.00  0.00           H
ATOM   5260 HG21 THR C 556     117.166  94.045 107.962  1.00  0.00           H
ATOM   5261 HG22 THR C 556     118.663  93.124 107.965  1.00  0.00           H
ATOM   5262 HG23 THR C 556     117.170  92.350 107.474  1.00  0.00           H
ATOM   5263  N   ARG C 557     116.710  90.321 111.938  1.00 30.00           N
ATOM   5264  CA  ARG C 557     116.618  89.780 113.294  1.00 30.00           C
ATOM   5265  C   ARG C 557     117.690  88.688 113.483  1.00 30.00           C
ATOM   5266  O   ARG C 557     117.945  87.932 112.552  1.00 30.00           O
ATOM   5267  CB  ARG C 557     115.178  89.294 113.573  1.00 20.00           C
ATOM   5268  CG  ARG C 557     114.764  87.988 112.862  1.00 20.00           C
ATOM   5269  CD  ARG C 557     113.274  87.649 113.000  1.00 20.00           C
ATOM   5270  NE  ARG C 557     112.397  88.695 112.456  1.00 20.00           N
ATOM   5271  CZ  ARG C 557     111.104  88.870 112.774  1.00 20.00           C
ATOM   5272  NH1 ARG C 557     110.507  88.126 113.716  1.00 20.00           N
ATOM   5273  NH2 ARG C 557     110.394  89.808 112.144  1.00 20.00           N1+
ATOM   5274  H   ARG C 557     116.000  90.039 111.275  1.00  0.00           H
ATOM   5275  HA  ARG C 557     116.830  90.597 113.985  1.00  0.00           H
ATOM   5276  HB2 ARG C 557     114.495  90.102 113.303  1.00  0.00           H
ATOM   5277  HB3 ARG C 557     115.049  89.155 114.648  1.00  0.00           H
ATOM   5278  HG2 ARG C 557     115.038  88.002 111.811  1.00  0.00           H
ATOM   5279  HG3 ARG C 557     115.334  87.161 113.288  1.00  0.00           H
ATOM   5280  HD2 ARG C 557     113.051  87.458 114.049  1.00  0.00           H
ATOM   5281  HD3 ARG C 557     113.047  86.739 112.444  1.00  0.00           H
ATOM   5282  HE  ARG C 557     112.787  89.225 111.683  1.00  0.00           H
ATOM   5283 HH11 ARG C 557     109.531  88.254 113.938  1.00  0.00           H
ATOM   5284 HH12 ARG C 557     111.039  87.463 114.261  1.00  0.00           H
ATOM   5285 HH21 ARG C 557     109.425  89.979 112.371  1.00  0.00           H
ATOM   5286 HH22 ARG C 557     110.840  90.420 111.468  1.00  0.00           H
ATOM   5287  N   GLY C 558     118.329  88.650 114.655  1.00 30.00           N
ATOM   5288  CA  GLY C 558     119.492  87.792 114.900  1.00 30.00           C
ATOM   5289  C   GLY C 558     120.810  88.555 114.651  1.00 30.00           C
ATOM   5290  O   GLY C 558     121.868  88.053 115.022  1.00 30.00           O
ATOM   5291  H   GLY C 558     118.083  89.310 115.379  1.00  0.00           H
ATOM   5292  HA2 GLY C 558     119.474  86.893 114.281  1.00  0.00           H
ATOM   5293  HA3 GLY C 558     119.458  87.451 115.933  1.00  0.00           H
ATOM   5294  N   PHE C 559     120.755  89.768 114.076  1.00 30.00           N
ATOM   5295  CA  PHE C 559     121.868  90.709 113.969  1.00 30.00           C
ATOM   5296  C   PHE C 559     121.360  92.005 114.607  1.00 30.00           C
ATOM   5297  O   PHE C 559     120.539  92.688 114.000  1.00 30.00           O
ATOM   5298  CB  PHE C 559     122.254  90.920 112.485  1.00 20.00           C
ATOM   5299  CG  PHE C 559     122.668  89.669 111.733  1.00 20.00           C
ATOM   5300  CD1 PHE C 559     123.967  89.141 111.883  1.00 20.00           C
ATOM   5301  CD2 PHE C 559     121.712  88.938 110.994  1.00 20.00           C
ATOM   5302  CE1 PHE C 559     124.311  87.955 111.247  1.00 20.00           C
ATOM   5303  CE2 PHE C 559     122.075  87.755 110.365  1.00 20.00           C
ATOM   5304  CZ  PHE C 559     123.372  87.271 110.484  1.00 20.00           C
ATOM   5305  H   PHE C 559     119.866  90.119 113.747  1.00  0.00           H
ATOM   5306  HA  PHE C 559     122.743  90.358 114.519  1.00  0.00           H
ATOM   5307  HB2 PHE C 559     121.440  91.389 111.933  1.00  0.00           H
ATOM   5308  HB3 PHE C 559     123.085  91.625 112.435  1.00  0.00           H
ATOM   5309  HD1 PHE C 559     124.699  89.659 112.485  1.00  0.00           H
ATOM   5310  HD2 PHE C 559     120.697  89.300 110.910  1.00  0.00           H
ATOM   5311  HE1 PHE C 559     125.310  87.558 111.351  1.00  0.00           H
ATOM   5312  HE2 PHE C 559     121.345  87.207 109.787  1.00  0.00           H
ATOM   5313  HZ  PHE C 559     123.647  86.349 109.994  1.00  0.00           H
ATOM   5314  N   GLN C 560     121.802  92.302 115.840  1.00 30.00           N
ATOM   5315  CA  GLN C 560     121.264  93.401 116.655  1.00 30.00           C
```

```
ATOM   5316  C   GLN C 560     121.475  94.810 116.054  1.00 30.00           C
ATOM   5317  O   GLN C 560     120.653  95.691 116.302  1.00 30.00           O
ATOM   5318  CB  GLN C 560     121.799  93.263 118.097  1.00 20.00           C
ATOM   5319  CG  GLN C 560     121.098  94.192 119.113  1.00 20.00           C
ATOM   5320  CD  GLN C 560     121.333  93.844 120.588  1.00 20.00           C
ATOM   5321  NE2 GLN C 560     122.416  93.133 120.912  1.00 20.00           N
ATOM   5322  OE1 GLN C 560     120.533  94.225 121.441  1.00 20.00           O
ATOM   5323  H   GLN C 560     122.478  91.701 116.288  1.00  0.00           H
ATOM   5324  HA  GLN C 560     120.183  93.254 116.693  1.00  0.00           H
ATOM   5325  HB2 GLN C 560     121.650  92.229 118.412  1.00  0.00           H
ATOM   5326  HB3 GLN C 560     122.876  93.432 118.113  1.00  0.00           H
ATOM   5327  HG2 GLN C 560     121.398  95.228 118.953  1.00  0.00           H
ATOM   5328  HG3 GLN C 560     120.022  94.147 118.941  1.00  0.00           H
ATOM   5329 HE21 GLN C 560     122.596  92.891 121.876  1.00  0.00           H
ATOM   5330 HE22 GLN C 560     123.066  92.835 120.199  1.00  0.00           H
ATOM   5331  N   GLN C 561     122.522  94.970 115.226  1.00 30.00           N
ATOM   5332  CA  GLN C 561     122.789  96.157 114.410  1.00 30.00           C
ATOM   5333  C   GLN C 561     121.701  96.412 113.349  1.00 30.00           C
ATOM   5334  O   GLN C 561     121.129  97.502 113.309  1.00 30.00           O
ATOM   5335  CB  GLN C 561     124.163  96.003 113.726  1.00 20.00           C
ATOM   5336  CG  GLN C 561     125.361  96.063 114.693  1.00 20.00           C
ATOM   5337  CD  GLN C 561     126.708  95.891 113.983  1.00 20.00           C
ATOM   5338  NE2 GLN C 561     127.774  95.687 114.759  1.00 20.00           N
ATOM   5339  OE1 GLN C 561     126.799  95.950 112.758  1.00 20.00           O
ATOM   5340  H   GLN C 561     123.145  94.190 115.077  1.00  0.00           H
ATOM   5341  HA  GLN C 561     122.821  97.025 115.072  1.00  0.00           H
ATOM   5342  HB2 GLN C 561     124.192  95.059 113.177  1.00  0.00           H
ATOM   5343  HB3 GLN C 561     124.281  96.784 112.974  1.00  0.00           H
ATOM   5344  HG2 GLN C 561     125.365  97.020 115.216  1.00  0.00           H
ATOM   5345  HG3 GLN C 561     125.267  95.287 115.453  1.00  0.00           H
ATOM   5346 HE21 GLN C 561     128.685  95.571 114.341  1.00  0.00           H
ATOM   5347 HE22 GLN C 561     127.681  95.648 115.764  1.00  0.00           H
ATOM   5348  N   MET C 562     121.410  95.377 112.551  1.00 30.00           N
ATOM   5349  CA  MET C 562     120.482  95.450 111.424  1.00 30.00           C
ATOM   5350  C   MET C 562     119.018  95.386 111.871  1.00 30.00           C
ATOM   5351  O   MET C 562     118.187  96.106 111.323  1.00 30.00           O
ATOM   5352  CB  MET C 562     120.764  94.310 110.419  1.00 20.00           C
ATOM   5353  CG  MET C 562     122.199  94.252 109.874  1.00 20.00           C
ATOM   5354  SD  MET C 562     122.710  95.741 108.977  1.00 20.00           S
ATOM   5355  CE  MET C 562     124.377  95.245 108.466  1.00 20.00           C
ATOM   5356  H   MET C 562     121.795  94.471 112.763  1.00  0.00           H
ATOM   5357  HA  MET C 562     120.626  96.405 110.915  1.00  0.00           H
ATOM   5358  HB2 MET C 562     120.084  94.414 109.572  1.00  0.00           H
ATOM   5359  HB3 MET C 562     120.520  93.347 110.871  1.00  0.00           H
ATOM   5360  HG2 MET C 562     122.907  94.070 110.683  1.00  0.00           H
ATOM   5361  HG3 MET C 562     122.288  93.402 109.196  1.00  0.00           H
ATOM   5362  HE1 MET C 562     124.854  96.044 107.900  1.00  0.00           H
ATOM   5363  HE2 MET C 562     124.997  95.025 109.335  1.00  0.00           H
ATOM   5364  HE3 MET C 562     124.335  94.359 107.832  1.00  0.00           H
ATOM   5365  N   GLY C 563     118.711  94.502 112.826  1.00 30.00           N
ATOM   5366  CA  GLY C 563     117.333  94.168 113.170  1.00 30.00           C
ATOM   5367  C   GLY C 563     116.630  95.323 113.882  1.00 30.00           C
ATOM   5368  O   GLY C 563     115.470  95.591 113.578  1.00 30.00           O
ATOM   5369  H   GLY C 563     119.435  93.902 113.214  1.00  0.00           H
ATOM   5370  HA2 GLY C 563     116.769  93.911 112.272  1.00  0.00           H
ATOM   5371  HA3 GLY C 563     117.329  93.282 113.795  1.00  0.00           H
ATOM   5372  N   ILE C 564     117.321  96.047 114.777  1.00 30.00           N
ATOM   5373  CA  ILE C 564     116.727  97.209 115.455  1.00 30.00           C
ATOM   5374  C   ILE C 564     116.455  98.358 114.459  1.00 30.00           C
ATOM   5375  O   ILE C 564     115.412  99.004 114.555  1.00 30.00           O
ATOM   5376  CB  ILE C 564     117.616  97.717 116.633  1.00 20.00           C
ATOM   5377  CG1 ILE C 564     117.741  96.618 117.715  1.00 20.00           C
ATOM   5378  CG2 ILE C 564     117.121  99.042 117.267  1.00 20.00           C
ATOM   5379  CD1 ILE C 564     118.738  96.931 118.840  1.00 20.00           C
ATOM   5380  H   ILE C 564     118.293  95.856 114.977  1.00  0.00           H
ATOM   5381  HA  ILE C 564     115.765  96.899 115.868  1.00  0.00           H
ATOM   5382  HB  ILE C 564     118.617  97.900 116.239  1.00  0.00           H
ATOM   5383 HG12 ILE C 564     116.760  96.427 118.145  1.00  0.00           H
ATOM   5384 HG13 ILE C 564     118.044  95.673 117.262  1.00  0.00           H
ATOM   5385 HG21 ILE C 564     117.760  99.370 118.085  1.00  0.00           H
ATOM   5386 HG22 ILE C 564     117.113  99.870 116.559  1.00  0.00           H
```

```
ATOM   5387 HG23 ILE C 564     116.111  98.933 117.663  1.00  0.00           H
ATOM   5388 HD11 ILE C 564     118.857  96.069 119.495  1.00  0.00           H
ATOM   5389 HD12 ILE C 564     119.719  97.189 118.442  1.00  0.00           H
ATOM   5390 HD13 ILE C 564     118.400  97.757 119.463  1.00  0.00           H
ATOM   5391  N   TYR C 565     117.328  98.503 113.452  1.00 30.00           N
ATOM   5392  CA  TYR C 565     117.178  99.426 112.324  1.00 30.00           C
ATOM   5393  C   TYR C 565     115.984  99.053 111.415  1.00 30.00           C
ATOM   5394  O   TYR C 565     115.199  99.924 111.045  1.00 30.00           O
ATOM   5395  CB  TYR C 565     118.530  99.463 111.576  1.00 20.00           C
ATOM   5396  CG  TYR C 565     118.721 100.495 110.478  1.00 20.00           C
ATOM   5397  CD1 TYR C 565     118.861 101.858 110.812  1.00 20.00           C
ATOM   5398  CD2 TYR C 565     118.844 100.092 109.130  1.00 20.00           C
ATOM   5399  CE1 TYR C 565     119.160 102.804 109.813  1.00 20.00           C
ATOM   5400  CE2 TYR C 565     119.123 101.043 108.129  1.00 20.00           C
ATOM   5401  CZ  TYR C 565     119.293 102.397 108.472  1.00 20.00           C
ATOM   5402  OH  TYR C 565     119.596 103.312 107.510  1.00 20.00           O
ATOM   5403  H   TYR C 565     118.111  97.868 113.405  1.00  0.00           H
ATOM   5404  HA  TYR C 565     116.979 100.420 112.731  1.00  0.00           H
ATOM   5405  HB2 TYR C 565     119.322  99.644 112.304  1.00  0.00           H
ATOM   5406  HB3 TYR C 565     118.754  98.486 111.156  1.00  0.00           H
ATOM   5407  HD1 TYR C 565     118.764 102.180 111.839  1.00  0.00           H
ATOM   5408  HD2 TYR C 565     118.747  99.051 108.856  1.00  0.00           H
ATOM   5409  HE1 TYR C 565     119.296 103.839 110.087  1.00  0.00           H
ATOM   5410  HE2 TYR C 565     119.227 100.726 107.101  1.00  0.00           H
ATOM   5411  HH  TYR C 565     119.591 104.213 107.844  1.00  0.00           H
ATOM   5412  N   ALA C 566     115.813  97.756 111.117  1.00 30.00           N
ATOM   5413  CA  ALA C 566     114.697  97.243 110.317  1.00 30.00           C
ATOM   5414  C   ALA C 566     113.324  97.415 110.994  1.00 30.00           C
ATOM   5415  O   ALA C 566     112.348  97.723 110.307  1.00 30.00           O
ATOM   5416  CB  ALA C 566     114.952  95.774 109.965  1.00 20.00           C
ATOM   5417  H   ALA C 566     116.507  97.080 111.421  1.00  0.00           H
ATOM   5418  HA  ALA C 566     114.670  97.810 109.384  1.00  0.00           H
ATOM   5419  HB1 ALA C 566     114.172  95.383 109.310  1.00  0.00           H
ATOM   5420  HB2 ALA C 566     115.903  95.652 109.446  1.00  0.00           H
ATOM   5421  HB3 ALA C 566     114.980  95.148 110.858  1.00  0.00           H
ATOM   5422  N   VAL C 567     113.273  97.260 112.326  1.00 30.00           N
ATOM   5423  CA  VAL C 567     112.075  97.514 113.132  1.00 30.00           C
ATOM   5424  C   VAL C 567     111.622  98.990 113.059  1.00 30.00           C
ATOM   5425  O   VAL C 567     110.421  99.255 112.998  1.00 30.00           O
ATOM   5426  CB  VAL C 567     112.273  97.082 114.617  1.00 20.00           C
ATOM   5427  CG1 VAL C 567     111.192  97.577 115.599  1.00 20.00           C
ATOM   5428  CG2 VAL C 567     112.348  95.549 114.732  1.00 20.00           C
ATOM   5429  H   VAL C 567     114.104  96.948 112.820  1.00  0.00           H
ATOM   5430  HA  VAL C 567     111.270  96.910 112.709  1.00  0.00           H
ATOM   5431  HB  VAL C 567     113.229  97.481 114.959  1.00  0.00           H
ATOM   5432 HG11 VAL C 567     111.378  97.175 116.591  1.00  0.00           H
ATOM   5433 HG12 VAL C 567     111.169  98.662 115.697  1.00  0.00           H
ATOM   5434 HG13 VAL C 567     110.199  97.252 115.286  1.00  0.00           H
ATOM   5435 HG21 VAL C 567     112.617  95.235 115.741  1.00  0.00           H
ATOM   5436 HG22 VAL C 567     111.386  95.099 114.490  1.00  0.00           H
ATOM   5437 HG23 VAL C 567     113.076  95.114 114.050  1.00  0.00           H
ATOM   5438  N   MET C 568     112.569  99.936 113.012  1.00 30.00           N
ATOM   5439  CA  MET C 568     112.269 101.352 112.771  1.00 30.00           C
ATOM   5440  C   MET C 568     111.613 101.587 111.400  1.00 30.00           C
ATOM   5441  O   MET C 568     110.671 102.369 111.323  1.00 30.00           O
ATOM   5442  CB  MET C 568     113.544 102.206 112.902  1.00 20.00           C
ATOM   5443  CG  MET C 568     114.142 102.239 114.313  1.00 20.00           C
ATOM   5444  SD  MET C 568     115.805 102.950 114.341  1.00 20.00           S
ATOM   5445  CE  MET C 568     116.045 103.141 116.123  1.00 20.00           C
ATOM   5446  H   MET C 568     113.546  99.687 113.087  1.00  0.00           H
ATOM   5447  HA  MET C 568     111.561 101.678 113.536  1.00  0.00           H
ATOM   5448  HB2 MET C 568     114.299 101.868 112.194  1.00  0.00           H
ATOM   5449  HB3 MET C 568     113.317 103.234 112.613  1.00  0.00           H
ATOM   5450  HG2 MET C 568     113.499 102.831 114.963  1.00  0.00           H
ATOM   5451  HG3 MET C 568     114.189 101.243 114.748  1.00  0.00           H
ATOM   5452  HE1 MET C 568     116.962 103.696 116.323  1.00  0.00           H
ATOM   5453  HE2 MET C 568     115.207 103.686 116.558  1.00  0.00           H
ATOM   5454  HE3 MET C 568     116.122 102.166 116.603  1.00  0.00           H
ATOM   5455  N   ILE C 569     112.115 100.927 110.349  1.00 30.00           N
ATOM   5456  CA  ILE C 569     111.582 101.069 108.993  1.00 30.00           C
ATOM   5457  C   ILE C 569     110.129 100.543 108.912  1.00 30.00           C
```

```
ATOM   5458  O   ILE C 569     109.260 101.250 108.404  1.00 30.00           O
ATOM   5459  CB  ILE C 569     112.504 100.383 107.938  1.00 20.00           C
ATOM   5460  CG1 ILE C 569     113.909 101.042 107.925  1.00 20.00           C
ATOM   5461  CG2 ILE C 569     111.927 100.362 106.505  1.00 20.00           C
ATOM   5462  CD1 ILE C 569     115.030 100.135 107.395  1.00 20.00           C
ATOM   5463  H   ILE C 569     112.908 100.310 110.478  1.00  0.00           H
ATOM   5464  HA  ILE C 569     111.548 102.136 108.762  1.00  0.00           H
ATOM   5465  HB  ILE C 569     112.631  99.345 108.246  1.00  0.00           H
ATOM   5466 HG12 ILE C 569     113.879 101.969 107.351  1.00  0.00           H
ATOM   5467 HG13 ILE C 569     114.201 101.346 108.930  1.00  0.00           H
ATOM   5468 HG21 ILE C 569     112.612  99.888 105.802  1.00  0.00           H
ATOM   5469 HG22 ILE C 569     110.989  99.812 106.450  1.00  0.00           H
ATOM   5470 HG23 ILE C 569     111.733 101.373 106.141  1.00  0.00           H
ATOM   5471 HD11 ILE C 569     115.620  99.726 108.216  1.00  0.00           H
ATOM   5472 HD12 ILE C 569     114.651  99.296 106.812  1.00  0.00           H
ATOM   5473 HD13 ILE C 569     115.716 100.701 106.765  1.00  0.00           H
ATOM   5474  N   GLU C 570     109.882  99.376 109.528  1.00  0.00           N
ATOM   5475  CA  GLU C 570     108.560  98.792 109.776  1.00  0.00           C
ATOM   5476  C   GLU C 570     107.587  99.794 110.447  1.00  0.00           C
ATOM   5477  O   GLU C 570     106.545 100.131 109.880  1.00  0.00           O
ATOM   5478  CB  GLU C 570     108.761  97.481 110.587  1.00 20.00           C
ATOM   5479  CG  GLU C 570     107.498  96.830 111.197  1.00 20.00           C
ATOM   5480  CD  GLU C 570     107.763  95.700 112.198  1.00 20.00           C
ATOM   5481  OE1 GLU C 570     108.941  95.355 112.436  1.00 20.00           O
ATOM   5482  OE2 GLU C 570     106.758  95.229 112.771  1.00 20.00           O1-
ATOM   5483  H   GLU C 570     110.670  98.880 109.933  1.00  0.00           H
ATOM   5484  HA  GLU C 570     108.122  98.531 108.810  1.00  0.00           H
ATOM   5485  HB2 GLU C 570     109.229  96.746 109.932  1.00  0.00           H
ATOM   5486  HB3 GLU C 570     109.483  97.656 111.379  1.00  0.00           H
ATOM   5487  HG2 GLU C 570     106.903  97.564 111.728  1.00  0.00           H
ATOM   5488  HG3 GLU C 570     106.866  96.449 110.397  1.00  0.00           H
ATOM   5489  N   LYS C 571     107.947 100.288 111.640  1.00  0.00           N
ATOM   5490  CA  LYS C 571     107.066 101.136 112.450  1.00  0.00           C
ATOM   5491  C   LYS C 571     106.818 102.558 111.890  1.00  0.00           C
ATOM   5492  O   LYS C 571     105.835 103.181 112.294  1.00  0.00           O
ATOM   5493  CB  LYS C 571     107.551 101.148 113.919  1.00 20.00           C
ATOM   5494  CG  LYS C 571     107.542  99.778 114.649  1.00 20.00           C
ATOM   5495  CD  LYS C 571     106.269  98.921 114.494  1.00 20.00           C
ATOM   5496  CE  LYS C 571     106.347  97.593 115.270  1.00 20.00           C
ATOM   5497  NZ  LYS C 571     105.282  96.656 114.874  1.00 20.00           N1+
ATOM   5498  H   LYS C 571     108.833 100.000 112.047  1.00  0.00           H
ATOM   5499  HA  LYS C 571     106.080 100.673 112.433  1.00  0.00           H
ATOM   5500  HB2 LYS C 571     108.558 101.567 113.963  1.00  0.00           H
ATOM   5501  HB3 LYS C 571     106.932 101.840 114.487  1.00  0.00           H
ATOM   5502  HG2 LYS C 571     108.385  99.184 114.308  1.00  0.00           H
ATOM   5503  HG3 LYS C 571     107.728  99.953 115.709  1.00  0.00           H
ATOM   5504  HD2 LYS C 571     105.396  99.486 114.821  1.00  0.00           H
ATOM   5505  HD3 LYS C 571     106.108  98.691 113.443  1.00  0.00           H
ATOM   5506  HE2 LYS C 571     107.302  97.098 115.087  1.00  0.00           H
ATOM   5507  HE3 LYS C 571     106.280  97.778 116.342  1.00  0.00           H
ATOM   5508  HZ1 LYS C 571     105.308  95.845 115.474  1.00  0.00           H
ATOM   5509  HZ2 LYS C 571     104.373  97.091 114.938  1.00  0.00           H
ATOM   5510  HZ3 LYS C 571     105.455  96.348 113.925  1.00  0.00           H
ATOM   5511  N   MET C 572     107.666 103.025 110.965  1.00  0.00           N
ATOM   5512  CA  MET C 572     107.543 104.293 110.237  1.00  0.00           C
ATOM   5513  C   MET C 572     106.713 104.157 108.948  1.00  0.00           C
ATOM   5514  O   MET C 572     106.187 105.165 108.483  1.00  0.00           O
ATOM   5515  CB  MET C 572     108.946 104.838 109.927  1.00 20.00           C
ATOM   5516  CG  MET C 572     109.737 105.228 111.185  1.00 20.00           C
ATOM   5517  SD  MET C 572     109.390 106.870 111.853  1.00 20.00           S
ATOM   5518  CE  MET C 572     110.660 106.867 113.139  1.00 20.00           C
ATOM   5519  H   MET C 572     108.449 102.441 110.704  1.00  0.00           H
ATOM   5520  HA  MET C 572     107.032 105.024 110.867  1.00  0.00           H
ATOM   5521  HB2 MET C 572     109.514 104.105 109.352  1.00  0.00           H
ATOM   5522  HB3 MET C 572     108.861 105.717 109.285  1.00  0.00           H
ATOM   5523  HG2 MET C 572     109.563 104.520 111.995  1.00  0.00           H
ATOM   5524  HG3 MET C 572     110.802 105.170 110.965  1.00  0.00           H
ATOM   5525  HE1 MET C 572     110.705 107.834 113.636  1.00  0.00           H
ATOM   5526  HE2 MET C 572     110.456 106.098 113.882  1.00  0.00           H
ATOM   5527  HE3 MET C 572     111.631 106.654 112.695  1.00  0.00           H
ATOM   5528  N   ILE C 573     106.574 102.937 108.408  1.00  0.00           N
```

```
ATOM   5529  CA  ILE C 573     105.581 102.621 107.378  1.00  0.00           C
ATOM   5530  C   ILE C 573     104.176 102.573 108.019  1.00  0.00           C
ATOM   5531  O   ILE C 573     103.227 103.166 107.505  1.00  0.00           O
ATOM   5532  CB  ILE C 573     105.908 101.279 106.646  1.00 20.00           C
ATOM   5533  CG1 ILE C 573     107.175 101.435 105.772  1.00 20.00           C
ATOM   5534  CG2 ILE C 573     104.759 100.692 105.794  1.00 20.00           C
ATOM   5535  CD1 ILE C 573     107.832 100.105 105.367  1.00 20.00           C
ATOM   5536  H   ILE C 573     107.106 102.155 108.773  1.00  0.00           H
ATOM   5537  HA  ILE C 573     105.578 103.419 106.635  1.00  0.00           H
ATOM   5538  HB  ILE C 573     106.134 100.534 107.409  1.00  0.00           H
ATOM   5539 HG12 ILE C 573     106.928 102.005 104.876  1.00  0.00           H
ATOM   5540 HG13 ILE C 573     107.923 102.031 106.296  1.00  0.00           H
ATOM   5541 HG21 ILE C 573     105.068  99.778 105.288  1.00  0.00           H
ATOM   5542 HG22 ILE C 573     103.893 100.421 106.398  1.00  0.00           H
ATOM   5543 HG23 ILE C 573     104.430 101.398 105.032  1.00  0.00           H
ATOM   5544 HD11 ILE C 573     108.869 100.264 105.070  1.00  0.00           H
ATOM   5545 HD12 ILE C 573     107.832  99.391 106.191  1.00  0.00           H
ATOM   5546 HD13 ILE C 573     107.317  99.645 104.523  1.00  0.00           H
ATOM   5547  N   LEU C 574     104.085 101.942 109.197  1.00  0.00           N
ATOM   5548  CA  LEU C 574     102.852 101.816 109.979  1.00  0.00           C
ATOM   5549  C   LEU C 574     102.350 103.138 110.613  1.00  0.00           C
ATOM   5550  O   LEU C 574     101.292 103.123 111.240  1.00  0.00           O
ATOM   5551  CB  LEU C 574     103.048 100.714 111.048  1.00 20.00           C
ATOM   5552  CG  LEU C 574     103.259  99.286 110.481  1.00 20.00           C
ATOM   5553  CD1 LEU C 574     103.674  98.298 111.592  1.00 20.00           C
ATOM   5554  CD2 LEU C 574     102.034  98.780 109.688  1.00 20.00           C
ATOM   5555  H   LEU C 574     104.901 101.443 109.538  1.00  0.00           H
ATOM   5556  HA  LEU C 574     102.060 101.502 109.297  1.00  0.00           H
ATOM   5557  HB2 LEU C 574     103.895 100.993 111.675  1.00  0.00           H
ATOM   5558  HB3 LEU C 574     102.186 100.691 111.717  1.00  0.00           H
ATOM   5559  HG  LEU C 574     104.093  99.321 109.780  1.00  0.00           H
ATOM   5560 HD11 LEU C 574     104.600  97.788 111.323  1.00  0.00           H
ATOM   5561 HD12 LEU C 574     103.844  98.798 112.544  1.00  0.00           H
ATOM   5562 HD13 LEU C 574     102.928  97.524 111.775  1.00  0.00           H
ATOM   5563 HD21 LEU C 574     101.782  97.743 109.912  1.00  0.00           H
ATOM   5564 HD22 LEU C 574     101.141  99.373 109.888  1.00  0.00           H
ATOM   5565 HD23 LEU C 574     102.224  98.832 108.615  1.00  0.00           H
ATOM   5566  N   ARG C 575     103.086 104.249 110.465  1.00  0.00           N
ATOM   5567  CA  ARG C 575     102.752 105.566 111.018  1.00  0.00           C
ATOM   5568  C   ARG C 575     102.891 106.650 109.936  1.00  0.00           C
ATOM   5569  O   ARG C 575     101.872 107.164 109.470  1.00  0.00           O
ATOM   5570  CB  ARG C 575     103.631 105.873 112.254  1.00 20.00           C
ATOM   5571  CG  ARG C 575     103.312 105.013 113.489  1.00 20.00           C
ATOM   5572  CD  ARG C 575     104.266 105.303 114.658  1.00 20.00           C
ATOM   5573  NE  ARG C 575     103.873 104.600 115.889  1.00 20.00           N
ATOM   5574  CZ  ARG C 575     103.964 103.278 116.120  1.00 20.00           C
ATOM   5575  NH1 ARG C 575     104.446 102.436 115.196  1.00 20.00           N
ATOM   5576  NH2 ARG C 575     103.573 102.792 117.305  1.00 20.00           N1+
ATOM   5577  H   ARG C 575     103.930 104.202 109.911  1.00  0.00           H
ATOM   5578  HA  ARG C 575     101.708 105.581 111.337  1.00  0.00           H
ATOM   5579  HB2 ARG C 575     104.686 105.763 111.997  1.00  0.00           H
ATOM   5580  HB3 ARG C 575     103.500 106.919 112.534  1.00  0.00           H
ATOM   5581  HG2 ARG C 575     102.272 105.124 113.799  1.00  0.00           H
ATOM   5582  HG3 ARG C 575     103.430 103.970 113.203  1.00  0.00           H
ATOM   5583  HD2 ARG C 575     105.272 104.960 114.413  1.00  0.00           H
ATOM   5584  HD3 ARG C 575     104.347 106.377 114.832  1.00  0.00           H
ATOM   5585  HE  ARG C 575     103.474 105.182 116.611  1.00  0.00           H
ATOM   5586 HH11 ARG C 575     104.513 101.447 115.386  1.00  0.00           H
ATOM   5587 HH12 ARG C 575     104.752 102.794 114.300  1.00  0.00           H
ATOM   5588 HH21 ARG C 575     103.654 101.806 117.509  1.00  0.00           H
ATOM   5589 HH22 ARG C 575     103.223 103.410 118.022  1.00  0.00           H
ATOM   5590  N   ASP C 576     104.143 106.984 109.573  1.00 30.00           N
ATOM   5591  CA  ASP C 576     104.539 108.071 108.661  1.00 30.00           C
ATOM   5592  C   ASP C 576     104.412 107.713 107.162  1.00 30.00           C
ATOM   5593  O   ASP C 576     105.207 108.185 106.349  1.00 30.00           O
ATOM   5594  CB  ASP C 576     105.943 108.662 108.981  1.00 20.00           C
ATOM   5595  CG  ASP C 576     106.209 109.078 110.434  1.00 20.00           C
ATOM   5596  OD1 ASP C 576     105.255 109.098 111.242  1.00 20.00           O
ATOM   5597  OD2 ASP C 576     107.375 109.438 110.700  1.00 20.00           O1-
ATOM   5598  H   ASP C 576     104.917 106.493 109.997  1.00  0.00           H
ATOM   5599  HA  ASP C 576     103.827 108.885 108.815  1.00  0.00           H
```

```
ATOM   5600  HB2 ASP C 576     106.703 107.928 108.706  1.00  0.00           H
ATOM   5601  HB3 ASP C 576     106.100 109.550 108.371  1.00  0.00           H
ATOM   5602  N   LEU C 577     103.469 106.846 106.797  1.00  0.00           N
ATOM   5603  CA  LEU C 577     103.079 106.630 105.404  1.00  0.00           C
ATOM   5604  C   LEU C 577     101.557 106.740 105.355  1.00  0.00           C
ATOM   5605  O   LEU C 577     101.052 107.501 104.540  1.00  0.00           O
ATOM   5606  CB  LEU C 577     103.603 105.298 104.804  1.00 20.00           C
ATOM   5607  CG  LEU C 577     104.904 105.409 103.967  1.00 20.00           C
ATOM   5608  CD1 LEU C 577     106.165 105.626 104.829  1.00 20.00           C
ATOM   5609  CD2 LEU C 577     105.060 104.196 103.025  1.00 20.00           C
ATOM   5610  H   LEU C 577     102.894 106.451 107.524  1.00  0.00           H
ATOM   5611  HA  LEU C 577     103.448 107.445 104.778  1.00  0.00           H
ATOM   5612  HB2 LEU C 577     103.712 104.538 105.569  1.00  0.00           H
ATOM   5613  HB3 LEU C 577     102.834 104.914 104.131  1.00  0.00           H
ATOM   5614  HG  LEU C 577     104.810 106.284 103.321  1.00  0.00           H
ATOM   5615 HD11 LEU C 577     106.633 106.583 104.596  1.00  0.00           H
ATOM   5616 HD12 LEU C 577     105.938 105.635 105.893  1.00  0.00           H
ATOM   5617 HD13 LEU C 577     106.923 104.858 104.686  1.00  0.00           H
ATOM   5618 HD21 LEU C 577     104.749 104.465 102.015  1.00  0.00           H
ATOM   5619 HD22 LEU C 577     106.087 103.838 102.952  1.00  0.00           H
ATOM   5620 HD23 LEU C 577     104.447 103.350 103.337  1.00  0.00           H
ATOM   5621  N   CYS C 578     100.853 106.073 106.281  1.00  0.00           N
ATOM   5622  CA  CYS C 578      99.396 106.156 106.444  1.00  0.00           C
ATOM   5623  C   CYS C 578      98.898 107.612 106.617  1.00  0.00           C
ATOM   5624  O   CYS C 578      98.042 108.072 105.861  1.00  0.00           O
ATOM   5625  CB  CYS C 578      98.929 105.227 107.589  1.00 20.00           C
ATOM   5626  SG  CYS C 578      97.125 105.202 107.820  1.00 20.00           S
ATOM   5627  H   CYS C 578     101.339 105.424 106.883  1.00  0.00           H
ATOM   5628  HA  CYS C 578      98.958 105.785 105.515  1.00  0.00           H
ATOM   5629  HB2 CYS C 578      99.252 104.204 107.393  1.00  0.00           H
ATOM   5630  HB3 CYS C 578      99.392 105.523 108.532  1.00  0.00           H
ATOM   5631  HG  CYS C 578      96.843 104.587 106.666  1.00  0.00           H
ATOM   5632  N   ARG C 579      99.500 108.328 107.574  1.00  0.00           N
ATOM   5633  CA  ARG C 579      99.201 109.730 107.862  1.00  0.00           C
ATOM   5634  C   ARG C 579      99.844 110.700 106.854  1.00  0.00           C
ATOM   5635  O   ARG C 579      99.236 111.735 106.582  1.00  0.00           O
ATOM   5636  CB  ARG C 579      99.628 110.043 109.311  1.00 20.00           C
ATOM   5637  CG  ARG C 579      98.794 109.269 110.352  1.00 20.00           C
ATOM   5638  CD  ARG C 579      99.106 109.616 111.816  1.00 20.00           C
ATOM   5639  NE  ARG C 579     100.502 109.343 112.182  1.00 20.00           N
ATOM   5640  CZ  ARG C 579     101.522 110.217 112.164  1.00 20.00           C
ATOM   5641  NH1 ARG C 579     101.346 111.501 111.824  1.00 20.00           N
ATOM   5642  NH2 ARG C 579     102.744 109.789 112.495  1.00 20.00           N1+
ATOM   5643  H   ARG C 579     100.277 107.926 108.081  1.00  0.00           H
ATOM   5644  HA  ARG C 579      98.121 109.874 107.797  1.00  0.00           H
ATOM   5645  HB2 ARG C 579     100.689 109.819 109.436  1.00  0.00           H
ATOM   5646  HB3 ARG C 579      99.515 111.111 109.502  1.00  0.00           H
ATOM   5647  HG2 ARG C 579      97.761 109.572 110.176  1.00  0.00           H
ATOM   5648  HG3 ARG C 579      98.813 108.188 110.204  1.00  0.00           H
ATOM   5649  HD2 ARG C 579      98.734 110.591 112.132  1.00  0.00           H
ATOM   5650  HD3 ARG C 579      98.556 108.891 112.416  1.00  0.00           H
ATOM   5651  HE  ARG C 579     100.709 108.380 112.406  1.00  0.00           H
ATOM   5652 HH11 ARG C 579     102.128 112.141 111.817  1.00  0.00           H
ATOM   5653 HH12 ARG C 579     100.427 111.838 111.576  1.00  0.00           H
ATOM   5654 HH21 ARG C 579     102.906 108.823 112.732  1.00  0.00           H
ATOM   5655 HH22 ARG C 579     103.549 110.396 112.416  1.00  0.00           H
ATOM   5656  N   PHE C 580     101.028 110.363 106.307  1.00  0.00           N
ATOM   5657  CA  PHE C 580     101.725 111.201 105.326  1.00  0.00           C
ATOM   5658  C   PHE C 580     101.085 111.174 103.928  1.00  0.00           C
ATOM   5659  O   PHE C 580     100.833 112.245 103.374  1.00  0.00           O
ATOM   5660  CB  PHE C 580     103.239 110.890 105.292  1.00 20.00           C
ATOM   5661  CG  PHE C 580     104.022 111.614 104.203  1.00 20.00           C
ATOM   5662  CD1 PHE C 580     104.322 112.987 104.338  1.00 20.00           C
ATOM   5663  CD2 PHE C 580     104.291 110.976 102.972  1.00 20.00           C
ATOM   5664  CE1 PHE C 580     104.919 113.677 103.291  1.00 20.00           C
ATOM   5665  CE2 PHE C 580     104.885 111.684 101.935  1.00 20.00           C
ATOM   5666  CZ  PHE C 580     105.202 113.027 102.096  1.00 20.00           C
ATOM   5667  H   PHE C 580     101.473 109.493 106.562  1.00  0.00           H
ATOM   5668  HA  PHE C 580     101.644 112.234 105.672  1.00  0.00           H
ATOM   5669  HB2 PHE C 580     103.677 111.166 106.253  1.00  0.00           H
ATOM   5670  HB3 PHE C 580     103.397 109.818 105.185  1.00  0.00           H
```

```
ATOM   5671  HD1 PHE C 580     104.083 113.507 105.254  1.00  0.00           H
ATOM   5672  HD2 PHE C 580     104.028 109.939 102.829  1.00  0.00           H
ATOM   5673  HE1 PHE C 580     105.157 114.724 103.403  1.00  0.00           H
ATOM   5674  HE2 PHE C 580     105.093 111.191 100.997  1.00  0.00           H
ATOM   5675  HZ  PHE C 580     105.661 113.572 101.285  1.00  0.00           H
ATOM   5676  N   MET C 581     100.820 109.975 103.395  1.00  0.00           N
ATOM   5677  CA  MET C 581     100.325 109.804 102.033  1.00  0.00           C
ATOM   5678  C   MET C 581      98.881 110.283 101.869  1.00  0.00           C
ATOM   5679  O   MET C 581      98.556 110.746 100.780  1.00  0.00           O
ATOM   5680  CB  MET C 581     100.499 108.354 101.538  1.00 20.00           C
ATOM   5681  CG  MET C 581     101.965 107.913 101.366  1.00 20.00           C
ATOM   5682  SD  MET C 581     102.946 108.869 100.170  1.00 20.00           S
ATOM   5683  CE  MET C 581     102.103 108.472  98.614  1.00 20.00           C
ATOM   5684  H   MET C 581     100.991 109.118 103.918  1.00  0.00           H
ATOM   5685  HA  MET C 581     100.928 110.442 101.387  1.00  0.00           H
ATOM   5686  HB2 MET C 581      99.985 107.668 102.211  1.00  0.00           H
ATOM   5687  HB3 MET C 581      99.988 108.237 100.582  1.00  0.00           H
ATOM   5688  HG2 MET C 581     102.481 107.965 102.325  1.00  0.00           H
ATOM   5689  HG3 MET C 581     101.997 106.865 101.069  1.00  0.00           H
ATOM   5690  HE1 MET C 581     102.611 108.956  97.780  1.00  0.00           H
ATOM   5691  HE2 MET C 581     102.107 107.395  98.439  1.00  0.00           H
ATOM   5692  HE3 MET C 581     101.071 108.823  98.625  1.00  0.00           H
ATOM   5693  N   PHE C 582      98.062 110.248 102.936  1.00  0.00           N
ATOM   5694  CA  PHE C 582      96.733 110.864 102.918  1.00  0.00           C
ATOM   5695  C   PHE C 582      96.789 112.370 102.604  1.00  0.00           C
ATOM   5696  O   PHE C 582      96.100 112.823 101.691  1.00  0.00           O
ATOM   5697  CB  PHE C 582      95.952 110.549 104.216  1.00 20.00           C
ATOM   5698  CG  PHE C 582      94.641 111.309 104.368  1.00 20.00           C
ATOM   5699  CD1 PHE C 582      93.515 110.933 103.605  1.00 20.00           C
ATOM   5700  CD2 PHE C 582      94.594 112.513 105.106  1.00 20.00           C
ATOM   5701  CE1 PHE C 582      92.368 111.717 103.622  1.00 20.00           C
ATOM   5702  CE2 PHE C 582      93.438 113.284 105.109  1.00 20.00           C
ATOM   5703  CZ  PHE C 582      92.330 112.886 104.371  1.00 20.00           C
ATOM   5704  H   PHE C 582      98.405 109.926 103.830  1.00  0.00           H
ATOM   5705  HA  PHE C 582      96.177 110.401 102.100  1.00  0.00           H
ATOM   5706  HB2 PHE C 582      95.723 109.482 104.235  1.00  0.00           H
ATOM   5707  HB3 PHE C 582      96.578 110.736 105.090  1.00  0.00           H
ATOM   5708  HD1 PHE C 582      93.547 110.044 102.993  1.00  0.00           H
ATOM   5709  HD2 PHE C 582      95.461 112.849 105.656  1.00  0.00           H
ATOM   5710  HE1 PHE C 582      91.507 111.423 103.040  1.00  0.00           H
ATOM   5711  HE2 PHE C 582      93.405 114.202 105.677  1.00  0.00           H
ATOM   5712  HZ  PHE C 582      91.437 113.494 104.373  1.00  0.00           H
ATOM   5713  N   VAL C 583      97.638 113.094 103.351  1.00  0.00           N
ATOM   5714  CA  VAL C 583      97.845 114.533 103.207  1.00  0.00           C
ATOM   5715  C   VAL C 583      98.522 114.892 101.868  1.00  0.00           C
ATOM   5716  O   VAL C 583      98.067 115.830 101.216  1.00  0.00           O
ATOM   5717  CB  VAL C 583      98.668 115.108 104.396  1.00 20.00           C
ATOM   5718  CG1 VAL C 583      99.052 116.594 104.252  1.00 20.00           C
ATOM   5719  CG2 VAL C 583      97.922 114.911 105.729  1.00 20.00           C
ATOM   5720  H   VAL C 583      98.185 112.637 104.067  1.00  0.00           H
ATOM   5721  HA  VAL C 583      96.861 115.008 103.215  1.00  0.00           H
ATOM   5722  HB  VAL C 583      99.600 114.544 104.468  1.00  0.00           H
ATOM   5723 HG11 VAL C 583      99.546 116.962 105.151  1.00  0.00           H
ATOM   5724 HG12 VAL C 583      99.742 116.758 103.426  1.00  0.00           H
ATOM   5725 HG13 VAL C 583      98.171 117.215 104.081  1.00  0.00           H
ATOM   5726 HG21 VAL C 583      98.508 115.284 106.569  1.00  0.00           H
ATOM   5727 HG22 VAL C 583      96.968 115.438 105.729  1.00  0.00           H
ATOM   5728 HG23 VAL C 583      97.717 113.859 105.926  1.00  0.00           H
ATOM   5729  N   TYR C 584      99.545 114.118 101.455  1.00  0.00           N
ATOM   5730  CA  TYR C 584     100.227 114.286 100.169  1.00  0.00           C
ATOM   5731  C   TYR C 584      99.305 114.061  98.954  1.00  0.00           C
ATOM   5732  O   TYR C 584      99.371 114.855  98.017  1.00  0.00           O
ATOM   5733  CB  TYR C 584     101.508 113.420 100.100  1.00 20.00           C
ATOM   5734  CG  TYR C 584     102.185 113.440  98.736  1.00 20.00           C
ATOM   5735  CD1 TYR C 584     102.774 114.633  98.270  1.00 20.00           C
ATOM   5736  CD2 TYR C 584     102.124 112.314  97.888  1.00 20.00           C
ATOM   5737  CE1 TYR C 584     103.243 114.722  96.946  1.00 20.00           C
ATOM   5738  CE2 TYR C 584     102.609 112.397  96.568  1.00 20.00           C
ATOM   5739  CZ  TYR C 584     103.144 113.609  96.089  1.00 20.00           C
ATOM   5740  OH  TYR C 584     103.544 113.712  94.789  1.00 20.00           O
ATOM   5741  H   TYR C 584      99.874 113.365 102.046  1.00  0.00           H
```

```
ATOM   5742  HA  TYR C 584     100.548 115.329 100.130  1.00  0.00           H
ATOM   5743  HB2 TYR C 584     102.224 113.747 100.856  1.00  0.00           H
ATOM   5744  HB3 TYR C 584     101.263 112.388 100.344  1.00  0.00           H
ATOM   5745  HD1 TYR C 584     102.811 115.500  98.913  1.00  0.00           H
ATOM   5746  HD2 TYR C 584     101.664 111.400  98.232  1.00  0.00           H
ATOM   5747  HE1 TYR C 584     103.650 115.655  96.584  1.00  0.00           H
ATOM   5748  HE2 TYR C 584     102.535 111.541  95.914  1.00  0.00           H
ATOM   5749  HH  TYR C 584     103.756 114.613  94.532  1.00  0.00           H
ATOM   5750  N   LEU C 585      98.461 113.013  98.991  1.00  0.00           N
ATOM   5751  CA  LEU C 585      97.506 112.702  97.921  1.00  0.00           C
ATOM   5752  C   LEU C 585      96.368 113.728  97.799  1.00  0.00           C
ATOM   5753  O   LEU C 585      95.859 113.891  96.693  1.00  0.00           O
ATOM   5754  CB  LEU C 585      96.952 111.269  98.071  1.00 20.00           C
ATOM   5755  CG  LEU C 585      97.967 110.161  97.695  1.00 20.00           C
ATOM   5756  CD1 LEU C 585      97.489 108.776  98.183  1.00 20.00           C
ATOM   5757  CD2 LEU C 585      98.321 110.168  96.191  1.00 20.00           C
ATOM   5758  H   LEU C 585      98.461 112.387  99.786  1.00  0.00           H
ATOM   5759  HA  LEU C 585      98.056 112.748  96.983  1.00  0.00           H
ATOM   5760  HB2 LEU C 585      96.599 111.139  99.094  1.00  0.00           H
ATOM   5761  HB3 LEU C 585      96.068 111.139  97.444  1.00  0.00           H
ATOM   5762  HG  LEU C 585      98.894 110.371  98.228  1.00  0.00           H
ATOM   5763 HD11 LEU C 585      98.251 108.302  98.801  1.00  0.00           H
ATOM   5764 HD12 LEU C 585      96.586 108.845  98.790  1.00  0.00           H
ATOM   5765 HD13 LEU C 585      97.264 108.091  97.365  1.00  0.00           H
ATOM   5766 HD21 LEU C 585      98.203 109.192  95.721  1.00  0.00           H
ATOM   5767 HD22 LEU C 585      97.704 110.867  95.625  1.00  0.00           H
ATOM   5768 HD23 LEU C 585      99.360 110.464  96.047  1.00  0.00           H
ATOM   5769  N   VAL C 586      96.021 114.433  98.892  1.00  0.00           N
ATOM   5770  CA  VAL C 586      95.094 115.568  98.875  1.00  0.00           C
ATOM   5771  C   VAL C 586      95.659 116.777  98.095  1.00  0.00           C
ATOM   5772  O   VAL C 586      94.915 117.370  97.313  1.00  0.00           O
ATOM   5773  CB  VAL C 586      94.656 115.976 100.317  1.00 20.00           C
ATOM   5774  CG1 VAL C 586      94.115 117.412 100.492  1.00 20.00           C
ATOM   5775  CG2 VAL C 586      93.621 114.976 100.865  1.00 20.00           C
ATOM   5776  H   VAL C 586      96.467 114.232  99.777  1.00  0.00           H
ATOM   5777  HA  VAL C 586      94.200 115.242  98.338  1.00  0.00           H
ATOM   5778  HB  VAL C 586      95.527 115.896 100.965  1.00  0.00           H
ATOM   5779 HG11 VAL C 586      93.745 117.569 101.506  1.00  0.00           H
ATOM   5780 HG12 VAL C 586      94.887 118.164 100.325  1.00  0.00           H
ATOM   5781 HG13 VAL C 586      93.291 117.613  99.807  1.00  0.00           H
ATOM   5782 HG21 VAL C 586      93.411 115.161 101.919  1.00  0.00           H
ATOM   5783 HG22 VAL C 586      92.679 115.048 100.321  1.00  0.00           H
ATOM   5784 HG23 VAL C 586      93.967 113.947 100.778  1.00  0.00           H
ATOM   5785  N   PHE C 587      96.960 117.081  98.268  1.00  0.00           N
ATOM   5786  CA  PHE C 587      97.658 118.108  97.487  1.00  0.00           C
ATOM   5787  C   PHE C 587      97.933 117.690  96.033  1.00  0.00           C
ATOM   5788  O   PHE C 587      97.728 118.507  95.139  1.00  0.00           O
ATOM   5789  CB  PHE C 587      98.949 118.577  98.190  1.00 20.00           C
ATOM   5790  CG  PHE C 587      98.713 119.453  99.407  1.00 20.00           C
ATOM   5791  CD1 PHE C 587      98.216 120.765  99.253  1.00 20.00           C
ATOM   5792  CD2 PHE C 587      98.876 118.935 100.708  1.00 20.00           C
ATOM   5793  CE1 PHE C 587      97.925 121.534 100.371  1.00 20.00           C
ATOM   5794  CE2 PHE C 587      98.589 119.722 101.814  1.00 20.00           C
ATOM   5795  CZ  PHE C 587      98.116 121.018 101.647  1.00 20.00           C
ATOM   5796  H   PHE C 587      97.517 116.556  98.928  1.00  0.00           H
ATOM   5797  HA  PHE C 587      96.996 118.974  97.423  1.00  0.00           H
ATOM   5798  HB2 PHE C 587      99.559 117.718  98.474  1.00  0.00           H
ATOM   5799  HB3 PHE C 587      99.558 119.156  97.492  1.00  0.00           H
ATOM   5800  HD1 PHE C 587      98.056 121.169  98.265  1.00  0.00           H
ATOM   5801  HD2 PHE C 587      99.247 117.933 100.845  1.00  0.00           H
ATOM   5802  HE1 PHE C 587      97.544 122.537 100.250  1.00  0.00           H
ATOM   5803  HE2 PHE C 587      98.732 119.326 102.808  1.00  0.00           H
ATOM   5804  HZ  PHE C 587      97.890 121.624 102.511  1.00  0.00           H
ATOM   5805  N   LEU C 588      98.366 116.437  95.810  1.00  0.00           N
ATOM   5806  CA  LEU C 588      98.648 115.888  94.481  1.00  0.00           C
ATOM   5807  C   LEU C 588      97.395 115.836  93.595  1.00  0.00           C
ATOM   5808  O   LEU C 588      97.419 116.426  92.519  1.00  0.00           O
ATOM   5809  CB  LEU C 588      99.370 114.525  94.606  1.00 20.00           C
ATOM   5810  CG  LEU C 588      99.608 113.735  93.291  1.00 20.00           C
ATOM   5811  CD1 LEU C 588     100.399 114.536  92.234  1.00 20.00           C
ATOM   5812  CD2 LEU C 588     100.243 112.360  93.584  1.00 20.00           C
```

```
ATOM   5813  H   LEU C 588      98.535 115.819  96.595  1.00  0.00           H
ATOM   5814  HA  LEU C 588      99.340 116.579  93.998  1.00  0.00           H
ATOM   5815  HB2 LEU C 588     100.326 114.679  95.109  1.00  0.00           H
ATOM   5816  HB3 LEU C 588      98.785 113.893  95.271  1.00  0.00           H
ATOM   5817  HG  LEU C 588      98.632 113.517  92.855  1.00  0.00           H
ATOM   5818 HD11 LEU C 588     101.247 113.986  91.826  1.00  0.00           H
ATOM   5819 HD12 LEU C 588      99.757 114.794  91.391  1.00  0.00           H
ATOM   5820 HD13 LEU C 588     100.794 115.468  92.633  1.00  0.00           H
ATOM   5821 HD21 LEU C 588     101.229 112.247  93.136  1.00  0.00           H
ATOM   5822 HD22 LEU C 588     100.362 112.187  94.653  1.00  0.00           H
ATOM   5823 HD23 LEU C 588      99.618 111.555  93.196  1.00  0.00           H
ATOM   5824  N   PHE C 589      96.329 115.168  94.068  1.00  0.00           N
ATOM   5825  CA  PHE C 589      95.068 115.012  93.337  1.00  0.00           C
ATOM   5826  C   PHE C 589      94.286 116.331  93.183  1.00  0.00           C
ATOM   5827  O   PHE C 589      93.636 116.514  92.154  1.00  0.00           O
ATOM   5828  CB  PHE C 589      94.222 113.906  94.005  1.00 20.00           C
ATOM   5829  CG  PHE C 589      92.956 113.508  93.267  1.00 20.00           C
ATOM   5830  CD1 PHE C 589      93.037 112.661  92.142  1.00 20.00           C
ATOM   5831  CD2 PHE C 589      91.715 114.094  93.597  1.00 20.00           C
ATOM   5832  CE1 PHE C 589      91.892 112.360  91.416  1.00 20.00           C
ATOM   5833  CE2 PHE C 589      90.582 113.784  92.855  1.00 20.00           C
ATOM   5834  CZ  PHE C 589      90.670 112.917  91.773  1.00 20.00           C
ATOM   5835  H   PHE C 589      96.374 114.726  94.977  1.00  0.00           H
ATOM   5836  HA  PHE C 589      95.323 114.664  92.334  1.00  0.00           H
ATOM   5837  HB2 PHE C 589      94.832 113.007  94.107  1.00  0.00           H
ATOM   5838  HB3 PHE C 589      93.949 114.204  95.018  1.00  0.00           H
ATOM   5839  HD1 PHE C 589      93.986 112.238  91.846  1.00  0.00           H
ATOM   5840  HD2 PHE C 589      91.641 114.784  94.424  1.00  0.00           H
ATOM   5841  HE1 PHE C 589      91.954 111.695  90.569  1.00  0.00           H
ATOM   5842  HE2 PHE C 589      89.632 114.226  93.114  1.00  0.00           H
ATOM   5843  HZ  PHE C 589      89.786 112.682  91.199  1.00  0.00           H
ATOM   5844  N   GLY C 590      94.382 117.225  94.185  1.00  0.00           N
ATOM   5845  CA  GLY C 590      93.711 118.525  94.207  1.00  0.00           C
ATOM   5846  C   GLY C 590      94.284 119.461  93.134  1.00  0.00           C
ATOM   5847  O   GLY C 590      93.526 119.969  92.307  1.00  0.00           O
ATOM   5848  H   GLY C 590      94.937 116.993  94.997  1.00  0.00           H
ATOM   5849  HA2 GLY C 590      92.638 118.395  94.061  1.00  0.00           H
ATOM   5850  HA3 GLY C 590      93.850 118.981  95.187  1.00  0.00           H
ATOM   5851  N   PHE C 591      95.617 119.646  93.120  1.00  0.00           N
ATOM   5852  CA  PHE C 591      96.323 120.435  92.107  1.00  0.00           C
ATOM   5853  C   PHE C 591      96.376 119.776  90.713  1.00  0.00           C
ATOM   5854  O   PHE C 591      96.446 120.510  89.727  1.00  0.00           O
ATOM   5855  CB  PHE C 591      97.722 120.849  92.614  1.00 20.00           C
ATOM   5856  CG  PHE C 591      97.736 122.042  93.561  1.00 20.00           C
ATOM   5857  CD1 PHE C 591      97.778 123.348  93.031  1.00 20.00           C
ATOM   5858  CD2 PHE C 591      97.571 121.883  94.955  1.00 20.00           C
ATOM   5859  CE1 PHE C 591      97.750 124.448  93.878  1.00 20.00           C
ATOM   5860  CE2 PHE C 591      97.544 122.996  95.786  1.00 20.00           C
ATOM   5861  CZ  PHE C 591      97.645 124.273  95.251  1.00 20.00           C
ATOM   5862  H   PHE C 591      96.194 119.198  93.820  1.00  0.00           H
ATOM   5863  HA  PHE C 591      95.758 121.358  91.965  1.00  0.00           H
ATOM   5864  HB2 PHE C 591      98.216 120.004  93.093  1.00  0.00           H
ATOM   5865  HB3 PHE C 591      98.361 121.111  91.769  1.00  0.00           H
ATOM   5866  HD1 PHE C 591      97.858 123.496  91.964  1.00  0.00           H
ATOM   5867  HD2 PHE C 591      97.478 120.903  95.391  1.00  0.00           H
ATOM   5868  HE1 PHE C 591      97.809 125.445  93.467  1.00  0.00           H
ATOM   5869  HE2 PHE C 591      97.439 122.871  96.853  1.00  0.00           H
ATOM   5870  HZ  PHE C 591      97.628 125.133  95.904  1.00  0.00           H
ATOM   5871  N   SER C 592      96.307 118.431  90.639  1.00  0.00           N
ATOM   5872  CA  SER C 592      96.223 117.679  89.380  1.00  0.00           C
ATOM   5873  C   SER C 592      94.878 117.885  88.670  1.00  0.00           C
ATOM   5874  O   SER C 592      94.875 118.095  87.461  1.00  0.00           O
ATOM   5875  CB  SER C 592      96.540 116.185  89.598  1.00 20.00           C
ATOM   5876  OG  SER C 592      96.521 115.466  88.382  1.00 20.00           O
ATOM   5877  H   SER C 592      96.291 117.883  91.489  1.00  0.00           H
ATOM   5878  HA  SER C 592      96.994 118.061  88.712  1.00  0.00           H
ATOM   5879  HB2 SER C 592      97.535 116.062  90.023  1.00  0.00           H
ATOM   5880  HB3 SER C 592      95.828 115.731  90.287  1.00  0.00           H
ATOM   5881  HG  SER C 592      95.620 115.436  88.053  1.00  0.00           H
ATOM   5882  N   THR C 593      93.769 117.867  89.427  1.00 30.00           N
ATOM   5883  CA  THR C 593      92.431 118.160  88.907  1.00 30.00           C
```

ATOM	5884	C	THR	C	593	92.280	119.638	88.481	1.00	30.00	C
ATOM	5885	O	THR	C	593	91.621	119.903	87.476	1.00	30.00	O
ATOM	5886	CB	THR	C	593	91.329	117.815	89.945	1.00	20.00	C
ATOM	5887	CG2	THR	C	593	89.882	118.108	89.504	1.00	20.00	C
ATOM	5888	OG1	THR	C	593	91.402	116.436	90.255	1.00	20.00	O
ATOM	5889	H	THR	C	593	93.838	117.690	90.421	1.00	0.00	H
ATOM	5890	HA	THR	C	593	92.272	117.539	88.022	1.00	0.00	H
ATOM	5891	HB	THR	C	593	91.525	118.358	90.872	1.00	0.00	H
ATOM	5892	HG1	THR	C	593	92.200	116.279	90.770	1.00	0.00	H
ATOM	5893	HG21	THR	C	593	89.167	117.733	90.237	1.00	0.00	H
ATOM	5894	HG22	THR	C	593	89.695	119.177	89.398	1.00	0.00	H
ATOM	5895	HG23	THR	C	593	89.657	117.632	88.549	1.00	0.00	H
ATOM	5896	N	ALA	C	594	92.933	120.560	89.212	1.00	30.00	N
ATOM	5897	CA	ALA	C	594	92.937	121.994	88.921	1.00	30.00	C
ATOM	5898	C	ALA	C	594	93.650	122.360	87.607	1.00	30.00	C
ATOM	5899	O	ALA	C	594	93.141	123.213	86.880	1.00	30.00	O
ATOM	5900	CB	ALA	C	594	93.551	122.756	90.104	1.00	30.00	C
ATOM	5901	H	ALA	C	594	93.458	120.270	90.026	1.00	0.00	H
ATOM	5902	HA	ALA	C	594	91.895	122.308	88.831	1.00	0.00	H
ATOM	5903	HB1	ALA	C	594	93.454	123.833	89.965	1.00	0.00	H
ATOM	5904	HB2	ALA	C	594	93.050	122.505	91.040	1.00	0.00	H
ATOM	5905	HB3	ALA	C	594	94.611	122.533	90.223	1.00	0.00	H
ATOM	5906	N	VAL	C	595	94.790	121.708	87.313	1.00	30.00	N
ATOM	5907	CA	VAL	C	595	95.570	121.956	86.095	1.00	30.00	C
ATOM	5908	C	VAL	C	595	95.021	121.204	84.856	1.00	30.00	C
ATOM	5909	O	VAL	C	595	95.243	121.682	83.746	1.00	30.00	O
ATOM	5910	CB	VAL	C	595	97.085	121.660	86.312	1.00	20.00	C
ATOM	5911	CG1	VAL	C	595	97.449	120.168	86.374	1.00	20.00	C
ATOM	5912	CG2	VAL	C	595	97.985	122.372	85.286	1.00	20.00	C
ATOM	5913	H	VAL	C	595	95.164	121.026	87.959	1.00	0.00	H
ATOM	5914	HA	VAL	C	595	95.489	123.021	85.868	1.00	0.00	H
ATOM	5915	HB	VAL	C	595	97.347	122.080	87.285	1.00	0.00	H
ATOM	5916	HG11	VAL	C	595	98.502	120.031	86.620	1.00	0.00	H
ATOM	5917	HG12	VAL	C	595	96.869	119.664	87.141	1.00	0.00	H
ATOM	5918	HG13	VAL	C	595	97.266	119.662	85.427	1.00	0.00	H
ATOM	5919	HG21	VAL	C	595	99.040	122.229	85.523	1.00	0.00	H
ATOM	5920	HG22	VAL	C	595	97.827	122.000	84.274	1.00	0.00	H
ATOM	5921	HG23	VAL	C	595	97.798	123.446	85.280	1.00	0.00	H
ATOM	5922	N	VAL	C	596	94.267	120.100	85.052	1.00	30.00	N
ATOM	5923	CA	VAL	C	596	93.524	119.390	83.995	1.00	30.00	C
ATOM	5924	C	VAL	C	596	92.473	120.280	83.310	1.00	30.00	C
ATOM	5925	O	VAL	C	596	92.417	120.295	82.083	1.00	30.00	O
ATOM	5926	CB	VAL	C	596	92.806	118.108	84.533	1.00	20.00	C
ATOM	5927	CG1	VAL	C	596	91.556	117.608	83.767	1.00	20.00	C
ATOM	5928	CG2	VAL	C	596	93.784	116.940	84.639	1.00	20.00	C
ATOM	5929	H	VAL	C	596	94.138	119.751	85.992	1.00	0.00	H
ATOM	5930	HA	VAL	C	596	94.249	119.092	83.234	1.00	0.00	H
ATOM	5931	HB	VAL	C	596	92.476	118.321	85.549	1.00	0.00	H
ATOM	5932	HG11	VAL	C	596	91.220	116.644	84.149	1.00	0.00	H
ATOM	5933	HG12	VAL	C	596	90.710	118.287	83.869	1.00	0.00	H
ATOM	5934	HG13	VAL	C	596	91.764	117.483	82.704	1.00	0.00	H
ATOM	5935	HG21	VAL	C	596	93.304	116.117	85.163	1.00	0.00	H
ATOM	5936	HG22	VAL	C	596	94.103	116.588	83.657	1.00	0.00	H
ATOM	5937	HG23	VAL	C	596	94.669	117.209	85.202	1.00	0.00	H
ATOM	5938	N	THR	C	597	91.664	120.981	84.121	1.00	30.00	N
ATOM	5939	CA	THR	C	597	90.575	121.842	83.655	1.00	30.00	C
ATOM	5940	C	THR	C	597	91.069	123.167	83.035	1.00	30.00	C
ATOM	5941	O	THR	C	597	90.336	123.745	82.233	1.00	30.00	O
ATOM	5942	CB	THR	C	597	89.586	122.174	84.804	1.00	20.00	C
ATOM	5943	CG2	THR	C	597	88.946	120.925	85.432	1.00	20.00	C
ATOM	5944	OG1	THR	C	597	90.176	122.963	85.823	1.00	20.00	O
ATOM	5945	H	THR	C	597	91.773	120.899	85.122	1.00	0.00	H
ATOM	5946	HA	THR	C	597	90.026	121.303	82.880	1.00	0.00	H
ATOM	5947	HB	THR	C	597	88.773	122.774	84.392	1.00	0.00	H
ATOM	5948	HG1	THR	C	597	90.833	122.438	86.287	1.00	0.00	H
ATOM	5949	HG21	THR	C	597	88.248	121.198	86.225	1.00	0.00	H
ATOM	5950	HG22	THR	C	597	88.389	120.360	84.685	1.00	0.00	H
ATOM	5951	HG23	THR	C	597	89.687	120.254	85.861	1.00	0.00	H
ATOM	5952	N	LEU	C	598	92.295	123.598	83.378	1.00	30.00	N
ATOM	5953	CA	LEU	C	598	92.955	124.758	82.780	1.00	30.00	C
ATOM	5954	C	LEU	C	598	93.585	124.422	81.414	1.00	30.00	C

```
ATOM   5955  O   LEU C 598      93.544 125.265  80.518  1.00 30.00           O
ATOM   5956  CB  LEU C 598      93.984 125.309  83.786  1.00 20.00           C
ATOM   5957  CG  LEU C 598      94.673 126.624  83.364  1.00 20.00           C
ATOM   5958  CD1 LEU C 598      93.684 127.803  83.245  1.00 20.00           C
ATOM   5959  CD2 LEU C 598      95.807 126.940  84.340  1.00 20.00           C
ATOM   5960  H   LEU C 598      92.835 123.072  84.050  1.00  0.00           H
ATOM   5961  HA  LEU C 598      92.199 125.525  82.610  1.00  0.00           H
ATOM   5962  HB2 LEU C 598      93.498 125.459  84.751  1.00  0.00           H
ATOM   5963  HB3 LEU C 598      94.748 124.547  83.956  1.00  0.00           H
ATOM   5964  HG  LEU C 598      95.143 126.492  82.389  1.00  0.00           H
ATOM   5965 HD11 LEU C 598      94.035 128.697  83.762  1.00  0.00           H
ATOM   5966 HD12 LEU C 598      93.533 128.075  82.200  1.00  0.00           H
ATOM   5967 HD13 LEU C 598      92.707 127.563  83.662  1.00  0.00           H
ATOM   5968 HD21 LEU C 598      96.125 127.978  84.261  1.00  0.00           H
ATOM   5969 HD22 LEU C 598      95.516 126.750  85.372  1.00  0.00           H
ATOM   5970 HD23 LEU C 598      96.667 126.307  84.125  1.00  0.00           H
ATOM   5971  N   ILE C 599      94.116 123.194  81.270  1.00 30.00           N
ATOM   5972  CA  ILE C 599      94.546 122.622  79.992  1.00 30.00           C
ATOM   5973  C   ILE C 599      93.317 122.339  79.107  1.00 30.00           C
ATOM   5974  O   ILE C 599      92.354 121.733  79.575  1.00 30.00           O
ATOM   5975  CB  ILE C 599      95.389 121.326  80.197  1.00 20.00           C
ATOM   5976  CG1 ILE C 599      96.764 121.672  80.820  1.00 20.00           C
ATOM   5977  CG2 ILE C 599      95.589 120.484  78.917  1.00 20.00           C
ATOM   5978  CD1 ILE C 599      97.469 120.476  81.480  1.00 20.00           C
ATOM   5979  H   ILE C 599      94.125 122.561  82.058  1.00  0.00           H
ATOM   5980  HA  ILE C 599      95.174 123.355  79.486  1.00  0.00           H
ATOM   5981  HB  ILE C 599      94.857 120.698  80.913  1.00  0.00           H
ATOM   5982 HG12 ILE C 599      97.414 122.101  80.056  1.00  0.00           H
ATOM   5983 HG13 ILE C 599      96.655 122.450  81.575  1.00  0.00           H
ATOM   5984 HG21 ILE C 599      96.277 119.656  79.079  1.00  0.00           H
ATOM   5985 HG22 ILE C 599      94.658 120.049  78.559  1.00  0.00           H
ATOM   5986 HG23 ILE C 599      95.989 121.097  78.113  1.00  0.00           H
ATOM   5987 HD11 ILE C 599      98.239 120.813  82.174  1.00  0.00           H
ATOM   5988 HD12 ILE C 599      96.771 119.853  82.040  1.00  0.00           H
ATOM   5989 HD13 ILE C 599      97.956 119.851  80.735  1.00  0.00           H
ATOM   5990  N   GLU C 600      93.364 122.824  77.860  1.00 30.00           N
ATOM   5991  CA  GLU C 600      92.222 122.816  76.945  1.00 30.00           C
ATOM   5992  C   GLU C 600      92.124 121.521  76.125  1.00 30.00           C
ATOM   5993  O   GLU C 600      91.022 120.999  75.962  1.00 30.00           O
ATOM   5994  CB  GLU C 600      92.312 124.062  76.046  1.00 20.00           C
ATOM   5995  CG  GLU C 600      92.130 125.363  76.857  1.00 20.00           C
ATOM   5996  CD  GLU C 600      92.301 126.649  76.051  1.00 20.00           C
ATOM   5997  OE1 GLU C 600      92.800 126.576  74.906  1.00 20.00           O
ATOM   5998  OE2 GLU C 600      91.948 127.701  76.625  1.00 20.00           O1-
ATOM   5999  H   GLU C 600      94.188 123.315  77.542  1.00  0.00           H
ATOM   6000  HA  GLU C 600      91.298 122.896  77.522  1.00  0.00           H
ATOM   6001  HB2 GLU C 600      93.278 124.072  75.537  1.00  0.00           H
ATOM   6002  HB3 GLU C 600      91.556 124.014  75.261  1.00  0.00           H
ATOM   6003  HG2 GLU C 600      91.136 125.373  77.307  1.00  0.00           H
ATOM   6004  HG3 GLU C 600      92.837 125.404  77.685  1.00  0.00           H
ATOM   6005  N   ASP C 601      93.273 121.024  75.646  1.00 30.00           N
ATOM   6006  CA  ASP C 601      93.395 119.832  74.803  1.00 30.00           C
ATOM   6007  C   ASP C 601      94.867 119.380  74.760  1.00 30.00           C
ATOM   6008  O   ASP C 601      95.719 120.012  75.384  1.00 30.00           O
ATOM   6009  CB  ASP C 601      92.758 119.974  73.385  1.00 20.00           C
ATOM   6010  CG  ASP C 601      93.017 121.290  72.635  1.00  0.00           C
ATOM   6011  OD1 ASP C 601      94.057 121.933  72.901  1.00  0.00           O
ATOM   6012  OD2 ASP C 601      92.204 121.578  71.730  1.00  0.00           O1-
ATOM   6013  H   ASP C 601      94.140 121.512  75.825  1.00  0.00           H
ATOM   6014  HA  ASP C 601      92.861 119.031  75.316  1.00  0.00           H
ATOM   6015  HB2 ASP C 601      93.055 119.153  72.733  1.00  0.00           H
ATOM   6016  HB3 ASP C 601      91.678 119.885  73.509  1.00  0.00           H
ATOM   6017  N   GLY C 602      95.142 118.282  74.039  1.00 30.00           N
ATOM   6018  CA  GLY C 602      96.487 117.737  73.863  1.00 30.00           C
ATOM   6019  C   GLY C 602      96.766 116.614  74.872  1.00 30.00           C
ATOM   6020  O   GLY C 602      95.853 116.069  75.493  1.00 30.00           O
ATOM   6021  H   GLY C 602      94.388 117.800  73.571  1.00  0.00           H
ATOM   6022  HA2 GLY C 602      96.562 117.327  72.855  1.00  0.00           H
ATOM   6023  HA3 GLY C 602      97.248 118.516  73.943  1.00  0.00           H
ATOM   6024  N   LYS C 603      98.056 116.249  74.978  1.00 30.00           N
ATOM   6025  CA  LYS C 603      98.600 115.125  75.751  1.00 30.00           C
```

```
ATOM   6026  C   LYS C 603      98.224 115.121  77.246  1.00 30.00           C
ATOM   6027  O   LYS C 603      97.804 114.086  77.763  1.00 30.00           O
ATOM   6028  CB  LYS C 603     100.135 115.105  75.539  1.00 20.00           C
ATOM   6029  CG  LYS C 603     100.914 114.037  76.332  1.00 20.00           C
ATOM   6030  CD  LYS C 603     102.422 114.082  76.046  1.00 20.00           C
ATOM   6031  CE  LYS C 603     103.209 113.059  76.877  1.00 20.00           C
ATOM   6032  NZ  LYS C 603     104.660 113.202  76.670  1.00 20.00           N1+
ATOM   6033  H   LYS C 603      98.737 116.754  74.430  1.00  0.00           H
ATOM   6034  HA  LYS C 603      98.188 114.215  75.312  1.00  0.00           H
ATOM   6035  HB2 LYS C 603     100.339 114.970  74.476  1.00  0.00           H
ATOM   6036  HB3 LYS C 603     100.541 116.084  75.800  1.00  0.00           H
ATOM   6037  HG2 LYS C 603     100.768 114.179  77.403  1.00  0.00           H
ATOM   6038  HG3 LYS C 603     100.522 113.047  76.095  1.00  0.00           H
ATOM   6039  HD2 LYS C 603     102.595 113.900  74.984  1.00  0.00           H
ATOM   6040  HD3 LYS C 603     102.798 115.086  76.248  1.00  0.00           H
ATOM   6041  HE2 LYS C 603     103.000 113.192  77.939  1.00  0.00           H
ATOM   6042  HE3 LYS C 603     102.908 112.044  76.614  1.00  0.00           H
ATOM   6043  HZ1 LYS C 603     104.950 114.124  76.966  1.00  0.00           H
ATOM   6044  HZ2 LYS C 603     105.149 112.508  77.217  1.00  0.00           H
ATOM   6045  HZ3 LYS C 603     104.879 113.075  75.692  1.00  0.00           H
ATOM   6046  N   TYR C 627      98.404 116.277  77.903  1.00 30.00           N
ATOM   6047  CA  TYR C 627      98.275 116.445  79.352  1.00 30.00           C
ATOM   6048  C   TYR C 627      96.850 116.792  79.825  1.00 30.00           C
ATOM   6049  O   TYR C 627      96.670 117.063  81.012  1.00 30.00           O
ATOM   6050  CB  TYR C 627      99.326 117.475  79.809  1.00 20.00           C
ATOM   6051  CG  TYR C 627     100.753 117.000  79.599  1.00 20.00           C
ATOM   6052  CD1 TYR C 627     101.263 115.967  80.410  1.00 20.00           C
ATOM   6053  CD2 TYR C 627     101.562 117.557  78.586  1.00 20.00           C
ATOM   6054  CE1 TYR C 627     102.571 115.492  80.211  1.00 20.00           C
ATOM   6055  CE2 TYR C 627     102.880 117.095  78.401  1.00 20.00           C
ATOM   6056  CZ  TYR C 627     103.389 116.066  79.220  1.00 20.00           C
ATOM   6057  OH  TYR C 627     104.672 115.629  79.066  1.00 20.00           O
ATOM   6058  H   TYR C 627      98.737 117.083  77.394  1.00  0.00           H
ATOM   6059  HA  TYR C 627      98.515 115.496  79.835  1.00  0.00           H
ATOM   6060  HB2 TYR C 627      99.177 118.424  79.291  1.00  0.00           H
ATOM   6061  HB3 TYR C 627      99.213 117.691  80.872  1.00  0.00           H
ATOM   6062  HD1 TYR C 627     100.650 115.535  81.186  1.00  0.00           H
ATOM   6063  HD2 TYR C 627     101.177 118.342  77.952  1.00  0.00           H
ATOM   6064  HE1 TYR C 627     102.948 114.696  80.836  1.00  0.00           H
ATOM   6065  HE2 TYR C 627     103.503 117.537  77.637  1.00  0.00           H
ATOM   6066  HH  TYR C 627     104.990 115.168  79.851  1.00  0.00           H
ATOM   6067  N   ASN C 628      95.864 116.747  78.912  1.00 30.00           N
ATOM   6068  CA  ASN C 628      94.444 116.968  79.198  1.00 30.00           C
ATOM   6069  C   ASN C 628      93.796 115.816  79.999  1.00 30.00           C
ATOM   6070  O   ASN C 628      92.809 116.065  80.690  1.00 30.00           O
ATOM   6071  CB  ASN C 628      93.716 117.231  77.858  1.00 20.00           C
ATOM   6072  CG  ASN C 628      92.295 117.798  77.972  1.00 20.00           C
ATOM   6073  ND2 ASN C 628      92.088 118.831  78.790  1.00 20.00           N
ATOM   6074  OD1 ASN C 628      91.387 117.313  77.302  1.00 20.00           O
ATOM   6075  H   ASN C 628      96.090 116.512  77.955  1.00  0.00           H
ATOM   6076  HA  ASN C 628      94.356 117.853  79.832  1.00  0.00           H
ATOM   6077  HB2 ASN C 628      94.288 117.958  77.280  1.00  0.00           H
ATOM   6078  HB3 ASN C 628      93.695 116.321  77.256  1.00  0.00           H
ATOM   6079 HD21 ASN C 628      91.167 119.238  78.862  1.00  0.00           H
ATOM   6080 HD22 ASN C 628      92.839 119.234  79.332  1.00  0.00           H
ATOM   6081  N   SER C 629      94.369 114.598  79.935  1.00 30.00           N
ATOM   6082  CA  SER C 629      93.974 113.472  80.786  1.00 30.00           C
ATOM   6083  C   SER C 629      94.549 113.625  82.209  1.00 30.00           C
ATOM   6084  O   SER C 629      95.646 114.163  82.373  1.00 30.00           O
ATOM   6085  CB  SER C 629      94.362 112.136  80.110  1.00 20.00           C
ATOM   6086  OG  SER C 629      95.714 111.764  80.300  1.00 20.00           O
ATOM   6087  H   SER C 629      95.185 114.461  79.357  1.00  0.00           H
ATOM   6088  HA  SER C 629      92.884 113.483  80.856  1.00  0.00           H
ATOM   6089  HB2 SER C 629      94.144 112.164  79.042  1.00  0.00           H
ATOM   6090  HB3 SER C 629      93.751 111.333  80.524  1.00  0.00           H
ATOM   6091  HG  SER C 629      96.269 112.286  79.714  1.00  0.00           H
ATOM   6092  N   LEU C 630      93.790 113.132  83.204  1.00 30.00           N
ATOM   6093  CA  LEU C 630      94.115 113.214  84.630  1.00 30.00           C
ATOM   6094  C   LEU C 630      95.414 112.487  85.014  1.00 30.00           C
ATOM   6095  O   LEU C 630      96.206 113.053  85.764  1.00 30.00           O
ATOM   6096  CB  LEU C 630      92.889 112.743  85.450  1.00 20.00           C
```

```
ATOM   6097  CG  LEU C 630      93.004 112.837  86.991  1.00 20.00           C
ATOM   6098  CD1 LEU C 630      93.193 114.285  87.479  1.00 20.00           C
ATOM   6099  CD2 LEU C 630      91.784 112.181  87.669  1.00 20.00           C
ATOM   6100  H   LEU C 630      92.903 112.704  82.981  1.00  0.00           H
ATOM   6101  HA  LEU C 630      94.294 114.262  84.847  1.00  0.00           H
ATOM   6102  HB2 LEU C 630      92.015 113.314  85.133  1.00  0.00           H
ATOM   6103  HB3 LEU C 630      92.679 111.706  85.181  1.00  0.00           H
ATOM   6104  HG  LEU C 630      93.878 112.270  87.314  1.00  0.00           H
ATOM   6105 HD11 LEU C 630      93.100 114.359  88.563  1.00  0.00           H
ATOM   6106 HD12 LEU C 630      94.179 114.663  87.214  1.00  0.00           H
ATOM   6107 HD13 LEU C 630      92.451 114.953  87.040  1.00  0.00           H
ATOM   6108 HD21 LEU C 630      91.268 112.866  88.344  1.00  0.00           H
ATOM   6109 HD22 LEU C 630      91.046 111.836  86.944  1.00  0.00           H
ATOM   6110 HD23 LEU C 630      92.087 111.313  88.254  1.00  0.00           H
ATOM   6111  N   TYR C 631      95.620 111.278  84.467  1.00 30.00           N
ATOM   6112  CA  TYR C 631      96.806 110.446  84.687  1.00 30.00           C
ATOM   6113  C   TYR C 631      98.117 111.081  84.184  1.00 30.00           C
ATOM   6114  O   TYR C 631      99.121 111.007  84.895  1.00 30.00           O
ATOM   6115  CB  TYR C 631      96.547 109.050  84.078  1.00 20.00           C
ATOM   6116  CG  TYR C 631      97.750 108.123  83.973  1.00 20.00           C
ATOM   6117  CD1 TYR C 631      98.308 107.546  85.133  1.00 20.00           C
ATOM   6118  CD2 TYR C 631      98.319 107.846  82.712  1.00 20.00           C
ATOM   6119  CE1 TYR C 631      99.422 106.688  85.031  1.00 20.00           C
ATOM   6120  CE2 TYR C 631      99.428 106.987  82.609  1.00 20.00           C
ATOM   6121  CZ  TYR C 631      99.977 106.403  83.767  1.00 20.00           C
ATOM   6122  OH  TYR C 631     101.044 105.564  83.656  1.00 20.00           O
ATOM   6123  H   TYR C 631      94.923 110.898  83.843  1.00  0.00           H
ATOM   6124  HA  TYR C 631      96.905 110.319  85.767  1.00  0.00           H
ATOM   6125  HB2 TYR C 631      95.769 108.540  84.647  1.00  0.00           H
ATOM   6126  HB3 TYR C 631      96.141 109.172  83.072  1.00  0.00           H
ATOM   6127  HD1 TYR C 631      97.884 107.760  86.103  1.00  0.00           H
ATOM   6128  HD2 TYR C 631      97.907 108.291  81.817  1.00  0.00           H
ATOM   6129  HE1 TYR C 631      99.843 106.248  85.923  1.00  0.00           H
ATOM   6130  HE2 TYR C 631      99.856 106.773  81.641  1.00  0.00           H
ATOM   6131  HH  TYR C 631     101.365 105.245  84.503  1.00  0.00           H
ATOM   6132  N   SER C 632      98.082 111.687  82.983  1.00 30.00           N
ATOM   6133  CA  SER C 632      99.254 112.257  82.315  1.00 30.00           C
ATOM   6134  C   SER C 632      99.895 113.433  83.069  1.00 30.00           C
ATOM   6135  O   SER C 632     101.122 113.495  83.116  1.00 30.00           O
ATOM   6136  CB  SER C 632      98.898 112.668  80.874  1.00 20.00           C
ATOM   6137  OG  SER C 632      98.828 111.530  80.040  1.00 20.00           O
ATOM   6138  H   SER C 632      97.218 111.712  82.459  1.00  0.00           H
ATOM   6139  HA  SER C 632     100.015 111.474  82.268  1.00  0.00           H
ATOM   6140  HB2 SER C 632      97.960 113.224  80.837  1.00  0.00           H
ATOM   6141  HB3 SER C 632      99.665 113.321  80.455  1.00  0.00           H
ATOM   6142  HG  SER C 632      99.715 111.184  79.917  1.00  0.00           H
ATOM   6143  N   THR C 633      99.071 114.321  83.651  1.00 30.00           N
ATOM   6144  CA  THR C 633      99.543 115.479  84.416  1.00 30.00           C
ATOM   6145  C   THR C 633      99.637 115.209  85.938  1.00 30.00           C
ATOM   6146  O   THR C 633     100.316 115.969  86.628  1.00 30.00           O
ATOM   6147  CB  THR C 633      98.680 116.735  84.127  1.00 20.00           C
ATOM   6148  CG2 THR C 633      97.231 116.655  84.624  1.00 20.00           C
ATOM   6149  OG1 THR C 633      99.254 117.888  84.712  1.00 20.00           O
ATOM   6150  H   THR C 633      98.069 114.208  83.573  1.00  0.00           H
ATOM   6151  HA  THR C 633     100.553 115.724  84.085  1.00  0.00           H
ATOM   6152  HB  THR C 633      98.664 116.899  83.050  1.00  0.00           H
ATOM   6153  HG1 THR C 633      99.291 117.760  85.664  1.00  0.00           H
ATOM   6154 HG21 THR C 633      96.681 117.534  84.287  1.00  0.00           H
ATOM   6155 HG22 THR C 633      96.719 115.778  84.227  1.00  0.00           H
ATOM   6156 HG23 THR C 633      97.164 116.618  85.712  1.00  0.00           H
ATOM   6157  N   CYS C 634      99.036 114.107  86.430  1.00 30.00           N
ATOM   6158  CA  CYS C 634      99.265 113.583  87.783  1.00 30.00           C
ATOM   6159  C   CYS C 634     100.709 113.063  87.937  1.00 30.00           C
ATOM   6160  O   CYS C 634     101.309 113.265  88.992  1.00 30.00           O
ATOM   6161  CB  CYS C 634      98.224 112.513  88.183  1.00 20.00           C
ATOM   6162  SG  CYS C 634      98.387 111.980  89.915  1.00 20.00           S
ATOM   6163  H   CYS C 634      98.465 113.535  85.823  1.00  0.00           H
ATOM   6164  HA  CYS C 634      99.150 114.420  88.475  1.00  0.00           H
ATOM   6165  HB2 CYS C 634      97.216 112.909  88.073  1.00  0.00           H
ATOM   6166  HB3 CYS C 634      98.294 111.640  87.532  1.00  0.00           H
ATOM   6167  HG  CYS C 634      99.578 111.392  89.771  1.00  0.00           H
```

```
ATOM   6168  N   LEU C 635     101.255 112.470  86.858  1.00 30.00           N
ATOM   6169  CA  LEU C 635     102.666 112.107  86.734  1.00 30.00           C
ATOM   6170  C   LEU C 635     103.596 113.326  86.745  1.00 30.00           C
ATOM   6171  O   LEU C 635     104.612 113.270  87.431  1.00 30.00           O
ATOM   6172  CB  LEU C 635     102.903 111.289  85.445  1.00 20.00           C
ATOM   6173  CG  LEU C 635     102.528 109.799  85.538  1.00 20.00           C
ATOM   6174  CD1 LEU C 635     102.473 109.159  84.134  1.00 20.00           C
ATOM   6175  CD2 LEU C 635     103.471 109.037  86.494  1.00 20.00           C
ATOM   6176  H   LEU C 635     100.687 112.324  86.034  1.00  0.00           H
ATOM   6177  HA  LEU C 635     102.917 111.509  87.610  1.00  0.00           H
ATOM   6178  HB2 LEU C 635     102.349 111.757  84.632  1.00  0.00           H
ATOM   6179  HB3 LEU C 635     103.953 111.344  85.149  1.00  0.00           H
ATOM   6180  HG  LEU C 635     101.522 109.743  85.956  1.00  0.00           H
ATOM   6181 HD11 LEU C 635     103.097 108.270  84.046  1.00  0.00           H
ATOM   6182 HD12 LEU C 635     101.453 108.862  83.892  1.00  0.00           H
ATOM   6183 HD13 LEU C 635     102.796 109.848  83.353  1.00  0.00           H
ATOM   6184 HD21 LEU C 635     102.918 108.322  87.104  1.00  0.00           H
ATOM   6185 HD22 LEU C 635     104.245 108.495  85.955  1.00  0.00           H
ATOM   6186 HD23 LEU C 635     104.003 109.695  87.179  1.00  0.00           H
ATOM   6187  N   GLU C 636     103.235 114.399  86.018  1.00 30.00           N
ATOM   6188  CA  GLU C 636     104.012 115.642  85.954  1.00 30.00           C
ATOM   6189  C   GLU C 636     104.079 116.397  87.291  1.00 30.00           C
ATOM   6190  O   GLU C 636     105.104 117.023  87.556  1.00 30.00           O
ATOM   6191  CB  GLU C 636     103.480 116.553  84.829  1.00 20.00           C
ATOM   6192  CG  GLU C 636     103.638 115.969  83.409  1.00 20.00           C
ATOM   6193  CD  GLU C 636     105.085 115.812  82.924  1.00 20.00           C
ATOM   6194  OE1 GLU C 636     105.952 116.576  83.401  1.00 20.00           O
ATOM   6195  OE2 GLU C 636     105.298 114.928  82.064  1.00 20.00           O1-
ATOM   6196  H   GLU C 636     102.387 114.373  85.471  1.00  0.00           H
ATOM   6197  HA  GLU C 636     105.039 115.367  85.716  1.00  0.00           H
ATOM   6198  HB2 GLU C 636     102.426 116.768  85.005  1.00  0.00           H
ATOM   6199  HB3 GLU C 636     103.973 117.527  84.871  1.00  0.00           H
ATOM   6200  HG2 GLU C 636     103.149 114.999  83.351  1.00  0.00           H
ATOM   6201  HG3 GLU C 636     103.118 116.611  82.704  1.00  0.00           H
ATOM   6202  N   LEU C 637     103.031 116.280  88.127  1.00 30.00           N
ATOM   6203  CA  LEU C 637     103.034 116.793  89.496  1.00 30.00           C
ATOM   6204  C   LEU C 637     103.777 115.871  90.481  1.00 30.00           C
ATOM   6205  O   LEU C 637     104.397 116.384  91.412  1.00 30.00           O
ATOM   6206  CB  LEU C 637     101.605 117.141  89.956  1.00 20.00           C
ATOM   6207  CG  LEU C 637     100.995 118.375  89.249  1.00 20.00           C
ATOM   6208  CD1 LEU C 637      99.600 118.676  89.808  1.00 20.00           C
ATOM   6209  CD2 LEU C 637     101.875 119.643  89.296  1.00 20.00           C
ATOM   6210  H   LEU C 637     102.208 115.771  87.833  1.00  0.00           H
ATOM   6211  HA  LEU C 637     103.590 117.721  89.495  1.00  0.00           H
ATOM   6212  HB2 LEU C 637     100.958 116.274  89.814  1.00  0.00           H
ATOM   6213  HB3 LEU C 637     101.612 117.337  91.029  1.00  0.00           H
ATOM   6214  HG  LEU C 637     100.864 118.113  88.198  1.00  0.00           H
ATOM   6215 HD11 LEU C 637      98.953 119.061  89.022  1.00  0.00           H
ATOM   6216 HD12 LEU C 637      99.132 117.788  90.232  1.00  0.00           H
ATOM   6217 HD13 LEU C 637      99.635 119.425  90.600  1.00  0.00           H
ATOM   6218 HD21 LEU C 637     101.286 120.548  89.448  1.00  0.00           H
ATOM   6219 HD22 LEU C 637     102.610 119.608  90.098  1.00  0.00           H
ATOM   6220 HD23 LEU C 637     102.417 119.769  88.358  1.00  0.00           H
ATOM   6221  N   PHE C 638     103.772 114.547  90.246  1.00 30.00           N
ATOM   6222  CA  PHE C 638     104.561 113.581  91.020  1.00 30.00           C
ATOM   6223  C   PHE C 638     106.079 113.681  90.740  1.00 30.00           C
ATOM   6224  O   PHE C 638     106.870 113.346  91.622  1.00 30.00           O
ATOM   6225  CB  PHE C 638     103.997 112.159  90.800  1.00 20.00           C
ATOM   6226  CG  PHE C 638     104.621 111.061  91.650  1.00 20.00           C
ATOM   6227  CD1 PHE C 638     104.198 110.875  92.984  1.00 20.00           C
ATOM   6228  CD2 PHE C 638     105.735 110.333  91.185  1.00 20.00           C
ATOM   6229  CE1 PHE C 638     104.833 109.943  93.794  1.00 20.00           C
ATOM   6230  CE2 PHE C 638     106.359 109.404  92.009  1.00 20.00           C
ATOM   6231  CZ  PHE C 638     105.904 109.205  93.306  1.00 20.00           C
ATOM   6232  H   PHE C 638     103.237 114.176  89.472  1.00  0.00           H
ATOM   6233  HA  PHE C 638     104.421 113.819  92.076  1.00  0.00           H
ATOM   6234  HB2 PHE C 638     102.929 112.167  91.021  1.00  0.00           H
ATOM   6235  HB3 PHE C 638     104.080 111.882  89.748  1.00  0.00           H
ATOM   6236  HD1 PHE C 638     103.380 111.455  93.380  1.00  0.00           H
ATOM   6237  HD2 PHE C 638     106.113 110.506  90.191  1.00  0.00           H
ATOM   6238  HE1 PHE C 638     104.496 109.798  94.809  1.00  0.00           H
```

```
ATOM   6239  HE2 PHE C 638     107.205 108.842  91.644  1.00  0.00           H
ATOM   6240  HZ  PHE C 638     106.392 108.482  93.943  1.00  0.00           H
ATOM   6241  N   LYS C 639     106.465 114.178  89.550  1.00 30.00           N
ATOM   6242  CA  LYS C 639     107.853 114.453  89.172  1.00 30.00           C
ATOM   6243  C   LYS C 639     108.518 115.558  90.014  1.00 30.00           C
ATOM   6244  O   LYS C 639     109.719 115.446  90.244  1.00 30.00           O
ATOM   6245  CB  LYS C 639     107.953 114.779  87.667  1.00 20.00           C
ATOM   6246  CG  LYS C 639     107.888 113.561  86.731  1.00 20.00           C
ATOM   6247  CD  LYS C 639     107.895 113.988  85.252  1.00 20.00           C
ATOM   6248  CE  LYS C 639     107.620 112.846  84.263  1.00 20.00           C
ATOM   6249  NZ  LYS C 639     108.708 111.854  84.256  1.00 20.00           N1+
ATOM   6250  H   LYS C 639     105.762 114.405  88.859  1.00  0.00           H
ATOM   6251  HA  LYS C 639     108.418 113.536  89.352  1.00  0.00           H
ATOM   6252  HB2 LYS C 639     107.197 115.514  87.396  1.00  0.00           H
ATOM   6253  HB3 LYS C 639     108.912 115.255  87.468  1.00  0.00           H
ATOM   6254  HG2 LYS C 639     108.741 112.910  86.930  1.00  0.00           H
ATOM   6255  HG3 LYS C 639     107.005 112.965  86.944  1.00  0.00           H
ATOM   6256  HD2 LYS C 639     107.155 114.771  85.096  1.00  0.00           H
ATOM   6257  HD3 LYS C 639     108.856 114.447  85.015  1.00  0.00           H
ATOM   6258  HE2 LYS C 639     106.678 112.352  84.502  1.00  0.00           H
ATOM   6259  HE3 LYS C 639     107.519 113.250  83.255  1.00  0.00           H
ATOM   6260  HZ1 LYS C 639     108.512 111.128  83.580  1.00  0.00           H
ATOM   6261  HZ2 LYS C 639     109.577 112.301  83.999  1.00  0.00           H
ATOM   6262  HZ3 LYS C 639     108.799 111.447  85.175  1.00  0.00           H
ATOM   6263  N   PHE C 640     107.763 116.572  90.486  1.00 30.00           N
ATOM   6264  CA  PHE C 640     108.282 117.617  91.387  1.00 30.00           C
ATOM   6265  C   PHE C 640     108.778 117.077  92.736  1.00 30.00           C
ATOM   6266  O   PHE C 640     109.794 117.564  93.228  1.00 30.00           O
ATOM   6267  CB  PHE C 640     107.248 118.732  91.635  1.00 20.00           C
ATOM   6268  CG  PHE C 640     107.025 119.690  90.482  1.00 20.00           C
ATOM   6269  CD1 PHE C 640     107.886 120.788  90.279  1.00 20.00           C
ATOM   6270  CD2 PHE C 640     106.042 119.409  89.518  1.00 20.00           C
ATOM   6271  CE1 PHE C 640     107.684 121.631  89.194  1.00 20.00           C
ATOM   6272  CE2 PHE C 640     105.849 120.261  88.441  1.00 20.00           C
ATOM   6273  CZ  PHE C 640     106.657 121.379  88.292  1.00 20.00           C
ATOM   6274  H   PHE C 640     106.779 116.613  90.264  1.00  0.00           H
ATOM   6275  HA  PHE C 640     109.150 118.068  90.902  1.00  0.00           H
ATOM   6276  HB2 PHE C 640     106.293 118.287  91.911  1.00  0.00           H
ATOM   6277  HB3 PHE C 640     107.547 119.335  92.494  1.00  0.00           H
ATOM   6278  HD1 PHE C 640     108.689 120.988  90.973  1.00  0.00           H
ATOM   6279  HD2 PHE C 640     105.444 118.527  89.633  1.00  0.00           H
ATOM   6280  HE1 PHE C 640     108.330 122.484  89.051  1.00  0.00           H
ATOM   6281  HE2 PHE C 640     105.077 120.050  87.716  1.00  0.00           H
ATOM   6282  HZ  PHE C 640     106.498 122.045  87.459  1.00  0.00           H
ATOM   6283  N   THR C 641     108.079 116.063  93.275  1.00 30.00           N
ATOM   6284  CA  THR C 641     108.453 115.344  94.493  1.00 30.00           C
ATOM   6285  C   THR C 641     109.777 114.564  94.341  1.00 30.00           C
ATOM   6286  O   THR C 641     110.533 114.512  95.308  1.00 30.00           O
ATOM   6287  CB  THR C 641     107.329 114.367  94.933  1.00 20.00           C
ATOM   6288  CG2 THR C 641     107.616 113.472  96.153  1.00 20.00           C
ATOM   6289  OG1 THR C 641     106.168 115.121  95.216  1.00 20.00           O
ATOM   6290  H   THR C 641     107.249 115.727  92.806  1.00  0.00           H
ATOM   6291  HA  THR C 641     108.590 116.084  95.283  1.00  0.00           H
ATOM   6292  HB  THR C 641     107.080 113.709  94.102  1.00  0.00           H
ATOM   6293  HG1 THR C 641     105.533 114.540  95.644  1.00  0.00           H
ATOM   6294 HG21 THR C 641     106.738 112.883  96.420  1.00  0.00           H
ATOM   6295 HG22 THR C 641     108.422 112.763  95.958  1.00  0.00           H
ATOM   6296 HG23 THR C 641     107.893 114.063  97.025  1.00  0.00           H
ATOM   6297  N   ILE C 642     110.049 114.033  93.132  1.00 30.00           N
ATOM   6298  CA  ILE C 642     111.308 113.372  92.762  1.00 30.00           C
ATOM   6299  C   ILE C 642     112.459 114.380  92.526  1.00 30.00           C
ATOM   6300  O   ILE C 642     113.604 114.057  92.835  1.00 30.00           O
ATOM   6301  CB  ILE C 642     111.140 112.475  91.491  1.00 20.00           C
ATOM   6302  CG1 ILE C 642     110.141 111.321  91.749  1.00 20.00           C
ATOM   6303  CG2 ILE C 642     112.462 111.899  90.919  1.00 20.00           C
ATOM   6304  CD1 ILE C 642     109.512 110.738  90.472  1.00 20.00           C
ATOM   6305  H   ILE C 642     109.370 114.130  92.390  1.00  0.00           H
ATOM   6306  HA  ILE C 642     111.598 112.719  93.585  1.00  0.00           H
ATOM   6307  HB  ILE C 642     110.706 113.097  90.709  1.00  0.00           H
ATOM   6308 HG12 ILE C 642     110.632 110.529  92.315  1.00  0.00           H
ATOM   6309 HG13 ILE C 642     109.321 111.662  92.381  1.00  0.00           H
```

```
ATOM   6310 HG21 ILE C 642     112.281 111.208  90.096  1.00  0.00           H
ATOM   6311 HG22 ILE C 642     113.115 112.675  90.520  1.00  0.00           H
ATOM   6312 HG23 ILE C 642     113.017 111.353  91.683  1.00  0.00           H
ATOM   6313 HD11 ILE C 642     108.501 111.120  90.334  1.00  0.00           H
ATOM   6314 HD12 ILE C 642     110.075 110.985  89.572  1.00  0.00           H
ATOM   6315 HD13 ILE C 642     109.446 109.651  90.533  1.00  0.00           H
ATOM   6316  N   GLY C 643     112.135 115.575  92.003  1.00 30.00           N
ATOM   6317  CA  GLY C 643     113.102 116.609  91.624  1.00 30.00           C
ATOM   6318  C   GLY C 643     113.278 116.699  90.096  1.00 30.00           C
ATOM   6319  O   GLY C 643     114.195 117.382  89.644  1.00 30.00           O
ATOM   6320  H   GLY C 643     111.163 115.772  91.807  1.00  0.00           H
ATOM   6321  HA2 GLY C 643     112.735 117.569  91.988  1.00  0.00           H
ATOM   6322  HA3 GLY C 643     114.075 116.449  92.091  1.00  0.00           H
ATOM   6323  N   MET C 644     112.413 116.033  89.309  1.00 30.00           N
ATOM   6324  CA  MET C 644     112.386 116.039  87.841  1.00 30.00           C
ATOM   6325  C   MET C 644     111.213 116.855  87.259  1.00 30.00           C
ATOM   6326  O   MET C 644     110.986 116.784  86.050  1.00 30.00           O
ATOM   6327  CB  MET C 644     112.346 114.572  87.343  1.00 20.00           C
ATOM   6328  CG  MET C 644     113.727 113.905  87.311  1.00 20.00           C
ATOM   6329  SD  MET C 644     114.909 114.638  86.141  1.00 20.00           S
ATOM   6330  CE  MET C 644     114.204 114.096  84.563  1.00 20.00           C
ATOM   6331  H   MET C 644     111.672 115.503  89.749  1.00  0.00           H
ATOM   6332  HA  MET C 644     113.281 116.526  87.451  1.00  0.00           H
ATOM   6333  HB2 MET C 644     111.674 113.984  87.970  1.00  0.00           H
ATOM   6334  HB3 MET C 644     111.924 114.499  86.341  1.00  0.00           H
ATOM   6335  HG2 MET C 644     114.165 113.928  88.307  1.00  0.00           H
ATOM   6336  HG3 MET C 644     113.618 112.853  87.053  1.00  0.00           H
ATOM   6337  HE1 MET C 644     114.860 114.389  83.743  1.00  0.00           H
ATOM   6338  HE2 MET C 644     114.094 113.011  84.546  1.00  0.00           H
ATOM   6339  HE3 MET C 644     113.227 114.551  84.399  1.00  0.00           H
ATOM   6340  N   GLY C 645     110.477 117.599  88.102  1.00 30.00           N
ATOM   6341  CA  GLY C 645     109.281 118.338  87.701  1.00 30.00           C
ATOM   6342  C   GLY C 645     109.664 119.586  86.901  1.00 30.00           C
ATOM   6343  O   GLY C 645     110.469 120.398  87.359  1.00 30.00           O
ATOM   6344  H   GLY C 645     110.728 117.626  89.079  1.00  0.00           H
ATOM   6345  HA2 GLY C 645     108.606 117.697  87.131  1.00  0.00           H
ATOM   6346  HA3 GLY C 645     108.744 118.644  88.593  1.00  0.00           H
ATOM   6347  N   ASP C 646     109.041 119.734  85.723  1.00 30.00           N
ATOM   6348  CA  ASP C 646     109.221 120.851  84.799  1.00 30.00           C
ATOM   6349  C   ASP C 646     108.032 121.812  84.973  1.00 30.00           C
ATOM   6350  O   ASP C 646     106.883 121.376  84.914  1.00 30.00           O
ATOM   6351  CB  ASP C 646     109.348 120.316  83.349  1.00 20.00           C
ATOM   6352  CG  ASP C 646     109.583 121.365  82.252  1.00 20.00           C
ATOM   6353  OD1 ASP C 646     109.913 122.525  82.587  1.00 20.00           O
ATOM   6354  OD2 ASP C 646     109.465 120.965  81.074  1.00 20.00           O1-
ATOM   6355  H   ASP C 646     108.374 119.035  85.428  1.00  0.00           H
ATOM   6356  HA  ASP C 646     110.143 121.382  85.043  1.00  0.00           H
ATOM   6357  HB2 ASP C 646     110.182 119.614  83.311  1.00  0.00           H
ATOM   6358  HB3 ASP C 646     108.446 119.759  83.099  1.00  0.00           H
ATOM   6359  N   LEU C 647     108.340 123.104  85.172  1.00 30.00           N
ATOM   6360  CA  LEU C 647     107.367 124.196  85.282  1.00 30.00           C
ATOM   6361  C   LEU C 647     106.700 124.551  83.941  1.00 30.00           C
ATOM   6362  O   LEU C 647     105.570 125.034  83.957  1.00 30.00           O
ATOM   6363  CB  LEU C 647     108.072 125.443  85.863  1.00 20.00           C
ATOM   6364  CG  LEU C 647     108.432 125.326  87.361  1.00 20.00           C
ATOM   6365  CD1 LEU C 647     109.417 126.436  87.786  1.00 20.00           C
ATOM   6366  CD2 LEU C 647     107.173 125.311  88.251  1.00 20.00           C
ATOM   6367  H   LEU C 647     109.313 123.376  85.188  1.00  0.00           H
ATOM   6368  HA  LEU C 647     106.570 123.886  85.959  1.00  0.00           H
ATOM   6369  HB2 LEU C 647     108.975 125.630  85.278  1.00  0.00           H
ATOM   6370  HB3 LEU C 647     107.449 126.329  85.726  1.00  0.00           H
ATOM   6371  HG  LEU C 647     108.953 124.379  87.510  1.00  0.00           H
ATOM   6372 HD11 LEU C 647     110.317 126.007  88.227  1.00  0.00           H
ATOM   6373 HD12 LEU C 647     109.734 127.050  86.943  1.00  0.00           H
ATOM   6374 HD13 LEU C 647     108.988 127.116  88.522  1.00  0.00           H
ATOM   6375 HD21 LEU C 647     107.347 125.766  89.225  1.00  0.00           H
ATOM   6376 HD22 LEU C 647     106.342 125.840  87.785  1.00  0.00           H
ATOM   6377 HD23 LEU C 647     106.842 124.292  88.440  1.00  0.00           H
ATOM   6378  N   GLU C 648     107.396 124.303  82.821  1.00 30.00           N
ATOM   6379  CA  GLU C 648     106.947 124.613  81.464  1.00 30.00           C
ATOM   6380  C   GLU C 648     106.731 123.310  80.671  1.00 30.00           C
```

```
ATOM   6381  O   GLU C 648     107.111 123.234  79.502  1.00 30.00           O
ATOM   6382  CB  GLU C 648     107.983 125.559  80.803  1.00 20.00           C
ATOM   6383  CG  GLU C 648     108.196 126.918  81.509  1.00  0.00           C
ATOM   6384  CD  GLU C 648     107.009 127.875  81.378  1.00  0.00           C
ATOM   6385  OE1 GLU C 648     106.029 127.705  82.136  1.00  0.00           O
ATOM   6386  OE2 GLU C 648     107.109 128.781  80.522  1.00  0.00           O1-
ATOM   6387  H   GLU C 648     108.308 123.867  82.888  1.00  0.00           H
ATOM   6388  HA  GLU C 648     105.979 125.114  81.478  1.00  0.00           H
ATOM   6389  HB2 GLU C 648     108.945 125.047  80.748  1.00  0.00           H
ATOM   6390  HB3 GLU C 648     107.697 125.752  79.768  1.00  0.00           H
ATOM   6391  HG2 GLU C 648     108.427 126.778  82.565  1.00  0.00           H
ATOM   6392  HG3 GLU C 648     109.072 127.404  81.078  1.00  0.00           H
ATOM   6393  N   PHE C 649     106.111 122.304  81.319  1.00 30.00           N
ATOM   6394  CA  PHE C 649     105.848 120.972  80.754  1.00 30.00           C
ATOM   6395  C   PHE C 649     104.779 120.959  79.639  1.00 30.00           C
ATOM   6396  O   PHE C 649     104.656 119.945  78.952  1.00 30.00           O
ATOM   6397  CB  PHE C 649     105.500 119.988  81.898  1.00 20.00           C
ATOM   6398  CG  PHE C 649     104.108 120.065  82.515  1.00 20.00           C
ATOM   6399  CD1 PHE C 649     103.813 121.019  83.514  1.00 20.00           C
ATOM   6400  CD2 PHE C 649     103.063 119.257  82.017  1.00 20.00           C
ATOM   6401  CE1 PHE C 649     102.533 121.092  84.049  1.00 20.00           C
ATOM   6402  CE2 PHE C 649     101.790 119.345  82.566  1.00 20.00           C
ATOM   6403  CZ  PHE C 649     101.529 120.254  83.583  1.00 20.00           C
ATOM   6404  H   PHE C 649     105.835 122.437  82.281  1.00  0.00           H
ATOM   6405  HA  PHE C 649     106.782 120.631  80.304  1.00  0.00           H
ATOM   6406  HB2 PHE C 649     106.232 120.088  82.697  1.00  0.00           H
ATOM   6407  HB3 PHE C 649     105.631 118.970  81.527  1.00  0.00           H
ATOM   6408  HD1 PHE C 649     104.578 121.688  83.876  1.00  0.00           H
ATOM   6409  HD2 PHE C 649     103.254 118.553  81.220  1.00  0.00           H
ATOM   6410  HE1 PHE C 649     102.314 121.812  84.823  1.00  0.00           H
ATOM   6411  HE2 PHE C 649     101.000 118.707  82.199  1.00  0.00           H
ATOM   6412  HZ  PHE C 649     100.536 120.320  84.002  1.00  0.00           H
ATOM   6413  N   THR C 650     104.031 122.063  79.479  1.00 30.00           N
ATOM   6414  CA  THR C 650     102.962 122.212  78.498  1.00 30.00           C
ATOM   6415  C   THR C 650     102.640 123.710  78.291  1.00 30.00           C
ATOM   6416  O   THR C 650     102.981 124.528  79.150  1.00 30.00           O
ATOM   6417  CB  THR C 650     101.701 121.405  78.930  1.00 20.00           C
ATOM   6418  CG2 THR C 650     101.016 121.890  80.219  1.00 20.00           C
ATOM   6419  OG1 THR C 650     100.720 121.421  77.919  1.00 20.00           O
ATOM   6420  H   THR C 650     104.201 122.868  80.065  1.00  0.00           H
ATOM   6421  HA  THR C 650     103.326 121.825  77.544  1.00  0.00           H
ATOM   6422  HB  THR C 650     101.983 120.363  79.076  1.00  0.00           H
ATOM   6423  HG1 THR C 650     101.063 120.957  77.150  1.00  0.00           H
ATOM   6424 HG21 THR C 650     100.223 121.205  80.516  1.00  0.00           H
ATOM   6425 HG22 THR C 650     101.724 121.942  81.045  1.00  0.00           H
ATOM   6426 HG23 THR C 650     100.565 122.873  80.100  1.00  0.00           H
ATOM   6427  N   GLU C 651     102.023 124.041  77.141  1.00 30.00           N
ATOM   6428  CA  GLU C 651     101.676 125.409  76.730  1.00 30.00           C
ATOM   6429  C   GLU C 651     100.229 125.545  76.210  1.00 30.00           C
ATOM   6430  O   GLU C 651      99.791 126.677  76.003  1.00 30.00           O
ATOM   6431  CB  GLU C 651     102.678 125.907  75.663  1.00 20.00           C
ATOM   6432  CG  GLU C 651     104.129 126.043  76.172  1.00  0.00           C
ATOM   6433  CD  GLU C 651     105.084 126.644  75.136  1.00  0.00           C
ATOM   6434  OE1 GLU C 651     104.840 126.445  73.925  1.00  0.00           O
ATOM   6435  OE2 GLU C 651     106.056 127.294  75.578  1.00  0.00           O1-
ATOM   6436  H   GLU C 651     101.801 123.314  76.477  1.00  0.00           H
ATOM   6437  HA  GLU C 651     101.744 126.080  77.585  1.00  0.00           H
ATOM   6438  HB2 GLU C 651     102.657 125.225  74.811  1.00  0.00           H
ATOM   6439  HB3 GLU C 651     102.354 126.875  75.277  1.00  0.00           H
ATOM   6440  HG2 GLU C 651     104.146 126.667  77.067  1.00  0.00           H
ATOM   6441  HG3 GLU C 651     104.526 125.070  76.462  1.00  0.00           H
ATOM   6442  N   ASN C 652      99.517 124.422  75.997  1.00 30.00           N
ATOM   6443  CA  ASN C 652      98.159 124.355  75.428  1.00 30.00           C
ATOM   6444  C   ASN C 652      97.068 124.722  76.456  1.00 30.00           C
ATOM   6445  O   ASN C 652      96.414 123.842  77.020  1.00 30.00           O
ATOM   6446  CB  ASN C 652      97.919 122.993  74.711  1.00 20.00           C
ATOM   6447  CG  ASN C 652      98.561 121.765  75.370  1.00 20.00           C
ATOM   6448  ND2 ASN C 652      98.074 121.392  76.551  1.00 20.00           N
ATOM   6449  OD1 ASN C 652      99.492 121.172  74.830  1.00 20.00           O
ATOM   6450  H   ASN C 652      99.931 123.531  76.227  1.00  0.00           H
ATOM   6451  HA  ASN C 652      98.163 125.108  74.636  1.00  0.00           H
```

```
ATOM   6452  HB2 ASN C 652      98.362 123.062  73.717  1.00  0.00           H
ATOM   6453  HB3 ASN C 652      96.856 122.815  74.534  1.00  0.00           H
ATOM   6454 HD21 ASN C 652      98.474 120.609  77.047  1.00  0.00           H
ATOM   6455 HD22 ASN C 652      97.309 121.908  76.964  1.00  0.00           H
ATOM   6456  N   TYR C 653      96.910 126.036  76.672  1.00 30.00           N
ATOM   6457  CA  TYR C 653      95.971 126.660  77.603  1.00 30.00           C
ATOM   6458  C   TYR C 653      95.895 128.169  77.323  1.00 30.00           C
ATOM   6459  O   TYR C 653      96.841 128.741  76.778  1.00 30.00           O
ATOM   6460  CB  TYR C 653      96.387 126.382  79.077  1.00 20.00           C
ATOM   6461  CG  TYR C 653      97.837 126.682  79.441  1.00 20.00           C
ATOM   6462  CD1 TYR C 653      98.734 125.620  79.680  1.00 20.00           C
ATOM   6463  CD2 TYR C 653      98.308 128.011  79.512  1.00 20.00           C
ATOM   6464  CE1 TYR C 653     100.091 125.889  79.936  1.00 20.00           C
ATOM   6465  CE2 TYR C 653      99.673 128.272  79.723  1.00 20.00           C
ATOM   6466  CZ  TYR C 653     100.571 127.212  79.924  1.00 20.00           C
ATOM   6467  OH  TYR C 653     101.901 127.466  80.098  1.00 20.00           O
ATOM   6468  H   TYR C 653      97.505 126.687  76.178  1.00  0.00           H
ATOM   6469  HA  TYR C 653      94.985 126.233  77.421  1.00  0.00           H
ATOM   6470  HB2 TYR C 653      96.201 125.333  79.302  1.00  0.00           H
ATOM   6471  HB3 TYR C 653      95.739 126.928  79.763  1.00  0.00           H
ATOM   6472  HD1 TYR C 653      98.392 124.596  79.649  1.00  0.00           H
ATOM   6473  HD2 TYR C 653      97.637 128.841  79.361  1.00  0.00           H
ATOM   6474  HE1 TYR C 653     100.767 125.073  80.125  1.00  0.00           H
ATOM   6475  HE2 TYR C 653     100.033 129.287  79.712  1.00  0.00           H
ATOM   6476  HH  TYR C 653     102.424 126.666  80.196  1.00  0.00           H
ATOM   6477  N   ASP C 654      94.817 128.794  77.820  1.00 30.00           N
ATOM   6478  CA  ASP C 654      94.786 130.220  78.159  1.00 30.00           C
ATOM   6479  C   ASP C 654      95.166 130.412  79.638  1.00 30.00           C
ATOM   6480  O   ASP C 654      95.066 129.476  80.433  1.00 30.00           O
ATOM   6481  CB  ASP C 654      93.421 130.895  77.867  1.00 20.00           C
ATOM   6482  CG  ASP C 654      92.973 130.872  76.401  1.00  0.00           C
ATOM   6483  OD1 ASP C 654      93.840 130.680  75.520  1.00  0.00           O
ATOM   6484  OD2 ASP C 654      91.779 131.172  76.185  1.00  0.00           O1-
ATOM   6485  H   ASP C 654      94.052 128.247  78.189  1.00  0.00           H
ATOM   6486  HA  ASP C 654      95.541 130.746  77.570  1.00  0.00           H
ATOM   6487  HB2 ASP C 654      92.655 130.387  78.454  1.00  0.00           H
ATOM   6488  HB3 ASP C 654      93.447 131.938  78.183  1.00  0.00           H
ATOM   6489  N   PHE C 655      95.567 131.650  79.973  1.00 30.00           N
ATOM   6490  CA  PHE C 655      95.883 132.142  81.320  1.00 30.00           C
ATOM   6491  C   PHE C 655      97.142 131.465  81.897  1.00 30.00           C
ATOM   6492  O   PHE C 655      97.039 130.634  82.802  1.00 30.00           O
ATOM   6493  CB  PHE C 655      94.658 132.028  82.266  1.00 20.00           C
ATOM   6494  CG  PHE C 655      93.379 132.653  81.733  1.00 20.00           C
ATOM   6495  CD1 PHE C 655      93.247 134.055  81.658  1.00 20.00           C
ATOM   6496  CD2 PHE C 655      92.378 131.838  81.162  1.00 20.00           C
ATOM   6497  CE1 PHE C 655      92.123 134.614  81.064  1.00 20.00           C
ATOM   6498  CE2 PHE C 655      91.263 132.416  80.571  1.00 20.00           C
ATOM   6499  CZ  PHE C 655      91.135 133.799  80.525  1.00 20.00           C
ATOM   6500  H   PHE C 655      95.605 132.348  79.245  1.00  0.00           H
ATOM   6501  HA  PHE C 655      96.114 133.202  81.208  1.00  0.00           H
ATOM   6502  HB2 PHE C 655      94.899 132.518  83.210  1.00  0.00           H
ATOM   6503  HB3 PHE C 655      94.450 130.987  82.521  1.00  0.00           H
ATOM   6504  HD1 PHE C 655      94.021 134.697  82.054  1.00  0.00           H
ATOM   6505  HD2 PHE C 655      92.480 130.762  81.169  1.00  0.00           H
ATOM   6506  HE1 PHE C 655      92.019 135.688  81.012  1.00  0.00           H
ATOM   6507  HE2 PHE C 655      90.499 131.789  80.135  1.00  0.00           H
ATOM   6508  HZ  PHE C 655      90.268 134.241  80.058  1.00  0.00           H
ATOM   6509  N   LYS C 656      98.316 131.843  81.357  1.00 30.00           N
ATOM   6510  CA  LYS C 656      99.624 131.346  81.803  1.00 30.00           C
ATOM   6511  C   LYS C 656      99.976 131.742  83.250  1.00 30.00           C
ATOM   6512  O   LYS C 656     100.648 130.966  83.930  1.00 30.00           O
ATOM   6513  CB  LYS C 656     100.728 131.778  80.812  1.00 20.00           C
ATOM   6514  CG  LYS C 656     102.091 131.125  81.116  1.00 20.00           C
ATOM   6515  CD  LYS C 656     103.139 131.314  80.013  1.00 20.00           C
ATOM   6516  CE  LYS C 656     104.421 130.536  80.338  1.00 20.00           C
ATOM   6517  NZ  LYS C 656     105.434 130.676  79.280  1.00 20.00           N1+
ATOM   6518  H   LYS C 656      98.326 132.512  80.600  1.00  0.00           H
ATOM   6519  HA  LYS C 656      99.564 130.258  81.780  1.00  0.00           H
ATOM   6520  HB2 LYS C 656     100.424 131.498  79.803  1.00  0.00           H
ATOM   6521  HB3 LYS C 656     100.830 132.865  80.803  1.00  0.00           H
ATOM   6522  HG2 LYS C 656     102.497 131.527  82.044  1.00  0.00           H
```

ATOM	6523	HG3	LYS	C	656	101.945	130.057	81.289	1.00	0.00	H
ATOM	6524	HD2	LYS	C	656	102.733	130.976	79.059	1.00	0.00	H
ATOM	6525	HD3	LYS	C	656	103.364	132.375	79.902	1.00	0.00	H
ATOM	6526	HE2	LYS	C	656	104.850	130.876	81.282	1.00	0.00	H
ATOM	6527	HE3	LYS	C	656	104.198	129.475	80.455	1.00	0.00	H
ATOM	6528	HZ1	LYS	C	656	106.231	130.104	79.547	1.00	0.00	H
ATOM	6529	HZ2	LYS	C	656	105.065	130.338	78.403	1.00	0.00	H
ATOM	6530	HZ3	LYS	C	656	105.719	131.639	79.192	1.00	0.00	H
ATOM	6531	N	ALA	C	657	99.484	132.911	83.699	1.00	30.00	N
ATOM	6532	CA	ALA	C	657	99.603	133.393	85.074	1.00	30.00	C
ATOM	6533	C	ALA	C	657	98.953	132.443	86.091	1.00	30.00	C
ATOM	6534	O	ALA	C	657	99.581	132.145	87.102	1.00	30.00	O
ATOM	6535	CB	ALA	C	657	99.000	134.802	85.176	1.00	30.00	C
ATOM	6536	H	ALA	C	657	98.947	133.490	83.071	1.00	0.00	H
ATOM	6537	HA	ALA	C	657	100.667	133.462	85.310	1.00	0.00	H
ATOM	6538	HB1	ALA	C	657	99.083	135.194	86.191	1.00	0.00	H
ATOM	6539	HB2	ALA	C	657	99.517	135.498	84.515	1.00	0.00	H
ATOM	6540	HB3	ALA	C	657	97.944	134.808	84.903	1.00	0.00	H
ATOM	6541	N	VAL	C	658	97.745	131.944	85.776	1.00	30.00	N
ATOM	6542	CA	VAL	C	658	97.014	130.962	86.579	1.00	30.00	C
ATOM	6543	C	VAL	C	658	97.710	129.584	86.589	1.00	30.00	C
ATOM	6544	O	VAL	C	658	97.762	128.957	87.646	1.00	30.00	O
ATOM	6545	CB	VAL	C	658	95.547	130.798	86.077	1.00	20.00	C
ATOM	6546	CG1	VAL	C	658	94.733	129.705	86.803	1.00	20.00	C
ATOM	6547	CG2	VAL	C	658	94.779	132.131	86.161	1.00	20.00	C
ATOM	6548	H	VAL	C	658	97.308	132.217	84.907	1.00	0.00	H
ATOM	6549	HA	VAL	C	658	96.985	131.328	87.607	1.00	0.00	H
ATOM	6550	HB	VAL	C	658	95.577	130.518	85.024	1.00	0.00	H
ATOM	6551	HG11	VAL	C	658	93.697	129.697	86.463	1.00	0.00	H
ATOM	6552	HG12	VAL	C	658	95.125	128.707	86.621	1.00	0.00	H
ATOM	6553	HG13	VAL	C	658	94.726	129.870	87.881	1.00	0.00	H
ATOM	6554	HG21	VAL	C	658	93.764	132.025	85.777	1.00	0.00	H
ATOM	6555	HG22	VAL	C	658	94.710	132.481	87.192	1.00	0.00	H
ATOM	6556	HG23	VAL	C	658	95.260	132.916	85.578	1.00	0.00	H
ATOM	6557	N	PHE	C	659	98.262	129.163	85.436	1.00	30.00	N
ATOM	6558	CA	PHE	C	659	98.987	127.901	85.256	1.00	30.00	C
ATOM	6559	C	PHE	C	659	100.234	127.770	86.142	1.00	30.00	C
ATOM	6560	O	PHE	C	659	100.371	126.768	86.844	1.00	30.00	O
ATOM	6561	CB	PHE	C	659	99.292	127.683	83.754	1.00	20.00	C
ATOM	6562	CG	PHE	C	659	100.200	126.515	83.403	1.00	20.00	C
ATOM	6563	CD1	PHE	C	659	99.705	125.194	83.407	1.00	20.00	C
ATOM	6564	CD2	PHE	C	659	101.578	126.730	83.182	1.00	20.00	C
ATOM	6565	CE1	PHE	C	659	100.557	124.133	83.125	1.00	20.00	C
ATOM	6566	CE2	PHE	C	659	102.409	125.659	82.884	1.00	20.00	C
ATOM	6567	CZ	PHE	C	659	101.900	124.368	82.853	1.00	20.00	C
ATOM	6568	H	PHE	C	659	98.177	129.743	84.613	1.00	0.00	H
ATOM	6569	HA	PHE	C	659	98.318	127.100	85.573	1.00	0.00	H
ATOM	6570	HB2	PHE	C	659	98.360	127.548	83.206	1.00	0.00	H
ATOM	6571	HB3	PHE	C	659	99.735	128.585	83.337	1.00	0.00	H
ATOM	6572	HD1	PHE	C	659	98.662	125.004	83.612	1.00	0.00	H
ATOM	6573	HD2	PHE	C	659	101.987	127.730	83.215	1.00	0.00	H
ATOM	6574	HE1	PHE	C	659	100.174	123.124	83.109	1.00	0.00	H
ATOM	6575	HE2	PHE	C	659	103.455	125.834	82.681	1.00	0.00	H
ATOM	6576	HZ	PHE	C	659	102.559	123.545	82.622	1.00	0.00	H
ATOM	6577	N	ILE	C	660	101.103	128.793	86.103	1.00	30.00	N
ATOM	6578	CA	ILE	C	660	102.342	128.813	86.875	1.00	30.00	C
ATOM	6579	C	ILE	C	660	102.114	129.158	88.363	1.00	30.00	C
ATOM	6580	O	ILE	C	660	102.919	128.729	89.185	1.00	30.00	O
ATOM	6581	CB	ILE	C	660	103.395	129.765	86.240	1.00	20.00	C
ATOM	6582	CG1	ILE	C	660	104.831	129.447	86.721	1.00	20.00	C
ATOM	6583	CG2	ILE	C	660	103.077	131.267	86.403	1.00	20.00	C
ATOM	6584	CD1	ILE	C	660	105.921	129.889	85.736	1.00	20.00	C
ATOM	6585	H	ILE	C	660	100.928	129.586	85.500	1.00	0.00	H
ATOM	6586	HA	ILE	C	660	102.754	127.802	86.842	1.00	0.00	H
ATOM	6587	HB	ILE	C	660	103.370	129.559	85.169	1.00	0.00	H
ATOM	6588	HG12	ILE	C	660	105.010	129.902	87.696	1.00	0.00	H
ATOM	6589	HG13	ILE	C	660	104.944	128.373	86.871	1.00	0.00	H
ATOM	6590	HG21	ILE	C	660	103.737	131.878	85.787	1.00	0.00	H
ATOM	6591	HG22	ILE	C	660	102.056	131.491	86.098	1.00	0.00	H
ATOM	6592	HG23	ILE	C	660	103.193	131.601	87.434	1.00	0.00	H
ATOM	6593	HD11	ILE	C	660	106.900	129.541	86.066	1.00	0.00	H

```
ATOM   6594 HD12 ILE C 660     105.748 129.478  84.741  1.00  0.00           H
ATOM   6595 HD13 ILE C 660     105.968 130.974  85.651  1.00  0.00           H
ATOM   6596  N   ILE C 661     101.003 129.846  88.700  1.00 30.00           N
ATOM   6597  CA  ILE C 661     100.549 130.040  90.083  1.00 30.00           C
ATOM   6598  C   ILE C 661     100.101 128.723  90.747  1.00 30.00           C
ATOM   6599  O   ILE C 661     100.423 128.534  91.916  1.00 30.00           O
ATOM   6600  CB  ILE C 661      99.442 131.137  90.209  1.00 20.00           C
ATOM   6601  CG1 ILE C 661     100.087 132.541  90.132  1.00 20.00           C
ATOM   6602  CG2 ILE C 661      98.533 131.060  91.461  1.00 20.00           C
ATOM   6603  CD1 ILE C 661      99.090 133.679  89.858  1.00 20.00           C
ATOM   6604  H   ILE C 661     100.387 130.189  87.976  1.00  0.00           H
ATOM   6605  HA  ILE C 661     101.415 130.387  90.650  1.00  0.00           H
ATOM   6606  HB  ILE C 661      98.783 131.025  89.347  1.00  0.00           H
ATOM   6607 HG12 ILE C 661     100.615 132.745  91.064  1.00  0.00           H
ATOM   6608 HG13 ILE C 661     100.854 132.559  89.357  1.00  0.00           H
ATOM   6609 HG21 ILE C 661      97.824 131.887  91.490  1.00  0.00           H
ATOM   6610 HG22 ILE C 661      97.932 130.150  91.479  1.00  0.00           H
ATOM   6611 HG23 ILE C 661      99.118 131.099  92.380  1.00  0.00           H
ATOM   6612 HD11 ILE C 661      99.552 134.461  89.256  1.00  0.00           H
ATOM   6613 HD12 ILE C 661      98.210 133.328  89.318  1.00  0.00           H
ATOM   6614 HD13 ILE C 661      98.751 134.135  90.789  1.00  0.00           H
ATOM   6615  N   LEU C 662      99.427 127.827  90.000  1.00 30.00           N
ATOM   6616  CA  LEU C 662      99.064 126.482  90.466  1.00 30.00           C
ATOM   6617  C   LEU C 662     100.294 125.595  90.722  1.00 30.00           C
ATOM   6618  O   LEU C 662     100.289 124.852  91.700  1.00 30.00           O
ATOM   6619  CB  LEU C 662      98.111 125.794  89.459  1.00 20.00           C
ATOM   6620  CG  LEU C 662      96.662 126.329  89.460  1.00 20.00           C
ATOM   6621  CD1 LEU C 662      95.873 125.777  88.254  1.00 20.00           C
ATOM   6622  CD2 LEU C 662      95.931 126.069  90.792  1.00 20.00           C
ATOM   6623  H   LEU C 662      99.179 128.055  89.047  1.00  0.00           H
ATOM   6624  HA  LEU C 662      98.549 126.591  91.421  1.00  0.00           H
ATOM   6625  HB2 LEU C 662      98.536 125.888  88.460  1.00  0.00           H
ATOM   6626  HB3 LEU C 662      98.071 124.721  89.657  1.00  0.00           H
ATOM   6627  HG  LEU C 662      96.709 127.409  89.334  1.00  0.00           H
ATOM   6628 HD11 LEU C 662      95.154 125.011  88.541  1.00  0.00           H
ATOM   6629 HD12 LEU C 662      95.317 126.575  87.763  1.00  0.00           H
ATOM   6630 HD13 LEU C 662      96.524 125.329  87.502  1.00  0.00           H
ATOM   6631 HD21 LEU C 662      94.902 125.743  90.647  1.00  0.00           H
ATOM   6632 HD22 LEU C 662      96.428 125.305  91.386  1.00  0.00           H
ATOM   6633 HD23 LEU C 662      95.893 126.978  91.394  1.00  0.00           H
ATOM   6634  N   LEU C 663     101.327 125.702  89.867  1.00 30.00           N
ATOM   6635  CA  LEU C 663     102.587 124.968  90.008  1.00 30.00           C
ATOM   6636  C   LEU C 663     103.431 125.467  91.191  1.00 30.00           C
ATOM   6637  O   LEU C 663     103.963 124.637  91.924  1.00 30.00           O
ATOM   6638  CB  LEU C 663     103.396 125.051  88.699  1.00 20.00           C
ATOM   6639  CG  LEU C 663     102.778 124.271  87.521  1.00 20.00           C
ATOM   6640  CD1 LEU C 663     103.368 124.747  86.182  1.00 20.00           C
ATOM   6641  CD2 LEU C 663     102.900 122.742  87.710  1.00 20.00           C
ATOM   6642  H   LEU C 663     101.256 126.326  89.075  1.00  0.00           H
ATOM   6643  HA  LEU C 663     102.345 123.922  90.206  1.00  0.00           H
ATOM   6644  HB2 LEU C 663     103.505 126.102  88.428  1.00  0.00           H
ATOM   6645  HB3 LEU C 663     104.409 124.682  88.862  1.00  0.00           H
ATOM   6646  HG  LEU C 663     101.713 124.502  87.485  1.00  0.00           H
ATOM   6647 HD11 LEU C 663     103.638 123.925  85.519  1.00  0.00           H
ATOM   6648 HD12 LEU C 663     102.642 125.361  85.651  1.00  0.00           H
ATOM   6649 HD13 LEU C 663     104.260 125.358  86.321  1.00  0.00           H
ATOM   6650 HD21 LEU C 663     103.520 122.269  86.948  1.00  0.00           H
ATOM   6651 HD22 LEU C 663     103.342 122.478  88.670  1.00  0.00           H
ATOM   6652 HD23 LEU C 663     101.918 122.271  87.665  1.00  0.00           H
ATOM   6653  N   LEU C 664     103.519 126.796  91.375  1.00 30.00           N
ATOM   6654  CA  LEU C 664     104.240 127.428  92.483  1.00 30.00           C
ATOM   6655  C   LEU C 664     103.529 127.198  93.826  1.00 30.00           C
ATOM   6656  O   LEU C 664     104.202 126.844  94.790  1.00 30.00           O
ATOM   6657  CB  LEU C 664     104.461 128.933  92.196  1.00 20.00           C
ATOM   6658  CG  LEU C 664     105.824 129.281  91.549  1.00 20.00           C
ATOM   6659  CD1 LEU C 664     106.163 128.477  90.274  1.00 20.00           C
ATOM   6660  CD2 LEU C 664     105.950 130.803  91.323  1.00 20.00           C
ATOM   6661  H   LEU C 664     103.070 127.422  90.719  1.00  0.00           H
ATOM   6662  HA  LEU C 664     105.219 126.949  92.563  1.00  0.00           H
ATOM   6663  HB2 LEU C 664     103.640 129.326  91.596  1.00  0.00           H
ATOM   6664  HB3 LEU C 664     104.410 129.489  93.134  1.00  0.00           H
```

```
ATOM   6665  HG  LEU C 664     106.584 129.018  92.287  1.00  0.00           H
ATOM   6666 HD11 LEU C 664     106.573 129.101  89.480  1.00  0.00           H
ATOM   6667 HD12 LEU C 664     106.916 127.721  90.495  1.00  0.00           H
ATOM   6668 HD13 LEU C 664     105.303 127.961  89.854  1.00  0.00           H
ATOM   6669 HD21 LEU C 664     106.884 131.181  91.739  1.00  0.00           H
ATOM   6670 HD22 LEU C 664     105.930 131.071  90.266  1.00  0.00           H
ATOM   6671 HD23 LEU C 664     105.139 131.355  91.800  1.00  0.00           H
ATOM   6672  N   ALA C 665     102.192 127.339  93.861  1.00 30.00           N
ATOM   6673  CA  ALA C 665     101.359 127.050  95.033  1.00 30.00           C
ATOM   6674  C   ALA C 665     101.317 125.560  95.414  1.00 30.00           C
ATOM   6675  O   ALA C 665     101.037 125.265  96.571  1.00 30.00           O
ATOM   6676  CB  ALA C 665      99.937 127.580  94.807  1.00 20.00           C
ATOM   6677  H   ALA C 665     101.696 127.641  93.031  1.00  0.00           H
ATOM   6678  HA  ALA C 665     101.786 127.588  95.882  1.00  0.00           H
ATOM   6679  HB1 ALA C 665      99.304 127.401  95.677  1.00  0.00           H
ATOM   6680  HB2 ALA C 665      99.943 128.655  94.626  1.00  0.00           H
ATOM   6681  HB3 ALA C 665      99.463 127.098  93.951  1.00  0.00           H
ATOM   6682  N   TYR C 666     101.615 124.660  94.463  1.00 30.00           N
ATOM   6683  CA  TYR C 666     101.803 123.235  94.712  1.00 30.00           C
ATOM   6684  C   TYR C 666     103.196 122.951  95.304  1.00 30.00           C
ATOM   6685  O   TYR C 666     103.267 122.353  96.374  1.00 30.00           O
ATOM   6686  CB  TYR C 666     101.522 122.441  93.419  1.00 20.00           C
ATOM   6687  CG  TYR C 666     101.829 120.957  93.490  1.00 20.00           C
ATOM   6688  CD1 TYR C 666     101.033 120.102  94.280  1.00 20.00           C
ATOM   6689  CD2 TYR C 666     102.932 120.435  92.785  1.00 20.00           C
ATOM   6690  CE1 TYR C 666     101.343 118.731  94.369  1.00 20.00           C
ATOM   6691  CE2 TYR C 666     103.237 119.067  92.871  1.00 20.00           C
ATOM   6692  CZ  TYR C 666     102.443 118.211  93.660  1.00 20.00           C
ATOM   6693  OH  TYR C 666     102.756 116.886  93.753  1.00 20.00           O
ATOM   6694  H   TYR C 666     101.798 124.976  93.520  1.00  0.00           H
ATOM   6695  HA  TYR C 666     101.061 122.917  95.448  1.00  0.00           H
ATOM   6696  HB2 TYR C 666     100.477 122.556  93.138  1.00  0.00           H
ATOM   6697  HB3 TYR C 666     102.094 122.866  92.595  1.00  0.00           H
ATOM   6698  HD1 TYR C 666     100.193 120.496  94.832  1.00  0.00           H
ATOM   6699  HD2 TYR C 666     103.552 121.083  92.183  1.00  0.00           H
ATOM   6700  HE1 TYR C 666     100.746 118.085  94.993  1.00  0.00           H
ATOM   6701  HE2 TYR C 666     104.089 118.687  92.332  1.00  0.00           H
ATOM   6702  HH  TYR C 666     103.465 116.641  93.151  1.00  0.00           H
ATOM   6703  N   VAL C 667     104.261 123.391  94.608  1.00 30.00           N
ATOM   6704  CA  VAL C 667     105.672 123.169  94.955  1.00 30.00           C
ATOM   6705  C   VAL C 667     106.090 123.775  96.310  1.00 30.00           C
ATOM   6706  O   VAL C 667     106.808 123.111  97.059  1.00 30.00           O
ATOM   6707  CB  VAL C 667     106.610 123.691  93.823  1.00 20.00           C
ATOM   6708  CG1 VAL C 667     108.097 123.896  94.195  1.00 20.00           C
ATOM   6709  CG2 VAL C 667     106.531 122.763  92.600  1.00 20.00           C
ATOM   6710  H   VAL C 667     104.104 123.882  93.736  1.00  0.00           H
ATOM   6711  HA  VAL C 667     105.808 122.089  95.036  1.00  0.00           H
ATOM   6712  HB  VAL C 667     106.234 124.666  93.507  1.00  0.00           H
ATOM   6713 HG11 VAL C 667     108.683 124.172  93.318  1.00  0.00           H
ATOM   6714 HG12 VAL C 667     108.236 124.697  94.922  1.00  0.00           H
ATOM   6715 HG13 VAL C 667     108.533 122.985  94.604  1.00  0.00           H
ATOM   6716 HG21 VAL C 667     107.082 123.179  91.757  1.00  0.00           H
ATOM   6717 HG22 VAL C 667     106.954 121.785  92.829  1.00  0.00           H
ATOM   6718 HG23 VAL C 667     105.506 122.601  92.269  1.00  0.00           H
ATOM   6719  N   ILE C 668     105.603 124.989  96.618  1.00  0.00           N
ATOM   6720  CA  ILE C 668     105.804 125.654  97.909  1.00  0.00           C
ATOM   6721  C   ILE C 668     105.101 124.922  99.074  1.00  0.00           C
ATOM   6722  O   ILE C 668     105.617 124.945 100.189  1.00  0.00           O
ATOM   6723  CB  ILE C 668     105.372 127.154  97.862  1.00 20.00           C
ATOM   6724  CG1 ILE C 668     106.310 127.986  96.949  1.00 20.00           C
ATOM   6725  CG2 ILE C 668     105.188 127.860  99.227  1.00 20.00           C
ATOM   6726  CD1 ILE C 668     107.767 128.111  97.424  1.00 20.00           C
ATOM   6727  H   ILE C 668     105.027 125.483  95.948  1.00  0.00           H
ATOM   6728  HA  ILE C 668     106.871 125.627  98.114  1.00  0.00           H
ATOM   6729  HB  ILE C 668     104.389 127.183  97.389  1.00  0.00           H
ATOM   6730 HG12 ILE C 668     105.890 128.984  96.811  1.00  0.00           H
ATOM   6731 HG13 ILE C 668     106.334 127.548  95.953  1.00  0.00           H
ATOM   6732 HG21 ILE C 668     104.987 128.923  99.093  1.00  0.00           H
ATOM   6733 HG22 ILE C 668     104.343 127.455  99.784  1.00  0.00           H
ATOM   6734 HG23 ILE C 668     106.072 127.766  99.857  1.00  0.00           H
ATOM   6735 HD11 ILE C 668     108.239 128.990  96.984  1.00  0.00           H
```

```
ATOM   6736 HD12 ILE C 668     107.849 128.207  98.505  1.00  0.00           H
ATOM   6737 HD13 ILE C 668     108.352 127.243  97.120  1.00  0.00           H
ATOM   6738  N   LEU C 669     103.964 124.266  98.786  1.00  0.00           N
ATOM   6739  CA  LEU C 669     103.158 123.543  99.767  1.00  0.00           C
ATOM   6740  C   LEU C 669     103.616 122.093  99.985  1.00  0.00           C
ATOM   6741  O   LEU C 669     103.315 121.585 101.062  1.00  0.00           O
ATOM   6742  CB  LEU C 669     101.667 123.579  99.353  1.00 20.00           C
ATOM   6743  CG  LEU C 669     100.863 124.783  99.902  1.00 20.00           C
ATOM   6744  CD1 LEU C 669     101.504 126.162  99.631  1.00 20.00           C
ATOM   6745  CD2 LEU C 669      99.399 124.726  99.416  1.00 20.00           C
ATOM   6746  H   LEU C 669     103.610 124.274  97.840  1.00  0.00           H
ATOM   6747  HA  LEU C 669     103.250 124.040 100.736  1.00  0.00           H
ATOM   6748  HB2 LEU C 669     101.585 123.512  98.270  1.00  0.00           H
ATOM   6749  HB3 LEU C 669     101.163 122.683  99.721  1.00  0.00           H
ATOM   6750  HG  LEU C 669     100.833 124.666 100.986  1.00  0.00           H
ATOM   6751 HD11 LEU C 669     102.096 126.484 100.488  1.00  0.00           H
ATOM   6752 HD12 LEU C 669     102.162 126.154  98.764  1.00  0.00           H
ATOM   6753 HD13 LEU C 669     100.759 126.938  99.452  1.00  0.00           H
ATOM   6754 HD21 LEU C 669      98.709 124.737 100.259  1.00  0.00           H
ATOM   6755 HD22 LEU C 669      99.139 125.565  98.771  1.00  0.00           H
ATOM   6756 HD23 LEU C 669      99.196 123.822  98.841  1.00  0.00           H
ATOM   6757  N   THR C 670     104.298 121.447  99.016  1.00  0.00           N
ATOM   6758  CA  THR C 670     104.610 120.015  99.102  1.00  0.00           C
ATOM   6759  C   THR C 670     106.117 119.695  99.099  1.00  0.00           C
ATOM   6760  O   THR C 670     106.544 118.883  99.918  1.00  0.00           O
ATOM   6761  CB  THR C 670     103.886 119.173  98.008  1.00 20.00           C
ATOM   6762  CG2 THR C 670     104.469 119.250  96.583  1.00 20.00           C
ATOM   6763  OG1 THR C 670     103.940 117.804  98.356  1.00 20.00           O
ATOM   6764  H   THR C 670     104.495 121.912  98.141  1.00  0.00           H
ATOM   6765  HA  THR C 670     104.241 119.631 100.051  1.00  0.00           H
ATOM   6766  HB  THR C 670     102.837 119.469  97.975  1.00  0.00           H
ATOM   6767  HG1 THR C 670     104.850 117.503  98.285  1.00  0.00           H
ATOM   6768 HG21 THR C 670     103.793 118.776  95.877  1.00  0.00           H
ATOM   6769 HG22 THR C 670     104.623 120.275  96.255  1.00  0.00           H
ATOM   6770 HG23 THR C 670     105.425 118.733  96.494  1.00  0.00           H
ATOM   6771  N   TYR C 671     106.897 120.297  98.185  1.00  0.00           N
ATOM   6772  CA  TYR C 671     108.288 119.901  97.950  1.00  0.00           C
ATOM   6773  C   TYR C 671     109.269 120.520  98.959  1.00  0.00           C
ATOM   6774  O   TYR C 671     110.193 119.832  99.388  1.00  0.00           O
ATOM   6775  CB  TYR C 671     108.681 120.200  96.488  1.00 20.00           C
ATOM   6776  CG  TYR C 671     110.104 119.804  96.129  1.00 20.00           C
ATOM   6777  CD1 TYR C 671     110.479 118.445  96.157  1.00 20.00           C
ATOM   6778  CD2 TYR C 671     111.064 120.786  95.807  1.00 20.00           C
ATOM   6779  CE1 TYR C 671     111.803 118.065  95.870  1.00 20.00           C
ATOM   6780  CE2 TYR C 671     112.391 120.407  95.524  1.00 20.00           C
ATOM   6781  CZ  TYR C 671     112.762 119.048  95.558  1.00 20.00           C
ATOM   6782  OH  TYR C 671     114.050 118.687  95.289  1.00 20.00           O
ATOM   6783  H   TYR C 671     106.511 120.990  97.557  1.00  0.00           H
ATOM   6784  HA  TYR C 671     108.349 118.818  98.076  1.00  0.00           H
ATOM   6785  HB2 TYR C 671     108.008 119.672  95.811  1.00  0.00           H
ATOM   6786  HB3 TYR C 671     108.548 121.261  96.279  1.00  0.00           H
ATOM   6787  HD1 TYR C 671     109.751 117.690  96.406  1.00  0.00           H
ATOM   6788  HD2 TYR C 671     110.791 121.830  95.786  1.00  0.00           H
ATOM   6789  HE1 TYR C 671     112.076 117.020  95.894  1.00  0.00           H
ATOM   6790  HE2 TYR C 671     113.125 121.162  95.282  1.00  0.00           H
ATOM   6791  HH  TYR C 671     114.196 117.740  95.342  1.00  0.00           H
ATOM   6792  N   ILE C 672     109.044 121.792  99.321  1.00  0.00           N
ATOM   6793  CA  ILE C 672     109.874 122.531 100.276  1.00  0.00           C
ATOM   6794  C   ILE C 672     109.189 122.684 101.652  1.00  0.00           C
ATOM   6795  O   ILE C 672     109.778 123.312 102.532  1.00  0.00           O
ATOM   6796  CB  ILE C 672     110.260 123.937  99.724  1.00 20.00           C
ATOM   6797  CG1 ILE C 672     109.076 124.925  99.576  1.00 20.00           C
ATOM   6798  CG2 ILE C 672     111.015 123.798  98.388  1.00 20.00           C
ATOM   6799  CD1 ILE C 672     108.844 125.860 100.774  1.00 20.00           C
ATOM   6800  H   ILE C 672     108.272 122.299  98.911  1.00  0.00           H
ATOM   6801  HA  ILE C 672     110.803 121.990 100.464  1.00  0.00           H
ATOM   6802  HB  ILE C 672     110.982 124.374 100.415  1.00  0.00           H
ATOM   6803 HG12 ILE C 672     108.167 124.362  99.372  1.00  0.00           H
ATOM   6804 HG13 ILE C 672     109.231 125.553  98.698  1.00  0.00           H
ATOM   6805 HG21 ILE C 672     111.428 124.755  98.069  1.00  0.00           H
ATOM   6806 HG22 ILE C 672     111.846 123.097  98.474  1.00  0.00           H
```

```
ATOM   6807 HG23 ILE C 672     110.361 123.442  97.591  1.00  0.00           H
ATOM   6808 HD11 ILE C 672     108.428 126.813 100.446  1.00  0.00           H
ATOM   6809 HD12 ILE C 672     108.136 125.429 101.481  1.00  0.00           H
ATOM   6810 HD13 ILE C 672     109.766 126.076 101.314  1.00  0.00           H
ATOM   6811  N   LEU C 673     107.985 122.104 101.825  1.00 30.00           N
ATOM   6812  CA  LEU C 673     107.216 122.174 103.065  1.00 30.00           C
ATOM   6813  C   LEU C 673     106.903 120.764 103.584  1.00 30.00           C
ATOM   6814  O   LEU C 673     107.497 120.405 104.594  1.00 30.00           O
ATOM   6815  CB  LEU C 673     106.014 123.128 102.895  1.00 20.00           C
ATOM   6816  CG  LEU C 673     105.273 123.530 104.195  1.00 20.00           C
ATOM   6817  CD1 LEU C 673     104.571 124.899 104.025  1.00 20.00           C
ATOM   6818  CD2 LEU C 673     104.300 122.441 104.696  1.00 20.00           C
ATOM   6819  H   LEU C 673     107.569 121.588 101.064  1.00  0.00           H
ATOM   6820  HA  LEU C 673     107.837 122.627 103.840  1.00  0.00           H
ATOM   6821  HB2 LEU C 673     106.430 124.041 102.470  1.00  0.00           H
ATOM   6822  HB3 LEU C 673     105.318 122.754 102.150  1.00  0.00           H
ATOM   6823  HG  LEU C 673     106.024 123.673 104.973  1.00  0.00           H
ATOM   6824 HD11 LEU C 673     103.524 124.885 104.325  1.00  0.00           H
ATOM   6825 HD12 LEU C 673     105.064 125.660 104.630  1.00  0.00           H
ATOM   6826 HD13 LEU C 673     104.590 125.249 102.992  1.00  0.00           H
ATOM   6827 HD21 LEU C 673     104.730 121.896 105.536  1.00  0.00           H
ATOM   6828 HD22 LEU C 673     103.345 122.843 105.036  1.00  0.00           H
ATOM   6829 HD23 LEU C 673     104.073 121.714 103.919  1.00  0.00           H
ATOM   6830  N   LEU C 674     106.024 119.979 102.925  1.00 30.00           N
ATOM   6831  CA  LEU C 674     105.615 118.645 103.412  1.00 30.00           C
ATOM   6832  C   LEU C 674     106.737 117.594 103.441  1.00 30.00           C
ATOM   6833  O   LEU C 674     106.755 116.786 104.367  1.00 30.00           O
ATOM   6834  CB  LEU C 674     104.431 118.079 102.602  1.00 20.00           C
ATOM   6835  CG  LEU C 674     103.089 118.789 102.851  1.00 20.00           C
ATOM   6836  CD1 LEU C 674     102.071 118.426 101.751  1.00 20.00           C
ATOM   6837  CD2 LEU C 674     102.550 118.543 104.276  1.00 20.00           C
ATOM   6838  H   LEU C 674     105.560 120.319 102.095  1.00  0.00           H
ATOM   6839  HA  LEU C 674     105.287 118.775 104.444  1.00  0.00           H
ATOM   6840  HB2 LEU C 674     104.691 118.104 101.546  1.00  0.00           H
ATOM   6841  HB3 LEU C 674     104.290 117.020 102.827  1.00  0.00           H
ATOM   6842  HG  LEU C 674     103.267 119.857 102.773  1.00  0.00           H
ATOM   6843 HD11 LEU C 674     101.230 117.854 102.134  1.00  0.00           H
ATOM   6844 HD12 LEU C 674     101.671 119.328 101.286  1.00  0.00           H
ATOM   6845 HD13 LEU C 674     102.513 117.828 100.954  1.00  0.00           H
ATOM   6846 HD21 LEU C 674     101.492 118.287 104.287  1.00  0.00           H
ATOM   6847 HD22 LEU C 674     103.082 117.739 104.783  1.00  0.00           H
ATOM   6848 HD23 LEU C 674     102.662 119.440 104.886  1.00  0.00           H
ATOM   6849  N   LEU C 675     107.643 117.616 102.450  1.00  0.00           N
ATOM   6850  CA  LEU C 675     108.795 116.711 102.369  1.00  0.00           C
ATOM   6851  C   LEU C 675     109.843 117.016 103.459  1.00  0.00           C
ATOM   6852  O   LEU C 675     110.453 116.080 103.974  1.00  0.00           O
ATOM   6853  CB  LEU C 675     109.413 116.776 100.951  1.00 20.00           C
ATOM   6854  CG  LEU C 675     108.950 115.680  99.959  1.00 20.00           C
ATOM   6855  CD1 LEU C 675     107.436 115.697  99.654  1.00 20.00           C
ATOM   6856  CD2 LEU C 675     109.797 115.709  98.668  1.00 20.00           C
ATOM   6857  H   LEU C 675     107.542 118.285 101.698  1.00  0.00           H
ATOM   6858  HA  LEU C 675     108.442 115.693 102.549  1.00  0.00           H
ATOM   6859  HB2 LEU C 675     109.239 117.759 100.516  1.00  0.00           H
ATOM   6860  HB3 LEU C 675     110.498 116.689 101.033  1.00  0.00           H
ATOM   6861  HG  LEU C 675     109.158 114.726 100.446  1.00  0.00           H
ATOM   6862 HD11 LEU C 675     107.044 114.680  99.610  1.00  0.00           H
ATOM   6863 HD12 LEU C 675     106.856 116.222 100.409  1.00  0.00           H
ATOM   6864 HD13 LEU C 675     107.209 116.172  98.700  1.00  0.00           H
ATOM   6865 HD21 LEU C 675     110.323 114.764  98.528  1.00  0.00           H
ATOM   6866 HD22 LEU C 675     109.196 115.874  97.775  1.00  0.00           H
ATOM   6867 HD23 LEU C 675     110.551 116.497  98.688  1.00  0.00           H
ATOM   6868  N   ASN C 676     109.995 118.304 103.819  1.00  0.00           N
ATOM   6869  CA  ASN C 676     110.856 118.771 104.912  1.00  0.00           C
ATOM   6870  C   ASN C 676     110.197 118.570 106.289  1.00  0.00           C
ATOM   6871  O   ASN C 676     110.912 118.324 107.259  1.00  0.00           O
ATOM   6872  CB  ASN C 676     111.223 120.257 104.682  1.00 20.00           C
ATOM   6873  CG  ASN C 676     112.125 120.513 103.465  1.00 20.00           C
ATOM   6874  ND2 ASN C 676     112.362 121.787 103.152  1.00 20.00           N
ATOM   6875  OD1 ASN C 676     112.617 119.589 102.821  1.00 20.00           O
ATOM   6876  H   ASN C 676     109.452 119.013 103.348  1.00  0.00           H
ATOM   6877  HA  ASN C 676     111.771 118.177 104.884  1.00  0.00           H
```

```
ATOM   6878  HB2 ASN C 676     111.761 120.635 105.553  1.00  0.00           H
ATOM   6879  HB3 ASN C 676     110.318 120.861 104.592  1.00  0.00           H
ATOM   6880 HD21 ASN C 676     112.958 122.011 102.369  1.00  0.00           H
ATOM   6881 HD22 ASN C 676     111.929 122.535 103.676  1.00  0.00           H
ATOM   6882  N   MET C 677     108.855 118.635 106.347  1.00  0.00           N
ATOM   6883  CA  MET C 677     108.045 118.347 107.531  1.00  0.00           C
ATOM   6884  C   MET C 677     108.050 116.851 107.880  1.00  0.00           C
ATOM   6885  O   MET C 677     108.008 116.518 109.061  1.00  0.00           O
ATOM   6886  CB  MET C 677     106.605 118.864 107.311  1.00 20.00           C
ATOM   6887  CG  MET C 677     105.762 118.999 108.588  1.00 20.00           C
ATOM   6888  SD  MET C 677     106.336 120.248 109.774  1.00 20.00           S
ATOM   6889  CE  MET C 677     106.124 121.765 108.800  1.00 20.00           C
ATOM   6890  H   MET C 677     108.340 118.889 105.514  1.00  0.00           H
ATOM   6891  HA  MET C 677     108.492 118.893 108.363  1.00  0.00           H
ATOM   6892  HB2 MET C 677     106.633 119.840 106.835  1.00  0.00           H
ATOM   6893  HB3 MET C 677     106.081 118.215 106.609  1.00  0.00           H
ATOM   6894  HG2 MET C 677     105.713 118.041 109.102  1.00  0.00           H
ATOM   6895  HG3 MET C 677     104.735 119.245 108.316  1.00  0.00           H
ATOM   6896  HE1 MET C 677     106.293 122.638 109.430  1.00  0.00           H
ATOM   6897  HE2 MET C 677     106.834 121.802 107.975  1.00  0.00           H
ATOM   6898  HE3 MET C 677     105.113 121.823 108.397  1.00  0.00           H
ATOM   6899  N   LEU C 678     108.137 115.984 106.854  1.00  0.00           N
ATOM   6900  CA  LEU C 678     108.271 114.538 107.004  1.00  0.00           C
ATOM   6901  C   LEU C 678     109.628 114.145 107.610  1.00  0.00           C
ATOM   6902  O   LEU C 678     109.647 113.267 108.465  1.00  0.00           O
ATOM   6903  CB  LEU C 678     108.009 113.839 105.650  1.00 20.00           C
ATOM   6904  CG  LEU C 678     107.949 112.295 105.727  1.00 20.00           C
ATOM   6905  CD1 LEU C 678     106.821 111.796 106.653  1.00 20.00           C
ATOM   6906  CD2 LEU C 678     107.871 111.658 104.330  1.00 20.00           C
ATOM   6907  H   LEU C 678     108.134 116.335 105.906  1.00  0.00           H
ATOM   6908  HA  LEU C 678     107.497 114.225 107.707  1.00  0.00           H
ATOM   6909  HB2 LEU C 678     107.070 114.203 105.233  1.00  0.00           H
ATOM   6910  HB3 LEU C 678     108.784 114.137 104.942  1.00  0.00           H
ATOM   6911  HG  LEU C 678     108.891 111.947 106.149  1.00  0.00           H
ATOM   6912 HD11 LEU C 678     106.321 110.911 106.260  1.00  0.00           H
ATOM   6913 HD12 LEU C 678     107.222 111.530 107.631  1.00  0.00           H
ATOM   6914 HD13 LEU C 678     106.056 112.556 106.812  1.00  0.00           H
ATOM   6915 HD21 LEU C 678     108.585 110.840 104.260  1.00  0.00           H
ATOM   6916 HD22 LEU C 678     106.891 111.236 104.117  1.00  0.00           H
ATOM   6917 HD23 LEU C 678     108.098 112.367 103.533  1.00  0.00           H
ATOM   6918  N   ILE C 679     110.716 114.824 107.205  1.00  0.00           N
ATOM   6919  CA  ILE C 679     112.056 114.666 107.784  1.00  0.00           C
ATOM   6920  C   ILE C 679     112.103 115.089 109.269  1.00  0.00           C
ATOM   6921  O   ILE C 679     112.731 114.396 110.069  1.00  0.00           O
ATOM   6922  CB  ILE C 679     113.127 115.469 106.978  1.00 20.00           C
ATOM   6923  CG1 ILE C 679     113.293 114.886 105.558  1.00 20.00           C
ATOM   6924  CG2 ILE C 679     114.514 115.579 107.650  1.00 20.00           C
ATOM   6925  CD1 ILE C 679     113.972 115.833 104.557  1.00 20.00           C
ATOM   6926  H   ILE C 679     110.621 115.532 106.490  1.00  0.00           H
ATOM   6927  HA  ILE C 679     112.314 113.605 107.734  1.00  0.00           H
ATOM   6928  HB  ILE C 679     112.749 116.485 106.865  1.00  0.00           H
ATOM   6929 HG12 ILE C 679     113.862 113.958 105.617  1.00  0.00           H
ATOM   6930 HG13 ILE C 679     112.323 114.610 105.149  1.00  0.00           H
ATOM   6931 HG21 ILE C 679     115.221 116.105 107.009  1.00  0.00           H
ATOM   6932 HG22 ILE C 679     114.484 116.136 108.586  1.00  0.00           H
ATOM   6933 HG23 ILE C 679     114.927 114.591 107.859  1.00  0.00           H
ATOM   6934 HD11 ILE C 679     113.616 115.642 103.544  1.00  0.00           H
ATOM   6935 HD12 ILE C 679     113.771 116.880 104.783  1.00  0.00           H
ATOM   6936 HD13 ILE C 679     115.053 115.696 104.556  1.00  0.00           H
ATOM   6937  N   ALA C 680     111.408 116.192 109.604  1.00  0.00           N
ATOM   6938  CA  ALA C 680     111.290 116.728 110.959  1.00  0.00           C
ATOM   6939  C   ALA C 680     110.468 115.830 111.900  1.00  0.00           C
ATOM   6940  O   ALA C 680     110.893 115.616 113.034  1.00  0.00           O
ATOM   6941  CB  ALA C 680     110.697 118.143 110.893  1.00 20.00           C
ATOM   6942  H   ALA C 680     110.925 116.707 108.881  1.00  0.00           H
ATOM   6943  HA  ALA C 680     112.298 116.807 111.372  1.00  0.00           H
ATOM   6944  HB1 ALA C 680     110.642 118.595 111.884  1.00  0.00           H
ATOM   6945  HB2 ALA C 680     111.310 118.796 110.270  1.00  0.00           H
ATOM   6946  HB3 ALA C 680     109.690 118.140 110.475  1.00  0.00           H
ATOM   6947  N   LEU C 681     109.327 115.310 111.412  1.00  0.00           N
ATOM   6948  CA  LEU C 681     108.438 114.418 112.162  1.00  0.00           C
```

```
ATOM   6949  C   LEU C 681     108.977 112.982 112.272  1.00  0.00           C
ATOM   6950  O   LEU C 681     108.725 112.344 113.293  1.00  0.00           O
ATOM   6951  CB  LEU C 681     107.019 114.446 111.554  1.00 20.00           C
ATOM   6952  CG  LEU C 681     106.242 115.756 111.829  1.00 20.00           C
ATOM   6953  CD1 LEU C 681     104.921 115.794 111.038  1.00 20.00           C
ATOM   6954  CD2 LEU C 681     106.004 116.006 113.334  1.00 20.00           C
ATOM   6955  H   LEU C 681     109.031 115.549 110.475  1.00  0.00           H
ATOM   6956  HA  LEU C 681     108.381 114.793 113.183  1.00  0.00           H
ATOM   6957  HB2 LEU C 681     107.092 114.268 110.480  1.00  0.00           H
ATOM   6958  HB3 LEU C 681     106.431 113.615 111.950  1.00  0.00           H
ATOM   6959  HG  LEU C 681     106.849 116.582 111.456  1.00  0.00           H
ATOM   6960 HD11 LEU C 681     104.677 116.812 110.739  1.00  0.00           H
ATOM   6961 HD12 LEU C 681     104.971 115.190 110.132  1.00  0.00           H
ATOM   6962 HD13 LEU C 681     104.084 115.419 111.628  1.00  0.00           H
ATOM   6963 HD21 LEU C 681     106.665 116.790 113.704  1.00  0.00           H
ATOM   6964 HD22 LEU C 681     104.983 116.325 113.545  1.00  0.00           H
ATOM   6965 HD23 LEU C 681     106.189 115.114 113.933  1.00  0.00           H
ATOM   6966  N   MET C 682     109.756 112.516 111.277  1.00  0.00           N
ATOM   6967  CA  MET C 682     110.544 111.284 111.374  1.00  0.00           C
ATOM   6968  C   MET C 682     111.646 111.406 112.435  1.00  0.00           C
ATOM   6969  O   MET C 682     111.812 110.464 113.198  1.00  0.00           O
ATOM   6970  CB  MET C 682     111.143 110.885 110.008  1.00 20.00           C
ATOM   6971  CG  MET C 682     110.128 110.201 109.081  1.00 20.00           C
ATOM   6972  SD  MET C 682     110.726 109.938 107.391  1.00 20.00           S
ATOM   6973  CE  MET C 682     109.373 108.909 106.761  1.00 20.00           C
ATOM   6974  H   MET C 682     109.895 113.071 110.443  1.00  0.00           H
ATOM   6975  HA  MET C 682     109.878 110.479 111.696  1.00  0.00           H
ATOM   6976  HB2 MET C 682     111.587 111.752 109.517  1.00  0.00           H
ATOM   6977  HB3 MET C 682     111.965 110.183 110.160  1.00  0.00           H
ATOM   6978  HG2 MET C 682     109.857 109.233 109.502  1.00  0.00           H
ATOM   6979  HG3 MET C 682     109.205 110.777 109.030  1.00  0.00           H
ATOM   6980  HE1 MET C 682     109.503 108.724 105.696  1.00  0.00           H
ATOM   6981  HE2 MET C 682     109.347 107.951 107.280  1.00  0.00           H
ATOM   6982  HE3 MET C 682     108.414 109.404 106.908  1.00  0.00           H
ATOM   6983  N   GLY C 683     112.334 112.561 112.499  1.00  0.00           N
ATOM   6984  CA  GLY C 683     113.386 112.851 113.477  1.00  0.00           C
ATOM   6985  C   GLY C 683     112.841 112.934 114.912  1.00  0.00           C
ATOM   6986  O   GLY C 683     113.486 112.428 115.829  1.00  0.00           O
ATOM   6987  H   GLY C 683     112.131 113.289 111.826  1.00  0.00           H
ATOM   6988  HA2 GLY C 683     114.170 112.096 113.426  1.00  0.00           H
ATOM   6989  HA3 GLY C 683     113.846 113.805 113.221  1.00  0.00           H
ATOM   6990  N   GLU C 684     111.650 113.531 115.094  1.00  0.00           N
ATOM   6991  CA  GLU C 684     110.952 113.659 116.375  1.00  0.00           C
ATOM   6992  C   GLU C 684     110.499 112.302 116.942  1.00  0.00           C
ATOM   6993  O   GLU C 684     110.796 112.005 118.101  1.00  0.00           O
ATOM   6994  CB  GLU C 684     109.832 114.722 116.226  1.00 20.00           C
ATOM   6995  CG  GLU C 684     108.854 114.913 117.411  1.00  0.00           C
ATOM   6996  CD  GLU C 684     107.660 113.953 117.410  1.00  0.00           C
ATOM   6997  OE1 GLU C 684     106.917 113.968 116.404  1.00  0.00           O
ATOM   6998  OE2 GLU C 684     107.505 113.227 118.416  1.00  0.00           O1-
ATOM   6999  H   GLU C 684     111.182 113.942 114.297  1.00  0.00           H
ATOM   7000  HA  GLU C 684     111.671 114.062 117.091  1.00  0.00           H
ATOM   7001  HB2 GLU C 684     110.312 115.683 116.041  1.00  0.00           H
ATOM   7002  HB3 GLU C 684     109.264 114.519 115.318  1.00  0.00           H
ATOM   7003  HG2 GLU C 684     109.390 114.848 118.359  1.00  0.00           H
ATOM   7004  HG3 GLU C 684     108.441 115.921 117.368  1.00  0.00           H
ATOM   7005  N   THR C 685     109.827 111.493 116.105  1.00  0.00           N
ATOM   7006  CA  THR C 685     109.361 110.154 116.469  1.00  0.00           C
ATOM   7007  C   THR C 685     110.490 109.099 116.511  1.00  0.00           C
ATOM   7008  O   THR C 685     110.299 108.093 117.192  1.00  0.00           O
ATOM   7009  CB  THR C 685     108.238 109.647 115.525  1.00 20.00           C
ATOM   7010  CG2 THR C 685     106.989 110.541 115.534  1.00 20.00           C
ATOM   7011  OG1 THR C 685     108.685 109.492 114.192  1.00 20.00           O
ATOM   7012  H   THR C 685     109.611 111.807 115.168  1.00  0.00           H
ATOM   7013  HA  THR C 685     108.939 110.211 117.475  1.00  0.00           H
ATOM   7014  HB  THR C 685     107.924 108.656 115.858  1.00  0.00           H
ATOM   7015  HG1 THR C 685     108.677 110.352 113.761  1.00  0.00           H
ATOM   7016 HG21 THR C 685     106.218 110.147 114.872  1.00  0.00           H
ATOM   7017 HG22 THR C 685     106.564 110.603 116.536  1.00  0.00           H
ATOM   7018 HG23 THR C 685     107.209 111.558 115.214  1.00  0.00           H
ATOM   7019  N   VAL C 686     111.650 109.349 115.866  1.00  0.00           N
```

```
ATOM   7020  CA  VAL C 686     112.864 108.522 115.970  1.00  0.00           C
ATOM   7021  C   VAL C 686     113.407 108.450 117.409  1.00  0.00           C
ATOM   7022  O   VAL C 686     113.838 107.374 117.812  1.00  0.00           O
ATOM   7023  CB  VAL C 686     114.004 108.969 114.980  1.00 20.00           C
ATOM   7024  CG1 VAL C 686     115.460 108.963 115.500  1.00 20.00           C
ATOM   7025  CG2 VAL C 686     113.964 108.166 113.668  1.00 20.00           C
ATOM   7026  H   VAL C 686     111.729 110.179 115.294  1.00  0.00           H
ATOM   7027  HA  VAL C 686     112.563 107.507 115.703  1.00  0.00           H
ATOM   7028  HB  VAL C 686     113.815 110.007 114.712  1.00  0.00           H
ATOM   7029 HG11 VAL C 686     116.158 109.257 114.715  1.00  0.00           H
ATOM   7030 HG12 VAL C 686     115.595 109.672 116.316  1.00  0.00           H
ATOM   7031 HG13 VAL C 686     115.764 107.975 115.848  1.00  0.00           H
ATOM   7032 HG21 VAL C 686     114.764 108.464 112.992  1.00  0.00           H
ATOM   7033 HG22 VAL C 686     114.070 107.096 113.852  1.00  0.00           H
ATOM   7034 HG23 VAL C 686     113.033 108.318 113.132  1.00  0.00           H
ATOM   7035  N   ASN C 687     113.361 109.573 118.150  1.00  0.00           N
ATOM   7036  CA  ASN C 687     113.839 109.663 119.536  1.00  0.00           C
ATOM   7037  C   ASN C 687     112.929 108.919 120.530  1.00  0.00           C
ATOM   7038  O   ASN C 687     113.444 108.363 121.499  1.00  0.00           O
ATOM   7039  CB  ASN C 687     114.001 111.141 119.961  1.00 20.00           C
ATOM   7040  CG  ASN C 687     114.977 111.950 119.099  1.00 20.00           C
ATOM   7041  ND2 ASN C 687     116.213 111.471 118.938  1.00 20.00           N
ATOM   7042  OD1 ASN C 687     114.621 113.013 118.596  1.00 20.00           O
ATOM   7043  H   ASN C 687     112.990 110.422 117.746  1.00  0.00           H
ATOM   7044  HA  ASN C 687     114.818 109.182 119.580  1.00  0.00           H
ATOM   7045  HB2 ASN C 687     113.029 111.639 119.947  1.00  0.00           H
ATOM   7046  HB3 ASN C 687     114.355 111.195 120.992  1.00  0.00           H
ATOM   7047 HD21 ASN C 687     116.881 111.985 118.382  1.00  0.00           H
ATOM   7048 HD22 ASN C 687     116.490 110.600 119.366  1.00  0.00           H
ATOM   7049  N   LYS C 688     111.611 108.893 120.260  1.00  0.00           N
ATOM   7050  CA  LYS C 688     110.625 108.118 121.016  1.00  0.00           C
ATOM   7051  C   LYS C 688     110.767 106.607 120.758  1.00  0.00           C
ATOM   7052  O   LYS C 688     110.755 105.823 121.707  1.00  0.00           O
ATOM   7053  CB  LYS C 688     109.211 108.644 120.678  1.00 20.00           C
ATOM   7054  CG  LYS C 688     108.059 107.895 121.375  1.00 20.00           C
ATOM   7055  CD  LYS C 688     106.687 108.520 121.084  1.00 20.00           C
ATOM   7056  CE  LYS C 688     105.544 107.754 121.764  1.00 20.00           C
ATOM   7057  NZ  LYS C 688     104.241 108.382 121.485  1.00 20.00           N1+
ATOM   7058  H   LYS C 688     111.266 109.382 119.446  1.00  0.00           H
ATOM   7059  HA  LYS C 688     110.802 108.294 122.079  1.00  0.00           H
ATOM   7060  HB2 LYS C 688     109.159 109.700 120.944  1.00  0.00           H
ATOM   7061  HB3 LYS C 688     109.052 108.600 119.599  1.00  0.00           H
ATOM   7062  HG2 LYS C 688     108.039 106.853 121.055  1.00  0.00           H
ATOM   7063  HG3 LYS C 688     108.234 107.883 122.452  1.00  0.00           H
ATOM   7064  HD2 LYS C 688     106.685 109.558 121.419  1.00  0.00           H
ATOM   7065  HD3 LYS C 688     106.523 108.545 120.006  1.00  0.00           H
ATOM   7066  HE2 LYS C 688     105.519 106.721 121.416  1.00  0.00           H
ATOM   7067  HE3 LYS C 688     105.695 107.728 122.844  1.00  0.00           H
ATOM   7068  HZ1 LYS C 688     104.076 108.388 120.488  1.00  0.00           H
ATOM   7069  HZ2 LYS C 688     103.509 107.859 121.944  1.00  0.00           H
ATOM   7070  HZ3 LYS C 688     104.244 109.331 121.830  1.00  0.00           H
ATOM   7071  N   ILE C 689     110.906 106.242 119.474  1.00  0.00           N
ATOM   7072  CA  ILE C 689     111.013 104.867 118.989  1.00  0.00           C
ATOM   7073  C   ILE C 689     112.415 104.243 119.207  1.00  0.00           C
ATOM   7074  O   ILE C 689     112.530 103.022 119.127  1.00  0.00           O
ATOM   7075  CB  ILE C 689     110.541 104.787 117.496  1.00 20.00           C
ATOM   7076  CG1 ILE C 689     109.006 105.007 117.427  1.00 20.00           C
ATOM   7077  CG2 ILE C 689     110.913 103.508 116.714  1.00 20.00           C
ATOM   7078  CD1 ILE C 689     108.465 105.391 116.042  1.00 20.00           C
ATOM   7079  H   ILE C 689     110.896 106.957 118.757  1.00  0.00           H
ATOM   7080  HA  ILE C 689     110.318 104.268 119.577  1.00  0.00           H
ATOM   7081  HB  ILE C 689     111.018 105.612 116.965  1.00  0.00           H
ATOM   7082 HG12 ILE C 689     108.494 104.111 117.778  1.00  0.00           H
ATOM   7083 HG13 ILE C 689     108.708 105.797 118.118  1.00  0.00           H
ATOM   7084 HG21 ILE C 689     110.534 103.534 115.693  1.00  0.00           H
ATOM   7085 HG22 ILE C 689     111.992 103.385 116.626  1.00  0.00           H
ATOM   7086 HG23 ILE C 689     110.501 102.621 117.194  1.00  0.00           H
ATOM   7087 HD11 ILE C 689     107.410 105.661 116.105  1.00  0.00           H
ATOM   7088 HD12 ILE C 689     108.993 106.253 115.637  1.00  0.00           H
ATOM   7089 HD13 ILE C 689     108.546 104.572 115.327  1.00  0.00           H
ATOM   7090  N   ALA C 690     113.438 105.061 119.525  1.00 30.00           N
```

```
ATOM   7091  CA  ALA C 690     114.844 104.677 119.718  1.00 30.00           C
ATOM   7092  C   ALA C 690     115.072 103.498 120.678  1.00 30.00           C
ATOM   7093  O   ALA C 690     115.777 102.557 120.311  1.00 30.00           O
ATOM   7094  CB  ALA C 690     115.662 105.897 120.176  1.00 20.00           C
ATOM   7095  H   ALA C 690     113.257 106.054 119.571  1.00  0.00           H
ATOM   7096  HA  ALA C 690     115.225 104.375 118.743  1.00  0.00           H
ATOM   7097  HB1 ALA C 690     116.699 105.625 120.379  1.00  0.00           H
ATOM   7098  HB2 ALA C 690     115.686 106.674 119.414  1.00  0.00           H
ATOM   7099  HB3 ALA C 690     115.252 106.335 121.087  1.00  0.00           H
ATOM   7100  N   GLN C 691     114.443 103.558 121.863  1.00 30.00           N
ATOM   7101  CA  GLN C 691     114.486 102.493 122.862  1.00 30.00           C
ATOM   7102  C   GLN C 691     113.385 101.441 122.632  1.00 30.00           C
ATOM   7103  O   GLN C 691     113.636 100.264 122.877  1.00 30.00           O
ATOM   7104  CB  GLN C 691     114.421 103.124 124.274  1.00 20.00           C
ATOM   7105  CG  GLN C 691     114.476 102.141 125.465  1.00 20.00           C
ATOM   7106  CD  GLN C 691     115.718 101.242 125.463  1.00 20.00           C
ATOM   7107  NE2 GLN C 691     115.513  99.925 125.504  1.00 20.00           N
ATOM   7108  OE1 GLN C 691     116.846 101.730 125.436  1.00 20.00           O
ATOM   7109  H   GLN C 691     113.877 104.365 122.086  1.00  0.00           H
ATOM   7110  HA  GLN C 691     115.445 101.978 122.773  1.00  0.00           H
ATOM   7111  HB2 GLN C 691     115.244 103.832 124.376  1.00  0.00           H
ATOM   7112  HB3 GLN C 691     113.512 103.721 124.361  1.00  0.00           H
ATOM   7113  HG2 GLN C 691     113.575 101.526 125.486  1.00  0.00           H
ATOM   7114  HG3 GLN C 691     114.473 102.705 126.398  1.00  0.00           H
ATOM   7115 HE21 GLN C 691     116.295  99.288 125.520  1.00  0.00           H
ATOM   7116 HE22 GLN C 691     114.569  99.559 125.495  1.00  0.00           H
ATOM   7117  N   GLU C 692     112.200 101.865 122.155  1.00 30.00           N
ATOM   7118  CA  GLU C 692     111.039 100.996 121.936  1.00 30.00           C
ATOM   7119  C   GLU C 692     111.281  99.915 120.863  1.00 30.00           C
ATOM   7120  O   GLU C 692     110.906  98.765 121.084  1.00 30.00           O
ATOM   7121  CB  GLU C 692     109.808 101.873 121.630  1.00 20.00           C
ATOM   7122  CG  GLU C 692     108.468 101.109 121.565  1.00  0.00           C
ATOM   7123  CD  GLU C 692     107.254 101.994 121.258  1.00  0.00           C
ATOM   7124  OE1 GLU C 692     107.435 103.221 121.084  1.00  0.00           O
ATOM   7125  OE2 GLU C 692     106.148 101.415 121.192  1.00  0.00           O1-
ATOM   7126  H   GLU C 692     112.065 102.846 121.958  1.00  0.00           H
ATOM   7127  HA  GLU C 692     110.850 100.480 122.880  1.00  0.00           H
ATOM   7128  HB2 GLU C 692     109.735 102.661 122.382  1.00  0.00           H
ATOM   7129  HB3 GLU C 692     109.970 102.380 120.679  1.00  0.00           H
ATOM   7130  HG2 GLU C 692     108.507 100.335 120.799  1.00  0.00           H
ATOM   7131  HG3 GLU C 692     108.288 100.601 122.513  1.00  0.00           H
ATOM   7132  N   SER C 693     111.951 100.288 119.757  1.00 30.00           N
ATOM   7133  CA  SER C 693     112.386  99.398 118.673  1.00 30.00           C
ATOM   7134  C   SER C 693     113.410  98.330 119.103  1.00 30.00           C
ATOM   7135  O   SER C 693     113.416  97.248 118.517  1.00 30.00           O
ATOM   7136  CB  SER C 693     112.908 100.235 117.486  1.00 20.00           C
ATOM   7137  OG  SER C 693     114.093 100.937 117.802  1.00 20.00           O
ATOM   7138  H   SER C 693     112.217 101.260 119.656  1.00  0.00           H
ATOM   7139  HA  SER C 693     111.497  98.870 118.329  1.00  0.00           H
ATOM   7140  HB2 SER C 693     112.150 100.937 117.146  1.00  0.00           H
ATOM   7141  HB3 SER C 693     113.132  99.588 116.638  1.00  0.00           H
ATOM   7142  HG  SER C 693     113.864 101.690 118.355  1.00  0.00           H
ATOM   7143  N   LYS C 694     114.229  98.640 120.125  1.00 30.00           N
ATOM   7144  CA  LYS C 694     115.166  97.705 120.744  1.00 30.00           C
ATOM   7145  C   LYS C 694     114.444  96.622 121.564  1.00 30.00           C
ATOM   7146  O   LYS C 694     114.843  95.462 121.485  1.00 30.00           O
ATOM   7147  CB  LYS C 694     116.221  98.493 121.550  1.00 20.00           C
ATOM   7148  CG  LYS C 694     117.277  97.638 122.273  1.00 20.00           C
ATOM   7149  CD  LYS C 694     118.365  98.508 122.924  1.00 20.00           C
ATOM   7150  CE  LYS C 694     119.499  97.701 123.571  1.00 20.00           C
ATOM   7151  NZ  LYS C 694     120.333  97.025 122.562  1.00 20.00           N1+
ATOM   7152  H   LYS C 694     114.162  99.553 120.553  1.00  0.00           H
ATOM   7153  HA  LYS C 694     115.695  97.195 119.939  1.00  0.00           H
ATOM   7154  HB2 LYS C 694     116.728  99.177 120.868  1.00  0.00           H
ATOM   7155  HB3 LYS C 694     115.736  99.122 122.292  1.00  0.00           H
ATOM   7156  HG2 LYS C 694     116.800  97.036 123.048  1.00  0.00           H
ATOM   7157  HG3 LYS C 694     117.720  96.933 121.571  1.00  0.00           H
ATOM   7158  HD2 LYS C 694     118.782  99.192 122.183  1.00  0.00           H
ATOM   7159  HD3 LYS C 694     117.905  99.141 123.684  1.00  0.00           H
ATOM   7160  HE2 LYS C 694     119.098  96.964 124.268  1.00  0.00           H
ATOM   7161  HE3 LYS C 694     120.140  98.369 124.146  1.00  0.00           H
```

```
ATOM   7162  HZ1 LYS C 694     119.777  96.363 122.039  1.00  0.00           H
ATOM   7163  HZ2 LYS C 694     120.719  97.710 121.929  1.00  0.00           H
ATOM   7164  HZ3 LYS C 694     121.087  96.531 123.019  1.00  0.00           H
ATOM   7165  N   ASN C 695     113.379  97.006 122.291  1.00 30.00           N
ATOM   7166  CA  ASN C 695     112.525  96.089 123.055  1.00 30.00           C
ATOM   7167  C   ASN C 695     111.612  95.237 122.147  1.00 30.00           C
ATOM   7168  O   ASN C 695     111.354  94.082 122.488  1.00 30.00           O
ATOM   7169  CB  ASN C 695     111.700  96.873 124.104  1.00 20.00           C
ATOM   7170  CG  ASN C 695     112.544  97.599 125.154  1.00 20.00           C
ATOM   7171  ND2 ASN C 695     113.167  96.844 126.061  1.00 20.00           N
ATOM   7172  OD1 ASN C 695     112.619  98.824 125.163  1.00 20.00           O
ATOM   7173  H   ASN C 695     113.110  97.981 122.302  1.00  0.00           H
ATOM   7174  HA  ASN C 695     113.183  95.400 123.589  1.00  0.00           H
ATOM   7175  HB2 ASN C 695     111.036  97.589 123.616  1.00  0.00           H
ATOM   7176  HB3 ASN C 695     111.065  96.191 124.665  1.00  0.00           H
ATOM   7177 HD21 ASN C 695     113.701  97.278 126.799  1.00  0.00           H
ATOM   7178 HD22 ASN C 695     113.043  95.842 126.061  1.00  0.00           H
ATOM   7179  N   ILE C 696     111.174  95.788 120.999  1.00 30.00           N
ATOM   7180  CA  ILE C 696     110.436  95.059 119.960  1.00 30.00           C
ATOM   7181  C   ILE C 696     111.315  94.001 119.260  1.00 30.00           C
ATOM   7182  O   ILE C 696     110.818  92.906 119.002  1.00 30.00           O
ATOM   7183  CB  ILE C 696     109.801  96.015 118.899  1.00 20.00           C
ATOM   7184  CG1 ILE C 696     108.654  96.849 119.516  1.00 20.00           C
ATOM   7185  CG2 ILE C 696     109.300  95.323 117.603  1.00 20.00           C
ATOM   7186  CD1 ILE C 696     108.375  98.170 118.779  1.00 20.00           C
ATOM   7187  H   ILE C 696     111.388  96.757 120.800  1.00  0.00           H
ATOM   7188  HA  ILE C 696     109.622  94.524 120.451  1.00  0.00           H
ATOM   7189  HB  ILE C 696     110.581  96.718 118.613  1.00  0.00           H
ATOM   7190 HG12 ILE C 696     107.742  96.253 119.558  1.00  0.00           H
ATOM   7191 HG13 ILE C 696     108.882  97.093 120.554  1.00  0.00           H
ATOM   7192 HG21 ILE C 696     108.803  96.022 116.933  1.00  0.00           H
ATOM   7193 HG22 ILE C 696     110.113  94.883 117.025  1.00  0.00           H
ATOM   7194 HG23 ILE C 696     108.586  94.531 117.832  1.00  0.00           H
ATOM   7195 HD11 ILE C 696     109.156  98.902 118.977  1.00  0.00           H
ATOM   7196 HD12 ILE C 696     108.320  98.043 117.700  1.00  0.00           H
ATOM   7197 HD13 ILE C 696     107.430  98.603 119.108  1.00  0.00           H
ATOM   7198  N   TRP C 697     112.599  94.313 119.013  1.00 30.00           N
ATOM   7199  CA  TRP C 697     113.578  93.376 118.447  1.00 30.00           C
ATOM   7200  C   TRP C 697     113.820  92.148 119.346  1.00 30.00           C
ATOM   7201  O   TRP C 697     113.897  91.029 118.836  1.00 30.00           O
ATOM   7202  CB  TRP C 697     114.899  94.104 118.119  1.00 20.00           C
ATOM   7203  CG  TRP C 697     116.029  93.250 117.608  1.00 20.00           C
ATOM   7204  CD1 TRP C 697     116.243  92.928 116.314  1.00 20.00           C
ATOM   7205  CD2 TRP C 697     117.057  92.537 118.363  1.00 20.00           C
ATOM   7206  CE2 TRP C 697     117.893  91.833 117.443  1.00 20.00           C
ATOM   7207  CE3 TRP C 697     117.374  92.416 119.737  1.00 20.00           C
ATOM   7208  NE1 TRP C 697     117.377  92.144 116.205  1.00 20.00           N
ATOM   7209  CZ2 TRP C 697     118.979  91.046 117.863  1.00 20.00           C
ATOM   7210  CZ3 TRP C 697     118.459  91.630 120.170  1.00 20.00           C
ATOM   7211  CH2 TRP C 697     119.260  90.947 119.237  1.00 20.00           C
ATOM   7212  H   TRP C 697     112.923  95.260 119.177  1.00  0.00           H
ATOM   7213  HA  TRP C 697     113.170  93.004 117.505  1.00  0.00           H
ATOM   7214  HB2 TRP C 697     114.710  94.887 117.383  1.00  0.00           H
ATOM   7215  HB3 TRP C 697     115.266  94.615 119.009  1.00  0.00           H
ATOM   7216  HD1 TRP C 697     115.619  93.279 115.504  1.00  0.00           H
ATOM   7217  HE1 TRP C 697     117.783  91.896 115.313  1.00  0.00           H
ATOM   7218  HE3 TRP C 697     116.772  92.945 120.460  1.00  0.00           H
ATOM   7219  HZ2 TRP C 697     119.604  90.542 117.142  1.00  0.00           H
ATOM   7220  HZ3 TRP C 697     118.695  91.563 121.221  1.00  0.00           H
ATOM   7221  HH2 TRP C 697     120.100  90.365 119.583  1.00  0.00           H
ATOM   7222  N   LYS C 698     113.886  92.364 120.671  1.00 30.00           N
ATOM   7223  CA  LYS C 698     114.025  91.289 121.663  1.00 30.00           C
ATOM   7224  C   LYS C 698     112.868  90.276 121.596  1.00 30.00           C
ATOM   7225  O   LYS C 698     113.117  89.076 121.712  1.00 30.00           O
ATOM   7226  CB  LYS C 698     114.124  91.882 123.088  1.00 20.00           C
ATOM   7227  CG  LYS C 698     115.399  92.692 123.379  1.00 20.00           C
ATOM   7228  CD  LYS C 698     116.656  91.835 123.598  1.00 20.00           C
ATOM   7229  CE  LYS C 698     117.919  92.673 123.857  1.00 20.00           C
ATOM   7230  NZ  LYS C 698     117.826  93.440 125.112  1.00 20.00           N1+
ATOM   7231  H   LYS C 698     113.747  93.295 121.038  1.00  0.00           H
ATOM   7232  HA  LYS C 698     114.946  90.750 121.437  1.00  0.00           H
```

ATOM	7233	HB2	LYS	C	698	113.257	92.517	123.270	1.00	0.00	H
ATOM	7234	HB3	LYS	C	698	114.054	91.084	123.829	1.00	0.00	H
ATOM	7235	HG2	LYS	C	698	115.590	93.381	122.561	1.00	0.00	H
ATOM	7236	HG3	LYS	C	698	115.217	93.312	124.258	1.00	0.00	H
ATOM	7237	HD2	LYS	C	698	116.493	91.152	124.432	1.00	0.00	H
ATOM	7238	HD3	LYS	C	698	116.825	91.204	122.726	1.00	0.00	H
ATOM	7239	HE2	LYS	C	698	118.094	93.364	123.030	1.00	0.00	H
ATOM	7240	HE3	LYS	C	698	118.791	92.020	123.916	1.00	0.00	H
ATOM	7241	HZ1	LYS	C	698	117.695	92.807	125.887	1.00	0.00	H
ATOM	7242	HZ2	LYS	C	698	118.677	93.966	125.252	1.00	0.00	H
ATOM	7243	HZ3	LYS	C	698	117.042	94.075	125.062	1.00	0.00	H
ATOM	7244	N	LEU	C	699	111.641	90.781	121.378	1.00	30.00	N
ATOM	7245	CA	LEU	C	699	110.428	89.980	121.225	1.00	30.00	C
ATOM	7246	C	LEU	C	699	110.332	89.303	119.844	1.00	30.00	C
ATOM	7247	O	LEU	C	699	109.827	88.184	119.777	1.00	30.00	O
ATOM	7248	CB	LEU	C	699	109.205	90.850	121.600	1.00	20.00	C
ATOM	7249	CG	LEU	C	699	107.867	90.082	121.766	1.00	20.00	C
ATOM	7250	CD1	LEU	C	699	106.968	90.694	122.858	1.00	20.00	C
ATOM	7251	CD2	LEU	C	699	107.101	89.908	120.439	1.00	20.00	C
ATOM	7252	H	LEU	C	699	111.528	91.781	121.287	1.00	0.00	H
ATOM	7253	HA	LEU	C	699	110.486	89.178	121.960	1.00	0.00	H
ATOM	7254	HB2	LEU	C	699	109.453	91.322	122.553	1.00	0.00	H
ATOM	7255	HB3	LEU	C	699	109.086	91.678	120.899	1.00	0.00	H
ATOM	7256	HG	LEU	C	699	108.122	89.084	122.127	1.00	0.00	H
ATOM	7257	HD11	LEU	C	699	106.424	89.915	123.393	1.00	0.00	H
ATOM	7258	HD12	LEU	C	699	107.533	91.249	123.604	1.00	0.00	H
ATOM	7259	HD13	LEU	C	699	106.234	91.383	122.442	1.00	0.00	H
ATOM	7260	HD21	LEU	C	699	106.931	88.851	120.241	1.00	0.00	H
ATOM	7261	HD22	LEU	C	699	106.124	90.392	120.449	1.00	0.00	H
ATOM	7262	HD23	LEU	C	699	107.644	90.319	119.588	1.00	0.00	H
ATOM	7263	N	GLN	C	700	110.862	89.939	118.787	1.00	30.00	N
ATOM	7264	CA	GLN	C	700	110.981	89.340	117.454	1.00	30.00	C
ATOM	7265	C	GLN	C	700	111.977	88.164	117.395	1.00	30.00	C
ATOM	7266	O	GLN	C	700	111.691	87.171	116.723	1.00	30.00	O
ATOM	7267	CB	GLN	C	700	111.287	90.418	116.392	1.00	20.00	C
ATOM	7268	CG	GLN	C	700	110.058	91.285	116.036	1.00	20.00	C
ATOM	7269	CD	GLN	C	700	110.320	92.291	114.912	1.00	20.00	C
ATOM	7270	NE2	GLN	C	700	109.307	93.070	114.545	1.00	20.00	N
ATOM	7271	OE1	GLN	C	700	111.414	92.365	114.364	1.00	20.00	O
ATOM	7272	H	GLN	C	700	111.207	90.886	118.897	1.00	0.00	H
ATOM	7273	HA	GLN	C	700	110.008	88.909	117.209	1.00	0.00	H
ATOM	7274	HB2	GLN	C	700	112.107	91.053	116.727	1.00	0.00	H
ATOM	7275	HB3	GLN	C	700	111.647	89.938	115.481	1.00	0.00	H
ATOM	7276	HG2	GLN	C	700	109.236	90.640	115.724	1.00	0.00	H
ATOM	7277	HG3	GLN	C	700	109.713	91.826	116.916	1.00	0.00	H
ATOM	7278	HE21	GLN	C	700	109.417	93.746	113.797	1.00	0.00	H
ATOM	7279	HE22	GLN	C	700	108.403	92.991	114.984	1.00	0.00	H
ATOM	7280	N	ARG	C	701	113.100	88.254	118.127	1.00	30.00	N
ATOM	7281	CA	ARG	C	701	114.026	87.132	118.324	1.00	30.00	C
ATOM	7282	C	ARG	C	701	113.382	85.997	119.149	1.00	30.00	C
ATOM	7283	O	ARG	C	701	113.569	84.835	118.793	1.00	30.00	O
ATOM	7284	CB	ARG	C	701	115.368	87.626	118.930	1.00	20.00	C
ATOM	7285	CG	ARG	C	701	116.362	86.488	119.266	1.00	20.00	C
ATOM	7286	CD	ARG	C	701	117.690	86.922	119.906	1.00	20.00	C
ATOM	7287	NE	ARG	C	701	118.673	87.420	118.934	1.00	20.00	N
ATOM	7288	CZ	ARG	C	701	119.955	87.706	119.232	1.00	20.00	C
ATOM	7289	NH1	ARG	C	701	120.424	87.581	120.482	1.00	20.00	N
ATOM	7290	NH2	ARG	C	701	120.790	88.114	118.269	1.00	20.00	N1+
ATOM	7291	H	ARG	C	701	113.296	89.098	118.648	1.00	0.00	H
ATOM	7292	HA	ARG	C	701	114.253	86.726	117.336	1.00	0.00	H
ATOM	7293	HB2	ARG	C	701	115.837	88.338	118.250	1.00	0.00	H
ATOM	7294	HB3	ARG	C	701	115.155	88.179	119.847	1.00	0.00	H
ATOM	7295	HG2	ARG	C	701	115.919	85.734	119.912	1.00	0.00	H
ATOM	7296	HG3	ARG	C	701	116.585	85.980	118.327	1.00	0.00	H
ATOM	7297	HD2	ARG	C	701	117.503	87.772	120.564	1.00	0.00	H
ATOM	7298	HD3	ARG	C	701	118.090	86.124	120.534	1.00	0.00	H
ATOM	7299	HE	ARG	C	701	118.348	87.532	117.986	1.00	0.00	H
ATOM	7300	HH11	ARG	C	701	121.389	87.785	120.698	1.00	0.00	H
ATOM	7301	HH12	ARG	C	701	119.817	87.259	121.223	1.00	0.00	H
ATOM	7302	HH21	ARG	C	701	121.761	88.293	118.480	1.00	0.00	H
ATOM	7303	HH22	ARG	C	701	120.480	88.178	117.312	1.00	0.00	H

ATOM	7304	N	ALA	C	702	112.630	86.350	120.210	1.00	30.00	N
ATOM	7305	CA	ALA	C	702	111.934	85.408	121.093	1.00	30.00	C
ATOM	7306	C	ALA	C	702	110.871	84.553	120.382	1.00	30.00	C
ATOM	7307	O	ALA	C	702	110.723	83.387	120.742	1.00	30.00	O
ATOM	7308	CB	ALA	C	702	111.317	86.160	122.279	1.00	20.00	C
ATOM	7309	H	ALA	C	702	112.532	87.329	120.444	1.00	0.00	H
ATOM	7310	HA	ALA	C	702	112.682	84.724	121.495	1.00	0.00	H
ATOM	7311	HB1	ALA	C	702	110.871	85.467	122.994	1.00	0.00	H
ATOM	7312	HB2	ALA	C	702	112.069	86.740	122.815	1.00	0.00	H
ATOM	7313	HB3	ALA	C	702	110.532	86.842	121.957	1.00	0.00	H
ATOM	7314	N	ILE	C	703	110.194	85.121	119.367	1.00	30.00	N
ATOM	7315	CA	ILE	C	703	109.262	84.401	118.497	1.00	30.00	C
ATOM	7316	C	ILE	C	703	109.974	83.310	117.668	1.00	30.00	C
ATOM	7317	O	ILE	C	703	109.532	82.165	117.716	1.00	30.00	O
ATOM	7318	CB	ILE	C	703	108.444	85.362	117.575	1.00	20.00	C
ATOM	7319	CG1	ILE	C	703	107.428	86.180	118.408	1.00	20.00	C
ATOM	7320	CG2	ILE	C	703	107.712	84.672	116.399	1.00	20.00	C
ATOM	7321	CD1	ILE	C	703	106.985	87.496	117.749	1.00	20.00	C
ATOM	7322	H	ILE	C	703	110.352	86.095	119.147	1.00	0.00	H
ATOM	7323	HA	ILE	C	703	108.549	83.890	119.148	1.00	0.00	H
ATOM	7324	HB	ILE	C	703	109.150	86.073	117.142	1.00	0.00	H
ATOM	7325	HG12	ILE	C	703	106.553	85.569	118.632	1.00	0.00	H
ATOM	7326	HG13	ILE	C	703	107.856	86.431	119.378	1.00	0.00	H
ATOM	7327	HG21	ILE	C	703	107.098	85.368	115.830	1.00	0.00	H
ATOM	7328	HG22	ILE	C	703	108.405	84.232	115.681	1.00	0.00	H
ATOM	7329	HG23	ILE	C	703	107.055	83.879	116.758	1.00	0.00	H
ATOM	7330	HD11	ILE	C	703	106.061	87.861	118.200	1.00	0.00	H
ATOM	7331	HD12	ILE	C	703	107.742	88.270	117.876	1.00	0.00	H
ATOM	7332	HD13	ILE	C	703	106.802	87.392	116.680	1.00	0.00	H
ATOM	7333	N	THR	C	704	111.078	83.657	116.977	1.00	30.00	N
ATOM	7334	CA	THR	C	704	111.855	82.713	116.157	1.00	30.00	C
ATOM	7335	C	THR	C	704	112.545	81.589	116.964	1.00	30.00	C
ATOM	7336	O	THR	C	704	112.711	80.498	116.420	1.00	30.00	O
ATOM	7337	CB	THR	C	704	112.930	83.415	115.282	1.00	20.00	C
ATOM	7338	CG2	THR	C	704	112.378	84.546	114.406	1.00	20.00	C
ATOM	7339	OG1	THR	C	704	114.049	83.879	116.017	1.00	20.00	O
ATOM	7340	H	THR	C	704	111.399	84.614	116.994	1.00	0.00	H
ATOM	7341	HA	THR	C	704	111.147	82.228	115.481	1.00	0.00	H
ATOM	7342	HB	THR	C	704	113.327	82.665	114.596	1.00	0.00	H
ATOM	7343	HG1	THR	C	704	113.742	84.474	116.707	1.00	0.00	H
ATOM	7344	HG21	THR	C	704	113.147	84.918	113.729	1.00	0.00	H
ATOM	7345	HG22	THR	C	704	111.545	84.193	113.799	1.00	0.00	H
ATOM	7346	HG23	THR	C	704	112.020	85.384	114.997	1.00	0.00	H
ATOM	7347	N	ILE	C	705	112.884	81.840	118.242	1.00	30.00	N
ATOM	7348	CA	ILE	C	705	113.388	80.821	119.168	1.00	30.00	C
ATOM	7349	C	ILE	C	705	112.328	79.744	119.483	1.00	30.00	C
ATOM	7350	O	ILE	C	705	112.666	78.561	119.489	1.00	30.00	O
ATOM	7351	CB	ILE	C	705	113.937	81.453	120.489	1.00	20.00	C
ATOM	7352	CG1	ILE	C	705	115.274	82.183	120.224	1.00	20.00	C
ATOM	7353	CG2	ILE	C	705	114.109	80.466	121.668	1.00	20.00	C
ATOM	7354	CD1	ILE	C	705	115.612	83.277	121.239	1.00	20.00	C
ATOM	7355	H	ILE	C	705	112.737	82.763	118.626	1.00	0.00	H
ATOM	7356	HA	ILE	C	705	114.216	80.311	118.672	1.00	0.00	H
ATOM	7357	HB	ILE	C	705	113.218	82.208	120.810	1.00	0.00	H
ATOM	7358	HG12	ILE	C	705	116.088	81.460	120.229	1.00	0.00	H
ATOM	7359	HG13	ILE	C	705	115.275	82.636	119.231	1.00	0.00	H
ATOM	7360	HG21	ILE	C	705	114.604	80.928	122.521	1.00	0.00	H
ATOM	7361	HG22	ILE	C	705	113.153	80.094	122.038	1.00	0.00	H
ATOM	7362	HG23	ILE	C	705	114.710	79.607	121.370	1.00	0.00	H
ATOM	7363	HD11	ILE	C	705	116.545	83.772	120.974	1.00	0.00	H
ATOM	7364	HD12	ILE	C	705	114.833	84.034	121.290	1.00	0.00	H
ATOM	7365	HD13	ILE	C	705	115.743	82.859	122.233	1.00	0.00	H
ATOM	7366	N	LEU	C	706	111.074	80.171	119.707	1.00	30.00	N
ATOM	7367	CA	LEU	C	706	109.956	79.284	120.027	1.00	30.00	C
ATOM	7368	C	LEU	C	706	109.421	78.525	118.804	1.00	30.00	C
ATOM	7369	O	LEU	C	706	109.148	77.335	118.938	1.00	30.00	O
ATOM	7370	CB	LEU	C	706	108.821	80.086	120.695	1.00	20.00	C
ATOM	7371	CG	LEU	C	706	109.165	80.658	122.087	1.00	20.00	C
ATOM	7372	CD1	LEU	C	706	108.029	81.576	122.574	1.00	20.00	C
ATOM	7373	CD2	LEU	C	706	109.526	79.572	123.123	1.00	20.00	C
ATOM	7374	H	LEU	C	706	110.865	81.159	119.668	1.00	0.00	H

```
ATOM   7375  HA  LEU C 706     110.317  78.530 120.728  1.00  0.00           H
ATOM   7376  HB2 LEU C 706     108.528  80.899 120.029  1.00  0.00           H
ATOM   7377  HB3 LEU C 706     107.936  79.455 120.798  1.00  0.00           H
ATOM   7378  HG  LEU C 706     110.047  81.286 121.977  1.00  0.00           H
ATOM   7379 HD11 LEU C 706     108.426  82.492 123.013  1.00  0.00           H
ATOM   7380 HD12 LEU C 706     107.361  81.871 121.764  1.00  0.00           H
ATOM   7381 HD13 LEU C 706     107.409  81.085 123.322  1.00  0.00           H
ATOM   7382 HD21 LEU C 706     108.982  79.688 124.060  1.00  0.00           H
ATOM   7383 HD22 LEU C 706     109.313  78.567 122.760  1.00  0.00           H
ATOM   7384 HD23 LEU C 706     110.588  79.610 123.366  1.00  0.00           H
ATOM   7385  N   ASP C 707     109.282  79.205 117.651  1.00 30.00           N
ATOM   7386  CA  ASP C 707     108.739  78.641 116.404  1.00 30.00           C
ATOM   7387  C   ASP C 707     109.586  77.500 115.813  1.00 30.00           C
ATOM   7388  O   ASP C 707     109.015  76.574 115.237  1.00 30.00           O
ATOM   7389  CB  ASP C 707     108.446  79.698 115.309  1.00 20.00           C
ATOM   7390  CG  ASP C 707     107.503  80.840 115.710  1.00  0.00           C
ATOM   7391  OD1 ASP C 707     106.911  80.781 116.812  1.00  0.00           O
ATOM   7392  OD2 ASP C 707     107.343  81.752 114.872  1.00  0.00           O1-
ATOM   7393  H   ASP C 707     109.498  80.194 117.631  1.00  0.00           H
ATOM   7394  HA  ASP C 707     107.780  78.191 116.672  1.00  0.00           H
ATOM   7395  HB2 ASP C 707     109.392  80.146 115.001  1.00  0.00           H
ATOM   7396  HB3 ASP C 707     108.009  79.202 114.441  1.00  0.00           H
ATOM   7397  N   THR C 708     110.916  77.561 115.991  1.00 30.00           N
ATOM   7398  CA  THR C 708     111.837  76.500 115.579  1.00 30.00           C
ATOM   7399  C   THR C 708     111.847  75.293 116.541  1.00 30.00           C
ATOM   7400  O   THR C 708     112.212  74.212 116.086  1.00 30.00           O
ATOM   7401  CB  THR C 708     113.288  77.014 115.424  1.00 20.00           C
ATOM   7402  CG2 THR C 708     113.418  78.065 114.308  1.00 20.00           C
ATOM   7403  OG1 THR C 708     113.786  77.520 116.648  1.00 20.00           O
ATOM   7404  H   THR C 708     111.321  78.352 116.473  1.00  0.00           H
ATOM   7405  HA  THR C 708     111.516  76.136 114.602  1.00  0.00           H
ATOM   7406  HB  THR C 708     113.937  76.179 115.153  1.00  0.00           H
ATOM   7407  HG1 THR C 708     113.412  78.393 116.798  1.00  0.00           H
ATOM   7408 HG21 THR C 708     114.434  78.447 114.227  1.00  0.00           H
ATOM   7409 HG22 THR C 708     113.162  77.629 113.342  1.00  0.00           H
ATOM   7410 HG23 THR C 708     112.753  78.914 114.459  1.00  0.00           H
ATOM   7411  N   GLU C 709     111.397  75.450 117.800  1.00 30.00           N
ATOM   7412  CA  GLU C 709     111.153  74.323 118.711  1.00 30.00           C
ATOM   7413  C   GLU C 709     109.848  73.569 118.391  1.00 30.00           C
ATOM   7414  O   GLU C 709     109.778  72.374 118.679  1.00 30.00           O
ATOM   7415  CB  GLU C 709     111.198  74.784 120.182  1.00 20.00           C
ATOM   7416  CG  GLU C 709     112.618  75.173 120.641  1.00 20.00           C
ATOM   7417  CD  GLU C 709     112.701  75.580 122.114  1.00 20.00           C
ATOM   7418  OE1 GLU C 709     111.703  76.117 122.644  1.00 20.00           O
ATOM   7419  OE2 GLU C 709     113.784  75.366 122.700  1.00 20.00           O1-
ATOM   7420  H   GLU C 709     111.116  76.363 118.129  1.00  0.00           H
ATOM   7421  HA  GLU C 709     111.958  73.596 118.581  1.00  0.00           H
ATOM   7422  HB2 GLU C 709     110.523  75.628 120.324  1.00  0.00           H
ATOM   7423  HB3 GLU C 709     110.823  73.990 120.830  1.00  0.00           H
ATOM   7424  HG2 GLU C 709     113.295  74.334 120.479  1.00  0.00           H
ATOM   7425  HG3 GLU C 709     113.000  75.996 120.038  1.00  0.00           H
ATOM   7426  N   LYS C 710     108.865  74.246 117.763  1.00 30.00           N
ATOM   7427  CA  LYS C 710     107.653  73.613 117.228  1.00 30.00           C
ATOM   7428  C   LYS C 710     107.942  72.847 115.921  1.00 30.00           C
ATOM   7429  O   LYS C 710     107.303  71.824 115.677  1.00 30.00           O
ATOM   7430  CB  LYS C 710     106.538  74.660 116.986  1.00 20.00           C
ATOM   7431  CG  LYS C 710     106.215  75.605 118.161  1.00  0.00           C
ATOM   7432  CD  LYS C 710     105.878  74.911 119.493  1.00  0.00           C
ATOM   7433  CE  LYS C 710     105.686  75.895 120.661  1.00  0.00           C
ATOM   7434  NZ  LYS C 710     106.951  76.557 121.026  1.00  0.00           N1+
ATOM   7435  H   LYS C 710     108.984  75.230 117.567  1.00  0.00           H
ATOM   7436  HA  LYS C 710     107.289  72.884 117.954  1.00  0.00           H
ATOM   7437  HB2 LYS C 710     106.805  75.284 116.132  1.00  0.00           H
ATOM   7438  HB3 LYS C 710     105.625  74.139 116.694  1.00  0.00           H
ATOM   7439  HG2 LYS C 710     107.046  76.289 118.295  1.00  0.00           H
ATOM   7440  HG3 LYS C 710     105.374  76.238 117.873  1.00  0.00           H
ATOM   7441  HD2 LYS C 710     104.966  74.326 119.369  1.00  0.00           H
ATOM   7442  HD3 LYS C 710     106.657  74.197 119.760  1.00  0.00           H
ATOM   7443  HE2 LYS C 710     104.938  76.650 120.417  1.00  0.00           H
ATOM   7444  HE3 LYS C 710     105.323  75.358 121.538  1.00  0.00           H
ATOM   7445  HZ1 LYS C 710     107.281  77.125 120.259  1.00  0.00           H
```

```
ATOM   7446  HZ2 LYS C 710     106.806  77.141 121.846  1.00  0.00           H
ATOM   7447  HZ3 LYS C 710     107.647  75.861 121.250  1.00  0.00           H
ATOM   7448  N   SER C 711     108.892  73.355 115.114  1.00 30.00           N
ATOM   7449  CA  SER C 711     109.291  72.787 113.826  1.00 30.00           C
ATOM   7450  C   SER C 711     110.268  71.606 113.978  1.00 30.00           C
ATOM   7451  O   SER C 711     110.067  70.576 113.337  1.00 30.00           O
ATOM   7452  CB  SER C 711     109.884  73.914 112.953  1.00 20.00           C
ATOM   7453  OG  SER C 711     110.192  73.472 111.645  1.00  0.00           O
ATOM   7454  H   SER C 711     109.358  74.209 115.387  1.00  0.00           H
ATOM   7455  HA  SER C 711     108.394  72.416 113.325  1.00  0.00           H
ATOM   7456  HB2 SER C 711     109.178  74.742 112.874  1.00  0.00           H
ATOM   7457  HB3 SER C 711     110.792  74.314 113.404  1.00  0.00           H
ATOM   7458  HG  SER C 711     110.902  72.827 111.695  1.00  0.00           H
ATOM   7459  N   PHE C 712     111.300  71.776 114.823  1.00 30.00           N
ATOM   7460  CA  PHE C 712     112.390  70.817 115.042  1.00 30.00           C
ATOM   7461  C   PHE C 712     112.093  69.935 116.278  1.00 30.00           C
ATOM   7462  O   PHE C 712     113.009  69.569 117.014  1.00 30.00           O
ATOM   7463  CB  PHE C 712     113.707  71.625 115.154  1.00 20.00           C
ATOM   7464  CG  PHE C 712     114.992  70.871 114.855  1.00  0.00           C
ATOM   7465  CD1 PHE C 712     115.324  70.554 113.521  1.00  0.00           C
ATOM   7466  CD2 PHE C 712     115.819  70.402 115.898  1.00  0.00           C
ATOM   7467  CE1 PHE C 712     116.481  69.838 113.245  1.00  0.00           C
ATOM   7468  CE2 PHE C 712     116.978  69.697 115.600  1.00  0.00           C
ATOM   7469  CZ  PHE C 712     117.307  69.418 114.279  1.00  0.00           C
ATOM   7470  H   PHE C 712     111.390  72.650 115.327  1.00  0.00           H
ATOM   7471  HA  PHE C 712     112.465  70.157 114.176  1.00  0.00           H
ATOM   7472  HB2 PHE C 712     113.677  72.456 114.448  1.00  0.00           H
ATOM   7473  HB3 PHE C 712     113.787  72.085 116.140  1.00  0.00           H
ATOM   7474  HD1 PHE C 712     114.686  70.876 112.711  1.00  0.00           H
ATOM   7475  HD2 PHE C 712     115.563  70.597 116.929  1.00  0.00           H
ATOM   7476  HE1 PHE C 712     116.739  69.607 112.221  1.00  0.00           H
ATOM   7477  HE2 PHE C 712     117.616  69.354 116.400  1.00  0.00           H
ATOM   7478  HZ  PHE C 712     118.206  68.862 114.057  1.00  0.00           H
ATOM   7479  N   LEU C 713     110.806  69.603 116.477  1.00 30.00           N
ATOM   7480  CA  LEU C 713     110.286  68.806 117.589  1.00 30.00           C
ATOM   7481  C   LEU C 713     110.641  67.306 117.493  1.00 30.00           C
ATOM   7482  O   LEU C 713     110.665  66.635 118.524  1.00 30.00           O
ATOM   7483  CB  LEU C 713     108.760  69.053 117.661  1.00 20.00           C
ATOM   7484  CG  LEU C 713     108.019  68.454 118.879  1.00 20.00           C
ATOM   7485  CD1 LEU C 713     108.588  68.959 120.223  1.00 20.00           C
ATOM   7486  CD2 LEU C 713     106.499  68.697 118.763  1.00 20.00           C
ATOM   7487  H   LEU C 713     110.113  69.948 115.828  1.00  0.00           H
ATOM   7488  HA  LEU C 713     110.748  69.190 118.499  1.00  0.00           H
ATOM   7489  HB2 LEU C 713     108.578  70.129 117.652  1.00  0.00           H
ATOM   7490  HB3 LEU C 713     108.303  68.673 116.745  1.00  0.00           H
ATOM   7491  HG  LEU C 713     108.150  67.372 118.857  1.00  0.00           H
ATOM   7492 HD11 LEU C 713     107.810  69.187 120.951  1.00  0.00           H
ATOM   7493 HD12 LEU C 713     109.230  68.203 120.674  1.00  0.00           H
ATOM   7494 HD13 LEU C 713     109.185  69.864 120.102  1.00  0.00           H
ATOM   7495 HD21 LEU C 713     105.949  67.760 118.854  1.00  0.00           H
ATOM   7496 HD22 LEU C 713     106.120  69.371 119.532  1.00  0.00           H
ATOM   7497 HD23 LEU C 713     106.226  69.137 117.803  1.00  0.00           H
ATOM   7498  N   LYS C 714     110.947  66.811 116.279  1.00 30.00           N
ATOM   7499  CA  LYS C 714     111.394  65.435 116.025  1.00 30.00           C
ATOM   7500  C   LYS C 714     112.804  65.133 116.586  1.00 30.00           C
ATOM   7501  O   LYS C 714     113.117  63.965 116.814  1.00 30.00           O
ATOM   7502  CB  LYS C 714     111.269  65.146 114.511  1.00 20.00           C
ATOM   7503  CG  LYS C 714     111.545  63.685 114.107  1.00  0.00           C
ATOM   7504  CD  LYS C 714     111.178  63.386 112.646  1.00  0.00           C
ATOM   7505  CE  LYS C 714     111.414  61.914 112.275  1.00  0.00           C
ATOM   7506  NZ  LYS C 714     111.037  61.644 110.877  1.00  0.00           N1+
ATOM   7507  H   LYS C 714     110.906  67.422 115.476  1.00  0.00           H
ATOM   7508  HA  LYS C 714     110.700  64.771 116.545  1.00  0.00           H
ATOM   7509  HB2 LYS C 714     110.251  65.392 114.205  1.00  0.00           H
ATOM   7510  HB3 LYS C 714     111.921  65.814 113.946  1.00  0.00           H
ATOM   7511  HG2 LYS C 714     112.599  63.451 114.256  1.00  0.00           H
ATOM   7512  HG3 LYS C 714     110.986  63.017 114.764  1.00  0.00           H
ATOM   7513  HD2 LYS C 714     110.131  63.643 112.475  1.00  0.00           H
ATOM   7514  HD3 LYS C 714     111.763  64.029 111.988  1.00  0.00           H
ATOM   7515  HE2 LYS C 714     112.463  61.652 112.414  1.00  0.00           H
ATOM   7516  HE3 LYS C 714     110.829  61.262 112.925  1.00  0.00           H
```

```
ATOM   7517  HZ1 LYS C 714     111.592  62.218 110.259  1.00  0.00           H
ATOM   7518  HZ2 LYS C 714     111.200  60.670 110.666  1.00  0.00           H
ATOM   7519  HZ3 LYS C 714     110.058  61.856 110.744  1.00  0.00           H
ATOM   7520  N   CYS C 715     113.602  66.184 116.839  1.00 30.00           N
ATOM   7521  CA  CYS C 715     114.907  66.119 117.491  1.00 30.00           C
ATOM   7522  C   CYS C 715     114.916  67.158 118.627  1.00 30.00           C
ATOM   7523  O   CYS C 715     115.644  68.150 118.569  1.00 30.00           O
ATOM   7524  CB  CYS C 715     116.051  66.250 116.460  1.00 20.00           C
ATOM   7525  SG  CYS C 715     117.702  66.163 117.219  1.00  0.00           S
ATOM   7526  H   CYS C 715     113.260  67.115 116.645  1.00  0.00           H
ATOM   7527  HA  CYS C 715     115.025  65.149 117.977  1.00  0.00           H
ATOM   7528  HB2 CYS C 715     115.978  65.448 115.724  1.00  0.00           H
ATOM   7529  HB3 CYS C 715     115.960  67.184 115.907  1.00  0.00           H
ATOM   7530  HG  CYS C 715     117.604  67.329 117.866  1.00  0.00           H
ATOM   7531  N   MET C 716     114.072  66.903 119.642  1.00 30.00           N
ATOM   7532  CA  MET C 716     113.874  67.750 120.824  1.00 30.00           C
ATOM   7533  C   MET C 716     114.890  67.490 121.960  1.00 30.00           C
ATOM   7534  O   MET C 716     114.600  67.825 123.109  1.00 30.00           O
ATOM   7535  CB  MET C 716     112.411  67.621 121.304  1.00 20.00           C
ATOM   7536  CG  MET C 716     111.982  66.203 121.728  1.00  0.00           C
ATOM   7537  SD  MET C 716     110.447  66.144 122.690  1.00  0.00           S
ATOM   7538  CE  MET C 716     111.058  66.724 124.297  1.00  0.00           C
ATOM   7539  H   MET C 716     113.491  66.079 119.596  1.00  0.00           H
ATOM   7540  HA  MET C 716     114.018  68.791 120.525  1.00  0.00           H
ATOM   7541  HB2 MET C 716     112.229  68.321 122.119  1.00  0.00           H
ATOM   7542  HB3 MET C 716     111.753  67.962 120.505  1.00  0.00           H
ATOM   7543  HG2 MET C 716     111.842  65.586 120.840  1.00  0.00           H
ATOM   7544  HG3 MET C 716     112.757  65.714 122.317  1.00  0.00           H
ATOM   7545  HE1 MET C 716     110.247  66.738 125.025  1.00  0.00           H
ATOM   7546  HE2 MET C 716     111.841  66.063 124.668  1.00  0.00           H
ATOM   7547  HE3 MET C 716     111.465  67.732 124.221  1.00  0.00           H
ATOM   7548  N   ARG C 717     116.066  66.924 121.636  1.00 30.00           N
ATOM   7549  CA  ARG C 717     117.164  66.668 122.578  1.00 30.00           C
ATOM   7550  C   ARG C 717     117.789  67.951 123.169  1.00 30.00           C
ATOM   7551  O   ARG C 717     118.345  67.887 124.264  1.00 30.00           O
ATOM   7552  CB  ARG C 717     118.245  65.798 121.899  1.00 20.00           C
ATOM   7553  CG  ARG C 717     117.733  64.430 121.403  1.00  0.00           C
ATOM   7554  CD  ARG C 717     118.830  63.545 120.787  1.00  0.00           C
ATOM   7555  NE  ARG C 717     119.265  64.030 119.468  1.00  0.00           N
ATOM   7556  CZ  ARG C 717     120.358  63.625 118.798  1.00  0.00           C
ATOM   7557  NH1 ARG C 717     121.198  62.716 119.312  1.00  0.00           N
ATOM   7558  NH2 ARG C 717     120.609  64.138 117.587  1.00  0.00           N1+
ATOM   7559  H   ARG C 717     116.241  66.682 120.672  1.00  0.00           H
ATOM   7560  HA  ARG C 717     116.750  66.101 123.414  1.00  0.00           H
ATOM   7561  HB2 ARG C 717     118.695  66.346 121.068  1.00  0.00           H
ATOM   7562  HB3 ARG C 717     119.048  65.622 122.616  1.00  0.00           H
ATOM   7563  HG2 ARG C 717     117.209  63.881 122.187  1.00  0.00           H
ATOM   7564  HG3 ARG C 717     116.989  64.625 120.629  1.00  0.00           H
ATOM   7565  HD2 ARG C 717     119.716  63.646 121.415  1.00  0.00           H
ATOM   7566  HD3 ARG C 717     118.570  62.487 120.794  1.00  0.00           H
ATOM   7567  HE  ARG C 717     118.642  64.682 119.011  1.00  0.00           H
ATOM   7568 HH11 ARG C 717     122.013  62.416 118.797  1.00  0.00           H
ATOM   7569 HH12 ARG C 717     121.015  62.318 120.222  1.00  0.00           H
ATOM   7570 HH21 ARG C 717     121.420  63.844 117.062  1.00  0.00           H
ATOM   7571 HH22 ARG C 717     119.978  64.815 117.180  1.00  0.00           H
ATOM   7572  N   LYS C 718     117.669  69.081 122.448  1.00 30.00           N
ATOM   7573  CA  LYS C 718     118.154  70.405 122.847  1.00 30.00           C
ATOM   7574  C   LYS C 718     117.016  71.375 123.235  1.00 30.00           C
ATOM   7575  O   LYS C 718     117.315  72.524 123.565  1.00 30.00           O
ATOM   7576  CB  LYS C 718     119.026  70.981 121.709  1.00 20.00           C
ATOM   7577  CG  LYS C 718     120.342  70.209 121.502  1.00  0.00           C
ATOM   7578  CD  LYS C 718     121.233  70.838 120.422  1.00  0.00           C
ATOM   7579  CE  LYS C 718     122.574  70.110 120.262  1.00  0.00           C
ATOM   7580  NZ  LYS C 718     123.410  70.749 119.231  1.00  0.00           N1+
ATOM   7581  H   LYS C 718     117.192  69.040 121.558  1.00  0.00           H
ATOM   7582  HA  LYS C 718     118.781  70.317 123.736  1.00  0.00           H
ATOM   7583  HB2 LYS C 718     118.456  71.008 120.778  1.00  0.00           H
ATOM   7584  HB3 LYS C 718     119.283  72.017 121.933  1.00  0.00           H
ATOM   7585  HG2 LYS C 718     120.888  70.169 122.446  1.00  0.00           H
ATOM   7586  HG3 LYS C 718     120.125  69.175 121.231  1.00  0.00           H
ATOM   7587  HD2 LYS C 718     120.700  70.827 119.470  1.00  0.00           H
```

```
ATOM   7588  HD3 LYS C 718     121.411  71.888 120.663  1.00  0.00           H
ATOM   7589  HE2 LYS C 718     123.121  70.111 121.205  1.00  0.00           H
ATOM   7590  HE3 LYS C 718     122.409  69.068 119.985  1.00  0.00           H
ATOM   7591  HZ1 LYS C 718     123.595  71.708 119.490  1.00  0.00           H
ATOM   7592  HZ2 LYS C 718     124.285  70.250 119.149  1.00  0.00           H
ATOM   7593  HZ3 LYS C 718     122.929  70.728 118.344  1.00  0.00           H
ATOM   7594  N   ALA C 719     115.750  70.910 123.221  1.00 30.00           N
ATOM   7595  CA  ALA C 719     114.574  71.678 123.653  1.00 30.00           C
ATOM   7596  C   ALA C 719     114.526  71.919 125.176  1.00 30.00           C
ATOM   7597  O   ALA C 719     113.946  72.913 125.611  1.00 30.00           O
ATOM   7598  CB  ALA C 719     113.301  70.964 123.176  1.00 20.00           C
ATOM   7599  H   ALA C 719     115.576  69.952 122.953  1.00  0.00           H
ATOM   7600  HA  ALA C 719     114.615  72.654 123.164  1.00  0.00           H
ATOM   7601  HB1 ALA C 719     112.415  71.572 123.363  1.00  0.00           H
ATOM   7602  HB2 ALA C 719     113.336  70.770 122.104  1.00  0.00           H
ATOM   7603  HB3 ALA C 719     113.159  70.010 123.685  1.00  0.00           H
ATOM   7604  N   PHE C 720     115.167  71.024 125.947  1.00 30.00           N
ATOM   7605  CA  PHE C 720     115.449  71.168 127.377  1.00 30.00           C
ATOM   7606  C   PHE C 720     116.619  72.144 127.599  1.00 30.00           C
ATOM   7607  O   PHE C 720     117.492  72.233 126.732  1.00 30.00           O
ATOM   7608  CB  PHE C 720     115.785  69.772 127.931  1.00 20.00           C
ATOM   7609  CG  PHE C 720     114.674  68.738 127.801  1.00  0.00           C
ATOM   7610  CD1 PHE C 720     113.364  69.011 128.255  1.00  0.00           C
ATOM   7611  CD2 PHE C 720     114.967  67.451 127.300  1.00  0.00           C
ATOM   7612  CE1 PHE C 720     112.385  68.029 128.189  1.00  0.00           C
ATOM   7613  CE2 PHE C 720     113.970  66.488 127.229  1.00  0.00           C
ATOM   7614  CZ  PHE C 720     112.685  66.776 127.672  1.00  0.00           C
ATOM   7615  H   PHE C 720     115.604  70.231 125.500  1.00  0.00           H
ATOM   7616  HA  PHE C 720     114.567  71.567 127.880  1.00  0.00           H
ATOM   7617  HB2 PHE C 720     116.671  69.393 127.417  1.00  0.00           H
ATOM   7618  HB3 PHE C 720     116.072  69.847 128.978  1.00  0.00           H
ATOM   7619  HD1 PHE C 720     113.113  69.979 128.660  1.00  0.00           H
ATOM   7620  HD2 PHE C 720     115.965  67.209 126.964  1.00  0.00           H
ATOM   7621  HE1 PHE C 720     111.384  68.241 128.534  1.00  0.00           H
ATOM   7622  HE2 PHE C 720     114.195  65.509 126.832  1.00  0.00           H
ATOM   7623  HZ  PHE C 720     111.916  66.019 127.616  1.00  0.00           H
ATOM   7624  N   ARG C 721     116.612  72.875 128.732  1.00 30.00           N
ATOM   7625  CA  ARG C 721     117.422  74.087 128.879  1.00 30.00           C
ATOM   7626  C   ARG C 721     118.177  74.168 130.214  1.00 30.00           C
ATOM   7627  O   ARG C 721     117.591  74.509 131.241  1.00 30.00           O
ATOM   7628  CB  ARG C 721     116.512  75.300 128.578  1.00 20.00           C
ATOM   7629  CG  ARG C 721     117.142  76.704 128.406  1.00  0.00           C
ATOM   7630  CD  ARG C 721     118.670  76.902 128.292  1.00  0.00           C
ATOM   7631  NE  ARG C 721     119.430  76.091 127.318  1.00  0.00           N
ATOM   7632  CZ  ARG C 721     119.049  75.427 126.210  1.00  0.00           C
ATOM   7633  NH1 ARG C 721     117.802  75.447 125.733  1.00  0.00           N
ATOM   7634  NH2 ARG C 721     119.958  74.701 125.553  1.00  0.00           N1+
ATOM   7635  H   ARG C 721     115.901  72.724 129.433  1.00  0.00           H
ATOM   7636  HA  ARG C 721     118.194  74.066 128.114  1.00  0.00           H
ATOM   7637  HB2 ARG C 721     115.997  75.083 127.642  1.00  0.00           H
ATOM   7638  HB3 ARG C 721     115.706  75.358 129.311  1.00  0.00           H
ATOM   7639  HG2 ARG C 721     116.594  77.299 127.677  1.00  0.00           H
ATOM   7640  HG3 ARG C 721     116.922  77.176 129.364  1.00  0.00           H
ATOM   7641  HD2 ARG C 721     118.768  77.889 127.840  1.00  0.00           H
ATOM   7642  HD3 ARG C 721     119.167  76.995 129.256  1.00  0.00           H
ATOM   7643  HE  ARG C 721     120.427  76.127 127.488  1.00  0.00           H
ATOM   7644 HH11 ARG C 721     117.549  74.898 124.922  1.00  0.00           H
ATOM   7645 HH12 ARG C 721     117.095  75.988 126.208  1.00  0.00           H
ATOM   7646 HH21 ARG C 721     119.690  74.179 124.732  1.00  0.00           H
ATOM   7647 HH22 ARG C 721     120.916  74.676 125.869  1.00  0.00           H
ATOM   7648  N   SER C 722     119.493  73.894 130.121  1.00 30.00           N
ATOM   7649  CA  SER C 722     120.532  74.060 131.144  1.00 30.00           C
ATOM   7650  C   SER C 722     120.331  73.177 132.397  1.00 30.00           C
ATOM   7651  O   SER C 722     120.647  73.604 133.507  1.00 30.00           O
ATOM   7652  CB  SER C 722     120.759  75.565 131.426  1.00 20.00           C
ATOM   7653  OG  SER C 722     121.938  75.813 132.164  1.00  0.00           O
ATOM   7654  H   SER C 722     119.844  73.614 129.218  1.00  0.00           H
ATOM   7655  HA  SER C 722     121.444  73.687 130.676  1.00  0.00           H
ATOM   7656  HB2 SER C 722     120.852  76.113 130.490  1.00  0.00           H
ATOM   7657  HB3 SER C 722     119.910  75.983 131.962  1.00  0.00           H
ATOM   7658  HG  SER C 722     122.026  76.760 132.303  1.00  0.00           H
```

```
ATOM   7659  N   GLY C 723     119.847  71.944 132.176  1.00 30.00           N
ATOM   7660  CA  GLY C 723     119.670  70.921 133.203  1.00 30.00           C
ATOM   7661  C   GLY C 723     118.213  70.445 133.233  1.00 30.00           C
ATOM   7662  O   GLY C 723     117.289  71.158 132.839  1.00 30.00           O
ATOM   7663  H   GLY C 723     119.587  71.685 131.236  1.00  0.00           H
ATOM   7664  HA2 GLY C 723     120.328  70.084 132.969  1.00  0.00           H
ATOM   7665  HA3 GLY C 723     119.945  71.275 134.197  1.00  0.00           H
ATOM   7666  N   LYS C 724     118.037  69.212 133.732  1.00 30.00           N
ATOM   7667  CA  LYS C 724     116.756  68.527 133.930  1.00 30.00           C
ATOM   7668  C   LYS C 724     116.712  67.741 135.254  1.00 30.00           C
ATOM   7669  O   LYS C 724     115.679  67.137 135.542  1.00 30.00           O
ATOM   7670  CB  LYS C 724     116.469  67.596 132.724  1.00 20.00           C
ATOM   7671  CG  LYS C 724     116.025  68.305 131.429  1.00 20.00           C
ATOM   7672  CD  LYS C 724     114.711  69.103 131.533  1.00 20.00           C
ATOM   7673  CE  LYS C 724     113.487  68.249 131.909  1.00 20.00           C
ATOM   7674  NZ  LYS C 724     112.233  69.018 131.811  1.00 20.00           N1+
ATOM   7675  H   LYS C 724     118.853  68.683 134.002  1.00  0.00           H
ATOM   7676  HA  LYS C 724     115.969  69.273 134.011  1.00  0.00           H
ATOM   7677  HB2 LYS C 724     117.350  66.987 132.520  1.00  0.00           H
ATOM   7678  HB3 LYS C 724     115.688  66.879 132.980  1.00  0.00           H
ATOM   7679  HG2 LYS C 724     116.821  68.969 131.093  1.00  0.00           H
ATOM   7680  HG3 LYS C 724     115.920  67.559 130.640  1.00  0.00           H
ATOM   7681  HD2 LYS C 724     114.844  69.926 132.229  1.00  0.00           H
ATOM   7682  HD3 LYS C 724     114.522  69.596 130.585  1.00  0.00           H
ATOM   7683  HE2 LYS C 724     113.411  67.390 131.241  1.00  0.00           H
ATOM   7684  HE3 LYS C 724     113.581  67.863 132.925  1.00  0.00           H
ATOM   7685  HZ1 LYS C 724     112.262  69.808 132.439  1.00  0.00           H
ATOM   7686  HZ2 LYS C 724     112.111  69.347 130.864  1.00  0.00           H
ATOM   7687  HZ3 LYS C 724     111.453  68.425 132.060  1.00  0.00           H
ATOM   7688  N   LEU C 725     117.817  67.747 136.021  1.00 30.00           N
ATOM   7689  CA  LEU C 725     118.023  66.937 137.220  1.00 30.00           C
ATOM   7690  C   LEU C 725     117.240  67.479 138.432  1.00 30.00           C
ATOM   7691  O   LEU C 725     116.075  67.119 138.599  1.00 30.00           O
ATOM   7692  CB  LEU C 725     119.546  66.798 137.487  1.00 20.00           C
ATOM   7693  CG  LEU C 725     120.340  66.068 136.377  1.00  0.00           C
ATOM   7694  CD1 LEU C 725     121.857  66.122 136.659  1.00  0.00           C
ATOM   7695  CD2 LEU C 725     119.843  64.625 136.142  1.00  0.00           C
ATOM   7696  H   LEU C 725     118.607  68.307 135.739  1.00  0.00           H
ATOM   7697  HA  LEU C 725     117.620  65.942 137.026  1.00  0.00           H
ATOM   7698  HB2 LEU C 725     119.976  67.792 137.625  1.00  0.00           H
ATOM   7699  HB3 LEU C 725     119.702  66.268 138.429  1.00  0.00           H
ATOM   7700  HG  LEU C 725     120.186  66.615 135.446  1.00  0.00           H
ATOM   7701 HD11 LEU C 725     122.393  66.546 135.809  1.00  0.00           H
ATOM   7702 HD12 LEU C 725     122.092  66.741 137.525  1.00  0.00           H
ATOM   7703 HD13 LEU C 725     122.285  65.139 136.854  1.00  0.00           H
ATOM   7704 HD21 LEU C 725     120.658  63.918 135.990  1.00  0.00           H
ATOM   7705 HD22 LEU C 725     119.253  64.255 136.981  1.00  0.00           H
ATOM   7706 HD23 LEU C 725     119.213  64.577 135.254  1.00  0.00           H
ATOM   7707  N   LEU C 726     117.909  68.266 139.290  1.00 30.00           N
ATOM   7708  CA  LEU C 726     117.452  68.575 140.644  1.00 30.00           C
ATOM   7709  C   LEU C 726     118.192  69.789 141.216  1.00 30.00           C
ATOM   7710  O   LEU C 726     119.287  70.112 140.751  1.00 30.00           O
ATOM   7711  CB  LEU C 726     117.665  67.322 141.530  1.00 20.00           C
ATOM   7712  CG  LEU C 726     119.149  66.965 141.850  1.00  0.00           C
ATOM   7713  CD1 LEU C 726     119.602  67.477 143.238  1.00  0.00           C
ATOM   7714  CD2 LEU C 726     119.473  65.471 141.637  1.00  0.00           C
ATOM   7715  H   LEU C 726     118.856  68.550 139.082  1.00  0.00           H
ATOM   7716  HA  LEU C 726     116.390  68.817 140.607  1.00  0.00           H
ATOM   7717  HB2 LEU C 726     117.104  67.454 142.453  1.00  0.00           H
ATOM   7718  HB3 LEU C 726     117.170  66.482 141.046  1.00  0.00           H
ATOM   7719  HG  LEU C 726     119.773  67.473 141.114  1.00  0.00           H
ATOM   7720 HD11 LEU C 726     120.413  68.199 143.134  1.00  0.00           H
ATOM   7721 HD12 LEU C 726     118.803  67.978 143.783  1.00  0.00           H
ATOM   7722 HD13 LEU C 726     119.968  66.678 143.883  1.00  0.00           H
ATOM   7723 HD21 LEU C 726     119.580  64.928 142.575  1.00  0.00           H
ATOM   7724 HD22 LEU C 726     118.712  64.954 141.053  1.00  0.00           H
ATOM   7725 HD23 LEU C 726     120.411  65.357 141.093  1.00  0.00           H
ATOM   7726  N   GLN C 727     117.598  70.385 142.264  1.00 30.00           N
ATOM   7727  CA  GLN C 727     118.262  71.360 143.131  1.00 30.00           C
ATOM   7728  C   GLN C 727     117.498  71.609 144.448  1.00 30.00           C
ATOM   7729  O   GLN C 727     118.072  72.239 145.336  1.00 30.00           O
```

ATOM	7730	CB	GLN	C	727	118.556	72.697	142.384	1.00	20.00	C
ATOM	7731	CG	GLN	C	727	119.617	73.616	143.035	1.00	0.00	C
ATOM	7732	CD	GLN	C	727	120.965	72.916	143.206	1.00	0.00	C
ATOM	7733	NE2	GLN	C	727	121.175	72.284	144.362	1.00	0.00	N
ATOM	7734	OE1	GLN	C	727	121.800	72.936	142.306	1.00	0.00	O
ATOM	7735	H	GLN	C	727	116.699	70.049	142.572	1.00	0.00	H
ATOM	7736	HA	GLN	C	727	119.193	70.873	143.420	1.00	0.00	H
ATOM	7737	HB2	GLN	C	727	118.892	72.502	141.367	1.00	0.00	H
ATOM	7738	HB3	GLN	C	727	117.628	73.255	142.263	1.00	0.00	H
ATOM	7739	HG2	GLN	C	727	119.767	74.497	142.414	1.00	0.00	H
ATOM	7740	HG3	GLN	C	727	119.285	73.999	143.999	1.00	0.00	H
ATOM	7741	HE21	GLN	C	727	122.049	71.806	144.529	1.00	0.00	H
ATOM	7742	HE22	GLN	C	727	120.473	72.299	145.090	1.00	0.00	H
ATOM	7743	N	VAL	C	728	116.239	71.146	144.578	1.00	30.00	N
ATOM	7744	CA	VAL	C	728	115.404	71.426	145.753	1.00	30.00	C
ATOM	7745	C	VAL	C	728	115.907	70.682	147.009	1.00	30.00	C
ATOM	7746	O	VAL	C	728	116.222	71.333	148.005	1.00	30.00	O
ATOM	7747	CB	VAL	C	728	113.903	71.059	145.557	1.00	20.00	C
ATOM	7748	CG1	VAL	C	728	113.020	71.744	146.614	1.00	0.00	C
ATOM	7749	CG2	VAL	C	728	113.334	71.370	144.166	1.00	0.00	C
ATOM	7750	H	VAL	C	728	115.793	70.638	143.829	1.00	0.00	H
ATOM	7751	HA	VAL	C	728	115.470	72.491	145.973	1.00	0.00	H
ATOM	7752	HB	VAL	C	728	113.786	69.985	145.681	1.00	0.00	H
ATOM	7753	HG11	VAL	C	728	111.989	71.398	146.545	1.00	0.00	H
ATOM	7754	HG12	VAL	C	728	113.368	71.550	147.627	1.00	0.00	H
ATOM	7755	HG13	VAL	C	728	113.014	72.825	146.482	1.00	0.00	H
ATOM	7756	HG21	VAL	C	728	113.872	70.853	143.373	1.00	0.00	H
ATOM	7757	HG22	VAL	C	728	112.297	71.050	144.106	1.00	0.00	H
ATOM	7758	HG23	VAL	C	728	113.328	72.435	143.960	1.00	0.00	H
ATOM	7759	N	GLY	C	729	115.971	69.342	146.921	1.00	30.00	N
ATOM	7760	CA	GLY	C	729	116.424	68.429	147.970	1.00	30.00	C
ATOM	7761	C	GLY	C	729	115.535	68.379	149.224	1.00	30.00	C
ATOM	7762	O	GLY	C	729	115.937	67.755	150.205	1.00	30.00	O
ATOM	7763	H	GLY	C	729	115.692	68.905	146.052	1.00	0.00	H
ATOM	7764	HA2	GLY	C	729	117.453	68.659	148.250	1.00	0.00	H
ATOM	7765	HA3	GLY	C	729	116.416	67.429	147.547	1.00	0.00	H
ATOM	7766	N	PHE	C	730	114.351	69.014	149.211	1.00	30.00	N
ATOM	7767	CA	PHE	C	730	113.439	69.067	150.351	1.00	30.00	C
ATOM	7768	C	PHE	C	730	112.017	69.293	149.827	1.00	30.00	C
ATOM	7769	O	PHE	C	730	111.824	70.043	148.872	1.00	30.00	O
ATOM	7770	CB	PHE	C	730	113.869	70.207	151.306	1.00	20.00	C
ATOM	7771	CG	PHE	C	730	113.411	70.008	152.740	1.00	0.00	C
ATOM	7772	CD1	PHE	C	730	112.088	70.311	153.129	1.00	0.00	C
ATOM	7773	CD2	PHE	C	730	114.254	69.335	153.649	1.00	0.00	C
ATOM	7774	CE1	PHE	C	730	111.648	69.977	154.404	1.00	0.00	C
ATOM	7775	CE2	PHE	C	730	113.804	69.038	154.928	1.00	0.00	C
ATOM	7776	CZ	PHE	C	730	112.506	69.359	155.304	1.00	0.00	C
ATOM	7777	H	PHE	C	730	114.056	69.513	148.383	1.00	0.00	H
ATOM	7778	HA	PHE	C	730	113.474	68.108	150.873	1.00	0.00	H
ATOM	7779	HB2	PHE	C	730	114.956	70.310	151.317	1.00	0.00	H
ATOM	7780	HB3	PHE	C	730	113.494	71.167	150.946	1.00	0.00	H
ATOM	7781	HD1	PHE	C	730	111.406	70.777	152.433	1.00	0.00	H
ATOM	7782	HD2	PHE	C	730	115.253	69.047	153.354	1.00	0.00	H
ATOM	7783	HE1	PHE	C	730	110.630	70.181	154.699	1.00	0.00	H
ATOM	7784	HE2	PHE	C	730	114.458	68.536	155.626	1.00	0.00	H
ATOM	7785	HZ	PHE	C	730	112.156	69.109	156.294	1.00	0.00	H
ATOM	7786	N	THR	C	731	111.036	68.672	150.492	1.00	30.00	N
ATOM	7787	CA	THR	C	731	109.614	68.810	150.185	1.00	30.00	C
ATOM	7788	C	THR	C	731	108.828	68.252	151.392	1.00	30.00	C
ATOM	7789	O	THR	C	731	109.295	67.301	152.021	1.00	30.00	O
ATOM	7790	CB	THR	C	731	109.270	68.034	148.867	1.00	20.00	C
ATOM	7791	CG2	THR	C	731	107.912	67.332	148.728	1.00	0.00	C
ATOM	7792	OG1	THR	C	731	109.349	68.931	147.787	1.00	0.00	O
ATOM	7793	H	THR	C	731	111.255	68.062	151.268	1.00	0.00	H
ATOM	7794	HA	THR	C	731	109.416	69.875	150.082	1.00	0.00	H
ATOM	7795	HB	THR	C	731	110.032	67.278	148.689	1.00	0.00	H
ATOM	7796	HG1	THR	C	731	110.255	69.249	147.711	1.00	0.00	H
ATOM	7797	HG21	THR	C	731	107.790	66.908	147.732	1.00	0.00	H
ATOM	7798	HG22	THR	C	731	107.849	66.496	149.413	1.00	0.00	H
ATOM	7799	HG23	THR	C	731	107.069	67.998	148.902	1.00	0.00	H
ATOM	7800	N	PRO	C	732	107.655	68.850	151.716	1.00	30.00	N

```
ATOM   7801  CA  PRO C 732     106.790  68.353 152.808  1.00 30.00           C
ATOM   7802  C   PRO C 732     106.242  66.925 152.602  1.00 30.00           C
ATOM   7803  O   PRO C 732     105.999  66.224 153.583  1.00 30.00           O
ATOM   7804  CB  PRO C 732     105.656  69.390 152.875  1.00 20.00           C
ATOM   7805  CG  PRO C 732     105.596  70.002 151.484  1.00 20.00           C
ATOM   7806  CD  PRO C 732     107.054  70.009 151.047  1.00 20.00           C
ATOM   7807  HA  PRO C 732     107.345  68.361 153.748  1.00  0.00           H
ATOM   7808  HB2 PRO C 732     104.695  68.977 153.186  1.00  0.00           H
ATOM   7809  HB3 PRO C 732     105.918  70.160 153.596  1.00  0.00           H
ATOM   7810  HG2 PRO C 732     105.021  69.348 150.826  1.00  0.00           H
ATOM   7811  HG3 PRO C 732     105.134  70.989 151.464  1.00  0.00           H
ATOM   7812  HD2 PRO C 732     107.131  69.982 149.962  1.00  0.00           H
ATOM   7813  HD3 PRO C 732     107.552  70.914 151.394  1.00  0.00           H
ATOM   7814  N   ASP C 733     106.084  66.531 151.330  1.00 30.00           N
ATOM   7815  CA  ASP C 733     105.571  65.240 150.866  1.00 30.00           C
ATOM   7816  C   ASP C 733     106.634  64.115 150.851  1.00 30.00           C
ATOM   7817  O   ASP C 733     106.242  62.957 150.710  1.00 30.00           O
ATOM   7818  CB  ASP C 733     104.917  65.343 149.459  1.00 20.00           C
ATOM   7819  CG  ASP C 733     104.038  66.579 149.207  1.00  0.00           C
ATOM   7820  OD1 ASP C 733     103.403  67.062 150.171  1.00  0.00           O
ATOM   7821  OD2 ASP C 733     103.965  66.981 148.025  1.00  0.00           O1-
ATOM   7822  H   ASP C 733     106.287  67.195 150.597  1.00  0.00           H
ATOM   7823  HA  ASP C 733     104.793  64.924 151.564  1.00  0.00           H
ATOM   7824  HB2 ASP C 733     105.708  65.350 148.707  1.00  0.00           H
ATOM   7825  HB3 ASP C 733     104.309  64.458 149.267  1.00  0.00           H
ATOM   7826  N   GLY C 734     107.935  64.447 150.974  1.00 30.00           N
ATOM   7827  CA  GLY C 734     109.035  63.486 150.860  1.00 30.00           C
ATOM   7828  C   GLY C 734     110.232  64.119 150.131  1.00 30.00           C
ATOM   7829  O   GLY C 734     110.485  65.315 150.251  1.00 30.00           O
ATOM   7830  H   GLY C 734     108.193  65.414 151.112  1.00  0.00           H
ATOM   7831  HA2 GLY C 734     109.347  63.183 151.860  1.00  0.00           H
ATOM   7832  HA3 GLY C 734     108.726  62.584 150.329  1.00  0.00           H
ATOM   7833  N   LYS C 735     111.004  63.279 149.419  1.00 30.00           N
ATOM   7834  CA  LYS C 735     112.235  63.626 148.691  1.00 30.00           C
ATOM   7835  C   LYS C 735     111.993  64.550 147.478  1.00 30.00           C
ATOM   7836  O   LYS C 735     110.908  64.517 146.895  1.00 30.00           O
ATOM   7837  CB  LYS C 735     112.919  62.297 148.287  1.00 20.00           C
ATOM   7838  CG  LYS C 735     114.246  62.432 147.514  1.00  0.00           C
ATOM   7839  CD  LYS C 735     114.941  61.083 147.290  1.00  0.00           C
ATOM   7840  CE  LYS C 735     116.257  61.236 146.516  1.00  0.00           C
ATOM   7841  NZ  LYS C 735     116.919  59.937 146.313  1.00  0.00           N1+
ATOM   7842  H   LYS C 735     110.740  62.306 149.374  1.00  0.00           H
ATOM   7843  HA  LYS C 735     112.890  64.153 149.388  1.00  0.00           H
ATOM   7844  HB2 LYS C 735     113.109  61.722 149.194  1.00  0.00           H
ATOM   7845  HB3 LYS C 735     112.228  61.698 147.691  1.00  0.00           H
ATOM   7846  HG2 LYS C 735     114.065  62.885 146.540  1.00  0.00           H
ATOM   7847  HG3 LYS C 735     114.917  63.104 148.052  1.00  0.00           H
ATOM   7848  HD2 LYS C 735     115.135  60.609 148.253  1.00  0.00           H
ATOM   7849  HD3 LYS C 735     114.270  60.416 146.746  1.00  0.00           H
ATOM   7850  HE2 LYS C 735     116.072  61.690 145.542  1.00  0.00           H
ATOM   7851  HE3 LYS C 735     116.938  61.891 147.061  1.00  0.00           H
ATOM   7852  HZ1 LYS C 735     116.312  59.325 145.787  1.00  0.00           H
ATOM   7853  HZ2 LYS C 735     117.779  60.076 145.802  1.00  0.00           H
ATOM   7854  HZ3 LYS C 735     117.125  59.519 147.209  1.00  0.00           H
ATOM   7855  N   ASP C 736     113.031  65.322 147.097  1.00 30.00           N
ATOM   7856  CA  ASP C 736     112.993  66.207 145.930  1.00 30.00           C
ATOM   7857  C   ASP C 736     114.398  66.421 145.314  1.00 30.00           C
ATOM   7858  O   ASP C 736     114.679  67.500 144.789  1.00 30.00           O
ATOM   7859  CB  ASP C 736     112.230  67.521 146.245  1.00 20.00           C
ATOM   7860  CG  ASP C 736     111.589  68.209 145.035  1.00  0.00           C
ATOM   7861  OD1 ASP C 736     111.793  67.739 143.895  1.00  0.00           O
ATOM   7862  OD2 ASP C 736     110.854  69.189 145.275  1.00  0.00           O1-
ATOM   7863  H   ASP C 736     113.892  65.311 147.625  1.00  0.00           H
ATOM   7864  HA  ASP C 736     112.447  65.664 145.155  1.00  0.00           H
ATOM   7865  HB2 ASP C 736     111.419  67.292 146.931  1.00  0.00           H
ATOM   7866  HB3 ASP C 736     112.893  68.221 146.753  1.00  0.00           H
ATOM   7867  N   ASP C 737     115.245  65.375 145.335  1.00 30.00           N
ATOM   7868  CA  ASP C 737     116.508  65.301 144.581  1.00 30.00           C
ATOM   7869  C   ASP C 737     116.307  64.597 143.224  1.00 30.00           C
ATOM   7870  O   ASP C 737     117.103  63.724 142.876  1.00 30.00           O
ATOM   7871  CB  ASP C 737     117.679  64.663 145.369  1.00 20.00           C
```

ATOM	7872	CG	ASP	C	737	117.975	65.317	146.712	1.00	0.00	C
ATOM	7873	OD1	ASP	C	737	118.593	66.403	146.680	1.00	0.00	O
ATOM	7874	OD2	ASP	C	737	117.607	64.708	147.740	1.00	0.00	O1-
ATOM	7875	H	ASP	C	737	114.964	64.525	145.801	1.00	0.00	H
ATOM	7876	HA	ASP	C	737	116.834	66.315	144.350	1.00	0.00	H
ATOM	7877	HB2	ASP	C	737	117.497	63.603	145.537	1.00	0.00	H
ATOM	7878	HB3	ASP	C	737	118.604	64.686	144.792	1.00	0.00	H
ATOM	7879	N	TYR	C	738	115.252	64.968	142.474	1.00	30.00	N
ATOM	7880	CA	TYR	C	738	114.936	64.365	141.172	1.00	30.00	C
ATOM	7881	C	TYR	C	738	113.916	65.160	140.330	1.00	30.00	C
ATOM	7882	O	TYR	C	738	113.585	64.682	139.244	1.00	30.00	O
ATOM	7883	CB	TYR	C	738	114.507	62.879	141.339	1.00	20.00	C
ATOM	7884	CG	TYR	C	738	113.177	62.637	142.037	1.00	0.00	C
ATOM	7885	CD1	TYR	C	738	113.075	62.763	143.439	1.00	0.00	C
ATOM	7886	CD2	TYR	C	738	112.039	62.277	141.286	1.00	0.00	C
ATOM	7887	CE1	TYR	C	738	111.839	62.558	144.081	1.00	0.00	C
ATOM	7888	CE2	TYR	C	738	110.804	62.067	141.928	1.00	0.00	C
ATOM	7889	CZ	TYR	C	738	110.701	62.218	143.325	1.00	0.00	C
ATOM	7890	OH	TYR	C	738	109.498	62.040	143.943	1.00	0.00	O
ATOM	7891	H	TYR	C	738	114.623	65.681	142.815	1.00	0.00	H
ATOM	7892	HA	TYR	C	738	115.855	64.380	140.583	1.00	0.00	H
ATOM	7893	HB2	TYR	C	738	114.453	62.418	140.351	1.00	0.00	H
ATOM	7894	HB3	TYR	C	738	115.270	62.302	141.859	1.00	0.00	H
ATOM	7895	HD1	TYR	C	738	113.941	63.022	144.028	1.00	0.00	H
ATOM	7896	HD2	TYR	C	738	112.105	62.173	140.213	1.00	0.00	H
ATOM	7897	HE1	TYR	C	738	111.770	62.664	145.153	1.00	0.00	H
ATOM	7898	HE2	TYR	C	738	109.933	61.803	141.347	1.00	0.00	H
ATOM	7899	HH	TYR	C	738	109.534	62.190	144.890	1.00	0.00	H
ATOM	7900	N	ARG	C	739	113.420	66.320	140.804	1.00	30.00	N
ATOM	7901	CA	ARG	C	739	112.374	67.094	140.116	1.00	30.00	C
ATOM	7902	C	ARG	C	739	112.737	68.583	139.989	1.00	30.00	C
ATOM	7903	O	ARG	C	739	113.780	69.015	140.479	1.00	30.00	O
ATOM	7904	CB	ARG	C	739	110.991	66.935	140.803	1.00	20.00	C
ATOM	7905	CG	ARG	C	739	110.674	65.551	141.402	1.00	0.00	C
ATOM	7906	CD	ARG	C	739	109.309	65.499	142.103	1.00	0.00	C
ATOM	7907	NE	ARG	C	739	109.288	66.362	143.291	1.00	0.00	N
ATOM	7908	CZ	ARG	C	739	108.253	66.535	144.129	1.00	0.00	C
ATOM	7909	NH1	ARG	C	739	107.124	65.824	144.001	1.00	0.00	N
ATOM	7910	NH2	ARG	C	739	108.352	67.444	145.105	1.00	0.00	N1+
ATOM	7911	H	ARG	C	739	113.741	66.701	141.682	1.00	0.00	H
ATOM	7912	HA	ARG	C	739	112.270	66.745	139.087	1.00	0.00	H
ATOM	7913	HB2	ARG	C	739	110.899	67.680	141.590	1.00	0.00	H
ATOM	7914	HB3	ARG	C	739	110.212	67.190	140.083	1.00	0.00	H
ATOM	7915	HG2	ARG	C	739	110.747	64.764	140.652	1.00	0.00	H
ATOM	7916	HG3	ARG	C	739	111.429	65.327	142.157	1.00	0.00	H
ATOM	7917	HD2	ARG	C	739	108.549	65.899	141.431	1.00	0.00	H
ATOM	7918	HD3	ARG	C	739	109.020	64.474	142.333	1.00	0.00	H
ATOM	7919	HE	ARG	C	739	110.132	66.907	143.454	1.00	0.00	H
ATOM	7920	HH11	ARG	C	739	106.353	65.966	144.637	1.00	0.00	H
ATOM	7921	HH12	ARG	C	739	107.046	65.132	143.271	1.00	0.00	H
ATOM	7922	HH21	ARG	C	739	107.587	67.609	145.744	1.00	0.00	H
ATOM	7923	HH22	ARG	C	739	109.190	68.010	145.201	1.00	0.00	H
ATOM	7924	N	TRP	C	740	111.834	69.331	139.327	1.00	30.00	N
ATOM	7925	CA	TRP	C	740	111.875	70.779	139.092	1.00	30.00	C
ATOM	7926	C	TRP	C	740	113.011	71.263	138.168	1.00	30.00	C
ATOM	7927	O	TRP	C	740	113.381	72.432	138.238	1.00	30.00	O
ATOM	7928	CB	TRP	C	740	111.736	71.582	140.405	1.00	20.00	C
ATOM	7929	CG	TRP	C	740	110.430	71.452	141.128	1.00	0.00	C
ATOM	7930	CD1	TRP	C	740	110.198	70.672	142.207	1.00	0.00	C
ATOM	7931	CD2	TRP	C	740	109.153	72.090	140.817	1.00	0.00	C
ATOM	7932	CE2	TRP	C	740	108.213	71.734	141.832	1.00	0.00	C
ATOM	7933	CE3	TRP	C	740	108.689	72.943	139.787	1.00	0.00	C
ATOM	7934	NE1	TRP	C	740	108.903	70.854	142.639	1.00	0.00	N
ATOM	7935	CZ2	TRP	C	740	106.904	72.247	141.858	1.00	0.00	C
ATOM	7936	CZ3	TRP	C	740	107.379	73.463	139.800	1.00	0.00	C
ATOM	7937	CH2	TRP	C	740	106.491	73.126	140.840	1.00	0.00	C
ATOM	7938	H	TRP	C	740	111.009	68.871	138.971	1.00	0.00	H
ATOM	7939	HA	TRP	C	740	110.980	70.997	138.509	1.00	0.00	H
ATOM	7940	HB2	TRP	C	740	112.524	71.310	141.103	1.00	0.00	H
ATOM	7941	HB3	TRP	C	740	111.863	72.648	140.216	1.00	0.00	H
ATOM	7942	HD1	TRP	C	740	110.935	70.031	142.666	1.00	0.00	H

```
ATOM   7943  HE1 TRP C 740     108.531  70.395 143.461  1.00  0.00           H
ATOM   7944  HE3 TRP C 740     109.350  73.198 138.972  1.00  0.00           H
ATOM   7945  HZ2 TRP C 740     106.223  71.964 142.648  1.00  0.00           H
ATOM   7946  HZ3 TRP C 740     107.054  74.120 139.006  1.00  0.00           H
ATOM   7947  HH2 TRP C 740     105.488  73.527 140.846  1.00  0.00           H
ATOM   7948  N   CYS C 741     113.472  70.367 137.275  1.00 30.00           N
ATOM   7949  CA  CYS C 741     114.115  70.593 135.970  1.00 30.00           C
ATOM   7950  C   CYS C 741     114.939  71.890 135.770  1.00 30.00           C
ATOM   7951  O   CYS C 741     114.657  72.669 134.860  1.00 30.00           O
ATOM   7952  CB  CYS C 741     113.091  70.343 134.841  1.00 20.00           C
ATOM   7953  SG  CYS C 741     111.702  71.518 134.827  1.00 20.00           S
ATOM   7954  H   CYS C 741     113.138  69.422 137.392  1.00  0.00           H
ATOM   7955  HA  CYS C 741     114.849  69.794 135.909  1.00  0.00           H
ATOM   7956  HB2 CYS C 741     112.696  69.328 134.905  1.00  0.00           H
ATOM   7957  HB3 CYS C 741     113.583  70.427 133.879  1.00  0.00           H
ATOM   7958  HG  CYS C 741     112.445  72.607 134.599  1.00  0.00           H
ATOM   7959  N   PHE C 742     115.924  72.103 136.653  1.00 30.00           N
ATOM   7960  CA  PHE C 742     116.599  73.384 136.861  1.00 30.00           C
ATOM   7961  C   PHE C 742     117.405  73.944 135.685  1.00 30.00           C
ATOM   7962  O   PHE C 742     118.177  73.210 135.075  1.00 30.00           O
ATOM   7963  CB  PHE C 742     117.482  73.295 138.118  1.00 20.00           C
ATOM   7964  CG  PHE C 742     116.650  73.312 139.377  1.00  0.00           C
ATOM   7965  CD1 PHE C 742     116.095  72.120 139.878  1.00  0.00           C
ATOM   7966  CD2 PHE C 742     116.216  74.543 139.902  1.00  0.00           C
ATOM   7967  CE1 PHE C 742     115.159  72.171 140.897  1.00  0.00           C
ATOM   7968  CE2 PHE C 742     115.276  74.577 140.914  1.00  0.00           C
ATOM   7969  CZ  PHE C 742     114.728  73.395 141.387  1.00  0.00           C
ATOM   7970  H   PHE C 742     116.096  71.406 137.363  1.00  0.00           H
ATOM   7971  HA  PHE C 742     115.804  74.103 137.062  1.00  0.00           H
ATOM   7972  HB2 PHE C 742     118.088  72.387 138.099  1.00  0.00           H
ATOM   7973  HB3 PHE C 742     118.184  74.129 138.157  1.00  0.00           H
ATOM   7974  HD1 PHE C 742     116.369  71.167 139.448  1.00  0.00           H
ATOM   7975  HD2 PHE C 742     116.577  75.466 139.484  1.00  0.00           H
ATOM   7976  HE1 PHE C 742     114.740  71.254 141.276  1.00  0.00           H
ATOM   7977  HE2 PHE C 742     114.948  75.529 141.303  1.00  0.00           H
ATOM   7978  HZ  PHE C 742     113.950  73.433 142.130  1.00  0.00           H
ATOM   7979  N   ARG C 743     117.261  75.262 135.474  1.00 30.00           N
ATOM   7980  CA  ARG C 743     118.157  76.086 134.667  1.00 30.00           C
ATOM   7981  C   ARG C 743     119.323  76.542 135.560  1.00 30.00           C
ATOM   7982  O   ARG C 743     119.052  77.142 136.598  1.00 30.00           O
ATOM   7983  CB  ARG C 743     117.341  77.283 134.106  1.00 20.00           C
ATOM   7984  CG  ARG C 743     118.129  78.272 133.221  1.00 20.00           C
ATOM   7985  CD  ARG C 743     118.825  79.429 133.963  1.00 20.00           C
ATOM   7986  NE  ARG C 743     119.900  79.996 133.139  1.00 20.00           N
ATOM   7987  CZ  ARG C 743     119.787  80.917 132.172  1.00 20.00           C
ATOM   7988  NH1 ARG C 743     118.628  81.544 131.944  1.00 20.00           N
ATOM   7989  NH2 ARG C 743     120.856  81.205 131.418  1.00 20.00           N1+
ATOM   7990  H   ARG C 743     116.588  75.770 136.032  1.00  0.00           H
ATOM   7991  HA  ARG C 743     118.540  75.498 133.832  1.00  0.00           H
ATOM   7992  HB2 ARG C 743     116.542  76.864 133.496  1.00  0.00           H
ATOM   7993  HB3 ARG C 743     116.842  77.826 134.911  1.00  0.00           H
ATOM   7994  HG2 ARG C 743     118.775  77.776 132.500  1.00  0.00           H
ATOM   7995  HG3 ARG C 743     117.364  78.760 132.615  1.00  0.00           H
ATOM   7996  HD2 ARG C 743     118.116  80.237 134.143  1.00  0.00           H
ATOM   7997  HD3 ARG C 743     119.186  79.143 134.946  1.00  0.00           H
ATOM   7998  HE  ARG C 743     120.808  79.564 133.275  1.00  0.00           H
ATOM   7999 HH11 ARG C 743     118.546  82.222 131.201  1.00  0.00           H
ATOM   8000 HH12 ARG C 743     117.823  81.355 132.524  1.00  0.00           H
ATOM   8001 HH21 ARG C 743     120.793  81.870 130.661  1.00  0.00           H
ATOM   8002 HH22 ARG C 743     121.742  80.756 131.603  1.00  0.00           H
ATOM   8003  N   VAL C 744     120.576  76.303 135.138  1.00 30.00           N
ATOM   8004  CA  VAL C 744     121.775  76.833 135.802  1.00 30.00           C
ATOM   8005  C   VAL C 744     122.101  78.232 135.239  1.00 30.00           C
ATOM   8006  O   VAL C 744     122.158  78.387 134.017  1.00 30.00           O
ATOM   8007  CB  VAL C 744     123.009  75.901 135.602  1.00 20.00           C
ATOM   8008  CG1 VAL C 744     124.348  76.494 136.095  1.00 20.00           C
ATOM   8009  CG2 VAL C 744     122.791  74.535 136.275  1.00 20.00           C
ATOM   8010  H   VAL C 744     120.734  75.774 134.291  1.00  0.00           H
ATOM   8011  HA  VAL C 744     121.590  76.921 136.873  1.00  0.00           H
ATOM   8012  HB  VAL C 744     123.120  75.708 134.535  1.00  0.00           H
ATOM   8013 HG11 VAL C 744     125.149  75.758 136.030  1.00  0.00           H
```

```
ATOM   8014 HG12 VAL C 744     124.666  77.351 135.501  1.00  0.00           H
ATOM   8015 HG13 VAL C 744     124.279  76.814 137.135  1.00  0.00           H
ATOM   8016 HG21 VAL C 744     123.606  73.849 136.041  1.00  0.00           H
ATOM   8017 HG22 VAL C 744     122.752  74.635 137.359  1.00  0.00           H
ATOM   8018 HG23 VAL C 744     121.867  74.059 135.947  1.00  0.00           H
ATOM   8019  N   ASP C 745     122.302  79.210 136.139  1.00 30.00           N
ATOM   8020  CA  ASP C 745     122.547  80.620 135.815  1.00 30.00           C
ATOM   8021  C   ASP C 745     123.749  81.149 136.627  1.00 30.00           C
ATOM   8022  O   ASP C 745     124.227  80.478 137.543  1.00 30.00           O
ATOM   8023  CB  ASP C 745     121.273  81.468 136.099  1.00 20.00           C
ATOM   8024  CG  ASP C 745     121.144  82.764 135.283  1.00  0.00           C
ATOM   8025  OD1 ASP C 745     121.845  82.884 134.255  1.00  0.00           O
ATOM   8026  OD2 ASP C 745     120.295  83.594 135.673  1.00  0.00           O1-
ATOM   8027  H   ASP C 745     122.240  79.000 137.127  1.00  0.00           H
ATOM   8028  HA  ASP C 745     122.831  80.689 134.764  1.00  0.00           H
ATOM   8029  HB2 ASP C 745     120.383  80.871 135.909  1.00  0.00           H
ATOM   8030  HB3 ASP C 745     121.253  81.737 137.157  1.00  0.00           H
ATOM   8031  N   GLU C 746     124.197  82.365 136.272  1.00 30.00           N
ATOM   8032  CA  GLU C 746     125.272  83.113 136.925  1.00 30.00           C
ATOM   8033  C   GLU C 746     124.871  84.592 137.080  1.00 30.00           C
ATOM   8034  O   GLU C 746     124.022  85.085 136.335  1.00 30.00           O
ATOM   8035  CB  GLU C 746     126.587  82.923 136.136  1.00 20.00           C
ATOM   8036  CG  GLU C 746     126.584  83.492 134.698  1.00  0.00           C
ATOM   8037  CD  GLU C 746     127.886  83.250 133.928  1.00  0.00           C
ATOM   8038  OE1 GLU C 746     128.869  82.786 134.548  1.00  0.00           O
ATOM   8039  OE2 GLU C 746     127.875  83.546 132.713  1.00  0.00           O1-
ATOM   8040  H   GLU C 746     123.730  82.853 135.517  1.00  0.00           H
ATOM   8041  HA  GLU C 746     125.425  82.721 137.929  1.00  0.00           H
ATOM   8042  HB2 GLU C 746     127.399  83.383 136.702  1.00  0.00           H
ATOM   8043  HB3 GLU C 746     126.823  81.858 136.104  1.00  0.00           H
ATOM   8044  HG2 GLU C 746     125.768  83.052 134.124  1.00  0.00           H
ATOM   8045  HG3 GLU C 746     126.409  84.568 134.714  1.00  0.00           H
ATOM   8046  N   VAL C 747     125.491  85.262 138.066  1.00 30.00           N
ATOM   8047  CA  VAL C 747     125.230  86.656 138.443  1.00 30.00           C
ATOM   8048  C   VAL C 747     126.578  87.376 138.678  1.00 30.00           C
ATOM   8049  O   VAL C 747     127.576  86.718 138.964  1.00 30.00           O
ATOM   8050  CB  VAL C 747     124.356  86.716 139.738  1.00 20.00           C
ATOM   8051  CG1 VAL C 747     124.124  88.123 140.326  1.00  0.00           C
ATOM   8052  CG2 VAL C 747     122.981  86.047 139.530  1.00  0.00           C
ATOM   8053  H   VAL C 747     126.198  84.793 138.614  1.00  0.00           H
ATOM   8054  HA  VAL C 747     124.715  87.176 137.633  1.00  0.00           H
ATOM   8055  HB  VAL C 747     124.873  86.147 140.510  1.00  0.00           H
ATOM   8056 HG11 VAL C 747     123.400  88.096 141.140  1.00  0.00           H
ATOM   8057 HG12 VAL C 747     125.032  88.548 140.747  1.00  0.00           H
ATOM   8058 HG13 VAL C 747     123.743  88.811 139.571  1.00  0.00           H
ATOM   8059 HG21 VAL C 747     122.372  86.102 140.433  1.00  0.00           H
ATOM   8060 HG22 VAL C 747     122.426  86.529 138.725  1.00  0.00           H
ATOM   8061 HG23 VAL C 747     123.072  84.990 139.282  1.00  0.00           H
ATOM   8062  N   ASN C 748     126.591  88.713 138.538  1.00 30.00           N
ATOM   8063  CA  ASN C 748     127.769  89.577 138.725  1.00 30.00           C
ATOM   8064  C   ASN C 748     128.118  89.809 140.212  1.00 30.00           C
ATOM   8065  O   ASN C 748     127.414  89.326 141.098  1.00 30.00           O
ATOM   8066  CB  ASN C 748     127.538  90.926 137.998  1.00 20.00           C
ATOM   8067  CG  ASN C 748     127.250  90.775 136.500  1.00  0.00           C
ATOM   8068  ND2 ASN C 748     128.246  90.345 135.722  1.00  0.00           N
ATOM   8069  OD1 ASN C 748     126.139  91.045 136.052  1.00  0.00           O
ATOM   8070  H   ASN C 748     125.733  89.189 138.298  1.00  0.00           H
ATOM   8071  HA  ASN C 748     128.632  89.083 138.274  1.00  0.00           H
ATOM   8072  HB2 ASN C 748     126.707  91.460 138.462  1.00  0.00           H
ATOM   8073  HB3 ASN C 748     128.410  91.574 138.098  1.00  0.00           H
ATOM   8074 HD21 ASN C 748     128.095  90.232 134.730  1.00  0.00           H
ATOM   8075 HD22 ASN C 748     129.149  90.121 136.114  1.00  0.00           H
ATOM   8076  N   TRP C 749     129.219  90.546 140.451  1.00 30.00           N
ATOM   8077  CA  TRP C 749     129.761  90.872 141.774  1.00 30.00           C
ATOM   8078  C   TRP C 749     128.797  91.682 142.658  1.00 30.00           C
ATOM   8079  O   TRP C 749     128.322  91.154 143.662  1.00 30.00           O
ATOM   8080  CB  TRP C 749     131.132  91.576 141.651  1.00 20.00           C
ATOM   8081  CG  TRP C 749     132.310  90.738 141.239  1.00  0.00           C
ATOM   8082  CD1 TRP C 749     132.339  89.809 140.253  1.00  0.00           C
ATOM   8083  CD2 TRP C 749     133.668  90.778 141.781  1.00  0.00           C
ATOM   8084  CE2 TRP C 749     134.461  89.810 141.094  1.00  0.00           C
```

```
ATOM   8085  CE3 TRP C 749     134.311  91.526 142.797  1.00  0.00           C
ATOM   8086  NE1 TRP C 749     133.597  89.251 140.176  1.00  0.00           N
ATOM   8087  CZ2 TRP C 749     135.816  89.590 141.401  1.00  0.00           C
ATOM   8088  CZ3 TRP C 749     135.669  91.315 143.114  1.00  0.00           C
ATOM   8089  CH2 TRP C 749     136.421  90.348 142.420  1.00  0.00           C
ATOM   8090  H   TRP C 749     129.744  90.904 139.668  1.00  0.00           H
ATOM   8091  HA  TRP C 749     129.927  89.931 142.297  1.00  0.00           H
ATOM   8092  HB2 TRP C 749     131.063  92.420 140.964  1.00  0.00           H
ATOM   8093  HB3 TRP C 749     131.396  92.004 142.619  1.00  0.00           H
ATOM   8094  HD1 TRP C 749     131.499  89.534 139.633  1.00  0.00           H
ATOM   8095  HE1 TRP C 749     133.832  88.525 139.514  1.00  0.00           H
ATOM   8096  HE3 TRP C 749     133.750  92.268 143.347  1.00  0.00           H
ATOM   8097  HZ2 TRP C 749     136.385  88.848 140.861  1.00  0.00           H
ATOM   8098  HZ3 TRP C 749     136.134  91.896 143.896  1.00  0.00           H
ATOM   8099  HH2 TRP C 749     137.460  90.190 142.667  1.00  0.00           H
ATOM   8100  N   THR C 750     128.550  92.949 142.274  1.00 30.00           N
ATOM   8101  CA  THR C 750     127.675  93.927 142.946  1.00 30.00           C
ATOM   8102  C   THR C 750     128.109  94.332 144.384  1.00 30.00           C
ATOM   8103  O   THR C 750     127.335  94.986 145.082  1.00 30.00           O
ATOM   8104  CB  THR C 750     126.168  93.509 142.926  1.00 20.00           C
ATOM   8105  CG2 THR C 750     125.679  92.967 141.572  1.00  0.00           C
ATOM   8106  OG1 THR C 750     125.804  92.610 143.958  1.00  0.00           O
ATOM   8107  H   THR C 750     128.989  93.285 141.429  1.00  0.00           H
ATOM   8108  HA  THR C 750     127.763  94.839 142.355  1.00  0.00           H
ATOM   8109  HB  THR C 750     125.587  94.413 143.116  1.00  0.00           H
ATOM   8110  HG1 THR C 750     126.338  91.814 143.868  1.00  0.00           H
ATOM   8111 HG21 THR C 750     124.599  92.814 141.581  1.00  0.00           H
ATOM   8112 HG22 THR C 750     125.904  93.665 140.765  1.00  0.00           H
ATOM   8113 HG23 THR C 750     126.139  92.011 141.324  1.00  0.00           H
ATOM   8114  N   THR C 751     129.349  93.973 144.780  1.00 30.00           N
ATOM   8115  CA  THR C 751     130.011  94.172 146.085  1.00 30.00           C
ATOM   8116  C   THR C 751     129.416  93.188 147.080  1.00 30.00           C
ATOM   8117  O   THR C 751     129.719  93.185 148.273  1.00 30.00           O
ATOM   8118  CB  THR C 751     130.111  95.648 146.603  1.00 20.00           C
ATOM   8119  CG2 THR C 751     130.619  96.645 145.548  1.00  0.00           C
ATOM   8120  OG1 THR C 751     128.895  96.148 147.132  1.00  0.00           O
ATOM   8121  H   THR C 751     129.903  93.455 144.114  1.00  0.00           H
ATOM   8122  HA  THR C 751     131.036  93.844 145.911  1.00  0.00           H
ATOM   8123  HB  THR C 751     130.822  95.664 147.431  1.00  0.00           H
ATOM   8124  HG1 THR C 751     128.215  96.064 146.456  1.00  0.00           H
ATOM   8125 HG21 THR C 751     130.747  97.639 145.979  1.00  0.00           H
ATOM   8126 HG22 THR C 751     131.584  96.333 145.150  1.00  0.00           H
ATOM   8127 HG23 THR C 751     129.927  96.737 144.710  1.00  0.00           H
ATOM   8128  HXT THR C 751     128.706  92.466 146.675  1.00  0.00           H
END
```

S2. Series B

Coordinates of compounds from Series B in Sdf file.

```
B_5aR
                   3D
 Structure written by MMmdl.
 66 67  0  0  1  0            999 V2000
  111.1304   92.9363  109.7991 S   0  0  0  0  0  0
  109.7072   92.6555  109.5820 O   0  0  0  0  0  0
  111.9891   91.9435  110.4564 O   0  0  0  0  0  0
  103.7886  102.1175  100.0899 C   0  0  0  0  0  0
  103.8360  101.1286  101.0708 C   0  0  0  0  0  0
  104.8948  100.2253  101.0878 C   0  0  0  0  0  0
  105.9025  100.3188  100.1241 C   0  0  0  0  0  0
  105.8557  101.3198   99.1516 C   0  0  0  0  0  0
  104.7930  102.2176   99.1293 C   0  0  0  0  0  0
  107.0550   99.3239  100.1376 C   0  0  0  0  0  0
  106.7259   97.9680   99.4645 C   0  0  2  0  0  0
  107.8743   96.9335   99.6329 C   0  0  0  0  0  0
  106.3213   98.1424   97.9808 C   0  0  0  0  0  0
  106.2406   96.8834   97.3152 O   0  0  0  0  0  0
  105.4199   96.6799   96.2584 C   0  0  0  0  0  0
  105.5849   95.2931   95.5674 C   0  0  0  0  0  0
  104.5973   97.4984   95.8440 O   0  0  0  0  0  0
  104.2081   94.5839   95.4951 C   0  0  0  0  0  0
  106.1079   95.5311   94.1293 C   0  0  0  0  0  0
  106.5769   94.3549   96.3080 C   0  0  0  0  0  0
  108.3589   96.6838  101.0030 N   0  0  0  0  0  0
  107.7649   95.8550  101.9131 C   0  0  0  0  0  0
  108.6559   95.4240  102.8572 N   0  0  0  0  0  0
  106.1512   95.3925  101.8547 S   0  0  0  0  0  0
  108.3437   94.4133  103.8775 C   0  0  0  0  0  0
  109.4163   94.1810  104.9348 C   0  0  0  0  0  0
  109.8566   95.2172  105.7681 C   0  0  0  0  0  0
  110.6727   94.9331  106.8657 C   0  0  0  0  0  0
  111.0780   93.6192  107.1053 C   0  0  0  0  0  0
  110.7183   92.6062  106.2157 C   0  0  0  0  0  0
  109.8787   92.8806  105.1421 C   0  0  0  0  0  0
  111.8560   93.2997  108.2246 N   0  0  0  0  0  0
  111.3189   94.5690  110.7053 C   0  0  0  0  0  0
  102.9655  102.8156  100.0751 H   0  0  0  0  0  0
  103.0562  101.0620  101.8163 H   0  0  0  0  0  0
  104.9303   99.4513  101.8401 H   0  0  0  0  0  0
  106.6413  101.4020   98.4166 H   0  0  0  0  0  0
  104.7488  102.9903   98.3725 H   0  0  0  0  0  0
  107.9176   99.7825   99.6570 H   0  0  0  0  0  0
  107.3470   99.1748  101.1770 H   0  0  0  0  0  0
  105.8421   97.5710   99.9622 H   0  0  0  0  0  0
  107.5938   95.9776   99.1849 H   0  0  0  0  0  0
  108.7302   97.2745   99.0504 H   0  0  0  0  0  0
  105.3688   98.6724   97.9291 H   0  0  0  0  0  0
  107.0561   98.7593   97.4636 H   0  0  0  0  0  0
  104.2808   93.6228   94.9819 H   0  0  0  0  0  0
  103.4710   95.1768   94.9485 H   0  0  0  0  0  0
  103.8010   94.3888   96.4878 H   0  0  0  0  0  0
  106.2552   94.5936   93.5958 H   0  0  0  0  0  0
  107.0679   96.0486   94.1296 H   0  0  0  0  0  0
  105.4147   96.1286   93.5356 H   0  0  0  0  0  0
  106.6556   93.3930   95.8056 H   0  0  0  0  0  0
  106.2615   94.1483   97.3326 H   0  0  0  0  0  0
  107.5827   94.7754   96.3527 H   0  0  0  0  0  0
  109.2648   97.0644  101.2333 H   0  0  0  0  0  0
```

```
 109.6348   95.5211  102.6234 H   0  0  0  0  0  0
 107.4299   94.6905  104.4080 H   0  0  0  0  0  0
 108.1308   93.4730  103.3655 H   0  0  0  0  0  0
 109.5056   96.2276  105.6163 H   0  0  0  0  0  0
 110.9623   95.7261  107.5405 H   0  0  0  0  0  0
 111.0557   91.5922  106.3848 H   0  0  0  0  0  0
 109.5567   92.0743  104.4980 H   0  0  0  0  0  0
 112.7880   92.9402  108.0667 H   0  0  0  0  0  0
 111.0556   94.4252  111.7519 H   0  0  0  0  0  0
 110.6564   95.3053  110.2541 H   0  0  0  0  0  0
 112.3520   94.8973  110.6287 H   0  0  0  0  0  0
 1  2  2  0  0  0
 1  3  2  0  0  0
 1 32  1  0  0  0
 1 33  1  0  0  0
 4  5  2  0  0  0
 4  9  1  0  0  0
 4 34  1  0  0  0
 5  6  1  0  0  0
 5 35  1  0  0  0
 6  7  2  0  0  0
 6 36  1  0  0  0
 7  8  1  0  0  0
 7 10  1  0  0  0
 8  9  2  0  0  0
 8 37  1  0  0  0
 9 38  1  0  0  0
10 11  1  0  0  0
10 39  1  0  0  0
10 40  1  0  0  0
11 12  1  0  0  0
11 13  1  0  0  0
11 41  1  0  0  0
12 21  1  0  0  0
12 42  1  0  0  0
12 43  1  0  0  0
13 14  1  0  0  0
13 44  1  0  0  0
13 45  1  0  0  0
14 15  1  0  0  0
15 16  1  0  0  0
15 17  2  0  0  0
16 18  1  0  0  0
16 19  1  0  0  0
16 20  1  0  0  0
18 46  1  0  0  0
18 47  1  0  0  0
18 48  1  0  0  0
19 49  1  0  0  0
19 50  1  0  0  0
19 51  1  0  0  0
20 52  1  0  0  0
20 53  1  0  0  0
20 54  1  0  0  0
21 22  1  0  0  0
21 55  1  0  0  0
22 23  1  0  0  0
22 24  2  0  0  0
23 25  1  0  0  0
23 56  1  0  0  0
25 26  1  0  0  0
25 57  1  0  0  0
25 58  1  0  0  0
26 27  2  0  0  0
26 31  1  0  0  0
27 28  1  0  0  0
27 59  1  0  0  0
28 29  2  0  0  0
28 60  1  0  0  0
29 30  1  0  0  0
29 32  1  0  0  0
```

```
 30 31  2  0  0  0
 30 61  1  0  0  0
 31 62  1  0  0  0
 32 63  1  0  0  0
 33 64  1  0  0  0
 33 65  1  0  0  0
 33 66  1  0  0  0
M  END
> <s_m_entry_id>
33

> <s_m_entry_name>
B_5aR.1

> <s_m_Source_Path>
C:\Users\julio\OneDrive - Universidad de Talca\Escritorio\Nueva carpeta

> <s_m_Source_File>
B_5aR.mol2

> <i_m_Source_File_Index>
1

$$$$
B_5aS
                    3D
 Structure written by MMmdl.
 66 67  0  0  1  0            999 V2000
  111.1523   92.9961  109.5780 S   0  0  0  0  0  0
  109.7584   92.5951  109.3585 O   0  0  0  0  0  0
  112.0826   92.0983  110.2673 O   0  0  0  0  0  0
  104.1291   94.1759   95.5251 C   0  0  0  0  0  0
  105.4831   93.8636   95.4245 C   0  0  0  0  0  0
  106.3957   94.4508   96.2960 C   0  0  0  0  0  0
  105.9619   95.3450   97.2822 C   0  0  0  0  0  0
  104.5950   95.6393   97.3886 C   0  0  0  0  0  0
  103.6832   95.0604   96.5037 C   0  0  0  0  0  0
  107.0125   95.9418   98.2286 C   0  0  0  0  0  0
  106.6238   97.1682   99.1028 C   0  0  1  0  0  0
  107.7923   97.6062  100.0346 C   0  0  0  0  0  0
  106.1303   98.3662   98.2541 C   0  0  0  0  0  0
  105.8369   99.4654   99.1039 O   0  0  0  0  0  0
  105.0243  100.4701   98.7189 C   0  0  0  0  0  0
  104.9238  101.5933   99.7899 C   0  0  0  0  0  0
  104.4058  100.5108   97.6530 O   0  0  0  0  0  0
  106.3413  102.1421  100.0920 C   0  0  0  0  0  0
  104.0345  102.7615   99.2959 C   0  0  0  0  0  0
  104.3089  101.0049  101.0849 C   0  0  0  0  0  0
  108.3001   96.5817  100.9566 N   0  0  0  0  0  0
  107.7129   96.2043  102.1292 C   0  0  0  0  0  0
  108.4404   95.2231  102.7288 N   0  0  0  0  0  0
  106.2897   96.8589  102.7443 S   0  0  0  0  0  0
  108.0705   94.4625  103.9259 C   0  0  0  0  0  0
  109.2376   94.2164  104.8721 C   0  0  0  0  0  0
  109.7321   95.2667  105.6524 C   0  0  0  0  0  0
  110.6374   95.0019  106.6813 C   0  0  0  0  0  0
  111.0560   93.6921  106.9095 C   0  0  0  0  0  0
  110.6534   92.6631  106.0568 C   0  0  0  0  0  0
  109.7280   92.9230  105.0515 C   0  0  0  0  0  0
  111.8556   93.3821  108.0081 N   0  0  0  0  0  0
  111.2017   94.6599  110.4274 C   0  0  0  0  0  0
  103.4216   93.7252   94.8429 H   0  0  0  0  0  0
  105.8243   93.1688   94.6659 H   0  0  0  0  0  0
  107.4444   94.1966   96.2099 H   0  0  0  0  0  0
  104.2266   96.3110   98.1456 H   0  0  0  0  0  0
  102.6316   95.2945   96.5784 H   0  0  0  0  0  0
  107.3307   95.1264   98.8722 H   0  0  0  0  0  0
  107.8882   96.2079   97.6291 H   0  0  0  0  0  0
  105.7973   96.8732   99.7540 H   0  0  0  0  0  0
  108.6331   97.9188   99.4168 H   0  0  0  0  0  0
  107.5208   98.4840  100.6191 H   0  0  0  0  0  0
```

```
 106.8952   98.6728   97.5387 H   0  0  0  0  0  0
 105.2465   98.0946   97.6802 H   0  0  0  0  0  0
 106.3010  102.9149  100.8570 H   0  0  0  0  0  0
 106.8018  102.5912   99.2122 H   0  0  0  0  0  0
 107.0221  101.3717  100.4591 H   0  0  0  0  0  0
 103.9250  103.5330  100.0604 H   0  0  0  0  0  0
 103.0261  102.4292   99.0400 H   0  0  0  0  0  0
 104.4554  103.2437   98.4169 H   0  0  0  0  0  0
 104.1656  101.7806  101.8372 H   0  0  0  0  0  0
 104.9476  100.2380  101.5272 H   0  0  0  0  0  0
 103.3298  100.5514  100.9041 H   0  0  0  0  0  0
 109.1832   96.1587  100.7185 H   0  0  0  0  0  0
 109.3174   94.9573  102.2891 H   0  0  0  0  0  0
 107.2766   94.9430  104.5002 H   0  0  0  0  0  0
 107.6589   93.5098  103.5913 H   0  0  0  0  0  0
 109.3700   96.2719  105.5030 H   0  0  0  0  0  0
 110.9656   95.8068  107.3249 H   0  0  0  0  0  0
 111.0021   91.6520  106.2134 H   0  0  0  0  0  0
 109.3523   92.1042  104.4539 H   0  0  0  0  0  0
 112.7948   93.0433  107.8519 H   0  0  0  0  0  0
 110.7171   94.5635  111.3947 H   0  0  0  0  0  0
 110.6596   95.3863  109.8191 H   0  0  0  0  0  0
 112.2393   94.9615  110.5570 H   0  0  0  0  0  0
  1  2  2  0  0  0
  1  3  2  0  0  0
  1 32  1  0  0  0
  1 33  1  0  0  0
  4  5  2  0  0  0
  4  9  1  0  0  0
  4 34  1  0  0  0
  5  6  1  0  0  0
  5 35  1  0  0  0
  6  7  2  0  0  0
  6 36  1  0  0  0
  7  8  1  0  0  0
  7 10  1  0  0  0
  8  9  2  0  0  0
  8 37  1  0  0  0
  9 38  1  0  0  0
 10 11  1  0  0  0
 10 39  1  0  0  0
 10 40  1  0  0  0
 11 12  1  0  0  0
 11 13  1  0  0  0
 11 41  1  0  0  0
 12 21  1  0  0  0
 12 42  1  0  0  0
 12 43  1  0  0  0
 13 14  1  0  0  0
 13 44  1  0  0  0
 13 45  1  0  0  0
 14 15  1  0  0  0
 15 16  1  0  0  0
 15 17  2  0  0  0
 16 18  1  0  0  0
 16 19  1  0  0  0
 16 20  1  0  0  0
 18 46  1  0  0  0
 18 47  1  0  0  0
 18 48  1  0  0  0
 19 49  1  0  0  0
 19 50  1  0  0  0
 19 51  1  0  0  0
 20 52  1  0  0  0
 20 53  1  0  0  0
 20 54  1  0  0  0
 21 22  1  0  0  0
 21 55  1  0  0  0
 22 23  1  0  0  0
 22 24  2  0  0  0
 23 25  1  0  0  0
```

```
 23 56  1  0  0  0
 25 26  1  0  0  0
 25 57  1  0  0  0
 25 58  1  0  0  0
 26 27  2  0  0  0
 26 31  1  0  0  0
 27 28  1  0  0  0
 27 59  1  0  0  0
 28 29  2  0  0  0
 28 60  1  0  0  0
 29 30  1  0  0  0
 29 32  1  0  0  0
 30 31  2  0  0  0
 30 61  1  0  0  0
 31 62  1  0  0  0
 32 63  1  0  0  0
 33 64  1  0  0  0
 33 65  1  0  0  0
 33 66  1  0  0  0
M  END
> <s_m_entry_id>
34

> <s_m_entry_name>
B_5aS.1

> <s_m_Source_Path>
C:\Users\julio\OneDrive - Universidad de Talca\Escritorio\Nueva carpeta

> <s_m_Source_File>
B_5aS.mol2

> <i_m_Source_File_Index>
1

$$$$
B_19
                   3D
 Structure written by MMmdl.
 69 70  0  0  1  0            999 V2000
  111.0473   92.7767  109.9661 S   0  0  0  0  0  0
  109.6236   92.6105  109.6570 O   0  0  0  0  0  0
  111.8384   91.6428  110.4575 O   0  0  0  0  0  0
  103.7514   99.5960   97.6546 C   0  0  0  0  0  0
  105.0526   99.8853   98.0578 C   0  0  0  0  0  0
  105.7199   99.0158   98.9123 C   0  0  0  0  0  0
  105.0722   97.8704   99.3865 C   0  0  0  0  0  0
  103.7653   97.5865   98.9831 C   0  0  0  0  0  0
  103.1056   98.4484   98.1114 C   0  0  0  0  0  0
  105.7667   96.9881  100.4117 C   0  0  0  0  0  0
  106.1596   95.5467   99.9852 C   0  0  2  0  0  0
  106.8926   94.7580  101.1028 C   0  0  0  0  0  0
  106.9427   95.4813   98.6661 C   0  0  0  0  0  0
  106.0431   95.5662   97.5702 O   0  0  0  0  0  0
  106.4675   95.2609   96.3308 C   0  0  0  0  0  0
  105.3488   95.2753   95.2469 C   0  0  0  0  0  0
  107.6381   94.9924   96.0570 O   0  0  0  0  0  0
  104.1985   96.2515   95.6093 C   0  0  0  0  0  0
  105.9298   95.7101   93.8786 C   0  0  0  0  0  0
  104.7749   93.8424   95.1191 C   0  0  0  0  0  0
  108.0999   95.3921  101.6627 N   0  0  0  0  0  0
  108.1556   96.0763  102.8413 C   0  0  0  0  0  0
  109.4419   96.2662  103.2563 N   0  0  0  0  0  0
  106.8005   96.5952  103.6572 S   0  0  0  0  0  0
  109.9047   96.9310  104.4852 C   0  0  1  0  0  0
  110.3433   95.9543  105.5977 C   0  0  0  0  0  0
  110.2084   94.5626  105.4572 C   0  0  0  0  0  0
  110.6641   93.6993  106.4493 C   0  0  0  0  0  0
  111.2736   94.2154  107.5903 C   0  0  0  0  0  0
  111.3771   95.5951  107.7650 C   0  0  0  0  0  0
  110.9050   96.4593  106.7784 C   0  0  0  0  0  0
```

```
 111.8569   93.3421  108.5090 N   0  0  0  0  0  0
 111.2773   94.2176  111.1448 C   0  0  0  0  0  0
 111.0266   97.9005  104.0558 C   0  0  0  0  0  0
 103.2394  100.2695   96.9829 H   0  0  0  0  0  0
 105.5483  100.7757   97.6991 H   0  0  0  0  0  0
 106.7272   99.2511   99.2248 H   0  0  0  0  0  0
 103.2603   96.7062   99.3516 H   0  0  0  0  0  0
 102.0954   98.2299   97.7964 H   0  0  0  0  0  0
 106.6523   97.5168  100.7652 H   0  0  0  0  0  0
 105.0901   96.9310  101.2650 H   0  0  0  0  0  0
 105.2381   94.9925   99.8213 H   0  0  0  0  0  0
 106.1851   94.4934  101.8893 H   0  0  0  0  0  0
 107.2186   93.7927  100.7173 H   0  0  0  0  0  0
 107.7026   96.2631   98.6141 H   0  0  0  0  0  0
 107.4561   94.5209   98.6045 H   0  0  0  0  0  0
 103.4502   96.3009   94.8172 H   0  0  0  0  0  0
 104.5655   97.2675   95.7665 H   0  0  0  0  0  0
 103.6730   95.9442   96.5146 H   0  0  0  0  0  0
 105.1449   95.7999   93.1278 H   0  0  0  0  0  0
 106.6563   94.9933   93.4962 H   0  0  0  0  0  0
 106.4261   96.6788   93.9348 H   0  0  0  0  0  0
 103.9971   93.7944   94.3549 H   0  0  0  0  0  0
 104.3254   93.4986   96.0524 H   0  0  0  0  0  0
 105.5427   93.1170   94.8429 H   0  0  0  0  0  0
 108.9735   95.2149  101.1848 H   0  0  0  0  0  0
 110.1745   95.9157  102.6497 H   0  0  0  0  0  0
 109.1103   97.5452  104.9090 H   0  0  0  0  0  0
 109.7679   94.1270  104.5724 H   0  0  0  0  0  0
 110.5835   92.6315  106.3077 H   0  0  0  0  0  0
 111.8502   96.0006  108.6474 H   0  0  0  0  0  0
 111.0038   97.5243  106.9272 H   0  0  0  0  0  0
 112.8013   93.0184  108.3457 H   0  0  0  0  0  0
 110.9508   93.9059  112.1350 H   0  0  0  0  0  0
 110.6621   95.0513  110.8077 H   0  0  0  0  0  0
 112.3284   94.4972  111.1687 H   0  0  0  0  0  0
 111.3932   98.5280  104.8576 H   0  0  0  0  0  0
 110.6834   98.5855  103.2771 H   0  0  0  0  0  0
 111.8834   97.3565  103.6666 H   0  0  0  0  0  0
  1  2  2  0  0  0
  1  3  2  0  0  0
  1 32  1  0  0  0
  1 33  1  0  0  0
  4  5  2  0  0  0
  4  9  1  0  0  0
  4 35  1  0  0  0
  5  6  1  0  0  0
  5 36  1  0  0  0
  6  7  2  0  0  0
  6 37  1  0  0  0
  7  8  1  0  0  0
  7 10  1  0  0  0
  8  9  2  0  0  0
  8 38  1  0  0  0
  9 39  1  0  0  0
 10 11  1  0  0  0
 10 40  1  0  0  0
 10 41  1  0  0  0
 11 12  1  0  0  0
 11 13  1  0  0  0
 11 42  1  0  0  0
 12 21  1  0  0  0
 12 43  1  0  0  0
 12 44  1  0  0  0
 13 14  1  0  0  0
 13 45  1  0  0  0
 13 46  1  0  0  0
 14 15  1  0  0  0
 15 16  1  0  0  0
 15 17  2  0  0  0
 16 18  1  0  0  0
 16 19  1  0  0  0
```

```
 16 20  1  0  0  0
 18 47  1  0  0  0
 18 48  1  0  0  0
 18 49  1  0  0  0
 19 50  1  0  0  0
 19 51  1  0  0  0
 19 52  1  0  0  0
 20 53  1  0  0  0
 20 54  1  0  0  0
 20 55  1  0  0  0
 21 22  1  0  0  0
 21 56  1  0  0  0
 22 23  1  0  0  0
 22 24  2  0  0  0
 23 25  1  0  0  0
 23 57  1  0  0  0
 25 26  1  0  0  0
 25 34  1  0  0  0
 25 58  1  0  0  0
 26 27  2  0  0  0
 26 31  1  0  0  0
 27 28  1  0  0  0
 27 59  1  0  0  0
 28 29  2  0  0  0
 28 60  1  0  0  0
 29 30  1  0  0  0
 29 32  1  0  0  0
 30 31  2  0  0  0
 30 61  1  0  0  0
 31 62  1  0  0  0
 32 63  1  0  0  0
 33 64  1  0  0  0
 33 65  1  0  0  0
 33 66  1  0  0  0
 34 67  1  0  0  0
 34 68  1  0  0  0
 34 69  1  0  0  0
M  END
> <s_m_entry_id>
35

> <s_m_entry_name>
B_19.1

> <s_m_Source_Path>
C:\Users\julio\OneDrive - Universidad de Talca\Escritorio\Nueva carpeta

> <s_m_Source_File>
B_19.mol2

> <i_m_Source_File_Index>
1

$$$$
B_20
                    3D
 Structure written by MMmdl.
 69 70  0  0  1  0            999 V2000
  111.3411   92.5942  110.3373 S   0  0  0  0  0  0
  109.9373   92.2711  110.0607 O   0  0  0  0  0  0
  112.2557   91.5686  110.8567 O   0  0  0  0  0  0
  106.2641  101.3235  100.5813 C   0  0  0  0  0  0
  106.2552  100.9535   99.2378 C   0  0  0  0  0  0
  106.0819   99.6165   98.8883 C   0  0  0  0  0  0
  105.9069   98.6522   99.8861 C   0  0  0  0  0  0
  105.9225   99.0305  101.2301 C   0  0  0  0  0  0
  106.1021  100.3658  101.5787 C   0  0  0  0  0  0
  105.6513   97.1942   99.5007 C   0  0  0  0  0  0
  106.9288   96.2874   99.4520 C   0  0  1  0  0  0
  106.9192   95.2053  100.5797 C   0  0  0  0  0  0
  107.2990   95.6748   98.0596 C   0  0  0  0  0  0
```

106.3901	95.9740	97.0015	O	0	0	0	0	0	0
105.9309	95.0210	96.1490	C	0	0	0	0	0	0
104.9042	95.5641	95.1039	C	0	0	0	0	0	0
106.2548	93.8325	96.1837	O	0	0	0	0	0	0
103.9963	96.6617	95.7153	C	0	0	0	0	0	0
105.6837	96.1620	93.9063	C	0	0	0	0	0	0
104.0052	94.4148	94.5827	C	0	0	0	0	0	0
107.9784	95.3942	101.5931	N	0	0	0	0	0	0
107.8092	95.6047	102.9272	C	0	0	0	0	0	0
109.0144	95.6318	103.5740	N	0	0	0	0	0	0
106.3172	95.7945	103.6564	S	0	0	0	0	0	0
109.3182	96.3318	104.8403	C	0	0	1	0	0	0
110.0156	95.4633	105.9164	C	0	0	0	0	0	0
110.2414	94.0924	105.7358	C	0	0	0	0	0	0
110.8881	93.3413	106.7164	C	0	0	0	0	0	0
111.3241	93.9480	107.8941	C	0	0	0	0	0	0
111.1017	95.3114	108.0906	C	0	0	0	0	0	0
110.4435	96.0574	107.1117	C	0	0	0	0	0	0
112.0465	93.2032	108.8387	N	0	0	0	0	0	0
111.4561	94.0864	111.4700	C	0	0	0	0	0	0
110.1509	97.5833	104.4941	C	0	0	0	0	0	0
106.3945	102.3631	100.8467	H	0	0	0	0	0	0
106.3899	101.7020	98.4709	H	0	0	0	0	0	0
106.0752	99.3353	97.8454	H	0	0	0	0	0	0
105.7822	98.2931	102.0078	H	0	0	0	0	0	0
106.1100	100.6637	102.6194	H	0	0	0	0	0	0
104.9151	96.8014	100.2058	H	0	0	0	0	0	0
105.0925	97.1900	98.5629	H	0	0	0	0	0	0
107.7712	96.9527	99.6591	H	0	0	0	0	0	0
105.9271	95.1121	101.0186	H	0	0	0	0	0	0
107.0716	94.2096	100.1551	H	0	0	0	0	0	0
108.2776	96.0506	97.7517	H	0	0	0	0	0	0
107.4404	94.5941	98.1552	H	0	0	0	0	0	0
103.2510	97.0083	94.9975	H	0	0	0	0	0	0
104.5565	97.5386	96.0383	H	0	0	0	0	0	0
103.4478	96.2914	96.5849	H	0	0	0	0	0	0
105.0046	96.4757	93.1120	H	0	0	0	0	0	0
106.3809	95.4466	93.4706	H	0	0	0	0	0	0
106.2562	97.0464	94.1926	H	0	0	0	0	0	0
103.2626	94.7845	93.8679	H	0	0	0	0	0	0
103.4575	93.9278	95.3917	H	0	0	0	0	0	0
104.5835	93.6413	94.0766	H	0	0	0	0	0	0
108.9244	95.2807	101.2607	H	0	0	0	0	0	0
109.8223	95.3226	103.0534	H	0	0	0	0	0	0
108.3989	96.6786	105.3086	H	0	0	0	0	0	0
109.9206	93.5884	104.8368	H	0	0	0	0	0	0
111.0719	92.2889	106.5501	H	0	0	0	0	0	0
111.4500	95.7944	108.9926	H	0	0	0	0	0	0
110.2757	97.1075	107.3011	H	0	0	0	0	0	0
112.9765	92.8811	108.5944	H	0	0	0	0	0	0
111.0768	93.8164	112.4555	H	0	0	0	0	0	0
110.8489	94.8876	111.0507	H	0	0	0	0	0	0
112.4962	94.3951	111.5445	H	0	0	0	0	0	0
110.3331	98.1940	105.3763	H	0	0	0	0	0	0
109.6396	98.2157	103.7632	H	0	0	0	0	0	0
111.1225	97.3164	104.0813	H	0	0	0	0	0	0

1	2	2	0	0	0
1	3	2	0	0	0
1	32	1	0	0	0
1	33	1	0	0	0
4	5	2	0	0	0
4	9	1	0	0	0
4	35	1	0	0	0
5	6	1	0	0	0
5	36	1	0	0	0
6	7	2	0	0	0
6	37	1	0	0	0
7	8	1	0	0	0
7	10	1	0	0	0
8	9	2	0	0	0
8	38	1	0	0	0

```
  9 39  1  0  0  0
 10 11  1  0  0  0
 10 40  1  0  0  0
 10 41  1  0  0  0
 11 12  1  0  0  0
 11 13  1  0  0  0
 11 42  1  0  0  0
 12 21  1  0  0  0
 12 43  1  0  0  0
 12 44  1  0  0  0
 13 14  1  0  0  0
 13 45  1  0  0  0
 13 46  1  0  0  0
 14 15  1  0  0  0
 15 16  1  0  0  0
 15 17  2  0  0  0
 16 18  1  0  0  0
 16 19  1  0  0  0
 16 20  1  0  0  0
 18 47  1  0  0  0
 18 48  1  0  0  0
 18 49  1  0  0  0
 19 50  1  0  0  0
 19 51  1  0  0  0
 19 52  1  0  0  0
 20 53  1  0  0  0
 20 54  1  0  0  0
 20 55  1  0  0  0
 21 22  1  0  0  0
 21 56  1  0  0  0
 22 23  1  0  0  0
 22 24  2  0  0  0
 23 25  1  0  0  0
 23 57  1  0  0  0
 25 26  1  0  0  0
 25 34  1  0  0  0
 25 58  1  0  0  0
 26 27  2  0  0  0
 26 31  1  0  0  0
 27 28  1  0  0  0
 27 59  1  0  0  0
 28 29  2  0  0  0
 28 60  1  0  0  0
 29 30  1  0  0  0
 29 32  1  0  0  0
 30 31  2  0  0  0
 30 61  1  0  0  0
 31 62  1  0  0  0
 32 63  1  0  0  0
 33 64  1  0  0  0
 33 65  1  0  0  0
 33 66  1  0  0  0
 34 67  1  0  0  0
 34 68  1  0  0  0
 34 69  1  0  0  0
M  END
> <s_m_entry_id>
36

> <s_m_entry_name>
B_20.1

> <s_m_Source_Path>
C:\Users\julio\OneDrive - Universidad de Talca\Escritorio\Nueva carpeta

> <s_m_Source_File>
B_20.mol2

> <i_m_Source_File_Index>
1
```

```
$$$$
B_21
                     3D
 Structure written by MMmdl.
 69 70  0  0  1  0            999 V2000
  110.7815   93.0373  109.5301 S   0  0  0  0  0  0
  109.4849   93.7166  109.4339 O   0  0  0  0  0  0
  110.8505   91.6283  109.9348 O   0  0  0  0  0  0
  104.6031   94.6354   94.7156 C   0  0  0  0  0  0
  105.0771   95.9437   94.7850 C   0  0  0  0  0  0
  106.0118   96.2891   95.7584 C   0  0  0  0  0  0
  106.4705   95.3247   96.6626 C   0  0  0  0  0  0
  105.9845   94.0146   96.5891 C   0  0  0  0  0  0
  105.0528   93.6693   95.6130 C   0  0  0  0  0  0
  107.4598   95.7099   97.7611 C   0  0  0  0  0  0
  106.9423   96.7240   98.8263 C   0  0  2  0  0  0
  106.4102   95.9998  100.0905 C   0  0  0  0  0  0
  105.8451   97.6802   98.2912 C   0  0  0  0  0  0
  105.6939   98.8488   99.0959 O   0  0  0  0  0  0
  104.7989   99.8206   98.7562 C   0  0  0  0  0  0
  104.7528  101.0216   99.7501 C   0  0  0  0  0  0
  104.0603   99.7728   97.7671 O   0  0  0  0  0  0
  104.0878  100.5957  101.0893 C   0  0  0  0  0  0
  106.1769  101.5575  100.0689 C   0  0  0  0  0  0
  103.9072  102.1537   99.1157 C   0  0  0  0  0  0
  107.4556   95.6874  101.0774 N   0  0  0  0  0  0
  107.9156   96.5342  102.0476 C   0  0  0  0  0  0
  109.0499   96.0295  102.6255 N   0  0  0  0  0  0
  107.1788   97.9895  102.4690 S   0  0  0  0  0  0
  109.8881   96.6757  103.6570 C   0  0  2  0  0  0
  110.3243   95.7488  104.8026 C   0  0  0  0  0  0
  110.0896   94.3691  104.7574 C   0  0  0  0  0  0
  110.5059   93.5523  105.8057 C   0  0  0  0  0  0
  111.1684   94.0945  106.9094 C   0  0  0  0  0  0
  111.3998   95.4754  106.9566 C   0  0  0  0  0  0
  110.9829   96.2935  105.9090 C   0  0  0  0  0  0
  111.5498   93.2014  107.9270 N   0  0  0  0  0  0
  111.9021   94.0750  110.6471 C   0  0  0  0  0  0
  109.1659   97.9204  104.2079 C   0  0  0  0  0  0
  103.8786   94.3682   93.9603 H   0  0  0  0  0  0
  104.7207   96.6873   94.0873 H   0  0  0  0  0  0
  106.3703   97.3072   95.8096 H   0  0  0  0  0  0
  106.3218   93.2637   97.2890 H   0  0  0  0  0  0
  104.6773   92.6582   95.5548 H   0  0  0  0  0  0
  107.8305   94.8107   98.2570 H   0  0  0  0  0  0
  108.3317   96.1174   97.2476 H   0  0  0  0  0  0
  107.7808   97.3391   99.1591 H   0  0  0  0  0  0
  105.6651   96.6327  100.5762 H   0  0  0  0  0  0
  105.9018   95.0816   99.7903 H   0  0  0  0  0  0
  106.0541   97.9595   97.2571 H   0  0  0  0  0  0
  104.8876   97.1555   98.2828 H   0  0  0  0  0  0
  104.0598  101.4209  101.8028 H   0  0  0  0  0  0
  104.6295   99.7804  101.5726 H   0  0  0  0  0  0
  103.0548  100.2684  100.9570 H   0  0  0  0  0  0
  106.8278  100.7753  100.4639 H   0  0  0  0  0  0
  106.6702  101.9840   99.1941 H   0  0  0  0  0  0
  106.1465  102.3502  100.8183 H   0  0  0  0  0  0
  103.8253  103.0132   99.7830 H   0  0  0  0  0  0
  102.8889  101.8304   98.8911 H   0  0  0  0  0  0
  104.3425  102.5192   98.1848 H   0  0  0  0  0  0
  107.8672   94.7669  101.0196 H   0  0  0  0  0  0
  109.3779   95.1335  102.2945 H   0  0  0  0  0  0
  110.7861   97.0630  103.1737 H   0  0  0  0  0  0
  109.5780   93.9207  103.9182 H   0  0  0  0  0  0
  110.3108   92.4903  105.7522 H   0  0  0  0  0  0
  111.8916   95.9425  107.7947 H   0  0  0  0  0  0
  111.1723   97.3559  105.9648 H   0  0  0  0  0  0
  112.3440   92.6077  107.7349 H   0  0  0  0  0  0
  111.4972   94.0663  111.6590 H   0  0  0  0  0  0
  111.9299   95.0981  110.2720 H   0  0  0  0  0  0
  112.9077   93.6566  110.6523 H   0  0  0  0  0  0
```

```
 109.7846   98.4540  104.9307 H   0  0  0  0  0  0
 108.2296   97.6587  104.7031 H   0  0  0  0  0  0
 108.9286   98.6245  103.4091 H   0  0  0  0  0  0
 1  2  2  0  0  0
 1  3  2  0  0  0
 1 32  1  0  0  0
 1 33  1  0  0  0
 4  5  2  0  0  0
 4  9  1  0  0  0
 4 35  1  0  0  0
 5  6  1  0  0  0
 5 36  1  0  0  0
 6  7  2  0  0  0
 6 37  1  0  0  0
 7  8  1  0  0  0
 7 10  1  0  0  0
 8  9  2  0  0  0
 8 38  1  0  0  0
 9 39  1  0  0  0
10 11  1  0  0  0
10 40  1  0  0  0
10 41  1  0  0  0
11 12  1  0  0  0
11 13  1  0  0  0
11 42  1  0  0  0
12 21  1  0  0  0
12 43  1  0  0  0
12 44  1  0  0  0
13 14  1  0  0  0
13 45  1  0  0  0
13 46  1  0  0  0
14 15  1  0  0  0
15 16  1  0  0  0
15 17  2  0  0  0
16 18  1  0  0  0
16 19  1  0  0  0
16 20  1  0  0  0
18 47  1  0  0  0
18 48  1  0  0  0
18 49  1  0  0  0
19 50  1  0  0  0
19 51  1  0  0  0
19 52  1  0  0  0
20 53  1  0  0  0
20 54  1  0  0  0
20 55  1  0  0  0
21 22  1  0  0  0
21 56  1  0  0  0
22 23  1  0  0  0
22 24  2  0  0  0
23 25  1  0  0  0
23 57  1  0  0  0
25 26  1  0  0  0
25 34  1  0  0  0
25 58  1  0  0  0
26 27  2  0  0  0
26 31  1  0  0  0
27 28  1  0  0  0
27 59  1  0  0  0
28 29  2  0  0  0
28 60  1  0  0  0
29 30  1  0  0  0
29 32  1  0  0  0
30 31  2  0  0  0
30 61  1  0  0  0
31 62  1  0  0  0
32 63  1  0  0  0
33 64  1  0  0  0
33 65  1  0  0  0
33 66  1  0  0  0
34 67  1  0  0  0
```

```
 34 68  1  0  0  0
 34 69  1  0  0  0
M  END
> <s_m_entry_id>
37

> <s_m_entry_name>
B_21.1

> <s_m_Source_Path>
C:\Users\julio\OneDrive - Universidad de Talca\Escritorio\Nueva carpeta

> <s_m_Source_File>
B_21.mol2

> <i_m_Source_File_Index>
1

$$$$
B_22
                    3D
 Structure written by MMmdl.
 69 70  0  0  1  0            999 V2000
  110.9149   92.9438  109.6328 S   0  0  0  0  0  0
  109.4562   92.8850  109.5614 O   0  0  0  0  0  0
  111.6646   91.8407  110.2457 O   0  0  0  0  0  0
  104.6417   93.8021   95.4671 C   0  0  0  0  0  0
  103.8233   94.7784   96.0303 C   0  0  0  0  0  0
  104.3839   95.7657   96.8369 C   0  0  0  0  0  0
  105.7609   95.7724   97.0814 C   0  0  0  0  0  0
  106.5762   94.7902   96.5123 C   0  0  0  0  0  0
  106.0136   93.8068   95.7023 C   0  0  0  0  0  0
  106.3644   96.8706   97.9493 C   0  0  0  0  0  0
  106.1798   96.6480   99.4771 C   0  0  1  0  0  0
  107.1313   95.5431   99.9910 C   0  0  0  0  0  0
  106.3737   97.9631  100.2628 C   0  0  0  0  0  0
  105.4199   98.9211   99.8259 O   0  0  0  0  0  0
  105.6567  100.2447   99.9407 C   0  0  0  0  0  0
  104.5474  101.1291   99.2993 C   0  0  0  0  0  0
  106.6560  100.7348  100.4750 O   0  0  0  0  0  0
  105.1386  102.4917   98.8639 C   0  0  0  0  0  0
  103.9148  100.4553   98.0509 C   0  0  0  0  0  0
  103.4478  101.3765  100.3599 C   0  0  0  0  0  0
  106.7988   94.9887  101.3063 N   0  0  0  0  0  0
  107.2940   95.4855  102.4693 C   0  0  0  0  0  0
  108.6560   95.6252  102.5477 N   0  0  0  0  0  0
  106.1830   95.7917  103.6569 S   0  0  0  0  0  0
  109.4576   96.2718  103.6190 C   0  0  2  0  0  0
  109.8712   95.4248  104.8491 C   0  0  0  0  0  0
  109.5756   94.0573  104.9525 C   0  0  0  0  0  0
  110.0805   93.2996  106.0147 C   0  0  0  0  0  0
  110.8868   93.8951  106.9884 C   0  0  0  0  0  0
  111.1603   95.2591  106.9104 C   0  0  0  0  0  0
  110.6560   96.0143  105.8527 C   0  0  0  0  0  0
  111.5087   93.1119  107.9731 N   0  0  0  0  0  0
  111.4446   94.5498  110.4510 C   0  0  0  0  0  0
  109.0330   97.7225  103.9451 C   0  0  0  0  0  0
  104.2101   93.0337   94.8446 H   0  0  0  0  0  0
  102.7600   94.7724   95.8375 H   0  0  0  0  0  0
  103.7498   96.5264   97.2699 H   0  0  0  0  0  0
  107.6419   94.7881   96.6918 H   0  0  0  0  0  0
  106.6425   93.0473   95.2610 H   0  0  0  0  0  0
  107.4220   96.9964   97.7112 H   0  0  0  0  0  0
  105.8984   97.8048   97.6327 H   0  0  0  0  0  0
  105.1518   96.3198   99.6486 H   0  0  0  0  0  0
  107.0917   94.6986   99.3020 H   0  0  0  0  0  0
  108.1552   95.9159   99.9698 H   0  0  0  0  0  0
  106.2294   97.8090  101.3320 H   0  0  0  0  0  0
  107.3959   98.3251  100.1247 H   0  0  0  0  0  0
  104.3707  103.1654   98.4803 H   0  0  0  0  0  0
  105.8903  102.3770   98.0813 H   0  0  0  0  0  0
```

```
 105.6194  102.9924   99.7035 H   0  0  0  0  0  0
 103.1836  101.1091   97.5741 H   0  0  0  0  0  0
 103.3924   99.5303   98.3024 H   0  0  0  0  0  0
 104.6660  100.2117   97.2981 H   0  0  0  0  0  0
 102.6542  102.0145   99.9682 H   0  0  0  0  0  0
 103.8431  101.8695  101.2498 H   0  0  0  0  0  0
 102.9781  100.4463  100.6841 H   0  0  0  0  0  0
 105.9939   94.3814  101.3583 H   0  0  0  0  0  0
 109.2028   95.2586  101.7784 H   0  0  0  0  0  0
 110.4136   96.3971  103.1089 H   0  0  0  0  0  0
 108.9849   93.5628  104.1949 H   0  0  0  0  0  0
 109.8773   92.2376  106.0556 H   0  0  0  0  0  0
 111.7989   95.7318  107.6423 H   0  0  0  0  0  0
 110.9249   97.0584  105.8024 H   0  0  0  0  0  0
 112.4147   92.7137  107.7710 H   0  0  0  0  0  0
 111.0575   94.5634  111.4697 H   0  0  0  0  0  0
 111.0339   95.3901  109.8960 H   0  0  0  0  0  0
 112.5312   94.5927  110.4638 H   0  0  0  0  0  0
 109.8686   98.3168  104.3122 H   0  0  0  0  0  0
 108.2554   97.7492  104.7058 H   0  0  0  0  0  0
 108.6538   98.2361  103.0613 H   0  0  0  0  0  0
 1  2  2  0  0  0
 1  3  2  0  0  0
 1 32  1  0  0  0
 1 33  1  0  0  0
 4  5  2  0  0  0
 4  9  1  0  0  0
 4 35  1  0  0  0
 5  6  1  0  0  0
 5 36  1  0  0  0
 6  7  2  0  0  0
 6 37  1  0  0  0
 7  8  1  0  0  0
 7 10  1  0  0  0
 8  9  2  0  0  0
 8 38  1  0  0  0
 9 39  1  0  0  0
10 11  1  0  0  0
10 40  1  0  0  0
10 41  1  0  0  0
11 12  1  0  0  0
11 13  1  0  0  0
11 42  1  0  0  0
12 21  1  0  0  0
12 43  1  0  0  0
12 44  1  0  0  0
13 14  1  0  0  0
13 45  1  0  0  0
13 46  1  0  0  0
14 15  1  0  0  0
15 16  1  0  0  0
15 17  2  0  0  0
16 18  1  0  0  0
16 19  1  0  0  0
16 20  1  0  0  0
18 47  1  0  0  0
18 48  1  0  0  0
18 49  1  0  0  0
19 50  1  0  0  0
19 51  1  0  0  0
19 52  1  0  0  0
20 53  1  0  0  0
20 54  1  0  0  0
20 55  1  0  0  0
21 22  1  0  0  0
21 56  1  0  0  0
22 23  1  0  0  0
22 24  2  0  0  0
23 25  1  0  0  0
23 57  1  0  0  0
25 26  1  0  0  0
```

```
 25 34  1  0  0  0
 25 58  1  0  0  0
 26 27  2  0  0  0
 26 31  1  0  0  0
 27 28  1  0  0  0
 27 59  1  0  0  0
 28 29  2  0  0  0
 28 60  1  0  0  0
 29 30  1  0  0  0
 29 32  1  0  0  0
 30 31  2  0  0  0
 30 61  1  0  0  0
 31 62  1  0  0  0
 32 63  1  0  0  0
 33 64  1  0  0  0
 33 65  1  0  0  0
 33 66  1  0  0  0
 34 67  1  0  0  0
 34 68  1  0  0  0
 34 69  1  0  0  0
M  END
> <s_m_entry_id>
38

> <s_m_entry_name>
B_22.1

> <s_m_Source_Path>
C:\Users\julio\OneDrive - Universidad de Talca\Escritorio\Nueva carpeta

> <s_m_Source_File>
B_22.mol2

> <i_m_Source_File_Index>
1

$$$$
B_25
                    3D
 Structure written by MMmdl.
 75 76  0  0  1  0            999 V2000
  111.0668   92.8895  110.0905 S   0  0  0  0  0  0
  109.6423   92.7021  109.7952 O   0  0  0  0  0  0
  111.8633   91.7904  110.6431 O   0  0  0  0  0  0
  104.6801  101.1141   98.9206 C   0  0  0  0  0  0
  103.9993   99.9199   98.6551 C   0  0  0  0  0  0
  104.3699   98.7367   99.2924 C   0  0  0  0  0  0
  105.4330   98.7276  100.1995 C   0  0  0  0  0  0
  106.1208   99.9167  100.4556 C   0  0  0  0  0  0
  105.7529  101.1089   99.8294 C   0  0  0  0  0  0
  105.8285   97.4528  100.9412 C   0  0  0  0  0  0
  106.1486   96.2247  100.0459 C   0  0  2  0  0  0
  106.6248   94.9998  100.8685 C   0  0  0  0  0  0
  107.1237   96.5498   98.8783 C   0  0  0  0  0  0
  106.4601   96.5448   97.6144 O   0  0  0  0  0  0
  106.2443   95.3846   96.9337 C   0  0  0  0  0  0
  105.3995   95.5714   95.6397 C   0  0  0  0  0  0
  106.6563   94.2774   97.2907 O   0  0  0  0  0  0
  104.1090   96.3721   95.9529 C   0  0  0  0  0  0
  106.2406   96.3433   94.5933 C   0  0  0  0  0  0
  104.9891   94.1998   95.0329 C   0  0  0  0  0  0
  107.8566   95.2232  101.6428 N   0  0  0  0  0  0
  107.9430   95.5343  102.9648 C   0  0  0  0  0  0
  109.1909   95.9936  103.2568 N   0  0  0  0  0  0
  106.6866   95.3920  104.0562 S   0  0  0  0  0  0
  109.6261   96.6803  104.4829 C   0  0  1  0  0  0
  110.1671   95.7622  105.5958 C   0  0  0  0  0  0
  110.2249   94.3675  105.4509 C   0  0  0  0  0  0
  110.7437   93.5722  106.4702 C   0  0  0  0  0  0
  111.2342   94.1648  107.6304 C   0  0  0  0  0  0
  111.1571   95.5474  107.7985 C   0  0  0  0  0  0
```

110.6141	96.3396	106.7908	C	0	0	0	0	0	0
111.8575	93.3727	108.5950	N	0	0	0	0	0	0
111.3179	94.3942	111.1847	C	0	0	0	0	0	0
110.6639	97.7345	104.0502	C	0	0	0	0	0	0
106.5119	102.3796	100.1745	C	0	0	0	0	0	0
104.2283	102.3965	98.2387	C	0	0	0	0	0	0
103.1719	99.9056	97.9601	H	0	0	0	0	0	0
103.8234	97.8292	99.0853	H	0	0	0	0	0	0
106.9398	99.9171	101.1608	H	0	0	0	0	0	0
106.6892	97.6779	101.5697	H	0	0	0	0	0	0
105.0191	97.2015	101.6261	H	0	0	0	0	0	0
105.2009	95.9124	99.6048	H	0	0	0	0	0	0
105.8148	94.6128	101.4883	H	0	0	0	0	0	0
106.8470	94.1945	100.1696	H	0	0	0	0	0	0
107.5731	97.5341	99.0191	H	0	0	0	0	0	0
107.9642	95.8575	98.8566	H	0	0	0	0	0	0
103.4811	96.4770	95.0661	H	0	0	0	0	0	0
104.3241	97.3821	96.3054	H	0	0	0	0	0	0
103.5058	95.8814	96.7175	H	0	0	0	0	0	0
107.1684	95.8224	94.3512	H	0	0	0	0	0	0
105.6949	96.4744	93.6586	H	0	0	0	0	0	0
106.5070	97.3413	94.9427	H	0	0	0	0	0	0
104.3976	94.3270	94.1239	H	0	0	0	0	0	0
104.3860	93.6155	95.7289	H	0	0	0	0	0	0
105.8553	93.5920	94.7685	H	0	0	0	0	0	0
108.7236	95.1730	101.1265	H	0	0	0	0	0	0
109.9093	95.8509	102.5562	H	0	0	0	0	0	0
108.7792	97.2265	104.9017	H	0	0	0	0	0	0
109.8784	93.8838	104.5490	H	0	0	0	0	0	0
110.7955	92.5025	106.3391	H	0	0	0	0	0	0
111.5292	96.0154	108.6972	H	0	0	0	0	0	0
110.5631	97.4092	106.9361	H	0	0	0	0	0	0
112.7905	93.0247	108.4178	H	0	0	0	0	0	0
111.0577	94.1266	112.2073	H	0	0	0	0	0	0
110.6585	95.1909	110.8431	H	0	0	0	0	0	0
112.3631	94.6977	111.1384	H	0	0	0	0	0	0
110.9548	98.3928	104.8631	H	0	0	0	0	0	0
110.2709	98.3776	103.2612	H	0	0	0	0	0	0
111.5708	97.2649	103.6705	H	0	0	0	0	0	0
107.4570	102.1647	100.6716	H	0	0	0	0	0	0
105.9156	102.9906	100.8515	H	0	0	0	0	0	0
106.7369	102.9590	99.2793	H	0	0	0	0	0	0
103.9152	103.1330	98.9810	H	0	0	0	0	0	0
103.3891	102.2319	97.5608	H	0	0	0	0	0	0
105.0425	102.8276	97.6577	H	0	0	0	0	0	0

1	2	2	0	0	0
1	3	2	0	0	0
1	32	1	0	0	0
1	33	1	0	0	0
4	5	2	0	0	0
4	9	1	0	0	0
4	36	1	0	0	0
5	6	1	0	0	0
5	37	1	0	0	0
6	7	2	0	0	0
6	38	1	0	0	0
7	8	1	0	0	0
7	10	1	0	0	0
8	9	2	0	0	0
8	39	1	0	0	0
9	35	1	0	0	0
10	11	1	0	0	0
10	40	1	0	0	0
10	41	1	0	0	0
11	12	1	0	0	0
11	13	1	0	0	0
11	42	1	0	0	0
12	21	1	0	0	0
12	43	1	0	0	0
12	44	1	0	0	0
13	14	1	0	0	0

```
 13 45  1  0  0  0
 13 46  1  0  0  0
 14 15  1  0  0  0
 15 16  1  0  0  0
 15 17  2  0  0  0
 16 18  1  0  0  0
 16 19  1  0  0  0
 16 20  1  0  0  0
 18 47  1  0  0  0
 18 48  1  0  0  0
 18 49  1  0  0  0
 19 50  1  0  0  0
 19 51  1  0  0  0
 19 52  1  0  0  0
 20 53  1  0  0  0
 20 54  1  0  0  0
 20 55  1  0  0  0
 21 22  1  0  0  0
 21 56  1  0  0  0
 22 23  1  0  0  0
 22 24  2  0  0  0
 23 25  1  0  0  0
 23 57  1  0  0  0
 25 26  1  0  0  0
 25 34  1  0  0  0
 25 58  1  0  0  0
 26 27  2  0  0  0
 26 31  1  0  0  0
 27 28  1  0  0  0
 27 59  1  0  0  0
 28 29  2  0  0  0
 28 60  1  0  0  0
 29 30  1  0  0  0
 29 32  1  0  0  0
 30 31  2  0  0  0
 30 61  1  0  0  0
 31 62  1  0  0  0
 32 63  1  0  0  0
 33 64  1  0  0  0
 33 65  1  0  0  0
 33 66  1  0  0  0
 34 67  1  0  0  0
 34 68  1  0  0  0
 34 69  1  0  0  0
 35 70  1  0  0  0
 35 71  1  0  0  0
 35 72  1  0  0  0
 36 73  1  0  0  0
 36 74  1  0  0  0
 36 75  1  0  0  0
M  END
> <s_m_entry_id>
39

> <s_m_entry_name>
B_25.1

> <s_m_Source_Path>
C:\Users\julio\OneDrive - Universidad de Talca\Escritorio\Nueva carpeta

> <s_m_Source_File>
B_25.mol2

> <i_m_Source_File_Index>
1

$$$$
B_26
                    3D
 Structure written by MMmdl.
 75 76  0  0  1  0            999 V2000
```

111.0802	92.7092	110.0014	S	0	0	0	0	0	0
109.6452	92.5919	109.7190	O	0	0	0	0	0	0
111.8562	91.5508	110.4576	O	0	0	0	0	0	0
105.6968	95.4870	95.3681	C	0	0	0	0	0	0
106.7429	95.5700	96.2890	C	0	0	0	0	0	0
106.4874	95.8905	97.6185	C	0	0	0	0	0	0
105.1780	96.1313	98.0562	C	0	0	0	0	0	0
104.1311	96.0185	97.1339	C	0	0	0	0	0	0
104.3753	95.6910	95.7964	C	0	0	0	0	0	0
104.8169	96.5195	99.4974	C	0	0	0	0	0	0
105.9303	96.5687	100.5781	C	0	0	1	0	0	0
106.5240	95.1597	100.8704	C	0	0	0	0	0	0
106.9695	97.7145	100.3886	C	0	0	0	0	0	0
106.3602	98.9976	100.4979	O	0	0	0	0	0	0
106.2489	99.8299	99.4320	C	0	0	0	0	0	0
105.5478	101.1750	99.7825	C	0	0	0	0	0	0
106.6530	99.5738	98.2956	O	0	0	0	0	0	0
106.4441	101.9824	100.7501	C	0	0	0	0	0	0
105.3103	102.0328	98.5132	C	0	0	0	0	0	0
104.1759	100.9083	100.4502	C	0	0	0	0	0	0
107.7632	95.1705	101.6569	N	0	0	0	0	0	0
107.8499	95.4897	102.9765	C	0	0	0	0	0	0
109.1001	95.9392	103.2712	N	0	0	0	0	0	0
106.5799	95.3924	104.0568	S	0	0	0	0	0	0
109.5381	96.6067	104.5073	C	0	0	1	0	0	0
110.0800	95.6769	105.6085	C	0	0	0	0	0	0
110.1646	94.2869	105.4354	C	0	0	0	0	0	0
110.7134	93.4868	106.4313	C	0	0	0	0	0	0
111.2048	94.0709	107.5963	C	0	0	0	0	0	0
111.0934	95.4433	107.7988	C	0	0	0	0	0	0
110.5156	96.2400	106.8148	C	0	0	0	0	0	0
111.8559	93.2704	108.5328	N	0	0	0	0	0	0
111.3766	94.1337	111.1872	C	0	0	0	0	0	0
110.5853	97.6598	104.1013	C	0	0	0	0	0	0
103.1994	95.5750	94.8346	C	0	0	0	0	0	0
106.0209	95.1878	93.9144	C	0	0	0	0	0	0
107.7650	95.4050	95.9739	H	0	0	0	0	0	0
107.3293	95.9492	98.2909	H	0	0	0	0	0	0
103.1160	96.1969	97.4611	H	0	0	0	0	0	0
104.3154	97.4890	99.4615	H	0	0	0	0	0	0
104.0503	95.8212	99.8356	H	0	0	0	0	0	0
105.4154	96.8383	101.5008	H	0	0	0	0	0	0
105.7754	94.5277	101.3528	H	0	0	0	0	0	0
106.7577	94.6371	99.9436	H	0	0	0	0	0	0
107.7053	97.6848	101.1916	H	0	0	0	0	0	0
107.5426	97.6088	99.4689	H	0	0	0	0	0	0
106.0508	102.9881	100.8899	H	0	0	0	0	0	0
107.4561	102.0994	100.3701	H	0	0	0	0	0	0
106.5160	101.5189	101.7333	H	0	0	0	0	0	0
104.7763	102.9547	98.7462	H	0	0	0	0	0	0
104.7197	101.5013	97.7650	H	0	0	0	0	0	0
106.2554	102.3262	98.0570	H	0	0	0	0	0	0
103.6572	101.8419	100.6773	H	0	0	0	0	0	0
104.2712	100.3669	101.3927	H	0	0	0	0	0	0
103.5166	100.3279	99.8034	H	0	0	0	0	0	0
108.6255	95.0517	101.1433	H	0	0	0	0	0	0
109.8190	95.7988	102.5704	H	0	0	0	0	0	0
108.6957	97.1548	104.9340	H	0	0	0	0	0	0
109.8238	93.8152	104.5257	H	0	0	0	0	0	0
110.7933	92.4192	106.2746	H	0	0	0	0	0	0
111.4666	95.8994	108.7048	H	0	0	0	0	0	0
110.4424	97.3045	106.9858	H	0	0	0	0	0	0
112.7782	92.9113	108.3203	H	0	0	0	0	0	0
111.0444	93.8369	112.1794	H	0	0	0	0	0	0
110.7982	94.9961	110.8568	H	0	0	0	0	0	0
112.4401	94.3674	111.2030	H	0	0	0	0	0	0
110.9284	98.2456	104.9498	H	0	0	0	0	0	0
110.1821	98.3627	103.3705	H	0	0	0	0	0	0
111.4635	97.1915	103.6571	H	0	0	0	0	0	0
102.2440	95.5774	95.3597	H	0	0	0	0	0	0
103.2567	94.6467	94.2630	H	0	0	0	0	0	0

```
 103.1998   96.4124   94.1333 H   0  0  0  0  0  0
 105.4015   95.7924   93.2541 H   0  0  0  0  0  0
 105.8487   94.1338   93.6979 H   0  0  0  0  0  0
 107.0564   95.4211   93.6759 H   0  0  0  0  0  0
 1  2  2  0  0  0
 1  3  2  0  0  0
 1 32  1  0  0  0
 1 33  1  0  0  0
 4  5  2  0  0  0
 4  9  1  0  0  0
 4 36  1  0  0  0
 5  6  1  0  0  0
 5 37  1  0  0  0
 6  7  2  0  0  0
 6 38  1  0  0  0
 7  8  1  0  0  0
 7 10  1  0  0  0
 8  9  2  0  0  0
 8 39  1  0  0  0
 9 35  1  0  0  0
10 11  1  0  0  0
10 40  1  0  0  0
10 41  1  0  0  0
11 12  1  0  0  0
11 13  1  0  0  0
11 42  1  0  0  0
12 21  1  0  0  0
12 43  1  0  0  0
12 44  1  0  0  0
13 14  1  0  0  0
13 45  1  0  0  0
13 46  1  0  0  0
14 15  1  0  0  0
15 16  1  0  0  0
15 17  2  0  0  0
16 18  1  0  0  0
16 19  1  0  0  0
16 20  1  0  0  0
18 47  1  0  0  0
18 48  1  0  0  0
18 49  1  0  0  0
19 50  1  0  0  0
19 51  1  0  0  0
19 52  1  0  0  0
20 53  1  0  0  0
20 54  1  0  0  0
20 55  1  0  0  0
21 22  1  0  0  0
21 56  1  0  0  0
22 23  1  0  0  0
22 24  2  0  0  0
23 25  1  0  0  0
23 57  1  0  0  0
25 26  1  0  0  0
25 34  1  0  0  0
25 58  1  0  0  0
26 27  2  0  0  0
26 31  1  0  0  0
27 28  1  0  0  0
27 59  1  0  0  0
28 29  2  0  0  0
28 60  1  0  0  0
29 30  1  0  0  0
29 32  1  0  0  0
30 31  2  0  0  0
30 61  1  0  0  0
31 62  1  0  0  0
32 63  1  0  0  0
33 64  1  0  0  0
33 65  1  0  0  0
33 66  1  0  0  0
```

```
 34 67  1  0  0  0
 34 68  1  0  0  0
 34 69  1  0  0  0
 35 70  1  0  0  0
 35 71  1  0  0  0
 35 72  1  0  0  0
 36 73  1  0  0  0
 36 74  1  0  0  0
 36 75  1  0  0  0
M  END
> <s_m_entry_id>
40

> <s_m_entry_name>
B_26.1

> <s_m_Source_Path>
C:\Users\julio\OneDrive - Universidad de Talca\Escritorio\Nueva carpeta

> <s_m_Source_File>
B_26.mol2

> <i_m_Source_File_Index>
1

$$$$
B_29
                    3D
 Structure written by MMmdl.
 81 82  0  0  1  0            999 V2000
  111.0690   92.7616  109.9472 S   0  0  0  0  0  0
  109.6415   92.5940  109.6567 O   0  0  0  0  0  0
  111.8566   91.6358  110.4571 O   0  0  0  0  0  0
  103.8258  101.3281   99.3488 C   0  0  0  0  0  0
  103.6594  100.4885  100.4604 C   0  0  0  0  0  0
  104.5259   99.4236  100.6862 C   0  0  0  0  0  0
  105.5657   99.1622   99.7882 C   0  0  0  0  0  0
  105.7738  100.0179   98.7070 C   0  0  0  0  0  0
  104.9142  101.0918   98.4971 C   0  0  0  0  0  0
  106.4784   97.9543   99.9809 C   0  0  0  0  0  0
  105.7369   96.6174  100.2703 C   0  0  2  0  0  0
  106.6920   95.4141  100.5040 C   0  0  0  0  0  0
  104.6582   96.2681   99.2246 C   0  0  0  0  0  0
  105.2541   95.9671   97.9736 O   0  0  0  0  0  0
  104.5055   95.5133   96.9466 C   0  0  0  0  0  0
  105.3331   95.1767   95.6738 C   0  0  0  0  0  0
  103.2812   95.3660   96.9785 O   0  0  0  0  0  0
  104.5072   95.4960   94.4015 C   0  0  0  0  0  0
  106.6555   95.9886   95.5997 C   0  0  0  0  0  0
  105.6642   93.6659   95.7134 C   0  0  0  0  0  0
  107.8545   95.6683  101.3704 N   0  0  0  0  0  0
  107.8197   95.8201  102.7264 C   0  0  0  0  0  0
  109.0744   96.0107  103.2197 N   0  0  0  0  0  0
  106.4319   95.7892  103.6567 S   0  0  0  0  0  0
  109.4484   96.6775  104.4787 C   0  0  1  0  0  0
  110.0320   95.7473  105.5621 C   0  0  0  0  0  0
  110.0795   94.3523  105.4010 C   0  0  0  0  0  0
  110.6555   93.5437  106.3794 C   0  0  0  0  0  0
  111.2028   94.1181  107.5276 C   0  0  0  0  0  0
  111.1329   95.4983  107.7148 C   0  0  0  0  0  0
  110.5405   96.3040  106.7437 C   0  0  0  0  0  0
  111.8546   93.3096  108.4642 N   0  0  0  0  0  0
  111.3185   94.2269  111.0949 C   0  0  0  0  0  0
  110.4298   97.8061  104.0905 C   0  0  0  0  0  0
  102.8208  102.4808   99.1003 C   0  0  0  0  0  0
  101.3929  101.9091   98.9333 C   0  0  0  0  0  0
  103.1395  103.3168   97.8304 C   0  0  0  0  0  0
  102.8440  103.4494  100.3020 C   0  0  0  0  0  0
  102.8474  100.6520  101.1530 H   0  0  0  0  0  0
  104.3645   98.7928  101.5481 H   0  0  0  0  0  0
  106.5926   99.8516   98.0205 H   0  0  0  0  0  0
```

```
  105.1081  101.7315   97.6569 H   0  0  0  0  0  0
  107.1098   97.8244   99.1000 H   0  0  0  0  0  0
  107.1536   98.1987  100.8005 H   0  0  0  0  0  0
  105.1883   96.7605  101.1974 H   0  0  0  0  0  0
  106.1346   94.5546  100.8827 H   0  0  0  0  0  0
  107.0916   95.0908   99.5428 H   0  0  0  0  0  0
  104.0810   95.4094   99.5740 H   0  0  0  0  0  0
  103.9490   97.0900   99.1106 H   0  0  0  0  0  0
  105.1102   95.3930   93.4999 H   0  0  0  0  0  0
  104.1354   96.5226   94.4072 H   0  0  0  0  0  0
  103.6421   94.8389   94.2931 H   0  0  0  0  0  0
  107.1995   95.7910   94.6756 H   0  0  0  0  0  0
  107.3342   95.7509   96.4220 H   0  0  0  0  0  0
  106.4627   97.0634   95.6289 H   0  0  0  0  0  0
  106.2532   93.3578   94.8542 H   0  0  0  0  0  0
  104.7637   93.0476   95.7121 H   0  0  0  0  0  0
  106.2417   93.4026   96.6004 H   0  0  0  0  0  0
  108.7576   95.6965  100.9172 H   0  0  0  0  0  0
  109.8466   95.7876  102.6042 H   0  0  0  0  0  0
  108.5741   97.1560  104.9237 H   0  0  0  0  0  0
  109.6806   93.8797  104.5154 H   0  0  0  0  0  0
  110.6976   92.4737  106.2330 H   0  0  0  0  0  0
  111.5449   95.9523  108.6037 H   0  0  0  0  0  0
  110.4992   97.3706  106.9101 H   0  0  0  0  0  0
  112.7908   92.9803  108.2692 H   0  0  0  0  0  0
  111.0380   93.9294  112.1039 H   0  0  0  0  0  0
  110.6783   95.0466  110.7696 H   0  0  0  0  0  0
  112.3675   94.5216  111.0772 H   0  0  0  0  0  0
  110.6439   98.4754  104.9180 H   0  0  0  0  0  0
  110.0185   98.4322  103.2977 H   0  0  0  0  0  0
  111.3811   97.4075  103.7355 H   0  0  0  0  0  0
  101.0528  101.3810   99.8249 H   0  0  0  0  0  0
  101.3412  101.2056   98.1006 H   0  0  0  0  0  0
  100.6644  102.6977   98.7378 H   0  0  0  0  0  0
  104.1200  103.7929   97.8934 H   0  0  0  0  0  0
  102.4116  104.1164   97.6828 H   0  0  0  0  0  0
  103.1241  102.7035   96.9281 H   0  0  0  0  0  0
  103.8561  103.8187  100.4567 H   0  0  0  0  0  0
  102.5269  102.9710  101.2288 H   0  0  0  0  0  0
  102.1943  104.3128  100.1459 H   0  0  0  0  0  0
  1  2  2  0  0  0
  1  3  2  0  0  0
  1 32  1  0  0  0
  1 33  1  0  0  0
  4  5  2  0  0  0
  4  9  1  0  0  0
  4 35  1  0  0  0
  5  6  1  0  0  0
  5 39  1  0  0  0
  6  7  2  0  0  0
  6 40  1  0  0  0
  7  8  1  0  0  0
  7 10  1  0  0  0
  8  9  2  0  0  0
  8 41  1  0  0  0
  9 42  1  0  0  0
 10 11  1  0  0  0
 10 43  1  0  0  0
 10 44  1  0  0  0
 11 12  1  0  0  0
 11 13  1  0  0  0
 11 45  1  0  0  0
 12 21  1  0  0  0
 12 46  1  0  0  0
 12 47  1  0  0  0
 13 14  1  0  0  0
 13 48  1  0  0  0
 13 49  1  0  0  0
 14 15  1  0  0  0
 15 16  1  0  0  0
 15 17  2  0  0  0
```

```
 16 18  1  0  0  0
 16 19  1  0  0  0
 16 20  1  0  0  0
 18 50  1  0  0  0
 18 51  1  0  0  0
 18 52  1  0  0  0
 19 53  1  0  0  0
 19 54  1  0  0  0
 19 55  1  0  0  0
 20 56  1  0  0  0
 20 57  1  0  0  0
 20 58  1  0  0  0
 21 22  1  0  0  0
 21 59  1  0  0  0
 22 23  1  0  0  0
 22 24  2  0  0  0
 23 25  1  0  0  0
 23 60  1  0  0  0
 25 26  1  0  0  0
 25 34  1  0  0  0
 25 61  1  0  0  0
 26 27  2  0  0  0
 26 31  1  0  0  0
 27 28  1  0  0  0
 27 62  1  0  0  0
 28 29  2  0  0  0
 28 63  1  0  0  0
 29 30  1  0  0  0
 29 32  1  0  0  0
 30 31  2  0  0  0
 30 64  1  0  0  0
 31 65  1  0  0  0
 32 66  1  0  0  0
 33 67  1  0  0  0
 33 68  1  0  0  0
 33 69  1  0  0  0
 34 70  1  0  0  0
 34 71  1  0  0  0
 34 72  1  0  0  0
 35 36  1  0  0  0
 35 37  1  0  0  0
 35 38  1  0  0  0
 36 73  1  0  0  0
 36 74  1  0  0  0
 36 75  1  0  0  0
 37 76  1  0  0  0
 37 77  1  0  0  0
 37 78  1  0  0  0
 38 79  1  0  0  0
 38 80  1  0  0  0
 38 81  1  0  0  0
M  END
> <s_m_entry_id>
41

> <s_m_entry_name>
B_29.1

> <s_m_Source_Path>
C:\Users\julio\OneDrive - Universidad de Talca\Escritorio\Nueva carpeta

> <s_m_Source_File>
B_29.mol2

> <i_m_Source_File_Index>
1

$$$$
B_30
                    3D
 Structure written by MMmdl.
```

```
81 82  0  0  1  0            999 V2000
 111.0713   92.8111  109.9324 S   0  0  0  0  0  0
 109.6437   92.6313  109.6546 O   0  0  0  0  0  0
 111.8604   91.6924  110.4572 O   0  0  0  0  0  0
 103.7436  101.2343   99.1559 C   0  0  0  0  0  0
 103.1290  100.4347  100.1271 C   0  0  0  0  0  0
 103.7909   99.3428  100.6789 C   0  0  0  0  0  0
 105.0939   99.0435  100.2750 C   0  0  0  0  0  0
 105.7341   99.8493   99.3373 C   0  0  0  0  0  0
 105.0660  100.9348   98.7808 C   0  0  0  0  0  0
 105.7912   97.8164  100.8431 C   0  0  0  0  0  0
 106.0507   96.6833   99.8166 C   0  0  1  0  0  0
 106.6481   95.3999  100.4587 C   0  0  0  0  0  0
 104.7735   96.3378   99.0036 C   0  0  0  0  0  0
 105.0231   95.5272   97.8608 O   0  0  0  0  0  0
 105.4919   96.0638   96.7027 C   0  0  0  0  0  0
 105.5484   95.0419   95.5312 C   0  0  0  0  0  0
 105.8509   97.2348   96.5687 O   0  0  0  0  0  0
 106.2158   95.6596   94.2737 C   0  0  0  0  0  0
 106.3544   93.7949   95.9604 C   0  0  0  0  0  0
 104.1039   94.6107   95.1700 C   0  0  0  0  0  0
 107.8203   95.6216  101.3250 N   0  0  0  0  0  0
 107.8080   95.7286  102.6860 C   0  0  0  0  0  0
 109.0565   95.9925  103.1560 N   0  0  0  0  0  0
 106.4521   95.5772  103.6569 S   0  0  0  0  0  0
 109.4179   96.6624  104.4196 C   0  0  1  0  0  0
 110.0131   95.7447  105.5107 C   0  0  0  0  0  0
 110.0781   94.3516  105.3575 C   0  0  0  0  0  0
 110.6560   93.5554  106.3463 C   0  0  0  0  0  0
 111.1929   94.1411  107.4932 C   0  0  0  0  0  0
 111.1096   95.5212  107.6707 C   0  0  0  0  0  0
 110.5110   96.3153  106.6923 C   0  0  0  0  0  0
 111.8535   93.3444  108.4380 N   0  0  0  0  0  0
 111.3152   94.2895  111.0614 C   0  0  0  0  0  0
 110.3788   97.8095  104.0369 C   0  0  0  0  0  0
 102.9695  102.4389   98.5617 C   0  0  0  0  0  0
 103.6530  103.0380   97.3074 C   0  0  0  0  0  0
 102.8694  103.5367   99.6420 C   0  0  0  0  0  0
 101.5425  102.0209   98.1204 C   0  0  0  0  0  0
 102.1274  100.6478  100.4670 H   0  0  0  0  0  0
 103.2862   98.7294  101.4114 H   0  0  0  0  0  0
 106.7452   99.6238   99.0240 H   0  0  0  0  0  0
 105.6049  101.5253   98.0566 H   0  0  0  0  0  0
 106.7306   98.1504  101.2857 H   0  0  0  0  0  0
 105.1948   97.4280  101.6706 H   0  0  0  0  0  0
 106.7918   97.0711   99.1168 H   0  0  0  0  0  0
 105.8705   94.8440  100.9869 H   0  0  0  0  0  0
 106.9662   94.7232   99.6648 H   0  0  0  0  0  0
 104.0683   95.8110   99.6478 H   0  0  0  0  0  0
 104.2576   97.2416   98.6775 H   0  0  0  0  0  0
 106.2558   94.9491   93.4480 H   0  0  0  0  0  0
 107.2419   95.9685   94.4750 H   0  0  0  0  0  0
 105.6763   96.5384   93.9139 H   0  0  0  0  0  0
 106.2488   92.9931   95.2296 H   0  0  0  0  0  0
 106.0201   93.3925   96.9169 H   0  0  0  0  0  0
 107.4175   94.0167   96.0630 H   0  0  0  0  0  0
 104.0970   93.8934   94.3473 H   0  0  0  0  0  0
 103.4917   95.4586   94.8566 H   0  0  0  0  0  0
 103.5963   94.1317   96.0085 H   0  0  0  0  0  0
 108.7127   95.6951  100.8570 H   0  0  0  0  0  0
 109.8327   95.7881  102.5392 H   0  0  0  0  0  0
 108.5319   97.1300  104.8545 H   0  0  0  0  0  0
 109.6906   93.8718  104.4712 H   0  0  0  0  0  0
 110.7128   92.4865  106.2021 H   0  0  0  0  0  0
 111.5129   95.9859  108.5570 H   0  0  0  0  0  0
 110.4568   97.3825  106.8535 H   0  0  0  0  0  0
 112.7836   93.0037  108.2412 H   0  0  0  0  0  0
 111.0363   94.0044  112.0744 H   0  0  0  0  0  0
 110.6697   95.1004  110.7259 H   0  0  0  0  0  0
 112.3593   94.5918  111.0384 H   0  0  0  0  0  0
 110.5973   98.4760  104.8662 H   0  0  0  0  0  0
```

109.9496	98.4398	103.2580	H	0	0	0	0	0	0
111.3296	97.4277	103.6618	H	0	0	0	0	0	0
103.7250	102.3048	96.5032	H	0	0	0	0	0	0
104.6559	103.3929	97.5242	H	0	0	0	0	0	0
103.1026	103.8943	96.9137	H	0	0	0	0	0	0
102.2719	103.2113	100.4960	H	0	0	0	0	0	0
102.4116	104.4492	99.2565	H	0	0	0	0	0	0
103.8567	103.7921	100.0294	H	0	0	0	0	0	0
100.9198	101.6984	98.9548	H	0	0	0	0	0	0
101.5733	101.2038	97.3983	H	0	0	0	0	0	0
101.0132	102.8495	97.6470	H	0	0	0	0	0	0

1	2	2	0	0	0
1	3	2	0	0	0
1	32	1	0	0	0
1	33	1	0	0	0
4	5	2	0	0	0
4	9	1	0	0	0
4	35	1	0	0	0
5	6	1	0	0	0
5	39	1	0	0	0
6	7	2	0	0	0
6	40	1	0	0	0
7	8	1	0	0	0
7	10	1	0	0	0
8	9	2	0	0	0
8	41	1	0	0	0
9	42	1	0	0	0
10	11	1	0	0	0
10	43	1	0	0	0
10	44	1	0	0	0
11	12	1	0	0	0
11	13	1	0	0	0
11	45	1	0	0	0
12	21	1	0	0	0
12	46	1	0	0	0
12	47	1	0	0	0
13	14	1	0	0	0
13	48	1	0	0	0
13	49	1	0	0	0
14	15	1	0	0	0
15	16	1	0	0	0
15	17	2	0	0	0
16	18	1	0	0	0
16	19	1	0	0	0
16	20	1	0	0	0
18	50	1	0	0	0
18	51	1	0	0	0
18	52	1	0	0	0
19	53	1	0	0	0
19	54	1	0	0	0
19	55	1	0	0	0
20	56	1	0	0	0
20	57	1	0	0	0
20	58	1	0	0	0
21	22	1	0	0	0
21	59	1	0	0	0
22	23	1	0	0	0
22	24	2	0	0	0
23	25	1	0	0	0
23	60	1	0	0	0
25	26	1	0	0	0
25	34	1	0	0	0
25	61	1	0	0	0
26	27	2	0	0	0
26	31	1	0	0	0
27	28	1	0	0	0
27	62	1	0	0	0
28	29	2	0	0	0
28	63	1	0	0	0
29	30	1	0	0	0
29	32	1	0	0	0

```
 30 31  2  0  0  0
 30 64  1  0  0  0
 31 65  1  0  0  0
 32 66  1  0  0  0
 33 67  1  0  0  0
 33 68  1  0  0  0
 33 69  1  0  0  0
 34 70  1  0  0  0
 34 71  1  0  0  0
 34 72  1  0  0  0
 35 36  1  0  0  0
 35 37  1  0  0  0
 35 38  1  0  0  0
 36 73  1  0  0  0
 36 74  1  0  0  0
 36 75  1  0  0  0
 37 76  1  0  0  0
 37 77  1  0  0  0
 37 78  1  0  0  0
 38 79  1  0  0  0
 38 80  1  0  0  0
 38 81  1  0  0  0
M  END
> <s_m_entry_id>
42

> <s_m_entry_name>
B_30.1

> <s_m_Source_Path>
C:\Users\julio\OneDrive - Universidad de Talca\Escritorio\Nueva carpeta

> <s_m_Source_File>
B_30.mol2

> <i_m_Source_File_Index>
1

$$$$
B_33
                    3D
 Structure written by MMmdl.
 66 67  0  0  1  0            999 V2000
  111.0698   92.7524  109.9474 S   0  0  0  0  0  0
  109.6413   92.5920  109.6581 O   0  0  0  0  0  0
  111.8568   91.6242  110.4574 O   0  0  0  0  0  0
  106.4878   97.1209  100.8568 C   0  0  2  0  0  0
  107.7074   96.6452  101.5487 N   0  0  0  0  0  0
  107.7808   96.0110  102.7598 C   0  0  0  0  0  0
  109.0564   95.9297  103.2204 N   0  0  0  0  0  0
  106.5091   95.3960  103.6570 S   0  0  0  0  0  0
  109.5000   96.6228  104.4414 C   0  0  1  0  0  0
  110.0757   95.6979  105.5313 C   0  0  0  0  0  0
  110.5843   96.2643  106.7088 C   0  0  0  0  0  0
  111.1597   95.4615  107.6920 C   0  0  0  0  0  0
  111.2105   94.0800  107.5178 C   0  0  0  0  0  0
  110.6568   93.4961  106.3795 C   0  0  0  0  0  0
  110.1019   94.3022  105.3869 C   0  0  0  0  0  0
  111.8559   93.2782  108.4598 N   0  0  0  0  0  0
  111.3200   94.2254  111.0884 C   0  0  0  0  0  0
  110.4983   97.7250  104.0273 C   0  0  0  0  0  0
  105.4105  101.9035   99.5303 C   0  0  0  0  0  0
  106.2533  101.7916  100.6358 C   0  0  0  0  0  0
  106.3070  100.5924  101.3429 C   0  0  0  0  0  0
  105.5354   99.5019  100.9296 C   0  0  0  0  0  0
  104.6824   99.6270   99.8294 C   0  0  0  0  0  0
  104.6222  100.8275   99.1259 C   0  0  0  0  0  0
  105.6524   98.1666  101.6539 C   0  0  0  0  0  0
  105.6341   95.9902  100.2261 C   0  0  0  0  0  0
  106.2204   95.4835   99.0293 O   0  0  0  0  0  0
  106.0019   96.0962   97.8311 C   0  0  0  0  0  0
```

```
  106.7016   95.3865   96.6356 C   0  0  0  0  0  0
  105.3271   97.1175   97.6816 O   0  0  0  0  0  0
  106.4601   96.1585   95.3089 C   0  0  0  0  0  0
  108.2225   95.2720   96.9309 C   0  0  0  0  0  0
  106.1301   93.9610   96.4702 C   0  0  0  0  0  0
  106.9055   97.6714  100.0098 H   0  0  0  0  0  0
  108.5782   96.8443  101.0788 H   0  0  0  0  0  0
  109.7832   95.6980  102.5543 H   0  0  0  0  0  0
  108.6471   97.1212  104.9057 H   0  0  0  0  0  0
  110.5584   97.3334  106.8602 H   0  0  0  0  0  0
  111.5789   95.9196  108.5773 H   0  0  0  0  0  0
  110.6870   92.4231  106.2435 H   0  0  0  0  0  0
  109.6998   93.8257  104.5039 H   0  0  0  0  0  0
  112.7828   92.9267  108.2604 H   0  0  0  0  0  0
  111.0474   93.9295  112.0985 H   0  0  0  0  0  0
  110.6707   95.0372  110.7609 H   0  0  0  0  0  0
  112.3635   94.5337  111.0566 H   0  0  0  0  0  0
  110.7681   98.3765  104.8524 H   0  0  0  0  0  0
  110.0755   98.3700  103.2576 H   0  0  0  0  0  0
  111.4218   97.2944  103.6360 H   0  0  0  0  0  0
  105.3705  102.8324   98.9827 H   0  0  0  0  0  0
  106.8622  102.6320  100.9417 H   0  0  0  0  0  0
  106.9704  100.5060  102.1913 H   0  0  0  0  0  0
  104.0839   98.7895   99.5024 H   0  0  0  0  0  0
  103.9700  100.9247   98.2704 H   0  0  0  0  0  0
  106.0766   98.3402  102.6437 H   0  0  0  0  0  0
  104.6452   97.7914  101.8506 H   0  0  0  0  0  0
  105.5102   95.1625  100.9217 H   0  0  0  0  0  0
  104.6255   96.3487  100.0121 H   0  0  0  0  0  0
  107.0796   95.7824   94.4945 H   0  0  0  0  0  0
  106.6561   97.2269   95.3995 H   0  0  0  0  0  0
  105.4219   96.0690   94.9808 H   0  0  0  0  0  0
  108.8038   95.0020   96.0538 H   0  0  0  0  0  0
  108.4260   94.4896   97.6566 H   0  0  0  0  0  0
  108.6520   96.1934   97.3203 H   0  0  0  0  0  0
  106.6375   93.4105   95.6720 H   0  0  0  0  0  0
  105.0642   93.9682   96.2321 H   0  0  0  0  0  0
  106.2558   93.3922   97.3926 H   0  0  0  0  0  0
  1  2  2  0  0  0
  1  3  2  0  0  0
  1 16  1  0  0  0
  1 17  1  0  0  0
  4  5  1  0  0  0
  4 25  1  0  0  0
  4 26  1  0  0  0
  4 34  1  0  0  0
  5  6  1  0  0  0
  5 35  1  0  0  0
  6  7  1  0  0  0
  6  8  2  0  0  0
  7  9  1  0  0  0
  7 36  1  0  0  0
  9 10  1  0  0  0
  9 18  1  0  0  0
  9 37  1  0  0  0
 10 11  2  0  0  0
 10 15  1  0  0  0
 11 12  1  0  0  0
 11 38  1  0  0  0
 12 13  2  0  0  0
 12 39  1  0  0  0
 13 14  1  0  0  0
 13 16  1  0  0  0
 14 15  2  0  0  0
 14 40  1  0  0  0
 15 41  1  0  0  0
 16 42  1  0  0  0
 17 43  1  0  0  0
 17 44  1  0  0  0
 17 45  1  0  0  0
 18 46  1  0  0  0
```

```
 18 47  1  0  0  0
 18 48  1  0  0  0
 19 20  2  0  0  0
 19 24  1  0  0  0
 19 49  1  0  0  0
 20 21  1  0  0  0
 20 50  1  0  0  0
 21 22  2  0  0  0
 21 51  1  0  0  0
 22 23  1  0  0  0
 22 25  1  0  0  0
 23 24  2  0  0  0
 23 52  1  0  0  0
 24 53  1  0  0  0
 25 54  1  0  0  0
 25 55  1  0  0  0
 26 27  1  0  0  0
 26 56  1  0  0  0
 26 57  1  0  0  0
 27 28  1  0  0  0
 28 29  1  0  0  0
 28 30  2  0  0  0
 29 31  1  0  0  0
 29 32  1  0  0  0
 29 33  1  0  0  0
 31 58  1  0  0  0
 31 59  1  0  0  0
 31 60  1  0  0  0
 32 61  1  0  0  0
 32 62  1  0  0  0
 32 63  1  0  0  0
 33 64  1  0  0  0
 33 65  1  0  0  0
 33 66  1  0  0  0
M  END
> <s_m_entry_id>
43

> <s_m_entry_name>
B_33.1

> <s_m_Source_Path>
C:\Users\julio\OneDrive - Universidad de Talca\Escritorio\Nueva carpeta

> <s_m_Source_File>
B_33.mol2

> <i_m_Source_File_Index>
1

$$$$
B_34
                    3D
 Structure written by MMmdl.
 66 67  0  0  1  0            999 V2000
  111.0831   92.8866  109.9331 S   0  0  0  0  0  0
  109.6513   92.7382  109.6595 O   0  0  0  0  0  0
  111.8562   91.7561  110.4571 O   0  0  0  0  0  0
  106.9770   96.5308  100.1023 C   0  0  1  0  0  0
  107.9595   96.1953  101.1588 N   0  0  0  0  0  0
  107.7551   96.2039  102.5079 C   0  0  0  0  0  0
  108.9165   95.9589  103.1874 N   0  0  0  0  0  0
  106.2837   96.4845  103.2565 S   0  0  0  0  0  0
  109.3356   96.6572  104.4208 C   0  0  1  0  0  0
  109.9695   95.7511  105.4985 C   0  0  0  0  0  0
  110.0530   94.3577  105.3500 C   0  0  0  0  0  0
  110.6562   93.5742  106.3356 C   0  0  0  0  0  0
  111.1920   94.1730  107.4764 C   0  0  0  0  0  0
  111.0938   95.5544  107.6460 C   0  0  0  0  0  0
  110.4749   96.3341  106.6706 C   0  0  0  0  0  0
  111.8556   93.3896  108.4283 N   0  0  0  0  0  0
```

```
 111.3814   94.3676  111.0507 C   0  0  0  0  0  0
 110.2782   97.8155  104.0183 C   0  0  0  0  0  0
 104.6649  100.9999  101.2094 C   0  0  0  0  0  0
 105.7812  100.5726  101.9255 C   0  0  0  0  0  0
 106.5995   99.5828  101.3915 C   0  0  0  0  0  0
 106.3066   99.0300  100.1406 C   0  0  0  0  0  0
 105.1846   99.4623   99.4299 C   0  0  0  0  0  0
 104.3613  100.4502   99.9643 C   0  0  0  0  0  0
 107.2159   97.9644   99.5437 C   0  0  0  0  0  0
 107.0560   95.4738   98.9814 C   0  0  0  0  0  0
 105.9714   95.6098   98.0646 O   0  0  0  0  0  0
 106.0768   95.1257   96.8010 C   0  0  0  0  0  0
 104.7922   95.2952   95.9348 C   0  0  0  0  0  0
 107.0872   94.5876   96.3413 O   0  0  0  0  0  0
 104.9639   96.5598   95.0574 C   0  0  0  0  0  0
 104.6105   94.0613   95.0090 C   0  0  0  0  0  0
 103.5115   95.4497   96.7997 C   0  0  0  0  0  0
 105.9603   96.4655  100.4904 H   0  0  0  0  0  0
 108.8947   95.9866  100.8392 H   0  0  0  0  0  0
 109.6655   95.5230  102.6640 H   0  0  0  0  0  0
 108.4671   97.1001  104.9137 H   0  0  0  0  0  0
 109.6642   93.8671  104.4694 H   0  0  0  0  0  0
 110.7186   92.5035  106.1986 H   0  0  0  0  0  0
 111.5034   96.0275  108.5288 H   0  0  0  0  0  0
 110.4047   97.3974  106.8322 H   0  0  0  0  0  0
 112.7770   93.0294  108.2245 H   0  0  0  0  0  0
 111.0537   94.1124  112.0576 H   0  0  0  0  0  0
 110.8039   95.2154  110.6867 H   0  0  0  0  0  0
 112.4437   94.6023  111.0579 H   0  0  0  0  0  0
 110.5189   98.4659  104.8555 H   0  0  0  0  0  0
 109.8177   98.4548  103.2629 H   0  0  0  0  0  0
 111.2163   97.4398  103.6094 H   0  0  0  0  0  0
 104.0253  101.7692  101.6196 H   0  0  0  0  0  0
 106.0052  101.0038  102.8901 H   0  0  0  0  0  0
 107.4555   99.2397  101.9535 H   0  0  0  0  0  0
 104.9469   99.0370   98.4646 H   0  0  0  0  0  0
 103.4937  100.7904   99.4155 H   0  0  0  0  0  0
 107.0748   97.9827   98.4611 H   0  0  0  0  0  0
 108.2567   98.2672   99.6666 H   0  0  0  0  0  0
 108.0104   95.5795   98.4573 H   0  0  0  0  0  0
 107.0215   94.4692   99.4035 H   0  0  0  0  0  0
 104.0928   96.7221   94.4182 H   0  0  0  0  0  0
 105.8326   96.4843   94.3996 H   0  0  0  0  0  0
 105.0867   97.4607   95.6610 H   0  0  0  0  0  0
 103.7189   94.1563   94.3883 H   0  0  0  0  0  0
 104.5064   93.1369   95.5803 H   0  0  0  0  0  0
 105.4552   93.9289   94.3310 H   0  0  0  0  0  0
 102.6194   95.5280   96.1796 H   0  0  0  0  0  0
 103.5409   96.3442   97.4228 H   0  0  0  0  0  0
 103.3660   94.5950   97.4611 H   0  0  0  0  0  0
  1  2  2  0  0  0
  1  3  2  0  0  0
  1 16  1  0  0  0
  1 17  1  0  0  0
  4  5  1  0  0  0
  4 25  1  0  0  0
  4 26  1  0  0  0
  4 34  1  0  0  0
  5  6  1  0  0  0
  5 35  1  0  0  0
  6  7  1  0  0  0
  6  8  2  0  0  0
  7  9  1  0  0  0
  7 36  1  0  0  0
  9 10  1  0  0  0
  9 18  1  0  0  0
  9 37  1  0  0  0
 10 11  2  0  0  0
 10 15  1  0  0  0
 11 12  1  0  0  0
 11 38  1  0  0  0
```

```
 12 13  2  0  0  0
 12 39  1  0  0  0
 13 14  1  0  0  0
 13 16  1  0  0  0
 14 15  2  0  0  0
 14 40  1  0  0  0
 15 41  1  0  0  0
 16 42  1  0  0  0
 17 43  1  0  0  0
 17 44  1  0  0  0
 17 45  1  0  0  0
 18 46  1  0  0  0
 18 47  1  0  0  0
 18 48  1  0  0  0
 19 20  2  0  0  0
 19 24  1  0  0  0
 19 49  1  0  0  0
 20 21  1  0  0  0
 20 50  1  0  0  0
 21 22  2  0  0  0
 21 51  1  0  0  0
 22 23  1  0  0  0
 22 25  1  0  0  0
 23 24  2  0  0  0
 23 52  1  0  0  0
 24 53  1  0  0  0
 25 54  1  0  0  0
 25 55  1  0  0  0
 26 27  1  0  0  0
 26 56  1  0  0  0
 26 57  1  0  0  0
 27 28  1  0  0  0
 28 29  1  0  0  0
 28 30  2  0  0  0
 29 31  1  0  0  0
 29 32  1  0  0  0
 29 33  1  0  0  0
 31 58  1  0  0  0
 31 59  1  0  0  0
 31 60  1  0  0  0
 32 61  1  0  0  0
 32 62  1  0  0  0
 32 63  1  0  0  0
 33 64  1  0  0  0
 33 65  1  0  0  0
 33 66  1  0  0  0
M  END
> <s_m_entry_id>
44

> <s_m_entry_name>
B_34.1

> <s_m_Source_Path>
C:\Users\julio\OneDrive - Universidad de Talca\Escritorio\Nueva carpeta

> <s_m_Source_File>
B_34.mol2

> <i_m_Source_File_Index>
1

$$$$
```

Coordinates of TRPV1 in PDB format in conformation for docking of compounds from series B.

```
ATOM      1  N   LYS B 571     132.999 108.143 111.393  1.00  0.00           N1+
ATOM      2  CA  LYS B 571     132.237 107.198 112.281  1.00  0.00           C
ATOM      3  C   LYS B 571     130.841 106.931 111.662  1.00  0.00           C
ATOM      4  O   LYS B 571     130.319 105.841 111.899  1.00  0.00           O
ATOM      5  CB  LYS B 571     132.181 107.669 113.753  1.00 20.00           C
ATOM      6  CG  LYS B 571     133.553 107.804 114.450  1.00 20.00           C
ATOM      7  CD  LYS B 571     134.373 106.500 114.501  1.00 20.00           C
ATOM      8  CE  LYS B 571     135.658 106.640 115.331  1.00 20.00           C
ATOM      9  NZ  LYS B 571     136.437 105.389 115.338  1.00 20.00           N1+
ATOM     10  H   LYS B 571     132.562 109.054 111.412  1.00  0.00           H
ATOM     11  HA  LYS B 571     132.804 106.270 112.199  1.00  0.00           H
ATOM     12  HB2 LYS B 571     131.668 108.631 113.809  1.00  0.00           H
ATOM     13  HB3 LYS B 571     131.569 106.981 114.336  1.00  0.00           H
ATOM     14  HG2 LYS B 571     134.138 108.583 113.960  1.00  0.00           H
ATOM     15  HG3 LYS B 571     133.386 108.159 115.468  1.00  0.00           H
ATOM     16  HD2 LYS B 571     133.757 105.697 114.908  1.00  0.00           H
ATOM     17  HD3 LYS B 571     134.647 106.196 113.491  1.00  0.00           H
ATOM     18  HE2 LYS B 571     136.283 107.438 114.929  1.00  0.00           H
ATOM     19  HE3 LYS B 571     135.417 106.909 116.361  1.00  0.00           H
ATOM     20  HZ1 LYS B 571     135.885 104.644 115.737  1.00  0.00           H
ATOM     21  HZ2 LYS B 571     136.702 105.146 114.395  1.00  0.00           H
ATOM     22  HZ3 LYS B 571     137.272 105.517 115.894  1.00  0.00           H
ATOM     23  H1  LYS B 571     133.028 107.779 110.451  1.00  0.00           H
ATOM     24  H2  LYS B 571     133.942 108.224 111.746  1.00  0.00           H
ATOM     25  N   MET B 572     130.260 107.871 110.887  1.00  0.00           N
ATOM     26  CA  MET B 572     128.999 107.686 110.156  1.00  0.00           C
ATOM     27  C   MET B 572     129.123 106.755 108.934  1.00  0.00           C
ATOM     28  O   MET B 572     128.113 106.195 108.522  1.00  0.00           O
ATOM     29  CB  MET B 572     128.429 109.054 109.725  1.00 20.00           C
ATOM     30  CG  MET B 572     128.109 110.008 110.884  1.00 20.00           C
ATOM     31  SD  MET B 572     126.807 109.437 112.008  1.00 20.00           S
ATOM     32  CE  MET B 572     126.837 110.808 113.186  1.00 20.00           C
ATOM     33  H   MET B 572     130.704 108.767 110.752  1.00  0.00           H
ATOM     34  HA  MET B 572     128.282 107.215 110.832  1.00  0.00           H
ATOM     35  HB2 MET B 572     129.126 109.545 109.044  1.00  0.00           H
ATOM     36  HB3 MET B 572     127.513 108.902 109.150  1.00  0.00           H
ATOM     37  HG2 MET B 572     129.006 110.210 111.470  1.00  0.00           H
ATOM     38  HG3 MET B 572     127.793 110.968 110.475  1.00  0.00           H
ATOM     39  HE1 MET B 572     126.007 110.729 113.887  1.00  0.00           H
ATOM     40  HE2 MET B 572     127.771 110.807 113.748  1.00  0.00           H
ATOM     41  HE3 MET B 572     126.761 111.755 112.654  1.00  0.00           H
ATOM     42  N   ILE B 573     130.340 106.575 108.393  1.00  0.00           N
ATOM     43  CA  ILE B 573     130.639 105.586 107.350  1.00  0.00           C
ATOM     44  C   ILE B 573     130.785 104.165 107.943  1.00  0.00           C
ATOM     45  O   ILE B 573     130.444 103.191 107.277  1.00  0.00           O
ATOM     46  CB  ILE B 573     131.929 105.975 106.561  1.00 20.00           C
ATOM     47  CG1 ILE B 573     131.698 107.282 105.764  1.00 20.00           C
ATOM     48  CG2 ILE B 573     132.475 104.876 105.617  1.00 20.00           C
ATOM     49  CD1 ILE B 573     132.983 107.922 105.216  1.00 20.00           C
ATOM     50  H   ILE B 573     131.126 107.096 108.750  1.00  0.00           H
ATOM     51  HA  ILE B 573     129.807 105.561 106.643  1.00  0.00           H
ATOM     52  HB  ILE B 573     132.710 106.178 107.296  1.00  0.00           H
ATOM     53 HG12 ILE B 573     131.016 107.083 104.936  1.00  0.00           H
ATOM     54 HG13 ILE B 573     131.195 108.024 106.383  1.00  0.00           H
ATOM     55 HG21 ILE B 573     133.337 105.227 105.049  1.00  0.00           H
ATOM     56 HG22 ILE B 573     132.814 103.993 106.157  1.00  0.00           H
ATOM     57 HG23 ILE B 573     131.717 104.560 104.899  1.00  0.00           H
ATOM     58 HD11 ILE B 573     132.870 109.003 105.124  1.00  0.00           H
ATOM     59 HD12 ILE B 573     133.838 107.736 105.866  1.00  0.00           H
```

```
ATOM     60 HD13 ILE B 573     133.224 107.534 104.226  1.00  0.00           H
ATOM     61  N   LEU B 574     131.249 104.070 109.199  1.00  0.00           N
ATOM     62  CA  LEU B 574     131.428 102.807 109.920  1.00  0.00           C
ATOM     63  C   LEU B 574     130.137 102.301 110.599  1.00  0.00           C
ATOM     64  O   LEU B 574     130.175 101.233 111.207  1.00  0.00           O
ATOM     65  CB  LEU B 574     132.584 102.975 110.934  1.00 20.00           C
ATOM     66  CG  LEU B 574     133.954 103.309 110.293  1.00 20.00           C
ATOM     67  CD1 LEU B 574     135.016 103.605 111.372  1.00 20.00           C
ATOM     68  CD2 LEU B 574     134.423 102.223 109.299  1.00 20.00           C
ATOM     69  H   LEU B 574     131.484 104.909 109.707  1.00  0.00           H
ATOM     70  HA  LEU B 574     131.707 102.030 109.207  1.00  0.00           H
ATOM     71  HB2 LEU B 574     132.316 103.758 111.645  1.00  0.00           H
ATOM     72  HB3 LEU B 574     132.693 102.063 111.524  1.00  0.00           H
ATOM     73  HG  LEU B 574     133.840 104.233 109.725  1.00  0.00           H
ATOM     74 HD11 LEU B 574     135.497 104.565 111.187  1.00  0.00           H
ATOM     75 HD12 LEU B 574     134.581 103.651 112.371  1.00  0.00           H
ATOM     76 HD13 LEU B 574     135.800 102.849 111.406  1.00  0.00           H
ATOM     77 HD21 LEU B 574     135.494 102.029 109.363  1.00  0.00           H
ATOM     78 HD22 LEU B 574     133.916 101.272 109.463  1.00  0.00           H
ATOM     79 HD23 LEU B 574     134.219 102.529 108.272  1.00  0.00           H
ATOM     80  N   ARG B 575     129.028 103.055 110.485  1.00  0.00           N
ATOM     81  CA  ARG B 575     127.718 102.719 111.042  1.00  0.00           C
ATOM     82  C   ARG B 575     126.643 102.862 109.954  1.00  0.00           C
ATOM     83  O   ARG B 575     126.082 101.854 109.524  1.00  0.00           O
ATOM     84  CB  ARG B 575     127.413 103.615 112.267  1.00 20.00           C
ATOM     85  CG  ARG B 575     128.326 103.359 113.477  1.00 20.00           C
ATOM     86  CD  ARG B 575     128.060 104.343 114.628  1.00 20.00           C
ATOM     87  NE  ARG B 575     128.754 103.946 115.863  1.00 20.00           N
ATOM     88  CZ  ARG B 575     130.081 103.956 116.082  1.00 20.00           C
ATOM     89  NH1 ARG B 575     130.943 104.387 115.151  1.00 20.00           N
ATOM     90  NH2 ARG B 575     130.551 103.527 117.260  1.00 20.00           N1+
ATOM     91  H   ARG B 575     129.075 103.916 109.958  1.00  0.00           H
ATOM     92  HA  ARG B 575     127.701 101.677 111.369  1.00  0.00           H
ATOM     93  HB2 ARG B 575     127.477 104.668 111.985  1.00  0.00           H
ATOM     94  HB3 ARG B 575     126.381 103.448 112.581  1.00  0.00           H
ATOM     95  HG2 ARG B 575     128.283 102.324 113.818  1.00  0.00           H
ATOM     96  HG3 ARG B 575     129.347 103.523 113.137  1.00  0.00           H
ATOM     97  HD2 ARG B 575     128.467 105.321 114.368  1.00  0.00           H
ATOM     98  HD3 ARG B 575     126.991 104.491 114.787  1.00  0.00           H
ATOM     99  HE  ARG B 575     128.155 103.585 116.593  1.00  0.00           H
ATOM    100 HH11 ARG B 575     131.937 104.377 115.326  1.00  0.00           H
ATOM    101 HH12 ARG B 575     130.595 104.705 114.257  1.00  0.00           H
ATOM    102 HH21 ARG B 575     131.542 103.524 117.449  1.00  0.00           H
ATOM    103 HH22 ARG B 575     129.916 103.196 117.973  1.00  0.00           H
ATOM    104  N   ASP B 576     126.375 104.113 109.543  1.00 30.00           N
ATOM    105  CA  ASP B 576     125.278 104.532 108.659  1.00 30.00           C
ATOM    106  C   ASP B 576     125.623 104.421 107.156  1.00 30.00           C
ATOM    107  O   ASP B 576     125.101 105.195 106.353  1.00 30.00           O
ATOM    108  CB  ASP B 576     124.713 105.942 109.007  1.00 20.00           C
ATOM    109  CG  ASP B 576     124.424 106.238 110.487  1.00 20.00           C
ATOM    110  OD1 ASP B 576     124.500 105.310 111.322  1.00 20.00           O
ATOM    111  OD2 ASP B 576     124.086 107.410 110.757  1.00 20.00           O1-
ATOM    112  H   ASP B 576     126.910 104.877 109.932  1.00  0.00           H
ATOM    113  HA  ASP B 576     124.455 103.831 108.813  1.00  0.00           H
ATOM    114  HB2 ASP B 576     125.417 106.699 108.658  1.00  0.00           H
ATOM    115  HB3 ASP B 576     123.777 106.085 108.469  1.00  0.00           H
ATOM    116  N   LEU B 577     126.461 103.438 106.795  1.00  0.00           N
ATOM    117  CA  LEU B 577     126.728 103.021 105.418  1.00  0.00           C
ATOM    118  C   LEU B 577     126.727 101.489 105.356  1.00  0.00           C
ATOM    119  O   LEU B 577     126.145 100.945 104.423  1.00  0.00           O
ATOM    120  CB  LEU B 577     128.017 103.690 104.886  1.00 20.00           C
ATOM    121  CG  LEU B 577     128.351 103.482 103.386  1.00 20.00           C
ATOM    122  CD1 LEU B 577     129.297 104.593 102.874  1.00 20.00           C
ATOM    123  CD2 LEU B 577     128.915 102.077 103.070  1.00 20.00           C
ATOM    124  H   LEU B 577     126.857 102.858 107.519  1.00  0.00           H
ATOM    125  HA  LEU B 577     125.902 103.351 104.784  1.00  0.00           H
ATOM    126  HB2 LEU B 577     127.901 104.760 105.066  1.00  0.00           H
ATOM    127  HB3 LEU B 577     128.870 103.384 105.480  1.00  0.00           H
ATOM    128  HG  LEU B 577     127.420 103.599 102.831  1.00  0.00           H
ATOM    129 HD11 LEU B 577     128.856 105.111 102.021  1.00  0.00           H
ATOM    130 HD12 LEU B 577     129.503 105.348 103.633  1.00  0.00           H
```

```
ATOM    131 HD13 LEU B 577     130.267 104.213 102.553  1.00  0.00           H
ATOM    132 HD21 LEU B 577     128.157 101.451 102.599  1.00  0.00           H
ATOM    133 HD22 LEU B 577     129.763 102.102 102.385  1.00  0.00           H
ATOM    134 HD23 LEU B 577     129.252 101.560 103.969  1.00  0.00           H
ATOM    135  N   CYS B 578     127.300 100.813 106.369  1.00  0.00           N
ATOM    136  CA  CYS B 578     127.218  99.359 106.547  1.00  0.00           C
ATOM    137  C   CYS B 578     125.758  98.882 106.715  1.00  0.00           C
ATOM    138  O   CYS B 578     125.352  97.921 106.060  1.00  0.00           O
ATOM    139  CB  CYS B 578     128.122  98.898 107.714  1.00 20.00           C
ATOM    140  SG  CYS B 578     128.085  97.100 107.988  1.00 20.00           S
ATOM    141  H   CYS B 578     127.786 101.319 107.094  1.00  0.00           H
ATOM    142  HA  CYS B 578     127.603  98.904 105.633  1.00  0.00           H
ATOM    143  HB2 CYS B 578     129.155  99.189 107.520  1.00  0.00           H
ATOM    144  HB3 CYS B 578     127.828  99.392 108.641  1.00  0.00           H
ATOM    145  HG  CYS B 578     128.690  96.768 106.843  1.00  0.00           H
ATOM    146  N   ARG B 579     125.003  99.593 107.567  1.00  0.00           N
ATOM    147  CA  ARG B 579     123.612  99.292 107.899  1.00  0.00           C
ATOM    148  C   ARG B 579     122.611  99.923 106.914  1.00  0.00           C
ATOM    149  O   ARG B 579     121.562  99.321 106.685  1.00  0.00           O
ATOM    150  CB  ARG B 579     123.343  99.719 109.359  1.00 20.00           C
ATOM    151  CG  ARG B 579     124.300  99.046 110.370  1.00 20.00           C
ATOM    152  CD  ARG B 579     124.058  99.420 111.839  1.00 20.00           C
ATOM    153  NE  ARG B 579     124.096 100.871 112.070  1.00 20.00           N
ATOM    154  CZ  ARG B 579     123.022 101.667 112.191  1.00 20.00           C
ATOM    155  NH1 ARG B 579     121.780 101.168 112.163  1.00 20.00           N
ATOM    156  NH2 ARG B 579     123.182 102.987 112.337  1.00 20.00           N1+
ATOM    157  H   ARG B 579     125.415 100.373 108.061  1.00  0.00           H
ATOM    158  HA  ARG B 579     123.471  98.213 107.839  1.00  0.00           H
ATOM    159  HB2 ARG B 579     123.439 100.803 109.431  1.00  0.00           H
ATOM    160  HB3 ARG B 579     122.312  99.484 109.629  1.00  0.00           H
ATOM    161  HG2 ARG B 579     124.103  97.978 110.284  1.00  0.00           H
ATOM    162  HG3 ARG B 579     125.356  99.169 110.126  1.00  0.00           H
ATOM    163  HD2 ARG B 579     123.224  98.895 112.304  1.00  0.00           H
ATOM    164  HD3 ARG B 579     124.944  99.081 112.376  1.00  0.00           H
ATOM    165  HE  ARG B 579     125.011 101.297 112.056  1.00  0.00           H
ATOM    166 HH11 ARG B 579     120.979 101.779 112.237  1.00  0.00           H
ATOM    167 HH12 ARG B 579     121.637 100.174 112.056  1.00  0.00           H
ATOM    168 HH21 ARG B 579     124.092 103.426 112.264  1.00  0.00           H
ATOM    169 HH22 ARG B 579     122.380 103.592 112.431  1.00  0.00           H
ATOM    170  N   PHE B 580     122.948 101.091 106.332  1.00  0.00           N
ATOM    171  CA  PHE B 580     122.114 101.774 105.337  1.00  0.00           C
ATOM    172  C   PHE B 580     122.156 101.117 103.947  1.00  0.00           C
ATOM    173  O   PHE B 580     121.092 100.855 103.387  1.00  0.00           O
ATOM    174  CB  PHE B 580     122.425 103.290 105.295  1.00 20.00           C
ATOM    175  CG  PHE B 580     121.755 104.076 104.173  1.00 20.00           C
ATOM    176  CD1 PHE B 580     120.384 104.403 104.248  1.00 20.00           C
ATOM    177  CD2 PHE B 580     122.448 104.337 102.971  1.00 20.00           C
ATOM    178  CE1 PHE B 580     119.747 105.009 103.173  1.00 20.00           C
ATOM    179  CE2 PHE B 580     121.795 104.943 101.906  1.00 20.00           C
ATOM    180  CZ  PHE B 580     120.451 105.279 102.007  1.00 20.00           C
ATOM    181  H   PHE B 580     123.829 101.527 106.558  1.00  0.00           H
ATOM    182  HA  PHE B 580     121.078 101.694 105.674  1.00  0.00           H
ATOM    183  HB2 PHE B 580     122.118 103.737 106.241  1.00  0.00           H
ATOM    184  HB3 PHE B 580     123.501 103.438 105.225  1.00  0.00           H
ATOM    185  HD1 PHE B 580     119.820 104.182 105.138  1.00  0.00           H
ATOM    186  HD2 PHE B 580     123.487 104.061 102.874  1.00  0.00           H
ATOM    187  HE1 PHE B 580     118.700 105.265 103.242  1.00  0.00           H
ATOM    188  HE2 PHE B 580     122.331 105.146 100.991  1.00  0.00           H
ATOM    189  HZ  PHE B 580     119.949 105.746 101.173  1.00  0.00           H
ATOM    190  N   MET B 581     123.369 100.875 103.412  1.00  0.00           N
ATOM    191  CA  MET B 581     123.558 100.331 102.062  1.00  0.00           C
ATOM    192  C   MET B 581     123.152  98.862 101.915  1.00  0.00           C
ATOM    193  O   MET B 581     122.818  98.485 100.797  1.00  0.00           O
ATOM    194  CB  MET B 581     124.987 100.557 101.526  1.00 20.00           C
ATOM    195  CG  MET B 581     125.370 102.034 101.338  1.00 20.00           C
ATOM    196  SD  MET B 581     124.383 102.961 100.124  1.00 20.00           S
ATOM    197  CE  MET B 581     124.843 102.121  98.585  1.00 20.00           C
ATOM    198  H   MET B 581     124.211 101.092 103.929  1.00  0.00           H
ATOM    199  HA  MET B 581     122.889 100.886 101.407  1.00  0.00           H
ATOM    200  HB2 MET B 581     125.714 100.059 102.165  1.00  0.00           H
ATOM    201  HB3 MET B 581     125.094 100.058 100.563  1.00  0.00           H
```

ATOM	202	HG2	MET	B	581	125.299	102.555	102.292	1.00	0.00	H
ATOM	203	HG3	MET	B	581	126.414	102.100	101.034	1.00	0.00	H
ATOM	204	HE1	MET	B	581	124.396	102.634	97.733	1.00	0.00	H
ATOM	205	HE2	MET	B	581	125.925	102.119	98.457	1.00	0.00	H
ATOM	206	HE3	MET	B	581	124.488	101.091	98.583	1.00	0.00	H
ATOM	207	N	PHE	B	582	123.120	98.073	103.007	1.00	0.00	N
ATOM	208	CA	PHE	B	582	122.515	96.736	102.990	1.00	0.00	C
ATOM	209	C	PHE	B	582	121.020	96.783	102.626	1.00	0.00	C
ATOM	210	O	PHE	B	582	120.595	96.077	101.712	1.00	0.00	O
ATOM	211	CB	PHE	B	582	122.785	95.966	104.306	1.00	20.00	C
ATOM	212	CG	PHE	B	582	122.015	94.655	104.436	1.00	20.00	C
ATOM	213	CD1	PHE	B	582	122.435	93.519	103.711	1.00	20.00	C
ATOM	214	CD2	PHE	B	582	120.759	94.622	105.083	1.00	20.00	C
ATOM	215	CE1	PHE	B	582	121.640	92.380	103.676	1.00	20.00	C
ATOM	216	CE2	PHE	B	582	119.973	93.479	105.024	1.00	20.00	C
ATOM	217	CZ	PHE	B	582	120.413	92.361	104.328	1.00	20.00	C
ATOM	218	H	PHE	B	582	123.418	98.431	103.903	1.00	0.00	H
ATOM	219	HA	PHE	B	582	123.006	96.173	102.193	1.00	0.00	H
ATOM	220	HB2	PHE	B	582	123.850	95.738	104.368	1.00	0.00	H
ATOM	221	HB3	PHE	B	582	122.563	96.597	105.169	1.00	0.00	H
ATOM	222	HD1	PHE	B	582	123.366	93.538	103.164	1.00	0.00	H
ATOM	223	HD2	PHE	B	582	120.390	95.497	105.598	1.00	0.00	H
ATOM	224	HE1	PHE	B	582	121.967	91.513	103.122	1.00	0.00	H
ATOM	225	HE2	PHE	B	582	119.006	93.466	105.503	1.00	0.00	H
ATOM	226	HZ	PHE	B	582	119.793	91.478	104.283	1.00	0.00	H
ATOM	227	N	VAL	B	583	120.266	97.651	103.319	1.00	0.00	N
ATOM	228	CA	VAL	B	583	118.828	97.803	103.113	1.00	0.00	C
ATOM	229	C	VAL	B	583	118.527	98.488	101.766	1.00	0.00	C
ATOM	230	O	VAL	B	583	117.678	97.998	101.020	1.00	0.00	O
ATOM	231	CB	VAL	B	583	118.159	98.606	104.266	1.00	20.00	C
ATOM	232	CG1	VAL	B	583	116.642	98.818	104.077	1.00	20.00	C
ATOM	233	CG2	VAL	B	583	118.403	97.938	105.633	1.00	20.00	C
ATOM	234	H	VAL	B	583	120.685	98.251	104.015	1.00	0.00	H
ATOM	235	HA	VAL	B	583	118.385	96.806	103.081	1.00	0.00	H
ATOM	236	HB	VAL	B	583	118.615	99.598	104.306	1.00	0.00	H
ATOM	237	HG11	VAL	B	583	116.209	99.342	104.925	1.00	0.00	H
ATOM	238	HG12	VAL	B	583	116.407	99.408	103.193	1.00	0.00	H
ATOM	239	HG13	VAL	B	583	116.119	97.868	103.978	1.00	0.00	H
ATOM	240	HG21	VAL	B	583	117.921	98.495	106.437	1.00	0.00	H
ATOM	241	HG22	VAL	B	583	118.010	96.921	105.654	1.00	0.00	H
ATOM	242	HG23	VAL	B	583	119.464	97.888	105.877	1.00	0.00	H
ATOM	243	N	TYR	B	584	119.267	99.562	101.443	1.00	0.00	N
ATOM	244	CA	TYR	B	584	119.113	100.265	100.165	1.00	0.00	C
ATOM	245	C	TYR	B	584	119.370	99.368	98.939	1.00	0.00	C
ATOM	246	O	TYR	B	584	118.585	99.437	97.995	1.00	0.00	O
ATOM	247	CB	TYR	B	584	119.956	101.564	100.125	1.00	20.00	C
ATOM	248	CG	TYR	B	584	119.938	102.252	98.766	1.00	20.00	C
ATOM	249	CD1	TYR	B	584	118.744	102.842	98.305	1.00	20.00	C
ATOM	250	CD2	TYR	B	584	121.060	102.189	97.913	1.00	20.00	C
ATOM	251	CE1	TYR	B	584	118.647	103.303	96.978	1.00	20.00	C
ATOM	252	CE2	TYR	B	584	120.969	102.664	96.590	1.00	20.00	C
ATOM	253	CZ	TYR	B	584	119.753	103.193	96.114	1.00	20.00	C
ATOM	254	OH	TYR	B	584	119.637	103.575	94.810	1.00	20.00	O
ATOM	255	H	TYR	B	584	119.992	99.902	102.062	1.00	0.00	H
ATOM	256	HA	TYR	B	584	118.065	100.569	100.115	1.00	0.00	H
ATOM	257	HB2	TYR	B	584	119.606	102.264	100.884	1.00	0.00	H
ATOM	258	HB3	TYR	B	584	120.990	101.336	100.377	1.00	0.00	H
ATOM	259	HD1	TYR	B	584	117.880	102.882	98.951	1.00	0.00	H
ATOM	260	HD2	TYR	B	584	121.976	101.728	98.253	1.00	0.00	H
ATOM	261	HE1	TYR	B	584	117.713	103.708	96.618	1.00	0.00	H
ATOM	262	HE2	TYR	B	584	121.821	102.583	95.930	1.00	0.00	H
ATOM	263	HH	TYR	B	584	118.730	103.768	94.557	1.00	0.00	H
ATOM	264	N	LEU	B	585	120.429	98.537	98.975	1.00	0.00	N
ATOM	265	CA	LEU	B	585	120.756	97.598	97.898	1.00	0.00	C
ATOM	266	C	LEU	B	585	119.734	96.459	97.752	1.00	0.00	C
ATOM	267	O	LEU	B	585	119.585	95.960	96.640	1.00	0.00	O
ATOM	268	CB	LEU	B	585	122.186	97.040	98.059	1.00	20.00	C
ATOM	269	CG	LEU	B	585	123.300	98.063	97.729	1.00	20.00	C
ATOM	270	CD1	LEU	B	585	124.679	97.560	98.210	1.00	20.00	C
ATOM	271	CD2	LEU	B	585	123.308	98.469	96.239	1.00	20.00	C
ATOM	272	H	LEU	B	585	121.045	98.531	99.778	1.00	0.00	H

ATOM	273	HA	LEU	B	585	120.721	98.158	96.966	1.00	0.00	H
ATOM	274	HB2	LEU	B	585	122.298	96.658	99.074	1.00	0.00	H
ATOM	275	HB3	LEU	B	585	122.326	96.173	97.410	1.00	0.00	H
ATOM	276	HG	LEU	B	585	123.089	98.970	98.295	1.00	0.00	H
ATOM	277	HD11	LEU	B	585	125.160	98.304	98.844	1.00	0.00	H
ATOM	278	HD12	LEU	B	585	124.599	96.646	98.799	1.00	0.00	H
ATOM	279	HD13	LEU	B	585	125.362	97.345	97.389	1.00	0.00	H
ATOM	280	HD21	LEU	B	585	124.293	98.387	95.780	1.00	0.00	H
ATOM	281	HD22	LEU	B	585	122.630	97.860	95.641	1.00	0.00	H
ATOM	282	HD23	LEU	B	585	122.994	99.508	96.127	1.00	0.00	H
ATOM	283	N	VAL	B	586	119.039	96.075	98.833	1.00	0.00	N
ATOM	284	CA	VAL	B	586	117.922	95.131	98.764	1.00	0.00	C
ATOM	285	C	VAL	B	586	116.719	95.743	98.011	1.00	0.00	C
ATOM	286	O	VAL	B	586	116.168	95.080	97.130	1.00	0.00	O
ATOM	287	CB	VAL	B	586	117.510	94.597	100.170	1.00	20.00	C
ATOM	288	CG1	VAL	B	586	116.120	93.934	100.240	1.00	20.00	C
ATOM	289	CG2	VAL	B	586	118.563	93.607	100.700	1.00	20.00	C
ATOM	290	H	VAL	B	586	119.226	96.495	99.735	1.00	0.00	H
ATOM	291	HA	VAL	B	586	118.254	94.274	98.173	1.00	0.00	H
ATOM	292	HB	VAL	B	586	117.493	95.437	100.865	1.00	0.00	H
ATOM	293	HG11	VAL	B	586	115.980	93.405	101.181	1.00	0.00	H
ATOM	294	HG12	VAL	B	586	115.316	94.666	100.160	1.00	0.00	H
ATOM	295	HG13	VAL	B	586	115.995	93.205	99.439	1.00	0.00	H
ATOM	296	HG21	VAL	B	586	118.373	93.339	101.740	1.00	0.00	H
ATOM	297	HG22	VAL	B	586	118.570	92.686	100.116	1.00	0.00	H
ATOM	298	HG23	VAL	B	586	119.569	94.023	100.656	1.00	0.00	H
ATOM	299	N	PHE	B	587	116.384	97.010	98.304	1.00	0.00	N
ATOM	300	CA	PHE	B	587	115.358	97.760	97.571	1.00	0.00	C
ATOM	301	C	PHE	B	587	115.733	97.942	96.090	1.00	0.00	C
ATOM	302	O	PHE	B	587	114.948	97.596	95.208	1.00	0.00	O
ATOM	303	CB	PHE	B	587	115.094	99.139	98.221	1.00	20.00	C
ATOM	304	CG	PHE	B	587	114.597	99.169	99.659	1.00	20.00	C
ATOM	305	CD1	PHE	B	587	113.864	98.097	100.218	1.00	20.00	C
ATOM	306	CD2	PHE	B	587	114.695	100.374	100.389	1.00	20.00	C
ATOM	307	CE1	PHE	B	587	113.309	98.219	101.483	1.00	20.00	C
ATOM	308	CE2	PHE	B	587	114.128	100.475	101.652	1.00	20.00	C
ATOM	309	CZ	PHE	B	587	113.447	99.398	102.203	1.00	20.00	C
ATOM	310	H	PHE	B	587	116.859	97.494	99.060	1.00	0.00	H
ATOM	311	HA	PHE	B	587	114.434	97.180	97.586	1.00	0.00	H
ATOM	312	HB2	PHE	B	587	115.996	99.751	98.171	1.00	0.00	H
ATOM	313	HB3	PHE	B	587	114.351	99.670	97.623	1.00	0.00	H
ATOM	314	HD1	PHE	B	587	113.714	97.175	99.679	1.00	0.00	H
ATOM	315	HD2	PHE	B	587	115.197	101.232	99.963	1.00	0.00	H
ATOM	316	HE1	PHE	B	587	112.747	97.394	101.890	1.00	0.00	H
ATOM	317	HE2	PHE	B	587	114.200	101.401	102.202	1.00	0.00	H
ATOM	318	HZ	PHE	B	587	113.001	99.487	103.183	1.00	0.00	H
ATOM	319	N	LEU	B	588	116.953	98.433	95.846	1.00	0.00	N
ATOM	320	CA	LEU	B	588	117.494	98.709	94.514	1.00	0.00	C
ATOM	321	C	LEU	B	588	117.531	97.453	93.633	1.00	0.00	C
ATOM	322	O	LEU	B	588	116.917	97.474	92.572	1.00	0.00	O
ATOM	323	CB	LEU	B	588	118.862	99.423	94.628	1.00	20.00	C
ATOM	324	CG	LEU	B	588	119.641	99.664	93.307	1.00	20.00	C
ATOM	325	CD1	LEU	B	588	118.834	100.458	92.256	1.00	20.00	C
ATOM	326	CD2	LEU	B	588	121.018	100.300	93.592	1.00	20.00	C
ATOM	327	H	LEU	B	588	117.582	98.588	96.624	1.00	0.00	H
ATOM	328	HA	LEU	B	588	116.804	99.404	94.035	1.00	0.00	H
ATOM	329	HB2	LEU	B	588	118.718	100.378	95.136	1.00	0.00	H
ATOM	330	HB3	LEU	B	588	119.497	98.831	95.283	1.00	0.00	H
ATOM	331	HG	LEU	B	588	119.855	98.689	92.868	1.00	0.00	H
ATOM	332	HD11	LEU	B	588	119.383	101.305	91.846	1.00	0.00	H
ATOM	333	HD12	LEU	B	588	118.569	99.818	91.414	1.00	0.00	H
ATOM	334	HD13	LEU	B	588	117.906	100.855	92.662	1.00	0.00	H
ATOM	335	HD21	LEU	B	588	121.113	101.304	93.183	1.00	0.00	H
ATOM	336	HD22	LEU	B	588	121.215	100.378	94.661	1.00	0.00	H
ATOM	337	HD23	LEU	B	588	121.819	99.697	93.162	1.00	0.00	H
ATOM	338	N	PHE	B	589	118.204	96.384	94.096	1.00	0.00	N
ATOM	339	CA	PHE	B	589	118.348	95.121	93.365	1.00	0.00	C
ATOM	340	C	PHE	B	589	117.020	94.356	93.205	1.00	0.00	C
ATOM	341	O	PHE	B	589	116.833	93.705	92.177	1.00	0.00	O
ATOM	342	CB	PHE	B	589	119.432	94.254	94.042	1.00	20.00	C
ATOM	343	CG	PHE	B	589	119.855	93.015	93.272	1.00	20.00	C

ATOM	344	CD1	PHE	B	589	120.761	93.138	92.197	1.00	20.00	C
ATOM	345	CD2	PHE	B	589	119.240	91.766	93.511	1.00	20.00	C
ATOM	346	CE1	PHE	B	589	121.086	92.028	91.432	1.00	20.00	C
ATOM	347	CE2	PHE	B	589	119.574	90.670	92.726	1.00	20.00	C
ATOM	348	CZ	PHE	B	589	120.497	90.798	91.696	1.00	20.00	C
ATOM	349	H	PHE	B	589	118.666	96.432	94.995	1.00	0.00	H
ATOM	350	HA	PHE	B	589	118.707	95.373	92.366	1.00	0.00	H
ATOM	351	HB2	PHE	B	589	120.328	94.859	94.190	1.00	0.00	H
ATOM	352	HB3	PHE	B	589	119.104	93.951	95.038	1.00	0.00	H
ATOM	353	HD1	PHE	B	589	121.208	94.095	91.970	1.00	0.00	H
ATOM	354	HD2	PHE	B	589	118.505	91.659	94.296	1.00	0.00	H
ATOM	355	HE1	PHE	B	589	121.796	92.123	90.625	1.00	0.00	H
ATOM	356	HE2	PHE	B	589	119.107	89.715	92.905	1.00	0.00	H
ATOM	357	HZ	PHE	B	589	120.748	89.940	91.090	1.00	0.00	H
ATOM	358	N	GLY	B	590	116.123	94.464	94.202	1.00	0.00	N
ATOM	359	CA	GLY	B	590	114.825	93.793	94.224	1.00	0.00	C
ATOM	360	C	GLY	B	590	113.881	94.360	93.160	1.00	0.00	C
ATOM	361	O	GLY	B	590	113.339	93.595	92.361	1.00	0.00	O
ATOM	362	H	GLY	B	590	116.357	95.019	95.015	1.00	0.00	H
ATOM	363	HA2	GLY	B	590	114.965	92.726	94.061	1.00	0.00	H
ATOM	364	HA3	GLY	B	590	114.374	93.923	95.207	1.00	0.00	H
ATOM	365	N	PHE	B	591	113.745	95.692	93.119	1.00	0.00	N
ATOM	366	CA	PHE	B	591	112.952	96.392	92.110	1.00	0.00	C
ATOM	367	C	PHE	B	591	113.619	96.406	90.724	1.00	0.00	C
ATOM	368	O	PHE	B	591	112.911	96.345	89.719	1.00	0.00	O
ATOM	369	CB	PHE	B	591	112.586	97.811	92.590	1.00	20.00	C
ATOM	370	CG	PHE	B	591	111.429	97.870	93.576	1.00	20.00	C
ATOM	371	CD1	PHE	B	591	110.108	98.006	93.100	1.00	20.00	C
ATOM	372	CD2	PHE	B	591	111.631	97.657	94.956	1.00	20.00	C
ATOM	373	CE1	PHE	B	591	109.049	98.044	93.996	1.00	20.00	C
ATOM	374	CE2	PHE	B	591	110.559	97.689	95.835	1.00	20.00	C
ATOM	375	CZ	PHE	B	591	109.275	97.908	95.359	1.00	20.00	C
ATOM	376	H	PHE	B	591	114.226	96.272	93.801	1.00	0.00	H
ATOM	377	HA	PHE	B	591	112.014	95.848	91.983	1.00	0.00	H
ATOM	378	HB2	PHE	B	591	113.457	98.296	93.032	1.00	0.00	H
ATOM	379	HB3	PHE	B	591	112.312	98.431	91.735	1.00	0.00	H
ATOM	380	HD1	PHE	B	591	109.918	98.125	92.043	1.00	0.00	H
ATOM	381	HD2	PHE	B	591	112.614	97.478	95.351	1.00	0.00	H
ATOM	382	HE1	PHE	B	591	108.045	98.185	93.626	1.00	0.00	H
ATOM	383	HE2	PHE	B	591	110.727	97.539	96.891	1.00	0.00	H
ATOM	384	HZ	PHE	B	591	108.451	97.950	96.055	1.00	0.00	H
ATOM	385	N	SER	B	592	114.960	96.431	90.677	1.00	0.00	N
ATOM	386	CA	SER	B	592	115.734	96.388	89.428	1.00	0.00	C
ATOM	387	C	SER	B	592	115.557	95.062	88.682	1.00	0.00	C
ATOM	388	O	SER	B	592	115.362	95.092	87.470	1.00	0.00	O
ATOM	389	CB	SER	B	592	117.224	96.709	89.671	1.00	20.00	C
ATOM	390	OG	SER	B	592	117.962	96.695	88.466	1.00	20.00	O
ATOM	391	H	SER	B	592	115.497	96.416	91.534	1.00	0.00	H
ATOM	392	HA	SER	B	592	115.355	97.170	88.773	1.00	0.00	H
ATOM	393	HB2	SER	B	592	117.337	97.703	90.101	1.00	0.00	H
ATOM	394	HB3	SER	B	592	117.669	95.995	90.363	1.00	0.00	H
ATOM	395	HG	SER	B	592	117.992	95.796	88.130	1.00	0.00	H
ATOM	396	N	THR	B	593	115.587	93.933	89.407	1.00	30.00	N
ATOM	397	CA	THR	B	593	115.307	92.621	88.828	1.00	30.00	C
ATOM	398	C	THR	B	593	113.830	92.493	88.398	1.00	30.00	C
ATOM	399	O	THR	B	593	113.569	91.947	87.330	1.00	30.00	O
ATOM	400	CB	THR	B	593	115.643	91.461	89.798	1.00	20.00	C
ATOM	401	CG2	THR	B	593	115.477	90.058	89.189	1.00	20.00	C
ATOM	402	OG1	THR	B	593	116.989	91.562	90.220	1.00	20.00	O
ATOM	403	H	THR	B	593	115.755	93.968	90.404	1.00	0.00	H
ATOM	404	HA	THR	B	593	115.928	92.507	87.936	1.00	0.00	H
ATOM	405	HB	THR	B	593	115.022	91.544	90.690	1.00	0.00	H
ATOM	406	HG1	THR	B	593	117.072	92.317	90.811	1.00	0.00	H
ATOM	407	HG21	THR	B	593	115.673	89.294	89.938	1.00	0.00	H
ATOM	408	HG22	THR	B	593	114.476	89.872	88.806	1.00	0.00	H
ATOM	409	HG23	THR	B	593	116.167	89.909	88.358	1.00	0.00	H
ATOM	410	N	ALA	B	594	112.901	93.043	89.201	1.00	30.00	N
ATOM	411	CA	ALA	B	594	111.465	93.033	88.919	1.00	30.00	C
ATOM	412	C	ALA	B	594	111.071	93.805	87.646	1.00	30.00	C
ATOM	413	O	ALA	B	594	110.216	93.319	86.910	1.00	30.00	O
ATOM	414	CB	ALA	B	594	110.700	93.556	90.141	1.00	30.00	C

```
ATOM    415  H   ALA B 594     113.183  93.487  90.064  1.00  0.00           H
ATOM    416  HA  ALA B 594     111.175  91.990  88.774  1.00  0.00           H
ATOM    417  HB1 ALA B 594     109.627  93.423  90.014  1.00  0.00           H
ATOM    418  HB2 ALA B 594     110.984  93.018  91.045  1.00  0.00           H
ATOM    419  HB3 ALA B 594     110.884  94.617  90.309  1.00  0.00           H
ATOM    420  N   VAL B 595     111.709  94.960  87.378  1.00 30.00           N
ATOM    421  CA  VAL B 595     111.429  95.763  86.183  1.00 30.00           C
ATOM    422  C   VAL B 595     112.113  95.219  84.903  1.00 30.00           C
ATOM    423  O   VAL B 595     111.562  95.430  83.823  1.00 30.00           O
ATOM    424  CB  VAL B 595     111.753  97.272  86.401  1.00 20.00           C
ATOM    425  CG1 VAL B 595     113.250  97.615  86.424  1.00 20.00           C
ATOM    426  CG2 VAL B 595     111.028  98.187  85.396  1.00 20.00           C
ATOM    427  H   VAL B 595     112.399  95.323  88.022  1.00  0.00           H
ATOM    428  HA  VAL B 595     110.354  95.704  86.000  1.00  0.00           H
ATOM    429  HB  VAL B 595     111.361  97.534  87.385  1.00  0.00           H
ATOM    430 HG11 VAL B 595     113.407  98.668  86.662  1.00  0.00           H
ATOM    431 HG12 VAL B 595     113.764  97.034  87.182  1.00  0.00           H
ATOM    432 HG13 VAL B 595     113.731  97.422  85.466  1.00  0.00           H
ATOM    433 HG21 VAL B 595     111.185  99.239  85.639  1.00  0.00           H
ATOM    434 HG22 VAL B 595     111.380  98.035  84.376  1.00  0.00           H
ATOM    435 HG23 VAL B 595     109.952  98.009  85.409  1.00  0.00           H
ATOM    436  N   VAL B 596     113.234  94.476  85.030  1.00 30.00           N
ATOM    437  CA  VAL B 596     113.863  93.745  83.916  1.00 30.00           C
ATOM    438  C   VAL B 596     112.946  92.658  83.337  1.00 30.00           C
ATOM    439  O   VAL B 596     112.827  92.570  82.118  1.00 30.00           O
ATOM    440  CB  VAL B 596     115.201  93.045  84.309  1.00 20.00           C
ATOM    441  CG1 VAL B 596     115.690  91.945  83.340  1.00 20.00           C
ATOM    442  CG2 VAL B 596     116.335  94.049  84.484  1.00 20.00           C
ATOM    443  H   VAL B 596     113.646  94.342  85.943  1.00  0.00           H
ATOM    444  HA  VAL B 596     114.071  94.468  83.123  1.00  0.00           H
ATOM    445  HB  VAL B 596     115.064  92.573  85.282  1.00  0.00           H
ATOM    446 HG11 VAL B 596     116.739  91.723  83.503  1.00  0.00           H
ATOM    447 HG12 VAL B 596     115.143  91.010  83.463  1.00  0.00           H
ATOM    448 HG13 VAL B 596     115.591  92.261  82.302  1.00  0.00           H
ATOM    449 HG21 VAL B 596     117.201  93.543  84.898  1.00  0.00           H
ATOM    450 HG22 VAL B 596     116.620  94.509  83.536  1.00  0.00           H
ATOM    451 HG23 VAL B 596     116.062  94.836  85.176  1.00  0.00           H
ATOM    452  N   THR B 597     112.348  91.842  84.220  1.00 30.00           N
ATOM    453  CA  THR B 597     111.501  90.711  83.838  1.00 30.00           C
ATOM    454  C   THR B 597     110.136  91.143  83.263  1.00 30.00           C
ATOM    455  O   THR B 597     109.525  90.346  82.556  1.00 30.00           O
ATOM    456  CB  THR B 597     111.279  89.742  85.029  1.00 20.00           C
ATOM    457  CG2 THR B 597     112.595  89.216  85.618  1.00 20.00           C
ATOM    458  OG1 THR B 597     110.497  90.307  86.063  1.00 20.00           O
ATOM    459  H   THR B 597     112.516  91.969  85.208  1.00  0.00           H
ATOM    460  HA  THR B 597     112.020  90.158  83.053  1.00  0.00           H
ATOM    461  HB  THR B 597     110.721  88.877  84.668  1.00  0.00           H
ATOM    462  HG1 THR B 597     110.987  91.028  86.467  1.00  0.00           H
ATOM    463 HG21 THR B 597     112.424  88.571  86.478  1.00  0.00           H
ATOM    464 HG22 THR B 597     113.136  88.648  84.868  1.00  0.00           H
ATOM    465 HG23 THR B 597     113.263  90.010  85.934  1.00  0.00           H
ATOM    466  N   LEU B 598     109.712  92.396  83.507  1.00 30.00           N
ATOM    467  CA  LEU B 598     108.554  92.999  82.852  1.00 30.00           C
ATOM    468  C   LEU B 598     108.902  93.548  81.453  1.00 30.00           C
ATOM    469  O   LEU B 598     108.083  93.410  80.544  1.00 30.00           O
ATOM    470  CB  LEU B 598     107.970  94.086  83.771  1.00 20.00           C
ATOM    471  CG  LEU B 598     106.600  94.633  83.321  1.00 20.00           C
ATOM    472  CD1 LEU B 598     105.481  93.570  83.330  1.00 20.00           C
ATOM    473  CD2 LEU B 598     106.227  95.831  84.188  1.00 20.00           C
ATOM    474  H   LEU B 598     110.258  93.000  84.105  1.00  0.00           H
ATOM    475  HA  LEU B 598     107.799  92.223  82.720  1.00  0.00           H
ATOM    476  HB2 LEU B 598     107.877  93.697  84.785  1.00  0.00           H
ATOM    477  HB3 LEU B 598     108.686  94.907  83.843  1.00  0.00           H
ATOM    478  HG  LEU B 598     106.686  95.013  82.302  1.00  0.00           H
ATOM    479 HD11 LEU B 598     104.597  93.909  83.869  1.00  0.00           H
ATOM    480 HD12 LEU B 598     105.166  93.332  82.313  1.00  0.00           H
ATOM    481 HD13 LEU B 598     105.797  92.638  83.797  1.00  0.00           H
ATOM    482 HD21 LEU B 598     105.252  96.224  83.905  1.00  0.00           H
ATOM    483 HD22 LEU B 598     106.198  95.563  85.242  1.00  0.00           H
ATOM    484 HD23 LEU B 598     106.965  96.623  84.072  1.00  0.00           H
ATOM    485  N   ILE B 599     110.112  94.119  81.296  1.00 30.00           N
```

```
ATOM    486  CA  ILE B 599     110.682  94.528  80.008  1.00 30.00           C
ATOM    487  C   ILE B 599     110.956  93.293  79.125  1.00 30.00           C
ATOM    488  O   ILE B 599     111.536  92.317  79.600  1.00 30.00           O
ATOM    489  CB  ILE B 599     111.976  95.384  80.191  1.00 20.00           C
ATOM    490  CG1 ILE B 599     111.628  96.769  80.789  1.00 20.00           C
ATOM    491  CG2 ILE B 599     112.821  95.565  78.910  1.00 20.00           C
ATOM    492  CD1 ILE B 599     112.823  97.491  81.432  1.00 20.00           C
ATOM    493  H   ILE B 599     110.729  94.202  82.093  1.00  0.00           H
ATOM    494  HA  ILE B 599     109.944  95.146  79.499  1.00  0.00           H
ATOM    495  HB  ILE B 599     112.608  94.871  80.916  1.00  0.00           H
ATOM    496 HG12 ILE B 599     111.196  97.403  80.013  1.00  0.00           H
ATOM    497 HG13 ILE B 599     110.850  96.672  81.546  1.00  0.00           H
ATOM    498 HG21 ILE B 599     113.658  96.244  79.070  1.00  0.00           H
ATOM    499 HG22 ILE B 599     113.248  94.628  78.558  1.00  0.00           H
ATOM    500 HG23 ILE B 599     112.216  95.969  78.101  1.00  0.00           H
ATOM    501 HD11 ILE B 599     112.485  98.243  82.144  1.00  0.00           H
ATOM    502 HD12 ILE B 599     113.474  96.800  81.969  1.00  0.00           H
ATOM    503 HD13 ILE B 599     113.423  98.000  80.679  1.00  0.00           H
ATOM    504  N   GLU B 600     110.487  93.345  77.872  1.00 30.00           N
ATOM    505  CA  GLU B 600     110.508  92.203  76.958  1.00 30.00           C
ATOM    506  C   GLU B 600     111.806  92.117  76.142  1.00 30.00           C
ATOM    507  O   GLU B 600     112.338  91.019  75.981  1.00 30.00           O
ATOM    508  CB  GLU B 600     109.256  92.261  76.063  1.00 20.00           C
ATOM    509  CG  GLU B 600     107.966  92.038  76.880  1.00 20.00           C
ATOM    510  CD  GLU B 600     106.695  92.046  76.034  1.00 20.00           C
ATOM    511  OE1 GLU B 600     106.585  92.935  75.163  1.00 20.00           O
ATOM    512  OE2 GLU B 600     105.849  91.160  76.292  1.00 20.00           O1-
ATOM    513  H   GLU B 600     110.016  94.177  77.542  1.00  0.00           H
ATOM    514  HA  GLU B 600     110.446  91.278  77.535  1.00  0.00           H
ATOM    515  HB2 GLU B 600     109.214  93.228  75.558  1.00  0.00           H
ATOM    516  HB3 GLU B 600     109.323  91.513  75.272  1.00  0.00           H
ATOM    517  HG2 GLU B 600     108.030  91.081  77.396  1.00  0.00           H
ATOM    518  HG3 GLU B 600     107.858  92.799  77.652  1.00  0.00           H
ATOM    519  N   ASP B 601     112.299  93.270  75.668  1.00 30.00           N
ATOM    520  CA  ASP B 601     113.493  93.399  74.828  1.00 30.00           C
ATOM    521  C   ASP B 601     113.926  94.877  74.771  1.00 30.00           C
ATOM    522  O   ASP B 601     113.244  95.734  75.331  1.00 30.00           O
ATOM    523  CB  ASP B 601     113.362  92.751  73.414  1.00 20.00           C
ATOM    524  CG  ASP B 601     112.035  92.967  72.671  1.00  0.00           C
ATOM    525  OD1 ASP B 601     111.376  94.002  72.916  1.00  0.00           O
ATOM    526  OD2 ASP B 601     111.743  92.119  71.801  1.00  0.00           O1-
ATOM    527  H   ASP B 601     111.805  94.133  75.845  1.00  0.00           H
ATOM    528  HA  ASP B 601     114.298  92.879  75.348  1.00  0.00           H
ATOM    529  HB2 ASP B 601     114.170  93.070  72.756  1.00  0.00           H
ATOM    530  HB3 ASP B 601     113.484  91.675  73.544  1.00  0.00           H
ATOM    531  N   GLY B 602     115.059  95.147  74.105  1.00 30.00           N
ATOM    532  CA  GLY B 602     115.595  96.495  73.916  1.00 30.00           C
ATOM    533  C   GLY B 602     116.732  96.781  74.907  1.00 30.00           C
ATOM    534  O   GLY B 602     117.286  95.871  75.526  1.00 30.00           O
ATOM    535  H   GLY B 602     115.578  94.388  73.690  1.00  0.00           H
ATOM    536  HA2 GLY B 602     115.988  96.568  72.902  1.00  0.00           H
ATOM    537  HA3 GLY B 602     114.817  97.256  74.007  1.00  0.00           H
ATOM    538  N   LYS B 603     117.097  98.071  75.003  1.00 30.00           N
ATOM    539  CA  LYS B 603     118.228  98.621  75.762  1.00 30.00           C
ATOM    540  C   LYS B 603     118.246  98.254  77.258  1.00 30.00           C
ATOM    541  O   LYS B 603     119.293  97.859  77.773  1.00 30.00           O
ATOM    542  CB  LYS B 603     118.240 100.154  75.541  1.00 20.00           C
ATOM    543  CG  LYS B 603     119.310 100.944  76.321  1.00 20.00           C
ATOM    544  CD  LYS B 603     119.243 102.450  76.035  1.00 20.00           C
ATOM    545  CE  LYS B 603     120.261 103.252  76.857  1.00 20.00           C
ATOM    546  NZ  LYS B 603     120.083 104.700  76.660  1.00 20.00           N1+
ATOM    547  H   LYS B 603     116.585  98.749  74.457  1.00  0.00           H
ATOM    548  HA  LYS B 603     119.135  98.209  75.316  1.00  0.00           H
ATOM    549  HB2 LYS B 603     118.364 100.354  74.476  1.00  0.00           H
ATOM    550  HB3 LYS B 603     117.261 100.557  75.808  1.00  0.00           H
ATOM    551  HG2 LYS B 603     119.183 100.797  77.394  1.00  0.00           H
ATOM    552  HG3 LYS B 603     120.301 100.562  76.072  1.00  0.00           H
ATOM    553  HD2 LYS B 603     119.416 102.624  74.972  1.00  0.00           H
ATOM    554  HD3 LYS B 603     118.236 102.813  76.243  1.00  0.00           H
ATOM    555  HE2 LYS B 603     120.146 103.034  77.919  1.00  0.00           H
ATOM    556  HE3 LYS B 603     121.278 102.973  76.578  1.00  0.00           H
```

ATOM	557	HZ1	LYS	B	603	119.158	104.966	76.972	1.00	0.00	H
ATOM	558	HZ2	LYS	B	603	120.773	105.202	77.201	1.00	0.00	H
ATOM	559	HZ3	LYS	B	603	120.189	104.928	75.682	1.00	0.00	H
ATOM	560	N	TYR	B	627	117.091	98.413	77.921	1.00	30.00	N
ATOM	561	CA	TYR	B	627	116.931	98.285	79.372	1.00	30.00	C
ATOM	562	C	TYR	B	627	116.590	96.858	79.845	1.00	30.00	C
ATOM	563	O	TYR	B	627	116.304	96.679	81.029	1.00	30.00	O
ATOM	564	CB	TYR	B	627	115.902	99.337	79.832	1.00	20.00	C
ATOM	565	CG	TYR	B	627	116.376	100.765	79.619	1.00	20.00	C
ATOM	566	CD1	TYR	B	627	117.422	101.273	80.415	1.00	20.00	C
ATOM	567	CD2	TYR	B	627	115.805	101.577	78.617	1.00	20.00	C
ATOM	568	CE1	TYR	B	627	117.895	102.581	80.213	1.00	20.00	C
ATOM	569	CE2	TYR	B	627	116.264	102.897	78.429	1.00	20.00	C
ATOM	570	CZ	TYR	B	627	117.305	103.402	79.234	1.00	20.00	C
ATOM	571	OH	TYR	B	627	117.741	104.686	79.079	1.00	20.00	O
ATOM	572	H	TYR	B	627	116.276	98.726	77.415	1.00	0.00	H
ATOM	573	HA	TYR	B	627	117.881	98.526	79.849	1.00	0.00	H
ATOM	574	HB2	TYR	B	627	114.952	99.187	79.316	1.00	0.00	H
ATOM	575	HB3	TYR	B	627	115.687	99.225	80.895	1.00	0.00	H
ATOM	576	HD1	TYR	B	627	117.865	100.657	81.183	1.00	0.00	H
ATOM	577	HD2	TYR	B	627	115.011	101.195	77.993	1.00	0.00	H
ATOM	578	HE1	TYR	B	627	118.701	102.955	80.827	1.00	0.00	H
ATOM	579	HE2	TYR	B	627	115.810	103.522	77.675	1.00	0.00	H
ATOM	580	HH	TYR	B	627	118.210	105.002	79.860	1.00	0.00	H
ATOM	581	N	ASN	B	628	116.658	95.870	78.936	1.00	30.00	N
ATOM	582	CA	ASN	B	628	116.437	94.449	79.218	1.00	30.00	C
ATOM	583	C	ASN	B	628	117.611	93.780	79.973	1.00	30.00	C
ATOM	584	O	ASN	B	628	117.397	92.725	80.568	1.00	30.00	O
ATOM	585	CB	ASN	B	628	116.121	93.735	77.880	1.00	20.00	C
ATOM	586	CG	ASN	B	628	115.512	92.331	78.002	1.00	20.00	C
ATOM	587	ND2	ASN	B	628	114.502	92.148	78.854	1.00	20.00	N
ATOM	588	OD1	ASN	B	628	115.945	91.415	77.308	1.00	20.00	O
ATOM	589	H	ASN	B	628	116.905	96.096	77.982	1.00	0.00	H
ATOM	590	HA	ASN	B	628	115.577	94.357	79.884	1.00	0.00	H
ATOM	591	HB2	ASN	B	628	115.396	94.329	77.322	1.00	0.00	H
ATOM	592	HB3	ASN	B	628	117.014	93.690	77.256	1.00	0.00	H
ATOM	593	HD21	ASN	B	628	114.071	91.238	78.934	1.00	0.00	H
ATOM	594	HD22	ASN	B	628	114.143	92.906	79.416	1.00	0.00	H
ATOM	595	N	SER	B	629	118.807	94.397	79.978	1.00	30.00	N
ATOM	596	CA	SER	B	629	119.927	93.977	80.827	1.00	30.00	C
ATOM	597	C	SER	B	629	119.764	94.527	82.258	1.00	30.00	C
ATOM	598	O	SER	B	629	119.200	95.610	82.437	1.00	30.00	O
ATOM	599	CB	SER	B	629	121.269	94.377	80.170	1.00	20.00	C
ATOM	600	OG	SER	B	629	121.638	95.725	80.385	1.00	20.00	O
ATOM	601	H	SER	B	629	118.922	95.266	79.477	1.00	0.00	H
ATOM	602	HA	SER	B	629	119.917	92.886	80.880	1.00	0.00	H
ATOM	603	HB2	SER	B	629	121.249	94.176	79.098	1.00	0.00	H
ATOM	604	HB3	SER	B	629	122.067	93.758	80.580	1.00	0.00	H
ATOM	605	HG	SER	B	629	121.140	96.288	79.786	1.00	0.00	H
ATOM	606	N	LEU	B	630	120.282	93.772	83.243	1.00	30.00	N
ATOM	607	CA	LEU	B	630	120.223	94.102	84.671	1.00	30.00	C
ATOM	608	C	LEU	B	630	120.923	95.423	85.031	1.00	30.00	C
ATOM	609	O	LEU	B	630	120.346	96.214	85.774	1.00	30.00	O
ATOM	610	CB	LEU	B	630	120.754	92.905	85.495	1.00	20.00	C
ATOM	611	CG	LEU	B	630	120.620	93.023	87.033	1.00	20.00	C
ATOM	612	CD1	LEU	B	630	119.153	93.193	87.482	1.00	20.00	C
ATOM	613	CD2	LEU	B	630	121.296	91.819	87.724	1.00	20.00	C
ATOM	614	H	LEU	B	630	120.723	92.893	83.012	1.00	0.00	H
ATOM	615	HA	LEU	B	630	119.176	94.248	84.915	1.00	0.00	H
ATOM	616	HB2	LEU	B	630	120.240	91.998	85.181	1.00	0.00	H
ATOM	617	HB3	LEU	B	630	121.804	92.749	85.243	1.00	0.00	H
ATOM	618	HG	LEU	B	630	121.164	93.909	87.363	1.00	0.00	H
ATOM	619	HD11	LEU	B	630	118.955	92.738	88.453	1.00	0.00	H
ATOM	620	HD12	LEU	B	630	118.901	94.249	87.572	1.00	0.00	H
ATOM	621	HD13	LEU	B	630	118.457	92.750	86.770	1.00	0.00	H
ATOM	622	HD21	LEU	B	630	120.617	91.271	88.378	1.00	0.00	H
ATOM	623	HD22	LEU	B	630	121.685	91.099	87.004	1.00	0.00	H
ATOM	624	HD23	LEU	B	630	122.139	92.148	88.329	1.00	0.00	H
ATOM	625	N	TYR	B	631	122.126	95.640	84.476	1.00	30.00	N
ATOM	626	CA	TYR	B	631	122.945	96.833	84.696	1.00	30.00	C
ATOM	627	C	TYR	B	631	122.297	98.136	84.193	1.00	30.00	C

ATOM	628	O	TYR	B	631	122.347	99.134	84.912	1.00	30.00	O
ATOM	629	CB	TYR	B	631	124.347	96.598	84.088	1.00	20.00	C
ATOM	630	CG	TYR	B	631	125.269	97.807	84.067	1.00	20.00	C
ATOM	631	CD1	TYR	B	631	125.781	98.327	85.274	1.00	20.00	C
ATOM	632	CD2	TYR	B	631	125.595	98.430	82.843	1.00	20.00	C
ATOM	633	CE1	TYR	B	631	126.609	99.467	85.259	1.00	20.00	C
ATOM	634	CE2	TYR	B	631	126.427	99.566	82.828	1.00	20.00	C
ATOM	635	CZ	TYR	B	631	126.932	100.087	84.035	1.00	20.00	C
ATOM	636	OH	TYR	B	631	127.726	101.195	84.018	1.00	20.00	O
ATOM	637	H	TYR	B	631	122.515	94.943	83.859	1.00	0.00	H
ATOM	638	HA	TYR	B	631	123.070	96.933	85.776	1.00	0.00	H
ATOM	639	HB2	TYR	B	631	124.849	95.793	84.627	1.00	0.00	H
ATOM	640	HB3	TYR	B	631	124.236	96.241	83.062	1.00	0.00	H
ATOM	641	HD1	TYR	B	631	125.531	97.861	86.215	1.00	0.00	H
ATOM	642	HD2	TYR	B	631	125.202	98.046	81.913	1.00	0.00	H
ATOM	643	HE1	TYR	B	631	126.990	99.863	86.188	1.00	0.00	H
ATOM	644	HE2	TYR	B	631	126.670	100.042	81.889	1.00	0.00	H
ATOM	645	HH	TYR	B	631	128.022	101.463	84.891	1.00	0.00	H
ATOM	646	N	SER	B	632	121.706	98.103	82.984	1.00	30.00	N
ATOM	647	CA	SER	B	632	121.145	99.276	82.310	1.00	30.00	C
ATOM	648	C	SER	B	632	119.989	99.949	83.067	1.00	30.00	C
ATOM	649	O	SER	B	632	119.954	101.177	83.105	1.00	30.00	O
ATOM	650	CB	SER	B	632	120.711	98.909	80.878	1.00	20.00	C
ATOM	651	OG	SER	B	632	121.837	98.816	80.030	1.00	20.00	O
ATOM	652	H	SER	B	632	121.699	97.242	82.454	1.00	0.00	H
ATOM	653	HA	SER	B	632	121.940	100.022	82.244	1.00	0.00	H
ATOM	654	HB2	SER	B	632	120.144	97.978	80.858	1.00	0.00	H
ATOM	655	HB3	SER	B	632	120.062	99.679	80.458	1.00	0.00	H
ATOM	656	HG	SER	B	632	122.193	99.697	79.893	1.00	0.00	H
ATOM	657	N	THR	B	633	119.088	99.147	83.662	1.00	30.00	N
ATOM	658	CA	THR	B	633	117.945	99.648	84.430	1.00	30.00	C
ATOM	659	C	THR	B	633	118.217	99.726	85.953	1.00	30.00	C
ATOM	660	O	THR	B	633	117.457	100.394	86.653	1.00	30.00	O
ATOM	661	CB	THR	B	633	116.667	98.822	84.138	1.00	20.00	C
ATOM	662	CG2	THR	B	633	116.710	97.372	84.633	1.00	20.00	C
ATOM	663	OG1	THR	B	633	115.527	99.421	84.724	1.00	20.00	O
ATOM	664	H	THR	B	633	119.181	98.143	83.591	1.00	0.00	H
ATOM	665	HA	THR	B	633	117.729	100.667	84.105	1.00	0.00	H
ATOM	666	HB	THR	B	633	116.504	98.811	83.061	1.00	0.00	H
ATOM	667	HG1	THR	B	633	115.655	99.451	85.676	1.00	0.00	H
ATOM	668	HG21	THR	B	633	115.814	96.852	84.296	1.00	0.00	H
ATOM	669	HG22	THR	B	633	117.571	96.839	84.231	1.00	0.00	H
ATOM	670	HG23	THR	B	633	116.748	97.301	85.720	1.00	0.00	H
ATOM	671	N	CYS	B	634	119.321	99.120	86.436	1.00	30.00	N
ATOM	672	CA	CYS	B	634	119.848	99.343	87.790	1.00	30.00	C
ATOM	673	C	CYS	B	634	120.369	100.785	87.948	1.00	30.00	C
ATOM	674	O	CYS	B	634	120.166	101.383	89.004	1.00	30.00	O
ATOM	675	CB	CYS	B	634	120.919	98.299	88.182	1.00	20.00	C
ATOM	676	SG	CYS	B	634	121.434	98.445	89.921	1.00	20.00	S
ATOM	677	H	CYS	B	634	119.895	98.559	85.822	1.00	0.00	H
ATOM	678	HA	CYS	B	634	119.013	99.228	88.484	1.00	0.00	H
ATOM	679	HB2	CYS	B	634	120.529	97.292	88.055	1.00	0.00	H
ATOM	680	HB3	CYS	B	634	121.797	98.382	87.541	1.00	0.00	H
ATOM	681	HG	CYS	B	634	122.014	99.642	89.797	1.00	0.00	H
ATOM	682	N	LEU	B	635	120.966	101.330	86.872	1.00	30.00	N
ATOM	683	CA	LEU	B	635	121.337	102.739	86.746	1.00	30.00	C
ATOM	684	C	LEU	B	635	120.112	103.667	86.748	1.00	30.00	C
ATOM	685	O	LEU	B	635	120.160	104.678	87.440	1.00	30.00	O
ATOM	686	CB	LEU	B	635	122.168	102.959	85.464	1.00	20.00	C
ATOM	687	CG	LEU	B	635	123.586	102.352	85.502	1.00	20.00	C
ATOM	688	CD1	LEU	B	635	124.197	102.316	84.086	1.00	20.00	C
ATOM	689	CD2	LEU	B	635	124.496	103.064	86.528	1.00	20.00	C
ATOM	690	H	LEU	B	635	121.110	100.761	86.048	1.00	0.00	H
ATOM	691	HA	LEU	B	635	121.945	102.999	87.613	1.00	0.00	H
ATOM	692	HB2	LEU	B	635	121.618	102.540	84.621	1.00	0.00	H
ATOM	693	HB3	LEU	B	635	122.256	104.027	85.253	1.00	0.00	H
ATOM	694	HG	LEU	B	635	123.499	101.315	85.826	1.00	0.00	H
ATOM	695	HD11	LEU	B	635	125.209	102.717	84.044	1.00	0.00	H
ATOM	696	HD12	LEU	B	635	124.243	101.289	83.722	1.00	0.00	H
ATOM	697	HD13	LEU	B	635	123.601	102.879	83.367	1.00	0.00	H
ATOM	698	HD21	LEU	B	635	124.741	102.393	87.352	1.00	0.00	H

```
ATOM    699 HD22 LEU B 635     125.441 103.398  86.101  1.00  0.00           H
ATOM    700 HD23 LEU B 635     124.018 103.944  86.960  1.00  0.00           H
ATOM    701  N   GLU B 636     119.041 103.305  86.016  1.00 30.00           N
ATOM    702  CA  GLU B 636     117.795 104.079  85.944  1.00 30.00           C
ATOM    703  C   GLU B 636     117.027 104.148  87.274  1.00 30.00           C
ATOM    704  O   GLU B 636     116.416 105.181  87.543  1.00 30.00           O
ATOM    705  CB  GLU B 636     116.884 103.544  84.820  1.00 20.00           C
ATOM    706  CG  GLU B 636     117.468 103.683  83.399  1.00 20.00           C
ATOM    707  CD  GLU B 636     117.632 105.122  82.897  1.00 20.00           C
ATOM    708  OE1 GLU B 636     116.876 106.000  83.366  1.00 20.00           O
ATOM    709  OE2 GLU B 636     118.514 105.320  82.030  1.00 20.00           O1-
ATOM    710  H   GLU B 636     119.071 102.457  85.470  1.00  0.00           H
ATOM    711  HA  GLU B 636     118.070 105.106  85.702  1.00  0.00           H
ATOM    712  HB2 GLU B 636     116.660 102.494  85.007  1.00  0.00           H
ATOM    713  HB3 GLU B 636     115.914 104.044  84.856  1.00  0.00           H
ATOM    714  HG2 GLU B 636     118.435 103.187  83.345  1.00  0.00           H
ATOM    715  HG3 GLU B 636     116.819 103.159  82.701  1.00  0.00           H
ATOM    716  N   LEU B 637     117.102 103.086  88.097  1.00 30.00           N
ATOM    717  CA  LEU B 637     116.566 103.096  89.458  1.00 30.00           C
ATOM    718  C   LEU B 637     117.478 103.838  90.451  1.00 30.00           C
ATOM    719  O   LEU B 637     116.960 104.465  91.374  1.00 30.00           O
ATOM    720  CB  LEU B 637     116.207 101.674  89.930  1.00 20.00           C
ATOM    721  CG  LEU B 637     114.954 101.076  89.248  1.00 20.00           C
ATOM    722  CD1 LEU B 637     114.629  99.704  89.847  1.00 20.00           C
ATOM    723  CD2 LEU B 637     113.704 101.981  89.293  1.00 20.00           C
ATOM    724  H   LEU B 637     117.592 102.251  87.803  1.00  0.00           H
ATOM    725  HA  LEU B 637     115.643 103.660  89.435  1.00  0.00           H
ATOM    726  HB2 LEU B 637     117.064 101.013  89.784  1.00  0.00           H
ATOM    727  HB3 LEU B 637     116.025 101.690  91.005  1.00  0.00           H
ATOM    728  HG  LEU B 637     115.202 100.916  88.197  1.00  0.00           H
ATOM    729 HD11 LEU B 637     114.207  99.049  89.087  1.00  0.00           H
ATOM    730 HD12 LEU B 637     115.514  99.223  90.262  1.00  0.00           H
ATOM    731 HD13 LEU B 637     113.901  99.778  90.656  1.00  0.00           H
ATOM    732 HD21 LEU B 637     112.789 101.410  89.454  1.00  0.00           H
ATOM    733 HD22 LEU B 637     113.756 102.721  90.090  1.00  0.00           H
ATOM    734 HD23 LEU B 637     113.584 102.517  88.351  1.00  0.00           H
ATOM    735  N   PHE B 638     118.803 103.819  90.227  1.00 30.00           N
ATOM    736  CA  PHE B 638     119.769 104.600  91.004  1.00 30.00           C
ATOM    737  C   PHE B 638     119.672 106.122  90.746  1.00 30.00           C
ATOM    738  O   PHE B 638     120.019 106.897  91.637  1.00 30.00           O
ATOM    739  CB  PHE B 638     121.186 104.025  90.785  1.00 20.00           C
ATOM    740  CG  PHE B 638     122.290 104.673  91.604  1.00 20.00           C
ATOM    741  CD1 PHE B 638     122.445 104.334  92.964  1.00 20.00           C
ATOM    742  CD2 PHE B 638     123.052 105.736  91.073  1.00 20.00           C
ATOM    743  CE1 PHE B 638     123.369 105.008  93.751  1.00 20.00           C
ATOM    744  CE2 PHE B 638     123.972 106.398  91.876  1.00 20.00           C
ATOM    745  CZ  PHE B 638     124.131 106.033  93.207  1.00 20.00           C
ATOM    746  H   PHE B 638     119.176 103.277  89.459  1.00  0.00           H
ATOM    747  HA  PHE B 638     119.529 104.451  92.059  1.00  0.00           H
ATOM    748  HB2 PHE B 638     121.178 102.966  91.046  1.00  0.00           H
ATOM    749  HB3 PHE B 638     121.454 104.069  89.729  1.00  0.00           H
ATOM    750  HD1 PHE B 638     121.842 103.552  93.400  1.00  0.00           H
ATOM    751  HD2 PHE B 638     122.915 106.043  90.047  1.00  0.00           H
ATOM    752  HE1 PHE B 638     123.487 104.739  94.790  1.00  0.00           H
ATOM    753  HE2 PHE B 638     124.558 107.208  91.466  1.00  0.00           H
ATOM    754  HZ  PHE B 638     124.845 106.557  93.825  1.00  0.00           H
ATOM    755  N   LYS B 639     119.159 106.531  89.569  1.00 30.00           N
ATOM    756  CA  LYS B 639     118.895 107.929  89.219  1.00 30.00           C
ATOM    757  C   LYS B 639     117.814 108.598  90.086  1.00 30.00           C
ATOM    758  O   LYS B 639     117.934 109.797  90.315  1.00 30.00           O
ATOM    759  CB  LYS B 639     118.531 108.065  87.727  1.00 20.00           C
ATOM    760  CG  LYS B 639     119.712 107.953  86.754  1.00 20.00           C
ATOM    761  CD  LYS B 639     119.238 107.978  85.290  1.00 20.00           C
ATOM    762  CE  LYS B 639     120.300 107.522  84.280  1.00 20.00           C
ATOM    763  NZ  LYS B 639     121.466 108.422  84.267  1.00 20.00           N1+
ATOM    764  H   LYS B 639     118.913 105.841  88.872  1.00  0.00           H
ATOM    765  HA  LYS B 639     119.822 108.480  89.388  1.00  0.00           H
ATOM    766  HB2 LYS B 639     117.754 107.346  87.472  1.00  0.00           H
ATOM    767  HB3 LYS B 639     118.095 109.047  87.551  1.00  0.00           H
ATOM    768  HG2 LYS B 639     120.401 108.779  86.930  1.00  0.00           H
ATOM    769  HG3 LYS B 639     120.281 107.049  86.949  1.00  0.00           H
```

```
ATOM    770  HD2 LYS B 639     118.354 107.351  85.178  1.00  0.00           H
ATOM    771  HD3 LYS B 639     118.909 108.987  85.037  1.00  0.00           H
ATOM    772  HE2 LYS B 639     120.628 106.507  84.507  1.00  0.00           H
ATOM    773  HE3 LYS B 639     119.870 107.500  83.278  1.00  0.00           H
ATOM    774  HZ1 LYS B 639     121.166 109.355  84.023  1.00  0.00           H
ATOM    775  HZ2 LYS B 639     121.892 108.433  85.183  1.00  0.00           H
ATOM    776  HZ3 LYS B 639     122.136 108.095  83.586  1.00  0.00           H
ATOM    777  N   PHE B 640     116.801 107.850  90.569  1.00 30.00           N
ATOM    778  CA  PHE B 640     115.768 108.385  91.473  1.00 30.00           C
ATOM    779  C   PHE B 640     116.324 108.877  92.817  1.00 30.00           C
ATOM    780  O   PHE B 640     115.839 109.890  93.316  1.00 30.00           O
ATOM    781  CB  PHE B 640     114.640 107.366  91.723  1.00 20.00           C
ATOM    782  CG  PHE B 640     113.708 107.120  90.553  1.00 20.00           C
ATOM    783  CD1 PHE B 640     112.617 107.979  90.304  1.00 20.00           C
ATOM    784  CD2 PHE B 640     114.009 106.116  89.616  1.00 20.00           C
ATOM    785  CE1 PHE B 640     111.799 107.755  89.205  1.00 20.00           C
ATOM    786  CE2 PHE B 640     113.181 105.901  88.524  1.00 20.00           C
ATOM    787  CZ  PHE B 640     112.068 106.707  88.332  1.00 20.00           C
ATOM    788  H   PHE B 640     116.750 106.865  90.350  1.00  0.00           H
ATOM    789  HA  PHE B 640     115.324 109.256  90.985  1.00  0.00           H
ATOM    790  HB2 PHE B 640     115.073 106.414  92.032  1.00  0.00           H
ATOM    791  HB3 PHE B 640     114.023 107.693  92.561  1.00  0.00           H
ATOM    792  HD1 PHE B 640     112.403 108.798  90.975  1.00  0.00           H
ATOM    793  HD2 PHE B 640     114.889 105.520  89.764  1.00  0.00           H
ATOM    794  HE1 PHE B 640     110.957 108.405  89.023  1.00  0.00           H
ATOM    795  HE2 PHE B 640     113.407 105.113  87.821  1.00  0.00           H
ATOM    796  HZ  PHE B 640     111.421 106.532  87.487  1.00  0.00           H
ATOM    797  N   THR B 641     117.348 108.180  93.342  1.00 30.00           N
ATOM    798  CA  THR B 641     118.090 108.540  94.555  1.00 30.00           C
ATOM    799  C   THR B 641     118.886 109.856  94.407  1.00 30.00           C
ATOM    800  O   THR B 641     119.002 110.596  95.384  1.00 30.00           O
ATOM    801  CB  THR B 641     119.062 107.401  94.963  1.00 20.00           C
ATOM    802  CG2 THR B 641     119.930 107.640  96.212  1.00 20.00           C
ATOM    803  OG1 THR B 641     118.309 106.226  95.186  1.00 20.00           O
ATOM    804  H   THR B 641     117.679 107.355  92.863  1.00  0.00           H
ATOM    805  HA  THR B 641     117.368 108.672  95.359  1.00  0.00           H
ATOM    806  HB  THR B 641     119.736 107.190  94.134  1.00  0.00           H
ATOM    807  HG1 THR B 641     118.894 105.568  95.570  1.00  0.00           H
ATOM    808 HG21 THR B 641     120.523 106.755  96.449  1.00  0.00           H
ATOM    809 HG22 THR B 641     120.634 108.461  96.073  1.00  0.00           H
ATOM    810 HG23 THR B 641     119.318 107.866  97.084  1.00  0.00           H
ATOM    811  N   ILE B 642     119.371 110.138  93.185  1.00 30.00           N
ATOM    812  CA  ILE B 642     120.036 111.390  92.805  1.00 30.00           C
ATOM    813  C   ILE B 642     119.019 112.540  92.596  1.00 30.00           C
ATOM    814  O   ILE B 642     119.372 113.699  92.814  1.00 30.00           O
ATOM    815  CB  ILE B 642     120.867 111.209  91.491  1.00 20.00           C
ATOM    816  CG1 ILE B 642     121.913 110.073  91.623  1.00 20.00           C
ATOM    817  CG2 ILE B 642     121.563 112.497  90.991  1.00 20.00           C
ATOM    818  CD1 ILE B 642     122.507 109.617  90.280  1.00 20.00           C
ATOM    819  H   ILE B 642     119.228 109.475  92.437  1.00  0.00           H
ATOM    820  HA  ILE B 642     120.716 111.681  93.608  1.00  0.00           H
ATOM    821  HB  ILE B 642     120.174 110.910  90.706  1.00  0.00           H
ATOM    822 HG12 ILE B 642     122.716 110.396  92.286  1.00  0.00           H
ATOM    823 HG13 ILE B 642     121.478 109.196  92.100  1.00  0.00           H
ATOM    824 HG21 ILE B 642     122.166 112.309  90.103  1.00  0.00           H
ATOM    825 HG22 ILE B 642     120.853 113.274  90.711  1.00  0.00           H
ATOM    826 HG23 ILE B 642     122.223 112.909  91.755  1.00  0.00           H
ATOM    827 HD11 ILE B 642     122.590 108.531  90.242  1.00  0.00           H
ATOM    828 HD12 ILE B 642     121.893 109.922  89.433  1.00  0.00           H
ATOM    829 HD13 ILE B 642     123.505 110.030  90.134  1.00  0.00           H
ATOM    830  N   GLY B 643     117.782 112.198  92.195  1.00 30.00           N
ATOM    831  CA  GLY B 643     116.723 113.144  91.844  1.00 30.00           C
ATOM    832  C   GLY B 643     116.665 113.383  90.325  1.00 30.00           C
ATOM    833  O   GLY B 643     116.121 114.400  89.901  1.00 30.00           O
ATOM    834  H   GLY B 643     117.565 111.218  92.080  1.00  0.00           H
ATOM    835  HA2 GLY B 643     115.774 112.715  92.163  1.00  0.00           H
ATOM    836  HA3 GLY B 643     116.836 114.093  92.372  1.00  0.00           H
ATOM    837  N   MET B 644     117.228 112.463  89.521  1.00 30.00           N
ATOM    838  CA  MET B 644     117.288 112.491  88.057  1.00 30.00           C
ATOM    839  C   MET B 644     116.469 111.362  87.395  1.00 30.00           C
ATOM    840  O   MET B 644     116.533 111.225  86.173  1.00 30.00           O
```

```
ATOM    841  CB  MET B 644     118.773 112.415  87.624  1.00 20.00           C
ATOM    842  CG  MET B 644     119.541 113.736  87.799  1.00 20.00           C
ATOM    843  SD  MET B 644     118.916 115.165  86.862  1.00 20.00           S
ATOM    844  CE  MET B 644     118.915 114.513  85.169  1.00 20.00           C
ATOM    845  H   MET B 644     117.652 111.650  89.949  1.00  0.00           H
ATOM    846  HA  MET B 644     116.860 113.419  87.688  1.00  0.00           H
ATOM    847  HB2 MET B 644     119.278 111.630  88.189  1.00  0.00           H
ATOM    848  HB3 MET B 644     118.865 112.110  86.583  1.00  0.00           H
ATOM    849  HG2 MET B 644     119.551 114.014  88.852  1.00  0.00           H
ATOM    850  HG3 MET B 644     120.583 113.583  87.517  1.00  0.00           H
ATOM    851  HE1 MET B 644     118.694 115.313  84.463  1.00  0.00           H
ATOM    852  HE2 MET B 644     119.888 114.090  84.921  1.00  0.00           H
ATOM    853  HE3 MET B 644     118.154 113.741  85.054  1.00  0.00           H
ATOM    854  N   GLY B 645     115.736 110.560  88.188  1.00 30.00           N
ATOM    855  CA  GLY B 645     115.050 109.359  87.715  1.00 30.00           C
ATOM    856  C   GLY B 645     113.774 109.724  86.953  1.00 30.00           C
ATOM    857  O   GLY B 645     112.900 110.406  87.490  1.00 30.00           O
ATOM    858  H   GLY B 645     115.709 110.743  89.180  1.00  0.00           H
ATOM    859  HA2 GLY B 645     115.714 108.755  87.095  1.00  0.00           H
ATOM    860  HA3 GLY B 645     114.785 108.749  88.575  1.00  0.00           H
ATOM    861  N   ASP B 646     113.669 109.215  85.716  1.00 30.00           N
ATOM    862  CA  ASP B 646     112.513 109.375  84.838  1.00 30.00           C
ATOM    863  C   ASP B 646     111.579 108.166  85.022  1.00 30.00           C
ATOM    864  O   ASP B 646     112.031 107.023  84.956  1.00 30.00           O
ATOM    865  CB  ASP B 646     112.967 109.540  83.365  1.00 20.00           C
ATOM    866  CG  ASP B 646     111.850 109.814  82.348  1.00 20.00           C
ATOM    867  OD1 ASP B 646     110.737 110.207  82.768  1.00 20.00           O
ATOM    868  OD2 ASP B 646     112.150 109.669  81.144  1.00 20.00           O1-
ATOM    869  H   ASP B 646     114.418 108.645  85.349  1.00  0.00           H
ATOM    870  HA  ASP B 646     111.977 110.284  85.123  1.00  0.00           H
ATOM    871  HB2 ASP B 646     113.678 110.364  83.305  1.00  0.00           H
ATOM    872  HB3 ASP B 646     113.492 108.635  83.057  1.00  0.00           H
ATOM    873  N   LEU B 647     110.286 108.459  85.227  1.00 30.00           N
ATOM    874  CA  LEU B 647     109.191 107.491  85.331  1.00 30.00           C
ATOM    875  C   LEU B 647     108.862 106.800  83.996  1.00 30.00           C
ATOM    876  O   LEU B 647     108.408 105.659  84.017  1.00 30.00           O
ATOM    877  CB  LEU B 647     107.937 108.229  85.846  1.00 20.00           C
ATOM    878  CG  LEU B 647     108.045 108.725  87.304  1.00 20.00           C
ATOM    879  CD1 LEU B 647     106.949 109.763  87.615  1.00 20.00           C
ATOM    880  CD2 LEU B 647     108.028 107.557  88.310  1.00 20.00           C
ATOM    881  H   LEU B 647     110.016 109.430  85.256  1.00  0.00           H
ATOM    882  HA  LEU B 647     109.475 106.715  86.043  1.00  0.00           H
ATOM    883  HB2 LEU B 647     107.746 109.074  85.182  1.00  0.00           H
ATOM    884  HB3 LEU B 647     107.057 107.588  85.759  1.00  0.00           H
ATOM    885  HG  LEU B 647     108.996 109.246  87.421  1.00  0.00           H
ATOM    886 HD11 LEU B 647     107.368 110.633  88.119  1.00  0.00           H
ATOM    887 HD12 LEU B 647     106.452 110.127  86.715  1.00  0.00           H
ATOM    888 HD13 LEU B 647     106.171 109.348  88.254  1.00  0.00           H
ATOM    889 HD21 LEU B 647     107.577 107.844  89.260  1.00  0.00           H
ATOM    890 HD22 LEU B 647     107.475 106.697  87.928  1.00  0.00           H
ATOM    891 HD23 LEU B 647     109.038 107.215  88.532  1.00  0.00           H
ATOM    892  N   GLU B 648     109.090 107.499  82.873  1.00 30.00           N
ATOM    893  CA  GLU B 648     108.762 107.051  81.520  1.00 30.00           C
ATOM    894  C   GLU B 648     110.057 106.826  80.713  1.00 30.00           C
ATOM    895  O   GLU B 648     110.120 107.203  79.543  1.00 30.00           O
ATOM    896  CB  GLU B 648     107.819 108.096  80.870  1.00 20.00           C
ATOM    897  CG  GLU B 648     106.475 108.339  81.599  1.00  0.00           C
ATOM    898  CD  GLU B 648     105.495 107.167  81.498  1.00  0.00           C
ATOM    899  OE1 GLU B 648     105.662 106.196  82.266  1.00  0.00           O
ATOM    900  OE2 GLU B 648     104.577 107.271  80.654  1.00  0.00           O1-
ATOM    901  H   GLU B 648     109.509 108.418  82.937  1.00  0.00           H
ATOM    902  HA  GLU B 648     108.251 106.087  81.542  1.00  0.00           H
ATOM    903  HB2 GLU B 648     108.344 109.050  80.796  1.00  0.00           H
ATOM    904  HB3 GLU B 648     107.601 107.804  79.841  1.00  0.00           H
ATOM    905  HG2 GLU B 648     106.636 108.578  82.650  1.00  0.00           H
ATOM    906  HG3 GLU B 648     105.995 109.219  81.168  1.00  0.00           H
ATOM    907  N   PHE B 649     111.070 106.209  81.353  1.00 30.00           N
ATOM    908  CA  PHE B 649     112.399 105.953  80.779  1.00 30.00           C
ATOM    909  C   PHE B 649     112.414 104.883  79.666  1.00 30.00           C
ATOM    910  O   PHE B 649     113.427 104.764  78.977  1.00 30.00           O
ATOM    911  CB  PHE B 649     113.393 105.615  81.919  1.00 20.00           C
```

```
ATOM    912  CG  PHE B 649     113.326 104.225  82.541  1.00 20.00           C
ATOM    913  CD1 PHE B 649     112.378 103.930  83.545  1.00 20.00           C
ATOM    914  CD2 PHE B 649     114.135 103.182  82.041  1.00 20.00           C
ATOM    915  CE1 PHE B 649     112.310 102.650  84.081  1.00 20.00           C
ATOM    916  CE2 PHE B 649     114.052 101.909  82.591  1.00 20.00           C
ATOM    917  CZ  PHE B 649     113.147 101.647  83.612  1.00 20.00           C
ATOM    918  H   PHE B 649     110.946 105.933  82.317  1.00  0.00           H
ATOM    919  HA  PHE B 649     112.730 106.890  80.327  1.00  0.00           H
ATOM    920  HB2 PHE B 649     113.297 106.352  82.715  1.00  0.00           H
ATOM    921  HB3 PHE B 649     114.408 105.749  81.540  1.00  0.00           H
ATOM    922  HD1 PHE B 649     111.710 104.695  83.910  1.00  0.00           H
ATOM    923  HD2 PHE B 649     114.836 103.373  81.242  1.00  0.00           H
ATOM    924  HE1 PHE B 649     111.594 102.431  84.860  1.00  0.00           H
ATOM    925  HE2 PHE B 649     114.690 101.120  82.222  1.00  0.00           H
ATOM    926  HZ  PHE B 649     113.084 100.654  84.033  1.00  0.00           H
ATOM    927  N   THR B 650     111.316 104.126  79.512  1.00 30.00           N
ATOM    928  CA  THR B 650     111.171 103.055  78.531  1.00 30.00           C
ATOM    929  C   THR B 650     109.675 102.724  78.324  1.00 30.00           C
ATOM    930  O   THR B 650     108.856 103.059  79.184  1.00 30.00           O
ATOM    931  CB  THR B 650     111.984 101.797  78.965  1.00 20.00           C
ATOM    932  CG2 THR B 650     111.500 101.110  80.253  1.00 20.00           C
ATOM    933  OG1 THR B 650     111.973 100.815  77.953  1.00 20.00           O
ATOM    934  H   THR B 650     110.511 104.290  80.100  1.00  0.00           H
ATOM    935  HA  THR B 650     111.556 103.419  77.577  1.00  0.00           H
ATOM    936  HB  THR B 650     113.024 102.085  79.111  1.00  0.00           H
ATOM    937  HG1 THR B 650     112.436 101.159  77.185  1.00  0.00           H
ATOM    938 HG21 THR B 650     112.188 100.318  80.549  1.00  0.00           H
ATOM    939 HG22 THR B 650     111.446 101.818  81.079  1.00  0.00           H
ATOM    940 HG23 THR B 650     110.519 100.656  80.133  1.00  0.00           H
ATOM    941  N   GLU B 651     109.348 102.106  77.174  1.00 30.00           N
ATOM    942  CA  GLU B 651     107.983 101.751  76.762  1.00 30.00           C
ATOM    943  C   GLU B 651     107.856 100.302  76.243  1.00 30.00           C
ATOM    944  O   GLU B 651     106.728  99.859  76.031  1.00 30.00           O
ATOM    945  CB  GLU B 651     107.481 102.752  75.696  1.00 20.00           C
ATOM    946  CG  GLU B 651     107.339 104.203  76.206  1.00  0.00           C
ATOM    947  CD  GLU B 651     106.743 105.163  75.172  1.00  0.00           C
ATOM    948  OE1 GLU B 651     106.907 104.901  73.959  1.00  0.00           O
ATOM    949  OE2 GLU B 651     106.132 106.159  75.617  1.00  0.00           O1-
ATOM    950  H   GLU B 651     110.077 101.887  76.512  1.00  0.00           H
ATOM    951  HA  GLU B 651     107.310 101.814  77.617  1.00  0.00           H
ATOM    952  HB2 GLU B 651     108.162 102.734  74.844  1.00  0.00           H
ATOM    953  HB3 GLU B 651     106.513 102.424  75.311  1.00  0.00           H
ATOM    954  HG2 GLU B 651     106.711 104.217  77.098  1.00  0.00           H
ATOM    955  HG3 GLU B 651     108.310 104.600  76.502  1.00  0.00           H
ATOM    956  N   ASN B 652     108.984  99.595  76.036  1.00 30.00           N
ATOM    957  CA  ASN B 652     109.061  98.237  75.468  1.00 30.00           C
ATOM    958  C   ASN B 652     108.699  97.144  76.496  1.00 30.00           C
ATOM    959  O   ASN B 652     109.583  96.496  77.061  1.00 30.00           O
ATOM    960  CB  ASN B 652     110.427  98.006  74.756  1.00 20.00           C
ATOM    961  CG  ASN B 652     111.651  98.650  75.420  1.00 20.00           C
ATOM    962  ND2 ASN B 652     112.036  98.151  76.593  1.00 20.00           N
ATOM    963  OD1 ASN B 652     112.232  99.595  74.890  1.00 20.00           O
ATOM    964  H   ASN B 652     109.871 100.015  76.270  1.00  0.00           H
ATOM    965  HA  ASN B 652     108.310  98.236  74.675  1.00  0.00           H
ATOM    966  HB2 ASN B 652     110.360  98.452  73.763  1.00  0.00           H
ATOM    967  HB3 ASN B 652     110.609  96.944  74.576  1.00  0.00           H
ATOM    968 HD21 ASN B 652     112.815  98.554  77.091  1.00  0.00           H
ATOM    969 HD22 ASN B 652     111.530  97.375  76.998  1.00  0.00           H
ATOM    970  N   TYR B 653     107.387  96.975  76.709  1.00 30.00           N
ATOM    971  CA  TYR B 653     106.773  96.030  77.642  1.00 30.00           C
ATOM    972  C   TYR B 653     105.267  95.929  77.361  1.00 30.00           C
ATOM    973  O   TYR B 653     104.687  96.840  76.766  1.00 30.00           O
ATOM    974  CB  TYR B 653     107.039  96.458  79.115  1.00 20.00           C
ATOM    975  CG  TYR B 653     106.724  97.907  79.470  1.00 20.00           C
ATOM    976  CD1 TYR B 653     107.777  98.811  79.718  1.00 20.00           C
ATOM    977  CD2 TYR B 653     105.391  98.368  79.530  1.00 20.00           C
ATOM    978  CE1 TYR B 653     107.498 100.166  79.975  1.00 20.00           C
ATOM    979  CE2 TYR B 653     105.118  99.731  79.745  1.00 20.00           C
ATOM    980  CZ  TYR B 653     106.172 100.636  79.958  1.00 20.00           C
ATOM    981  OH  TYR B 653     105.908 101.962  80.139  1.00 20.00           O
ATOM    982  H   TYR B 653     106.730  97.563  76.214  1.00  0.00           H
```

```
ATOM    983  HA  TYR B 653     107.215  95.048  77.466  1.00  0.00           H
ATOM    984  HB2 TYR B 653     108.088  96.288  79.345  1.00  0.00           H
ATOM    985  HB3 TYR B 653     106.499  95.807  79.804  1.00  0.00           H
ATOM    986  HD1 TYR B 653     108.804  98.476  79.694  1.00  0.00           H
ATOM    987  HD2 TYR B 653     104.566  97.691  79.370  1.00  0.00           H
ATOM    988  HE1 TYR B 653     108.308 100.846  80.170  1.00  0.00           H
ATOM    989  HE2 TYR B 653     104.101 100.083  79.730  1.00  0.00           H
ATOM    990  HH  TYR B 653     106.704 102.490  80.245  1.00  0.00           H
ATOM    991  N   ASP B 654     104.654  94.864  77.898  1.00 30.00           N
ATOM    992  CA  ASP B 654     103.224  94.822  78.218  1.00 30.00           C
ATOM    993  C   ASP B 654     103.016  95.215  79.692  1.00 30.00           C
ATOM    994  O   ASP B 654     103.935  95.095  80.504  1.00 30.00           O
ATOM    995  CB  ASP B 654     102.569  93.446  77.938  1.00 20.00           C
ATOM    996  CG  ASP B 654     102.626  92.966  76.484  1.00  0.00           C
ATOM    997  OD1 ASP B 654     102.849  93.810  75.589  1.00  0.00           O
ATOM    998  OD2 ASP B 654     102.324  91.769  76.289  1.00  0.00           O1-
ATOM    999  H   ASP B 654     105.212  94.143  78.332  1.00  0.00           H
ATOM   1000  HA  ASP B 654     102.695  95.563  77.615  1.00  0.00           H
ATOM   1001  HB2 ASP B 654     103.070  92.696  78.552  1.00  0.00           H
ATOM   1002  HB3 ASP B 654     101.518  93.471  78.231  1.00  0.00           H
ATOM   1003  N   PHE B 655     101.779  95.637  80.003  1.00 30.00           N
ATOM   1004  CA  PHE B 655     101.259  95.950  81.340  1.00 30.00           C
ATOM   1005  C   PHE B 655     101.935  97.203  81.934  1.00 30.00           C
ATOM   1006  O   PHE B 655     102.765  97.088  82.837  1.00 30.00           O
ATOM   1007  CB  PHE B 655     101.339  94.715  82.282  1.00 20.00           C
ATOM   1008  CG  PHE B 655     100.761  93.436  81.698  1.00 20.00           C
ATOM   1009  CD1 PHE B 655      99.368  93.301  81.518  1.00 20.00           C
ATOM   1010  CD2 PHE B 655     101.620  92.448  81.169  1.00 20.00           C
ATOM   1011  CE1 PHE B 655      98.861  92.204  80.833  1.00 20.00           C
ATOM   1012  CE2 PHE B 655     101.094  91.362  80.484  1.00 20.00           C
ATOM   1013  CZ  PHE B 655      99.721  91.248  80.308  1.00 20.00           C
ATOM   1014  H   PHE B 655     101.097  95.689  79.260  1.00  0.00           H
ATOM   1015  HA  PHE B 655     100.204  96.190  81.204  1.00  0.00           H
ATOM   1016  HB2 PHE B 655     100.789  94.941  83.194  1.00  0.00           H
ATOM   1017  HB3 PHE B 655     102.366  94.518  82.595  1.00  0.00           H
ATOM   1018  HD1 PHE B 655      98.694  94.058  81.891  1.00  0.00           H
ATOM   1019  HD2 PHE B 655     102.692  92.547  81.267  1.00  0.00           H
ATOM   1020  HE1 PHE B 655      97.795  92.102  80.697  1.00  0.00           H
ATOM   1021  HE2 PHE B 655     101.755  90.613  80.073  1.00  0.00           H
ATOM   1022  HZ  PHE B 655      99.321  90.405  79.764  1.00  0.00           H
ATOM   1023  N   LYS B 656     101.559  98.385  81.409  1.00 30.00           N
ATOM   1024  CA  LYS B 656     102.062  99.688  81.865  1.00 30.00           C
ATOM   1025  C   LYS B 656     101.674 100.029  83.316  1.00 30.00           C
ATOM   1026  O   LYS B 656     102.452 100.695  83.999  1.00 30.00           O
ATOM   1027  CB  LYS B 656     101.629 100.806  80.889  1.00 20.00           C
ATOM   1028  CG  LYS B 656     102.288 102.164  81.206  1.00 20.00           C
ATOM   1029  CD  LYS B 656     102.082 103.234  80.128  1.00 20.00           C
ATOM   1030  CE  LYS B 656     102.870 104.508  80.463  1.00 20.00           C
ATOM   1031  NZ  LYS B 656     102.697 105.548  79.439  1.00 20.00           N1+
ATOM   1032  H   LYS B 656     100.889  98.405  80.653  1.00  0.00           H
ATOM   1033  HA  LYS B 656     103.150  99.625  81.836  1.00  0.00           H
ATOM   1034  HB2 LYS B 656     101.903 100.514  79.875  1.00  0.00           H
ATOM   1035  HB3 LYS B 656     100.543 100.910  80.887  1.00  0.00           H
ATOM   1036  HG2 LYS B 656     101.900 102.555  82.147  1.00  0.00           H
ATOM   1037  HG3 LYS B 656     103.357 102.014  81.360  1.00  0.00           H
ATOM   1038  HD2 LYS B 656     102.399 102.846  79.159  1.00  0.00           H
ATOM   1039  HD3 LYS B 656     101.020 103.465  80.043  1.00  0.00           H
ATOM   1040  HE2 LYS B 656     102.559 104.912  81.427  1.00  0.00           H
ATOM   1041  HE3 LYS B 656     103.935 104.283  80.543  1.00  0.00           H
ATOM   1042  HZ1 LYS B 656     103.269 106.342  79.711  1.00  0.00           H
ATOM   1043  HZ2 LYS B 656     103.012 105.204  78.543  1.00  0.00           H
ATOM   1044  HZ3 LYS B 656     101.729 105.827  79.385  1.00  0.00           H
ATOM   1045  N   ALA B 657     100.504  99.539  83.766  1.00 30.00           N
ATOM   1046  CA  ALA B 657     100.031  99.651  85.145  1.00 30.00           C
ATOM   1047  C   ALA B 657     100.982  98.984  86.151  1.00 30.00           C
ATOM   1048  O   ALA B 657     101.282  99.594  87.171  1.00 30.00           O
ATOM   1049  CB  ALA B 657      98.618  99.059  85.250  1.00 30.00           C
ATOM   1050  H   ALA B 657      99.920  99.007  83.138  1.00  0.00           H
ATOM   1051  HA  ALA B 657      99.973 100.713  85.390  1.00  0.00           H
ATOM   1052  HB1 ALA B 657      98.231  99.138  86.267  1.00  0.00           H
ATOM   1053  HB2 ALA B 657      97.923  99.587  84.596  1.00  0.00           H
```

```
ATOM   1054  HB3 ALA B 657      98.601  98.005  84.970  1.00  0.00           H
ATOM   1055  N   VAL B 658     101.483  97.782  85.815  1.00 30.00           N
ATOM   1056  CA  VAL B 658     102.462  97.038  86.611  1.00 30.00           C
ATOM   1057  C   VAL B 658     103.830  97.749  86.641  1.00 30.00           C
ATOM   1058  O   VAL B 658     104.412  97.859  87.718  1.00 30.00           O
ATOM   1059  CB  VAL B 658     102.660  95.593  86.066  1.00 20.00           C
ATOM   1060  CG1 VAL B 658     103.700  94.749  86.837  1.00 20.00           C
ATOM   1061  CG2 VAL B 658     101.329  94.824  86.029  1.00 20.00           C
ATOM   1062  H   VAL B 658     101.212  97.359  84.940  1.00  0.00           H
ATOM   1063  HA  VAL B 658     102.087  96.976  87.634  1.00  0.00           H
ATOM   1064  HB  VAL B 658     103.012  95.667  85.037  1.00  0.00           H
ATOM   1065 HG11 VAL B 658     103.807  93.761  86.391  1.00  0.00           H
ATOM   1066 HG12 VAL B 658     104.691  95.197  86.843  1.00  0.00           H
ATOM   1067 HG13 VAL B 658     103.405  94.607  87.875  1.00  0.00           H
ATOM   1068 HG21 VAL B 658     101.459  93.874  85.516  1.00  0.00           H
ATOM   1069 HG22 VAL B 658     100.957  94.622  87.034  1.00  0.00           H
ATOM   1070 HG23 VAL B 658     100.550  95.365  85.492  1.00  0.00           H
ATOM   1071  N   PHE B 659     104.284  98.251  85.476  1.00 30.00           N
ATOM   1072  CA  PHE B 659     105.542  98.983  85.290  1.00 30.00           C
ATOM   1073  C   PHE B 659     105.676 100.223  86.182  1.00 30.00           C
ATOM   1074  O   PHE B 659     106.705 100.394  86.834  1.00 30.00           O
ATOM   1075  CB  PHE B 659     105.733  99.323  83.790  1.00 20.00           C
ATOM   1076  CG  PHE B 659     106.892 100.242  83.438  1.00 20.00           C
ATOM   1077  CD1 PHE B 659     108.216  99.753  83.425  1.00 20.00           C
ATOM   1078  CD2 PHE B 659     106.668 101.621  83.236  1.00 20.00           C
ATOM   1079  CE1 PHE B 659     109.270 100.615  83.148  1.00 20.00           C
ATOM   1080  CE2 PHE B 659     107.732 102.463  82.944  1.00 20.00           C
ATOM   1081  CZ  PHE B 659     109.026 101.961  82.898  1.00 20.00           C
ATOM   1082  H   PHE B 659     103.729  98.117  84.641  1.00  0.00           H
ATOM   1083  HA  PHE B 659     106.350  98.312  85.586  1.00  0.00           H
ATOM   1084  HB2 PHE B 659     105.861  98.409  83.213  1.00  0.00           H
ATOM   1085  HB3 PHE B 659     104.823  99.774  83.401  1.00  0.00           H
ATOM   1086  HD1 PHE B 659     108.412  98.709  83.615  1.00  0.00           H
ATOM   1087  HD2 PHE B 659     105.666 102.024  83.283  1.00  0.00           H
ATOM   1088  HE1 PHE B 659     110.281 100.239  83.120  1.00  0.00           H
ATOM   1089  HE2 PHE B 659     107.552 103.511  82.760  1.00  0.00           H
ATOM   1090  HZ  PHE B 659     109.845 102.628  82.673  1.00  0.00           H
ATOM   1091  N   ILE B 660     104.626 101.056  86.190  1.00 30.00           N
ATOM   1092  CA  ILE B 660     104.617 102.317  86.919  1.00 30.00           C
ATOM   1093  C   ILE B 660     104.237 102.143  88.404  1.00 30.00           C
ATOM   1094  O   ILE B 660     104.624 102.993  89.203  1.00 30.00           O
ATOM   1095  CB  ILE B 660     103.690 103.346  86.217  1.00 20.00           C
ATOM   1096  CG1 ILE B 660     104.111 104.802  86.517  1.00 20.00           C
ATOM   1097  CG2 ILE B 660     102.179 103.143  86.455  1.00 20.00           C
ATOM   1098  CD1 ILE B 660     103.808 105.765  85.362  1.00 20.00           C
ATOM   1099  H   ILE B 660     103.813 100.852  85.623  1.00  0.00           H
ATOM   1100  HA  ILE B 660     105.634 102.712  86.898  1.00  0.00           H
ATOM   1101  HB  ILE B 660     103.848 103.182  85.150  1.00  0.00           H
ATOM   1102 HG12 ILE B 660     103.634 105.151  87.433  1.00  0.00           H
ATOM   1103 HG13 ILE B 660     105.183 104.857  86.709  1.00  0.00           H
ATOM   1104 HG21 ILE B 660     101.590 103.791  85.806  1.00  0.00           H
ATOM   1105 HG22 ILE B 660     101.881 102.117  86.244  1.00  0.00           H
ATOM   1106 HG23 ILE B 660     101.891 103.367  87.483  1.00  0.00           H
ATOM   1107 HD11 ILE B 660     104.649 106.436  85.192  1.00  0.00           H
ATOM   1108 HD12 ILE B 660     103.633 105.250  84.417  1.00  0.00           H
ATOM   1109 HD13 ILE B 660     102.926 106.366  85.580  1.00  0.00           H
ATOM   1110  N   ILE B 661     103.564 101.035  88.765  1.00 30.00           N
ATOM   1111  CA  ILE B 661     103.384 100.616  90.157  1.00 30.00           C
ATOM   1112  C   ILE B 661     104.731 100.273  90.817  1.00 30.00           C
ATOM   1113  O   ILE B 661     104.975 100.753  91.923  1.00 30.00           O
ATOM   1114  CB  ILE B 661     102.372  99.434  90.321  1.00 20.00           C
ATOM   1115  CG1 ILE B 661     100.923  99.961  90.200  1.00 20.00           C
ATOM   1116  CG2 ILE B 661     102.493  98.601  91.622  1.00 20.00           C
ATOM   1117  CD1 ILE B 661      99.879  98.862  89.936  1.00 20.00           C
ATOM   1118  H   ILE B 661     103.225 100.398  88.055  1.00  0.00           H
ATOM   1119  HA  ILE B 661     102.984 101.475  90.699  1.00  0.00           H
ATOM   1120  HB  ILE B 661     102.546  98.747  89.491  1.00  0.00           H
ATOM   1121 HG12 ILE B 661     100.656 100.498  91.110  1.00  0.00           H
ATOM   1122 HG13 ILE B 661     100.858 100.704  89.404  1.00  0.00           H
ATOM   1123 HG21 ILE B 661     101.731  97.823  91.673  1.00  0.00           H
ATOM   1124 HG22 ILE B 661     103.451  98.088  91.703  1.00  0.00           H
```

```
ATOM   1125 HG23 ILE B 661     102.378  99.229  92.506  1.00  0.00           H
ATOM   1126 HD11 ILE B 661      99.036  99.261  89.372  1.00  0.00           H
ATOM   1127 HD12 ILE B 661     100.296  98.039  89.354  1.00  0.00           H
ATOM   1128 HD13 ILE B 661      99.490  98.452  90.868  1.00  0.00           H
ATOM   1129  N   LEU B 662     105.595  99.512  90.120  1.00 30.00           N
ATOM   1130  CA  LEU B 662     106.955  99.201  90.580  1.00 30.00           C
ATOM   1131  C   LEU B 662     107.772 100.473  90.856  1.00 30.00           C
ATOM   1132  O   LEU B 662     108.427 100.556  91.894  1.00 30.00           O
ATOM   1133  CB  LEU B 662     107.715  98.339  89.548  1.00 20.00           C
ATOM   1134  CG  LEU B 662     107.236  96.882  89.393  1.00 20.00           C
ATOM   1135  CD1 LEU B 662     107.893  96.253  88.144  1.00 20.00           C
ATOM   1136  CD2 LEU B 662     107.457  96.043  90.671  1.00 20.00           C
ATOM   1137  H   LEU B 662     105.320  99.142  89.215  1.00  0.00           H
ATOM   1138  HA  LEU B 662     106.873  98.655  91.521  1.00  0.00           H
ATOM   1139  HB2 LEU B 662     107.667  98.841  88.582  1.00  0.00           H
ATOM   1140  HB3 LEU B 662     108.777  98.317  89.802  1.00  0.00           H
ATOM   1141  HG  LEU B 662     106.163  96.899  89.209  1.00  0.00           H
ATOM   1142 HD11 LEU B 662     108.539  95.410  88.381  1.00  0.00           H
ATOM   1143 HD12 LEU B 662     107.133  95.905  87.446  1.00  0.00           H
ATOM   1144 HD13 LEU B 662     108.512  96.963  87.595  1.00  0.00           H
ATOM   1145 HD21 LEU B 662     107.879  95.061  90.464  1.00  0.00           H
ATOM   1146 HD22 LEU B 662     108.126  96.535  91.377  1.00  0.00           H
ATOM   1147 HD23 LEU B 662     106.512  95.875  91.187  1.00  0.00           H
ATOM   1148  N   LEU B 663     107.687 101.450  89.943  1.00 30.00           N
ATOM   1149  CA  LEU B 663     108.409 102.718  90.063  1.00 30.00           C
ATOM   1150  C   LEU B 663     107.910 103.573  91.236  1.00 30.00           C
ATOM   1151  O   LEU B 663     108.740 104.110  91.965  1.00 30.00           O
ATOM   1152  CB  LEU B 663     108.314 103.509  88.743  1.00 20.00           C
ATOM   1153  CG  LEU B 663     109.095 102.879  87.570  1.00 20.00           C
ATOM   1154  CD1 LEU B 663     108.612 103.446  86.225  1.00 20.00           C
ATOM   1155  CD2 LEU B 663     110.623 103.010  87.750  1.00 20.00           C
ATOM   1156  H   LEU B 663     107.095 101.347  89.131  1.00  0.00           H
ATOM   1157  HA  LEU B 663     109.457 102.486  90.260  1.00  0.00           H
ATOM   1158  HB2 LEU B 663     107.261 103.601  88.473  1.00  0.00           H
ATOM   1159  HB3 LEU B 663     108.669 104.531  88.886  1.00  0.00           H
ATOM   1160  HG  LEU B 663     108.870 101.812  87.550  1.00  0.00           H
ATOM   1161 HD11 LEU B 663     109.432 103.703  85.554  1.00  0.00           H
ATOM   1162 HD12 LEU B 663     107.995 102.712  85.708  1.00  0.00           H
ATOM   1163 HD13 LEU B 663     108.003 104.342  86.353  1.00  0.00           H
ATOM   1164 HD21 LEU B 663     111.089 103.630  86.985  1.00  0.00           H
ATOM   1165 HD22 LEU B 663     110.891 103.457  88.708  1.00  0.00           H
ATOM   1166 HD23 LEU B 663     111.099 102.030  87.705  1.00  0.00           H
ATOM   1167  N   LEU B 664     106.585 103.714  91.372  1.00 30.00           N
ATOM   1168  CA  LEU B 664     105.974 104.505  92.439  1.00 30.00           C
ATOM   1169  C   LEU B 664     106.207 103.846  93.807  1.00 30.00           C
ATOM   1170  O   LEU B 664     106.597 104.545  94.739  1.00 30.00           O
ATOM   1171  CB  LEU B 664     104.474 104.751  92.146  1.00 20.00           C
ATOM   1172  CG  LEU B 664     104.165 106.085  91.419  1.00 20.00           C
ATOM   1173  CD1 LEU B 664     104.944 106.302  90.104  1.00 20.00           C
ATOM   1174  CD2 LEU B 664     102.647 106.269  91.221  1.00 20.00           C
ATOM   1175  H   LEU B 664     105.956 103.281  90.703  1.00  0.00           H
ATOM   1176  HA  LEU B 664     106.477 105.474  92.474  1.00  0.00           H
ATOM   1177  HB2 LEU B 664     104.051 103.907  91.600  1.00  0.00           H
ATOM   1178  HB3 LEU B 664     103.931 104.774  93.093  1.00  0.00           H
ATOM   1179  HG  LEU B 664     104.479 106.880  92.098  1.00  0.00           H
ATOM   1180 HD11 LEU B 664     104.321 106.704  89.305  1.00  0.00           H
ATOM   1181 HD12 LEU B 664     105.746 107.020  90.263  1.00  0.00           H
ATOM   1182 HD13 LEU B 664     105.397 105.390  89.722  1.00  0.00           H
ATOM   1183 HD21 LEU B 664     102.327 107.251  91.572  1.00  0.00           H
ATOM   1184 HD22 LEU B 664     102.347 106.182  90.177  1.00  0.00           H
ATOM   1185 HD23 LEU B 664     102.069 105.528  91.774  1.00  0.00           H
ATOM   1186  N   ALA B 665     106.051 102.515  93.885  1.00 30.00           N
ATOM   1187  CA  ALA B 665     106.377 101.711  95.063  1.00 30.00           C
ATOM   1188  C   ALA B 665     107.833 101.898  95.508  1.00 30.00           C
ATOM   1189  O   ALA B 665     108.066 102.236  96.665  1.00 30.00           O
ATOM   1190  CB  ALA B 665     106.088 100.230  94.782  1.00 20.00           C
ATOM   1191  H   ALA B 665     105.738 101.998  93.066  1.00  0.00           H
ATOM   1192  HA  ALA B 665     105.741 102.045  95.884  1.00  0.00           H
ATOM   1193  HB1 ALA B 665     106.437  99.602  95.602  1.00  0.00           H
ATOM   1194  HB2 ALA B 665     105.021 100.053  94.655  1.00  0.00           H
ATOM   1195  HB3 ALA B 665     106.589  99.894  93.876  1.00  0.00           H
```

```
ATOM   1196  N   TYR B 666     108.770 101.748  94.562  1.00 30.00           N
ATOM   1197  CA  TYR B 666     110.195 101.988  94.769  1.00 30.00           C
ATOM   1198  C   TYR B 666     110.484 103.413  95.276  1.00 30.00           C
ATOM   1199  O   TYR B 666     111.074 103.552  96.346  1.00 30.00           O
ATOM   1200  CB  TYR B 666     110.978 101.637  93.486  1.00 20.00           C
ATOM   1201  CG  TYR B 666     112.459 101.972  93.505  1.00 20.00           C
ATOM   1202  CD1 TYR B 666     113.360 101.217  94.288  1.00 20.00           C
ATOM   1203  CD2 TYR B 666     112.932 103.068  92.758  1.00 20.00           C
ATOM   1204  CE1 TYR B 666     114.725 101.567  94.330  1.00 20.00           C
ATOM   1205  CE2 TYR B 666     114.294 103.410  92.796  1.00 20.00           C
ATOM   1206  CZ  TYR B 666     115.194 102.664  93.582  1.00 20.00           C
ATOM   1207  OH  TYR B 666     116.512 103.020  93.622  1.00 20.00           O
ATOM   1208  H   TYR B 666     108.496 101.459  93.627  1.00  0.00           H
ATOM   1209  HA  TYR B 666     110.519 101.296  95.550  1.00  0.00           H
ATOM   1210  HB2 TYR B 666     110.873 100.577  93.271  1.00  0.00           H
ATOM   1211  HB3 TYR B 666     110.528 102.151  92.637  1.00  0.00           H
ATOM   1212  HD1 TYR B 666     113.006 100.377  94.869  1.00  0.00           H
ATOM   1213  HD2 TYR B 666     112.254 103.659  92.157  1.00  0.00           H
ATOM   1214  HE1 TYR B 666     115.408 101.002  94.947  1.00  0.00           H
ATOM   1215  HE2 TYR B 666     114.628 104.253  92.216  1.00  0.00           H
ATOM   1216  HH  TYR B 666     116.707 103.659  92.917  1.00  0.00           H
ATOM   1217  N   VAL B 667     110.024 104.441  94.543  1.00 30.00           N
ATOM   1218  CA  VAL B 667     110.223 105.852  94.885  1.00 30.00           C
ATOM   1219  C   VAL B 667     109.691 106.214  96.287  1.00 30.00           C
ATOM   1220  O   VAL B 667     110.423 106.848  97.053  1.00 30.00           O
ATOM   1221  CB  VAL B 667     109.617 106.803  93.806  1.00 20.00           C
ATOM   1222  CG1 VAL B 667     109.423 108.280  94.222  1.00 20.00           C
ATOM   1223  CG2 VAL B 667     110.475 106.766  92.530  1.00 20.00           C
ATOM   1224  H   VAL B 667     109.507 104.254  93.687  1.00  0.00           H
ATOM   1225  HA  VAL B 667     111.303 106.013  94.911  1.00  0.00           H
ATOM   1226  HB  VAL B 667     108.630 106.418  93.544  1.00  0.00           H
ATOM   1227 HG11 VAL B 667     109.075 108.881  93.382  1.00  0.00           H
ATOM   1228 HG12 VAL B 667     108.677 108.395  95.010  1.00  0.00           H
ATOM   1229 HG13 VAL B 667     110.355 108.722  94.575  1.00  0.00           H
ATOM   1230 HG21 VAL B 667     110.004 107.322  91.719  1.00  0.00           H
ATOM   1231 HG22 VAL B 667     111.455 107.204  92.714  1.00  0.00           H
ATOM   1232 HG23 VAL B 667     110.643 105.752  92.170  1.00  0.00           H
ATOM   1233  N   ILE B 668     108.472 105.763  96.615  1.00  0.00           N
ATOM   1234  CA  ILE B 668     107.878 105.981  97.931  1.00  0.00           C
ATOM   1235  C   ILE B 668     108.627 105.218  99.051  1.00  0.00           C
ATOM   1236  O   ILE B 668     108.808 105.769 100.134  1.00  0.00           O
ATOM   1237  CB  ILE B 668     106.347 105.689  97.961  1.00 20.00           C
ATOM   1238  CG1 ILE B 668     105.557 106.629  97.010  1.00 20.00           C
ATOM   1239  CG2 ILE B 668     105.715 105.721  99.371  1.00 20.00           C
ATOM   1240  CD1 ILE B 668     105.611 108.128  97.347  1.00 20.00           C
ATOM   1241  H   ILE B 668     107.925 105.221  95.950  1.00  0.00           H
ATOM   1242  HA  ILE B 668     107.994 107.039  98.139  1.00  0.00           H
ATOM   1243  HB  ILE B 668     106.204 104.675  97.580  1.00  0.00           H
ATOM   1244 HG12 ILE B 668     104.516 106.307  96.966  1.00  0.00           H
ATOM   1245 HG13 ILE B 668     105.928 106.523  95.993  1.00  0.00           H
ATOM   1246 HG21 ILE B 668     104.629 105.666  99.324  1.00  0.00           H
ATOM   1247 HG22 ILE B 668     106.056 104.888  99.984  1.00  0.00           H
ATOM   1248 HG23 ILE B 668     105.969 106.635  99.906  1.00  0.00           H
ATOM   1249 HD11 ILE B 668     104.790 108.658  96.866  1.00  0.00           H
ATOM   1250 HD12 ILE B 668     105.532 108.320  98.416  1.00  0.00           H
ATOM   1251 HD13 ILE B 668     106.536 108.578  96.986  1.00  0.00           H
ATOM   1252  N   LEU B 669     109.100 104.000  98.760  1.00  0.00           N
ATOM   1253  CA  LEU B 669     109.857 103.159  99.687  1.00  0.00           C
ATOM   1254  C   LEU B 669     111.271 103.695 100.001  1.00  0.00           C
ATOM   1255  O   LEU B 669     111.751 103.506 101.120  1.00  0.00           O
ATOM   1256  CB  LEU B 669     109.889 101.722  99.112  1.00 20.00           C
ATOM   1257  CG  LEU B 669     110.614 100.633  99.932  1.00 20.00           C
ATOM   1258  CD1 LEU B 669     109.936 100.384 101.295  1.00 20.00           C
ATOM   1259  CD2 LEU B 669     110.752  99.343  99.100  1.00 20.00           C
ATOM   1260  H   LEU B 669     108.891 103.585  97.856  1.00  0.00           H
ATOM   1261  HA  LEU B 669     109.306 103.148 100.627  1.00  0.00           H
ATOM   1262  HB2 LEU B 669     108.865 101.392  98.941  1.00  0.00           H
ATOM   1263  HB3 LEU B 669     110.355 101.763  98.128  1.00  0.00           H
ATOM   1264  HG  LEU B 669     111.631 100.970 100.135  1.00  0.00           H
ATOM   1265 HD11 LEU B 669     110.662 100.464 102.104  1.00  0.00           H
ATOM   1266 HD12 LEU B 669     109.146 101.101 101.511  1.00  0.00           H
```

```
ATOM   1267 HD13 LEU B 669     109.476  99.399 101.353  1.00  0.00           H
ATOM   1268 HD21 LEU B 669     111.761  99.257  98.696  1.00  0.00           H
ATOM   1269 HD22 LEU B 669     110.568  98.439  99.680  1.00  0.00           H
ATOM   1270 HD23 LEU B 669     110.070  99.335  98.250  1.00  0.00           H
ATOM   1271  N   THR B 670     111.926 104.344  99.031  1.00  0.00           N
ATOM   1272  CA  THR B 670     113.358 104.652  99.118  1.00  0.00           C
ATOM   1273  C   THR B 670     113.672 106.161  99.135  1.00  0.00           C
ATOM   1274  O   THR B 670     114.486 106.576  99.958  1.00  0.00           O
ATOM   1275  CB  THR B 670     114.201 103.948  98.012  1.00 20.00           C
ATOM   1276  CG2 THR B 670     114.147 104.562  96.599  1.00 20.00           C
ATOM   1277  OG1 THR B 670     115.567 103.981  98.377  1.00 20.00           O
ATOM   1278  H   THR B 670     111.480 104.473  98.133  1.00  0.00           H
ATOM   1279  HA  THR B 670     113.744 104.265 100.059  1.00  0.00           H
ATOM   1280  HB  THR B 670     113.896 102.902  97.952  1.00  0.00           H
ATOM   1281  HG1 THR B 670     115.873 104.890  98.341  1.00  0.00           H
ATOM   1282 HG21 THR B 670     114.632 103.902  95.885  1.00  0.00           H
ATOM   1283 HG22 THR B 670     113.129 104.726  96.258  1.00  0.00           H
ATOM   1284 HG23 THR B 670     114.668 105.519  96.540  1.00  0.00           H
ATOM   1285  N   TYR B 671     113.063 106.952  98.235  1.00  0.00           N
ATOM   1286  CA  TYR B 671     113.467 108.343  98.016  1.00  0.00           C
ATOM   1287  C   TYR B 671     112.851 109.320  99.028  1.00  0.00           C
ATOM   1288  O   TYR B 671     113.551 110.223  99.481  1.00  0.00           O
ATOM   1289  CB  TYR B 671     113.181 108.771  96.561  1.00 20.00           C
ATOM   1290  CG  TYR B 671     113.685 110.168  96.242  1.00 20.00           C
ATOM   1291  CD1 TYR B 671     115.071 110.418  96.247  1.00 20.00           C
ATOM   1292  CD2 TYR B 671     112.787 111.231  96.014  1.00 20.00           C
ATOM   1293  CE1 TYR B 671     115.568 111.714  96.019  1.00 20.00           C
ATOM   1294  CE2 TYR B 671     113.286 112.530  95.802  1.00 20.00           C
ATOM   1295  CZ  TYR B 671     114.672 112.773  95.795  1.00 20.00           C
ATOM   1296  OH  TYR B 671     115.143 114.033  95.571  1.00 20.00           O
ATOM   1297  H   TYR B 671     112.366 106.576  97.606  1.00  0.00           H
ATOM   1298  HA  TYR B 671     114.550 108.395  98.151  1.00  0.00           H
ATOM   1299  HB2 TYR B 671     113.661 108.077  95.869  1.00  0.00           H
ATOM   1300  HB3 TYR B 671     112.113 108.718  96.353  1.00  0.00           H
ATOM   1301  HD1 TYR B 671     115.756 109.610  96.440  1.00  0.00           H
ATOM   1302  HD2 TYR B 671     111.721 111.061  96.023  1.00  0.00           H
ATOM   1303  HE1 TYR B 671     116.634 111.893  96.020  1.00  0.00           H
ATOM   1304  HE2 TYR B 671     112.607 113.348  95.644  1.00  0.00           H
ATOM   1305  HH  TYR B 671     114.450 114.645  95.313  1.00  0.00           H
ATOM   1306  N   ILE B 672     111.567 109.123  99.361  1.00  0.00           N
ATOM   1307  CA  ILE B 672     110.850 109.957 100.329  1.00  0.00           C
ATOM   1308  C   ILE B 672     110.715 109.267 101.701  1.00  0.00           C
ATOM   1309  O   ILE B 672     110.135 109.879 102.595  1.00  0.00           O
ATOM   1310  CB  ILE B 672     109.438 110.371  99.810  1.00 20.00           C
ATOM   1311  CG1 ILE B 672     108.409 109.216  99.725  1.00 20.00           C
ATOM   1312  CG2 ILE B 672     109.551 111.100  98.457  1.00 20.00           C
ATOM   1313  CD1 ILE B 672     107.487 109.059 100.948  1.00 20.00           C
ATOM   1314  H   ILE B 672     111.043 108.370  98.935  1.00  0.00           H
ATOM   1315  HA  ILE B 672     111.403 110.880 100.514  1.00  0.00           H
ATOM   1316  HB  ILE B 672     109.048 111.120 100.500  1.00  0.00           H
ATOM   1317 HG12 ILE B 672     108.942 108.284  99.551  1.00  0.00           H
ATOM   1318 HG13 ILE B 672     107.773 109.357  98.852  1.00  0.00           H
ATOM   1319 HG21 ILE B 672     108.591 111.525  98.165  1.00  0.00           H
ATOM   1320 HG22 ILE B 672     110.269 111.920  98.508  1.00  0.00           H
ATOM   1321 HG23 ILE B 672     109.869 110.426  97.660  1.00  0.00           H
ATOM   1322 HD11 ILE B 672     106.474 108.798 100.641  1.00  0.00           H
ATOM   1323 HD12 ILE B 672     107.840 108.265 101.606  1.00  0.00           H
ATOM   1324 HD13 ILE B 672     107.420 109.972 101.540  1.00  0.00           H
ATOM   1325  N   LEU B 673     111.222 108.035 101.850  1.00 30.00           N
ATOM   1326  CA  LEU B 673     111.184 107.276 103.098  1.00 30.00           C
ATOM   1327  C   LEU B 673     112.623 107.032 103.560  1.00 30.00           C
ATOM   1328  O   LEU B 673     113.073 107.736 104.458  1.00 30.00           O
ATOM   1329  CB  LEU B 673     110.315 106.006 102.931  1.00 20.00           C
ATOM   1330  CG  LEU B 673     110.368 104.955 104.071  1.00 20.00           C
ATOM   1331  CD1 LEU B 673     109.926 105.546 105.425  1.00 20.00           C
ATOM   1332  CD2 LEU B 673     109.547 103.704 103.697  1.00 20.00           C
ATOM   1333  H   LEU B 673     111.692 107.611 101.066  1.00  0.00           H
ATOM   1334  HA  LEU B 673     110.718 107.872 103.885  1.00  0.00           H
ATOM   1335  HB2 LEU B 673     109.280 106.316 102.778  1.00  0.00           H
ATOM   1336  HB3 LEU B 673     110.604 105.523 102.004  1.00  0.00           H
ATOM   1337  HG  LEU B 673     111.394 104.607 104.190  1.00  0.00           H
```

```
ATOM   1338 HD11 LEU B 673     110.781 105.953 105.965  1.00  0.00           H
ATOM   1339 HD12 LEU B 673     109.206 106.354 105.289  1.00  0.00           H
ATOM   1340 HD13 LEU B 673     109.459 104.808 106.077  1.00  0.00           H
ATOM   1341 HD21 LEU B 673     110.202 102.858 103.485  1.00  0.00           H
ATOM   1342 HD22 LEU B 673     108.868 103.393 104.487  1.00  0.00           H
ATOM   1343 HD23 LEU B 673     108.938 103.869 102.808  1.00  0.00           H
ATOM   1344  N   LEU B 674     113.341 106.071 102.956  1.00 30.00           N
ATOM   1345  CA  LEU B 674     114.665 105.637 103.429  1.00 30.00           C
ATOM   1346  C   LEU B 674     115.727 106.762 103.485  1.00 30.00           C
ATOM   1347  O   LEU B 674     116.496 106.822 104.443  1.00 30.00           O
ATOM   1348  CB  LEU B 674     115.154 104.426 102.600  1.00 20.00           C
ATOM   1349  CG  LEU B 674     116.163 103.516 103.342  1.00 20.00           C
ATOM   1350  CD1 LEU B 674     115.492 102.706 104.473  1.00 20.00           C
ATOM   1351  CD2 LEU B 674     116.963 102.629 102.363  1.00 20.00           C
ATOM   1352  H   LEU B 674     112.903 105.495 102.248  1.00  0.00           H
ATOM   1353  HA  LEU B 674     114.511 105.304 104.456  1.00  0.00           H
ATOM   1354  HB2 LEU B 674     114.307 103.811 102.293  1.00  0.00           H
ATOM   1355  HB3 LEU B 674     115.597 104.798 101.675  1.00  0.00           H
ATOM   1356  HG  LEU B 674     116.894 104.165 103.824  1.00  0.00           H
ATOM   1357 HD11 LEU B 674     115.645 101.632 104.371  1.00  0.00           H
ATOM   1358 HD12 LEU B 674     115.897 102.991 105.445  1.00  0.00           H
ATOM   1359 HD13 LEU B 674     114.414 102.865 104.514  1.00  0.00           H
ATOM   1360 HD21 LEU B 674     116.744 101.568 102.470  1.00  0.00           H
ATOM   1361 HD22 LEU B 674     116.772 102.881 101.320  1.00  0.00           H
ATOM   1362 HD23 LEU B 674     118.033 102.745 102.532  1.00  0.00           H
ATOM   1363  N   LEU B 675     115.714 107.673 102.504  1.00  0.00           N
ATOM   1364  CA  LEU B 675     116.612 108.830 102.425  1.00  0.00           C
ATOM   1365  C   LEU B 675     116.282 109.881 103.504  1.00  0.00           C
ATOM   1366  O   LEU B 675     117.206 110.502 104.027  1.00  0.00           O
ATOM   1367  CB  LEU B 675     116.560 109.435 101.002  1.00 20.00           C
ATOM   1368  CG  LEU B 675     117.666 108.967 100.024  1.00 20.00           C
ATOM   1369  CD1 LEU B 675     117.639 107.455  99.715  1.00 20.00           C
ATOM   1370  CD2 LEU B 675     117.657 109.813  98.733  1.00 20.00           C
ATOM   1371  H   LEU B 675     115.038 107.576 101.759  1.00  0.00           H
ATOM   1372  HA  LEU B 675     117.631 108.487 102.620  1.00  0.00           H
ATOM   1373  HB2 LEU B 675     115.584 109.251 100.559  1.00  0.00           H
ATOM   1374  HB3 LEU B 675     116.641 110.522 101.076  1.00  0.00           H
ATOM   1375  HG  LEU B 675     118.616 109.166 100.522  1.00  0.00           H
ATOM   1376 HD11 LEU B 675     118.652 107.068  99.611  1.00  0.00           H
ATOM   1377 HD12 LEU B 675     117.160 106.870 100.498  1.00  0.00           H
ATOM   1378 HD13 LEU B 675     117.109 107.233  98.788  1.00  0.00           H
ATOM   1379 HD21 LEU B 675     118.589 110.369  98.626  1.00  0.00           H
ATOM   1380 HD22 LEU B 675     117.546 109.206  97.837  1.00  0.00           H
ATOM   1381 HD23 LEU B 675     116.845 110.541  98.724  1.00  0.00           H
ATOM   1382  N   ASN B 676     114.991 110.019 103.856  1.00  0.00           N
ATOM   1383  CA  ASN B 676     114.512 110.861 104.959  1.00  0.00           C
ATOM   1384  C   ASN B 676     114.772 110.205 106.331  1.00  0.00           C
ATOM   1385  O   ASN B 676     115.010 110.929 107.297  1.00  0.00           O
ATOM   1386  CB  ASN B 676     113.007 111.174 104.765  1.00 20.00           C
ATOM   1387  CG  ASN B 676     112.682 112.089 103.574  1.00 20.00           C
ATOM   1388  ND2 ASN B 676     111.419 112.507 103.471  1.00 20.00           N
ATOM   1389  OD1 ASN B 676     113.543 112.434 102.767  1.00 20.00           O
ATOM   1390  H   ASN B 676     114.289 109.472 103.379  1.00  0.00           H
ATOM   1391  HA  ASN B 676     115.069 111.798 104.913  1.00  0.00           H
ATOM   1392  HB2 ASN B 676     112.631 111.680 105.656  1.00  0.00           H
ATOM   1393  HB3 ASN B 676     112.432 110.251 104.671  1.00  0.00           H
ATOM   1394 HD21 ASN B 676     111.151 113.125 102.720  1.00  0.00           H
ATOM   1395 HD22 ASN B 676     110.722 112.201 104.135  1.00  0.00           H
ATOM   1396  N   MET B 677     114.759 108.860 106.385  1.00  0.00           N
ATOM   1397  CA  MET B 677     115.076 108.050 107.563  1.00  0.00           C
ATOM   1398  C   MET B 677     116.571 108.085 107.912  1.00  0.00           C
ATOM   1399  O   MET B 677     116.907 108.055 109.094  1.00  0.00           O
ATOM   1400  CB  MET B 677     114.612 106.591 107.341  1.00 20.00           C
ATOM   1401  CG  MET B 677     114.410 105.774 108.627  1.00 20.00           C
ATOM   1402  SD  MET B 677     113.224 106.462 109.820  1.00 20.00           S
ATOM   1403  CE  MET B 677     111.770 106.663 108.754  1.00 20.00           C
ATOM   1404  H   MET B 677     114.508 108.342 105.553  1.00  0.00           H
ATOM   1405  HA  MET B 677     114.520 108.486 108.394  1.00  0.00           H
ATOM   1406  HB2 MET B 677     113.678 106.565 106.789  1.00  0.00           H
ATOM   1407  HB3 MET B 677     115.323 106.067 106.703  1.00  0.00           H
ATOM   1408  HG2 MET B 677     115.366 105.647 109.131  1.00  0.00           H
```

```
ATOM   1409  HG3 MET B 677     114.073 104.770 108.364  1.00  0.00           H
ATOM   1410  HE1 MET B 677     110.939 107.066 109.329  1.00  0.00           H
ATOM   1411  HE2 MET B 677     111.972 107.362 107.946  1.00  0.00           H
ATOM   1412  HE3 MET B 677     111.469 105.707 108.326  1.00  0.00           H
ATOM   1413  N   LEU B 678     117.429 108.166 106.879  1.00  0.00           N
ATOM   1414  CA  LEU B 678     118.876 108.307 107.004  1.00  0.00           C
ATOM   1415  C   LEU B 678     119.265 109.655 107.632  1.00  0.00           C
ATOM   1416  O   LEU B 678     120.141 109.667 108.489  1.00  0.00           O
ATOM   1417  CB  LEU B 678     119.538 108.085 105.624  1.00 20.00           C
ATOM   1418  CG  LEU B 678     121.085 108.084 105.626  1.00 20.00           C
ATOM   1419  CD1 LEU B 678     121.661 106.964 106.518  1.00 20.00           C
ATOM   1420  CD2 LEU B 678     121.645 108.037 104.189  1.00 20.00           C
ATOM   1421  H   LEU B 678     117.066 108.148 105.936  1.00  0.00           H
ATOM   1422  HA  LEU B 678     119.212 107.519 107.681  1.00  0.00           H
ATOM   1423  HB2 LEU B 678     119.190 107.138 105.210  1.00  0.00           H
ATOM   1424  HB3 LEU B 678     119.183 108.855 104.938  1.00  0.00           H
ATOM   1425  HG  LEU B 678     121.426 109.033 106.041  1.00  0.00           H
ATOM   1426 HD11 LEU B 678     122.549 106.499 106.091  1.00  0.00           H
ATOM   1427 HD12 LEU B 678     121.946 107.362 107.492  1.00  0.00           H
ATOM   1428 HD13 LEU B 678     120.936 106.169 106.694  1.00  0.00           H
ATOM   1429 HD21 LEU B 678     122.275 108.905 103.992  1.00  0.00           H
ATOM   1430 HD22 LEU B 678     122.251 107.151 104.002  1.00  0.00           H
ATOM   1431 HD23 LEU B 678     120.852 108.040 103.440  1.00  0.00           H
ATOM   1432  N   ILE B 679     118.576 110.743 107.240  1.00  0.00           N
ATOM   1433  CA  ILE B 679     118.743 112.083 107.812  1.00  0.00           C
ATOM   1434  C   ILE B 679     118.275 112.149 109.283  1.00  0.00           C
ATOM   1435  O   ILE B 679     118.959 112.763 110.101  1.00  0.00           O
ATOM   1436  CB  ILE B 679     117.984 113.160 106.975  1.00 20.00           C
ATOM   1437  CG1 ILE B 679     118.554 113.257 105.540  1.00 20.00           C
ATOM   1438  CG2 ILE B 679     117.955 114.570 107.612  1.00 20.00           C
ATOM   1439  CD1 ILE B 679     117.597 113.921 104.537  1.00 20.00           C
ATOM   1440  H   ILE B 679     117.868 110.649 106.525  1.00  0.00           H
ATOM   1441  HA  ILE B 679     119.809 112.322 107.792  1.00  0.00           H
ATOM   1442  HB  ILE B 679     116.949 112.827 106.889  1.00  0.00           H
ATOM   1443 HG12 ILE B 679     119.497 113.806 105.560  1.00  0.00           H
ATOM   1444 HG13 ILE B 679     118.808 112.270 105.156  1.00  0.00           H
ATOM   1445 HG21 ILE B 679     117.488 115.296 106.947  1.00  0.00           H
ATOM   1446 HG22 ILE B 679     117.388 114.596 108.542  1.00  0.00           H
ATOM   1447 HG23 ILE B 679     118.963 114.925 107.825  1.00  0.00           H
ATOM   1448 HD11 ILE B 679     117.765 113.536 103.530  1.00  0.00           H
ATOM   1449 HD12 ILE B 679     116.553 113.732 104.786  1.00  0.00           H
ATOM   1450 HD13 ILE B 679     117.744 115.000 104.506  1.00  0.00           H
ATOM   1451  N   ALA B 680     117.145 111.485 109.588  1.00  0.00           N
ATOM   1452  CA  ALA B 680     116.564 111.378 110.928  1.00  0.00           C
ATOM   1453  C   ALA B 680     117.451 110.605 111.921  1.00  0.00           C
ATOM   1454  O   ALA B 680     117.622 111.063 113.050  1.00  0.00           O
ATOM   1455  CB  ALA B 680     115.171 110.738 110.818  1.00 20.00           C
ATOM   1456  H   ALA B 680     116.638 111.013 108.852  1.00  0.00           H
ATOM   1457  HA  ALA B 680     116.438 112.391 111.317  1.00  0.00           H
ATOM   1458  HB1 ALA B 680     114.710 110.610 111.797  1.00  0.00           H
ATOM   1459  HB2 ALA B 680     114.500 111.357 110.220  1.00  0.00           H
ATOM   1460  HB3 ALA B 680     115.214 109.754 110.353  1.00  0.00           H
ATOM   1461  N   LEU B 681     118.011 109.466 111.475  1.00  0.00           N
ATOM   1462  CA  LEU B 681     118.867 108.595 112.283  1.00  0.00           C
ATOM   1463  C   LEU B 681     120.331 109.061 112.351  1.00  0.00           C
ATOM   1464  O   LEU B 681     120.983 108.774 113.354  1.00  0.00           O
ATOM   1465  CB  LEU B 681     118.748 107.138 111.785  1.00 20.00           C
ATOM   1466  CG  LEU B 681     117.422 106.458 112.200  1.00 20.00           C
ATOM   1467  CD1 LEU B 681     117.122 105.203 111.365  1.00 20.00           C
ATOM   1468  CD2 LEU B 681     117.368 106.155 113.711  1.00 20.00           C
ATOM   1469  H   LEU B 681     117.807 109.145 110.537  1.00  0.00           H
ATOM   1470  HA  LEU B 681     118.503 108.640 113.308  1.00  0.00           H
ATOM   1471  HB2 LEU B 681     118.863 107.130 110.700  1.00  0.00           H
ATOM   1472  HB3 LEU B 681     119.573 106.536 112.169  1.00  0.00           H
ATOM   1473  HG  LEU B 681     116.622 107.164 111.977  1.00  0.00           H
ATOM   1474 HD11 LEU B 681     116.047 105.033 111.306  1.00  0.00           H
ATOM   1475 HD12 LEU B 681     117.491 105.301 110.345  1.00  0.00           H
ATOM   1476 HD13 LEU B 681     117.567 104.308 111.798  1.00  0.00           H
ATOM   1477 HD21 LEU B 681     116.377 106.358 114.115  1.00  0.00           H
ATOM   1478 HD22 LEU B 681     117.602 105.114 113.925  1.00  0.00           H
ATOM   1479 HD23 LEU B 681     118.079 106.743 114.286  1.00  0.00           H
```

```
ATOM   1480  N   MET B 682     120.811 109.823 111.351  1.00  0.00           N
ATOM   1481  CA  MET B 682     122.071 110.568 111.446  1.00  0.00           C
ATOM   1482  C   MET B 682     121.984 111.684 112.498  1.00  0.00           C
ATOM   1483  O   MET B 682     122.941 111.851 113.246  1.00  0.00           O
ATOM   1484  CB  MET B 682     122.489 111.147 110.078  1.00 20.00           C
ATOM   1485  CG  MET B 682     123.210 110.127 109.186  1.00 20.00           C
ATOM   1486  SD  MET B 682     123.536 110.718 107.504  1.00 20.00           S
ATOM   1487  CE  MET B 682     124.605 109.375 106.923  1.00 20.00           C
ATOM   1488  H   MET B 682     120.248 109.995 110.528  1.00  0.00           H
ATOM   1489  HA  MET B 682     122.853 109.880 111.774  1.00  0.00           H
ATOM   1490  HB2 MET B 682     121.626 111.569 109.561  1.00  0.00           H
ATOM   1491  HB3 MET B 682     123.174 111.983 110.226  1.00  0.00           H
ATOM   1492  HG2 MET B 682     124.166 109.870 109.643  1.00  0.00           H
ATOM   1493  HG3 MET B 682     122.640 109.201 109.123  1.00  0.00           H
ATOM   1494  HE1 MET B 682     124.880 109.534 105.881  1.00  0.00           H
ATOM   1495  HE2 MET B 682     125.517 109.329 107.519  1.00  0.00           H
ATOM   1496  HE3 MET B 682     124.097 108.415 107.004  1.00  0.00           H
ATOM   1497  N   GLY B 683     120.837 112.384 112.570  1.00  0.00           N
ATOM   1498  CA  GLY B 683     120.581 113.456 113.533  1.00  0.00           C
ATOM   1499  C   GLY B 683     120.491 112.935 114.977  1.00  0.00           C
ATOM   1500  O   GLY B 683     120.967 113.611 115.887  1.00  0.00           O
ATOM   1501  H   GLY B 683     120.096 112.179 111.913  1.00  0.00           H
ATOM   1502  HA2 GLY B 683     121.361 114.215 113.456  1.00  0.00           H
ATOM   1503  HA3 GLY B 683     119.639 113.939 113.275  1.00  0.00           H
ATOM   1504  N   GLU B 684     119.923 111.732 115.179  1.00  0.00           N
ATOM   1505  CA  GLU B 684     119.814 111.065 116.479  1.00  0.00           C
ATOM   1506  C   GLU B 684     121.173 110.581 117.018  1.00  0.00           C
ATOM   1507  O   GLU B 684     121.482 110.842 118.182  1.00  0.00           O
ATOM   1508  CB  GLU B 684     118.706 109.986 116.396  1.00 20.00           C
ATOM   1509  CG  GLU B 684     118.536 109.022 117.597  1.00  0.00           C
ATOM   1510  CD  GLU B 684     119.460 107.799 117.569  1.00  0.00           C
ATOM   1511  OE1 GLU B 684     119.451 107.096 116.534  1.00  0.00           O
ATOM   1512  OE2 GLU B 684     120.155 107.583 118.585  1.00  0.00           O1-
ATOM   1513  H   GLU B 684     119.538 111.231 114.390  1.00  0.00           H
ATOM   1514  HA  GLU B 684     119.460 111.811 117.194  1.00  0.00           H
ATOM   1515  HB2 GLU B 684     117.763 110.515 116.266  1.00  0.00           H
ATOM   1516  HB3 GLU B 684     118.828 109.409 115.479  1.00  0.00           H
ATOM   1517  HG2 GLU B 684     118.659 109.564 118.535  1.00  0.00           H
ATOM   1518  HG3 GLU B 684     117.517 108.637 117.604  1.00  0.00           H
ATOM   1519  N   THR B 685     121.971 109.926 116.157  1.00  0.00           N
ATOM   1520  CA  THR B 685     123.313 109.445 116.496  1.00  0.00           C
ATOM   1521  C   THR B 685     124.377 110.568 116.547  1.00  0.00           C
ATOM   1522  O   THR B 685     125.403 110.353 117.189  1.00  0.00           O
ATOM   1523  CB  THR B 685     123.803 108.342 115.521  1.00 20.00           C
ATOM   1524  CG2 THR B 685     122.907 107.093 115.525  1.00 20.00           C
ATOM   1525  OG1 THR B 685     123.936 108.818 114.195  1.00 20.00           O
ATOM   1526  H   THR B 685     121.648 109.737 115.218  1.00  0.00           H
ATOM   1527  HA  THR B 685     123.267 109.003 117.493  1.00  0.00           H
ATOM   1528  HB  THR B 685     124.799 108.021 115.829  1.00  0.00           H
ATOM   1529  HG1 THR B 685     123.071 108.802 113.774  1.00  0.00           H
ATOM   1530 HG21 THR B 685     123.275 106.342 114.825  1.00  0.00           H
ATOM   1531 HG22 THR B 685     122.883 106.637 116.515  1.00  0.00           H
ATOM   1532 HG23 THR B 685     121.878 107.321 115.253  1.00  0.00           H
ATOM   1533  N   VAL B 686     124.112 111.747 115.947  1.00  0.00           N
ATOM   1534  CA  VAL B 686     124.929 112.964 116.078  1.00  0.00           C
ATOM   1535  C   VAL B 686     125.006 113.474 117.531  1.00  0.00           C
ATOM   1536  O   VAL B 686     126.081 113.897 117.954  1.00  0.00           O
ATOM   1537  CB  VAL B 686     124.438 114.109 115.123  1.00 20.00           C
ATOM   1538  CG1 VAL B 686     124.463 115.564 115.652  1.00 20.00           C
ATOM   1539  CG2 VAL B 686     125.190 114.066 113.782  1.00 20.00           C
ATOM   1540  H   VAL B 686     123.266 111.841 115.402  1.00  0.00           H
ATOM   1541  HA  VAL B 686     125.946 112.686 115.792  1.00  0.00           H
ATOM   1542  HB  VAL B 686     123.394 113.910 114.889  1.00  0.00           H
ATOM   1543 HG11 VAL B 686     124.138 116.263 114.881  1.00  0.00           H
ATOM   1544 HG12 VAL B 686     123.785 115.703 116.495  1.00  0.00           H
ATOM   1545 HG13 VAL B 686     125.464 115.864 115.961  1.00  0.00           H
ATOM   1546 HG21 VAL B 686     124.793 114.799 113.080  1.00  0.00           H
ATOM   1547 HG22 VAL B 686     126.251 114.274 113.916  1.00  0.00           H
ATOM   1548 HG23 VAL B 686     125.102 113.092 113.310  1.00  0.00           H
ATOM   1549  N   ASN B 687     123.880 113.400 118.263  1.00  0.00           N
ATOM   1550  CA  ASN B 687     123.779 113.814 119.667  1.00  0.00           C
```

```
ATOM   1551  C   ASN B 687     124.539 112.872 120.619  1.00  0.00           C
ATOM   1552  O   ASN B 687     125.044 113.341 121.638  1.00  0.00           O
ATOM   1553  CB  ASN B 687     122.296 113.923 120.096  1.00 20.00           C
ATOM   1554  CG  ASN B 687     121.453 114.882 119.247  1.00 20.00           C
ATOM   1555  ND2 ASN B 687     121.903 116.129 119.085  1.00 20.00           N
ATOM   1556  OD1 ASN B 687     120.394 114.503 118.754  1.00 20.00           O
ATOM   1557  H   ASN B 687     123.036 113.035 117.842  1.00  0.00           H
ATOM   1558  HA  ASN B 687     124.243 114.798 119.764  1.00  0.00           H
ATOM   1559  HB2 ASN B 687     121.830 112.937 120.066  1.00  0.00           H
ATOM   1560  HB3 ASN B 687     122.233 114.257 121.133  1.00  0.00           H
ATOM   1561 HD21 ASN B 687     121.369 116.785 118.534  1.00  0.00           H
ATOM   1562 HD22 ASN B 687     122.772 116.424 119.505  1.00  0.00           H
ATOM   1563  N   LYS B 688     124.625 111.579 120.259  1.00  0.00           N
ATOM   1564  CA  LYS B 688     125.386 110.561 120.982  1.00  0.00           C
ATOM   1565  C   LYS B 688     126.902 110.648 120.708  1.00  0.00           C
ATOM   1566  O   LYS B 688     127.688 110.404 121.622  1.00  0.00           O
ATOM   1567  CB  LYS B 688     124.805 109.175 120.625  1.00 20.00           C
ATOM   1568  CG  LYS B 688     125.485 107.988 121.332  1.00 20.00           C
ATOM   1569  CD  LYS B 688     124.785 106.653 121.048  1.00 20.00           C
ATOM   1570  CE  LYS B 688     125.467 105.478 121.761  1.00 20.00           C
ATOM   1571  NZ  LYS B 688     124.759 104.214 121.504  1.00 20.00           N1+
ATOM   1572  H   LYS B 688     124.172 111.276 119.408  1.00  0.00           H
ATOM   1573  HA  LYS B 688     125.235 110.719 122.052  1.00  0.00           H
ATOM   1574  HB2 LYS B 688     123.743 109.170 120.878  1.00  0.00           H
ATOM   1575  HB3 LYS B 688     124.856 109.020 119.546  1.00  0.00           H
ATOM   1576  HG2 LYS B 688     126.525 107.910 121.014  1.00  0.00           H
ATOM   1577  HG3 LYS B 688     125.503 108.170 122.407  1.00  0.00           H
ATOM   1578  HD2 LYS B 688     123.741 106.718 121.361  1.00  0.00           H
ATOM   1579  HD3 LYS B 688     124.771 106.474 119.972  1.00  0.00           H
ATOM   1580  HE2 LYS B 688     126.500 105.379 121.426  1.00  0.00           H
ATOM   1581  HE3 LYS B 688     125.491 105.651 122.837  1.00  0.00           H
ATOM   1582  HZ1 LYS B 688     124.750 104.025 120.512  1.00  0.00           H
ATOM   1583  HZ2 LYS B 688     125.226 103.459 121.987  1.00  0.00           H
ATOM   1584  HZ3 LYS B 688     123.808 104.287 121.839  1.00  0.00           H
ATOM   1585  N   ILE B 689     127.277 110.990 119.462  1.00  0.00           N
ATOM   1586  CA  ILE B 689     128.657 111.057 118.958  1.00  0.00           C
ATOM   1587  C   ILE B 689     129.076 112.506 119.142  1.00  0.00           C
ATOM   1588  O   ILE B 689     130.257 112.850 119.191  1.00  0.00           O
ATOM   1589  CB  ILE B 689     128.738 110.541 117.481  1.00 20.00           C
ATOM   1590  CG1 ILE B 689     128.417 109.026 117.401  1.00 20.00           C
ATOM   1591  CG2 ILE B 689     130.074 110.840 116.760  1.00 20.00           C
ATOM   1592  CD1 ILE B 689     128.065 108.518 115.994  1.00 20.00           C
ATOM   1593  H   ILE B 689     126.558 111.159 118.771  1.00  0.00           H
ATOM   1594  HA  ILE B 689     129.312 110.431 119.569  1.00  0.00           H
ATOM   1595  HB  ILE B 689     127.969 111.067 116.912  1.00  0.00           H
ATOM   1596 HG12 ILE B 689     129.261 108.455 117.790  1.00  0.00           H
ATOM   1597 HG13 ILE B 689     127.578 108.784 118.054  1.00  0.00           H
ATOM   1598 HG21 ILE B 689     130.074 110.456 115.740  1.00  0.00           H
ATOM   1599 HG22 ILE B 689     130.265 111.910 116.683  1.00  0.00           H
ATOM   1600 HG23 ILE B 689     130.915 110.387 117.285  1.00  0.00           H
ATOM   1601 HD11 ILE B 689     127.620 107.525 116.044  1.00  0.00           H
ATOM   1602 HD12 ILE B 689     127.342 109.171 115.506  1.00  0.00           H
ATOM   1603 HD13 ILE B 689     128.946 108.448 115.356  1.00  0.00           H
ATOM   1604  HXT ILE B 689     128.272 113.239 119.222  1.00  0.00           H
TER    1605      ILE B 689
ATOM   1606  N   THR C 335      97.045  79.138 134.838  1.00 36.89           N1+
ATOM   1607  CA  THR C 335      97.667  79.340 136.195  1.00 36.74           C
ATOM   1608  C   THR C 335      97.713  80.877 136.459  1.00 36.68           C
ATOM   1609  O   THR C 335      97.592  81.665 135.519  1.00 36.82           O
ATOM   1610  CB  THR C 335      99.108  78.746 136.295  1.00 36.74           C
ATOM   1611  CG2 THR C 335      99.147  77.225 136.083  1.00 36.34           C
ATOM   1612  OG1 THR C 335      99.998  79.334 135.360  1.00 37.07           O
ATOM   1613  HA  THR C 335      96.975  78.839 136.870  1.00  0.00           H
ATOM   1614  HB  THR C 335      99.522  78.939 137.285  1.00  0.00           H
ATOM   1615  HG1 THR C 335     100.802  78.808 135.330  1.00  0.00           H
ATOM   1616 HG21 THR C 335     100.158  76.835 136.209  1.00  0.00           H
ATOM   1617 HG22 THR C 335      98.510  76.713 136.805  1.00  0.00           H
ATOM   1618 HG23 THR C 335      98.810  76.945 135.085  1.00  0.00           H
ATOM   1619  H1  THR C 335      96.981  78.148 134.648  1.00  0.00           H
ATOM   1620  H2  THR C 335      96.123  79.551 134.826  1.00  0.00           H
ATOM   1621  H   THR C 335      97.636  79.577 134.146  1.00  0.00           H
```

```
ATOM   1622  N   PRO C 336      97.917  81.293 137.735  1.00 36.46           N
ATOM   1623  CA  PRO C 336      98.170  82.708 138.088  1.00 35.71           C
ATOM   1624  C   PRO C 336      99.431  83.333 137.457  1.00 35.26           C
ATOM   1625  O   PRO C 336      99.430  84.536 137.199  1.00 35.27           O
ATOM   1626  CB  PRO C 336      98.250  82.706 139.625  1.00 36.20           C
ATOM   1627  CG  PRO C 336      97.483  81.466 140.046  1.00 36.12           C
ATOM   1628  CD  PRO C 336      97.815  80.472 138.942  1.00 36.23           C
ATOM   1629  HA  PRO C 336      97.293  83.281 137.783  1.00  0.00           H
ATOM   1630  HB2 PRO C 336      99.285  82.617 139.960  1.00  0.00           H
ATOM   1631  HB3 PRO C 336      97.840  83.614 140.068  1.00  0.00           H
ATOM   1632  HG2 PRO C 336      97.744  81.110 141.044  1.00  0.00           H
ATOM   1633  HG3 PRO C 336      96.413  81.679 140.034  1.00  0.00           H
ATOM   1634  HD2 PRO C 336      97.053  79.695 138.891  1.00  0.00           H
ATOM   1635  HD3 PRO C 336      98.779  80.002 139.142  1.00  0.00           H
ATOM   1636  N   LEU C 337     100.463  82.508 137.199  1.00 35.26           N
ATOM   1637  CA  LEU C 337     101.693  82.890 136.502  1.00 34.53           C
ATOM   1638  C   LEU C 337     101.451  83.182 135.011  1.00 34.37           C
ATOM   1639  O   LEU C 337     101.933  84.200 134.515  1.00 34.02           O
ATOM   1640  CB  LEU C 337     102.758  81.789 136.734  1.00 34.61           C
ATOM   1641  CG  LEU C 337     104.091  81.918 135.953  1.00 34.07           C
ATOM   1642  CD1 LEU C 337     104.796  83.280 136.159  1.00 34.18           C
ATOM   1643  CD2 LEU C 337     105.016  80.717 136.252  1.00 32.60           C
ATOM   1644  H   LEU C 337     100.388  81.535 137.454  1.00  0.00           H
ATOM   1645  HA  LEU C 337     102.047  83.816 136.959  1.00  0.00           H
ATOM   1646  HB2 LEU C 337     102.969  81.736 137.802  1.00  0.00           H
ATOM   1647  HB3 LEU C 337     102.316  80.825 136.478  1.00  0.00           H
ATOM   1648  HG  LEU C 337     103.852  81.850 134.893  1.00  0.00           H
ATOM   1649 HD11 LEU C 337     104.799  83.855 135.232  1.00  0.00           H
ATOM   1650 HD12 LEU C 337     104.304  83.893 136.912  1.00  0.00           H
ATOM   1651 HD13 LEU C 337     105.833  83.182 136.472  1.00  0.00           H
ATOM   1652 HD21 LEU C 337     105.992  81.016 136.632  1.00  0.00           H
ATOM   1653 HD22 LEU C 337     104.582  80.042 136.990  1.00  0.00           H
ATOM   1654 HD23 LEU C 337     105.192  80.128 135.352  1.00  0.00           H
ATOM   1655  N   ALA C 338     100.679  82.307 134.341  1.00 33.95           N
ATOM   1656  CA  ALA C 338     100.247  82.480 132.953  1.00 34.02           C
ATOM   1657  C   ALA C 338      99.228  83.620 132.776  1.00 34.07           C
ATOM   1658  O   ALA C 338      99.200  84.229 131.708  1.00 34.62           O
ATOM   1659  CB  ALA C 338      99.678  81.153 132.431  1.00 34.42           C
ATOM   1660  H   ALA C 338     100.329  81.487 134.816  1.00  0.00           H
ATOM   1661  HA  ALA C 338     101.128  82.722 132.356  1.00  0.00           H
ATOM   1662  HB1 ALA C 338      99.381  81.236 131.384  1.00  0.00           H
ATOM   1663  HB2 ALA C 338     100.420  80.357 132.494  1.00  0.00           H
ATOM   1664  HB3 ALA C 338      98.803  80.841 133.001  1.00  0.00           H
ATOM   1665  N   LEU C 339      98.434  83.909 133.823  1.00 33.85           N
ATOM   1666  CA  LEU C 339      97.490  85.024 133.872  1.00 33.53           C
ATOM   1667  C   LEU C 339      98.217  86.372 133.999  1.00 33.66           C
ATOM   1668  O   LEU C 339      97.858  87.299 133.278  1.00 34.18           O
ATOM   1669  CB  LEU C 339      96.442  84.770 134.979  1.00 33.69           C
ATOM   1670  CG  LEU C 339      95.251  85.765 135.023  1.00 33.84           C
ATOM   1671  CD1 LEU C 339      93.985  85.106 135.617  1.00 31.31           C
ATOM   1672  CD2 LEU C 339      95.590  87.093 135.738  1.00 33.49           C
ATOM   1673  H   LEU C 339      98.495  83.343 134.658  1.00  0.00           H
ATOM   1674  HA  LEU C 339      96.951  85.028 132.924  1.00  0.00           H
ATOM   1675  HB2 LEU C 339      96.045  83.773 134.789  1.00  0.00           H
ATOM   1676  HB3 LEU C 339      96.917  84.712 135.958  1.00  0.00           H
ATOM   1677  HG  LEU C 339      95.002  86.007 133.988  1.00  0.00           H
ATOM   1678 HD11 LEU C 339      93.125  85.271 134.967  1.00  0.00           H
ATOM   1679 HD12 LEU C 339      94.088  84.028 135.738  1.00  0.00           H
ATOM   1680 HD13 LEU C 339      93.726  85.501 136.599  1.00  0.00           H
ATOM   1681 HD21 LEU C 339      94.835  87.388 136.466  1.00  0.00           H
ATOM   1682 HD22 LEU C 339      96.539  87.040 136.273  1.00  0.00           H
ATOM   1683 HD23 LEU C 339      95.667  87.909 135.019  1.00  0.00           H
ATOM   1684  N   ALA C 340      99.253  86.443 134.857  1.00 33.60           N
ATOM   1685  CA  ALA C 340     100.150  87.596 134.986  1.00 33.36           C
ATOM   1686  C   ALA C 340     100.957  87.879 133.705  1.00 33.23           C
ATOM   1687  O   ALA C 340     101.195  89.045 133.394  1.00 33.09           O
ATOM   1688  CB  ALA C 340     101.089  87.381 136.184  1.00 33.41           C
ATOM   1689  H   ALA C 340      99.476  85.642 135.433  1.00  0.00           H
ATOM   1690  HA  ALA C 340      99.537  88.474 135.196  1.00  0.00           H
ATOM   1691  HB1 ALA C 340     101.754  88.234 136.324  1.00  0.00           H
ATOM   1692  HB2 ALA C 340     100.520  87.255 137.105  1.00  0.00           H
```

ATOM	1693	HB3	ALA	C	340	101.711	86.494	136.057	1.00	0.00	H
ATOM	1694	N	ALA	C	341	101.318	86.809	132.975	1.00	33.42	N
ATOM	1695	CA	ALA	C	341	101.999	86.858	131.684	1.00	33.00	C
ATOM	1696	C	ALA	C	341	101.091	87.296	130.520	1.00	33.03	C
ATOM	1697	O	ALA	C	341	101.591	87.934	129.598	1.00	33.45	O
ATOM	1698	CB	ALA	C	341	102.626	85.485	131.402	1.00	32.81	C
ATOM	1699	H	ALA	C	341	101.104	85.884	133.324	1.00	0.00	H
ATOM	1700	HA	ALA	C	341	102.806	87.584	131.760	1.00	0.00	H
ATOM	1701	HB1	ALA	C	341	103.213	85.500	130.486	1.00	0.00	H
ATOM	1702	HB2	ALA	C	341	103.292	85.181	132.209	1.00	0.00	H
ATOM	1703	HB3	ALA	C	341	101.868	84.711	131.291	1.00	0.00	H
ATOM	1704	N	SER	C	342	99.792	86.952	130.566	1.00	32.96	N
ATOM	1705	CA	SER	C	342	98.812	87.316	129.536	1.00	32.56	C
ATOM	1706	C	SER	C	342	98.229	88.729	129.729	1.00	32.96	C
ATOM	1707	O	SER	C	342	97.857	89.354	128.738	1.00	33.30	O
ATOM	1708	CB	SER	C	342	97.715	86.231	129.447	1.00	32.35	C
ATOM	1709	OG	SER	C	342	96.806	86.267	130.529	1.00	30.21	O
ATOM	1710	H	SER	C	342	99.451	86.395	131.337	1.00	0.00	H
ATOM	1711	HA	SER	C	342	99.324	87.314	128.571	1.00	0.00	H
ATOM	1712	HB2	SER	C	342	98.153	85.235	129.386	1.00	0.00	H
ATOM	1713	HB3	SER	C	342	97.141	86.367	128.530	1.00	0.00	H
ATOM	1714	HG	SER	C	342	97.268	85.991	131.325	1.00	0.00	H
ATOM	1715	N	SER	C	343	98.170	89.205	130.986	1.00	32.75	N
ATOM	1716	CA	SER	C	343	97.669	90.529	131.366	1.00	32.98	C
ATOM	1717	C	SER	C	343	98.763	91.617	131.405	1.00	33.17	C
ATOM	1718	O	SER	C	343	98.417	92.789	131.553	1.00	32.85	O
ATOM	1719	CB	SER	C	343	96.896	90.412	132.697	1.00	32.58	C
ATOM	1720	OG	SER	C	343	97.738	90.101	133.789	1.00	32.92	O
ATOM	1721	H	SER	C	343	98.473	88.613	131.747	1.00	0.00	H
ATOM	1722	HA	SER	C	343	96.946	90.850	130.614	1.00	0.00	H
ATOM	1723	HB2	SER	C	343	96.121	89.648	132.621	1.00	0.00	H
ATOM	1724	HB3	SER	C	343	96.386	91.350	132.919	1.00	0.00	H
ATOM	1725	HG	SER	C	343	97.916	89.155	133.766	1.00	0.00	H
ATOM	1726	N	GLY	C	344	100.042	91.232	131.245	1.00	33.56	N
ATOM	1727	CA	GLY	C	344	101.168	92.157	131.121	1.00	34.45	C
ATOM	1728	C	GLY	C	344	101.663	92.726	132.460	1.00	35.18	C
ATOM	1729	O	GLY	C	344	102.356	93.743	132.449	1.00	35.31	O
ATOM	1730	H	GLY	C	344	100.248	90.249	131.141	1.00	0.00	H
ATOM	1731	HA2	GLY	C	344	100.927	92.968	130.436	1.00	0.00	H
ATOM	1732	HA3	GLY	C	344	101.990	91.615	130.670	1.00	0.00	H
ATOM	1733	N	LYS	C	345	101.315	92.094	133.595	1.00	35.75	N
ATOM	1734	CA	LYS	C	345	101.703	92.494	134.950	1.00	36.22	C
ATOM	1735	C	LYS	C	345	103.168	92.115	135.241	1.00	36.63	C
ATOM	1736	O	LYS	C	345	103.438	91.036	135.770	1.00	36.92	O
ATOM	1737	CB	LYS	C	345	100.712	91.870	135.960	1.00	36.19	C
ATOM	1738	CG	LYS	C	345	99.250	92.344	135.800	1.00	36.14	C
ATOM	1739	CD	LYS	C	345	98.982	93.830	136.115	1.00	36.58	C
ATOM	1740	CE	LYS	C	345	99.157	94.222	137.591	1.00	37.13	C
ATOM	1741	NZ	LYS	C	345	98.170	93.551	138.455	1.00	37.20	N1+
ATOM	1742	H	LYS	C	345	100.763	91.250	133.528	1.00	0.00	H
ATOM	1743	HA	LYS	C	345	101.629	93.580	135.025	1.00	0.00	H
ATOM	1744	HB2	LYS	C	345	100.734	90.784	135.858	1.00	0.00	H
ATOM	1745	HB3	LYS	C	345	101.046	92.075	136.977	1.00	0.00	H
ATOM	1746	HG2	LYS	C	345	98.932	92.165	134.775	1.00	0.00	H
ATOM	1747	HG3	LYS	C	345	98.604	91.715	136.412	1.00	0.00	H
ATOM	1748	HD2	LYS	C	345	99.622	94.461	135.499	1.00	0.00	H
ATOM	1749	HD3	LYS	C	345	97.966	94.072	135.801	1.00	0.00	H
ATOM	1750	HE2	LYS	C	345	100.163	93.991	137.942	1.00	0.00	H
ATOM	1751	HE3	LYS	C	345	99.027	95.300	137.700	1.00	0.00	H
ATOM	1752	HZ1	LYS	C	345	98.288	92.550	138.389	1.00	0.00	H
ATOM	1753	HZ2	LYS	C	345	98.302	93.844	139.413	1.00	0.00	H
ATOM	1754	HZ3	LYS	C	345	97.237	93.799	138.156	1.00	0.00	H
ATOM	1755	N	ILE	C	346	104.080	93.024	134.857	1.00	36.53	N
ATOM	1756	CA	ILE	C	346	105.538	92.886	134.914	1.00	36.02	C
ATOM	1757	C	ILE	C	346	106.140	92.659	136.317	1.00	36.09	C
ATOM	1758	O	ILE	C	346	107.079	91.871	136.430	1.00	36.19	O
ATOM	1759	CB	ILE	C	346	106.256	94.111	134.267	1.00	35.75	C
ATOM	1760	CG1	ILE	C	346	105.707	95.483	134.739	1.00	35.02	C
ATOM	1761	CG2	ILE	C	346	106.248	94.004	132.733	1.00	35.66	C
ATOM	1762	CD1	ILE	C	346	106.559	96.683	134.299	1.00	35.16	C
ATOM	1763	H	ILE	C	346	103.751	93.862	134.399	1.00	0.00	H

```
ATOM   1764  HA  ILE C 346     105.780  91.997 134.328  1.00  0.00           H
ATOM   1765  HB  ILE C 346     107.308  94.081 134.558  1.00  0.00           H
ATOM   1766 HG12 ILE C 346     104.693  95.622 134.359  1.00  0.00           H
ATOM   1767 HG13 ILE C 346     105.625  95.509 135.824  1.00  0.00           H
ATOM   1768 HG21 ILE C 346     106.735  94.857 132.262  1.00  0.00           H
ATOM   1769 HG22 ILE C 346     106.797  93.121 132.411  1.00  0.00           H
ATOM   1770 HG23 ILE C 346     105.234  93.935 132.339  1.00  0.00           H
ATOM   1771 HD11 ILE C 346     106.457  97.511 135.001  1.00  0.00           H
ATOM   1772 HD12 ILE C 346     107.618  96.429 134.241  1.00  0.00           H
ATOM   1773 HD13 ILE C 346     106.250  97.045 133.317  1.00  0.00           H
ATOM   1774  N   GLY C 347     105.592  93.321 137.352  1.00 35.51           N
ATOM   1775  CA  GLY C 347     106.097  93.238 138.725  1.00 35.19           C
ATOM   1776  C   GLY C 347     105.624  91.957 139.429  1.00 34.92           C
ATOM   1777  O   GLY C 347     106.344  91.434 140.280  1.00 34.78           O
ATOM   1778  H   GLY C 347     104.818  93.948 137.181  1.00  0.00           H
ATOM   1779  HA2 GLY C 347     107.187  93.290 138.735  1.00  0.00           H
ATOM   1780  HA3 GLY C 347     105.734  94.100 139.285  1.00  0.00           H
ATOM   1781  N   VAL C 348     104.440  91.441 139.056  1.00 34.20           N
ATOM   1782  CA  VAL C 348     103.880  90.181 139.551  1.00 33.89           C
ATOM   1783  C   VAL C 348     104.641  88.971 138.974  1.00 34.13           C
ATOM   1784  O   VAL C 348     104.955  88.045 139.721  1.00 34.36           O
ATOM   1785  CB  VAL C 348     102.370  90.055 139.189  1.00 33.36           C
ATOM   1786  CG1 VAL C 348     101.734  88.697 139.558  1.00 33.30           C
ATOM   1787  CG2 VAL C 348     101.548  91.192 139.823  1.00 34.16           C
ATOM   1788  H   VAL C 348     103.904  91.910 138.341  1.00  0.00           H
ATOM   1789  HA  VAL C 348     103.981  90.161 140.638  1.00  0.00           H
ATOM   1790  HB  VAL C 348     102.270  90.162 138.108  1.00  0.00           H
ATOM   1791 HG11 VAL C 348     100.660  88.702 139.370  1.00  0.00           H
ATOM   1792 HG12 VAL C 348     102.146  87.879 138.969  1.00  0.00           H
ATOM   1793 HG13 VAL C 348     101.885  88.462 140.611  1.00  0.00           H
ATOM   1794 HG21 VAL C 348     100.493  91.111 139.559  1.00  0.00           H
ATOM   1795 HG22 VAL C 348     101.620  91.172 140.911  1.00  0.00           H
ATOM   1796 HG23 VAL C 348     101.886  92.171 139.485  1.00  0.00           H
ATOM   1797  N   LEU C 349     104.957  89.031 137.669  1.00 34.10           N
ATOM   1798  CA  LEU C 349     105.756  88.039 136.953  1.00 33.94           C
ATOM   1799  C   LEU C 349     107.220  87.998 137.435  1.00 33.11           C
ATOM   1800  O   LEU C 349     107.768  86.905 137.557  1.00 32.65           O
ATOM   1801  CB  LEU C 349     105.571  88.270 135.434  1.00 34.24           C
ATOM   1802  CG  LEU C 349     106.361  87.315 134.500  1.00 34.70           C
ATOM   1803  CD1 LEU C 349     105.563  86.951 133.229  1.00 35.42           C
ATOM   1804  CD2 LEU C 349     107.757  87.868 134.140  1.00 37.49           C
ATOM   1805  H   LEU C 349     104.652  89.827 137.124  1.00  0.00           H
ATOM   1806  HA  LEU C 349     105.327  87.061 137.181  1.00  0.00           H
ATOM   1807  HB2 LEU C 349     104.502  88.150 135.250  1.00  0.00           H
ATOM   1808  HB3 LEU C 349     105.779  89.309 135.175  1.00  0.00           H
ATOM   1809  HG  LEU C 349     106.498  86.375 135.036  1.00  0.00           H
ATOM   1810 HD11 LEU C 349     105.579  85.874 133.060  1.00  0.00           H
ATOM   1811 HD12 LEU C 349     104.517  87.246 133.293  1.00  0.00           H
ATOM   1812 HD13 LEU C 349     105.964  87.425 132.333  1.00  0.00           H
ATOM   1813 HD21 LEU C 349     108.031  87.681 133.102  1.00  0.00           H
ATOM   1814 HD22 LEU C 349     107.812  88.945 134.293  1.00  0.00           H
ATOM   1815 HD23 LEU C 349     108.530  87.412 134.757  1.00  0.00           H
ATOM   1816  N   ALA C 350     107.805  89.163 137.768  1.00 32.35           N
ATOM   1817  CA  ALA C 350     109.138  89.271 138.368  1.00 31.91           C
ATOM   1818  C   ALA C 350     109.234  88.660 139.779  1.00 31.54           C
ATOM   1819  O   ALA C 350     110.260  88.058 140.091  1.00 31.60           O
ATOM   1820  CB  ALA C 350     109.585  90.739 138.371  1.00 31.68           C
ATOM   1821  H   ALA C 350     107.306  90.032 137.630  1.00  0.00           H
ATOM   1822  HA  ALA C 350     109.830  88.716 137.734  1.00  0.00           H
ATOM   1823  HB1 ALA C 350     110.575  90.851 138.814  1.00  0.00           H
ATOM   1824  HB2 ALA C 350     109.638  91.132 137.356  1.00  0.00           H
ATOM   1825  HB3 ALA C 350     108.894  91.367 138.936  1.00  0.00           H
ATOM   1826  N   TYR C 351     108.164  88.792 140.584  1.00 30.99           N
ATOM   1827  CA  TYR C 351     108.050  88.209 141.924  1.00 30.59           C
ATOM   1828  C   TYR C 351     107.980  86.669 141.911  1.00 31.05           C
ATOM   1829  O   TYR C 351     108.686  86.032 142.693  1.00 31.61           O
ATOM   1830  CB  TYR C 351     106.854  88.853 142.665  1.00 29.56           C
ATOM   1831  CG  TYR C 351     106.474  88.208 143.990  1.00 28.61           C
ATOM   1832  CD1 TYR C 351     107.258  88.441 145.140  1.00 29.08           C
ATOM   1833  CD2 TYR C 351     105.354  87.353 144.070  1.00 26.21           C
ATOM   1834  CE1 TYR C 351     106.929  87.816 146.359  1.00 28.61           C
```

```
ATOM   1835  CE2 TYR C 351     105.027  86.727 145.288  1.00 23.37           C
ATOM   1836  CZ  TYR C 351     105.814  86.957 146.433  1.00 27.49           C
ATOM   1837  OH  TYR C 351     105.496  86.345 147.610  1.00 29.18           O
ATOM   1838  H   TYR C 351     107.362  89.313 140.256  1.00  0.00           H
ATOM   1839  HA  TYR C 351     108.955  88.484 142.470  1.00  0.00           H
ATOM   1840  HB2 TYR C 351     107.056  89.910 142.837  1.00  0.00           H
ATOM   1841  HB3 TYR C 351     105.973  88.829 142.023  1.00  0.00           H
ATOM   1842  HD1 TYR C 351     108.119  89.091 145.087  1.00  0.00           H
ATOM   1843  HD2 TYR C 351     104.749  87.167 143.195  1.00  0.00           H
ATOM   1844  HE1 TYR C 351     107.537  87.996 147.233  1.00  0.00           H
ATOM   1845  HE2 TYR C 351     104.170  86.072 145.344  1.00  0.00           H
ATOM   1846  HH  TYR C 351     106.087  86.577 148.330  1.00  0.00           H
ATOM   1847  N   ILE C 352     107.135  86.105 141.031  1.00 30.80           N
ATOM   1848  CA  ILE C 352     106.905  84.660 140.910  1.00 30.99           C
ATOM   1849  C   ILE C 352     108.143  83.891 140.401  1.00 31.33           C
ATOM   1850  O   ILE C 352     108.374  82.765 140.846  1.00 32.20           O
ATOM   1851  CB  ILE C 352     105.682  84.352 139.987  1.00 31.03           C
ATOM   1852  CG1 ILE C 352     104.367  84.883 140.608  1.00 29.77           C
ATOM   1853  CG2 ILE C 352     105.505  82.859 139.622  1.00 30.83           C
ATOM   1854  CD1 ILE C 352     103.227  85.069 139.594  1.00 28.73           C
ATOM   1855  H   ILE C 352     106.586  86.696 140.420  1.00  0.00           H
ATOM   1856  HA  ILE C 352     106.677  84.282 141.908  1.00  0.00           H
ATOM   1857  HB  ILE C 352     105.841  84.895 139.053  1.00  0.00           H
ATOM   1858 HG12 ILE C 352     104.038  84.206 141.395  1.00  0.00           H
ATOM   1859 HG13 ILE C 352     104.532  85.839 141.102  1.00  0.00           H
ATOM   1860 HG21 ILE C 352     104.590  82.693 139.061  1.00  0.00           H
ATOM   1861 HG22 ILE C 352     106.314  82.485 138.996  1.00  0.00           H
ATOM   1862 HG23 ILE C 352     105.455  82.238 140.517  1.00  0.00           H
ATOM   1863 HD11 ILE C 352     102.440  85.693 140.015  1.00  0.00           H
ATOM   1864 HD12 ILE C 352     103.575  85.552 138.681  1.00  0.00           H
ATOM   1865 HD13 ILE C 352     102.773  84.116 139.323  1.00  0.00           H
ATOM   1866  N   LEU C 353     108.912  84.517 139.495  1.00 30.90           N
ATOM   1867  CA  LEU C 353     110.085  83.921 138.858  1.00 30.99           C
ATOM   1868  C   LEU C 353     111.399  84.120 139.642  1.00 31.13           C
ATOM   1869  O   LEU C 353     112.407  83.558 139.216  1.00 31.12           O
ATOM   1870  CB  LEU C 353     110.197  84.436 137.404  1.00 30.86           C
ATOM   1871  CG  LEU C 353     109.013  84.047 136.486  1.00 29.88           C
ATOM   1872  CD1 LEU C 353     109.264  84.503 135.036  1.00 29.83           C
ATOM   1873  CD2 LEU C 353     108.665  82.552 136.546  1.00 30.44           C
ATOM   1874  H   LEU C 353     108.657  85.443 139.178  1.00  0.00           H
ATOM   1875  HA  LEU C 353     109.941  82.844 138.806  1.00  0.00           H
ATOM   1876  HB2 LEU C 353     110.321  85.521 137.406  1.00  0.00           H
ATOM   1877  HB3 LEU C 353     111.103  84.032 136.954  1.00  0.00           H
ATOM   1878  HG  LEU C 353     108.126  84.571 136.834  1.00  0.00           H
ATOM   1879 HD11 LEU C 353     108.378  84.981 134.619  1.00  0.00           H
ATOM   1880 HD12 LEU C 353     110.083  85.220 134.967  1.00  0.00           H
ATOM   1881 HD13 LEU C 353     109.514  83.667 134.382  1.00  0.00           H
ATOM   1882 HD21 LEU C 353     108.082  82.251 135.677  1.00  0.00           H
ATOM   1883 HD22 LEU C 353     109.561  81.931 136.593  1.00  0.00           H
ATOM   1884 HD23 LEU C 353     108.058  82.325 137.422  1.00  0.00           H
ATOM   1885  N   GLN C 354     111.388  84.873 140.760  1.00 31.81           N
ATOM   1886  CA  GLN C 354     112.600  85.205 141.524  1.00 32.41           C
ATOM   1887  C   GLN C 354     112.455  85.033 143.052  1.00 33.15           C
ATOM   1888  O   GLN C 354     113.385  85.412 143.765  1.00 33.51           O
ATOM   1889  CB  GLN C 354     113.089  86.625 141.149  1.00 32.04           C
ATOM   1890  CG  GLN C 354     113.447  86.797 139.652  1.00 30.84           C
ATOM   1891  CD  GLN C 354     113.957  88.186 139.253  1.00 31.08           C
ATOM   1892  NE2 GLN C 354     113.970  89.157 140.169  1.00 31.66           N
ATOM   1893  OE1 GLN C 354     114.334  88.384 138.101  1.00 32.26           O
ATOM   1894  H   GLN C 354     110.530  85.315 141.060  1.00  0.00           H
ATOM   1895  HA  GLN C 354     113.398  84.512 141.257  1.00  0.00           H
ATOM   1896  HB2 GLN C 354     112.328  87.350 141.439  1.00  0.00           H
ATOM   1897  HB3 GLN C 354     113.974  86.868 141.740  1.00  0.00           H
ATOM   1898  HG2 GLN C 354     114.200  86.061 139.367  1.00  0.00           H
ATOM   1899  HG3 GLN C 354     112.573  86.602 139.031  1.00  0.00           H
ATOM   1900 HE21 GLN C 354     114.297  90.080 139.919  1.00  0.00           H
ATOM   1901 HE22 GLN C 354     113.643  88.981 141.107  1.00  0.00           H
ATOM   1902  N   ARG C 355     111.334  84.466 143.543  1.00 34.49           N
ATOM   1903  CA  ARG C 355     111.086  84.225 144.976  1.00 35.92           C
ATOM   1904  C   ARG C 355     112.108  83.249 145.600  1.00 36.56           C
ATOM   1905  O   ARG C 355     112.457  82.254 144.967  1.00 37.15           O
```

```
ATOM   1906  CB  ARG C 355     109.631  83.741 145.196  1.00 36.12           C
ATOM   1907  CG  ARG C 355     109.251  82.440 144.461  1.00 37.70           C
ATOM   1908  CD  ARG C 355     107.875  81.875 144.832  1.00 41.27           C
ATOM   1909  NE  ARG C 355     106.777  82.726 144.356  1.00 44.15           N
ATOM   1910  CZ  ARG C 355     105.471  82.431 144.474  1.00 43.72           C
ATOM   1911  NH1 ARG C 355     105.065  81.283 145.038  1.00 44.30           N
ATOM   1912  NH2 ARG C 355     104.560  83.300 144.021  1.00 41.81           N1+
ATOM   1913  H   ARG C 355     110.602  84.181 142.909  1.00  0.00           H
ATOM   1914  HA  ARG C 355     111.189  85.189 145.477  1.00  0.00           H
ATOM   1915  HB2 ARG C 355     108.939  84.532 144.907  1.00  0.00           H
ATOM   1916  HB3 ARG C 355     109.477  83.599 146.268  1.00  0.00           H
ATOM   1917  HG2 ARG C 355     109.272  82.642 143.391  1.00  0.00           H
ATOM   1918  HG3 ARG C 355     109.980  81.654 144.636  1.00  0.00           H
ATOM   1919  HD2 ARG C 355     107.784  81.832 145.918  1.00  0.00           H
ATOM   1920  HD3 ARG C 355     107.777  80.853 144.466  1.00  0.00           H
ATOM   1921  HE  ARG C 355     107.045  83.613 143.954  1.00  0.00           H
ATOM   1922 HH11 ARG C 355     104.083  81.070 145.130  1.00  0.00           H
ATOM   1923 HH12 ARG C 355     105.749  80.623 145.382  1.00  0.00           H
ATOM   1924 HH21 ARG C 355     103.574  83.099 144.107  1.00  0.00           H
ATOM   1925 HH22 ARG C 355     104.850  84.176 143.610  1.00  0.00           H
ATOM   1926  N   GLU C 356     112.549  83.559 146.829  1.00 30.00           N
ATOM   1927  CA  GLU C 356     113.541  82.791 147.584  1.00 30.00           C
ATOM   1928  C   GLU C 356     113.012  82.516 149.000  1.00 30.00           C
ATOM   1929  O   GLU C 356     112.529  83.440 149.655  1.00 30.00           O
ATOM   1930  CB  GLU C 356     114.887  83.560 147.583  1.00 20.00           C
ATOM   1931  CG  GLU C 356     116.029  82.997 148.466  1.00  0.00           C
ATOM   1932  CD  GLU C 356     116.518  81.607 148.049  1.00  0.00           C
ATOM   1933  OE1 GLU C 356     115.829  80.623 148.398  1.00  0.00           O
ATOM   1934  OE2 GLU C 356     117.587  81.544 147.404  1.00  0.00           O1-
ATOM   1935  H   GLU C 356     112.199  84.384 147.294  1.00  0.00           H
ATOM   1936  HA  GLU C 356     113.714  81.834 147.096  1.00  0.00           H
ATOM   1937  HB2 GLU C 356     115.248  83.620 146.555  1.00  0.00           H
ATOM   1938  HB3 GLU C 356     114.705  84.593 147.884  1.00  0.00           H
ATOM   1939  HG2 GLU C 356     116.878  83.681 148.423  1.00  0.00           H
ATOM   1940  HG3 GLU C 356     115.731  82.966 149.514  1.00  0.00           H
ATOM   1941  N   ILE C 357     113.150  81.257 149.445  1.00 30.00           N
ATOM   1942  CA  ILE C 357     112.882  80.803 150.807  1.00 30.00           C
ATOM   1943  C   ILE C 357     113.900  79.695 151.149  1.00 30.00           C
ATOM   1944  O   ILE C 357     114.071  78.758 150.366  1.00 30.00           O
ATOM   1945  CB  ILE C 357     111.460  80.181 151.003  1.00 20.00           C
ATOM   1946  CG1 ILE C 357     110.285  80.999 150.415  1.00  0.00           C
ATOM   1947  CG2 ILE C 357     111.189  79.833 152.478  1.00  0.00           C
ATOM   1948  CD1 ILE C 357     109.941  82.283 151.184  1.00  0.00           C
ATOM   1949  H   ILE C 357     113.610  80.583 148.849  1.00  0.00           H
ATOM   1950  HA  ILE C 357     113.020  81.635 151.500  1.00  0.00           H
ATOM   1951  HB  ILE C 357     111.433  79.226 150.478  1.00  0.00           H
ATOM   1952 HG12 ILE C 357     110.465  81.235 149.366  1.00  0.00           H
ATOM   1953 HG13 ILE C 357     109.399  80.365 150.402  1.00  0.00           H
ATOM   1954 HG21 ILE C 357     110.174  79.479 152.628  1.00  0.00           H
ATOM   1955 HG22 ILE C 357     111.853  79.042 152.816  1.00  0.00           H
ATOM   1956 HG23 ILE C 357     111.333  80.694 153.132  1.00  0.00           H
ATOM   1957 HD11 ILE C 357     109.528  83.038 150.514  1.00  0.00           H
ATOM   1958 HD12 ILE C 357     109.199  82.086 151.958  1.00  0.00           H
ATOM   1959 HD13 ILE C 357     110.816  82.717 151.667  1.00  0.00           H
ATOM   1960  N   HIS C 358     114.507  79.804 152.342  1.00 30.00           N
ATOM   1961  CA  HIS C 358     115.315  78.764 152.979  1.00 30.00           C
ATOM   1962  C   HIS C 358     114.647  78.428 154.321  1.00 30.00           C
ATOM   1963  O   HIS C 358     114.921  79.086 155.325  1.00 30.00           O
ATOM   1964  CB  HIS C 358     116.772  79.252 153.159  1.00 20.00           C
ATOM   1965  CG  HIS C 358     117.686  79.040 151.973  1.00  0.00           C
ATOM   1966  CD2 HIS C 358     118.927  78.446 151.887  1.00  0.00           C
ATOM   1967  ND1 HIS C 358     117.382  79.435 150.681  1.00  0.00           N
ATOM   1968  CE1 HIS C 358     118.415  79.096 149.906  1.00  0.00           C
ATOM   1969  NE2 HIS C 358     119.388  78.484 150.569  1.00  0.00           N
ATOM   1970  H   HIS C 358     114.308  80.607 152.921  1.00  0.00           H
ATOM   1971  HA  HIS C 358     115.329  77.846 152.386  1.00  0.00           H
ATOM   1972  HB2 HIS C 358     116.791  80.310 153.424  1.00  0.00           H
ATOM   1973  HB3 HIS C 358     117.237  78.730 153.996  1.00  0.00           H
ATOM   1974  HD1 HIS C 358     116.540  79.897 150.357  1.00  0.00           H
ATOM   1975  HD2 HIS C 358     119.524  77.999 152.669  1.00  0.00           H
ATOM   1976  HE1 HIS C 358     118.450  79.290 148.843  1.00  0.00           H
```

```
ATOM   1977  N   GLU C 359     113.745  77.434 154.291  1.00 30.00           N
ATOM   1978  CA  GLU C 359     112.890  77.033 155.410  1.00 30.00           C
ATOM   1979  C   GLU C 359     112.370  75.608 155.121  1.00 30.00           C
ATOM   1980  O   GLU C 359     112.055  75.325 153.961  1.00 30.00           O
ATOM   1981  CB  GLU C 359     111.718  78.041 155.506  1.00 20.00           C
ATOM   1982  CG  GLU C 359     110.722  77.826 156.666  1.00 20.00           C
ATOM   1983  CD  GLU C 359     109.545  78.808 156.657  1.00 20.00           C
ATOM   1984  OE1 GLU C 359     109.357  79.504 155.634  1.00 20.00           O
ATOM   1985  OE2 GLU C 359     108.839  78.837 157.688  1.00 20.00           O1-
ATOM   1986  H   GLU C 359     113.571  76.946 153.424  1.00  0.00           H
ATOM   1987  HA  GLU C 359     113.492  77.063 156.317  1.00  0.00           H
ATOM   1988  HB2 GLU C 359     112.107  79.059 155.564  1.00  0.00           H
ATOM   1989  HB3 GLU C 359     111.158  77.986 154.577  1.00  0.00           H
ATOM   1990  HG2 GLU C 359     110.303  76.821 156.635  1.00  0.00           H
ATOM   1991  HG3 GLU C 359     111.241  77.926 157.619  1.00  0.00           H
ATOM   1992  N   PRO C 360     112.260  74.736 156.154  1.00 30.00           N
ATOM   1993  CA  PRO C 360     111.634  73.409 155.995  1.00 30.00           C
ATOM   1994  C   PRO C 360     110.152  73.500 155.583  1.00 30.00           C
ATOM   1995  O   PRO C 360     109.408  74.304 156.144  1.00 30.00           O
ATOM   1996  CB  PRO C 360     111.816  72.746 157.370  1.00 20.00           C
ATOM   1997  CG  PRO C 360     111.914  73.904 158.349  1.00  0.00           C
ATOM   1998  CD  PRO C 360     112.655  74.966 157.546  1.00  0.00           C
ATOM   1999  HA  PRO C 360     112.181  72.837 155.244  1.00  0.00           H
ATOM   2000  HB2 PRO C 360     111.019  72.049 157.632  1.00  0.00           H
ATOM   2001  HB3 PRO C 360     112.751  72.184 157.375  1.00  0.00           H
ATOM   2002  HG2 PRO C 360     110.912  74.262 158.587  1.00  0.00           H
ATOM   2003  HG3 PRO C 360     112.410  73.644 159.284  1.00  0.00           H
ATOM   2004  HD2 PRO C 360     112.411  75.958 157.923  1.00  0.00           H
ATOM   2005  HD3 PRO C 360     113.734  74.827 157.627  1.00  0.00           H
ATOM   2006  N   GLU C 361     109.787  72.687 154.578  1.00 30.00           N
ATOM   2007  CA  GLU C 361     108.467  72.574 153.943  1.00 30.00           C
ATOM   2008  C   GLU C 361     108.116  73.729 152.980  1.00 30.00           C
ATOM   2009  O   GLU C 361     107.035  73.690 152.390  1.00 30.00           O
ATOM   2010  CB  GLU C 361     107.336  72.316 154.973  1.00 20.00           C
ATOM   2011  CG  GLU C 361     107.570  71.079 155.864  1.00  0.00           C
ATOM   2012  CD  GLU C 361     106.386  70.813 156.793  1.00  0.00           C
ATOM   2013  OE1 GLU C 361     105.316  70.442 156.261  1.00  0.00           O
ATOM   2014  OE2 GLU C 361     106.570  70.981 158.018  1.00  0.00           O1-
ATOM   2015  H   GLU C 361     110.490  72.072 154.195  1.00  0.00           H
ATOM   2016  HA  GLU C 361     108.538  71.685 153.317  1.00  0.00           H
ATOM   2017  HB2 GLU C 361     107.188  73.193 155.603  1.00  0.00           H
ATOM   2018  HB3 GLU C 361     106.391  72.197 154.443  1.00  0.00           H
ATOM   2019  HG2 GLU C 361     107.734  70.195 155.248  1.00  0.00           H
ATOM   2020  HG3 GLU C 361     108.470  71.205 156.467  1.00  0.00           H
ATOM   2021  N   CYS C 362     109.019  74.713 152.812  1.00 30.00           N
ATOM   2022  CA  CYS C 362     108.780  75.935 152.038  1.00 30.00           C
ATOM   2023  C   CYS C 362     109.927  76.251 151.048  1.00 30.00           C
ATOM   2024  O   CYS C 362     109.762  77.137 150.210  1.00 30.00           O
ATOM   2025  CB  CYS C 362     108.409  77.099 152.984  1.00 20.00           C
ATOM   2026  SG  CYS C 362     107.607  78.484 152.124  1.00 20.00           S
ATOM   2027  H   CYS C 362     109.879  74.686 153.343  1.00  0.00           H
ATOM   2028  HA  CYS C 362     107.915  75.769 151.394  1.00  0.00           H
ATOM   2029  HB2 CYS C 362     107.705  76.752 153.741  1.00  0.00           H
ATOM   2030  HB3 CYS C 362     109.284  77.455 153.523  1.00  0.00           H
ATOM   2031  HG  CYS C 362     108.676  78.829 151.400  1.00  0.00           H
ATOM   2032  N   ARG C 363     111.029  75.474 151.078  1.00 30.00           N
ATOM   2033  CA  ARG C 363     112.047  75.408 150.016  1.00 30.00           C
ATOM   2034  C   ARG C 363     111.476  74.817 148.704  1.00 30.00           C
ATOM   2035  O   ARG C 363     111.946  75.180 147.628  1.00 30.00           O
ATOM   2036  CB  ARG C 363     113.285  74.639 150.555  1.00 20.00           C
ATOM   2037  CG  ARG C 363     114.399  74.270 149.545  1.00  0.00           C
ATOM   2038  CD  ARG C 363     115.094  75.461 148.861  1.00  0.00           C
ATOM   2039  NE  ARG C 363     116.172  75.001 147.970  1.00  0.00           N
ATOM   2040  CZ  ARG C 363     117.499  75.108 148.163  1.00  0.00           C
ATOM   2041  NH1 ARG C 363     118.005  75.755 149.221  1.00  0.00           N
ATOM   2042  NH2 ARG C 363     118.336  74.557 147.274  1.00  0.00           N1+
ATOM   2043  H   ARG C 363     111.123  74.789 151.815  1.00  0.00           H
ATOM   2044  HA  ARG C 363     112.360  76.432 149.803  1.00  0.00           H
ATOM   2045  HB2 ARG C 363     113.724  75.204 151.378  1.00  0.00           H
ATOM   2046  HB3 ARG C 363     112.935  73.704 150.995  1.00  0.00           H
ATOM   2047  HG2 ARG C 363     115.149  73.629 150.010  1.00  0.00           H
```

```
ATOM   2048  HG3 ARG C 363     113.949  73.649 148.770  1.00  0.00           H
ATOM   2049  HD2 ARG C 363     114.376  75.928 148.190  1.00  0.00           H
ATOM   2050  HD3 ARG C 363     115.392  76.246 149.557  1.00  0.00           H
ATOM   2051  HE  ARG C 363     115.860  74.519 147.138  1.00  0.00           H
ATOM   2052 HH11 ARG C 363     119.006  75.834 149.342  1.00  0.00           H
ATOM   2053 HH12 ARG C 363     117.391  76.195 149.890  1.00  0.00           H
ATOM   2054 HH21 ARG C 363     119.346  74.632 147.383  1.00  0.00           H
ATOM   2055 HH22 ARG C 363     117.980  74.010 146.500  1.00  0.00           H
ATOM   2056  N   HIS C 364     110.439  73.968 148.811  1.00 30.00           N
ATOM   2057  CA  HIS C 364     109.631  73.475 147.692  1.00 30.00           C
ATOM   2058  C   HIS C 364     108.822  74.590 146.999  1.00 30.00           C
ATOM   2059  O   HIS C 364     108.677  74.561 145.777  1.00 30.00           O
ATOM   2060  CB  HIS C 364     108.695  72.374 148.227  1.00 20.00           C
ATOM   2061  CG  HIS C 364     107.869  71.647 147.192  1.00  0.00           C
ATOM   2062  CD2 HIS C 364     108.144  71.279 145.894  1.00  0.00           C
ATOM   2063  ND1 HIS C 364     106.592  71.177 147.447  1.00  0.00           N
ATOM   2064  CE1 HIS C 364     106.167  70.566 146.338  1.00  0.00           C
ATOM   2065  NE2 HIS C 364     107.058  70.588 145.352  1.00  0.00           N
ATOM   2066  H   HIS C 364     110.115  73.708 149.731  1.00  0.00           H
ATOM   2067  HA  HIS C 364     110.307  73.038 146.955  1.00  0.00           H
ATOM   2068  HB2 HIS C 364     109.298  71.623 148.732  1.00  0.00           H
ATOM   2069  HB3 HIS C 364     108.024  72.784 148.985  1.00  0.00           H
ATOM   2070  HD1 HIS C 364     106.077  71.265 148.311  1.00  0.00           H
ATOM   2071  HD2 HIS C 364     109.044  71.448 145.321  1.00  0.00           H
ATOM   2072  HE1 HIS C 364     105.200  70.094 146.252  1.00  0.00           H
ATOM   2073  N   LEU C 365     108.319  75.547 147.797  1.00 30.00           N
ATOM   2074  CA  LEU C 365     107.483  76.666 147.355  1.00 30.00           C
ATOM   2075  C   LEU C 365     108.301  77.846 146.789  1.00 30.00           C
ATOM   2076  O   LEU C 365     107.722  78.704 146.121  1.00 30.00           O
ATOM   2077  CB  LEU C 365     106.590  77.121 148.534  1.00 20.00           C
ATOM   2078  CG  LEU C 365     105.739  76.001 149.185  1.00  0.00           C
ATOM   2079  CD1 LEU C 365     104.962  76.536 150.408  1.00  0.00           C
ATOM   2080  CD2 LEU C 365     104.819  75.287 148.172  1.00  0.00           C
ATOM   2081  H   LEU C 365     108.504  75.507 148.789  1.00  0.00           H
ATOM   2082  HA  LEU C 365     106.841  76.317 146.546  1.00  0.00           H
ATOM   2083  HB2 LEU C 365     107.224  77.569 149.299  1.00  0.00           H
ATOM   2084  HB3 LEU C 365     105.927  77.918 148.194  1.00  0.00           H
ATOM   2085  HG  LEU C 365     106.419  75.243 149.577  1.00  0.00           H
ATOM   2086 HD11 LEU C 365     105.266  76.015 151.317  1.00  0.00           H
ATOM   2087 HD12 LEU C 365     105.142  77.598 150.571  1.00  0.00           H
ATOM   2088 HD13 LEU C 365     103.884  76.414 150.312  1.00  0.00           H
ATOM   2089 HD21 LEU C 365     103.838  75.052 148.585  1.00  0.00           H
ATOM   2090 HD22 LEU C 365     104.654  75.888 147.278  1.00  0.00           H
ATOM   2091 HD23 LEU C 365     105.262  74.343 147.853  1.00  0.00           H
ATOM   2092  N   SER C 366     109.622  77.857 147.042  1.00 30.00           N
ATOM   2093  CA  SER C 366     110.603  78.772 146.459  1.00 30.00           C
ATOM   2094  C   SER C 366     110.903  78.427 144.983  1.00 30.00           C
ATOM   2095  O   SER C 366     110.690  77.290 144.558  1.00 30.00           O
ATOM   2096  CB  SER C 366     111.854  78.747 147.366  1.00 20.00           C
ATOM   2097  OG  SER C 366     112.956  79.446 146.828  1.00 20.00           O
ATOM   2098  H   SER C 366     110.007  77.111 147.604  1.00  0.00           H
ATOM   2099  HA  SER C 366     110.190  79.783 146.484  1.00  0.00           H
ATOM   2100  HB2 SER C 366     111.604  79.205 148.317  1.00  0.00           H
ATOM   2101  HB3 SER C 366     112.164  77.731 147.596  1.00  0.00           H
ATOM   2102  HG  SER C 366     113.736  79.271 147.362  1.00  0.00           H
ATOM   2103  N   ARG C 367     111.397  79.432 144.243  1.00 30.00           N
ATOM   2104  CA  ARG C 367     111.723  79.367 142.821  1.00 30.00           C
ATOM   2105  C   ARG C 367     113.241  79.468 142.623  1.00 30.00           C
ATOM   2106  O   ARG C 367     113.861  78.495 142.197  1.00 30.00           O
ATOM   2107  CB  ARG C 367     110.955  80.485 142.084  1.00 20.00           C
ATOM   2108  CG  ARG C 367     111.265  80.616 140.582  1.00 20.00           C
ATOM   2109  CD  ARG C 367     110.576  79.588 139.679  1.00 20.00           C
ATOM   2110  NE  ARG C 367     109.134  79.845 139.560  1.00 20.00           N
ATOM   2111  CZ  ARG C 367     108.262  79.108 138.851  1.00 20.00           C
ATOM   2112  NH1 ARG C 367     108.659  78.048 138.134  1.00 20.00           N
ATOM   2113  NH2 ARG C 367     106.967  79.442 138.856  1.00 20.00           N1+
ATOM   2114  H   ARG C 367     111.605  80.313 144.696  1.00  0.00           H
ATOM   2115  HA  ARG C 367     111.398  78.416 142.409  1.00  0.00           H
ATOM   2116  HB2 ARG C 367     109.882  80.354 142.231  1.00  0.00           H
ATOM   2117  HB3 ARG C 367     111.202  81.441 142.547  1.00  0.00           H
ATOM   2118  HG2 ARG C 367     112.323  80.728 140.346  1.00  0.00           H
```

```
ATOM   2119  HG3 ARG C 367     110.821  81.570 140.327  1.00  0.00           H
ATOM   2120  HD2 ARG C 367     110.625  78.626 140.182  1.00  0.00           H
ATOM   2121  HD3 ARG C 367     111.085  79.474 138.722  1.00  0.00           H
ATOM   2122  HE  ARG C 367     108.787  80.649 140.068  1.00  0.00           H
ATOM   2123 HH11 ARG C 367     107.983  77.519 137.592  1.00  0.00           H
ATOM   2124 HH12 ARG C 367     109.630  77.774 138.127  1.00  0.00           H
ATOM   2125 HH21 ARG C 367     106.302  78.894 138.328  1.00  0.00           H
ATOM   2126 HH22 ARG C 367     106.645  80.235 139.391  1.00  0.00           H
ATOM   2127  N   LYS C 368     113.794  80.655 142.921  1.00 30.00           N
ATOM   2128  CA  LYS C 368     115.217  80.960 142.864  1.00 30.00           C
ATOM   2129  C   LYS C 368     115.917  80.354 144.084  1.00 30.00           C
ATOM   2130  O   LYS C 368     115.556  80.707 145.203  1.00 30.00           O
ATOM   2131  CB  LYS C 368     115.395  82.492 142.816  1.00 20.00           C
ATOM   2132  CG  LYS C 368     116.862  82.955 142.727  1.00 20.00           C
ATOM   2133  CD  LYS C 368     116.994  84.479 142.603  1.00 20.00           C
ATOM   2134  CE  LYS C 368     118.452  84.919 142.410  1.00 20.00           C
ATOM   2135  NZ  LYS C 368     118.555  86.376 142.238  1.00 20.00           N1+
ATOM   2136  H   LYS C 368     113.212  81.384 143.311  1.00  0.00           H
ATOM   2137  HA  LYS C 368     115.629  80.541 141.946  1.00  0.00           H
ATOM   2138  HB2 LYS C 368     114.851  82.874 141.954  1.00  0.00           H
ATOM   2139  HB3 LYS C 368     114.931  82.947 143.693  1.00  0.00           H
ATOM   2140  HG2 LYS C 368     117.416  82.629 143.608  1.00  0.00           H
ATOM   2141  HG3 LYS C 368     117.339  82.474 141.873  1.00  0.00           H
ATOM   2142  HD2 LYS C 368     116.386  84.840 141.773  1.00  0.00           H
ATOM   2143  HD3 LYS C 368     116.588  84.947 143.501  1.00  0.00           H
ATOM   2144  HE2 LYS C 368     119.055  84.621 143.267  1.00  0.00           H
ATOM   2145  HE3 LYS C 368     118.879  84.436 141.530  1.00  0.00           H
ATOM   2146  HZ1 LYS C 368     118.028  86.657 141.423  1.00  0.00           H
ATOM   2147  HZ2 LYS C 368     118.185  86.840 143.055  1.00  0.00           H
ATOM   2148  HZ3 LYS C 368     119.524  86.633 142.115  1.00  0.00           H
ATOM   2149  N   PHE C 369     116.900  79.477 143.837  1.00 30.00           N
ATOM   2150  CA  PHE C 369     117.755  78.885 144.861  1.00 30.00           C
ATOM   2151  C   PHE C 369     119.180  79.374 144.589  1.00 30.00           C
ATOM   2152  O   PHE C 369     119.754  79.020 143.559  1.00 30.00           O
ATOM   2153  CB  PHE C 369     117.726  77.343 144.777  1.00 20.00           C
ATOM   2154  CG  PHE C 369     116.378  76.648 144.789  1.00 20.00           C
ATOM   2155  CD1 PHE C 369     115.235  77.196 145.411  1.00 20.00           C
ATOM   2156  CD2 PHE C 369     116.313  75.330 144.303  1.00 20.00           C
ATOM   2157  CE1 PHE C 369     114.046  76.480 145.424  1.00 20.00           C
ATOM   2158  CE2 PHE C 369     115.130  74.621 144.371  1.00 20.00           C
ATOM   2159  CZ  PHE C 369     113.989  75.197 144.905  1.00 20.00           C
ATOM   2160  H   PHE C 369     117.099  79.206 142.882  1.00  0.00           H
ATOM   2161  HA  PHE C 369     117.447  79.190 145.864  1.00  0.00           H
ATOM   2162  HB2 PHE C 369     118.244  77.004 143.878  1.00  0.00           H
ATOM   2163  HB3 PHE C 369     118.296  76.942 145.616  1.00  0.00           H
ATOM   2164  HD1 PHE C 369     115.264  78.176 145.862  1.00  0.00           H
ATOM   2165  HD2 PHE C 369     117.187  74.867 143.876  1.00  0.00           H
ATOM   2166  HE1 PHE C 369     113.160  76.908 145.855  1.00  0.00           H
ATOM   2167  HE2 PHE C 369     115.115  73.615 143.996  1.00  0.00           H
ATOM   2168  HZ  PHE C 369     113.060  74.647 144.934  1.00  0.00           H
ATOM   2169  N   THR C 370     119.735  80.155 145.525  1.00 30.00           N
ATOM   2170  CA  THR C 370     121.110  80.657 145.482  1.00 30.00           C
ATOM   2171  C   THR C 370     122.085  79.620 146.098  1.00 30.00           C
ATOM   2172  O   THR C 370     122.752  79.898 147.096  1.00 30.00           O
ATOM   2173  CB  THR C 370     121.201  82.026 146.217  1.00 20.00           C
ATOM   2174  CG2 THR C 370     120.292  83.103 145.606  1.00 20.00           C
ATOM   2175  OG1 THR C 370     120.904  81.927 147.599  1.00 20.00           O
ATOM   2176  H   THR C 370     119.189  80.439 146.330  1.00  0.00           H
ATOM   2177  HA  THR C 370     121.411  80.816 144.444  1.00  0.00           H
ATOM   2178  HB  THR C 370     122.227  82.389 146.138  1.00  0.00           H
ATOM   2179  HG1 THR C 370     121.534  81.319 147.997  1.00  0.00           H
ATOM   2180 HG21 THR C 370     120.413  84.056 146.121  1.00  0.00           H
ATOM   2181 HG22 THR C 370     120.539  83.265 144.558  1.00  0.00           H
ATOM   2182 HG23 THR C 370     119.238  82.831 145.661  1.00  0.00           H
ATOM   2183  N   GLU C 371     122.139  78.421 145.485  1.00 30.00           N
ATOM   2184  CA  GLU C 371     122.908  77.265 145.959  1.00 30.00           C
ATOM   2185  C   GLU C 371     124.425  77.521 146.026  1.00 30.00           C
ATOM   2186  O   GLU C 371     125.060  77.133 147.005  1.00 30.00           O
ATOM   2187  CB  GLU C 371     122.601  76.041 145.066  1.00 20.00           C
ATOM   2188  CG  GLU C 371     123.146  74.703 145.616  1.00  0.00           C
ATOM   2189  CD  GLU C 371     122.399  74.174 146.849  1.00  0.00           C
```

ATOM	2190	OE1	GLU	C	371	121.236	74.588	147.064	1.00	0.00	O
ATOM	2191	OE2	GLU	C	371	123.001	73.331	147.546	1.00	0.00	O1-
ATOM	2192	H	GLU	C	371	121.565	78.265	144.668	1.00	0.00	H
ATOM	2193	HA	GLU	C	371	122.559	77.057	146.972	1.00	0.00	H
ATOM	2194	HB2	GLU	C	371	121.526	75.961	144.899	1.00	0.00	H
ATOM	2195	HB3	GLU	C	371	123.024	76.204	144.073	1.00	0.00	H
ATOM	2196	HG2	GLU	C	371	123.105	73.949	144.835	1.00	0.00	H
ATOM	2197	HG3	GLU	C	371	124.206	74.785	145.859	1.00	0.00	H
ATOM	2198	N	TRP	C	372	124.958	78.193	144.993	1.00	30.00	N
ATOM	2199	CA	TRP	C	372	126.353	78.604	144.890	1.00	30.00	C
ATOM	2200	C	TRP	C	372	126.469	80.127	145.054	1.00	30.00	C
ATOM	2201	O	TRP	C	372	126.973	80.820	144.169	1.00	30.00	O
ATOM	2202	CB	TRP	C	372	126.967	78.044	143.590	1.00	20.00	C
ATOM	2203	CG	TRP	C	372	127.479	76.640	143.692	1.00	0.00	C
ATOM	2204	CD1	TRP	C	372	126.744	75.510	143.583	1.00	0.00	C
ATOM	2205	CD2	TRP	C	372	128.838	76.206	144.008	1.00	0.00	C
ATOM	2206	CE2	TRP	C	372	128.859	74.780	144.058	1.00	0.00	C
ATOM	2207	CE3	TRP	C	372	130.060	76.876	144.259	1.00	0.00	C
ATOM	2208	NE1	TRP	C	372	127.555	74.412	143.789	1.00	0.00	N
ATOM	2209	CZ2	TRP	C	372	130.032	74.056	144.340	1.00	0.00	C
ATOM	2210	CZ3	TRP	C	372	131.241	76.162	144.548	1.00	0.00	C
ATOM	2211	CH2	TRP	C	372	131.228	74.754	144.587	1.00	0.00	C
ATOM	2212	H	TRP	C	372	124.357	78.491	144.237	1.00	0.00	H
ATOM	2213	HA	TRP	C	372	126.944	78.205	145.717	1.00	0.00	H
ATOM	2214	HB2	TRP	C	372	126.243	78.098	142.776	1.00	0.00	H
ATOM	2215	HB3	TRP	C	372	127.825	78.640	143.281	1.00	0.00	H
ATOM	2216	HD1	TRP	C	372	125.684	75.489	143.376	1.00	0.00	H
ATOM	2217	HE1	TRP	C	372	127.206	73.465	143.752	1.00	0.00	H
ATOM	2218	HE3	TRP	C	372	130.089	77.954	144.222	1.00	0.00	H
ATOM	2219	HZ2	TRP	C	372	130.013	72.977	144.371	1.00	0.00	H
ATOM	2220	HZ3	TRP	C	372	132.159	76.699	144.737	1.00	0.00	H
ATOM	2221	HH2	TRP	C	372	132.136	74.212	144.808	1.00	0.00	H
ATOM	2222	N	ALA	C	373	126.058	80.599	146.243	1.00	30.00	N
ATOM	2223	CA	ALA	C	373	126.381	81.917	146.788	1.00	30.00	C
ATOM	2224	C	ALA	C	373	127.774	81.862	147.448	1.00	30.00	C
ATOM	2225	O	ALA	C	373	127.886	81.930	148.673	1.00	30.00	O
ATOM	2226	CB	ALA	C	373	125.271	82.326	147.773	1.00	30.00	C
ATOM	2227	H	ALA	C	373	125.627	79.958	146.894	1.00	0.00	H
ATOM	2228	HA	ALA	C	373	126.414	82.656	145.985	1.00	0.00	H
ATOM	2229	HB1	ALA	C	373	125.493	83.284	148.244	1.00	0.00	H
ATOM	2230	HB2	ALA	C	373	124.316	82.430	147.259	1.00	0.00	H
ATOM	2231	HB3	ALA	C	373	125.141	81.587	148.565	1.00	0.00	H
ATOM	2232	N	TYR	C	374	128.800	81.674	146.604	1.00	30.00	N
ATOM	2233	CA	TYR	C	374	130.190	81.410	146.972	1.00	30.00	C
ATOM	2234	C	TYR	C	374	130.942	82.745	147.132	1.00	30.00	C
ATOM	2235	O	TYR	C	374	130.595	83.524	148.020	1.00	30.00	O
ATOM	2236	CB	TYR	C	374	130.753	80.433	145.908	1.00	20.00	C
ATOM	2237	CG	TYR	C	374	132.181	79.950	146.104	1.00	0.00	C
ATOM	2238	CD1	TYR	C	374	132.511	79.169	147.231	1.00	0.00	C
ATOM	2239	CD2	TYR	C	374	133.180	80.268	145.158	1.00	0.00	C
ATOM	2240	CE1	TYR	C	374	133.832	78.720	147.419	1.00	0.00	C
ATOM	2241	CE2	TYR	C	374	134.502	79.824	145.350	1.00	0.00	C
ATOM	2242	CZ	TYR	C	374	134.829	79.051	146.481	1.00	0.00	C
ATOM	2243	OH	TYR	C	374	136.111	78.621	146.663	1.00	0.00	O
ATOM	2244	H	TYR	C	374	128.603	81.646	145.613	1.00	0.00	H
ATOM	2245	HA	TYR	C	374	130.215	80.909	147.942	1.00	0.00	H
ATOM	2246	HB2	TYR	C	374	130.123	79.543	145.893	1.00	0.00	H
ATOM	2247	HB3	TYR	C	374	130.658	80.872	144.913	1.00	0.00	H
ATOM	2248	HD1	TYR	C	374	131.751	78.912	147.955	1.00	0.00	H
ATOM	2249	HD2	TYR	C	374	132.939	80.855	144.285	1.00	0.00	H
ATOM	2250	HE1	TYR	C	374	134.075	78.124	148.286	1.00	0.00	H
ATOM	2251	HE2	TYR	C	374	135.264	80.073	144.625	1.00	0.00	H
ATOM	2252	HH	TYR	C	374	136.223	78.093	147.456	1.00	0.00	H
ATOM	2253	N	GLY	C	375	131.910	83.022	146.241	1.00	30.00	N
ATOM	2254	CA	GLY	C	375	132.497	84.346	146.042	1.00	30.00	C
ATOM	2255	C	GLY	C	375	131.487	85.268	145.325	1.00	30.00	C
ATOM	2256	O	GLY	C	375	130.327	84.888	145.152	1.00	30.00	O
ATOM	2257	H	GLY	C	375	132.152	82.318	145.559	1.00	0.00	H
ATOM	2258	HA2	GLY	C	375	133.400	84.239	145.439	1.00	0.00	H
ATOM	2259	HA3	GLY	C	375	132.785	84.779	147.001	1.00	0.00	H
ATOM	2260	N	PRO	C	376	131.900	86.488	144.914	1.00	30.00	N

ATOM	2261	CA	PRO	C	376	131.000	87.520	144.355	1.00	30.00	C
ATOM	2262	C	PRO	C	376	130.030	87.114	143.223	1.00	30.00	C
ATOM	2263	O	PRO	C	376	128.941	87.683	143.166	1.00	30.00	O
ATOM	2264	CB	PRO	C	376	131.960	88.637	143.935	1.00	20.00	C
ATOM	2265	CG	PRO	C	376	133.121	88.518	144.906	1.00	20.00	C
ATOM	2266	CD	PRO	C	376	133.256	87.013	145.094	1.00	20.00	C
ATOM	2267	HA	PRO	C	376	130.398	87.878	145.192	1.00	0.00	H
ATOM	2268	HB2	PRO	C	376	132.316	88.461	142.920	1.00	0.00	H
ATOM	2269	HB3	PRO	C	376	131.502	89.626	143.973	1.00	0.00	H
ATOM	2270	HG2	PRO	C	376	134.036	88.995	144.553	1.00	0.00	H
ATOM	2271	HG3	PRO	C	376	132.844	88.981	145.854	1.00	0.00	H
ATOM	2272	HD2	PRO	C	376	133.901	86.586	144.325	1.00	0.00	H
ATOM	2273	HD3	PRO	C	376	133.686	86.784	146.070	1.00	0.00	H
ATOM	2274	N	VAL	C	377	130.390	86.112	142.399	1.00	30.00	N
ATOM	2275	CA	VAL	C	377	129.479	85.477	141.444	1.00	30.00	C
ATOM	2276	C	VAL	C	377	128.501	84.532	142.175	1.00	30.00	C
ATOM	2277	O	VAL	C	377	128.924	83.497	142.694	1.00	30.00	O
ATOM	2278	CB	VAL	C	377	130.256	84.695	140.341	1.00	20.00	C
ATOM	2279	CG1	VAL	C	377	129.390	83.773	139.448	1.00	20.00	C
ATOM	2280	CG2	VAL	C	377	131.054	85.666	139.452	1.00	20.00	C
ATOM	2281	H	VAL	C	377	131.293	85.676	142.508	1.00	0.00	H
ATOM	2282	HA	VAL	C	377	128.908	86.270	140.965	1.00	0.00	H
ATOM	2283	HB	VAL	C	377	130.986	84.052	140.836	1.00	0.00	H
ATOM	2284	HG11	VAL	C	377	129.980	83.347	138.636	1.00	0.00	H
ATOM	2285	HG12	VAL	C	377	128.972	82.931	139.999	1.00	0.00	H
ATOM	2286	HG13	VAL	C	377	128.561	84.315	138.994	1.00	0.00	H
ATOM	2287	HG21	VAL	C	377	131.626	85.131	138.693	1.00	0.00	H
ATOM	2288	HG22	VAL	C	377	130.393	86.364	138.936	1.00	0.00	H
ATOM	2289	HG23	VAL	C	377	131.763	86.254	140.035	1.00	0.00	H
ATOM	2290	N	HIS	C	378	127.216	84.923	142.206	1.00	30.00	N
ATOM	2291	CA	HIS	C	378	126.140	84.200	142.887	1.00	30.00	C
ATOM	2292	C	HIS	C	378	125.356	83.341	141.884	1.00	30.00	C
ATOM	2293	O	HIS	C	378	124.303	83.761	141.403	1.00	30.00	O
ATOM	2294	CB	HIS	C	378	125.227	85.201	143.630	1.00	20.00	C
ATOM	2295	CG	HIS	C	378	125.791	85.717	144.930	1.00	0.00	C
ATOM	2296	CD2	HIS	C	378	125.341	85.590	146.226	1.00	0.00	C
ATOM	2297	ND1	HIS	C	378	126.960	86.452	145.017	1.00	0.00	N
ATOM	2298	CE1	HIS	C	378	127.169	86.717	146.308	1.00	0.00	C
ATOM	2299	NE2	HIS	C	378	126.225	86.225	147.101	1.00	0.00	N
ATOM	2300	H	HIS	C	378	126.953	85.788	141.756	1.00	0.00	H
ATOM	2301	HA	HIS	C	378	126.550	83.518	143.635	1.00	0.00	H
ATOM	2302	HB2	HIS	C	378	124.989	86.054	142.992	1.00	0.00	H
ATOM	2303	HB3	HIS	C	378	124.272	84.730	143.872	1.00	0.00	H
ATOM	2304	HD1	HIS	C	378	127.557	86.737	144.249	1.00	0.00	H
ATOM	2305	HD2	HIS	C	378	124.460	85.085	146.596	1.00	0.00	H
ATOM	2306	HE1	HIS	C	378	128.020	87.275	146.670	1.00	0.00	H
ATOM	2307	N	SER	C	379	125.887	82.142	141.591	1.00	30.00	N
ATOM	2308	CA	SER	C	379	125.248	81.145	140.732	1.00	30.00	C
ATOM	2309	C	SER	C	379	123.931	80.628	141.344	1.00	30.00	C
ATOM	2310	O	SER	C	379	123.931	80.105	142.460	1.00	30.00	O
ATOM	2311	CB	SER	C	379	126.267	80.042	140.385	1.00	20.00	C
ATOM	2312	OG	SER	C	379	125.688	78.980	139.651	1.00	20.00	O
ATOM	2313	H	SER	C	379	126.746	81.858	142.042	1.00	0.00	H
ATOM	2314	HA	SER	C	379	124.998	81.651	139.800	1.00	0.00	H
ATOM	2315	HB2	SER	C	379	127.078	80.464	139.789	1.00	0.00	H
ATOM	2316	HB3	SER	C	379	126.726	79.645	141.286	1.00	0.00	H
ATOM	2317	HG	SER	C	379	125.375	79.325	138.808	1.00	0.00	H
ATOM	2318	N	SER	C	380	122.840	80.844	140.593	1.00	30.00	N
ATOM	2319	CA	SER	C	380	121.457	80.632	141.016	1.00	30.00	C
ATOM	2320	C	SER	C	380	120.754	79.657	140.063	1.00	30.00	C
ATOM	2321	O	SER	C	380	121.147	79.553	138.900	1.00	30.00	O
ATOM	2322	CB	SER	C	380	120.734	81.994	140.998	1.00	20.00	C
ATOM	2323	OG	SER	C	380	121.227	82.830	142.024	1.00	20.00	O
ATOM	2324	H	SER	C	380	122.952	81.239	139.670	1.00	0.00	H
ATOM	2325	HA	SER	C	380	121.425	80.213	142.022	1.00	0.00	H
ATOM	2326	HB2	SER	C	380	119.664	81.862	141.162	1.00	0.00	H
ATOM	2327	HB3	SER	C	380	120.849	82.493	140.034	1.00	0.00	H
ATOM	2328	HG	SER	C	380	122.146	83.038	141.831	1.00	0.00	H
ATOM	2329	N	LEU	C	381	119.717	78.973	140.574	1.00	30.00	N
ATOM	2330	CA	LEU	C	381	118.928	78.002	139.818	1.00	30.00	C
ATOM	2331	C	LEU	C	381	117.425	78.249	140.015	1.00	30.00	C

```
ATOM   2332  O   LEU C 381     117.007  78.553 141.128  1.00 30.00           O
ATOM   2333  CB  LEU C 381     119.304  76.559 140.222  1.00 20.00           C
ATOM   2334  CG  LEU C 381     120.766  76.144 139.935  1.00 20.00           C
ATOM   2335  CD1 LEU C 381     121.723  76.493 141.098  1.00 20.00           C
ATOM   2336  CD2 LEU C 381     120.850  74.655 139.548  1.00 20.00           C
ATOM   2337  H   LEU C 381     119.459  79.098 141.544  1.00  0.00           H
ATOM   2338  HA  LEU C 381     119.129  78.140 138.761  1.00  0.00           H
ATOM   2339  HB2 LEU C 381     119.037  76.360 141.260  1.00  0.00           H
ATOM   2340  HB3 LEU C 381     118.672  75.905 139.626  1.00  0.00           H
ATOM   2341  HG  LEU C 381     121.106  76.702 139.064  1.00  0.00           H
ATOM   2342 HD11 LEU C 381     122.204  75.612 141.523  1.00  0.00           H
ATOM   2343 HD12 LEU C 381     122.515  77.162 140.761  1.00  0.00           H
ATOM   2344 HD13 LEU C 381     121.208  76.993 141.919  1.00  0.00           H
ATOM   2345 HD21 LEU C 381     121.813  74.212 139.802  1.00  0.00           H
ATOM   2346 HD22 LEU C 381     120.078  74.062 140.038  1.00  0.00           H
ATOM   2347 HD23 LEU C 381     120.711  74.532 138.474  1.00  0.00           H
ATOM   2348  N   TYR C 382     116.648  78.097 138.928  1.00 30.00           N
ATOM   2349  CA  TYR C 382     115.211  78.400 138.863  1.00 30.00           C
ATOM   2350  C   TYR C 382     114.420  77.148 138.448  1.00 30.00           C
ATOM   2351  O   TYR C 382     114.881  76.428 137.561  1.00 30.00           O
ATOM   2352  CB  TYR C 382     114.990  79.534 137.837  1.00 20.00           C
ATOM   2353  CG  TYR C 382     115.815  80.784 138.094  1.00 20.00           C
ATOM   2354  CD1 TYR C 382     115.355  81.768 138.993  1.00 20.00           C
ATOM   2355  CD2 TYR C 382     117.059  80.955 137.450  1.00 20.00           C
ATOM   2356  CE1 TYR C 382     116.130  82.918 139.239  1.00 20.00           C
ATOM   2357  CE2 TYR C 382     117.837  82.098 137.707  1.00 20.00           C
ATOM   2358  CZ  TYR C 382     117.373  83.084 138.599  1.00 20.00           C
ATOM   2359  OH  TYR C 382     118.125  84.199 138.832  1.00 20.00           O
ATOM   2360  H   TYR C 382     117.074  77.807 138.058  1.00  0.00           H
ATOM   2361  HA  TYR C 382     114.847  78.739 139.832  1.00  0.00           H
ATOM   2362  HB2 TYR C 382     115.225  79.178 136.832  1.00  0.00           H
ATOM   2363  HB3 TYR C 382     113.936  79.813 137.815  1.00  0.00           H
ATOM   2364  HD1 TYR C 382     114.406  81.646 139.495  1.00  0.00           H
ATOM   2365  HD2 TYR C 382     117.428  80.203 136.767  1.00  0.00           H
ATOM   2366  HE1 TYR C 382     115.761  83.677 139.911  1.00  0.00           H
ATOM   2367  HE2 TYR C 382     118.789  82.216 137.217  1.00  0.00           H
ATOM   2368  HH  TYR C 382     118.908  84.237 138.276  1.00  0.00           H
ATOM   2369  N   ASP C 383     113.244  76.923 139.074  1.00 30.00           N
ATOM   2370  CA  ASP C 383     112.400  75.719 138.907  1.00 30.00           C
ATOM   2371  C   ASP C 383     111.903  75.428 137.473  1.00 30.00           C
ATOM   2372  O   ASP C 383     111.523  74.289 137.207  1.00 30.00           O
ATOM   2373  CB  ASP C 383     111.163  75.737 139.839  1.00 20.00           C
ATOM   2374  CG  ASP C 383     111.411  75.856 141.343  1.00  0.00           C
ATOM   2375  OD1 ASP C 383     112.510  75.805 141.884  1.00  0.00           O
ATOM   2376  OD2 ASP C 383     110.243  76.025 142.025  1.00  0.00           O
ATOM   2377  H   ASP C 383     112.961  77.553 139.811  1.00  0.00           H
ATOM   2378  HA  ASP C 383     113.019  74.868 139.193  1.00  0.00           H
ATOM   2379  HB2 ASP C 383     110.486  76.533 139.539  1.00  0.00           H
ATOM   2380  HB3 ASP C 383     110.606  74.816 139.705  1.00  0.00           H
ATOM   2381  HD2 ASP C 383     110.383  76.132 142.973  1.00  0.00           H
ATOM   2382  N   LEU C 384     111.905  76.439 136.586  1.00 30.00           N
ATOM   2383  CA  LEU C 384     111.706  76.326 135.136  1.00 30.00           C
ATOM   2384  C   LEU C 384     110.413  75.588 134.712  1.00 30.00           C
ATOM   2385  O   LEU C 384     110.469  74.585 134.003  1.00 30.00           O
ATOM   2386  CB  LEU C 384     113.009  75.768 134.497  1.00 20.00           C
ATOM   2387  CG  LEU C 384     113.089  75.796 132.951  1.00 20.00           C
ATOM   2388  CD1 LEU C 384     112.778  77.190 132.380  1.00 20.00           C
ATOM   2389  CD2 LEU C 384     114.439  75.246 132.445  1.00 20.00           C
ATOM   2390  H   LEU C 384     112.225  77.342 136.903  1.00  0.00           H
ATOM   2391  HA  LEU C 384     111.581  77.355 134.800  1.00  0.00           H
ATOM   2392  HB2 LEU C 384     113.855  76.337 134.886  1.00  0.00           H
ATOM   2393  HB3 LEU C 384     113.161  74.746 134.842  1.00  0.00           H
ATOM   2394  HG  LEU C 384     112.340  75.118 132.547  1.00  0.00           H
ATOM   2395 HD11 LEU C 384     113.292  77.355 131.438  1.00  0.00           H
ATOM   2396 HD12 LEU C 384     111.711  77.297 132.179  1.00  0.00           H
ATOM   2397 HD13 LEU C 384     113.074  77.989 133.061  1.00  0.00           H
ATOM   2398 HD21 LEU C 384     114.288  74.389 131.788  1.00  0.00           H
ATOM   2399 HD22 LEU C 384     115.009  75.985 131.881  1.00  0.00           H
ATOM   2400 HD23 LEU C 384     115.077  74.908 133.263  1.00  0.00           H
ATOM   2401  N   SER C 385     109.260  76.132 135.131  1.00 30.00           N
ATOM   2402  CA  SER C 385     107.939  75.784 134.591  1.00 30.00           C
```

```
ATOM   2403  C   SER C 385     107.520  76.760 133.466  1.00 30.00           C
ATOM   2404  O   SER C 385     106.336  76.839 133.141  1.00 30.00           O
ATOM   2405  CB  SER C 385     106.926  75.709 135.757  1.00 20.00           C
ATOM   2406  OG  SER C 385     106.590  76.986 136.270  1.00  0.00           O
ATOM   2407  H   SER C 385     109.284  76.942 135.732  1.00  0.00           H
ATOM   2408  HA  SER C 385     107.976  74.791 134.141  1.00  0.00           H
ATOM   2409  HB2 SER C 385     106.007  75.229 135.416  1.00  0.00           H
ATOM   2410  HB3 SER C 385     107.315  75.086 136.564  1.00  0.00           H
ATOM   2411  HG  SER C 385     106.019  77.418 135.626  1.00  0.00           H
ATOM   2412  N   CYS C 386     108.496  77.518 132.934  1.00 30.00           N
ATOM   2413  CA  CYS C 386     108.294  78.670 132.060  1.00 30.00           C
ATOM   2414  C   CYS C 386     108.363  78.292 130.578  1.00 30.00           C
ATOM   2415  O   CYS C 386     107.613  78.862 129.788  1.00 30.00           O
ATOM   2416  CB  CYS C 386     109.347  79.763 132.324  1.00 20.00           C
ATOM   2417  SG  CYS C 386     109.314  80.269 134.060  1.00  0.00           S
ATOM   2418  H   CYS C 386     109.441  77.378 133.258  1.00  0.00           H
ATOM   2419  HA  CYS C 386     107.308  79.091 132.257  1.00  0.00           H
ATOM   2420  HB2 CYS C 386     110.352  79.413 132.085  1.00  0.00           H
ATOM   2421  HB3 CYS C 386     109.152  80.638 131.705  1.00  0.00           H
ATOM   2422  HG  CYS C 386     110.243  81.224 133.946  1.00  0.00           H
ATOM   2423  N   ILE C 387     109.275  77.372 130.225  1.00 30.00           N
ATOM   2424  CA  ILE C 387     109.504  76.917 128.854  1.00 30.00           C
ATOM   2425  C   ILE C 387     108.451  75.901 128.382  1.00 30.00           C
ATOM   2426  O   ILE C 387     107.912  75.136 129.184  1.00 30.00           O
ATOM   2427  CB  ILE C 387     110.928  76.321 128.659  1.00 20.00           C
ATOM   2428  CG1 ILE C 387     111.339  75.240 129.694  1.00  0.00           C
ATOM   2429  CG2 ILE C 387     111.959  77.459 128.612  1.00  0.00           C
ATOM   2430  CD1 ILE C 387     111.351  73.822 129.124  1.00  0.00           C
ATOM   2431  H   ILE C 387     109.843  76.936 130.936  1.00  0.00           H
ATOM   2432  HA  ILE C 387     109.415  77.786 128.200  1.00  0.00           H
ATOM   2433  HB  ILE C 387     110.967  75.859 127.671  1.00  0.00           H
ATOM   2434 HG12 ILE C 387     112.344  75.445 130.061  1.00  0.00           H
ATOM   2435 HG13 ILE C 387     110.702  75.259 130.579  1.00  0.00           H
ATOM   2436 HG21 ILE C 387     112.961  77.077 128.420  1.00  0.00           H
ATOM   2437 HG22 ILE C 387     111.724  78.169 127.818  1.00  0.00           H
ATOM   2438 HG23 ILE C 387     111.978  78.012 129.550  1.00  0.00           H
ATOM   2439 HD11 ILE C 387     111.571  73.088 129.901  1.00  0.00           H
ATOM   2440 HD12 ILE C 387     110.383  73.576 128.697  1.00  0.00           H
ATOM   2441 HD13 ILE C 387     112.104  73.717 128.342  1.00  0.00           H
ATOM   2442  N   ASP C 388     108.175  75.958 127.070  1.00 30.00           N
ATOM   2443  CA  ASP C 388     107.108  75.237 126.365  1.00 30.00           C
ATOM   2444  C   ASP C 388     107.295  73.707 126.323  1.00 30.00           C
ATOM   2445  O   ASP C 388     106.324  72.998 126.067  1.00 30.00           O
ATOM   2446  CB  ASP C 388     106.854  75.782 124.933  1.00 20.00           C
ATOM   2447  CG  ASP C 388     106.831  77.311 124.755  1.00  0.00           C
ATOM   2448  OD1 ASP C 388     106.722  78.049 125.760  1.00  0.00           O
ATOM   2449  OD2 ASP C 388     106.876  77.722 123.576  1.00  0.00           O1-
ATOM   2450  H   ASP C 388     108.652  76.649 126.509  1.00  0.00           H
ATOM   2451  HA  ASP C 388     106.194  75.399 126.935  1.00  0.00           H
ATOM   2452  HB2 ASP C 388     107.634  75.393 124.275  1.00  0.00           H
ATOM   2453  HB3 ASP C 388     105.898  75.398 124.573  1.00  0.00           H
ATOM   2454  N   THR C 389     108.520  73.223 126.588  1.00 30.00           N
ATOM   2455  CA  THR C 389     108.853  71.799 126.689  1.00 30.00           C
ATOM   2456  C   THR C 389     108.437  71.168 128.042  1.00 30.00           C
ATOM   2457  O   THR C 389     108.350  69.944 128.131  1.00 30.00           O
ATOM   2458  CB  THR C 389     110.379  71.549 126.491  1.00 20.00           C
ATOM   2459  CG2 THR C 389     110.718  70.145 125.963  1.00  0.00           C
ATOM   2460  OG1 THR C 389     110.920  72.472 125.563  1.00  0.00           O
ATOM   2461  H   THR C 389     109.283  73.864 126.744  1.00  0.00           H
ATOM   2462  HA  THR C 389     108.313  71.268 125.902  1.00  0.00           H
ATOM   2463  HB  THR C 389     110.913  71.688 127.431  1.00  0.00           H
ATOM   2464  HG1 THR C 389     111.865  72.316 125.480  1.00  0.00           H
ATOM   2465 HG21 THR C 389     111.795  70.018 125.846  1.00  0.00           H
ATOM   2466 HG22 THR C 389     110.372  69.361 126.634  1.00  0.00           H
ATOM   2467 HG23 THR C 389     110.258  69.968 124.990  1.00  0.00           H
ATOM   2468  N   CYS C 390     108.185  72.012 129.063  1.00 30.00           N
ATOM   2469  CA  CYS C 390     107.804  71.614 130.419  1.00 30.00           C
ATOM   2470  C   CYS C 390     106.274  71.560 130.579  1.00 30.00           C
ATOM   2471  O   CYS C 390     105.764  70.545 131.054  1.00 30.00           O
ATOM   2472  CB  CYS C 390     108.460  72.532 131.478  1.00 20.00           C
ATOM   2473  SG  CYS C 390     108.174  71.950 133.176  1.00  0.00           S
```

ATOM	2474	H	CYS	C	390	108.247	73.008	128.901	1.00	0.00	H
ATOM	2475	HA	CYS	C	390	108.184	70.606	130.600	1.00	0.00	H
ATOM	2476	HB2	CYS	C	390	109.538	72.571	131.321	1.00	0.00	H
ATOM	2477	HB3	CYS	C	390	108.096	73.556	131.392	1.00	0.00	H
ATOM	2478	HG	CYS	C	390	106.859	72.198	133.212	1.00	0.00	H
ATOM	2479	N	GLU	C	391	105.583	72.645	130.188	1.00	30.00	N
ATOM	2480	CA	GLU	C	391	104.135	72.817	130.350	1.00	30.00	C
ATOM	2481	C	GLU	C	391	103.460	73.038	128.987	1.00	30.00	C
ATOM	2482	O	GLU	C	391	104.109	73.489	128.044	1.00	30.00	O
ATOM	2483	CB	GLU	C	391	103.859	74.043	131.256	1.00	20.00	C
ATOM	2484	CG	GLU	C	391	104.549	74.048	132.638	1.00	0.00	C
ATOM	2485	CD	GLU	C	391	104.165	72.885	133.554	1.00	0.00	C
ATOM	2486	OE1	GLU	C	391	103.019	72.398	133.437	1.00	0.00	O
ATOM	2487	OE2	GLU	C	391	105.030	72.519	134.379	1.00	0.00	O1-
ATOM	2488	H	GLU	C	391	106.072	73.426	129.775	1.00	0.00	H
ATOM	2489	HA	GLU	C	391	103.691	71.929	130.803	1.00	0.00	H
ATOM	2490	HB2	GLU	C	391	104.155	74.951	130.733	1.00	0.00	H
ATOM	2491	HB3	GLU	C	391	102.783	74.146	131.404	1.00	0.00	H
ATOM	2492	HG2	GLU	C	391	105.633	74.062	132.515	1.00	0.00	H
ATOM	2493	HG3	GLU	C	391	104.295	74.971	133.159	1.00	0.00	H
ATOM	2494	N	LYS	C	392	102.146	72.766	128.937	1.00	30.00	N
ATOM	2495	CA	LYS	C	392	101.267	73.127	127.823	1.00	30.00	C
ATOM	2496	C	LYS	C	392	100.640	74.505	128.104	1.00	30.00	C
ATOM	2497	O	LYS	C	392	100.184	74.737	129.224	1.00	30.00	O
ATOM	2498	CB	LYS	C	392	100.203	72.020	127.655	1.00	20.00	C
ATOM	2499	CG	LYS	C	392	99.231	72.242	126.481	1.00	0.00	C
ATOM	2500	CD	LYS	C	392	98.287	71.050	126.264	1.00	0.00	C
ATOM	2501	CE	LYS	C	392	97.315	71.277	125.099	1.00	0.00	C
ATOM	2502	NZ	LYS	C	392	96.428	70.117	124.906	1.00	0.00	N1+
ATOM	2503	H	LYS	C	392	101.685	72.413	129.763	1.00	0.00	H
ATOM	2504	HA	LYS	C	392	101.847	73.183	126.899	1.00	0.00	H
ATOM	2505	HB2	LYS	C	392	100.717	71.070	127.504	1.00	0.00	H
ATOM	2506	HB3	LYS	C	392	99.632	71.911	128.578	1.00	0.00	H
ATOM	2507	HG2	LYS	C	392	98.635	73.137	126.657	1.00	0.00	H
ATOM	2508	HG3	LYS	C	392	99.800	72.426	125.569	1.00	0.00	H
ATOM	2509	HD2	LYS	C	392	98.877	70.151	126.079	1.00	0.00	H
ATOM	2510	HD3	LYS	C	392	97.725	70.865	127.181	1.00	0.00	H
ATOM	2511	HE2	LYS	C	392	96.702	72.161	125.284	1.00	0.00	H
ATOM	2512	HE3	LYS	C	392	97.866	71.454	124.174	1.00	0.00	H
ATOM	2513	HZ1	LYS	C	392	95.801	70.296	124.135	1.00	0.00	H
ATOM	2514	HZ2	LYS	C	392	96.983	69.298	124.704	1.00	0.00	H
ATOM	2515	HZ3	LYS	C	392	95.891	69.960	125.747	1.00	0.00	H
ATOM	2516	N	ASN	C	393	100.632	75.384	127.081	1.00	30.00	N
ATOM	2517	CA	ASN	C	393	100.188	76.790	127.141	1.00	30.00	C
ATOM	2518	C	ASN	C	393	101.076	77.606	128.104	1.00	30.00	C
ATOM	2519	O	ASN	C	393	100.571	78.249	129.026	1.00	30.00	O
ATOM	2520	CB	ASN	C	393	98.670	76.922	127.457	1.00	20.00	C
ATOM	2521	CG	ASN	C	393	97.750	76.152	126.500	1.00	0.00	C
ATOM	2522	ND2	ASN	C	393	97.922	76.338	125.189	1.00	0.00	N
ATOM	2523	OD1	ASN	C	393	96.883	75.402	126.942	1.00	0.00	O
ATOM	2524	H	ASN	C	393	101.034	75.105	126.197	1.00	0.00	H
ATOM	2525	HA	ASN	C	393	100.356	77.226	126.157	1.00	0.00	H
ATOM	2526	HB2	ASN	C	393	98.461	76.591	128.475	1.00	0.00	H
ATOM	2527	HB3	ASN	C	393	98.377	77.972	127.417	1.00	0.00	H
ATOM	2528	HD21	ASN	C	393	97.329	75.857	124.529	1.00	0.00	H
ATOM	2529	HD22	ASN	C	393	98.644	76.956	124.846	1.00	0.00	H
ATOM	2530	N	SER	C	394	102.398	77.502	127.892	1.00	30.00	N
ATOM	2531	CA	SER	C	394	103.432	77.937	128.833	1.00	30.00	C
ATOM	2532	C	SER	C	394	103.773	79.438	128.712	1.00	30.00	C
ATOM	2533	O	SER	C	394	103.253	80.121	127.831	1.00	30.00	O
ATOM	2534	CB	SER	C	394	104.653	77.023	128.630	1.00	20.00	C
ATOM	2535	OG	SER	C	394	105.455	76.986	129.789	1.00	20.00	O
ATOM	2536	H	SER	C	394	102.724	76.994	127.081	1.00	0.00	H
ATOM	2537	HA	SER	C	394	103.048	77.766	129.841	1.00	0.00	H
ATOM	2538	HB2	SER	C	394	104.339	76.003	128.419	1.00	0.00	H
ATOM	2539	HB3	SER	C	394	105.250	77.356	127.782	1.00	0.00	H
ATOM	2540	HG	SER	C	394	106.213	76.419	129.618	1.00	0.00	H
ATOM	2541	N	VAL	C	395	104.613	79.930	129.640	1.00	30.00	N
ATOM	2542	CA	VAL	C	395	104.894	81.351	129.879	1.00	30.00	C
ATOM	2543	C	VAL	C	395	105.492	82.111	128.676	1.00	30.00	C
ATOM	2544	O	VAL	C	395	104.996	83.197	128.377	1.00	30.00	O

ATOM	2545	CB	VAL	C	395	105.806	81.558	131.126	1.00	20.00	C
ATOM	2546	CG1	VAL	C	395	106.234	83.018	131.397	1.00	20.00	C
ATOM	2547	CG2	VAL	C	395	105.146	80.983	132.391	1.00	20.00	C
ATOM	2548	H	VAL	C	395	105.035	79.283	130.294	1.00	0.00	H
ATOM	2549	HA	VAL	C	395	103.930	81.817	130.095	1.00	0.00	H
ATOM	2550	HB	VAL	C	395	106.726	81.001	130.964	1.00	0.00	H
ATOM	2551	HG11	VAL	C	395	106.775	83.101	132.340	1.00	0.00	H
ATOM	2552	HG12	VAL	C	395	106.902	83.400	130.625	1.00	0.00	H
ATOM	2553	HG13	VAL	C	395	105.369	83.679	131.454	1.00	0.00	H
ATOM	2554	HG21	VAL	C	395	105.783	81.130	133.263	1.00	0.00	H
ATOM	2555	HG22	VAL	C	395	104.189	81.468	132.588	1.00	0.00	H
ATOM	2556	HG23	VAL	C	395	104.964	79.912	132.305	1.00	0.00	H
ATOM	2557	N	LEU	C	396	106.512	81.547	127.998	1.00	30.00	N
ATOM	2558	CA	LEU	C	396	107.120	82.178	126.816	1.00	30.00	C
ATOM	2559	C	LEU	C	396	106.158	82.219	125.617	1.00	30.00	C
ATOM	2560	O	LEU	C	396	106.114	83.241	124.933	1.00	30.00	O
ATOM	2561	CB	LEU	C	396	108.450	81.504	126.407	1.00	20.00	C
ATOM	2562	CG	LEU	C	396	109.663	81.856	127.298	1.00	20.00	C
ATOM	2563	CD1	LEU	C	396	109.664	81.075	128.616	1.00	20.00	C
ATOM	2564	CD2	LEU	C	396	110.993	81.645	126.549	1.00	20.00	C
ATOM	2565	H	LEU	C	396	106.873	80.644	128.277	1.00	0.00	H
ATOM	2566	HA	LEU	C	396	107.338	83.217	127.072	1.00	0.00	H
ATOM	2567	HB2	LEU	C	396	108.329	80.424	126.317	1.00	0.00	H
ATOM	2568	HB3	LEU	C	396	108.683	81.854	125.401	1.00	0.00	H
ATOM	2569	HG	LEU	C	396	109.611	82.920	127.536	1.00	0.00	H
ATOM	2570	HD11	LEU	C	396	110.666	80.966	129.032	1.00	0.00	H
ATOM	2571	HD12	LEU	C	396	109.042	81.561	129.368	1.00	0.00	H
ATOM	2572	HD13	LEU	C	396	109.282	80.073	128.450	1.00	0.00	H
ATOM	2573	HD21	LEU	C	396	111.676	82.472	126.744	1.00	0.00	H
ATOM	2574	HD22	LEU	C	396	111.491	80.726	126.857	1.00	0.00	H
ATOM	2575	HD23	LEU	C	396	110.862	81.579	125.469	1.00	0.00	H
ATOM	2576	N	GLU	C	397	105.394	81.132	125.409	1.00	30.00	N
ATOM	2577	CA	GLU	C	397	104.335	81.012	124.404	1.00	30.00	C
ATOM	2578	C	GLU	C	397	103.225	82.073	124.551	1.00	30.00	C
ATOM	2579	O	GLU	C	397	102.797	82.636	123.545	1.00	30.00	O
ATOM	2580	CB	GLU	C	397	103.832	79.548	124.400	1.00	20.00	C
ATOM	2581	CG	GLU	C	397	102.493	79.265	123.691	1.00	0.00	C
ATOM	2582	CD	GLU	C	397	102.205	77.766	123.570	1.00	0.00	C
ATOM	2583	OE1	GLU	C	397	102.365	77.056	124.588	1.00	0.00	O
ATOM	2584	OE2	GLU	C	397	101.821	77.353	122.453	1.00	0.00	O1-
ATOM	2585	H	GLU	C	397	105.522	80.324	126.003	1.00	0.00	H
ATOM	2586	HA	GLU	C	397	104.796	81.193	123.431	1.00	0.00	H
ATOM	2587	HB2	GLU	C	397	104.596	78.943	123.915	1.00	0.00	H
ATOM	2588	HB3	GLU	C	397	103.775	79.176	125.424	1.00	0.00	H
ATOM	2589	HG2	GLU	C	397	101.667	79.726	124.232	1.00	0.00	H
ATOM	2590	HG3	GLU	C	397	102.506	79.703	122.696	1.00	0.00	H
ATOM	2591	N	VAL	C	398	102.826	82.360	125.800	1.00	30.00	N
ATOM	2592	CA	VAL	C	398	101.851	83.391	126.157	1.00	30.00	C
ATOM	2593	C	VAL	C	398	102.346	84.830	125.874	1.00	30.00	C
ATOM	2594	O	VAL	C	398	101.581	85.618	125.317	1.00	30.00	O
ATOM	2595	CB	VAL	C	398	101.416	83.236	127.647	1.00	20.00	C
ATOM	2596	CG1	VAL	C	398	100.763	84.473	128.294	1.00	20.00	C
ATOM	2597	CG2	VAL	C	398	100.477	82.027	127.817	1.00	20.00	C
ATOM	2598	H	VAL	C	398	103.220	81.838	126.572	1.00	0.00	H
ATOM	2599	HA	VAL	C	398	100.971	83.230	125.530	1.00	0.00	H
ATOM	2600	HB	VAL	C	398	102.309	83.019	128.233	1.00	0.00	H
ATOM	2601	HG11	VAL	C	398	100.406	84.243	129.296	1.00	0.00	H
ATOM	2602	HG12	VAL	C	398	101.467	85.298	128.397	1.00	0.00	H
ATOM	2603	HG13	VAL	C	398	99.913	84.824	127.708	1.00	0.00	H
ATOM	2604	HG21	VAL	C	398	100.267	81.835	128.869	1.00	0.00	H
ATOM	2605	HG22	VAL	C	398	99.525	82.195	127.314	1.00	0.00	H
ATOM	2606	HG23	VAL	C	398	100.906	81.116	127.401	1.00	0.00	H
ATOM	2607	N	ILE	C	399	103.601	85.144	126.245	1.00	30.00	N
ATOM	2608	CA	ILE	C	399	104.199	86.477	126.081	1.00	30.00	C
ATOM	2609	C	ILE	C	399	104.516	86.807	124.605	1.00	30.00	C
ATOM	2610	O	ILE	C	399	104.263	87.932	124.169	1.00	30.00	O
ATOM	2611	CB	ILE	C	399	105.497	86.645	126.937	1.00	20.00	C
ATOM	2612	CG1	ILE	C	399	105.188	86.532	128.448	1.00	20.00	C
ATOM	2613	CG2	ILE	C	399	106.282	87.952	126.668	1.00	20.00	C
ATOM	2614	CD1	ILE	C	399	106.407	86.167	129.309	1.00	20.00	C
ATOM	2615	H	ILE	C	399	104.176	84.446	126.698	1.00	0.00	H

ATOM	2616	HA	ILE	C	399	103.471	87.212	126.432	1.00	0.00	H
ATOM	2617	HB	ILE	C	399	106.158	85.814	126.684	1.00	0.00	H
ATOM	2618	HG12	ILE	C	399	104.746	87.461	128.810	1.00	0.00	H
ATOM	2619	HG13	ILE	C	399	104.430	85.770	128.618	1.00	0.00	H
ATOM	2620	HG21	ILE	C	399	107.101	88.082	127.373	1.00	0.00	H
ATOM	2621	HG22	ILE	C	399	106.728	87.973	125.673	1.00	0.00	H
ATOM	2622	HG23	ILE	C	399	105.636	88.826	126.762	1.00	0.00	H
ATOM	2623	HD11	ILE	C	399	106.097	85.908	130.321	1.00	0.00	H
ATOM	2624	HD12	ILE	C	399	106.941	85.309	128.899	1.00	0.00	H
ATOM	2625	HD13	ILE	C	399	107.113	86.993	129.386	1.00	0.00	H
ATOM	2626	N	ALA	C	400	105.053	85.819	123.869	1.00	30.00	N
ATOM	2627	CA	ALA	C	400	105.460	85.947	122.470	1.00	30.00	C
ATOM	2628	C	ALA	C	400	104.286	86.035	121.482	1.00	30.00	C
ATOM	2629	O	ALA	C	400	104.448	86.676	120.444	1.00	30.00	O
ATOM	2630	CB	ALA	C	400	106.374	84.770	122.102	1.00	20.00	C
ATOM	2631	H	ALA	C	400	105.230	84.920	124.299	1.00	0.00	H
ATOM	2632	HA	ALA	C	400	106.043	86.866	122.372	1.00	0.00	H
ATOM	2633	HB1	ALA	C	400	106.727	84.843	121.073	1.00	0.00	H
ATOM	2634	HB2	ALA	C	400	107.254	84.741	122.745	1.00	0.00	H
ATOM	2635	HB3	ALA	C	400	105.853	83.816	122.205	1.00	0.00	H
ATOM	2636	N	TYR	C	401	103.145	85.398	121.806	1.00	30.00	N
ATOM	2637	CA	TYR	C	401	101.958	85.332	120.942	1.00	30.00	C
ATOM	2638	C	TYR	C	401	100.803	86.213	121.472	1.00	30.00	C
ATOM	2639	O	TYR	C	401	99.649	85.963	121.120	1.00	30.00	O
ATOM	2640	CB	TYR	C	401	101.520	83.855	120.741	1.00	20.00	C
ATOM	2641	CG	TYR	C	401	102.559	82.838	120.263	1.00	20.00	C
ATOM	2642	CD1	TYR	C	401	103.726	83.219	119.562	1.00	20.00	C
ATOM	2643	CD2	TYR	C	401	102.338	81.468	120.519	1.00	20.00	C
ATOM	2644	CE1	TYR	C	401	104.668	82.254	119.158	1.00	20.00	C
ATOM	2645	CE2	TYR	C	401	103.272	80.498	120.102	1.00	20.00	C
ATOM	2646	CZ	TYR	C	401	104.440	80.890	119.422	1.00	20.00	C
ATOM	2647	OH	TYR	C	401	105.337	79.949	119.003	1.00	20.00	O
ATOM	2648	H	TYR	C	401	103.093	84.876	122.671	1.00	0.00	H
ATOM	2649	HA	TYR	C	401	102.189	85.733	119.954	1.00	0.00	H
ATOM	2650	HB2	TYR	C	401	101.111	83.484	121.682	1.00	0.00	H
ATOM	2651	HB3	TYR	C	401	100.696	83.815	120.027	1.00	0.00	H
ATOM	2652	HD1	TYR	C	401	103.914	84.254	119.322	1.00	0.00	H
ATOM	2653	HD2	TYR	C	401	101.448	81.154	121.045	1.00	0.00	H
ATOM	2654	HE1	TYR	C	401	105.559	82.562	118.630	1.00	0.00	H
ATOM	2655	HE2	TYR	C	401	103.088	79.453	120.306	1.00	0.00	H
ATOM	2656	HH	TYR	C	401	105.995	80.298	118.387	1.00	0.00	H
ATOM	2657	N	SER	C	402	101.121	87.236	122.288	1.00	30.00	N
ATOM	2658	CA	SER	C	402	100.180	88.232	122.817	1.00	30.00	C
ATOM	2659	C	SER	C	402	99.560	89.137	121.730	1.00	30.00	C
ATOM	2660	O	SER	C	402	100.138	89.296	120.655	1.00	30.00	O
ATOM	2661	CB	SER	C	402	100.897	89.068	123.901	1.00	20.00	C
ATOM	2662	OG	SER	C	402	100.731	88.477	125.173	1.00	20.00	O
ATOM	2663	H	SER	C	402	102.089	87.370	122.541	1.00	0.00	H
ATOM	2664	HA	SER	C	402	99.350	87.692	123.278	1.00	0.00	H
ATOM	2665	HB2	SER	C	402	101.957	89.185	123.685	1.00	0.00	H
ATOM	2666	HB3	SER	C	402	100.484	90.077	123.968	1.00	0.00	H
ATOM	2667	HG	SER	C	402	101.133	87.602	125.164	1.00	0.00	H
ATOM	2668	N	SER	C	403	98.389	89.718	122.047	1.00	30.00	N
ATOM	2669	CA	SER	C	403	97.570	90.552	121.155	1.00	30.00	C
ATOM	2670	C	SER	C	403	98.154	91.936	120.791	1.00	30.00	C
ATOM	2671	O	SER	C	403	97.613	92.581	119.894	1.00	30.00	O
ATOM	2672	CB	SER	C	403	96.166	90.690	121.782	1.00	20.00	C
ATOM	2673	OG	SER	C	403	96.181	91.501	122.944	1.00	0.00	O
ATOM	2674	H	SER	C	403	97.990	89.551	122.959	1.00	0.00	H
ATOM	2675	HA	SER	C	403	97.461	90.002	120.218	1.00	0.00	H
ATOM	2676	HB2	SER	C	403	95.476	91.138	121.066	1.00	0.00	H
ATOM	2677	HB3	SER	C	403	95.757	89.710	122.033	1.00	0.00	H
ATOM	2678	HG	SER	C	403	95.281	91.580	123.272	1.00	0.00	H
ATOM	2679	N	SER	C	404	99.211	92.370	121.502	1.00	30.00	N
ATOM	2680	CA	SER	C	404	99.872	93.681	121.420	1.00	30.00	C
ATOM	2681	C	SER	C	404	99.122	94.814	122.159	1.00	30.00	C
ATOM	2682	O	SER	C	404	99.667	95.913	122.264	1.00	30.00	O
ATOM	2683	CB	SER	C	404	100.251	94.046	119.962	1.00	20.00	C
ATOM	2684	OG	SER	C	404	101.249	95.046	119.927	1.00	0.00	O
ATOM	2685	H	SER	C	404	99.580	91.752	122.211	1.00	0.00	H
ATOM	2686	HA	SER	C	404	100.801	93.549	121.970	1.00	0.00	H

```
ATOM   2687  HB2 SER C 404     100.630  93.175 119.429  1.00  0.00           H
ATOM   2688  HB3 SER C 404      99.386  94.406 119.405  1.00  0.00           H
ATOM   2689  HG  SER C 404     100.893  95.843 120.331  1.00  0.00           H
ATOM   2690  N   GLU C 405      97.919  94.528 122.686  1.00 30.00           N
ATOM   2691  CA  GLU C 405      97.107  95.444 123.496  1.00 30.00           C
ATOM   2692  C   GLU C 405      97.464  95.381 124.997  1.00 30.00           C
ATOM   2693  O   GLU C 405      97.037  96.252 125.753  1.00 30.00           O
ATOM   2694  CB  GLU C 405      95.615  95.131 123.257  1.00 20.00           C
ATOM   2695  CG  GLU C 405      95.199  95.273 121.776  1.00  0.00           C
ATOM   2696  CD  GLU C 405      93.727  94.945 121.528  1.00  0.00           C
ATOM   2697  OE1 GLU C 405      92.875  95.444 122.294  1.00  0.00           O
ATOM   2698  OE2 GLU C 405      93.467  94.217 120.545  1.00  0.00           O1-
ATOM   2699  H   GLU C 405      97.528  93.607 122.545  1.00  0.00           H
ATOM   2700  HA  GLU C 405      97.289  96.468 123.167  1.00  0.00           H
ATOM   2701  HB2 GLU C 405      95.388  94.123 123.606  1.00  0.00           H
ATOM   2702  HB3 GLU C 405      95.006  95.802 123.867  1.00  0.00           H
ATOM   2703  HG2 GLU C 405      95.384  96.291 121.434  1.00  0.00           H
ATOM   2704  HG3 GLU C 405      95.803  94.619 121.146  1.00  0.00           H
ATOM   2705  N   THR C 406      98.263  94.373 125.390  1.00 30.00           N
ATOM   2706  CA  THR C 406      98.872  94.201 126.710  1.00 30.00           C
ATOM   2707  C   THR C 406      99.923  95.313 126.985  1.00 30.00           C
ATOM   2708  O   THR C 406     100.617  95.716 126.049  1.00 30.00           O
ATOM   2709  CB  THR C 406      99.610  92.835 126.748  1.00 20.00           C
ATOM   2710  CG2 THR C 406      98.670  91.636 126.562  1.00 20.00           C
ATOM   2711  OG1 THR C 406     100.608  92.756 125.748  1.00 20.00           O
ATOM   2712  H   THR C 406      98.570  93.709 124.694  1.00  0.00           H
ATOM   2713  HA  THR C 406      98.065  94.211 127.441  1.00  0.00           H
ATOM   2714  HB  THR C 406     100.109  92.723 127.712  1.00  0.00           H
ATOM   2715  HG1 THR C 406     101.195  93.511 125.845  1.00  0.00           H
ATOM   2716 HG21 THR C 406      99.211  90.695 126.664  1.00  0.00           H
ATOM   2717 HG22 THR C 406      97.879  91.646 127.311  1.00  0.00           H
ATOM   2718 HG23 THR C 406      98.197  91.637 125.579  1.00  0.00           H
ATOM   2719  N   PRO C 407     100.003  95.807 128.243  1.00 30.00           N
ATOM   2720  CA  PRO C 407     100.771  97.020 128.587  1.00 30.00           C
ATOM   2721  C   PRO C 407     102.289  96.972 128.319  1.00 30.00           C
ATOM   2722  O   PRO C 407     102.795  97.912 127.707  1.00 30.00           O
ATOM   2723  CB  PRO C 407     100.435  97.269 130.071  1.00 20.00           C
ATOM   2724  CG  PRO C 407      99.997  95.915 130.605  1.00  0.00           C
ATOM   2725  CD  PRO C 407      99.279  95.303 129.410  1.00  0.00           C
ATOM   2726  HA  PRO C 407     100.369  97.851 128.003  1.00  0.00           H
ATOM   2727  HB2 PRO C 407     101.258  97.693 130.649  1.00  0.00           H
ATOM   2728  HB3 PRO C 407      99.604  97.971 130.138  1.00  0.00           H
ATOM   2729  HG2 PRO C 407     100.882  95.327 130.846  1.00  0.00           H
ATOM   2730  HG3 PRO C 407      99.379  95.974 131.501  1.00  0.00           H
ATOM   2731  HD2 PRO C 407      99.265  94.216 129.466  1.00  0.00           H
ATOM   2732  HD3 PRO C 407      98.248  95.656 129.365  1.00  0.00           H
ATOM   2733  N   ASN C 408     102.989  95.918 128.780  1.00 30.00           N
ATOM   2734  CA  ASN C 408     104.458  95.823 128.735  1.00 30.00           C
ATOM   2735  C   ASN C 408     104.899  94.384 128.396  1.00 30.00           C
ATOM   2736  O   ASN C 408     105.601  93.758 129.191  1.00 30.00           O
ATOM   2737  CB  ASN C 408     105.076  96.327 130.072  1.00 20.00           C
ATOM   2738  CG  ASN C 408     104.905  97.825 130.345  1.00  0.00           C
ATOM   2739  ND2 ASN C 408     105.894  98.636 129.965  1.00  0.00           N
ATOM   2740  OD1 ASN C 408     103.899  98.246 130.913  1.00  0.00           O
ATOM   2741  H   ASN C 408     102.511  95.181 129.278  1.00  0.00           H
ATOM   2742  HA  ASN C 408     104.831  96.457 127.928  1.00  0.00           H
ATOM   2743  HB2 ASN C 408     104.658  95.770 130.911  1.00  0.00           H
ATOM   2744  HB3 ASN C 408     106.148  96.129 130.079  1.00  0.00           H
ATOM   2745 HD21 ASN C 408     105.821  99.630 130.120  1.00  0.00           H
ATOM   2746 HD22 ASN C 408     106.745  98.262 129.554  1.00  0.00           H
ATOM   2747  N   ARG C 409     104.512  93.886 127.206  1.00 30.00           N
ATOM   2748  CA  ARG C 409     104.942  92.575 126.688  1.00 30.00           C
ATOM   2749  C   ARG C 409     106.425  92.509 126.296  1.00 30.00           C
ATOM   2750  O   ARG C 409     107.042  91.454 126.445  1.00 30.00           O
ATOM   2751  CB  ARG C 409     104.038  92.125 125.526  1.00 20.00           C
ATOM   2752  CG  ARG C 409     104.096  93.015 124.268  1.00 20.00           C
ATOM   2753  CD  ARG C 409     103.157  92.551 123.154  1.00 20.00           C
ATOM   2754  NE  ARG C 409     103.521  91.221 122.650  1.00 20.00           N
ATOM   2755  CZ  ARG C 409     103.173  90.721 121.454  1.00 20.00           C
ATOM   2756  NH1 ARG C 409     102.489  91.451 120.569  1.00 20.00           N
ATOM   2757  NH2 ARG C 409     103.517  89.471 121.132  1.00 20.00           N1+
```

```
ATOM   2758  H   ARG C 409     103.930  94.444 126.599  1.00  0.00           H
ATOM   2759  HA  ARG C 409     104.822  91.846 127.480  1.00  0.00           H
ATOM   2760  HB2 ARG C 409     103.016  92.070 125.891  1.00  0.00           H
ATOM   2761  HB3 ARG C 409     104.304  91.101 125.259  1.00  0.00           H
ATOM   2762  HG2 ARG C 409     103.852  94.047 124.522  1.00  0.00           H
ATOM   2763  HG3 ARG C 409     105.109  93.039 123.870  1.00  0.00           H
ATOM   2764  HD2 ARG C 409     103.203  93.283 122.347  1.00  0.00           H
ATOM   2765  HD3 ARG C 409     102.125  92.515 123.501  1.00  0.00           H
ATOM   2766  HE  ARG C 409     104.007  90.616 123.297  1.00  0.00           H
ATOM   2767 HH11 ARG C 409     102.261  91.052 119.659  1.00  0.00           H
ATOM   2768 HH12 ARG C 409     102.251  92.412 120.762  1.00  0.00           H
ATOM   2769 HH21 ARG C 409     103.257  89.098 120.228  1.00  0.00           H
ATOM   2770 HH22 ARG C 409     104.050  88.897 121.769  1.00  0.00           H
ATOM   2771  N   HIS C 410     106.963  93.641 125.819  1.00 30.00           N
ATOM   2772  CA  HIS C 410     108.346  93.794 125.379  1.00 30.00           C
ATOM   2773  C   HIS C 410     109.353  93.913 126.543  1.00 30.00           C
ATOM   2774  O   HIS C 410     110.554  93.926 126.276  1.00 30.00           O
ATOM   2775  CB  HIS C 410     108.413  95.002 124.423  1.00 20.00           C
ATOM   2776  CG  HIS C 410     107.461  94.953 123.251  1.00 20.00           C
ATOM   2777  CD2 HIS C 410     106.389  95.747 122.905  1.00 20.00           C
ATOM   2778  ND1 HIS C 410     107.543  93.991 122.260  1.00 20.00           N
ATOM   2779  CE1 HIS C 410     106.553  94.225 121.395  1.00 20.00           C
ATOM   2780  NE2 HIS C 410     105.810  95.274 121.725  1.00 20.00           N
ATOM   2781  H   HIS C 410     106.383  94.464 125.736  1.00  0.00           H
ATOM   2782  HA  HIS C 410     108.628  92.905 124.813  1.00  0.00           H
ATOM   2783  HB2 HIS C 410     109.407  95.060 123.995  1.00  0.00           H
ATOM   2784  HB3 HIS C 410     108.250  95.932 124.970  1.00  0.00           H
ATOM   2785  HD1 HIS C 410     108.223  93.246 122.202  1.00  0.00           H
ATOM   2786  HD2 HIS C 410     105.987  96.612 123.413  1.00  0.00           H
ATOM   2787  HE1 HIS C 410     106.373  93.621 120.518  1.00  0.00           H
ATOM   2788  N   ASP C 411     108.851  93.983 127.792  1.00 30.00           N
ATOM   2789  CA  ASP C 411     109.623  94.138 129.032  1.00 30.00           C
ATOM   2790  C   ASP C 411     109.486  92.931 129.983  1.00 30.00           C
ATOM   2791  O   ASP C 411     110.271  92.850 130.927  1.00 30.00           O
ATOM   2792  CB  ASP C 411     109.283  95.445 129.791  1.00 20.00           C
ATOM   2793  CG  ASP C 411     109.285  96.716 128.932  1.00 20.00           C
ATOM   2794  OD1 ASP C 411     110.138  96.808 128.021  1.00 20.00           O
ATOM   2795  OD2 ASP C 411     108.486  97.618 129.264  1.00 20.00           O1-
ATOM   2796  H   ASP C 411     107.848  93.973 127.908  1.00  0.00           H
ATOM   2797  HA  ASP C 411     110.684  94.189 128.782  1.00  0.00           H
ATOM   2798  HB2 ASP C 411     108.292  95.335 130.231  1.00  0.00           H
ATOM   2799  HB3 ASP C 411     109.980  95.598 130.616  1.00  0.00           H
ATOM   2800  N   MET C 412     108.546  91.995 129.738  1.00 30.00           N
ATOM   2801  CA  MET C 412     108.457  90.732 130.492  1.00 30.00           C
ATOM   2802  C   MET C 412     109.579  89.737 130.141  1.00 30.00           C
ATOM   2803  O   MET C 412     109.933  88.919 130.990  1.00 30.00           O
ATOM   2804  CB  MET C 412     107.077  90.070 130.303  1.00 20.00           C
ATOM   2805  CG  MET C 412     105.974  90.724 131.145  1.00 20.00           C
ATOM   2806  SD  MET C 412     104.376  89.879 131.088  1.00 20.00           S
ATOM   2807  CE  MET C 412     103.877  90.330 129.412  1.00 20.00           C
ATOM   2808  H   MET C 412     107.911  92.107 128.960  1.00  0.00           H
ATOM   2809  HA  MET C 412     108.575  90.957 131.554  1.00  0.00           H
ATOM   2810  HB2 MET C 412     106.807  90.076 129.247  1.00  0.00           H
ATOM   2811  HB3 MET C 412     107.126  89.020 130.594  1.00  0.00           H
ATOM   2812  HG2 MET C 412     106.283  90.749 132.191  1.00  0.00           H
ATOM   2813  HG3 MET C 412     105.827  91.757 130.836  1.00  0.00           H
ATOM   2814  HE1 MET C 412     102.876  89.976 129.185  1.00  0.00           H
ATOM   2815  HE2 MET C 412     103.864  91.416 129.332  1.00  0.00           H
ATOM   2816  HE3 MET C 412     104.562  89.914 128.676  1.00  0.00           H
ATOM   2817  N   LEU C 413     110.135  89.843 128.922  1.00 30.00           N
ATOM   2818  CA  LEU C 413     111.276  89.054 128.450  1.00 30.00           C
ATOM   2819  C   LEU C 413     112.623  89.520 129.041  1.00 30.00           C
ATOM   2820  O   LEU C 413     113.597  88.777 128.931  1.00 30.00           O
ATOM   2821  CB  LEU C 413     111.316  89.104 126.904  1.00 20.00           C
ATOM   2822  CG  LEU C 413     110.079  88.490 126.207  1.00 20.00           C
ATOM   2823  CD1 LEU C 413     110.057  88.848 124.708  1.00 20.00           C
ATOM   2824  CD2 LEU C 413     109.960  86.968 126.457  1.00 20.00           C
ATOM   2825  H   LEU C 413     109.780  90.537 128.280  1.00  0.00           H
ATOM   2826  HA  LEU C 413     111.136  88.020 128.770  1.00  0.00           H
ATOM   2827  HB2 LEU C 413     111.418  90.147 126.596  1.00  0.00           H
ATOM   2828  HB3 LEU C 413     112.209  88.597 126.537  1.00  0.00           H
```

```
ATOM   2829  HG  LEU C 413     109.189  88.955 126.633  1.00  0.00           H
ATOM   2830 HD11 LEU C 413     109.859  87.990 124.067  1.00  0.00           H
ATOM   2831 HD12 LEU C 413     109.276  89.580 124.504  1.00  0.00           H
ATOM   2832 HD13 LEU C 413     111.001  89.285 124.378  1.00  0.00           H
ATOM   2833 HD21 LEU C 413     109.874  86.389 125.538  1.00  0.00           H
ATOM   2834 HD22 LEU C 413     110.822  86.573 126.993  1.00  0.00           H
ATOM   2835 HD23 LEU C 413     109.080  86.741 127.059  1.00  0.00           H
ATOM   2836  N   LEU C 414     112.657  90.719 129.651  1.00 30.00           N
ATOM   2837  CA  LEU C 414     113.834  91.314 130.292  1.00 30.00           C
ATOM   2838  C   LEU C 414     114.061  90.802 131.729  1.00 30.00           C
ATOM   2839  O   LEU C 414     115.141  91.043 132.267  1.00 30.00           O
ATOM   2840  CB  LEU C 414     113.733  92.860 130.254  1.00 20.00           C
ATOM   2841  CG  LEU C 414     114.164  93.518 128.922  1.00 20.00           C
ATOM   2842  CD1 LEU C 414     113.366  93.043 127.692  1.00 20.00           C
ATOM   2843  CD2 LEU C 414     114.135  95.055 129.044  1.00 20.00           C
ATOM   2844  H   LEU C 414     111.809  91.265 129.706  1.00  0.00           H
ATOM   2845  HA  LEU C 414     114.720  91.031 129.728  1.00  0.00           H
ATOM   2846  HB2 LEU C 414     112.728  93.181 130.522  1.00  0.00           H
ATOM   2847  HB3 LEU C 414     114.379  93.273 131.031  1.00  0.00           H
ATOM   2848  HG  LEU C 414     115.205  93.239 128.755  1.00  0.00           H
ATOM   2849 HD11 LEU C 414     113.865  92.204 127.206  1.00  0.00           H
ATOM   2850 HD12 LEU C 414     112.361  92.719 127.957  1.00  0.00           H
ATOM   2851 HD13 LEU C 414     113.264  93.828 126.942  1.00  0.00           H
ATOM   2852 HD21 LEU C 414     115.033  95.499 128.613  1.00  0.00           H
ATOM   2853 HD22 LEU C 414     113.273  95.488 128.535  1.00  0.00           H
ATOM   2854 HD23 LEU C 414     114.079  95.385 130.083  1.00  0.00           H
ATOM   2855  N   VAL C 415     113.078  90.095 132.318  1.00 30.00           N
ATOM   2856  CA  VAL C 415     113.170  89.483 133.647  1.00 30.00           C
ATOM   2857  C   VAL C 415     114.180  88.312 133.632  1.00 30.00           C
ATOM   2858  O   VAL C 415     114.153  87.502 132.704  1.00 30.00           O
ATOM   2859  CB  VAL C 415     111.771  89.001 134.131  1.00 20.00           C
ATOM   2860  CG1 VAL C 415     111.769  88.307 135.508  1.00 20.00           C
ATOM   2861  CG2 VAL C 415     110.780  90.181 134.178  1.00 20.00           C
ATOM   2862  H   VAL C 415     112.221  89.924 131.811  1.00  0.00           H
ATOM   2863  HA  VAL C 415     113.530  90.245 134.341  1.00  0.00           H
ATOM   2864  HB  VAL C 415     111.387  88.278 133.408  1.00  0.00           H
ATOM   2865 HG11 VAL C 415     110.762  87.990 135.776  1.00  0.00           H
ATOM   2866 HG12 VAL C 415     112.384  87.415 135.530  1.00  0.00           H
ATOM   2867 HG13 VAL C 415     112.128  88.976 136.290  1.00  0.00           H
ATOM   2868 HG21 VAL C 415     109.822  89.882 134.597  1.00  0.00           H
ATOM   2869 HG22 VAL C 415     111.163  90.992 134.799  1.00  0.00           H
ATOM   2870 HG23 VAL C 415     110.586  90.589 133.186  1.00  0.00           H
ATOM   2871  N   GLU C 416     115.080  88.301 134.633  1.00 30.00           N
ATOM   2872  CA  GLU C 416     116.375  87.603 134.652  1.00 30.00           C
ATOM   2873  C   GLU C 416     116.443  86.125 134.195  1.00 30.00           C
ATOM   2874  O   GLU C 416     117.363  85.818 133.433  1.00 30.00           O
ATOM   2875  CB  GLU C 416     117.049  87.768 136.039  1.00 20.00           C
ATOM   2876  CG  GLU C 416     117.290  89.222 136.496  1.00  0.00           C
ATOM   2877  CD  GLU C 416     118.308  89.958 135.626  1.00  0.00           C
ATOM   2878  OE1 GLU C 416     119.515  89.694 135.824  1.00  0.00           O
ATOM   2879  OE2 GLU C 416     117.865  90.754 134.773  1.00  0.00           O1-
ATOM   2880  H   GLU C 416     114.979  88.993 135.361  1.00  0.00           H
ATOM   2881  HA  GLU C 416     116.984  88.146 133.927  1.00  0.00           H
ATOM   2882  HB2 GLU C 416     116.453  87.274 136.804  1.00  0.00           H
ATOM   2883  HB3 GLU C 416     118.002  87.237 136.047  1.00  0.00           H
ATOM   2884  HG2 GLU C 416     116.356  89.783 136.519  1.00  0.00           H
ATOM   2885  HG3 GLU C 416     117.661  89.220 137.522  1.00  0.00           H
ATOM   2886  N   PRO C 417     115.502  85.237 134.608  1.00 30.00           N
ATOM   2887  CA  PRO C 417     115.531  83.824 134.185  1.00 30.00           C
ATOM   2888  C   PRO C 417     115.182  83.613 132.704  1.00 30.00           C
ATOM   2889  O   PRO C 417     115.754  82.713 132.096  1.00 30.00           O
ATOM   2890  CB  PRO C 417     114.522  83.117 135.113  1.00 20.00           C
ATOM   2891  CG  PRO C 417     114.296  84.091 136.255  1.00 20.00           C
ATOM   2892  CD  PRO C 417     114.425  85.439 135.572  1.00 20.00           C
ATOM   2893  HA  PRO C 417     116.530  83.430 134.385  1.00  0.00           H
ATOM   2894  HB2 PRO C 417     113.573  82.942 134.603  1.00  0.00           H
ATOM   2895  HB3 PRO C 417     114.884  82.151 135.467  1.00  0.00           H
ATOM   2896  HG2 PRO C 417     113.346  83.948 136.765  1.00  0.00           H
ATOM   2897  HG3 PRO C 417     115.093  83.991 136.989  1.00  0.00           H
ATOM   2898  HD2 PRO C 417     114.588  86.233 136.298  1.00  0.00           H
ATOM   2899  HD3 PRO C 417     113.516  85.654 135.013  1.00  0.00           H
```

```
ATOM   2900  N   LEU C 418     114.270  84.440 132.162  1.00 30.00           N
ATOM   2901  CA  LEU C 418     113.819  84.393 130.769  1.00 30.00           C
ATOM   2902  C   LEU C 418     114.826  85.040 129.805  1.00 30.00           C
ATOM   2903  O   LEU C 418     114.994  84.537 128.694  1.00 30.00           O
ATOM   2904  CB  LEU C 418     112.447  85.097 130.629  1.00 20.00           C
ATOM   2905  CG  LEU C 418     111.311  84.546 131.522  1.00 20.00           C
ATOM   2906  CD1 LEU C 418     110.018  85.369 131.328  1.00 20.00           C
ATOM   2907  CD2 LEU C 418     111.074  83.035 131.322  1.00 20.00           C
ATOM   2908  H   LEU C 418     113.864  85.171 132.730  1.00  0.00           H
ATOM   2909  HA  LEU C 418     113.711  83.347 130.477  1.00  0.00           H
ATOM   2910  HB2 LEU C 418     112.127  85.041 129.586  1.00  0.00           H
ATOM   2911  HB3 LEU C 418     112.568  86.161 130.842  1.00  0.00           H
ATOM   2912  HG  LEU C 418     111.610  84.688 132.562  1.00  0.00           H
ATOM   2913 HD11 LEU C 418     109.676  85.784 132.277  1.00  0.00           H
ATOM   2914 HD12 LEU C 418     110.168  86.209 130.650  1.00  0.00           H
ATOM   2915 HD13 LEU C 418     109.196  84.782 130.917  1.00  0.00           H
ATOM   2916 HD21 LEU C 418     110.015  82.781 131.285  1.00  0.00           H
ATOM   2917 HD22 LEU C 418     111.523  82.672 130.398  1.00  0.00           H
ATOM   2918 HD23 LEU C 418     111.508  82.464 132.144  1.00  0.00           H
ATOM   2919  N   ASN C 419     115.464  86.140 130.242  1.00 30.00           N
ATOM   2920  CA  ASN C 419     116.347  86.977 129.429  1.00 30.00           C
ATOM   2921  C   ASN C 419     117.629  86.254 128.977  1.00 30.00           C
ATOM   2922  O   ASN C 419     117.970  86.325 127.796  1.00 30.00           O
ATOM   2923  CB  ASN C 419     116.662  88.282 130.201  1.00 20.00           C
ATOM   2924  CG  ASN C 419     117.198  89.423 129.328  1.00 20.00           C
ATOM   2925  ND2 ASN C 419     116.500  89.757 128.240  1.00 20.00           N
ATOM   2926  OD1 ASN C 419     118.229  90.011 129.640  1.00 20.00           O
ATOM   2927  H   ASN C 419     115.257  86.488 131.169  1.00  0.00           H
ATOM   2928  HA  ASN C 419     115.817  87.223 128.507  1.00  0.00           H
ATOM   2929  HB2 ASN C 419     115.746  88.657 130.657  1.00  0.00           H
ATOM   2930  HB3 ASN C 419     117.345  88.089 131.031  1.00  0.00           H
ATOM   2931 HD21 ASN C 419     116.820  90.505 127.646  1.00  0.00           H
ATOM   2932 HD22 ASN C 419     115.632  89.284 128.028  1.00  0.00           H
ATOM   2933  N   ARG C 420     118.284  85.543 129.910  1.00 30.00           N
ATOM   2934  CA  ARG C 420     119.475  84.737 129.629  1.00 30.00           C
ATOM   2935  C   ARG C 420     119.145  83.337 129.081  1.00 30.00           C
ATOM   2936  O   ARG C 420     120.048  82.689 128.556  1.00 30.00           O
ATOM   2937  CB  ARG C 420     120.356  84.650 130.891  1.00 20.00           C
ATOM   2938  CG  ARG C 420     120.899  86.015 131.342  1.00 20.00           C
ATOM   2939  CD  ARG C 420     121.961  85.899 132.443  1.00 20.00           C
ATOM   2940  NE  ARG C 420     122.308  87.215 132.991  1.00 20.00           N
ATOM   2941  CZ  ARG C 420     121.576  87.878 133.901  1.00 20.00           C
ATOM   2942  NH1 ARG C 420     121.934  89.112 134.271  1.00 20.00           N
ATOM   2943  NH2 ARG C 420     120.481  87.335 134.449  1.00 20.00           N1+
ATOM   2944  H   ARG C 420     117.945  85.535 130.862  1.00  0.00           H
ATOM   2945  HA  ARG C 420     120.061  85.244 128.860  1.00  0.00           H
ATOM   2946  HB2 ARG C 420     119.802  84.189 131.710  1.00  0.00           H
ATOM   2947  HB3 ARG C 420     121.208  83.999 130.691  1.00  0.00           H
ATOM   2948  HG2 ARG C 420     121.395  86.441 130.468  1.00  0.00           H
ATOM   2949  HG3 ARG C 420     120.111  86.720 131.613  1.00  0.00           H
ATOM   2950  HD2 ARG C 420     121.725  85.157 133.203  1.00  0.00           H
ATOM   2951  HD3 ARG C 420     122.887  85.571 131.969  1.00  0.00           H
ATOM   2952  HE  ARG C 420     123.117  87.668 132.591  1.00  0.00           H
ATOM   2953 HH11 ARG C 420     121.339  89.616 134.922  1.00  0.00           H
ATOM   2954 HH12 ARG C 420     122.757  89.557 133.894  1.00  0.00           H
ATOM   2955 HH21 ARG C 420     119.938  87.888 135.104  1.00  0.00           H
ATOM   2956 HH22 ARG C 420     120.201  86.389 134.226  1.00  0.00           H
ATOM   2957  N   LEU C 421     117.884  82.885 129.208  1.00 30.00           N
ATOM   2958  CA  LEU C 421     117.404  81.605 128.677  1.00 30.00           C
ATOM   2959  C   LEU C 421     117.197  81.656 127.158  1.00 30.00           C
ATOM   2960  O   LEU C 421     117.575  80.707 126.474  1.00 30.00           O
ATOM   2961  CB  LEU C 421     116.146  81.175 129.460  1.00 20.00           C
ATOM   2962  CG  LEU C 421     115.349  79.974 128.907  1.00 20.00           C
ATOM   2963  CD1 LEU C 421     114.730  79.144 130.045  1.00 20.00           C
ATOM   2964  CD2 LEU C 421     114.286  80.382 127.866  1.00 20.00           C
ATOM   2965  H   LEU C 421     117.186  83.476 129.637  1.00  0.00           H
ATOM   2966  HA  LEU C 421     118.171  80.850 128.866  1.00  0.00           H
ATOM   2967  HB2 LEU C 421     116.494  80.929 130.461  1.00  0.00           H
ATOM   2968  HB3 LEU C 421     115.469  82.019 129.587  1.00  0.00           H
ATOM   2969  HG  LEU C 421     116.068  79.322 128.415  1.00  0.00           H
ATOM   2970 HD11 LEU C 421     114.683  78.093 129.761  1.00  0.00           H
```

```
ATOM   2971 HD12 LEU C 421     115.305  79.192 130.970  1.00  0.00           H
ATOM   2972 HD13 LEU C 421     113.720  79.475 130.284  1.00  0.00           H
ATOM   2973 HD21 LEU C 421     114.545  80.002 126.879  1.00  0.00           H
ATOM   2974 HD22 LEU C 421     113.300  79.994 128.113  1.00  0.00           H
ATOM   2975 HD23 LEU C 421     114.187  81.464 127.777  1.00  0.00           H
ATOM   2976  N   LEU C 422     116.634  82.772 126.665  1.00 30.00           N
ATOM   2977  CA  LEU C 422     116.482  83.064 125.240  1.00 30.00           C
ATOM   2978  C   LEU C 422     117.840  83.309 124.557  1.00 30.00           C
ATOM   2979  O   LEU C 422     118.003  82.910 123.407  1.00 30.00           O
ATOM   2980  CB  LEU C 422     115.537  84.273 125.065  1.00 20.00           C
ATOM   2981  CG  LEU C 422     114.064  83.983 125.439  1.00 20.00           C
ATOM   2982  CD1 LEU C 422     113.262  85.292 125.594  1.00 20.00           C
ATOM   2983  CD2 LEU C 422     113.393  83.002 124.455  1.00 20.00           C
ATOM   2984  H   LEU C 422     116.325  83.495 127.301  1.00  0.00           H
ATOM   2985  HA  LEU C 422     116.030  82.194 124.760  1.00  0.00           H
ATOM   2986  HB2 LEU C 422     115.913  85.093 125.681  1.00  0.00           H
ATOM   2987  HB3 LEU C 422     115.574  84.638 124.037  1.00  0.00           H
ATOM   2988  HG  LEU C 422     114.049  83.505 126.419  1.00  0.00           H
ATOM   2989 HD11 LEU C 422     112.354  85.309 124.992  1.00  0.00           H
ATOM   2990 HD12 LEU C 422     112.961  85.434 126.632  1.00  0.00           H
ATOM   2991 HD13 LEU C 422     113.845  86.168 125.308  1.00  0.00           H
ATOM   2992 HD21 LEU C 422     112.343  83.232 124.276  1.00  0.00           H
ATOM   2993 HD22 LEU C 422     113.880  83.008 123.483  1.00  0.00           H
ATOM   2994 HD23 LEU C 422     113.436  81.981 124.836  1.00  0.00           H
ATOM   2995  N   GLN C 423     118.802  83.909 125.283  1.00 30.00           N
ATOM   2996  CA  GLN C 423     120.170  84.125 124.808  1.00 30.00           C
ATOM   2997  C   GLN C 423     120.976  82.813 124.717  1.00 30.00           C
ATOM   2998  O   GLN C 423     121.722  82.644 123.753  1.00 30.00           O
ATOM   2999  CB  GLN C 423     120.858  85.174 125.711  1.00 20.00           C
ATOM   3000  CG  GLN C 423     122.251  85.648 125.239  1.00 20.00           C
ATOM   3001  CD  GLN C 423     122.235  86.310 123.855  1.00 20.00           C
ATOM   3002  NE2 GLN C 423     123.192  85.956 122.997  1.00 20.00           N
ATOM   3003  OE1 GLN C 423     121.378  87.143 123.566  1.00 20.00           O
ATOM   3004  H   GLN C 423     118.600  84.215 126.224  1.00  0.00           H
ATOM   3005  HA  GLN C 423     120.094  84.532 123.797  1.00  0.00           H
ATOM   3006  HB2 GLN C 423     120.207  86.046 125.802  1.00  0.00           H
ATOM   3007  HB3 GLN C 423     120.950  84.774 126.720  1.00  0.00           H
ATOM   3008  HG2 GLN C 423     122.955  84.815 125.242  1.00  0.00           H
ATOM   3009  HG3 GLN C 423     122.643  86.375 125.951  1.00  0.00           H
ATOM   3010 HE21 GLN C 423     123.239  86.371 122.080  1.00  0.00           H
ATOM   3011 HE22 GLN C 423     123.868  85.236 123.236  1.00  0.00           H
ATOM   3012  N   ASP C 424     120.775  81.902 125.690  1.00 30.00           N
ATOM   3013  CA  ASP C 424     121.335  80.545 125.720  1.00 30.00           C
ATOM   3014  C   ASP C 424     120.770  79.712 124.555  1.00 30.00           C
ATOM   3015  O   ASP C 424     121.566  79.207 123.766  1.00 30.00           O
ATOM   3016  CB  ASP C 424     121.148  79.887 127.115  1.00 20.00           C
ATOM   3017  CG  ASP C 424     121.777  78.500 127.357  1.00 20.00           C
ATOM   3018  OD1 ASP C 424     121.897  78.155 128.552  1.00 20.00           O
ATOM   3019  OD2 ASP C 424     122.051  77.757 126.389  1.00 20.00           O1-
ATOM   3020  H   ASP C 424     120.162  82.135 126.459  1.00  0.00           H
ATOM   3021  HA  ASP C 424     122.410  80.645 125.555  1.00  0.00           H
ATOM   3022  HB2 ASP C 424     121.589  80.561 127.849  1.00  0.00           H
ATOM   3023  HB3 ASP C 424     120.085  79.819 127.341  1.00  0.00           H
ATOM   3024  N   LYS C 425     119.429  79.659 124.417  1.00  0.00           N
ATOM   3025  CA  LYS C 425     118.726  79.030 123.290  1.00  0.00           C
ATOM   3026  C   LYS C 425     119.184  79.523 121.907  1.00  0.00           C
ATOM   3027  O   LYS C 425     119.235  78.711 120.985  1.00  0.00           O
ATOM   3028  CB  LYS C 425     117.195  79.200 123.428  1.00 20.00           C
ATOM   3029  CG  LYS C 425     116.540  78.190 124.382  1.00 20.00           C
ATOM   3030  CD  LYS C 425     115.004  78.232 124.350  1.00 20.00           C
ATOM   3031  CE  LYS C 425     114.360  77.221 125.314  1.00 20.00           C
ATOM   3032  NZ  LYS C 425     112.902  77.140 125.119  1.00 20.00           N1+
ATOM   3033  H   LYS C 425     118.843  80.115 125.104  1.00  0.00           H
ATOM   3034  HA  LYS C 425     118.963  77.965 123.336  1.00  0.00           H
ATOM   3035  HB2 LYS C 425     116.941  80.224 123.701  1.00  0.00           H
ATOM   3036  HB3 LYS C 425     116.738  79.034 122.451  1.00  0.00           H
ATOM   3037  HG2 LYS C 425     116.853  77.193 124.079  1.00  0.00           H
ATOM   3038  HG3 LYS C 425     116.904  78.345 125.398  1.00  0.00           H
ATOM   3039  HD2 LYS C 425     114.659  79.240 124.583  1.00  0.00           H
ATOM   3040  HD3 LYS C 425     114.665  78.022 123.335  1.00  0.00           H
ATOM   3041  HE2 LYS C 425     114.777  76.226 125.156  1.00  0.00           H
```

```
ATOM   3042  HE3 LYS C 425     114.567  77.498 126.347  1.00  0.00           H
ATOM   3043  HZ1 LYS C 425     112.706  76.809 124.178  1.00  0.00           H
ATOM   3044  HZ2 LYS C 425     112.507  76.482 125.774  1.00  0.00           H
ATOM   3045  HZ3 LYS C 425     112.480  78.047 125.246  1.00  0.00           H
ATOM   3046  N   TRP C 426     119.530  80.819 121.798  1.00  0.00           N
ATOM   3047  CA  TRP C 426     120.048  81.417 120.575  1.00  0.00           C
ATOM   3048  C   TRP C 426     121.467  80.912 120.255  1.00  0.00           C
ATOM   3049  O   TRP C 426     121.592  80.103 119.338  1.00  0.00           O
ATOM   3050  CB  TRP C 426     119.870  82.949 120.588  1.00 20.00           C
ATOM   3051  CG  TRP C 426     119.999  83.593 119.240  1.00 20.00           C
ATOM   3052  CD1 TRP C 426     119.092  83.488 118.241  1.00 20.00           C
ATOM   3053  CD2 TRP C 426     121.101  84.373 118.692  1.00 20.00           C
ATOM   3054  CE2 TRP C 426     120.830  84.633 117.315  1.00 20.00           C
ATOM   3055  CE3 TRP C 426     122.314  84.874 119.214  1.00 20.00           C
ATOM   3056  NE1 TRP C 426     119.586  84.082 117.099  1.00 20.00           N
ATOM   3057  CZ2 TRP C 426     121.739  85.312 116.488  1.00 20.00           C
ATOM   3058  CZ3 TRP C 426     123.222  85.584 118.402  1.00 20.00           C
ATOM   3059  CH2 TRP C 426     122.941  85.794 117.039  1.00 20.00           C
ATOM   3060  H   TRP C 426     119.453  81.428 122.601  1.00  0.00           H
ATOM   3061  HA  TRP C 426     119.411  81.059 119.773  1.00  0.00           H
ATOM   3062  HB2 TRP C 426     118.867  83.189 120.940  1.00  0.00           H
ATOM   3063  HB3 TRP C 426     120.556  83.420 121.294  1.00  0.00           H
ATOM   3064  HD1 TRP C 426     118.144  82.980 118.329  1.00  0.00           H
ATOM   3065  HE1 TRP C 426     119.081  84.083 116.223  1.00  0.00           H
ATOM   3066  HE3 TRP C 426     122.540  84.709 120.255  1.00  0.00           H
ATOM   3067  HZ2 TRP C 426     121.514  85.468 115.443  1.00  0.00           H
ATOM   3068  HZ3 TRP C 426     124.144  85.950 118.828  1.00  0.00           H
ATOM   3069  HH2 TRP C 426     123.645  86.327 116.417  1.00  0.00           H
ATOM   3070  N   ASP C 427     122.483  81.338 121.031  1.00 30.00           N
ATOM   3071  CA  ASP C 427     123.903  80.977 120.855  1.00 30.00           C
ATOM   3072  C   ASP C 427     124.213  79.471 120.755  1.00 30.00           C
ATOM   3073  O   ASP C 427     125.064  79.100 119.945  1.00 30.00           O
ATOM   3074  CB  ASP C 427     124.833  81.610 121.921  1.00 20.00           C
ATOM   3075  CG  ASP C 427     124.815  83.139 121.974  1.00  0.00           C
ATOM   3076  OD1 ASP C 427     124.748  83.754 120.887  1.00  0.00           O
ATOM   3077  OD2 ASP C 427     124.990  83.673 123.091  1.00  0.00           O1-
ATOM   3078  H   ASP C 427     122.286  81.971 121.795  1.00  0.00           H
ATOM   3079  HA  ASP C 427     124.183  81.395 119.887  1.00  0.00           H
ATOM   3080  HB2 ASP C 427     124.522  81.241 122.900  1.00  0.00           H
ATOM   3081  HB3 ASP C 427     125.866  81.293 121.776  1.00  0.00           H
ATOM   3082  N   ARG C 428     123.535  78.638 121.563  1.00 30.00           N
ATOM   3083  CA  ARG C 428     123.757  77.191 121.617  1.00 30.00           C
ATOM   3084  C   ARG C 428     123.052  76.414 120.489  1.00 30.00           C
ATOM   3085  O   ARG C 428     123.608  75.410 120.042  1.00 30.00           O
ATOM   3086  CB  ARG C 428     123.342  76.675 123.013  1.00 20.00           C
ATOM   3087  CG  ARG C 428     123.460  75.156 123.235  1.00 20.00           C
ATOM   3088  CD  ARG C 428     123.270  74.778 124.708  1.00 20.00           C
ATOM   3089  NE  ARG C 428     122.988  73.344 124.867  1.00 20.00           N
ATOM   3090  CZ  ARG C 428     122.846  72.700 126.038  1.00 20.00           C
ATOM   3091  NH1 ARG C 428     122.980  73.343 127.207  1.00 20.00           N
ATOM   3092  NH2 ARG C 428     122.558  71.392 126.035  1.00 20.00           N1+
ATOM   3093  H   ARG C 428     122.851  79.013 122.209  1.00  0.00           H
ATOM   3094  HA  ARG C 428     124.829  77.011 121.513  1.00  0.00           H
ATOM   3095  HB2 ARG C 428     123.926  77.200 123.770  1.00  0.00           H
ATOM   3096  HB3 ARG C 428     122.300  76.933 123.194  1.00  0.00           H
ATOM   3097  HG2 ARG C 428     122.800  74.577 122.587  1.00  0.00           H
ATOM   3098  HG3 ARG C 428     124.480  74.886 122.959  1.00  0.00           H
ATOM   3099  HD2 ARG C 428     124.228  74.918 125.210  1.00  0.00           H
ATOM   3100  HD3 ARG C 428     122.559  75.425 125.220  1.00  0.00           H
ATOM   3101  HE  ARG C 428     122.890  72.818 124.012  1.00  0.00           H
ATOM   3102 HH11 ARG C 428     122.894  72.845 128.081  1.00  0.00           H
ATOM   3103 HH12 ARG C 428     123.232  74.322 127.220  1.00  0.00           H
ATOM   3104 HH21 ARG C 428     122.454  70.889 126.904  1.00  0.00           H
ATOM   3105 HH22 ARG C 428     122.455  70.890 125.165  1.00  0.00           H
ATOM   3106  N   PHE C 429     121.845  76.852 120.084  1.00 30.00           N
ATOM   3107  CA  PHE C 429     120.940  76.057 119.254  1.00 30.00           C
ATOM   3108  C   PHE C 429     120.467  76.795 117.990  1.00 30.00           C
ATOM   3109  O   PHE C 429     120.991  76.499 116.915  1.00 30.00           O
ATOM   3110  CB  PHE C 429     119.832  75.423 120.135  1.00 20.00           C
ATOM   3111  CG  PHE C 429     118.709  74.698 119.411  1.00 20.00           C
ATOM   3112  CD1 PHE C 429     118.955  73.459 118.782  1.00 20.00           C
```

ATOM	3113	CD2	PHE	C	429	117.444	75.305	119.259	1.00	20.00	C
ATOM	3114	CE1	PHE	C	429	117.944	72.829	118.069	1.00	20.00	C
ATOM	3115	CE2	PHE	C	429	116.447	74.661	118.538	1.00	20.00	C
ATOM	3116	CZ	PHE	C	429	116.695	73.427	117.950	1.00	20.00	C
ATOM	3117	H	PHE	C	429	121.470	77.711	120.463	1.00	0.00	H
ATOM	3118	HA	PHE	C	429	121.487	75.203	118.850	1.00	0.00	H
ATOM	3119	HB2	PHE	C	429	120.303	74.690	120.792	1.00	0.00	H
ATOM	3120	HB3	PHE	C	429	119.399	76.167	120.802	1.00	0.00	H
ATOM	3121	HD1	PHE	C	429	119.930	73.000	118.853	1.00	0.00	H
ATOM	3122	HD2	PHE	C	429	117.245	76.270	119.703	1.00	0.00	H
ATOM	3123	HE1	PHE	C	429	118.131	71.877	117.595	1.00	0.00	H
ATOM	3124	HE2	PHE	C	429	115.477	75.125	118.429	1.00	0.00	H
ATOM	3125	HZ	PHE	C	429	115.913	72.938	117.389	1.00	0.00	H
ATOM	3126	N	VAL	C	430	119.469	77.690	118.113	1.00	30.00	N
ATOM	3127	CA	VAL	C	430	118.740	78.229	116.956	1.00	30.00	C
ATOM	3128	C	VAL	C	430	119.542	79.193	116.046	1.00	30.00	C
ATOM	3129	O	VAL	C	430	119.184	79.320	114.878	1.00	30.00	O
ATOM	3130	CB	VAL	C	430	117.361	78.849	117.337	1.00	20.00	C
ATOM	3131	CG1	VAL	C	430	117.458	80.090	118.224	1.00	20.00	C
ATOM	3132	CG2	VAL	C	430	116.462	79.183	116.132	1.00	20.00	C
ATOM	3133	H	VAL	C	430	119.104	77.923	119.027	1.00	0.00	H
ATOM	3134	HA	VAL	C	430	118.511	77.356	116.347	1.00	0.00	H
ATOM	3135	HB	VAL	C	430	116.817	78.093	117.906	1.00	0.00	H
ATOM	3136	HG11	VAL	C	430	116.511	80.623	118.239	1.00	0.00	H
ATOM	3137	HG12	VAL	C	430	117.692	79.812	119.248	1.00	0.00	H
ATOM	3138	HG13	VAL	C	430	118.210	80.787	117.861	1.00	0.00	H
ATOM	3139	HG21	VAL	C	430	115.469	79.485	116.460	1.00	0.00	H
ATOM	3140	HG22	VAL	C	430	116.847	80.011	115.537	1.00	0.00	H
ATOM	3141	HG23	VAL	C	430	116.347	78.317	115.482	1.00	0.00	H
ATOM	3142	N	LYS	C	431	120.640	79.797	116.538	1.00	0.00	N
ATOM	3143	CA	LYS	C	431	121.570	80.622	115.747	1.00	0.00	C
ATOM	3144	C	LYS	C	431	122.168	79.882	114.530	1.00	0.00	C
ATOM	3145	O	LYS	C	431	122.387	80.513	113.497	1.00	0.00	O
ATOM	3146	CB	LYS	C	431	122.657	81.175	116.694	1.00	20.00	C
ATOM	3147	CG	LYS	C	431	123.749	82.064	116.072	1.00	20.00	C
ATOM	3148	CD	LYS	C	431	124.741	82.543	117.147	1.00	20.00	C
ATOM	3149	CE	LYS	C	431	125.783	83.551	116.644	1.00	20.00	C
ATOM	3150	NZ	LYS	C	431	126.694	82.953	115.656	1.00	20.00	N1+
ATOM	3151	H	LYS	C	431	120.890	79.657	117.509	1.00	0.00	H
ATOM	3152	HA	LYS	C	431	120.997	81.469	115.364	1.00	0.00	H
ATOM	3153	HB2	LYS	C	431	122.162	81.763	117.467	1.00	0.00	H
ATOM	3154	HB3	LYS	C	431	123.144	80.341	117.202	1.00	0.00	H
ATOM	3155	HG2	LYS	C	431	124.291	81.519	115.299	1.00	0.00	H
ATOM	3156	HG3	LYS	C	431	123.285	82.921	115.582	1.00	0.00	H
ATOM	3157	HD2	LYS	C	431	124.190	82.990	117.974	1.00	0.00	H
ATOM	3158	HD3	LYS	C	431	125.253	81.680	117.575	1.00	0.00	H
ATOM	3159	HE2	LYS	C	431	125.296	84.418	116.200	1.00	0.00	H
ATOM	3160	HE3	LYS	C	431	126.376	83.916	117.484	1.00	0.00	H
ATOM	3161	HZ1	LYS	C	431	126.159	82.632	114.862	1.00	0.00	H
ATOM	3162	HZ2	LYS	C	431	127.364	83.646	115.353	1.00	0.00	H
ATOM	3163	HZ3	LYS	C	431	127.181	82.173	116.074	1.00	0.00	H
ATOM	3164	N	ARG	C	432	122.371	78.559	114.665	1.00	0.00	N
ATOM	3165	CA	ARG	C	432	122.825	77.661	113.605	1.00	0.00	C
ATOM	3166	C	ARG	C	432	121.773	77.468	112.498	1.00	0.00	C
ATOM	3167	O	ARG	C	432	122.090	77.711	111.333	1.00	0.00	O
ATOM	3168	CB	ARG	C	432	123.231	76.301	114.211	1.00	20.00	C
ATOM	3169	CG	ARG	C	432	124.406	76.384	115.204	1.00	20.00	C
ATOM	3170	CD	ARG	C	432	124.813	75.015	115.774	1.00	20.00	C
ATOM	3171	NE	ARG	C	432	125.397	74.133	114.751	1.00	20.00	N
ATOM	3172	CZ	ARG	C	432	126.666	74.158	114.309	1.00	20.00	C
ATOM	3173	NH1	ARG	C	432	127.567	75.012	114.817	1.00	20.00	N
ATOM	3174	NH2	ARG	C	432	127.038	73.311	113.341	1.00	20.00	N1+
ATOM	3175	H	ARG	C	432	122.139	78.119	115.546	1.00	0.00	H
ATOM	3176	HA	ARG	C	432	123.711	78.107	113.149	1.00	0.00	H
ATOM	3177	HB2	ARG	C	432	122.371	75.852	114.709	1.00	0.00	H
ATOM	3178	HB3	ARG	C	432	123.501	75.619	113.404	1.00	0.00	H
ATOM	3179	HG2	ARG	C	432	125.250	76.784	114.641	1.00	0.00	H
ATOM	3180	HG3	ARG	C	432	124.228	77.088	116.018	1.00	0.00	H
ATOM	3181	HD2	ARG	C	432	123.893	74.501	116.054	1.00	0.00	H
ATOM	3182	HD3	ARG	C	432	125.400	75.085	116.690	1.00	0.00	H
ATOM	3183	HE	ARG	C	432	124.750	73.500	114.303	1.00	0.00	H

```
ATOM   3184 HH11 ARG C 432     128.517  75.022 114.478  1.00  0.00           H
ATOM   3185 HH12 ARG C 432     127.295  75.651 115.550  1.00  0.00           H
ATOM   3186 HH21 ARG C 432     127.987  73.314 112.994  1.00  0.00           H
ATOM   3187 HH22 ARG C 432     126.375  72.661 112.945  1.00  0.00           H
ATOM   3188  N   ILE C 433     120.553  77.035 112.873  1.00  0.00           N
ATOM   3189  CA  ILE C 433     119.479  76.725 111.920  1.00  0.00           C
ATOM   3190  C   ILE C 433     118.847  77.987 111.287  1.00  0.00           C
ATOM   3191  O   ILE C 433     118.380  77.906 110.154  1.00  0.00           O
ATOM   3192  CB  ILE C 433     118.371  75.824 112.548  1.00 20.00           C
ATOM   3193  CG1 ILE C 433     117.524  75.094 111.478  1.00 20.00           C
ATOM   3194  CG2 ILE C 433     117.450  76.550 113.547  1.00 20.00           C
ATOM   3195  CD1 ILE C 433     116.669  73.947 112.041  1.00 20.00           C
ATOM   3196  H   ILE C 433     120.352  76.873 113.849  1.00  0.00           H
ATOM   3197  HA  ILE C 433     119.939  76.151 111.115  1.00  0.00           H
ATOM   3198  HB  ILE C 433     118.898  75.053 113.113  1.00  0.00           H
ATOM   3199 HG12 ILE C 433     116.870  75.803 110.970  1.00  0.00           H
ATOM   3200 HG13 ILE C 433     118.177  74.687 110.705  1.00  0.00           H
ATOM   3201 HG21 ILE C 433     116.839  75.849 114.114  1.00  0.00           H
ATOM   3202 HG22 ILE C 433     118.042  77.111 114.260  1.00  0.00           H
ATOM   3203 HG23 ILE C 433     116.774  77.249 113.054  1.00  0.00           H
ATOM   3204 HD11 ILE C 433     116.392  73.248 111.252  1.00  0.00           H
ATOM   3205 HD12 ILE C 433     117.203  73.383 112.806  1.00  0.00           H
ATOM   3206 HD13 ILE C 433     115.747  74.323 112.484  1.00  0.00           H
ATOM   3207  N   PHE C 434     118.883  79.129 111.996  1.00  0.00           N
ATOM   3208  CA  PHE C 434     118.412  80.427 111.510  1.00  0.00           C
ATOM   3209  C   PHE C 434     119.304  80.999 110.392  1.00  0.00           C
ATOM   3210  O   PHE C 434     118.770  81.527 109.416  1.00  0.00           O
ATOM   3211  CB  PHE C 434     118.248  81.397 112.700  1.00 20.00           C
ATOM   3212  CG  PHE C 434     117.706  82.771 112.350  1.00 20.00           C
ATOM   3213  CD1 PHE C 434     116.316  82.960 112.188  1.00 20.00           C
ATOM   3214  CD2 PHE C 434     118.585  83.819 112.006  1.00 20.00           C
ATOM   3215  CE1 PHE C 434     115.828  84.186 111.756  1.00 20.00           C
ATOM   3216  CE2 PHE C 434     118.080  85.028 111.552  1.00 20.00           C
ATOM   3217  CZ  PHE C 434     116.707  85.214 111.444  1.00 20.00           C
ATOM   3218  H   PHE C 434     119.265  79.119 112.934  1.00  0.00           H
ATOM   3219  HA  PHE C 434     117.421  80.271 111.080  1.00  0.00           H
ATOM   3220  HB2 PHE C 434     117.561  80.955 113.422  1.00  0.00           H
ATOM   3221  HB3 PHE C 434     119.200  81.515 113.221  1.00  0.00           H
ATOM   3222  HD1 PHE C 434     115.629  82.155 112.403  1.00  0.00           H
ATOM   3223  HD2 PHE C 434     119.652  83.679 112.084  1.00  0.00           H
ATOM   3224  HE1 PHE C 434     114.764  84.338 111.651  1.00  0.00           H
ATOM   3225  HE2 PHE C 434     118.757  85.825 111.285  1.00  0.00           H
ATOM   3226  HZ  PHE C 434     116.320  86.165 111.113  1.00  0.00           H
ATOM   3227  N   TYR C 435     120.635  80.847 110.532  1.00  0.00           N
ATOM   3228  CA  TYR C 435     121.616  81.191 109.495  1.00  0.00           C
ATOM   3229  C   TYR C 435     121.545  80.247 108.281  1.00  0.00           C
ATOM   3230  O   TYR C 435     121.795  80.703 107.166  1.00  0.00           O
ATOM   3231  CB  TYR C 435     123.043  81.208 110.087  1.00 20.00           C
ATOM   3232  CG  TYR C 435     123.415  82.322 111.061  1.00 20.00           C
ATOM   3233  CD1 TYR C 435     122.528  83.373 111.393  1.00 20.00           C
ATOM   3234  CD2 TYR C 435     124.703  82.308 111.635  1.00 20.00           C
ATOM   3235  CE1 TYR C 435     122.920  84.381 112.294  1.00 20.00           C
ATOM   3236  CE2 TYR C 435     125.103  83.325 112.522  1.00 20.00           C
ATOM   3237  CZ  TYR C 435     124.211  84.362 112.856  1.00 20.00           C
ATOM   3238  OH  TYR C 435     124.600  85.349 113.713  1.00 20.00           O
ATOM   3239  H   TYR C 435     121.005  80.414 111.367  1.00  0.00           H
ATOM   3240  HA  TYR C 435     121.383  82.189 109.121  1.00  0.00           H
ATOM   3241  HB2 TYR C 435     123.249  80.251 110.569  1.00  0.00           H
ATOM   3242  HB3 TYR C 435     123.761  81.287 109.268  1.00  0.00           H
ATOM   3243  HD1 TYR C 435     121.542  83.425 110.962  1.00  0.00           H
ATOM   3244  HD2 TYR C 435     125.396  81.517 111.386  1.00  0.00           H
ATOM   3245  HE1 TYR C 435     122.230  85.175 112.540  1.00  0.00           H
ATOM   3246  HE2 TYR C 435     126.100  83.312 112.936  1.00  0.00           H
ATOM   3247  HH  TYR C 435     123.941  86.041 113.812  1.00  0.00           H
ATOM   3248  N   PHE C 436     121.163  78.976 108.505  1.00  0.00           N
ATOM   3249  CA  PHE C 436     120.867  78.006 107.451  1.00  0.00           C
ATOM   3250  C   PHE C 436     119.617  78.370 106.628  1.00  0.00           C
ATOM   3251  O   PHE C 436     119.674  78.293 105.404  1.00  0.00           O
ATOM   3252  CB  PHE C 436     120.838  76.576 108.037  1.00 20.00           C
ATOM   3253  CG  PHE C 436     120.299  75.483 107.129  1.00 20.00           C
ATOM   3254  CD1 PHE C 436     121.113  74.945 106.111  1.00 20.00           C
```

```
ATOM   3255  CD2 PHE C 436     118.936  75.118 107.185  1.00 20.00           C
ATOM   3256  CE1 PHE C 436     120.587  74.020 105.219  1.00 20.00           C
ATOM   3257  CE2 PHE C 436     118.429  74.193 106.282  1.00 20.00           C
ATOM   3258  CZ  PHE C 436     119.253  73.642 105.309  1.00 20.00           C
ATOM   3259  H   PHE C 436     120.995  78.669 109.453  1.00  0.00           H
ATOM   3260  HA  PHE C 436     121.705  78.030 106.750  1.00  0.00           H
ATOM   3261  HB2 PHE C 436     121.852  76.297 108.327  1.00  0.00           H
ATOM   3262  HB3 PHE C 436     120.257  76.565 108.955  1.00  0.00           H
ATOM   3263  HD1 PHE C 436     122.145  75.251 106.020  1.00  0.00           H
ATOM   3264  HD2 PHE C 436     118.283  75.559 107.925  1.00  0.00           H
ATOM   3265  HE1 PHE C 436     121.214  73.602 104.446  1.00  0.00           H
ATOM   3266  HE2 PHE C 436     117.388  73.910 106.332  1.00  0.00           H
ATOM   3267  HZ  PHE C 436     118.849  72.927 104.607  1.00  0.00           H
ATOM   3268  N   ASN C 437     118.534  78.796 107.304  1.00 30.00           N
ATOM   3269  CA  ASN C 437     117.289  79.258 106.676  1.00 30.00           C
ATOM   3270  C   ASN C 437     117.469  80.574 105.898  1.00 30.00           C
ATOM   3271  O   ASN C 437     116.810  80.742 104.874  1.00 30.00           O
ATOM   3272  CB  ASN C 437     116.179  79.408 107.744  1.00 20.00           C
ATOM   3273  CG  ASN C 437     115.694  78.082 108.346  1.00 20.00           C
ATOM   3274  ND2 ASN C 437     115.222  78.124 109.593  1.00 20.00           N
ATOM   3275  OD1 ASN C 437     115.720  77.039 107.696  1.00 20.00           O
ATOM   3276  H   ASN C 437     118.555  78.806 108.315  1.00  0.00           H
ATOM   3277  HA  ASN C 437     116.999  78.479 105.968  1.00  0.00           H
ATOM   3278  HB2 ASN C 437     116.510  80.078 108.539  1.00  0.00           H
ATOM   3279  HB3 ASN C 437     115.302  79.877 107.294  1.00  0.00           H
ATOM   3280 HD21 ASN C 437     114.891  77.278 110.033  1.00  0.00           H
ATOM   3281 HD22 ASN C 437     115.213  78.990 110.111  1.00  0.00           H
ATOM   3282  N   PHE C 438     118.372  81.456 106.365  1.00  0.00           N
ATOM   3283  CA  PHE C 438     118.775  82.677 105.666  1.00  0.00           C
ATOM   3284  C   PHE C 438     119.590  82.384 104.392  1.00  0.00           C
ATOM   3285  O   PHE C 438     119.334  83.004 103.362  1.00  0.00           O
ATOM   3286  CB  PHE C 438     119.527  83.606 106.646  1.00 20.00           C
ATOM   3287  CG  PHE C 438     120.078  84.913 106.090  1.00 20.00           C
ATOM   3288  CD1 PHE C 438     119.329  85.694 105.180  1.00 20.00           C
ATOM   3289  CD2 PHE C 438     121.270  85.450 106.622  1.00 20.00           C
ATOM   3290  CE1 PHE C 438     119.798  86.936 104.775  1.00 20.00           C
ATOM   3291  CE2 PHE C 438     121.724  86.693 106.201  1.00 20.00           C
ATOM   3292  CZ  PHE C 438     120.995  87.430 105.277  1.00 20.00           C
ATOM   3293  H   PHE C 438     118.852  81.259 107.233  1.00  0.00           H
ATOM   3294  HA  PHE C 438     117.860  83.186 105.355  1.00  0.00           H
ATOM   3295  HB2 PHE C 438     118.876  83.859 107.481  1.00  0.00           H
ATOM   3296  HB3 PHE C 438     120.356  83.049 107.083  1.00  0.00           H
ATOM   3297  HD1 PHE C 438     118.384  85.341 104.795  1.00  0.00           H
ATOM   3298  HD2 PHE C 438     121.834  84.898 107.359  1.00  0.00           H
ATOM   3299  HE1 PHE C 438     119.221  87.526 104.080  1.00  0.00           H
ATOM   3300  HE2 PHE C 438     122.643  87.094 106.604  1.00  0.00           H
ATOM   3301  HZ  PHE C 438     121.351  88.399 104.960  1.00  0.00           H
ATOM   3302  N   PHE C 439     120.526  81.422 104.483  1.00  0.00           N
ATOM   3303  CA  PHE C 439     121.339  80.925 103.371  1.00  0.00           C
ATOM   3304  C   PHE C 439     120.507  80.237 102.274  1.00  0.00           C
ATOM   3305  O   PHE C 439     120.721  80.511 101.094  1.00  0.00           O
ATOM   3306  CB  PHE C 439     122.472  80.035 103.933  1.00 20.00           C
ATOM   3307  CG  PHE C 439     123.343  79.321 102.914  1.00 20.00           C
ATOM   3308  CD1 PHE C 439     124.375  80.020 102.252  1.00 20.00           C
ATOM   3309  CD2 PHE C 439     123.036  78.004 102.507  1.00 20.00           C
ATOM   3310  CE1 PHE C 439     125.117  79.389 101.262  1.00 20.00           C
ATOM   3311  CE2 PHE C 439     123.788  77.393 101.513  1.00 20.00           C
ATOM   3312  CZ  PHE C 439     124.828  78.080 100.899  1.00 20.00           C
ATOM   3313  H   PHE C 439     120.684  80.974 105.376  1.00  0.00           H
ATOM   3314  HA  PHE C 439     121.811  81.793 102.906  1.00  0.00           H
ATOM   3315  HB2 PHE C 439     123.128  80.655 104.546  1.00  0.00           H
ATOM   3316  HB3 PHE C 439     122.057  79.289 104.611  1.00  0.00           H
ATOM   3317  HD1 PHE C 439     124.593  81.045 102.515  1.00  0.00           H
ATOM   3318  HD2 PHE C 439     122.218  77.470 102.967  1.00  0.00           H
ATOM   3319  HE1 PHE C 439     125.914  79.922 100.766  1.00  0.00           H
ATOM   3320  HE2 PHE C 439     123.558  76.382 101.210  1.00  0.00           H
ATOM   3321  HZ  PHE C 439     125.405  77.598 100.124  1.00  0.00           H
ATOM   3322  N   VAL C 440     119.545  79.396 102.691  1.00  0.00           N
ATOM   3323  CA  VAL C 440     118.566  78.737 101.826  1.00  0.00           C
ATOM   3324  C   VAL C 440     117.618  79.738 101.134  1.00  0.00           C
ATOM   3325  O   VAL C 440     117.317  79.540  99.957  1.00  0.00           O
```

```
ATOM   3326  CB  VAL C 440     117.749  77.668 102.616  1.00 20.00           C
ATOM   3327  CG1 VAL C 440     116.416  77.213 101.981  1.00 20.00           C
ATOM   3328  CG2 VAL C 440     118.617  76.428 102.897  1.00 20.00           C
ATOM   3329  H   VAL C 440     119.454  79.205 103.681  1.00  0.00           H
ATOM   3330  HA  VAL C 440     119.124  78.227 101.038  1.00  0.00           H
ATOM   3331  HB  VAL C 440     117.495  78.100 103.586  1.00  0.00           H
ATOM   3332 HG11 VAL C 440     115.971  76.396 102.550  1.00  0.00           H
ATOM   3333 HG12 VAL C 440     115.675  78.013 101.960  1.00  0.00           H
ATOM   3334 HG13 VAL C 440     116.563  76.859 100.960  1.00  0.00           H
ATOM   3335 HG21 VAL C 440     118.098  75.726 103.549  1.00  0.00           H
ATOM   3336 HG22 VAL C 440     118.864  75.903 101.973  1.00  0.00           H
ATOM   3337 HG23 VAL C 440     119.557  76.687 103.382  1.00  0.00           H
ATOM   3338  N   TYR C 441     117.197  80.800 101.848  1.00  0.00           N
ATOM   3339  CA  TYR C 441     116.347  81.855 101.292  1.00  0.00           C
ATOM   3340  C   TYR C 441     117.086  82.760 100.290  1.00  0.00           C
ATOM   3341  O   TYR C 441     116.482  83.171  99.300  1.00  0.00           O
ATOM   3342  CB  TYR C 441     115.686  82.682 102.411  1.00 20.00           C
ATOM   3343  CG  TYR C 441     114.607  83.611 101.886  1.00 20.00           C
ATOM   3344  CD1 TYR C 441     113.391  83.057 101.439  1.00 20.00           C
ATOM   3345  CD2 TYR C 441     114.827  85.002 101.778  1.00 20.00           C
ATOM   3346  CE1 TYR C 441     112.397  83.881 100.886  1.00 20.00           C
ATOM   3347  CE2 TYR C 441     113.829  85.829 101.225  1.00 20.00           C
ATOM   3348  CZ  TYR C 441     112.617  85.265 100.780  1.00 20.00           C
ATOM   3349  OH  TYR C 441     111.648  86.046 100.234  1.00 20.00           O
ATOM   3350  H   TYR C 441     117.467  80.897 102.818  1.00  0.00           H
ATOM   3351  HA  TYR C 441     115.545  81.354 100.751  1.00  0.00           H
ATOM   3352  HB2 TYR C 441     115.211  82.011 103.128  1.00  0.00           H
ATOM   3353  HB3 TYR C 441     116.433  83.246 102.973  1.00  0.00           H
ATOM   3354  HD1 TYR C 441     113.231  81.991 101.499  1.00  0.00           H
ATOM   3355  HD2 TYR C 441     115.761  85.433 102.104  1.00  0.00           H
ATOM   3356  HE1 TYR C 441     111.470  83.454 100.537  1.00  0.00           H
ATOM   3357  HE2 TYR C 441     114.000  86.892 101.136  1.00  0.00           H
ATOM   3358  HH  TYR C 441     111.941  86.951 100.078  1.00  0.00           H
ATOM   3359  N   CYS C 442     118.379  83.023 100.546  1.00  0.00           N
ATOM   3360  CA  CYS C 442     119.284  83.748  99.655  1.00  0.00           C
ATOM   3361  C   CYS C 442     119.445  83.020  98.306  1.00  0.00           C
ATOM   3362  O   CYS C 442     119.307  83.653  97.261  1.00  0.00           O
ATOM   3363  CB  CYS C 442     120.638  84.020 100.347  1.00 20.00           C
ATOM   3364  SG  CYS C 442     121.781  84.983  99.313  1.00 20.00           S
ATOM   3365  H   CYS C 442     118.791  82.671 101.401  1.00  0.00           H
ATOM   3366  HA  CYS C 442     118.820  84.715  99.451  1.00  0.00           H
ATOM   3367  HB2 CYS C 442     120.480  84.568 101.276  1.00  0.00           H
ATOM   3368  HB3 CYS C 442     121.128  83.085 100.619  1.00  0.00           H
ATOM   3369  HG  CYS C 442     121.965  84.038  98.386  1.00  0.00           H
ATOM   3370  N   LEU C 443     119.674  81.696  98.368  1.00  0.00           N
ATOM   3371  CA  LEU C 443     119.781  80.806  97.211  1.00  0.00           C
ATOM   3372  C   LEU C 443     118.446  80.650  96.458  1.00  0.00           C
ATOM   3373  O   LEU C 443     118.473  80.596  95.230  1.00  0.00           O
ATOM   3374  CB  LEU C 443     120.342  79.434  97.656  1.00 20.00           C
ATOM   3375  CG  LEU C 443     121.880  79.286  97.567  1.00 20.00           C
ATOM   3376  CD1 LEU C 443     122.664  80.241  98.489  1.00 20.00           C
ATOM   3377  CD2 LEU C 443     122.314  77.821  97.780  1.00 20.00           C
ATOM   3378  H   LEU C 443     119.772  81.255  99.272  1.00  0.00           H
ATOM   3379  HA  LEU C 443     120.486  81.257  96.509  1.00  0.00           H
ATOM   3380  HB2 LEU C 443     119.994  79.188  98.661  1.00  0.00           H
ATOM   3381  HB3 LEU C 443     119.919  78.659  97.013  1.00  0.00           H
ATOM   3382  HG  LEU C 443     122.155  79.548  96.545  1.00  0.00           H
ATOM   3383 HD11 LEU C 443     123.651  80.450  98.075  1.00  0.00           H
ATOM   3384 HD12 LEU C 443     122.176  81.202  98.628  1.00  0.00           H
ATOM   3385 HD13 LEU C 443     122.810  79.812  99.480  1.00  0.00           H
ATOM   3386 HD21 LEU C 443     123.067  77.720  98.561  1.00  0.00           H
ATOM   3387 HD22 LEU C 443     121.477  77.181  98.061  1.00  0.00           H
ATOM   3388 HD23 LEU C 443     122.744  77.409  96.867  1.00  0.00           H
ATOM   3389  N   TYR C 444     117.314  80.615  97.185  1.00  0.00           N
ATOM   3390  CA  TYR C 444     115.960  80.573  96.624  1.00  0.00           C
ATOM   3391  C   TYR C 444     115.631  81.804  95.761  1.00  0.00           C
ATOM   3392  O   TYR C 444     115.136  81.633  94.647  1.00  0.00           O
ATOM   3393  CB  TYR C 444     114.926  80.351  97.753  1.00 20.00           C
ATOM   3394  CG  TYR C 444     113.473  80.569  97.360  1.00 20.00           C
ATOM   3395  CD1 TYR C 444     112.800  79.601  96.588  1.00 20.00           C
ATOM   3396  CD2 TYR C 444     112.809  81.761  97.721  1.00 20.00           C
```

```
ATOM   3397  CE1 TYR C 444     111.479  79.835  96.164  1.00 20.00           C
ATOM   3398  CE2 TYR C 444     111.489  81.993  97.291  1.00 20.00           C
ATOM   3399  CZ  TYR C 444     110.824  81.034  96.504  1.00 20.00           C
ATOM   3400  OH  TYR C 444     109.549  81.267  96.076  1.00 20.00           O
ATOM   3401  H   TYR C 444     117.375  80.635  98.195  1.00  0.00           H
ATOM   3402  HA  TYR C 444     115.919  79.700  95.969  1.00  0.00           H
ATOM   3403  HB2 TYR C 444     115.037  79.349  98.169  1.00  0.00           H
ATOM   3404  HB3 TYR C 444     115.140  81.036  98.570  1.00  0.00           H
ATOM   3405  HD1 TYR C 444     113.298  78.684  96.308  1.00  0.00           H
ATOM   3406  HD2 TYR C 444     113.318  82.514  98.306  1.00  0.00           H
ATOM   3407  HE1 TYR C 444     110.966  79.081  95.589  1.00  0.00           H
ATOM   3408  HE2 TYR C 444     110.993  82.913  97.559  1.00  0.00           H
ATOM   3409  HH  TYR C 444     109.231  80.609  95.449  1.00  0.00           H
ATOM   3410  N   MET C 445     115.929  83.007  96.282  1.00  0.00           N
ATOM   3411  CA  MET C 445     115.715  84.272  95.578  1.00  0.00           C
ATOM   3412  C   MET C 445     116.654  84.476  94.378  1.00  0.00           C
ATOM   3413  O   MET C 445     116.227  85.106  93.414  1.00  0.00           O
ATOM   3414  CB  MET C 445     115.789  85.459  96.561  1.00 20.00           C
ATOM   3415  CG  MET C 445     114.600  85.560  97.536  1.00 20.00           C
ATOM   3416  SD  MET C 445     112.935  85.580  96.799  1.00 20.00           S
ATOM   3417  CE  MET C 445     113.124  86.869  95.537  1.00 20.00           C
ATOM   3418  H   MET C 445     116.324  83.069  97.211  1.00  0.00           H
ATOM   3419  HA  MET C 445     114.711  84.237  95.154  1.00  0.00           H
ATOM   3420  HB2 MET C 445     116.716  85.400  97.134  1.00  0.00           H
ATOM   3421  HB3 MET C 445     115.854  86.396  96.010  1.00  0.00           H
ATOM   3422  HG2 MET C 445     114.707  86.466  98.132  1.00  0.00           H
ATOM   3423  HG3 MET C 445     114.630  84.732  98.243  1.00  0.00           H
ATOM   3424  HE1 MET C 445     112.152  87.143  95.127  1.00  0.00           H
ATOM   3425  HE2 MET C 445     113.582  87.758  95.967  1.00  0.00           H
ATOM   3426  HE3 MET C 445     113.752  86.521  94.717  1.00  0.00           H
ATOM   3427  N   ILE C 446     117.876  83.915  94.429  1.00  0.00           N
ATOM   3428  CA  ILE C 446     118.830  83.894  93.315  1.00  0.00           C
ATOM   3429  C   ILE C 446     118.380  82.966  92.165  1.00  0.00           C
ATOM   3430  O   ILE C 446     118.518  83.347  91.002  1.00  0.00           O
ATOM   3431  CB  ILE C 446     120.264  83.515  93.804  1.00 20.00           C
ATOM   3432  CG1 ILE C 446     120.877  84.691  94.601  1.00 20.00           C
ATOM   3433  CG2 ILE C 446     121.253  83.054  92.706  1.00 20.00           C
ATOM   3434  CD1 ILE C 446     122.080  84.304  95.473  1.00 20.00           C
ATOM   3435  H   ILE C 446     118.162  83.428  95.268  1.00  0.00           H
ATOM   3436  HA  ILE C 446     118.877  84.906  92.908  1.00  0.00           H
ATOM   3437  HB  ILE C 446     120.155  82.679  94.496  1.00  0.00           H
ATOM   3438 HG12 ILE C 446     121.170  85.483  93.911  1.00  0.00           H
ATOM   3439 HG13 ILE C 446     120.124  85.136  95.249  1.00  0.00           H
ATOM   3440 HG21 ILE C 446     122.242  82.855  93.118  1.00  0.00           H
ATOM   3441 HG22 ILE C 446     120.937  82.127  92.227  1.00  0.00           H
ATOM   3442 HG23 ILE C 446     121.365  83.813  91.932  1.00  0.00           H
ATOM   3443 HD11 ILE C 446     122.238  85.045  96.256  1.00  0.00           H
ATOM   3444 HD12 ILE C 446     121.933  83.339  95.959  1.00  0.00           H
ATOM   3445 HD13 ILE C 446     122.996  84.251  94.886  1.00  0.00           H
ATOM   3446  N   ILE C 447     117.824  81.790  92.505  1.00  0.00           N
ATOM   3447  CA  ILE C 447     117.258  80.828  91.554  1.00  0.00           C
ATOM   3448  C   ILE C 447     115.955  81.331  90.898  1.00  0.00           C
ATOM   3449  O   ILE C 447     115.779  81.134  89.695  1.00  0.00           O
ATOM   3450  CB  ILE C 447     117.041  79.435  92.228  1.00 20.00           C
ATOM   3451  CG1 ILE C 447     118.389  78.711  92.465  1.00 20.00           C
ATOM   3452  CG2 ILE C 447     116.032  78.490  91.538  1.00 20.00           C
ATOM   3453  CD1 ILE C 447     119.162  78.311  91.197  1.00 20.00           C
ATOM   3454  H   ILE C 447     117.764  81.535  93.482  1.00  0.00           H
ATOM   3455  HA  ILE C 447     117.980  80.712  90.745  1.00  0.00           H
ATOM   3456  HB  ILE C 447     116.630  79.624  93.221  1.00  0.00           H
ATOM   3457 HG12 ILE C 447     118.221  77.825  93.079  1.00  0.00           H
ATOM   3458 HG13 ILE C 447     119.041  79.351  93.059  1.00  0.00           H
ATOM   3459 HG21 ILE C 447     116.048  77.498  91.989  1.00  0.00           H
ATOM   3460 HG22 ILE C 447     115.008  78.855  91.624  1.00  0.00           H
ATOM   3461 HG23 ILE C 447     116.257  78.378  90.479  1.00  0.00           H
ATOM   3462 HD11 ILE C 447     119.804  77.453  91.394  1.00  0.00           H
ATOM   3463 HD12 ILE C 447     118.506  78.043  90.371  1.00  0.00           H
ATOM   3464 HD13 ILE C 447     119.804  79.125  90.859  1.00  0.00           H
ATOM   3465  N   PHE C 448     115.097  82.002  91.684  1.00  0.00           N
ATOM   3466  CA  PHE C 448     113.875  82.657  91.214  1.00  0.00           C
ATOM   3467  C   PHE C 448     114.161  83.868  90.302  1.00  0.00           C
```

ATOM	3468	O	PHE	C	448	113.472	84.035	89.295	1.00	0.00	O
ATOM	3469	CB	PHE	C	448	112.989	82.999	92.433	1.00	20.00	C
ATOM	3470	CG	PHE	C	448	111.693	83.760	92.185	1.00	20.00	C
ATOM	3471	CD1	PHE	C	448	110.912	83.539	91.027	1.00	20.00	C
ATOM	3472	CD2	PHE	C	448	111.157	84.556	93.219	1.00	20.00	C
ATOM	3473	CE1	PHE	C	448	109.698	84.188	90.870	1.00	20.00	C
ATOM	3474	CE2	PHE	C	448	109.927	85.176	93.054	1.00	20.00	C
ATOM	3475	CZ	PHE	C	448	109.213	85.012	91.875	1.00	20.00	C
ATOM	3476	H	PHE	C	448	115.300	82.096	92.671	1.00	0.00	H
ATOM	3477	HA	PHE	C	448	113.338	81.926	90.607	1.00	0.00	H
ATOM	3478	HB2	PHE	C	448	112.725	82.076	92.951	1.00	0.00	H
ATOM	3479	HB3	PHE	C	448	113.583	83.574	93.144	1.00	0.00	H
ATOM	3480	HD1	PHE	C	448	111.252	82.874	90.248	1.00	0.00	H
ATOM	3481	HD2	PHE	C	448	111.697	84.680	94.146	1.00	0.00	H
ATOM	3482	HE1	PHE	C	448	109.117	84.031	89.974	1.00	0.00	H
ATOM	3483	HE2	PHE	C	448	109.520	85.781	93.850	1.00	0.00	H
ATOM	3484	HZ	PHE	C	448	108.262	85.502	91.747	1.00	0.00	H
ATOM	3485	N	THR	C	449	115.211	84.641	90.632	1.00	30.00	N
ATOM	3486	CA	THR	C	449	115.742	85.730	89.810	1.00	30.00	C
ATOM	3487	C	THR	C	449	116.243	85.227	88.446	1.00	30.00	C
ATOM	3488	O	THR	C	449	115.878	85.827	87.439	1.00	30.00	O
ATOM	3489	CB	THR	C	449	116.899	86.471	90.538	1.00	20.00	C
ATOM	3490	CG2	THR	C	449	117.856	87.334	89.690	1.00	20.00	C
ATOM	3491	OG1	THR	C	449	116.322	87.279	91.539	1.00	20.00	O
ATOM	3492	H	THR	C	449	115.721	84.442	91.482	1.00	0.00	H
ATOM	3493	HA	THR	C	449	114.930	86.436	89.626	1.00	0.00	H
ATOM	3494	HB	THR	C	449	117.513	85.742	91.064	1.00	0.00	H
ATOM	3495	HG1	THR	C	449	115.978	86.695	92.223	1.00	0.00	H
ATOM	3496	HG21	THR	C	449	118.475	87.973	90.320	1.00	0.00	H
ATOM	3497	HG22	THR	C	449	118.535	86.714	89.103	1.00	0.00	H
ATOM	3498	HG23	THR	C	449	117.317	87.973	88.993	1.00	0.00	H
ATOM	3499	N	ALA	C	450	117.022	84.131	88.433	1.00	30.00	N
ATOM	3500	CA	ALA	C	450	117.559	83.510	87.221	1.00	30.00	C
ATOM	3501	C	ALA	C	450	116.473	82.939	86.291	1.00	30.00	C
ATOM	3502	O	ALA	C	450	116.539	83.176	85.086	1.00	30.00	O
ATOM	3503	CB	ALA	C	450	118.575	82.428	87.615	1.00	30.00	C
ATOM	3504	H	ALA	C	450	117.280	83.693	89.307	1.00	0.00	H
ATOM	3505	HA	ALA	C	450	118.094	84.283	86.666	1.00	0.00	H
ATOM	3506	HB1	ALA	C	450	119.011	81.955	86.735	1.00	0.00	H
ATOM	3507	HB2	ALA	C	450	119.393	82.855	88.196	1.00	0.00	H
ATOM	3508	HB3	ALA	C	450	118.115	81.645	88.220	1.00	0.00	H
ATOM	3509	N	ALA	C	451	115.482	82.236	86.867	1.00	30.00	N
ATOM	3510	CA	ALA	C	451	114.351	81.643	86.148	1.00	30.00	C
ATOM	3511	C	ALA	C	451	113.426	82.680	85.485	1.00	30.00	C
ATOM	3512	O	ALA	C	451	112.943	82.430	84.380	1.00	30.00	O
ATOM	3513	CB	ALA	C	451	113.561	80.745	87.113	1.00	30.00	C
ATOM	3514	H	ALA	C	451	115.502	82.080	87.867	1.00	0.00	H
ATOM	3515	HA	ALA	C	451	114.757	81.010	85.357	1.00	0.00	H
ATOM	3516	HB1	ALA	C	451	112.730	80.251	86.607	1.00	0.00	H
ATOM	3517	HB2	ALA	C	451	114.201	79.963	87.523	1.00	0.00	H
ATOM	3518	HB3	ALA	C	451	113.154	81.312	87.951	1.00	0.00	H
ATOM	3519	N	ALA	C	452	113.223	83.826	86.158	1.00	30.00	N
ATOM	3520	CA	ALA	C	452	112.423	84.946	85.666	1.00	30.00	C
ATOM	3521	C	ALA	C	452	113.183	85.830	84.660	1.00	30.00	C
ATOM	3522	O	ALA	C	452	112.559	86.314	83.717	1.00	30.00	O
ATOM	3523	CB	ALA	C	452	111.932	85.769	86.866	1.00	30.00	C
ATOM	3524	H	ALA	C	452	113.645	83.948	87.069	1.00	0.00	H
ATOM	3525	HA	ALA	C	452	111.543	84.545	85.159	1.00	0.00	H
ATOM	3526	HB1	ALA	C	452	111.251	86.559	86.549	1.00	0.00	H
ATOM	3527	HB2	ALA	C	452	111.387	85.143	87.574	1.00	0.00	H
ATOM	3528	HB3	ALA	C	452	112.762	86.230	87.404	1.00	0.00	H
ATOM	3529	N	TYR	C	453	114.501	86.020	84.865	1.00	30.00	N
ATOM	3530	CA	TYR	C	453	115.397	86.800	83.999	1.00	30.00	C
ATOM	3531	C	TYR	C	453	115.594	86.153	82.617	1.00	30.00	C
ATOM	3532	O	TYR	C	453	115.616	86.873	81.620	1.00	30.00	O
ATOM	3533	CB	TYR	C	453	116.738	87.038	84.735	1.00	20.00	C
ATOM	3534	CG	TYR	C	453	117.789	87.868	84.011	1.00	20.00	C
ATOM	3535	CD1	TYR	C	453	117.846	89.263	84.206	1.00	20.00	C
ATOM	3536	CD2	TYR	C	453	118.730	87.250	83.162	1.00	20.00	C
ATOM	3537	CE1	TYR	C	453	118.825	90.031	83.546	1.00	20.00	C
ATOM	3538	CE2	TYR	C	453	119.708	88.018	82.500	1.00	20.00	C

ATOM	3539	CZ	TYR C	453	119.755	89.413	82.691	1.00	20.00	C
ATOM	3540	OH	TYR C	453	120.697	90.168	82.053	1.00	20.00	O
ATOM	3541	H	TYR C	453	114.933	85.609	85.683	1.00	0.00	H
ATOM	3542	HA	TYR C	453	114.938	87.775	83.833	1.00	0.00	H
ATOM	3543	HB2	TYR C	453	116.536	87.543	85.680	1.00	0.00	H
ATOM	3544	HB3	TYR C	453	117.188	86.080	84.998	1.00	0.00	H
ATOM	3545	HD1	TYR C	453	117.138	89.747	84.862	1.00	0.00	H
ATOM	3546	HD2	TYR C	453	118.696	86.182	83.013	1.00	0.00	H
ATOM	3547	HE1	TYR C	453	118.860	91.100	83.689	1.00	0.00	H
ATOM	3548	HE2	TYR C	453	120.420	87.535	81.848	1.00	0.00	H
ATOM	3549	HH	TYR C	453	121.268	89.647	81.484	1.00	0.00	H
ATOM	3550	N	TYR C	454	115.696	84.813	82.590	1.00	30.00	N
ATOM	3551	CA	TYR C	454	115.828	84.007	81.377	1.00	30.00	C
ATOM	3552	C	TYR C	454	114.485	83.380	80.955	1.00	30.00	C
ATOM	3553	O	TYR C	454	114.478	82.257	80.450	1.00	30.00	O
ATOM	3554	CB	TYR C	454	116.945	82.953	81.575	1.00	20.00	C
ATOM	3555	CG	TYR C	454	118.344	83.521	81.745	1.00	20.00	C
ATOM	3556	CD1	TYR C	454	118.931	84.272	80.704	1.00	20.00	C
ATOM	3557	CD2	TYR C	454	119.076	83.286	82.928	1.00	20.00	C
ATOM	3558	CE1	TYR C	454	120.231	84.791	80.848	1.00	20.00	C
ATOM	3559	CE2	TYR C	454	120.375	83.810	83.075	1.00	20.00	C
ATOM	3560	CZ	TYR C	454	120.953	84.565	82.036	1.00	20.00	C
ATOM	3561	OH	TYR C	454	122.207	85.083	82.178	1.00	20.00	O
ATOM	3562	H	TYR C	454	115.677	84.299	83.461	1.00	0.00	H
ATOM	3563	HA	TYR C	454	116.121	84.645	80.543	1.00	0.00	H
ATOM	3564	HB2	TYR C	454	116.702	82.308	82.421	1.00	0.00	H
ATOM	3565	HB3	TYR C	454	116.998	82.296	80.706	1.00	0.00	H
ATOM	3566	HD1	TYR C	454	118.385	84.451	79.789	1.00	0.00	H
ATOM	3567	HD2	TYR C	454	118.650	82.699	83.728	1.00	0.00	H
ATOM	3568	HE1	TYR C	454	120.671	85.368	80.048	1.00	0.00	H
ATOM	3569	HE2	TYR C	454	120.924	83.628	83.988	1.00	0.00	H
ATOM	3570	HH	TYR C	454	122.611	84.868	83.022	1.00	0.00	H
ATOM	3571	N	ARG C	455	113.368	84.114	81.123	1.00	30.00	N
ATOM	3572	CA	ARG C	455	112.067	83.728	80.567	1.00	30.00	C
ATOM	3573	C	ARG C	455	112.036	83.958	79.035	1.00	30.00	C
ATOM	3574	O	ARG C	455	112.663	84.911	78.567	1.00	30.00	O
ATOM	3575	CB	ARG C	455	110.929	84.483	81.297	1.00	20.00	C
ATOM	3576	CG	ARG C	455	110.744	85.969	80.931	1.00	20.00	C
ATOM	3577	CD	ARG C	455	109.547	86.597	81.657	1.00	20.00	C
ATOM	3578	NE	ARG C	455	109.241	87.935	81.142	1.00	20.00	N
ATOM	3579	CZ	ARG C	455	108.632	88.238	79.984	1.00	20.00	C
ATOM	3580	NH1	ARG C	455	108.211	87.285	79.139	1.00	20.00	N
ATOM	3581	NH2	ARG C	455	108.444	89.527	79.673	1.00	20.00	N1+
ATOM	3582	H	ARG C	455	113.428	85.031	81.543	1.00	0.00	H
ATOM	3583	HA	ARG C	455	111.945	82.669	80.790	1.00	0.00	H
ATOM	3584	HB2	ARG C	455	111.050	84.376	82.376	1.00	0.00	H
ATOM	3585	HB3	ARG C	455	109.994	83.974	81.060	1.00	0.00	H
ATOM	3586	HG2	ARG C	455	111.651	86.485	81.245	1.00	0.00	H
ATOM	3587	HG3	ARG C	455	110.655	86.146	79.859	1.00	0.00	H
ATOM	3588	HD2	ARG C	455	108.677	85.942	81.721	1.00	0.00	H
ATOM	3589	HD3	ARG C	455	109.859	86.789	82.685	1.00	0.00	H
ATOM	3590	HE	ARG C	455	109.530	88.710	81.730	1.00	0.00	H
ATOM	3591	HH11	ARG C	455	107.777	87.533	78.262	1.00	0.00	H
ATOM	3592	HH12	ARG C	455	108.359	86.311	79.358	1.00	0.00	H
ATOM	3593	HH21	ARG C	455	107.983	89.792	78.814	1.00	0.00	H
ATOM	3594	HH22	ARG C	455	108.760	90.250	80.305	1.00	0.00	H
ATOM	3595	N	PRO C	456	111.293	83.115	78.281	1.00	30.00	N
ATOM	3596	CA	PRO C	456	111.098	83.311	76.833	1.00	30.00	C
ATOM	3597	C	PRO C	456	110.264	84.566	76.514	1.00	30.00	C
ATOM	3598	O	PRO C	456	109.195	84.761	77.095	1.00	30.00	O
ATOM	3599	CB	PRO C	456	110.398	82.018	76.381	1.00	20.00	C
ATOM	3600	CG	PRO C	456	109.670	81.522	77.614	1.00	20.00	C
ATOM	3601	CD	PRO C	456	110.598	81.916	78.749	1.00	20.00	C
ATOM	3602	HA	PRO C	456	112.065	83.386	76.333	1.00	0.00	H
ATOM	3603	HB2	PRO C	456	109.721	82.156	75.536	1.00	0.00	H
ATOM	3604	HB3	PRO C	456	111.150	81.288	76.077	1.00	0.00	H
ATOM	3605	HG2	PRO C	456	108.730	82.065	77.715	1.00	0.00	H
ATOM	3606	HG3	PRO C	456	109.452	80.455	77.589	1.00	0.00	H
ATOM	3607	HD2	PRO C	456	110.035	82.066	79.669	1.00	0.00	H
ATOM	3608	HD3	PRO C	456	111.341	81.135	78.909	1.00	0.00	H
ATOM	3609	N	VAL C	457	110.788	85.384	75.587	1.00	30.00	N

ATOM	3610	CA	VAL	C	457	110.128	86.571	75.037	1.00	30.00	C
ATOM	3611	C	VAL	C	457	108.946	86.227	74.096	1.00	30.00	C
ATOM	3612	O	VAL	C	457	108.070	87.071	73.908	1.00	30.00	O
ATOM	3613	CB	VAL	C	457	111.162	87.468	74.291	1.00	20.00	C
ATOM	3614	CG1	VAL	C	457	111.746	86.834	73.011	1.00	20.00	C
ATOM	3615	CG2	VAL	C	457	110.634	88.883	73.987	1.00	20.00	C
ATOM	3616	H	VAL	C	457	111.666	85.134	75.155	1.00	0.00	H
ATOM	3617	HA	VAL	C	457	109.722	87.141	75.875	1.00	0.00	H
ATOM	3618	HB	VAL	C	457	111.999	87.605	74.978	1.00	0.00	H
ATOM	3619	HG11	VAL	C	457	112.534	87.461	72.594	1.00	0.00	H
ATOM	3620	HG12	VAL	C	457	112.184	85.857	73.213	1.00	0.00	H
ATOM	3621	HG13	VAL	C	457	110.992	86.706	72.234	1.00	0.00	H
ATOM	3622	HG21	VAL	C	457	111.433	89.530	73.622	1.00	0.00	H
ATOM	3623	HG22	VAL	C	457	109.851	88.880	73.229	1.00	0.00	H
ATOM	3624	HG23	VAL	C	457	110.228	89.348	74.885	1.00	0.00	H
ATOM	3625	N	GLU	C	458	108.930	84.987	73.566	1.00	30.00	N
ATOM	3626	CA	GLU	C	458	107.911	84.398	72.692	1.00	30.00	C
ATOM	3627	C	GLU	C	458	106.468	84.539	73.224	1.00	30.00	C
ATOM	3628	O	GLU	C	458	106.253	84.640	74.433	1.00	30.00	O
ATOM	3629	CB	GLU	C	458	108.235	82.901	72.442	1.00	20.00	C
ATOM	3630	CG	GLU	C	458	109.550	82.601	71.680	1.00	0.00	C
ATOM	3631	CD	GLU	C	458	110.761	82.291	72.570	1.00	0.00	C
ATOM	3632	OE1	GLU	C	458	111.138	83.174	73.370	1.00	0.00	O
ATOM	3633	OE2	GLU	C	458	111.304	81.170	72.430	1.00	0.00	O1-
ATOM	3634	H	GLU	C	458	109.715	84.375	73.762	1.00	0.00	H
ATOM	3635	HA	GLU	C	458	107.963	84.931	71.742	1.00	0.00	H
ATOM	3636	HB2	GLU	C	458	108.238	82.370	73.395	1.00	0.00	H
ATOM	3637	HB3	GLU	C	458	107.417	82.452	71.876	1.00	0.00	H
ATOM	3638	HG2	GLU	C	458	109.387	81.738	71.034	1.00	0.00	H
ATOM	3639	HG3	GLU	C	458	109.802	83.426	71.013	1.00	0.00	H
ATOM	3640	N	GLY	C	459	105.499	84.526	72.296	1.00	30.00	N
ATOM	3641	CA	GLY	C	459	104.074	84.596	72.609	1.00	30.00	C
ATOM	3642	C	GLY	C	459	103.541	83.225	73.054	1.00	30.00	C
ATOM	3643	O	GLY	C	459	104.108	82.186	72.713	1.00	30.00	O
ATOM	3644	H	GLY	C	459	105.748	84.427	71.323	1.00	0.00	H
ATOM	3645	HA2	GLY	C	459	103.885	85.348	73.376	1.00	0.00	H
ATOM	3646	HA3	GLY	C	459	103.535	84.909	71.715	1.00	0.00	H
ATOM	3647	N	LEU	C	460	102.405	83.256	73.772	1.00	30.00	N
ATOM	3648	CA	LEU	C	460	101.577	82.119	74.190	1.00	30.00	C
ATOM	3649	C	LEU	C	460	102.286	81.143	75.163	1.00	30.00	C
ATOM	3650	O	LEU	C	460	102.878	80.164	74.707	1.00	30.00	O
ATOM	3651	CB	LEU	C	460	100.990	81.403	72.940	1.00	20.00	C
ATOM	3652	CG	LEU	C	460	99.909	80.336	73.238	1.00	0.00	C
ATOM	3653	CD1	LEU	C	460	98.703	80.937	73.980	1.00	0.00	C
ATOM	3654	CD2	LEU	C	460	99.477	79.602	71.952	1.00	0.00	C
ATOM	3655	H	LEU	C	460	102.014	84.159	73.997	1.00	0.00	H
ATOM	3656	HA	LEU	C	460	100.733	82.569	74.711	1.00	0.00	H
ATOM	3657	HB2	LEU	C	460	100.559	82.151	72.272	1.00	0.00	H
ATOM	3658	HB3	LEU	C	460	101.796	80.935	72.374	1.00	0.00	H
ATOM	3659	HG	LEU	C	460	100.342	79.576	73.889	1.00	0.00	H
ATOM	3660	HD11	LEU	C	460	97.803	80.334	73.859	1.00	0.00	H
ATOM	3661	HD12	LEU	C	460	98.915	80.996	75.046	1.00	0.00	H
ATOM	3662	HD13	LEU	C	460	98.478	81.946	73.636	1.00	0.00	H
ATOM	3663	HD21	LEU	C	460	99.618	78.526	72.055	1.00	0.00	H
ATOM	3664	HD22	LEU	C	460	98.426	79.769	71.713	1.00	0.00	H
ATOM	3665	HD23	LEU	C	460	100.054	79.921	71.084	1.00	0.00	H
ATOM	3666	N	PRO	C	461	102.157	81.378	76.491	1.00	30.00	N
ATOM	3667	CA	PRO	C	461	102.401	80.334	77.512	1.00	30.00	C
ATOM	3668	C	PRO	C	461	101.423	79.133	77.404	1.00	30.00	C
ATOM	3669	O	PRO	C	461	100.373	79.274	76.775	1.00	30.00	O
ATOM	3670	CB	PRO	C	461	102.186	81.084	78.845	1.00	20.00	C
ATOM	3671	CG	PRO	C	461	102.293	82.562	78.518	1.00	20.00	C
ATOM	3672	CD	PRO	C	461	101.740	82.638	77.104	1.00	20.00	C
ATOM	3673	HA	PRO	C	461	103.440	80.019	77.413	1.00	0.00	H
ATOM	3674	HB2	PRO	C	461	101.185	80.900	79.237	1.00	0.00	H
ATOM	3675	HB3	PRO	C	461	102.896	80.783	79.616	1.00	0.00	H
ATOM	3676	HG2	PRO	C	461	101.761	83.197	79.226	1.00	0.00	H
ATOM	3677	HG3	PRO	C	461	103.340	82.860	78.521	1.00	0.00	H
ATOM	3678	HD2	PRO	C	461	102.118	83.518	76.583	1.00	0.00	H
ATOM	3679	HD3	PRO	C	461	100.651	82.684	77.120	1.00	0.00	H
ATOM	3680	N	PRO	C	462	101.723	78.000	78.080	1.00	30.00	N

```
ATOM   3681  CA  PRO C 462     103.050  77.599  78.581  1.00 30.00           C
ATOM   3682  C   PRO C 462     103.985  77.173  77.429  1.00 30.00           C
ATOM   3683  O   PRO C 462     103.510  76.814  76.350  1.00 30.00           O
ATOM   3684  CB  PRO C 462     102.713  76.418  79.505  1.00 20.00           C
ATOM   3685  CG  PRO C 462     101.520  75.750  78.842  1.00 20.00           C
ATOM   3686  CD  PRO C 462     100.739  76.932  78.278  1.00 20.00           C
ATOM   3687  HA  PRO C 462     103.518  78.397  79.158  1.00  0.00           H
ATOM   3688  HB2 PRO C 462     103.545  75.730  79.663  1.00  0.00           H
ATOM   3689  HB3 PRO C 462     102.415  76.800  80.483  1.00  0.00           H
ATOM   3690  HG2 PRO C 462     101.865  75.117  78.024  1.00  0.00           H
ATOM   3691  HG3 PRO C 462     100.932  75.130  79.518  1.00  0.00           H
ATOM   3692  HD2 PRO C 462     100.224  76.658  77.356  1.00  0.00           H
ATOM   3693  HD3 PRO C 462      99.996  77.274  78.999  1.00  0.00           H
ATOM   3694  N   TYR C 463     105.300  77.236  77.679  1.00 30.00           N
ATOM   3695  CA  TYR C 463     106.333  77.015  76.664  1.00 30.00           C
ATOM   3696  C   TYR C 463     106.925  75.604  76.771  1.00 30.00           C
ATOM   3697  O   TYR C 463     107.202  75.139  77.878  1.00 30.00           O
ATOM   3698  CB  TYR C 463     107.430  78.084  76.831  1.00 20.00           C
ATOM   3699  CG  TYR C 463     106.893  79.503  76.798  1.00 20.00           C
ATOM   3700  CD1 TYR C 463     106.687  80.163  75.570  1.00 20.00           C
ATOM   3701  CD2 TYR C 463     106.558  80.151  78.004  1.00 20.00           C
ATOM   3702  CE1 TYR C 463     106.152  81.464  75.555  1.00 20.00           C
ATOM   3703  CE2 TYR C 463     106.011  81.448  77.989  1.00 20.00           C
ATOM   3704  CZ  TYR C 463     105.813  82.107  76.762  1.00 20.00           C
ATOM   3705  OH  TYR C 463     105.302  83.370  76.744  1.00 20.00           O
ATOM   3706  H   TYR C 463     105.627  77.516  78.594  1.00  0.00           H
ATOM   3707  HA  TYR C 463     105.908  77.139  75.665  1.00  0.00           H
ATOM   3708  HB2 TYR C 463     108.183  77.975  76.048  1.00  0.00           H
ATOM   3709  HB3 TYR C 463     107.957  77.938  77.776  1.00  0.00           H
ATOM   3710  HD1 TYR C 463     106.933  79.674  74.639  1.00  0.00           H
ATOM   3711  HD2 TYR C 463     106.721  79.642  78.939  1.00  0.00           H
ATOM   3712  HE1 TYR C 463     105.997  81.960  74.609  1.00  0.00           H
ATOM   3713  HE2 TYR C 463     105.754  81.937  78.917  1.00  0.00           H
ATOM   3714  HH  TYR C 463     105.400  83.780  75.878  1.00  0.00           H
ATOM   3715  N   LYS C 464     107.141  74.971  75.605  1.00 30.00           N
ATOM   3716  CA  LYS C 464     107.827  73.686  75.465  1.00 30.00           C
ATOM   3717  C   LYS C 464     109.342  73.878  75.642  1.00 30.00           C
ATOM   3718  O   LYS C 464     109.927  74.719  74.957  1.00 30.00           O
ATOM   3719  CB  LYS C 464     107.446  73.066  74.097  1.00  0.00           C
ATOM   3720  CG  LYS C 464     108.049  71.676  73.791  1.00  0.00           C
ATOM   3721  CD  LYS C 464     109.365  71.712  72.994  1.00  0.00           C
ATOM   3722  CE  LYS C 464     109.996  70.320  72.831  1.00  0.00           C
ATOM   3723  NZ  LYS C 464     111.355  70.414  72.270  1.00  0.00           N1+
ATOM   3724  H   LYS C 464     106.885  75.428  74.742  1.00  0.00           H
ATOM   3725  HA  LYS C 464     107.462  73.019  76.248  1.00  0.00           H
ATOM   3726  HB2 LYS C 464     106.360  72.960  74.083  1.00  0.00           H
ATOM   3727  HB3 LYS C 464     107.679  73.754  73.283  1.00  0.00           H
ATOM   3728  HG2 LYS C 464     108.198  71.133  74.725  1.00  0.00           H
ATOM   3729  HG3 LYS C 464     107.319  71.094  73.227  1.00  0.00           H
ATOM   3730  HD2 LYS C 464     109.167  72.129  72.006  1.00  0.00           H
ATOM   3731  HD3 LYS C 464     110.078  72.389  73.458  1.00  0.00           H
ATOM   3732  HE2 LYS C 464     110.066  69.821  73.798  1.00  0.00           H
ATOM   3733  HE3 LYS C 464     109.378  69.693  72.188  1.00  0.00           H
ATOM   3734  HZ1 LYS C 464     111.930  70.952  72.904  1.00  0.00           H
ATOM   3735  HZ2 LYS C 464     111.755  69.491  72.170  1.00  0.00           H
ATOM   3736  HZ3 LYS C 464     111.325  70.868  71.369  1.00  0.00           H
ATOM   3737  N   LEU C 465     109.943  73.085  76.547  1.00 30.00           N
ATOM   3738  CA  LEU C 465     111.379  73.095  76.831  1.00 30.00           C
ATOM   3739  C   LEU C 465     112.191  72.530  75.654  1.00 30.00           C
ATOM   3740  O   LEU C 465     111.879  71.453  75.144  1.00 30.00           O
ATOM   3741  CB  LEU C 465     111.668  72.406  78.189  1.00  0.00           C
ATOM   3742  CG  LEU C 465     111.659  70.856  78.254  1.00  0.00           C
ATOM   3743  CD1 LEU C 465     112.172  70.366  79.624  1.00  0.00           C
ATOM   3744  CD2 LEU C 465     110.284  70.240  77.923  1.00  0.00           C
ATOM   3745  H   LEU C 465     109.394  72.419  77.070  1.00  0.00           H
ATOM   3746  HA  LEU C 465     111.658  74.144  76.957  1.00  0.00           H
ATOM   3747  HB2 LEU C 465     112.658  72.743  78.501  1.00  0.00           H
ATOM   3748  HB3 LEU C 465     110.976  72.800  78.933  1.00  0.00           H
ATOM   3749  HG  LEU C 465     112.377  70.479  77.526  1.00  0.00           H
ATOM   3750 HD11 LEU C 465     112.844  69.517  79.501  1.00  0.00           H
ATOM   3751 HD12 LEU C 465     112.728  71.137  80.159  1.00  0.00           H
```

```
ATOM   3752 HD13 LEU C 465     111.360  70.048  80.278  1.00  0.00           H
ATOM   3753 HD21 LEU C 465     110.091  69.336  78.502  1.00  0.00           H
ATOM   3754 HD22 LEU C 465     109.467  70.931  78.129  1.00  0.00           H
ATOM   3755 HD23 LEU C 465     110.228  69.961  76.871  1.00  0.00           H
ATOM   3756  N   LYS C 466     113.207  73.293  75.234  1.00 30.00           N
ATOM   3757  CA  LYS C 466     114.062  72.988  74.090  1.00 30.00           C
ATOM   3758  C   LYS C 466     115.135  71.960  74.492  1.00 30.00           C
ATOM   3759  O   LYS C 466     115.491  71.862  75.667  1.00 30.00           O
ATOM   3760  CB  LYS C 466     114.656  74.311  73.553  1.00  0.00           C
ATOM   3761  CG  LYS C 466     113.572  75.323  73.111  1.00  0.00           C
ATOM   3762  CD  LYS C 466     114.096  76.749  72.872  1.00  0.00           C
ATOM   3763  CE  LYS C 466     112.948  77.747  72.625  1.00  0.00           C
ATOM   3764  NZ  LYS C 466     113.432  79.132  72.492  1.00  0.00           N1+
ATOM   3765  H   LYS C 466     113.439  74.146  75.734  1.00  0.00           H
ATOM   3766  HA  LYS C 466     113.448  72.549  73.302  1.00  0.00           H
ATOM   3767  HB2 LYS C 466     115.278  74.764  74.323  1.00  0.00           H
ATOM   3768  HB3 LYS C 466     115.323  74.111  72.713  1.00  0.00           H
ATOM   3769  HG2 LYS C 466     113.088  74.957  72.205  1.00  0.00           H
ATOM   3770  HG3 LYS C 466     112.784  75.383  73.861  1.00  0.00           H
ATOM   3771  HD2 LYS C 466     114.681  77.069  73.736  1.00  0.00           H
ATOM   3772  HD3 LYS C 466     114.776  76.749  72.020  1.00  0.00           H
ATOM   3773  HE2 LYS C 466     112.394  77.476  71.725  1.00  0.00           H
ATOM   3774  HE3 LYS C 466     112.242  77.717  73.456  1.00  0.00           H
ATOM   3775  HZ1 LYS C 466     112.643  79.762  72.376  1.00  0.00           H
ATOM   3776  HZ2 LYS C 466     113.935  79.396  73.326  1.00  0.00           H
ATOM   3777  HZ3 LYS C 466     114.041  79.210  71.690  1.00  0.00           H
ATOM   3778  N   ASN C 467     115.608  71.183  73.505  1.00 30.00           N
ATOM   3779  CA  ASN C 467     116.524  70.050  73.713  1.00 30.00           C
ATOM   3780  C   ASN C 467     117.980  70.461  74.016  1.00 30.00           C
ATOM   3781  O   ASN C 467     118.765  69.587  74.384  1.00 30.00           O
ATOM   3782  CB  ASN C 467     116.433  69.088  72.506  1.00  0.00           C
ATOM   3783  CG  ASN C 467     115.064  68.404  72.398  1.00  0.00           C
ATOM   3784  ND2 ASN C 467     114.971  67.152  72.853  1.00  0.00           N
ATOM   3785  OD1 ASN C 467     114.102  68.997  71.915  1.00  0.00           O
ATOM   3786  H   ASN C 467     115.272  71.312  72.562  1.00  0.00           H
ATOM   3787  HA  ASN C 467     116.148  69.518  74.589  1.00  0.00           H
ATOM   3788  HB2 ASN C 467     116.646  69.619  71.576  1.00  0.00           H
ATOM   3789  HB3 ASN C 467     117.193  68.309  72.589  1.00  0.00           H
ATOM   3790 HD21 ASN C 467     114.092  66.658  72.803  1.00  0.00           H
ATOM   3791 HD22 ASN C 467     115.777  66.687  73.245  1.00  0.00           H
ATOM   3792  N   THR C 468     118.309  71.761  73.913  1.00 30.00           N
ATOM   3793  CA  THR C 468     119.561  72.345  74.400  1.00 30.00           C
ATOM   3794  C   THR C 468     119.628  72.316  75.943  1.00 30.00           C
ATOM   3795  O   THR C 468     118.608  72.524  76.596  1.00 30.00           O
ATOM   3796  CB  THR C 468     119.729  73.808  73.895  1.00  0.00           C
ATOM   3797  CG2 THR C 468     118.620  74.788  74.328  1.00  0.00           C
ATOM   3798  OG1 THR C 468     120.955  74.365  74.337  1.00  0.00           O
ATOM   3799  H   THR C 468     117.601  72.421  73.626  1.00  0.00           H
ATOM   3800  HA  THR C 468     120.385  71.747  74.003  1.00  0.00           H
ATOM   3801  HB  THR C 468     119.761  73.789  72.806  1.00  0.00           H
ATOM   3802  HG1 THR C 468     120.987  75.286  74.066  1.00  0.00           H
ATOM   3803 HG21 THR C 468     118.753  75.762  73.857  1.00  0.00           H
ATOM   3804 HG22 THR C 468     117.635  74.423  74.043  1.00  0.00           H
ATOM   3805 HG23 THR C 468     118.612  74.950  75.407  1.00  0.00           H
ATOM   3806  N   VAL C 469     120.830  72.064  76.488  1.00 30.00           N
ATOM   3807  CA  VAL C 469     121.104  71.926  77.925  1.00 30.00           C
ATOM   3808  C   VAL C 469     120.846  73.193  78.781  1.00 30.00           C
ATOM   3809  O   VAL C 469     120.782  73.075  80.005  1.00 30.00           O
ATOM   3810  CB  VAL C 469     122.573  71.471  78.166  1.00  0.00           C
ATOM   3811  CG1 VAL C 469     122.837  70.076  77.572  1.00  0.00           C
ATOM   3812  CG2 VAL C 469     123.641  72.481  77.697  1.00  0.00           C
ATOM   3813  H   VAL C 469     121.623  71.923  75.879  1.00  0.00           H
ATOM   3814  HA  VAL C 469     120.448  71.143  78.305  1.00  0.00           H
ATOM   3815  HB  VAL C 469     122.710  71.356  79.243  1.00  0.00           H
ATOM   3816 HG11 VAL C 469     123.839  69.727  77.822  1.00  0.00           H
ATOM   3817 HG12 VAL C 469     122.130  69.343  77.960  1.00  0.00           H
ATOM   3818 HG13 VAL C 469     122.752  70.078  76.485  1.00  0.00           H
ATOM   3819 HG21 VAL C 469     124.646  72.093  77.871  1.00  0.00           H
ATOM   3820 HG22 VAL C 469     123.553  72.691  76.631  1.00  0.00           H
ATOM   3821 HG23 VAL C 469     123.572  73.429  78.230  1.00  0.00           H
ATOM   3822  N   GLY C 470     120.709  74.365  78.135  1.00 30.00           N
```

ATOM	3823	CA	GLY	C	470	120.592	75.670	78.783	1.00	30.00	C
ATOM	3824	C	GLY	C	470	119.262	75.852	79.530	1.00	30.00	C
ATOM	3825	O	GLY	C	470	119.288	76.238	80.698	1.00	30.00	O
ATOM	3826	H	GLY	C	470	120.748	74.363	77.125	1.00	0.00	H
ATOM	3827	HA2	GLY	C	470	121.427	75.813	79.470	1.00	0.00	H
ATOM	3828	HA3	GLY	C	470	120.673	76.445	78.021	1.00	0.00	H
ATOM	3829	N	ASP	C	471	118.113	75.580	78.880	1.00	30.00	N
ATOM	3830	CA	ASP	C	471	116.780	75.808	79.462	1.00	30.00	C
ATOM	3831	C	ASP	C	471	116.287	74.662	80.370	1.00	30.00	C
ATOM	3832	O	ASP	C	471	115.216	74.803	80.956	1.00	30.00	O
ATOM	3833	CB	ASP	C	471	115.684	76.264	78.460	1.00	20.00	C
ATOM	3834	CG	ASP	C	471	115.438	75.389	77.228	1.00	20.00	C
ATOM	3835	OD1	ASP	C	471	116.327	74.588	76.870	1.00	20.00	O
ATOM	3836	OD2	ASP	C	471	114.363	75.593	76.622	1.00	20.00	O1-
ATOM	3837	H	ASP	C	471	118.123	75.222	77.933	1.00	0.00	H
ATOM	3838	HA	ASP	C	471	116.880	76.657	80.140	1.00	0.00	H
ATOM	3839	HB2	ASP	C	471	115.950	77.257	78.097	1.00	0.00	H
ATOM	3840	HB3	ASP	C	471	114.735	76.375	78.987	1.00	0.00	H
ATOM	3841	N	TYR	C	472	117.084	73.594	80.560	1.00	0.00	N
ATOM	3842	CA	TYR	C	472	116.875	72.642	81.658	1.00	0.00	C
ATOM	3843	C	TYR	C	472	117.199	73.271	83.024	1.00	0.00	C
ATOM	3844	O	TYR	C	472	116.505	72.958	83.988	1.00	0.00	O
ATOM	3845	CB	TYR	C	472	117.667	71.342	81.428	1.00	20.00	C
ATOM	3846	CG	TYR	C	472	117.109	70.481	80.310	1.00	20.00	C
ATOM	3847	CD1	TYR	C	472	116.133	69.497	80.577	1.00	20.00	C
ATOM	3848	CD2	TYR	C	472	117.550	70.680	78.989	1.00	20.00	C
ATOM	3849	CE1	TYR	C	472	115.608	68.720	79.525	1.00	20.00	C
ATOM	3850	CE2	TYR	C	472	117.026	69.907	77.937	1.00	20.00	C
ATOM	3851	CZ	TYR	C	472	116.050	68.928	78.203	1.00	20.00	C
ATOM	3852	OH	TYR	C	472	115.531	68.185	77.183	1.00	20.00	O
ATOM	3853	H	TYR	C	472	117.947	73.511	80.041	1.00	0.00	H
ATOM	3854	HA	TYR	C	472	115.815	72.376	81.672	1.00	0.00	H
ATOM	3855	HB2	TYR	C	472	118.718	71.563	81.236	1.00	0.00	H
ATOM	3856	HB3	TYR	C	472	117.652	70.738	82.337	1.00	0.00	H
ATOM	3857	HD1	TYR	C	472	115.782	69.339	81.586	1.00	0.00	H
ATOM	3858	HD2	TYR	C	472	118.282	71.443	78.789	1.00	0.00	H
ATOM	3859	HE1	TYR	C	472	114.860	67.969	79.734	1.00	0.00	H
ATOM	3860	HE2	TYR	C	472	117.368	70.077	76.927	1.00	0.00	H
ATOM	3861	HH	TYR	C	472	115.896	68.427	76.329	1.00	0.00	H
ATOM	3862	N	PHE	C	473	118.175	74.197	83.076	1.00	0.00	N
ATOM	3863	CA	PHE	C	473	118.452	75.024	84.256	1.00	0.00	C
ATOM	3864	C	PHE	C	473	117.372	76.090	84.517	1.00	0.00	C
ATOM	3865	O	PHE	C	473	117.174	76.452	85.676	1.00	0.00	O
ATOM	3866	CB	PHE	C	473	119.848	75.671	84.146	1.00	20.00	C
ATOM	3867	CG	PHE	C	473	121.030	74.714	84.098	1.00	20.00	C
ATOM	3868	CD1	PHE	C	473	121.122	73.617	84.986	1.00	20.00	C
ATOM	3869	CD2	PHE	C	473	122.132	75.008	83.267	1.00	20.00	C
ATOM	3870	CE1	PHE	C	473	122.253	72.812	84.984	1.00	20.00	C
ATOM	3871	CE2	PHE	C	473	123.257	74.194	83.285	1.00	20.00	C
ATOM	3872	CZ	PHE	C	473	123.314	73.098	84.136	1.00	20.00	C
ATOM	3873	H	PHE	C	473	118.708	74.414	82.245	1.00	0.00	H
ATOM	3874	HA	PHE	C	473	118.447	74.376	85.131	1.00	0.00	H
ATOM	3875	HB2	PHE	C	473	119.875	76.295	83.253	1.00	0.00	H
ATOM	3876	HB3	PHE	C	473	120.013	76.350	84.984	1.00	0.00	H
ATOM	3877	HD1	PHE	C	473	120.323	73.392	85.676	1.00	0.00	H
ATOM	3878	HD2	PHE	C	473	122.106	75.865	82.610	1.00	0.00	H
ATOM	3879	HE1	PHE	C	473	122.312	71.967	85.654	1.00	0.00	H
ATOM	3880	HE2	PHE	C	473	124.092	74.417	82.637	1.00	0.00	H
ATOM	3881	HZ	PHE	C	473	124.194	72.470	84.145	1.00	0.00	H
ATOM	3882	N	ARG	C	474	116.670	76.541	83.460	1.00	30.00	N
ATOM	3883	CA	ARG	C	474	115.500	77.412	83.576	1.00	30.00	C
ATOM	3884	C	ARG	C	474	114.289	76.654	84.149	1.00	30.00	C
ATOM	3885	O	ARG	C	474	113.639	77.186	85.042	1.00	30.00	O
ATOM	3886	CB	ARG	C	474	115.156	78.049	82.211	1.00	20.00	C
ATOM	3887	CG	ARG	C	474	114.001	79.069	82.274	1.00	20.00	C
ATOM	3888	CD	ARG	C	474	113.205	79.165	80.967	1.00	20.00	C
ATOM	3889	NE	ARG	C	474	111.952	79.897	81.184	1.00	20.00	N
ATOM	3890	CZ	ARG	C	474	110.799	79.359	81.621	1.00	20.00	C
ATOM	3891	NH1	ARG	C	474	109.742	80.151	81.838	1.00	20.00	N
ATOM	3892	NH2	ARG	C	474	110.686	78.041	81.849	1.00	20.00	N1+
ATOM	3893	H	ARG	C	474	116.892	76.204	82.534	1.00	0.00	H

```
ATOM   3894  HA  ARG C 474     115.748  78.222  84.266  1.00  0.00           H
ATOM   3895  HB2 ARG C 474     116.033  78.516  81.761  1.00  0.00           H
ATOM   3896  HB3 ARG C 474     114.872  77.246  81.533  1.00  0.00           H
ATOM   3897  HG2 ARG C 474     113.294  78.882  83.082  1.00  0.00           H
ATOM   3898  HG3 ARG C 474     114.450  80.035  82.508  1.00  0.00           H
ATOM   3899  HD2 ARG C 474     113.769  79.807  80.293  1.00  0.00           H
ATOM   3900  HD3 ARG C 474     113.083  78.218  80.439  1.00  0.00           H
ATOM   3901  HE  ARG C 474     112.016  80.900  81.089  1.00  0.00           H
ATOM   3902 HH11 ARG C 474     108.869  79.758  82.162  1.00  0.00           H
ATOM   3903 HH12 ARG C 474     109.810  81.146  81.690  1.00  0.00           H
ATOM   3904 HH21 ARG C 474     109.817  77.648  82.183  1.00  0.00           H
ATOM   3905 HH22 ARG C 474     111.476  77.434  81.691  1.00  0.00           H
ATOM   3906  N   VAL C 475     114.002  75.450  83.622  1.00  0.00           N
ATOM   3907  CA  VAL C 475     112.855  74.619  84.001  1.00  0.00           C
ATOM   3908  C   VAL C 475     112.974  74.033  85.422  1.00  0.00           C
ATOM   3909  O   VAL C 475     111.992  74.075  86.163  1.00  0.00           O
ATOM   3910  CB  VAL C 475     112.615  73.490  82.956  1.00 20.00           C
ATOM   3911  CG1 VAL C 475     111.719  72.316  83.410  1.00 20.00           C
ATOM   3912  CG2 VAL C 475     112.038  74.087  81.661  1.00 20.00           C
ATOM   3913  H   VAL C 475     114.578  75.090  82.873  1.00  0.00           H
ATOM   3914  HA  VAL C 475     111.975  75.266  84.000  1.00  0.00           H
ATOM   3915  HB  VAL C 475     113.587  73.062  82.702  1.00  0.00           H
ATOM   3916 HG11 VAL C 475     111.522  71.632  82.583  1.00  0.00           H
ATOM   3917 HG12 VAL C 475     112.186  71.723  84.197  1.00  0.00           H
ATOM   3918 HG13 VAL C 475     110.757  72.671  83.779  1.00  0.00           H
ATOM   3919 HG21 VAL C 475     111.986  73.330  80.883  1.00  0.00           H
ATOM   3920 HG22 VAL C 475     111.031  74.473  81.817  1.00  0.00           H
ATOM   3921 HG23 VAL C 475     112.647  74.905  81.277  1.00  0.00           H
ATOM   3922  N   THR C 476     114.176  73.558  85.795  1.00  0.00           N
ATOM   3923  CA  THR C 476     114.485  73.135  87.165  1.00  0.00           C
ATOM   3924  C   THR C 476     114.588  74.326  88.144  1.00  0.00           C
ATOM   3925  O   THR C 476     114.348  74.119  89.329  1.00  0.00           O
ATOM   3926  CB  THR C 476     115.794  72.311  87.268  1.00 20.00           C
ATOM   3927  CG2 THR C 476     115.737  70.990  86.485  1.00 20.00           C
ATOM   3928  OG1 THR C 476     116.929  73.054  86.866  1.00 20.00           O
ATOM   3929  H   THR C 476     114.941  73.536  85.133  1.00  0.00           H
ATOM   3930  HA  THR C 476     113.676  72.486  87.499  1.00  0.00           H
ATOM   3931  HB  THR C 476     115.961  72.051  88.315  1.00  0.00           H
ATOM   3932  HG1 THR C 476     116.935  73.095  85.905  1.00  0.00           H
ATOM   3933 HG21 THR C 476     116.687  70.458  86.540  1.00  0.00           H
ATOM   3934 HG22 THR C 476     114.971  70.333  86.898  1.00  0.00           H
ATOM   3935 HG23 THR C 476     115.498  71.137  85.433  1.00  0.00           H
ATOM   3936  N   GLY C 477     114.879  75.542  87.647  1.00  0.00           N
ATOM   3937  CA  GLY C 477     114.877  76.764  88.452  1.00  0.00           C
ATOM   3938  C   GLY C 477     113.451  77.307  88.643  1.00  0.00           C
ATOM   3939  O   GLY C 477     113.166  77.906  89.678  1.00  0.00           O
ATOM   3940  H   GLY C 477     115.084  75.642  86.663  1.00  0.00           H
ATOM   3941  HA2 GLY C 477     115.340  76.575  89.420  1.00  0.00           H
ATOM   3942  HA3 GLY C 477     115.479  77.520  87.948  1.00  0.00           H
ATOM   3943  N   GLU C 478     112.561  77.084  87.659  1.00  0.00           N
ATOM   3944  CA  GLU C 478     111.158  77.494  87.660  1.00  0.00           C
ATOM   3945  C   GLU C 478     110.337  76.669  88.659  1.00  0.00           C
ATOM   3946  O   GLU C 478     109.635  77.262  89.473  1.00  0.00           O
ATOM   3947  CB  GLU C 478     110.619  77.495  86.206  1.00 20.00           C
ATOM   3948  CG  GLU C 478     109.141  77.907  85.985  1.00 20.00           C
ATOM   3949  CD  GLU C 478     108.087  76.832  86.276  1.00 20.00           C
ATOM   3950  OE1 GLU C 478     108.390  75.640  86.052  1.00 20.00           O
ATOM   3951  OE2 GLU C 478     106.982  77.230  86.705  1.00 20.00           O1-
ATOM   3952  H   GLU C 478     112.873  76.612  86.821  1.00  0.00           H
ATOM   3953  HA  GLU C 478     111.127  78.530  88.004  1.00  0.00           H
ATOM   3954  HB2 GLU C 478     111.229  78.192  85.630  1.00  0.00           H
ATOM   3955  HB3 GLU C 478     110.802  76.523  85.747  1.00  0.00           H
ATOM   3956  HG2 GLU C 478     108.919  78.801  86.569  1.00  0.00           H
ATOM   3957  HG3 GLU C 478     109.011  78.191  84.940  1.00  0.00           H
ATOM   3958  N   ILE C 479     110.476  75.332  88.626  1.00  0.00           N
ATOM   3959  CA  ILE C 479     109.789  74.433  89.559  1.00  0.00           C
ATOM   3960  C   ILE C 479     110.320  74.546  91.010  1.00  0.00           C
ATOM   3961  O   ILE C 479     109.546  74.308  91.935  1.00  0.00           O
ATOM   3962  CB  ILE C 479     109.837  72.955  89.074  1.00 20.00           C
ATOM   3963  CG1 ILE C 479     108.771  72.070  89.762  1.00 20.00           C
ATOM   3964  CG2 ILE C 479     111.230  72.309  89.181  1.00 20.00           C
```

ATOM	3965	CD1	ILE	C	479	108.537	70.727	89.058	1.00	20.00	C
ATOM	3966	H	ILE	C	479	111.053	74.900	87.917	1.00	0.00	H
ATOM	3967	HA	ILE	C	479	108.742	74.741	89.571	1.00	0.00	H
ATOM	3968	HB	ILE	C	479	109.587	72.985	88.012	1.00	0.00	H
ATOM	3969	HG12	ILE	C	479	109.052	71.882	90.799	1.00	0.00	H
ATOM	3970	HG13	ILE	C	479	107.821	72.605	89.803	1.00	0.00	H
ATOM	3971	HG21	ILE	C	479	111.288	71.388	88.601	1.00	0.00	H
ATOM	3972	HG22	ILE	C	479	111.988	72.986	88.798	1.00	0.00	H
ATOM	3973	HG23	ILE	C	479	111.490	72.067	90.211	1.00	0.00	H
ATOM	3974	HD11	ILE	C	479	107.589	70.288	89.369	1.00	0.00	H
ATOM	3975	HD12	ILE	C	479	108.508	70.841	87.974	1.00	0.00	H
ATOM	3976	HD13	ILE	C	479	109.324	70.012	89.300	1.00	0.00	H
ATOM	3977	N	LEU	C	480	111.587	74.971	91.190	1.00	0.00	N
ATOM	3978	CA	LEU	C	480	112.178	75.307	92.492	1.00	0.00	C
ATOM	3979	C	LEU	C	480	111.619	76.623	93.062	1.00	0.00	C
ATOM	3980	O	LEU	C	480	111.391	76.692	94.270	1.00	0.00	O
ATOM	3981	CB	LEU	C	480	113.724	75.367	92.390	1.00	20.00	C
ATOM	3982	CG	LEU	C	480	114.466	74.117	92.922	1.00	20.00	C
ATOM	3983	CD1	LEU	C	480	114.340	73.991	94.456	1.00	20.00	C
ATOM	3984	CD2	LEU	C	480	114.106	72.809	92.186	1.00	20.00	C
ATOM	3985	H	LEU	C	480	112.170	75.139	90.381	1.00	0.00	H
ATOM	3986	HA	LEU	C	480	111.905	74.516	93.192	1.00	0.00	H
ATOM	3987	HB2	LEU	C	480	114.023	75.574	91.364	1.00	0.00	H
ATOM	3988	HB3	LEU	C	480	114.106	76.223	92.950	1.00	0.00	H
ATOM	3989	HG	LEU	C	480	115.523	74.302	92.721	1.00	0.00	H
ATOM	3990	HD11	LEU	C	480	115.321	73.868	94.916	1.00	0.00	H
ATOM	3991	HD12	LEU	C	480	113.888	74.877	94.903	1.00	0.00	H
ATOM	3992	HD13	LEU	C	480	113.730	73.140	94.760	1.00	0.00	H
ATOM	3993	HD21	LEU	C	480	113.737	72.033	92.856	1.00	0.00	H
ATOM	3994	HD22	LEU	C	480	113.339	72.957	91.429	1.00	0.00	H
ATOM	3995	HD23	LEU	C	480	114.980	72.401	91.677	1.00	0.00	H
ATOM	3996	N	SER	C	481	111.386	77.627	92.195	1.00	0.00	N
ATOM	3997	CA	SER	C	481	110.759	78.898	92.571	1.00	0.00	C
ATOM	3998	C	SER	C	481	109.273	78.737	92.956	1.00	0.00	C
ATOM	3999	O	SER	C	481	108.817	79.402	93.888	1.00	0.00	O
ATOM	4000	CB	SER	C	481	111.018	79.969	91.492	1.00	20.00	C
ATOM	4001	OG	SER	C	481	110.159	79.898	90.373	1.00	20.00	O
ATOM	4002	H	SER	C	481	111.622	77.513	91.219	1.00	0.00	H
ATOM	4003	HA	SER	C	481	111.293	79.247	93.452	1.00	0.00	H
ATOM	4004	HB2	SER	C	481	112.056	79.944	91.158	1.00	0.00	H
ATOM	4005	HB3	SER	C	481	110.861	80.947	91.942	1.00	0.00	H
ATOM	4006	HG	SER	C	481	110.239	79.028	89.970	1.00	0.00	H
ATOM	4007	N	VAL	C	482	108.572	77.816	92.271	1.00	0.00	N
ATOM	4008	CA	VAL	C	482	107.197	77.409	92.558	1.00	0.00	C
ATOM	4009	C	VAL	C	482	107.086	76.561	93.841	1.00	0.00	C
ATOM	4010	O	VAL	C	482	106.082	76.691	94.537	1.00	0.00	O
ATOM	4011	CB	VAL	C	482	106.586	76.629	91.354	1.00	20.00	C
ATOM	4012	CG1	VAL	C	482	105.271	75.868	91.638	1.00	20.00	C
ATOM	4013	CG2	VAL	C	482	106.373	77.571	90.158	1.00	20.00	C
ATOM	4014	H	VAL	C	482	109.019	77.336	91.501	1.00	0.00	H
ATOM	4015	HA	VAL	C	482	106.607	78.314	92.715	1.00	0.00	H
ATOM	4016	HB	VAL	C	482	107.313	75.881	91.038	1.00	0.00	H
ATOM	4017	HG11	VAL	C	482	104.845	75.465	90.719	1.00	0.00	H
ATOM	4018	HG12	VAL	C	482	105.423	75.020	92.306	1.00	0.00	H
ATOM	4019	HG13	VAL	C	482	104.524	76.522	92.089	1.00	0.00	H
ATOM	4020	HG21	VAL	C	482	105.981	77.035	89.294	1.00	0.00	H
ATOM	4021	HG22	VAL	C	482	105.671	78.364	90.412	1.00	0.00	H
ATOM	4022	HG23	VAL	C	482	107.294	78.055	89.843	1.00	0.00	H
ATOM	4023	N	SER	C	483	108.112	75.748	94.156	1.00	0.00	N
ATOM	4024	CA	SER	C	483	108.166	74.912	95.361	1.00	0.00	C
ATOM	4025	C	SER	C	483	108.255	75.722	96.668	1.00	0.00	C
ATOM	4026	O	SER	C	483	107.697	75.284	97.674	1.00	0.00	O
ATOM	4027	CB	SER	C	483	109.332	73.908	95.263	1.00	20.00	C
ATOM	4028	OG	SER	C	483	109.001	72.852	94.385	1.00	20.00	O
ATOM	4029	H	SER	C	483	108.901	75.679	93.529	1.00	0.00	H
ATOM	4030	HA	SER	C	483	107.234	74.345	95.416	1.00	0.00	H
ATOM	4031	HB2	SER	C	483	109.543	73.462	96.236	1.00	0.00	H
ATOM	4032	HB3	SER	C	483	110.251	74.391	94.933	1.00	0.00	H
ATOM	4033	HG	SER	C	483	109.027	73.189	93.484	1.00	0.00	H
ATOM	4034	N	GLY	C	484	108.895	76.904	96.620	1.00	0.00	N
ATOM	4035	CA	GLY	C	484	108.899	77.860	97.729	1.00	0.00	C

```
ATOM   4036  C   GLY C 484     107.574  78.635  97.788  1.00  0.00           C
ATOM   4037  O   GLY C 484     107.136  78.990  98.879  1.00  0.00           O
ATOM   4038  H   GLY C 484     109.342  77.190  95.760  1.00  0.00           H
ATOM   4039  HA2 GLY C 484     109.087  77.348  98.672  1.00  0.00           H
ATOM   4040  HA3 GLY C 484     109.713  78.567  97.604  1.00  0.00           H
ATOM   4041  N   GLY C 485     106.908  78.844  96.636  1.00  0.00           N
ATOM   4042  CA  GLY C 485     105.571  79.437  96.535  1.00  0.00           C
ATOM   4043  C   GLY C 485     104.469  78.500  97.062  1.00  0.00           C
ATOM   4044  O   GLY C 485     103.384  78.975  97.386  1.00  0.00           O
ATOM   4045  H   GLY C 485     107.318  78.518  95.772  1.00  0.00           H
ATOM   4046  HA2 GLY C 485     105.543  80.387  97.070  1.00  0.00           H
ATOM   4047  HA3 GLY C 485     105.364  79.650  95.487  1.00  0.00           H
ATOM   4048  N   VAL C 486     104.747  77.191  97.172  1.00  0.00           N
ATOM   4049  CA  VAL C 486     103.890  76.191  97.806  1.00  0.00           C
ATOM   4050  C   VAL C 486     104.214  76.067  99.309  1.00  0.00           C
ATOM   4051  O   VAL C 486     103.285  75.955 100.110  1.00  0.00           O
ATOM   4052  CB  VAL C 486     104.049  74.808  97.103  1.00 20.00           C
ATOM   4053  CG1 VAL C 486     103.497  73.595  97.880  1.00 20.00           C
ATOM   4054  CG2 VAL C 486     103.406  74.838  95.704  1.00 20.00           C
ATOM   4055  H   VAL C 486     105.639  76.856  96.836  1.00  0.00           H
ATOM   4056  HA  VAL C 486     102.845  76.498  97.716  1.00  0.00           H
ATOM   4057  HB  VAL C 486     105.115  74.628  96.955  1.00  0.00           H
ATOM   4058 HG11 VAL C 486     103.551  72.686  97.281  1.00  0.00           H
ATOM   4059 HG12 VAL C 486     104.056  73.397  98.796  1.00  0.00           H
ATOM   4060 HG13 VAL C 486     102.453  73.753  98.146  1.00  0.00           H
ATOM   4061 HG21 VAL C 486     103.598  73.913  95.161  1.00  0.00           H
ATOM   4062 HG22 VAL C 486     102.325  74.964  95.773  1.00  0.00           H
ATOM   4063 HG23 VAL C 486     103.789  75.655  95.096  1.00  0.00           H
ATOM   4064  N   TYR C 487     105.511  76.117  99.668  1.00  0.00           N
ATOM   4065  CA  TYR C 487     105.998  76.026 101.047  1.00  0.00           C
ATOM   4066  C   TYR C 487     105.543  77.201 101.927  1.00  0.00           C
ATOM   4067  O   TYR C 487     104.972  76.953 102.986  1.00  0.00           O
ATOM   4068  CB  TYR C 487     107.532  75.852 101.057  1.00 20.00           C
ATOM   4069  CG  TYR C 487     108.180  75.847 102.435  1.00 20.00           C
ATOM   4070  CD1 TYR C 487     107.920  74.790 103.331  1.00 20.00           C
ATOM   4071  CD2 TYR C 487     109.022  76.908 102.832  1.00 20.00           C
ATOM   4072  CE1 TYR C 487     108.493  74.795 104.618  1.00 20.00           C
ATOM   4073  CE2 TYR C 487     109.600  76.910 104.116  1.00 20.00           C
ATOM   4074  CZ  TYR C 487     109.334  75.854 105.011  1.00 20.00           C
ATOM   4075  OH  TYR C 487     109.883  75.857 106.260  1.00 20.00           O
ATOM   4076  H   TYR C 487     106.220  76.197  98.952  1.00  0.00           H
ATOM   4077  HA  TYR C 487     105.565  75.118 101.470  1.00  0.00           H
ATOM   4078  HB2 TYR C 487     107.802  74.926 100.548  1.00  0.00           H
ATOM   4079  HB3 TYR C 487     107.983  76.656 100.477  1.00  0.00           H
ATOM   4080  HD1 TYR C 487     107.273  73.976 103.038  1.00  0.00           H
ATOM   4081  HD2 TYR C 487     109.223  77.728 102.156  1.00  0.00           H
ATOM   4082  HE1 TYR C 487     108.285  73.986 105.302  1.00  0.00           H
ATOM   4083  HE2 TYR C 487     110.244  77.726 104.409  1.00  0.00           H
ATOM   4084  HH  TYR C 487     110.475  76.596 106.416  1.00  0.00           H
ATOM   4085  N   PHE C 488     105.778  78.443 101.468  1.00  0.00           N
ATOM   4086  CA  PHE C 488     105.384  79.672 102.166  1.00  0.00           C
ATOM   4087  C   PHE C 488     103.864  79.918 102.199  1.00  0.00           C
ATOM   4088  O   PHE C 488     103.400  80.650 103.073  1.00  0.00           O
ATOM   4089  CB  PHE C 488     106.142  80.880 101.576  1.00 20.00           C
ATOM   4090  CG  PHE C 488     107.648  80.872 101.789  1.00 20.00           C
ATOM   4091  CD1 PHE C 488     108.194  80.702 103.081  1.00 20.00           C
ATOM   4092  CD2 PHE C 488     108.520  81.123 100.707  1.00 20.00           C
ATOM   4093  CE1 PHE C 488     109.570  80.708 103.258  1.00 20.00           C
ATOM   4094  CE2 PHE C 488     109.894  81.113 100.904  1.00 20.00           C
ATOM   4095  CZ  PHE C 488     110.415  80.897 102.173  1.00 20.00           C
ATOM   4096  H   PHE C 488     106.255  78.568 100.583  1.00  0.00           H
ATOM   4097  HA  PHE C 488     105.681  79.563 103.207  1.00  0.00           H
ATOM   4098  HB2 PHE C 488     105.959  80.907 100.502  1.00  0.00           H
ATOM   4099  HB3 PHE C 488     105.752  81.816 101.978  1.00  0.00           H
ATOM   4100  HD1 PHE C 488     107.551  80.560 103.937  1.00  0.00           H
ATOM   4101  HD2 PHE C 488     108.128  81.301  99.717  1.00  0.00           H
ATOM   4102  HE1 PHE C 488     109.986  80.564 104.244  1.00  0.00           H
ATOM   4103  HE2 PHE C 488     110.558  81.274 100.068  1.00  0.00           H
ATOM   4104  HZ  PHE C 488     111.483  80.886 102.324  1.00  0.00           H
ATOM   4105  N   PHE C 489     103.120  79.275 101.282  1.00  0.00           N
ATOM   4106  CA  PHE C 489     101.659  79.255 101.258  1.00  0.00           C
```

ATOM	4107	C	PHE	C	489	101.084	78.397	102.394	1.00	0.00	C
ATOM	4108	O	PHE	C	489	100.261	78.897	103.159	1.00	0.00	O
ATOM	4109	CB	PHE	C	489	101.184	78.837	99.852	1.00	20.00	C
ATOM	4110	CG	PHE	C	489	99.688	78.708	99.636	1.00	20.00	C
ATOM	4111	CD1	PHE	C	489	98.901	79.863	99.441	1.00	20.00	C
ATOM	4112	CD2	PHE	C	489	99.055	77.449	99.724	1.00	20.00	C
ATOM	4113	CE1	PHE	C	489	97.530	79.745	99.262	1.00	20.00	C
ATOM	4114	CE2	PHE	C	489	97.682	77.353	99.539	1.00	20.00	C
ATOM	4115	CZ	PHE	C	489	96.925	78.494	99.299	1.00	20.00	C
ATOM	4116	H	PHE	C	489	103.580	78.696	100.595	1.00	0.00	H
ATOM	4117	HA	PHE	C	489	101.306	80.273	101.424	1.00	0.00	H
ATOM	4118	HB2	PHE	C	489	101.539	79.584	99.144	1.00	0.00	H
ATOM	4119	HB3	PHE	C	489	101.657	77.903	99.553	1.00	0.00	H
ATOM	4120	HD1	PHE	C	489	99.366	80.838	99.411	1.00	0.00	H
ATOM	4121	HD2	PHE	C	489	99.636	76.559	99.917	1.00	0.00	H
ATOM	4122	HE1	PHE	C	489	96.932	80.628	99.094	1.00	0.00	H
ATOM	4123	HE2	PHE	C	489	97.201	76.387	99.587	1.00	0.00	H
ATOM	4124	HZ	PHE	C	489	95.858	78.410	99.159	1.00	0.00	H
ATOM	4125	N	PHE	C	490	101.573	77.150	102.520	1.00	0.00	N
ATOM	4126	CA	PHE	C	490	101.225	76.239	103.614	1.00	0.00	C
ATOM	4127	C	PHE	C	490	101.758	76.669	104.991	1.00	0.00	C
ATOM	4128	O	PHE	C	490	101.075	76.428	105.985	1.00	0.00	O
ATOM	4129	CB	PHE	C	490	101.666	74.799	103.277	1.00	20.00	C
ATOM	4130	CG	PHE	C	490	100.822	74.086	102.233	1.00	20.00	C
ATOM	4131	CD1	PHE	C	490	99.424	73.966	102.398	1.00	20.00	C
ATOM	4132	CD2	PHE	C	490	101.436	73.445	101.135	1.00	20.00	C
ATOM	4133	CE1	PHE	C	490	98.670	73.265	101.467	1.00	20.00	C
ATOM	4134	CE2	PHE	C	490	100.663	72.743	100.219	1.00	20.00	C
ATOM	4135	CZ	PHE	C	490	99.287	72.659	100.381	1.00	20.00	C
ATOM	4136	H	PHE	C	490	102.248	76.806	101.850	1.00	0.00	H
ATOM	4137	HA	PHE	C	490	100.139	76.253	103.707	1.00	0.00	H
ATOM	4138	HB2	PHE	C	490	102.704	74.818	102.941	1.00	0.00	H
ATOM	4139	HB3	PHE	C	490	101.657	74.177	104.174	1.00	0.00	H
ATOM	4140	HD1	PHE	C	490	98.930	74.412	103.249	1.00	0.00	H
ATOM	4141	HD2	PHE	C	490	102.507	73.493	101.003	1.00	0.00	H
ATOM	4142	HE1	PHE	C	490	97.600	73.184	101.593	1.00	0.00	H
ATOM	4143	HE2	PHE	C	490	101.134	72.253	99.381	1.00	0.00	H
ATOM	4144	HZ	PHE	C	490	98.694	72.111	99.664	1.00	0.00	H
ATOM	4145	N	ARG	C	491	102.940	77.311	105.029	1.00	0.00	N
ATOM	4146	CA	ARG	C	491	103.564	77.820	106.252	1.00	0.00	C
ATOM	4147	C	ARG	C	491	102.830	79.046	106.825	1.00	0.00	C
ATOM	4148	O	ARG	C	491	102.699	79.142	108.044	1.00	0.00	O
ATOM	4149	CB	ARG	C	491	105.064	78.082	106.011	1.00	20.00	C
ATOM	4150	CG	ARG	C	491	105.868	78.383	107.289	1.00	20.00	C
ATOM	4151	CD	ARG	C	491	107.365	78.564	107.006	1.00	20.00	C
ATOM	4152	NE	ARG	C	491	108.153	78.552	108.246	1.00	20.00	N
ATOM	4153	CZ	ARG	C	491	109.467	78.811	108.345	1.00	20.00	C
ATOM	4154	NH1	ARG	C	491	110.061	78.738	109.543	1.00	20.00	N
ATOM	4155	NH2	ARG	C	491	110.195	79.138	107.267	1.00	20.00	N1+
ATOM	4156	H	ARG	C	491	103.462	77.444	104.172	1.00	0.00	H
ATOM	4157	HA	ARG	C	491	103.506	77.024	106.992	1.00	0.00	H
ATOM	4158	HB2	ARG	C	491	105.496	77.193	105.551	1.00	0.00	H
ATOM	4159	HB3	ARG	C	491	105.193	78.894	105.297	1.00	0.00	H
ATOM	4160	HG2	ARG	C	491	105.492	79.324	107.693	1.00	0.00	H
ATOM	4161	HG3	ARG	C	491	105.707	77.630	108.061	1.00	0.00	H
ATOM	4162	HD2	ARG	C	491	107.736	77.875	106.249	1.00	0.00	H
ATOM	4163	HD3	ARG	C	491	107.503	79.570	106.607	1.00	0.00	H
ATOM	4164	HE	ARG	C	491	107.647	78.303	109.084	1.00	0.00	H
ATOM	4165	HH11	ARG	C	491	111.048	78.930	109.639	1.00	0.00	H
ATOM	4166	HH12	ARG	C	491	109.525	78.497	110.364	1.00	0.00	H
ATOM	4167	HH21	ARG	C	491	111.180	79.346	107.352	1.00	0.00	H
ATOM	4168	HH22	ARG	C	491	109.744	79.221	106.368	1.00	0.00	H
ATOM	4169	N	GLY	C	492	102.331	79.927	105.939	1.00	0.00	N
ATOM	4170	CA	GLY	C	492	101.521	81.088	106.310	1.00	0.00	C
ATOM	4171	C	GLY	C	492	100.094	80.675	106.708	1.00	0.00	C
ATOM	4172	O	GLY	C	492	99.490	81.338	107.552	1.00	0.00	O
ATOM	4173	H	GLY	C	492	102.494	79.785	104.952	1.00	0.00	H
ATOM	4174	HA2	GLY	C	492	101.996	81.639	107.123	1.00	0.00	H
ATOM	4175	HA3	GLY	C	492	101.468	81.763	105.455	1.00	0.00	H
ATOM	4176	N	ILE	C	493	99.571	79.573	106.137	1.00	30.00	N
ATOM	4177	CA	ILE	C	493	98.273	78.991	106.486	1.00	30.00	C

```
ATOM   4178  C   ILE C 493      98.279  78.306 107.861  1.00 30.00           C
ATOM   4179  O   ILE C 493      97.381  78.590 108.648  1.00 30.00           O
ATOM   4180  CB  ILE C 493      97.742  78.023 105.384  1.00 20.00           C
ATOM   4181  CG1 ILE C 493      97.185  78.852 104.208  1.00 20.00           C
ATOM   4182  CG2 ILE C 493      96.688  76.978 105.828  1.00 20.00           C
ATOM   4183  CD1 ILE C 493      97.047  78.070 102.897  1.00 20.00           C
ATOM   4184  H   ILE C 493     100.101  79.082 105.431  1.00  0.00           H
ATOM   4185  HA  ILE C 493      97.563  79.819 106.559  1.00  0.00           H
ATOM   4186  HB  ILE C 493      98.599  77.462 105.012  1.00  0.00           H
ATOM   4187 HG12 ILE C 493      96.216  79.271 104.482  1.00  0.00           H
ATOM   4188 HG13 ILE C 493      97.830  79.710 104.026  1.00  0.00           H
ATOM   4189 HG21 ILE C 493      96.336  76.386 104.984  1.00  0.00           H
ATOM   4190 HG22 ILE C 493      97.090  76.265 106.548  1.00  0.00           H
ATOM   4191 HG23 ILE C 493      95.818  77.458 106.277  1.00  0.00           H
ATOM   4192 HD11 ILE C 493      97.061  78.757 102.053  1.00  0.00           H
ATOM   4193 HD12 ILE C 493      97.863  77.360 102.757  1.00  0.00           H
ATOM   4194 HD13 ILE C 493      96.107  77.519 102.857  1.00  0.00           H
ATOM   4195  N   GLN C 494      99.278  77.447 108.141  1.00 30.00           N
ATOM   4196  CA  GLN C 494      99.381  76.727 109.417  1.00 30.00           C
ATOM   4197  C   GLN C 494      99.756  77.648 110.597  1.00 30.00           C
ATOM   4198  O   GLN C 494      99.402  77.324 111.729  1.00 30.00           O
ATOM   4199  CB  GLN C 494     100.309  75.500 109.276  1.00 20.00           C
ATOM   4200  CG  GLN C 494     101.821  75.799 109.252  1.00 20.00           C
ATOM   4201  CD  GLN C 494     102.680  74.584 108.883  1.00 20.00           C
ATOM   4202  NE2 GLN C 494     103.974  74.812 108.655  1.00 20.00           N
ATOM   4203  OE1 GLN C 494     102.199  73.454 108.816  1.00 20.00           O
ATOM   4204  H   GLN C 494      99.987  77.246 107.446  1.00  0.00           H
ATOM   4205  HA  GLN C 494      98.388  76.330 109.633  1.00  0.00           H
ATOM   4206  HB2 GLN C 494     100.020  74.953 108.377  1.00  0.00           H
ATOM   4207  HB3 GLN C 494     100.110  74.820 110.107  1.00  0.00           H
ATOM   4208  HG2 GLN C 494     102.011  76.592 108.534  1.00  0.00           H
ATOM   4209  HG3 GLN C 494     102.152  76.170 110.222  1.00  0.00           H
ATOM   4210 HE21 GLN C 494     104.585  74.045 108.415  1.00  0.00           H
ATOM   4211 HE22 GLN C 494     104.351  75.745 108.729  1.00  0.00           H
ATOM   4212  N   TYR C 495     100.400  78.795 110.310  1.00 30.00           N
ATOM   4213  CA  TYR C 495     100.604  79.901 111.248  1.00 30.00           C
ATOM   4214  C   TYR C 495      99.266  80.522 111.699  1.00 30.00           C
ATOM   4215  O   TYR C 495      99.035  80.666 112.900  1.00 30.00           O
ATOM   4216  CB  TYR C 495     101.543  80.948 110.600  1.00 20.00           C
ATOM   4217  CG  TYR C 495     101.792  82.203 111.421  1.00 20.00           C
ATOM   4218  CD1 TYR C 495     102.889  82.252 112.304  1.00 20.00           C
ATOM   4219  CD2 TYR C 495     100.924  83.313 111.323  1.00 20.00           C
ATOM   4220  CE1 TYR C 495     103.098  83.389 113.105  1.00 20.00           C
ATOM   4221  CE2 TYR C 495     101.117  84.439 112.147  1.00 20.00           C
ATOM   4222  CZ  TYR C 495     102.197  84.469 113.050  1.00 20.00           C
ATOM   4223  OH  TYR C 495     102.369  85.538 113.879  1.00 20.00           O
ATOM   4224  H   TYR C 495     100.683  78.972 109.356  1.00  0.00           H
ATOM   4225  HA  TYR C 495     101.102  79.498 112.133  1.00  0.00           H
ATOM   4226  HB2 TYR C 495     102.507  80.486 110.382  1.00  0.00           H
ATOM   4227  HB3 TYR C 495     101.145  81.259 109.635  1.00  0.00           H
ATOM   4228  HD1 TYR C 495     103.572  81.417 112.373  1.00  0.00           H
ATOM   4229  HD2 TYR C 495     100.089  83.291 110.638  1.00  0.00           H
ATOM   4230  HE1 TYR C 495     103.947  83.419 113.771  1.00  0.00           H
ATOM   4231  HE2 TYR C 495     100.431  85.271 112.086  1.00  0.00           H
ATOM   4232  HH  TYR C 495     101.697  86.212 113.761  1.00  0.00           H
ATOM   4233  N   PHE C 496      98.419  80.870 110.714  1.00 30.00           N
ATOM   4234  CA  PHE C 496      97.109  81.490 110.915  1.00 30.00           C
ATOM   4235  C   PHE C 496      96.066  80.535 111.527  1.00 30.00           C
ATOM   4236  O   PHE C 496      95.244  80.981 112.326  1.00 30.00           O
ATOM   4237  CB  PHE C 496      96.640  82.112 109.580  1.00 20.00           C
ATOM   4238  CG  PHE C 496      95.280  82.792 109.614  1.00 20.00           C
ATOM   4239  CD1 PHE C 496      95.166  84.105 110.118  1.00 20.00           C
ATOM   4240  CD2 PHE C 496      94.105  82.068 109.317  1.00 20.00           C
ATOM   4241  CE1 PHE C 496      93.914  84.687 110.269  1.00 20.00           C
ATOM   4242  CE2 PHE C 496      92.862  82.667 109.475  1.00 20.00           C
ATOM   4243  CZ  PHE C 496      92.767  83.971 109.946  1.00 20.00           C
ATOM   4244  H   PHE C 496      98.689  80.713 109.753  1.00  0.00           H
ATOM   4245  HA  PHE C 496      97.243  82.308 111.626  1.00  0.00           H
ATOM   4246  HB2 PHE C 496      97.371  82.857 109.264  1.00  0.00           H
ATOM   4247  HB3 PHE C 496      96.634  81.352 108.796  1.00  0.00           H
ATOM   4248  HD1 PHE C 496      96.053  84.659 110.387  1.00  0.00           H
```

```
ATOM   4249  HD2 PHE C 496      94.168  81.045 108.976  1.00  0.00           H
ATOM   4250  HE1 PHE C 496      93.831  85.696 110.643  1.00  0.00           H
ATOM   4251  HE2 PHE C 496      91.965  82.114 109.241  1.00  0.00           H
ATOM   4252  HZ  PHE C 496      91.797  84.429 110.069  1.00  0.00           H
ATOM   4253  N   LEU C 497      96.125  79.247 111.150  1.00 30.00           N
ATOM   4254  CA  LEU C 497      95.242  78.178 111.621  1.00 30.00           C
ATOM   4255  C   LEU C 497      95.427  77.865 113.119  1.00 30.00           C
ATOM   4256  O   LEU C 497      94.449  77.510 113.776  1.00 30.00           O
ATOM   4257  CB  LEU C 497      95.466  76.937 110.725  1.00 20.00           C
ATOM   4258  CG  LEU C 497      94.495  75.752 110.930  1.00  0.00           C
ATOM   4259  CD1 LEU C 497      93.025  76.147 110.675  1.00  0.00           C
ATOM   4260  CD2 LEU C 497      94.929  74.542 110.074  1.00  0.00           C
ATOM   4261  H   LEU C 497      96.814  78.973 110.460  1.00  0.00           H
ATOM   4262  HA  LEU C 497      94.219  78.532 111.484  1.00  0.00           H
ATOM   4263  HB2 LEU C 497      95.405  77.238 109.679  1.00  0.00           H
ATOM   4264  HB3 LEU C 497      96.487  76.587 110.880  1.00  0.00           H
ATOM   4265  HG  LEU C 497      94.571  75.429 111.969  1.00  0.00           H
ATOM   4266 HD11 LEU C 497      92.477  75.393 110.110  1.00  0.00           H
ATOM   4267 HD12 LEU C 497      92.496  76.278 111.620  1.00  0.00           H
ATOM   4268 HD13 LEU C 497      92.942  77.083 110.123  1.00  0.00           H
ATOM   4269 HD21 LEU C 497      95.034  73.649 110.691  1.00  0.00           H
ATOM   4270 HD22 LEU C 497      94.217  74.307 109.283  1.00  0.00           H
ATOM   4271 HD23 LEU C 497      95.892  74.707 109.588  1.00  0.00           H
ATOM   4272  N   GLN C 498      96.657  78.047 113.631  1.00 30.00           N
ATOM   4273  CA  GLN C 498      96.993  77.971 115.051  1.00 30.00           C
ATOM   4274  C   GLN C 498      96.463  79.177 115.846  1.00 30.00           C
ATOM   4275  O   GLN C 498      95.638  78.997 116.740  1.00 30.00           O
ATOM   4276  CB  GLN C 498      98.523  77.844 115.221  1.00 20.00           C
ATOM   4277  CG  GLN C 498      99.081  76.447 114.896  1.00 20.00           C
ATOM   4278  CD  GLN C 498     100.614  76.408 114.895  1.00 20.00           C
ATOM   4279  NE2 GLN C 498     101.185  75.328 114.358  1.00 20.00           N
ATOM   4280  OE1 GLN C 498     101.280  77.324 115.376  1.00 20.00           O
ATOM   4281  H   GLN C 498      97.408  78.328 113.016  1.00  0.00           H
ATOM   4282  HA  GLN C 498      96.530  77.074 115.467  1.00  0.00           H
ATOM   4283  HB2 GLN C 498      99.016  78.587 114.592  1.00  0.00           H
ATOM   4284  HB3 GLN C 498      98.802  78.090 116.247  1.00  0.00           H
ATOM   4285  HG2 GLN C 498      98.713  75.724 115.625  1.00  0.00           H
ATOM   4286  HG3 GLN C 498      98.720  76.116 113.923  1.00  0.00           H
ATOM   4287 HE21 GLN C 498     102.191  75.248 114.338  1.00  0.00           H
ATOM   4288 HE22 GLN C 498     100.618  74.588 113.970  1.00  0.00           H
ATOM   4289  N   ARG C 499      96.998  80.369 115.531  1.00 30.00           N
ATOM   4290  CA  ARG C 499      96.881  81.568 116.363  1.00 30.00           C
ATOM   4291  C   ARG C 499      95.579  82.348 116.127  1.00 30.00           C
ATOM   4292  O   ARG C 499      94.940  82.742 117.101  1.00 30.00           O
ATOM   4293  CB  ARG C 499      98.112  82.476 116.139  1.00 20.00           C
ATOM   4294  CG  ARG C 499      99.455  81.748 116.324  1.00 20.00           C
ATOM   4295  CD  ARG C 499     100.673  82.671 116.195  1.00 20.00           C
ATOM   4296  NE  ARG C 499     101.904  81.880 116.080  1.00 20.00           N
ATOM   4297  CZ  ARG C 499     103.163  82.341 116.142  1.00 20.00           C
ATOM   4298  NH1 ARG C 499     103.432  83.636 116.363  1.00 20.00           N
ATOM   4299  NH2 ARG C 499     104.166  81.473 115.970  1.00 20.00           N1+
ATOM   4300  H   ARG C 499      97.649  80.427 114.760  1.00  0.00           H
ATOM   4301  HA  ARG C 499      96.890  81.260 117.411  1.00  0.00           H
ATOM   4302  HB2 ARG C 499      98.065  83.327 116.819  1.00  0.00           H
ATOM   4303  HB3 ARG C 499      98.081  82.890 115.129  1.00  0.00           H
ATOM   4304  HG2 ARG C 499      99.447  81.356 117.343  1.00  0.00           H
ATOM   4305  HG3 ARG C 499      99.564  80.883 115.669  1.00  0.00           H
ATOM   4306  HD2 ARG C 499     100.560  83.425 115.416  1.00  0.00           H
ATOM   4307  HD3 ARG C 499     100.785  83.203 117.140  1.00  0.00           H
ATOM   4308  HE  ARG C 499     101.770  80.898 115.887  1.00  0.00           H
ATOM   4309 HH11 ARG C 499     104.388  83.959 116.398  1.00  0.00           H
ATOM   4310 HH12 ARG C 499     102.678  84.300 116.462  1.00  0.00           H
ATOM   4311 HH21 ARG C 499     105.137  81.763 116.023  1.00  0.00           H
ATOM   4312 HH22 ARG C 499     103.973  80.494 115.818  1.00  0.00           H
ATOM   4313  N   ARG C 500      95.234  82.560 114.845  1.00 30.00           N
ATOM   4314  CA  ARG C 500      94.108  83.355 114.343  1.00 30.00           C
ATOM   4315  C   ARG C 500      94.070  84.792 114.927  1.00 30.00           C
ATOM   4316  O   ARG C 500      93.256  85.049 115.815  1.00 30.00           O
ATOM   4317  CB  ARG C 500      92.782  82.574 114.527  1.00 20.00           C
ATOM   4318  CG  ARG C 500      91.565  83.220 113.830  1.00 20.00           C
ATOM   4319  CD  ARG C 500      90.264  82.421 114.000  1.00 20.00           C
```

ATOM	4320	NE	ARG	C	500	90.277	81.166	113.233	1.00	20.00	N
ATOM	4321	CZ	ARG	C	500	89.345	80.200	113.300	1.00	20.00	C
ATOM	4322	NH1	ARG	C	500	89.486	79.101	112.549	1.00	20.00	N
ATOM	4323	NH2	ARG	C	500	88.276	80.315	114.101	1.00	20.00	N1+
ATOM	4324	H	ARG	C	500	95.805	82.141	114.124	1.00	0.00	H
ATOM	4325	HA	ARG	C	500	94.261	83.450	113.267	1.00	0.00	H
ATOM	4326	HB2	ARG	C	500	92.913	81.563	114.140	1.00	0.00	H
ATOM	4327	HB3	ARG	C	500	92.570	82.460	115.592	1.00	0.00	H
ATOM	4328	HG2	ARG	C	500	91.395	84.255	114.128	1.00	0.00	H
ATOM	4329	HG3	ARG	C	500	91.805	83.255	112.767	1.00	0.00	H
ATOM	4330	HD2	ARG	C	500	90.206	82.100	115.041	1.00	0.00	H
ATOM	4331	HD3	ARG	C	500	89.379	83.030	113.816	1.00	0.00	H
ATOM	4332	HE	ARG	C	500	91.059	81.042	112.605	1.00	0.00	H
ATOM	4333	HH11	ARG	C	500	88.798	78.363	112.585	1.00	0.00	H
ATOM	4334	HH12	ARG	C	500	90.289	78.994	111.945	1.00	0.00	H
ATOM	4335	HH21	ARG	C	500	87.582	79.583	114.146	1.00	0.00	H
ATOM	4336	HH22	ARG	C	500	88.162	81.140	114.672	1.00	0.00	H
ATOM	4337	N	PRO	C	501	94.946	85.708	114.440	1.00	30.00	N
ATOM	4338	CA	PRO	C	501	94.978	87.121	114.888	1.00	30.00	C
ATOM	4339	C	PRO	C	501	93.712	87.977	114.634	1.00	30.00	C
ATOM	4340	O	PRO	C	501	93.685	89.129	115.065	1.00	30.00	O
ATOM	4341	CB	PRO	C	501	96.212	87.706	114.178	1.00	20.00	C
ATOM	4342	CG	PRO	C	501	97.078	86.506	113.839	1.00	0.00	C
ATOM	4343	CD	PRO	C	501	96.041	85.445	113.505	1.00	0.00	C
ATOM	4344	HA	PRO	C	501	95.168	87.109	115.963	1.00	0.00	H
ATOM	4345	HB2	PRO	C	501	95.915	88.200	113.253	1.00	0.00	H
ATOM	4346	HB3	PRO	C	501	96.745	88.436	114.788	1.00	0.00	H
ATOM	4347	HG2	PRO	C	501	97.790	86.692	113.034	1.00	0.00	H
ATOM	4348	HG3	PRO	C	501	97.642	86.200	114.721	1.00	0.00	H
ATOM	4349	HD2	PRO	C	501	95.668	85.587	112.492	1.00	0.00	H
ATOM	4350	HD3	PRO	C	501	96.480	84.451	113.574	1.00	0.00	H
ATOM	4351	N	SER	C	502	92.689	87.409	113.968	1.00	30.00	N
ATOM	4352	CA	SER	C	502	91.336	87.953	113.798	1.00	30.00	C
ATOM	4353	C	SER	C	502	91.286	89.276	112.998	1.00	30.00	C
ATOM	4354	O	SER	C	502	90.419	90.114	113.247	1.00	30.00	O
ATOM	4355	CB	SER	C	502	90.620	88.005	115.172	1.00	20.00	C
ATOM	4356	OG	SER	C	502	89.230	88.231	115.041	1.00	0.00	O
ATOM	4357	H	SER	C	502	92.804	86.459	113.646	1.00	0.00	H
ATOM	4358	HA	SER	C	502	90.811	87.219	113.185	1.00	0.00	H
ATOM	4359	HB2	SER	C	502	90.752	87.060	115.700	1.00	0.00	H
ATOM	4360	HB3	SER	C	502	91.044	88.784	115.807	1.00	0.00	H
ATOM	4361	HG	SER	C	502	89.103	89.106	114.663	1.00	0.00	H
ATOM	4362	N	LEU	C	503	92.223	89.424	112.044	1.00	30.00	N
ATOM	4363	CA	LEU	C	503	92.372	90.513	111.069	1.00	30.00	C
ATOM	4364	C	LEU	C	503	92.836	91.870	111.641	1.00	30.00	C
ATOM	4365	O	LEU	C	503	93.423	92.641	110.882	1.00	30.00	O
ATOM	4366	CB	LEU	C	503	91.104	90.677	110.188	1.00	20.00	C
ATOM	4367	CG	LEU	C	503	90.661	89.399	109.434	1.00	0.00	C
ATOM	4368	CD1	LEU	C	503	89.311	89.619	108.718	1.00	0.00	C
ATOM	4369	CD2	LEU	C	503	91.748	88.873	108.473	1.00	0.00	C
ATOM	4370	H	LEU	C	503	92.899	88.681	111.942	1.00	0.00	H
ATOM	4371	HA	LEU	C	503	93.184	90.189	110.420	1.00	0.00	H
ATOM	4372	HB2	LEU	C	503	90.280	91.034	110.806	1.00	0.00	H
ATOM	4373	HB3	LEU	C	503	91.276	91.467	109.455	1.00	0.00	H
ATOM	4374	HG	LEU	C	503	90.481	88.618	110.173	1.00	0.00	H
ATOM	4375	HD11	LEU	C	503	88.587	88.858	109.012	1.00	0.00	H
ATOM	4376	HD12	LEU	C	503	88.872	90.587	108.961	1.00	0.00	H
ATOM	4377	HD13	LEU	C	503	89.399	89.580	107.632	1.00	0.00	H
ATOM	4378	HD21	LEU	C	503	91.336	88.503	107.534	1.00	0.00	H
ATOM	4379	HD22	LEU	C	503	92.473	89.646	108.219	1.00	0.00	H
ATOM	4380	HD23	LEU	C	503	92.296	88.046	108.927	1.00	0.00	H
ATOM	4381	N	LYS	C	504	92.601	92.146	112.938	1.00	30.00	N
ATOM	4382	CA	LYS	C	504	92.995	93.394	113.595	1.00	30.00	C
ATOM	4383	C	LYS	C	504	94.444	93.316	114.107	1.00	30.00	C
ATOM	4384	O	LYS	C	504	95.252	94.166	113.736	1.00	30.00	O
ATOM	4385	CB	LYS	C	504	92.001	93.737	114.726	1.00	20.00	C
ATOM	4386	CG	LYS	C	504	92.148	95.185	115.240	1.00	0.00	C
ATOM	4387	CD	LYS	C	504	91.350	95.486	116.518	1.00	0.00	C
ATOM	4388	CE	LYS	C	504	92.011	94.908	117.779	1.00	0.00	C
ATOM	4389	NZ	LYS	C	504	91.291	95.292	119.004	1.00	0.00	N1+
ATOM	4390	H	LYS	C	504	92.095	91.476	113.500	1.00	0.00	H

```
ATOM   4391  HA  LYS C 504      92.945  94.204 112.864  1.00  0.00           H
ATOM   4392  HB2 LYS C 504      90.981  93.616 114.358  1.00  0.00           H
ATOM   4393  HB3 LYS C 504      92.109  93.019 115.540  1.00  0.00           H
ATOM   4394  HG2 LYS C 504      93.196  95.430 115.415  1.00  0.00           H
ATOM   4395  HG3 LYS C 504      91.818  95.864 114.454  1.00  0.00           H
ATOM   4396  HD2 LYS C 504      91.242  96.567 116.623  1.00  0.00           H
ATOM   4397  HD3 LYS C 504      90.338  95.089 116.420  1.00  0.00           H
ATOM   4398  HE2 LYS C 504      92.062  93.820 117.732  1.00  0.00           H
ATOM   4399  HE3 LYS C 504      93.036  95.271 117.863  1.00  0.00           H
ATOM   4400  HZ1 LYS C 504      91.803  94.918 119.797  1.00  0.00           H
ATOM   4401  HZ2 LYS C 504      91.256  96.298 119.083  1.00  0.00           H
ATOM   4402  HZ3 LYS C 504      90.356  94.913 118.994  1.00  0.00           H
ATOM   4403  N   SER C 505      94.753  92.285 114.917  1.00 30.00           N
ATOM   4404  CA  SER C 505      96.102  92.012 115.432  1.00 30.00           C
ATOM   4405  C   SER C 505      97.062  91.474 114.343  1.00 30.00           C
ATOM   4406  O   SER C 505      98.274  91.474 114.559  1.00 30.00           O
ATOM   4407  CB  SER C 505      95.995  91.071 116.654  1.00 20.00           C
ATOM   4408  OG  SER C 505      97.229  90.900 117.323  1.00  0.00           O
ATOM   4409  H   SER C 505      94.040  91.614 115.167  1.00  0.00           H
ATOM   4410  HA  SER C 505      96.512  92.957 115.789  1.00  0.00           H
ATOM   4411  HB2 SER C 505      95.278  91.468 117.374  1.00  0.00           H
ATOM   4412  HB3 SER C 505      95.637  90.087 116.360  1.00  0.00           H
ATOM   4413  HG  SER C 505      97.408  91.683 117.852  1.00  0.00           H
ATOM   4414  N   LEU C 506      96.508  91.064 113.190  1.00 30.00           N
ATOM   4415  CA  LEU C 506      97.242  90.581 112.026  1.00 30.00           C
ATOM   4416  C   LEU C 506      98.265  91.628 111.554  1.00 30.00           C
ATOM   4417  O   LEU C 506      99.454  91.463 111.801  1.00 30.00           O
ATOM   4418  CB  LEU C 506      96.229  90.152 110.932  1.00 20.00           C
ATOM   4419  CG  LEU C 506      96.835  89.447 109.697  1.00  0.00           C
ATOM   4420  CD1 LEU C 506      97.607  88.161 110.056  1.00  0.00           C
ATOM   4421  CD2 LEU C 506      95.758  89.176 108.630  1.00  0.00           C
ATOM   4422  H   LEU C 506      95.510  91.163 113.097  1.00  0.00           H
ATOM   4423  HA  LEU C 506      97.805  89.705 112.351  1.00  0.00           H
ATOM   4424  HB2 LEU C 506      95.486  89.487 111.370  1.00  0.00           H
ATOM   4425  HB3 LEU C 506      95.675  91.031 110.597  1.00  0.00           H
ATOM   4426  HG  LEU C 506      97.541  90.139 109.239  1.00  0.00           H
ATOM   4427 HD11 LEU C 506      97.406  87.345 109.361  1.00  0.00           H
ATOM   4428 HD12 LEU C 506      98.683  88.335 110.029  1.00  0.00           H
ATOM   4429 HD13 LEU C 506      97.358  87.797 111.051  1.00  0.00           H
ATOM   4430 HD21 LEU C 506      96.143  89.394 107.633  1.00  0.00           H
ATOM   4431 HD22 LEU C 506      95.419  88.140 108.630  1.00  0.00           H
ATOM   4432 HD23 LEU C 506      94.876  89.801 108.769  1.00  0.00           H
ATOM   4433  N   PHE C 507      97.787  92.722 110.959  1.00 30.00           N
ATOM   4434  CA  PHE C 507      98.646  93.761 110.385  1.00 30.00           C
ATOM   4435  C   PHE C 507      99.388  94.606 111.440  1.00 30.00           C
ATOM   4436  O   PHE C 507     100.368  95.262 111.092  1.00 30.00           O
ATOM   4437  CB  PHE C 507      97.802  94.650 109.451  1.00 20.00           C
ATOM   4438  CG  PHE C 507      97.017  93.866 108.411  1.00  0.00           C
ATOM   4439  CD1 PHE C 507      95.674  93.510 108.659  1.00  0.00           C
ATOM   4440  CD2 PHE C 507      97.671  93.323 107.285  1.00  0.00           C
ATOM   4441  CE1 PHE C 507      95.002  92.670 107.783  1.00  0.00           C
ATOM   4442  CE2 PHE C 507      96.981  92.484 106.420  1.00  0.00           C
ATOM   4443  CZ  PHE C 507      95.652  92.161 106.666  1.00  0.00           C
ATOM   4444  H   PHE C 507      96.798  92.808 110.776  1.00  0.00           H
ATOM   4445  HA  PHE C 507      99.412  93.270 109.782  1.00  0.00           H
ATOM   4446  HB2 PHE C 507      97.109  95.260 110.033  1.00  0.00           H
ATOM   4447  HB3 PHE C 507      98.459  95.353 108.935  1.00  0.00           H
ATOM   4448  HD1 PHE C 507      95.161  93.888 109.532  1.00  0.00           H
ATOM   4449  HD2 PHE C 507      98.712  93.544 107.098  1.00  0.00           H
ATOM   4450  HE1 PHE C 507      93.972  92.407 107.979  1.00  0.00           H
ATOM   4451  HE2 PHE C 507      97.482  92.071 105.556  1.00  0.00           H
ATOM   4452  HZ  PHE C 507      95.124  91.503 105.991  1.00  0.00           H
ATOM   4453  N   VAL C 508      98.917  94.585 112.695  1.00 30.00           N
ATOM   4454  CA  VAL C 508      99.532  95.303 113.814  1.00 30.00           C
ATOM   4455  C   VAL C 508     100.836  94.640 114.303  1.00 30.00           C
ATOM   4456  O   VAL C 508     101.824  95.354 114.487  1.00 30.00           O
ATOM   4457  CB  VAL C 508      98.526  95.447 114.998  1.00 20.00           C
ATOM   4458  CG1 VAL C 508      99.130  95.820 116.371  1.00  0.00           C
ATOM   4459  CG2 VAL C 508      97.419  96.454 114.642  1.00  0.00           C
ATOM   4460  H   VAL C 508      98.103  94.030 112.917  1.00  0.00           H
ATOM   4461  HA  VAL C 508      99.788  96.306 113.467  1.00  0.00           H
```

ATOM	4462	HB	VAL	C	508	98.040	94.481	115.128	1.00	0.00	H
ATOM	4463	HG11	VAL	C	508	98.346	95.997	117.108	1.00	0.00	H
ATOM	4464	HG12	VAL	C	508	99.759	95.026	116.777	1.00	0.00	H
ATOM	4465	HG13	VAL	C	508	99.731	96.727	116.306	1.00	0.00	H
ATOM	4466	HG21	VAL	C	508	96.642	96.475	115.408	1.00	0.00	H
ATOM	4467	HG22	VAL	C	508	97.820	97.464	114.548	1.00	0.00	H
ATOM	4468	HG23	VAL	C	508	96.936	96.207	113.697	1.00	0.00	H
ATOM	4469	N	ASP	C	509	100.805	93.312	114.515	1.00	30.00	N
ATOM	4470	CA	ASP	C	509	101.885	92.559	115.157	1.00	30.00	C
ATOM	4471	C	ASP	C	509	102.561	91.603	114.158	1.00	30.00	C
ATOM	4472	O	ASP	C	509	103.782	91.663	114.015	1.00	30.00	O
ATOM	4473	CB	ASP	C	509	101.363	91.803	116.405	1.00	20.00	C
ATOM	4474	CG	ASP	C	509	102.450	91.183	117.294	1.00	0.00	C
ATOM	4475	OD1	ASP	C	509	103.585	91.708	117.305	1.00	0.00	O
ATOM	4476	OD2	ASP	C	509	102.095	90.247	118.043	1.00	0.00	O1-
ATOM	4477	H	ASP	C	509	99.955	92.796	114.326	1.00	0.00	H
ATOM	4478	HA	ASP	C	509	102.655	93.256	115.493	1.00	0.00	H
ATOM	4479	HB2	ASP	C	509	100.775	92.483	117.022	1.00	0.00	H
ATOM	4480	HB3	ASP	C	509	100.691	91.006	116.084	1.00	0.00	H
ATOM	4481	N	SER	C	510	101.779	90.831	113.396	1.00	30.00	N
ATOM	4482	CA	SER	C	510	102.232	89.737	112.531	1.00	30.00	C
ATOM	4483	C	SER	C	510	102.928	90.179	111.211	1.00	30.00	C
ATOM	4484	O	SER	C	510	102.705	89.564	110.163	1.00	30.00	O
ATOM	4485	CB	SER	C	510	101.023	88.799	112.308	1.00	20.00	C
ATOM	4486	OG	SER	C	510	101.410	87.524	111.856	1.00	20.00	O
ATOM	4487	H	SER	C	510	100.779	90.993	113.398	1.00	0.00	H
ATOM	4488	HA	SER	C	510	102.981	89.179	113.097	1.00	0.00	H
ATOM	4489	HB2	SER	C	510	100.339	89.209	111.569	1.00	0.00	H
ATOM	4490	HB3	SER	C	510	100.456	88.669	113.231	1.00	0.00	H
ATOM	4491	HG	SER	C	510	101.857	87.651	111.008	1.00	0.00	H
ATOM	4492	N	TYR	C	511	103.777	91.214	111.278	1.00	30.00	N
ATOM	4493	CA	TYR	C	511	104.563	91.760	110.167	1.00	30.00	C
ATOM	4494	C	TYR	C	511	105.570	90.724	109.617	1.00	30.00	C
ATOM	4495	O	TYR	C	511	105.908	89.755	110.301	1.00	30.00	O
ATOM	4496	CB	TYR	C	511	105.276	93.054	110.627	1.00	20.00	C
ATOM	4497	CG	TYR	C	511	105.449	94.113	109.545	1.00	0.00	C
ATOM	4498	CD1	TYR	C	511	106.556	94.088	108.668	1.00	0.00	C
ATOM	4499	CD2	TYR	C	511	104.503	95.155	109.433	1.00	0.00	C
ATOM	4500	CE1	TYR	C	511	106.705	95.081	107.679	1.00	0.00	C
ATOM	4501	CE2	TYR	C	511	104.668	96.163	108.465	1.00	0.00	C
ATOM	4502	CZ	TYR	C	511	105.763	96.124	107.583	1.00	0.00	C
ATOM	4503	OH	TYR	C	511	105.892	97.093	106.635	1.00	0.00	O
ATOM	4504	H	TYR	C	511	103.958	91.591	112.206	1.00	0.00	H
ATOM	4505	HA	TYR	C	511	103.860	92.005	109.368	1.00	0.00	H
ATOM	4506	HB2	TYR	C	511	104.692	93.527	111.420	1.00	0.00	H
ATOM	4507	HB3	TYR	C	511	106.239	92.832	111.090	1.00	0.00	H
ATOM	4508	HD1	TYR	C	511	107.306	93.318	108.752	1.00	0.00	H
ATOM	4509	HD2	TYR	C	511	103.654	95.202	110.102	1.00	0.00	H
ATOM	4510	HE1	TYR	C	511	107.556	95.047	107.013	1.00	0.00	H
ATOM	4511	HE2	TYR	C	511	103.949	96.965	108.393	1.00	0.00	H
ATOM	4512	HH	TYR	C	511	106.243	96.748	105.803	1.00	0.00	H
ATOM	4513	N	SER	C	512	106.015	90.922	108.370	1.00	30.00	N
ATOM	4514	CA	SER	C	512	106.838	90.010	107.565	1.00	30.00	C
ATOM	4515	C	SER	C	512	106.099	88.717	107.151	1.00	30.00	C
ATOM	4516	O	SER	C	512	106.029	88.435	105.958	1.00	30.00	O
ATOM	4517	CB	SER	C	512	108.226	89.724	108.198	1.00	20.00	C
ATOM	4518	OG	SER	C	512	109.231	90.563	107.653	1.00	0.00	O
ATOM	4519	H	SER	C	512	105.729	91.765	107.894	1.00	0.00	H
ATOM	4520	HA	SER	C	512	107.015	90.537	106.625	1.00	0.00	H
ATOM	4521	HB2	SER	C	512	108.230	89.831	109.284	1.00	0.00	H
ATOM	4522	HB3	SER	C	512	108.527	88.692	107.997	1.00	0.00	H
ATOM	4523	HG	SER	C	512	109.251	91.398	108.143	1.00	0.00	H
ATOM	4524	N	GLU	C	513	105.505	87.979	108.101	1.00	30.00	N
ATOM	4525	CA	GLU	C	513	104.836	86.691	107.868	1.00	30.00	C
ATOM	4526	C	GLU	C	513	103.684	86.786	106.844	1.00	30.00	C
ATOM	4527	O	GLU	C	513	103.588	85.957	105.937	1.00	30.00	O
ATOM	4528	CB	GLU	C	513	104.387	86.103	109.228	1.00	20.00	C
ATOM	4529	CG	GLU	C	513	103.741	84.697	109.187	1.00	20.00	C
ATOM	4530	CD	GLU	C	513	104.649	83.587	108.648	1.00	20.00	C
ATOM	4531	OE1	GLU	C	513	105.886	83.763	108.686	1.00	20.00	O
ATOM	4532	OE2	GLU	C	513	104.088	82.563	108.206	1.00	20.00	O1-

```
ATOM   4533  H   GLU C 513     105.556  88.310 109.062  1.00  0.00           H
ATOM   4534  HA  GLU C 513     105.589  86.032 107.433  1.00  0.00           H
ATOM   4535  HB2 GLU C 513     105.244  86.070 109.903  1.00  0.00           H
ATOM   4536  HB3 GLU C 513     103.681  86.789 109.698  1.00  0.00           H
ATOM   4537  HG2 GLU C 513     102.818  84.713 108.606  1.00  0.00           H
ATOM   4538  HG3 GLU C 513     103.453  84.414 110.199  1.00  0.00           H
ATOM   4539  N   ILE C 514     102.894  87.864 106.949  1.00  0.00           N
ATOM   4540  CA  ILE C 514     101.875  88.251 105.969  1.00  0.00           C
ATOM   4541  C   ILE C 514     102.467  88.414 104.552  1.00  0.00           C
ATOM   4542  O   ILE C 514     101.903  87.901 103.589  1.00  0.00           O
ATOM   4543  CB  ILE C 514     101.195  89.594 106.383  1.00 20.00           C
ATOM   4544  CG1 ILE C 514     100.423  89.448 107.712  1.00 20.00           C
ATOM   4545  CG2 ILE C 514     100.268  90.235 105.322  1.00 20.00           C
ATOM   4546  CD1 ILE C 514     100.211  90.785 108.433  1.00 20.00           C
ATOM   4547  H   ILE C 514     103.022  88.480 107.741  1.00  0.00           H
ATOM   4548  HA  ILE C 514     101.122  87.461 105.933  1.00  0.00           H
ATOM   4549  HB  ILE C 514     102.003  90.307 106.556  1.00  0.00           H
ATOM   4550 HG12 ILE C 514      99.457  88.975 107.526  1.00  0.00           H
ATOM   4551 HG13 ILE C 514     100.940  88.775 108.395  1.00  0.00           H
ATOM   4552 HG21 ILE C 514      99.803  91.144 105.702  1.00  0.00           H
ATOM   4553 HG22 ILE C 514     100.802  90.525 104.416  1.00  0.00           H
ATOM   4554 HG23 ILE C 514      99.468  89.553 105.033  1.00  0.00           H
ATOM   4555 HD11 ILE C 514     100.188  90.630 109.509  1.00  0.00           H
ATOM   4556 HD12 ILE C 514     101.009  91.499 108.233  1.00  0.00           H
ATOM   4557 HD13 ILE C 514      99.268  91.244 108.143  1.00  0.00           H
ATOM   4558  N   LEU C 515     103.618  89.085 104.460  1.00  0.00           N
ATOM   4559  CA  LEU C 515     104.297  89.406 103.212  1.00  0.00           C
ATOM   4560  C   LEU C 515     104.910  88.158 102.548  1.00  0.00           C
ATOM   4561  O   LEU C 515     104.891  88.067 101.320  1.00  0.00           O
ATOM   4562  CB  LEU C 515     105.337  90.527 103.438  1.00 20.00           C
ATOM   4563  CG  LEU C 515     104.799  91.899 103.921  1.00 20.00           C
ATOM   4564  CD1 LEU C 515     103.676  92.462 103.027  1.00 20.00           C
ATOM   4565  CD2 LEU C 515     104.444  91.960 105.422  1.00 20.00           C
ATOM   4566  H   LEU C 515     104.116  89.336 105.301  1.00  0.00           H
ATOM   4567  HA  LEU C 515     103.544  89.781 102.522  1.00  0.00           H
ATOM   4568  HB2 LEU C 515     106.138  90.189 104.095  1.00  0.00           H
ATOM   4569  HB3 LEU C 515     105.829  90.700 102.482  1.00  0.00           H
ATOM   4570  HG  LEU C 515     105.646  92.577 103.793  1.00  0.00           H
ATOM   4571 HD11 LEU C 515     103.819  93.530 102.859  1.00  0.00           H
ATOM   4572 HD12 LEU C 515     103.663  91.993 102.044  1.00  0.00           H
ATOM   4573 HD13 LEU C 515     102.686  92.327 103.463  1.00  0.00           H
ATOM   4574 HD21 LEU C 515     103.389  92.174 105.594  1.00  0.00           H
ATOM   4575 HD22 LEU C 515     104.673  91.031 105.935  1.00  0.00           H
ATOM   4576 HD23 LEU C 515     105.012  92.751 105.915  1.00  0.00           H
ATOM   4577  N   PHE C 516     105.376  87.185 103.347  1.00  0.00           N
ATOM   4578  CA  PHE C 516     105.745  85.837 102.884  1.00  0.00           C
ATOM   4579  C   PHE C 516     104.552  85.074 102.274  1.00  0.00           C
ATOM   4580  O   PHE C 516     104.690  84.444 101.222  1.00  0.00           O
ATOM   4581  CB  PHE C 516     106.423  85.018 104.008  1.00 20.00           C
ATOM   4582  CG  PHE C 516     107.930  85.190 104.141  1.00 20.00           C
ATOM   4583  CD1 PHE C 516     108.804  84.335 103.436  1.00 20.00           C
ATOM   4584  CD2 PHE C 516     108.480  86.269 104.865  1.00 20.00           C
ATOM   4585  CE1 PHE C 516     110.178  84.506 103.538  1.00 20.00           C
ATOM   4586  CE2 PHE C 516     109.856  86.432 104.941  1.00 20.00           C
ATOM   4587  CZ  PHE C 516     110.704  85.548 104.289  1.00 20.00           C
ATOM   4588  H   PHE C 516     105.438  87.367 104.344  1.00  0.00           H
ATOM   4589  HA  PHE C 516     106.471  85.942 102.078  1.00  0.00           H
ATOM   4590  HB2 PHE C 516     105.963  85.247 104.969  1.00  0.00           H
ATOM   4591  HB3 PHE C 516     106.240  83.953 103.853  1.00  0.00           H
ATOM   4592  HD1 PHE C 516     108.415  83.535 102.824  1.00  0.00           H
ATOM   4593  HD2 PHE C 516     107.840  86.969 105.372  1.00  0.00           H
ATOM   4594  HE1 PHE C 516     110.834  83.827 103.017  1.00  0.00           H
ATOM   4595  HE2 PHE C 516     110.267  87.259 105.498  1.00  0.00           H
ATOM   4596  HZ  PHE C 516     111.773  85.690 104.347  1.00  0.00           H
ATOM   4597  N   PHE C 517     103.373  85.189 102.900  1.00  0.00           N
ATOM   4598  CA  PHE C 517     102.138  84.608 102.382  1.00  0.00           C
ATOM   4599  C   PHE C 517     101.624  85.332 101.121  1.00  0.00           C
ATOM   4600  O   PHE C 517     101.142  84.661 100.212  1.00  0.00           O
ATOM   4601  CB  PHE C 517     101.085  84.502 103.505  1.00 20.00           C
ATOM   4602  CG  PHE C 517      99.774  83.868 103.074  1.00 20.00           C
ATOM   4603  CD1 PHE C 517      99.709  82.475 102.861  1.00 20.00           C
```

```
ATOM   4604  CD2 PHE C 517      98.677  84.672 102.695  1.00 20.00           C
ATOM   4605  CE1 PHE C 517      98.563  81.908 102.323  1.00 20.00           C
ATOM   4606  CE2 PHE C 517      97.535  84.082 102.168  1.00 20.00           C
ATOM   4607  CZ  PHE C 517      97.478  82.706 101.984  1.00 20.00           C
ATOM   4608  H   PHE C 517     103.289  85.769 103.724  1.00  0.00           H
ATOM   4609  HA  PHE C 517     102.368  83.584 102.086  1.00  0.00           H
ATOM   4610  HB2 PHE C 517     101.495  83.888 104.309  1.00  0.00           H
ATOM   4611  HB3 PHE C 517     100.888  85.479 103.948  1.00  0.00           H
ATOM   4612  HD1 PHE C 517     100.557  81.848 103.097  1.00  0.00           H
ATOM   4613  HD2 PHE C 517      98.726  85.746 102.802  1.00  0.00           H
ATOM   4614  HE1 PHE C 517      98.521  80.843 102.153  1.00  0.00           H
ATOM   4615  HE2 PHE C 517      96.694  84.698 101.885  1.00  0.00           H
ATOM   4616  HZ  PHE C 517      96.592  82.256 101.562  1.00  0.00           H
ATOM   4617  N   VAL C 518     101.791  86.660 101.034  1.00  0.00           N
ATOM   4618  CA  VAL C 518     101.478  87.452  99.839  1.00  0.00           C
ATOM   4619  C   VAL C 518     102.420  87.127  98.656  1.00  0.00           C
ATOM   4620  O   VAL C 518     101.933  86.889  97.550  1.00  0.00           O
ATOM   4621  CB  VAL C 518     101.481  88.980 100.140  1.00 20.00           C
ATOM   4622  CG1 VAL C 518     101.491  89.902  98.904  1.00 20.00           C
ATOM   4623  CG2 VAL C 518     100.286  89.361 101.035  1.00 20.00           C
ATOM   4624  H   VAL C 518     102.101  87.168 101.857  1.00  0.00           H
ATOM   4625  HA  VAL C 518     100.468  87.180  99.526  1.00  0.00           H
ATOM   4626  HB  VAL C 518     102.387  89.204 100.700  1.00  0.00           H
ATOM   4627 HG11 VAL C 518     101.333  90.944  99.182  1.00  0.00           H
ATOM   4628 HG12 VAL C 518     102.445  89.859  98.378  1.00  0.00           H
ATOM   4629 HG13 VAL C 518     100.705  89.631  98.196  1.00  0.00           H
ATOM   4630 HG21 VAL C 518     100.353  90.399 101.363  1.00  0.00           H
ATOM   4631 HG22 VAL C 518      99.342  89.242 100.501  1.00  0.00           H
ATOM   4632 HG23 VAL C 518     100.226  88.744 101.929  1.00  0.00           H
ATOM   4633  N   GLN C 519     103.736  87.040  98.919  1.00  0.00           N
ATOM   4634  CA  GLN C 519     104.764  86.559  97.985  1.00  0.00           C
ATOM   4635  C   GLN C 519     104.384  85.218  97.340  1.00  0.00           C
ATOM   4636  O   GLN C 519     104.470  85.068  96.120  1.00  0.00           O
ATOM   4637  CB  GLN C 519     106.122  86.473  98.725  1.00 20.00           C
ATOM   4638  CG  GLN C 519     107.259  85.651  98.060  1.00 20.00           C
ATOM   4639  CD  GLN C 519     108.459  85.463  98.988  1.00 20.00           C
ATOM   4640  NE2 GLN C 519     109.619  85.127  98.447  1.00 20.00           N
ATOM   4641  OE1 GLN C 519     108.359  85.636 100.198  1.00 20.00           O
ATOM   4642  H   GLN C 519     104.061  87.284  99.852  1.00  0.00           H
ATOM   4643  HA  GLN C 519     104.853  87.294  97.183  1.00  0.00           H
ATOM   4644  HB2 GLN C 519     106.489  87.482  98.909  1.00  0.00           H
ATOM   4645  HB3 GLN C 519     105.935  86.049  99.709  1.00  0.00           H
ATOM   4646  HG2 GLN C 519     106.933  84.645  97.796  1.00  0.00           H
ATOM   4647  HG3 GLN C 519     107.578  86.136  97.135  1.00  0.00           H
ATOM   4648 HE21 GLN C 519     110.436  85.138  99.057  1.00  0.00           H
ATOM   4649 HE22 GLN C 519     109.730  84.960  97.458  1.00  0.00           H
ATOM   4650  N   SER C 520     103.963  84.281  98.195  1.00  0.00           N
ATOM   4651  CA  SER C 520     103.580  82.927  97.802  1.00  0.00           C
ATOM   4652  C   SER C 520     102.244  82.873  97.037  1.00  0.00           C
ATOM   4653  O   SER C 520     102.111  82.056  96.129  1.00  0.00           O
ATOM   4654  CB  SER C 520     103.600  82.038  99.063  1.00 20.00           C
ATOM   4655  OG  SER C 520     102.409  82.101  99.820  1.00 20.00           O
ATOM   4656  H   SER C 520     103.969  84.464  99.189  1.00  0.00           H
ATOM   4657  HA  SER C 520     104.360  82.564  97.132  1.00  0.00           H
ATOM   4658  HB2 SER C 520     104.437  82.307  99.702  1.00  0.00           H
ATOM   4659  HB3 SER C 520     103.756  80.999  98.788  1.00  0.00           H
ATOM   4660  HG  SER C 520     102.257  83.009 100.097  1.00  0.00           H
ATOM   4661  N   LEU C 521     101.304  83.767  97.391  1.00  0.00           N
ATOM   4662  CA  LEU C 521     100.012  83.943  96.733  1.00  0.00           C
ATOM   4663  C   LEU C 521     100.180  84.494  95.308  1.00  0.00           C
ATOM   4664  O   LEU C 521      99.581  83.939  94.389  1.00  0.00           O
ATOM   4665  CB  LEU C 521      99.102  84.798  97.647  1.00 20.00           C
ATOM   4666  CG  LEU C 521      97.660  85.050  97.144  1.00 20.00           C
ATOM   4667  CD1 LEU C 521      96.695  85.279  98.323  1.00 20.00           C
ATOM   4668  CD2 LEU C 521      97.560  86.204  96.123  1.00 20.00           C
ATOM   4669  H   LEU C 521     101.486  84.388  98.168  1.00  0.00           H
ATOM   4670  HA  LEU C 521      99.556  82.954  96.648  1.00  0.00           H
ATOM   4671  HB2 LEU C 521      99.046  84.264  98.596  1.00  0.00           H
ATOM   4672  HB3 LEU C 521      99.579  85.749  97.882  1.00  0.00           H
ATOM   4673  HG  LEU C 521      97.326  84.135  96.652  1.00  0.00           H
ATOM   4674 HD11 LEU C 521      95.672  85.036  98.036  1.00  0.00           H
```

```
ATOM   4675 HD12 LEU C 521      96.940  84.656  99.184  1.00  0.00           H
ATOM   4676 HD13 LEU C 521      96.708  86.317  98.658  1.00  0.00           H
ATOM   4677 HD21 LEU C 521      96.774  86.915  96.380  1.00  0.00           H
ATOM   4678 HD22 LEU C 521      98.488  86.771  96.048  1.00  0.00           H
ATOM   4679 HD23 LEU C 521      97.331  85.821  95.129  1.00  0.00           H
ATOM   4680  N   PHE C 522     101.049  85.502  95.147  1.00  0.00           N
ATOM   4681  CA  PHE C 522     101.486  86.008  93.847  1.00  0.00           C
ATOM   4682  C   PHE C 522     102.159  84.919  92.984  1.00  0.00           C
ATOM   4683  O   PHE C 522     101.876  84.834  91.787  1.00  0.00           O
ATOM   4684  CB  PHE C 522     102.451  87.200  94.029  1.00 20.00           C
ATOM   4685  CG  PHE C 522     101.914  88.536  94.513  1.00 20.00           C
ATOM   4686  CD1 PHE C 522     100.740  89.095  93.964  1.00 20.00           C
ATOM   4687  CD2 PHE C 522     102.724  89.341  95.345  1.00 20.00           C
ATOM   4688  CE1 PHE C 522     100.373  90.395  94.288  1.00 20.00           C
ATOM   4689  CE2 PHE C 522     102.342  90.638  95.653  1.00 20.00           C
ATOM   4690  CZ  PHE C 522     101.168  91.161  95.131  1.00 20.00           C
ATOM   4691  H   PHE C 522     101.455  85.940  95.972  1.00  0.00           H
ATOM   4692  HA  PHE C 522     100.602  86.335  93.299  1.00  0.00           H
ATOM   4693  HB2 PHE C 522     103.244  86.896  94.712  1.00  0.00           H
ATOM   4694  HB3 PHE C 522     102.931  87.422  93.079  1.00  0.00           H
ATOM   4695  HD1 PHE C 522     100.126  88.534  93.276  1.00  0.00           H
ATOM   4696  HD2 PHE C 522     103.657  88.962  95.737  1.00  0.00           H
ATOM   4697  HE1 PHE C 522      99.472  90.820  93.869  1.00  0.00           H
ATOM   4698  HE2 PHE C 522     102.968  91.246  96.290  1.00  0.00           H
ATOM   4699  HZ  PHE C 522     100.878  92.175  95.368  1.00  0.00           H
ATOM   4700  N   MET C 523     102.997  84.074  93.607  1.00  0.00           N
ATOM   4701  CA  MET C 523     103.709  82.990  92.924  1.00  0.00           C
ATOM   4702  C   MET C 523     102.763  81.911  92.365  1.00  0.00           C
ATOM   4703  O   MET C 523     103.004  81.419  91.265  1.00  0.00           O
ATOM   4704  CB  MET C 523     104.777  82.377  93.860  1.00 20.00           C
ATOM   4705  CG  MET C 523     105.801  81.481  93.140  1.00 20.00           C
ATOM   4706  SD  MET C 523     106.822  82.363  91.926  1.00 20.00           S
ATOM   4707  CE  MET C 523     106.989  81.095  90.647  1.00 20.00           C
ATOM   4708  H   MET C 523     103.106  84.124  94.611  1.00  0.00           H
ATOM   4709  HA  MET C 523     104.219  83.449  92.076  1.00  0.00           H
ATOM   4710  HB2 MET C 523     105.316  83.169  94.376  1.00  0.00           H
ATOM   4711  HB3 MET C 523     104.293  81.798  94.647  1.00  0.00           H
ATOM   4712  HG2 MET C 523     105.299  80.646  92.652  1.00  0.00           H
ATOM   4713  HG3 MET C 523     106.477  81.038  93.871  1.00  0.00           H
ATOM   4714  HE1 MET C 523     107.627  81.456  89.840  1.00  0.00           H
ATOM   4715  HE2 MET C 523     107.437  80.196  91.066  1.00  0.00           H
ATOM   4716  HE3 MET C 523     106.014  80.842  90.232  1.00  0.00           H
ATOM   4717  N   LEU C 524     101.700  81.578  93.118  1.00  0.00           N
ATOM   4718  CA  LEU C 524     100.701  80.582  92.730  1.00  0.00           C
ATOM   4719  C   LEU C 524      99.690  81.114  91.701  1.00  0.00           C
ATOM   4720  O   LEU C 524      99.237  80.328  90.871  1.00  0.00           O
ATOM   4721  CB  LEU C 524      99.992  80.042  93.988  1.00 20.00           C
ATOM   4722  CG  LEU C 524     100.901  79.188  94.903  1.00 20.00           C
ATOM   4723  CD1 LEU C 524     100.247  78.991  96.282  1.00 20.00           C
ATOM   4724  CD2 LEU C 524     101.314  77.850  94.250  1.00 20.00           C
ATOM   4725  H   LEU C 524     101.574  82.013  94.022  1.00  0.00           H
ATOM   4726  HA  LEU C 524     101.222  79.753  92.248  1.00  0.00           H
ATOM   4727  HB2 LEU C 524      99.599  80.889  94.555  1.00  0.00           H
ATOM   4728  HB3 LEU C 524      99.122  79.446  93.705  1.00  0.00           H
ATOM   4729  HG  LEU C 524     101.825  79.738  95.078  1.00  0.00           H
ATOM   4730 HD11 LEU C 524     100.438  78.006  96.708  1.00  0.00           H
ATOM   4731 HD12 LEU C 524     100.641  79.724  96.985  1.00  0.00           H
ATOM   4732 HD13 LEU C 524      99.166  79.129  96.248  1.00  0.00           H
ATOM   4733 HD21 LEU C 524     101.046  76.985  94.855  1.00  0.00           H
ATOM   4734 HD22 LEU C 524     100.847  77.705  93.276  1.00  0.00           H
ATOM   4735 HD23 LEU C 524     102.394  77.813  94.100  1.00  0.00           H
ATOM   4736  N   VAL C 525      99.389  82.426  91.720  1.00  0.00           N
ATOM   4737  CA  VAL C 525      98.596  83.088  90.676  1.00  0.00           C
ATOM   4738  C   VAL C 525      99.377  83.191  89.346  1.00  0.00           C
ATOM   4739  O   VAL C 525      98.762  83.071  88.288  1.00  0.00           O
ATOM   4740  CB  VAL C 525      98.097  84.494  91.125  1.00 20.00           C
ATOM   4741  CG1 VAL C 525      97.473  85.359  90.006  1.00 20.00           C
ATOM   4742  CG2 VAL C 525      97.063  84.364  92.259  1.00 20.00           C
ATOM   4743  H   VAL C 525      99.765  83.019  92.448  1.00  0.00           H
ATOM   4744  HA  VAL C 525      97.716  82.471  90.486  1.00  0.00           H
ATOM   4745  HB  VAL C 525      98.953  85.042  91.522  1.00  0.00           H
```

```
ATOM   4746 HG11 VAL C 525      97.104  86.305  90.403  1.00  0.00           H
ATOM   4747 HG12 VAL C 525      98.183  85.606  89.219  1.00  0.00           H
ATOM   4748 HG13 VAL C 525      96.631  84.847  89.538  1.00  0.00           H
ATOM   4749 HG21 VAL C 525      96.797  85.341  92.662  1.00  0.00           H
ATOM   4750 HG22 VAL C 525      96.147  83.892  91.902  1.00  0.00           H
ATOM   4751 HG23 VAL C 525      97.431  83.760  93.086  1.00  0.00           H
ATOM   4752  N   SER C 526     100.713  83.338  89.420  1.00  0.00           N
ATOM   4753  CA  SER C 526     101.612  83.266  88.266  1.00  0.00           C
ATOM   4754  C   SER C 526     101.632  81.866  87.616  1.00  0.00           C
ATOM   4755  O   SER C 526     101.654  81.789  86.392  1.00  0.00           O
ATOM   4756  CB  SER C 526     103.019  83.742  88.677  1.00 20.00           C
ATOM   4757  OG  SER C 526     103.890  83.790  87.568  1.00 20.00           O
ATOM   4758  H   SER C 526     101.154  83.438  90.324  1.00  0.00           H
ATOM   4759  HA  SER C 526     101.231  83.966  87.519  1.00  0.00           H
ATOM   4760  HB2 SER C 526     102.970  84.736  89.118  1.00  0.00           H
ATOM   4761  HB3 SER C 526     103.458  83.088  89.426  1.00  0.00           H
ATOM   4762  HG  SER C 526     103.630  84.531  87.010  1.00  0.00           H
ATOM   4763  N   VAL C 527     101.562  80.794  88.427  1.00 30.00           N
ATOM   4764  CA  VAL C 527     101.429  79.412  87.952  1.00 30.00           C
ATOM   4765  C   VAL C 527     100.081  79.160  87.245  1.00 30.00           C
ATOM   4766  O   VAL C 527     100.078  78.562  86.170  1.00 30.00           O
ATOM   4767  CB  VAL C 527     101.622  78.388  89.111  1.00 20.00           C
ATOM   4768  CG1 VAL C 527     101.165  76.941  88.823  1.00 20.00           C
ATOM   4769  CG2 VAL C 527     103.089  78.363  89.559  1.00 20.00           C
ATOM   4770  H   VAL C 527     101.559  80.926  89.429  1.00  0.00           H
ATOM   4771  HA  VAL C 527     102.218  79.242  87.216  1.00  0.00           H
ATOM   4772  HB  VAL C 527     101.043  78.732  89.966  1.00  0.00           H
ATOM   4773 HG11 VAL C 527     101.431  76.276  89.646  1.00  0.00           H
ATOM   4774 HG12 VAL C 527     100.085  76.863  88.700  1.00  0.00           H
ATOM   4775 HG13 VAL C 527     101.639  76.552  87.922  1.00  0.00           H
ATOM   4776 HG21 VAL C 527     103.219  77.735  90.440  1.00  0.00           H
ATOM   4777 HG22 VAL C 527     103.731  77.970  88.769  1.00  0.00           H
ATOM   4778 HG23 VAL C 527     103.455  79.357  89.811  1.00  0.00           H
ATOM   4779  N   VAL C 528      98.978  79.651  87.838  1.00  0.00           N
ATOM   4780  CA  VAL C 528      97.622  79.541  87.292  1.00  0.00           C
ATOM   4781  C   VAL C 528      97.469  80.224  85.919  1.00  0.00           C
ATOM   4782  O   VAL C 528      96.938  79.594  85.007  1.00  0.00           O
ATOM   4783  CB  VAL C 528      96.556  80.086  88.294  1.00 20.00           C
ATOM   4784  CG1 VAL C 528      95.162  80.394  87.703  1.00 20.00           C
ATOM   4785  CG2 VAL C 528      96.390  79.119  89.479  1.00 20.00           C
ATOM   4786  H   VAL C 528      99.063  80.126  88.726  1.00  0.00           H
ATOM   4787  HA  VAL C 528      97.429  78.477  87.142  1.00  0.00           H
ATOM   4788  HB  VAL C 528      96.936  81.023  88.702  1.00  0.00           H
ATOM   4789 HG11 VAL C 528      94.458  80.673  88.488  1.00  0.00           H
ATOM   4790 HG12 VAL C 528      95.183  81.226  86.999  1.00  0.00           H
ATOM   4791 HG13 VAL C 528      94.750  79.526  87.186  1.00  0.00           H
ATOM   4792 HG21 VAL C 528      95.751  79.548  90.252  1.00  0.00           H
ATOM   4793 HG22 VAL C 528      95.939  78.179  89.159  1.00  0.00           H
ATOM   4794 HG23 VAL C 528      97.344  78.876  89.944  1.00  0.00           H
ATOM   4795  N   LEU C 529      97.972  81.465  85.787  1.00  0.00           N
ATOM   4796  CA  LEU C 529      97.914  82.243  84.546  1.00  0.00           C
ATOM   4797  C   LEU C 529      98.857  81.707  83.451  1.00  0.00           C
ATOM   4798  O   LEU C 529      98.524  81.844  82.274  1.00  0.00           O
ATOM   4799  CB  LEU C 529      98.147  83.745  84.835  1.00 20.00           C
ATOM   4800  CG  LEU C 529      96.853  84.560  85.081  1.00 20.00           C
ATOM   4801  CD1 LEU C 529      96.065  84.114  86.333  1.00 20.00           C
ATOM   4802  CD2 LEU C 529      97.149  86.073  85.092  1.00 20.00           C
ATOM   4803  H   LEU C 529      98.399  81.919  86.583  1.00  0.00           H
ATOM   4804  HA  LEU C 529      96.906  82.145  84.151  1.00  0.00           H
ATOM   4805  HB2 LEU C 529      98.853  83.876  85.656  1.00  0.00           H
ATOM   4806  HB3 LEU C 529      98.627  84.197  83.965  1.00  0.00           H
ATOM   4807  HG  LEU C 529      96.200  84.385  84.224  1.00  0.00           H
ATOM   4808 HD11 LEU C 529      95.099  83.696  86.050  1.00  0.00           H
ATOM   4809 HD12 LEU C 529      96.585  83.353  86.908  1.00  0.00           H
ATOM   4810 HD13 LEU C 529      95.867  84.932  87.025  1.00  0.00           H
ATOM   4811 HD21 LEU C 529      96.616  86.612  85.875  1.00  0.00           H
ATOM   4812 HD22 LEU C 529      98.209  86.276  85.239  1.00  0.00           H
ATOM   4813 HD23 LEU C 529      96.855  86.526  84.145  1.00  0.00           H
ATOM   4814  N   TYR C 530      99.984  81.086  83.845  1.00  0.00           N
ATOM   4815  CA  TYR C 530     100.946  80.448  82.943  1.00  0.00           C
ATOM   4816  C   TYR C 530     100.360  79.222  82.219  1.00  0.00           C
```

```
ATOM   4817  O   TYR C 530     100.569  79.088  81.015  1.00  0.00           O
ATOM   4818  CB  TYR C 530     102.228  80.104  83.732  1.00 20.00           C
ATOM   4819  CG  TYR C 530     103.342  79.437  82.946  1.00 20.00           C
ATOM   4820  CD1 TYR C 530     104.181  80.219  82.130  1.00 20.00           C
ATOM   4821  CD2 TYR C 530     103.559  78.046  83.041  1.00 20.00           C
ATOM   4822  CE1 TYR C 530     105.238  79.620  81.422  1.00 20.00           C
ATOM   4823  CE2 TYR C 530     104.608  77.442  82.320  1.00 20.00           C
ATOM   4824  CZ  TYR C 530     105.448  78.229  81.506  1.00 20.00           C
ATOM   4825  OH  TYR C 530     106.459  77.647  80.798  1.00 20.00           O
ATOM   4826  H   TYR C 530     100.192  81.020  84.832  1.00  0.00           H
ATOM   4827  HA  TYR C 530     101.212  81.185  82.182  1.00  0.00           H
ATOM   4828  HB2 TYR C 530     102.643  81.019  84.154  1.00  0.00           H
ATOM   4829  HB3 TYR C 530     101.984  79.470  84.584  1.00  0.00           H
ATOM   4830  HD1 TYR C 530     104.018  81.282  82.049  1.00  0.00           H
ATOM   4831  HD2 TYR C 530     102.919  77.439  83.664  1.00  0.00           H
ATOM   4832  HE1 TYR C 530     105.882  80.241  80.821  1.00  0.00           H
ATOM   4833  HE2 TYR C 530     104.760  76.376  82.394  1.00  0.00           H
ATOM   4834  HH  TYR C 530     106.511  76.697  80.932  1.00  0.00           H
ATOM   4835  N   PHE C 531      99.620  78.375  82.956  1.00  0.00           N
ATOM   4836  CA  PHE C 531      98.958  77.188  82.409  1.00  0.00           C
ATOM   4837  C   PHE C 531      97.573  77.466  81.794  1.00  0.00           C
ATOM   4838  O   PHE C 531      97.161  76.691  80.932  1.00  0.00           O
ATOM   4839  CB  PHE C 531      98.923  76.059  83.460  1.00 20.00           C
ATOM   4840  CG  PHE C 531     100.286  75.445  83.746  1.00 20.00           C
ATOM   4841  CD1 PHE C 531     100.986  74.765  82.726  1.00 20.00           C
ATOM   4842  CD2 PHE C 531     100.896  75.594  85.010  1.00 20.00           C
ATOM   4843  CE1 PHE C 531     102.257  74.262  82.969  1.00 20.00           C
ATOM   4844  CE2 PHE C 531     102.166  75.079  85.234  1.00 20.00           C
ATOM   4845  CZ  PHE C 531     102.844  74.419  84.217  1.00 20.00           C
ATOM   4846  H   PHE C 531      99.499  78.544  83.946  1.00  0.00           H
ATOM   4847  HA  PHE C 531      99.558  76.823  81.574  1.00  0.00           H
ATOM   4848  HB2 PHE C 531      98.489  76.429  84.390  1.00  0.00           H
ATOM   4849  HB3 PHE C 531      98.268  75.255  83.121  1.00  0.00           H
ATOM   4850  HD1 PHE C 531     100.539  74.641  81.752  1.00  0.00           H
ATOM   4851  HD2 PHE C 531     100.378  76.102  85.809  1.00  0.00           H
ATOM   4852  HE1 PHE C 531     102.791  73.748  82.183  1.00  0.00           H
ATOM   4853  HE2 PHE C 531     102.632  75.195  86.201  1.00  0.00           H
ATOM   4854  HZ  PHE C 531     103.834  74.027  84.400  1.00  0.00           H
ATOM   4855  N   SER C 532      96.911  78.577  82.177  1.00 30.00           N
ATOM   4856  CA  SER C 532      95.698  79.084  81.515  1.00 30.00           C
ATOM   4857  C   SER C 532      96.000  79.931  80.255  1.00 30.00           C
ATOM   4858  O   SER C 532      95.075  80.524  79.700  1.00 30.00           O
ATOM   4859  CB  SER C 532      94.774  79.792  82.533  1.00 20.00           C
ATOM   4860  OG  SER C 532      95.104  81.150  82.743  1.00 20.00           O
ATOM   4861  H   SER C 532      97.289  79.160  82.912  1.00  0.00           H
ATOM   4862  HA  SER C 532      95.137  78.218  81.161  1.00  0.00           H
ATOM   4863  HB2 SER C 532      94.762  79.265  83.487  1.00  0.00           H
ATOM   4864  HB3 SER C 532      93.749  79.769  82.163  1.00  0.00           H
ATOM   4865  HG  SER C 532      94.907  81.635  81.937  1.00  0.00           H
ATOM   4866  N   GLN C 533      97.271  79.932  79.814  1.00 30.00           N
ATOM   4867  CA  GLN C 533      97.770  80.408  78.522  1.00 30.00           C
ATOM   4868  C   GLN C 533      97.802  81.939  78.340  1.00 30.00           C
ATOM   4869  O   GLN C 533      97.931  82.402  77.206  1.00 30.00           O
ATOM   4870  CB  GLN C 533      97.051  79.685  77.352  1.00 20.00           C
ATOM   4871  CG  GLN C 533      97.155  78.147  77.406  1.00 20.00           C
ATOM   4872  CD  GLN C 533      96.587  77.453  76.166  1.00 20.00           C
ATOM   4873  NE2 GLN C 533      96.827  76.145  76.055  1.00 20.00           N
ATOM   4874  OE1 GLN C 533      95.932  78.068  75.327  1.00 20.00           O
ATOM   4875  H   GLN C 533      97.959  79.443  80.370  1.00  0.00           H
ATOM   4876  HA  GLN C 533      98.814  80.108  78.518  1.00  0.00           H
ATOM   4877  HB2 GLN C 533      96.001  79.976  77.317  1.00  0.00           H
ATOM   4878  HB3 GLN C 533      97.476  80.019  76.408  1.00  0.00           H
ATOM   4879  HG2 GLN C 533      98.198  77.859  77.510  1.00  0.00           H
ATOM   4880  HG3 GLN C 533      96.634  77.759  78.282  1.00  0.00           H
ATOM   4881 HE21 GLN C 533      96.473  75.635  75.259  1.00  0.00           H
ATOM   4882 HE22 GLN C 533      97.362  75.660  76.760  1.00  0.00           H
ATOM   4883  N   ARG C 534      97.708  82.701  79.441  1.00 30.00           N
ATOM   4884  CA  ARG C 534      97.674  84.162  79.423  1.00 30.00           C
ATOM   4885  C   ARG C 534      99.057  84.734  79.776  1.00 30.00           C
ATOM   4886  O   ARG C 534      99.642  84.330  80.781  1.00 30.00           O
ATOM   4887  CB  ARG C 534      96.572  84.635  80.394  1.00 20.00           C
```

```
ATOM   4888  CG  ARG C 534      96.225  86.130  80.259  1.00 20.00           C
ATOM   4889  CD  ARG C 534      94.981  86.551  81.059  1.00 20.00           C
ATOM   4890  NE  ARG C 534      93.742  85.998  80.489  1.00 20.00           N
ATOM   4891  CZ  ARG C 534      93.045  86.519  79.463  1.00 20.00           C
ATOM   4892  NH1 ARG C 534      93.413  87.670  78.882  1.00 20.00           N
ATOM   4893  NH2 ARG C 534      91.961  85.874  79.012  1.00 20.00           N1+
ATOM   4894  H   ARG C 534      97.660  82.262  80.351  1.00  0.00           H
ATOM   4895  HA  ARG C 534      97.403  84.513  78.424  1.00  0.00           H
ATOM   4896  HB2 ARG C 534      95.670  84.054  80.197  1.00  0.00           H
ATOM   4897  HB3 ARG C 534      96.855  84.409  81.423  1.00  0.00           H
ATOM   4898  HG2 ARG C 534      97.068  86.785  80.470  1.00  0.00           H
ATOM   4899  HG3 ARG C 534      95.993  86.285  79.204  1.00  0.00           H
ATOM   4900  HD2 ARG C 534      95.047  86.059  82.030  1.00  0.00           H
ATOM   4901  HD3 ARG C 534      94.941  87.619  81.273  1.00  0.00           H
ATOM   4902  HE  ARG C 534      93.439  85.116  80.876  1.00  0.00           H
ATOM   4903 HH11 ARG C 534      92.884  88.055  78.114  1.00  0.00           H
ATOM   4904 HH12 ARG C 534      94.216  88.173  79.233  1.00  0.00           H
ATOM   4905 HH21 ARG C 534      91.425  86.249  78.244  1.00  0.00           H
ATOM   4906 HH22 ARG C 534      91.668  85.007  79.439  1.00  0.00           H
ATOM   4907  N   LYS C 535      99.537  85.688  78.956  1.00 30.00           N
ATOM   4908  CA  LYS C 535     100.822  86.387  79.120  1.00 30.00           C
ATOM   4909  C   LYS C 535     100.913  87.260  80.394  1.00 30.00           C
ATOM   4910  O   LYS C 535     102.023  87.537  80.845  1.00 30.00           O
ATOM   4911  CB  LYS C 535     101.134  87.165  77.818  1.00 20.00           C
ATOM   4912  CG  LYS C 535     102.467  87.946  77.811  1.00 20.00           C
ATOM   4913  CD  LYS C 535     102.837  88.537  76.437  1.00 20.00           C
ATOM   4914  CE  LYS C 535     103.449  87.536  75.442  1.00 20.00           C
ATOM   4915  NZ  LYS C 535     104.836  87.190  75.801  1.00 20.00           N1+
ATOM   4916  H   LYS C 535      98.993  85.960  78.150  1.00  0.00           H
ATOM   4917  HA  LYS C 535     101.587  85.616  79.216  1.00  0.00           H
ATOM   4918  HB2 LYS C 535     101.143  86.451  76.994  1.00  0.00           H
ATOM   4919  HB3 LYS C 535     100.320  87.860  77.604  1.00  0.00           H
ATOM   4920  HG2 LYS C 535     102.397  88.777  78.513  1.00  0.00           H
ATOM   4921  HG3 LYS C 535     103.275  87.312  78.177  1.00  0.00           H
ATOM   4922  HD2 LYS C 535     101.945  88.976  75.988  1.00  0.00           H
ATOM   4923  HD3 LYS C 535     103.527  89.370  76.581  1.00  0.00           H
ATOM   4924  HE2 LYS C 535     102.848  86.629  75.372  1.00  0.00           H
ATOM   4925  HE3 LYS C 535     103.469  87.981  74.446  1.00  0.00           H
ATOM   4926  HZ1 LYS C 535     105.225  86.551  75.119  1.00  0.00           H
ATOM   4927  HZ2 LYS C 535     105.392  88.034  75.816  1.00  0.00           H
ATOM   4928  HZ3 LYS C 535     104.855  86.759  76.713  1.00  0.00           H
ATOM   4929  N   GLU C 536      99.756  87.615  80.983  1.00 30.00           N
ATOM   4930  CA  GLU C 536      99.600  88.364  82.236  1.00 30.00           C
ATOM   4931  C   GLU C 536     100.116  87.636  83.499  1.00 30.00           C
ATOM   4932  O   GLU C 536     100.164  88.253  84.562  1.00 30.00           O
ATOM   4933  CB  GLU C 536      98.128  88.825  82.328  1.00 20.00           C
ATOM   4934  CG  GLU C 536      97.795  89.811  83.468  1.00  0.00           C
ATOM   4935  CD  GLU C 536      96.391  90.407  83.339  1.00  0.00           C
ATOM   4936  OE1 GLU C 536      95.475  89.655  82.938  1.00  0.00           O
ATOM   4937  OE2 GLU C 536      96.260  91.612  83.643  1.00  0.00           O1-
ATOM   4938  H   GLU C 536      98.893  87.324  80.549  1.00  0.00           H
ATOM   4939  HA  GLU C 536     100.221  89.255  82.149  1.00  0.00           H
ATOM   4940  HB2 GLU C 536      97.840  89.279  81.378  1.00  0.00           H
ATOM   4941  HB3 GLU C 536      97.493  87.946  82.432  1.00  0.00           H
ATOM   4942  HG2 GLU C 536      97.849  89.316  84.437  1.00  0.00           H
ATOM   4943  HG3 GLU C 536      98.525  90.621  83.483  1.00  0.00           H
ATOM   4944  N   TYR C 537     100.570  86.374  83.362  1.00 30.00           N
ATOM   4945  CA  TYR C 537     101.319  85.624  84.378  1.00 30.00           C
ATOM   4946  C   TYR C 537     102.593  86.357  84.856  1.00 30.00           C
ATOM   4947  O   TYR C 537     102.979  86.190  86.013  1.00 30.00           O
ATOM   4948  CB  TYR C 537     101.644  84.211  83.826  1.00 20.00           C
ATOM   4949  CG  TYR C 537     102.982  84.028  83.120  1.00 20.00           C
ATOM   4950  CD1 TYR C 537     103.111  84.353  81.755  1.00 20.00           C
ATOM   4951  CD2 TYR C 537     104.113  83.582  83.839  1.00 20.00           C
ATOM   4952  CE1 TYR C 537     104.364  84.262  81.118  1.00 20.00           C
ATOM   4953  CE2 TYR C 537     105.367  83.495  83.205  1.00 20.00           C
ATOM   4954  CZ  TYR C 537     105.495  83.841  81.845  1.00 20.00           C
ATOM   4955  OH  TYR C 537     106.712  83.765  81.234  1.00 20.00           O
ATOM   4956  H   TYR C 537     100.492  85.927  82.460  1.00  0.00           H
ATOM   4957  HA  TYR C 537     100.665  85.513  85.245  1.00  0.00           H
ATOM   4958  HB2 TYR C 537     100.844  83.849  83.182  1.00  0.00           H
```

```
ATOM   4959  HB3 TYR C 537     101.651  83.529  84.671  1.00  0.00           H
ATOM   4960  HD1 TYR C 537     102.251  84.690  81.201  1.00  0.00           H
ATOM   4961  HD2 TYR C 537     104.025  83.325  84.884  1.00  0.00           H
ATOM   4962  HE1 TYR C 537     104.457  84.523  80.074  1.00  0.00           H
ATOM   4963  HE2 TYR C 537     106.227  83.165  83.768  1.00  0.00           H
ATOM   4964  HH  TYR C 537     107.406  83.474  81.829  1.00  0.00           H
ATOM   4965  N   VAL C 538     103.208  87.144  83.951  1.00 30.00           N
ATOM   4966  CA  VAL C 538     104.413  87.933  84.189  1.00 30.00           C
ATOM   4967  C   VAL C 538     104.204  89.058  85.223  1.00 30.00           C
ATOM   4968  O   VAL C 538     105.133  89.334  85.974  1.00 30.00           O
ATOM   4969  CB  VAL C 538     104.973  88.533  82.863  1.00 20.00           C
ATOM   4970  CG1 VAL C 538     104.140  89.692  82.273  1.00 20.00           C
ATOM   4971  CG2 VAL C 538     106.443  88.970  82.997  1.00 20.00           C
ATOM   4972  H   VAL C 538     102.819  87.212  83.020  1.00  0.00           H
ATOM   4973  HA  VAL C 538     105.160  87.246  84.588  1.00  0.00           H
ATOM   4974  HB  VAL C 538     104.951  87.728  82.127  1.00  0.00           H
ATOM   4975 HG11 VAL C 538     104.453  89.915  81.252  1.00  0.00           H
ATOM   4976 HG12 VAL C 538     103.078  89.452  82.243  1.00  0.00           H
ATOM   4977 HG13 VAL C 538     104.251  90.611  82.850  1.00  0.00           H
ATOM   4978 HG21 VAL C 538     106.834  89.308  82.037  1.00  0.00           H
ATOM   4979 HG22 VAL C 538     106.566  89.795  83.700  1.00  0.00           H
ATOM   4980 HG23 VAL C 538     107.073  88.147  83.336  1.00  0.00           H
ATOM   4981  N   ALA C 539     102.990  89.636  85.293  1.00  0.00           N
ATOM   4982  CA  ALA C 539     102.607  90.643  86.286  1.00  0.00           C
ATOM   4983  C   ALA C 539     102.659  90.108  87.726  1.00  0.00           C
ATOM   4984  O   ALA C 539     103.165  90.795  88.612  1.00  0.00           O
ATOM   4985  CB  ALA C 539     101.197  91.161  85.962  1.00  0.00           C
ATOM   4986  H   ALA C 539     102.261  89.337  84.661  1.00  0.00           H
ATOM   4987  HA  ALA C 539     103.309  91.474  86.204  1.00  0.00           H
ATOM   4988  HB1 ALA C 539     100.899  91.944  86.659  1.00  0.00           H
ATOM   4989  HB2 ALA C 539     101.149  91.572  84.953  1.00  0.00           H
ATOM   4990  HB3 ALA C 539     100.449  90.371  86.033  1.00  0.00           H
ATOM   4991  N   SER C 540     102.170  88.873  87.909  1.00  0.00           N
ATOM   4992  CA  SER C 540     102.158  88.159  89.181  1.00  0.00           C
ATOM   4993  C   SER C 540     103.538  87.589  89.564  1.00  0.00           C
ATOM   4994  O   SER C 540     103.837  87.522  90.754  1.00  0.00           O
ATOM   4995  CB  SER C 540     101.076  87.069  89.108  1.00 20.00           C
ATOM   4996  OG  SER C 540      99.796  87.668  89.117  1.00 20.00           O
ATOM   4997  H   SER C 540     101.768  88.381  87.123  1.00  0.00           H
ATOM   4998  HA  SER C 540     101.882  88.861  89.972  1.00  0.00           H
ATOM   4999  HB2 SER C 540     101.186  86.451  88.216  1.00  0.00           H
ATOM   5000  HB3 SER C 540     101.127  86.405  89.967  1.00  0.00           H
ATOM   5001  HG  SER C 540      99.637  88.044  89.986  1.00  0.00           H
ATOM   5002  N   MET C 541     104.375  87.241  88.566  1.00  0.00           N
ATOM   5003  CA  MET C 541     105.782  86.860  88.748  1.00  0.00           C
ATOM   5004  C   MET C 541     106.632  88.035  89.262  1.00  0.00           C
ATOM   5005  O   MET C 541     107.419  87.855  90.188  1.00  0.00           O
ATOM   5006  CB  MET C 541     106.342  86.292  87.423  1.00 20.00           C
ATOM   5007  CG  MET C 541     107.832  85.902  87.449  1.00 20.00           C
ATOM   5008  SD  MET C 541     108.396  84.994  85.982  1.00 20.00           S
ATOM   5009  CE  MET C 541     107.724  83.350  86.347  1.00 20.00           C
ATOM   5010  H   MET C 541     104.055  87.310  87.609  1.00  0.00           H
ATOM   5011  HA  MET C 541     105.819  86.066  89.498  1.00  0.00           H
ATOM   5012  HB2 MET C 541     105.752  85.436  87.111  1.00  0.00           H
ATOM   5013  HB3 MET C 541     106.211  87.032  86.635  1.00  0.00           H
ATOM   5014  HG2 MET C 541     108.451  86.796  87.528  1.00  0.00           H
ATOM   5015  HG3 MET C 541     108.045  85.300  88.332  1.00  0.00           H
ATOM   5016  HE1 MET C 541     108.024  82.643  85.573  1.00  0.00           H
ATOM   5017  HE2 MET C 541     108.097  82.988  87.305  1.00  0.00           H
ATOM   5018  HE3 MET C 541     106.635  83.374  86.385  1.00  0.00           H
ATOM   5019  N   VAL C 542     106.425  89.211  88.649  1.00  0.00           N
ATOM   5020  CA  VAL C 542     107.057  90.490  88.964  1.00  0.00           C
ATOM   5021  C   VAL C 542     106.761  90.973  90.397  1.00  0.00           C
ATOM   5022  O   VAL C 542     107.693  91.391  91.084  1.00  0.00           O
ATOM   5023  CB  VAL C 542     106.613  91.558  87.919  1.00 20.00           C
ATOM   5024  CG1 VAL C 542     106.744  93.034  88.334  1.00 20.00           C
ATOM   5025  CG2 VAL C 542     107.348  91.344  86.586  1.00 20.00           C
ATOM   5026  H   VAL C 542     105.770  89.242  87.878  1.00  0.00           H
ATOM   5027  HA  VAL C 542     108.137  90.349  88.883  1.00  0.00           H
ATOM   5028  HB  VAL C 542     105.556  91.395  87.714  1.00  0.00           H
ATOM   5029 HG11 VAL C 542     106.512  93.684  87.492  1.00  0.00           H
```

ATOM	5030	HG12	VAL	C	542	106.058	93.305	89.138	1.00	0.00	H
ATOM	5031	HG13	VAL	C	542	107.759	93.255	88.657	1.00	0.00	H
ATOM	5032	HG21	VAL	C	542	106.845	91.866	85.774	1.00	0.00	H
ATOM	5033	HG22	VAL	C	542	108.365	91.717	86.640	1.00	0.00	H
ATOM	5034	HG23	VAL	C	542	107.417	90.293	86.308	1.00	0.00	H
ATOM	5035	N	PHE	C	543	105.499	90.878	90.836	1.00	0.00	N
ATOM	5036	CA	PHE	C	543	105.123	91.264	92.195	1.00	0.00	C
ATOM	5037	C	PHE	C	543	105.554	90.244	93.262	1.00	0.00	C
ATOM	5038	O	PHE	C	543	105.885	90.661	94.372	1.00	0.00	O
ATOM	5039	CB	PHE	C	543	103.622	91.595	92.276	1.00	20.00	C
ATOM	5040	CG	PHE	C	543	103.159	92.812	91.484	1.00	20.00	C
ATOM	5041	CD1	PHE	C	543	103.916	94.008	91.447	1.00	20.00	C
ATOM	5042	CD2	PHE	C	543	101.866	92.817	90.919	1.00	20.00	C
ATOM	5043	CE1	PHE	C	543	103.417	95.132	90.806	1.00	20.00	C
ATOM	5044	CE2	PHE	C	543	101.379	93.956	90.292	1.00	20.00	C
ATOM	5045	CZ	PHE	C	543	102.151	95.108	90.237	1.00	20.00	C
ATOM	5046	H	PHE	C	543	104.756	90.643	90.187	1.00	0.00	H
ATOM	5047	HA	PHE	C	543	105.676	92.166	92.455	1.00	0.00	H
ATOM	5048	HB2	PHE	C	543	103.051	90.725	91.949	1.00	0.00	H
ATOM	5049	HB3	PHE	C	543	103.341	91.763	93.315	1.00	0.00	H
ATOM	5050	HD1	PHE	C	543	104.890	94.073	91.907	1.00	0.00	H
ATOM	5051	HD2	PHE	C	543	101.247	91.932	90.968	1.00	0.00	H
ATOM	5052	HE1	PHE	C	543	104.011	96.032	90.754	1.00	0.00	H
ATOM	5053	HE2	PHE	C	543	100.393	93.946	89.851	1.00	0.00	H
ATOM	5054	HZ	PHE	C	543	101.766	95.992	89.751	1.00	0.00	H
ATOM	5055	N	SER	C	544	105.621	88.947	92.918	1.00	0.00	N
ATOM	5056	CA	SER	C	544	106.206	87.930	93.797	1.00	0.00	C
ATOM	5057	C	SER	C	544	107.716	88.159	94.013	1.00	0.00	C
ATOM	5058	O	SER	C	544	108.197	87.989	95.131	1.00	0.00	O
ATOM	5059	CB	SER	C	544	105.917	86.528	93.222	1.00	20.00	C
ATOM	5060	OG	SER	C	544	106.454	85.503	94.032	1.00	20.00	O
ATOM	5061	H	SER	C	544	105.409	88.655	91.973	1.00	0.00	H
ATOM	5062	HA	SER	C	544	105.716	88.001	94.771	1.00	0.00	H
ATOM	5063	HB2	SER	C	544	104.847	86.357	93.145	1.00	0.00	H
ATOM	5064	HB3	SER	C	544	106.311	86.431	92.212	1.00	0.00	H
ATOM	5065	HG	SER	C	544	105.879	85.385	94.795	1.00	0.00	H
ATOM	5066	N	LEU	C	545	108.427	88.514	92.932	1.00	0.00	N
ATOM	5067	CA	LEU	C	545	109.874	88.704	92.915	1.00	0.00	C
ATOM	5068	C	LEU	C	545	110.286	89.970	93.677	1.00	0.00	C
ATOM	5069	O	LEU	C	545	111.178	89.897	94.524	1.00	0.00	O
ATOM	5070	CB	LEU	C	545	110.355	88.644	91.447	1.00	20.00	C
ATOM	5071	CG	LEU	C	545	111.884	88.559	91.234	1.00	20.00	C
ATOM	5072	CD1	LEU	C	545	112.234	87.684	90.012	1.00	20.00	C
ATOM	5073	CD2	LEU	C	545	112.527	89.952	91.120	1.00	20.00	C
ATOM	5074	H	LEU	C	545	107.970	88.553	92.028	1.00	0.00	H
ATOM	5075	HA	LEU	C	545	110.327	87.866	93.445	1.00	0.00	H
ATOM	5076	HB2	LEU	C	545	109.921	87.734	91.032	1.00	0.00	H
ATOM	5077	HB3	LEU	C	545	109.930	89.459	90.858	1.00	0.00	H
ATOM	5078	HG	LEU	C	545	112.317	88.067	92.107	1.00	0.00	H
ATOM	5079	HD11	LEU	C	545	113.071	88.081	89.439	1.00	0.00	H
ATOM	5080	HD12	LEU	C	545	112.513	86.677	90.328	1.00	0.00	H
ATOM	5081	HD13	LEU	C	545	111.396	87.581	89.322	1.00	0.00	H
ATOM	5082	HD21	LEU	C	545	113.522	89.954	91.561	1.00	0.00	H
ATOM	5083	HD22	LEU	C	545	112.622	90.260	90.080	1.00	0.00	H
ATOM	5084	HD23	LEU	C	545	111.951	90.732	91.616	1.00	0.00	H
ATOM	5085	N	ALA	C	546	109.581	91.084	93.418	1.00	0.00	N
ATOM	5086	CA	ALA	C	546	109.756	92.351	94.131	1.00	0.00	C
ATOM	5087	C	ALA	C	546	109.529	92.198	95.646	1.00	0.00	C
ATOM	5088	O	ALA	C	546	110.392	92.602	96.428	1.00	0.00	O
ATOM	5089	CB	ALA	C	546	108.822	93.418	93.538	1.00	20.00	C
ATOM	5090	H	ALA	C	546	108.878	91.066	92.685	1.00	0.00	H
ATOM	5091	HA	ALA	C	546	110.788	92.675	93.980	1.00	0.00	H
ATOM	5092	HB1	ALA	C	546	108.954	94.375	94.045	1.00	0.00	H
ATOM	5093	HB2	ALA	C	546	109.028	93.582	92.482	1.00	0.00	H
ATOM	5094	HB3	ALA	C	546	107.774	93.133	93.628	1.00	0.00	H
ATOM	5095	N	MET	C	547	108.412	91.548	96.019	1.00	0.00	N
ATOM	5096	CA	MET	C	547	108.071	91.231	97.408	1.00	0.00	C
ATOM	5097	C	MET	C	547	109.111	90.293	98.052	1.00	0.00	C
ATOM	5098	O	MET	C	547	109.540	90.529	99.178	1.00	0.00	O
ATOM	5099	CB	MET	C	547	106.639	90.649	97.486	1.00	20.00	C
ATOM	5100	CG	MET	C	547	106.113	90.405	98.915	1.00	20.00	C

ATOM	5101	SD	MET	C	547	105.912	91.893	99.931	1.00	20.00	S
ATOM	5102	CE	MET	C	547	104.347	92.540	99.291	1.00	20.00	C
ATOM	5103	H	MET	C	547	107.759	91.233	95.312	1.00	0.00	H
ATOM	5104	HA	MET	C	547	108.073	92.172	97.960	1.00	0.00	H
ATOM	5105	HB2	MET	C	547	105.951	91.323	96.979	1.00	0.00	H
ATOM	5106	HB3	MET	C	547	106.596	89.710	96.931	1.00	0.00	H
ATOM	5107	HG2	MET	C	547	106.785	89.740	99.454	1.00	0.00	H
ATOM	5108	HG3	MET	C	547	105.156	89.886	98.874	1.00	0.00	H
ATOM	5109	HE1	MET	C	547	104.116	93.492	99.768	1.00	0.00	H
ATOM	5110	HE2	MET	C	547	104.404	92.700	98.217	1.00	0.00	H
ATOM	5111	HE3	MET	C	547	103.537	91.844	99.501	1.00	0.00	H
ATOM	5112	N	GLY	C	548	109.536	89.260	97.316	1.00	0.00	N
ATOM	5113	CA	GLY	C	548	110.454	88.239	97.810	1.00	0.00	C
ATOM	5114	C	GLY	C	548	111.829	88.806	98.179	1.00	0.00	C
ATOM	5115	O	GLY	C	548	112.395	88.402	99.199	1.00	0.00	O
ATOM	5116	H	GLY	C	548	109.142	89.105	96.392	1.00	0.00	H
ATOM	5117	HA2	GLY	C	548	110.017	87.783	98.696	1.00	0.00	H
ATOM	5118	HA3	GLY	C	548	110.567	87.462	97.056	1.00	0.00	H
ATOM	5119	N	TRP	C	549	112.354	89.761	97.400	1.00	0.00	N
ATOM	5120	CA	TRP	C	549	113.619	90.419	97.724	1.00	0.00	C
ATOM	5121	C	TRP	C	549	113.534	91.284	98.993	1.00	0.00	C
ATOM	5122	O	TRP	C	549	114.400	91.143	99.854	1.00	0.00	O
ATOM	5123	CB	TRP	C	549	114.207	91.170	96.516	1.00	20.00	C
ATOM	5124	CG	TRP	C	549	114.973	90.306	95.554	1.00	20.00	C
ATOM	5125	CD1	TRP	C	549	114.599	89.960	94.300	1.00	20.00	C
ATOM	5126	CD2	TRP	C	549	116.249	89.634	95.788	1.00	20.00	C
ATOM	5127	CE2	TRP	C	549	116.597	88.896	94.618	1.00	20.00	C
ATOM	5128	CE3	TRP	C	549	117.151	89.570	96.877	1.00	20.00	C
ATOM	5129	NE1	TRP	C	549	115.569	89.147	93.738	1.00	20.00	N
ATOM	5130	CZ2	TRP	C	549	117.768	88.123	94.535	1.00	20.00	C
ATOM	5131	CZ3	TRP	C	549	118.336	88.809	96.801	1.00	20.00	C
ATOM	5132	CH2	TRP	C	549	118.642	88.080	95.635	1.00	20.00	C
ATOM	5133	H	TRP	C	549	111.861	90.058	96.562	1.00	0.00	H
ATOM	5134	HA	TRP	C	549	114.339	89.638	97.982	1.00	0.00	H
ATOM	5135	HB2	TRP	C	549	113.426	91.720	95.989	1.00	0.00	H
ATOM	5136	HB3	TRP	C	549	114.920	91.920	96.865	1.00	0.00	H
ATOM	5137	HD1	TRP	C	549	113.688	90.298	93.827	1.00	0.00	H
ATOM	5138	HE1	TRP	C	549	115.556	88.801	92.778	1.00	0.00	H
ATOM	5139	HE3	TRP	C	549	116.930	90.118	97.782	1.00	0.00	H
ATOM	5140	HZ2	TRP	C	549	117.998	87.574	93.634	1.00	0.00	H
ATOM	5141	HZ3	TRP	C	549	119.011	88.783	97.645	1.00	0.00	H
ATOM	5142	HH2	TRP	C	549	119.550	87.497	95.584	1.00	0.00	H
ATOM	5143	N	THR	C	550	112.480	92.097	99.152	1.00	0.00	N
ATOM	5144	CA	THR	C	550	112.300	92.888	100.376	1.00	0.00	C
ATOM	5145	C	THR	C	550	111.938	92.024	101.609	1.00	0.00	C
ATOM	5146	O	THR	C	550	112.305	92.406	102.718	1.00	0.00	O
ATOM	5147	CB	THR	C	550	111.289	94.052	100.200	1.00	20.00	C
ATOM	5148	OG1	THR	C	550	111.165	94.849	101.370	1.00	0.00	O
ATOM	5149	CG2	THR	C	550	109.890	93.588	99.799	1.00	0.00	C
ATOM	5150	H	THR	C	550	111.749	92.141	98.451	1.00	0.00	H
ATOM	5151	HA	THR	C	550	113.263	93.351	100.602	1.00	0.00	H
ATOM	5152	HB1	THR	C	550	111.666	94.708	99.414	1.00	0.00	H
ATOM	5153	HG1	THR	C	550	111.365	94.291	102.136	1.00	0.00	H
ATOM	5154	HG21	THR	C	550	109.251	94.428	99.567	1.00	0.00	H
ATOM	5155	HG22	THR	C	550	109.939	93.005	98.888	1.00	0.00	H
ATOM	5156	HG23	THR	C	550	109.398	92.987	100.565	1.00	0.00	H
ATOM	5157	N	ASN	C	551	111.313	90.851	101.418	1.00	0.00	N
ATOM	5158	CA	ASN	C	551	111.084	89.852	102.477	1.00	0.00	C
ATOM	5159	C	ASN	C	551	112.390	89.296	103.066	1.00	0.00	C
ATOM	5160	O	ASN	C	551	112.372	88.807	104.194	1.00	0.00	O
ATOM	5161	CB	ASN	C	551	110.208	88.694	101.949	1.00	20.00	C
ATOM	5162	CG	ASN	C	551	108.721	89.016	101.868	1.00	20.00	C
ATOM	5163	ND2	ASN	C	551	107.929	88.110	101.315	1.00	20.00	N
ATOM	5164	OD1	ASN	C	551	108.270	90.065	102.309	1.00	20.00	O
ATOM	5165	H	ASN	C	551	110.903	90.647	100.509	1.00	0.00	H
ATOM	5166	HA	ASN	C	551	110.550	90.388	103.266	1.00	0.00	H
ATOM	5167	HB2	ASN	C	551	110.561	88.347	100.982	1.00	0.00	H
ATOM	5168	HB3	ASN	C	551	110.282	87.839	102.621	1.00	0.00	H
ATOM	5169	HD21	ASN	C	551	106.935	88.284	101.275	1.00	0.00	H
ATOM	5170	HD22	ASN	C	551	108.282	87.203	100.997	1.00	0.00	H
ATOM	5171	N	MET	C	552	113.523	89.431	102.358	1.00	30.00	N

```
ATOM   5172  CA  MET C 552     114.846  89.094 102.888  1.00 30.00           C
ATOM   5173  C   MET C 552     115.254  89.960 104.100  1.00 30.00           C
ATOM   5174  O   MET C 552     116.043  89.498 104.924  1.00 30.00           O
ATOM   5175  CB  MET C 552     115.889  89.136 101.755  1.00 20.00           C
ATOM   5176  CG  MET C 552     117.188  88.383 102.087  1.00 20.00           C
ATOM   5177  SD  MET C 552     118.319  88.131 100.690  1.00 20.00           S
ATOM   5178  CE  MET C 552     117.318  87.084  99.596  1.00 20.00           C
ATOM   5179  H   MET C 552     113.490  89.881 101.450  1.00  0.00           H
ATOM   5180  HA  MET C 552     114.774  88.064 103.240  1.00  0.00           H
ATOM   5181  HB2 MET C 552     115.451  88.730 100.846  1.00  0.00           H
ATOM   5182  HB3 MET C 552     116.137  90.173 101.524  1.00  0.00           H
ATOM   5183  HG2 MET C 552     117.737  88.930 102.854  1.00  0.00           H
ATOM   5184  HG3 MET C 552     116.962  87.406 102.515  1.00  0.00           H
ATOM   5185  HE1 MET C 552     117.924  86.744  98.757  1.00  0.00           H
ATOM   5186  HE2 MET C 552     116.941  86.208 100.123  1.00  0.00           H
ATOM   5187  HE3 MET C 552     116.477  87.641  99.187  1.00  0.00           H
ATOM   5188  N   LEU C 553     114.647  91.150 104.244  1.00 30.00           N
ATOM   5189  CA  LEU C 553     114.801  92.036 105.401  1.00 30.00           C
ATOM   5190  C   LEU C 553     114.227  91.450 106.705  1.00 30.00           C
ATOM   5191  O   LEU C 553     114.521  91.991 107.770  1.00 30.00           O
ATOM   5192  CB  LEU C 553     114.172  93.417 105.117  1.00 20.00           C
ATOM   5193  CG  LEU C 553     114.788  94.168 103.918  1.00 20.00           C
ATOM   5194  CD1 LEU C 553     113.929  95.385 103.527  1.00 20.00           C
ATOM   5195  CD2 LEU C 553     116.266  94.543 104.150  1.00 20.00           C
ATOM   5196  H   LEU C 553     113.960  91.448 103.558  1.00  0.00           H
ATOM   5197  HA  LEU C 553     115.871  92.170 105.564  1.00  0.00           H
ATOM   5198  HB2 LEU C 553     113.102  93.282 104.960  1.00  0.00           H
ATOM   5199  HB3 LEU C 553     114.249  94.050 106.004  1.00  0.00           H
ATOM   5200  HG  LEU C 553     114.753  93.498 103.061  1.00  0.00           H
ATOM   5201 HD11 LEU C 553     114.408  96.335 103.759  1.00  0.00           H
ATOM   5202 HD12 LEU C 553     113.729  95.376 102.455  1.00  0.00           H
ATOM   5203 HD13 LEU C 553     112.964  95.388 104.034  1.00  0.00           H
ATOM   5204 HD21 LEU C 553     116.507  95.533 103.767  1.00  0.00           H
ATOM   5205 HD22 LEU C 553     116.533  94.532 105.207  1.00  0.00           H
ATOM   5206 HD23 LEU C 553     116.927  93.842 103.639  1.00  0.00           H
ATOM   5207  N   TYR C 554     113.466  90.345 106.634  1.00 30.00           N
ATOM   5208  CA  TYR C 554     113.080  89.533 107.791  1.00 30.00           C
ATOM   5209  C   TYR C 554     114.304  89.059 108.598  1.00 30.00           C
ATOM   5210  O   TYR C 554     114.321  89.178 109.823  1.00 30.00           O
ATOM   5211  CB  TYR C 554     112.207  88.346 107.327  1.00 20.00           C
ATOM   5212  CG  TYR C 554     111.929  87.271 108.366  1.00 20.00           C
ATOM   5213  CD1 TYR C 554     110.942  87.472 109.351  1.00 20.00           C
ATOM   5214  CD2 TYR C 554     112.677  86.075 108.366  1.00 20.00           C
ATOM   5215  CE1 TYR C 554     110.691  86.477 110.314  1.00 20.00           C
ATOM   5216  CE2 TYR C 554     112.443  85.089 109.340  1.00 20.00           C
ATOM   5217  CZ  TYR C 554     111.443  85.286 110.309  1.00 20.00           C
ATOM   5218  OH  TYR C 554     111.207  84.320 111.239  1.00 20.00           O
ATOM   5219  H   TYR C 554     113.217  89.971 105.725  1.00  0.00           H
ATOM   5220  HA  TYR C 554     112.470  90.159 108.439  1.00  0.00           H
ATOM   5221  HB2 TYR C 554     111.259  88.725 106.943  1.00  0.00           H
ATOM   5222  HB3 TYR C 554     112.695  87.846 106.491  1.00  0.00           H
ATOM   5223  HD1 TYR C 554     110.382  88.395 109.374  1.00  0.00           H
ATOM   5224  HD2 TYR C 554     113.442  85.911 107.624  1.00  0.00           H
ATOM   5225  HE1 TYR C 554     109.927  86.645 111.060  1.00  0.00           H
ATOM   5226  HE2 TYR C 554     113.023  84.177 109.332  1.00  0.00           H
ATOM   5227  HH  TYR C 554     110.379  84.454 111.710  1.00  0.00           H
ATOM   5228  N   TYR C 555     115.337  88.593 107.882  1.00 30.00           N
ATOM   5229  CA  TYR C 555     116.546  87.979 108.434  1.00 30.00           C
ATOM   5230  C   TYR C 555     117.561  88.990 109.002  1.00 30.00           C
ATOM   5231  O   TYR C 555     118.702  88.619 109.263  1.00 30.00           O
ATOM   5232  CB  TYR C 555     117.184  87.059 107.375  1.00 20.00           C
ATOM   5233  CG  TYR C 555     116.287  85.927 106.899  1.00 20.00           C
ATOM   5234  CD1 TYR C 555     116.191  84.738 107.652  1.00 20.00           C
ATOM   5235  CD2 TYR C 555     115.547  86.057 105.705  1.00 20.00           C
ATOM   5236  CE1 TYR C 555     115.371  83.684 107.207  1.00 20.00           C
ATOM   5237  CE2 TYR C 555     114.712  85.011 105.272  1.00 20.00           C
ATOM   5238  CZ  TYR C 555     114.629  83.820 106.018  1.00 20.00           C
ATOM   5239  OH  TYR C 555     113.829  82.799 105.599  1.00 20.00           O
ATOM   5240  H   TYR C 555     115.298  88.646 106.872  1.00  0.00           H
ATOM   5241  HA  TYR C 555     116.245  87.353 109.273  1.00  0.00           H
ATOM   5242  HB2 TYR C 555     117.504  87.652 106.517  1.00  0.00           H
```

```
ATOM   5243  HB3 TYR C 555     118.095  86.609 107.774  1.00  0.00           H
ATOM   5244  HD1 TYR C 555     116.748  84.624 108.572  1.00  0.00           H
ATOM   5245  HD2 TYR C 555     115.612  86.963 105.124  1.00  0.00           H
ATOM   5246  HE1 TYR C 555     115.307  82.772 107.783  1.00  0.00           H
ATOM   5247  HE2 TYR C 555     114.134  85.133 104.369  1.00  0.00           H
ATOM   5248  HH  TYR C 555     113.311  83.019 104.820  1.00  0.00           H
ATOM   5249  N   THR C 556     117.138  90.242 109.206  1.00 30.00           N
ATOM   5250  CA  THR C 556     117.893  91.239 109.955  1.00 30.00           C
ATOM   5251  C   THR C 556     117.785  90.987 111.479  1.00 30.00           C
ATOM   5252  O   THR C 556     118.704  91.345 112.217  1.00 30.00           O
ATOM   5253  CB  THR C 556     117.354  92.654 109.636  1.00 20.00           C
ATOM   5254  CG2 THR C 556     117.594  93.065 108.176  1.00 20.00           C
ATOM   5255  OG1 THR C 556     115.968  92.754 109.921  1.00 20.00           O
ATOM   5256  H   THR C 556     116.175  90.476 109.009  1.00  0.00           H
ATOM   5257  HA  THR C 556     118.948  91.186 109.677  1.00  0.00           H
ATOM   5258  HB  THR C 556     117.865  93.377 110.267  1.00  0.00           H
ATOM   5259  HG1 THR C 556     115.446  92.527 109.130  1.00  0.00           H
ATOM   5260 HG21 THR C 556     117.166  94.045 107.962  1.00  0.00           H
ATOM   5261 HG22 THR C 556     118.663  93.124 107.965  1.00  0.00           H
ATOM   5262 HG23 THR C 556     117.170  92.350 107.474  1.00  0.00           H
ATOM   5263  N   ARG C 557     116.710  90.321 111.938  1.00 30.00           N
ATOM   5264  CA  ARG C 557     116.618  89.780 113.294  1.00 30.00           C
ATOM   5265  C   ARG C 557     117.690  88.688 113.483  1.00 30.00           C
ATOM   5266  O   ARG C 557     117.945  87.932 112.552  1.00 30.00           O
ATOM   5267  CB  ARG C 557     115.178  89.294 113.573  1.00 20.00           C
ATOM   5268  CG  ARG C 557     114.764  87.988 112.862  1.00 20.00           C
ATOM   5269  CD  ARG C 557     113.274  87.649 113.000  1.00 20.00           C
ATOM   5270  NE  ARG C 557     112.397  88.695 112.456  1.00 20.00           N
ATOM   5271  CZ  ARG C 557     111.104  88.870 112.774  1.00 20.00           C
ATOM   5272  NH1 ARG C 557     110.507  88.126 113.716  1.00 20.00           N
ATOM   5273  NH2 ARG C 557     110.394  89.808 112.144  1.00 20.00           N1+
ATOM   5274  H   ARG C 557     116.000  90.039 111.275  1.00  0.00           H
ATOM   5275  HA  ARG C 557     116.830  90.597 113.985  1.00  0.00           H
ATOM   5276  HB2 ARG C 557     114.495  90.102 113.303  1.00  0.00           H
ATOM   5277  HB3 ARG C 557     115.049  89.155 114.648  1.00  0.00           H
ATOM   5278  HG2 ARG C 557     115.038  88.002 111.811  1.00  0.00           H
ATOM   5279  HG3 ARG C 557     115.334  87.161 113.288  1.00  0.00           H
ATOM   5280  HD2 ARG C 557     113.051  87.458 114.049  1.00  0.00           H
ATOM   5281  HD3 ARG C 557     113.047  86.739 112.444  1.00  0.00           H
ATOM   5282  HE  ARG C 557     112.787  89.225 111.683  1.00  0.00           H
ATOM   5283 HH11 ARG C 557     109.531  88.254 113.938  1.00  0.00           H
ATOM   5284 HH12 ARG C 557     111.039  87.463 114.261  1.00  0.00           H
ATOM   5285 HH21 ARG C 557     109.425  89.979 112.371  1.00  0.00           H
ATOM   5286 HH22 ARG C 557     110.840  90.420 111.468  1.00  0.00           H
ATOM   5287  N   GLY C 558     118.329  88.650 114.655  1.00 30.00           N
ATOM   5288  CA  GLY C 558     119.492  87.792 114.900  1.00 30.00           C
ATOM   5289  C   GLY C 558     120.810  88.555 114.651  1.00 30.00           C
ATOM   5290  O   GLY C 558     121.868  88.053 115.022  1.00 30.00           O
ATOM   5291  H   GLY C 558     118.083  89.310 115.379  1.00  0.00           H
ATOM   5292  HA2 GLY C 558     119.474  86.893 114.281  1.00  0.00           H
ATOM   5293  HA3 GLY C 558     119.458  87.451 115.933  1.00  0.00           H
ATOM   5294  N   PHE C 559     120.755  89.768 114.076  1.00 30.00           N
ATOM   5295  CA  PHE C 559     121.868  90.709 113.969  1.00 30.00           C
ATOM   5296  C   PHE C 559     121.360  92.005 114.607  1.00 30.00           C
ATOM   5297  O   PHE C 559     120.539  92.688 114.000  1.00 30.00           O
ATOM   5298  CB  PHE C 559     122.254  90.920 112.485  1.00 20.00           C
ATOM   5299  CG  PHE C 559     122.668  89.669 111.733  1.00 20.00           C
ATOM   5300  CD1 PHE C 559     123.967  89.141 111.883  1.00 20.00           C
ATOM   5301  CD2 PHE C 559     121.712  88.938 110.994  1.00 20.00           C
ATOM   5302  CE1 PHE C 559     124.311  87.955 111.247  1.00 20.00           C
ATOM   5303  CE2 PHE C 559     122.075  87.755 110.365  1.00 20.00           C
ATOM   5304  CZ  PHE C 559     123.372  87.271 110.484  1.00 20.00           C
ATOM   5305  H   PHE C 559     119.866  90.119 113.747  1.00  0.00           H
ATOM   5306  HA  PHE C 559     122.743  90.358 114.519  1.00  0.00           H
ATOM   5307  HB2 PHE C 559     121.440  91.389 111.933  1.00  0.00           H
ATOM   5308  HB3 PHE C 559     123.085  91.625 112.435  1.00  0.00           H
ATOM   5309  HD1 PHE C 559     124.699  89.659 112.485  1.00  0.00           H
ATOM   5310  HD2 PHE C 559     120.697  89.300 110.910  1.00  0.00           H
ATOM   5311  HE1 PHE C 559     125.310  87.558 111.351  1.00  0.00           H
ATOM   5312  HE2 PHE C 559     121.345  87.207 109.787  1.00  0.00           H
ATOM   5313  HZ  PHE C 559     123.647  86.349 109.994  1.00  0.00           H
```

```
ATOM   5314  N   GLN C 560     121.802  92.302 115.840  1.00 30.00           N
ATOM   5315  CA  GLN C 560     121.264  93.401 116.655  1.00 30.00           C
ATOM   5316  C   GLN C 560     121.475  94.810 116.054  1.00 30.00           C
ATOM   5317  O   GLN C 560     120.653  95.691 116.302  1.00 30.00           O
ATOM   5318  CB  GLN C 560     121.799  93.263 118.097  1.00 20.00           C
ATOM   5319  CG  GLN C 560     121.098  94.192 119.113  1.00 20.00           C
ATOM   5320  CD  GLN C 560     121.333  93.844 120.588  1.00 20.00           C
ATOM   5321  NE2 GLN C 560     122.416  93.133 120.912  1.00 20.00           N
ATOM   5322  OE1 GLN C 560     120.533  94.225 121.441  1.00 20.00           O
ATOM   5323  H   GLN C 560     122.478  91.701 116.288  1.00  0.00           H
ATOM   5324  HA  GLN C 560     120.183  93.254 116.693  1.00  0.00           H
ATOM   5325  HB2 GLN C 560     121.650  92.229 118.412  1.00  0.00           H
ATOM   5326  HB3 GLN C 560     122.876  93.432 118.113  1.00  0.00           H
ATOM   5327  HG2 GLN C 560     121.398  95.228 118.953  1.00  0.00           H
ATOM   5328  HG3 GLN C 560     120.022  94.147 118.941  1.00  0.00           H
ATOM   5329 HE21 GLN C 560     122.596  92.891 121.876  1.00  0.00           H
ATOM   5330 HE22 GLN C 560     123.066  92.835 120.199  1.00  0.00           H
ATOM   5331  N   GLN C 561     122.522  94.970 115.226  1.00 30.00           N
ATOM   5332  CA  GLN C 561     122.789  96.157 114.410  1.00 30.00           C
ATOM   5333  C   GLN C 561     121.701  96.412 113.349  1.00 30.00           C
ATOM   5334  O   GLN C 561     121.129  97.502 113.309  1.00 30.00           O
ATOM   5335  CB  GLN C 561     124.163  96.003 113.726  1.00 20.00           C
ATOM   5336  CG  GLN C 561     125.361  96.063 114.693  1.00 20.00           C
ATOM   5337  CD  GLN C 561     126.708  95.891 113.983  1.00 20.00           C
ATOM   5338  NE2 GLN C 561     127.774  95.687 114.759  1.00 20.00           N
ATOM   5339  OE1 GLN C 561     126.799  95.950 112.758  1.00 20.00           O
ATOM   5340  H   GLN C 561     123.145  94.190 115.077  1.00  0.00           H
ATOM   5341  HA  GLN C 561     122.821  97.025 115.072  1.00  0.00           H
ATOM   5342  HB2 GLN C 561     124.192  95.059 113.177  1.00  0.00           H
ATOM   5343  HB3 GLN C 561     124.281  96.784 112.974  1.00  0.00           H
ATOM   5344  HG2 GLN C 561     125.365  97.020 115.216  1.00  0.00           H
ATOM   5345  HG3 GLN C 561     125.267  95.287 115.453  1.00  0.00           H
ATOM   5346 HE21 GLN C 561     128.685  95.571 114.341  1.00  0.00           H
ATOM   5347 HE22 GLN C 561     127.681  95.648 115.764  1.00  0.00           H
ATOM   5348  N   MET C 562     121.410  95.377 112.551  1.00 30.00           N
ATOM   5349  CA  MET C 562     120.482  95.450 111.424  1.00 30.00           C
ATOM   5350  C   MET C 562     119.018  95.386 111.871  1.00 30.00           C
ATOM   5351  O   MET C 562     118.187  96.106 111.323  1.00 30.00           O
ATOM   5352  CB  MET C 562     120.764  94.310 110.419  1.00 20.00           C
ATOM   5353  CG  MET C 562     122.199  94.252 109.874  1.00 20.00           C
ATOM   5354  SD  MET C 562     122.710  95.741 108.977  1.00 20.00           S
ATOM   5355  CE  MET C 562     124.377  95.245 108.466  1.00 20.00           C
ATOM   5356  H   MET C 562     121.795  94.471 112.763  1.00  0.00           H
ATOM   5357  HA  MET C 562     120.626  96.405 110.915  1.00  0.00           H
ATOM   5358  HB2 MET C 562     120.084  94.414 109.572  1.00  0.00           H
ATOM   5359  HB3 MET C 562     120.520  93.347 110.871  1.00  0.00           H
ATOM   5360  HG2 MET C 562     122.907  94.070 110.683  1.00  0.00           H
ATOM   5361  HG3 MET C 562     122.288  93.402 109.196  1.00  0.00           H
ATOM   5362  HE1 MET C 562     124.854  96.044 107.900  1.00  0.00           H
ATOM   5363  HE2 MET C 562     124.997  95.025 109.335  1.00  0.00           H
ATOM   5364  HE3 MET C 562     124.335  94.359 107.832  1.00  0.00           H
ATOM   5365  N   GLY C 563     118.711  94.502 112.826  1.00 30.00           N
ATOM   5366  CA  GLY C 563     117.333  94.168 113.170  1.00 30.00           C
ATOM   5367  C   GLY C 563     116.630  95.323 113.882  1.00 30.00           C
ATOM   5368  O   GLY C 563     115.470  95.591 113.578  1.00 30.00           O
ATOM   5369  H   GLY C 563     119.435  93.902 113.214  1.00  0.00           H
ATOM   5370  HA2 GLY C 563     116.769  93.911 112.272  1.00  0.00           H
ATOM   5371  HA3 GLY C 563     117.329  93.282 113.795  1.00  0.00           H
ATOM   5372  N   ILE C 564     117.321  96.047 114.777  1.00 30.00           N
ATOM   5373  CA  ILE C 564     116.727  97.209 115.455  1.00 30.00           C
ATOM   5374  C   ILE C 564     116.455  98.358 114.459  1.00 30.00           C
ATOM   5375  O   ILE C 564     115.412  99.004 114.555  1.00 30.00           O
ATOM   5376  CB  ILE C 564     117.616  97.717 116.633  1.00 20.00           C
ATOM   5377  CG1 ILE C 564     117.741  96.618 117.715  1.00 20.00           C
ATOM   5378  CG2 ILE C 564     117.121  99.042 117.267  1.00 20.00           C
ATOM   5379  CD1 ILE C 564     118.738  96.931 118.840  1.00 20.00           C
ATOM   5380  H   ILE C 564     118.293  95.856 114.977  1.00  0.00           H
ATOM   5381  HA  ILE C 564     115.765  96.899 115.868  1.00  0.00           H
ATOM   5382  HB  ILE C 564     118.617  97.900 116.239  1.00  0.00           H
ATOM   5383 HG12 ILE C 564     116.760  96.427 118.145  1.00  0.00           H
ATOM   5384 HG13 ILE C 564     118.044  95.673 117.262  1.00  0.00           H
```

```
ATOM   5385 HG21 ILE C 564     117.760  99.370 118.085  1.00  0.00           H
ATOM   5386 HG22 ILE C 564     117.113  99.870 116.559  1.00  0.00           H
ATOM   5387 HG23 ILE C 564     116.111  98.933 117.663  1.00  0.00           H
ATOM   5388 HD11 ILE C 564     118.857  96.069 119.495  1.00  0.00           H
ATOM   5389 HD12 ILE C 564     119.719  97.189 118.442  1.00  0.00           H
ATOM   5390 HD13 ILE C 564     118.400  97.757 119.463  1.00  0.00           H
ATOM   5391  N   TYR C 565     117.328  98.503 113.452  1.00 30.00           N
ATOM   5392  CA  TYR C 565     117.178  99.426 112.324  1.00 30.00           C
ATOM   5393  C   TYR C 565     115.984  99.053 111.415  1.00 30.00           C
ATOM   5394  O   TYR C 565     115.199  99.924 111.045  1.00 30.00           O
ATOM   5395  CB  TYR C 565     118.530  99.463 111.576  1.00 20.00           C
ATOM   5396  CG  TYR C 565     118.721 100.495 110.478  1.00 20.00           C
ATOM   5397  CD1 TYR C 565     118.861 101.858 110.812  1.00 20.00           C
ATOM   5398  CD2 TYR C 565     118.844 100.092 109.130  1.00 20.00           C
ATOM   5399  CE1 TYR C 565     119.160 102.804 109.813  1.00 20.00           C
ATOM   5400  CE2 TYR C 565     119.123 101.043 108.129  1.00 20.00           C
ATOM   5401  CZ  TYR C 565     119.293 102.397 108.472  1.00 20.00           C
ATOM   5402  OH  TYR C 565     119.596 103.312 107.510  1.00 20.00           O
ATOM   5403  H   TYR C 565     118.111  97.868 113.405  1.00  0.00           H
ATOM   5404  HA  TYR C 565     116.979 100.420 112.731  1.00  0.00           H
ATOM   5405  HB2 TYR C 565     119.322  99.644 112.304  1.00  0.00           H
ATOM   5406  HB3 TYR C 565     118.754  98.486 111.156  1.00  0.00           H
ATOM   5407  HD1 TYR C 565     118.764 102.180 111.839  1.00  0.00           H
ATOM   5408  HD2 TYR C 565     118.747  99.051 108.856  1.00  0.00           H
ATOM   5409  HE1 TYR C 565     119.296 103.839 110.087  1.00  0.00           H
ATOM   5410  HE2 TYR C 565     119.227 100.726 107.101  1.00  0.00           H
ATOM   5411  HH  TYR C 565     119.591 104.213 107.844  1.00  0.00           H
ATOM   5412  N   ALA C 566     115.813  97.756 111.117  1.00 30.00           N
ATOM   5413  CA  ALA C 566     114.697  97.243 110.317  1.00 30.00           C
ATOM   5414  C   ALA C 566     113.324  97.415 110.994  1.00 30.00           C
ATOM   5415  O   ALA C 566     112.348  97.723 110.307  1.00 30.00           O
ATOM   5416  CB  ALA C 566     114.952  95.774 109.965  1.00 20.00           C
ATOM   5417  H   ALA C 566     116.507  97.080 111.421  1.00  0.00           H
ATOM   5418  HA  ALA C 566     114.670  97.810 109.384  1.00  0.00           H
ATOM   5419  HB1 ALA C 566     114.172  95.383 109.310  1.00  0.00           H
ATOM   5420  HB2 ALA C 566     115.903  95.652 109.446  1.00  0.00           H
ATOM   5421  HB3 ALA C 566     114.980  95.148 110.858  1.00  0.00           H
ATOM   5422  N   VAL C 567     113.273  97.260 112.326  1.00 30.00           N
ATOM   5423  CA  VAL C 567     112.075  97.514 113.132  1.00 30.00           C
ATOM   5424  C   VAL C 567     111.622  98.990 113.059  1.00 30.00           C
ATOM   5425  O   VAL C 567     110.421  99.255 112.998  1.00 30.00           O
ATOM   5426  CB  VAL C 567     112.273  97.082 114.617  1.00 20.00           C
ATOM   5427  CG1 VAL C 567     111.192  97.577 115.599  1.00 20.00           C
ATOM   5428  CG2 VAL C 567     112.348  95.549 114.732  1.00 20.00           C
ATOM   5429  H   VAL C 567     114.104  96.948 112.820  1.00  0.00           H
ATOM   5430  HA  VAL C 567     111.270  96.910 112.709  1.00  0.00           H
ATOM   5431  HB  VAL C 567     113.229  97.481 114.959  1.00  0.00           H
ATOM   5432 HG11 VAL C 567     111.378  97.175 116.591  1.00  0.00           H
ATOM   5433 HG12 VAL C 567     111.169  98.662 115.697  1.00  0.00           H
ATOM   5434 HG13 VAL C 567     110.199  97.252 115.286  1.00  0.00           H
ATOM   5435 HG21 VAL C 567     112.617  95.235 115.741  1.00  0.00           H
ATOM   5436 HG22 VAL C 567     111.386  95.099 114.490  1.00  0.00           H
ATOM   5437 HG23 VAL C 567     113.076  95.114 114.050  1.00  0.00           H
ATOM   5438  N   MET C 568     112.569  99.936 113.012  1.00 30.00           N
ATOM   5439  CA  MET C 568     112.269 101.352 112.771  1.00 30.00           C
ATOM   5440  C   MET C 568     111.613 101.587 111.400  1.00 30.00           C
ATOM   5441  O   MET C 568     110.671 102.369 111.323  1.00 30.00           O
ATOM   5442  CB  MET C 568     113.544 102.206 112.902  1.00 20.00           C
ATOM   5443  CG  MET C 568     114.142 102.239 114.313  1.00 20.00           C
ATOM   5444  SD  MET C 568     115.805 102.950 114.341  1.00 20.00           S
ATOM   5445  CE  MET C 568     116.045 103.141 116.123  1.00 20.00           C
ATOM   5446  H   MET C 568     113.546  99.687 113.087  1.00  0.00           H
ATOM   5447  HA  MET C 568     111.561 101.678 113.536  1.00  0.00           H
ATOM   5448  HB2 MET C 568     114.299 101.868 112.194  1.00  0.00           H
ATOM   5449  HB3 MET C 568     113.317 103.234 112.613  1.00  0.00           H
ATOM   5450  HG2 MET C 568     113.499 102.831 114.963  1.00  0.00           H
ATOM   5451  HG3 MET C 568     114.189 101.243 114.748  1.00  0.00           H
ATOM   5452  HE1 MET C 568     116.962 103.696 116.323  1.00  0.00           H
ATOM   5453  HE2 MET C 568     115.207 103.686 116.558  1.00  0.00           H
ATOM   5454  HE3 MET C 568     116.122 102.166 116.603  1.00  0.00           H
ATOM   5455  N   ILE C 569     112.115 100.927 110.349  1.00 30.00           N
```

```
ATOM   5456  CA  ILE C 569     111.582 101.069 108.993  1.00 30.00           C
ATOM   5457  C   ILE C 569     110.129 100.543 108.912  1.00 30.00           C
ATOM   5458  O   ILE C 569     109.260 101.250 108.404  1.00 30.00           O
ATOM   5459  CB  ILE C 569     112.504 100.383 107.938  1.00 20.00           C
ATOM   5460  CG1 ILE C 569     113.909 101.042 107.925  1.00 20.00           C
ATOM   5461  CG2 ILE C 569     111.927 100.362 106.505  1.00 20.00           C
ATOM   5462  CD1 ILE C 569     115.030 100.135 107.395  1.00 20.00           C
ATOM   5463  H   ILE C 569     112.908 100.310 110.478  1.00  0.00           H
ATOM   5464  HA  ILE C 569     111.548 102.136 108.762  1.00  0.00           H
ATOM   5465  HB  ILE C 569     112.631  99.345 108.246  1.00  0.00           H
ATOM   5466 HG12 ILE C 569     113.879 101.969 107.351  1.00  0.00           H
ATOM   5467 HG13 ILE C 569     114.201 101.346 108.930  1.00  0.00           H
ATOM   5468 HG21 ILE C 569     112.612  99.888 105.802  1.00  0.00           H
ATOM   5469 HG22 ILE C 569     110.989  99.812 106.450  1.00  0.00           H
ATOM   5470 HG23 ILE C 569     111.733 101.373 106.141  1.00  0.00           H
ATOM   5471 HD11 ILE C 569     115.620  99.726 108.216  1.00  0.00           H
ATOM   5472 HD12 ILE C 569     114.651  99.296 106.812  1.00  0.00           H
ATOM   5473 HD13 ILE C 569     115.716 100.701 106.765  1.00  0.00           H
ATOM   5474  N   GLU C 570     109.882  99.376 109.528  1.00  0.00           N
ATOM   5475  CA  GLU C 570     108.560  98.792 109.776  1.00  0.00           C
ATOM   5476  C   GLU C 570     107.587  99.794 110.447  1.00  0.00           C
ATOM   5477  O   GLU C 570     106.545 100.131 109.880  1.00  0.00           O
ATOM   5478  CB  GLU C 570     108.761  97.481 110.587  1.00 20.00           C
ATOM   5479  CG  GLU C 570     107.498  96.830 111.197  1.00 20.00           C
ATOM   5480  CD  GLU C 570     107.763  95.700 112.198  1.00 20.00           C
ATOM   5481  OE1 GLU C 570     108.941  95.355 112.436  1.00 20.00           O
ATOM   5482  OE2 GLU C 570     106.758  95.229 112.771  1.00 20.00           O1-
ATOM   5483  H   GLU C 570     110.670  98.880 109.933  1.00  0.00           H
ATOM   5484  HA  GLU C 570     108.122  98.531 108.810  1.00  0.00           H
ATOM   5485  HB2 GLU C 570     109.229  96.746 109.932  1.00  0.00           H
ATOM   5486  HB3 GLU C 570     109.483  97.656 111.379  1.00  0.00           H
ATOM   5487  HG2 GLU C 570     106.903  97.564 111.728  1.00  0.00           H
ATOM   5488  HG3 GLU C 570     106.866  96.449 110.397  1.00  0.00           H
ATOM   5489  N   LYS C 571     107.947 100.288 111.640  1.00  0.00           N
ATOM   5490  CA  LYS C 571     107.066 101.136 112.450  1.00  0.00           C
ATOM   5491  C   LYS C 571     106.818 102.558 111.890  1.00  0.00           C
ATOM   5492  O   LYS C 571     105.835 103.181 112.294  1.00  0.00           O
ATOM   5493  CB  LYS C 571     107.551 101.148 113.919  1.00 20.00           C
ATOM   5494  CG  LYS C 571     107.542  99.778 114.649  1.00 20.00           C
ATOM   5495  CD  LYS C 571     106.269  98.921 114.494  1.00 20.00           C
ATOM   5496  CE  LYS C 571     106.347  97.593 115.270  1.00 20.00           C
ATOM   5497  NZ  LYS C 571     105.282  96.656 114.874  1.00 20.00           N1+
ATOM   5498  H   LYS C 571     108.833 100.000 112.047  1.00  0.00           H
ATOM   5499  HA  LYS C 571     106.080 100.673 112.433  1.00  0.00           H
ATOM   5500  HB2 LYS C 571     108.558 101.567 113.963  1.00  0.00           H
ATOM   5501  HB3 LYS C 571     106.932 101.840 114.487  1.00  0.00           H
ATOM   5502  HG2 LYS C 571     108.385  99.184 114.308  1.00  0.00           H
ATOM   5503  HG3 LYS C 571     107.728  99.953 115.709  1.00  0.00           H
ATOM   5504  HD2 LYS C 571     105.396  99.486 114.821  1.00  0.00           H
ATOM   5505  HD3 LYS C 571     106.108  98.691 113.443  1.00  0.00           H
ATOM   5506  HE2 LYS C 571     107.302  97.098 115.087  1.00  0.00           H
ATOM   5507  HE3 LYS C 571     106.280  97.778 116.342  1.00  0.00           H
ATOM   5508  HZ1 LYS C 571     105.308  95.845 115.474  1.00  0.00           H
ATOM   5509  HZ2 LYS C 571     104.373  97.091 114.938  1.00  0.00           H
ATOM   5510  HZ3 LYS C 571     105.455  96.348 113.925  1.00  0.00           H
ATOM   5511  N   MET C 572     107.666 103.025 110.965  1.00  0.00           N
ATOM   5512  CA  MET C 572     107.543 104.293 110.237  1.00  0.00           C
ATOM   5513  C   MET C 572     106.713 104.157 108.948  1.00  0.00           C
ATOM   5514  O   MET C 572     106.187 105.165 108.483  1.00  0.00           O
ATOM   5515  CB  MET C 572     108.946 104.838 109.927  1.00 20.00           C
ATOM   5516  CG  MET C 572     109.737 105.228 111.185  1.00 20.00           C
ATOM   5517  SD  MET C 572     109.390 106.870 111.853  1.00 20.00           S
ATOM   5518  CE  MET C 572     110.660 106.867 113.139  1.00 20.00           C
ATOM   5519  H   MET C 572     108.449 102.441 110.704  1.00  0.00           H
ATOM   5520  HA  MET C 572     107.032 105.024 110.867  1.00  0.00           H
ATOM   5521  HB2 MET C 572     109.514 104.105 109.352  1.00  0.00           H
ATOM   5522  HB3 MET C 572     108.861 105.717 109.285  1.00  0.00           H
ATOM   5523  HG2 MET C 572     109.563 104.520 111.995  1.00  0.00           H
ATOM   5524  HG3 MET C 572     110.802 105.170 110.965  1.00  0.00           H
ATOM   5525  HE1 MET C 572     110.705 107.834 113.636  1.00  0.00           H
ATOM   5526  HE2 MET C 572     110.456 106.098 113.882  1.00  0.00           H
```

```
ATOM   5527  HE3 MET C 572     111.631 106.654 112.695  1.00  0.00           H
ATOM   5528  N   ILE C 573     106.574 102.937 108.408  1.00  0.00           N
ATOM   5529  CA  ILE C 573     105.581 102.621 107.378  1.00  0.00           C
ATOM   5530  C   ILE C 573     104.176 102.573 108.019  1.00  0.00           C
ATOM   5531  O   ILE C 573     103.227 103.166 107.505  1.00  0.00           O
ATOM   5532  CB  ILE C 573     105.908 101.279 106.646  1.00 20.00           C
ATOM   5533  CG1 ILE C 573     107.175 101.435 105.772  1.00 20.00           C
ATOM   5534  CG2 ILE C 573     104.759 100.692 105.794  1.00 20.00           C
ATOM   5535  CD1 ILE C 573     107.832 100.105 105.367  1.00 20.00           C
ATOM   5536  H   ILE C 573     107.106 102.155 108.773  1.00  0.00           H
ATOM   5537  HA  ILE C 573     105.578 103.419 106.635  1.00  0.00           H
ATOM   5538  HB  ILE C 573     106.134 100.534 107.409  1.00  0.00           H
ATOM   5539 HG12 ILE C 573     106.928 102.005 104.876  1.00  0.00           H
ATOM   5540 HG13 ILE C 573     107.923 102.031 106.296  1.00  0.00           H
ATOM   5541 HG21 ILE C 573     105.068  99.778 105.288  1.00  0.00           H
ATOM   5542 HG22 ILE C 573     103.893 100.421 106.398  1.00  0.00           H
ATOM   5543 HG23 ILE C 573     104.430 101.398 105.032  1.00  0.00           H
ATOM   5544 HD11 ILE C 573     108.869 100.264 105.070  1.00  0.00           H
ATOM   5545 HD12 ILE C 573     107.832  99.391 106.191  1.00  0.00           H
ATOM   5546 HD13 ILE C 573     107.317  99.645 104.523  1.00  0.00           H
ATOM   5547  N   LEU C 574     104.085 101.942 109.197  1.00  0.00           N
ATOM   5548  CA  LEU C 574     102.852 101.816 109.979  1.00  0.00           C
ATOM   5549  C   LEU C 574     102.350 103.138 110.613  1.00  0.00           C
ATOM   5550  O   LEU C 574     101.292 103.123 111.240  1.00  0.00           O
ATOM   5551  CB  LEU C 574     103.048 100.714 111.048  1.00 20.00           C
ATOM   5552  CG  LEU C 574     103.259  99.286 110.481  1.00 20.00           C
ATOM   5553  CD1 LEU C 574     103.674  98.298 111.592  1.00 20.00           C
ATOM   5554  CD2 LEU C 574     102.034  98.780 109.688  1.00 20.00           C
ATOM   5555  H   LEU C 574     104.901 101.443 109.538  1.00  0.00           H
ATOM   5556  HA  LEU C 574     102.060 101.502 109.297  1.00  0.00           H
ATOM   5557  HB2 LEU C 574     103.895 100.993 111.675  1.00  0.00           H
ATOM   5558  HB3 LEU C 574     102.186 100.691 111.717  1.00  0.00           H
ATOM   5559  HG  LEU C 574     104.093  99.321 109.780  1.00  0.00           H
ATOM   5560 HD11 LEU C 574     104.600  97.788 111.323  1.00  0.00           H
ATOM   5561 HD12 LEU C 574     103.844  98.798 112.544  1.00  0.00           H
ATOM   5562 HD13 LEU C 574     102.928  97.524 111.775  1.00  0.00           H
ATOM   5563 HD21 LEU C 574     101.782  97.743 109.912  1.00  0.00           H
ATOM   5564 HD22 LEU C 574     101.141  99.373 109.888  1.00  0.00           H
ATOM   5565 HD23 LEU C 574     102.224  98.832 108.615  1.00  0.00           H
ATOM   5566  N   ARG C 575     103.086 104.249 110.465  1.00  0.00           N
ATOM   5567  CA  ARG C 575     102.752 105.566 111.018  1.00  0.00           C
ATOM   5568  C   ARG C 575     102.891 106.650 109.936  1.00  0.00           C
ATOM   5569  O   ARG C 575     101.872 107.164 109.470  1.00  0.00           O
ATOM   5570  CB  ARG C 575     103.631 105.873 112.254  1.00 20.00           C
ATOM   5571  CG  ARG C 575     103.312 105.013 113.489  1.00 20.00           C
ATOM   5572  CD  ARG C 575     104.266 105.303 114.658  1.00 20.00           C
ATOM   5573  NE  ARG C 575     103.873 104.600 115.889  1.00 20.00           N
ATOM   5574  CZ  ARG C 575     103.964 103.278 116.120  1.00 20.00           C
ATOM   5575  NH1 ARG C 575     104.446 102.436 115.196  1.00 20.00           N
ATOM   5576  NH2 ARG C 575     103.573 102.792 117.305  1.00 20.00           N1+
ATOM   5577  H   ARG C 575     103.930 104.202 109.911  1.00  0.00           H
ATOM   5578  HA  ARG C 575     101.708 105.581 111.337  1.00  0.00           H
ATOM   5579  HB2 ARG C 575     104.686 105.763 111.997  1.00  0.00           H
ATOM   5580  HB3 ARG C 575     103.500 106.919 112.534  1.00  0.00           H
ATOM   5581  HG2 ARG C 575     102.272 105.124 113.799  1.00  0.00           H
ATOM   5582  HG3 ARG C 575     103.430 103.970 113.203  1.00  0.00           H
ATOM   5583  HD2 ARG C 575     105.272 104.960 114.413  1.00  0.00           H
ATOM   5584  HD3 ARG C 575     104.347 106.377 114.832  1.00  0.00           H
ATOM   5585  HE  ARG C 575     103.474 105.182 116.611  1.00  0.00           H
ATOM   5586 HH11 ARG C 575     104.513 101.447 115.386  1.00  0.00           H
ATOM   5587 HH12 ARG C 575     104.752 102.794 114.300  1.00  0.00           H
ATOM   5588 HH21 ARG C 575     103.654 101.806 117.509  1.00  0.00           H
ATOM   5589 HH22 ARG C 575     103.223 103.410 118.022  1.00  0.00           H
ATOM   5590  N   ASP C 576     104.143 106.984 109.573  1.00 30.00           N
ATOM   5591  CA  ASP C 576     104.539 108.071 108.661  1.00 30.00           C
ATOM   5592  C   ASP C 576     104.412 107.713 107.162  1.00 30.00           C
ATOM   5593  O   ASP C 576     105.207 108.185 106.349  1.00 30.00           O
ATOM   5594  CB  ASP C 576     105.943 108.662 108.981  1.00 20.00           C
ATOM   5595  CG  ASP C 576     106.209 109.078 110.434  1.00 20.00           C
ATOM   5596  OD1 ASP C 576     105.255 109.098 111.242  1.00 20.00           O
ATOM   5597  OD2 ASP C 576     107.375 109.438 110.700  1.00 20.00           O1-
```

```
ATOM   5598  H   ASP C 576     104.917 106.493 109.997  1.00  0.00           H
ATOM   5599  HA  ASP C 576     103.827 108.885 108.815  1.00  0.00           H
ATOM   5600  HB2 ASP C 576     106.703 107.928 108.706  1.00  0.00           H
ATOM   5601  HB3 ASP C 576     106.100 109.550 108.371  1.00  0.00           H
ATOM   5602  N   LEU C 577     103.469 106.846 106.797  1.00  0.00           N
ATOM   5603  CA  LEU C 577     103.079 106.630 105.404  1.00  0.00           C
ATOM   5604  C   LEU C 577     101.557 106.740 105.355  1.00  0.00           C
ATOM   5605  O   LEU C 577     101.052 107.501 104.540  1.00  0.00           O
ATOM   5606  CB  LEU C 577     103.603 105.298 104.804  1.00 20.00           C
ATOM   5607  CG  LEU C 577     104.904 105.409 103.967  1.00 20.00           C
ATOM   5608  CD1 LEU C 577     106.165 105.626 104.829  1.00 20.00           C
ATOM   5609  CD2 LEU C 577     105.060 104.196 103.025  1.00 20.00           C
ATOM   5610  H   LEU C 577     102.894 106.451 107.524  1.00  0.00           H
ATOM   5611  HA  LEU C 577     103.448 107.445 104.778  1.00  0.00           H
ATOM   5612  HB2 LEU C 577     103.712 104.538 105.569  1.00  0.00           H
ATOM   5613  HB3 LEU C 577     102.834 104.914 104.131  1.00  0.00           H
ATOM   5614  HG  LEU C 577     104.810 106.284 103.321  1.00  0.00           H
ATOM   5615 HD11 LEU C 577     106.633 106.583 104.596  1.00  0.00           H
ATOM   5616 HD12 LEU C 577     105.938 105.635 105.893  1.00  0.00           H
ATOM   5617 HD13 LEU C 577     106.923 104.858 104.686  1.00  0.00           H
ATOM   5618 HD21 LEU C 577     104.749 104.465 102.015  1.00  0.00           H
ATOM   5619 HD22 LEU C 577     106.087 103.838 102.952  1.00  0.00           H
ATOM   5620 HD23 LEU C 577     104.447 103.350 103.337  1.00  0.00           H
ATOM   5621  N   CYS C 578     100.853 106.073 106.281  1.00  0.00           N
ATOM   5622  CA  CYS C 578      99.396 106.156 106.444  1.00  0.00           C
ATOM   5623  C   CYS C 578      98.898 107.612 106.617  1.00  0.00           C
ATOM   5624  O   CYS C 578      98.042 108.072 105.861  1.00  0.00           O
ATOM   5625  CB  CYS C 578      98.929 105.227 107.589  1.00 20.00           C
ATOM   5626  SG  CYS C 578      97.125 105.202 107.820  1.00 20.00           S
ATOM   5627  H   CYS C 578     101.339 105.424 106.883  1.00  0.00           H
ATOM   5628  HA  CYS C 578      98.958 105.785 105.515  1.00  0.00           H
ATOM   5629  HB2 CYS C 578      99.252 104.204 107.393  1.00  0.00           H
ATOM   5630  HB3 CYS C 578      99.392 105.523 108.532  1.00  0.00           H
ATOM   5631  HG  CYS C 578      96.843 104.587 106.666  1.00  0.00           H
ATOM   5632  N   ARG C 579      99.500 108.328 107.574  1.00  0.00           N
ATOM   5633  CA  ARG C 579      99.201 109.730 107.862  1.00  0.00           C
ATOM   5634  C   ARG C 579      99.844 110.700 106.854  1.00  0.00           C
ATOM   5635  O   ARG C 579      99.236 111.735 106.582  1.00  0.00           O
ATOM   5636  CB  ARG C 579      99.628 110.043 109.311  1.00 20.00           C
ATOM   5637  CG  ARG C 579      98.794 109.269 110.352  1.00 20.00           C
ATOM   5638  CD  ARG C 579      99.106 109.616 111.816  1.00 20.00           C
ATOM   5639  NE  ARG C 579     100.502 109.343 112.182  1.00 20.00           N
ATOM   5640  CZ  ARG C 579     101.522 110.217 112.164  1.00 20.00           C
ATOM   5641  NH1 ARG C 579     101.346 111.501 111.824  1.00 20.00           N
ATOM   5642  NH2 ARG C 579     102.744 109.789 112.495  1.00 20.00           N1+
ATOM   5643  H   ARG C 579     100.277 107.926 108.081  1.00  0.00           H
ATOM   5644  HA  ARG C 579      98.121 109.874 107.797  1.00  0.00           H
ATOM   5645  HB2 ARG C 579     100.689 109.819 109.436  1.00  0.00           H
ATOM   5646  HB3 ARG C 579      99.515 111.111 109.502  1.00  0.00           H
ATOM   5647  HG2 ARG C 579      97.761 109.572 110.176  1.00  0.00           H
ATOM   5648  HG3 ARG C 579      98.813 108.188 110.204  1.00  0.00           H
ATOM   5649  HD2 ARG C 579      98.734 110.591 112.132  1.00  0.00           H
ATOM   5650  HD3 ARG C 579      98.556 108.891 112.416  1.00  0.00           H
ATOM   5651  HE  ARG C 579     100.709 108.380 112.406  1.00  0.00           H
ATOM   5652 HH11 ARG C 579     102.128 112.141 111.817  1.00  0.00           H
ATOM   5653 HH12 ARG C 579     100.427 111.838 111.576  1.00  0.00           H
ATOM   5654 HH21 ARG C 579     102.906 108.823 112.732  1.00  0.00           H
ATOM   5655 HH22 ARG C 579     103.549 110.396 112.416  1.00  0.00           H
ATOM   5656  N   PHE C 580     101.028 110.363 106.307  1.00  0.00           N
ATOM   5657  CA  PHE C 580     101.725 111.201 105.326  1.00  0.00           C
ATOM   5658  C   PHE C 580     101.085 111.174 103.928  1.00  0.00           C
ATOM   5659  O   PHE C 580     100.833 112.245 103.374  1.00  0.00           O
ATOM   5660  CB  PHE C 580     103.239 110.890 105.292  1.00 20.00           C
ATOM   5661  CG  PHE C 580     104.022 111.614 104.203  1.00 20.00           C
ATOM   5662  CD1 PHE C 580     104.322 112.987 104.338  1.00 20.00           C
ATOM   5663  CD2 PHE C 580     104.291 110.976 102.972  1.00 20.00           C
ATOM   5664  CE1 PHE C 580     104.919 113.677 103.291  1.00 20.00           C
ATOM   5665  CE2 PHE C 580     104.885 111.684 101.935  1.00 20.00           C
ATOM   5666  CZ  PHE C 580     105.202 113.027 102.096  1.00 20.00           C
ATOM   5667  H   PHE C 580     101.473 109.493 106.562  1.00  0.00           H
ATOM   5668  HA  PHE C 580     101.644 112.234 105.672  1.00  0.00           H
```

```
ATOM   5669  HB2 PHE C 580     103.677 111.166 106.253  1.00  0.00           H
ATOM   5670  HB3 PHE C 580     103.397 109.818 105.185  1.00  0.00           H
ATOM   5671  HD1 PHE C 580     104.083 113.507 105.254  1.00  0.00           H
ATOM   5672  HD2 PHE C 580     104.028 109.939 102.829  1.00  0.00           H
ATOM   5673  HE1 PHE C 580     105.157 114.724 103.403  1.00  0.00           H
ATOM   5674  HE2 PHE C 580     105.093 111.191 100.997  1.00  0.00           H
ATOM   5675  HZ  PHE C 580     105.661 113.572 101.285  1.00  0.00           H
ATOM   5676  N   MET C 581     100.820 109.975 103.395  1.00  0.00           N
ATOM   5677  CA  MET C 581     100.325 109.804 102.033  1.00  0.00           C
ATOM   5678  C   MET C 581      98.881 110.283 101.869  1.00  0.00           C
ATOM   5679  O   MET C 581      98.556 110.746 100.780  1.00  0.00           O
ATOM   5680  CB  MET C 581     100.499 108.354 101.538  1.00 20.00           C
ATOM   5681  CG  MET C 581     101.965 107.913 101.366  1.00 20.00           C
ATOM   5682  SD  MET C 581     102.946 108.869 100.170  1.00 20.00           S
ATOM   5683  CE  MET C 581     102.103 108.472  98.614  1.00 20.00           C
ATOM   5684  H   MET C 581     100.991 109.118 103.918  1.00  0.00           H
ATOM   5685  HA  MET C 581     100.928 110.442 101.387  1.00  0.00           H
ATOM   5686  HB2 MET C 581      99.985 107.668 102.211  1.00  0.00           H
ATOM   5687  HB3 MET C 581      99.988 108.237 100.582  1.00  0.00           H
ATOM   5688  HG2 MET C 581     102.481 107.965 102.325  1.00  0.00           H
ATOM   5689  HG3 MET C 581     101.997 106.865 101.069  1.00  0.00           H
ATOM   5690  HE1 MET C 581     102.611 108.956  97.780  1.00  0.00           H
ATOM   5691  HE2 MET C 581     102.107 107.395  98.439  1.00  0.00           H
ATOM   5692  HE3 MET C 581     101.071 108.823  98.625  1.00  0.00           H
ATOM   5693  N   PHE C 582      98.062 110.248 102.936  1.00  0.00           N
ATOM   5694  CA  PHE C 582      96.733 110.864 102.918  1.00  0.00           C
ATOM   5695  C   PHE C 582      96.789 112.370 102.604  1.00  0.00           C
ATOM   5696  O   PHE C 582      96.100 112.823 101.691  1.00  0.00           O
ATOM   5697  CB  PHE C 582      95.952 110.549 104.216  1.00 20.00           C
ATOM   5698  CG  PHE C 582      94.641 111.309 104.368  1.00 20.00           C
ATOM   5699  CD1 PHE C 582      93.515 110.933 103.605  1.00 20.00           C
ATOM   5700  CD2 PHE C 582      94.594 112.513 105.106  1.00 20.00           C
ATOM   5701  CE1 PHE C 582      92.368 111.717 103.622  1.00 20.00           C
ATOM   5702  CE2 PHE C 582      93.438 113.284 105.109  1.00 20.00           C
ATOM   5703  CZ  PHE C 582      92.330 112.886 104.371  1.00 20.00           C
ATOM   5704  H   PHE C 582      98.405 109.926 103.830  1.00  0.00           H
ATOM   5705  HA  PHE C 582      96.177 110.401 102.100  1.00  0.00           H
ATOM   5706  HB2 PHE C 582      95.723 109.482 104.235  1.00  0.00           H
ATOM   5707  HB3 PHE C 582      96.578 110.736 105.090  1.00  0.00           H
ATOM   5708  HD1 PHE C 582      93.547 110.044 102.993  1.00  0.00           H
ATOM   5709  HD2 PHE C 582      95.461 112.849 105.656  1.00  0.00           H
ATOM   5710  HE1 PHE C 582      91.507 111.423 103.040  1.00  0.00           H
ATOM   5711  HE2 PHE C 582      93.405 114.202 105.677  1.00  0.00           H
ATOM   5712  HZ  PHE C 582      91.437 113.494 104.373  1.00  0.00           H
ATOM   5713  N   VAL C 583      97.638 113.094 103.351  1.00  0.00           N
ATOM   5714  CA  VAL C 583      97.845 114.533 103.207  1.00  0.00           C
ATOM   5715  C   VAL C 583      98.522 114.892 101.868  1.00  0.00           C
ATOM   5716  O   VAL C 583      98.067 115.830 101.216  1.00  0.00           O
ATOM   5717  CB  VAL C 583      98.668 115.108 104.396  1.00 20.00           C
ATOM   5718  CG1 VAL C 583      99.052 116.594 104.252  1.00 20.00           C
ATOM   5719  CG2 VAL C 583      97.922 114.911 105.729  1.00 20.00           C
ATOM   5720  H   VAL C 583      98.185 112.637 104.067  1.00  0.00           H
ATOM   5721  HA  VAL C 583      96.861 115.008 103.215  1.00  0.00           H
ATOM   5722  HB  VAL C 583      99.600 114.544 104.468  1.00  0.00           H
ATOM   5723 HG11 VAL C 583      99.546 116.962 105.151  1.00  0.00           H
ATOM   5724 HG12 VAL C 583      99.742 116.758 103.426  1.00  0.00           H
ATOM   5725 HG13 VAL C 583      98.171 117.215 104.081  1.00  0.00           H
ATOM   5726 HG21 VAL C 583      98.508 115.284 106.569  1.00  0.00           H
ATOM   5727 HG22 VAL C 583      96.968 115.438 105.729  1.00  0.00           H
ATOM   5728 HG23 VAL C 583      97.717 113.859 105.926  1.00  0.00           H
ATOM   5729  N   TYR C 584      99.545 114.118 101.455  1.00  0.00           N
ATOM   5730  CA  TYR C 584     100.227 114.286 100.169  1.00  0.00           C
ATOM   5731  C   TYR C 584      99.305 114.061  98.954  1.00  0.00           C
ATOM   5732  O   TYR C 584      99.371 114.855  98.017  1.00  0.00           O
ATOM   5733  CB  TYR C 584     101.508 113.420 100.100  1.00 20.00           C
ATOM   5734  CG  TYR C 584     102.185 113.440  98.736  1.00 20.00           C
ATOM   5735  CD1 TYR C 584     102.774 114.633  98.270  1.00 20.00           C
ATOM   5736  CD2 TYR C 584     102.124 112.314  97.888  1.00 20.00           C
ATOM   5737  CE1 TYR C 584     103.243 114.722  96.946  1.00 20.00           C
ATOM   5738  CE2 TYR C 584     102.609 112.397  96.568  1.00 20.00           C
ATOM   5739  CZ  TYR C 584     103.144 113.609  96.089  1.00 20.00           C
```

```
ATOM   5740  OH  TYR C 584     103.544 113.712  94.789  1.00 20.00           O
ATOM   5741  H   TYR C 584      99.874 113.365 102.046  1.00  0.00           H
ATOM   5742  HA  TYR C 584     100.548 115.329 100.130  1.00  0.00           H
ATOM   5743  HB2 TYR C 584     102.224 113.747 100.856  1.00  0.00           H
ATOM   5744  HB3 TYR C 584     101.263 112.388 100.344  1.00  0.00           H
ATOM   5745  HD1 TYR C 584     102.811 115.500  98.913  1.00  0.00           H
ATOM   5746  HD2 TYR C 584     101.664 111.400  98.232  1.00  0.00           H
ATOM   5747  HE1 TYR C 584     103.650 115.655  96.584  1.00  0.00           H
ATOM   5748  HE2 TYR C 584     102.535 111.541  95.914  1.00  0.00           H
ATOM   5749  HH  TYR C 584     103.756 114.613  94.532  1.00  0.00           H
ATOM   5750  N   LEU C 585      98.461 113.013  98.991  1.00  0.00           N
ATOM   5751  CA  LEU C 585      97.506 112.702  97.921  1.00  0.00           C
ATOM   5752  C   LEU C 585      96.368 113.728  97.799  1.00  0.00           C
ATOM   5753  O   LEU C 585      95.859 113.891  96.693  1.00  0.00           O
ATOM   5754  CB  LEU C 585      96.952 111.269  98.071  1.00 20.00           C
ATOM   5755  CG  LEU C 585      97.967 110.161  97.695  1.00 20.00           C
ATOM   5756  CD1 LEU C 585      97.489 108.776  98.183  1.00 20.00           C
ATOM   5757  CD2 LEU C 585      98.321 110.168  96.191  1.00 20.00           C
ATOM   5758  H   LEU C 585      98.461 112.387  99.786  1.00  0.00           H
ATOM   5759  HA  LEU C 585      98.056 112.748  96.983  1.00  0.00           H
ATOM   5760  HB2 LEU C 585      96.599 111.139  99.094  1.00  0.00           H
ATOM   5761  HB3 LEU C 585      96.068 111.139  97.444  1.00  0.00           H
ATOM   5762  HG  LEU C 585      98.894 110.371  98.228  1.00  0.00           H
ATOM   5763 HD11 LEU C 585      98.251 108.302  98.801  1.00  0.00           H
ATOM   5764 HD12 LEU C 585      96.586 108.845  98.790  1.00  0.00           H
ATOM   5765 HD13 LEU C 585      97.264 108.091  97.365  1.00  0.00           H
ATOM   5766 HD21 LEU C 585      98.203 109.192  95.721  1.00  0.00           H
ATOM   5767 HD22 LEU C 585      97.704 110.867  95.625  1.00  0.00           H
ATOM   5768 HD23 LEU C 585      99.360 110.464  96.047  1.00  0.00           H
ATOM   5769  N   VAL C 586      96.021 114.433  98.892  1.00  0.00           N
ATOM   5770  CA  VAL C 586      95.094 115.568  98.875  1.00  0.00           C
ATOM   5771  C   VAL C 586      95.659 116.777  98.095  1.00  0.00           C
ATOM   5772  O   VAL C 586      94.915 117.370  97.313  1.00  0.00           O
ATOM   5773  CB  VAL C 586      94.656 115.976 100.317  1.00 20.00           C
ATOM   5774  CG1 VAL C 586      94.115 117.412 100.492  1.00 20.00           C
ATOM   5775  CG2 VAL C 586      93.621 114.976 100.865  1.00 20.00           C
ATOM   5776  H   VAL C 586      96.467 114.232  99.777  1.00  0.00           H
ATOM   5777  HA  VAL C 586      94.200 115.242  98.338  1.00  0.00           H
ATOM   5778  HB  VAL C 586      95.527 115.896 100.965  1.00  0.00           H
ATOM   5779 HG11 VAL C 586      93.745 117.569 101.506  1.00  0.00           H
ATOM   5780 HG12 VAL C 586      94.887 118.164 100.325  1.00  0.00           H
ATOM   5781 HG13 VAL C 586      93.291 117.613  99.807  1.00  0.00           H
ATOM   5782 HG21 VAL C 586      93.411 115.161 101.919  1.00  0.00           H
ATOM   5783 HG22 VAL C 586      92.679 115.048 100.321  1.00  0.00           H
ATOM   5784 HG23 VAL C 586      93.967 113.947 100.778  1.00  0.00           H
ATOM   5785  N   PHE C 587      96.960 117.081  98.268  1.00  0.00           N
ATOM   5786  CA  PHE C 587      97.658 118.108  97.487  1.00  0.00           C
ATOM   5787  C   PHE C 587      97.933 117.690  96.033  1.00  0.00           C
ATOM   5788  O   PHE C 587      97.728 118.507  95.139  1.00  0.00           O
ATOM   5789  CB  PHE C 587      98.949 118.577  98.190  1.00 20.00           C
ATOM   5790  CG  PHE C 587      98.713 119.453  99.407  1.00 20.00           C
ATOM   5791  CD1 PHE C 587      98.216 120.765  99.253  1.00 20.00           C
ATOM   5792  CD2 PHE C 587      98.876 118.935 100.708  1.00 20.00           C
ATOM   5793  CE1 PHE C 587      97.925 121.534 100.371  1.00 20.00           C
ATOM   5794  CE2 PHE C 587      98.589 119.722 101.814  1.00 20.00           C
ATOM   5795  CZ  PHE C 587      98.116 121.018 101.647  1.00 20.00           C
ATOM   5796  H   PHE C 587      97.517 116.556  98.928  1.00  0.00           H
ATOM   5797  HA  PHE C 587      96.996 118.974  97.423  1.00  0.00           H
ATOM   5798  HB2 PHE C 587      99.559 117.718  98.474  1.00  0.00           H
ATOM   5799  HB3 PHE C 587      99.558 119.156  97.492  1.00  0.00           H
ATOM   5800  HD1 PHE C 587      98.056 121.169  98.265  1.00  0.00           H
ATOM   5801  HD2 PHE C 587      99.247 117.933 100.845  1.00  0.00           H
ATOM   5802  HE1 PHE C 587      97.544 122.537 100.250  1.00  0.00           H
ATOM   5803  HE2 PHE C 587      98.732 119.326 102.808  1.00  0.00           H
ATOM   5804  HZ  PHE C 587      97.890 121.624 102.511  1.00  0.00           H
ATOM   5805  N   LEU C 588      98.366 116.437  95.810  1.00  0.00           N
ATOM   5806  CA  LEU C 588      98.648 115.888  94.481  1.00  0.00           C
ATOM   5807  C   LEU C 588      97.395 115.836  93.595  1.00  0.00           C
ATOM   5808  O   LEU C 588      97.419 116.426  92.519  1.00  0.00           O
ATOM   5809  CB  LEU C 588      99.370 114.525  94.606  1.00 20.00           C
ATOM   5810  CG  LEU C 588      99.608 113.735  93.291  1.00 20.00           C
```

```
ATOM   5811  CD1 LEU C 588     100.399 114.536  92.234  1.00 20.00           C
ATOM   5812  CD2 LEU C 588     100.243 112.360  93.584  1.00 20.00           C
ATOM   5813  H   LEU C 588      98.535 115.819  96.595  1.00  0.00           H
ATOM   5814  HA  LEU C 588      99.340 116.579  93.998  1.00  0.00           H
ATOM   5815  HB2 LEU C 588     100.326 114.679  95.109  1.00  0.00           H
ATOM   5816  HB3 LEU C 588      98.785 113.893  95.271  1.00  0.00           H
ATOM   5817  HG  LEU C 588      98.632 113.517  92.855  1.00  0.00           H
ATOM   5818 HD11 LEU C 588     101.247 113.986  91.826  1.00  0.00           H
ATOM   5819 HD12 LEU C 588      99.757 114.794  91.391  1.00  0.00           H
ATOM   5820 HD13 LEU C 588     100.794 115.468  92.633  1.00  0.00           H
ATOM   5821 HD21 LEU C 588     101.229 112.247  93.136  1.00  0.00           H
ATOM   5822 HD22 LEU C 588     100.362 112.187  94.653  1.00  0.00           H
ATOM   5823 HD23 LEU C 588      99.618 111.555  93.196  1.00  0.00           H
ATOM   5824  N   PHE C 589      96.329 115.168  94.068  1.00  0.00           N
ATOM   5825  CA  PHE C 589      95.068 115.012  93.337  1.00  0.00           C
ATOM   5826  C   PHE C 589      94.286 116.331  93.183  1.00  0.00           C
ATOM   5827  O   PHE C 589      93.636 116.514  92.154  1.00  0.00           O
ATOM   5828  CB  PHE C 589      94.222 113.906  94.005  1.00 20.00           C
ATOM   5829  CG  PHE C 589      92.956 113.508  93.267  1.00 20.00           C
ATOM   5830  CD1 PHE C 589      93.037 112.661  92.142  1.00 20.00           C
ATOM   5831  CD2 PHE C 589      91.715 114.094  93.597  1.00 20.00           C
ATOM   5832  CE1 PHE C 589      91.892 112.360  91.416  1.00 20.00           C
ATOM   5833  CE2 PHE C 589      90.582 113.784  92.855  1.00 20.00           C
ATOM   5834  CZ  PHE C 589      90.670 112.917  91.773  1.00 20.00           C
ATOM   5835  H   PHE C 589      96.374 114.726  94.977  1.00  0.00           H
ATOM   5836  HA  PHE C 589      95.323 114.664  92.334  1.00  0.00           H
ATOM   5837  HB2 PHE C 589      94.832 113.007  94.107  1.00  0.00           H
ATOM   5838  HB3 PHE C 589      93.949 114.204  95.018  1.00  0.00           H
ATOM   5839  HD1 PHE C 589      93.986 112.238  91.846  1.00  0.00           H
ATOM   5840  HD2 PHE C 589      91.641 114.784  94.424  1.00  0.00           H
ATOM   5841  HE1 PHE C 589      91.954 111.695  90.569  1.00  0.00           H
ATOM   5842  HE2 PHE C 589      89.632 114.226  93.114  1.00  0.00           H
ATOM   5843  HZ  PHE C 589      89.786 112.682  91.199  1.00  0.00           H
ATOM   5844  N   GLY C 590      94.382 117.225  94.185  1.00  0.00           N
ATOM   5845  CA  GLY C 590      93.711 118.525  94.207  1.00  0.00           C
ATOM   5846  C   GLY C 590      94.284 119.461  93.134  1.00  0.00           C
ATOM   5847  O   GLY C 590      93.526 119.969  92.307  1.00  0.00           O
ATOM   5848  H   GLY C 590      94.937 116.993  94.997  1.00  0.00           H
ATOM   5849  HA2 GLY C 590      92.638 118.395  94.061  1.00  0.00           H
ATOM   5850  HA3 GLY C 590      93.850 118.981  95.187  1.00  0.00           H
ATOM   5851  N   PHE C 591      95.617 119.646  93.120  1.00  0.00           N
ATOM   5852  CA  PHE C 591      96.323 120.435  92.107  1.00  0.00           C
ATOM   5853  C   PHE C 591      96.376 119.776  90.713  1.00  0.00           C
ATOM   5854  O   PHE C 591      96.446 120.510  89.727  1.00  0.00           O
ATOM   5855  CB  PHE C 591      97.722 120.849  92.614  1.00 20.00           C
ATOM   5856  CG  PHE C 591      97.736 122.042  93.561  1.00 20.00           C
ATOM   5857  CD1 PHE C 591      97.778 123.348  93.031  1.00 20.00           C
ATOM   5858  CD2 PHE C 591      97.571 121.883  94.955  1.00 20.00           C
ATOM   5859  CE1 PHE C 591      97.750 124.448  93.878  1.00 20.00           C
ATOM   5860  CE2 PHE C 591      97.544 122.996  95.786  1.00 20.00           C
ATOM   5861  CZ  PHE C 591      97.645 124.273  95.251  1.00 20.00           C
ATOM   5862  H   PHE C 591      96.194 119.198  93.820  1.00  0.00           H
ATOM   5863  HA  PHE C 591      95.758 121.358  91.965  1.00  0.00           H
ATOM   5864  HB2 PHE C 591      98.216 120.004  93.093  1.00  0.00           H
ATOM   5865  HB3 PHE C 591      98.361 121.111  91.769  1.00  0.00           H
ATOM   5866  HD1 PHE C 591      97.858 123.496  91.964  1.00  0.00           H
ATOM   5867  HD2 PHE C 591      97.478 120.903  95.391  1.00  0.00           H
ATOM   5868  HE1 PHE C 591      97.809 125.445  93.467  1.00  0.00           H
ATOM   5869  HE2 PHE C 591      97.439 122.871  96.853  1.00  0.00           H
ATOM   5870  HZ  PHE C 591      97.628 125.133  95.904  1.00  0.00           H
ATOM   5871  N   SER C 592      96.307 118.431  90.639  1.00  0.00           N
ATOM   5872  CA  SER C 592      96.223 117.679  89.380  1.00  0.00           C
ATOM   5873  C   SER C 592      94.878 117.885  88.670  1.00  0.00           C
ATOM   5874  O   SER C 592      94.875 118.095  87.461  1.00  0.00           O
ATOM   5875  CB  SER C 592      96.540 116.185  89.598  1.00 20.00           C
ATOM   5876  OG  SER C 592      96.521 115.466  88.382  1.00 20.00           O
ATOM   5877  H   SER C 592      96.291 117.883  91.489  1.00  0.00           H
ATOM   5878  HA  SER C 592      96.994 118.061  88.712  1.00  0.00           H
ATOM   5879  HB2 SER C 592      97.535 116.062  90.023  1.00  0.00           H
ATOM   5880  HB3 SER C 592      95.828 115.731  90.287  1.00  0.00           H
ATOM   5881  HG  SER C 592      95.620 115.436  88.053  1.00  0.00           H
```

```
ATOM   5882  N   THR C 593      93.769 117.867  89.427  1.00 30.00           N
ATOM   5883  CA  THR C 593      92.431 118.160  88.907  1.00 30.00           C
ATOM   5884  C   THR C 593      92.280 119.638  88.481  1.00 30.00           C
ATOM   5885  O   THR C 593      91.621 119.903  87.476  1.00 30.00           O
ATOM   5886  CB  THR C 593      91.329 117.815  89.945  1.00 20.00           C
ATOM   5887  CG2 THR C 593      89.882 118.108  89.504  1.00 20.00           C
ATOM   5888  OG1 THR C 593      91.402 116.436  90.255  1.00 20.00           O
ATOM   5889  H   THR C 593      93.838 117.690  90.421  1.00  0.00           H
ATOM   5890  HA  THR C 593      92.272 117.539  88.022  1.00  0.00           H
ATOM   5891  HB  THR C 593      91.525 118.358  90.872  1.00  0.00           H
ATOM   5892  HG1 THR C 593      92.200 116.279  90.770  1.00  0.00           H
ATOM   5893 HG21 THR C 593      89.167 117.733  90.237  1.00  0.00           H
ATOM   5894 HG22 THR C 593      89.695 119.177  89.398  1.00  0.00           H
ATOM   5895 HG23 THR C 593      89.657 117.632  88.549  1.00  0.00           H
ATOM   5896  N   ALA C 594      92.933 120.560  89.212  1.00 30.00           N
ATOM   5897  CA  ALA C 594      92.937 121.994  88.921  1.00 30.00           C
ATOM   5898  C   ALA C 594      93.650 122.360  87.607  1.00 30.00           C
ATOM   5899  O   ALA C 594      93.141 123.213  86.880  1.00 30.00           O
ATOM   5900  CB  ALA C 594      93.551 122.756  90.104  1.00 30.00           C
ATOM   5901  H   ALA C 594      93.458 120.270  90.026  1.00  0.00           H
ATOM   5902  HA  ALA C 594      91.895 122.308  88.831  1.00  0.00           H
ATOM   5903  HB1 ALA C 594      93.454 123.833  89.965  1.00  0.00           H
ATOM   5904  HB2 ALA C 594      93.050 122.505  91.040  1.00  0.00           H
ATOM   5905  HB3 ALA C 594      94.611 122.533  90.223  1.00  0.00           H
ATOM   5906  N   VAL C 595      94.790 121.708  87.313  1.00 30.00           N
ATOM   5907  CA  VAL C 595      95.570 121.956  86.095  1.00 30.00           C
ATOM   5908  C   VAL C 595      95.021 121.204  84.856  1.00 30.00           C
ATOM   5909  O   VAL C 595      95.243 121.682  83.746  1.00 30.00           O
ATOM   5910  CB  VAL C 595      97.085 121.660  86.312  1.00 20.00           C
ATOM   5911  CG1 VAL C 595      97.449 120.168  86.374  1.00 20.00           C
ATOM   5912  CG2 VAL C 595      97.985 122.372  85.286  1.00 20.00           C
ATOM   5913  H   VAL C 595      95.164 121.026  87.959  1.00  0.00           H
ATOM   5914  HA  VAL C 595      95.489 123.021  85.868  1.00  0.00           H
ATOM   5915  HB  VAL C 595      97.347 122.080  87.285  1.00  0.00           H
ATOM   5916 HG11 VAL C 595      98.502 120.031  86.620  1.00  0.00           H
ATOM   5917 HG12 VAL C 595      96.869 119.664  87.141  1.00  0.00           H
ATOM   5918 HG13 VAL C 595      97.266 119.662  85.427  1.00  0.00           H
ATOM   5919 HG21 VAL C 595      99.040 122.229  85.523  1.00  0.00           H
ATOM   5920 HG22 VAL C 595      97.827 122.000  84.274  1.00  0.00           H
ATOM   5921 HG23 VAL C 595      97.798 123.446  85.280  1.00  0.00           H
ATOM   5922  N   VAL C 596      94.267 120.100  85.052  1.00 30.00           N
ATOM   5923  CA  VAL C 596      93.524 119.390  83.995  1.00 30.00           C
ATOM   5924  C   VAL C 596      92.473 120.280  83.310  1.00 30.00           C
ATOM   5925  O   VAL C 596      92.417 120.295  82.083  1.00 30.00           O
ATOM   5926  CB  VAL C 596      92.806 118.108  84.533  1.00 20.00           C
ATOM   5927  CG1 VAL C 596      91.556 117.608  83.767  1.00 20.00           C
ATOM   5928  CG2 VAL C 596      93.784 116.940  84.639  1.00 20.00           C
ATOM   5929  H   VAL C 596      94.138 119.751  85.992  1.00  0.00           H
ATOM   5930  HA  VAL C 596      94.249 119.092  83.234  1.00  0.00           H
ATOM   5931  HB  VAL C 596      92.476 118.321  85.549  1.00  0.00           H
ATOM   5932 HG11 VAL C 596      91.220 116.644  84.149  1.00  0.00           H
ATOM   5933 HG12 VAL C 596      90.710 118.287  83.869  1.00  0.00           H
ATOM   5934 HG13 VAL C 596      91.764 117.483  82.704  1.00  0.00           H
ATOM   5935 HG21 VAL C 596      93.304 116.117  85.163  1.00  0.00           H
ATOM   5936 HG22 VAL C 596      94.103 116.588  83.657  1.00  0.00           H
ATOM   5937 HG23 VAL C 596      94.669 117.209  85.202  1.00  0.00           H
ATOM   5938  N   THR C 597      91.664 120.981  84.121  1.00 30.00           N
ATOM   5939  CA  THR C 597      90.575 121.842  83.655  1.00 30.00           C
ATOM   5940  C   THR C 597      91.069 123.167  83.035  1.00 30.00           C
ATOM   5941  O   THR C 597      90.336 123.745  82.233  1.00 30.00           O
ATOM   5942  CB  THR C 597      89.586 122.174  84.804  1.00 20.00           C
ATOM   5943  CG2 THR C 597      88.946 120.925  85.432  1.00 20.00           C
ATOM   5944  OG1 THR C 597      90.176 122.963  85.823  1.00 20.00           O
ATOM   5945  H   THR C 597      91.773 120.899  85.122  1.00  0.00           H
ATOM   5946  HA  THR C 597      90.026 121.303  82.880  1.00  0.00           H
ATOM   5947  HB  THR C 597      88.773 122.774  84.392  1.00  0.00           H
ATOM   5948  HG1 THR C 597      90.833 122.438  86.287  1.00  0.00           H
ATOM   5949 HG21 THR C 597      88.248 121.198  86.225  1.00  0.00           H
ATOM   5950 HG22 THR C 597      88.389 120.360  84.685  1.00  0.00           H
ATOM   5951 HG23 THR C 597      89.687 120.254  85.861  1.00  0.00           H
ATOM   5952  N   LEU C 598      92.295 123.598  83.378  1.00 30.00           N
```

```
ATOM   5953  CA  LEU C 598      92.955 124.758  82.780  1.00 30.00           C
ATOM   5954  C   LEU C 598      93.585 124.422  81.414  1.00 30.00           C
ATOM   5955  O   LEU C 598      93.544 125.265  80.518  1.00 30.00           O
ATOM   5956  CB  LEU C 598      93.984 125.309  83.786  1.00 20.00           C
ATOM   5957  CG  LEU C 598      94.673 126.624  83.364  1.00 20.00           C
ATOM   5958  CD1 LEU C 598      93.684 127.803  83.245  1.00 20.00           C
ATOM   5959  CD2 LEU C 598      95.807 126.940  84.340  1.00 20.00           C
ATOM   5960  H   LEU C 598      92.835 123.072  84.050  1.00  0.00           H
ATOM   5961  HA  LEU C 598      92.199 125.525  82.610  1.00  0.00           H
ATOM   5962  HB2 LEU C 598      93.498 125.459  84.751  1.00  0.00           H
ATOM   5963  HB3 LEU C 598      94.748 124.547  83.956  1.00  0.00           H
ATOM   5964  HG  LEU C 598      95.143 126.492  82.389  1.00  0.00           H
ATOM   5965 HD11 LEU C 598      94.035 128.697  83.762  1.00  0.00           H
ATOM   5966 HD12 LEU C 598      93.533 128.075  82.200  1.00  0.00           H
ATOM   5967 HD13 LEU C 598      92.707 127.563  83.662  1.00  0.00           H
ATOM   5968 HD21 LEU C 598      96.125 127.978  84.261  1.00  0.00           H
ATOM   5969 HD22 LEU C 598      95.516 126.750  85.372  1.00  0.00           H
ATOM   5970 HD23 LEU C 598      96.667 126.307  84.125  1.00  0.00           H
ATOM   5971  N   ILE C 599      94.116 123.194  81.270  1.00 30.00           N
ATOM   5972  CA  ILE C 599      94.546 122.622  79.992  1.00 30.00           C
ATOM   5973  C   ILE C 599      93.317 122.339  79.107  1.00 30.00           C
ATOM   5974  O   ILE C 599      92.354 121.733  79.575  1.00 30.00           O
ATOM   5975  CB  ILE C 599      95.389 121.326  80.197  1.00 20.00           C
ATOM   5976  CG1 ILE C 599      96.764 121.672  80.820  1.00 20.00           C
ATOM   5977  CG2 ILE C 599      95.589 120.484  78.917  1.00 20.00           C
ATOM   5978  CD1 ILE C 599      97.469 120.476  81.480  1.00 20.00           C
ATOM   5979  H   ILE C 599      94.125 122.561  82.058  1.00  0.00           H
ATOM   5980  HA  ILE C 599      95.174 123.355  79.486  1.00  0.00           H
ATOM   5981  HB  ILE C 599      94.857 120.698  80.913  1.00  0.00           H
ATOM   5982 HG12 ILE C 599      97.414 122.101  80.056  1.00  0.00           H
ATOM   5983 HG13 ILE C 599      96.655 122.450  81.575  1.00  0.00           H
ATOM   5984 HG21 ILE C 599      96.277 119.656  79.079  1.00  0.00           H
ATOM   5985 HG22 ILE C 599      94.658 120.049  78.559  1.00  0.00           H
ATOM   5986 HG23 ILE C 599      95.989 121.097  78.113  1.00  0.00           H
ATOM   5987 HD11 ILE C 599      98.239 120.813  82.174  1.00  0.00           H
ATOM   5988 HD12 ILE C 599      96.771 119.853  82.040  1.00  0.00           H
ATOM   5989 HD13 ILE C 599      97.956 119.851  80.735  1.00  0.00           H
ATOM   5990  N   GLU C 600      93.364 122.824  77.860  1.00 30.00           N
ATOM   5991  CA  GLU C 600      92.222 122.816  76.945  1.00 30.00           C
ATOM   5992  C   GLU C 600      92.124 121.521  76.125  1.00 30.00           C
ATOM   5993  O   GLU C 600      91.022 120.999  75.962  1.00 30.00           O
ATOM   5994  CB  GLU C 600      92.312 124.062  76.046  1.00 20.00           C
ATOM   5995  CG  GLU C 600      92.130 125.363  76.857  1.00 20.00           C
ATOM   5996  CD  GLU C 600      92.301 126.649  76.051  1.00 20.00           C
ATOM   5997  OE1 GLU C 600      92.800 126.576  74.906  1.00 20.00           O
ATOM   5998  OE2 GLU C 600      91.948 127.701  76.625  1.00 20.00           O1-
ATOM   5999  H   GLU C 600      94.188 123.315  77.542  1.00  0.00           H
ATOM   6000  HA  GLU C 600      91.298 122.896  77.522  1.00  0.00           H
ATOM   6001  HB2 GLU C 600      93.278 124.072  75.537  1.00  0.00           H
ATOM   6002  HB3 GLU C 600      91.556 124.014  75.261  1.00  0.00           H
ATOM   6003  HG2 GLU C 600      91.136 125.373  77.307  1.00  0.00           H
ATOM   6004  HG3 GLU C 600      92.837 125.404  77.685  1.00  0.00           H
ATOM   6005  N   ASP C 601      93.273 121.024  75.646  1.00 30.00           N
ATOM   6006  CA  ASP C 601      93.395 119.832  74.803  1.00 30.00           C
ATOM   6007  C   ASP C 601      94.867 119.380  74.760  1.00 30.00           C
ATOM   6008  O   ASP C 601      95.719 120.012  75.384  1.00 30.00           O
ATOM   6009  CB  ASP C 601      92.758 119.974  73.385  1.00 20.00           C
ATOM   6010  CG  ASP C 601      93.017 121.290  72.635  1.00  0.00           C
ATOM   6011  OD1 ASP C 601      94.057 121.933  72.901  1.00  0.00           O
ATOM   6012  OD2 ASP C 601      92.204 121.578  71.730  1.00  0.00           O1-
ATOM   6013  H   ASP C 601      94.140 121.512  75.825  1.00  0.00           H
ATOM   6014  HA  ASP C 601      92.861 119.031  75.316  1.00  0.00           H
ATOM   6015  HB2 ASP C 601      93.055 119.153  72.733  1.00  0.00           H
ATOM   6016  HB3 ASP C 601      91.678 119.885  73.509  1.00  0.00           H
ATOM   6017  N   GLY C 602      95.142 118.282  74.039  1.00 30.00           N
ATOM   6018  CA  GLY C 602      96.487 117.737  73.863  1.00 30.00           C
ATOM   6019  C   GLY C 602      96.766 116.614  74.872  1.00 30.00           C
ATOM   6020  O   GLY C 602      95.853 116.069  75.493  1.00 30.00           O
ATOM   6021  H   GLY C 602      94.388 117.800  73.571  1.00  0.00           H
ATOM   6022  HA2 GLY C 602      96.562 117.327  72.855  1.00  0.00           H
ATOM   6023  HA3 GLY C 602      97.248 118.516  73.943  1.00  0.00           H
```

```
ATOM   6024  N   LYS C 603      98.056 116.249  74.978  1.00 30.00           N
ATOM   6025  CA  LYS C 603      98.600 115.125  75.751  1.00 30.00           C
ATOM   6026  C   LYS C 603      98.224 115.121  77.246  1.00 30.00           C
ATOM   6027  O   LYS C 603      97.804 114.086  77.763  1.00 30.00           O
ATOM   6028  CB  LYS C 603     100.135 115.105  75.539  1.00 20.00           C
ATOM   6029  CG  LYS C 603     100.914 114.037  76.332  1.00 20.00           C
ATOM   6030  CD  LYS C 603     102.422 114.082  76.046  1.00 20.00           C
ATOM   6031  CE  LYS C 603     103.209 113.059  76.877  1.00 20.00           C
ATOM   6032  NZ  LYS C 603     104.660 113.202  76.670  1.00 20.00           N1+
ATOM   6033  H   LYS C 603      98.737 116.754  74.430  1.00  0.00           H
ATOM   6034  HA  LYS C 603      98.188 114.215  75.312  1.00  0.00           H
ATOM   6035  HB2 LYS C 603     100.339 114.970  74.476  1.00  0.00           H
ATOM   6036  HB3 LYS C 603     100.541 116.084  75.800  1.00  0.00           H
ATOM   6037  HG2 LYS C 603     100.768 114.179  77.403  1.00  0.00           H
ATOM   6038  HG3 LYS C 603     100.522 113.047  76.095  1.00  0.00           H
ATOM   6039  HD2 LYS C 603     102.595 113.900  74.984  1.00  0.00           H
ATOM   6040  HD3 LYS C 603     102.798 115.086  76.248  1.00  0.00           H
ATOM   6041  HE2 LYS C 603     103.000 113.192  77.939  1.00  0.00           H
ATOM   6042  HE3 LYS C 603     102.908 112.044  76.614  1.00  0.00           H
ATOM   6043  HZ1 LYS C 603     104.950 114.124  76.966  1.00  0.00           H
ATOM   6044  HZ2 LYS C 603     105.149 112.508  77.217  1.00  0.00           H
ATOM   6045  HZ3 LYS C 603     104.879 113.075  75.692  1.00  0.00           H
ATOM   6046  N   TYR C 627      98.404 116.277  77.903  1.00 30.00           N
ATOM   6047  CA  TYR C 627      98.275 116.445  79.352  1.00 30.00           C
ATOM   6048  C   TYR C 627      96.850 116.792  79.825  1.00 30.00           C
ATOM   6049  O   TYR C 627      96.670 117.063  81.012  1.00 30.00           O
ATOM   6050  CB  TYR C 627      99.326 117.475  79.809  1.00 20.00           C
ATOM   6051  CG  TYR C 627     100.753 117.000  79.599  1.00 20.00           C
ATOM   6052  CD1 TYR C 627     101.263 115.967  80.410  1.00 20.00           C
ATOM   6053  CD2 TYR C 627     101.562 117.557  78.586  1.00 20.00           C
ATOM   6054  CE1 TYR C 627     102.571 115.492  80.211  1.00 20.00           C
ATOM   6055  CE2 TYR C 627     102.880 117.095  78.401  1.00 20.00           C
ATOM   6056  CZ  TYR C 627     103.389 116.066  79.220  1.00 20.00           C
ATOM   6057  OH  TYR C 627     104.672 115.629  79.066  1.00 20.00           O
ATOM   6058  H   TYR C 627      98.737 117.083  77.394  1.00  0.00           H
ATOM   6059  HA  TYR C 627      98.515 115.496  79.835  1.00  0.00           H
ATOM   6060  HB2 TYR C 627      99.177 118.424  79.291  1.00  0.00           H
ATOM   6061  HB3 TYR C 627      99.213 117.691  80.872  1.00  0.00           H
ATOM   6062  HD1 TYR C 627     100.650 115.535  81.186  1.00  0.00           H
ATOM   6063  HD2 TYR C 627     101.177 118.342  77.952  1.00  0.00           H
ATOM   6064  HE1 TYR C 627     102.948 114.696  80.836  1.00  0.00           H
ATOM   6065  HE2 TYR C 627     103.503 117.537  77.637  1.00  0.00           H
ATOM   6066  HH  TYR C 627     104.990 115.168  79.851  1.00  0.00           H
ATOM   6067  N   ASN C 628      95.864 116.747  78.912  1.00 30.00           N
ATOM   6068  CA  ASN C 628      94.444 116.968  79.198  1.00 30.00           C
ATOM   6069  C   ASN C 628      93.796 115.816  79.999  1.00 30.00           C
ATOM   6070  O   ASN C 628      92.809 116.065  80.690  1.00 30.00           O
ATOM   6071  CB  ASN C 628      93.716 117.231  77.858  1.00 20.00           C
ATOM   6072  CG  ASN C 628      92.295 117.798  77.972  1.00 20.00           C
ATOM   6073  ND2 ASN C 628      92.088 118.831  78.790  1.00 20.00           N
ATOM   6074  OD1 ASN C 628      91.387 117.313  77.302  1.00 20.00           O
ATOM   6075  H   ASN C 628      96.090 116.512  77.955  1.00  0.00           H
ATOM   6076  HA  ASN C 628      94.356 117.853  79.832  1.00  0.00           H
ATOM   6077  HB2 ASN C 628      94.288 117.958  77.280  1.00  0.00           H
ATOM   6078  HB3 ASN C 628      93.695 116.321  77.256  1.00  0.00           H
ATOM   6079 HD21 ASN C 628      91.167 119.238  78.862  1.00  0.00           H
ATOM   6080 HD22 ASN C 628      92.839 119.234  79.332  1.00  0.00           H
ATOM   6081  N   SER C 629      94.369 114.598  79.935  1.00 30.00           N
ATOM   6082  CA  SER C 629      93.974 113.472  80.786  1.00 30.00           C
ATOM   6083  C   SER C 629      94.549 113.625  82.209  1.00 30.00           C
ATOM   6084  O   SER C 629      95.646 114.163  82.373  1.00 30.00           O
ATOM   6085  CB  SER C 629      94.362 112.136  80.110  1.00 20.00           C
ATOM   6086  OG  SER C 629      95.714 111.764  80.300  1.00 20.00           O
ATOM   6087  H   SER C 629      95.185 114.461  79.357  1.00  0.00           H
ATOM   6088  HA  SER C 629      92.884 113.483  80.856  1.00  0.00           H
ATOM   6089  HB2 SER C 629      94.144 112.164  79.042  1.00  0.00           H
ATOM   6090  HB3 SER C 629      93.751 111.333  80.524  1.00  0.00           H
ATOM   6091  HG  SER C 629      96.269 112.286  79.714  1.00  0.00           H
ATOM   6092  N   LEU C 630      93.790 113.132  83.204  1.00 30.00           N
ATOM   6093  CA  LEU C 630      94.115 113.214  84.630  1.00 30.00           C
ATOM   6094  C   LEU C 630      95.414 112.487  85.014  1.00 30.00           C
```

```
ATOM   6095  O   LEU C 630      96.206 113.053  85.764  1.00 30.00           O
ATOM   6096  CB  LEU C 630      92.889 112.743  85.450  1.00 20.00           C
ATOM   6097  CG  LEU C 630      93.004 112.837  86.991  1.00 20.00           C
ATOM   6098  CD1 LEU C 630      93.193 114.285  87.479  1.00 20.00           C
ATOM   6099  CD2 LEU C 630      91.784 112.181  87.669  1.00 20.00           C
ATOM   6100  H   LEU C 630      92.903 112.704  82.981  1.00  0.00           H
ATOM   6101  HA  LEU C 630      94.294 114.262  84.847  1.00  0.00           H
ATOM   6102  HB2 LEU C 630      92.015 113.314  85.133  1.00  0.00           H
ATOM   6103  HB3 LEU C 630      92.679 111.706  85.181  1.00  0.00           H
ATOM   6104  HG  LEU C 630      93.878 112.270  87.314  1.00  0.00           H
ATOM   6105 HD11 LEU C 630      93.100 114.359  88.563  1.00  0.00           H
ATOM   6106 HD12 LEU C 630      94.179 114.663  87.214  1.00  0.00           H
ATOM   6107 HD13 LEU C 630      92.451 114.953  87.040  1.00  0.00           H
ATOM   6108 HD21 LEU C 630      91.268 112.866  88.344  1.00  0.00           H
ATOM   6109 HD22 LEU C 630      91.046 111.836  86.944  1.00  0.00           H
ATOM   6110 HD23 LEU C 630      92.087 111.313  88.254  1.00  0.00           H
ATOM   6111  N   TYR C 631      95.620 111.278  84.467  1.00 30.00           N
ATOM   6112  CA  TYR C 631      96.806 110.446  84.687  1.00 30.00           C
ATOM   6113  C   TYR C 631      98.117 111.081  84.184  1.00 30.00           C
ATOM   6114  O   TYR C 631      99.121 111.007  84.895  1.00 30.00           O
ATOM   6115  CB  TYR C 631      96.547 109.050  84.078  1.00 20.00           C
ATOM   6116  CG  TYR C 631      97.750 108.123  83.973  1.00 20.00           C
ATOM   6117  CD1 TYR C 631      98.308 107.546  85.133  1.00 20.00           C
ATOM   6118  CD2 TYR C 631      98.319 107.846  82.712  1.00 20.00           C
ATOM   6119  CE1 TYR C 631      99.422 106.688  85.031  1.00 20.00           C
ATOM   6120  CE2 TYR C 631      99.428 106.987  82.609  1.00 20.00           C
ATOM   6121  CZ  TYR C 631      99.977 106.403  83.767  1.00 20.00           C
ATOM   6122  OH  TYR C 631     101.044 105.564  83.656  1.00 20.00           O
ATOM   6123  H   TYR C 631      94.923 110.898  83.843  1.00  0.00           H
ATOM   6124  HA  TYR C 631      96.905 110.319  85.767  1.00  0.00           H
ATOM   6125  HB2 TYR C 631      95.769 108.540  84.647  1.00  0.00           H
ATOM   6126  HB3 TYR C 631      96.141 109.172  83.072  1.00  0.00           H
ATOM   6127  HD1 TYR C 631      97.884 107.760  86.103  1.00  0.00           H
ATOM   6128  HD2 TYR C 631      97.907 108.291  81.817  1.00  0.00           H
ATOM   6129  HE1 TYR C 631      99.843 106.248  85.923  1.00  0.00           H
ATOM   6130  HE2 TYR C 631      99.856 106.773  81.641  1.00  0.00           H
ATOM   6131  HH  TYR C 631     101.365 105.245  84.503  1.00  0.00           H
ATOM   6132  N   SER C 632      98.082 111.687  82.983  1.00 30.00           N
ATOM   6133  CA  SER C 632      99.254 112.257  82.315  1.00 30.00           C
ATOM   6134  C   SER C 632      99.895 113.433  83.069  1.00 30.00           C
ATOM   6135  O   SER C 632     101.122 113.495  83.116  1.00 30.00           O
ATOM   6136  CB  SER C 632      98.898 112.668  80.874  1.00 20.00           C
ATOM   6137  OG  SER C 632      98.828 111.530  80.040  1.00 20.00           O
ATOM   6138  H   SER C 632      97.218 111.712  82.459  1.00  0.00           H
ATOM   6139  HA  SER C 632     100.015 111.474  82.268  1.00  0.00           H
ATOM   6140  HB2 SER C 632      97.960 113.224  80.837  1.00  0.00           H
ATOM   6141  HB3 SER C 632      99.665 113.321  80.455  1.00  0.00           H
ATOM   6142  HG  SER C 632      99.715 111.184  79.917  1.00  0.00           H
ATOM   6143  N   THR C 633      99.071 114.321  83.651  1.00 30.00           N
ATOM   6144  CA  THR C 633      99.543 115.479  84.416  1.00 30.00           C
ATOM   6145  C   THR C 633      99.637 115.209  85.938  1.00 30.00           C
ATOM   6146  O   THR C 633     100.316 115.969  86.628  1.00 30.00           O
ATOM   6147  CB  THR C 633      98.680 116.735  84.127  1.00 20.00           C
ATOM   6148  CG2 THR C 633      97.231 116.655  84.624  1.00 20.00           C
ATOM   6149  OG1 THR C 633      99.254 117.888  84.712  1.00 20.00           O
ATOM   6150  H   THR C 633      98.069 114.208  83.573  1.00  0.00           H
ATOM   6151  HA  THR C 633     100.553 115.724  84.085  1.00  0.00           H
ATOM   6152  HB  THR C 633      98.664 116.899  83.050  1.00  0.00           H
ATOM   6153  HG1 THR C 633      99.291 117.760  85.664  1.00  0.00           H
ATOM   6154 HG21 THR C 633      96.681 117.534  84.287  1.00  0.00           H
ATOM   6155 HG22 THR C 633      96.719 115.778  84.227  1.00  0.00           H
ATOM   6156 HG23 THR C 633      97.164 116.618  85.712  1.00  0.00           H
ATOM   6157  N   CYS C 634      99.036 114.107  86.430  1.00 30.00           N
ATOM   6158  CA  CYS C 634      99.265 113.583  87.783  1.00 30.00           C
ATOM   6159  C   CYS C 634     100.709 113.063  87.937  1.00 30.00           C
ATOM   6160  O   CYS C 634     101.309 113.265  88.992  1.00 30.00           O
ATOM   6161  CB  CYS C 634      98.224 112.513  88.183  1.00 20.00           C
ATOM   6162  SG  CYS C 634      98.387 111.980  89.915  1.00 20.00           S
ATOM   6163  H   CYS C 634      98.465 113.535  85.823  1.00  0.00           H
ATOM   6164  HA  CYS C 634      99.150 114.420  88.475  1.00  0.00           H
ATOM   6165  HB2 CYS C 634      97.216 112.909  88.073  1.00  0.00           H
```

```
ATOM   6166  HB3 CYS C 634      98.294 111.640  87.532  1.00  0.00           H
ATOM   6167  HG  CYS C 634      99.578 111.392  89.771  1.00  0.00           H
ATOM   6168  N   LEU C 635     101.255 112.470  86.858  1.00 30.00           N
ATOM   6169  CA  LEU C 635     102.666 112.107  86.734  1.00 30.00           C
ATOM   6170  C   LEU C 635     103.596 113.326  86.745  1.00 30.00           C
ATOM   6171  O   LEU C 635     104.612 113.270  87.431  1.00 30.00           O
ATOM   6172  CB  LEU C 635     102.903 111.289  85.445  1.00 20.00           C
ATOM   6173  CG  LEU C 635     102.528 109.799  85.538  1.00 20.00           C
ATOM   6174  CD1 LEU C 635     102.473 109.159  84.134  1.00 20.00           C
ATOM   6175  CD2 LEU C 635     103.471 109.037  86.494  1.00 20.00           C
ATOM   6176  H   LEU C 635     100.687 112.324  86.034  1.00  0.00           H
ATOM   6177  HA  LEU C 635     102.917 111.509  87.610  1.00  0.00           H
ATOM   6178  HB2 LEU C 635     102.349 111.757  84.632  1.00  0.00           H
ATOM   6179  HB3 LEU C 635     103.953 111.344  85.149  1.00  0.00           H
ATOM   6180  HG  LEU C 635     101.522 109.743  85.956  1.00  0.00           H
ATOM   6181 HD11 LEU C 635     103.097 108.270  84.046  1.00  0.00           H
ATOM   6182 HD12 LEU C 635     101.453 108.862  83.892  1.00  0.00           H
ATOM   6183 HD13 LEU C 635     102.796 109.848  83.353  1.00  0.00           H
ATOM   6184 HD21 LEU C 635     102.918 108.322  87.104  1.00  0.00           H
ATOM   6185 HD22 LEU C 635     104.245 108.495  85.955  1.00  0.00           H
ATOM   6186 HD23 LEU C 635     104.003 109.695  87.179  1.00  0.00           H
ATOM   6187  N   GLU C 636     103.235 114.399  86.018  1.00 30.00           N
ATOM   6188  CA  GLU C 636     104.012 115.642  85.954  1.00 30.00           C
ATOM   6189  C   GLU C 636     104.079 116.397  87.291  1.00 30.00           C
ATOM   6190  O   GLU C 636     105.104 117.023  87.556  1.00 30.00           O
ATOM   6191  CB  GLU C 636     103.480 116.553  84.829  1.00 20.00           C
ATOM   6192  CG  GLU C 636     103.638 115.969  83.409  1.00 20.00           C
ATOM   6193  CD  GLU C 636     105.085 115.812  82.924  1.00 20.00           C
ATOM   6194  OE1 GLU C 636     105.952 116.576  83.401  1.00 20.00           O
ATOM   6195  OE2 GLU C 636     105.298 114.928  82.064  1.00 20.00           O1-
ATOM   6196  H   GLU C 636     102.387 114.373  85.471  1.00  0.00           H
ATOM   6197  HA  GLU C 636     105.039 115.367  85.716  1.00  0.00           H
ATOM   6198  HB2 GLU C 636     102.426 116.768  85.005  1.00  0.00           H
ATOM   6199  HB3 GLU C 636     103.973 117.527  84.871  1.00  0.00           H
ATOM   6200  HG2 GLU C 636     103.149 114.999  83.351  1.00  0.00           H
ATOM   6201  HG3 GLU C 636     103.118 116.611  82.704  1.00  0.00           H
ATOM   6202  N   LEU C 637     103.031 116.280  88.127  1.00 30.00           N
ATOM   6203  CA  LEU C 637     103.034 116.793  89.496  1.00 30.00           C
ATOM   6204  C   LEU C 637     103.777 115.871  90.481  1.00 30.00           C
ATOM   6205  O   LEU C 637     104.397 116.384  91.412  1.00 30.00           O
ATOM   6206  CB  LEU C 637     101.605 117.141  89.956  1.00 20.00           C
ATOM   6207  CG  LEU C 637     100.995 118.375  89.249  1.00 20.00           C
ATOM   6208  CD1 LEU C 637      99.600 118.676  89.808  1.00 20.00           C
ATOM   6209  CD2 LEU C 637     101.875 119.643  89.296  1.00 20.00           C
ATOM   6210  H   LEU C 637     102.208 115.771  87.833  1.00  0.00           H
ATOM   6211  HA  LEU C 637     103.590 117.721  89.495  1.00  0.00           H
ATOM   6212  HB2 LEU C 637     100.958 116.274  89.814  1.00  0.00           H
ATOM   6213  HB3 LEU C 637     101.612 117.337  91.029  1.00  0.00           H
ATOM   6214  HG  LEU C 637     100.864 118.113  88.198  1.00  0.00           H
ATOM   6215 HD11 LEU C 637      98.953 119.061  89.022  1.00  0.00           H
ATOM   6216 HD12 LEU C 637      99.132 117.788  90.232  1.00  0.00           H
ATOM   6217 HD13 LEU C 637      99.635 119.425  90.600  1.00  0.00           H
ATOM   6218 HD21 LEU C 637     101.286 120.548  89.448  1.00  0.00           H
ATOM   6219 HD22 LEU C 637     102.610 119.608  90.098  1.00  0.00           H
ATOM   6220 HD23 LEU C 637     102.417 119.769  88.358  1.00  0.00           H
ATOM   6221  N   PHE C 638     103.772 114.547  90.246  1.00 30.00           N
ATOM   6222  CA  PHE C 638     104.561 113.581  91.020  1.00 30.00           C
ATOM   6223  C   PHE C 638     106.079 113.681  90.740  1.00 30.00           C
ATOM   6224  O   PHE C 638     106.870 113.346  91.622  1.00 30.00           O
ATOM   6225  CB  PHE C 638     103.997 112.159  90.800  1.00 20.00           C
ATOM   6226  CG  PHE C 638     104.621 111.061  91.650  1.00 20.00           C
ATOM   6227  CD1 PHE C 638     104.198 110.875  92.984  1.00 20.00           C
ATOM   6228  CD2 PHE C 638     105.735 110.333  91.185  1.00 20.00           C
ATOM   6229  CE1 PHE C 638     104.833 109.943  93.794  1.00 20.00           C
ATOM   6230  CE2 PHE C 638     106.359 109.404  92.009  1.00 20.00           C
ATOM   6231  CZ  PHE C 638     105.904 109.205  93.306  1.00 20.00           C
ATOM   6232  H   PHE C 638     103.237 114.176  89.472  1.00  0.00           H
ATOM   6233  HA  PHE C 638     104.421 113.819  92.076  1.00  0.00           H
ATOM   6234  HB2 PHE C 638     102.929 112.167  91.021  1.00  0.00           H
ATOM   6235  HB3 PHE C 638     104.080 111.882  89.748  1.00  0.00           H
ATOM   6236  HD1 PHE C 638     103.380 111.455  93.380  1.00  0.00           H
```

```
ATOM   6237  HD2 PHE C 638     106.113 110.506  90.191  1.00  0.00           H
ATOM   6238  HE1 PHE C 638     104.496 109.798  94.809  1.00  0.00           H
ATOM   6239  HE2 PHE C 638     107.205 108.842  91.644  1.00  0.00           H
ATOM   6240  HZ  PHE C 638     106.392 108.482  93.943  1.00  0.00           H
ATOM   6241  N   LYS C 639     106.465 114.178  89.550  1.00 30.00           N
ATOM   6242  CA  LYS C 639     107.853 114.453  89.172  1.00 30.00           C
ATOM   6243  C   LYS C 639     108.518 115.558  90.014  1.00 30.00           C
ATOM   6244  O   LYS C 639     109.719 115.446  90.244  1.00 30.00           O
ATOM   6245  CB  LYS C 639     107.953 114.779  87.667  1.00 20.00           C
ATOM   6246  CG  LYS C 639     107.888 113.561  86.731  1.00 20.00           C
ATOM   6247  CD  LYS C 639     107.895 113.988  85.252  1.00 20.00           C
ATOM   6248  CE  LYS C 639     107.620 112.846  84.263  1.00 20.00           C
ATOM   6249  NZ  LYS C 639     108.708 111.854  84.256  1.00 20.00           N1+
ATOM   6250  H   LYS C 639     105.762 114.405  88.859  1.00  0.00           H
ATOM   6251  HA  LYS C 639     108.418 113.536  89.352  1.00  0.00           H
ATOM   6252  HB2 LYS C 639     107.197 115.514  87.396  1.00  0.00           H
ATOM   6253  HB3 LYS C 639     108.912 115.255  87.468  1.00  0.00           H
ATOM   6254  HG2 LYS C 639     108.741 112.910  86.930  1.00  0.00           H
ATOM   6255  HG3 LYS C 639     107.005 112.965  86.944  1.00  0.00           H
ATOM   6256  HD2 LYS C 639     107.155 114.771  85.096  1.00  0.00           H
ATOM   6257  HD3 LYS C 639     108.856 114.447  85.015  1.00  0.00           H
ATOM   6258  HE2 LYS C 639     106.678 112.352  84.502  1.00  0.00           H
ATOM   6259  HE3 LYS C 639     107.519 113.250  83.255  1.00  0.00           H
ATOM   6260  HZ1 LYS C 639     108.512 111.128  83.580  1.00  0.00           H
ATOM   6261  HZ2 LYS C 639     109.577 112.301  83.999  1.00  0.00           H
ATOM   6262  HZ3 LYS C 639     108.799 111.447  85.175  1.00  0.00           H
ATOM   6263  N   PHE C 640     107.763 116.572  90.486  1.00 30.00           N
ATOM   6264  CA  PHE C 640     108.282 117.617  91.387  1.00 30.00           C
ATOM   6265  C   PHE C 640     108.778 117.077  92.736  1.00 30.00           C
ATOM   6266  O   PHE C 640     109.794 117.564  93.228  1.00 30.00           O
ATOM   6267  CB  PHE C 640     107.248 118.732  91.635  1.00 20.00           C
ATOM   6268  CG  PHE C 640     107.025 119.690  90.482  1.00 20.00           C
ATOM   6269  CD1 PHE C 640     107.886 120.788  90.279  1.00 20.00           C
ATOM   6270  CD2 PHE C 640     106.042 119.409  89.518  1.00 20.00           C
ATOM   6271  CE1 PHE C 640     107.684 121.631  89.194  1.00 20.00           C
ATOM   6272  CE2 PHE C 640     105.849 120.261  88.441  1.00 20.00           C
ATOM   6273  CZ  PHE C 640     106.657 121.379  88.292  1.00 20.00           C
ATOM   6274  H   PHE C 640     106.779 116.613  90.264  1.00  0.00           H
ATOM   6275  HA  PHE C 640     109.150 118.068  90.902  1.00  0.00           H
ATOM   6276  HB2 PHE C 640     106.293 118.287  91.911  1.00  0.00           H
ATOM   6277  HB3 PHE C 640     107.547 119.335  92.494  1.00  0.00           H
ATOM   6278  HD1 PHE C 640     108.689 120.988  90.973  1.00  0.00           H
ATOM   6279  HD2 PHE C 640     105.444 118.527  89.633  1.00  0.00           H
ATOM   6280  HE1 PHE C 640     108.330 122.484  89.051  1.00  0.00           H
ATOM   6281  HE2 PHE C 640     105.077 120.050  87.716  1.00  0.00           H
ATOM   6282  HZ  PHE C 640     106.498 122.045  87.459  1.00  0.00           H
ATOM   6283  N   THR C 641     108.079 116.063  93.275  1.00 30.00           N
ATOM   6284  CA  THR C 641     108.453 115.344  94.493  1.00 30.00           C
ATOM   6285  C   THR C 641     109.777 114.564  94.341  1.00 30.00           C
ATOM   6286  O   THR C 641     110.533 114.512  95.308  1.00 30.00           O
ATOM   6287  CB  THR C 641     107.329 114.367  94.933  1.00 20.00           C
ATOM   6288  CG2 THR C 641     107.616 113.472  96.153  1.00 20.00           C
ATOM   6289  OG1 THR C 641     106.168 115.121  95.216  1.00 20.00           O
ATOM   6290  H   THR C 641     107.249 115.727  92.806  1.00  0.00           H
ATOM   6291  HA  THR C 641     108.590 116.084  95.283  1.00  0.00           H
ATOM   6292  HB  THR C 641     107.080 113.709  94.102  1.00  0.00           H
ATOM   6293  HG1 THR C 641     105.533 114.540  95.644  1.00  0.00           H
ATOM   6294 HG21 THR C 641     106.738 112.883  96.420  1.00  0.00           H
ATOM   6295 HG22 THR C 641     108.422 112.763  95.958  1.00  0.00           H
ATOM   6296 HG23 THR C 641     107.893 114.063  97.025  1.00  0.00           H
ATOM   6297  N   ILE C 642     110.049 114.033  93.132  1.00 30.00           N
ATOM   6298  CA  ILE C 642     111.308 113.372  92.762  1.00 30.00           C
ATOM   6299  C   ILE C 642     112.459 114.380  92.526  1.00 30.00           C
ATOM   6300  O   ILE C 642     113.604 114.057  92.835  1.00 30.00           O
ATOM   6301  CB  ILE C 642     111.140 112.475  91.491  1.00 20.00           C
ATOM   6302  CG1 ILE C 642     110.141 111.321  91.749  1.00 20.00           C
ATOM   6303  CG2 ILE C 642     112.462 111.899  90.919  1.00 20.00           C
ATOM   6304  CD1 ILE C 642     109.512 110.738  90.472  1.00 20.00           C
ATOM   6305  H   ILE C 642     109.370 114.130  92.390  1.00  0.00           H
ATOM   6306  HA  ILE C 642     111.598 112.719  93.585  1.00  0.00           H
ATOM   6307  HB  ILE C 642     110.706 113.097  90.709  1.00  0.00           H
```

```
ATOM   6308 HG12 ILE C 642     110.632 110.529  92.315  1.00  0.00           H
ATOM   6309 HG13 ILE C 642     109.321 111.662  92.381  1.00  0.00           H
ATOM   6310 HG21 ILE C 642     112.281 111.208  90.096  1.00  0.00           H
ATOM   6311 HG22 ILE C 642     113.115 112.675  90.520  1.00  0.00           H
ATOM   6312 HG23 ILE C 642     113.017 111.353  91.683  1.00  0.00           H
ATOM   6313 HD11 ILE C 642     108.501 111.120  90.334  1.00  0.00           H
ATOM   6314 HD12 ILE C 642     110.075 110.985  89.572  1.00  0.00           H
ATOM   6315 HD13 ILE C 642     109.446 109.651  90.533  1.00  0.00           H
ATOM   6316  N   GLY C 643     112.135 115.575  92.003  1.00 30.00           N
ATOM   6317  CA  GLY C 643     113.102 116.609  91.624  1.00 30.00           C
ATOM   6318  C   GLY C 643     113.278 116.699  90.096  1.00 30.00           C
ATOM   6319  O   GLY C 643     114.195 117.382  89.644  1.00 30.00           O
ATOM   6320  H   GLY C 643     111.163 115.772  91.807  1.00  0.00           H
ATOM   6321  HA2 GLY C 643     112.735 117.569  91.988  1.00  0.00           H
ATOM   6322  HA3 GLY C 643     114.075 116.449  92.091  1.00  0.00           H
ATOM   6323  N   MET C 644     112.413 116.033  89.309  1.00 30.00           N
ATOM   6324  CA  MET C 644     112.386 116.039  87.841  1.00 30.00           C
ATOM   6325  C   MET C 644     111.213 116.855  87.259  1.00 30.00           C
ATOM   6326  O   MET C 644     110.986 116.784  86.050  1.00 30.00           O
ATOM   6327  CB  MET C 644     112.346 114.572  87.343  1.00 20.00           C
ATOM   6328  CG  MET C 644     113.727 113.905  87.311  1.00 20.00           C
ATOM   6329  SD  MET C 644     114.909 114.638  86.141  1.00 20.00           S
ATOM   6330  CE  MET C 644     114.204 114.096  84.563  1.00 20.00           C
ATOM   6331  H   MET C 644     111.672 115.503  89.749  1.00  0.00           H
ATOM   6332  HA  MET C 644     113.281 116.526  87.451  1.00  0.00           H
ATOM   6333  HB2 MET C 644     111.674 113.984  87.970  1.00  0.00           H
ATOM   6334  HB3 MET C 644     111.924 114.499  86.341  1.00  0.00           H
ATOM   6335  HG2 MET C 644     114.165 113.928  88.307  1.00  0.00           H
ATOM   6336  HG3 MET C 644     113.618 112.853  87.053  1.00  0.00           H
ATOM   6337  HE1 MET C 644     114.860 114.389  83.743  1.00  0.00           H
ATOM   6338  HE2 MET C 644     114.094 113.011  84.546  1.00  0.00           H
ATOM   6339  HE3 MET C 644     113.227 114.551  84.399  1.00  0.00           H
ATOM   6340  N   GLY C 645     110.477 117.599  88.102  1.00 30.00           N
ATOM   6341  CA  GLY C 645     109.281 118.338  87.701  1.00 30.00           C
ATOM   6342  C   GLY C 645     109.664 119.586  86.901  1.00 30.00           C
ATOM   6343  O   GLY C 645     110.469 120.398  87.359  1.00 30.00           O
ATOM   6344  H   GLY C 645     110.728 117.626  89.079  1.00  0.00           H
ATOM   6345  HA2 GLY C 645     108.606 117.697  87.131  1.00  0.00           H
ATOM   6346  HA3 GLY C 645     108.744 118.644  88.593  1.00  0.00           H
ATOM   6347  N   ASP C 646     109.041 119.734  85.723  1.00 30.00           N
ATOM   6348  CA  ASP C 646     109.221 120.851  84.799  1.00 30.00           C
ATOM   6349  C   ASP C 646     108.032 121.812  84.973  1.00 30.00           C
ATOM   6350  O   ASP C 646     106.883 121.376  84.914  1.00 30.00           O
ATOM   6351  CB  ASP C 646     109.348 120.316  83.349  1.00 20.00           C
ATOM   6352  CG  ASP C 646     109.583 121.365  82.252  1.00 20.00           C
ATOM   6353  OD1 ASP C 646     109.913 122.525  82.587  1.00 20.00           O
ATOM   6354  OD2 ASP C 646     109.465 120.965  81.074  1.00 20.00           O1-
ATOM   6355  H   ASP C 646     108.374 119.035  85.428  1.00  0.00           H
ATOM   6356  HA  ASP C 646     110.143 121.382  85.043  1.00  0.00           H
ATOM   6357  HB2 ASP C 646     110.182 119.614  83.311  1.00  0.00           H
ATOM   6358  HB3 ASP C 646     108.446 119.759  83.099  1.00  0.00           H
ATOM   6359  N   LEU C 647     108.340 123.104  85.172  1.00 30.00           N
ATOM   6360  CA  LEU C 647     107.367 124.196  85.282  1.00 30.00           C
ATOM   6361  C   LEU C 647     106.700 124.551  83.941  1.00 30.00           C
ATOM   6362  O   LEU C 647     105.570 125.034  83.957  1.00 30.00           O
ATOM   6363  CB  LEU C 647     108.072 125.443  85.863  1.00 20.00           C
ATOM   6364  CG  LEU C 647     108.432 125.326  87.361  1.00 20.00           C
ATOM   6365  CD1 LEU C 647     109.417 126.436  87.786  1.00 20.00           C
ATOM   6366  CD2 LEU C 647     107.173 125.311  88.251  1.00 20.00           C
ATOM   6367  H   LEU C 647     109.313 123.376  85.188  1.00  0.00           H
ATOM   6368  HA  LEU C 647     106.570 123.886  85.959  1.00  0.00           H
ATOM   6369  HB2 LEU C 647     108.975 125.630  85.278  1.00  0.00           H
ATOM   6370  HB3 LEU C 647     107.449 126.329  85.726  1.00  0.00           H
ATOM   6371  HG  LEU C 647     108.953 124.379  87.510  1.00  0.00           H
ATOM   6372 HD11 LEU C 647     110.317 126.007  88.227  1.00  0.00           H
ATOM   6373 HD12 LEU C 647     109.734 127.050  86.943  1.00  0.00           H
ATOM   6374 HD13 LEU C 647     108.988 127.116  88.522  1.00  0.00           H
ATOM   6375 HD21 LEU C 647     107.347 125.766  89.225  1.00  0.00           H
ATOM   6376 HD22 LEU C 647     106.342 125.840  87.785  1.00  0.00           H
ATOM   6377 HD23 LEU C 647     106.842 124.292  88.440  1.00  0.00           H
ATOM   6378  N   GLU C 648     107.396 124.303  82.821  1.00 30.00           N
```

```
ATOM   6379  CA  GLU C 648     106.947 124.613  81.464  1.00 30.00           C
ATOM   6380  C   GLU C 648     106.731 123.310  80.671  1.00 30.00           C
ATOM   6381  O   GLU C 648     107.111 123.234  79.502  1.00 30.00           O
ATOM   6382  CB  GLU C 648     107.983 125.559  80.803  1.00 20.00           C
ATOM   6383  CG  GLU C 648     108.196 126.918  81.509  1.00  0.00           C
ATOM   6384  CD  GLU C 648     107.009 127.875  81.378  1.00  0.00           C
ATOM   6385  OE1 GLU C 648     106.029 127.705  82.136  1.00  0.00           O
ATOM   6386  OE2 GLU C 648     107.109 128.781  80.522  1.00  0.00           O1-
ATOM   6387  H   GLU C 648     108.308 123.867  82.888  1.00  0.00           H
ATOM   6388  HA  GLU C 648     105.979 125.114  81.478  1.00  0.00           H
ATOM   6389  HB2 GLU C 648     108.945 125.047  80.748  1.00  0.00           H
ATOM   6390  HB3 GLU C 648     107.697 125.752  79.768  1.00  0.00           H
ATOM   6391  HG2 GLU C 648     108.427 126.778  82.565  1.00  0.00           H
ATOM   6392  HG3 GLU C 648     109.072 127.404  81.078  1.00  0.00           H
ATOM   6393  N   PHE C 649     106.111 122.304  81.319  1.00 30.00           N
ATOM   6394  CA  PHE C 649     105.848 120.972  80.754  1.00 30.00           C
ATOM   6395  C   PHE C 649     104.779 120.959  79.639  1.00 30.00           C
ATOM   6396  O   PHE C 649     104.656 119.945  78.952  1.00 30.00           O
ATOM   6397  CB  PHE C 649     105.500 119.988  81.898  1.00 20.00           C
ATOM   6398  CG  PHE C 649     104.108 120.065  82.515  1.00 20.00           C
ATOM   6399  CD1 PHE C 649     103.813 121.019  83.514  1.00 20.00           C
ATOM   6400  CD2 PHE C 649     103.063 119.257  82.017  1.00 20.00           C
ATOM   6401  CE1 PHE C 649     102.533 121.092  84.049  1.00 20.00           C
ATOM   6402  CE2 PHE C 649     101.790 119.345  82.566  1.00 20.00           C
ATOM   6403  CZ  PHE C 649     101.529 120.254  83.583  1.00 20.00           C
ATOM   6404  H   PHE C 649     105.835 122.437  82.281  1.00  0.00           H
ATOM   6405  HA  PHE C 649     106.782 120.631  80.304  1.00  0.00           H
ATOM   6406  HB2 PHE C 649     106.232 120.088  82.697  1.00  0.00           H
ATOM   6407  HB3 PHE C 649     105.631 118.970  81.527  1.00  0.00           H
ATOM   6408  HD1 PHE C 649     104.578 121.688  83.876  1.00  0.00           H
ATOM   6409  HD2 PHE C 649     103.254 118.553  81.220  1.00  0.00           H
ATOM   6410  HE1 PHE C 649     102.314 121.812  84.823  1.00  0.00           H
ATOM   6411  HE2 PHE C 649     101.000 118.707  82.199  1.00  0.00           H
ATOM   6412  HZ  PHE C 649     100.536 120.320  84.002  1.00  0.00           H
ATOM   6413  N   THR C 650     104.031 122.063  79.479  1.00 30.00           N
ATOM   6414  CA  THR C 650     102.962 122.212  78.498  1.00 30.00           C
ATOM   6415  C   THR C 650     102.640 123.710  78.291  1.00 30.00           C
ATOM   6416  O   THR C 650     102.981 124.528  79.150  1.00 30.00           O
ATOM   6417  CB  THR C 650     101.701 121.405  78.930  1.00 20.00           C
ATOM   6418  CG2 THR C 650     101.016 121.890  80.219  1.00 20.00           C
ATOM   6419  OG1 THR C 650     100.720 121.421  77.919  1.00 20.00           O
ATOM   6420  H   THR C 650     104.201 122.868  80.065  1.00  0.00           H
ATOM   6421  HA  THR C 650     103.326 121.825  77.544  1.00  0.00           H
ATOM   6422  HB  THR C 650     101.983 120.363  79.076  1.00  0.00           H
ATOM   6423  HG1 THR C 650     101.063 120.957  77.150  1.00  0.00           H
ATOM   6424 HG21 THR C 650     100.223 121.205  80.516  1.00  0.00           H
ATOM   6425 HG22 THR C 650     101.724 121.942  81.045  1.00  0.00           H
ATOM   6426 HG23 THR C 650     100.565 122.873  80.100  1.00  0.00           H
ATOM   6427  N   GLU C 651     102.023 124.041  77.141  1.00 30.00           N
ATOM   6428  CA  GLU C 651     101.676 125.409  76.730  1.00 30.00           C
ATOM   6429  C   GLU C 651     100.229 125.545  76.210  1.00 30.00           C
ATOM   6430  O   GLU C 651      99.791 126.677  76.003  1.00 30.00           O
ATOM   6431  CB  GLU C 651     102.678 125.907  75.663  1.00 20.00           C
ATOM   6432  CG  GLU C 651     104.129 126.043  76.172  1.00  0.00           C
ATOM   6433  CD  GLU C 651     105.084 126.644  75.136  1.00  0.00           C
ATOM   6434  OE1 GLU C 651     104.840 126.445  73.925  1.00  0.00           O
ATOM   6435  OE2 GLU C 651     106.056 127.294  75.578  1.00  0.00           O1-
ATOM   6436  H   GLU C 651     101.801 123.314  76.477  1.00  0.00           H
ATOM   6437  HA  GLU C 651     101.744 126.080  77.585  1.00  0.00           H
ATOM   6438  HB2 GLU C 651     102.657 125.225  74.811  1.00  0.00           H
ATOM   6439  HB3 GLU C 651     102.354 126.875  75.277  1.00  0.00           H
ATOM   6440  HG2 GLU C 651     104.146 126.667  77.067  1.00  0.00           H
ATOM   6441  HG3 GLU C 651     104.526 125.070  76.462  1.00  0.00           H
ATOM   6442  N   ASN C 652      99.517 124.422  75.997  1.00 30.00           N
ATOM   6443  CA  ASN C 652      98.159 124.355  75.428  1.00 30.00           C
ATOM   6444  C   ASN C 652      97.068 124.722  76.456  1.00 30.00           C
ATOM   6445  O   ASN C 652      96.414 123.842  77.020  1.00 30.00           O
ATOM   6446  CB  ASN C 652      97.919 122.993  74.711  1.00 20.00           C
ATOM   6447  CG  ASN C 652      98.561 121.765  75.370  1.00 20.00           C
ATOM   6448  ND2 ASN C 652      98.074 121.392  76.551  1.00 20.00           N
ATOM   6449  OD1 ASN C 652      99.492 121.172  74.830  1.00 20.00           O
```

```
ATOM   6450  H   ASN C 652      99.931 123.531  76.227  1.00  0.00           H
ATOM   6451  HA  ASN C 652      98.163 125.108  74.636  1.00  0.00           H
ATOM   6452  HB2 ASN C 652      98.362 123.062  73.717  1.00  0.00           H
ATOM   6453  HB3 ASN C 652      96.856 122.815  74.534  1.00  0.00           H
ATOM   6454 HD21 ASN C 652      98.474 120.609  77.047  1.00  0.00           H
ATOM   6455 HD22 ASN C 652      97.309 121.908  76.964  1.00  0.00           H
ATOM   6456  N   TYR C 653      96.910 126.036  76.672  1.00 30.00           N
ATOM   6457  CA  TYR C 653      95.971 126.660  77.603  1.00 30.00           C
ATOM   6458  C   TYR C 653      95.895 128.169  77.323  1.00 30.00           C
ATOM   6459  O   TYR C 653      96.841 128.741  76.778  1.00 30.00           O
ATOM   6460  CB  TYR C 653      96.387 126.382  79.077  1.00 20.00           C
ATOM   6461  CG  TYR C 653      97.837 126.682  79.441  1.00 20.00           C
ATOM   6462  CD1 TYR C 653      98.734 125.620  79.680  1.00 20.00           C
ATOM   6463  CD2 TYR C 653      98.308 128.011  79.512  1.00 20.00           C
ATOM   6464  CE1 TYR C 653     100.091 125.889  79.936  1.00 20.00           C
ATOM   6465  CE2 TYR C 653      99.673 128.272  79.723  1.00 20.00           C
ATOM   6466  CZ  TYR C 653     100.571 127.212  79.924  1.00 20.00           C
ATOM   6467  OH  TYR C 653     101.901 127.466  80.098  1.00 20.00           O
ATOM   6468  H   TYR C 653      97.505 126.687  76.178  1.00  0.00           H
ATOM   6469  HA  TYR C 653      94.985 126.233  77.421  1.00  0.00           H
ATOM   6470  HB2 TYR C 653      96.201 125.333  79.302  1.00  0.00           H
ATOM   6471  HB3 TYR C 653      95.739 126.928  79.763  1.00  0.00           H
ATOM   6472  HD1 TYR C 653      98.392 124.596  79.649  1.00  0.00           H
ATOM   6473  HD2 TYR C 653      97.637 128.841  79.361  1.00  0.00           H
ATOM   6474  HE1 TYR C 653     100.767 125.073  80.125  1.00  0.00           H
ATOM   6475  HE2 TYR C 653     100.033 129.287  79.712  1.00  0.00           H
ATOM   6476  HH  TYR C 653     102.424 126.666  80.196  1.00  0.00           H
ATOM   6477  N   ASP C 654      94.817 128.794  77.820  1.00 30.00           N
ATOM   6478  CA  ASP C 654      94.786 130.220  78.159  1.00 30.00           C
ATOM   6479  C   ASP C 654      95.166 130.412  79.638  1.00 30.00           C
ATOM   6480  O   ASP C 654      95.066 129.476  80.433  1.00 30.00           O
ATOM   6481  CB  ASP C 654      93.421 130.895  77.867  1.00 20.00           C
ATOM   6482  CG  ASP C 654      92.973 130.872  76.401  1.00  0.00           C
ATOM   6483  OD1 ASP C 654      93.840 130.680  75.520  1.00  0.00           O
ATOM   6484  OD2 ASP C 654      91.779 131.172  76.185  1.00  0.00           O1-
ATOM   6485  H   ASP C 654      94.052 128.247  78.189  1.00  0.00           H
ATOM   6486  HA  ASP C 654      95.541 130.746  77.570  1.00  0.00           H
ATOM   6487  HB2 ASP C 654      92.655 130.387  78.454  1.00  0.00           H
ATOM   6488  HB3 ASP C 654      93.447 131.938  78.183  1.00  0.00           H
ATOM   6489  N   PHE C 655      95.567 131.650  79.973  1.00 30.00           N
ATOM   6490  CA  PHE C 655      95.883 132.142  81.320  1.00 30.00           C
ATOM   6491  C   PHE C 655      97.142 131.465  81.897  1.00 30.00           C
ATOM   6492  O   PHE C 655      97.039 130.634  82.802  1.00 30.00           O
ATOM   6493  CB  PHE C 655      94.658 132.028  82.266  1.00 20.00           C
ATOM   6494  CG  PHE C 655      93.379 132.653  81.733  1.00 20.00           C
ATOM   6495  CD1 PHE C 655      93.247 134.055  81.658  1.00 20.00           C
ATOM   6496  CD2 PHE C 655      92.378 131.838  81.162  1.00 20.00           C
ATOM   6497  CE1 PHE C 655      92.123 134.614  81.064  1.00 20.00           C
ATOM   6498  CE2 PHE C 655      91.263 132.416  80.571  1.00 20.00           C
ATOM   6499  CZ  PHE C 655      91.135 133.799  80.525  1.00 20.00           C
ATOM   6500  H   PHE C 655      95.605 132.348  79.245  1.00  0.00           H
ATOM   6501  HA  PHE C 655      96.114 133.202  81.208  1.00  0.00           H
ATOM   6502  HB2 PHE C 655      94.899 132.518  83.210  1.00  0.00           H
ATOM   6503  HB3 PHE C 655      94.450 130.987  82.521  1.00  0.00           H
ATOM   6504  HD1 PHE C 655      94.021 134.697  82.054  1.00  0.00           H
ATOM   6505  HD2 PHE C 655      92.480 130.762  81.169  1.00  0.00           H
ATOM   6506  HE1 PHE C 655      92.019 135.688  81.012  1.00  0.00           H
ATOM   6507  HE2 PHE C 655      90.499 131.789  80.135  1.00  0.00           H
ATOM   6508  HZ  PHE C 655      90.268 134.241  80.058  1.00  0.00           H
ATOM   6509  N   LYS C 656      98.316 131.843  81.357  1.00 30.00           N
ATOM   6510  CA  LYS C 656      99.624 131.346  81.803  1.00 30.00           C
ATOM   6511  C   LYS C 656      99.976 131.742  83.250  1.00 30.00           C
ATOM   6512  O   LYS C 656     100.648 130.966  83.930  1.00 30.00           O
ATOM   6513  CB  LYS C 656     100.728 131.778  80.812  1.00 20.00           C
ATOM   6514  CG  LYS C 656     102.091 131.125  81.116  1.00 20.00           C
ATOM   6515  CD  LYS C 656     103.139 131.314  80.013  1.00 20.00           C
ATOM   6516  CE  LYS C 656     104.421 130.536  80.338  1.00 20.00           C
ATOM   6517  NZ  LYS C 656     105.434 130.676  79.280  1.00 20.00           N1+
ATOM   6518  H   LYS C 656      98.326 132.512  80.600  1.00  0.00           H
ATOM   6519  HA  LYS C 656      99.564 130.258  81.780  1.00  0.00           H
ATOM   6520  HB2 LYS C 656     100.424 131.498  79.803  1.00  0.00           H
```

```
ATOM   6521  HB3 LYS C 656     100.830 132.865  80.803  1.00  0.00           H
ATOM   6522  HG2 LYS C 656     102.497 131.527  82.044  1.00  0.00           H
ATOM   6523  HG3 LYS C 656     101.945 130.057  81.289  1.00  0.00           H
ATOM   6524  HD2 LYS C 656     102.733 130.976  79.059  1.00  0.00           H
ATOM   6525  HD3 LYS C 656     103.364 132.375  79.902  1.00  0.00           H
ATOM   6526  HE2 LYS C 656     104.850 130.876  81.282  1.00  0.00           H
ATOM   6527  HE3 LYS C 656     104.198 129.475  80.455  1.00  0.00           H
ATOM   6528  HZ1 LYS C 656     106.231 130.104  79.547  1.00  0.00           H
ATOM   6529  HZ2 LYS C 656     105.065 130.338  78.403  1.00  0.00           H
ATOM   6530  HZ3 LYS C 656     105.719 131.639  79.192  1.00  0.00           H
ATOM   6531  N   ALA C 657      99.484 132.911  83.699  1.00 30.00           N
ATOM   6532  CA  ALA C 657      99.603 133.393  85.074  1.00 30.00           C
ATOM   6533  C   ALA C 657      98.953 132.443  86.091  1.00 30.00           C
ATOM   6534  O   ALA C 657      99.581 132.145  87.102  1.00 30.00           O
ATOM   6535  CB  ALA C 657      99.000 134.802  85.176  1.00 30.00           C
ATOM   6536  H   ALA C 657      98.947 133.490  83.071  1.00  0.00           H
ATOM   6537  HA  ALA C 657     100.667 133.462  85.310  1.00  0.00           H
ATOM   6538  HB1 ALA C 657      99.083 135.194  86.191  1.00  0.00           H
ATOM   6539  HB2 ALA C 657      99.517 135.498  84.515  1.00  0.00           H
ATOM   6540  HB3 ALA C 657      97.944 134.808  84.903  1.00  0.00           H
ATOM   6541  N   VAL C 658      97.745 131.944  85.776  1.00 30.00           N
ATOM   6542  CA  VAL C 658      97.014 130.962  86.579  1.00 30.00           C
ATOM   6543  C   VAL C 658      97.710 129.584  86.589  1.00 30.00           C
ATOM   6544  O   VAL C 658      97.762 128.957  87.646  1.00 30.00           O
ATOM   6545  CB  VAL C 658      95.547 130.798  86.077  1.00 20.00           C
ATOM   6546  CG1 VAL C 658      94.733 129.705  86.803  1.00 20.00           C
ATOM   6547  CG2 VAL C 658      94.779 132.131  86.161  1.00 20.00           C
ATOM   6548  H   VAL C 658      97.308 132.217  84.907  1.00  0.00           H
ATOM   6549  HA  VAL C 658      96.985 131.328  87.607  1.00  0.00           H
ATOM   6550  HB  VAL C 658      95.577 130.518  85.024  1.00  0.00           H
ATOM   6551 HG11 VAL C 658      93.697 129.697  86.463  1.00  0.00           H
ATOM   6552 HG12 VAL C 658      95.125 128.707  86.621  1.00  0.00           H
ATOM   6553 HG13 VAL C 658      94.726 129.870  87.881  1.00  0.00           H
ATOM   6554 HG21 VAL C 658      93.764 132.025  85.777  1.00  0.00           H
ATOM   6555 HG22 VAL C 658      94.710 132.481  87.192  1.00  0.00           H
ATOM   6556 HG23 VAL C 658      95.260 132.916  85.578  1.00  0.00           H
ATOM   6557  N   PHE C 659      98.262 129.163  85.436  1.00 30.00           N
ATOM   6558  CA  PHE C 659      98.987 127.901  85.256  1.00 30.00           C
ATOM   6559  C   PHE C 659     100.234 127.770  86.142  1.00 30.00           C
ATOM   6560  O   PHE C 659     100.371 126.768  86.844  1.00 30.00           O
ATOM   6561  CB  PHE C 659      99.292 127.683  83.754  1.00 20.00           C
ATOM   6562  CG  PHE C 659     100.200 126.515  83.403  1.00 20.00           C
ATOM   6563  CD1 PHE C 659      99.705 125.194  83.407  1.00 20.00           C
ATOM   6564  CD2 PHE C 659     101.578 126.730  83.182  1.00 20.00           C
ATOM   6565  CE1 PHE C 659     100.557 124.133  83.125  1.00 20.00           C
ATOM   6566  CE2 PHE C 659     102.409 125.659  82.884  1.00 20.00           C
ATOM   6567  CZ  PHE C 659     101.900 124.368  82.853  1.00 20.00           C
ATOM   6568  H   PHE C 659      98.177 129.743  84.613  1.00  0.00           H
ATOM   6569  HA  PHE C 659      98.318 127.100  85.573  1.00  0.00           H
ATOM   6570  HB2 PHE C 659      98.360 127.548  83.206  1.00  0.00           H
ATOM   6571  HB3 PHE C 659      99.735 128.585  83.337  1.00  0.00           H
ATOM   6572  HD1 PHE C 659      98.662 125.004  83.612  1.00  0.00           H
ATOM   6573  HD2 PHE C 659     101.987 127.730  83.215  1.00  0.00           H
ATOM   6574  HE1 PHE C 659     100.174 123.124  83.109  1.00  0.00           H
ATOM   6575  HE2 PHE C 659     103.455 125.834  82.681  1.00  0.00           H
ATOM   6576  HZ  PHE C 659     102.559 123.545  82.622  1.00  0.00           H
ATOM   6577  N   ILE C 660     101.103 128.793  86.103  1.00 30.00           N
ATOM   6578  CA  ILE C 660     102.342 128.813  86.875  1.00 30.00           C
ATOM   6579  C   ILE C 660     102.114 129.158  88.363  1.00 30.00           C
ATOM   6580  O   ILE C 660     102.919 128.729  89.185  1.00 30.00           O
ATOM   6581  CB  ILE C 660     103.395 129.765  86.240  1.00 20.00           C
ATOM   6582  CG1 ILE C 660     104.831 129.447  86.721  1.00 20.00           C
ATOM   6583  CG2 ILE C 660     103.077 131.267  86.403  1.00 20.00           C
ATOM   6584  CD1 ILE C 660     105.921 129.889  85.736  1.00 20.00           C
ATOM   6585  H   ILE C 660     100.928 129.586  85.500  1.00  0.00           H
ATOM   6586  HA  ILE C 660     102.754 127.802  86.842  1.00  0.00           H
ATOM   6587  HB  ILE C 660     103.370 129.559  85.169  1.00  0.00           H
ATOM   6588 HG12 ILE C 660     105.010 129.902  87.696  1.00  0.00           H
ATOM   6589 HG13 ILE C 660     104.944 128.373  86.871  1.00  0.00           H
ATOM   6590 HG21 ILE C 660     103.737 131.878  85.787  1.00  0.00           H
ATOM   6591 HG22 ILE C 660     102.056 131.491  86.098  1.00  0.00           H
```

```
ATOM   6592 HG23 ILE C 660     103.193 131.601  87.434  1.00  0.00           H
ATOM   6593 HD11 ILE C 660     106.900 129.541  86.066  1.00  0.00           H
ATOM   6594 HD12 ILE C 660     105.748 129.478  84.741  1.00  0.00           H
ATOM   6595 HD13 ILE C 660     105.968 130.974  85.651  1.00  0.00           H
ATOM   6596  N   ILE C 661     101.003 129.846  88.700  1.00 30.00           N
ATOM   6597  CA  ILE C 661     100.549 130.040  90.083  1.00 30.00           C
ATOM   6598  C   ILE C 661     100.101 128.723  90.747  1.00 30.00           C
ATOM   6599  O   ILE C 661     100.423 128.534  91.916  1.00 30.00           O
ATOM   6600  CB  ILE C 661      99.442 131.137  90.209  1.00 20.00           C
ATOM   6601  CG1 ILE C 661     100.087 132.541  90.132  1.00 20.00           C
ATOM   6602  CG2 ILE C 661      98.533 131.060  91.461  1.00 20.00           C
ATOM   6603  CD1 ILE C 661      99.090 133.679  89.858  1.00 20.00           C
ATOM   6604  H   ILE C 661     100.387 130.189  87.976  1.00  0.00           H
ATOM   6605  HA  ILE C 661     101.415 130.387  90.650  1.00  0.00           H
ATOM   6606  HB  ILE C 661      98.783 131.025  89.347  1.00  0.00           H
ATOM   6607 HG12 ILE C 661     100.615 132.745  91.064  1.00  0.00           H
ATOM   6608 HG13 ILE C 661     100.854 132.559  89.357  1.00  0.00           H
ATOM   6609 HG21 ILE C 661      97.824 131.887  91.490  1.00  0.00           H
ATOM   6610 HG22 ILE C 661      97.932 130.150  91.479  1.00  0.00           H
ATOM   6611 HG23 ILE C 661      99.118 131.099  92.380  1.00  0.00           H
ATOM   6612 HD11 ILE C 661      99.552 134.461  89.256  1.00  0.00           H
ATOM   6613 HD12 ILE C 661      98.210 133.328  89.318  1.00  0.00           H
ATOM   6614 HD13 ILE C 661      98.751 134.135  90.789  1.00  0.00           H
ATOM   6615  N   LEU C 662      99.427 127.827  90.000  1.00 30.00           N
ATOM   6616  CA  LEU C 662      99.064 126.482  90.466  1.00 30.00           C
ATOM   6617  C   LEU C 662     100.294 125.595  90.722  1.00 30.00           C
ATOM   6618  O   LEU C 662     100.289 124.852  91.700  1.00 30.00           O
ATOM   6619  CB  LEU C 662      98.111 125.794  89.459  1.00 20.00           C
ATOM   6620  CG  LEU C 662      96.662 126.329  89.460  1.00 20.00           C
ATOM   6621  CD1 LEU C 662      95.873 125.777  88.254  1.00 20.00           C
ATOM   6622  CD2 LEU C 662      95.931 126.069  90.792  1.00 20.00           C
ATOM   6623  H   LEU C 662      99.179 128.055  89.047  1.00  0.00           H
ATOM   6624  HA  LEU C 662      98.549 126.591  91.421  1.00  0.00           H
ATOM   6625  HB2 LEU C 662      98.536 125.888  88.460  1.00  0.00           H
ATOM   6626  HB3 LEU C 662      98.071 124.721  89.657  1.00  0.00           H
ATOM   6627  HG  LEU C 662      96.709 127.409  89.334  1.00  0.00           H
ATOM   6628 HD11 LEU C 662      95.154 125.011  88.541  1.00  0.00           H
ATOM   6629 HD12 LEU C 662      95.317 126.575  87.763  1.00  0.00           H
ATOM   6630 HD13 LEU C 662      96.524 125.329  87.502  1.00  0.00           H
ATOM   6631 HD21 LEU C 662      94.902 125.743  90.647  1.00  0.00           H
ATOM   6632 HD22 LEU C 662      96.428 125.305  91.386  1.00  0.00           H
ATOM   6633 HD23 LEU C 662      95.893 126.978  91.394  1.00  0.00           H
ATOM   6634  N   LEU C 663     101.327 125.702  89.867  1.00 30.00           N
ATOM   6635  CA  LEU C 663     102.587 124.968  90.008  1.00 30.00           C
ATOM   6636  C   LEU C 663     103.431 125.467  91.191  1.00 30.00           C
ATOM   6637  O   LEU C 663     103.963 124.637  91.924  1.00 30.00           O
ATOM   6638  CB  LEU C 663     103.396 125.051  88.699  1.00 20.00           C
ATOM   6639  CG  LEU C 663     102.778 124.271  87.521  1.00 20.00           C
ATOM   6640  CD1 LEU C 663     103.368 124.747  86.182  1.00 20.00           C
ATOM   6641  CD2 LEU C 663     102.900 122.742  87.710  1.00 20.00           C
ATOM   6642  H   LEU C 663     101.256 126.326  89.075  1.00  0.00           H
ATOM   6643  HA  LEU C 663     102.345 123.922  90.206  1.00  0.00           H
ATOM   6644  HB2 LEU C 663     103.505 126.102  88.428  1.00  0.00           H
ATOM   6645  HB3 LEU C 663     104.409 124.682  88.862  1.00  0.00           H
ATOM   6646  HG  LEU C 663     101.713 124.502  87.485  1.00  0.00           H
ATOM   6647 HD11 LEU C 663     103.638 123.925  85.519  1.00  0.00           H
ATOM   6648 HD12 LEU C 663     102.642 125.361  85.651  1.00  0.00           H
ATOM   6649 HD13 LEU C 663     104.260 125.358  86.321  1.00  0.00           H
ATOM   6650 HD21 LEU C 663     103.520 122.269  86.948  1.00  0.00           H
ATOM   6651 HD22 LEU C 663     103.342 122.478  88.670  1.00  0.00           H
ATOM   6652 HD23 LEU C 663     101.918 122.271  87.665  1.00  0.00           H
ATOM   6653  N   LEU C 664     103.519 126.796  91.375  1.00 30.00           N
ATOM   6654  CA  LEU C 664     104.240 127.428  92.483  1.00 30.00           C
ATOM   6655  C   LEU C 664     103.529 127.198  93.826  1.00 30.00           C
ATOM   6656  O   LEU C 664     104.202 126.844  94.790  1.00 30.00           O
ATOM   6657  CB  LEU C 664     104.461 128.933  92.196  1.00 20.00           C
ATOM   6658  CG  LEU C 664     105.824 129.281  91.549  1.00 20.00           C
ATOM   6659  CD1 LEU C 664     106.163 128.477  90.274  1.00 20.00           C
ATOM   6660  CD2 LEU C 664     105.950 130.803  91.323  1.00 20.00           C
ATOM   6661  H   LEU C 664     103.070 127.422  90.719  1.00  0.00           H
ATOM   6662  HA  LEU C 664     105.219 126.949  92.563  1.00  0.00           H
```

```
ATOM   6663  HB2 LEU C 664     103.640 129.326  91.596  1.00  0.00           H
ATOM   6664  HB3 LEU C 664     104.410 129.489  93.134  1.00  0.00           H
ATOM   6665  HG  LEU C 664     106.584 129.018  92.287  1.00  0.00           H
ATOM   6666 HD11 LEU C 664     106.573 129.101  89.480  1.00  0.00           H
ATOM   6667 HD12 LEU C 664     106.916 127.721  90.495  1.00  0.00           H
ATOM   6668 HD13 LEU C 664     105.303 127.961  89.854  1.00  0.00           H
ATOM   6669 HD21 LEU C 664     106.884 131.181  91.739  1.00  0.00           H
ATOM   6670 HD22 LEU C 664     105.930 131.071  90.266  1.00  0.00           H
ATOM   6671 HD23 LEU C 664     105.139 131.355  91.800  1.00  0.00           H
ATOM   6672  N   ALA C 665     102.192 127.339  93.861  1.00 30.00           N
ATOM   6673  CA  ALA C 665     101.359 127.050  95.033  1.00 30.00           C
ATOM   6674  C   ALA C 665     101.317 125.560  95.414  1.00 30.00           C
ATOM   6675  O   ALA C 665     101.037 125.265  96.571  1.00 30.00           O
ATOM   6676  CB  ALA C 665      99.937 127.580  94.807  1.00 20.00           C
ATOM   6677  H   ALA C 665     101.696 127.641  93.031  1.00  0.00           H
ATOM   6678  HA  ALA C 665     101.786 127.588  95.882  1.00  0.00           H
ATOM   6679  HB1 ALA C 665      99.304 127.401  95.677  1.00  0.00           H
ATOM   6680  HB2 ALA C 665      99.943 128.655  94.626  1.00  0.00           H
ATOM   6681  HB3 ALA C 665      99.463 127.098  93.951  1.00  0.00           H
ATOM   6682  N   TYR C 666     101.615 124.660  94.463  1.00 30.00           N
ATOM   6683  CA  TYR C 666     101.803 123.235  94.712  1.00 30.00           C
ATOM   6684  C   TYR C 666     103.196 122.951  95.304  1.00 30.00           C
ATOM   6685  O   TYR C 666     103.267 122.353  96.374  1.00 30.00           O
ATOM   6686  CB  TYR C 666     101.522 122.441  93.419  1.00 20.00           C
ATOM   6687  CG  TYR C 666     101.829 120.957  93.490  1.00 20.00           C
ATOM   6688  CD1 TYR C 666     101.033 120.102  94.280  1.00 20.00           C
ATOM   6689  CD2 TYR C 666     102.932 120.435  92.785  1.00 20.00           C
ATOM   6690  CE1 TYR C 666     101.343 118.731  94.369  1.00 20.00           C
ATOM   6691  CE2 TYR C 666     103.237 119.067  92.871  1.00 20.00           C
ATOM   6692  CZ  TYR C 666     102.443 118.211  93.660  1.00 20.00           C
ATOM   6693  OH  TYR C 666     102.756 116.886  93.753  1.00 20.00           O
ATOM   6694  H   TYR C 666     101.798 124.976  93.520  1.00  0.00           H
ATOM   6695  HA  TYR C 666     101.061 122.917  95.448  1.00  0.00           H
ATOM   6696  HB2 TYR C 666     100.477 122.556  93.138  1.00  0.00           H
ATOM   6697  HB3 TYR C 666     102.094 122.866  92.595  1.00  0.00           H
ATOM   6698  HD1 TYR C 666     100.193 120.496  94.832  1.00  0.00           H
ATOM   6699  HD2 TYR C 666     103.552 121.083  92.183  1.00  0.00           H
ATOM   6700  HE1 TYR C 666     100.746 118.085  94.993  1.00  0.00           H
ATOM   6701  HE2 TYR C 666     104.089 118.687  92.332  1.00  0.00           H
ATOM   6702  HH  TYR C 666     103.465 116.641  93.151  1.00  0.00           H
ATOM   6703  N   VAL C 667     104.261 123.391  94.608  1.00 30.00           N
ATOM   6704  CA  VAL C 667     105.672 123.169  94.955  1.00 30.00           C
ATOM   6705  C   VAL C 667     106.090 123.775  96.310  1.00 30.00           C
ATOM   6706  O   VAL C 667     106.808 123.111  97.059  1.00 30.00           O
ATOM   6707  CB  VAL C 667     106.610 123.691  93.823  1.00 20.00           C
ATOM   6708  CG1 VAL C 667     108.097 123.896  94.195  1.00 20.00           C
ATOM   6709  CG2 VAL C 667     106.531 122.763  92.600  1.00 20.00           C
ATOM   6710  H   VAL C 667     104.104 123.882  93.736  1.00  0.00           H
ATOM   6711  HA  VAL C 667     105.808 122.089  95.036  1.00  0.00           H
ATOM   6712  HB  VAL C 667     106.234 124.666  93.507  1.00  0.00           H
ATOM   6713 HG11 VAL C 667     108.683 124.172  93.318  1.00  0.00           H
ATOM   6714 HG12 VAL C 667     108.236 124.697  94.922  1.00  0.00           H
ATOM   6715 HG13 VAL C 667     108.533 122.985  94.604  1.00  0.00           H
ATOM   6716 HG21 VAL C 667     107.082 123.179  91.757  1.00  0.00           H
ATOM   6717 HG22 VAL C 667     106.954 121.785  92.829  1.00  0.00           H
ATOM   6718 HG23 VAL C 667     105.506 122.601  92.269  1.00  0.00           H
ATOM   6719  N   ILE C 668     105.603 124.989  96.618  1.00  0.00           N
ATOM   6720  CA  ILE C 668     105.804 125.654  97.909  1.00  0.00           C
ATOM   6721  C   ILE C 668     105.101 124.922  99.074  1.00  0.00           C
ATOM   6722  O   ILE C 668     105.617 124.945 100.189  1.00  0.00           O
ATOM   6723  CB  ILE C 668     105.372 127.154  97.862  1.00 20.00           C
ATOM   6724  CG1 ILE C 668     106.310 127.986  96.949  1.00 20.00           C
ATOM   6725  CG2 ILE C 668     105.188 127.860  99.227  1.00 20.00           C
ATOM   6726  CD1 ILE C 668     107.767 128.111  97.424  1.00 20.00           C
ATOM   6727  H   ILE C 668     105.027 125.483  95.948  1.00  0.00           H
ATOM   6728  HA  ILE C 668     106.871 125.627  98.114  1.00  0.00           H
ATOM   6729  HB  ILE C 668     104.389 127.183  97.389  1.00  0.00           H
ATOM   6730 HG12 ILE C 668     105.890 128.984  96.811  1.00  0.00           H
ATOM   6731 HG13 ILE C 668     106.334 127.548  95.953  1.00  0.00           H
ATOM   6732 HG21 ILE C 668     104.987 128.923  99.093  1.00  0.00           H
ATOM   6733 HG22 ILE C 668     104.343 127.455  99.784  1.00  0.00           H
```

ATOM	6734	HG23	ILE	C	668	106.072	127.766	99.857	1.00	0.00	H
ATOM	6735	HD11	ILE	C	668	108.239	128.990	96.984	1.00	0.00	H
ATOM	6736	HD12	ILE	C	668	107.849	128.207	98.505	1.00	0.00	H
ATOM	6737	HD13	ILE	C	668	108.352	127.243	97.120	1.00	0.00	H
ATOM	6738	N	LEU	C	669	103.964	124.266	98.786	1.00	0.00	N
ATOM	6739	CA	LEU	C	669	103.158	123.543	99.767	1.00	0.00	C
ATOM	6740	C	LEU	C	669	103.616	122.093	99.985	1.00	0.00	C
ATOM	6741	O	LEU	C	669	103.315	121.585	101.062	1.00	0.00	O
ATOM	6742	CB	LEU	C	669	101.667	123.579	99.353	1.00	20.00	C
ATOM	6743	CG	LEU	C	669	100.863	124.783	99.902	1.00	20.00	C
ATOM	6744	CD1	LEU	C	669	101.504	126.162	99.631	1.00	20.00	C
ATOM	6745	CD2	LEU	C	669	99.399	124.726	99.416	1.00	20.00	C
ATOM	6746	H	LEU	C	669	103.610	124.274	97.840	1.00	0.00	H
ATOM	6747	HA	LEU	C	669	103.250	124.040	100.736	1.00	0.00	H
ATOM	6748	HB2	LEU	C	669	101.585	123.512	98.270	1.00	0.00	H
ATOM	6749	HB3	LEU	C	669	101.163	122.683	99.721	1.00	0.00	H
ATOM	6750	HG	LEU	C	669	100.833	124.666	100.986	1.00	0.00	H
ATOM	6751	HD11	LEU	C	669	102.096	126.484	100.488	1.00	0.00	H
ATOM	6752	HD12	LEU	C	669	102.162	126.154	98.764	1.00	0.00	H
ATOM	6753	HD13	LEU	C	669	100.759	126.938	99.452	1.00	0.00	H
ATOM	6754	HD21	LEU	C	669	98.709	124.737	100.259	1.00	0.00	H
ATOM	6755	HD22	LEU	C	669	99.139	125.565	98.771	1.00	0.00	H
ATOM	6756	HD23	LEU	C	669	99.196	123.822	98.841	1.00	0.00	H
ATOM	6757	N	THR	C	670	104.298	121.447	99.016	1.00	0.00	N
ATOM	6758	CA	THR	C	670	104.610	120.015	99.102	1.00	0.00	C
ATOM	6759	C	THR	C	670	106.117	119.695	99.099	1.00	0.00	C
ATOM	6760	O	THR	C	670	106.544	118.883	99.918	1.00	0.00	O
ATOM	6761	CB	THR	C	670	103.886	119.173	98.008	1.00	20.00	C
ATOM	6762	CG2	THR	C	670	104.469	119.250	96.583	1.00	20.00	C
ATOM	6763	OG1	THR	C	670	103.940	117.804	98.356	1.00	20.00	O
ATOM	6764	H	THR	C	670	104.495	121.912	98.141	1.00	0.00	H
ATOM	6765	HA	THR	C	670	104.241	119.631	100.051	1.00	0.00	H
ATOM	6766	HB	THR	C	670	102.837	119.469	97.975	1.00	0.00	H
ATOM	6767	HG1	THR	C	670	104.850	117.503	98.285	1.00	0.00	H
ATOM	6768	HG21	THR	C	670	103.793	118.776	95.877	1.00	0.00	H
ATOM	6769	HG22	THR	C	670	104.623	120.275	96.255	1.00	0.00	H
ATOM	6770	HG23	THR	C	670	105.425	118.733	96.494	1.00	0.00	H
ATOM	6771	N	TYR	C	671	106.897	120.297	98.185	1.00	0.00	N
ATOM	6772	CA	TYR	C	671	108.288	119.901	97.950	1.00	0.00	C
ATOM	6773	C	TYR	C	671	109.269	120.520	98.959	1.00	0.00	C
ATOM	6774	O	TYR	C	671	110.193	119.832	99.388	1.00	0.00	O
ATOM	6775	CB	TYR	C	671	108.681	120.200	96.488	1.00	20.00	C
ATOM	6776	CG	TYR	C	671	110.104	119.804	96.129	1.00	20.00	C
ATOM	6777	CD1	TYR	C	671	110.479	118.445	96.157	1.00	20.00	C
ATOM	6778	CD2	TYR	C	671	111.064	120.786	95.807	1.00	20.00	C
ATOM	6779	CE1	TYR	C	671	111.803	118.065	95.870	1.00	20.00	C
ATOM	6780	CE2	TYR	C	671	112.391	120.407	95.524	1.00	20.00	C
ATOM	6781	CZ	TYR	C	671	112.762	119.048	95.558	1.00	20.00	C
ATOM	6782	OH	TYR	C	671	114.050	118.687	95.289	1.00	20.00	O
ATOM	6783	H	TYR	C	671	106.511	120.990	97.557	1.00	0.00	H
ATOM	6784	HA	TYR	C	671	108.349	118.818	98.076	1.00	0.00	H
ATOM	6785	HB2	TYR	C	671	108.008	119.672	95.811	1.00	0.00	H
ATOM	6786	HB3	TYR	C	671	108.548	121.261	96.279	1.00	0.00	H
ATOM	6787	HD1	TYR	C	671	109.751	117.690	96.406	1.00	0.00	H
ATOM	6788	HD2	TYR	C	671	110.791	121.830	95.786	1.00	0.00	H
ATOM	6789	HE1	TYR	C	671	112.076	117.020	95.894	1.00	0.00	H
ATOM	6790	HE2	TYR	C	671	113.125	121.162	95.282	1.00	0.00	H
ATOM	6791	HH	TYR	C	671	114.196	117.740	95.342	1.00	0.00	H
ATOM	6792	N	ILE	C	672	109.044	121.792	99.321	1.00	0.00	N
ATOM	6793	CA	ILE	C	672	109.874	122.531	100.276	1.00	0.00	C
ATOM	6794	C	ILE	C	672	109.189	122.684	101.652	1.00	0.00	C
ATOM	6795	O	ILE	C	672	109.778	123.312	102.532	1.00	0.00	O
ATOM	6796	CB	ILE	C	672	110.260	123.937	99.724	1.00	20.00	C
ATOM	6797	CG1	ILE	C	672	109.076	124.925	99.576	1.00	20.00	C
ATOM	6798	CG2	ILE	C	672	111.015	123.798	98.388	1.00	20.00	C
ATOM	6799	CD1	ILE	C	672	108.844	125.860	100.774	1.00	20.00	C
ATOM	6800	H	ILE	C	672	108.272	122.299	98.911	1.00	0.00	H
ATOM	6801	HA	ILE	C	672	110.803	121.990	100.464	1.00	0.00	H
ATOM	6802	HB	ILE	C	672	110.982	124.374	100.415	1.00	0.00	H
ATOM	6803	HG12	ILE	C	672	108.167	124.362	99.372	1.00	0.00	H
ATOM	6804	HG13	ILE	C	672	109.231	125.553	98.698	1.00	0.00	H

```
ATOM   6805 HG21 ILE C 672     111.428 124.755  98.069  1.00  0.00           H
ATOM   6806 HG22 ILE C 672     111.846 123.097  98.474  1.00  0.00           H
ATOM   6807 HG23 ILE C 672     110.361 123.442  97.591  1.00  0.00           H
ATOM   6808 HD11 ILE C 672     108.428 126.813 100.446  1.00  0.00           H
ATOM   6809 HD12 ILE C 672     108.136 125.429 101.481  1.00  0.00           H
ATOM   6810 HD13 ILE C 672     109.766 126.076 101.314  1.00  0.00           H
ATOM   6811  N   LEU C 673     107.985 122.104 101.825  1.00 30.00           N
ATOM   6812  CA  LEU C 673     107.216 122.174 103.065  1.00 30.00           C
ATOM   6813  C   LEU C 673     106.903 120.764 103.584  1.00 30.00           C
ATOM   6814  O   LEU C 673     107.497 120.405 104.594  1.00 30.00           O
ATOM   6815  CB  LEU C 673     106.014 123.128 102.895  1.00 20.00           C
ATOM   6816  CG  LEU C 673     105.273 123.530 104.195  1.00 20.00           C
ATOM   6817  CD1 LEU C 673     104.571 124.899 104.025  1.00 20.00           C
ATOM   6818  CD2 LEU C 673     104.300 122.441 104.696  1.00 20.00           C
ATOM   6819  H   LEU C 673     107.569 121.588 101.064  1.00  0.00           H
ATOM   6820  HA  LEU C 673     107.837 122.627 103.840  1.00  0.00           H
ATOM   6821  HB2 LEU C 673     106.430 124.041 102.470  1.00  0.00           H
ATOM   6822  HB3 LEU C 673     105.318 122.754 102.150  1.00  0.00           H
ATOM   6823  HG  LEU C 673     106.024 123.673 104.973  1.00  0.00           H
ATOM   6824 HD11 LEU C 673     103.524 124.885 104.325  1.00  0.00           H
ATOM   6825 HD12 LEU C 673     105.064 125.660 104.630  1.00  0.00           H
ATOM   6826 HD13 LEU C 673     104.590 125.249 102.992  1.00  0.00           H
ATOM   6827 HD21 LEU C 673     104.730 121.896 105.536  1.00  0.00           H
ATOM   6828 HD22 LEU C 673     103.345 122.843 105.036  1.00  0.00           H
ATOM   6829 HD23 LEU C 673     104.073 121.714 103.919  1.00  0.00           H
ATOM   6830  N   LEU C 674     106.024 119.979 102.925  1.00 30.00           N
ATOM   6831  CA  LEU C 674     105.615 118.645 103.412  1.00 30.00           C
ATOM   6832  C   LEU C 674     106.737 117.594 103.441  1.00 30.00           C
ATOM   6833  O   LEU C 674     106.755 116.786 104.367  1.00 30.00           O
ATOM   6834  CB  LEU C 674     104.431 118.079 102.602  1.00 20.00           C
ATOM   6835  CG  LEU C 674     103.089 118.789 102.851  1.00 20.00           C
ATOM   6836  CD1 LEU C 674     102.071 118.426 101.751  1.00 20.00           C
ATOM   6837  CD2 LEU C 674     102.550 118.543 104.276  1.00 20.00           C
ATOM   6838  H   LEU C 674     105.560 120.319 102.095  1.00  0.00           H
ATOM   6839  HA  LEU C 674     105.287 118.775 104.444  1.00  0.00           H
ATOM   6840  HB2 LEU C 674     104.691 118.104 101.546  1.00  0.00           H
ATOM   6841  HB3 LEU C 674     104.290 117.020 102.827  1.00  0.00           H
ATOM   6842  HG  LEU C 674     103.267 119.857 102.773  1.00  0.00           H
ATOM   6843 HD11 LEU C 674     101.230 117.854 102.134  1.00  0.00           H
ATOM   6844 HD12 LEU C 674     101.671 119.328 101.286  1.00  0.00           H
ATOM   6845 HD13 LEU C 674     102.513 117.828 100.954  1.00  0.00           H
ATOM   6846 HD21 LEU C 674     101.492 118.287 104.287  1.00  0.00           H
ATOM   6847 HD22 LEU C 674     103.082 117.739 104.783  1.00  0.00           H
ATOM   6848 HD23 LEU C 674     102.662 119.440 104.886  1.00  0.00           H
ATOM   6849  N   LEU C 675     107.643 117.616 102.450  1.00  0.00           N
ATOM   6850  CA  LEU C 675     108.795 116.711 102.369  1.00  0.00           C
ATOM   6851  C   LEU C 675     109.843 117.016 103.459  1.00  0.00           C
ATOM   6852  O   LEU C 675     110.453 116.080 103.974  1.00  0.00           O
ATOM   6853  CB  LEU C 675     109.413 116.776 100.951  1.00 20.00           C
ATOM   6854  CG  LEU C 675     108.950 115.680  99.959  1.00 20.00           C
ATOM   6855  CD1 LEU C 675     107.436 115.697  99.654  1.00 20.00           C
ATOM   6856  CD2 LEU C 675     109.797 115.709  98.668  1.00 20.00           C
ATOM   6857  H   LEU C 675     107.542 118.285 101.698  1.00  0.00           H
ATOM   6858  HA  LEU C 675     108.442 115.693 102.549  1.00  0.00           H
ATOM   6859  HB2 LEU C 675     109.239 117.759 100.516  1.00  0.00           H
ATOM   6860  HB3 LEU C 675     110.498 116.689 101.033  1.00  0.00           H
ATOM   6861  HG  LEU C 675     109.158 114.726 100.446  1.00  0.00           H
ATOM   6862 HD11 LEU C 675     107.044 114.680  99.610  1.00  0.00           H
ATOM   6863 HD12 LEU C 675     106.856 116.222 100.409  1.00  0.00           H
ATOM   6864 HD13 LEU C 675     107.209 116.172  98.700  1.00  0.00           H
ATOM   6865 HD21 LEU C 675     110.323 114.764  98.528  1.00  0.00           H
ATOM   6866 HD22 LEU C 675     109.196 115.874  97.775  1.00  0.00           H
ATOM   6867 HD23 LEU C 675     110.551 116.497  98.688  1.00  0.00           H
ATOM   6868  N   ASN C 676     109.995 118.304 103.819  1.00  0.00           N
ATOM   6869  CA  ASN C 676     110.856 118.771 104.912  1.00  0.00           C
ATOM   6870  C   ASN C 676     110.197 118.570 106.289  1.00  0.00           C
ATOM   6871  O   ASN C 676     110.912 118.324 107.259  1.00  0.00           O
ATOM   6872  CB  ASN C 676     111.223 120.257 104.682  1.00 20.00           C
ATOM   6873  CG  ASN C 676     112.125 120.513 103.465  1.00 20.00           C
ATOM   6874  ND2 ASN C 676     112.362 121.787 103.152  1.00 20.00           N
ATOM   6875  OD1 ASN C 676     112.617 119.589 102.821  1.00 20.00           O
```

```
ATOM   6876  H   ASN C 676     109.452 119.013 103.348  1.00  0.00           H
ATOM   6877  HA  ASN C 676     111.771 118.177 104.884  1.00  0.00           H
ATOM   6878  HB2 ASN C 676     111.761 120.635 105.553  1.00  0.00           H
ATOM   6879  HB3 ASN C 676     110.318 120.861 104.592  1.00  0.00           H
ATOM   6880 HD21 ASN C 676     112.958 122.011 102.369  1.00  0.00           H
ATOM   6881 HD22 ASN C 676     111.929 122.535 103.676  1.00  0.00           H
ATOM   6882  N   MET C 677     108.855 118.635 106.347  1.00  0.00           N
ATOM   6883  CA  MET C 677     108.045 118.347 107.531  1.00  0.00           C
ATOM   6884  C   MET C 677     108.050 116.851 107.880  1.00  0.00           C
ATOM   6885  O   MET C 677     108.008 116.518 109.061  1.00  0.00           O
ATOM   6886  CB  MET C 677     106.605 118.864 107.311  1.00 20.00           C
ATOM   6887  CG  MET C 677     105.762 118.999 108.588  1.00 20.00           C
ATOM   6888  SD  MET C 677     106.336 120.248 109.774  1.00 20.00           S
ATOM   6889  CE  MET C 677     106.124 121.765 108.800  1.00 20.00           C
ATOM   6890  H   MET C 677     108.340 118.889 105.514  1.00  0.00           H
ATOM   6891  HA  MET C 677     108.492 118.893 108.363  1.00  0.00           H
ATOM   6892  HB2 MET C 677     106.633 119.840 106.835  1.00  0.00           H
ATOM   6893  HB3 MET C 677     106.081 118.215 106.609  1.00  0.00           H
ATOM   6894  HG2 MET C 677     105.713 118.041 109.102  1.00  0.00           H
ATOM   6895  HG3 MET C 677     104.735 119.245 108.316  1.00  0.00           H
ATOM   6896  HE1 MET C 677     106.293 122.638 109.430  1.00  0.00           H
ATOM   6897  HE2 MET C 677     106.834 121.802 107.975  1.00  0.00           H
ATOM   6898  HE3 MET C 677     105.113 121.823 108.397  1.00  0.00           H
ATOM   6899  N   LEU C 678     108.137 115.984 106.854  1.00  0.00           N
ATOM   6900  CA  LEU C 678     108.271 114.538 107.004  1.00  0.00           C
ATOM   6901  C   LEU C 678     109.628 114.145 107.610  1.00  0.00           C
ATOM   6902  O   LEU C 678     109.647 113.267 108.465  1.00  0.00           O
ATOM   6903  CB  LEU C 678     108.009 113.839 105.650  1.00 20.00           C
ATOM   6904  CG  LEU C 678     107.949 112.295 105.727  1.00 20.00           C
ATOM   6905  CD1 LEU C 678     106.821 111.796 106.653  1.00 20.00           C
ATOM   6906  CD2 LEU C 678     107.871 111.658 104.330  1.00 20.00           C
ATOM   6907  H   LEU C 678     108.134 116.335 105.906  1.00  0.00           H
ATOM   6908  HA  LEU C 678     107.497 114.225 107.707  1.00  0.00           H
ATOM   6909  HB2 LEU C 678     107.070 114.203 105.233  1.00  0.00           H
ATOM   6910  HB3 LEU C 678     108.784 114.137 104.942  1.00  0.00           H
ATOM   6911  HG  LEU C 678     108.891 111.947 106.149  1.00  0.00           H
ATOM   6912 HD11 LEU C 678     106.321 110.911 106.260  1.00  0.00           H
ATOM   6913 HD12 LEU C 678     107.222 111.530 107.631  1.00  0.00           H
ATOM   6914 HD13 LEU C 678     106.056 112.556 106.812  1.00  0.00           H
ATOM   6915 HD21 LEU C 678     108.585 110.840 104.260  1.00  0.00           H
ATOM   6916 HD22 LEU C 678     106.891 111.236 104.117  1.00  0.00           H
ATOM   6917 HD23 LEU C 678     108.098 112.367 103.533  1.00  0.00           H
ATOM   6918  N   ILE C 679     110.716 114.824 107.205  1.00  0.00           N
ATOM   6919  CA  ILE C 679     112.056 114.666 107.784  1.00  0.00           C
ATOM   6920  C   ILE C 679     112.103 115.089 109.269  1.00  0.00           C
ATOM   6921  O   ILE C 679     112.731 114.396 110.069  1.00  0.00           O
ATOM   6922  CB  ILE C 679     113.127 115.469 106.978  1.00 20.00           C
ATOM   6923  CG1 ILE C 679     113.293 114.886 105.558  1.00 20.00           C
ATOM   6924  CG2 ILE C 679     114.514 115.579 107.650  1.00 20.00           C
ATOM   6925  CD1 ILE C 679     113.972 115.833 104.557  1.00 20.00           C
ATOM   6926  H   ILE C 679     110.621 115.532 106.490  1.00  0.00           H
ATOM   6927  HA  ILE C 679     112.314 113.605 107.734  1.00  0.00           H
ATOM   6928  HB  ILE C 679     112.749 116.485 106.865  1.00  0.00           H
ATOM   6929 HG12 ILE C 679     113.862 113.958 105.617  1.00  0.00           H
ATOM   6930 HG13 ILE C 679     112.323 114.610 105.149  1.00  0.00           H
ATOM   6931 HG21 ILE C 679     115.221 116.105 107.009  1.00  0.00           H
ATOM   6932 HG22 ILE C 679     114.484 116.136 108.586  1.00  0.00           H
ATOM   6933 HG23 ILE C 679     114.927 114.591 107.859  1.00  0.00           H
ATOM   6934 HD11 ILE C 679     113.616 115.642 103.544  1.00  0.00           H
ATOM   6935 HD12 ILE C 679     113.771 116.880 104.783  1.00  0.00           H
ATOM   6936 HD13 ILE C 679     115.053 115.696 104.556  1.00  0.00           H
ATOM   6937  N   ALA C 680     111.408 116.192 109.604  1.00  0.00           N
ATOM   6938  CA  ALA C 680     111.290 116.728 110.959  1.00  0.00           C
ATOM   6939  C   ALA C 680     110.468 115.830 111.900  1.00  0.00           C
ATOM   6940  O   ALA C 680     110.893 115.616 113.034  1.00  0.00           O
ATOM   6941  CB  ALA C 680     110.697 118.143 110.893  1.00 20.00           C
ATOM   6942  H   ALA C 680     110.925 116.707 108.881  1.00  0.00           H
ATOM   6943  HA  ALA C 680     112.298 116.807 111.372  1.00  0.00           H
ATOM   6944  HB1 ALA C 680     110.642 118.595 111.884  1.00  0.00           H
ATOM   6945  HB2 ALA C 680     111.310 118.796 110.270  1.00  0.00           H
ATOM   6946  HB3 ALA C 680     109.690 118.140 110.475  1.00  0.00           H
```

```
ATOM   6947  N   LEU C 681     109.327 115.310 111.412  1.00  0.00           N
ATOM   6948  CA  LEU C 681     108.438 114.418 112.162  1.00  0.00           C
ATOM   6949  C   LEU C 681     108.977 112.982 112.272  1.00  0.00           C
ATOM   6950  O   LEU C 681     108.725 112.344 113.293  1.00  0.00           O
ATOM   6951  CB  LEU C 681     107.019 114.446 111.554  1.00 20.00           C
ATOM   6952  CG  LEU C 681     106.242 115.756 111.829  1.00 20.00           C
ATOM   6953  CD1 LEU C 681     104.921 115.794 111.038  1.00 20.00           C
ATOM   6954  CD2 LEU C 681     106.004 116.006 113.334  1.00 20.00           C
ATOM   6955  H   LEU C 681     109.031 115.549 110.475  1.00  0.00           H
ATOM   6956  HA  LEU C 681     108.381 114.793 113.183  1.00  0.00           H
ATOM   6957  HB2 LEU C 681     107.092 114.268 110.480  1.00  0.00           H
ATOM   6958  HB3 LEU C 681     106.431 113.615 111.950  1.00  0.00           H
ATOM   6959  HG  LEU C 681     106.849 116.582 111.456  1.00  0.00           H
ATOM   6960 HD11 LEU C 681     104.677 116.812 110.739  1.00  0.00           H
ATOM   6961 HD12 LEU C 681     104.971 115.190 110.132  1.00  0.00           H
ATOM   6962 HD13 LEU C 681     104.084 115.419 111.628  1.00  0.00           H
ATOM   6963 HD21 LEU C 681     106.665 116.790 113.704  1.00  0.00           H
ATOM   6964 HD22 LEU C 681     104.983 116.325 113.545  1.00  0.00           H
ATOM   6965 HD23 LEU C 681     106.189 115.114 113.933  1.00  0.00           H
ATOM   6966  N   MET C 682     109.756 112.516 111.277  1.00  0.00           N
ATOM   6967  CA  MET C 682     110.544 111.284 111.374  1.00  0.00           C
ATOM   6968  C   MET C 682     111.646 111.406 112.435  1.00  0.00           C
ATOM   6969  O   MET C 682     111.812 110.464 113.198  1.00  0.00           O
ATOM   6970  CB  MET C 682     111.143 110.885 110.008  1.00 20.00           C
ATOM   6971  CG  MET C 682     110.128 110.201 109.081  1.00 20.00           C
ATOM   6972  SD  MET C 682     110.726 109.938 107.391  1.00 20.00           S
ATOM   6973  CE  MET C 682     109.373 108.909 106.761  1.00 20.00           C
ATOM   6974  H   MET C 682     109.895 113.071 110.443  1.00  0.00           H
ATOM   6975  HA  MET C 682     109.878 110.479 111.696  1.00  0.00           H
ATOM   6976  HB2 MET C 682     111.587 111.752 109.517  1.00  0.00           H
ATOM   6977  HB3 MET C 682     111.965 110.183 110.160  1.00  0.00           H
ATOM   6978  HG2 MET C 682     109.857 109.233 109.502  1.00  0.00           H
ATOM   6979  HG3 MET C 682     109.205 110.777 109.030  1.00  0.00           H
ATOM   6980  HE1 MET C 682     109.503 108.724 105.696  1.00  0.00           H
ATOM   6981  HE2 MET C 682     109.347 107.951 107.280  1.00  0.00           H
ATOM   6982  HE3 MET C 682     108.414 109.404 106.908  1.00  0.00           H
ATOM   6983  N   GLY C 683     112.334 112.561 112.499  1.00  0.00           N
ATOM   6984  CA  GLY C 683     113.386 112.851 113.477  1.00  0.00           C
ATOM   6985  C   GLY C 683     112.841 112.934 114.912  1.00  0.00           C
ATOM   6986  O   GLY C 683     113.486 112.428 115.829  1.00  0.00           O
ATOM   6987  H   GLY C 683     112.131 113.289 111.826  1.00  0.00           H
ATOM   6988  HA2 GLY C 683     114.170 112.096 113.426  1.00  0.00           H
ATOM   6989  HA3 GLY C 683     113.846 113.805 113.221  1.00  0.00           H
ATOM   6990  N   GLU C 684     111.650 113.531 115.094  1.00  0.00           N
ATOM   6991  CA  GLU C 684     110.952 113.659 116.375  1.00  0.00           C
ATOM   6992  C   GLU C 684     110.499 112.302 116.942  1.00  0.00           C
ATOM   6993  O   GLU C 684     110.796 112.005 118.101  1.00  0.00           O
ATOM   6994  CB  GLU C 684     109.832 114.722 116.226  1.00 20.00           C
ATOM   6995  CG  GLU C 684     108.854 114.913 117.411  1.00  0.00           C
ATOM   6996  CD  GLU C 684     107.660 113.953 117.410  1.00  0.00           C
ATOM   6997  OE1 GLU C 684     106.917 113.968 116.404  1.00  0.00           O
ATOM   6998  OE2 GLU C 684     107.505 113.227 118.416  1.00  0.00           O1-
ATOM   6999  H   GLU C 684     111.182 113.942 114.297  1.00  0.00           H
ATOM   7000  HA  GLU C 684     111.671 114.062 117.091  1.00  0.00           H
ATOM   7001  HB2 GLU C 684     110.312 115.683 116.041  1.00  0.00           H
ATOM   7002  HB3 GLU C 684     109.264 114.519 115.318  1.00  0.00           H
ATOM   7003  HG2 GLU C 684     109.390 114.848 118.359  1.00  0.00           H
ATOM   7004  HG3 GLU C 684     108.441 115.921 117.368  1.00  0.00           H
ATOM   7005  N   THR C 685     109.827 111.493 116.105  1.00  0.00           N
ATOM   7006  CA  THR C 685     109.361 110.154 116.469  1.00  0.00           C
ATOM   7007  C   THR C 685     110.490 109.099 116.511  1.00  0.00           C
ATOM   7008  O   THR C 685     110.299 108.093 117.192  1.00  0.00           O
ATOM   7009  CB  THR C 685     108.238 109.647 115.525  1.00 20.00           C
ATOM   7010  CG2 THR C 685     106.989 110.541 115.534  1.00 20.00           C
ATOM   7011  OG1 THR C 685     108.685 109.492 114.192  1.00 20.00           O
ATOM   7012  H   THR C 685     109.611 111.807 115.168  1.00  0.00           H
ATOM   7013  HA  THR C 685     108.939 110.211 117.475  1.00  0.00           H
ATOM   7014  HB  THR C 685     107.924 108.656 115.858  1.00  0.00           H
ATOM   7015  HG1 THR C 685     108.677 110.352 113.761  1.00  0.00           H
ATOM   7016 HG21 THR C 685     106.218 110.147 114.872  1.00  0.00           H
ATOM   7017 HG22 THR C 685     106.564 110.603 116.536  1.00  0.00           H
```

```
ATOM   7018 HG23 THR C 685     107.209 111.558 115.214  1.00  0.00           H
ATOM   7019  N   VAL C 686     111.650 109.349 115.866  1.00  0.00           N
ATOM   7020  CA  VAL C 686     112.864 108.522 115.970  1.00  0.00           C
ATOM   7021  C   VAL C 686     113.407 108.450 117.409  1.00  0.00           C
ATOM   7022  O   VAL C 686     113.838 107.374 117.812  1.00  0.00           O
ATOM   7023  CB  VAL C 686     114.004 108.969 114.980  1.00 20.00           C
ATOM   7024  CG1 VAL C 686     115.460 108.963 115.500  1.00 20.00           C
ATOM   7025  CG2 VAL C 686     113.964 108.166 113.668  1.00 20.00           C
ATOM   7026  H   VAL C 686     111.729 110.179 115.294  1.00  0.00           H
ATOM   7027  HA  VAL C 686     112.563 107.507 115.703  1.00  0.00           H
ATOM   7028  HB  VAL C 686     113.815 110.007 114.712  1.00  0.00           H
ATOM   7029 HG11 VAL C 686     116.158 109.257 114.715  1.00  0.00           H
ATOM   7030 HG12 VAL C 686     115.595 109.672 116.316  1.00  0.00           H
ATOM   7031 HG13 VAL C 686     115.764 107.975 115.848  1.00  0.00           H
ATOM   7032 HG21 VAL C 686     114.764 108.464 112.992  1.00  0.00           H
ATOM   7033 HG22 VAL C 686     114.070 107.096 113.852  1.00  0.00           H
ATOM   7034 HG23 VAL C 686     113.033 108.318 113.132  1.00  0.00           H
ATOM   7035  N   ASN C 687     113.361 109.573 118.150  1.00  0.00           N
ATOM   7036  CA  ASN C 687     113.839 109.663 119.536  1.00  0.00           C
ATOM   7037  C   ASN C 687     112.929 108.919 120.530  1.00  0.00           C
ATOM   7038  O   ASN C 687     113.444 108.363 121.499  1.00  0.00           O
ATOM   7039  CB  ASN C 687     114.001 111.141 119.961  1.00 20.00           C
ATOM   7040  CG  ASN C 687     114.977 111.950 119.099  1.00 20.00           C
ATOM   7041  ND2 ASN C 687     116.213 111.471 118.938  1.00 20.00           N
ATOM   7042  OD1 ASN C 687     114.621 113.013 118.596  1.00 20.00           O
ATOM   7043  H   ASN C 687     112.990 110.422 117.746  1.00  0.00           H
ATOM   7044  HA  ASN C 687     114.818 109.182 119.580  1.00  0.00           H
ATOM   7045  HB2 ASN C 687     113.029 111.639 119.947  1.00  0.00           H
ATOM   7046  HB3 ASN C 687     114.355 111.195 120.992  1.00  0.00           H
ATOM   7047 HD21 ASN C 687     116.881 111.985 118.382  1.00  0.00           H
ATOM   7048 HD22 ASN C 687     116.490 110.600 119.366  1.00  0.00           H
ATOM   7049  N   LYS C 688     111.611 108.893 120.260  1.00  0.00           N
ATOM   7050  CA  LYS C 688     110.625 108.118 121.016  1.00  0.00           C
ATOM   7051  C   LYS C 688     110.767 106.607 120.758  1.00  0.00           C
ATOM   7052  O   LYS C 688     110.755 105.823 121.707  1.00  0.00           O
ATOM   7053  CB  LYS C 688     109.211 108.644 120.678  1.00 20.00           C
ATOM   7054  CG  LYS C 688     108.059 107.895 121.375  1.00 20.00           C
ATOM   7055  CD  LYS C 688     106.687 108.520 121.084  1.00 20.00           C
ATOM   7056  CE  LYS C 688     105.544 107.754 121.764  1.00 20.00           C
ATOM   7057  NZ  LYS C 688     104.241 108.382 121.485  1.00 20.00           N1+
ATOM   7058  H   LYS C 688     111.266 109.382 119.446  1.00  0.00           H
ATOM   7059  HA  LYS C 688     110.802 108.294 122.079  1.00  0.00           H
ATOM   7060  HB2 LYS C 688     109.159 109.700 120.944  1.00  0.00           H
ATOM   7061  HB3 LYS C 688     109.052 108.600 119.599  1.00  0.00           H
ATOM   7062  HG2 LYS C 688     108.039 106.853 121.055  1.00  0.00           H
ATOM   7063  HG3 LYS C 688     108.234 107.883 122.452  1.00  0.00           H
ATOM   7064  HD2 LYS C 688     106.685 109.558 121.419  1.00  0.00           H
ATOM   7065  HD3 LYS C 688     106.523 108.545 120.006  1.00  0.00           H
ATOM   7066  HE2 LYS C 688     105.519 106.721 121.416  1.00  0.00           H
ATOM   7067  HE3 LYS C 688     105.695 107.728 122.844  1.00  0.00           H
ATOM   7068  HZ1 LYS C 688     104.076 108.388 120.488  1.00  0.00           H
ATOM   7069  HZ2 LYS C 688     103.509 107.859 121.944  1.00  0.00           H
ATOM   7070  HZ3 LYS C 688     104.244 109.331 121.830  1.00  0.00           H
ATOM   7071  N   ILE C 689     110.906 106.242 119.474  1.00  0.00           N
ATOM   7072  CA  ILE C 689     111.013 104.867 118.989  1.00  0.00           C
ATOM   7073  C   ILE C 689     112.415 104.243 119.207  1.00  0.00           C
ATOM   7074  O   ILE C 689     112.530 103.022 119.127  1.00  0.00           O
ATOM   7075  CB  ILE C 689     110.541 104.787 117.496  1.00 20.00           C
ATOM   7076  CG1 ILE C 689     109.006 105.007 117.427  1.00 20.00           C
ATOM   7077  CG2 ILE C 689     110.913 103.508 116.714  1.00 20.00           C
ATOM   7078  CD1 ILE C 689     108.465 105.391 116.042  1.00 20.00           C
ATOM   7079  H   ILE C 689     110.896 106.957 118.757  1.00  0.00           H
ATOM   7080  HA  ILE C 689     110.318 104.268 119.577  1.00  0.00           H
ATOM   7081  HB  ILE C 689     111.018 105.612 116.965  1.00  0.00           H
ATOM   7082 HG12 ILE C 689     108.494 104.111 117.778  1.00  0.00           H
ATOM   7083 HG13 ILE C 689     108.708 105.797 118.118  1.00  0.00           H
ATOM   7084 HG21 ILE C 689     110.534 103.534 115.693  1.00  0.00           H
ATOM   7085 HG22 ILE C 689     111.992 103.385 116.626  1.00  0.00           H
ATOM   7086 HG23 ILE C 689     110.501 102.621 117.194  1.00  0.00           H
ATOM   7087 HD11 ILE C 689     107.410 105.661 116.105  1.00  0.00           H
ATOM   7088 HD12 ILE C 689     108.993 106.253 115.637  1.00  0.00           H
```

```
ATOM   7089 HD13 ILE C 689     108.546 104.572 115.327  1.00  0.00           H
ATOM   7090  N   ALA C 690     113.438 105.061 119.525  1.00 30.00           N
ATOM   7091  CA  ALA C 690     114.844 104.677 119.718  1.00 30.00           C
ATOM   7092  C   ALA C 690     115.072 103.498 120.678  1.00 30.00           C
ATOM   7093  O   ALA C 690     115.777 102.557 120.311  1.00 30.00           O
ATOM   7094  CB  ALA C 690     115.662 105.897 120.176  1.00 20.00           C
ATOM   7095  H   ALA C 690     113.257 106.054 119.571  1.00  0.00           H
ATOM   7096  HA  ALA C 690     115.225 104.375 118.743  1.00  0.00           H
ATOM   7097  HB1 ALA C 690     116.699 105.625 120.379  1.00  0.00           H
ATOM   7098  HB2 ALA C 690     115.686 106.674 119.414  1.00  0.00           H
ATOM   7099  HB3 ALA C 690     115.252 106.335 121.087  1.00  0.00           H
ATOM   7100  N   GLN C 691     114.443 103.558 121.863  1.00 30.00           N
ATOM   7101  CA  GLN C 691     114.486 102.493 122.862  1.00 30.00           C
ATOM   7102  C   GLN C 691     113.385 101.441 122.632  1.00 30.00           C
ATOM   7103  O   GLN C 691     113.636 100.264 122.877  1.00 30.00           O
ATOM   7104  CB  GLN C 691     114.421 103.124 124.274  1.00 20.00           C
ATOM   7105  CG  GLN C 691     114.476 102.141 125.465  1.00 20.00           C
ATOM   7106  CD  GLN C 691     115.718 101.242 125.463  1.00 20.00           C
ATOM   7107  NE2 GLN C 691     115.513  99.925 125.504  1.00 20.00           N
ATOM   7108  OE1 GLN C 691     116.846 101.730 125.436  1.00 20.00           O
ATOM   7109  H   GLN C 691     113.877 104.365 122.086  1.00  0.00           H
ATOM   7110  HA  GLN C 691     115.445 101.978 122.773  1.00  0.00           H
ATOM   7111  HB2 GLN C 691     115.244 103.832 124.376  1.00  0.00           H
ATOM   7112  HB3 GLN C 691     113.512 103.721 124.361  1.00  0.00           H
ATOM   7113  HG2 GLN C 691     113.575 101.526 125.486  1.00  0.00           H
ATOM   7114  HG3 GLN C 691     114.473 102.705 126.398  1.00  0.00           H
ATOM   7115 HE21 GLN C 691     116.295  99.288 125.520  1.00  0.00           H
ATOM   7116 HE22 GLN C 691     114.569  99.559 125.495  1.00  0.00           H
ATOM   7117  N   GLU C 692     112.200 101.865 122.155  1.00 30.00           N
ATOM   7118  CA  GLU C 692     111.039 100.996 121.936  1.00 30.00           C
ATOM   7119  C   GLU C 692     111.281  99.915 120.863  1.00 30.00           C
ATOM   7120  O   GLU C 692     110.906  98.765 121.084  1.00 30.00           O
ATOM   7121  CB  GLU C 692     109.808 101.873 121.630  1.00 20.00           C
ATOM   7122  CG  GLU C 692     108.468 101.109 121.565  1.00  0.00           C
ATOM   7123  CD  GLU C 692     107.254 101.994 121.258  1.00  0.00           C
ATOM   7124  OE1 GLU C 692     107.435 103.221 121.084  1.00  0.00           O
ATOM   7125  OE2 GLU C 692     106.148 101.415 121.192  1.00  0.00           O1-
ATOM   7126  H   GLU C 692     112.065 102.846 121.958  1.00  0.00           H
ATOM   7127  HA  GLU C 692     110.850 100.480 122.880  1.00  0.00           H
ATOM   7128  HB2 GLU C 692     109.735 102.661 122.382  1.00  0.00           H
ATOM   7129  HB3 GLU C 692     109.970 102.380 120.679  1.00  0.00           H
ATOM   7130  HG2 GLU C 692     108.507 100.335 120.799  1.00  0.00           H
ATOM   7131  HG3 GLU C 692     108.288 100.601 122.513  1.00  0.00           H
ATOM   7132  N   SER C 693     111.951 100.288 119.757  1.00 30.00           N
ATOM   7133  CA  SER C 693     112.386  99.398 118.673  1.00 30.00           C
ATOM   7134  C   SER C 693     113.410  98.330 119.103  1.00 30.00           C
ATOM   7135  O   SER C 693     113.416  97.248 118.517  1.00 30.00           O
ATOM   7136  CB  SER C 693     112.908 100.235 117.486  1.00 20.00           C
ATOM   7137  OG  SER C 693     114.093 100.937 117.802  1.00 20.00           O
ATOM   7138  H   SER C 693     112.217 101.260 119.656  1.00  0.00           H
ATOM   7139  HA  SER C 693     111.497  98.870 118.329  1.00  0.00           H
ATOM   7140  HB2 SER C 693     112.150 100.937 117.146  1.00  0.00           H
ATOM   7141  HB3 SER C 693     113.132  99.588 116.638  1.00  0.00           H
ATOM   7142  HG  SER C 693     113.864 101.690 118.355  1.00  0.00           H
ATOM   7143  N   LYS C 694     114.229  98.640 120.125  1.00 30.00           N
ATOM   7144  CA  LYS C 694     115.166  97.705 120.744  1.00 30.00           C
ATOM   7145  C   LYS C 694     114.444  96.622 121.564  1.00 30.00           C
ATOM   7146  O   LYS C 694     114.843  95.462 121.485  1.00 30.00           O
ATOM   7147  CB  LYS C 694     116.221  98.493 121.550  1.00 20.00           C
ATOM   7148  CG  LYS C 694     117.277  97.638 122.273  1.00 20.00           C
ATOM   7149  CD  LYS C 694     118.365  98.508 122.924  1.00 20.00           C
ATOM   7150  CE  LYS C 694     119.499  97.701 123.571  1.00 20.00           C
ATOM   7151  NZ  LYS C 694     120.333  97.025 122.562  1.00 20.00           N1+
ATOM   7152  H   LYS C 694     114.162  99.553 120.553  1.00  0.00           H
ATOM   7153  HA  LYS C 694     115.695  97.195 119.939  1.00  0.00           H
ATOM   7154  HB2 LYS C 694     116.728  99.177 120.868  1.00  0.00           H
ATOM   7155  HB3 LYS C 694     115.736  99.122 122.292  1.00  0.00           H
ATOM   7156  HG2 LYS C 694     116.800  97.036 123.048  1.00  0.00           H
ATOM   7157  HG3 LYS C 694     117.720  96.933 121.571  1.00  0.00           H
ATOM   7158  HD2 LYS C 694     118.782  99.192 122.183  1.00  0.00           H
ATOM   7159  HD3 LYS C 694     117.905  99.141 123.684  1.00  0.00           H
```

ATOM	7160	HE2	LYS	C	694	119.098	96.964	124.268	1.00	0.00	H
ATOM	7161	HE3	LYS	C	694	120.140	98.369	124.146	1.00	0.00	H
ATOM	7162	HZ1	LYS	C	694	119.777	96.363	122.039	1.00	0.00	H
ATOM	7163	HZ2	LYS	C	694	120.719	97.710	121.929	1.00	0.00	H
ATOM	7164	HZ3	LYS	C	694	121.087	96.531	123.019	1.00	0.00	H
ATOM	7165	N	ASN	C	695	113.379	97.006	122.291	1.00	30.00	N
ATOM	7166	CA	ASN	C	695	112.525	96.089	123.055	1.00	30.00	C
ATOM	7167	C	ASN	C	695	111.612	95.237	122.147	1.00	30.00	C
ATOM	7168	O	ASN	C	695	111.354	94.082	122.488	1.00	30.00	O
ATOM	7169	CB	ASN	C	695	111.700	96.873	124.104	1.00	20.00	C
ATOM	7170	CG	ASN	C	695	112.544	97.599	125.154	1.00	20.00	C
ATOM	7171	ND2	ASN	C	695	113.167	96.844	126.061	1.00	20.00	N
ATOM	7172	OD1	ASN	C	695	112.619	98.824	125.163	1.00	20.00	O
ATOM	7173	H	ASN	C	695	113.110	97.981	122.302	1.00	0.00	H
ATOM	7174	HA	ASN	C	695	113.183	95.400	123.589	1.00	0.00	H
ATOM	7175	HB2	ASN	C	695	111.036	97.589	123.616	1.00	0.00	H
ATOM	7176	HB3	ASN	C	695	111.065	96.191	124.665	1.00	0.00	H
ATOM	7177	HD21	ASN	C	695	113.701	97.278	126.799	1.00	0.00	H
ATOM	7178	HD22	ASN	C	695	113.043	95.842	126.061	1.00	0.00	H
ATOM	7179	N	ILE	C	696	111.174	95.788	120.999	1.00	30.00	N
ATOM	7180	CA	ILE	C	696	110.436	95.059	119.960	1.00	30.00	C
ATOM	7181	C	ILE	C	696	111.315	94.001	119.260	1.00	30.00	C
ATOM	7182	O	ILE	C	696	110.818	92.906	119.002	1.00	30.00	O
ATOM	7183	CB	ILE	C	696	109.801	96.015	118.899	1.00	20.00	C
ATOM	7184	CG1	ILE	C	696	108.654	96.849	119.516	1.00	20.00	C
ATOM	7185	CG2	ILE	C	696	109.300	95.323	117.603	1.00	20.00	C
ATOM	7186	CD1	ILE	C	696	108.375	98.170	118.779	1.00	20.00	C
ATOM	7187	H	ILE	C	696	111.388	96.757	120.800	1.00	0.00	H
ATOM	7188	HA	ILE	C	696	109.622	94.524	120.451	1.00	0.00	H
ATOM	7189	HB	ILE	C	696	110.581	96.718	118.613	1.00	0.00	H
ATOM	7190	HG12	ILE	C	696	107.742	96.253	119.558	1.00	0.00	H
ATOM	7191	HG13	ILE	C	696	108.882	97.093	120.554	1.00	0.00	H
ATOM	7192	HG21	ILE	C	696	108.803	96.022	116.933	1.00	0.00	H
ATOM	7193	HG22	ILE	C	696	110.113	94.883	117.025	1.00	0.00	H
ATOM	7194	HG23	ILE	C	696	108.586	94.531	117.832	1.00	0.00	H
ATOM	7195	HD11	ILE	C	696	109.156	98.902	118.977	1.00	0.00	H
ATOM	7196	HD12	ILE	C	696	108.320	98.043	117.700	1.00	0.00	H
ATOM	7197	HD13	ILE	C	696	107.430	98.603	119.108	1.00	0.00	H
ATOM	7198	N	TRP	C	697	112.599	94.313	119.013	1.00	30.00	N
ATOM	7199	CA	TRP	C	697	113.578	93.376	118.447	1.00	30.00	C
ATOM	7200	C	TRP	C	697	113.820	92.148	119.346	1.00	30.00	C
ATOM	7201	O	TRP	C	697	113.897	91.029	118.836	1.00	30.00	O
ATOM	7202	CB	TRP	C	697	114.899	94.104	118.119	1.00	20.00	C
ATOM	7203	CG	TRP	C	697	116.029	93.250	117.608	1.00	20.00	C
ATOM	7204	CD1	TRP	C	697	116.243	92.928	116.314	1.00	20.00	C
ATOM	7205	CD2	TRP	C	697	117.057	92.537	118.363	1.00	20.00	C
ATOM	7206	CE2	TRP	C	697	117.893	91.833	117.443	1.00	20.00	C
ATOM	7207	CE3	TRP	C	697	117.374	92.416	119.737	1.00	20.00	C
ATOM	7208	NE1	TRP	C	697	117.377	92.144	116.205	1.00	20.00	N
ATOM	7209	CZ2	TRP	C	697	118.979	91.046	117.863	1.00	20.00	C
ATOM	7210	CZ3	TRP	C	697	118.459	91.630	120.170	1.00	20.00	C
ATOM	7211	CH2	TRP	C	697	119.260	90.947	119.237	1.00	20.00	C
ATOM	7212	H	TRP	C	697	112.923	95.260	119.177	1.00	0.00	H
ATOM	7213	HA	TRP	C	697	113.170	93.004	117.505	1.00	0.00	H
ATOM	7214	HB2	TRP	C	697	114.710	94.887	117.383	1.00	0.00	H
ATOM	7215	HB3	TRP	C	697	115.266	94.615	119.009	1.00	0.00	H
ATOM	7216	HD1	TRP	C	697	115.619	93.279	115.504	1.00	0.00	H
ATOM	7217	HE1	TRP	C	697	117.783	91.896	115.313	1.00	0.00	H
ATOM	7218	HE3	TRP	C	697	116.772	92.945	120.460	1.00	0.00	H
ATOM	7219	HZ2	TRP	C	697	119.604	90.542	117.142	1.00	0.00	H
ATOM	7220	HZ3	TRP	C	697	118.695	91.563	121.221	1.00	0.00	H
ATOM	7221	HH2	TRP	C	697	120.100	90.365	119.583	1.00	0.00	H
ATOM	7222	N	LYS	C	698	113.886	92.364	120.671	1.00	30.00	N
ATOM	7223	CA	LYS	C	698	114.025	91.289	121.663	1.00	30.00	C
ATOM	7224	C	LYS	C	698	112.868	90.276	121.596	1.00	30.00	C
ATOM	7225	O	LYS	C	698	113.117	89.076	121.712	1.00	30.00	O
ATOM	7226	CB	LYS	C	698	114.124	91.882	123.088	1.00	20.00	C
ATOM	7227	CG	LYS	C	698	115.399	92.692	123.379	1.00	20.00	C
ATOM	7228	CD	LYS	C	698	116.656	91.835	123.598	1.00	20.00	C
ATOM	7229	CE	LYS	C	698	117.919	92.673	123.857	1.00	20.00	C
ATOM	7230	NZ	LYS	C	698	117.826	93.440	125.112	1.00	20.00	N1+

```
ATOM   7231  H   LYS C 698     113.747  93.295 121.038  1.00  0.00           H
ATOM   7232  HA  LYS C 698     114.946  90.750 121.437  1.00  0.00           H
ATOM   7233  HB2 LYS C 698     113.257  92.517 123.270  1.00  0.00           H
ATOM   7234  HB3 LYS C 698     114.054  91.084 123.829  1.00  0.00           H
ATOM   7235  HG2 LYS C 698     115.590  93.381 122.561  1.00  0.00           H
ATOM   7236  HG3 LYS C 698     115.217  93.312 124.258  1.00  0.00           H
ATOM   7237  HD2 LYS C 698     116.493  91.152 124.432  1.00  0.00           H
ATOM   7238  HD3 LYS C 698     116.825  91.204 122.726  1.00  0.00           H
ATOM   7239  HE2 LYS C 698     118.094  93.364 123.030  1.00  0.00           H
ATOM   7240  HE3 LYS C 698     118.791  92.020 123.916  1.00  0.00           H
ATOM   7241  HZ1 LYS C 698     117.695  92.807 125.887  1.00  0.00           H
ATOM   7242  HZ2 LYS C 698     118.677  93.966 125.252  1.00  0.00           H
ATOM   7243  HZ3 LYS C 698     117.042  94.075 125.062  1.00  0.00           H
ATOM   7244  N   LEU C 699     111.641  90.781 121.378  1.00 30.00           N
ATOM   7245  CA  LEU C 699     110.428  89.980 121.225  1.00 30.00           C
ATOM   7246  C   LEU C 699     110.332  89.303 119.844  1.00 30.00           C
ATOM   7247  O   LEU C 699     109.827  88.184 119.777  1.00 30.00           O
ATOM   7248  CB  LEU C 699     109.205  90.850 121.600  1.00 20.00           C
ATOM   7249  CG  LEU C 699     107.867  90.082 121.766  1.00 20.00           C
ATOM   7250  CD1 LEU C 699     106.968  90.694 122.858  1.00 20.00           C
ATOM   7251  CD2 LEU C 699     107.101  89.908 120.439  1.00 20.00           C
ATOM   7252  H   LEU C 699     111.528  91.781 121.287  1.00  0.00           H
ATOM   7253  HA  LEU C 699     110.486  89.178 121.960  1.00  0.00           H
ATOM   7254  HB2 LEU C 699     109.453  91.322 122.553  1.00  0.00           H
ATOM   7255  HB3 LEU C 699     109.086  91.678 120.899  1.00  0.00           H
ATOM   7256  HG  LEU C 699     108.122  89.084 122.127  1.00  0.00           H
ATOM   7257 HD11 LEU C 699     106.424  89.915 123.393  1.00  0.00           H
ATOM   7258 HD12 LEU C 699     107.533  91.249 123.604  1.00  0.00           H
ATOM   7259 HD13 LEU C 699     106.234  91.383 122.442  1.00  0.00           H
ATOM   7260 HD21 LEU C 699     106.931  88.851 120.241  1.00  0.00           H
ATOM   7261 HD22 LEU C 699     106.124  90.392 120.449  1.00  0.00           H
ATOM   7262 HD23 LEU C 699     107.644  90.319 119.588  1.00  0.00           H
ATOM   7263  N   GLN C 700     110.862  89.939 118.787  1.00 30.00           N
ATOM   7264  CA  GLN C 700     110.981  89.340 117.454  1.00 30.00           C
ATOM   7265  C   GLN C 700     111.977  88.164 117.395  1.00 30.00           C
ATOM   7266  O   GLN C 700     111.691  87.171 116.723  1.00 30.00           O
ATOM   7267  CB  GLN C 700     111.287  90.418 116.392  1.00 20.00           C
ATOM   7268  CG  GLN C 700     110.058  91.285 116.036  1.00 20.00           C
ATOM   7269  CD  GLN C 700     110.320  92.291 114.912  1.00 20.00           C
ATOM   7270  NE2 GLN C 700     109.307  93.070 114.545  1.00 20.00           N
ATOM   7271  OE1 GLN C 700     111.414  92.365 114.364  1.00 20.00           O
ATOM   7272  H   GLN C 700     111.207  90.886 118.897  1.00  0.00           H
ATOM   7273  HA  GLN C 700     110.008  88.909 117.209  1.00  0.00           H
ATOM   7274  HB2 GLN C 700     112.107  91.053 116.727  1.00  0.00           H
ATOM   7275  HB3 GLN C 700     111.647  89.938 115.481  1.00  0.00           H
ATOM   7276  HG2 GLN C 700     109.236  90.640 115.724  1.00  0.00           H
ATOM   7277  HG3 GLN C 700     109.713  91.826 116.916  1.00  0.00           H
ATOM   7278 HE21 GLN C 700     109.417  93.746 113.797  1.00  0.00           H
ATOM   7279 HE22 GLN C 700     108.403  92.991 114.984  1.00  0.00           H
ATOM   7280  N   ARG C 701     113.100  88.254 118.127  1.00 30.00           N
ATOM   7281  CA  ARG C 701     114.026  87.132 118.324  1.00 30.00           C
ATOM   7282  C   ARG C 701     113.382  85.997 119.149  1.00 30.00           C
ATOM   7283  O   ARG C 701     113.569  84.835 118.793  1.00 30.00           O
ATOM   7284  CB  ARG C 701     115.368  87.626 118.930  1.00 20.00           C
ATOM   7285  CG  ARG C 701     116.362  86.488 119.266  1.00 20.00           C
ATOM   7286  CD  ARG C 701     117.690  86.922 119.906  1.00 20.00           C
ATOM   7287  NE  ARG C 701     118.673  87.420 118.934  1.00 20.00           N
ATOM   7288  CZ  ARG C 701     119.955  87.706 119.232  1.00 20.00           C
ATOM   7289  NH1 ARG C 701     120.424  87.581 120.482  1.00 20.00           N
ATOM   7290  NH2 ARG C 701     120.790  88.114 118.269  1.00 20.00           N1+
ATOM   7291  H   ARG C 701     113.296  89.098 118.648  1.00  0.00           H
ATOM   7292  HA  ARG C 701     114.253  86.726 117.336  1.00  0.00           H
ATOM   7293  HB2 ARG C 701     115.837  88.338 118.250  1.00  0.00           H
ATOM   7294  HB3 ARG C 701     115.155  88.179 119.847  1.00  0.00           H
ATOM   7295  HG2 ARG C 701     115.919  85.734 119.912  1.00  0.00           H
ATOM   7296  HG3 ARG C 701     116.585  85.980 118.327  1.00  0.00           H
ATOM   7297  HD2 ARG C 701     117.503  87.772 120.564  1.00  0.00           H
ATOM   7298  HD3 ARG C 701     118.090  86.124 120.534  1.00  0.00           H
ATOM   7299  HE  ARG C 701     118.348  87.532 117.986  1.00  0.00           H
ATOM   7300 HH11 ARG C 701     121.389  87.785 120.698  1.00  0.00           H
ATOM   7301 HH12 ARG C 701     119.817  87.259 121.223  1.00  0.00           H
```

ATOM	7302	HH21	ARG	C	701	121.761	88.293	118.480	1.00	0.00	H
ATOM	7303	HH22	ARG	C	701	120.480	88.178	117.312	1.00	0.00	H
ATOM	7304	N	ALA	C	702	112.630	86.350	120.210	1.00	30.00	N
ATOM	7305	CA	ALA	C	702	111.934	85.408	121.093	1.00	30.00	C
ATOM	7306	C	ALA	C	702	110.871	84.553	120.382	1.00	30.00	C
ATOM	7307	O	ALA	C	702	110.723	83.387	120.742	1.00	30.00	O
ATOM	7308	CB	ALA	C	702	111.317	86.160	122.279	1.00	20.00	C
ATOM	7309	H	ALA	C	702	112.532	87.329	120.444	1.00	0.00	H
ATOM	7310	HA	ALA	C	702	112.682	84.724	121.495	1.00	0.00	H
ATOM	7311	HB1	ALA	C	702	110.871	85.467	122.994	1.00	0.00	H
ATOM	7312	HB2	ALA	C	702	112.069	86.740	122.815	1.00	0.00	H
ATOM	7313	HB3	ALA	C	702	110.532	86.842	121.957	1.00	0.00	H
ATOM	7314	N	ILE	C	703	110.194	85.121	119.367	1.00	30.00	N
ATOM	7315	CA	ILE	C	703	109.262	84.401	118.497	1.00	30.00	C
ATOM	7316	C	ILE	C	703	109.974	83.310	117.668	1.00	30.00	C
ATOM	7317	O	ILE	C	703	109.532	82.165	117.716	1.00	30.00	O
ATOM	7318	CB	ILE	C	703	108.444	85.362	117.575	1.00	20.00	C
ATOM	7319	CG1	ILE	C	703	107.428	86.180	118.408	1.00	20.00	C
ATOM	7320	CG2	ILE	C	703	107.712	84.672	116.399	1.00	20.00	C
ATOM	7321	CD1	ILE	C	703	106.985	87.496	117.749	1.00	20.00	C
ATOM	7322	H	ILE	C	703	110.352	86.095	119.147	1.00	0.00	H
ATOM	7323	HA	ILE	C	703	108.549	83.890	119.148	1.00	0.00	H
ATOM	7324	HB	ILE	C	703	109.150	86.073	117.142	1.00	0.00	H
ATOM	7325	HG12	ILE	C	703	106.553	85.569	118.632	1.00	0.00	H
ATOM	7326	HG13	ILE	C	703	107.856	86.431	119.378	1.00	0.00	H
ATOM	7327	HG21	ILE	C	703	107.098	85.368	115.830	1.00	0.00	H
ATOM	7328	HG22	ILE	C	703	108.405	84.232	115.681	1.00	0.00	H
ATOM	7329	HG23	ILE	C	703	107.055	83.879	116.758	1.00	0.00	H
ATOM	7330	HD11	ILE	C	703	106.061	87.861	118.200	1.00	0.00	H
ATOM	7331	HD12	ILE	C	703	107.742	88.270	117.876	1.00	0.00	H
ATOM	7332	HD13	ILE	C	703	106.802	87.392	116.680	1.00	0.00	H
ATOM	7333	N	THR	C	704	111.078	83.657	116.977	1.00	30.00	N
ATOM	7334	CA	THR	C	704	111.855	82.713	116.157	1.00	30.00	C
ATOM	7335	C	THR	C	704	112.545	81.589	116.964	1.00	30.00	C
ATOM	7336	O	THR	C	704	112.711	80.498	116.420	1.00	30.00	O
ATOM	7337	CB	THR	C	704	112.930	83.415	115.282	1.00	20.00	C
ATOM	7338	CG2	THR	C	704	112.378	84.546	114.406	1.00	20.00	C
ATOM	7339	OG1	THR	C	704	114.049	83.879	116.017	1.00	20.00	O
ATOM	7340	H	THR	C	704	111.399	84.614	116.994	1.00	0.00	H
ATOM	7341	HA	THR	C	704	111.147	82.228	115.481	1.00	0.00	H
ATOM	7342	HB	THR	C	704	113.327	82.665	114.596	1.00	0.00	H
ATOM	7343	HG1	THR	C	704	113.742	84.474	116.707	1.00	0.00	H
ATOM	7344	HG21	THR	C	704	113.147	84.918	113.729	1.00	0.00	H
ATOM	7345	HG22	THR	C	704	111.545	84.193	113.799	1.00	0.00	H
ATOM	7346	HG23	THR	C	704	112.020	85.384	114.997	1.00	0.00	H
ATOM	7347	N	ILE	C	705	112.884	81.840	118.242	1.00	30.00	N
ATOM	7348	CA	ILE	C	705	113.388	80.821	119.168	1.00	30.00	C
ATOM	7349	C	ILE	C	705	112.328	79.744	119.483	1.00	30.00	C
ATOM	7350	O	ILE	C	705	112.666	78.561	119.489	1.00	30.00	O
ATOM	7351	CB	ILE	C	705	113.937	81.453	120.489	1.00	20.00	C
ATOM	7352	CG1	ILE	C	705	115.274	82.183	120.224	1.00	20.00	C
ATOM	7353	CG2	ILE	C	705	114.109	80.466	121.668	1.00	20.00	C
ATOM	7354	CD1	ILE	C	705	115.612	83.277	121.239	1.00	20.00	C
ATOM	7355	H	ILE	C	705	112.737	82.763	118.626	1.00	0.00	H
ATOM	7356	HA	ILE	C	705	114.216	80.311	118.672	1.00	0.00	H
ATOM	7357	HB	ILE	C	705	113.218	82.208	120.810	1.00	0.00	H
ATOM	7358	HG12	ILE	C	705	116.088	81.460	120.229	1.00	0.00	H
ATOM	7359	HG13	ILE	C	705	115.275	82.636	119.231	1.00	0.00	H
ATOM	7360	HG21	ILE	C	705	114.604	80.928	122.521	1.00	0.00	H
ATOM	7361	HG22	ILE	C	705	113.153	80.094	122.038	1.00	0.00	H
ATOM	7362	HG23	ILE	C	705	114.710	79.607	121.370	1.00	0.00	H
ATOM	7363	HD11	ILE	C	705	116.545	83.772	120.974	1.00	0.00	H
ATOM	7364	HD12	ILE	C	705	114.833	84.034	121.290	1.00	0.00	H
ATOM	7365	HD13	ILE	C	705	115.743	82.859	122.233	1.00	0.00	H
ATOM	7366	N	LEU	C	706	111.074	80.171	119.707	1.00	30.00	N
ATOM	7367	CA	LEU	C	706	109.956	79.284	120.027	1.00	30.00	C
ATOM	7368	C	LEU	C	706	109.421	78.525	118.804	1.00	30.00	C
ATOM	7369	O	LEU	C	706	109.148	77.335	118.938	1.00	30.00	O
ATOM	7370	CB	LEU	C	706	108.821	80.086	120.695	1.00	20.00	C
ATOM	7371	CG	LEU	C	706	109.165	80.658	122.087	1.00	20.00	C
ATOM	7372	CD1	LEU	C	706	108.029	81.576	122.574	1.00	20.00	C

```
ATOM   7373  CD2 LEU C 706     109.526  79.572 123.123  1.00 20.00           C
ATOM   7374  H   LEU C 706     110.865  81.159 119.668  1.00  0.00           H
ATOM   7375  HA  LEU C 706     110.317  78.530 120.728  1.00  0.00           H
ATOM   7376  HB2 LEU C 706     108.528  80.899 120.029  1.00  0.00           H
ATOM   7377  HB3 LEU C 706     107.936  79.455 120.798  1.00  0.00           H
ATOM   7378  HG  LEU C 706     110.047  81.286 121.977  1.00  0.00           H
ATOM   7379 HD11 LEU C 706     108.426  82.492 123.013  1.00  0.00           H
ATOM   7380 HD12 LEU C 706     107.361  81.871 121.764  1.00  0.00           H
ATOM   7381 HD13 LEU C 706     107.409  81.085 123.322  1.00  0.00           H
ATOM   7382 HD21 LEU C 706     108.982  79.688 124.060  1.00  0.00           H
ATOM   7383 HD22 LEU C 706     109.313  78.567 122.760  1.00  0.00           H
ATOM   7384 HD23 LEU C 706     110.588  79.610 123.366  1.00  0.00           H
ATOM   7385  N   ASP C 707     109.282  79.205 117.651  1.00 30.00           N
ATOM   7386  CA  ASP C 707     108.739  78.641 116.404  1.00 30.00           C
ATOM   7387  C   ASP C 707     109.586  77.500 115.813  1.00 30.00           C
ATOM   7388  O   ASP C 707     109.015  76.574 115.237  1.00 30.00           O
ATOM   7389  CB  ASP C 707     108.446  79.698 115.309  1.00 20.00           C
ATOM   7390  CG  ASP C 707     107.503  80.840 115.710  1.00  0.00           C
ATOM   7391  OD1 ASP C 707     106.911  80.781 116.812  1.00  0.00           O
ATOM   7392  OD2 ASP C 707     107.343  81.752 114.872  1.00  0.00           O1-
ATOM   7393  H   ASP C 707     109.498  80.194 117.631  1.00  0.00           H
ATOM   7394  HA  ASP C 707     107.780  78.191 116.672  1.00  0.00           H
ATOM   7395  HB2 ASP C 707     109.392  80.146 115.001  1.00  0.00           H
ATOM   7396  HB3 ASP C 707     108.009  79.202 114.441  1.00  0.00           H
ATOM   7397  N   THR C 708     110.916  77.561 115.991  1.00 30.00           N
ATOM   7398  CA  THR C 708     111.837  76.500 115.579  1.00 30.00           C
ATOM   7399  C   THR C 708     111.847  75.293 116.541  1.00 30.00           C
ATOM   7400  O   THR C 708     112.212  74.212 116.086  1.00 30.00           O
ATOM   7401  CB  THR C 708     113.288  77.014 115.424  1.00 20.00           C
ATOM   7402  CG2 THR C 708     113.418  78.065 114.308  1.00 20.00           C
ATOM   7403  OG1 THR C 708     113.786  77.520 116.648  1.00 20.00           O
ATOM   7404  H   THR C 708     111.321  78.352 116.473  1.00  0.00           H
ATOM   7405  HA  THR C 708     111.516  76.136 114.602  1.00  0.00           H
ATOM   7406  HB  THR C 708     113.937  76.179 115.153  1.00  0.00           H
ATOM   7407  HG1 THR C 708     113.412  78.393 116.798  1.00  0.00           H
ATOM   7408 HG21 THR C 708     114.434  78.447 114.227  1.00  0.00           H
ATOM   7409 HG22 THR C 708     113.162  77.629 113.342  1.00  0.00           H
ATOM   7410 HG23 THR C 708     112.753  78.914 114.459  1.00  0.00           H
ATOM   7411  N   GLU C 709     111.397  75.450 117.800  1.00 30.00           N
ATOM   7412  CA  GLU C 709     111.153  74.323 118.711  1.00 30.00           C
ATOM   7413  C   GLU C 709     109.848  73.569 118.391  1.00 30.00           C
ATOM   7414  O   GLU C 709     109.778  72.374 118.679  1.00 30.00           O
ATOM   7415  CB  GLU C 709     111.198  74.784 120.182  1.00 20.00           C
ATOM   7416  CG  GLU C 709     112.618  75.173 120.641  1.00 20.00           C
ATOM   7417  CD  GLU C 709     112.701  75.580 122.114  1.00 20.00           C
ATOM   7418  OE1 GLU C 709     111.703  76.117 122.644  1.00 20.00           O
ATOM   7419  OE2 GLU C 709     113.784  75.366 122.700  1.00 20.00           O1-
ATOM   7420  H   GLU C 709     111.116  76.363 118.129  1.00  0.00           H
ATOM   7421  HA  GLU C 709     111.958  73.596 118.581  1.00  0.00           H
ATOM   7422  HB2 GLU C 709     110.523  75.628 120.324  1.00  0.00           H
ATOM   7423  HB3 GLU C 709     110.823  73.990 120.830  1.00  0.00           H
ATOM   7424  HG2 GLU C 709     113.295  74.334 120.479  1.00  0.00           H
ATOM   7425  HG3 GLU C 709     113.000  75.996 120.038  1.00  0.00           H
ATOM   7426  N   LYS C 710     108.865  74.246 117.763  1.00 30.00           N
ATOM   7427  CA  LYS C 710     107.653  73.613 117.228  1.00 30.00           C
ATOM   7428  C   LYS C 710     107.942  72.847 115.921  1.00 30.00           C
ATOM   7429  O   LYS C 710     107.303  71.824 115.677  1.00 30.00           O
ATOM   7430  CB  LYS C 710     106.538  74.660 116.986  1.00 20.00           C
ATOM   7431  CG  LYS C 710     106.215  75.605 118.161  1.00  0.00           C
ATOM   7432  CD  LYS C 710     105.878  74.911 119.493  1.00  0.00           C
ATOM   7433  CE  LYS C 710     105.686  75.895 120.661  1.00  0.00           C
ATOM   7434  NZ  LYS C 710     106.951  76.557 121.026  1.00  0.00           N1+
ATOM   7435  H   LYS C 710     108.984  75.230 117.567  1.00  0.00           H
ATOM   7436  HA  LYS C 710     107.289  72.884 117.954  1.00  0.00           H
ATOM   7437  HB2 LYS C 710     106.805  75.284 116.132  1.00  0.00           H
ATOM   7438  HB3 LYS C 710     105.625  74.139 116.694  1.00  0.00           H
ATOM   7439  HG2 LYS C 710     107.046  76.289 118.295  1.00  0.00           H
ATOM   7440  HG3 LYS C 710     105.374  76.238 117.873  1.00  0.00           H
ATOM   7441  HD2 LYS C 710     104.966  74.326 119.369  1.00  0.00           H
ATOM   7442  HD3 LYS C 710     106.657  74.197 119.760  1.00  0.00           H
ATOM   7443  HE2 LYS C 710     104.938  76.650 120.417  1.00  0.00           H
```

```
ATOM   7444  HE3 LYS C 710     105.323  75.358 121.538  1.00  0.00           H
ATOM   7445  HZ1 LYS C 710     107.281  77.125 120.259  1.00  0.00           H
ATOM   7446  HZ2 LYS C 710     106.806  77.141 121.846  1.00  0.00           H
ATOM   7447  HZ3 LYS C 710     107.647  75.861 121.250  1.00  0.00           H
ATOM   7448  N   SER C 711     108.892  73.355 115.114  1.00 30.00           N
ATOM   7449  CA  SER C 711     109.291  72.787 113.826  1.00 30.00           C
ATOM   7450  C   SER C 711     110.268  71.606 113.978  1.00 30.00           C
ATOM   7451  O   SER C 711     110.067  70.576 113.337  1.00 30.00           O
ATOM   7452  CB  SER C 711     109.884  73.914 112.953  1.00 20.00           C
ATOM   7453  OG  SER C 711     110.192  73.472 111.645  1.00  0.00           O
ATOM   7454  H   SER C 711     109.358  74.209 115.387  1.00  0.00           H
ATOM   7455  HA  SER C 711     108.394  72.416 113.325  1.00  0.00           H
ATOM   7456  HB2 SER C 711     109.178  74.742 112.874  1.00  0.00           H
ATOM   7457  HB3 SER C 711     110.792  74.314 113.404  1.00  0.00           H
ATOM   7458  HG  SER C 711     110.902  72.827 111.695  1.00  0.00           H
ATOM   7459  N   PHE C 712     111.300  71.776 114.823  1.00 30.00           N
ATOM   7460  CA  PHE C 712     112.390  70.817 115.042  1.00 30.00           C
ATOM   7461  C   PHE C 712     112.093  69.935 116.278  1.00 30.00           C
ATOM   7462  O   PHE C 712     113.009  69.569 117.014  1.00 30.00           O
ATOM   7463  CB  PHE C 712     113.707  71.625 115.154  1.00 20.00           C
ATOM   7464  CG  PHE C 712     114.992  70.871 114.855  1.00  0.00           C
ATOM   7465  CD1 PHE C 712     115.324  70.554 113.521  1.00  0.00           C
ATOM   7466  CD2 PHE C 712     115.819  70.402 115.898  1.00  0.00           C
ATOM   7467  CE1 PHE C 712     116.481  69.838 113.245  1.00  0.00           C
ATOM   7468  CE2 PHE C 712     116.978  69.697 115.600  1.00  0.00           C
ATOM   7469  CZ  PHE C 712     117.307  69.418 114.279  1.00  0.00           C
ATOM   7470  H   PHE C 712     111.390  72.650 115.327  1.00  0.00           H
ATOM   7471  HA  PHE C 712     112.465  70.157 114.176  1.00  0.00           H
ATOM   7472  HB2 PHE C 712     113.677  72.456 114.448  1.00  0.00           H
ATOM   7473  HB3 PHE C 712     113.787  72.085 116.140  1.00  0.00           H
ATOM   7474  HD1 PHE C 712     114.686  70.876 112.711  1.00  0.00           H
ATOM   7475  HD2 PHE C 712     115.563  70.597 116.929  1.00  0.00           H
ATOM   7476  HE1 PHE C 712     116.739  69.607 112.221  1.00  0.00           H
ATOM   7477  HE2 PHE C 712     117.616  69.354 116.400  1.00  0.00           H
ATOM   7478  HZ  PHE C 712     118.206  68.862 114.057  1.00  0.00           H
ATOM   7479  N   LEU C 713     110.806  69.603 116.477  1.00 30.00           N
ATOM   7480  CA  LEU C 713     110.286  68.806 117.589  1.00 30.00           C
ATOM   7481  C   LEU C 713     110.641  67.306 117.493  1.00 30.00           C
ATOM   7482  O   LEU C 713     110.665  66.635 118.524  1.00 30.00           O
ATOM   7483  CB  LEU C 713     108.760  69.053 117.661  1.00 20.00           C
ATOM   7484  CG  LEU C 713     108.019  68.454 118.879  1.00 20.00           C
ATOM   7485  CD1 LEU C 713     108.588  68.959 120.223  1.00 20.00           C
ATOM   7486  CD2 LEU C 713     106.499  68.697 118.763  1.00 20.00           C
ATOM   7487  H   LEU C 713     110.113  69.948 115.828  1.00  0.00           H
ATOM   7488  HA  LEU C 713     110.748  69.190 118.499  1.00  0.00           H
ATOM   7489  HB2 LEU C 713     108.578  70.129 117.652  1.00  0.00           H
ATOM   7490  HB3 LEU C 713     108.303  68.673 116.745  1.00  0.00           H
ATOM   7491  HG  LEU C 713     108.150  67.372 118.857  1.00  0.00           H
ATOM   7492 HD11 LEU C 713     107.810  69.187 120.951  1.00  0.00           H
ATOM   7493 HD12 LEU C 713     109.230  68.203 120.674  1.00  0.00           H
ATOM   7494 HD13 LEU C 713     109.185  69.864 120.102  1.00  0.00           H
ATOM   7495 HD21 LEU C 713     105.949  67.760 118.854  1.00  0.00           H
ATOM   7496 HD22 LEU C 713     106.120  69.371 119.532  1.00  0.00           H
ATOM   7497 HD23 LEU C 713     106.226  69.137 117.803  1.00  0.00           H
ATOM   7498  N   LYS C 714     110.947  66.811 116.279  1.00 30.00           N
ATOM   7499  CA  LYS C 714     111.394  65.435 116.025  1.00 30.00           C
ATOM   7500  C   LYS C 714     112.804  65.133 116.586  1.00 30.00           C
ATOM   7501  O   LYS C 714     113.117  63.965 116.814  1.00 30.00           O
ATOM   7502  CB  LYS C 714     111.269  65.146 114.511  1.00 20.00           C
ATOM   7503  CG  LYS C 714     111.545  63.685 114.107  1.00  0.00           C
ATOM   7504  CD  LYS C 714     111.178  63.386 112.646  1.00  0.00           C
ATOM   7505  CE  LYS C 714     111.414  61.914 112.275  1.00  0.00           C
ATOM   7506  NZ  LYS C 714     111.037  61.644 110.877  1.00  0.00           N1+
ATOM   7507  H   LYS C 714     110.906  67.422 115.476  1.00  0.00           H
ATOM   7508  HA  LYS C 714     110.700  64.771 116.545  1.00  0.00           H
ATOM   7509  HB2 LYS C 714     110.251  65.392 114.205  1.00  0.00           H
ATOM   7510  HB3 LYS C 714     111.921  65.814 113.946  1.00  0.00           H
ATOM   7511  HG2 LYS C 714     112.599  63.451 114.256  1.00  0.00           H
ATOM   7512  HG3 LYS C 714     110.986  63.017 114.764  1.00  0.00           H
ATOM   7513  HD2 LYS C 714     110.131  63.643 112.475  1.00  0.00           H
ATOM   7514  HD3 LYS C 714     111.763  64.029 111.988  1.00  0.00           H
```

ATOM	7515	HE2	LYS	C	714	112.463	61.652	112.414	1.00	0.00	H
ATOM	7516	HE3	LYS	C	714	110.829	61.262	112.925	1.00	0.00	H
ATOM	7517	HZ1	LYS	C	714	111.592	62.218	110.259	1.00	0.00	H
ATOM	7518	HZ2	LYS	C	714	111.200	60.670	110.666	1.00	0.00	H
ATOM	7519	HZ3	LYS	C	714	110.058	61.856	110.744	1.00	0.00	H
ATOM	7520	N	CYS	C	715	113.602	66.184	116.839	1.00	30.00	N
ATOM	7521	CA	CYS	C	715	114.907	66.119	117.491	1.00	30.00	C
ATOM	7522	C	CYS	C	715	114.916	67.158	118.627	1.00	30.00	C
ATOM	7523	O	CYS	C	715	115.644	68.150	118.569	1.00	30.00	O
ATOM	7524	CB	CYS	C	715	116.051	66.250	116.460	1.00	20.00	C
ATOM	7525	SG	CYS	C	715	117.702	66.163	117.219	1.00	0.00	S
ATOM	7526	H	CYS	C	715	113.260	67.115	116.645	1.00	0.00	H
ATOM	7527	HA	CYS	C	715	115.025	65.149	117.977	1.00	0.00	H
ATOM	7528	HB2	CYS	C	715	115.978	65.448	115.724	1.00	0.00	H
ATOM	7529	HB3	CYS	C	715	115.960	67.184	115.907	1.00	0.00	H
ATOM	7530	HG	CYS	C	715	117.604	67.329	117.866	1.00	0.00	H
ATOM	7531	N	MET	C	716	114.072	66.903	119.642	1.00	30.00	N
ATOM	7532	CA	MET	C	716	113.874	67.750	120.824	1.00	30.00	C
ATOM	7533	C	MET	C	716	114.890	67.490	121.960	1.00	30.00	C
ATOM	7534	O	MET	C	716	114.600	67.825	123.109	1.00	30.00	O
ATOM	7535	CB	MET	C	716	112.411	67.621	121.304	1.00	20.00	C
ATOM	7536	CG	MET	C	716	111.982	66.203	121.728	1.00	0.00	C
ATOM	7537	SD	MET	C	716	110.447	66.144	122.690	1.00	0.00	S
ATOM	7538	CE	MET	C	716	111.058	66.724	124.297	1.00	0.00	C
ATOM	7539	H	MET	C	716	113.491	66.079	119.596	1.00	0.00	H
ATOM	7540	HA	MET	C	716	114.018	68.791	120.525	1.00	0.00	H
ATOM	7541	HB2	MET	C	716	112.229	68.321	122.119	1.00	0.00	H
ATOM	7542	HB3	MET	C	716	111.753	67.962	120.505	1.00	0.00	H
ATOM	7543	HG2	MET	C	716	111.842	65.586	120.840	1.00	0.00	H
ATOM	7544	HG3	MET	C	716	112.757	65.714	122.317	1.00	0.00	H
ATOM	7545	HE1	MET	C	716	110.247	66.738	125.025	1.00	0.00	H
ATOM	7546	HE2	MET	C	716	111.841	66.063	124.668	1.00	0.00	H
ATOM	7547	HE3	MET	C	716	111.465	67.732	124.221	1.00	0.00	H
ATOM	7548	N	ARG	C	717	116.066	66.924	121.636	1.00	30.00	N
ATOM	7549	CA	ARG	C	717	117.164	66.668	122.578	1.00	30.00	C
ATOM	7550	C	ARG	C	717	117.789	67.951	123.169	1.00	30.00	C
ATOM	7551	O	ARG	C	717	118.345	67.887	124.264	1.00	30.00	O
ATOM	7552	CB	ARG	C	717	118.245	65.798	121.899	1.00	20.00	C
ATOM	7553	CG	ARG	C	717	117.733	64.430	121.403	1.00	0.00	C
ATOM	7554	CD	ARG	C	717	118.830	63.545	120.787	1.00	0.00	C
ATOM	7555	NE	ARG	C	717	119.265	64.030	119.468	1.00	0.00	N
ATOM	7556	CZ	ARG	C	717	120.358	63.625	118.798	1.00	0.00	C
ATOM	7557	NH1	ARG	C	717	121.198	62.716	119.312	1.00	0.00	N
ATOM	7558	NH2	ARG	C	717	120.609	64.138	117.587	1.00	0.00	N1+
ATOM	7559	H	ARG	C	717	116.241	66.682	120.672	1.00	0.00	H
ATOM	7560	HA	ARG	C	717	116.750	66.101	123.414	1.00	0.00	H
ATOM	7561	HB2	ARG	C	717	118.695	66.346	121.068	1.00	0.00	H
ATOM	7562	HB3	ARG	C	717	119.048	65.622	122.616	1.00	0.00	H
ATOM	7563	HG2	ARG	C	717	117.209	63.881	122.187	1.00	0.00	H
ATOM	7564	HG3	ARG	C	717	116.989	64.625	120.629	1.00	0.00	H
ATOM	7565	HD2	ARG	C	717	119.716	63.646	121.415	1.00	0.00	H
ATOM	7566	HD3	ARG	C	717	118.570	62.487	120.794	1.00	0.00	H
ATOM	7567	HE	ARG	C	717	118.642	64.682	119.011	1.00	0.00	H
ATOM	7568	HH11	ARG	C	717	122.013	62.416	118.797	1.00	0.00	H
ATOM	7569	HH12	ARG	C	717	121.015	62.318	120.222	1.00	0.00	H
ATOM	7570	HH21	ARG	C	717	121.420	63.844	117.062	1.00	0.00	H
ATOM	7571	HH22	ARG	C	717	119.978	64.815	117.180	1.00	0.00	H
ATOM	7572	N	LYS	C	718	117.669	69.081	122.448	1.00	30.00	N
ATOM	7573	CA	LYS	C	718	118.154	70.405	122.847	1.00	30.00	C
ATOM	7574	C	LYS	C	718	117.016	71.375	123.235	1.00	30.00	C
ATOM	7575	O	LYS	C	718	117.315	72.524	123.565	1.00	30.00	O
ATOM	7576	CB	LYS	C	718	119.026	70.981	121.709	1.00	20.00	C
ATOM	7577	CG	LYS	C	718	120.342	70.209	121.502	1.00	0.00	C
ATOM	7578	CD	LYS	C	718	121.233	70.838	120.422	1.00	0.00	C
ATOM	7579	CE	LYS	C	718	122.574	70.110	120.262	1.00	0.00	C
ATOM	7580	NZ	LYS	C	718	123.410	70.749	119.231	1.00	0.00	N1+
ATOM	7581	H	LYS	C	718	117.192	69.040	121.558	1.00	0.00	H
ATOM	7582	HA	LYS	C	718	118.781	70.317	123.736	1.00	0.00	H
ATOM	7583	HB2	LYS	C	718	118.456	71.008	120.778	1.00	0.00	H
ATOM	7584	HB3	LYS	C	718	119.283	72.017	121.933	1.00	0.00	H
ATOM	7585	HG2	LYS	C	718	120.888	70.169	122.446	1.00	0.00	H

```
ATOM   7586  HG3 LYS C 718     120.125  69.175 121.231  1.00  0.00           H
ATOM   7587  HD2 LYS C 718     120.700  70.827 119.470  1.00  0.00           H
ATOM   7588  HD3 LYS C 718     121.411  71.888 120.663  1.00  0.00           H
ATOM   7589  HE2 LYS C 718     123.121  70.111 121.205  1.00  0.00           H
ATOM   7590  HE3 LYS C 718     122.409  69.068 119.985  1.00  0.00           H
ATOM   7591  HZ1 LYS C 718     123.595  71.708 119.490  1.00  0.00           H
ATOM   7592  HZ2 LYS C 718     124.285  70.250 119.149  1.00  0.00           H
ATOM   7593  HZ3 LYS C 718     122.929  70.728 118.344  1.00  0.00           H
ATOM   7594  N   ALA C 719     115.750  70.910 123.221  1.00 30.00           N
ATOM   7595  CA  ALA C 719     114.574  71.678 123.653  1.00 30.00           C
ATOM   7596  C   ALA C 719     114.526  71.919 125.176  1.00 30.00           C
ATOM   7597  O   ALA C 719     113.946  72.913 125.611  1.00 30.00           O
ATOM   7598  CB  ALA C 719     113.301  70.964 123.176  1.00 20.00           C
ATOM   7599  H   ALA C 719     115.576  69.952 122.953  1.00  0.00           H
ATOM   7600  HA  ALA C 719     114.615  72.654 123.164  1.00  0.00           H
ATOM   7601  HB1 ALA C 719     112.415  71.572 123.363  1.00  0.00           H
ATOM   7602  HB2 ALA C 719     113.336  70.770 122.104  1.00  0.00           H
ATOM   7603  HB3 ALA C 719     113.159  70.010 123.685  1.00  0.00           H
ATOM   7604  N   PHE C 720     115.167  71.024 125.947  1.00 30.00           N
ATOM   7605  CA  PHE C 720     115.449  71.168 127.377  1.00 30.00           C
ATOM   7606  C   PHE C 720     116.619  72.144 127.599  1.00 30.00           C
ATOM   7607  O   PHE C 720     117.492  72.233 126.732  1.00 30.00           O
ATOM   7608  CB  PHE C 720     115.785  69.772 127.931  1.00 20.00           C
ATOM   7609  CG  PHE C 720     114.674  68.738 127.801  1.00  0.00           C
ATOM   7610  CD1 PHE C 720     113.364  69.011 128.255  1.00  0.00           C
ATOM   7611  CD2 PHE C 720     114.967  67.451 127.300  1.00  0.00           C
ATOM   7612  CE1 PHE C 720     112.385  68.029 128.189  1.00  0.00           C
ATOM   7613  CE2 PHE C 720     113.970  66.488 127.229  1.00  0.00           C
ATOM   7614  CZ  PHE C 720     112.685  66.776 127.672  1.00  0.00           C
ATOM   7615  H   PHE C 720     115.604  70.231 125.500  1.00  0.00           H
ATOM   7616  HA  PHE C 720     114.567  71.567 127.880  1.00  0.00           H
ATOM   7617  HB2 PHE C 720     116.671  69.393 127.417  1.00  0.00           H
ATOM   7618  HB3 PHE C 720     116.072  69.847 128.978  1.00  0.00           H
ATOM   7619  HD1 PHE C 720     113.113  69.979 128.660  1.00  0.00           H
ATOM   7620  HD2 PHE C 720     115.965  67.209 126.964  1.00  0.00           H
ATOM   7621  HE1 PHE C 720     111.384  68.241 128.534  1.00  0.00           H
ATOM   7622  HE2 PHE C 720     114.195  65.509 126.832  1.00  0.00           H
ATOM   7623  HZ  PHE C 720     111.916  66.019 127.616  1.00  0.00           H
ATOM   7624  N   ARG C 721     116.612  72.875 128.732  1.00 30.00           N
ATOM   7625  CA  ARG C 721     117.422  74.087 128.879  1.00 30.00           C
ATOM   7626  C   ARG C 721     118.177  74.168 130.214  1.00 30.00           C
ATOM   7627  O   ARG C 721     117.591  74.509 131.241  1.00 30.00           O
ATOM   7628  CB  ARG C 721     116.512  75.300 128.578  1.00 20.00           C
ATOM   7629  CG  ARG C 721     117.142  76.704 128.406  1.00  0.00           C
ATOM   7630  CD  ARG C 721     118.670  76.902 128.292  1.00  0.00           C
ATOM   7631  NE  ARG C 721     119.430  76.091 127.318  1.00  0.00           N
ATOM   7632  CZ  ARG C 721     119.049  75.427 126.210  1.00  0.00           C
ATOM   7633  NH1 ARG C 721     117.802  75.447 125.733  1.00  0.00           N
ATOM   7634  NH2 ARG C 721     119.958  74.701 125.553  1.00  0.00           N1+
ATOM   7635  H   ARG C 721     115.901  72.724 129.433  1.00  0.00           H
ATOM   7636  HA  ARG C 721     118.194  74.066 128.114  1.00  0.00           H
ATOM   7637  HB2 ARG C 721     115.997  75.083 127.642  1.00  0.00           H
ATOM   7638  HB3 ARG C 721     115.706  75.358 129.311  1.00  0.00           H
ATOM   7639  HG2 ARG C 721     116.594  77.299 127.677  1.00  0.00           H
ATOM   7640  HG3 ARG C 721     116.922  77.176 129.364  1.00  0.00           H
ATOM   7641  HD2 ARG C 721     118.768  77.889 127.840  1.00  0.00           H
ATOM   7642  HD3 ARG C 721     119.167  76.995 129.256  1.00  0.00           H
ATOM   7643  HE  ARG C 721     120.427  76.127 127.488  1.00  0.00           H
ATOM   7644 HH11 ARG C 721     117.549  74.898 124.922  1.00  0.00           H
ATOM   7645 HH12 ARG C 721     117.095  75.988 126.208  1.00  0.00           H
ATOM   7646 HH21 ARG C 721     119.690  74.179 124.732  1.00  0.00           H
ATOM   7647 HH22 ARG C 721     120.916  74.676 125.869  1.00  0.00           H
ATOM   7648  N   SER C 722     119.493  73.894 130.121  1.00 30.00           N
ATOM   7649  CA  SER C 722     120.532  74.060 131.144  1.00 30.00           C
ATOM   7650  C   SER C 722     120.331  73.177 132.397  1.00 30.00           C
ATOM   7651  O   SER C 722     120.647  73.604 133.507  1.00 30.00           O
ATOM   7652  CB  SER C 722     120.759  75.565 131.426  1.00 20.00           C
ATOM   7653  OG  SER C 722     121.938  75.813 132.164  1.00  0.00           O
ATOM   7654  H   SER C 722     119.844  73.614 129.218  1.00  0.00           H
ATOM   7655  HA  SER C 722     121.444  73.687 130.676  1.00  0.00           H
ATOM   7656  HB2 SER C 722     120.852  76.113 130.490  1.00  0.00           H
```

```
ATOM   7657  HB3 SER C 722     119.910  75.983 131.962  1.00  0.00           H
ATOM   7658  HG  SER C 722     122.026  76.760 132.303  1.00  0.00           H
ATOM   7659  N   GLY C 723     119.847  71.944 132.176  1.00 30.00           N
ATOM   7660  CA  GLY C 723     119.670  70.921 133.203  1.00 30.00           C
ATOM   7661  C   GLY C 723     118.213  70.445 133.233  1.00 30.00           C
ATOM   7662  O   GLY C 723     117.289  71.158 132.839  1.00 30.00           O
ATOM   7663  H   GLY C 723     119.587  71.685 131.236  1.00  0.00           H
ATOM   7664  HA2 GLY C 723     120.328  70.084 132.969  1.00  0.00           H
ATOM   7665  HA3 GLY C 723     119.945  71.275 134.197  1.00  0.00           H
ATOM   7666  N   LYS C 724     118.037  69.212 133.732  1.00 30.00           N
ATOM   7667  CA  LYS C 724     116.756  68.527 133.930  1.00 30.00           C
ATOM   7668  C   LYS C 724     116.712  67.741 135.254  1.00 30.00           C
ATOM   7669  O   LYS C 724     115.679  67.137 135.542  1.00 30.00           O
ATOM   7670  CB  LYS C 724     116.469  67.596 132.724  1.00 20.00           C
ATOM   7671  CG  LYS C 724     116.025  68.305 131.429  1.00 20.00           C
ATOM   7672  CD  LYS C 724     114.711  69.103 131.533  1.00 20.00           C
ATOM   7673  CE  LYS C 724     113.487  68.249 131.909  1.00 20.00           C
ATOM   7674  NZ  LYS C 724     112.233  69.018 131.811  1.00 20.00           N1+
ATOM   7675  H   LYS C 724     118.853  68.683 134.002  1.00  0.00           H
ATOM   7676  HA  LYS C 724     115.969  69.273 134.011  1.00  0.00           H
ATOM   7677  HB2 LYS C 724     117.350  66.987 132.520  1.00  0.00           H
ATOM   7678  HB3 LYS C 724     115.688  66.879 132.980  1.00  0.00           H
ATOM   7679  HG2 LYS C 724     116.821  68.969 131.093  1.00  0.00           H
ATOM   7680  HG3 LYS C 724     115.920  67.559 130.640  1.00  0.00           H
ATOM   7681  HD2 LYS C 724     114.844  69.926 132.229  1.00  0.00           H
ATOM   7682  HD3 LYS C 724     114.522  69.596 130.585  1.00  0.00           H
ATOM   7683  HE2 LYS C 724     113.411  67.390 131.241  1.00  0.00           H
ATOM   7684  HE3 LYS C 724     113.581  67.863 132.925  1.00  0.00           H
ATOM   7685  HZ1 LYS C 724     112.262  69.808 132.439  1.00  0.00           H
ATOM   7686  HZ2 LYS C 724     112.111  69.347 130.864  1.00  0.00           H
ATOM   7687  HZ3 LYS C 724     111.453  68.425 132.060  1.00  0.00           H
ATOM   7688  N   LEU C 725     117.817  67.747 136.021  1.00 30.00           N
ATOM   7689  CA  LEU C 725     118.023  66.937 137.220  1.00 30.00           C
ATOM   7690  C   LEU C 725     117.240  67.479 138.432  1.00 30.00           C
ATOM   7691  O   LEU C 725     116.075  67.119 138.599  1.00 30.00           O
ATOM   7692  CB  LEU C 725     119.546  66.798 137.487  1.00 20.00           C
ATOM   7693  CG  LEU C 725     120.340  66.068 136.377  1.00  0.00           C
ATOM   7694  CD1 LEU C 725     121.857  66.122 136.659  1.00  0.00           C
ATOM   7695  CD2 LEU C 725     119.843  64.625 136.142  1.00  0.00           C
ATOM   7696  H   LEU C 725     118.607  68.307 135.739  1.00  0.00           H
ATOM   7697  HA  LEU C 725     117.620  65.942 137.026  1.00  0.00           H
ATOM   7698  HB2 LEU C 725     119.976  67.792 137.625  1.00  0.00           H
ATOM   7699  HB3 LEU C 725     119.702  66.268 138.429  1.00  0.00           H
ATOM   7700  HG  LEU C 725     120.186  66.615 135.446  1.00  0.00           H
ATOM   7701 HD11 LEU C 725     122.393  66.546 135.809  1.00  0.00           H
ATOM   7702 HD12 LEU C 725     122.092  66.741 137.525  1.00  0.00           H
ATOM   7703 HD13 LEU C 725     122.285  65.139 136.854  1.00  0.00           H
ATOM   7704 HD21 LEU C 725     120.658  63.918 135.990  1.00  0.00           H
ATOM   7705 HD22 LEU C 725     119.253  64.255 136.981  1.00  0.00           H
ATOM   7706 HD23 LEU C 725     119.213  64.577 135.254  1.00  0.00           H
ATOM   7707  N   LEU C 726     117.909  68.266 139.290  1.00 30.00           N
ATOM   7708  CA  LEU C 726     117.452  68.575 140.644  1.00 30.00           C
ATOM   7709  C   LEU C 726     118.192  69.789 141.216  1.00 30.00           C
ATOM   7710  O   LEU C 726     119.287  70.112 140.751  1.00 30.00           O
ATOM   7711  CB  LEU C 726     117.665  67.322 141.530  1.00 20.00           C
ATOM   7712  CG  LEU C 726     119.149  66.965 141.850  1.00  0.00           C
ATOM   7713  CD1 LEU C 726     119.602  67.477 143.238  1.00  0.00           C
ATOM   7714  CD2 LEU C 726     119.473  65.471 141.637  1.00  0.00           C
ATOM   7715  H   LEU C 726     118.856  68.550 139.082  1.00  0.00           H
ATOM   7716  HA  LEU C 726     116.390  68.817 140.607  1.00  0.00           H
ATOM   7717  HB2 LEU C 726     117.104  67.454 142.453  1.00  0.00           H
ATOM   7718  HB3 LEU C 726     117.170  66.482 141.046  1.00  0.00           H
ATOM   7719  HG  LEU C 726     119.773  67.473 141.114  1.00  0.00           H
ATOM   7720 HD11 LEU C 726     120.413  68.199 143.134  1.00  0.00           H
ATOM   7721 HD12 LEU C 726     118.803  67.978 143.783  1.00  0.00           H
ATOM   7722 HD13 LEU C 726     119.968  66.678 143.883  1.00  0.00           H
ATOM   7723 HD21 LEU C 726     119.580  64.928 142.575  1.00  0.00           H
ATOM   7724 HD22 LEU C 726     118.712  64.954 141.053  1.00  0.00           H
ATOM   7725 HD23 LEU C 726     120.411  65.357 141.093  1.00  0.00           H
ATOM   7726  N   GLN C 727     117.598  70.385 142.264  1.00 30.00           N
ATOM   7727  CA  GLN C 727     118.262  71.360 143.131  1.00 30.00           C
```

```
ATOM   7728  C   GLN C 727     117.498  71.609 144.448  1.00 30.00           C
ATOM   7729  O   GLN C 727     118.072  72.239 145.336  1.00 30.00           O
ATOM   7730  CB  GLN C 727     118.556  72.697 142.384  1.00 20.00           C
ATOM   7731  CG  GLN C 727     119.617  73.616 143.035  1.00  0.00           C
ATOM   7732  CD  GLN C 727     120.965  72.916 143.206  1.00  0.00           C
ATOM   7733  NE2 GLN C 727     121.175  72.284 144.362  1.00  0.00           N
ATOM   7734  OE1 GLN C 727     121.800  72.936 142.306  1.00  0.00           O
ATOM   7735  H   GLN C 727     116.699  70.049 142.572  1.00  0.00           H
ATOM   7736  HA  GLN C 727     119.193  70.873 143.420  1.00  0.00           H
ATOM   7737  HB2 GLN C 727     118.892  72.502 141.367  1.00  0.00           H
ATOM   7738  HB3 GLN C 727     117.628  73.255 142.263  1.00  0.00           H
ATOM   7739  HG2 GLN C 727     119.767  74.497 142.414  1.00  0.00           H
ATOM   7740  HG3 GLN C 727     119.285  73.999 143.999  1.00  0.00           H
ATOM   7741 HE21 GLN C 727     122.049  71.806 144.529  1.00  0.00           H
ATOM   7742 HE22 GLN C 727     120.473  72.299 145.090  1.00  0.00           H
ATOM   7743  N   VAL C 728     116.239  71.146 144.578  1.00 30.00           N
ATOM   7744  CA  VAL C 728     115.404  71.426 145.753  1.00 30.00           C
ATOM   7745  C   VAL C 728     115.907  70.682 147.009  1.00 30.00           C
ATOM   7746  O   VAL C 728     116.222  71.333 148.005  1.00 30.00           O
ATOM   7747  CB  VAL C 728     113.903  71.059 145.557  1.00 20.00           C
ATOM   7748  CG1 VAL C 728     113.020  71.744 146.614  1.00  0.00           C
ATOM   7749  CG2 VAL C 728     113.334  71.370 144.166  1.00  0.00           C
ATOM   7750  H   VAL C 728     115.793  70.638 143.829  1.00  0.00           H
ATOM   7751  HA  VAL C 728     115.470  72.491 145.973  1.00  0.00           H
ATOM   7752  HB  VAL C 728     113.786  69.985 145.681  1.00  0.00           H
ATOM   7753 HG11 VAL C 728     111.989  71.398 146.545  1.00  0.00           H
ATOM   7754 HG12 VAL C 728     113.368  71.550 147.627  1.00  0.00           H
ATOM   7755 HG13 VAL C 728     113.014  72.825 146.482  1.00  0.00           H
ATOM   7756 HG21 VAL C 728     113.872  70.853 143.373  1.00  0.00           H
ATOM   7757 HG22 VAL C 728     112.297  71.050 144.106  1.00  0.00           H
ATOM   7758 HG23 VAL C 728     113.328  72.435 143.960  1.00  0.00           H
ATOM   7759  N   GLY C 729     115.971  69.342 146.921  1.00 30.00           N
ATOM   7760  CA  GLY C 729     116.424  68.429 147.970  1.00 30.00           C
ATOM   7761  C   GLY C 729     115.535  68.379 149.224  1.00 30.00           C
ATOM   7762  O   GLY C 729     115.937  67.755 150.205  1.00 30.00           O
ATOM   7763  H   GLY C 729     115.692  68.905 146.052  1.00  0.00           H
ATOM   7764  HA2 GLY C 729     117.453  68.659 148.250  1.00  0.00           H
ATOM   7765  HA3 GLY C 729     116.416  67.429 147.547  1.00  0.00           H
ATOM   7766  N   PHE C 730     114.351  69.014 149.211  1.00 30.00           N
ATOM   7767  CA  PHE C 730     113.439  69.067 150.351  1.00 30.00           C
ATOM   7768  C   PHE C 730     112.017  69.293 149.827  1.00 30.00           C
ATOM   7769  O   PHE C 730     111.824  70.043 148.872  1.00 30.00           O
ATOM   7770  CB  PHE C 730     113.869  70.207 151.306  1.00 20.00           C
ATOM   7771  CG  PHE C 730     113.411  70.008 152.740  1.00  0.00           C
ATOM   7772  CD1 PHE C 730     112.088  70.311 153.129  1.00  0.00           C
ATOM   7773  CD2 PHE C 730     114.254  69.335 153.649  1.00  0.00           C
ATOM   7774  CE1 PHE C 730     111.648  69.977 154.404  1.00  0.00           C
ATOM   7775  CE2 PHE C 730     113.804  69.038 154.928  1.00  0.00           C
ATOM   7776  CZ  PHE C 730     112.506  69.359 155.304  1.00  0.00           C
ATOM   7777  H   PHE C 730     114.056  69.513 148.383  1.00  0.00           H
ATOM   7778  HA  PHE C 730     113.474  68.108 150.873  1.00  0.00           H
ATOM   7779  HB2 PHE C 730     114.956  70.310 151.317  1.00  0.00           H
ATOM   7780  HB3 PHE C 730     113.494  71.167 150.946  1.00  0.00           H
ATOM   7781  HD1 PHE C 730     111.406  70.777 152.433  1.00  0.00           H
ATOM   7782  HD2 PHE C 730     115.253  69.047 153.354  1.00  0.00           H
ATOM   7783  HE1 PHE C 730     110.630  70.181 154.699  1.00  0.00           H
ATOM   7784  HE2 PHE C 730     114.458  68.536 155.626  1.00  0.00           H
ATOM   7785  HZ  PHE C 730     112.156  69.109 156.294  1.00  0.00           H
ATOM   7786  N   THR C 731     111.036  68.672 150.492  1.00 30.00           N
ATOM   7787  CA  THR C 731     109.614  68.810 150.185  1.00 30.00           C
ATOM   7788  C   THR C 731     108.828  68.252 151.392  1.00 30.00           C
ATOM   7789  O   THR C 731     109.295  67.301 152.021  1.00 30.00           O
ATOM   7790  CB  THR C 731     109.270  68.034 148.867  1.00 20.00           C
ATOM   7791  CG2 THR C 731     107.912  67.332 148.728  1.00  0.00           C
ATOM   7792  OG1 THR C 731     109.349  68.931 147.787  1.00  0.00           O
ATOM   7793  H   THR C 731     111.255  68.062 151.268  1.00  0.00           H
ATOM   7794  HA  THR C 731     109.416  69.875 150.082  1.00  0.00           H
ATOM   7795  HB  THR C 731     110.032  67.278 148.689  1.00  0.00           H
ATOM   7796  HG1 THR C 731     110.255  69.249 147.711  1.00  0.00           H
ATOM   7797 HG21 THR C 731     107.790  66.908 147.732  1.00  0.00           H
ATOM   7798 HG22 THR C 731     107.849  66.496 149.413  1.00  0.00           H
```

```
ATOM   7799 HG23 THR C 731      107.069  67.998 148.902  1.00  0.00           H
ATOM   7800  N   PRO C 732      107.655  68.850 151.716  1.00 30.00           N
ATOM   7801  CA  PRO C 732      106.790  68.353 152.808  1.00 30.00           C
ATOM   7802  C   PRO C 732      106.242  66.925 152.602  1.00 30.00           C
ATOM   7803  O   PRO C 732      105.999  66.224 153.583  1.00 30.00           O
ATOM   7804  CB  PRO C 732      105.656  69.390 152.875  1.00 20.00           C
ATOM   7805  CG  PRO C 732      105.596  70.002 151.484  1.00 20.00           C
ATOM   7806  CD  PRO C 732      107.054  70.009 151.047  1.00 20.00           C
ATOM   7807  HA  PRO C 732      107.345  68.361 153.748  1.00  0.00           H
ATOM   7808  HB2 PRO C 732      104.695  68.977 153.186  1.00  0.00           H
ATOM   7809  HB3 PRO C 732      105.918  70.160 153.596  1.00  0.00           H
ATOM   7810  HG2 PRO C 732      105.021  69.348 150.826  1.00  0.00           H
ATOM   7811  HG3 PRO C 732      105.134  70.989 151.464  1.00  0.00           H
ATOM   7812  HD2 PRO C 732      107.131  69.982 149.962  1.00  0.00           H
ATOM   7813  HD3 PRO C 732      107.552  70.914 151.394  1.00  0.00           H
ATOM   7814  N   ASP C 733      106.084  66.531 151.330  1.00 30.00           N
ATOM   7815  CA  ASP C 733      105.571  65.240 150.866  1.00 30.00           C
ATOM   7816  C   ASP C 733      106.634  64.115 150.851  1.00 30.00           C
ATOM   7817  O   ASP C 733      106.242  62.957 150.710  1.00 30.00           O
ATOM   7818  CB  ASP C 733      104.917  65.343 149.459  1.00 20.00           C
ATOM   7819  CG  ASP C 733      104.038  66.579 149.207  1.00  0.00           C
ATOM   7820  OD1 ASP C 733      103.403  67.062 150.171  1.00  0.00           O
ATOM   7821  OD2 ASP C 733      103.965  66.981 148.025  1.00  0.00           O1-
ATOM   7822  H   ASP C 733      106.287  67.195 150.597  1.00  0.00           H
ATOM   7823  HA  ASP C 733      104.793  64.924 151.564  1.00  0.00           H
ATOM   7824  HB2 ASP C 733      105.708  65.350 148.707  1.00  0.00           H
ATOM   7825  HB3 ASP C 733      104.309  64.458 149.267  1.00  0.00           H
ATOM   7826  N   GLY C 734      107.935  64.447 150.974  1.00 30.00           N
ATOM   7827  CA  GLY C 734      109.035  63.486 150.860  1.00 30.00           C
ATOM   7828  C   GLY C 734      110.232  64.119 150.131  1.00 30.00           C
ATOM   7829  O   GLY C 734      110.485  65.315 150.251  1.00 30.00           O
ATOM   7830  H   GLY C 734      108.193  65.414 151.112  1.00  0.00           H
ATOM   7831  HA2 GLY C 734      109.347  63.183 151.860  1.00  0.00           H
ATOM   7832  HA3 GLY C 734      108.726  62.584 150.329  1.00  0.00           H
ATOM   7833  N   LYS C 735      111.004  63.279 149.419  1.00 30.00           N
ATOM   7834  CA  LYS C 735      112.235  63.626 148.691  1.00 30.00           C
ATOM   7835  C   LYS C 735      111.993  64.550 147.478  1.00 30.00           C
ATOM   7836  O   LYS C 735      110.908  64.517 146.895  1.00 30.00           O
ATOM   7837  CB  LYS C 735      112.919  62.297 148.287  1.00 20.00           C
ATOM   7838  CG  LYS C 735      114.246  62.432 147.514  1.00  0.00           C
ATOM   7839  CD  LYS C 735      114.941  61.083 147.290  1.00  0.00           C
ATOM   7840  CE  LYS C 735      116.257  61.236 146.516  1.00  0.00           C
ATOM   7841  NZ  LYS C 735      116.919  59.937 146.313  1.00  0.00           N1+
ATOM   7842  H   LYS C 735      110.740  62.306 149.374  1.00  0.00           H
ATOM   7843  HA  LYS C 735      112.890  64.153 149.388  1.00  0.00           H
ATOM   7844  HB2 LYS C 735      113.109  61.722 149.194  1.00  0.00           H
ATOM   7845  HB3 LYS C 735      112.228  61.698 147.691  1.00  0.00           H
ATOM   7846  HG2 LYS C 735      114.065  62.885 146.540  1.00  0.00           H
ATOM   7847  HG3 LYS C 735      114.917  63.104 148.052  1.00  0.00           H
ATOM   7848  HD2 LYS C 735      115.135  60.609 148.253  1.00  0.00           H
ATOM   7849  HD3 LYS C 735      114.270  60.416 146.746  1.00  0.00           H
ATOM   7850  HE2 LYS C 735      116.072  61.690 145.542  1.00  0.00           H
ATOM   7851  HE3 LYS C 735      116.938  61.891 147.061  1.00  0.00           H
ATOM   7852  HZ1 LYS C 735      116.312  59.325 145.787  1.00  0.00           H
ATOM   7853  HZ2 LYS C 735      117.779  60.076 145.802  1.00  0.00           H
ATOM   7854  HZ3 LYS C 735      117.125  59.519 147.209  1.00  0.00           H
ATOM   7855  N   ASP C 736      113.031  65.322 147.097  1.00 30.00           N
ATOM   7856  CA  ASP C 736      112.993  66.207 145.930  1.00 30.00           C
ATOM   7857  C   ASP C 736      114.398  66.421 145.314  1.00 30.00           C
ATOM   7858  O   ASP C 736      114.679  67.500 144.789  1.00 30.00           O
ATOM   7859  CB  ASP C 736      112.230  67.521 146.245  1.00 20.00           C
ATOM   7860  CG  ASP C 736      111.589  68.209 145.035  1.00  0.00           C
ATOM   7861  OD1 ASP C 736      111.793  67.739 143.895  1.00  0.00           O
ATOM   7862  OD2 ASP C 736      110.854  69.189 145.275  1.00  0.00           O1-
ATOM   7863  H   ASP C 736      113.892  65.311 147.625  1.00  0.00           H
ATOM   7864  HA  ASP C 736      112.447  65.664 145.155  1.00  0.00           H
ATOM   7865  HB2 ASP C 736      111.419  67.292 146.931  1.00  0.00           H
ATOM   7866  HB3 ASP C 736      112.893  68.221 146.753  1.00  0.00           H
ATOM   7867  N   ASP C 737      115.245  65.375 145.335  1.00 30.00           N
ATOM   7868  CA  ASP C 737      116.508  65.301 144.581  1.00 30.00           C
ATOM   7869  C   ASP C 737      116.307  64.597 143.224  1.00 30.00           C
```

```
ATOM   7870  O   ASP C 737     117.103  63.724 142.876  1.00 30.00           O
ATOM   7871  CB  ASP C 737     117.679  64.663 145.369  1.00 20.00           C
ATOM   7872  CG  ASP C 737     117.975  65.317 146.712  1.00  0.00           C
ATOM   7873  OD1 ASP C 737     118.593  66.403 146.680  1.00  0.00           O
ATOM   7874  OD2 ASP C 737     117.607  64.708 147.740  1.00  0.00           O1-
ATOM   7875  H   ASP C 737     114.964  64.525 145.801  1.00  0.00           H
ATOM   7876  HA  ASP C 737     116.834  66.315 144.350  1.00  0.00           H
ATOM   7877  HB2 ASP C 737     117.497  63.603 145.537  1.00  0.00           H
ATOM   7878  HB3 ASP C 737     118.604  64.686 144.792  1.00  0.00           H
ATOM   7879  N   TYR C 738     115.252  64.968 142.474  1.00 30.00           N
ATOM   7880  CA  TYR C 738     114.936  64.365 141.172  1.00 30.00           C
ATOM   7881  C   TYR C 738     113.916  65.160 140.330  1.00 30.00           C
ATOM   7882  O   TYR C 738     113.585  64.682 139.244  1.00 30.00           O
ATOM   7883  CB  TYR C 738     114.507  62.879 141.339  1.00 20.00           C
ATOM   7884  CG  TYR C 738     113.177  62.637 142.037  1.00  0.00           C
ATOM   7885  CD1 TYR C 738     113.075  62.763 143.439  1.00  0.00           C
ATOM   7886  CD2 TYR C 738     112.039  62.277 141.286  1.00  0.00           C
ATOM   7887  CE1 TYR C 738     111.839  62.558 144.081  1.00  0.00           C
ATOM   7888  CE2 TYR C 738     110.804  62.067 141.928  1.00  0.00           C
ATOM   7889  CZ  TYR C 738     110.701  62.218 143.325  1.00  0.00           C
ATOM   7890  OH  TYR C 738     109.498  62.040 143.943  1.00  0.00           O
ATOM   7891  H   TYR C 738     114.623  65.681 142.815  1.00  0.00           H
ATOM   7892  HA  TYR C 738     115.855  64.380 140.583  1.00  0.00           H
ATOM   7893  HB2 TYR C 738     114.453  62.418 140.351  1.00  0.00           H
ATOM   7894  HB3 TYR C 738     115.270  62.302 141.859  1.00  0.00           H
ATOM   7895  HD1 TYR C 738     113.941  63.022 144.028  1.00  0.00           H
ATOM   7896  HD2 TYR C 738     112.105  62.173 140.213  1.00  0.00           H
ATOM   7897  HE1 TYR C 738     111.770  62.664 145.153  1.00  0.00           H
ATOM   7898  HE2 TYR C 738     109.933  61.803 141.347  1.00  0.00           H
ATOM   7899  HH  TYR C 738     109.534  62.190 144.890  1.00  0.00           H
ATOM   7900  N   ARG C 739     113.420  66.320 140.804  1.00 30.00           N
ATOM   7901  CA  ARG C 739     112.374  67.094 140.116  1.00 30.00           C
ATOM   7902  C   ARG C 739     112.737  68.583 139.989  1.00 30.00           C
ATOM   7903  O   ARG C 739     113.780  69.015 140.479  1.00 30.00           O
ATOM   7904  CB  ARG C 739     110.991  66.935 140.803  1.00 20.00           C
ATOM   7905  CG  ARG C 739     110.674  65.551 141.402  1.00  0.00           C
ATOM   7906  CD  ARG C 739     109.309  65.499 142.103  1.00  0.00           C
ATOM   7907  NE  ARG C 739     109.288  66.362 143.291  1.00  0.00           N
ATOM   7908  CZ  ARG C 739     108.253  66.535 144.129  1.00  0.00           C
ATOM   7909  NH1 ARG C 739     107.124  65.824 144.001  1.00  0.00           N
ATOM   7910  NH2 ARG C 739     108.352  67.444 145.105  1.00  0.00           N1+
ATOM   7911  H   ARG C 739     113.741  66.701 141.682  1.00  0.00           H
ATOM   7912  HA  ARG C 739     112.270  66.745 139.087  1.00  0.00           H
ATOM   7913  HB2 ARG C 739     110.899  67.680 141.590  1.00  0.00           H
ATOM   7914  HB3 ARG C 739     110.212  67.190 140.083  1.00  0.00           H
ATOM   7915  HG2 ARG C 739     110.747  64.764 140.652  1.00  0.00           H
ATOM   7916  HG3 ARG C 739     111.429  65.327 142.157  1.00  0.00           H
ATOM   7917  HD2 ARG C 739     108.549  65.899 141.431  1.00  0.00           H
ATOM   7918  HD3 ARG C 739     109.020  64.474 142.333  1.00  0.00           H
ATOM   7919  HE  ARG C 739     110.132  66.907 143.454  1.00  0.00           H
ATOM   7920 HH11 ARG C 739     106.353  65.966 144.637  1.00  0.00           H
ATOM   7921 HH12 ARG C 739     107.046  65.132 143.271  1.00  0.00           H
ATOM   7922 HH21 ARG C 739     107.587  67.609 145.744  1.00  0.00           H
ATOM   7923 HH22 ARG C 739     109.190  68.010 145.201  1.00  0.00           H
ATOM   7924  N   TRP C 740     111.834  69.331 139.327  1.00 30.00           N
ATOM   7925  CA  TRP C 740     111.875  70.779 139.092  1.00 30.00           C
ATOM   7926  C   TRP C 740     113.011  71.263 138.168  1.00 30.00           C
ATOM   7927  O   TRP C 740     113.381  72.432 138.238  1.00 30.00           O
ATOM   7928  CB  TRP C 740     111.736  71.582 140.405  1.00 20.00           C
ATOM   7929  CG  TRP C 740     110.430  71.452 141.128  1.00  0.00           C
ATOM   7930  CD1 TRP C 740     110.198  70.672 142.207  1.00  0.00           C
ATOM   7931  CD2 TRP C 740     109.153  72.090 140.817  1.00  0.00           C
ATOM   7932  CE2 TRP C 740     108.213  71.734 141.832  1.00  0.00           C
ATOM   7933  CE3 TRP C 740     108.689  72.943 139.787  1.00  0.00           C
ATOM   7934  NE1 TRP C 740     108.903  70.854 142.639  1.00  0.00           N
ATOM   7935  CZ2 TRP C 740     106.904  72.247 141.858  1.00  0.00           C
ATOM   7936  CZ3 TRP C 740     107.379  73.463 139.800  1.00  0.00           C
ATOM   7937  CH2 TRP C 740     106.491  73.126 140.840  1.00  0.00           C
ATOM   7938  H   TRP C 740     111.009  68.871 138.971  1.00  0.00           H
ATOM   7939  HA  TRP C 740     110.980  70.997 138.509  1.00  0.00           H
ATOM   7940  HB2 TRP C 740     112.524  71.310 141.103  1.00  0.00           H
```

```
ATOM   7941  HB3 TRP C 740      111.863  72.648 140.216  1.00  0.00           H
ATOM   7942  HD1 TRP C 740      110.935  70.031 142.666  1.00  0.00           H
ATOM   7943  HE1 TRP C 740      108.531  70.395 143.461  1.00  0.00           H
ATOM   7944  HE3 TRP C 740      109.350  73.198 138.972  1.00  0.00           H
ATOM   7945  HZ2 TRP C 740      106.223  71.964 142.648  1.00  0.00           H
ATOM   7946  HZ3 TRP C 740      107.054  74.120 139.006  1.00  0.00           H
ATOM   7947  HH2 TRP C 740      105.488  73.527 140.846  1.00  0.00           H
ATOM   7948  N   CYS C 741      113.472  70.367 137.275  1.00 30.00           N
ATOM   7949  CA  CYS C 741      114.115  70.593 135.970  1.00 30.00           C
ATOM   7950  C   CYS C 741      114.939  71.890 135.770  1.00 30.00           C
ATOM   7951  O   CYS C 741      114.657  72.669 134.860  1.00 30.00           O
ATOM   7952  CB  CYS C 741      113.091  70.343 134.841  1.00 20.00           C
ATOM   7953  SG  CYS C 741      111.702  71.518 134.827  1.00 20.00           S
ATOM   7954  H   CYS C 741      113.138  69.422 137.392  1.00  0.00           H
ATOM   7955  HA  CYS C 741      114.849  69.794 135.909  1.00  0.00           H
ATOM   7956  HB2 CYS C 741      112.696  69.328 134.905  1.00  0.00           H
ATOM   7957  HB3 CYS C 741      113.583  70.427 133.879  1.00  0.00           H
ATOM   7958  HG  CYS C 741      112.445  72.607 134.599  1.00  0.00           H
ATOM   7959  N   PHE C 742      115.924  72.103 136.653  1.00 30.00           N
ATOM   7960  CA  PHE C 742      116.599  73.384 136.861  1.00 30.00           C
ATOM   7961  C   PHE C 742      117.405  73.944 135.685  1.00 30.00           C
ATOM   7962  O   PHE C 742      118.177  73.210 135.075  1.00 30.00           O
ATOM   7963  CB  PHE C 742      117.482  73.295 138.118  1.00 20.00           C
ATOM   7964  CG  PHE C 742      116.650  73.312 139.377  1.00  0.00           C
ATOM   7965  CD1 PHE C 742      116.095  72.120 139.878  1.00  0.00           C
ATOM   7966  CD2 PHE C 742      116.216  74.543 139.902  1.00  0.00           C
ATOM   7967  CE1 PHE C 742      115.159  72.171 140.897  1.00  0.00           C
ATOM   7968  CE2 PHE C 742      115.276  74.577 140.914  1.00  0.00           C
ATOM   7969  CZ  PHE C 742      114.728  73.395 141.387  1.00  0.00           C
ATOM   7970  H   PHE C 742      116.096  71.406 137.363  1.00  0.00           H
ATOM   7971  HA  PHE C 742      115.804  74.103 137.062  1.00  0.00           H
ATOM   7972  HB2 PHE C 742      118.088  72.387 138.099  1.00  0.00           H
ATOM   7973  HB3 PHE C 742      118.184  74.129 138.157  1.00  0.00           H
ATOM   7974  HD1 PHE C 742      116.369  71.167 139.448  1.00  0.00           H
ATOM   7975  HD2 PHE C 742      116.577  75.466 139.484  1.00  0.00           H
ATOM   7976  HE1 PHE C 742      114.740  71.254 141.276  1.00  0.00           H
ATOM   7977  HE2 PHE C 742      114.948  75.529 141.303  1.00  0.00           H
ATOM   7978  HZ  PHE C 742      113.950  73.433 142.130  1.00  0.00           H
ATOM   7979  N   ARG C 743      117.261  75.262 135.474  1.00 30.00           N
ATOM   7980  CA  ARG C 743      118.157  76.086 134.667  1.00 30.00           C
ATOM   7981  C   ARG C 743      119.323  76.542 135.560  1.00 30.00           C
ATOM   7982  O   ARG C 743      119.052  77.142 136.598  1.00 30.00           O
ATOM   7983  CB  ARG C 743      117.341  77.283 134.106  1.00 20.00           C
ATOM   7984  CG  ARG C 743      118.129  78.272 133.221  1.00 20.00           C
ATOM   7985  CD  ARG C 743      118.825  79.429 133.963  1.00 20.00           C
ATOM   7986  NE  ARG C 743      119.900  79.996 133.139  1.00 20.00           N
ATOM   7987  CZ  ARG C 743      119.787  80.917 132.172  1.00 20.00           C
ATOM   7988  NH1 ARG C 743      118.628  81.544 131.944  1.00 20.00           N
ATOM   7989  NH2 ARG C 743      120.856  81.205 131.418  1.00 20.00           N1+
ATOM   7990  H   ARG C 743      116.588  75.770 136.032  1.00  0.00           H
ATOM   7991  HA  ARG C 743      118.540  75.498 133.832  1.00  0.00           H
ATOM   7992  HB2 ARG C 743      116.542  76.864 133.496  1.00  0.00           H
ATOM   7993  HB3 ARG C 743      116.842  77.826 134.911  1.00  0.00           H
ATOM   7994  HG2 ARG C 743      118.775  77.776 132.500  1.00  0.00           H
ATOM   7995  HG3 ARG C 743      117.364  78.760 132.615  1.00  0.00           H
ATOM   7996  HD2 ARG C 743      118.116  80.237 134.143  1.00  0.00           H
ATOM   7997  HD3 ARG C 743      119.186  79.143 134.946  1.00  0.00           H
ATOM   7998  HE  ARG C 743      120.808  79.564 133.275  1.00  0.00           H
ATOM   7999 HH11 ARG C 743      118.546  82.222 131.201  1.00  0.00           H
ATOM   8000 HH12 ARG C 743      117.823  81.355 132.524  1.00  0.00           H
ATOM   8001 HH21 ARG C 743      120.793  81.870 130.661  1.00  0.00           H
ATOM   8002 HH22 ARG C 743      121.742  80.756 131.603  1.00  0.00           H
ATOM   8003  N   VAL C 744      120.576  76.303 135.138  1.00 30.00           N
ATOM   8004  CA  VAL C 744      121.775  76.833 135.802  1.00 30.00           C
ATOM   8005  C   VAL C 744      122.101  78.232 135.239  1.00 30.00           C
ATOM   8006  O   VAL C 744      122.158  78.387 134.017  1.00 30.00           O
ATOM   8007  CB  VAL C 744      123.009  75.901 135.602  1.00 20.00           C
ATOM   8008  CG1 VAL C 744      124.348  76.494 136.095  1.00 20.00           C
ATOM   8009  CG2 VAL C 744      122.791  74.535 136.275  1.00 20.00           C
ATOM   8010  H   VAL C 744      120.734  75.774 134.291  1.00  0.00           H
ATOM   8011  HA  VAL C 744      121.590  76.921 136.873  1.00  0.00           H
```

```
ATOM   8012  HB  VAL C 744     123.120  75.708 134.535  1.00  0.00           H
ATOM   8013 HG11 VAL C 744     125.149  75.758 136.030  1.00  0.00           H
ATOM   8014 HG12 VAL C 744     124.666  77.351 135.501  1.00  0.00           H
ATOM   8015 HG13 VAL C 744     124.279  76.814 137.135  1.00  0.00           H
ATOM   8016 HG21 VAL C 744     123.606  73.849 136.041  1.00  0.00           H
ATOM   8017 HG22 VAL C 744     122.752  74.635 137.359  1.00  0.00           H
ATOM   8018 HG23 VAL C 744     121.867  74.059 135.947  1.00  0.00           H
ATOM   8019  N   ASP C 745     122.302  79.210 136.139  1.00 30.00           N
ATOM   8020  CA  ASP C 745     122.547  80.620 135.815  1.00 30.00           C
ATOM   8021  C   ASP C 745     123.749  81.149 136.627  1.00 30.00           C
ATOM   8022  O   ASP C 745     124.227  80.478 137.543  1.00 30.00           O
ATOM   8023  CB  ASP C 745     121.273  81.468 136.099  1.00 20.00           C
ATOM   8024  CG  ASP C 745     121.144  82.764 135.283  1.00  0.00           C
ATOM   8025  OD1 ASP C 745     121.845  82.884 134.255  1.00  0.00           O
ATOM   8026  OD2 ASP C 745     120.295  83.594 135.673  1.00  0.00           O1-
ATOM   8027  H   ASP C 745     122.240  79.000 137.127  1.00  0.00           H
ATOM   8028  HA  ASP C 745     122.831  80.689 134.764  1.00  0.00           H
ATOM   8029  HB2 ASP C 745     120.383  80.871 135.909  1.00  0.00           H
ATOM   8030  HB3 ASP C 745     121.253  81.737 137.157  1.00  0.00           H
ATOM   8031  N   GLU C 746     124.197  82.365 136.272  1.00 30.00           N
ATOM   8032  CA  GLU C 746     125.272  83.113 136.925  1.00 30.00           C
ATOM   8033  C   GLU C 746     124.871  84.592 137.080  1.00 30.00           C
ATOM   8034  O   GLU C 746     124.022  85.085 136.335  1.00 30.00           O
ATOM   8035  CB  GLU C 746     126.587  82.923 136.136  1.00 20.00           C
ATOM   8036  CG  GLU C 746     126.584  83.492 134.698  1.00  0.00           C
ATOM   8037  CD  GLU C 746     127.886  83.250 133.928  1.00  0.00           C
ATOM   8038  OE1 GLU C 746     128.869  82.786 134.548  1.00  0.00           O
ATOM   8039  OE2 GLU C 746     127.875  83.546 132.713  1.00  0.00           O1-
ATOM   8040  H   GLU C 746     123.730  82.853 135.517  1.00  0.00           H
ATOM   8041  HA  GLU C 746     125.425  82.721 137.929  1.00  0.00           H
ATOM   8042  HB2 GLU C 746     127.399  83.383 136.702  1.00  0.00           H
ATOM   8043  HB3 GLU C 746     126.823  81.858 136.104  1.00  0.00           H
ATOM   8044  HG2 GLU C 746     125.768  83.052 134.124  1.00  0.00           H
ATOM   8045  HG3 GLU C 746     126.409  84.568 134.714  1.00  0.00           H
ATOM   8046  N   VAL C 747     125.491  85.262 138.066  1.00 30.00           N
ATOM   8047  CA  VAL C 747     125.230  86.656 138.443  1.00 30.00           C
ATOM   8048  C   VAL C 747     126.578  87.376 138.678  1.00 30.00           C
ATOM   8049  O   VAL C 747     127.576  86.718 138.964  1.00 30.00           O
ATOM   8050  CB  VAL C 747     124.356  86.716 139.738  1.00 20.00           C
ATOM   8051  CG1 VAL C 747     124.124  88.123 140.326  1.00  0.00           C
ATOM   8052  CG2 VAL C 747     122.981  86.047 139.530  1.00  0.00           C
ATOM   8053  H   VAL C 747     126.198  84.793 138.614  1.00  0.00           H
ATOM   8054  HA  VAL C 747     124.715  87.176 137.633  1.00  0.00           H
ATOM   8055  HB  VAL C 747     124.873  86.147 140.510  1.00  0.00           H
ATOM   8056 HG11 VAL C 747     123.400  88.096 141.140  1.00  0.00           H
ATOM   8057 HG12 VAL C 747     125.032  88.548 140.747  1.00  0.00           H
ATOM   8058 HG13 VAL C 747     123.743  88.811 139.571  1.00  0.00           H
ATOM   8059 HG21 VAL C 747     122.372  86.102 140.433  1.00  0.00           H
ATOM   8060 HG22 VAL C 747     122.426  86.529 138.725  1.00  0.00           H
ATOM   8061 HG23 VAL C 747     123.072  84.990 139.282  1.00  0.00           H
ATOM   8062  N   ASN C 748     126.591  88.713 138.538  1.00 30.00           N
ATOM   8063  CA  ASN C 748     127.769  89.577 138.725  1.00 30.00           C
ATOM   8064  C   ASN C 748     128.118  89.809 140.212  1.00 30.00           C
ATOM   8065  O   ASN C 748     127.414  89.326 141.098  1.00 30.00           O
ATOM   8066  CB  ASN C 748     127.538  90.926 137.998  1.00 20.00           C
ATOM   8067  CG  ASN C 748     127.250  90.775 136.500  1.00  0.00           C
ATOM   8068  ND2 ASN C 748     128.246  90.345 135.722  1.00  0.00           N
ATOM   8069  OD1 ASN C 748     126.139  91.045 136.052  1.00  0.00           O
ATOM   8070  H   ASN C 748     125.733  89.189 138.298  1.00  0.00           H
ATOM   8071  HA  ASN C 748     128.632  89.083 138.274  1.00  0.00           H
ATOM   8072  HB2 ASN C 748     126.707  91.460 138.462  1.00  0.00           H
ATOM   8073  HB3 ASN C 748     128.410  91.574 138.098  1.00  0.00           H
ATOM   8074 HD21 ASN C 748     128.095  90.232 134.730  1.00  0.00           H
ATOM   8075 HD22 ASN C 748     129.149  90.121 136.114  1.00  0.00           H
ATOM   8076  N   TRP C 749     129.219  90.546 140.451  1.00 30.00           N
ATOM   8077  CA  TRP C 749     129.761  90.872 141.774  1.00 30.00           C
ATOM   8078  C   TRP C 749     128.797  91.682 142.658  1.00 30.00           C
ATOM   8079  O   TRP C 749     128.322  91.154 143.662  1.00 30.00           O
ATOM   8080  CB  TRP C 749     131.132  91.576 141.651  1.00 20.00           C
ATOM   8081  CG  TRP C 749     132.310  90.738 141.239  1.00  0.00           C
ATOM   8082  CD1 TRP C 749     132.339  89.809 140.253  1.00  0.00           C
```

```
ATOM   8083  CD2 TRP C 749     133.668  90.778 141.781  1.00  0.00           C
ATOM   8084  CE2 TRP C 749     134.461  89.810 141.094  1.00  0.00           C
ATOM   8085  CE3 TRP C 749     134.311  91.526 142.797  1.00  0.00           C
ATOM   8086  NE1 TRP C 749     133.597  89.251 140.176  1.00  0.00           N
ATOM   8087  CZ2 TRP C 749     135.816  89.590 141.401  1.00  0.00           C
ATOM   8088  CZ3 TRP C 749     135.669  91.315 143.114  1.00  0.00           C
ATOM   8089  CH2 TRP C 749     136.421  90.348 142.420  1.00  0.00           C
ATOM   8090  H   TRP C 749     129.744  90.904 139.668  1.00  0.00           H
ATOM   8091  HA  TRP C 749     129.927  89.931 142.297  1.00  0.00           H
ATOM   8092  HB2 TRP C 749     131.063  92.420 140.964  1.00  0.00           H
ATOM   8093  HB3 TRP C 749     131.396  92.004 142.619  1.00  0.00           H
ATOM   8094  HD1 TRP C 749     131.499  89.534 139.633  1.00  0.00           H
ATOM   8095  HE1 TRP C 749     133.832  88.525 139.514  1.00  0.00           H
ATOM   8096  HE3 TRP C 749     133.750  92.268 143.347  1.00  0.00           H
ATOM   8097  HZ2 TRP C 749     136.385  88.848 140.861  1.00  0.00           H
ATOM   8098  HZ3 TRP C 749     136.134  91.896 143.896  1.00  0.00           H
ATOM   8099  HH2 TRP C 749     137.460  90.190 142.667  1.00  0.00           H
ATOM   8100  N   THR C 750     128.550  92.949 142.274  1.00 30.00           N
ATOM   8101  CA  THR C 750     127.675  93.927 142.946  1.00 30.00           C
ATOM   8102  C   THR C 750     128.109  94.332 144.384  1.00 30.00           C
ATOM   8103  O   THR C 750     127.335  94.986 145.082  1.00 30.00           O
ATOM   8104  CB  THR C 750     126.168  93.509 142.926  1.00 20.00           C
ATOM   8105  CG2 THR C 750     125.679  92.967 141.572  1.00  0.00           C
ATOM   8106  OG1 THR C 750     125.804  92.610 143.958  1.00  0.00           O
ATOM   8107  H   THR C 750     128.989  93.285 141.429  1.00  0.00           H
ATOM   8108  HA  THR C 750     127.763  94.839 142.355  1.00  0.00           H
ATOM   8109  HB  THR C 750     125.587  94.413 143.116  1.00  0.00           H
ATOM   8110  HG1 THR C 750     126.338  91.814 143.868  1.00  0.00           H
ATOM   8111 HG21 THR C 750     124.599  92.814 141.581  1.00  0.00           H
ATOM   8112 HG22 THR C 750     125.904  93.665 140.765  1.00  0.00           H
ATOM   8113 HG23 THR C 750     126.139  92.011 141.324  1.00  0.00           H
ATOM   8114  N   THR C 751     129.349  93.973 144.780  1.00 30.00           N
ATOM   8115  CA  THR C 751     130.011  94.172 146.085  1.00 30.00           C
ATOM   8116  C   THR C 751     129.416  93.188 147.080  1.00 30.00           C
ATOM   8117  O   THR C 751     129.719  93.185 148.273  1.00 30.00           O
ATOM   8118  CB  THR C 751     130.111  95.648 146.603  1.00 20.00           C
ATOM   8119  CG2 THR C 751     130.619  96.645 145.548  1.00  0.00           C
ATOM   8120  OG1 THR C 751     128.895  96.148 147.132  1.00  0.00           O
ATOM   8121  H   THR C 751     129.903  93.455 144.114  1.00  0.00           H
ATOM   8122  HA  THR C 751     131.036  93.844 145.911  1.00  0.00           H
ATOM   8123  HB  THR C 751     130.822  95.664 147.431  1.00  0.00           H
ATOM   8124  HG1 THR C 751     128.215  96.064 146.456  1.00  0.00           H
ATOM   8125 HG21 THR C 751     130.747  97.639 145.979  1.00  0.00           H
ATOM   8126 HG22 THR C 751     131.584  96.333 145.150  1.00  0.00           H
ATOM   8127 HG23 THR C 751     129.927  96.737 144.710  1.00  0.00           H
ATOM   8128  HXT THR C 751     128.706  92.466 146.675  1.00  0.00           H
END
```

S3. Series C

Coordinates of compounds from Series C in Sdf file.

```
C_1a
                   3D
 Structure written by MMmdl.
 39 40  0  0  1  0            999 V2000
   93.9841  116.9379  128.4737 C   0  0  0  0  0  0
   90.7402  118.8413  124.7966 C   0  0  0  0  0  0
   95.0084  116.1264  127.4178 S   0  0  0  0  0  0
   93.3836  118.1346  128.2146 N   0  0  0  0  0  0
   93.6704  116.5162  129.7329 N   0  0  0  0  0  0
   91.3888  118.8692  123.5547 C   0  0  0  0  0  0
   91.5203  118.9163  125.9609 C   0  0  0  0  0  0
   92.7820  118.8986  123.4743 C   0  0  0  0  0  0
   92.9181  118.9552  125.8848 C   0  0  0  0  0  0
   93.7589  119.0686  127.1455 C   0  0  0  0  0  0
   94.4589  115.5821  130.5518 C   0  0  0  0  0  0
   97.4386  117.4256  133.0734 C   0  0  0  0  0  0
   95.4996  116.2560  131.4469 C   0  0  0  0  0  0
   93.5448  118.9165  124.6379 C   0  0  0  0  0  0
   89.3631  118.7180  124.7866 O   0  0  0  0  0  0
   98.6778  118.1190  134.0462 Cl  0  0  0  0  0  0
   90.6598  118.8732  122.3947 O   0  0  0  0  0  0
   97.2536  117.8929  131.7762 C   0  0  0  0  0  0
   96.6522  116.3927  133.5747 C   0  0  0  0  0  0
   96.2804  117.3153  130.9681 C   0  0  0  0  0  0
   95.6826  115.8072  132.7603 C   0  0  0  0  0  0
   88.6277  118.7724  125.9971 C   0  0  0  0  0  0
   92.9829  117.0648  130.2401 H   0  0  0  0  0  0
   91.0599  118.9159  126.9347 H   0  0  0  0  0  0
   93.2815  118.9173  122.5155 H   0  0  0  0  0  0
   93.6053  120.0757  127.5324 H   0  0  0  0  0  0
   94.8230  118.9970  126.9124 H   0  0  0  0  0  0
   94.9490  114.8149  129.9505 H   0  0  0  0  0  0
   93.7486  115.0518  131.1876 H   0  0  0  0  0  0
   94.6236  118.9426  124.5647 H   0  0  0  0  0  0
   89.7019  118.8814  122.5521 H   0  0  0  0  0  0
   97.8669  118.6968  131.3952 H   0  0  0  0  0  0
   96.8056  116.0295  134.5805 H   0  0  0  0  0  0
   96.1663  117.6855  129.9613 H   0  0  0  0  0  0
   95.0878  114.9934  133.1479 H   0  0  0  0  0  0
   87.5856  118.5190  125.7973 H   0  0  0  0  0  0
   88.6491  119.7848  126.3951 H   0  0  0  0  0  0
   88.9943  118.0710  126.7484 H   0  0  0  0  0  0
   92.6777  118.4523  128.8699 H   0  0  0  0  0  0
  1  3  2  0  0  0
  1  4  1  0  0  0
  1  5  1  0  0  0
  2  7  2  0  0  0
  2 15  1  0  0  0
  2  6  1  0  0  0
  4 10  1  0  0  0
  4 39  1  0  0  0
  5 11  1  0  0  0
  5 23  1  0  0  0
  6  8  2  0  0  0
  6 17  1  0  0  0
  7  9  1  0  0  0
  7 24  1  0  0  0
  8 14  1  0  0  0
  8 25  1  0  0  0
```

```
  9 10  1  0  0  0
  9 14  2  0  0  0
 10 26  1  0  0  0
 10 27  1  0  0  0
 11 13  1  0  0  0
 11 28  1  0  0  0
 11 29  1  0  0  0
 12 19  2  0  0  0
 12 16  1  0  0  0
 12 18  1  0  0  0
 13 20  1  0  0  0
 13 21  2  0  0  0
 14 30  1  0  0  0
 15 22  1  0  0  0
 17 31  1  0  0  0
 18 20  2  0  0  0
 18 32  1  0  0  0
 19 21  1  0  0  0
 19 33  1  0  0  0
 20 34  1  0  0  0
 21 35  1  0  0  0
 22 36  1  0  0  0
 22 37  1  0  0  0
 22 38  1  0  0  0
M  END
> <s_m_entry_id>
45

> <s_m_entry_name>
C_1a.1

> <s_m_Source_Path>
C:\Users\julio\OneDrive - Universidad de Talca\Escritorio\Nueva carpeta

> <s_m_Source_File>
C_1a.mol2

> <i_m_Source_File_Index>
1

$$$$
C_1b
                   3D
 Structure written by MMmdl.
 42 43  0  0  1  0            999 V2000
   93.6729  116.8518  128.3106 C   0  0  0  0  0  0
   90.6978  118.7956  124.8023 C   0  0  0  0  0  0
   94.6820  116.1173  127.1926 S   0  0  0  0  0  0
   93.2890  118.1582  128.2513 N   0  0  0  0  0  0
   91.3656  118.7903  123.5690 C   0  0  0  0  0  0
   91.4609  118.9165  125.9735 C   0  0  0  0  0  0
   93.1672  116.2475  129.4258 N   0  0  0  0  0  0
   92.7595  118.8456  123.5121 C   0  0  0  0  0  0
   92.8559  118.9831  125.9200 C   0  0  0  0  0  0
   93.6631  119.1177  127.2027 C   0  0  0  0  0  0
   96.8894  117.3236  133.0149 C   0  0  0  0  0  0
   93.5042  118.9190  124.6863 C   0  0  0  0  0  0
   89.3214  118.6546  124.7900 O   0  0  0  0  0  0
   95.4559  115.6390  131.3443 C   0  0  0  0  0  0
   97.9067  118.2961  134.0028 Cl  0  0  0  0  0  0
   90.6598  118.7291  122.3951 O   0  0  0  0  0  0
   96.7282  117.6660  131.6778 C   0  0  0  0  0  0
   96.2706  116.1896  133.5420 C   0  0  0  0  0  0
   96.0019  116.8271  130.8438 C   0  0  0  0  0  0
   95.5439  115.3484  132.7068 C   0  0  0  0  0  0
   93.4364  114.8755  129.8739 C   0  0  0  0  0  0
   94.8653  114.6002  130.3952 C   0  0  0  0  0  0
   88.5789  118.7475  125.9980 C   0  0  0  0  0  0
   90.9901  118.9165  126.9427 H   0  0  0  0  0  0
   92.6188  116.8213  130.0550 H   0  0  0  0  0  0
   93.2763  118.8373  122.5638 H   0  0  0  0  0  0
```

```
   94.7342  119.0744  127.0000 H   0  0  0  0  0  0
   93.4776  120.1170  127.5911 H   0  0  0  0  0  0
   94.5830  118.9590  124.6309 H   0  0  0  0  0  0
   89.6984  118.7067  122.5378 H   0  0  0  0  0  0
   97.2111  118.5431  131.2748 H   0  0  0  0  0  0
   96.4095  115.9315  134.5803 H   0  0  0  0  0  0
   95.9566  117.0604  129.7926 H   0  0  0  0  0  0
   95.1199  114.4378  133.1050 H   0  0  0  0  0  0
   92.7303  114.6790  130.6815 H   0  0  0  0  0  0
   93.1844  114.1703  129.0817 H   0  0  0  0  0  0
   94.8876  113.6170  130.8677 H   0  0  0  0  0  0
   95.5517  114.4999  129.5546 H   0  0  0  0  0  0
   87.5318  118.5203  125.7965 H   0  0  0  0  0  0
   88.6235  119.7606  126.3936 H   0  0  0  0  0  0
   88.9222  118.0420  126.7554 H   0  0  0  0  0  0
   92.7256  118.5124  129.0155 H   0  0  0  0  0  0
  1  3  2  0  0  0
  1  4  1  0  0  0
  1  7  1  0  0  0
  2  6  2  0  0  0
  2 13  1  0  0  0
  2  5  1  0  0  0
  4 10  1  0  0  0
  4 42  1  0  0  0
  5  8  2  0  0  0
  5 16  1  0  0  0
  6  9  1  0  0  0
  6 24  1  0  0  0
  7 21  1  0  0  0
  7 25  1  0  0  0
  8 12  1  0  0  0
  8 26  1  0  0  0
  9 10  1  0  0  0
  9 12  2  0  0  0
 10 27  1  0  0  0
 10 28  1  0  0  0
 11 18  2  0  0  0
 11 15  1  0  0  0
 11 17  1  0  0  0
 12 29  1  0  0  0
 13 23  1  0  0  0
 14 22  1  0  0  0
 14 19  1  0  0  0
 14 20  2  0  0  0
 16 30  1  0  0  0
 17 19  2  0  0  0
 17 31  1  0  0  0
 18 20  1  0  0  0
 18 32  1  0  0  0
 19 33  1  0  0  0
 20 34  1  0  0  0
 21 22  1  0  0  0
 21 35  1  0  0  0
 21 36  1  0  0  0
 22 37  1  0  0  0
 22 38  1  0  0  0
 23 39  1  0  0  0
 23 40  1  0  0  0
 23 41  1  0  0  0
M  END
> <s_m_entry_id>
46

> <s_m_entry_name>
C_1b.1

> <s_m_Source_Path>
C:\Users\julio\OneDrive - Universidad de Talca\Escritorio\Nueva carpeta

> <s_m_Source_File>
C_1b.mol2
```

```
> <i_m_Source_File_Index>
1

$$$$
C_1c
                    3D
 Structure written by MMmdl.
 40 41  0  0  1  0            999 V2000
   94.1914  116.5849  128.0372 C   0  0  0  0  0  0
   90.8303  118.8791  124.7984 C   0  0  0  0  0  0
   95.2581  116.3398  126.7950 S   0  0  0  0  0  0
   93.4690  117.7254  128.2589 N   0  0  0  0  0  0
   93.5724  115.5582  130.1622 C   0  0  0  0  0  0
   91.5920  118.8991  123.6208 C   0  0  0  0  0  0
   94.0395  115.5407  128.8921 N   0  0  0  0  0  0
   91.4963  118.9159  126.0321 C   0  0  0  0  0  0
   93.8233  114.5742  131.0416 C   0  0  0  0  0  0
   92.9854  118.9157  123.6801 C   0  0  0  0  0  0
   92.8888  118.9146  126.0981 C   0  0  0  0  0  0
   93.4165  114.5664  132.4535 C   0  0  0  0  0  0
   93.5718  118.9555  127.4589 C   0  0  0  0  0  0
   92.6788  114.5150  135.1318 C   0  0  0  0  0  0
   93.6315  118.9162  124.9138 C   0  0  0  0  0  0
   89.4580  118.8110  124.6734 O   0  0  0  0  0  0
   92.2598  114.5168  136.7946 Cl  0  0  0  0  0  0
   90.9728  118.8964  122.3960 O   0  0  0  0  0  0
   93.0295  115.7423  133.1004 C   0  0  0  0  0  0
   93.4279  113.3579  133.1594 C   0  0  0  0  0  0
   93.0590  113.3322  134.5024 C   0  0  0  0  0  0
   92.6613  115.7166  134.4369 C   0  0  0  0  0  0
   88.6402  118.7249  125.8369 C   0  0  0  0  0  0
   92.9046  117.7818  129.0982 H   0  0  0  0  0  0
   92.9971  116.4373  130.4130 H   0  0  0  0  0  0
   94.5042  114.6856  128.6228 H   0  0  0  0  0  0
   90.9473  118.9136  126.9590 H   0  0  0  0  0  0
   94.4020  113.7223  130.7114 H   0  0  0  0  0  0
   93.5714  118.9245  122.7722 H   0  0  0  0  0  0
   94.6067  119.2874  127.3768 H   0  0  0  0  0  0
   93.0617  119.7168  128.0334 H   0  0  0  0  0  0
   94.7109  118.9272  124.9471 H   0  0  0  0  0  0
   90.0111  118.8752  122.5099 H   0  0  0  0  0  0
   93.0228  116.6910  132.5855 H   0  0  0  0  0  0
   93.7280  112.4394  132.6746 H   0  0  0  0  0  0
   93.0747  112.4034  135.0537 H   0  0  0  0  0  0
   92.3764  116.6272  134.9389 H   0  0  0  0  0  0
   87.6084  118.5393  125.5378 H   0  0  0  0  0  0
   88.6524  119.6618  126.3947 H   0  0  0  0  0  0
   88.9452  117.9069  126.4912 H   0  0  0  0  0  0
  1  3  2  0  0  0
  1  4  1  0  0  0
  1  7  1  0  0  0
  2  8  2  0  0  0
  2 16  1  0  0  0
  2  6  1  0  0  0
  4 13  1  0  0  0
  4 24  1  0  0  0
  5  7  1  0  0  0
  5  9  2  0  0  0
  5 25  1  0  0  0
  6 10  2  0  0  0
  6 18  1  0  0  0
  7 26  1  0  0  0
  8 11  1  0  0  0
  8 27  1  0  0  0
  9 12  1  0  0  0
  9 28  1  0  0  0
 10 15  1  0  0  0
 10 29  1  0  0  0
 11 13  1  0  0  0
 11 15  2  0  0  0
```

```
 12 19  2  0  0  0
 12 20  1  0  0  0
 13 30  1  0  0  0
 13 31  1  0  0  0
 14 21  1  0  0  0
 14 17  1  0  0  0
 14 22  2  0  0  0
 15 32  1  0  0  0
 16 23  1  0  0  0
 18 33  1  0  0  0
 19 22  1  0  0  0
 19 34  1  0  0  0
 20 21  2  0  0  0
 20 35  1  0  0  0
 21 36  1  0  0  0
 22 37  1  0  0  0
 23 38  1  0  0  0
 23 39  1  0  0  0
 23 40  1  0  0  0
M  END
> <s_m_entry_id>
47

> <s_m_entry_name>
C_1c.1

> <s_m_Source_Path>
C:\Users\julio\OneDrive - Universidad de Talca\Escritorio\Nueva carpeta

> <s_m_Source_File>
C_1c.mol2

> <i_m_Source_File_Index>
1

$$$$
C_1d
                    3D
 Structure written by MMmdl.
 40 41  0  0  1  0            999 V2000
   93.8845  116.9946  128.2999 C   0  0  0  0  0  0
   90.7579  118.7592  124.7750 C   0  0  0  0  0  0
   94.8531  116.1764  127.1952 S   0  0  0  0  0  0
   93.4591  118.2805  128.1611 N   0  0  0  0  0  0
   93.6820  115.2420  130.0013 C   0  0  0  0  0  0
   91.3930  118.7320  123.5263 C   0  0  0  0  0  0
   93.4593  116.4791  129.4893 N   0  0  0  0  0  0
   91.5517  118.9169  125.9206 C   0  0  0  0  0  0
   94.5071  114.9276  131.0188 C   0  0  0  0  0  0
   92.7815  118.8055  123.4226 C   0  0  0  0  0  0
   92.9457  119.0014  125.8201 C   0  0  0  0  0  0
   95.4301  115.8099  131.7651 C   0  0  0  0  0  0
   93.8017  119.1843  127.0613 C   0  0  0  0  0  0
   97.3131  117.2889  133.1906 C   0  0  0  0  0  0
   93.5579  118.9168  124.5699 C   0  0  0  0  0  0
   89.3817  118.6079  124.7953 O   0  0  0  0  0  0
   98.5692  118.1174  134.0260 Cl  0  0  0  0  0  0
   90.6599  118.6345  122.3770 O   0  0  0  0  0  0
   95.7165  115.4851  133.0924 C   0  0  0  0  0  0
   96.0596  116.9166  131.1763 C   0  0  0  0  0  0
   96.9961  117.6585  131.8887 C   0  0  0  0  0  0
   96.6639  116.2155  133.7991 C   0  0  0  0  0  0
   88.6648  118.6921  126.0212 C   0  0  0  0  0  0
   92.8469  118.6512  128.8775 H   0  0  0  0  0  0
   93.0866  114.4949  129.4934 H   0  0  0  0  0  0
   92.9730  117.1176  130.1113 H   0  0  0  0  0  0
   91.0988  118.9494  126.8986 H   0  0  0  0  0  0
   94.5384  113.8857  131.3066 H   0  0  0  0  0  0
   93.2695  118.7842  122.4602 H   0  0  0  0  0  0
   94.8654  119.1256  126.8230 H   0  0  0  0  0  0
   93.6266  120.1945  127.4220 H   0  0  0  0  0  0
```

```
   94.6344  118.9719  124.4806 H   0  0  0  0  0  0
   89.7029  118.6180  122.5406 H   0  0  0  0  0  0
   95.2420  114.6381  133.5671 H   0  0  0  0  0  0
   95.8796  117.1817  130.1492 H   0  0  0  0  0  0
   97.5025  118.4889  131.4185 H   0  0  0  0  0  0
   96.9248  115.9316  134.8070 H   0  0  0  0  0  0
   87.6271  118.4089  125.8495 H   0  0  0  0  0  0
   88.6600  119.7150  126.3951 H   0  0  0  0  0  0
   89.0582  118.0283  126.7932 H   0  0  0  0  0  0
  1  3  2  0  0  0
  1  4  1  0  0  0
  1  7  1  0  0  0
  2  8  2  0  0  0
  2 16  1  0  0  0
  2  6  1  0  0  0
  4 13  1  0  0  0
  4 24  1  0  0  0
  5  7  1  0  0  0
  5  9  2  0  0  0
  5 25  1  0  0  0
  6 10  2  0  0  0
  6 18  1  0  0  0
  7 26  1  0  0  0
  8 11  1  0  0  0
  8 27  1  0  0  0
  9 12  1  0  0  0
  9 28  1  0  0  0
 10 15  1  0  0  0
 10 29  1  0  0  0
 11 13  1  0  0  0
 11 15  2  0  0  0
 12 19  2  0  0  0
 12 20  1  0  0  0
 13 30  1  0  0  0
 13 31  1  0  0  0
 14 21  1  0  0  0
 14 17  1  0  0  0
 14 22  2  0  0  0
 15 32  1  0  0  0
 16 23  1  0  0  0
 18 33  1  0  0  0
 19 22  1  0  0  0
 19 34  1  0  0  0
 20 21  2  0  0  0
 20 35  1  0  0  0
 21 36  1  0  0  0
 22 37  1  0  0  0
 23 38  1  0  0  0
 23 39  1  0  0  0
 23 40  1  0  0  0
M  END
> <s_m_entry_id>
48

> <s_m_entry_name>
C_1d.1

> <s_m_Source_Path>
C:\Users\julio\OneDrive - Universidad de Talca\Escritorio\Nueva carpeta

> <s_m_Source_File>
C_1d.mol2

> <i_m_Source_File_Index>
1

$$$$
C_1e
                    3D
 Structure written by MMmdl.
 42 43  0  0  1  0            999 V2000
```

```
   94.0257  117.0041  128.2549 C   0  0  0  0  0  0
   90.8226  118.7926  124.7824 C   0  0  0  0  0  0
   95.0618  116.3848  127.0795 S   0  0  0  0  0  0
   93.4866  118.2608  128.2377 N   0  0  0  0  0  0
   91.5194  118.6533  123.5730 C   0  0  0  0  0  0
   91.5613  118.9918  125.9645 C   0  0  0  0  0  0
   93.6071  116.3159  129.3596 N   0  0  0  0  0  0
   92.9136  118.7145  123.5447 C   0  0  0  0  0  0
   92.9596  119.0405  125.9400 C   0  0  0  0  0  0
   93.7423  119.2901  127.2209 C   0  0  0  0  0  0
   97.2959  117.5537  132.7815 C   0  0  0  0  0  0
   93.6311  118.9121  124.7222 C   0  0  0  0  0  0
   89.4399  118.7261  124.7305 O   0  0  0  0  0  0
   95.3362  115.8271  131.8173 C   0  0  0  0  0  0
   98.2479  118.3925  133.2498 F   0  0  0  0  0  0
   90.8417  118.4575  122.3959 O   0  0  0  0  0  0
   96.8100  116.5344  133.5985 C   0  0  0  0  0  0
   96.8084  117.7159  131.4859 C   0  0  0  0  0  0
   95.8295  115.6703  133.1167 C   0  0  0  0  0  0
   95.8280  116.8524  131.0030 C   0  0  0  0  0  0
   94.0747  114.9845  129.7649 C   0  0  0  0  0  0
   94.2645  114.8810  131.2891 C   0  0  0  0  0  0
   88.6761  118.8610  125.9254 C   0  0  0  0  0  0
   91.0645  119.1088  126.9160 H   0  0  0  0  0  0
   92.9177  116.7514  129.9555 H   0  0  0  0  0  0
   93.4446  118.6113  122.6092 H   0  0  0  0  0  0
   94.8096  119.3100  126.9941 H   0  0  0  0  0  0
   93.4816  120.2848  127.5858 H   0  0  0  0  0  0
   94.7098  118.9626  124.6836 H   0  0  0  0  0  0
   89.8975  118.3385  122.5222 H   0  0  0  0  0  0
   97.1912  116.4137  134.6020 H   0  0  0  0  0  0
   97.1884  118.5079  130.8572 H   0  0  0  0  0  0
   95.4564  114.8824  133.7551 H   0  0  0  0  0  0
   95.4536  116.9835  129.9979 H   0  0  0  0  0  0
   93.3560  114.2299  129.4405 H   0  0  0  0  0  0
   95.0216  114.7628  129.2694 H   0  0  0  0  0  0
   93.3219  115.0891  131.7990 H   0  0  0  0  0  0
   94.5241  113.8574  131.5657 H   0  0  0  0  0  0
   87.6170  118.7795  125.6808 H   0  0  0  0  0  0
   88.8266  119.8346  126.3945 H   0  0  0  0  0  0
   88.9048  118.0718  126.6434 H   0  0  0  0  0  0
   92.6961  118.4292  128.8433 H   0  0  0  0  0  0
  1  3  2  0  0  0
  1  4  1  0  0  0
  1  7  1  0  0  0
  2  6  2  0  0  0
  2 13  1  0  0  0
  2  5  1  0  0  0
  4 10  1  0  0  0
  4 42  1  0  0  0
  5  8  2  0  0  0
  5 16  1  0  0  0
  6  9  1  0  0  0
  6 24  1  0  0  0
  7 21  1  0  0  0
  7 25  1  0  0  0
  8 12  1  0  0  0
  8 26  1  0  0  0
  9 10  1  0  0  0
  9 12  2  0  0  0
 10 27  1  0  0  0
 10 28  1  0  0  0
 11 18  2  0  0  0
 11 15  1  0  0  0
 11 17  1  0  0  0
 12 29  1  0  0  0
 13 23  1  0  0  0
 14 22  1  0  0  0
 14 19  1  0  0  0
 14 20  2  0  0  0
 16 30  1  0  0  0
```

```
 17 19  2  0  0  0
 17 31  1  0  0  0
 18 20  1  0  0  0
 18 32  1  0  0  0
 19 33  1  0  0  0
 20 34  1  0  0  0
 21 22  1  0  0  0
 21 35  1  0  0  0
 21 36  1  0  0  0
 22 37  1  0  0  0
 22 38  1  0  0  0
 23 39  1  0  0  0
 23 40  1  0  0  0
 23 41  1  0  0  0
M  END
> <s_m_entry_id>
49

> <s_m_entry_name>
C_1e.1

> <s_m_Source_Path>
C:\Users\julio\OneDrive - Universidad de Talca\Escritorio\Nueva carpeta

> <s_m_Source_File>
C_1e.mol2

> <i_m_Source_File_Index>
1

$$$$
C_1f
                    3D
 Structure written by MMmdl.
 42 43  0  0  1  0            999 V2000
   93.9655  116.8747  128.3023 C   0  0  0  0  0  0
   95.8595  115.4067  133.4096 C   0  0  0  0  0  0
   96.9065  116.1313  133.9926 C   0  0  0  0  0  0
   90.7224  118.7913  124.8028 C   0  0  0  0  0  0
   95.0597  116.2462  127.1949 S   0  0  0  0  0  0
   93.4096  118.1171  128.2145 N   0  0  0  0  0  0
   91.3793  118.7848  123.5637 C   0  0  0  0  0  0
   95.3627  115.7620  132.1387 C   0  0  0  0  0  0
   91.4924  118.9165  125.9705 C   0  0  0  0  0  0
   93.5382  116.2297  129.4285 N   0  0  0  0  0  0
   92.7734  118.8421  123.4987 C   0  0  0  0  0  0
   95.9391  116.8611  131.4833 C   0  0  0  0  0  0
   97.4589  117.2246  133.3171 C   0  0  0  0  0  0
   92.8874  118.9775  125.9084 C   0  0  0  0  0  0
   95.2304  114.0516  134.2833 Cl  0  0  0  0  0  0
   93.7064  119.1229  127.1819 C   0  0  0  0  0  0
   96.9758  117.5857  132.0655 C   0  0  0  0  0  0
   93.5249  118.9164  124.6681 C   0  0  0  0  0  0
   89.3464  118.6504  124.7922 O   0  0  0  0  0  0
   98.7690  118.1161  133.9959 Cl  0  0  0  0  0  0
   90.6611  118.7225  122.3951 O   0  0  0  0  0  0
   94.0875  114.9745  129.9635 C   0  0  0  0  0  0
   94.2047  114.9712  131.5049 C   0  0  0  0  0  0
   88.6097  118.7419  126.0024 C   0  0  0  0  0  0
   97.2943  115.8459  134.9591 H   0  0  0  0  0  0
   91.0269  118.9166  126.9437 H   0  0  0  0  0  0
   92.7581  116.6327  129.9313 H   0  0  0  0  0  0
   93.2851  118.8301  122.5488 H   0  0  0  0  0  0
   95.6023  117.1810  130.5136 H   0  0  0  0  0  0
   94.7755  119.1547  126.9598 H   0  0  0  0  0  0
   93.4657  120.0994  127.5954 H   0  0  0  0  0  0
   97.4084  118.4249  131.5392 H   0  0  0  0  0  0
   94.6038  118.9577  124.6062 H   0  0  0  0  0  0
   89.7010  118.6994  122.5455 H   0  0  0  0  0  0
   93.4225  114.1699  129.6480 H   0  0  0  0  0  0
   95.0691  114.7342  129.5530 H   0  0  0  0  0  0
```

```
   93.2679  115.3058  131.9538 H   0  0  0  0  0  0
   94.3131  113.9248  131.7914 H   0  0  0  0  0  0
   87.5616  118.5170  125.8069 H   0  0  0  0  0  0
   88.6574  119.7561  126.3951 H   0  0  0  0  0  0
   88.9550  118.0363  126.7583 H   0  0  0  0  0  0
   92.7791  118.4000  128.9547 H   0  0  0  0  0  0
  1  5  2  0  0  0
  1  6  1  0  0  0
  1 10  1  0  0  0
  2  8  2  0  0  0
  2  3  1  0  0  0
  2 15  1  0  0  0
  3 13  2  0  0  0
  3 25  1  0  0  0
  4  9  2  0  0  0
  4 19  1  0  0  0
  4  7  1  0  0  0
  6 16  1  0  0  0
  6 42  1  0  0  0
  7 11  2  0  0  0
  7 21  1  0  0  0
  8 23  1  0  0  0
  8 12  1  0  0  0
  9 14  1  0  0  0
  9 26  1  0  0  0
 10 22  1  0  0  0
 10 27  1  0  0  0
 11 18  1  0  0  0
 11 28  1  0  0  0
 12 17  2  0  0  0
 12 29  1  0  0  0
 13 17  1  0  0  0
 13 20  1  0  0  0
 14 16  1  0  0  0
 14 18  2  0  0  0
 16 30  1  0  0  0
 16 31  1  0  0  0
 17 32  1  0  0  0
 18 33  1  0  0  0
 19 24  1  0  0  0
 21 34  1  0  0  0
 22 23  1  0  0  0
 22 35  1  0  0  0
 22 36  1  0  0  0
 23 37  1  0  0  0
 23 38  1  0  0  0
 24 39  1  0  0  0
 24 40  1  0  0  0
 24 41  1  0  0  0
M  END
> <s_m_entry_id>
50

> <s_m_entry_name>
C_1f.1

> <s_m_Source_Path>
C:\Users\julio\OneDrive - Universidad de Talca\Escritorio\Nueva carpeta

> <s_m_Source_File>
C_1f.mol2

> <i_m_Source_File_Index>
1

$$$$
C_1g
                    3D
 Structure written by MMmdl.
 42 43  0  0  1  0            999 V2000
   93.9379  116.8827  128.2823 C   0  0  0  0  0  0
```

90.7207	118.7996	124.8011	C	0	0	0	0	0	0
95.0524	116.2534	127.1906	S	0	0	0	0	0	0
93.4003	118.1325	128.2016	N	0	0	0	0	0	0
91.3789	118.8034	123.5644	C	0	0	0	0	0	0
91.4925	118.9152	125.9691	C	0	0	0	0	0	0
93.4912	116.2426	129.4028	N	0	0	0	0	0	0
92.7719	118.8553	123.4968	C	0	0	0	0	0	0
92.8881	118.9850	125.9018	C	0	0	0	0	0	0
93.7060	119.1350	127.1742	C	0	0	0	0	0	0
93.5255	118.9249	124.6633	C	0	0	0	0	0	0
89.3456	118.6496	124.7939	O	0	0	0	0	0	0
90.6608	118.7553	122.3953	O	0	0	0	0	0	0
94.0720	115.0271	129.9901	C	0	0	0	0	0	0
95.5741	116.1095	131.8729	C	0	0	0	0	0	0
94.4050	115.1909	131.4903	C	0	0	0	0	0	0
88.6086	118.7402	126.0016	C	0	0	0	0	0	0
95.9906	116.1165	133.2070	C	0	0	0	0	0	0
96.2457	116.9203	130.9510	C	0	0	0	0	0	0
97.3111	117.7184	131.3485	C	0	0	0	0	0	0
97.0618	116.9163	133.6057	C	0	0	0	0	0	0
97.7127	117.7206	132.6783	C	0	0	0	0	0	0
91.0260	118.9174	126.9398	H	0	0	0	0	0	0
92.7099	116.6561	129.8965	H	0	0	0	0	0	0
93.2811	118.8513	122.5451	H	0	0	0	0	0	0
94.7759	119.1747	126.9577	H	0	0	0	0	0	0
93.4593	120.1096	127.5910	H	0	0	0	0	0	0
94.6058	118.9576	124.6008	H	0	0	0	0	0	0
89.7033	118.7445	122.5465	H	0	0	0	0	0	0
93.3423	114.2243	129.8719	H	0	0	0	0	0	0
94.9721	114.6879	129.4744	H	0	0	0	0	0	0
93.5170	115.5254	132.0294	H	0	0	0	0	0	0
94.6407	114.2014	131.8848	H	0	0	0	0	0	0
87.5598	118.5182	125.8029	H	0	0	0	0	0	0
88.6589	119.7532	126.3952	H	0	0	0	0	0	0
88.9538	118.0371	126.7587	H	0	0	0	0	0	0
95.4907	115.4979	133.9396	H	0	0	0	0	0	0
95.9830	116.9304	129.9103	H	0	0	0	0	0	0
97.8228	118.3389	130.6242	H	0	0	0	0	0	0
97.3820	116.9141	134.6388	H	0	0	0	0	0	0
98.5417	118.3397	132.9868	H	0	0	0	0	0	0
92.8146	118.4352	128.9698	H	0	0	0	0	0	0

1	3	2	0	0	0
1	4	1	0	0	0
1	7	1	0	0	0
2	6	2	0	0	0
2	12	1	0	0	0
2	5	1	0	0	0
4	10	1	0	0	0
4	42	1	0	0	0
5	8	2	0	0	0
5	13	1	0	0	0
6	9	1	0	0	0
6	23	1	0	0	0
7	14	1	0	0	0
7	24	1	0	0	0
8	11	1	0	0	0
8	25	1	0	0	0
9	10	1	0	0	0
9	11	2	0	0	0
10	26	1	0	0	0
10	27	1	0	0	0
11	28	1	0	0	0
12	17	1	0	0	0
13	29	1	0	0	0
14	16	1	0	0	0
14	30	1	0	0	0
14	31	1	0	0	0
15	16	1	0	0	0
15	18	2	0	0	0
15	19	1	0	0	0
16	32	1	0	0	0

```
 16 33  1  0  0  0
 17 34  1  0  0  0
 17 35  1  0  0  0
 17 36  1  0  0  0
 18 21  1  0  0  0
 18 37  1  0  0  0
 19 20  2  0  0  0
 19 38  1  0  0  0
 20 22  1  0  0  0
 20 39  1  0  0  0
 21 22  2  0  0  0
 21 40  1  0  0  0
 22 41  1  0  0  0
M  END
> <s_m_entry_id>
51

> <s_m_entry_name>
C_1g.1

> <s_m_Source_Path>
C:\Users\julio\OneDrive - Universidad de Talca\Escritorio\Nueva carpeta

> <s_m_Source_File>
C_1g.mol2

> <i_m_Source_File_Index>
1

$$$$
C_1h
                    3D
 Structure written by MMmdl.
 45 46  0  0  1  0            999 V2000
   93.8468  116.8427  128.1987 C   0  0  0  0  0  0
   90.7207  118.7814  124.7993 C   0  0  0  0  0  0
   95.0589  116.2553  127.1956 S   0  0  0  0  0  0
   93.3896  118.1281  128.1859 N   0  0  0  0  0  0
   91.3762  118.7696  123.5596 C   0  0  0  0  0  0
   91.4944  118.9164  125.9611 C   0  0  0  0  0  0
   93.1993  116.1102  129.1506 N   0  0  0  0  0  0
   92.7689  118.8291  123.4869 C   0  0  0  0  0  0
   92.8891  118.9906  125.8914 C   0  0  0  0  0  0
   93.7055  119.1406  127.1687 C   0  0  0  0  0  0
   98.0746  117.7153  133.3318 C   0  0  0  0  0  0
   93.5256  118.9188  124.6519 C   0  0  0  0  0  0
   89.3443  118.6326  124.7953 O   0  0  0  0  0  0
   99.4593  118.5209  133.9648 Cl  0  0  0  0  0  0
   95.8599  116.3673  132.3282 C   0  0  0  0  0  0
   90.6597  118.6999  122.3957 O   0  0  0  0  0  0
   97.4596  116.7241  134.0925 C   0  0  0  0  0  0
   97.6032  118.0194  132.0577 C   0  0  0  0  0  0
   96.3451  116.0558  133.5950 C   0  0  0  0  0  0
   96.5006  117.3351  131.5510 C   0  0  0  0  0  0
   93.5985  114.7715  129.6225 C   0  0  0  0  0  0
   94.5882  115.7055  131.8179 C   0  0  0  0  0  0
   88.6097  118.7336  126.0065 C   0  0  0  0  0  0
   94.8085  114.8052  130.5829 C   0  0  0  0  0  0
   91.0370  118.9247  126.9371 H   0  0  0  0  0  0
   92.4596  116.5666  129.6663 H   0  0  0  0  0  0
   93.2746  118.8149  122.5332 H   0  0  0  0  0  0
   94.7754  119.1714  126.9552 H   0  0  0  0  0  0
   93.4604  120.1164  127.5949 H   0  0  0  0  0  0
   94.6034  118.9670  124.5846 H   0  0  0  0  0  0
   89.6985  118.6855  122.5462 H   0  0  0  0  0  0
   97.8396  116.4797  135.0730 H   0  0  0  0  0  0
   98.0877  118.7813  131.4644 H   0  0  0  0  0  0
   95.8631  115.3040  134.2019 H   0  0  0  0  0  0
   96.1266  117.5830  130.5690 H   0  0  0  0  0  0
   92.7396  114.3452  130.1436 H   0  0  0  0  0  0
   93.7937  114.1063  128.7794 H   0  0  0  0  0  0
```

```
   93.8951  116.5165  131.5924 H   0  0  0  0  0  0
   94.1003  115.1349  132.6112 H   0  0  0  0  0  0
   87.5607  118.5126  125.8107 H   0  0  0  0  0  0
   88.6565  119.7482  126.3918 H   0  0  0  0  0  0
   88.9545  118.0296  126.7644 H   0  0  0  0  0  0
   95.0512  113.7929  130.9102 H   0  0  0  0  0  0
   95.6800  115.1414  130.0204 H   0  0  0  0  0  0
   92.7901  118.4191  128.9481 H   0  0  0  0  0  0
  1  3  2  0  0  0
  1  4  1  0  0  0
  1  7  1  0  0  0
  2  6  2  0  0  0
  2 13  1  0  0  0
  2  5  1  0  0  0
  4 10  1  0  0  0
  4 45  1  0  0  0
  5  8  2  0  0  0
  5 16  1  0  0  0
  6  9  1  0  0  0
  6 25  1  0  0  0
  7 21  1  0  0  0
  7 26  1  0  0  0
  8 12  1  0  0  0
  8 27  1  0  0  0
  9 10  1  0  0  0
  9 12  2  0  0  0
 10 28  1  0  0  0
 10 29  1  0  0  0
 11 18  2  0  0  0
 11 14  1  0  0  0
 11 17  1  0  0  0
 12 30  1  0  0  0
 13 23  1  0  0  0
 15 22  1  0  0  0
 15 19  1  0  0  0
 15 20  2  0  0  0
 16 31  1  0  0  0
 17 19  2  0  0  0
 17 32  1  0  0  0
 18 20  1  0  0  0
 18 33  1  0  0  0
 19 34  1  0  0  0
 20 35  1  0  0  0
 21 24  1  0  0  0
 21 36  1  0  0  0
 21 37  1  0  0  0
 22 24  1  0  0  0
 22 38  1  0  0  0
 22 39  1  0  0  0
 23 40  1  0  0  0
 23 41  1  0  0  0
 23 42  1  0  0  0
 24 43  1  0  0  0
 24 44  1  0  0  0
M  END
> <s_m_entry_id>
52

> <s_m_entry_name>
C_1h.1

> <s_m_Source_Path>
C:\Users\julio\OneDrive - Universidad de Talca\Escritorio\Nueva carpeta

> <s_m_Source_File>
C_1h.mol2

> <i_m_Source_File_Index>
1

$$$$
```

```
C_1i
                    3D
 Structure written by MMmdl.
 51 52  0  0  1  0            999 V2000
   92.7181  114.1667  134.2857 C   0  0  0  0  0  0
   92.7429  115.4732  133.7740 C   0  0  0  0  0  0
   93.1022  115.7165  132.4516 C   0  0  0  0  0  0
   93.4595  114.6615  131.6069 C   0  0  0  0  0  0
   93.4558  113.3601  132.1077 C   0  0  0  0  0  0
   93.0865  113.1148  133.4300 C   0  0  0  0  0  0
   93.8285  114.8912  130.1391 C   0  0  0  0  0  0
   93.4118  116.1927  129.5941 N   0  0  0  0  0  0
   93.9265  116.8028  128.4876 C   0  0  0  0  0  0
   93.4018  118.0571  128.3388 N   0  0  0  0  0  0
   95.0596  116.1192  127.4577 S   0  0  0  0  0  0
   93.7667  119.0409  127.3066 C   0  0  0  0  0  0
   92.9762  118.9308  126.0083 C   0  0  0  0  0  0
   93.6452  118.8824  124.7848 C   0  0  0  0  0  0
   92.9285  118.7970  123.5955 C   0  0  0  0  0  0
   91.5343  118.7373  123.6216 C   0  0  0  0  0  0
   90.8396  118.7710  124.8402 C   0  0  0  0  0  0
   91.5775  118.8923  126.0302 C   0  0  0  0  0  0
   89.4594  118.6560  124.7952 O   0  0  0  0  0  0
   88.6916  118.7046  125.9948 C   0  0  0  0  0  0
   90.8523  118.6349  122.4348 O   0  0  0  0  0  0
   92.2801  113.8551  135.7413 C   0  0  0  0  0  0
   93.4291  113.1406  136.4964 C   0  0  0  0  0  0
   91.0349  112.9287  135.7431 C   0  0  0  0  0  0
   91.8957  115.1327  136.5309 C   0  0  0  0  0  0
   92.4789  116.3241  134.3793 H   0  0  0  0  0  0
   93.0967  116.7350  132.0936 H   0  0  0  0  0  0
   93.7209  112.5286  131.4693 H   0  0  0  0  0  0
   93.0805  112.0949  133.7848 H   0  0  0  0  0  0
   94.9115  114.7700  130.0566 H   0  0  0  0  0  0
   93.3797  114.1023  129.5309 H   0  0  0  0  0  0
   92.5999  116.6188  130.0220 H   0  0  0  0  0  0
   92.7323  118.3642  129.0353 H   0  0  0  0  0  0
   93.5504  120.0310  127.7097 H   0  0  0  0  0  0
   94.8406  119.0273  127.1117 H   0  0  0  0  0  0
   94.7241  118.9186  124.7517 H   0  0  0  0  0  0
   93.4626  118.7732  122.6560 H   0  0  0  0  0  0
   91.0806  118.9170  126.9879 H   0  0  0  0  0  0
   87.6622  118.4242  125.7706 H   0  0  0  0  0  0
   88.6652  119.7160  126.3954 H   0  0  0  0  0  0
   89.0539  118.0146  126.7594 H   0  0  0  0  0  0
   89.9011  118.5685  122.5859 H   0  0  0  0  0  0
   94.3543  113.7178  136.4734 H   0  0  0  0  0  0
   93.6559  112.1624  136.0658 H   0  0  0  0  0  0
   93.1805  112.9706  137.5451 H   0  0  0  0  0  0
   90.1644  113.4220  135.3087 H   0  0  0  0  0  0
   90.7532  112.6350  136.7557 H   0  0  0  0  0  0
   91.2031  112.0074  135.1843 H   0  0  0  0  0  0
   91.0632  115.6582  136.0600 H   0  0  0  0  0  0
   92.7235  115.8319  136.6036 H   0  0  0  0  0  0
   91.5893  114.9170  137.5545 H   0  0  0  0  0  0
  1  6  2  0  0  0
  1  2  1  0  0  0
  1 22  1  0  0  0
  2  3  2  0  0  0
  2 26  1  0  0  0
  3  4  1  0  0  0
  3 27  1  0  0  0
  4  7  1  0  0  0
  4  5  2  0  0  0
  5  6  1  0  0  0
  5 28  1  0  0  0
  6 29  1  0  0  0
  7  8  1  0  0  0
  7 30  1  0  0  0
  7 31  1  0  0  0
  8  9  1  0  0  0
```

```
  8 32  1  0  0  0
  9 10  1  0  0  0
  9 11  2  0  0  0
 10 12  1  0  0  0
 10 33  1  0  0  0
 12 13  1  0  0  0
 12 34  1  0  0  0
 12 35  1  0  0  0
 13 14  2  0  0  0
 13 18  1  0  0  0
 14 15  1  0  0  0
 14 36  1  0  0  0
 15 16  2  0  0  0
 15 37  1  0  0  0
 16 17  1  0  0  0
 16 21  1  0  0  0
 17 18  2  0  0  0
 17 19  1  0  0  0
 18 38  1  0  0  0
 19 20  1  0  0  0
 20 39  1  0  0  0
 20 40  1  0  0  0
 20 41  1  0  0  0
 21 42  1  0  0  0
 22 23  1  0  0  0
 22 24  1  0  0  0
 22 25  1  0  0  0
 23 43  1  0  0  0
 23 44  1  0  0  0
 23 45  1  0  0  0
 24 46  1  0  0  0
 24 47  1  0  0  0
 24 48  1  0  0  0
 25 49  1  0  0  0
 25 50  1  0  0  0
 25 51  1  0  0  0
M  END
> <s_m_entry_id>
53

> <s_m_entry_name>
C_1i.1

> <s_m_Source_Path>
C:\Users\julio\OneDrive - Universidad de Talca\Escritorio\Nueva carpeta

> <s_m_Source_File>
C_1i.mol2

> <i_m_Source_File_Index>
1

$$$$
C_2a
                    3D
 Structure written by MMmdl.
 51 52  0  0  1  0            999 V2000
   94.4651  115.9644  132.2667 C   0  0  0  0  0  0
   93.7843  117.0556  127.3822 C   0  0  0  0  0  0
   95.4769  114.8039  131.5958 S   0  0  0  0  0  0
   93.2092  116.2386  131.8073 N   0  0  0  0  0  0
   92.6439  117.7515  126.9538 C   0  0  0  0  0  0
   93.7339  116.3470  128.5957 C   0  0  0  0  0  0
   94.7803  116.7540  133.3387 N   0  0  0  0  0  0
   91.5166  117.7943  127.7839 C   0  0  0  0  0  0
   92.5902  116.3645  129.3971 C   0  0  0  0  0  0
   92.5311  115.5637  130.6884 C   0  0  0  0  0  0
   93.6981  114.7318  138.0079 C   0  0  0  0  0  0
   91.4812  117.0995  128.9867 C   0  0  0  0  0  0
   94.8975  117.0744  126.5588 O   0  0  0  0  0  0
   95.5136  115.3364  135.9934 C   0  0  0  0  0  0
```

```
   92.5697  114.3694  139.2582 Cl  0  0  0  0  0  0
   94.7814  115.5633  138.2793 C   0  0  0  0  0  0
   93.5191  114.1934  136.7350 C   0  0  0  0  0  0
   89.0827  118.7630  125.2695 N   0  3  0  0  0  0
   92.6666  118.3837  125.7252 O   0  0  0  0  0  0
   94.4316  114.4928  135.7269 C   0  0  0  0  0  0
   95.6912  115.8698  137.2720 C   0  0  0  0  0  0
   96.0970  116.8533  133.9936 C   0  0  0  0  0  0
   96.5106  115.6646  134.8936 C   0  0  0  0  0  0
   90.4926  119.3518  125.0853 C   0  0  0  0  0  0
   91.5261  118.2266  124.8763 C   0  0  0  0  0  0
   96.1396  116.5953  127.0710 C   0  0  0  0  0  0
   94.5814  115.7694  128.9262 H   0  0  0  0  0  0
   94.0627  117.3745  133.6875 H   0  0  0  0  0  0
   90.6489  118.3613  127.5102 H   0  0  0  0  0  0
   92.9071  114.5557  130.5092 H   0  0  0  0  0  0
   91.4868  115.4289  130.9672 H   0  0  0  0  0  0
   90.5886  117.1351  129.5956 H   0  0  0  0  0  0
   94.9163  115.9682  139.2690 H   0  0  0  0  0  0
   92.6780  113.5474  136.5305 H   0  0  0  0  0  0
   88.4308  119.4876  125.5368 H   0  0  0  0  0  0
   88.7496  118.3629  124.3977 H   0  0  0  0  0  0
   94.2946  114.0741  134.7393 H   0  0  0  0  0  0
   96.5297  116.5158  137.4883 H   0  0  0  0  0  0
   96.0554  117.7476  134.6169 H   0  0  0  0  0  0
   96.8680  117.0641  133.2545 H   0  0  0  0  0  0
   97.4813  115.8750  135.3421 H   0  0  0  0  0  0
   96.6775  114.7659  134.3013 H   0  0  0  0  0  0
   90.6848  119.9535  125.9750 H   0  0  0  0  0  0
   90.4145  120.0089  124.2200 H   0  0  0  0  0  0
   91.8961  118.2900  123.8526 H   0  0  0  0  0  0
   91.0990  117.2242  124.9589 H   0  0  0  0  0  0
   96.9290  116.7565  126.3398 H   0  0  0  0  0  0
   96.1053  115.5250  127.2727 H   0  0  0  0  0  0
   96.4281  117.1209  127.9828 H   0  0  0  0  0  0
   92.7839  117.1060  132.1093 H   0  0  0  0  0  0
   89.0745  118.0532  125.9907 H   0  0  0  0  0  0
  1  3  2  0  0  0
  1  4  1  0  0  0
  1  7  1  0  0  0
  2  6  2  0  0  0
  2 13  1  0  0  0
  2  5  1  0  0  0
  4 10  1  0  0  0
  4 50  1  0  0  0
  5  8  2  0  0  0
  5 19  1  0  0  0
  6  9  1  0  0  0
  6 27  1  0  0  0
  7 22  1  0  0  0
  7 28  1  0  0  0
  8 12  1  0  0  0
  8 29  1  0  0  0
  9 10  1  0  0  0
  9 12  2  0  0  0
 10 30  1  0  0  0
 10 31  1  0  0  0
 11 16  2  0  0  0
 11 15  1  0  0  0
 11 17  1  0  0  0
 12 32  1  0  0  0
 13 26  1  0  0  0
 14 23  1  0  0  0
 14 20  1  0  0  0
 14 21  2  0  0  0
 16 21  1  0  0  0
 16 33  1  0  0  0
 17 20  2  0  0  0
 17 34  1  0  0  0
 18 24  1  0  0  0
 18 35  1  0  0  0
```

```
 18 36  1  0  0  0
 18 51  1  0  0  0
 19 25  1  0  0  0
 20 37  1  0  0  0
 21 38  1  0  0  0
 22 23  1  0  0  0
 22 39  1  0  0  0
 22 40  1  0  0  0
 23 41  1  0  0  0
 23 42  1  0  0  0
 24 25  1  0  0  0
 24 43  1  0  0  0
 24 44  1  0  0  0
 25 45  1  0  0  0
 25 46  1  0  0  0
 26 47  1  0  0  0
 26 48  1  0  0  0
 26 49  1  0  0  0
M  CHG  1  18   1
M  END
> <s_m_entry_id>
54

> <s_m_entry_name>
C_2a.1

> <s_m_Source_Path>
C:\Users\julio\OneDrive - Universidad de Talca\Escritorio\Nueva carpeta

> <s_m_Source_File>
C_2a.mol2

> <i_m_Source_File_Index>
1

$$$$
C_2b
                    3D
 Structure written by MMmdl.
 51 52  0  0  1  0            999 V2000
   95.1159  116.6038  133.7439 C   0  0  0  0  0  0
   94.1412  116.6940  128.0215 C   0  0  0  0  0  0
   95.4315  117.9616  134.6903 S   0  0  0  0  0  0
   94.2272  116.5647  132.7052 N   0  0  0  0  0  0
   92.7840  117.0064  127.8371 C   0  0  0  0  0  0
   94.5763  116.2205  129.2736 C   0  0  0  0  0  0
   95.7150  115.3863  133.9110 N   0  0  0  0  0  0
   91.8877  116.8358  128.9076 C   0  0  0  0  0  0
   93.6807  116.0670  130.3348 C   0  0  0  0  0  0
   94.1605  115.5220  131.6721 C   0  0  0  0  0  0
   92.3324  116.3663  130.1411 C   0  0  0  0  0  0
   94.9770  116.8759  126.9317 O   0  0  0  0  0  0
   89.1713  118.5384  125.2819 N   0  3  0  0  0  0
   92.4132  117.4687  126.5844 O   0  0  0  0  0  0
   96.7832  115.0842  134.8718 C   0  0  0  0  0  0
   95.0880  115.2688  136.7708 C   0  0  0  0  0  0
   96.4760  115.6503  136.2700 C   0  0  0  0  0  0
   90.6611  118.2262  125.1206 C   0  0  0  0  0  0
   91.3861  118.4497  126.4546 C   0  0  0  0  0  0
   96.3692  116.5951  127.0445 C   0  0  0  0  0  0
   94.8625  115.0896  138.1396 C   0  0  0  0  0  0
   94.0388  115.0968  135.8618 C   0  0  0  0  0  0
   92.7708  114.7468  136.3199 C   0  0  0  0  0  0
   93.5941  114.7396  138.5966 C   0  0  0  0  0  0
   92.5543  114.5697  137.6849 C   0  0  0  0  0  0
   95.6123  115.9678  129.4426 H   0  0  0  0  0  0
   95.4044  114.6220  133.3284 H   0  0  0  0  0  0
   90.8365  117.0589  128.8074 H   0  0  0  0  0  0
   95.1500  115.0787  131.5480 H   0  0  0  0  0  0
   93.4898  114.7128  131.9653 H   0  0  0  0  0  0
   91.6229  116.2390  130.9462 H   0  0  0  0  0  0
```

```
   88.6897  118.3927  124.4062 H   0  0  0  0  0  0
   88.7646  117.9351  125.9825 H   0  0  0  0  0  0
   97.7258  115.5024  134.5144 H   0  0  0  0  0  0
   96.9160  114.0033  134.9434 H   0  0  0  0  0  0
   96.5658  116.7383  136.2619 H   0  0  0  0  0  0
   97.2221  115.3030  136.9872 H   0  0  0  0  0  0
   91.0075  118.8903  124.3282 H   0  0  0  0  0  0
   90.7005  117.1913  124.7797 H   0  0  0  0  0  0
   90.6725  118.3701  127.2772 H   0  0  0  0  0  0
   91.8103  119.4556  126.4770 H   0  0  0  0  0  0
   96.8513  116.8011  126.0888 H   0  0  0  0  0  0
   96.5529  115.5457  127.2806 H   0  0  0  0  0  0
   96.8505  117.2264  127.7931 H   0  0  0  0  0  0
   95.6654  115.2207  138.8508 H   0  0  0  0  0  0
   94.2012  115.2337  134.8023 H   0  0  0  0  0  0
   91.9589  114.6134  135.6201 H   0  0  0  0  0  0
   93.4174  114.6006  139.6530 H   0  0  0  0  0  0
   91.5708  114.2983  138.0397 H   0  0  0  0  0  0
   93.7172  117.4132  132.5056 H   0  0  0  0  0  0
   89.0473  119.4994  125.5668 H   0  0  0  0  0  0
  1  3  2  0  0  0
  1  4  1  0  0  0
  1  7  1  0  0  0
  2  6  2  0  0  0
  2 12  1  0  0  0
  2  5  1  0  0  0
  4 10  1  0  0  0
  4 50  1  0  0  0
  5  8  2  0  0  0
  5 14  1  0  0  0
  6  9  1  0  0  0
  6 26  1  0  0  0
  7 15  1  0  0  0
  7 27  1  0  0  0
  8 11  1  0  0  0
  8 28  1  0  0  0
  9 10  1  0  0  0
  9 11  2  0  0  0
 10 29  1  0  0  0
 10 30  1  0  0  0
 11 31  1  0  0  0
 12 20  1  0  0  0
 13 18  1  0  0  0
 13 32  1  0  0  0
 13 33  1  0  0  0
 13 51  1  0  0  0
 14 19  1  0  0  0
 15 17  1  0  0  0
 15 34  1  0  0  0
 15 35  1  0  0  0
 16 17  1  0  0  0
 16 21  2  0  0  0
 16 22  1  0  0  0
 17 36  1  0  0  0
 17 37  1  0  0  0
 18 19  1  0  0  0
 18 38  1  0  0  0
 18 39  1  0  0  0
 19 40  1  0  0  0
 19 41  1  0  0  0
 20 42  1  0  0  0
 20 43  1  0  0  0
 20 44  1  0  0  0
 21 24  1  0  0  0
 21 45  1  0  0  0
 22 23  2  0  0  0
 22 46  1  0  0  0
 23 25  1  0  0  0
 23 47  1  0  0  0
 24 25  2  0  0  0
 24 48  1  0  0  0
```

```
 25 49  1  0  0  0
M  CHG  1  13   1
M  END
> <s_m_entry_id>
55

> <s_m_entry_name>
C_2b.1

> <s_m_Source_Path>
C:\Users\julio\OneDrive - Universidad de Talca\Escritorio\Nueva carpeta

> <s_m_Source_File>
C_2b.mol2

> <i_m_Source_File_Index>
1

$$$$
C_2c
                    3D
 Structure written by MMmdl.
 51 52  0  0  1  0            999 V2000
   94.7125  116.3266  133.5056 C   0  0  0  0  0  0
   94.0505  116.7654  127.7125 C   0  0  0  0  0  0
   95.8771  115.1634  133.8178 S   0  0  0  0  0  0
   93.9420  116.3632  132.3797 N   0  0  0  0  0  0
   92.7112  117.1632  127.5416 C   0  0  0  0  0  0
   94.4523  116.2133  128.9432 C   0  0  0  0  0  0
   94.3958  117.3577  134.3434 N   0  0  0  0  0  0
   91.7946  116.9353  128.5824 C   0  0  0  0  0  0
   93.5335  116.0107  129.9732 C   0  0  0  0  0  0
   93.9667  115.3849  131.2863 C   0  0  0  0  0  0
   92.3794  113.6321  137.7387 C   0  0  0  0  0  0
   92.2002  116.3580  129.7790 C   0  0  0  0  0  0
   94.9110  116.9176  126.6371 O   0  0  0  0  0  0
   94.2121  115.6248  137.0573 C   0  0  0  0  0  0
   91.4885  112.6714  138.0662 F   0  0  0  0  0  0
   92.0483  114.5720  136.7683 C   0  0  0  0  0  0
   93.6180  113.6763  138.3731 C   0  0  0  0  0  0
   88.8014  118.3752  125.6694 N   0  3  0  0  0  0
   92.3340  117.6896  126.3156 O   0  0  0  0  0  0
   94.5311  114.6716  138.0314 C   0  0  0  0  0  0
   92.9636  115.5628  136.4258 C   0  0  0  0  0  0
   95.0761  117.7334  135.5967 C   0  0  0  0  0  0
   95.2649  116.6994  136.7512 C   0  0  0  0  0  0
   90.2407  118.2044  125.1565 C   0  0  0  0  0  0
   91.2484  118.6193  126.2476 C   0  0  0  0  0  0
   96.2283  116.3721  126.7147 C   0  0  0  0  0  0
   95.4779  115.9232  129.1121 H   0  0  0  0  0  0
   93.7147  118.0243  134.0064 H   0  0  0  0  0  0
   90.7437  117.1537  128.4742 H   0  0  0  0  0  0
   94.9543  114.9401  131.1557 H   0  0  0  0  0  0
   93.2907  114.5650  131.5329 H   0  0  0  0  0  0
   91.4773  116.1835  130.5609 H   0  0  0  0  0  0
   91.0786  114.5363  136.2930 H   0  0  0  0  0  0
   93.8661  112.9422  139.1259 H   0  0  0  0  0  0
   88.1369  117.9964  125.0072 H   0  0  0  0  0  0
   88.6787  117.9114  126.5655 H   0  0  0  0  0  0
   95.4916  114.7023  138.5264 H   0  0  0  0  0  0
   92.6576  116.2902  135.6939 H   0  0  0  0  0  0
   94.5053  118.5723  135.9924 H   0  0  0  0  0  0
   96.0482  118.1551  135.3374 H   0  0  0  0  0  0
   95.4347  117.2552  137.6690 H   0  0  0  0  0  0
   96.2076  116.1766  136.5835 H   0  0  0  0  0  0
   90.3023  118.8197  124.2579 H   0  0  0  0  0  0
   90.3206  117.1579  124.8696 H   0  0  0  0  0  0
   90.7532  118.7875  127.2012 H   0  0  0  0  0  0
   91.6794  119.5816  125.9832 H   0  0  0  0  0  0
   96.6789  116.3627  125.7243 H   0  0  0  0  0  0
   96.2250  115.3377  127.0569 H   0  0  0  0  0  0
```

```
   96.8623  116.9645  127.3757 H   0  0  0  0  0  0
   93.2011  117.0519  132.3394 H   0  0  0  0  0  0
   88.5913  119.3532  125.8062 H   0  0  0  0  0  0
  1  3  2  0  0  0
  1  4  1  0  0  0
  1  7  1  0  0  0
  2  6  2  0  0  0
  2 13  1  0  0  0
  2  5  1  0  0  0
  4 10  1  0  0  0
  4 50  1  0  0  0
  5  8  2  0  0  0
  5 19  1  0  0  0
  6  9  1  0  0  0
  6 27  1  0  0  0
  7 22  1  0  0  0
  7 28  1  0  0  0
  8 12  1  0  0  0
  8 29  1  0  0  0
  9 10  1  0  0  0
  9 12  2  0  0  0
 10 30  1  0  0  0
 10 31  1  0  0  0
 11 16  2  0  0  0
 11 15  1  0  0  0
 11 17  1  0  0  0
 12 32  1  0  0  0
 13 26  1  0  0  0
 14 23  1  0  0  0
 14 20  1  0  0  0
 14 21  2  0  0  0
 16 21  1  0  0  0
 16 33  1  0  0  0
 17 20  2  0  0  0
 17 34  1  0  0  0
 18 24  1  0  0  0
 18 35  1  0  0  0
 18 36  1  0  0  0
 18 51  1  0  0  0
 19 25  1  0  0  0
 20 37  1  0  0  0
 21 38  1  0  0  0
 22 23  1  0  0  0
 22 39  1  0  0  0
 22 40  1  0  0  0
 23 41  1  0  0  0
 23 42  1  0  0  0
 24 25  1  0  0  0
 24 43  1  0  0  0
 24 44  1  0  0  0
 25 45  1  0  0  0
 25 46  1  0  0  0
 26 47  1  0  0  0
 26 48  1  0  0  0
 26 49  1  0  0  0
M  CHG  1  18   1
M  END
> <s_m_entry_id>
56

> <s_m_entry_name>
C_2c.1

> <s_m_Source_Path>
C:\Users\julio\OneDrive - Universidad de Talca\Escritorio\Nueva carpeta

> <s_m_Source_File>
C_2c.mol2

> <i_m_Source_File_Index>
1
```

```
$$$$
C_2d
                    3D
 Structure written by MMmdl.
 51 52  0  0  1  0            999 V2000
   94.0324  115.9408  132.6150 C   0  0  0  0  0  0
   94.5832  115.3061  137.1735 C   0  0  0  0  0  0
   93.7331  114.2746  137.5797 C   0  0  0  0  0  0
   93.8565  116.8217  127.5209 C   0  0  0  0  0  0
   95.1276  114.6616  132.5623 S   0  0  0  0  0  0
   92.8402  115.9768  131.9459 N   0  0  0  0  0  0
   92.7523  117.5873  127.1107 C   0  0  0  0  0  0
   94.2487  116.1096  136.0654 C   0  0  0  0  0  0
   93.7686  116.0622  128.7028 C   0  0  0  0  0  0
   94.2154  117.0889  133.3345 N   0  0  0  0  0  0
   91.5805  117.5755  127.8885 C   0  0  0  0  0  0
   93.0466  115.8461  135.3868 C   0  0  0  0  0  0
   92.5450  114.0278  136.8947 C   0  0  0  0  0  0
   92.6049  116.0676  129.4757 C   0  0  0  0  0  0
   96.0430  115.5540  138.0730 Cl  0  0  0  0  0  0
   92.5138  115.2148  130.7328 C   0  0  0  0  0  0
   92.2027  114.8147  135.7978 C   0  0  0  0  0  0
   91.5078  116.8193  129.0558 C   0  0  0  0  0  0
   94.9815  116.8666  126.7136 O   0  0  0  0  0  0
   91.5005  112.7507  137.4040 Cl  0  0  0  0  0  0
   89.3139  118.3749  125.6509 N   0  3  0  0  0  0
   92.8960  118.3151  125.9402 O   0  0  0  0  0  0
   95.4149  117.4300  134.1100 C   0  0  0  0  0  0
   95.1916  117.2425  135.6266 C   0  0  0  0  0  0
   90.6147  119.0679  126.0640 C   0  0  0  0  0  0
   91.7564  118.6231  125.1399 C   0  0  0  0  0  0
   96.2564  116.5178  127.2452 C   0  0  0  0  0  0
   93.9973  113.6630  138.4306 H   0  0  0  0  0  0
   94.5995  115.4606  129.0396 H   0  0  0  0  0  0
   93.4675  117.7677  133.3407 H   0  0  0  0  0  0
   90.7079  118.1450  127.6071 H   0  0  0  0  0  0
   92.7415  116.4301  134.5322 H   0  0  0  0  0  0
   93.1990  114.3697  130.6470 H   0  0  0  0  0  0
   91.5071  114.7971  130.7832 H   0  0  0  0  0  0
   91.2831  114.6294  135.2617 H   0  0  0  0  0  0
   90.5943  116.8231  129.6331 H   0  0  0  0  0  0
   88.5593  118.6647  126.2564 H   0  0  0  0  0  0
   89.0796  118.6167  124.6986 H   0  0  0  0  0  0
   95.6890  118.4673  133.9101 H   0  0  0  0  0  0
   96.2440  116.7987  133.7854 H   0  0  0  0  0  0
   94.8037  118.1769  136.0362 H   0  0  0  0  0  0
   96.1606  117.0720  136.0993 H   0  0  0  0  0  0
   90.7710  118.7841  127.1051 H   0  0  0  0  0  0
   90.4023  120.1354  125.9993 H   0  0  0  0  0  0
   91.9879  119.4223  124.4329 H   0  0  0  0  0  0
   91.4430  117.7493  124.5649 H   0  0  0  0  0  0
   97.0071  116.6176  126.4611 H   0  0  0  0  0  0
   96.2825  115.4825  127.5889 H   0  0  0  0  0  0
   96.5501  117.1800  128.0612 H   0  0  0  0  0  0
   92.2102  116.7334  132.1714 H   0  0  0  0  0  0
   89.4189  117.3726  125.7168 H   0  0  0  0  0  0
  1  5  2  0  0  0
  1  6  1  0  0  0
  1 10  1  0  0  0
  2  8  2  0  0  0
  2  3  1  0  0  0
  2 15  1  0  0  0
  3 13  2  0  0  0
  3 28  1  0  0  0
  4  9  2  0  0  0
  4 19  1  0  0  0
  4  7  1  0  0  0
  6 16  1  0  0  0
  6 50  1  0  0  0
  7 11  2  0  0  0
```

```
  7 22  1  0  0  0
  8 24  1  0  0  0
  8 12  1  0  0  0
  9 14  1  0  0  0
  9 29  1  0  0  0
 10 23  1  0  0  0
 10 30  1  0  0  0
 11 18  1  0  0  0
 11 31  1  0  0  0
 12 17  2  0  0  0
 12 32  1  0  0  0
 13 17  1  0  0  0
 13 20  1  0  0  0
 14 16  1  0  0  0
 14 18  2  0  0  0
 16 33  1  0  0  0
 16 34  1  0  0  0
 17 35  1  0  0  0
 18 36  1  0  0  0
 19 27  1  0  0  0
 21 25  1  0  0  0
 21 37  1  0  0  0
 21 38  1  0  0  0
 21 51  1  0  0  0
 22 26  1  0  0  0
 23 24  1  0  0  0
 23 39  1  0  0  0
 23 40  1  0  0  0
 24 41  1  0  0  0
 24 42  1  0  0  0
 25 26  1  0  0  0
 25 43  1  0  0  0
 25 44  1  0  0  0
 26 45  1  0  0  0
 26 46  1  0  0  0
 27 47  1  0  0  0
 27 48  1  0  0  0
 27 49  1  0  0  0
M  CHG  1  21   1
M  END
> <s_m_entry_id>
57

> <s_m_entry_name>
C_2d.1

> <s_m_Source_Path>
C:\Users\julio\OneDrive - Universidad de Talca\Escritorio\Nueva carpeta

> <s_m_Source_File>
C_2d.mol2

> <i_m_Source_File_Index>
1

$$$$
C_2e
                    3D
 Structure written by MMmdl.
 48 49  0  0  1  0            999 V2000
   94.1986  116.0724  132.8193 C   0  0  0  0  0  0
   93.9657  117.0623  127.3344 C   0  0  0  0  0  0
   95.0777  114.6496  132.9683 S   0  0  0  0  0  0
   93.3730  116.3629  131.7764 N   0  0  0  0  0  0
   94.1989  117.0977  133.7240 N   0  0  0  0  0  0
   92.7511  117.6528  126.9463 C   0  0  0  0  0  0
   94.0278  116.3634  128.5528 C   0  0  0  0  0  0
   91.6410  117.5631  127.8010 C   0  0  0  0  0  0
   92.9096  116.2558  129.3775 C   0  0  0  0  0  0
   92.9999  115.4757  130.6731 C   0  0  0  0  0  0
   94.8559  117.0917  135.0422 C   0  0  0  0  0  0
```

93.4331	114.2271	137.9290	C	0	0	0	0	0	0
94.3152	116.1068	136.0802	C	0	0	0	0	0	0
91.7111	116.8524	128.9949	C	0	0	0	0	0	0
95.0401	117.1609	126.4667	O	0	0	0	0	0	0
92.7912	112.7632	139.2694	I	0	0	0	0	0	0
92.5868	114.6234	136.8981	C	0	0	0	0	0	0
94.7057	114.7785	138.0603	C	0	0	0	0	0	0
89.0773	118.7623	125.2696	N	0	3	0	0	0	0
93.0280	115.5628	135.9728	C	0	0	0	0	0	0
95.1485	115.7163	137.1319	C	0	0	0	0	0	0
92.6965	118.3079	125.7280	O	0	0	0	0	0	0
90.5140	119.3114	125.2156	C	0	0	0	0	0	0
91.5145	118.1729	124.9358	C	0	0	0	0	0	0
96.3034	116.6325	126.8681	C	0	0	0	0	0	0
93.7575	117.9644	133.4471	H	0	0	0	0	0	0
94.9406	115.8883	128.8777	H	0	0	0	0	0	0
90.6975	118.0171	127.5489	H	0	0	0	0	0	0
93.6970	114.6489	130.5331	H	0	0	0	0	0	0
92.0349	115.0208	130.8987	H	0	0	0	0	0	0
94.7576	118.0923	135.4636	H	0	0	0	0	0	0
95.9266	116.9327	134.9026	H	0	0	0	0	0	0
90.8336	116.7795	129.6225	H	0	0	0	0	0	0
91.5988	114.1948	136.8087	H	0	0	0	0	0	0
95.3534	114.4679	138.8661	H	0	0	0	0	0	0
88.4313	119.4904	125.5411	H	0	0	0	0	0	0
88.7856	118.4155	124.3602	H	0	0	0	0	0	0
92.3673	115.8558	135.1704	H	0	0	0	0	0	0
96.1446	116.1250	137.2247	H	0	0	0	0	0	0
90.6789	119.7915	126.1790	H	0	0	0	0	0	0
90.5174	120.0749	124.4380	H	0	0	0	0	0	0
91.8247	118.2322	123.8918	H	0	0	0	0	0	0
91.0779	117.1781	125.0410	H	0	0	0	0	0	0
97.0288	116.7626	126.0698	H	0	0	0	0	0	0
96.2547	115.5626	127.0690	H	0	0	0	0	0	0
96.6879	117.1460	127.7504	H	0	0	0	0	0	0
92.9026	117.2647	131.7933	H	0	0	0	0	0	0
89.0014	118.0149	125.9473	H	0	0	0	0	0	0

1	3	2	0	0	0
1	4	1	0	0	0
1	5	1	0	0	0
2	7	2	0	0	0
2	15	1	0	0	0
2	6	1	0	0	0
4	10	1	0	0	0
4	47	1	0	0	0
5	11	1	0	0	0
5	26	1	0	0	0
6	8	2	0	0	0
6	22	1	0	0	0
7	9	1	0	0	0
7	27	1	0	0	0
8	14	1	0	0	0
8	28	1	0	0	0
9	10	1	0	0	0
9	14	2	0	0	0
10	29	1	0	0	0
10	30	1	0	0	0
11	13	1	0	0	0
11	31	1	0	0	0
11	32	1	0	0	0
12	18	2	0	0	0
12	16	1	0	0	0
12	17	1	0	0	0
13	20	1	0	0	0
13	21	2	0	0	0
14	33	1	0	0	0
15	25	1	0	0	0
17	20	2	0	0	0
17	34	1	0	0	0
18	21	1	0	0	0
18	35	1	0	0	0

```
 19 23  1  0  0  0
 19 36  1  0  0  0
 19 37  1  0  0  0
 19 48  1  0  0  0
 20 38  1  0  0  0
 21 39  1  0  0  0
 22 24  1  0  0  0
 23 24  1  0  0  0
 23 40  1  0  0  0
 23 41  1  0  0  0
 24 42  1  0  0  0
 24 43  1  0  0  0
 25 44  1  0  0  0
 25 45  1  0  0  0
 25 46  1  0  0  0
M  CHG  1  19   1
M  END
> <s_m_entry_id>
58

> <s_m_entry_name>
C_2e.1

> <s_m_Source_Path>
C:\Users\julio\OneDrive - Universidad de Talca\Escritorio\Nueva carpeta

> <s_m_Source_File>
C_2e.mol2

> <i_m_Source_File_Index>
1

$$$$
C_2g
                    3D
 Structure written by MMmdl.
 57 58  0  0  1  0            999 V2000
   93.9591  116.1186  131.8577 C   0  0  0  0  0  0
   93.7016  116.7893  127.3734 C   0  0  0  0  0  0
   93.8728  116.7373  133.0747 N   0  0  0  0  0  0
   95.2121  116.3629  130.7582 S   0  0  0  0  0  0
   92.8950  115.2824  131.6611 N   0  0  0  0  0  0
   92.9975  114.5634  138.3453 C   0  0  0  0  0  0
   93.2632  115.1542  136.9303 C   0  0  0  0  0  0
   92.6153  117.6536  127.1577 C   0  0  0  0  0  0
   93.6411  115.8538  128.4235 C   0  0  0  0  0  0
   92.3099  114.9710  135.9133 C   0  0  0  0  0  0
   94.4299  115.8781  136.6223 C   0  0  0  0  0  0
   91.4882  117.5630  127.9942 C   0  0  0  0  0  0
   93.6848  116.1821  134.3490 C   0  0  0  0  0  0
   92.5226  115.7806  129.2573 C   0  0  0  0  0  0
   92.4585  114.7412  130.3669 C   0  0  0  0  0  0
   92.5119  115.4840  134.6345 C   0  0  0  0  0  0
   94.6379  116.3938  135.3451 C   0  0  0  0  0  0
   91.4421  116.6325  129.0294 C   0  0  0  0  0  0
   94.7822  116.9192  126.5161 O   0  0  0  0  0  0
   89.1965  118.5861  125.2689 N   0  3  0  0  0  0
   92.7306  118.5538  126.1105 O   0  0  0  0  0  0
   92.3353  115.6376  139.2492 C   0  0  0  0  0  0
   94.2875  114.0858  139.0724 C   0  0  0  0  0  0
   92.0553  113.3345  138.2356 C   0  0  0  0  0  0
   90.6541  118.2625  124.9316 C   0  0  0  0  0  0
   91.5841  119.2582  125.6378 C   0  0  0  0  0  0
   96.0457  116.3645  126.8704 C   0  0  0  0  0  0
   93.9542  117.7439  133.0661 H   0  0  0  0  0  0
   94.4589  115.1738  128.6095 H   0  0  0  0  0  0
   91.3951  114.4294  136.1030 H   0  0  0  0  0  0
   95.1985  116.0629  137.3561 H   0  0  0  0  0  0
   90.6302  118.2041  127.8602 H   0  0  0  0  0  0
   93.0910  113.8916  130.1037 H   0  0  0  0  0  0
   91.4353  114.3654  130.4166 H   0  0  0  0  0  0
```

```
  91.7579  115.3361  133.8743 H   0  0  0  0  0  0
  95.5382  116.9543  135.1365 H   0  0  0  0  0  0
  90.5625  116.5793  129.6548 H   0  0  0  0  0  0
  88.5804  117.9331  124.8062 H   0  0  0  0  0  0
  89.0493  118.5234  126.2661 H   0  0  0  0  0  0
  91.3664  115.9645  138.8704 H   0  0  0  0  0  0
  92.9619  116.5264  139.3398 H   0  0  0  0  0  0
  92.1611  115.2649  140.2600 H   0  0  0  0  0  0
  94.8027  113.2985  138.5196 H   0  0  0  0  0  0
  94.0662  113.6747  140.0589 H   0  0  0  0  0  0
  94.9952  114.9011  139.2312 H   0  0  0  0  0  0
  92.4793  112.5607  137.5934 H   0  0  0  0  0  0
  91.0773  113.5911  137.8273 H   0  0  0  0  0  0
  91.8717  112.8767  139.2091 H   0  0  0  0  0  0
  90.7143  118.3171  123.8442 H   0  0  0  0  0  0
  90.8009  117.2328  125.2590 H   0  0  0  0  0  0
  91.0537  119.7297  126.4675 H   0  0  0  0  0  0
  91.8772  120.0445  124.9391 H   0  0  0  0  0  0
  96.7603  116.5722  126.0738 H   0  0  0  0  0  0
  95.9944  115.2806  126.9852 H   0  0  0  0  0  0
  96.4414  116.8093  127.7848 H   0  0  0  0  0  0
  92.2331  115.1956  132.4190 H   0  0  0  0  0  0
  88.9695  119.5217  124.9635 H   0  0  0  0  0  0
  1  3  1  0  0  0
  1  4  2  0  0  0
  1  5  1  0  0  0
  2  9  2  0  0  0
  2 19  1  0  0  0
  2  8  1  0  0  0
  3 13  1  0  0  0
  3 28  1  0  0  0
  5 15  1  0  0  0
  5 56  1  0  0  0
  6  7  1  0  0  0
  6 22  1  0  0  0
  6 23  1  0  0  0
  6 24  1  0  0  0
  7 11  2  0  0  0
  7 10  1  0  0  0
  8 12  2  0  0  0
  8 21  1  0  0  0
  9 14  1  0  0  0
  9 29  1  0  0  0
 10 16  2  0  0  0
 10 30  1  0  0  0
 11 17  1  0  0  0
 11 31  1  0  0  0
 12 18  1  0  0  0
 12 32  1  0  0  0
 13 16  1  0  0  0
 13 17  2  0  0  0
 14 15  1  0  0  0
 14 18  2  0  0  0
 15 33  1  0  0  0
 15 34  1  0  0  0
 16 35  1  0  0  0
 17 36  1  0  0  0
 18 37  1  0  0  0
 19 27  1  0  0  0
 20 25  1  0  0  0
 20 38  1  0  0  0
 20 39  1  0  0  0
 20 57  1  0  0  0
 21 26  1  0  0  0
 22 40  1  0  0  0
 22 41  1  0  0  0
 22 42  1  0  0  0
 23 43  1  0  0  0
 23 44  1  0  0  0
 23 45  1  0  0  0
 24 46  1  0  0  0
```

```
 24 47  1  0  0  0
 24 48  1  0  0  0
 25 26  1  0  0  0
 25 49  1  0  0  0
 25 50  1  0  0  0
 26 51  1  0  0  0
 26 52  1  0  0  0
 27 53  1  0  0  0
 27 54  1  0  0  0
 27 55  1  0  0  0
M  CHG  1  20   1
M  END
> <s_m_entry_id>
59

> <s_m_entry_name>
C_2g.1

> <s_m_Source_Path>
C:\Users\julio\OneDrive - Universidad de Talca\Escritorio\Nueva carpeta

> <s_m_Source_File>
C_2g.mol2

> <i_m_Source_File_Index>
1

$$$$
C_2h
                  3D
 Structure written by MMmdl.
 60 61  0  0  1  0            999 V2000
   94.1119  115.5146  133.3103 C   0  0  0  0  0  0
   94.0876  117.5151  127.4462 C   0  0  0  0  0  0
   95.0416  114.1269  133.4848 S   0  0  0  0  0  0
   94.0623  116.2490  132.1616 N   0  0  0  0  0  0
   93.3135  116.0689  134.2763 N   0  0  0  0  0  0
   91.9379  110.1489  136.9541 C   0  0  0  0  0  0
   92.2875  111.6351  136.6882 C   0  0  0  0  0  0
   92.6930  117.6584  127.4535 C   0  0  0  0  0  0
   94.7413  116.9275  128.5455 C   0  0  0  0  0  0
   93.5578  112.1591  136.9717 C   0  0  0  0  0  0
   91.3099  112.5004  136.1739 C   0  0  0  0  0  0
   91.9928  117.2705  128.6006 C   0  0  0  0  0  0
   94.0219  116.5223  129.6714 C   0  0  0  0  0  0
   94.7265  115.9242  130.8845 C   0  0  0  0  0  0
   93.2758  115.7371  135.7157 C   0  0  0  0  0  0
   92.8969  114.3113  136.1018 C   0  0  0  0  0  0
   92.6429  116.7109  129.6873 C   0  0  0  0  0  0
   93.8551  113.4883  136.6880 C   0  0  0  0  0  0
   91.6086  113.8303  135.8868 C   0  0  0  0  0  0
   94.7686  117.9388  126.3290 O   0  0  0  0  0  0
   88.6298  118.8694  125.0854 N   0  3  0  0  0  0
   92.0736  118.2067  126.3415 O   0  0  0  0  0  0
   91.0233  110.0735  138.1910 C   0  0  0  0  0  0
   93.1855  109.2676  137.2334 C   0  0  0  0  0  0
   91.2171  109.5288  135.7255 C   0  0  0  0  0  0
   90.1579  118.7867  124.9771 C   0  0  0  0  0  0
   90.6895  117.9302  126.1259 C   0  0  0  0  0  0
   96.0062  118.5995  126.5502 C   0  0  0  0  0  0
   93.6322  117.1676  132.2089 H   0  0  0  0  0  0
   92.8253  116.9176  134.0253 H   0  0  0  0  0  0
   95.8109  116.7846  128.5272 H   0  0  0  0  0  0
   94.3376  111.5501  137.3981 H   0  0  0  0  0  0
   90.3039  112.1524  135.9931 H   0  0  0  0  0  0
   90.9297  117.3657  128.6851 H   0  0  0  0  0  0
   95.7587  116.2695  130.9284 H   0  0  0  0  0  0
   94.7667  114.8472  130.7251 H   0  0  0  0  0  0
   94.2363  115.9944  136.1605 H   0  0  0  0  0  0
   92.5514  116.4066  136.1763 H   0  0  0  0  0  0
   92.0540  116.4216  130.5443 H   0  0  0  0  0  0
```

```
   94.8504  113.8597  136.8882 H   0  0  0  0  0  0
   90.8413  114.4708  135.4855 H   0  0  0  0  0  0
   88.2420  119.2428  124.2246 H   0  0  0  0  0  0
   88.3486  119.4494  125.8653 H   0  0  0  0  0  0
   90.6617  109.0617  138.3739 H   0  0  0  0  0  0
   90.1597  110.7253  138.0895 H   0  0  0  0  0  0
   91.5541  110.3981  139.0844 H   0  0  0  0  0  0
   93.7000  109.5603  138.1512 H   0  0  0  0  0  0
   93.9066  109.3132  136.4159 H   0  0  0  0  0  0
   92.9157  108.2180  137.3641 H   0  0  0  0  0  0
   90.9879  108.4752  135.8866 H   0  0  0  0  0  0
   91.8367  109.5921  134.8313 H   0  0  0  0  0  0
   90.2695  110.0123  135.4922 H   0  0  0  0  0  0
   90.3793  118.3627  123.9972 H   0  0  0  0  0  0
   90.5002  119.8223  125.0140 H   0  0  0  0  0  0
   90.5511  116.8730  125.8854 H   0  0  0  0  0  0
   90.0913  118.1448  127.0076 H   0  0  0  0  0  0
   96.2354  119.2106  125.6771 H   0  0  0  0  0  0
   96.8105  117.8768  126.6860 H   0  0  0  0  0  0
   95.9816  119.2678  127.4124 H   0  0  0  0  0  0
   88.2315  117.9474  125.2087 H   0  0  0  0  0  0
  1  3  2  0  0  0
  1  4  1  0  0  0
  1  5  1  0  0  0
  2  9  2  0  0  0
  2 20  1  0  0  0
  2  8  1  0  0  0
  4 14  1  0  0  0
  4 29  1  0  0  0
  5 15  1  0  0  0
  5 30  1  0  0  0
  6  7  1  0  0  0
  6 23  1  0  0  0
  6 24  1  0  0  0
  6 25  1  0  0  0
  7 11  2  0  0  0
  7 10  1  0  0  0
  8 12  2  0  0  0
  8 22  1  0  0  0
  9 13  1  0  0  0
  9 31  1  0  0  0
 10 18  2  0  0  0
 10 32  1  0  0  0
 11 19  1  0  0  0
 11 33  1  0  0  0
 12 17  1  0  0  0
 12 34  1  0  0  0
 13 14  1  0  0  0
 13 17  2  0  0  0
 14 35  1  0  0  0
 14 36  1  0  0  0
 15 16  1  0  0  0
 15 37  1  0  0  0
 15 38  1  0  0  0
 16 18  1  0  0  0
 16 19  2  0  0  0
 17 39  1  0  0  0
 18 40  1  0  0  0
 19 41  1  0  0  0
 20 28  1  0  0  0
 21 26  1  0  0  0
 21 42  1  0  0  0
 21 43  1  0  0  0
 21 60  1  0  0  0
 22 27  1  0  0  0
 23 44  1  0  0  0
 23 45  1  0  0  0
 23 46  1  0  0  0
 24 47  1  0  0  0
 24 48  1  0  0  0
 24 49  1  0  0  0
```

```
 25 50  1  0  0  0
 25 51  1  0  0  0
 25 52  1  0  0  0
 26 27  1  0  0  0
 26 53  1  0  0  0
 26 54  1  0  0  0
 27 55  1  0  0  0
 27 56  1  0  0  0
 28 57  1  0  0  0
 28 58  1  0  0  0
 28 59  1  0  0  0
M  CHG  1  21   1
M  END
> <s_m_entry_id>
60

> <s_m_entry_name>
C_2h.1

> <s_m_Source_Path>
C:\Users\julio\OneDrive - Universidad de Talca\Escritorio\Nueva carpeta

> <s_m_Source_File>
C_2h.mol2

> <i_m_Source_File_Index>
1

$$$$
C_2i
                   3D
 Structure written by MMmdl.
 72 73  0  0  1  0            999 V2000
   94.2640  115.7725  132.9015 C   0  0  0  0  0  0
   93.7885  113.1512  136.8974 C   0  0  0  0  0  0
   92.8834  111.3665  135.4540 C   0  0  0  0  0  0
   92.9740  112.0235  136.6937 C   0  0  0  0  0  0
   93.9437  117.0008  127.5571 C   0  0  0  0  0  0
   91.9649  110.1187  135.3045 C   0  0  0  0  0  0
   93.8436  113.8230  138.3004 C   0  0  0  0  0  0
   94.8757  116.5685  134.2539 S   0  0  0  0  0  0
   94.5580  114.4814  132.5593 N   0  0  0  0  0  0
   93.3993  116.3215  131.9942 N   0  0  0  0  0  0
   94.5295  113.6247  135.8019 C   0  0  0  0  0  0
   93.6438  111.8784  134.3895 C   0  0  0  0  0  0
   92.7823  117.6107  127.0553 C   0  0  0  0  0  0
   94.4527  113.0044  134.5534 C   0  0  0  0  0  0
   93.9011  116.3632  128.8113 C   0  0  0  0  0  0
   91.6001  117.5679  127.8160 C   0  0  0  0  0  0
   92.7249  116.3352  129.5661 C   0  0  0  0  0  0
   95.2916  113.5175  133.3912 C   0  0  0  0  0  0
   92.6903  115.6174  130.9129 C   0  0  0  0  0  0
   91.5730  116.9335  129.0555 C   0  0  0  0  0  0
   95.0771  117.0689  126.7633 O   0  0  0  0  0  0
   89.2688  118.5047  125.6449 N   0  3  0  0  0  0
   92.8828  118.2236  125.8164 O   0  0  0  0  0  0
   90.8885  110.3828  134.2175 C   0  0  0  0  0  0
   91.2100  109.7414  136.6118 C   0  0  0  0  0  0
   92.8135  108.8844  134.8970 C   0  0  0  0  0  0
   94.2431  112.7739  139.3727 C   0  0  0  0  0  0
   94.8863  114.9731  138.2945 C   0  0  0  0  0  0
   92.4871  114.4395  138.7490 C   0  0  0  0  0  0
   90.6404  119.0698  126.0221 C   0  0  0  0  0  0
   91.7084  118.5237  125.0648 C   0  0  0  0  0  0
   96.3177  116.5595  127.2442 C   0  0  0  0  0  0
   92.3974  111.6447  137.5236 H   0  0  0  0  0  0
   94.4529  114.2298  131.5868 H   0  0  0  0  0  0
   95.1771  114.4830  135.8927 H   0  0  0  0  0  0
   93.6227  111.4180  133.4139 H   0  0  0  0  0  0
   94.7771  115.8809  129.2186 H   0  0  0  0  0  0
   90.6844  118.0193  127.4657 H   0  0  0  0  0  0
```

```
   96.2022  113.9568  133.8015 H   0  0  0  0  0  0
   95.6038  112.6758  132.7706 H   0  0  0  0  0  0
   93.2066  114.6667  130.7738 H   0  0  0  0  0  0
   91.6698  115.3514  131.1918 H   0  0  0  0  0  0
   90.6493  116.9101  129.6156 H   0  0  0  0  0  0
   88.5623  118.8614  126.2723 H   0  0  0  0  0  0
   89.0316  118.7720  124.7002 H   0  0  0  0  0  0
   91.3214  110.5575  133.2321 H   0  0  0  0  0  0
   90.2793  111.2549  134.4607 H   0  0  0  0  0  0
   90.2071  109.5373  134.1086 H   0  0  0  0  0  0
   91.8949  109.5009  137.4266 H   0  0  0  0  0  0
   90.5768  108.8633  136.4733 H   0  0  0  0  0  0
   90.5529  110.5432  136.9526 H   0  0  0  0  0  0
   93.5939  108.6751  135.6305 H   0  0  0  0  0  0
   93.3051  109.0131  133.9322 H   0  0  0  0  0  0
   92.2036  107.9833  134.8135 H   0  0  0  0  0  0
   94.2611  113.2057  140.3748 H   0  0  0  0  0  0
   95.2350  112.3551  139.2008 H   0  0  0  0  0  0
   93.5409  111.9390  139.3971 H   0  0  0  0  0  0
   94.9283  115.4866  139.2565 H   0  0  0  0  0  0
   94.6482  115.7255  137.5408 H   0  0  0  0  0  0
   95.8978  114.6213  138.0894 H   0  0  0  0  0  0
   92.5598  114.9007  139.7355 H   0  0  0  0  0  0
   91.6979  113.6890  138.8180 H   0  0  0  0  0  0
   92.1465  115.2217  138.0686 H   0  0  0  0  0  0
   90.7985  118.7676  127.0577 H   0  0  0  0  0  0
   90.5281  120.1530  125.9657 H   0  0  0  0  0  0
   91.9246  119.2670  124.2947 H   0  0  0  0  0  0
   91.3310  117.6280  124.5675 H   0  0  0  0  0  0
   97.0816  116.7066  126.4806 H   0  0  0  0  0  0
   96.2645  115.4888  127.4473 H   0  0  0  0  0  0
   96.6507  117.0842  128.1411 H   0  0  0  0  0  0
   93.2111  117.3107  132.0719 H   0  0  0  0  0  0
   89.2806  117.4966  125.7057 H   0  0  0  0  0  0
  1  8  2  0  0  0
  1  9  1  0  0  0
  1 10  1  0  0  0
  2 11  2  0  0  0
  2  7  1  0  0  0
  2  4  1  0  0  0
  3 12  1  0  0  0
  3  4  2  0  0  0
  3  6  1  0  0  0
  4 33  1  0  0  0
  5 15  2  0  0  0
  5 21  1  0  0  0
  5 13  1  0  0  0
  6 24  1  0  0  0
  6 25  1  0  0  0
  6 26  1  0  0  0
  7 27  1  0  0  0
  7 28  1  0  0  0
  7 29  1  0  0  0
  9 18  1  0  0  0
  9 34  1  0  0  0
 10 19  1  0  0  0
 10 71  1  0  0  0
 11 14  1  0  0  0
 11 35  1  0  0  0
 12 14  2  0  0  0
 12 36  1  0  0  0
 13 16  2  0  0  0
 13 23  1  0  0  0
 14 18  1  0  0  0
 15 17  1  0  0  0
 15 37  1  0  0  0
 16 20  1  0  0  0
 16 38  1  0  0  0
 17 19  1  0  0  0
 17 20  2  0  0  0
 18 39  1  0  0  0
```

```
 18 40  1  0  0  0
 19 41  1  0  0  0
 19 42  1  0  0  0
 20 43  1  0  0  0
 21 32  1  0  0  0
 22 30  1  0  0  0
 22 44  1  0  0  0
 22 45  1  0  0  0
 22 72  1  0  0  0
 23 31  1  0  0  0
 24 46  1  0  0  0
 24 47  1  0  0  0
 24 48  1  0  0  0
 25 49  1  0  0  0
 25 50  1  0  0  0
 25 51  1  0  0  0
 26 52  1  0  0  0
 26 53  1  0  0  0
 26 54  1  0  0  0
 27 55  1  0  0  0
 27 56  1  0  0  0
 27 57  1  0  0  0
 28 58  1  0  0  0
 28 59  1  0  0  0
 28 60  1  0  0  0
 29 61  1  0  0  0
 29 62  1  0  0  0
 29 63  1  0  0  0
 30 31  1  0  0  0
 30 64  1  0  0  0
 30 65  1  0  0  0
 31 66  1  0  0  0
 31 67  1  0  0  0
 32 68  1  0  0  0
 32 69  1  0  0  0
 32 70  1  0  0  0
M  CHG  1  22   1
M  END
> <s_m_entry_id>
61

> <s_m_entry_name>
C_2i.1

> <s_m_Source_Path>
C:\Users\julio\OneDrive - Universidad de Talca\Escritorio\Nueva carpeta

> <s_m_Source_File>
C_2i.mol2

> <i_m_Source_File_Index>
1

$$$$
C_2j
                    3D
 Structure written by MMmdl.
 63 64  0  0  1  0            999 V2000
   94.4205  115.8284  132.2132 C   0  0  0  0  0  0
   93.8654  117.0150  127.4512 C   0  0  0  0  0  0
   95.4780  114.6969  131.5734 S   0  0  0  0  0  0
   93.1232  115.9631  131.8130 N   0  0  0  0  0  0
   92.8046  114.0193  138.5314 C   0  0  0  0  0  0
   93.8830  114.5403  137.5490 C   0  0  0  0  0  0
   92.7119  117.6583  126.9844 C   0  0  0  0  0  0
   93.7961  116.2598  128.6357 C   0  0  0  0  0  0
   94.7346  116.7294  133.1956 N   0  0  0  0  0  0
   94.1454  113.8315  136.3654 C   0  0  0  0  0  0
   94.6354  115.6954  137.8156 C   0  0  0  0  0  0
   91.5319  117.5666  127.7360 C   0  0  0  0  0  0
   92.6110  116.1763  129.3773 C   0  0  0  0  0  0
```

92.5224	115.3229	130.6325	C	0	0	0	0	0	0
91.4756	116.8365	128.9156	C	0	0	0	0	0	0
95.0166	117.1394	126.6907	O	0	0	0	0	0	0
95.0745	114.2971	135.4470	C	0	0	0	0	0	0
95.5764	116.1623	136.8999	C	0	0	0	0	0	0
95.7773	115.4709	135.7042	C	0	0	0	0	0	0
89.2333	119.0033	125.2698	N	0	3	0	0	0	0
92.7864	118.3604	125.7905	O	0	0	0	0	0	0
96.0907	117.0302	133.6909	C	0	0	0	0	0	0
91.5738	113.4509	137.7726	C	0	0	0	0	0	0
92.2609	115.1342	139.4631	C	0	0	0	0	0	0
93.4181	112.8988	139.3950	C	0	0	0	0	0	0
96.7364	116.0055	134.6555	C	0	0	0	0	0	0
90.6828	119.5229	125.2690	C	0	0	0	0	0	0
91.6481	118.3658	124.9293	C	0	0	0	0	0	0
96.2332	116.5766	127.1734	C	0	0	0	0	0	0
94.6617	115.7260	128.9904	H	0	0	0	0	0	0
93.9947	117.3292	133.5363	H	0	0	0	0	0	0
93.6319	112.9089	136.1401	H	0	0	0	0	0	0
94.5026	116.2566	138.7293	H	0	0	0	0	0	0
90.6333	118.0583	127.4201	H	0	0	0	0	0	0
92.9367	114.3359	130.4319	H	0	0	0	0	0	0
91.4747	115.1561	130.8620	H	0	0	0	0	0	0
90.5457	116.7813	129.4578	H	0	0	0	0	0	0
95.2374	113.7476	134.5286	H	0	0	0	0	0	0
96.1317	117.0657	137.1149	H	0	0	0	0	0	0
88.6110	119.7284	125.5876	H	0	0	0	0	0	0
88.9403	118.7393	124.3318	H	0	0	0	0	0	0
95.9995	117.9766	134.2194	H	0	0	0	0	0	0
96.7578	117.2298	132.8544	H	0	0	0	0	0	0
91.7979	112.5526	137.1980	H	0	0	0	0	0	0
91.1472	114.1891	137.0913	H	0	0	0	0	0	0
90.7895	113.1616	138.4685	H	0	0	0	0	0	0
93.0287	115.5532	140.1129	H	0	0	0	0	0	0
91.4781	114.7640	140.1246	H	0	0	0	0	0	0
91.8283	115.9539	138.8882	H	0	0	0	0	0	0
94.2404	113.2634	140.0110	H	0	0	0	0	0	0
93.8048	112.0842	138.7821	H	0	0	0	0	0	0
92.6781	112.4680	140.0692	H	0	0	0	0	0	0
97.6007	116.4497	135.1513	H	0	0	0	0	0	0
97.1431	115.1662	134.0925	H	0	0	0	0	0	0
90.8403	119.9149	126.2776	H	0	0	0	0	0	0
90.7110	120.3438	124.5493	H	0	0	0	0	0	0
92.0126	118.4994	123.9104	H	0	0	0	0	0	0
91.1598	117.3883	124.9350	H	0	0	0	0	0	0
97.0440	116.7600	126.4664	H	0	0	0	0	0	0
96.1583	115.4949	127.2829	H	0	0	0	0	0	0
96.5215	117.0131	128.1292	H	0	0	0	0	0	0
92.5891	116.7129	132.2232	H	0	0	0	0	0	0
89.1252	118.2108	125.8909	H	0	0	0	0	0	0

1	3	2	0	0	0
1	4	1	0	0	0
1	9	1	0	0	0
2	8	2	0	0	0
2	16	1	0	0	0
2	7	1	0	0	0
4	14	1	0	0	0
4	62	1	0	0	0
5	6	1	0	0	0
5	23	1	0	0	0
5	24	1	0	0	0
5	25	1	0	0	0
6	11	2	0	0	0
6	10	1	0	0	0
7	12	2	0	0	0
7	21	1	0	0	0
8	13	1	0	0	0
8	30	1	0	0	0
9	22	1	0	0	0
9	31	1	0	0	0
10	17	2	0	0	0

```
 10 32  1  0  0  0
 11 18  1  0  0  0
 11 33  1  0  0  0
 12 15  1  0  0  0
 12 34  1  0  0  0
 13 14  1  0  0  0
 13 15  2  0  0  0
 14 35  1  0  0  0
 14 36  1  0  0  0
 15 37  1  0  0  0
 16 29  1  0  0  0
 17 19  1  0  0  0
 17 38  1  0  0  0
 18 19  2  0  0  0
 18 39  1  0  0  0
 19 26  1  0  0  0
 20 27  1  0  0  0
 20 40  1  0  0  0
 20 41  1  0  0  0
 20 63  1  0  0  0
 21 28  1  0  0  0
 22 26  1  0  0  0
 22 42  1  0  0  0
 22 43  1  0  0  0
 23 44  1  0  0  0
 23 45  1  0  0  0
 23 46  1  0  0  0
 24 47  1  0  0  0
 24 48  1  0  0  0
 24 49  1  0  0  0
 25 50  1  0  0  0
 25 51  1  0  0  0
 25 52  1  0  0  0
 26 53  1  0  0  0
 26 54  1  0  0  0
 27 28  1  0  0  0
 27 55  1  0  0  0
 27 56  1  0  0  0
 28 57  1  0  0  0
 28 58  1  0  0  0
 29 59  1  0  0  0
 29 60  1  0  0  0
 29 61  1  0  0  0
M  CHG  1  20   1
M  END
> <s_m_entry_id>
62

> <s_m_entry_name>
C_2j.1

> <s_m_Source_Path>
C:\Users\julio\OneDrive - Universidad de Talca\Escritorio\Nueva carpeta

> <s_m_Source_File>
C_2j.mol2

> <i_m_Source_File_Index>
1

$$$$
C_3
                    3D
 Structure written by MMmdl.
 54 55  0  0  1  0            999 V2000
   95.2623  116.6702  133.9736 C   0  0  0  0  0  0
   94.1285  116.6813  128.1890 C   0  0  0  0  0  0
   96.1942  115.3715  134.5062 S   0  0  0  0  0  0
   94.5114  116.6764  132.8307 N   0  0  0  0  0  0
   92.7544  116.9582  128.0948 C   0  0  0  0  0  0
   94.6588  116.2287  129.4118 C   0  0  0  0  0  0
```

```
   95.1528  117.8656  134.6281 N   0  0  0  0  0  0
   91.9381  116.7804  129.2264 C   0  0  0  0  0  0
   93.8375  116.0424  130.5265 C   0  0  0  0  0  0
   94.4318  115.5848  131.8505 C   0  0  0  0  0  0
   93.1359  114.1375  137.4939 C   0  0  0  0  0  0
   92.4767  116.3318  130.4299 C   0  0  0  0  0  0
   94.8848  116.8795  127.0453 O   0  0  0  0  0  0
   95.1744  115.9543  136.9469 C   0  0  0  0  0  0
   91.8720  113.0111  137.8329 Cl  0  0  0  0  0  0
   93.1152  114.8629  136.3034 C   0  0  0  0  0  0
   94.1693  114.3152  138.4127 C   0  0  0  0  0  0
   90.6771  119.1592  123.0552 N   0  3  0  0  0  0
   92.2881  117.4001  126.8669 O   0  0  0  0  0  0
   95.1889  115.2239  138.1394 C   0  0  0  0  0  0
   94.1346  115.7717  136.0296 C   0  0  0  0  0  0
   95.7917  118.2015  135.9064 C   0  0  0  0  0  0
   96.2907  116.9476  136.6470 C   0  0  0  0  0  0
   91.1876  119.2142  124.4967 C   0  0  0  0  0  0
   91.0217  118.0503  126.7690 C   0  0  0  0  0  0
   96.2809  116.5956  127.0541 C   0  0  0  0  0  0
   90.5801  118.0535  125.2973 C   0  0  0  0  0  0
   93.7982  117.3875  132.7542 H   0  0  0  0  0  0
   95.7117  116.0128  129.5148 H   0  0  0  0  0  0
   94.5867  118.5869  134.2045 H   0  0  0  0  0  0
   90.8790  116.9862  129.1989 H   0  0  0  0  0  0
   95.4156  115.1586  131.6478 H   0  0  0  0  0  0
   93.8210  114.7794  132.2620 H   0  0  0  0  0  0
   91.8292  116.2082  131.2862 H   0  0  0  0  0  0
   92.3134  114.7240  135.5928 H   0  0  0  0  0  0
   94.1833  113.7524  139.3348 H   0  0  0  0  0  0
   91.0708  119.9190  122.5187 H   0  0  0  0  0  0
   90.9434  118.2844  122.6263 H   0  0  0  0  0  0
   95.9867  115.3574  138.8560 H   0  0  0  0  0  0
   94.1125  116.3313  135.1054 H   0  0  0  0  0  0
   95.0818  118.7347  136.5413 H   0  0  0  0  0  0
   96.6340  118.8728  135.7293 H   0  0  0  0  0  0
   96.7755  117.2329  137.5826 H   0  0  0  0  0  0
   97.0642  116.4486  136.0600 H   0  0  0  0  0  0
   90.8863  120.1959  124.8630 H   0  0  0  0  0  0
   92.2731  119.1587  124.4115 H   0  0  0  0  0  0
   90.2796  117.5255  127.3738 H   0  0  0  0  0  0
   91.0951  119.0773  127.1324 H   0  0  0  0  0  0
   96.6933  116.8126  126.0688 H   0  0  0  0  0  0
   96.4789  115.5433  127.2638 H   0  0  0  0  0  0
   96.8161  117.2173  127.7735 H   0  0  0  0  0  0
   90.8618  117.1077  124.8321 H   0  0  0  0  0  0
   89.4918  118.1098  125.2453 H   0  0  0  0  0  0
   89.6702  119.2367  123.0408 H   0  0  0  0  0  0
  1  3  2  0  0  0
  1  4  1  0  0  0
  1  7  1  0  0  0
  2  6  2  0  0  0
  2 13  1  0  0  0
  2  5  1  0  0  0
  4 10  1  0  0  0
  4 28  1  0  0  0
  5  8  2  0  0  0
  5 19  1  0  0  0
  6  9  1  0  0  0
  6 29  1  0  0  0
  7 22  1  0  0  0
  7 30  1  0  0  0
  8 12  1  0  0  0
  8 31  1  0  0  0
  9 10  1  0  0  0
  9 12  2  0  0  0
 10 32  1  0  0  0
 10 33  1  0  0  0
 11 16  2  0  0  0
 11 15  1  0  0  0
 11 17  1  0  0  0
```

```
 12 34  1  0  0  0
 13 26  1  0  0  0
 14 23  1  0  0  0
 14 20  1  0  0  0
 14 21  2  0  0  0
 16 21  1  0  0  0
 16 35  1  0  0  0
 17 20  2  0  0  0
 17 36  1  0  0  0
 18 24  1  0  0  0
 18 37  1  0  0  0
 18 38  1  0  0  0
 18 54  1  0  0  0
 19 25  1  0  0  0
 20 39  1  0  0  0
 21 40  1  0  0  0
 22 23  1  0  0  0
 22 41  1  0  0  0
 22 42  1  0  0  0
 23 43  1  0  0  0
 23 44  1  0  0  0
 24 27  1  0  0  0
 24 45  1  0  0  0
 24 46  1  0  0  0
 25 27  1  0  0  0
 25 47  1  0  0  0
 25 48  1  0  0  0
 26 49  1  0  0  0
 26 50  1  0  0  0
 26 51  1  0  0  0
 27 52  1  0  0  0
 27 53  1  0  0  0
M  CHG  1  18   1
M  END
> <s_m_entry_id>
63

> <s_m_entry_name>
C_3.1

> <s_m_Source_Path>
C:\Users\julio\OneDrive - Universidad de Talca\Escritorio\Nueva carpeta

> <s_m_Source_File>
C_3.mol2

> <i_m_Source_File_Index>
1

$$$$
C_4
                   3D
 Structure written by MMmdl.
 54 55  0  0  1  0            999 V2000
   94.0868  115.7857  132.6319 C   0  0  0  0  0  0
   94.2277  117.0124  127.2925 C   0  0  0  0  0  0
   94.4225  114.1391  132.7536 S   0  0  0  0  0  0
   93.3599  116.3629  131.6276 N   0  0  0  0  0  0
   93.1563  117.7549  126.7689 C   0  0  0  0  0  0
   94.0618  116.3266  128.5105 C   0  0  0  0  0  0
   94.5050  116.7303  133.5275 N   0  0  0  0  0  0
   91.9409  117.8001  127.4754 C   0  0  0  0  0  0
   92.8464  116.3667  129.1982 C   0  0  0  0  0  0
   92.6937  115.6476  130.5306 C   0  0  0  0  0  0
   93.9521  114.5382  137.9674 C   0  0  0  0  0  0
   91.7911  117.1166  128.6796 C   0  0  0  0  0  0
   95.4021  117.0080  126.5575 O   0  0  0  0  0  0
   89.8837  118.6007  124.3749 N   0  3  0  0  0  0
   95.5902  115.3495  135.8666 C   0  0  0  0  0  0
   92.9364  114.0352  139.2699 Cl  0  0  0  0  0  0
   93.6229  114.1885  136.6585 C   0  0  0  0  0  0
```

```
   95.0955  115.2907  138.2324 C   0  0  0  0  0  0
   93.3767  118.4087  125.5670 O   0  0  0  0  0  0
   94.4421  114.5941  135.6076 C   0  0  0  0  0  0
   95.9150  115.6966  137.1818 C   0  0  0  0  0  0
   95.8974  116.9753  133.9234 C   0  0  0  0  0  0
   96.4860  115.7947  134.7167 C   0  0  0  0  0  0
   91.2843  117.9758  124.4604 C   0  0  0  0  0  0
   89.3591  119.1284  125.7166 C   0  0  0  0  0  0
   92.2978  119.0469  124.8867 C   0  0  0  0  0  0
   96.5130  116.2283  126.9908 C   0  0  0  0  0  0
   94.8694  115.7522  128.9392 H   0  0  0  0  0  0
   93.8046  117.3157  133.9596 H   0  0  0  0  0  0
   91.0946  118.3625  127.1115 H   0  0  0  0  0  0
   93.0899  114.6376  130.4146 H   0  0  0  0  0  0
   91.6331  115.5423  130.7653 H   0  0  0  0  0  0
   90.8488  117.1714  129.2057 H   0  0  0  0  0  0
   89.9077  119.3639  123.7138 H   0  0  0  0  0  0
   92.7366  113.6050  136.4553 H   0  0  0  0  0  0
   95.3490  115.5604  139.2474 H   0  0  0  0  0  0
   94.1818  114.3192  134.5955 H   0  0  0  0  0  0
   96.8001  116.2791  137.3939 H   0  0  0  0  0  0
   95.9508  117.8794  134.5325 H   0  0  0  0  0  0
   96.5055  117.1489  133.0338 H   0  0  0  0  0  0
   97.4681  116.0612  135.1119 H   0  0  0  0  0  0
   96.6612  114.9467  134.0519 H   0  0  0  0  0  0
   91.4961  117.5733  123.4688 H   0  0  0  0  0  0
   91.2083  117.1629  125.1840 H   0  0  0  0  0  0
   88.3702  119.5387  125.5119 H   0  0  0  0  0  0
   90.0515  119.8988  126.0572 H   0  0  0  0  0  0
   89.3092  118.2822  126.4027 H   0  0  0  0  0  0
   91.8096  119.7746  125.5381 H   0  0  0  0  0  0
   92.6609  119.5792  124.0052 H   0  0  0  0  0  0
   97.3331  116.3557  126.2841 H   0  0  0  0  0  0
   96.2738  115.1641  127.0213 H   0  0  0  0  0  0
   96.8755  116.5483  127.9689 H   0  0  0  0  0  0
   93.0801  117.3245  131.7581 H   0  0  0  0  0  0
   89.2385  117.9068  124.0252 H   0  0  0  0  0  0
  1  3  2  0  0  0
  1  4  1  0  0  0
  1  7  1  0  0  0
  2  6  2  0  0  0
  2 13  1  0  0  0
  2  5  1  0  0  0
  4 10  1  0  0  0
  4 53  1  0  0  0
  5  8  2  0  0  0
  5 19  1  0  0  0
  6  9  1  0  0  0
  6 28  1  0  0  0
  7 22  1  0  0  0
  7 29  1  0  0  0
  8 12  1  0  0  0
  8 30  1  0  0  0
  9 10  1  0  0  0
  9 12  2  0  0  0
 10 31  1  0  0  0
 10 32  1  0  0  0
 11 18  2  0  0  0
 11 16  1  0  0  0
 11 17  1  0  0  0
 12 33  1  0  0  0
 13 27  1  0  0  0
 14 24  1  0  0  0
 14 25  1  0  0  0
 14 34  1  0  0  0
 14 54  1  0  0  0
 15 23  1  0  0  0
 15 20  1  0  0  0
 15 21  2  0  0  0
 17 20  2  0  0  0
 17 35  1  0  0  0
```

```
 18 21  1  0  0  0
 18 36  1  0  0  0
 19 26  1  0  0  0
 20 37  1  0  0  0
 21 38  1  0  0  0
 22 23  1  0  0  0
 22 39  1  0  0  0
 22 40  1  0  0  0
 23 41  1  0  0  0
 23 42  1  0  0  0
 24 26  1  0  0  0
 24 43  1  0  0  0
 24 44  1  0  0  0
 25 45  1  0  0  0
 25 46  1  0  0  0
 25 47  1  0  0  0
 26 48  1  0  0  0
 26 49  1  0  0  0
 27 50  1  0  0  0
 27 51  1  0  0  0
 27 52  1  0  0  0
M  CHG  1  14   1
M  END
> <s_m_entry_id>
64

> <s_m_entry_name>
C_4.1

> <s_m_Source_Path>
C:\Users\julio\OneDrive - Universidad de Talca\Escritorio\Nueva carpeta

> <s_m_Source_File>
C_4.mol2

> <i_m_Source_File_Index>
1

$$$$
C_5
                   3D
 Structure written by MMmdl.
 57 58  0  0  1  0            999 V2000
   94.5439  115.7042  132.8777 C   0  0  0  0  0  0
   93.8055  116.6233  128.0777 C   0  0  0  0  0  0
   95.4750  114.4525  132.2537 S   0  0  0  0  0  0
   93.2610  115.9639  132.4921 N   0  0  0  0  0  0
   92.6861  117.3432  127.6451 C   0  0  0  0  0  0
   93.7421  115.9310  129.2972 C   0  0  0  0  0  0
   94.9675  116.5833  133.8360 N   0  0  0  0  0  0
   91.5581  117.4399  128.4700 C   0  0  0  0  0  0
   92.6004  116.0002  130.0982 C   0  0  0  0  0  0
   92.5335  115.2641  131.4244 C   0  0  0  0  0  0
   93.5622  114.6578  138.1809 C   0  0  0  0  0  0
   90.6773  118.4994  123.3635 N   0  3  0  0  0  0
   91.5158  116.7597  129.6727 C   0  0  0  0  0  0
   94.9211  116.6323  127.2567 O   0  0  0  0  0  0
   92.7584  117.9253  126.4017 O   0  0  0  0  0  0
   95.6109  115.1923  136.4041 C   0  0  0  0  0  0
   92.2772  114.3472  139.2693 Cl  0  0  0  0  0  0
   93.6878  113.8825  137.0331 C   0  0  0  0  0  0
   94.4555  115.6864  138.4586 C   0  0  0  0  0  0
   94.7168  114.1519  136.1393 C   0  0  0  0  0  0
   95.4847  115.9555  137.5642 C   0  0  0  0  0  0
   96.3196  116.6565  134.4248 C   0  0  0  0  0  0
   91.5687  118.3657  125.7743 C   0  0  0  0  0  0
   91.6907  117.8731  124.3307 C   0  0  0  0  0  0
   96.7048  115.5272  135.4089 C   0  0  0  0  0  0
   90.6726  120.0008  123.5004 C   0  0  0  0  0  0
   90.9515  118.1808  121.8849 C   0  0  0  0  0  0
   96.1711  116.2190  127.7940 C   0  0  0  0  0  0
```

```
   94.5816  115.3465  129.6346 H   0  0  0  0  0  0
   94.2932  117.2427  134.1967 H   0  0  0  0  0  0
   90.6989  118.0409  128.2173 H   0  0  0  0  0  0
   92.8665  114.2386  131.2870 H   0  0  0  0  0  0
   91.4973  115.1717  131.7372 H   0  0  0  0  0  0
   90.6405  116.8652  130.2838 H   0  0  0  0  0  0
   92.9873  113.0869  136.8299 H   0  0  0  0  0  0
   94.3406  116.2872  139.3471 H   0  0  0  0  0  0
   94.8148  113.5579  135.2450 H   0  0  0  0  0  0
   96.1722  116.7640  137.7538 H   0  0  0  0  0  0
   96.3475  117.5977  134.9766 H   0  0  0  0  0  0
   97.0815  116.7593  133.6532 H   0  0  0  0  0  0
   90.6318  117.9704  126.1600 H   0  0  0  0  0  0
   91.5567  119.4459  125.9000 H   0  0  0  0  0  0
   92.6741  118.0959  123.9156 H   0  0  0  0  0  0
   91.4671  116.8097  124.2873 H   0  0  0  0  0  0
   97.6086  115.8030  135.9484 H   0  0  0  0  0  0
   96.9862  114.6225  134.8683 H   0  0  0  0  0  0
   89.9847  120.3628  122.7305 H   0  0  0  0  0  0
   91.6910  120.3705  123.3549 H   0  0  0  0  0  0
   90.2789  120.2699  124.4768 H   0  0  0  0  0  0
   90.2771  118.7676  121.2630 H   0  0  0  0  0  0
   90.6773  117.1627  121.7385 H   0  0  0  0  0  0
   91.9964  118.3821  121.6721 H   0  0  0  0  0  0
   96.9570  116.3683  127.0536 H   0  0  0  0  0  0
   96.1519  115.1626  128.0541 H   0  0  0  0  0  0
   96.4459  116.7970  128.6772 H   0  0  0  0  0  0
   92.8444  116.8317  132.8042 H   0  0  0  0  0  0
   89.7441  118.1712  123.5950 H   0  0  0  0  0  0
  1  3  2  0  0  0
  1  4  1  0  0  0
  1  7  1  0  0  0
  2  6  2  0  0  0
  2 14  1  0  0  0
  2  5  1  0  0  0
  4 10  1  0  0  0
  4 56  1  0  0  0
  5  8  2  0  0  0
  5 15  1  0  0  0
  6  9  1  0  0  0
  6 29  1  0  0  0
  7 22  1  0  0  0
  7 30  1  0  0  0
  8 13  1  0  0  0
  8 31  1  0  0  0
  9 10  1  0  0  0
  9 13  2  0  0  0
 10 32  1  0  0  0
 10 33  1  0  0  0
 11 19  2  0  0  0
 11 17  1  0  0  0
 11 18  1  0  0  0
 12 24  1  0  0  0
 12 26  1  0  0  0
 12 27  1  0  0  0
 12 57  1  0  0  0
 13 34  1  0  0  0
 14 28  1  0  0  0
 15 23  1  0  0  0
 16 25  1  0  0  0
 16 20  1  0  0  0
 16 21  2  0  0  0
 18 20  2  0  0  0
 18 35  1  0  0  0
 19 21  1  0  0  0
 19 36  1  0  0  0
 20 37  1  0  0  0
 21 38  1  0  0  0
 22 25  1  0  0  0
 22 39  1  0  0  0
 22 40  1  0  0  0
```

```
 23 24  1  0  0  0
 23 41  1  0  0  0
 23 42  1  0  0  0
 24 43  1  0  0  0
 24 44  1  0  0  0
 25 45  1  0  0  0
 25 46  1  0  0  0
 26 47  1  0  0  0
 26 48  1  0  0  0
 26 49  1  0  0  0
 27 50  1  0  0  0
 27 51  1  0  0  0
 27 52  1  0  0  0
 28 53  1  0  0  0
 28 54  1  0  0  0
 28 55  1  0  0  0
M  CHG  1  12   1
M  END
> <s_m_entry_id>
65

> <s_m_entry_name>
C_5.1

> <s_m_Source_Path>
C:\Users\julio\OneDrive - Universidad de Talca\Escritorio\Nueva carpeta

> <s_m_Source_File>
C_5.mol2

> <i_m_Source_File_Index>
1

$$$$
C_8
                    3D
 Structure written by MMmdl.
 55 56  0  0  1  0            999 V2000
   94.7369  116.3483  133.6522 C   0  0  0  0  0  0
   93.9676  116.7228  127.9107 C   0  0  0  0  0  0
   95.8774  115.1630  133.9635 S   0  0  0  0  0  0
   93.9418  116.3630  132.5452 N   0  0  0  0  0  0
   91.9411  119.1838  123.4843 C   0  0  0  0  0  0
   92.6369  117.1629  127.7820 C   0  0  0  0  0  0
   94.3856  116.1402  129.1208 C   0  0  0  0  0  0
   94.4653  117.4144  134.4621 N   0  0  0  0  0  0
   93.0205  118.7632  123.8907 O   0  0  0  0  0  0
   91.7535  116.9905  128.8652 C   0  0  0  0  0  0
   90.7410  118.7524  123.8845 N   0  0  0  0  0  0
   93.4900  115.9613  130.1734 C   0  0  0  0  0  0
   93.9373  115.3512  131.4820 C   0  0  0  0  0  0
   93.3209  113.4244  137.8126 C   0  0  0  0  0  0
   92.1692  116.3628  130.0300 C   0  0  0  0  0  0
   94.8040  116.8680  126.8149 O   0  0  0  0  0  0
   94.7482  115.7407  137.1898 C   0  0  0  0  0  0
   92.4725  111.9598  138.1296 Cl  0  0  0  0  0  0
   94.6049  113.6011  138.3172 C   0  0  0  0  0  0
   92.7267  114.4112  137.0334 C   0  0  0  0  0  0
   92.2673  117.7163  126.5653 O   0  0  0  0  0  0
   95.3106  114.7638  138.0163 C   0  0  0  0  0  0
   93.4423  115.5626  136.7218 C   0  0  0  0  0  0
   95.2484  117.8857  135.6198 C   0  0  0  0  0  0
   91.8991  120.2583  122.4282 C   0  0  0  0  0  0
   95.6311  116.9331  136.7927 C   0  0  0  0  0  0
   90.4953  117.7196  124.8731 C   0  0  0  0  0  0
   90.9073  118.0873  126.3100 C   0  0  0  0  0  0
   96.1366  116.3631  126.8790 C   0  0  0  0  0  0
   95.4052  115.8158  129.2601 H   0  0  0  0  0  0
   93.7543  118.0569  134.1390 H   0  0  0  0  0  0
   90.7298  117.3246  128.8460 H   0  0  0  0  0  0
   89.9182  119.1628  123.4650 H   0  0  0  0  0  0
```

```
   93.2516  114.5494  131.7598 H   0  0  0  0  0  0
   94.9157  114.8900  131.3398 H   0  0  0  0  0  0
   91.4774  116.2355  130.8472 H   0  0  0  0  0  0
   95.0620  112.8248  138.9132 H   0  0  0  0  0  0
   91.7253  114.2734  136.6518 H   0  0  0  0  0  0
   96.3171  114.8837  138.3923 H   0  0  0  0  0  0
   92.9658  116.3011  136.0985 H   0  0  0  0  0  0
   96.1557  118.3533  135.2344 H   0  0  0  0  0  0
   94.6767  118.7088  136.0472 H   0  0  0  0  0  0
   90.8780  120.3631  122.0693 H   0  0  0  0  0  0
   92.5368  119.9625  121.6005 H   0  0  0  0  0  0
   92.2773  121.1997  122.8319 H   0  0  0  0  0  0
   96.6159  116.5246  136.5607 H   0  0  0  0  0  0
   95.8017  117.5433  137.6693 H   0  0  0  0  0  0
   89.4163  117.5635  124.8697 H   0  0  0  0  0  0
   90.9443  116.7775  124.5518 H   0  0  0  0  0  0
   90.7658  119.1497  126.5106 H   0  0  0  0  0  0
   90.1916  117.5631  126.9300 H   0  0  0  0  0  0
   96.7595  116.9721  127.5357 H   0  0  0  0  0  0
   96.5816  116.3628  125.8854 H   0  0  0  0  0  0
   96.1613  115.3289  127.2216 H   0  0  0  0  0  0
   93.2153  117.0652  132.4892 H   0  0  0  0  0  0
  1  3  2  0  0  0
  1  4  1  0  0  0
  1  8  1  0  0  0
  2  7  2  0  0  0
  2 16  1  0  0  0
  2  6  1  0  0  0
  4 13  1  0  0  0
  4 55  1  0  0  0
  5 11  1  0  0  0
  5  9  2  0  0  0
  5 25  1  0  0  0
  6 10  2  0  0  0
  6 21  1  0  0  0
  7 12  1  0  0  0
  7 30  1  0  0  0
  8 24  1  0  0  0
  8 31  1  0  0  0
 10 15  1  0  0  0
 10 32  1  0  0  0
 11 27  1  0  0  0
 11 33  1  0  0  0
 12 13  1  0  0  0
 12 15  2  0  0  0
 13 34  1  0  0  0
 13 35  1  0  0  0
 14 20  2  0  0  0
 14 18  1  0  0  0
 14 19  1  0  0  0
 15 36  1  0  0  0
 16 29  1  0  0  0
 17 26  1  0  0  0
 17 22  1  0  0  0
 17 23  2  0  0  0
 19 22  2  0  0  0
 19 37  1  0  0  0
 20 23  1  0  0  0
 20 38  1  0  0  0
 21 28  1  0  0  0
 22 39  1  0  0  0
 23 40  1  0  0  0
 24 26  1  0  0  0
 24 41  1  0  0  0
 24 42  1  0  0  0
 25 43  1  0  0  0
 25 44  1  0  0  0
 25 45  1  0  0  0
 26 46  1  0  0  0
 26 47  1  0  0  0
 27 28  1  0  0  0
```

```
 27 48  1  0  0  0
 27 49  1  0  0  0
 28 50  1  0  0  0
 28 51  1  0  0  0
 29 52  1  0  0  0
 29 53  1  0  0  0
 29 54  1  0  0  0
M  END
> <s_m_entry_id>
66

> <s_m_entry_name>
C_8.1

> <s_m_Source_Path>
C:\Users\julio\OneDrive - Universidad de Talca\Escritorio\Nueva carpeta

> <s_m_Source_File>
C_8.mol2

> <i_m_Source_File_Index>
1

$$$$
```

Coordinates of TRPV1 in PDB format in conformation for docking of compounds from series C_1.

```
ATOM      1  N   THR A 335      75.504 109.932  96.831  1.00 36.89           N1+
ATOM      2  CA  THR A 335      75.759 110.586  95.499  1.00 36.74           C
ATOM      3  C   THR A 335      77.271 110.382  95.168  1.00 36.68           C
ATOM      4  O   THR A 335      78.057 110.081  96.068  1.00 36.82           O
ATOM      5  CB  THR A 335      75.418 112.109  95.494  1.00 36.74           C
ATOM      6  CG2 THR A 335      73.937 112.394  95.787  1.00 36.34           C
ATOM      7  OG1 THR A 335      76.191 112.835  96.438  1.00 37.07           O
ATOM      8  HA  THR A 335      75.121 110.025  94.816  1.00  0.00           H
ATOM      9  HB  THR A 335      75.637 112.536  94.516  1.00  0.00           H
ATOM     10  HG1 THR A 335      75.833 113.725  96.507  1.00  0.00           H
ATOM     11 HG21 THR A 335      73.723 113.462  95.729  1.00  0.00           H
ATOM     12 HG22 THR A 335      73.292 111.893  95.065  1.00  0.00           H
ATOM     13 HG23 THR A 335      73.648 112.056  96.783  1.00  0.00           H
ATOM     14  H1  THR A 335      74.526 110.025  97.065  1.00  0.00           H
ATOM     15  H2  THR A 335      75.754 108.954  96.779  1.00  0.00           H
ATOM     16  H   THR A 335      76.066 110.400  97.529  1.00  0.00           H
ATOM     17  N   PRO A 336      77.671 110.560  93.883  1.00 36.46           N
ATOM     18  CA  PRO A 336      79.091 110.496  93.469  1.00 35.71           C
ATOM     19  C   PRO A 336      80.028 111.532  94.123  1.00 35.26           C
ATOM     20  O   PRO A 336      81.189 111.205  94.368  1.00 35.27           O
ATOM     21  CB  PRO A 336      79.048 110.659  91.939  1.00 36.20           C
ATOM     22  CG  PRO A 336      77.646 110.224  91.547  1.00 36.12           C
ATOM     23  CD  PRO A 336      76.802 110.715  92.714  1.00 36.23           C
ATOM     24  HA  PRO A 336      79.451 109.492  93.706  1.00  0.00           H
ATOM     25  HB2 PRO A 336      79.192 111.703  91.654  1.00  0.00           H
ATOM     26  HB3 PRO A 336      79.816 110.073  91.433  1.00  0.00           H
ATOM     27  HG2 PRO A 336      77.323 110.614  90.582  1.00  0.00           H
ATOM     28  HG3 PRO A 336      77.605 109.135  91.501  1.00  0.00           H
ATOM     29  HD2 PRO A 336      75.870 110.152  92.768  1.00  0.00           H
ATOM     30  HD3 PRO A 336      76.563 111.770  92.583  1.00  0.00           H
ATOM     31  N   LEU A 337      79.508 112.740  94.414  1.00 35.26           N
ATOM     32  CA  LEU A 337      80.230 113.815  95.103  1.00 34.53           C
ATOM     33  C   LEU A 337      80.503 113.482  96.581  1.00 34.37           C
ATOM     34  O   LEU A 337      81.622 113.693  97.047  1.00 34.02           O
ATOM     35  CB  LEU A 337      79.449 115.142  94.930  1.00 34.61           C
ATOM     36  CG  LEU A 337      80.056 116.393  95.617  1.00 34.07           C
ATOM     37  CD1 LEU A 337      81.512 116.674  95.183  1.00 34.18           C
ATOM     38  CD2 LEU A 337      79.141 117.623  95.439  1.00 32.60           C
ATOM     39  H   LEU A 337      78.548 112.935  94.174  1.00  0.00           H
ATOM     40  HA  LEU A 337      81.198 113.915  94.610  1.00  0.00           H
ATOM     41  HB2 LEU A 337      79.334 115.343  93.865  1.00  0.00           H
ATOM     42  HB3 LEU A 337      78.437 114.999  95.312  1.00  0.00           H
ATOM     43  HG  LEU A 337      80.079 116.201  96.690  1.00  0.00           H
ATOM     44 HD11 LEU A 337      81.687 117.719  94.928  1.00  0.00           H
ATOM     45 HD12 LEU A 337      82.201 116.427  95.990  1.00  0.00           H
ATOM     46 HD13 LEU A 337      81.812 116.083  94.320  1.00  0.00           H
ATOM     47 HD21 LEU A 337      78.869 118.042  96.409  1.00  0.00           H
ATOM     48 HD22 LEU A 337      79.609 118.422  94.864  1.00  0.00           H
ATOM     49 HD23 LEU A 337      78.212 117.370  94.928  1.00  0.00           H
ATOM     50  N   ALA A 338      79.487 112.934  97.273  1.00 33.95           N
ATOM     51  CA  ALA A 338      79.596 112.450  98.649  1.00 34.02           C
ATOM     52  C   ALA A 338      80.452 111.176  98.773  1.00 34.07           C
ATOM     53  O   ALA A 338      81.095 110.995  99.803  1.00 34.62           O
ATOM     54  CB  ALA A 338      78.188 112.221  99.218  1.00 34.42           C
ATOM     55  H   ALA A 338      78.593 112.797  96.823  1.00  0.00           H
ATOM     56  HA  ALA A 338      80.076 113.229  99.245  1.00  0.00           H
ATOM     57  HB1 ALA A 338      78.231 111.876 100.252  1.00  0.00           H
ATOM     58  HB2 ALA A 338      77.608 113.144  99.209  1.00  0.00           H
ATOM     59  HB3 ALA A 338      77.640 111.476  98.641  1.00  0.00           H
ATOM     60  N   LEU A 339      80.477 110.335  97.722  1.00 33.85           N
ATOM     61  CA  LEU A 339      81.305 109.130  97.644  1.00 33.53           C
```

```
ATOM     62  C   LEU A 339      82.803 109.465  97.519  1.00 33.66           C
ATOM     63  O   LEU A 339      83.615 108.796  98.157  1.00 34.18           O
ATOM     64  CB  LEU A 339      80.774 108.206  96.524  1.00 33.69           C
ATOM     65  CG  LEU A 339      81.368 106.775  96.501  1.00 33.84           C
ATOM     66  CD1 LEU A 339      80.351 105.761  95.931  1.00 31.31           C
ATOM     67  CD2 LEU A 339      82.727 106.701  95.766  1.00 33.49           C
ATOM     68  H   LEU A 339      79.903 110.534  96.914  1.00  0.00           H
ATOM     69  HA  LEU A 339      81.163 108.594  98.581  1.00  0.00           H
ATOM     70  HB2 LEU A 339      79.702 108.110  96.696  1.00  0.00           H
ATOM     71  HB3 LEU A 339      80.870 108.678  95.545  1.00  0.00           H
ATOM     72  HG  LEU A 339      81.546 106.474  97.535  1.00  0.00           H
ATOM     73 HD11 LEU A 339      80.806 105.020  95.275  1.00  0.00           H
ATOM     74 HD12 LEU A 339      79.868 105.210  96.739  1.00  0.00           H
ATOM     75 HD13 LEU A 339      79.563 106.249  95.357  1.00  0.00           H
ATOM     76 HD21 LEU A 339      82.737 105.969  94.959  1.00  0.00           H
ATOM     77 HD22 LEU A 339      83.005 107.655  95.318  1.00  0.00           H
ATOM     78 HD23 LEU A 339      83.524 106.426  96.458  1.00  0.00           H
ATOM     79  N   ALA A 340      83.132 110.519  96.749  1.00 33.60           N
ATOM     80  CA  ALA A 340      84.486 111.059  96.606  1.00 33.36           C
ATOM     81  C   ALA A 340      85.039 111.665  97.910  1.00 33.23           C
ATOM     82  O   ALA A 340      86.233 111.524  98.168  1.00 33.09           O
ATOM     83  CB  ALA A 340      84.501 112.090  95.466  1.00 33.41           C
ATOM     84  H   ALA A 340      82.406 111.009  96.245  1.00  0.00           H
ATOM     85  HA  ALA A 340      85.142 110.234  96.320  1.00  0.00           H
ATOM     86  HB1 ALA A 340      85.511 112.454  95.274  1.00  0.00           H
ATOM     87  HB2 ALA A 340      84.133 111.651  94.538  1.00  0.00           H
ATOM     88  HB3 ALA A 340      83.875 112.954  95.693  1.00  0.00           H
ATOM     89  N   ALA A 341      84.160 112.282  98.722  1.00 33.42           N
ATOM     90  CA  ALA A 341      84.482 112.811 100.049  1.00 33.00           C
ATOM     91  C   ALA A 341      84.670 111.706 101.104  1.00 33.03           C
ATOM     92  O   ALA A 341      85.585 111.811 101.920  1.00 33.45           O
ATOM     93  CB  ALA A 341      83.383 113.793 100.484  1.00 32.81           C
ATOM     94  H   ALA A 341      83.197 112.372  98.430  1.00  0.00           H
ATOM     95  HA  ALA A 341      85.420 113.368  99.980  1.00  0.00           H
ATOM     96  HB1 ALA A 341      83.590 114.215 101.468  1.00  0.00           H
ATOM     97  HB2 ALA A 341      83.306 114.623  99.787  1.00  0.00           H
ATOM     98  HB3 ALA A 341      82.405 113.315 100.523  1.00  0.00           H
ATOM     99  N   SER A 342      83.818 110.665 101.058  1.00 32.96           N
ATOM    100  CA  SER A 342      83.824 109.528 101.988  1.00 32.56           C
ATOM    101  C   SER A 342      84.929 108.485 101.723  1.00 32.96           C
ATOM    102  O   SER A 342      85.004 107.512 102.473  1.00 33.30           O
ATOM    103  CB  SER A 342      82.419 108.882 102.030  1.00 32.35           C
ATOM    104  OG  SER A 342      82.154 108.068 100.906  1.00 30.21           O
ATOM    105  H   SER A 342      83.082 110.664 100.365  1.00  0.00           H
ATOM    106  HA  SER A 342      84.014 109.933 102.984  1.00  0.00           H
ATOM    107  HB2 SER A 342      81.636 109.632 102.114  1.00  0.00           H
ATOM    108  HB3 SER A 342      82.331 108.254 102.917  1.00  0.00           H
ATOM    109  HG  SER A 342      82.105 108.630 100.130  1.00  0.00           H
ATOM    110  N   SER A 343      85.756 108.694 100.686  1.00 32.75           N
ATOM    111  CA  SER A 343      86.871 107.820 100.319  1.00 32.98           C
ATOM    112  C   SER A 343      88.196 108.581 100.119  1.00 33.17           C
ATOM    113  O   SER A 343      89.223 107.921  99.956  1.00 32.85           O
ATOM    114  CB  SER A 343      86.459 106.940  99.118  1.00 32.58           C
ATOM    115  OG  SER A 343      86.299 107.678  97.922  1.00 32.92           O
ATOM    116  H   SER A 343      85.613 109.504 100.101  1.00  0.00           H
ATOM    117  HA  SER A 343      87.073 107.137 101.147  1.00  0.00           H
ATOM    118  HB2 SER A 343      85.528 106.415  99.335  1.00  0.00           H
ATOM    119  HB3 SER A 343      87.213 106.171  98.945  1.00  0.00           H
ATOM    120  HG  SER A 343      85.463 108.153  97.976  1.00  0.00           H
ATOM    121  N   GLY A 344      88.173 109.927 100.190  1.00 33.56           N
ATOM    122  CA  GLY A 344      89.344 110.803 100.090  1.00 34.45           C
ATOM    123  C   GLY A 344      89.907 110.760  98.667  1.00 35.18           C
ATOM    124  O   GLY A 344      90.850 110.016  98.399  1.00 35.31           O
ATOM    125  H   GLY A 344      87.290 110.391 100.346  1.00  0.00           H
ATOM    126  HA2 GLY A 344      90.110 110.498 100.804  1.00  0.00           H
ATOM    127  HA3 GLY A 344      89.055 111.823 100.347  1.00  0.00           H
ATOM    128  N   LYS A 345      89.302 111.540  97.760  1.00 35.75           N
ATOM    129  CA  LYS A 345      89.635 111.572  96.338  1.00 36.22           C
ATOM    130  C   LYS A 345      89.688 113.041  95.905  1.00 36.63           C
ATOM    131  O   LYS A 345      88.710 113.546  95.354  1.00 36.92           O
ATOM    132  CB  LYS A 345      88.588 110.760  95.532  1.00 36.19           C
```

```
ATOM    133  CG  LYS A 345      88.419 109.287  95.943  1.00 36.14           C
ATOM    134  CD  LYS A 345      89.627 108.390  95.624  1.00 36.58           C
ATOM    135  CE  LYS A 345      89.578 107.028  96.333  1.00 37.13           C
ATOM    136  NZ  LYS A 345      88.337 106.290  96.042  1.00 37.20           N1+
ATOM    137  H   LYS A 345      88.522 112.118  98.043  1.00  0.00           H
ATOM    138  HA  LYS A 345      90.627 111.155  96.158  1.00  0.00           H
ATOM    139  HB2 LYS A 345      87.611 111.236  95.630  1.00  0.00           H
ATOM    140  HB3 LYS A 345      88.841 110.798  94.473  1.00  0.00           H
ATOM    141  HG2 LYS A 345      88.191 109.233  97.005  1.00  0.00           H
ATOM    142  HG3 LYS A 345      87.537 108.892  95.438  1.00  0.00           H
ATOM    143  HD2 LYS A 345      89.681 108.234  94.545  1.00  0.00           H
ATOM    144  HD3 LYS A 345      90.556 108.890  95.896  1.00  0.00           H
ATOM    145  HE2 LYS A 345      89.655 107.164  97.412  1.00  0.00           H
ATOM    146  HE3 LYS A 345      90.431 106.420  96.029  1.00  0.00           H
ATOM    147  HZ1 LYS A 345      87.549 106.816  96.397  1.00  0.00           H
ATOM    148  HZ2 LYS A 345      88.240 106.162  95.045  1.00  0.00           H
ATOM    149  HZ3 LYS A 345      88.365 105.389  96.497  1.00  0.00           H
ATOM    150  N   ILE A 346      90.825 113.702  96.190  1.00 36.53           N
ATOM    151  CA  ILE A 346      91.063 115.132  95.946  1.00 36.02           C
ATOM    152  C   ILE A 346      90.827 115.557  94.482  1.00 36.09           C
ATOM    153  O   ILE A 346      90.151 116.559  94.268  1.00 36.19           O
ATOM    154  CB  ILE A 346      92.501 115.568  96.380  1.00 35.75           C
ATOM    155  CG1 ILE A 346      92.697 115.424  97.907  1.00 35.02           C
ATOM    156  CG2 ILE A 346      92.915 116.997  95.944  1.00 35.66           C
ATOM    157  CD1 ILE A 346      94.165 115.257  98.328  1.00 35.16           C
ATOM    158  H   ILE A 346      91.569 113.214  96.666  1.00  0.00           H
ATOM    159  HA  ILE A 346      90.344 115.677  96.564  1.00  0.00           H
ATOM    160  HB  ILE A 346      93.197 114.877  95.903  1.00  0.00           H
ATOM    161 HG12 ILE A 346      92.144 114.566  98.288  1.00  0.00           H
ATOM    162 HG13 ILE A 346      92.270 116.289  98.415  1.00  0.00           H
ATOM    163 HG21 ILE A 346      93.900 117.263  96.324  1.00  0.00           H
ATOM    164 HG22 ILE A 346      92.975 117.107  94.862  1.00  0.00           H
ATOM    165 HG23 ILE A 346      92.210 117.741  96.318  1.00  0.00           H
ATOM    166 HD11 ILE A 346      94.237 115.091  99.403  1.00  0.00           H
ATOM    167 HD12 ILE A 346      94.625 114.403  97.830  1.00  0.00           H
ATOM    168 HD13 ILE A 346      94.759 116.140  98.097  1.00  0.00           H
ATOM    169  N   GLY A 347      91.360 114.787  93.517  1.00 35.51           N
ATOM    170  CA  GLY A 347      91.288 115.104  92.088  1.00 35.19           C
ATOM    171  C   GLY A 347      89.894 114.850  91.490  1.00 34.92           C
ATOM    172  O   GLY A 347      89.498 115.565  90.570  1.00 34.78           O
ATOM    173  H   GLY A 347      91.892 113.970  93.779  1.00  0.00           H
ATOM    174  HA2 GLY A 347      91.577 116.144  91.922  1.00  0.00           H
ATOM    175  HA3 GLY A 347      92.013 114.487  91.556  1.00  0.00           H
ATOM    176  N   VAL A 348      89.146 113.863  92.018  1.00 34.20           N
ATOM    177  CA  VAL A 348      87.774 113.548  91.602  1.00 33.89           C
ATOM    178  C   VAL A 348      86.783 114.602  92.130  1.00 34.13           C
ATOM    179  O   VAL A 348      85.941 115.080  91.370  1.00 34.36           O
ATOM    180  CB  VAL A 348      87.329 112.146  92.117  1.00 33.36           C
ATOM    181  CG1 VAL A 348      85.852 111.793  91.834  1.00 33.30           C
ATOM    182  CG2 VAL A 348      88.236 111.031  91.566  1.00 34.16           C
ATOM    183  H   VAL A 348      89.516 113.318  92.783  1.00  0.00           H
ATOM    184  HA  VAL A 348      87.730 113.550  90.511  1.00  0.00           H
ATOM    185  HB  VAL A 348      87.442 112.138  93.201  1.00  0.00           H
ATOM    186 HG11 VAL A 348      85.628 110.771  92.142  1.00  0.00           H
ATOM    187 HG12 VAL A 348      85.160 112.436  92.378  1.00  0.00           H
ATOM    188 HG13 VAL A 348      85.622 111.876  90.772  1.00  0.00           H
ATOM    189 HG21 VAL A 348      87.931 110.054  91.940  1.00  0.00           H
ATOM    190 HG22 VAL A 348      88.200 110.996  90.476  1.00  0.00           H
ATOM    191 HG23 VAL A 348      89.276 111.174  91.860  1.00  0.00           H
ATOM    192  N   LEU A 349      86.931 114.952  93.417  1.00 34.10           N
ATOM    193  CA  LEU A 349      86.126 115.940  94.128  1.00 33.94           C
ATOM    194  C   LEU A 349      86.382 117.374  93.628  1.00 33.11           C
ATOM    195  O   LEU A 349      85.422 118.132  93.503  1.00 32.65           O
ATOM    196  CB  LEU A 349      86.370 115.742  95.641  1.00 34.24           C
ATOM    197  CG  LEU A 349      85.421 116.516  96.585  1.00 34.70           C
ATOM    198  CD1 LEU A 349      84.915 115.640  97.747  1.00 35.42           C
ATOM    199  CD2 LEU A 349      86.091 117.780  97.136  1.00 37.49           C
ATOM    200  H   LEU A 349      87.654 114.505  93.968  1.00  0.00           H
ATOM    201  HA  LEU A 349      85.078 115.705  93.927  1.00  0.00           H
ATOM    202  HB2 LEU A 349      86.243 114.676  95.829  1.00  0.00           H
ATOM    203  HB3 LEU A 349      87.412 115.949  95.893  1.00  0.00           H
```

```
ATOM    204  HG  LEU A 349      84.544 116.828  96.018  1.00  0.00           H
ATOM    205 HD11 LEU A 349      84.940 116.170  98.700  1.00  0.00           H
ATOM    206 HD12 LEU A 349      83.884 115.331  97.574  1.00  0.00           H
ATOM    207 HD13 LEU A 349      85.511 114.738  97.880  1.00  0.00           H
ATOM    208 HD21 LEU A 349      85.416 118.342  97.781  1.00  0.00           H
ATOM    209 HD22 LEU A 349      86.975 117.530  97.724  1.00  0.00           H
ATOM    210 HD23 LEU A 349      86.401 118.440  96.329  1.00  0.00           H
ATOM    211  N   ALA A 350      87.644 117.689  93.277  1.00 32.35           N
ATOM    212  CA  ALA A 350      88.043 118.940  92.628  1.00 31.91           C
ATOM    213  C   ALA A 350      87.485 119.095  91.208  1.00 31.54           C
ATOM    214  O   ALA A 350      87.113 120.209  90.852  1.00 31.60           O
ATOM    215  CB  ALA A 350      89.573 119.069  92.592  1.00 31.68           C
ATOM    216  H   ALA A 350      88.386 117.018  93.431  1.00  0.00           H
ATOM    217  HA  ALA A 350      87.650 119.761  93.231  1.00  0.00           H
ATOM    218  HB1 ALA A 350      89.882 119.987  92.092  1.00  0.00           H
ATOM    219  HB2 ALA A 350      89.992 119.100  93.594  1.00  0.00           H
ATOM    220  HB3 ALA A 350      90.033 118.234  92.063  1.00  0.00           H
ATOM    221  N   TYR A 351      87.411 117.997  90.431  1.00 30.99           N
ATOM    222  CA  TYR A 351      86.812 117.997  89.093  1.00 30.59           C
ATOM    223  C   TYR A 351      85.308 118.325  89.119  1.00 31.05           C
ATOM    224  O   TYR A 351      84.868 119.165  88.336  1.00 31.61           O
ATOM    225  CB  TYR A 351      87.110 116.673  88.350  1.00 29.56           C
ATOM    226  CG  TYR A 351      86.345 116.518  87.042  1.00 28.61           C
ATOM    227  CD1 TYR A 351      86.697 117.305  85.926  1.00 26.21           C
ATOM    228  CD2 TYR A 351      85.224 115.662  86.968  1.00 29.08           C
ATOM    229  CE1 TYR A 351      85.916 117.262  84.754  1.00 23.37           C
ATOM    230  CE2 TYR A 351      84.444 115.621  85.797  1.00 28.61           C
ATOM    231  CZ  TYR A 351      84.783 116.428  84.693  1.00 27.49           C
ATOM    232  OH  TYR A 351      84.009 116.404  83.570  1.00 29.18           O
ATOM    233  H   TYR A 351      87.753 117.110  90.775  1.00  0.00           H
ATOM    234  HA  TYR A 351      87.303 118.794  88.530  1.00  0.00           H
ATOM    235  HB2 TYR A 351      88.178 116.591  88.148  1.00  0.00           H
ATOM    236  HB3 TYR A 351      86.863 115.826  88.990  1.00  0.00           H
ATOM    237  HD1 TYR A 351      87.547 117.970  85.978  1.00  0.00           H
ATOM    238  HD2 TYR A 351      84.941 115.061  87.820  1.00  0.00           H
ATOM    239  HE1 TYR A 351      86.183 117.883  83.911  1.00  0.00           H
ATOM    240  HE2 TYR A 351      83.577 114.977  85.749  1.00  0.00           H
ATOM    241  HH  TYR A 351      84.342 116.968  82.868  1.00  0.00           H
ATOM    242  N   ILE A 352      84.562 117.677  90.029  1.00 30.80           N
ATOM    243  CA  ILE A 352      83.121 117.879  90.219  1.00 30.99           C
ATOM    244  C   ILE A 352      82.768 119.306  90.704  1.00 31.33           C
ATOM    245  O   ILE A 352      81.667 119.781  90.422  1.00 32.20           O
ATOM    246  CB  ILE A 352      82.538 116.821  91.210  1.00 31.03           C
ATOM    247  CG1 ILE A 352      82.660 115.388  90.633  1.00 29.77           C
ATOM    248  CG2 ILE A 352      81.077 117.072  91.649  1.00 30.83           C
ATOM    249  CD1 ILE A 352      82.581 114.279  91.695  1.00 28.73           C
ATOM    250  H   ILE A 352      85.001 116.999  90.637  1.00  0.00           H
ATOM    251  HA  ILE A 352      82.642 117.738  89.250  1.00  0.00           H
ATOM    252  HB  ILE A 352      83.153 116.857  92.112  1.00  0.00           H
ATOM    253 HG12 ILE A 352      81.883 115.229  89.885  1.00  0.00           H
ATOM    254 HG13 ILE A 352      83.601 115.264  90.098  1.00  0.00           H
ATOM    255 HG21 ILE A 352      80.687 116.241  92.232  1.00  0.00           H
ATOM    256 HG22 ILE A 352      80.982 117.957  92.278  1.00  0.00           H
ATOM    257 HG23 ILE A 352      80.422 117.195  90.785  1.00  0.00           H
ATOM    258 HD11 ILE A 352      83.024 113.356  91.321  1.00  0.00           H
ATOM    259 HD12 ILE A 352      83.117 114.551  92.604  1.00  0.00           H
ATOM    260 HD13 ILE A 352      81.548 114.057  91.967  1.00  0.00           H
ATOM    261  N   LEU A 353      83.715 119.958  91.398  1.00 30.90           N
ATOM    262  CA  LEU A 353      83.544 121.274  92.001  1.00 30.99           C
ATOM    263  C   LEU A 353      84.009 122.445  91.115  1.00 31.13           C
ATOM    264  O   LEU A 353      83.408 123.514  91.211  1.00 31.12           O
ATOM    265  CB  LEU A 353      84.234 121.279  93.374  1.00 30.86           C
ATOM    266  CG  LEU A 353      83.387 120.575  94.465  1.00 29.88           C
ATOM    267  CD1 LEU A 353      84.211 120.275  95.727  1.00 29.83           C
ATOM    268  CD2 LEU A 353      82.079 121.330  94.795  1.00 30.44           C
ATOM    269  H   LEU A 353      84.596 119.498  91.583  1.00  0.00           H
ATOM    270  HA  LEU A 353      82.488 121.420  92.193  1.00  0.00           H
ATOM    271  HB2 LEU A 353      85.211 120.803  93.282  1.00  0.00           H
ATOM    272  HB3 LEU A 353      84.443 122.304  93.671  1.00  0.00           H
ATOM    273  HG  LEU A 353      83.100 119.602  94.062  1.00  0.00           H
ATOM    274 HD11 LEU A 353      84.006 119.262  96.071  1.00  0.00           H
```

```
ATOM    275 HD12 LEU A 353      85.280 120.348  95.535  1.00  0.00           H
ATOM    276 HD13 LEU A 353      83.985 120.947  96.553  1.00  0.00           H
ATOM    277 HD21 LEU A 353      81.956 121.504  95.864  1.00  0.00           H
ATOM    278 HD22 LEU A 353      82.035 122.307  94.312  1.00  0.00           H
ATOM    279 HD23 LEU A 353      81.210 120.762  94.462  1.00  0.00           H
ATOM    280  N   GLN A 354      85.045 122.246  90.282  1.00 31.81           N
ATOM    281  CA  GLN A 354      85.649 123.287  89.436  1.00 32.41           C
ATOM    282  C   GLN A 354      85.383 123.089  87.930  1.00 33.15           C
ATOM    283  O   GLN A 354      85.996 123.797  87.130  1.00 33.51           O
ATOM    284  CB  GLN A 354      87.163 123.454  89.754  1.00 32.04           C
ATOM    285  CG  GLN A 354      87.501 124.377  90.947  1.00 30.84           C
ATOM    286  CD  GLN A 354      87.032 123.888  92.318  1.00 31.08           C
ATOM    287  NE2 GLN A 354      87.340 122.639  92.669  1.00 31.66           N
ATOM    288  OE1 GLN A 354      86.410 124.637  93.063  1.00 32.26           O
ATOM    289  H   GLN A 354      85.504 121.344  90.266  1.00  0.00           H
ATOM    290  HA  GLN A 354      85.181 124.249  89.654  1.00  0.00           H
ATOM    291  HB2 GLN A 354      87.635 122.481  89.880  1.00  0.00           H
ATOM    292  HB3 GLN A 354      87.683 123.891  88.902  1.00  0.00           H
ATOM    293  HG2 GLN A 354      87.088 125.370  90.770  1.00  0.00           H
ATOM    294  HG3 GLN A 354      88.582 124.509  91.003  1.00  0.00           H
ATOM    295 HE21 GLN A 354      87.075 122.295  93.579  1.00  0.00           H
ATOM    296 HE22 GLN A 354      87.840 122.034  92.032  1.00  0.00           H
ATOM    297  N   ARG A 355      84.465 122.176  87.554  1.00 34.49           N
ATOM    298  CA  ARG A 355      84.038 121.968  86.164  1.00 35.92           C
ATOM    299  C   ARG A 355      83.409 123.242  85.563  1.00 36.56           C
ATOM    300  O   ARG A 355      82.562 123.871  86.199  1.00 37.15           O
ATOM    301  CB  ARG A 355      83.069 120.762  86.045  1.00 20.00           C
ATOM    302  CG  ARG A 355      81.825 120.838  86.948  1.00 20.00           C
ATOM    303  CD  ARG A 355      80.718 119.831  86.618  1.00 20.00           C
ATOM    304  NE  ARG A 355      81.074 118.455  86.984  1.00 20.00           N
ATOM    305  CZ  ARG A 355      80.226 117.413  86.953  1.00 20.00           C
ATOM    306  NH1 ARG A 355      78.948 117.564  86.571  1.00 20.00           N
ATOM    307  NH2 ARG A 355      80.666 116.200  87.310  1.00 20.00           N1+
ATOM    308  H   ARG A 355      84.007 121.612  88.256  1.00  0.00           H
ATOM    309  HA  ARG A 355      84.936 121.722  85.593  1.00  0.00           H
ATOM    310  HB2 ARG A 355      83.601 119.832  86.235  1.00  0.00           H
ATOM    311  HB3 ARG A 355      82.746 120.696  85.006  1.00  0.00           H
ATOM    312  HG2 ARG A 355      82.153 120.694  87.977  1.00  0.00           H
ATOM    313  HG3 ARG A 355      81.373 121.821  86.914  1.00  0.00           H
ATOM    314  HD2 ARG A 355      80.545 119.821  85.541  1.00  0.00           H
ATOM    315  HD3 ARG A 355      79.784 120.143  87.085  1.00  0.00           H
ATOM    316  HE  ARG A 355      82.040 118.298  87.235  1.00  0.00           H
ATOM    317 HH11 ARG A 355      78.319 116.776  86.549  1.00  0.00           H
ATOM    318 HH12 ARG A 355      78.609 118.475  86.287  1.00  0.00           H
ATOM    319 HH21 ARG A 355      80.046 115.403  87.285  1.00  0.00           H
ATOM    320 HH22 ARG A 355      81.629 116.066  87.584  1.00  0.00           H
ATOM    321  N   GLU A 356      83.835 123.566  84.336  1.00 30.00           N
ATOM    322  CA  GLU A 356      83.268 124.621  83.507  1.00 30.00           C
ATOM    323  C   GLU A 356      83.115 124.032  82.104  1.00 30.00           C
ATOM    324  O   GLU A 356      84.082 124.014  81.341  1.00 30.00           O
ATOM    325  CB  GLU A 356      84.120 125.910  83.592  1.00 20.00           C
ATOM    326  CG  GLU A 356      83.713 127.071  82.650  1.00  0.00           C
ATOM    327  CD  GLU A 356      82.303 127.626  82.883  1.00  0.00           C
ATOM    328  OE1 GLU A 356      81.330 126.901  82.573  1.00  0.00           O
ATOM    329  OE2 GLU A 356      82.221 128.780  83.359  1.00  0.00           O1-
ATOM    330  H   GLU A 356      84.548 123.006  83.893  1.00  0.00           H
ATOM    331  HA  GLU A 356      82.275 124.863  83.870  1.00  0.00           H
ATOM    332  HB2 GLU A 356      84.096 126.277  84.619  1.00  0.00           H
ATOM    333  HB3 GLU A 356      85.165 125.663  83.400  1.00  0.00           H
ATOM    334  HG2 GLU A 356      84.427 127.887  82.764  1.00  0.00           H
ATOM    335  HG3 GLU A 356      83.786 126.761  81.609  1.00  0.00           H
ATOM    336  N   ILE A 357      81.917 123.491  81.833  1.00 30.00           N
ATOM    337  CA  ILE A 357      81.599 122.767  80.604  1.00 30.00           C
ATOM    338  C   ILE A 357      80.799 123.719  79.683  1.00 30.00           C
ATOM    339  O   ILE A 357      79.599 123.538  79.469  1.00 30.00           O
ATOM    340  CB  ILE A 357      80.793 121.458  80.895  1.00 20.00           C
ATOM    341  CG1 ILE A 357      81.337 120.629  82.087  1.00  0.00           C
ATOM    342  CG2 ILE A 357      80.675 120.565  79.640  1.00  0.00           C
ATOM    343  CD1 ILE A 357      82.786 120.135  81.942  1.00  0.00           C
ATOM    344  H   ILE A 357      81.177 123.558  82.516  1.00  0.00           H
ATOM    345  HA  ILE A 357      82.512 122.491  80.072  1.00  0.00           H
```

```
ATOM    346  HB  ILE A 357      79.778 121.736  81.185  1.00  0.00           H
ATOM    347 HG12 ILE A 357      81.254 121.208  83.007  1.00  0.00           H
ATOM    348 HG13 ILE A 357      80.688 119.766  82.242  1.00  0.00           H
ATOM    349 HG21 ILE A 357      80.016 119.715  79.821  1.00  0.00           H
ATOM    350 HG22 ILE A 357      80.282 121.105  78.782  1.00  0.00           H
ATOM    351 HG23 ILE A 357      81.645 120.173  79.335  1.00  0.00           H
ATOM    352 HD11 ILE A 357      83.124 119.657  82.862  1.00  0.00           H
ATOM    353 HD12 ILE A 357      82.880 119.398  81.145  1.00  0.00           H
ATOM    354 HD13 ILE A 357      83.475 120.952  81.728  1.00  0.00           H
ATOM    355  N   HIS A 358      81.492 124.760  79.190  1.00 30.00           N
ATOM    356  CA  HIS A 358      80.957 125.799  78.307  1.00 30.00           C
ATOM    357  C   HIS A 358      80.705 125.248  76.890  1.00 30.00           C
ATOM    358  O   HIS A 358      81.627 125.186  76.076  1.00 30.00           O
ATOM    359  CB  HIS A 358      81.885 127.037  78.322  1.00 20.00           C
ATOM    360  CG  HIS A 358      83.354 126.795  78.051  1.00 20.00           C
ATOM    361  CD2 HIS A 358      84.079 127.072  76.910  1.00 20.00           C
ATOM    362  ND1 HIS A 358      84.213 126.228  78.998  1.00 20.00           N
ATOM    363  CE1 HIS A 358      85.397 126.176  78.404  1.00 20.00           C
ATOM    364  NE2 HIS A 358      85.376 126.662  77.163  1.00 20.00           N
ATOM    365  H   HIS A 358      82.468 124.859  79.435  1.00  0.00           H
ATOM    366  HA  HIS A 358      79.994 126.123  78.709  1.00  0.00           H
ATOM    367  HB2 HIS A 358      81.812 127.518  79.299  1.00  0.00           H
ATOM    368  HB3 HIS A 358      81.523 127.776  77.605  1.00  0.00           H
ATOM    369  HD2 HIS A 358      83.781 127.505  75.967  1.00  0.00           H
ATOM    370  HE1 HIS A 358      86.283 125.781  78.877  1.00  0.00           H
ATOM    371  HE2 HIS A 358      86.160 126.719  76.528  1.00  0.00           H
ATOM    372  N   GLU A 359      79.458 124.818  76.651  1.00 30.00           N
ATOM    373  CA  GLU A 359      79.023 124.095  75.457  1.00 30.00           C
ATOM    374  C   GLU A 359      77.478 124.121  75.421  1.00 30.00           C
ATOM    375  O   GLU A 359      76.870 123.988  76.485  1.00 30.00           O
ATOM    376  CB  GLU A 359      79.588 122.652  75.541  1.00 20.00           C
ATOM    377  CG  GLU A 359      79.138 121.679  74.429  1.00 20.00           C
ATOM    378  CD  GLU A 359      79.715 120.266  74.567  1.00 20.00           C
ATOM    379  OE1 GLU A 359      80.263 119.950  75.646  1.00 20.00           O
ATOM    380  OE2 GLU A 359      79.587 119.515  73.576  1.00 20.00           O1-
ATOM    381  H   GLU A 359      78.766 124.884  77.385  1.00  0.00           H
ATOM    382  HA  GLU A 359      79.442 124.608  74.592  1.00  0.00           H
ATOM    383  HB2 GLU A 359      80.678 122.696  75.526  1.00  0.00           H
ATOM    384  HB3 GLU A 359      79.331 122.234  76.514  1.00  0.00           H
ATOM    385  HG2 GLU A 359      78.053 121.585  74.409  1.00  0.00           H
ATOM    386  HG3 GLU A 359      79.444 122.065  73.457  1.00  0.00           H
ATOM    387  N   PRO A 360      76.855 124.288  74.227  1.00 30.00           N
ATOM    388  CA  PRO A 360      75.385 124.223  74.083  1.00 30.00           C
ATOM    389  C   PRO A 360      74.812 122.846  74.471  1.00 30.00           C
ATOM    390  O   PRO A 360      75.315 121.823  74.009  1.00 30.00           O
ATOM    391  CB  PRO A 360      75.139 124.562  72.603  1.00 20.00           C
ATOM    392  CG  PRO A 360      76.427 124.177  71.896  1.00 20.00           C
ATOM    393  CD  PRO A 360      77.496 124.514  72.928  1.00 20.00           C
ATOM    394  HA  PRO A 360      74.925 124.991  74.708  1.00  0.00           H
ATOM    395  HB2 PRO A 360      74.268 124.063  72.176  1.00  0.00           H
ATOM    396  HB3 PRO A 360      74.976 125.637  72.504  1.00  0.00           H
ATOM    397  HG2 PRO A 360      76.430 123.103  71.708  1.00  0.00           H
ATOM    398  HG3 PRO A 360      76.569 124.685  70.942  1.00  0.00           H
ATOM    399  HD2 PRO A 360      78.385 123.911  72.757  1.00  0.00           H
ATOM    400  HD3 PRO A 360      77.779 125.565  72.859  1.00  0.00           H
ATOM    401  N   GLU A 361      73.798 122.870  75.353  1.00 30.00           N
ATOM    402  CA  GLU A 361      73.178 121.709  76.006  1.00 30.00           C
ATOM    403  C   GLU A 361      74.137 120.968  76.961  1.00 30.00           C
ATOM    404  O   GLU A 361      74.000 119.759  77.151  1.00 30.00           O
ATOM    405  CB  GLU A 361      72.484 120.764  74.986  1.00 20.00           C
ATOM    406  CG  GLU A 361      71.489 121.446  74.021  1.00  0.00           C
ATOM    407  CD  GLU A 361      70.356 122.207  74.716  1.00  0.00           C
ATOM    408  OE1 GLU A 361      69.932 121.758  75.804  1.00  0.00           O
ATOM    409  OE2 GLU A 361      69.941 123.241  74.150  1.00  0.00           O1-
ATOM    410  H   GLU A 361      73.434 123.766  75.643  1.00  0.00           H
ATOM    411  HA  GLU A 361      72.411 122.124  76.659  1.00  0.00           H
ATOM    412  HB2 GLU A 361      73.236 120.239  74.396  1.00  0.00           H
ATOM    413  HB3 GLU A 361      71.951 119.980  75.527  1.00  0.00           H
ATOM    414  HG2 GLU A 361      72.021 122.131  73.361  1.00  0.00           H
ATOM    415  HG3 GLU A 361      71.037 120.693  73.374  1.00  0.00           H
ATOM    416  N   CYS A 362      75.065 121.719  77.577  1.00 30.00           N
```

ATOM	417	CA	CYS	A	362	76.061	121.199	78.510	1.00	30.00	C
ATOM	418	C	CYS	A	362	76.497	122.236	79.562	1.00	30.00	C
ATOM	419	O	CYS	A	362	77.129	121.836	80.537	1.00	30.00	O
ATOM	420	CB	CYS	A	362	77.272	120.585	77.769	1.00	20.00	C
ATOM	421	SG	CYS	A	362	77.373	118.802	78.076	1.00	0.00	S
ATOM	422	H	CYS	A	362	75.152	122.693	77.325	1.00	0.00	H
ATOM	423	HA	CYS	A	362	75.571	120.424	79.102	1.00	0.00	H
ATOM	424	HB2	CYS	A	362	77.212	120.755	76.693	1.00	0.00	H
ATOM	425	HB3	CYS	A	362	78.207	121.032	78.096	1.00	0.00	H
ATOM	426	HG	CYS	A	362	76.284	118.481	77.370	1.00	0.00	H
ATOM	427	N	ARG	A	363	76.135	123.525	79.405	1.00	30.00	N
ATOM	428	CA	ARG	A	363	76.401	124.570	80.403	1.00	30.00	C
ATOM	429	C	ARG	A	363	75.560	124.403	81.689	1.00	30.00	C
ATOM	430	O	ARG	A	363	75.980	124.874	82.740	1.00	30.00	O
ATOM	431	CB	ARG	A	363	76.261	125.968	79.757	1.00	20.00	C
ATOM	432	CG	ARG	A	363	76.747	127.124	80.657	1.00	0.00	C
ATOM	433	CD	ARG	A	363	76.855	128.475	79.938	1.00	0.00	C
ATOM	434	NE	ARG	A	363	77.197	129.564	80.868	1.00	0.00	N
ATOM	435	CZ	ARG	A	363	76.339	130.253	81.641	1.00	0.00	C
ATOM	436	NH1	ARG	A	363	75.023	130.011	81.614	1.00	0.00	N
ATOM	437	NH2	ARG	A	363	76.804	131.206	82.459	1.00	0.00	N1+
ATOM	438	H	ARG	A	363	75.656	123.819	78.567	1.00	0.00	H
ATOM	439	HA	ARG	A	363	77.444	124.465	80.709	1.00	0.00	H
ATOM	440	HB2	ARG	A	363	76.845	125.982	78.835	1.00	0.00	H
ATOM	441	HB3	ARG	A	363	75.225	126.143	79.463	1.00	0.00	H
ATOM	442	HG2	ARG	A	363	76.170	127.225	81.574	1.00	0.00	H
ATOM	443	HG3	ARG	A	363	77.759	126.850	80.963	1.00	0.00	H
ATOM	444	HD2	ARG	A	363	77.735	128.417	79.296	1.00	0.00	H
ATOM	445	HD3	ARG	A	363	76.017	128.694	79.275	1.00	0.00	H
ATOM	446	HE	ARG	A	363	78.185	129.756	80.967	1.00	0.00	H
ATOM	447	HH11	ARG	A	363	74.395	130.537	82.207	1.00	0.00	H
ATOM	448	HH12	ARG	A	363	74.643	129.306	80.999	1.00	0.00	H
ATOM	449	HH21	ARG	A	363	76.168	131.721	83.053	1.00	0.00	H
ATOM	450	HH22	ARG	A	363	77.791	131.419	82.493	1.00	0.00	H
ATOM	451	N	HIS	A	364	74.431	123.680	81.610	1.00	30.00	N
ATOM	452	CA	HIS	A	364	73.646	123.234	82.767	1.00	30.00	C
ATOM	453	C	HIS	A	364	74.352	122.164	83.626	1.00	30.00	C
ATOM	454	O	HIS	A	364	74.050	122.069	84.816	1.00	30.00	O
ATOM	455	CB	HIS	A	364	72.267	122.746	82.278	1.00	20.00	C
ATOM	456	CG	HIS	A	364	72.313	121.511	81.407	1.00	20.00	C
ATOM	457	CD2	HIS	A	364	72.393	121.381	80.033	1.00	20.00	C
ATOM	458	ND1	HIS	A	364	72.316	120.228	81.937	1.00	20.00	N1+
ATOM	459	CE1	HIS	A	364	72.419	119.384	80.914	1.00	20.00	C
ATOM	460	NE2	HIS	A	364	72.472	120.028	79.752	1.00	20.00	N
ATOM	461	H	HIS	A	364	74.140	123.329	80.710	1.00	0.00	H
ATOM	462	HA	HIS	A	364	73.495	124.092	83.419	1.00	0.00	H
ATOM	463	HB2	HIS	A	364	71.769	123.541	81.722	1.00	0.00	H
ATOM	464	HB3	HIS	A	364	71.622	122.539	83.134	1.00	0.00	H
ATOM	465	HD1	HIS	A	364	72.269	119.980	82.916	1.00	0.00	H
ATOM	466	HD2	HIS	A	364	72.419	122.119	79.245	1.00	0.00	H
ATOM	467	HE1	HIS	A	364	72.459	118.309	81.014	1.00	0.00	H
ATOM	468	HE2	HIS	A	364	72.562	119.612	78.832	1.00	0.00	H
ATOM	469	N	LEU	A	365	75.260	121.385	83.010	1.00	30.00	N
ATOM	470	CA	LEU	A	365	76.057	120.336	83.652	1.00	30.00	C
ATOM	471	C	LEU	A	365	77.240	120.911	84.459	1.00	30.00	C
ATOM	472	O	LEU	A	365	77.756	120.215	85.334	1.00	30.00	O
ATOM	473	CB	LEU	A	365	76.533	119.350	82.556	1.00	20.00	C
ATOM	474	CG	LEU	A	365	77.027	117.973	83.052	1.00	20.00	C
ATOM	475	CD1	LEU	A	365	75.913	117.176	83.763	1.00	20.00	C
ATOM	476	CD2	LEU	A	365	77.674	117.175	81.899	1.00	20.00	C
ATOM	477	H	LEU	A	365	75.467	121.546	82.033	1.00	0.00	H
ATOM	478	HA	LEU	A	365	75.406	119.808	84.351	1.00	0.00	H
ATOM	479	HB2	LEU	A	365	75.728	119.185	81.838	1.00	0.00	H
ATOM	480	HB3	LEU	A	365	77.340	119.818	81.993	1.00	0.00	H
ATOM	481	HG	LEU	A	365	77.823	118.141	83.777	1.00	0.00	H
ATOM	482	HD11	LEU	A	365	75.884	116.129	83.461	1.00	0.00	H
ATOM	483	HD12	LEU	A	365	76.063	117.190	84.843	1.00	0.00	H
ATOM	484	HD13	LEU	A	365	74.925	117.592	83.566	1.00	0.00	H
ATOM	485	HD21	LEU	A	365	78.683	116.858	82.167	1.00	0.00	H
ATOM	486	HD22	LEU	A	365	77.112	116.277	81.637	1.00	0.00	H
ATOM	487	HD23	LEU	A	365	77.759	117.766	80.987	1.00	0.00	H

```
ATOM    488  N   SER A 366      77.625 122.168  84.166  1.00 30.00           N
ATOM    489  CA  SER A 366      78.653 122.943  84.859  1.00 30.00           C
ATOM    490  C   SER A 366      78.290 123.225  86.332  1.00 30.00           C
ATOM    491  O   SER A 366      77.110 123.278  86.684  1.00 30.00           O
ATOM    492  CB  SER A 366      78.880 124.254  84.075  1.00 20.00           C
ATOM    493  OG  SER A 366      79.942 125.010  84.608  1.00 20.00           O
ATOM    494  H   SER A 366      77.131 122.663  83.437  1.00  0.00           H
ATOM    495  HA  SER A 366      79.575 122.358  84.833  1.00  0.00           H
ATOM    496  HB2 SER A 366      79.086 124.051  83.023  1.00  0.00           H
ATOM    497  HB3 SER A 366      77.994 124.883  84.120  1.00  0.00           H
ATOM    498  HG  SER A 366      80.117 125.766  84.035  1.00  0.00           H
ATOM    499  N   ARG A 367      79.340 123.418  87.141  1.00 30.00           N
ATOM    500  CA  ARG A 367      79.274 123.754  88.558  1.00 30.00           C
ATOM    501  C   ARG A 367      79.785 125.182  88.777  1.00 30.00           C
ATOM    502  O   ARG A 367      79.103 125.969  89.427  1.00 30.00           O
ATOM    503  CB  ARG A 367      80.127 122.742  89.350  1.00 20.00           C
ATOM    504  CG  ARG A 367      80.166 122.989  90.865  1.00 20.00           C
ATOM    505  CD  ARG A 367      78.954 122.455  91.625  1.00 20.00           C
ATOM    506  NE  ARG A 367      78.951 120.988  91.716  1.00 20.00           N
ATOM    507  CZ  ARG A 367      77.926 120.266  92.196  1.00 20.00           C
ATOM    508  NH1 ARG A 367      76.816 120.873  92.637  1.00 20.00           N
ATOM    509  NH2 ARG A 367      78.015 118.931  92.234  1.00 20.00           N1+
ATOM    510  H   ARG A 367      80.266 123.411  86.736  1.00  0.00           H
ATOM    511  HA  ARG A 367      78.244 123.704  88.912  1.00  0.00           H
ATOM    512  HB2 ARG A 367      79.789 121.726  89.147  1.00  0.00           H
ATOM    513  HB3 ARG A 367      81.155 122.793  88.989  1.00  0.00           H
ATOM    514  HG2 ARG A 367      80.427 124.006  91.158  1.00  0.00           H
ATOM    515  HG3 ARG A 367      80.996 122.387  91.199  1.00  0.00           H
ATOM    516  HD2 ARG A 367      78.078 122.668  91.015  1.00  0.00           H
ATOM    517  HD3 ARG A 367      78.812 122.954  92.582  1.00  0.00           H
ATOM    518  HE  ARG A 367      79.787 120.516  91.395  1.00  0.00           H
ATOM    519 HH11 ARG A 367      76.024 120.351  92.994  1.00  0.00           H
ATOM    520 HH12 ARG A 367      76.752 121.879  92.590  1.00  0.00           H
ATOM    521 HH21 ARG A 367      77.246 118.380  92.587  1.00  0.00           H
ATOM    522 HH22 ARG A 367      78.848 118.467  91.903  1.00  0.00           H
ATOM    523  N   LYS A 368      80.984 125.468  88.244  1.00 30.00           N
ATOM    524  CA  LYS A 368      81.634 126.767  88.320  1.00 30.00           C
ATOM    525  C   LYS A 368      81.310 127.545  87.042  1.00 30.00           C
ATOM    526  O   LYS A 368      81.721 127.129  85.961  1.00 30.00           O
ATOM    527  CB  LYS A 368      83.152 126.564  88.497  1.00 20.00           C
ATOM    528  CG  LYS A 368      83.914 127.883  88.729  1.00 20.00           C
ATOM    529  CD  LYS A 368      85.434 127.706  88.850  1.00 20.00           C
ATOM    530  CE  LYS A 368      86.120 127.403  87.510  1.00 20.00           C
ATOM    531  NZ  LYS A 368      87.571 127.240  87.688  1.00 20.00           N1+
ATOM    532  H   LYS A 368      81.470 124.773  87.693  1.00  0.00           H
ATOM    533  HA  LYS A 368      81.269 127.320  89.186  1.00  0.00           H
ATOM    534  HB2 LYS A 368      83.325 125.908  89.350  1.00  0.00           H
ATOM    535  HB3 LYS A 368      83.556 126.039  87.631  1.00  0.00           H
ATOM    536  HG2 LYS A 368      83.706 128.599  87.933  1.00  0.00           H
ATOM    537  HG3 LYS A 368      83.540 128.340  89.646  1.00  0.00           H
ATOM    538  HD2 LYS A 368      85.649 126.901  89.551  1.00  0.00           H
ATOM    539  HD3 LYS A 368      85.863 128.609  89.287  1.00  0.00           H
ATOM    540  HE2 LYS A 368      85.723 126.492  87.063  1.00  0.00           H
ATOM    541  HE3 LYS A 368      85.940 128.213  86.801  1.00  0.00           H
ATOM    542  HZ1 LYS A 368      87.749 126.469  88.315  1.00  0.00           H
ATOM    543  HZ2 LYS A 368      88.002 127.053  86.793  1.00  0.00           H
ATOM    544  HZ3 LYS A 368      87.960 128.087  88.078  1.00  0.00           H
ATOM    545  N   PHE A 369      80.595 128.661  87.213  1.00 30.00           N
ATOM    546  CA  PHE A 369      80.197 129.591  86.167  1.00 30.00           C
ATOM    547  C   PHE A 369      81.061 130.847  86.322  1.00 30.00           C
ATOM    548  O   PHE A 369      80.827 131.634  87.242  1.00 30.00           O
ATOM    549  CB  PHE A 369      78.701 129.932  86.341  1.00 20.00           C
ATOM    550  CG  PHE A 369      77.740 128.768  86.165  1.00 20.00           C
ATOM    551  CD1 PHE A 369      77.186 128.489  84.899  1.00 20.00           C
ATOM    552  CD2 PHE A 369      77.504 127.868  87.227  1.00 20.00           C
ATOM    553  CE1 PHE A 369      76.361 127.387  84.733  1.00 20.00           C
ATOM    554  CE2 PHE A 369      76.702 126.753  87.029  1.00 20.00           C
ATOM    555  CZ  PHE A 369      76.128 126.516  85.788  1.00 20.00           C
ATOM    556  H   PHE A 369      80.302 128.919  88.149  1.00  0.00           H
ATOM    557  HA  PHE A 369      80.344 129.160  85.176  1.00  0.00           H
ATOM    558  HB2 PHE A 369      78.418 130.713  85.635  1.00  0.00           H
```

```
ATOM    559  HB3 PHE A 369      78.540 130.355  87.331  1.00  0.00           H
ATOM    560  HD1 PHE A 369      77.389 129.136  84.061  1.00  0.00           H
ATOM    561  HD2 PHE A 369      77.946 128.042  88.197  1.00  0.00           H
ATOM    562  HE1 PHE A 369      75.905 127.200  83.775  1.00  0.00           H
ATOM    563  HE2 PHE A 369      76.529 126.063  87.840  1.00  0.00           H
ATOM    564  HZ  PHE A 369      75.501 125.648  85.643  1.00  0.00           H
ATOM    565  N   THR A 370      82.055 131.007  85.434  1.00 30.00           N
ATOM    566  CA  THR A 370      82.930 132.181  85.401  1.00 30.00           C
ATOM    567  C   THR A 370      82.142 133.410  84.889  1.00 30.00           C
ATOM    568  O   THR A 370      81.784 133.473  83.712  1.00 30.00           O
ATOM    569  CB  THR A 370      84.194 131.932  84.528  1.00 20.00           C
ATOM    570  CG2 THR A 370      85.013 130.713  84.985  1.00 20.00           C
ATOM    571  OG1 THR A 370      83.913 131.772  83.150  1.00 20.00           O
ATOM    572  H   THR A 370      82.199 130.317  84.704  1.00  0.00           H
ATOM    573  HA  THR A 370      83.273 132.382  86.416  1.00  0.00           H
ATOM    574  HB  THR A 370      84.837 132.810  84.612  1.00  0.00           H
ATOM    575  HG1 THR A 370      83.354 132.503  82.870  1.00  0.00           H
ATOM    576 HG21 THR A 370      85.924 130.611  84.394  1.00  0.00           H
ATOM    577 HG22 THR A 370      85.309 130.806  86.028  1.00  0.00           H
ATOM    578 HG23 THR A 370      84.450 129.785  84.881  1.00  0.00           H
ATOM    579  N   GLU A 371      81.839 134.341  85.811  1.00 30.00           N
ATOM    580  CA  GLU A 371      81.030 135.537  85.547  1.00 30.00           C
ATOM    581  C   GLU A 371      81.806 136.563  84.708  1.00 30.00           C
ATOM    582  O   GLU A 371      81.270 137.075  83.726  1.00 30.00           O
ATOM    583  CB  GLU A 371      80.554 136.155  86.888  1.00 20.00           C
ATOM    584  CG  GLU A 371      79.023 136.297  87.016  1.00  0.00           C
ATOM    585  CD  GLU A 371      78.398 137.225  85.974  1.00  0.00           C
ATOM    586  OE1 GLU A 371      77.537 136.731  85.213  1.00  0.00           O
ATOM    587  OE2 GLU A 371      78.783 138.415  85.970  1.00  0.00           O1-
ATOM    588  H   GLU A 371      82.168 134.230  86.760  1.00  0.00           H
ATOM    589  HA  GLU A 371      80.161 135.217  84.969  1.00  0.00           H
ATOM    590  HB2 GLU A 371      80.925 135.562  87.723  1.00  0.00           H
ATOM    591  HB3 GLU A 371      81.003 137.137  87.049  1.00  0.00           H
ATOM    592  HG2 GLU A 371      78.553 135.314  86.960  1.00  0.00           H
ATOM    593  HG3 GLU A 371      78.776 136.704  87.995  1.00  0.00           H
ATOM    594  N   TRP A 372      83.063 136.812  85.107  1.00 30.00           N
ATOM    595  CA  TRP A 372      83.996 137.695  84.419  1.00 30.00           C
ATOM    596  C   TRP A 372      85.427 137.390  84.875  1.00 30.00           C
ATOM    597  O   TRP A 372      85.641 136.926  85.997  1.00 30.00           O
ATOM    598  CB  TRP A 372      83.622 139.184  84.626  1.00 20.00           C
ATOM    599  CG  TRP A 372      83.505 139.691  86.037  1.00 20.00           C
ATOM    600  CD1 TRP A 372      82.354 139.760  86.745  1.00 20.00           C
ATOM    601  CD2 TRP A 372      84.557 140.158  86.940  1.00 20.00           C
ATOM    602  CE2 TRP A 372      83.953 140.529  88.181  1.00 20.00           C
ATOM    603  CE3 TRP A 372      85.962 140.312  86.843  1.00 20.00           C
ATOM    604  NE1 TRP A 372      82.611 140.263  88.002  1.00 20.00           N
ATOM    605  CZ2 TRP A 372      84.697 141.032  89.262  1.00 20.00           C
ATOM    606  CZ3 TRP A 372      86.720 140.806  87.923  1.00 20.00           C
ATOM    607  CH2 TRP A 372      86.090 141.170  89.129  1.00 20.00           C
ATOM    608  H   TRP A 372      83.428 136.343  85.924  1.00  0.00           H
ATOM    609  HA  TRP A 372      83.946 137.472  83.351  1.00  0.00           H
ATOM    610  HB2 TRP A 372      82.676 139.388  84.125  1.00  0.00           H
ATOM    611  HB3 TRP A 372      84.353 139.812  84.115  1.00  0.00           H
ATOM    612  HD1 TRP A 372      81.386 139.471  86.362  1.00  0.00           H
ATOM    613  HE1 TRP A 372      81.886 140.407  88.690  1.00  0.00           H
ATOM    614  HE3 TRP A 372      86.463 140.047  85.924  1.00  0.00           H
ATOM    615  HZ2 TRP A 372      84.208 141.298  90.188  1.00  0.00           H
ATOM    616  HZ3 TRP A 372      87.790 140.907  87.828  1.00  0.00           H
ATOM    617  HH2 TRP A 372      86.671 141.551  89.955  1.00  0.00           H
ATOM    618  N   ALA A 373      86.377 137.699  83.982  1.00 30.00           N
ATOM    619  CA  ALA A 373      87.811 137.612  84.220  1.00 30.00           C
ATOM    620  C   ALA A 373      88.455 138.953  83.856  1.00 30.00           C
ATOM    621  O   ALA A 373      88.121 139.541  82.826  1.00 30.00           O
ATOM    622  CB  ALA A 373      88.395 136.473  83.374  1.00 30.00           C
ATOM    623  H   ALA A 373      86.111 138.088  83.089  1.00  0.00           H
ATOM    624  HA  ALA A 373      88.013 137.403  85.272  1.00  0.00           H
ATOM    625  HB1 ALA A 373      89.474 136.392  83.516  1.00  0.00           H
ATOM    626  HB2 ALA A 373      87.954 135.516  83.653  1.00  0.00           H
ATOM    627  HB3 ALA A 373      88.211 136.625  82.309  1.00  0.00           H
ATOM    628  N   TYR A 374      89.380 139.396  84.715  1.00 30.00           N
ATOM    629  CA  TYR A 374      90.152 140.622  84.569  1.00 30.00           C
```

```
ATOM    630  C   TYR A 374      91.463 140.390  85.332  1.00 30.00           C
ATOM    631  O   TYR A 374      91.601 140.856  86.464  1.00 30.00           O
ATOM    632  CB  TYR A 374      89.314 141.826  85.063  1.00 20.00           C
ATOM    633  CG  TYR A 374      89.882 143.189  84.713  1.00 20.00           C
ATOM    634  CD1 TYR A 374      89.683 143.717  83.420  1.00 20.00           C
ATOM    635  CD2 TYR A 374      90.596 143.938  85.671  1.00 20.00           C
ATOM    636  CE1 TYR A 374      90.194 144.985  83.086  1.00 20.00           C
ATOM    637  CE2 TYR A 374      91.109 145.207  85.336  1.00 20.00           C
ATOM    638  CZ  TYR A 374      90.907 145.731  84.044  1.00 20.00           C
ATOM    639  OH  TYR A 374      91.403 146.961  83.723  1.00 20.00           O
ATOM    640  H   TYR A 374      89.592 138.847  85.540  1.00  0.00           H
ATOM    641  HA  TYR A 374      90.400 140.771  83.515  1.00  0.00           H
ATOM    642  HB2 TYR A 374      88.319 141.784  84.619  1.00  0.00           H
ATOM    643  HB3 TYR A 374      89.148 141.764  86.139  1.00  0.00           H
ATOM    644  HD1 TYR A 374      89.135 143.149  82.681  1.00  0.00           H
ATOM    645  HD2 TYR A 374      90.753 143.542  86.664  1.00  0.00           H
ATOM    646  HE1 TYR A 374      90.036 145.381  82.093  1.00  0.00           H
ATOM    647  HE2 TYR A 374      91.656 145.776  86.073  1.00  0.00           H
ATOM    648  HH  TYR A 374      91.214 147.220  82.819  1.00  0.00           H
ATOM    649  N   GLY A 375      92.348 139.587  84.709  1.00 30.00           N
ATOM    650  CA  GLY A 375      93.563 138.964  85.253  1.00 30.00           C
ATOM    651  C   GLY A 375      94.402 139.885  86.165  1.00 30.00           C
ATOM    652  O   GLY A 375      94.651 141.027  85.776  1.00 30.00           O
ATOM    653  H   GLY A 375      92.102 139.280  83.779  1.00  0.00           H
ATOM    654  HA2 GLY A 375      94.192 138.662  84.415  1.00  0.00           H
ATOM    655  HA3 GLY A 375      93.256 138.044  85.745  1.00  0.00           H
ATOM    656  N   PRO A 376      94.875 139.417  87.345  1.00 30.00           N
ATOM    657  CA  PRO A 376      94.733 138.046  87.883  1.00 30.00           C
ATOM    658  C   PRO A 376      93.353 137.683  88.475  1.00 30.00           C
ATOM    659  O   PRO A 376      93.178 136.529  88.865  1.00 30.00           O
ATOM    660  CB  PRO A 376      95.813 137.997  88.978  1.00 20.00           C
ATOM    661  CG  PRO A 376      95.887 139.424  89.496  1.00 20.00           C
ATOM    662  CD  PRO A 376      95.694 140.249  88.231  1.00 20.00           C
ATOM    663  HA  PRO A 376      94.974 137.306  87.119  1.00  0.00           H
ATOM    664  HB2 PRO A 376      95.606 137.284  89.776  1.00  0.00           H
ATOM    665  HB3 PRO A 376      96.772 137.716  88.543  1.00  0.00           H
ATOM    666  HG2 PRO A 376      95.058 139.605  90.183  1.00  0.00           H
ATOM    667  HG3 PRO A 376      96.815 139.648  90.023  1.00  0.00           H
ATOM    668  HD2 PRO A 376      95.231 141.213  88.448  1.00  0.00           H
ATOM    669  HD3 PRO A 376      96.655 140.432  87.747  1.00  0.00           H
ATOM    670  N   VAL A 377      92.415 138.643  88.552  1.00 30.00           N
ATOM    671  CA  VAL A 377      91.112 138.471  89.200  1.00 30.00           C
ATOM    672  C   VAL A 377      90.148 137.647  88.320  1.00 30.00           C
ATOM    673  O   VAL A 377      89.985 137.966  87.143  1.00 30.00           O
ATOM    674  CB  VAL A 377      90.441 139.837  89.522  1.00 20.00           C
ATOM    675  CG1 VAL A 377      89.139 139.684  90.332  1.00  0.00           C
ATOM    676  CG2 VAL A 377      91.396 140.777  90.283  1.00  0.00           C
ATOM    677  H   VAL A 377      92.607 139.567  88.190  1.00  0.00           H
ATOM    678  HA  VAL A 377      91.276 137.957  90.145  1.00  0.00           H
ATOM    679  HB  VAL A 377      90.178 140.335  88.588  1.00  0.00           H
ATOM    680 HG11 VAL A 377      88.728 140.656  90.602  1.00  0.00           H
ATOM    681 HG12 VAL A 377      88.363 139.149  89.786  1.00  0.00           H
ATOM    682 HG13 VAL A 377      89.330 139.145  91.258  1.00  0.00           H
ATOM    683 HG21 VAL A 377      90.901 141.712  90.548  1.00  0.00           H
ATOM    684 HG22 VAL A 377      91.752 140.316  91.205  1.00  0.00           H
ATOM    685 HG23 VAL A 377      92.267 141.040  89.683  1.00  0.00           H
ATOM    686  N   HIS A 378      89.533 136.608  88.908  1.00 30.00           N
ATOM    687  CA  HIS A 378      88.593 135.707  88.238  1.00 30.00           C
ATOM    688  C   HIS A 378      87.401 135.437  89.169  1.00 30.00           C
ATOM    689  O   HIS A 378      87.450 134.492  89.958  1.00 30.00           O
ATOM    690  CB  HIS A 378      89.303 134.396  87.822  1.00 20.00           C
ATOM    691  CG  HIS A 378      90.325 134.517  86.717  1.00  0.00           C
ATOM    692  CD2 HIS A 378      90.253 134.226  85.372  1.00  0.00           C
ATOM    693  ND1 HIS A 378      91.626 134.942  86.930  1.00  0.00           N
ATOM    694  CE1 HIS A 378      92.252 134.917  85.752  1.00  0.00           C
ATOM    695  NE2 HIS A 378      91.480 134.494  84.759  1.00  0.00           N
ATOM    696  H   HIS A 378      89.742 136.393  89.874  1.00  0.00           H
ATOM    697  HA  HIS A 378      88.191 136.174  87.337  1.00  0.00           H
ATOM    698  HB2 HIS A 378      89.800 133.952  88.683  1.00  0.00           H
ATOM    699  HB3 HIS A 378      88.562 133.666  87.492  1.00  0.00           H
ATOM    700  HD1 HIS A 378      92.032 135.240  87.810  1.00  0.00           H
```

```
ATOM    701  HD2 HIS A 378      89.417 133.851  84.801  1.00  0.00           H
ATOM    702  HE1 HIS A 378      93.284 135.209  85.622  1.00  0.00           H
ATOM    703  N   SER A 379      86.354 136.276  89.059  1.00 30.00           N
ATOM    704  CA  SER A 379      85.100 136.145  89.806  1.00 30.00           C
ATOM    705  C   SER A 379      84.254 134.990  89.243  1.00 30.00           C
ATOM    706  O   SER A 379      83.843 135.050  88.083  1.00 30.00           O
ATOM    707  CB  SER A 379      84.349 137.489  89.772  1.00 20.00           C
ATOM    708  OG  SER A 379      83.240 137.483  90.651  1.00 20.00           O
ATOM    709  H   SER A 379      86.382 137.016  88.372  1.00  0.00           H
ATOM    710  HA  SER A 379      85.343 135.929  90.846  1.00  0.00           H
ATOM    711  HB2 SER A 379      85.014 138.296  90.080  1.00  0.00           H
ATOM    712  HB3 SER A 379      84.008 137.721  88.763  1.00  0.00           H
ATOM    713  HG  SER A 379      83.588 137.544  91.550  1.00  0.00           H
ATOM    714  N   SER A 380      84.052 133.952  90.069  1.00 30.00           N
ATOM    715  CA  SER A 380      83.451 132.683  89.667  1.00 30.00           C
ATOM    716  C   SER A 380      82.368 132.266  90.670  1.00 30.00           C
ATOM    717  O   SER A 380      82.685 132.036  91.837  1.00 30.00           O
ATOM    718  CB  SER A 380      84.561 131.616  89.594  1.00 20.00           C
ATOM    719  OG  SER A 380      85.403 131.845  88.484  1.00  0.00           O
ATOM    720  H   SER A 380      84.433 133.981  91.007  1.00  0.00           H
ATOM    721  HA  SER A 380      82.979 132.767  88.687  1.00  0.00           H
ATOM    722  HB2 SER A 380      85.160 131.599  90.507  1.00  0.00           H
ATOM    723  HB3 SER A 380      84.125 130.624  89.480  1.00  0.00           H
ATOM    724  HG  SER A 380      85.901 132.650  88.648  1.00  0.00           H
ATOM    725  N   LEU A 381      81.122 132.150  90.181  1.00 30.00           N
ATOM    726  CA  LEU A 381      79.963 131.661  90.927  1.00 30.00           C
ATOM    727  C   LEU A 381      79.924 130.131  90.841  1.00 30.00           C
ATOM    728  O   LEU A 381      79.789 129.593  89.745  1.00 30.00           O
ATOM    729  CB  LEU A 381      78.654 132.237  90.337  1.00 20.00           C
ATOM    730  CG  LEU A 381      78.330 133.703  90.681  1.00  0.00           C
ATOM    731  CD1 LEU A 381      79.358 134.679  90.089  1.00  0.00           C
ATOM    732  CD2 LEU A 381      76.897 134.081  90.247  1.00  0.00           C
ATOM    733  H   LEU A 381      80.961 132.322  89.197  1.00  0.00           H
ATOM    734  HA  LEU A 381      80.029 131.955  91.975  1.00  0.00           H
ATOM    735  HB2 LEU A 381      78.643 132.102  89.254  1.00  0.00           H
ATOM    736  HB3 LEU A 381      77.832 131.638  90.731  1.00  0.00           H
ATOM    737  HG  LEU A 381      78.350 133.783  91.765  1.00  0.00           H
ATOM    738 HD11 LEU A 381      78.958 135.687  90.011  1.00  0.00           H
ATOM    739 HD12 LEU A 381      80.259 134.739  90.696  1.00  0.00           H
ATOM    740 HD13 LEU A 381      79.658 134.353  89.095  1.00  0.00           H
ATOM    741 HD21 LEU A 381      76.332 134.491  91.085  1.00  0.00           H
ATOM    742 HD22 LEU A 381      76.889 134.828  89.453  1.00  0.00           H
ATOM    743 HD23 LEU A 381      76.338 133.222  89.875  1.00  0.00           H
ATOM    744  N   TYR A 382      79.995 129.464  92.000  1.00 30.00           N
ATOM    745  CA  TYR A 382      79.839 128.013  92.127  1.00 30.00           C
ATOM    746  C   TYR A 382      78.351 127.607  92.208  1.00 30.00           C
ATOM    747  O   TYR A 382      77.477 128.453  92.015  1.00 30.00           O
ATOM    748  CB  TYR A 382      80.675 127.557  93.344  1.00 20.00           C
ATOM    749  CG  TYR A 382      82.173 127.666  93.109  1.00 20.00           C
ATOM    750  CD1 TYR A 382      82.828 126.671  92.352  1.00 20.00           C
ATOM    751  CD2 TYR A 382      82.914 128.757  93.612  1.00 20.00           C
ATOM    752  CE1 TYR A 382      84.210 126.764  92.103  1.00 20.00           C
ATOM    753  CE2 TYR A 382      84.298 128.846  93.359  1.00 20.00           C
ATOM    754  CZ  TYR A 382      84.948 127.852  92.604  1.00 20.00           C
ATOM    755  OH  TYR A 382      86.288 127.941  92.355  1.00 20.00           O
ATOM    756  H   TYR A 382      80.094 129.975  92.866  1.00  0.00           H
ATOM    757  HA  TYR A 382      80.250 127.529  91.242  1.00  0.00           H
ATOM    758  HB2 TYR A 382      80.394 128.122  94.235  1.00  0.00           H
ATOM    759  HB3 TYR A 382      80.473 126.511  93.581  1.00  0.00           H
ATOM    760  HD1 TYR A 382      82.269 125.837  91.955  1.00  0.00           H
ATOM    761  HD2 TYR A 382      82.425 129.530  94.187  1.00  0.00           H
ATOM    762  HE1 TYR A 382      84.699 125.995  91.524  1.00  0.00           H
ATOM    763  HE2 TYR A 382      84.868 129.675  93.744  1.00  0.00           H
ATOM    764  HH  TYR A 382      86.718 128.669  92.813  1.00  0.00           H
ATOM    765  N   ASP A 383      78.085 126.321  92.489  1.00 30.00           N
ATOM    766  CA  ASP A 383      76.785 125.837  92.967  1.00 30.00           C
ATOM    767  C   ASP A 383      76.992 125.175  94.332  1.00 30.00           C
ATOM    768  O   ASP A 383      77.976 124.461  94.529  1.00 30.00           O
ATOM    769  CB  ASP A 383      76.048 124.832  92.047  1.00 20.00           C
ATOM    770  CG  ASP A 383      75.870 125.245  90.588  1.00  0.00           C
ATOM    771  OD1 ASP A 383      75.734 126.459  90.332  1.00  0.00           O
```

```
ATOM    772  OD2 ASP A 383      75.734 124.319  89.760  1.00  0.00           O1-
ATOM    773  H   ASP A 383      78.850 125.669  92.583  1.00  0.00           H
ATOM    774  HA  ASP A 383      76.115 126.685  93.109  1.00  0.00           H
ATOM    775  HB2 ASP A 383      76.576 123.883  92.066  1.00  0.00           H
ATOM    776  HB3 ASP A 383      75.052 124.639  92.448  1.00  0.00           H
ATOM    777  N   LEU A 384      76.015 125.389  95.220  1.00 30.00           N
ATOM    778  CA  LEU A 384      75.945 124.801  96.551  1.00 30.00           C
ATOM    779  C   LEU A 384      74.709 123.882  96.603  1.00 30.00           C
ATOM    780  O   LEU A 384      73.803 124.127  97.398  1.00 30.00           O
ATOM    781  CB  LEU A 384      75.896 125.926  97.615  1.00 20.00           C
ATOM    782  CG  LEU A 384      77.024 126.982  97.566  1.00 20.00           C
ATOM    783  CD1 LEU A 384      76.735 128.102  98.587  1.00 20.00           C
ATOM    784  CD2 LEU A 384      78.423 126.366  97.759  1.00 20.00           C
ATOM    785  H   LEU A 384      75.266 126.026  94.983  1.00  0.00           H
ATOM    786  HA  LEU A 384      76.821 124.184  96.746  1.00  0.00           H
ATOM    787  HB2 LEU A 384      75.894 125.466  98.601  1.00  0.00           H
ATOM    788  HB3 LEU A 384      74.948 126.457  97.533  1.00  0.00           H
ATOM    789  HG  LEU A 384      77.021 127.457  96.585  1.00  0.00           H
ATOM    790 HD11 LEU A 384      77.614 128.388  99.163  1.00  0.00           H
ATOM    791 HD12 LEU A 384      76.371 128.996  98.080  1.00  0.00           H
ATOM    792 HD13 LEU A 384      75.968 127.813  99.307  1.00  0.00           H
ATOM    793 HD21 LEU A 384      79.117 127.048  98.250  1.00  0.00           H
ATOM    794 HD22 LEU A 384      78.377 125.463  98.363  1.00  0.00           H
ATOM    795 HD23 LEU A 384      78.866 126.103  96.798  1.00  0.00           H
ATOM    796  N   SER A 385      74.666 122.863  95.723  1.00 30.00           N
ATOM    797  CA  SER A 385      73.541 121.923  95.619  1.00 30.00           C
ATOM    798  C   SER A 385      73.624 120.770  96.637  1.00 30.00           C
ATOM    799  O   SER A 385      72.581 120.306  97.097  1.00 30.00           O
ATOM    800  CB  SER A 385      73.408 121.417  94.161  1.00 20.00           C
ATOM    801  OG  SER A 385      74.254 120.322  93.856  1.00  0.00           O
ATOM    802  H   SER A 385      75.426 122.733  95.072  1.00  0.00           H
ATOM    803  HA  SER A 385      72.626 122.479  95.836  1.00  0.00           H
ATOM    804  HB2 SER A 385      72.382 121.086  93.991  1.00  0.00           H
ATOM    805  HB3 SER A 385      73.588 122.225  93.450  1.00  0.00           H
ATOM    806  HG  SER A 385      73.894 119.538  94.280  1.00  0.00           H
ATOM    807  N   CYS A 386      74.854 120.325  96.942  1.00 30.00           N
ATOM    808  CA  CYS A 386      75.173 119.214  97.844  1.00 30.00           C
ATOM    809  C   CYS A 386      76.368 119.574  98.750  1.00 30.00           C
ATOM    810  O   CYS A 386      76.884 118.689  99.430  1.00 30.00           O
ATOM    811  CB  CYS A 386      75.463 117.908  97.063  1.00 20.00           C
ATOM    812  SG  CYS A 386      73.960 117.225  96.309  1.00  0.00           S
ATOM    813  H   CYS A 386      75.647 120.761  96.495  1.00  0.00           H
ATOM    814  HA  CYS A 386      74.338 119.029  98.520  1.00  0.00           H
ATOM    815  HB2 CYS A 386      76.204 118.084  96.283  1.00  0.00           H
ATOM    816  HB3 CYS A 386      75.866 117.131  97.714  1.00  0.00           H
ATOM    817  HG  CYS A 386      73.341 116.944  97.459  1.00  0.00           H
ATOM    818  N   ILE A 387      76.821 120.841  98.729  1.00 30.00           N
ATOM    819  CA  ILE A 387      78.048 121.277  99.393  1.00 30.00           C
ATOM    820  C   ILE A 387      77.811 121.601 100.884  1.00 30.00           C
ATOM    821  O   ILE A 387      78.523 121.048 101.722  1.00 30.00           O
ATOM    822  CB  ILE A 387      78.736 122.469  98.652  1.00 20.00           C
ATOM    823  CG1 ILE A 387      79.369 122.040  97.301  1.00  0.00           C
ATOM    824  CG2 ILE A 387      79.828 123.188  99.478  1.00  0.00           C
ATOM    825  CD1 ILE A 387      78.406 121.521  96.221  1.00  0.00           C
ATOM    826  H   ILE A 387      76.339 121.536  98.178  1.00  0.00           H
ATOM    827  HA  ILE A 387      78.747 120.443  99.353  1.00  0.00           H
ATOM    828  HB  ILE A 387      77.965 123.206  98.435  1.00  0.00           H
ATOM    829 HG12 ILE A 387      79.903 122.890  96.872  1.00  0.00           H
ATOM    830 HG13 ILE A 387      80.129 121.282  97.488  1.00  0.00           H
ATOM    831 HG21 ILE A 387      80.324 123.963  98.893  1.00  0.00           H
ATOM    832 HG22 ILE A 387      79.419 123.685 100.357  1.00  0.00           H
ATOM    833 HG23 ILE A 387      80.596 122.491  99.815  1.00  0.00           H
ATOM    834 HD11 ILE A 387      78.812 121.696  95.224  1.00  0.00           H
ATOM    835 HD12 ILE A 387      78.242 120.449  96.321  1.00  0.00           H
ATOM    836 HD13 ILE A 387      77.437 122.015  96.267  1.00  0.00           H
ATOM    837  N   ASP A 388      76.810 122.451 101.180  1.00 30.00           N
ATOM    838  CA  ASP A 388      76.431 122.850 102.545  1.00 30.00           C
ATOM    839  C   ASP A 388      74.982 122.456 102.897  1.00 30.00           C
ATOM    840  O   ASP A 388      74.596 122.637 104.052  1.00 30.00           O
ATOM    841  CB  ASP A 388      76.661 124.363 102.835  1.00 20.00           C
ATOM    842  CG  ASP A 388      76.051 125.376 101.852  1.00  0.00           C
```

```
ATOM    843  OD1 ASP A 388      75.270 124.961 100.967  1.00  0.00           O
ATOM    844  OD2 ASP A 388      76.349 126.576 102.044  1.00  0.00           O1-
ATOM    845  H   ASP A 388      76.281 122.891 100.439  1.00  0.00           H
ATOM    846  HA  ASP A 388      77.047 122.303 103.261  1.00  0.00           H
ATOM    847  HB2 ASP A 388      76.295 124.603 103.834  1.00  0.00           H
ATOM    848  HB3 ASP A 388      77.737 124.526 102.847  1.00  0.00           H
ATOM    849  N   THR A 389      74.203 121.953 101.920  1.00 30.00           N
ATOM    850  CA  THR A 389      72.779 121.633 102.062  1.00 30.00           C
ATOM    851  C   THR A 389      72.561 120.432 103.009  1.00 30.00           C
ATOM    852  O   THR A 389      72.804 119.278 102.655  1.00 30.00           O
ATOM    853  CB  THR A 389      72.105 121.394 100.683  1.00 20.00           C
ATOM    854  CG2 THR A 389      72.251 122.593  99.737  1.00  0.00           C
ATOM    855  OG1 THR A 389      72.574 120.238 100.017  1.00  0.00           O
ATOM    856  H   THR A 389      74.590 121.819 100.998  1.00  0.00           H
ATOM    857  HA  THR A 389      72.297 122.509 102.502  1.00  0.00           H
ATOM    858  HB  THR A 389      71.039 121.238 100.852  1.00  0.00           H
ATOM    859  HG1 THR A 389      72.284 120.270  99.100  1.00  0.00           H
ATOM    860 HG21 THR A 389      71.722 122.429  98.798  1.00  0.00           H
ATOM    861 HG22 THR A 389      71.848 123.501 100.186  1.00  0.00           H
ATOM    862 HG23 THR A 389      73.299 122.778  99.502  1.00  0.00           H
ATOM    863  N   CYS A 390      72.154 120.772 104.241  1.00 30.00           N
ATOM    864  CA  CYS A 390      72.111 119.891 105.407  1.00 30.00           C
ATOM    865  C   CYS A 390      70.919 118.912 105.457  1.00 30.00           C
ATOM    866  O   CYS A 390      70.794 118.195 106.451  1.00 30.00           O
ATOM    867  CB  CYS A 390      72.250 120.723 106.698  1.00 20.00           C
ATOM    868  SG  CYS A 390      70.790 121.750 107.040  1.00  0.00           S
ATOM    869  H   CYS A 390      71.988 121.748 104.442  1.00  0.00           H
ATOM    870  HA  CYS A 390      72.998 119.256 105.352  1.00  0.00           H
ATOM    871  HB2 CYS A 390      72.383 120.055 107.545  1.00  0.00           H
ATOM    872  HB3 CYS A 390      73.138 121.355 106.655  1.00  0.00           H
ATOM    873  HG  CYS A 390      69.963 120.732 107.299  1.00  0.00           H
ATOM    874  N   GLU A 391      70.082 118.876 104.404  1.00 30.00           N
ATOM    875  CA  GLU A 391      69.005 117.899 104.217  1.00 30.00           C
ATOM    876  C   GLU A 391      69.550 116.463 104.103  1.00 30.00           C
ATOM    877  O   GLU A 391      69.088 115.575 104.819  1.00 30.00           O
ATOM    878  CB  GLU A 391      68.186 118.293 102.967  1.00 20.00           C
ATOM    879  CG  GLU A 391      67.025 117.336 102.621  1.00  0.00           C
ATOM    880  CD  GLU A 391      66.380 117.700 101.288  1.00  0.00           C
ATOM    881  OE1 GLU A 391      66.938 117.260 100.258  1.00  0.00           O
ATOM    882  OE2 GLU A 391      65.350 118.405 101.317  1.00  0.00           O1-
ATOM    883  H   GLU A 391      70.250 119.502 103.630  1.00  0.00           H
ATOM    884  HA  GLU A 391      68.349 117.946 105.089  1.00  0.00           H
ATOM    885  HB2 GLU A 391      67.779 119.294 103.115  1.00  0.00           H
ATOM    886  HB3 GLU A 391      68.857 118.374 102.109  1.00  0.00           H
ATOM    887  HG2 GLU A 391      67.362 116.302 102.543  1.00  0.00           H
ATOM    888  HG3 GLU A 391      66.271 117.359 103.409  1.00  0.00           H
ATOM    889  N   LYS A 392      70.528 116.295 103.201  1.00 30.00           N
ATOM    890  CA  LYS A 392      71.247 115.051 102.945  1.00 30.00           C
ATOM    891  C   LYS A 392      72.675 115.160 103.512  1.00 30.00           C
ATOM    892  O   LYS A 392      73.026 116.175 104.117  1.00 30.00           O
ATOM    893  CB  LYS A 392      71.258 114.796 101.418  1.00 20.00           C
ATOM    894  CG  LYS A 392      69.853 114.674 100.795  1.00  0.00           C
ATOM    895  CD  LYS A 392      69.902 114.214  99.329  1.00  0.00           C
ATOM    896  CE  LYS A 392      68.520 114.107  98.664  1.00  0.00           C
ATOM    897  NZ  LYS A 392      67.929 115.428  98.385  1.00  0.00           N1+
ATOM    898  H   LYS A 392      70.861 117.100 102.692  1.00  0.00           H
ATOM    899  HA  LYS A 392      70.757 114.212 103.441  1.00  0.00           H
ATOM    900  HB2 LYS A 392      71.808 115.591 100.911  1.00  0.00           H
ATOM    901  HB3 LYS A 392      71.801 113.873 101.214  1.00  0.00           H
ATOM    902  HG2 LYS A 392      69.256 113.972 101.378  1.00  0.00           H
ATOM    903  HG3 LYS A 392      69.341 115.635 100.857  1.00  0.00           H
ATOM    904  HD2 LYS A 392      70.535 114.891  98.754  1.00  0.00           H
ATOM    905  HD3 LYS A 392      70.388 113.239  99.280  1.00  0.00           H
ATOM    906  HE2 LYS A 392      68.616 113.582  97.713  1.00  0.00           H
ATOM    907  HE3 LYS A 392      67.837 113.523  99.282  1.00  0.00           H
ATOM    908  HZ1 LYS A 392      67.757 115.939  99.247  1.00  0.00           H
ATOM    909  HZ2 LYS A 392      68.552 115.968  97.804  1.00  0.00           H
ATOM    910  HZ3 LYS A 392      67.047 115.310  97.908  1.00  0.00           H
ATOM    911  N   ASN A 393      73.477 114.102 103.302  1.00 30.00           N
ATOM    912  CA  ASN A 393      74.903 114.043 103.642  1.00 30.00           C
ATOM    913  C   ASN A 393      75.706 114.992 102.734  1.00 30.00           C
```

```
ATOM    914  O   ASN A 393      76.136 114.584 101.654  1.00 30.00           O
ATOM    915  CB  ASN A 393      75.404 112.583 103.516  1.00 20.00           C
ATOM    916  CG  ASN A 393      74.769 111.630 104.532  1.00  0.00           C
ATOM    917  ND2 ASN A 393      75.464 111.365 105.640  1.00  0.00           N
ATOM    918  OD1 ASN A 393      73.669 111.128 104.312  1.00  0.00           O
ATOM    919  H   ASN A 393      73.108 113.291 102.828  1.00  0.00           H
ATOM    920  HA  ASN A 393      75.005 114.359 104.683  1.00  0.00           H
ATOM    921  HB2 ASN A 393      75.213 112.197 102.513  1.00  0.00           H
ATOM    922  HB3 ASN A 393      76.485 112.551 103.650  1.00  0.00           H
ATOM    923 HD21 ASN A 393      75.082 110.739 106.334  1.00  0.00           H
ATOM    924 HD22 ASN A 393      76.366 111.789 105.797  1.00  0.00           H
ATOM    925  N   SER A 394      75.866 116.249 103.186  1.00 30.00           N
ATOM    926  CA  SER A 394      76.596 117.300 102.475  1.00 30.00           C
ATOM    927  C   SER A 394      78.109 117.015 102.442  1.00 30.00           C
ATOM    928  O   SER A 394      78.614 116.330 103.332  1.00 30.00           O
ATOM    929  CB  SER A 394      76.242 118.675 103.088  1.00 20.00           C
ATOM    930  OG  SER A 394      76.983 119.000 104.248  1.00  0.00           O
ATOM    931  H   SER A 394      75.454 116.515 104.070  1.00  0.00           H
ATOM    932  HA  SER A 394      76.220 117.288 101.452  1.00  0.00           H
ATOM    933  HB2 SER A 394      76.413 119.459 102.352  1.00  0.00           H
ATOM    934  HB3 SER A 394      75.184 118.721 103.338  1.00  0.00           H
ATOM    935  HG  SER A 394      76.403 118.917 105.013  1.00  0.00           H
ATOM    936  N   VAL A 395      78.808 117.542 101.421  1.00 30.00           N
ATOM    937  CA  VAL A 395      80.235 117.281 101.188  1.00 30.00           C
ATOM    938  C   VAL A 395      81.148 117.732 102.353  1.00 30.00           C
ATOM    939  O   VAL A 395      82.155 117.070 102.604  1.00 30.00           O
ATOM    940  CB  VAL A 395      80.725 117.898  99.838  1.00 20.00           C
ATOM    941  CG1 VAL A 395      81.266 119.342  99.913  1.00  0.00           C
ATOM    942  CG2 VAL A 395      81.779 117.008  99.162  1.00  0.00           C
ATOM    943  H   VAL A 395      78.330 118.100 100.725  1.00  0.00           H
ATOM    944  HA  VAL A 395      80.332 116.195 101.121  1.00  0.00           H
ATOM    945  HB  VAL A 395      79.864 117.928  99.168  1.00  0.00           H
ATOM    946 HG11 VAL A 395      81.392 119.764  98.916  1.00  0.00           H
ATOM    947 HG12 VAL A 395      80.601 119.998 100.470  1.00  0.00           H
ATOM    948 HG13 VAL A 395      82.239 119.378 100.402  1.00  0.00           H
ATOM    949 HG21 VAL A 395      82.091 117.427  98.205  1.00  0.00           H
ATOM    950 HG22 VAL A 395      82.671 116.910  99.782  1.00  0.00           H
ATOM    951 HG23 VAL A 395      81.390 116.008  98.969  1.00  0.00           H
ATOM    952  N   LEU A 396      80.744 118.806 103.060  1.00 30.00           N
ATOM    953  CA  LEU A 396      81.394 119.332 104.261  1.00 30.00           C
ATOM    954  C   LEU A 396      81.207 118.410 105.478  1.00 30.00           C
ATOM    955  O   LEU A 396      82.190 118.141 106.167  1.00 30.00           O
ATOM    956  CB  LEU A 396      80.857 120.750 104.564  1.00 20.00           C
ATOM    957  CG  LEU A 396      81.341 121.836 103.577  1.00 20.00           C
ATOM    958  CD1 LEU A 396      80.522 123.136 103.736  1.00 20.00           C
ATOM    959  CD2 LEU A 396      82.864 122.079 103.687  1.00 20.00           C
ATOM    960  H   LEU A 396      79.898 119.282 102.778  1.00  0.00           H
ATOM    961  HA  LEU A 396      82.464 119.396 104.063  1.00  0.00           H
ATOM    962  HB2 LEU A 396      79.765 120.714 104.565  1.00  0.00           H
ATOM    963  HB3 LEU A 396      81.143 121.052 105.574  1.00  0.00           H
ATOM    964  HG  LEU A 396      81.147 121.476 102.566  1.00  0.00           H
ATOM    965 HD11 LEU A 396      81.135 124.004 103.977  1.00  0.00           H
ATOM    966 HD12 LEU A 396      79.993 123.364 102.811  1.00  0.00           H
ATOM    967 HD13 LEU A 396      79.768 123.054 104.520  1.00  0.00           H
ATOM    968 HD21 LEU A 396      83.124 123.135 103.747  1.00  0.00           H
ATOM    969 HD22 LEU A 396      83.295 121.598 104.565  1.00  0.00           H
ATOM    970 HD23 LEU A 396      83.380 121.677 102.815  1.00  0.00           H
ATOM    971  N   GLU A 397      79.971 117.931 105.709  1.00 30.00           N
ATOM    972  CA  GLU A 397      79.623 116.999 106.790  1.00 30.00           C
ATOM    973  C   GLU A 397      80.318 115.636 106.662  1.00 30.00           C
ATOM    974  O   GLU A 397      80.751 115.091 107.675  1.00 30.00           O
ATOM    975  CB  GLU A 397      78.094 116.836 106.874  1.00 20.00           C
ATOM    976  CG  GLU A 397      77.392 118.097 107.407  1.00  0.00           C
ATOM    977  CD  GLU A 397      75.870 117.989 107.352  1.00  0.00           C
ATOM    978  OE1 GLU A 397      75.336 117.000 107.898  1.00  0.00           O
ATOM    979  OE2 GLU A 397      75.258 118.924 106.790  1.00  0.00           O1-
ATOM    980  H   GLU A 397      79.214 118.193 105.092  1.00  0.00           H
ATOM    981  HA  GLU A 397      79.960 117.419 107.737  1.00  0.00           H
ATOM    982  HB2 GLU A 397      77.695 116.584 105.890  1.00  0.00           H
ATOM    983  HB3 GLU A 397      77.847 115.992 107.522  1.00  0.00           H
ATOM    984  HG2 GLU A 397      77.689 118.281 108.440  1.00  0.00           H
```

```
ATOM    985  HG3 GLU A 397      77.703 118.974 106.839  1.00  0.00           H
ATOM    986  N   VAL A 398      80.439 115.131 105.425  1.00 30.00           N
ATOM    987  CA  VAL A 398      81.070 113.852 105.116  1.00 30.00           C
ATOM    988  C   VAL A 398      82.592 113.848 105.374  1.00 30.00           C
ATOM    989  O   VAL A 398      83.065 112.926 106.037  1.00 30.00           O
ATOM    990  CB  VAL A 398      80.763 113.415 103.655  1.00 20.00           C
ATOM    991  CG1 VAL A 398      81.608 112.235 103.147  1.00 20.00           C
ATOM    992  CG2 VAL A 398      79.275 113.055 103.490  1.00 20.00           C
ATOM    993  H   VAL A 398      80.049 115.640 104.642  1.00  0.00           H
ATOM    994  HA  VAL A 398      80.630 113.107 105.784  1.00  0.00           H
ATOM    995  HB  VAL A 398      80.964 114.265 102.999  1.00  0.00           H
ATOM    996 HG11 VAL A 398      81.271 111.926 102.161  1.00  0.00           H
ATOM    997 HG12 VAL A 398      82.663 112.489 103.055  1.00  0.00           H
ATOM    998 HG13 VAL A 398      81.527 111.375 103.813  1.00  0.00           H
ATOM    999 HG21 VAL A 398      79.030 112.864 102.445  1.00  0.00           H
ATOM   1000 HG22 VAL A 398      79.022 112.160 104.060  1.00  0.00           H
ATOM   1001 HG23 VAL A 398      78.619 113.851 103.836  1.00  0.00           H
ATOM   1002  N   ILE A 399      83.322 114.863 104.873  1.00 30.00           N
ATOM   1003  CA  ILE A 399      84.780 114.962 105.030  1.00 30.00           C
ATOM   1004  C   ILE A 399      85.216 115.316 106.474  1.00 30.00           C
ATOM   1005  O   ILE A 399      86.295 114.893 106.891  1.00 30.00           O
ATOM   1006  CB  ILE A 399      85.406 115.975 104.017  1.00 20.00           C
ATOM   1007  CG1 ILE A 399      86.949 115.895 103.900  1.00 20.00           C
ATOM   1008  CG2 ILE A 399      85.004 117.440 104.274  1.00 20.00           C
ATOM   1009  CD1 ILE A 399      87.485 114.516 103.492  1.00 20.00           C
ATOM   1010  H   ILE A 399      82.873 115.596 104.340  1.00  0.00           H
ATOM   1011  HA  ILE A 399      85.182 113.972 104.810  1.00  0.00           H
ATOM   1012  HB  ILE A 399      85.008 115.715 103.034  1.00  0.00           H
ATOM   1013 HG12 ILE A 399      87.419 116.206 104.833  1.00  0.00           H
ATOM   1014 HG13 ILE A 399      87.285 116.615 103.154  1.00  0.00           H
ATOM   1015 HG21 ILE A 399      85.340 118.091 103.466  1.00  0.00           H
ATOM   1016 HG22 ILE A 399      83.927 117.545 104.346  1.00  0.00           H
ATOM   1017 HG23 ILE A 399      85.430 117.821 105.199  1.00  0.00           H
ATOM   1018 HD11 ILE A 399      88.504 114.593 103.113  1.00  0.00           H
ATOM   1019 HD12 ILE A 399      87.500 113.825 104.336  1.00  0.00           H
ATOM   1020 HD13 ILE A 399      86.878 114.073 102.703  1.00  0.00           H
ATOM   1021  N   ALA A 400      84.363 116.047 107.214  1.00 30.00           N
ATOM   1022  CA  ALA A 400      84.590 116.429 108.608  1.00 30.00           C
ATOM   1023  C   ALA A 400      84.356 115.273 109.593  1.00 30.00           C
ATOM   1024  O   ALA A 400      85.192 115.070 110.474  1.00 30.00           O
ATOM   1025  CB  ALA A 400      83.695 117.624 108.955  1.00 20.00           C
ATOM   1026  H   ALA A 400      83.496 116.366 106.802  1.00  0.00           H
ATOM   1027  HA  ALA A 400      85.627 116.752 108.710  1.00  0.00           H
ATOM   1028  HB1 ALA A 400      83.825 117.938 109.990  1.00  0.00           H
ATOM   1029  HB2 ALA A 400      83.928 118.482 108.325  1.00  0.00           H
ATOM   1030  HB3 ALA A 400      82.640 117.382 108.815  1.00  0.00           H
ATOM   1031  N   TYR A 401      83.242 114.536 109.421  1.00 30.00           N
ATOM   1032  CA  TYR A 401      82.849 113.416 110.286  1.00 30.00           C
ATOM   1033  C   TYR A 401      83.441 112.058 109.859  1.00 30.00           C
ATOM   1034  O   TYR A 401      83.122 111.056 110.501  1.00 30.00           O
ATOM   1035  CB  TYR A 401      81.308 113.346 110.411  1.00 20.00           C
ATOM   1036  CG  TYR A 401      80.591 114.577 110.959  1.00 20.00           C
ATOM   1037  CD1 TYR A 401      81.190 115.410 111.932  1.00 20.00           C
ATOM   1038  CD2 TYR A 401      79.285 114.872 110.512  1.00 20.00           C
ATOM   1039  CE1 TYR A 401      80.490 116.517 112.449  1.00 20.00           C
ATOM   1040  CE2 TYR A 401      78.585 115.981 111.028  1.00 20.00           C
ATOM   1041  CZ  TYR A 401      79.185 116.800 112.004  1.00 20.00           C
ATOM   1042  OH  TYR A 401      78.500 117.862 112.521  1.00 20.00           O
ATOM   1043  H   TYR A 401      82.597 114.768 108.677  1.00  0.00           H
ATOM   1044  HA  TYR A 401      83.256 113.587 111.282  1.00  0.00           H
ATOM   1045  HB2 TYR A 401      80.883 113.103 109.436  1.00  0.00           H
ATOM   1046  HB3 TYR A 401      81.031 112.519 111.066  1.00  0.00           H
ATOM   1047  HD1 TYR A 401      82.186 115.215 112.296  1.00  0.00           H
ATOM   1048  HD2 TYR A 401      78.811 114.248 109.768  1.00  0.00           H
ATOM   1049  HE1 TYR A 401      80.961 117.144 113.192  1.00  0.00           H
ATOM   1050  HE2 TYR A 401      77.587 116.198 110.676  1.00  0.00           H
ATOM   1051  HH  TYR A 401      78.961 118.278 113.263  1.00  0.00           H
ATOM   1052  N   SER A 402      84.318 112.036 108.836  1.00 30.00           N
ATOM   1053  CA  SER A 402      85.140 110.877 108.474  1.00 30.00           C
ATOM   1054  C   SER A 402      86.083 110.514 109.641  1.00 30.00           C
ATOM   1055  O   SER A 402      86.781 111.394 110.148  1.00 30.00           O
```

```
ATOM   1056  CB  SER A 402      85.896 111.177 107.161  1.00 20.00           C
ATOM   1057  OG  SER A 402      86.686 110.076 106.758  1.00  0.00           O
ATOM   1058  H   SER A 402      84.519 112.894 108.342  1.00  0.00           H
ATOM   1059  HA  SER A 402      84.466 110.038 108.291  1.00  0.00           H
ATOM   1060  HB2 SER A 402      85.192 111.397 106.358  1.00  0.00           H
ATOM   1061  HB3 SER A 402      86.533 112.056 107.267  1.00  0.00           H
ATOM   1062  HG  SER A 402      87.187 110.322 105.973  1.00  0.00           H
ATOM   1063  N   SER A 403      86.036 109.245 110.077  1.00 30.00           N
ATOM   1064  CA  SER A 403      86.611 108.797 111.346  1.00 30.00           C
ATOM   1065  C   SER A 403      88.084 108.365 111.197  1.00 30.00           C
ATOM   1066  O   SER A 403      88.381 107.171 111.183  1.00 30.00           O
ATOM   1067  CB  SER A 403      85.686 107.712 111.937  1.00 20.00           C
ATOM   1068  OG  SER A 403      86.025 107.440 113.281  1.00  0.00           O
ATOM   1069  H   SER A 403      85.460 108.574 109.590  1.00  0.00           H
ATOM   1070  HA  SER A 403      86.594 109.632 112.044  1.00  0.00           H
ATOM   1071  HB2 SER A 403      84.650 108.052 111.923  1.00  0.00           H
ATOM   1072  HB3 SER A 403      85.726 106.790 111.354  1.00  0.00           H
ATOM   1073  HG  SER A 403      85.590 106.629 113.557  1.00  0.00           H
ATOM   1074  N   SER A 404      88.970 109.371 111.073  1.00 30.00           N
ATOM   1075  CA  SER A 404      90.436 109.307 110.954  1.00 30.00           C
ATOM   1076  C   SER A 404      90.993 108.693 109.647  1.00 30.00           C
ATOM   1077  O   SER A 404      92.098 109.066 109.250  1.00 30.00           O
ATOM   1078  CB  SER A 404      91.064 108.657 112.209  1.00 20.00           C
ATOM   1079  OG  SER A 404      92.474 108.781 112.194  1.00  0.00           O
ATOM   1080  H   SER A 404      88.597 110.310 111.116  1.00  0.00           H
ATOM   1081  HA  SER A 404      90.750 110.352 110.945  1.00  0.00           H
ATOM   1082  HB2 SER A 404      90.693 109.138 113.115  1.00  0.00           H
ATOM   1083  HB3 SER A 404      90.807 107.600 112.280  1.00  0.00           H
ATOM   1084  HG  SER A 404      92.813 108.318 111.423  1.00  0.00           H
ATOM   1085  N   GLU A 405      90.236 107.802 108.985  1.00 30.00           N
ATOM   1086  CA  GLU A 405      90.566 107.191 107.693  1.00 30.00           C
ATOM   1087  C   GLU A 405      90.356 108.176 106.523  1.00 30.00           C
ATOM   1088  O   GLU A 405      90.225 109.377 106.757  1.00 30.00           O
ATOM   1089  CB  GLU A 405      89.774 105.873 107.542  1.00 20.00           C
ATOM   1090  CG  GLU A 405      90.102 104.841 108.644  1.00  0.00           C
ATOM   1091  CD  GLU A 405      89.394 103.494 108.480  1.00  0.00           C
ATOM   1092  OE1 GLU A 405      88.591 103.354 107.531  1.00  0.00           O
ATOM   1093  OE2 GLU A 405      89.677 102.613 109.321  1.00  0.00           O1-
ATOM   1094  H   GLU A 405      89.352 107.522 109.388  1.00  0.00           H
ATOM   1095  HA  GLU A 405      91.629 106.940 107.696  1.00  0.00           H
ATOM   1096  HB2 GLU A 405      88.703 106.089 107.549  1.00  0.00           H
ATOM   1097  HB3 GLU A 405      89.986 105.420 106.572  1.00  0.00           H
ATOM   1098  HG2 GLU A 405      91.177 104.657 108.665  1.00  0.00           H
ATOM   1099  HG3 GLU A 405      89.836 105.235 109.625  1.00  0.00           H
ATOM   1100  N   THR A 406      90.374 107.669 105.276  1.00 30.00           N
ATOM   1101  CA  THR A 406      90.318 108.456 104.034  1.00 30.00           C
ATOM   1102  C   THR A 406      91.570 109.375 103.914  1.00 30.00           C
ATOM   1103  O   THR A 406      91.469 110.588 104.109  1.00 30.00           O
ATOM   1104  CB  THR A 406      88.962 109.217 103.827  1.00 20.00           C
ATOM   1105  CG2 THR A 406      87.728 108.342 104.083  1.00 20.00           C
ATOM   1106  OG1 THR A 406      88.791 110.447 104.510  1.00 20.00           O
ATOM   1107  H   THR A 406      90.476 106.672 105.158  1.00  0.00           H
ATOM   1108  HA  THR A 406      90.355 107.731 103.223  1.00  0.00           H
ATOM   1109  HB  THR A 406      88.930 109.497 102.777  1.00  0.00           H
ATOM   1110  HG1 THR A 406      89.532 111.014 104.277  1.00  0.00           H
ATOM   1111 HG21 THR A 406      86.815 108.900 103.884  1.00  0.00           H
ATOM   1112 HG22 THR A 406      87.732 107.461 103.442  1.00  0.00           H
ATOM   1113 HG23 THR A 406      87.677 108.000 105.117  1.00  0.00           H
ATOM   1114  N   PRO A 407      92.762 108.757 103.707  1.00 30.00           N
ATOM   1115  CA  PRO A 407      94.068 109.380 104.019  1.00 30.00           C
ATOM   1116  C   PRO A 407      94.473 110.606 103.180  1.00 30.00           C
ATOM   1117  O   PRO A 407      95.384 111.323 103.594  1.00 30.00           O
ATOM   1118  CB  PRO A 407      95.074 108.225 103.880  1.00 20.00           C
ATOM   1119  CG  PRO A 407      94.423 107.272 102.894  1.00  0.00           C
ATOM   1120  CD  PRO A 407      92.946 107.375 103.253  1.00  0.00           C
ATOM   1121  HA  PRO A 407      94.055 109.707 105.060  1.00  0.00           H
ATOM   1122  HB2 PRO A 407      96.069 108.537 103.558  1.00  0.00           H
ATOM   1123  HB3 PRO A 407      95.188 107.730 104.845  1.00  0.00           H
ATOM   1124  HG2 PRO A 407      94.579 107.639 101.879  1.00  0.00           H
ATOM   1125  HG3 PRO A 407      94.812 106.255 102.949  1.00  0.00           H
ATOM   1126  HD2 PRO A 407      92.325 107.122 102.393  1.00  0.00           H
```

```
ATOM   1127  HD3 PRO A 407      92.707 106.693 104.070  1.00  0.00           H
ATOM   1128  N   ASN A 408      93.753 110.873 102.077  1.00 30.00           N
ATOM   1129  CA  ASN A 408      93.809 112.125 101.318  1.00 30.00           C
ATOM   1130  C   ASN A 408      92.862 113.162 101.959  1.00 30.00           C
ATOM   1131  O   ASN A 408      91.961 113.670 101.294  1.00 30.00           O
ATOM   1132  CB  ASN A 408      93.445 111.842  99.843  1.00 20.00           C
ATOM   1133  CG  ASN A 408      94.418 110.904  99.125  1.00  0.00           C
ATOM   1134  ND2 ASN A 408      93.885 109.973  98.332  1.00  0.00           N
ATOM   1135  OD1 ASN A 408      95.632 111.028  99.264  1.00  0.00           O
ATOM   1136  H   ASN A 408      93.021 110.232 101.807  1.00  0.00           H
ATOM   1137  HA  ASN A 408      94.821 112.533 101.357  1.00  0.00           H
ATOM   1138  HB2 ASN A 408      92.438 111.435  99.797  1.00  0.00           H
ATOM   1139  HB3 ASN A 408      93.426 112.769  99.275  1.00  0.00           H
ATOM   1140 HD21 ASN A 408      94.484 109.338  97.825  1.00  0.00           H
ATOM   1141 HD22 ASN A 408      92.880 109.895  98.239  1.00  0.00           H
ATOM   1142  N   ARG A 409      93.079 113.435 103.257  1.00 30.00           N
ATOM   1143  CA  ARG A 409      92.325 114.394 104.059  1.00 30.00           C
ATOM   1144  C   ARG A 409      92.810 115.830 103.796  1.00 30.00           C
ATOM   1145  O   ARG A 409      93.882 116.029 103.221  1.00 30.00           O
ATOM   1146  CB  ARG A 409      92.448 113.979 105.548  1.00 20.00           C
ATOM   1147  CG  ARG A 409      91.548 114.735 106.555  1.00 20.00           C
ATOM   1148  CD  ARG A 409      90.046 114.415 106.431  1.00 20.00           C
ATOM   1149  NE  ARG A 409      89.760 113.048 106.883  1.00 20.00           N
ATOM   1150  CZ  ARG A 409      89.126 112.701 108.013  1.00 20.00           C
ATOM   1151  NH1 ARG A 409      88.538 113.603 108.810  1.00 20.00           N
ATOM   1152  NH2 ARG A 409      89.080 111.411 108.347  1.00 20.00           N1+
ATOM   1153  H   ARG A 409      93.844 112.970 103.726  1.00  0.00           H
ATOM   1154  HA  ARG A 409      91.280 114.334 103.751  1.00  0.00           H
ATOM   1155  HB2 ARG A 409      92.237 112.912 105.633  1.00  0.00           H
ATOM   1156  HB3 ARG A 409      93.488 114.092 105.858  1.00  0.00           H
ATOM   1157  HG2 ARG A 409      91.732 115.807 106.607  1.00  0.00           H
ATOM   1158  HG3 ARG A 409      91.862 114.362 107.532  1.00  0.00           H
ATOM   1159  HD2 ARG A 409      89.796 114.367 105.374  1.00  0.00           H
ATOM   1160  HD3 ARG A 409      89.394 115.188 106.837  1.00  0.00           H
ATOM   1161  HE  ARG A 409      90.160 112.307 106.323  1.00  0.00           H
ATOM   1162 HH11 ARG A 409      88.038 113.304 109.638  1.00  0.00           H
ATOM   1163 HH12 ARG A 409      88.524 114.578 108.550  1.00  0.00           H
ATOM   1164 HH21 ARG A 409      88.548 111.121 109.157  1.00  0.00           H
ATOM   1165 HH22 ARG A 409      89.513 110.705 107.757  1.00  0.00           H
ATOM   1166  N   HIS A 410      91.998 116.802 104.238  1.00 30.00           N
ATOM   1167  CA  HIS A 410      92.187 118.243 104.062  1.00 30.00           C
ATOM   1168  C   HIS A 410      91.869 118.685 102.628  1.00 30.00           C
ATOM   1169  O   HIS A 410      92.571 119.532 102.079  1.00 30.00           O
ATOM   1170  CB  HIS A 410      93.551 118.754 104.595  1.00 20.00           C
ATOM   1171  CG  HIS A 410      93.840 118.359 106.022  1.00 20.00           C
ATOM   1172  CD2 HIS A 410      93.308 118.805 107.209  1.00 20.00           C
ATOM   1173  ND1 HIS A 410      94.777 117.401 106.369  1.00 20.00           N
ATOM   1174  CE1 HIS A 410      94.759 117.300 107.701  1.00 20.00           C
ATOM   1175  NE2 HIS A 410      93.885 118.116 108.278  1.00 20.00           N
ATOM   1176  H   HIS A 410      91.146 116.537 104.709  1.00  0.00           H
ATOM   1177  HA  HIS A 410      91.419 118.704 104.684  1.00  0.00           H
ATOM   1178  HB2 HIS A 410      93.567 119.843 104.562  1.00  0.00           H
ATOM   1179  HB3 HIS A 410      94.375 118.430 103.958  1.00  0.00           H
ATOM   1180  HD1 HIS A 410      95.357 116.871 105.733  1.00  0.00           H
ATOM   1181  HD2 HIS A 410      92.563 119.569 107.370  1.00  0.00           H
ATOM   1182  HE1 HIS A 410      95.389 116.620 108.256  1.00  0.00           H
ATOM   1183  N   ASP A 411      90.747 118.169 102.088  1.00 30.00           N
ATOM   1184  CA  ASP A 411      90.095 118.649 100.859  1.00 30.00           C
ATOM   1185  C   ASP A 411      89.713 120.143 100.908  1.00 30.00           C
ATOM   1186  O   ASP A 411      89.542 120.735  99.847  1.00 30.00           O
ATOM   1187  CB  ASP A 411      88.856 117.813 100.443  1.00 20.00           C
ATOM   1188  CG  ASP A 411      89.081 116.302 100.314  1.00 20.00           C
ATOM   1189  OD1 ASP A 411      90.248 115.894 100.131  1.00 20.00           O
ATOM   1190  OD2 ASP A 411      88.059 115.582 100.314  1.00 20.00           O1-
ATOM   1191  H   ASP A 411      90.253 117.440 102.582  1.00  0.00           H
ATOM   1192  HA  ASP A 411      90.831 118.544 100.062  1.00  0.00           H
ATOM   1193  HB2 ASP A 411      88.072 117.970 101.185  1.00  0.00           H
ATOM   1194  HB3 ASP A 411      88.479 118.173  99.485  1.00  0.00           H
ATOM   1195  N   MET A 412      89.639 120.718 102.126  1.00 30.00           N
ATOM   1196  CA  MET A 412      89.430 122.128 102.461  1.00 30.00           C
ATOM   1197  C   MET A 412      90.232 123.087 101.563  1.00 30.00           C
```

```
ATOM   1198  O   MET A 412      89.635 123.738 100.708  1.00 30.00           O
ATOM   1199  CB  MET A 412      89.728 122.376 103.965  1.00 20.00           C
ATOM   1200  CG  MET A 412      89.135 121.351 104.948  1.00 20.00           C
ATOM   1201  SD  MET A 412      87.339 121.118 104.870  1.00 20.00           S
ATOM   1202  CE  MET A 412      87.161 119.862 106.162  1.00 20.00           C
ATOM   1203  H   MET A 412      89.791 120.119 102.923  1.00  0.00           H
ATOM   1204  HA  MET A 412      88.373 122.328 102.304  1.00  0.00           H
ATOM   1205  HB2 MET A 412      89.382 123.374 104.239  1.00  0.00           H
ATOM   1206  HB3 MET A 412      90.804 122.384 104.138  1.00  0.00           H
ATOM   1207  HG2 MET A 412      89.384 121.659 105.965  1.00  0.00           H
ATOM   1208  HG3 MET A 412      89.610 120.380 104.808  1.00  0.00           H
ATOM   1209  HE1 MET A 412      86.107 119.701 106.389  1.00  0.00           H
ATOM   1210  HE2 MET A 412      87.600 118.918 105.841  1.00  0.00           H
ATOM   1211  HE3 MET A 412      87.662 120.183 107.074  1.00  0.00           H
ATOM   1212  N   LEU A 413      91.563 123.105 101.744  1.00 30.00           N
ATOM   1213  CA  LEU A 413      92.505 123.963 101.025  1.00 30.00           C
ATOM   1214  C   LEU A 413      93.087 123.335  99.745  1.00 30.00           C
ATOM   1215  O   LEU A 413      93.971 123.948  99.148  1.00 30.00           O
ATOM   1216  CB  LEU A 413      93.582 124.490 102.009  1.00 20.00           C
ATOM   1217  CG  LEU A 413      94.735 123.540 102.432  1.00  0.00           C
ATOM   1218  CD1 LEU A 413      95.851 124.321 103.160  1.00  0.00           C
ATOM   1219  CD2 LEU A 413      94.259 122.337 103.272  1.00  0.00           C
ATOM   1220  H   LEU A 413      91.972 122.490 102.433  1.00  0.00           H
ATOM   1221  HA  LEU A 413      91.957 124.841 100.687  1.00  0.00           H
ATOM   1222  HB2 LEU A 413      94.030 125.365 101.536  1.00  0.00           H
ATOM   1223  HB3 LEU A 413      93.081 124.869 102.899  1.00  0.00           H
ATOM   1224  HG  LEU A 413      95.195 123.140 101.528  1.00  0.00           H
ATOM   1225 HD11 LEU A 413      96.825 124.101 102.722  1.00  0.00           H
ATOM   1226 HD12 LEU A 413      95.711 125.400 103.098  1.00  0.00           H
ATOM   1227 HD13 LEU A 413      95.913 124.077 104.222  1.00  0.00           H
ATOM   1228 HD21 LEU A 413      94.945 122.094 104.084  1.00  0.00           H
ATOM   1229 HD22 LEU A 413      93.283 122.506 103.723  1.00  0.00           H
ATOM   1230 HD23 LEU A 413      94.191 121.447 102.649  1.00  0.00           H
ATOM   1231  N   LEU A 414      92.595 122.150  99.342  1.00 30.00           N
ATOM   1232  CA  LEU A 414      93.077 121.412  98.170  1.00 30.00           C
ATOM   1233  C   LEU A 414      92.075 121.402  97.000  1.00 30.00           C
ATOM   1234  O   LEU A 414      92.457 120.924  95.931  1.00 30.00           O
ATOM   1235  CB  LEU A 414      93.441 119.970  98.593  1.00 20.00           C
ATOM   1236  CG  LEU A 414      94.660 119.863  99.538  1.00  0.00           C
ATOM   1237  CD1 LEU A 414      94.819 118.426 100.080  1.00  0.00           C
ATOM   1238  CD2 LEU A 414      95.959 120.385  98.890  1.00  0.00           C
ATOM   1239  H   LEU A 414      91.867 121.704  99.882  1.00  0.00           H
ATOM   1240  HA  LEU A 414      93.973 121.887  97.769  1.00  0.00           H
ATOM   1241  HB2 LEU A 414      92.570 119.517  99.066  1.00  0.00           H
ATOM   1242  HB3 LEU A 414      93.647 119.361  97.712  1.00  0.00           H
ATOM   1243  HG  LEU A 414      94.459 120.494 100.404  1.00  0.00           H
ATOM   1244 HD11 LEU A 414      94.970 118.434 101.160  1.00  0.00           H
ATOM   1245 HD12 LEU A 414      93.934 117.818  99.891  1.00  0.00           H
ATOM   1246 HD13 LEU A 414      95.666 117.904  99.638  1.00  0.00           H
ATOM   1247 HD21 LEU A 414      96.814 119.737  99.084  1.00  0.00           H
ATOM   1248 HD22 LEU A 414      95.867 120.478  97.808  1.00  0.00           H
ATOM   1249 HD23 LEU A 414      96.212 121.370  99.283  1.00  0.00           H
ATOM   1250  N   VAL A 415      90.841 121.911  97.189  1.00 30.00           N
ATOM   1251  CA  VAL A 415      89.813 121.947  96.139  1.00 30.00           C
ATOM   1252  C   VAL A 415      89.190 123.357  96.009  1.00 30.00           C
ATOM   1253  O   VAL A 415      89.088 123.855  94.890  1.00 30.00           O
ATOM   1254  CB  VAL A 415      88.737 120.828  96.313  1.00 20.00           C
ATOM   1255  CG1 VAL A 415      89.387 119.439  96.491  1.00  0.00           C
ATOM   1256  CG2 VAL A 415      87.651 121.061  97.384  1.00  0.00           C
ATOM   1257  H   VAL A 415      90.584 122.278  98.094  1.00  0.00           H
ATOM   1258  HA  VAL A 415      90.285 121.768  95.171  1.00  0.00           H
ATOM   1259  HB  VAL A 415      88.210 120.767  95.360  1.00  0.00           H
ATOM   1260 HG11 VAL A 415      88.672 118.633  96.337  1.00  0.00           H
ATOM   1261 HG12 VAL A 415      90.196 119.281  95.781  1.00  0.00           H
ATOM   1262 HG13 VAL A 415      89.809 119.317  97.489  1.00  0.00           H
ATOM   1263 HG21 VAL A 415      87.364 120.134  97.878  1.00  0.00           H
ATOM   1264 HG22 VAL A 415      87.967 121.747  98.167  1.00  0.00           H
ATOM   1265 HG23 VAL A 415      86.747 121.457  96.931  1.00  0.00           H
ATOM   1266  N   GLU A 416      88.884 124.017  97.140  1.00 30.00           N
ATOM   1267  CA  GLU A 416      88.650 125.462  97.287  1.00 30.00           C
ATOM   1268  C   GLU A 416      87.377 126.169  96.724  1.00 30.00           C
```

```
ATOM   1269  O   GLU A 416      87.415 127.400  96.664  1.00 30.00           O
ATOM   1270  CB  GLU A 416      89.944 126.261  96.974  1.00 20.00           C
ATOM   1271  CG  GLU A 416      91.190 125.767  97.732  1.00  0.00           C
ATOM   1272  CD  GLU A 416      92.327 126.783  97.670  1.00  0.00           C
ATOM   1273  OE1 GLU A 416      93.319 126.498  96.966  1.00  0.00           O
ATOM   1274  OE2 GLU A 416      92.181 127.831  98.339  1.00  0.00           O1-
ATOM   1275  H   GLU A 416      88.986 123.522  98.014  1.00  0.00           H
ATOM   1276  HA  GLU A 416      88.495 125.556  98.360  1.00  0.00           H
ATOM   1277  HB2 GLU A 416      90.143 126.233  95.902  1.00  0.00           H
ATOM   1278  HB3 GLU A 416      89.797 127.315  97.217  1.00  0.00           H
ATOM   1279  HG2 GLU A 416      90.949 125.595  98.779  1.00  0.00           H
ATOM   1280  HG3 GLU A 416      91.535 124.813  97.332  1.00  0.00           H
ATOM   1281  N   PRO A 417      86.224 125.502  96.449  1.00 30.00           N
ATOM   1282  CA  PRO A 417      84.933 126.222  96.499  1.00 30.00           C
ATOM   1283  C   PRO A 417      84.449 126.452  97.951  1.00 30.00           C
ATOM   1284  O   PRO A 417      83.566 127.279  98.169  1.00 30.00           O
ATOM   1285  CB  PRO A 417      83.986 125.264  95.771  1.00 20.00           C
ATOM   1286  CG  PRO A 417      84.458 123.900  96.240  1.00 20.00           C
ATOM   1287  CD  PRO A 417      85.976 124.064  96.348  1.00 20.00           C
ATOM   1288  HA  PRO A 417      84.966 127.178  95.971  1.00  0.00           H
ATOM   1289  HB2 PRO A 417      82.930 125.440  95.982  1.00  0.00           H
ATOM   1290  HB3 PRO A 417      84.126 125.355  94.694  1.00  0.00           H
ATOM   1291  HG2 PRO A 417      84.034 123.682  97.220  1.00  0.00           H
ATOM   1292  HG3 PRO A 417      84.149 123.097  95.587  1.00  0.00           H
ATOM   1293  HD2 PRO A 417      86.348 123.547  97.226  1.00  0.00           H
ATOM   1294  HD3 PRO A 417      86.458 123.662  95.459  1.00  0.00           H
ATOM   1295  N   LEU A 418      85.015 125.668  98.889  1.00 30.00           N
ATOM   1296  CA  LEU A 418      84.565 125.492 100.261  1.00 30.00           C
ATOM   1297  C   LEU A 418      85.628 125.852 101.311  1.00 30.00           C
ATOM   1298  O   LEU A 418      85.279 125.898 102.489  1.00 30.00           O
ATOM   1299  CB  LEU A 418      83.942 124.082 100.432  1.00 20.00           C
ATOM   1300  CG  LEU A 418      84.846 122.865 100.099  1.00 20.00           C
ATOM   1301  CD1 LEU A 418      85.979 122.641 101.115  1.00 20.00           C
ATOM   1302  CD2 LEU A 418      84.009 121.585  99.897  1.00 20.00           C
ATOM   1303  H   LEU A 418      85.729 125.018  98.592  1.00  0.00           H
ATOM   1304  HA  LEU A 418      83.766 126.205 100.422  1.00  0.00           H
ATOM   1305  HB2 LEU A 418      83.071 124.051  99.774  1.00  0.00           H
ATOM   1306  HB3 LEU A 418      83.537 123.975 101.439  1.00  0.00           H
ATOM   1307  HG  LEU A 418      85.322 123.052  99.140  1.00  0.00           H
ATOM   1308 HD11 LEU A 418      86.198 121.586 101.278  1.00  0.00           H
ATOM   1309 HD12 LEU A 418      86.898 123.100 100.751  1.00  0.00           H
ATOM   1310 HD13 LEU A 418      85.751 123.076 102.087  1.00  0.00           H
ATOM   1311 HD21 LEU A 418      84.210 121.140  98.923  1.00  0.00           H
ATOM   1312 HD22 LEU A 418      84.220 120.822 100.646  1.00  0.00           H
ATOM   1313 HD23 LEU A 418      82.939 121.783  99.947  1.00  0.00           H
ATOM   1314  N   ASN A 419      86.869 126.172 100.890  1.00 30.00           N
ATOM   1315  CA  ASN A 419      87.895 126.797 101.740  1.00 30.00           C
ATOM   1316  C   ASN A 419      87.416 128.167 102.250  1.00 30.00           C
ATOM   1317  O   ASN A 419      87.546 128.465 103.437  1.00 30.00           O
ATOM   1318  CB  ASN A 419      89.209 126.989 100.939  1.00 20.00           C
ATOM   1319  CG  ASN A 419      90.486 127.148 101.779  1.00  0.00           C
ATOM   1320  ND2 ASN A 419      91.556 127.630 101.145  1.00  0.00           N
ATOM   1321  OD1 ASN A 419      90.530 126.830 102.967  1.00  0.00           O
ATOM   1322  H   ASN A 419      87.088 126.106  99.907  1.00  0.00           H
ATOM   1323  HA  ASN A 419      88.070 126.181 102.624  1.00  0.00           H
ATOM   1324  HB2 ASN A 419      89.379 126.111 100.330  1.00  0.00           H
ATOM   1325  HB3 ASN A 419      89.125 127.812 100.226  1.00  0.00           H
ATOM   1326 HD21 ASN A 419      92.429 127.767 101.627  1.00  0.00           H
ATOM   1327 HD22 ASN A 419      91.524 127.833 100.149  1.00  0.00           H
ATOM   1328  N   ARG A 420      86.834 128.944 101.322  1.00 30.00           N
ATOM   1329  CA  ARG A 420      86.262 130.256 101.578  1.00 30.00           C
ATOM   1330  C   ARG A 420      84.808 130.194 102.068  1.00 30.00           C
ATOM   1331  O   ARG A 420      84.396 131.157 102.704  1.00 30.00           O
ATOM   1332  CB  ARG A 420      86.397 131.133 100.320  1.00 20.00           C
ATOM   1333  CG  ARG A 420      87.861 131.363  99.900  1.00 20.00           C
ATOM   1334  CD  ARG A 420      87.983 132.450  98.827  1.00 20.00           C
ATOM   1335  NE  ARG A 420      89.337 132.552  98.264  1.00 20.00           N
ATOM   1336  CZ  ARG A 420      89.864 131.747  97.325  1.00 20.00           C
ATOM   1337  NH1 ARG A 420      89.209 130.671  96.872  1.00 20.00           N
ATOM   1338  NH2 ARG A 420      91.066 132.035  96.813  1.00 20.00           N1+
ATOM   1339  H   ARG A 420      86.767 128.610 100.372  1.00  0.00           H
```

```
ATOM   1340  HA  ARG A 420      86.843 130.733 102.369  1.00  0.00           H
ATOM   1341  HB2 ARG A 420      85.841 130.691  99.492  1.00  0.00           H
ATOM   1342  HB3 ARG A 420      85.935 132.102 100.514  1.00  0.00           H
ATOM   1343  HG2 ARG A 420      88.370 131.738 100.789  1.00  0.00           H
ATOM   1344  HG3 ARG A 420      88.383 130.448  99.618  1.00  0.00           H
ATOM   1345  HD2 ARG A 420      87.189 132.414  98.086  1.00  0.00           H
ATOM   1346  HD3 ARG A 420      87.867 133.406  99.338  1.00  0.00           H
ATOM   1347  HE  ARG A 420      89.896 133.322  98.603  1.00  0.00           H
ATOM   1348 HH11 ARG A 420      89.573 130.147  96.083  1.00  0.00           H
ATOM   1349 HH12 ARG A 420      88.301 130.430  97.241  1.00  0.00           H
ATOM   1350 HH21 ARG A 420      91.403 131.500  96.018  1.00  0.00           H
ATOM   1351 HH22 ARG A 420      91.596 132.830  97.136  1.00  0.00           H
ATOM   1352  N   LEU A 421      84.060 129.100 101.813  1.00 30.00           N
ATOM   1353  CA  LEU A 421      82.701 128.923 102.351  1.00 30.00           C
ATOM   1354  C   LEU A 421      82.713 128.708 103.873  1.00 30.00           C
ATOM   1355  O   LEU A 421      81.858 129.265 104.557  1.00 30.00           O
ATOM   1356  CB  LEU A 421      81.968 127.765 101.641  1.00 20.00           C
ATOM   1357  CG  LEU A 421      80.468 127.614 101.982  1.00 20.00           C
ATOM   1358  CD1 LEU A 421      79.658 128.866 101.602  1.00 20.00           C
ATOM   1359  CD2 LEU A 421      79.892 126.336 101.342  1.00 20.00           C
ATOM   1360  H   LEU A 421      84.444 128.334 101.280  1.00  0.00           H
ATOM   1361  HA  LEU A 421      82.154 129.846 102.151  1.00  0.00           H
ATOM   1362  HB2 LEU A 421      82.079 127.870 100.560  1.00  0.00           H
ATOM   1363  HB3 LEU A 421      82.455 126.833 101.927  1.00  0.00           H
ATOM   1364  HG  LEU A 421      80.368 127.480 103.059  1.00  0.00           H
ATOM   1365 HD11 LEU A 421      78.627 128.630 101.338  1.00  0.00           H
ATOM   1366 HD12 LEU A 421      79.626 129.568 102.434  1.00  0.00           H
ATOM   1367 HD13 LEU A 421      80.103 129.388 100.756  1.00  0.00           H
ATOM   1368 HD21 LEU A 421      79.589 125.630 102.112  1.00  0.00           H
ATOM   1369 HD22 LEU A 421      79.017 126.528 100.721  1.00  0.00           H
ATOM   1370 HD23 LEU A 421      80.624 125.830 100.711  1.00  0.00           H
ATOM   1371  N   LEU A 422      83.704 127.945 104.368  1.00 30.00           N
ATOM   1372  CA  LEU A 422      83.970 127.756 105.794  1.00 30.00           C
ATOM   1373  C   LEU A 422      84.440 129.058 106.467  1.00 30.00           C
ATOM   1374  O   LEU A 422      84.028 129.319 107.593  1.00 30.00           O
ATOM   1375  CB  LEU A 422      85.004 126.623 105.985  1.00 20.00           C
ATOM   1376  CG  LEU A 422      84.479 125.212 105.624  1.00 20.00           C
ATOM   1377  CD1 LEU A 422      85.643 124.210 105.474  1.00 20.00           C
ATOM   1378  CD2 LEU A 422      83.394 124.712 106.603  1.00 20.00           C
ATOM   1379  H   LEU A 422      84.356 127.504 103.734  1.00  0.00           H
ATOM   1380  HA  LEU A 422      83.035 127.462 106.275  1.00  0.00           H
ATOM   1381  HB2 LEU A 422      85.878 126.853 105.372  1.00  0.00           H
ATOM   1382  HB3 LEU A 422      85.363 126.608 107.015  1.00  0.00           H
ATOM   1383  HG  LEU A 422      84.004 125.273 104.645  1.00  0.00           H
ATOM   1384 HD11 LEU A 422      85.540 123.339 106.118  1.00  0.00           H
ATOM   1385 HD12 LEU A 422      85.701 123.840 104.450  1.00  0.00           H
ATOM   1386 HD13 LEU A 422      86.609 124.660 105.705  1.00  0.00           H
ATOM   1387 HD21 LEU A 422      83.561 123.690 106.943  1.00  0.00           H
ATOM   1388 HD22 LEU A 422      83.335 125.332 107.494  1.00  0.00           H
ATOM   1389 HD23 LEU A 422      82.412 124.730 106.130  1.00  0.00           H
ATOM   1390  N   GLN A 423      85.241 129.880 105.762  1.00 30.00           N
ATOM   1391  CA  GLN A 423      85.685 131.191 106.244  1.00 30.00           C
ATOM   1392  C   GLN A 423      84.543 132.229 106.283  1.00 30.00           C
ATOM   1393  O   GLN A 423      84.522 133.062 107.188  1.00 30.00           O
ATOM   1394  CB  GLN A 423      86.867 131.680 105.375  1.00 20.00           C
ATOM   1395  CG  GLN A 423      87.591 132.944 105.891  1.00 20.00           C
ATOM   1396  CD  GLN A 423      88.216 132.759 107.278  1.00 20.00           C
ATOM   1397  NE2 GLN A 423      88.025 133.729 108.173  1.00 20.00           N
ATOM   1398  OE1 GLN A 423      88.879 131.757 107.540  1.00 20.00           O
ATOM   1399  H   GLN A 423      85.548 129.611 104.838  1.00  0.00           H
ATOM   1400  HA  GLN A 423      86.039 131.049 107.267  1.00  0.00           H
ATOM   1401  HB2 GLN A 423      87.598 130.874 105.280  1.00  0.00           H
ATOM   1402  HB3 GLN A 423      86.514 131.874 104.363  1.00  0.00           H
ATOM   1403  HG2 GLN A 423      86.909 133.796 105.900  1.00  0.00           H
ATOM   1404  HG3 GLN A 423      88.391 133.208 105.199  1.00  0.00           H
ATOM   1405 HE21 GLN A 423      88.425 133.656 109.095  1.00  0.00           H
ATOM   1406 HE22 GLN A 423      87.459 134.545 107.954  1.00  0.00           H
ATOM   1407  N   ASP A 424      83.611 132.143 105.318  1.00 30.00           N
ATOM   1408  CA  ASP A 424      82.450 133.025 105.200  1.00 30.00           C
ATOM   1409  C   ASP A 424      81.351 132.640 106.214  1.00 30.00           C
ATOM   1410  O   ASP A 424      80.686 133.538 106.724  1.00 30.00           O
```

```
ATOM   1411  CB  ASP A 424      81.906 133.044 103.750  1.00 20.00           C
ATOM   1412  CG  ASP A 424      81.190 134.342 103.369  1.00  0.00           C
ATOM   1413  OD1 ASP A 424      81.791 135.415 103.585  1.00  0.00           O
ATOM   1414  OD2 ASP A 424      80.096 134.236 102.775  1.00  0.00           O1-
ATOM   1415  H   ASP A 424      83.717 131.451 104.588  1.00  0.00           H
ATOM   1416  HA  ASP A 424      82.790 134.029 105.464  1.00  0.00           H
ATOM   1417  HB2 ASP A 424      82.709 132.927 103.026  1.00  0.00           H
ATOM   1418  HB3 ASP A 424      81.245 132.192 103.587  1.00  0.00           H
ATOM   1419  N   LYS A 425      81.215 131.337 106.533  1.00  0.00           N
ATOM   1420  CA  LYS A 425      80.415 130.830 107.656  1.00  0.00           C
ATOM   1421  C   LYS A 425      81.016 131.206 109.024  1.00  0.00           C
ATOM   1422  O   LYS A 425      80.247 131.436 109.957  1.00  0.00           O
ATOM   1423  CB  LYS A 425      80.233 129.297 107.545  1.00 20.00           C
ATOM   1424  CG  LYS A 425      79.188 128.853 106.503  1.00 20.00           C
ATOM   1425  CD  LYS A 425      79.108 127.320 106.369  1.00 20.00           C
ATOM   1426  CE  LYS A 425      78.109 126.820 105.309  1.00 20.00           C
ATOM   1427  NZ  LYS A 425      76.708 127.091 105.678  1.00 20.00           N1+
ATOM   1428  H   LYS A 425      81.769 130.649 106.040  1.00  0.00           H
ATOM   1429  HA  LYS A 425      79.429 131.296 107.596  1.00  0.00           H
ATOM   1430  HB2 LYS A 425      81.197 128.824 107.349  1.00  0.00           H
ATOM   1431  HB3 LYS A 425      79.903 128.904 108.508  1.00  0.00           H
ATOM   1432  HG2 LYS A 425      78.213 129.242 106.796  1.00  0.00           H
ATOM   1433  HG3 LYS A 425      79.414 129.295 105.532  1.00  0.00           H
ATOM   1434  HD2 LYS A 425      80.099 126.935 106.122  1.00  0.00           H
ATOM   1435  HD3 LYS A 425      78.854 126.883 107.336  1.00  0.00           H
ATOM   1436  HE2 LYS A 425      78.315 127.274 104.340  1.00  0.00           H
ATOM   1437  HE3 LYS A 425      78.219 125.743 105.184  1.00  0.00           H
ATOM   1438  HZ1 LYS A 425      76.559 128.084 105.772  1.00  0.00           H
ATOM   1439  HZ2 LYS A 425      76.091 126.732 104.963  1.00  0.00           H
ATOM   1440  HZ3 LYS A 425      76.481 126.645 106.563  1.00  0.00           H
ATOM   1441  N   TRP A 426      82.359 131.280 109.113  1.00  0.00           N
ATOM   1442  CA  TRP A 426      83.107 131.665 110.309  1.00  0.00           C
ATOM   1443  C   TRP A 426      82.838 133.123 110.713  1.00  0.00           C
ATOM   1444  O   TRP A 426      82.041 133.332 111.625  1.00  0.00           O
ATOM   1445  CB  TRP A 426      84.602 131.299 110.173  1.00 20.00           C
ATOM   1446  CG  TRP A 426      85.459 131.550 111.380  1.00 20.00           C
ATOM   1447  CD1 TRP A 426      86.307 132.590 111.537  1.00 20.00           C
ATOM   1448  CD2 TRP A 426      85.523 130.793 112.628  1.00 20.00           C
ATOM   1449  CE2 TRP A 426      86.444 131.443 113.505  1.00 20.00           C
ATOM   1450  CE3 TRP A 426      84.899 129.619 113.111  1.00 20.00           C
ATOM   1451  NE1 TRP A 426      86.889 132.533 112.785  1.00 20.00           N
ATOM   1452  CZ2 TRP A 426      86.732 130.956 114.792  1.00 20.00           C
ATOM   1453  CZ3 TRP A 426      85.187 129.114 114.396  1.00 20.00           C
ATOM   1454  CH2 TRP A 426      86.102 129.780 115.235  1.00 20.00           C
ATOM   1455  H   TRP A 426      82.917 131.055 108.300  1.00  0.00           H
ATOM   1456  HA  TRP A 426      82.738 131.050 111.124  1.00  0.00           H
ATOM   1457  HB2 TRP A 426      84.687 130.235 109.957  1.00  0.00           H
ATOM   1458  HB3 TRP A 426      85.051 131.812 109.326  1.00  0.00           H
ATOM   1459  HD1 TRP A 426      86.484 133.344 110.786  1.00  0.00           H
ATOM   1460  HE1 TRP A 426      87.550 133.222 113.111  1.00  0.00           H
ATOM   1461  HE3 TRP A 426      84.196 129.096 112.480  1.00  0.00           H
ATOM   1462  HZ2 TRP A 426      87.435 131.472 115.429  1.00  0.00           H
ATOM   1463  HZ3 TRP A 426      84.705 128.211 114.739  1.00  0.00           H
ATOM   1464  HH2 TRP A 426      86.323 129.383 116.214  1.00  0.00           H
ATOM   1465  N   ASP A 427      83.466 134.093 110.025  1.00 30.00           N
ATOM   1466  CA  ASP A 427      83.394 135.536 110.321  1.00 30.00           C
ATOM   1467  C   ASP A 427      81.981 136.149 110.422  1.00 30.00           C
ATOM   1468  O   ASP A 427      81.832 137.154 111.115  1.00 30.00           O
ATOM   1469  CB  ASP A 427      84.261 136.391 109.362  1.00 20.00           C
ATOM   1470  CG  ASP A 427      85.751 136.034 109.335  1.00  0.00           C
ATOM   1471  OD1 ASP A 427      86.321 135.829 110.431  1.00  0.00           O
ATOM   1472  OD2 ASP A 427      86.327 136.077 108.227  1.00  0.00           O1-
ATOM   1473  H   ASP A 427      84.075 133.836 109.259  1.00  0.00           H
ATOM   1474  HA  ASP A 427      83.817 135.642 111.320  1.00  0.00           H
ATOM   1475  HB2 ASP A 427      83.868 136.263 108.351  1.00  0.00           H
ATOM   1476  HB3 ASP A 427      84.176 137.453 109.593  1.00  0.00           H
ATOM   1477  N   ARG A 428      80.980 135.548 109.755  1.00 30.00           N
ATOM   1478  CA  ARG A 428      79.606 136.048 109.714  1.00 30.00           C
ATOM   1479  C   ARG A 428      78.694 135.489 110.824  1.00 30.00           C
ATOM   1480  O   ARG A 428      77.772 136.202 111.223  1.00 30.00           O
ATOM   1481  CB  ARG A 428      79.029 135.730 108.322  1.00 20.00           C
```

```
ATOM   1482  CG  ARG A 428      77.622 136.280 108.030  1.00 20.00           C
ATOM   1483  CD  ARG A 428      77.252 136.060 106.560  1.00 20.00           C
ATOM   1484  NE  ARG A 428      75.830 136.310 106.285  1.00 20.00           N
ATOM   1485  CZ  ARG A 428      74.840 135.410 106.420  1.00 20.00           C
ATOM   1486  NH1 ARG A 428      73.586 135.762 106.106  1.00 20.00           N
ATOM   1487  NH2 ARG A 428      75.086 134.165 106.853  1.00 20.00           N1+
ATOM   1488  H   ARG A 428      81.177 134.724 109.206  1.00  0.00           H
ATOM   1489  HA  ARG A 428      79.621 137.135 109.820  1.00  0.00           H
ATOM   1490  HB2 ARG A 428      79.719 136.105 107.564  1.00  0.00           H
ATOM   1491  HB3 ARG A 428      78.993 134.647 108.200  1.00  0.00           H
ATOM   1492  HG2 ARG A 428      76.851 135.893 108.697  1.00  0.00           H
ATOM   1493  HG3 ARG A 428      77.675 137.356 108.204  1.00  0.00           H
ATOM   1494  HD2 ARG A 428      77.748 136.850 105.996  1.00  0.00           H
ATOM   1495  HD3 ARG A 428      77.636 135.123 106.161  1.00  0.00           H
ATOM   1496  HE  ARG A 428      75.601 137.249 105.990  1.00  0.00           H
ATOM   1497 HH11 ARG A 428      72.833 135.093 106.180  1.00  0.00           H
ATOM   1498 HH12 ARG A 428      73.389 136.691 105.760  1.00  0.00           H
ATOM   1499 HH21 ARG A 428      74.345 133.483 106.944  1.00  0.00           H
ATOM   1500 HH22 ARG A 428      76.031 133.893 107.090  1.00  0.00           H
ATOM   1501  N   PHE A 429      78.912 134.233 111.262  1.00 30.00           N
ATOM   1502  CA  PHE A 429      77.915 133.492 112.043  1.00 30.00           C
ATOM   1503  C   PHE A 429      78.495 132.601 113.153  1.00 30.00           C
ATOM   1504  O   PHE A 429      77.995 132.668 114.277  1.00 30.00           O
ATOM   1505  CB  PHE A 429      76.953 132.750 111.080  1.00 20.00           C
ATOM   1506  CG  PHE A 429      75.894 131.866 111.720  1.00 20.00           C
ATOM   1507  CD1 PHE A 429      74.757 132.449 112.319  1.00 20.00           C
ATOM   1508  CD2 PHE A 429      76.096 130.475 111.845  1.00 20.00           C
ATOM   1509  CE1 PHE A 429      73.823 131.648 112.963  1.00 20.00           C
ATOM   1510  CE2 PHE A 429      75.153 129.692 112.497  1.00 20.00           C
ATOM   1511  CZ  PHE A 429      74.018 130.275 113.046  1.00 20.00           C
ATOM   1512  H   PHE A 429      79.712 133.711 110.929  1.00  0.00           H
ATOM   1513  HA  PHE A 429      77.301 134.212 112.587  1.00  0.00           H
ATOM   1514  HB2 PHE A 429      76.423 133.491 110.480  1.00  0.00           H
ATOM   1515  HB3 PHE A 429      77.527 132.154 110.369  1.00  0.00           H
ATOM   1516  HD1 PHE A 429      74.605 133.515 112.262  1.00  0.00           H
ATOM   1517  HD2 PHE A 429      76.979 130.013 111.429  1.00  0.00           H
ATOM   1518  HE1 PHE A 429      72.941 132.091 113.402  1.00  0.00           H
ATOM   1519  HE2 PHE A 429      75.305 128.625 112.579  1.00  0.00           H
ATOM   1520  HZ  PHE A 429      73.288 129.656 113.549  1.00  0.00           H
ATOM   1521  N   VAL A 430      79.506 131.774 112.835  1.00 30.00           N
ATOM   1522  CA  VAL A 430      80.061 130.794 113.776  1.00 30.00           C
ATOM   1523  C   VAL A 430      81.078 131.401 114.763  1.00 30.00           C
ATOM   1524  O   VAL A 430      81.127 130.955 115.906  1.00 30.00           O
ATOM   1525  CB  VAL A 430      80.713 129.582 113.053  1.00 20.00           C
ATOM   1526  CG1 VAL A 430      81.525 128.634 113.968  1.00 20.00           C
ATOM   1527  CG2 VAL A 430      79.653 128.796 112.263  1.00 20.00           C
ATOM   1528  H   VAL A 430      79.870 131.750 111.890  1.00  0.00           H
ATOM   1529  HA  VAL A 430      79.233 130.391 114.359  1.00  0.00           H
ATOM   1530  HB  VAL A 430      81.416 129.964 112.321  1.00  0.00           H
ATOM   1531 HG11 VAL A 430      81.941 127.799 113.415  1.00  0.00           H
ATOM   1532 HG12 VAL A 430      82.383 129.125 114.421  1.00  0.00           H
ATOM   1533 HG13 VAL A 430      80.916 128.239 114.778  1.00  0.00           H
ATOM   1534 HG21 VAL A 430      80.080 127.917 111.787  1.00  0.00           H
ATOM   1535 HG22 VAL A 430      78.839 128.461 112.905  1.00  0.00           H
ATOM   1536 HG23 VAL A 430      79.219 129.403 111.470  1.00  0.00           H
ATOM   1537  N   LYS A 431      81.871 132.389 114.323  1.00  0.00           N
ATOM   1538  CA  LYS A 431      82.915 133.045 115.114  1.00  0.00           C
ATOM   1539  C   LYS A 431      82.363 133.718 116.381  1.00  0.00           C
ATOM   1540  O   LYS A 431      82.901 133.492 117.463  1.00  0.00           O
ATOM   1541  CB  LYS A 431      83.653 134.033 114.193  1.00 20.00           C
ATOM   1542  CG  LYS A 431      84.841 134.796 114.799  1.00 20.00           C
ATOM   1543  CD  LYS A 431      85.466 135.706 113.733  1.00 20.00           C
ATOM   1544  CE  LYS A 431      86.796 136.346 114.134  1.00 20.00           C
ATOM   1545  NZ  LYS A 431      87.315 137.169 113.028  1.00 20.00           N1+
ATOM   1546  H   LYS A 431      81.781 132.705 113.365  1.00  0.00           H
ATOM   1547  HA  LYS A 431      83.623 132.273 115.423  1.00  0.00           H
ATOM   1548  HB2 LYS A 431      84.032 133.470 113.341  1.00  0.00           H
ATOM   1549  HB3 LYS A 431      82.940 134.754 113.790  1.00  0.00           H
ATOM   1550  HG2 LYS A 431      84.524 135.396 115.652  1.00  0.00           H
ATOM   1551  HG3 LYS A 431      85.580 134.083 115.166  1.00  0.00           H
ATOM   1552  HD2 LYS A 431      85.616 135.128 112.822  1.00  0.00           H
```

```
ATOM   1553  HD3 LYS A 431      84.753 136.490 113.476  1.00  0.00           H
ATOM   1554  HE2 LYS A 431      86.677 136.967 115.023  1.00  0.00           H
ATOM   1555  HE3 LYS A 431      87.528 135.573 114.371  1.00  0.00           H
ATOM   1556  HZ1 LYS A 431      87.368 136.608 112.186  1.00  0.00           H
ATOM   1557  HZ2 LYS A 431      86.684 137.940 112.858  1.00  0.00           H
ATOM   1558  HZ3 LYS A 431      88.230 137.528 113.259  1.00  0.00           H
ATOM   1559  N   ARG A 432      81.269 134.481 116.216  1.00  0.00           N
ATOM   1560  CA  ARG A 432      80.569 135.188 117.288  1.00  0.00           C
ATOM   1561  C   ARG A 432      79.938 134.252 118.339  1.00  0.00           C
ATOM   1562  O   ARG A 432      80.007 134.580 119.524  1.00  0.00           O
ATOM   1563  CB  ARG A 432      79.537 136.166 116.676  1.00 20.00           C
ATOM   1564  CG  ARG A 432      78.407 135.503 115.863  1.00  0.00           C
ATOM   1565  CD  ARG A 432      77.416 136.504 115.261  1.00  0.00           C
ATOM   1566  NE  ARG A 432      76.211 135.811 114.786  1.00  0.00           N
ATOM   1567  CZ  ARG A 432      75.076 136.400 114.375  1.00  0.00           C
ATOM   1568  NH1 ARG A 432      74.963 137.734 114.322  1.00  0.00           N
ATOM   1569  NH2 ARG A 432      74.033 135.635 114.029  1.00  0.00           N1+
ATOM   1570  H   ARG A 432      80.885 134.590 115.289  1.00  0.00           H
ATOM   1571  HA  ARG A 432      81.317 135.791 117.807  1.00  0.00           H
ATOM   1572  HB2 ARG A 432      79.093 136.748 117.485  1.00  0.00           H
ATOM   1573  HB3 ARG A 432      80.057 136.886 116.043  1.00  0.00           H
ATOM   1574  HG2 ARG A 432      78.892 134.992 115.032  1.00  0.00           H
ATOM   1575  HG3 ARG A 432      77.870 134.740 116.426  1.00  0.00           H
ATOM   1576  HD2 ARG A 432      77.200 137.320 115.952  1.00  0.00           H
ATOM   1577  HD3 ARG A 432      77.852 136.946 114.364  1.00  0.00           H
ATOM   1578  HE  ARG A 432      76.262 134.801 114.785  1.00  0.00           H
ATOM   1579 HH11 ARG A 432      74.096 138.173 114.047  1.00  0.00           H
ATOM   1580 HH12 ARG A 432      75.746 138.313 114.588  1.00  0.00           H
ATOM   1581 HH21 ARG A 432      73.153 136.055 113.768  1.00  0.00           H
ATOM   1582 HH22 ARG A 432      74.098 134.629 114.098  1.00  0.00           H
ATOM   1583  N   ILE A 433      79.363 133.109 117.910  1.00  0.00           N
ATOM   1584  CA  ILE A 433      78.743 132.143 118.823  1.00  0.00           C
ATOM   1585  C   ILE A 433      79.791 131.221 119.483  1.00  0.00           C
ATOM   1586  O   ILE A 433      79.563 130.801 120.613  1.00  0.00           O
ATOM   1587  CB  ILE A 433      77.610 131.305 118.146  1.00 20.00           C
ATOM   1588  CG1 ILE A 433      76.607 130.745 119.182  1.00 20.00           C
ATOM   1589  CG2 ILE A 433      78.092 130.172 117.219  1.00 20.00           C
ATOM   1590  CD1 ILE A 433      75.308 130.207 118.561  1.00 20.00           C
ATOM   1591  H   ILE A 433      79.354 132.879 116.927  1.00  0.00           H
ATOM   1592  HA  ILE A 433      78.273 132.715 119.623  1.00  0.00           H
ATOM   1593  HB  ILE A 433      77.056 132.003 117.516  1.00  0.00           H
ATOM   1594 HG12 ILE A 433      77.078 129.951 119.763  1.00  0.00           H
ATOM   1595 HG13 ILE A 433      76.344 131.522 119.900  1.00  0.00           H
ATOM   1596 HG21 ILE A 433      77.265 129.722 116.671  1.00  0.00           H
ATOM   1597 HG22 ILE A 433      78.776 130.567 116.480  1.00  0.00           H
ATOM   1598 HG23 ILE A 433      78.598 129.372 117.761  1.00  0.00           H
ATOM   1599 HD11 ILE A 433      74.485 130.263 119.274  1.00  0.00           H
ATOM   1600 HD12 ILE A 433      75.015 130.774 117.677  1.00  0.00           H
ATOM   1601 HD13 ILE A 433      75.417 129.162 118.267  1.00  0.00           H
ATOM   1602  N   PHE A 434      80.932 130.972 118.811  1.00  0.00           N
ATOM   1603  CA  PHE A 434      82.066 130.205 119.335  1.00  0.00           C
ATOM   1604  C   PHE A 434      82.795 130.913 120.490  1.00  0.00           C
ATOM   1605  O   PHE A 434      83.108 130.258 121.485  1.00  0.00           O
ATOM   1606  CB  PHE A 434      83.019 129.811 118.184  1.00 20.00           C
ATOM   1607  CG  PHE A 434      84.292 129.090 118.597  1.00 20.00           C
ATOM   1608  CD1 PHE A 434      84.251 127.716 118.914  1.00 20.00           C
ATOM   1609  CD2 PHE A 434      85.485 129.810 118.829  1.00 20.00           C
ATOM   1610  CE1 PHE A 434      85.396 127.070 119.361  1.00 20.00           C
ATOM   1611  CE2 PHE A 434      86.617 129.146 119.282  1.00 20.00           C
ATOM   1612  CZ  PHE A 434      86.576 127.781 119.531  1.00 20.00           C
ATOM   1613  H   PHE A 434      81.043 131.336 117.874  1.00  0.00           H
ATOM   1614  HA  PHE A 434      81.662 129.277 119.744  1.00  0.00           H
ATOM   1615  HB2 PHE A 434      82.483 129.155 117.497  1.00  0.00           H
ATOM   1616  HB3 PHE A 434      83.292 130.693 117.604  1.00  0.00           H
ATOM   1617  HD1 PHE A 434      83.332 127.159 118.798  1.00  0.00           H
ATOM   1618  HD2 PHE A 434      85.523 130.875 118.649  1.00  0.00           H
ATOM   1619  HE1 PHE A 434      85.369 126.012 119.579  1.00  0.00           H
ATOM   1620  HE2 PHE A 434      87.533 129.690 119.444  1.00  0.00           H
ATOM   1621  HZ  PHE A 434      87.466 127.272 119.867  1.00  0.00           H
ATOM   1622  N   TYR A 435      83.018 132.234 120.359  1.00  0.00           N
ATOM   1623  CA  TYR A 435      83.547 133.076 121.436  1.00  0.00           C
```

```
ATOM   1624  C   TYR A 435      82.563 133.237 122.608  1.00  0.00           C
ATOM   1625  O   TYR A 435      83.023 133.386 123.739  1.00  0.00           O
ATOM   1626  CB  TYR A 435      83.994 134.449 120.889  1.00 20.00           C
ATOM   1627  CG  TYR A 435      85.309 134.441 120.126  1.00 20.00           C
ATOM   1628  CD1 TYR A 435      86.492 134.006 120.762  1.00 20.00           C
ATOM   1629  CD2 TYR A 435      85.369 134.898 118.793  1.00 20.00           C
ATOM   1630  CE1 TYR A 435      87.715 134.003 120.064  1.00 20.00           C
ATOM   1631  CE2 TYR A 435      86.590 134.883 118.091  1.00 20.00           C
ATOM   1632  CZ  TYR A 435      87.763 134.429 118.723  1.00 20.00           C
ATOM   1633  OH  TYR A 435      88.942 134.410 118.037  1.00 20.00           O
ATOM   1634  H   TYR A 435      82.763 132.705 119.500  1.00  0.00           H
ATOM   1635  HA  TYR A 435      84.422 132.565 121.839  1.00  0.00           H
ATOM   1636  HB2 TYR A 435      83.202 134.888 120.280  1.00  0.00           H
ATOM   1637  HB3 TYR A 435      84.143 135.141 121.720  1.00  0.00           H
ATOM   1638  HD1 TYR A 435      86.469 133.678 121.791  1.00  0.00           H
ATOM   1639  HD2 TYR A 435      84.481 135.268 118.304  1.00  0.00           H
ATOM   1640  HE1 TYR A 435      88.614 133.676 120.565  1.00  0.00           H
ATOM   1641  HE2 TYR A 435      86.627 135.228 117.069  1.00  0.00           H
ATOM   1642  HH  TYR A 435      89.679 134.090 118.563  1.00  0.00           H
ATOM   1643  N   PHE A 436      81.248 133.151 122.336  1.00  0.00           N
ATOM   1644  CA  PHE A 436      80.207 133.087 123.360  1.00  0.00           C
ATOM   1645  C   PHE A 436      80.205 131.749 124.129  1.00  0.00           C
ATOM   1646  O   PHE A 436      80.060 131.777 125.348  1.00  0.00           O
ATOM   1647  CB  PHE A 436      78.838 133.462 122.752  1.00 20.00           C
ATOM   1648  CG  PHE A 436      77.646 133.355 123.686  1.00 20.00           C
ATOM   1649  CD1 PHE A 436      77.422 134.353 124.658  1.00 20.00           C
ATOM   1650  CD2 PHE A 436      76.850 132.189 123.704  1.00 20.00           C
ATOM   1651  CE1 PHE A 436      76.392 134.209 125.577  1.00 20.00           C
ATOM   1652  CE2 PHE A 436      75.824 132.065 124.631  1.00 20.00           C
ATOM   1653  CZ  PHE A 436      75.593 133.073 125.559  1.00 20.00           C
ATOM   1654  H   PHE A 436      80.942 133.042 121.379  1.00  0.00           H
ATOM   1655  HA  PHE A 436      80.433 133.861 124.097  1.00  0.00           H
ATOM   1656  HB2 PHE A 436      78.885 134.493 122.399  1.00  0.00           H
ATOM   1657  HB3 PHE A 436      78.639 132.859 121.869  1.00  0.00           H
ATOM   1658  HD1 PHE A 436      78.052 135.231 124.691  1.00  0.00           H
ATOM   1659  HD2 PHE A 436      77.037 131.391 122.999  1.00  0.00           H
ATOM   1660  HE1 PHE A 436      76.217 134.979 126.314  1.00  0.00           H
ATOM   1661  HE2 PHE A 436      75.209 131.177 124.638  1.00  0.00           H
ATOM   1662  HZ  PHE A 436      74.797 132.965 126.282  1.00  0.00           H
ATOM   1663  N   ASN A 437      80.420 130.619 123.427  1.00 30.00           N
ATOM   1664  CA  ASN A 437      80.560 129.281 124.025  1.00 30.00           C
ATOM   1665  C   ASN A 437      81.823 129.161 124.895  1.00 30.00           C
ATOM   1666  O   ASN A 437      81.756 128.534 125.951  1.00 30.00           O
ATOM   1667  CB  ASN A 437      80.575 128.171 122.942  1.00 20.00           C
ATOM   1668  CG  ASN A 437      79.311 128.044 122.082  1.00 20.00           C
ATOM   1669  ND2 ASN A 437      78.125 128.263 122.657  1.00 20.00           N
ATOM   1670  OD1 ASN A 437      79.405 127.728 120.898  1.00 20.00           O
ATOM   1671  H   ASN A 437      80.510 130.669 122.420  1.00  0.00           H
ATOM   1672  HA  ASN A 437      79.700 129.127 124.680  1.00  0.00           H
ATOM   1673  HB2 ASN A 437      81.429 128.310 122.278  1.00  0.00           H
ATOM   1674  HB3 ASN A 437      80.722 127.201 123.420  1.00  0.00           H
ATOM   1675 HD21 ASN A 437      77.281 128.182 122.110  1.00  0.00           H
ATOM   1676 HD22 ASN A 437      78.063 128.518 123.632  1.00  0.00           H
ATOM   1677  N   PHE A 438      82.932 129.782 124.454  1.00  0.00           N
ATOM   1678  CA  PHE A 438      84.182 129.880 125.206  1.00  0.00           C
ATOM   1679  C   PHE A 438      84.043 130.737 126.475  1.00  0.00           C
ATOM   1680  O   PHE A 438      84.503 130.309 127.531  1.00  0.00           O
ATOM   1681  CB  PHE A 438      85.313 130.388 124.284  1.00 20.00           C
ATOM   1682  CG  PHE A 438      86.668 130.618 124.941  1.00 20.00           C
ATOM   1683  CD1 PHE A 438      87.254 129.623 125.755  1.00 20.00           C
ATOM   1684  CD2 PHE A 438      87.407 131.785 124.654  1.00 20.00           C
ATOM   1685  CE1 PHE A 438      88.523 129.810 126.282  1.00 20.00           C
ATOM   1686  CE2 PHE A 438      88.671 131.959 125.203  1.00 20.00           C
ATOM   1687  CZ  PHE A 438      89.226 130.977 126.014  1.00 20.00           C
ATOM   1688  H   PHE A 438      82.914 130.248 123.556  1.00  0.00           H
ATOM   1689  HA  PHE A 438      84.441 128.868 125.526  1.00  0.00           H
ATOM   1690  HB2 PHE A 438      85.462 129.691 123.460  1.00  0.00           H
ATOM   1691  HB3 PHE A 438      84.991 131.321 123.821  1.00  0.00           H
ATOM   1692  HD1 PHE A 438      86.721 128.710 125.974  1.00  0.00           H
ATOM   1693  HD2 PHE A 438      86.993 132.548 124.012  1.00  0.00           H
ATOM   1694  HE1 PHE A 438      88.966 129.046 126.903  1.00  0.00           H
```

```
ATOM   1695  HE2 PHE A 438      89.229 132.859 124.989  1.00  0.00           H
ATOM   1696  HZ  PHE A 438      90.212 131.118 126.431  1.00  0.00           H
ATOM   1697  N   PHE A 439      83.382 131.902 126.352  1.00  0.00           N
ATOM   1698  CA  PHE A 439      83.089 132.818 127.456  1.00  0.00           C
ATOM   1699  C   PHE A 439      82.182 132.204 128.538  1.00  0.00           C
ATOM   1700  O   PHE A 439      82.473 132.363 129.722  1.00  0.00           O
ATOM   1701  CB  PHE A 439      82.541 134.152 126.899  1.00 20.00           C
ATOM   1702  CG  PHE A 439      82.071 135.155 127.940  1.00 20.00           C
ATOM   1703  CD1 PHE A 439      83.017 135.922 128.654  1.00 20.00           C
ATOM   1704  CD2 PHE A 439      80.715 135.200 128.331  1.00 20.00           C
ATOM   1705  CE1 PHE A 439      82.601 136.746 129.692  1.00 20.00           C
ATOM   1706  CE2 PHE A 439      80.321 136.028 129.374  1.00 20.00           C
ATOM   1707  CZ  PHE A 439      81.259 136.800 130.048  1.00 20.00           C
ATOM   1708  H   PHE A 439      83.046 132.188 125.442  1.00  0.00           H
ATOM   1709  HA  PHE A 439      84.041 133.041 127.941  1.00  0.00           H
ATOM   1710  HB2 PHE A 439      83.324 134.632 126.310  1.00  0.00           H
ATOM   1711  HB3 PHE A 439      81.722 133.959 126.206  1.00  0.00           H
ATOM   1712  HD1 PHE A 439      84.065 135.864 128.402  1.00  0.00           H
ATOM   1713  HD2 PHE A 439      79.983 134.582 127.830  1.00  0.00           H
ATOM   1714  HE1 PHE A 439      83.325 137.338 130.232  1.00  0.00           H
ATOM   1715  HE2 PHE A 439      79.282 136.064 129.668  1.00  0.00           H
ATOM   1716  HZ  PHE A 439      80.945 137.437 130.861  1.00  0.00           H
ATOM   1717  N   VAL A 440      81.128 131.491 128.106  1.00  0.00           N
ATOM   1718  CA  VAL A 440      80.215 130.745 128.972  1.00  0.00           C
ATOM   1719  C   VAL A 440      80.900 129.553 129.672  1.00  0.00           C
ATOM   1720  O   VAL A 440      80.552 129.273 130.819  1.00  0.00           O
ATOM   1721  CB  VAL A 440      78.952 130.277 128.185  1.00 20.00           C
ATOM   1722  CG1 VAL A 440      78.117 129.152 128.836  1.00 20.00           C
ATOM   1723  CG2 VAL A 440      78.030 131.476 127.894  1.00 20.00           C
ATOM   1724  H   VAL A 440      80.943 131.432 127.113  1.00  0.00           H
ATOM   1725  HA  VAL A 440      79.886 131.429 129.757  1.00  0.00           H
ATOM   1726  HB  VAL A 440      79.287 129.892 127.220  1.00  0.00           H
ATOM   1727 HG11 VAL A 440      77.206 128.962 128.268  1.00  0.00           H
ATOM   1728 HG12 VAL A 440      78.660 128.207 128.874  1.00  0.00           H
ATOM   1729 HG13 VAL A 440      77.822 129.414 129.853  1.00  0.00           H
ATOM   1730 HG21 VAL A 440      77.200 131.186 127.250  1.00  0.00           H
ATOM   1731 HG22 VAL A 440      77.610 131.883 128.814  1.00  0.00           H
ATOM   1732 HG23 VAL A 440      78.559 132.287 127.394  1.00  0.00           H
ATOM   1733  N   TYR A 441      81.883 128.910 129.012  1.00  0.00           N
ATOM   1734  CA  TYR A 441      82.686 127.847 129.618  1.00  0.00           C
ATOM   1735  C   TYR A 441      83.703 128.377 130.645  1.00  0.00           C
ATOM   1736  O   TYR A 441      83.898 127.731 131.673  1.00  0.00           O
ATOM   1737  CB  TYR A 441      83.369 126.974 128.548  1.00 20.00           C
ATOM   1738  CG  TYR A 441      84.011 125.730 129.139  1.00 20.00           C
ATOM   1739  CD1 TYR A 441      83.182 124.744 129.712  1.00 20.00           C
ATOM   1740  CD2 TYR A 441      85.413 125.577 129.185  1.00 20.00           C
ATOM   1741  CE1 TYR A 441      83.743 123.611 130.323  1.00 20.00           C
ATOM   1742  CE2 TYR A 441      85.976 124.436 129.794  1.00 20.00           C
ATOM   1743  CZ  TYR A 441      85.138 123.454 130.359  1.00 20.00           C
ATOM   1744  OH  TYR A 441      85.663 122.345 130.945  1.00 20.00           O
ATOM   1745  H   TYR A 441      82.117 129.175 128.064  1.00  0.00           H
ATOM   1746  HA  TYR A 441      81.991 127.204 130.159  1.00  0.00           H
ATOM   1747  HB2 TYR A 441      82.629 126.641 127.819  1.00  0.00           H
ATOM   1748  HB3 TYR A 441      84.107 127.550 127.987  1.00  0.00           H
ATOM   1749  HD1 TYR A 441      82.110 124.869 129.704  1.00  0.00           H
ATOM   1750  HD2 TYR A 441      86.060 126.334 128.765  1.00  0.00           H
ATOM   1751  HE1 TYR A 441      83.107 122.862 130.768  1.00  0.00           H
ATOM   1752  HE2 TYR A 441      87.047 124.317 129.831  1.00  0.00           H
ATOM   1753  HH  TYR A 441      86.620 122.393 131.039  1.00  0.00           H
ATOM   1754  N   CYS A 442      84.302 129.550 130.373  1.00  0.00           N
ATOM   1755  CA  CYS A 442      85.187 130.262 131.299  1.00  0.00           C
ATOM   1756  C   CYS A 442      84.429 130.703 132.561  1.00  0.00           C
ATOM   1757  O   CYS A 442      84.940 130.500 133.659  1.00  0.00           O
ATOM   1758  CB  CYS A 442      85.866 131.481 130.647  1.00 20.00           C
ATOM   1759  SG  CYS A 442      87.059 130.940 129.397  1.00 20.00           S
ATOM   1760  H   CYS A 442      84.114 130.011 129.492  1.00  0.00           H
ATOM   1761  HA  CYS A 442      85.967 129.566 131.616  1.00  0.00           H
ATOM   1762  HB2 CYS A 442      85.134 132.142 130.182  1.00  0.00           H
ATOM   1763  HB3 CYS A 442      86.404 132.070 131.391  1.00  0.00           H
ATOM   1764  HG  CYS A 442      86.149 130.509 128.516  1.00  0.00           H
ATOM   1765  N   LEU A 443      83.210 131.238 132.376  1.00  0.00           N
```

```
ATOM   1766  CA  LEU A 443      82.277 131.621 133.435  1.00  0.00           C
ATOM   1767  C   LEU A 443      81.812 130.420 134.283  1.00  0.00           C
ATOM   1768  O   LEU A 443      81.740 130.553 135.503  1.00  0.00           O
ATOM   1769  CB  LEU A 443      81.099 132.388 132.790  1.00 20.00           C
ATOM   1770  CG  LEU A 443      80.042 132.961 133.762  1.00 20.00           C
ATOM   1771  CD1 LEU A 443      80.655 133.953 134.774  1.00 20.00           C
ATOM   1772  CD2 LEU A 443      78.862 133.577 132.978  1.00 20.00           C
ATOM   1773  H   LEU A 443      82.876 131.379 131.431  1.00  0.00           H
ATOM   1774  HA  LEU A 443      82.815 132.301 134.096  1.00  0.00           H
ATOM   1775  HB2 LEU A 443      81.497 133.209 132.192  1.00  0.00           H
ATOM   1776  HB3 LEU A 443      80.600 131.724 132.084  1.00  0.00           H
ATOM   1777  HG  LEU A 443      79.622 132.133 134.335  1.00  0.00           H
ATOM   1778 HD11 LEU A 443      80.031 134.831 134.937  1.00  0.00           H
ATOM   1779 HD12 LEU A 443      80.788 133.475 135.745  1.00  0.00           H
ATOM   1780 HD13 LEU A 443      81.633 134.312 134.453  1.00  0.00           H
ATOM   1781 HD21 LEU A 443      77.919 133.115 133.272  1.00  0.00           H
ATOM   1782 HD22 LEU A 443      78.760 134.650 133.140  1.00  0.00           H
ATOM   1783 HD23 LEU A 443      78.966 133.433 131.902  1.00  0.00           H
ATOM   1784  N   TYR A 444      81.552 129.271 133.633  1.00  0.00           N
ATOM   1785  CA  TYR A 444      81.216 127.997 134.275  1.00  0.00           C
ATOM   1786  C   TYR A 444      82.342 127.466 135.179  1.00  0.00           C
ATOM   1787  O   TYR A 444      82.060 127.092 136.316  1.00  0.00           O
ATOM   1788  CB  TYR A 444      80.785 126.966 133.205  1.00 20.00           C
ATOM   1789  CG  TYR A 444      80.672 125.528 133.689  1.00 20.00           C
ATOM   1790  CD1 TYR A 444      79.577 125.140 134.485  1.00 20.00           C
ATOM   1791  CD2 TYR A 444      81.684 124.590 133.387  1.00 20.00           C
ATOM   1792  CE1 TYR A 444      79.503 123.829 134.988  1.00 20.00           C
ATOM   1793  CE2 TYR A 444      81.609 123.278 133.893  1.00 20.00           C
ATOM   1794  CZ  TYR A 444      80.519 122.897 134.700  1.00 20.00           C
ATOM   1795  OH  TYR A 444      80.448 121.630 135.203  1.00 20.00           O
ATOM   1796  H   TYR A 444      81.610 129.253 132.624  1.00  0.00           H
ATOM   1797  HA  TYR A 444      80.351 128.185 134.914  1.00  0.00           H
ATOM   1798  HB2 TYR A 444      79.834 127.266 132.763  1.00  0.00           H
ATOM   1799  HB3 TYR A 444      81.503 126.973 132.388  1.00  0.00           H
ATOM   1800  HD1 TYR A 444      78.797 125.849 134.723  1.00  0.00           H
ATOM   1801  HD2 TYR A 444      82.533 124.880 132.785  1.00  0.00           H
ATOM   1802  HE1 TYR A 444      78.652 123.540 135.582  1.00  0.00           H
ATOM   1803  HE2 TYR A 444      82.392 122.570 133.669  1.00  0.00           H
ATOM   1804  HH  TYR A 444      79.717 121.506 135.815  1.00  0.00           H
ATOM   1805  N   MET A 445      83.585 127.455 134.666  1.00  0.00           N
ATOM   1806  CA  MET A 445      84.767 127.009 135.404  1.00  0.00           C
ATOM   1807  C   MET A 445      85.180 127.963 136.538  1.00  0.00           C
ATOM   1808  O   MET A 445      85.686 127.477 137.547  1.00  0.00           O
ATOM   1809  CB  MET A 445      85.936 126.732 134.435  1.00 20.00           C
ATOM   1810  CG  MET A 445      85.753 125.495 133.533  1.00 20.00           C
ATOM   1811  SD  MET A 445      85.375 123.919 134.361  1.00 20.00           S
ATOM   1812  CE  MET A 445      86.693 123.849 135.605  1.00 20.00           C
ATOM   1813  H   MET A 445      83.737 127.762 133.714  1.00  0.00           H
ATOM   1814  HA  MET A 445      84.505 126.072 135.897  1.00  0.00           H
ATOM   1815  HB2 MET A 445      86.105 127.609 133.808  1.00  0.00           H
ATOM   1816  HB3 MET A 445      86.859 126.600 134.998  1.00  0.00           H
ATOM   1817  HG2 MET A 445      86.658 125.349 132.944  1.00  0.00           H
ATOM   1818  HG3 MET A 445      84.956 125.675 132.813  1.00  0.00           H
ATOM   1819  HE1 MET A 445      86.695 122.877 136.098  1.00  0.00           H
ATOM   1820  HE2 MET A 445      87.665 124.002 135.139  1.00  0.00           H
ATOM   1821  HE3 MET A 445      86.549 124.614 136.367  1.00  0.00           H
ATOM   1822  N   ILE A 446      84.918 129.275 136.385  1.00  0.00           N
ATOM   1823  CA  ILE A 446      85.084 130.295 137.427  1.00  0.00           C
ATOM   1824  C   ILE A 446      84.092 130.112 138.597  1.00  0.00           C
ATOM   1825  O   ILE A 446      84.497 130.240 139.752  1.00  0.00           O
ATOM   1826  CB  ILE A 446      84.985 131.738 136.835  1.00 20.00           C
ATOM   1827  CG1 ILE A 446      86.281 132.081 136.064  1.00 20.00           C
ATOM   1828  CG2 ILE A 446      84.663 132.870 137.840  1.00 20.00           C
ATOM   1829  CD1 ILE A 446      86.134 133.249 135.076  1.00 20.00           C
ATOM   1830  H   ILE A 446      84.530 129.599 135.509  1.00  0.00           H
ATOM   1831  HA  ILE A 446      86.090 130.178 137.833  1.00  0.00           H
ATOM   1832  HB  ILE A 446      84.170 131.727 136.111  1.00  0.00           H
ATOM   1833 HG12 ILE A 446      87.077 132.308 136.774  1.00  0.00           H
ATOM   1834 HG13 ILE A 446      86.632 131.211 135.509  1.00  0.00           H
ATOM   1835 HG21 ILE A 446      84.669 133.845 137.353  1.00  0.00           H
ATOM   1836 HG22 ILE A 446      83.673 132.760 138.282  1.00  0.00           H
```

```
ATOM   1837 HG23 ILE A 446      85.394 132.902 138.649  1.00  0.00           H
ATOM   1838 HD11 ILE A 446      86.820 133.134 134.236  1.00  0.00           H
ATOM   1839 HD12 ILE A 446      85.125 133.310 134.666  1.00  0.00           H
ATOM   1840 HD13 ILE A 446      86.358 134.202 135.556  1.00  0.00           H
ATOM   1841  N   ILE A 447      82.830 129.780 138.277  1.00  0.00           N
ATOM   1842  CA  ILE A 447      81.786 129.443 139.247  1.00  0.00           C
ATOM   1843  C   ILE A 447      82.051 128.102 139.964  1.00  0.00           C
ATOM   1844  O   ILE A 447      81.835 128.023 141.173  1.00  0.00           O
ATOM   1845  CB  ILE A 447      80.375 129.448 138.573  1.00 20.00           C
ATOM   1846  CG1 ILE A 447      79.893 130.890 138.278  1.00 20.00           C
ATOM   1847  CG2 ILE A 447      79.266 128.634 139.276  1.00 20.00           C
ATOM   1848  CD1 ILE A 447      79.560 131.746 139.512  1.00 20.00           C
ATOM   1849  H   ILE A 447      82.567 129.713 137.302  1.00  0.00           H
ATOM   1850  HA  ILE A 447      81.804 130.212 140.021  1.00  0.00           H
ATOM   1851  HB  ILE A 447      80.494 128.980 137.594  1.00  0.00           H
ATOM   1852 HG12 ILE A 447      79.020 130.851 137.626  1.00  0.00           H
ATOM   1853 HG13 ILE A 447      80.656 131.416 137.704  1.00  0.00           H
ATOM   1854 HG21 ILE A 447      78.299 128.791 138.798  1.00  0.00           H
ATOM   1855 HG22 ILE A 447      79.462 127.562 139.231  1.00  0.00           H
ATOM   1856 HG23 ILE A 447      79.168 128.910 140.326  1.00  0.00           H
ATOM   1857 HD11 ILE A 447      78.957 132.608 139.226  1.00  0.00           H
ATOM   1858 HD12 ILE A 447      78.996 131.195 140.263  1.00  0.00           H
ATOM   1859 HD13 ILE A 447      80.464 132.128 139.984  1.00  0.00           H
ATOM   1860  N   PHE A 448      82.531 127.092 139.217  1.00  0.00           N
ATOM   1861  CA  PHE A 448      82.818 125.751 139.723  1.00  0.00           C
ATOM   1862  C   PHE A 448      84.061 125.674 140.622  1.00  0.00           C
ATOM   1863  O   PHE A 448      83.992 125.022 141.666  1.00  0.00           O
ATOM   1864  CB  PHE A 448      82.812 124.737 138.555  1.00 20.00           C
ATOM   1865  CG  PHE A 448      83.195 123.295 138.861  1.00 20.00           C
ATOM   1866  CD1 PHE A 448      82.774 122.659 140.051  1.00 20.00           C
ATOM   1867  CD2 PHE A 448      83.828 122.518 137.868  1.00 20.00           C
ATOM   1868  CE1 PHE A 448      83.095 121.331 140.283  1.00 20.00           C
ATOM   1869  CE2 PHE A 448      84.111 121.180 138.106  1.00 20.00           C
ATOM   1870  CZ  PHE A 448      83.770 120.596 139.318  1.00 20.00           C
ATOM   1871  H   PHE A 448      82.660 127.229 138.223  1.00  0.00           H
ATOM   1872  HA  PHE A 448      81.994 125.488 140.382  1.00  0.00           H
ATOM   1873  HB2 PHE A 448      81.816 124.711 138.110  1.00  0.00           H
ATOM   1874  HB3 PHE A 448      83.477 125.105 137.773  1.00  0.00           H
ATOM   1875  HD1 PHE A 448      82.219 123.202 140.802  1.00  0.00           H
ATOM   1876  HD2 PHE A 448      84.094 122.959 136.919  1.00  0.00           H
ATOM   1877  HE1 PHE A 448      82.797 120.864 141.210  1.00  0.00           H
ATOM   1878  HE2 PHE A 448      84.595 120.591 137.342  1.00  0.00           H
ATOM   1879  HZ  PHE A 448      84.002 119.559 139.501  1.00  0.00           H
ATOM   1880  N   THR A 449      85.151 126.371 140.251  1.00 30.00           N
ATOM   1881  CA  THR A 449      86.354 126.436 141.084  1.00 30.00           C
ATOM   1882  C   THR A 449      86.106 127.183 142.414  1.00 30.00           C
ATOM   1883  O   THR A 449      86.659 126.768 143.427  1.00 30.00           O
ATOM   1884  CB  THR A 449      87.584 127.039 140.345  1.00 20.00           C
ATOM   1885  CG2 THR A 449      87.548 128.558 140.105  1.00  0.00           C
ATOM   1886  OG1 THR A 449      88.759 126.800 141.092  1.00  0.00           O
ATOM   1887  H   THR A 449      85.164 126.887 139.381  1.00  0.00           H
ATOM   1888  HA  THR A 449      86.613 125.408 141.341  1.00  0.00           H
ATOM   1889  HB  THR A 449      87.697 126.533 139.385  1.00  0.00           H
ATOM   1890  HG1 THR A 449      88.702 127.286 141.919  1.00  0.00           H
ATOM   1891 HG21 THR A 449      88.411 128.885 139.526  1.00  0.00           H
ATOM   1892 HG22 THR A 449      86.656 128.835 139.554  1.00  0.00           H
ATOM   1893 HG23 THR A 449      87.555 129.130 141.032  1.00  0.00           H
ATOM   1894  N   ALA A 450      85.236 128.212 142.391  1.00 30.00           N
ATOM   1895  CA  ALA A 450      84.818 128.987 143.559  1.00 30.00           C
ATOM   1896  C   ALA A 450      83.887 128.210 144.507  1.00 30.00           C
ATOM   1897  O   ALA A 450      84.083 128.288 145.719  1.00 30.00           O
ATOM   1898  CB  ALA A 450      84.155 130.290 143.091  1.00 30.00           C
ATOM   1899  H   ALA A 450      84.813 128.479 141.513  1.00  0.00           H
ATOM   1900  HA  ALA A 450      85.715 129.253 144.122  1.00  0.00           H
ATOM   1901  HB1 ALA A 450      83.835 130.898 143.939  1.00  0.00           H
ATOM   1902  HB2 ALA A 450      84.849 130.892 142.504  1.00  0.00           H
ATOM   1903  HB3 ALA A 450      83.279 130.096 142.471  1.00  0.00           H
ATOM   1904  N   ALA A 451      82.920 127.457 143.949  1.00 30.00           N
ATOM   1905  CA  ALA A 451      81.983 126.609 144.698  1.00 30.00           C
ATOM   1906  C   ALA A 451      82.678 125.481 145.485  1.00 30.00           C
ATOM   1907  O   ALA A 451      82.250 125.173 146.600  1.00 30.00           O
```

```
ATOM   1908  CB  ALA A 451      80.929 126.039 143.735  1.00 30.00           C
ATOM   1909  H   ALA A 451      82.809 127.454 142.943  1.00  0.00           H
ATOM   1910  HA  ALA A 451      81.469 127.245 145.421  1.00  0.00           H
ATOM   1911  HB1 ALA A 451      80.210 125.409 144.260  1.00  0.00           H
ATOM   1912  HB2 ALA A 451      80.364 126.838 143.253  1.00  0.00           H
ATOM   1913  HB3 ALA A 451      81.385 125.435 142.949  1.00  0.00           H
ATOM   1914  N   ALA A 452      83.760 124.935 144.905  1.00 30.00           N
ATOM   1915  CA  ALA A 452      84.643 123.953 145.527  1.00 30.00           C
ATOM   1916  C   ALA A 452      85.630 124.570 146.536  1.00 30.00           C
ATOM   1917  O   ALA A 452      85.974 123.889 147.501  1.00 30.00           O
ATOM   1918  CB  ALA A 452      85.405 123.213 144.420  1.00 30.00           C
ATOM   1919  H   ALA A 452      84.023 125.237 143.977  1.00  0.00           H
ATOM   1920  HA  ALA A 452      84.030 123.223 146.061  1.00  0.00           H
ATOM   1921  HB1 ALA A 452      86.085 122.465 144.832  1.00  0.00           H
ATOM   1922  HB2 ALA A 452      84.719 122.696 143.749  1.00  0.00           H
ATOM   1923  HB3 ALA A 452      85.997 123.906 143.821  1.00  0.00           H
ATOM   1924  N   TYR A 453      86.071 125.824 146.310  1.00 30.00           N
ATOM   1925  CA  TYR A 453      87.033 126.538 147.165  1.00 30.00           C
ATOM   1926  C   TYR A 453      86.457 126.934 148.534  1.00 30.00           C
ATOM   1927  O   TYR A 453      87.208 126.968 149.507  1.00 30.00           O
ATOM   1928  CB  TYR A 453      87.612 127.755 146.413  1.00 20.00           C
ATOM   1929  CG  TYR A 453      88.909 128.312 146.975  1.00 20.00           C
ATOM   1930  CD1 TYR A 453      90.145 127.806 146.517  1.00 20.00           C
ATOM   1931  CD2 TYR A 453      88.892 129.338 147.942  1.00 20.00           C
ATOM   1932  CE1 TYR A 453      91.353 128.326 147.020  1.00 20.00           C
ATOM   1933  CE2 TYR A 453      90.100 129.849 148.455  1.00 20.00           C
ATOM   1934  CZ  TYR A 453      91.333 129.344 147.993  1.00 20.00           C
ATOM   1935  OH  TYR A 453      92.507 129.836 148.485  1.00 20.00           O
ATOM   1936  H   TYR A 453      85.757 126.318 145.486  1.00  0.00           H
ATOM   1937  HA  TYR A 453      87.864 125.874 147.377  1.00  0.00           H
ATOM   1938  HB2 TYR A 453      87.856 127.461 145.397  1.00  0.00           H
ATOM   1939  HB3 TYR A 453      86.869 128.549 146.325  1.00  0.00           H
ATOM   1940  HD1 TYR A 453      90.171 127.020 145.777  1.00  0.00           H
ATOM   1941  HD2 TYR A 453      87.950 129.725 148.298  1.00  0.00           H
ATOM   1942  HE1 TYR A 453      92.293 127.937 146.660  1.00  0.00           H
ATOM   1943  HE2 TYR A 453      90.075 130.630 149.200  1.00  0.00           H
ATOM   1944  HH  TYR A 453      92.375 130.496 149.170  1.00  0.00           H
ATOM   1945  N   TYR A 454      85.142 127.199 148.584  1.00 30.00           N
ATOM   1946  CA  TYR A 454      84.421 127.604 149.790  1.00 30.00           C
ATOM   1947  C   TYR A 454      83.460 126.506 150.283  1.00 30.00           C
ATOM   1948  O   TYR A 454      82.461 126.831 150.925  1.00 30.00           O
ATOM   1949  CB  TYR A 454      83.720 128.958 149.527  1.00 20.00           C
ATOM   1950  CG  TYR A 454      84.674 130.093 149.186  1.00 20.00           C
ATOM   1951  CD1 TYR A 454      85.600 130.547 150.148  1.00 20.00           C
ATOM   1952  CD2 TYR A 454      84.653 130.689 147.907  1.00 20.00           C
ATOM   1953  CE1 TYR A 454      86.506 131.578 149.832  1.00 20.00           C
ATOM   1954  CE2 TYR A 454      85.565 131.714 147.586  1.00 20.00           C
ATOM   1955  CZ  TYR A 454      86.494 132.157 148.548  1.00 20.00           C
ATOM   1956  OH  TYR A 454      87.386 133.142 148.238  1.00 20.00           O
ATOM   1957  H   TYR A 454      84.597 127.153 147.733  1.00  0.00           H
ATOM   1958  HA  TYR A 454      85.120 127.754 150.614  1.00  0.00           H
ATOM   1959  HB2 TYR A 454      82.987 128.845 148.726  1.00  0.00           H
ATOM   1960  HB3 TYR A 454      83.158 129.270 150.408  1.00  0.00           H
ATOM   1961  HD1 TYR A 454      85.625 130.100 151.131  1.00  0.00           H
ATOM   1962  HD2 TYR A 454      83.941 130.363 147.165  1.00  0.00           H
ATOM   1963  HE1 TYR A 454      87.217 131.914 150.572  1.00  0.00           H
ATOM   1964  HE2 TYR A 454      85.547 132.155 146.601  1.00  0.00           H
ATOM   1965  HH  TYR A 454      87.292 133.467 147.340  1.00  0.00           H
ATOM   1966  N   ARG A 455      83.779 125.226 150.011  1.00 30.00           N
ATOM   1967  CA  ARG A 455      83.023 124.066 150.495  1.00 30.00           C
ATOM   1968  C   ARG A 455      83.113 123.928 152.039  1.00 30.00           C
ATOM   1969  O   ARG A 455      84.199 124.135 152.585  1.00 30.00           O
ATOM   1970  CB  ARG A 455      83.532 122.787 149.783  1.00 20.00           C
ATOM   1971  CG  ARG A 455      84.924 122.268 150.206  1.00 20.00           C
ATOM   1972  CD  ARG A 455      85.346 121.011 149.435  1.00 20.00           C
ATOM   1973  NE  ARG A 455      86.569 120.421 149.991  1.00 20.00           N
ATOM   1974  CZ  ARG A 455      86.671 119.675 151.104  1.00 20.00           C
ATOM   1975  NH1 ARG A 455      85.599 119.397 151.862  1.00 20.00           N
ATOM   1976  NH2 ARG A 455      87.871 119.197 151.458  1.00 20.00           N1+
ATOM   1977  H   ARG A 455      84.620 125.026 149.488  1.00  0.00           H
ATOM   1978  HA  ARG A 455      81.999 124.224 150.168  1.00  0.00           H
```

```
ATOM   1979  HB2 ARG A 455      83.512 122.943 148.703  1.00  0.00           H
ATOM   1980  HB3 ARG A 455      82.810 121.993 149.975  1.00  0.00           H
ATOM   1981  HG2 ARG A 455      85.635 123.061 149.977  1.00  0.00           H
ATOM   1982  HG3 ARG A 455      85.013 122.086 151.277  1.00  0.00           H
ATOM   1983  HD2 ARG A 455      84.539 120.293 149.285  1.00  0.00           H
ATOM   1984  HD3 ARG A 455      85.654 121.331 148.439  1.00  0.00           H
ATOM   1985  HE  ARG A 455      87.418 120.587 149.462  1.00  0.00           H
ATOM   1986 HH11 ARG A 455      85.689 118.831 152.693  1.00  0.00           H
ATOM   1987 HH12 ARG A 455      84.696 119.772 151.613  1.00  0.00           H
ATOM   1988 HH21 ARG A 455      87.984 118.635 152.289  1.00  0.00           H
ATOM   1989 HH22 ARG A 455      88.678 119.380 150.875  1.00  0.00           H
ATOM   1990  N   PRO A 456      82.005 123.541 152.716  1.00 30.00           N
ATOM   1991  CA  PRO A 456      82.051 123.053 154.111  1.00 30.00           C
ATOM   1992  C   PRO A 456      82.966 121.822 154.281  1.00 30.00           C
ATOM   1993  O   PRO A 456      82.765 120.817 153.598  1.00 30.00           O
ATOM   1994  CB  PRO A 456      80.581 122.730 154.444  1.00 20.00           C
ATOM   1995  CG  PRO A 456      79.766 123.550 153.460  1.00 20.00           C
ATOM   1996  CD  PRO A 456      80.633 123.550 152.210  1.00 20.00           C
ATOM   1997  HA  PRO A 456      82.394 123.877 154.740  1.00  0.00           H
ATOM   1998  HB2 PRO A 456      80.366 121.673 154.292  1.00  0.00           H
ATOM   1999  HB3 PRO A 456      80.328 122.962 155.479  1.00  0.00           H
ATOM   2000  HG2 PRO A 456      78.761 123.160 153.293  1.00  0.00           H
ATOM   2001  HG3 PRO A 456      79.671 124.570 153.834  1.00  0.00           H
ATOM   2002  HD2 PRO A 456      80.405 124.428 151.606  1.00  0.00           H
ATOM   2003  HD3 PRO A 456      80.464 122.653 151.613  1.00  0.00           H
ATOM   2004  N   VAL A 457      83.975 121.952 155.158  1.00 30.00           N
ATOM   2005  CA  VAL A 457      84.970 120.911 155.427  1.00 30.00           C
ATOM   2006  C   VAL A 457      84.454 119.865 156.437  1.00 30.00           C
ATOM   2007  O   VAL A 457      84.633 118.670 156.200  1.00 30.00           O
ATOM   2008  CB  VAL A 457      86.306 121.530 155.938  1.00 20.00           C
ATOM   2009  CG1 VAL A 457      87.351 120.508 156.437  1.00 20.00           C
ATOM   2010  CG2 VAL A 457      86.946 122.419 154.854  1.00 20.00           C
ATOM   2011  H   VAL A 457      84.095 122.822 155.655  1.00  0.00           H
ATOM   2012  HA  VAL A 457      85.185 120.387 154.494  1.00  0.00           H
ATOM   2013  HB  VAL A 457      86.076 122.181 156.783  1.00  0.00           H
ATOM   2014 HG11 VAL A 457      88.296 120.998 156.677  1.00  0.00           H
ATOM   2015 HG12 VAL A 457      87.029 119.995 157.343  1.00  0.00           H
ATOM   2016 HG13 VAL A 457      87.558 119.754 155.677  1.00  0.00           H
ATOM   2017 HG21 VAL A 457      87.876 122.866 155.205  1.00  0.00           H
ATOM   2018 HG22 VAL A 457      87.176 121.843 153.957  1.00  0.00           H
ATOM   2019 HG23 VAL A 457      86.288 123.237 154.561  1.00  0.00           H
ATOM   2020  N   GLU A 458      83.820 120.332 157.528  1.00 30.00           N
ATOM   2021  CA  GLU A 458      83.267 119.494 158.593  1.00 30.00           C
ATOM   2022  C   GLU A 458      81.971 118.786 158.155  1.00 30.00           C
ATOM   2023  O   GLU A 458      81.155 119.377 157.448  1.00 30.00           O
ATOM   2024  CB  GLU A 458      83.084 120.358 159.867  1.00  0.00           C
ATOM   2025  CG  GLU A 458      82.415 119.677 161.087  1.00  0.00           C
ATOM   2026  CD  GLU A 458      83.111 118.394 161.555  1.00  0.00           C
ATOM   2027  OE1 GLU A 458      84.361 118.389 161.585  1.00  0.00           O
ATOM   2028  OE2 GLU A 458      82.375 117.425 161.843  1.00  0.00           O1-
ATOM   2029  H   GLU A 458      83.694 121.327 157.640  1.00  0.00           H
ATOM   2030  HA  GLU A 458      84.009 118.724 158.815  1.00  0.00           H
ATOM   2031  HB2 GLU A 458      84.060 120.737 160.175  1.00  0.00           H
ATOM   2032  HB3 GLU A 458      82.501 121.244 159.614  1.00  0.00           H
ATOM   2033  HG2 GLU A 458      82.401 120.373 161.925  1.00  0.00           H
ATOM   2034  HG3 GLU A 458      81.369 119.456 160.867  1.00  0.00           H
ATOM   2035  N   GLY A 459      81.814 117.536 158.620  1.00 30.00           N
ATOM   2036  CA  GLY A 459      80.656 116.684 158.370  1.00 30.00           C
ATOM   2037  C   GLY A 459      80.799 115.919 157.047  1.00 30.00           C
ATOM   2038  O   GLY A 459      81.640 116.234 156.204  1.00 30.00           O
ATOM   2039  H   GLY A 459      82.524 117.156 159.231  1.00  0.00           H
ATOM   2040  HA2 GLY A 459      79.738 117.275 158.356  1.00  0.00           H
ATOM   2041  HA3 GLY A 459      80.567 115.976 159.195  1.00  0.00           H
ATOM   2042  N   LEU A 460      79.922 114.918 156.878  1.00 30.00           N
ATOM   2043  CA  LEU A 460      79.765 114.100 155.678  1.00 30.00           C
ATOM   2044  C   LEU A 460      78.374 114.095 154.983  1.00 30.00           C
ATOM   2045  O   LEU A 460      78.332 113.521 153.894  1.00 30.00           O
ATOM   2046  CB  LEU A 460      80.295 112.666 155.946  1.00 20.00           C
ATOM   2047  CG  LEU A 460      79.413 111.730 156.815  1.00  0.00           C
ATOM   2048  CD1 LEU A 460      79.927 110.275 156.742  1.00  0.00           C
ATOM   2049  CD2 LEU A 460      79.278 112.198 158.279  1.00  0.00           C
```

```
ATOM   2050  H   LEU A 460      79.267 114.718 157.620  1.00  0.00           H
ATOM   2051  HA  LEU A 460      80.423 114.513 154.911  1.00  0.00           H
ATOM   2052  HB2 LEU A 460      80.425 112.190 154.973  1.00  0.00           H
ATOM   2053  HB3 LEU A 460      81.297 112.719 156.373  1.00  0.00           H
ATOM   2054  HG  LEU A 460      78.411 111.713 156.386  1.00  0.00           H
ATOM   2055 HD11 LEU A 460      79.105 109.583 156.557  1.00  0.00           H
ATOM   2056 HD12 LEU A 460      80.651 110.132 155.940  1.00  0.00           H
ATOM   2057 HD13 LEU A 460      80.416 109.960 157.664  1.00  0.00           H
ATOM   2058 HD21 LEU A 460      79.262 111.364 158.981  1.00  0.00           H
ATOM   2059 HD22 LEU A 460      80.099 112.848 158.579  1.00  0.00           H
ATOM   2060 HD23 LEU A 460      78.347 112.745 158.425  1.00  0.00           H
ATOM   2061  N   PRO A 461      77.279 114.721 155.503  1.00 30.00           N
ATOM   2062  CA  PRO A 461      75.987 114.701 154.782  1.00 30.00           C
ATOM   2063  C   PRO A 461      75.926 115.753 153.648  1.00 30.00           C
ATOM   2064  O   PRO A 461      76.828 116.589 153.558  1.00 30.00           O
ATOM   2065  CB  PRO A 461      74.967 114.982 155.901  1.00 20.00           C
ATOM   2066  CG  PRO A 461      75.692 115.923 156.839  1.00  0.00           C
ATOM   2067  CD  PRO A 461      77.131 115.428 156.784  1.00  0.00           C
ATOM   2068  HA  PRO A 461      75.795 113.714 154.362  1.00  0.00           H
ATOM   2069  HB2 PRO A 461      74.026 115.414 155.563  1.00  0.00           H
ATOM   2070  HB3 PRO A 461      74.733 114.049 156.417  1.00  0.00           H
ATOM   2071  HG2 PRO A 461      75.642 116.938 156.441  1.00  0.00           H
ATOM   2072  HG3 PRO A 461      75.278 115.939 157.848  1.00  0.00           H
ATOM   2073  HD2 PRO A 461      77.801 116.283 156.871  1.00  0.00           H
ATOM   2074  HD3 PRO A 461      77.318 114.735 157.604  1.00  0.00           H
ATOM   2075  N   PRO A 462      74.834 115.752 152.845  1.00 30.00           N
ATOM   2076  CA  PRO A 462      74.523 116.874 151.945  1.00 30.00           C
ATOM   2077  C   PRO A 462      74.150 118.133 152.752  1.00 30.00           C
ATOM   2078  O   PRO A 462      73.082 118.171 153.365  1.00 30.00           O
ATOM   2079  CB  PRO A 462      73.373 116.339 151.077  1.00 20.00           C
ATOM   2080  CG  PRO A 462      72.676 115.306 151.948  1.00  0.00           C
ATOM   2081  CD  PRO A 462      73.821 114.698 152.748  1.00  0.00           C
ATOM   2082  HA  PRO A 462      75.383 117.089 151.309  1.00  0.00           H
ATOM   2083  HB2 PRO A 462      72.693 117.114 150.723  1.00  0.00           H
ATOM   2084  HB3 PRO A 462      73.794 115.852 150.196  1.00  0.00           H
ATOM   2085  HG2 PRO A 462      71.986 115.808 152.626  1.00  0.00           H
ATOM   2086  HG3 PRO A 462      72.108 114.567 151.383  1.00  0.00           H
ATOM   2087  HD2 PRO A 462      73.458 114.339 153.708  1.00  0.00           H
ATOM   2088  HD3 PRO A 462      74.253 113.855 152.207  1.00  0.00           H
ATOM   2089  N   TYR A 463      75.088 119.093 152.792  1.00 30.00           N
ATOM   2090  CA  TYR A 463      75.073 120.270 153.665  1.00 30.00           C
ATOM   2091  C   TYR A 463      73.852 121.175 153.441  1.00 30.00           C
ATOM   2092  O   TYR A 463      73.527 121.491 152.296  1.00 30.00           O
ATOM   2093  CB  TYR A 463      76.379 121.071 153.474  1.00 20.00           C
ATOM   2094  CG  TYR A 463      77.657 120.257 153.599  1.00 20.00           C
ATOM   2095  CD1 TYR A 463      78.540 120.161 152.504  1.00 20.00           C
ATOM   2096  CD2 TYR A 463      77.962 119.580 154.800  1.00 20.00           C
ATOM   2097  CE1 TYR A 463      79.710 119.386 152.606  1.00 20.00           C
ATOM   2098  CE2 TYR A 463      79.132 118.801 154.898  1.00 20.00           C
ATOM   2099  CZ  TYR A 463      80.009 118.704 153.800  1.00 20.00           C
ATOM   2100  OH  TYR A 463      81.147 117.956 153.886  1.00 20.00           O
ATOM   2101  H   TYR A 463      75.934 118.969 152.255  1.00  0.00           H
ATOM   2102  HA  TYR A 463      75.044 119.899 154.692  1.00  0.00           H
ATOM   2103  HB2 TYR A 463      76.423 121.886 154.197  1.00  0.00           H
ATOM   2104  HB3 TYR A 463      76.372 121.545 152.490  1.00  0.00           H
ATOM   2105  HD1 TYR A 463      78.323 120.676 151.580  1.00  0.00           H
ATOM   2106  HD2 TYR A 463      77.294 119.644 155.646  1.00  0.00           H
ATOM   2107  HE1 TYR A 463      80.377 119.317 151.763  1.00  0.00           H
ATOM   2108  HE2 TYR A 463      79.350 118.278 155.816  1.00  0.00           H
ATOM   2109  HH  TYR A 463      81.277 117.550 154.749  1.00  0.00           H
ATOM   2110  N   LYS A 464      73.218 121.572 154.555  1.00 30.00           N
ATOM   2111  CA  LYS A 464      72.052 122.450 154.585  1.00 30.00           C
ATOM   2112  C   LYS A 464      72.521 123.821 155.086  1.00 30.00           C
ATOM   2113  O   LYS A 464      72.952 123.934 156.235  1.00 30.00           O
ATOM   2114  CB  LYS A 464      70.968 121.846 155.510  1.00  0.00           C
ATOM   2115  CG  LYS A 464      70.566 120.390 155.195  1.00  0.00           C
ATOM   2116  CD  LYS A 464      70.136 120.138 153.741  1.00  0.00           C
ATOM   2117  CE  LYS A 464      69.678 118.690 153.514  1.00  0.00           C
ATOM   2118  NZ  LYS A 464      69.394 118.435 152.092  1.00  0.00           N1+
ATOM   2119  H   LYS A 464      73.564 121.276 155.457  1.00  0.00           H
ATOM   2120  HA  LYS A 464      71.626 122.570 153.587  1.00  0.00           H
```

```
ATOM   2121  HB2 LYS A 464      71.304 121.876 156.548  1.00  0.00           H
ATOM   2122  HB3 LYS A 464      70.079 122.477 155.469  1.00  0.00           H
ATOM   2123  HG2 LYS A 464      71.394 119.725 155.444  1.00  0.00           H
ATOM   2124  HG3 LYS A 464      69.752 120.105 155.862  1.00  0.00           H
ATOM   2125  HD2 LYS A 464      69.335 120.827 153.471  1.00  0.00           H
ATOM   2126  HD3 LYS A 464      70.966 120.351 153.068  1.00  0.00           H
ATOM   2127  HE2 LYS A 464      70.449 117.992 153.841  1.00  0.00           H
ATOM   2128  HE3 LYS A 464      68.784 118.478 154.101  1.00  0.00           H
ATOM   2129  HZ1 LYS A 464      70.231 118.598 151.550  1.00  0.00           H
ATOM   2130  HZ2 LYS A 464      69.099 117.476 151.974  1.00  0.00           H
ATOM   2131  HZ3 LYS A 464      68.661 119.054 151.777  1.00  0.00           H
ATOM   2132  N   LEU A 465      72.469 124.822 154.196  1.00 30.00           N
ATOM   2133  CA  LEU A 465      73.004 126.160 154.435  1.00 30.00           C
ATOM   2134  C   LEU A 465      71.915 127.097 154.974  1.00 30.00           C
ATOM   2135  O   LEU A 465      70.788 127.090 154.476  1.00 30.00           O
ATOM   2136  CB  LEU A 465      73.599 126.720 153.121  1.00 20.00           C
ATOM   2137  CG  LEU A 465      74.968 126.129 152.694  1.00  0.00           C
ATOM   2138  CD1 LEU A 465      76.085 126.337 153.738  1.00  0.00           C
ATOM   2139  CD2 LEU A 465      74.878 124.671 152.195  1.00  0.00           C
ATOM   2140  H   LEU A 465      72.090 124.655 153.275  1.00  0.00           H
ATOM   2141  HA  LEU A 465      73.796 126.103 155.184  1.00  0.00           H
ATOM   2142  HB2 LEU A 465      72.876 126.607 152.314  1.00  0.00           H
ATOM   2143  HB3 LEU A 465      73.722 127.797 153.230  1.00  0.00           H
ATOM   2144  HG  LEU A 465      75.265 126.723 151.831  1.00  0.00           H
ATOM   2145 HD11 LEU A 465      76.977 126.753 153.268  1.00  0.00           H
ATOM   2146 HD12 LEU A 465      75.790 127.033 154.523  1.00  0.00           H
ATOM   2147 HD13 LEU A 465      76.379 125.407 154.225  1.00  0.00           H
ATOM   2148 HD21 LEU A 465      75.475 124.530 151.296  1.00  0.00           H
ATOM   2149 HD22 LEU A 465      75.233 123.957 152.939  1.00  0.00           H
ATOM   2150 HD23 LEU A 465      73.858 124.390 151.937  1.00  0.00           H
ATOM   2151  N   LYS A 466      72.310 127.916 155.961  1.00 30.00           N
ATOM   2152  CA  LYS A 466      71.508 128.984 156.560  1.00 30.00           C
ATOM   2153  C   LYS A 466      71.426 130.222 155.644  1.00 30.00           C
ATOM   2154  O   LYS A 466      72.192 130.336 154.684  1.00 30.00           O
ATOM   2155  CB  LYS A 466      72.119 129.324 157.940  1.00  0.00           C
ATOM   2156  CG  LYS A 466      73.515 129.979 157.895  1.00  0.00           C
ATOM   2157  CD  LYS A 466      74.139 130.149 159.289  1.00  0.00           C
ATOM   2158  CE  LYS A 466      75.450 130.952 159.287  1.00  0.00           C
ATOM   2159  NZ  LYS A 466      76.499 130.319 158.468  1.00  0.00           N1+
ATOM   2160  H   LYS A 466      73.262 127.855 156.290  1.00  0.00           H
ATOM   2161  HA  LYS A 466      70.494 128.610 156.712  1.00  0.00           H
ATOM   2162  HB2 LYS A 466      71.443 129.993 158.474  1.00  0.00           H
ATOM   2163  HB3 LYS A 466      72.163 128.413 158.540  1.00  0.00           H
ATOM   2164  HG2 LYS A 466      74.177 129.376 157.275  1.00  0.00           H
ATOM   2165  HG3 LYS A 466      73.451 130.956 157.415  1.00  0.00           H
ATOM   2166  HD2 LYS A 466      73.423 130.642 159.947  1.00  0.00           H
ATOM   2167  HD3 LYS A 466      74.319 129.165 159.726  1.00  0.00           H
ATOM   2168  HE2 LYS A 466      75.276 131.962 158.918  1.00  0.00           H
ATOM   2169  HE3 LYS A 466      75.822 131.049 160.307  1.00  0.00           H
ATOM   2170  HZ1 LYS A 466      76.203 130.267 157.496  1.00  0.00           H
ATOM   2171  HZ2 LYS A 466      77.344 130.870 158.515  1.00  0.00           H
ATOM   2172  HZ3 LYS A 466      76.686 129.387 158.807  1.00  0.00           H
ATOM   2173  N   ASN A 467      70.513 131.145 155.991  1.00 30.00           N
ATOM   2174  CA  ASN A 467      70.339 132.435 155.316  1.00 30.00           C
ATOM   2175  C   ASN A 467      71.523 133.367 155.625  1.00 30.00           C
ATOM   2176  O   ASN A 467      71.576 133.966 156.699  1.00 30.00           O
ATOM   2177  CB  ASN A 467      68.994 133.086 155.726  1.00 20.00           C
ATOM   2178  CG  ASN A 467      67.756 132.254 155.375  1.00  0.00           C
ATOM   2179  ND2 ASN A 467      67.602 131.870 154.105  1.00  0.00           N
ATOM   2180  OD1 ASN A 467      66.938 131.967 156.244  1.00  0.00           O
ATOM   2181  H   ASN A 467      69.922 130.983 156.794  1.00  0.00           H
ATOM   2182  HA  ASN A 467      70.317 132.251 154.240  1.00  0.00           H
ATOM   2183  HB2 ASN A 467      68.983 133.281 156.799  1.00  0.00           H
ATOM   2184  HB3 ASN A 467      68.889 134.056 155.238  1.00  0.00           H
ATOM   2185 HD21 ASN A 467      66.797 131.321 153.843  1.00  0.00           H
ATOM   2186 HD22 ASN A 467      68.282 132.120 153.402  1.00  0.00           H
ATOM   2187  N   THR A 468      72.458 133.441 154.669  1.00 30.00           N
ATOM   2188  CA  THR A 468      73.665 134.263 154.702  1.00 30.00           C
ATOM   2189  C   THR A 468      74.064 134.540 153.243  1.00 30.00           C
ATOM   2190  O   THR A 468      73.887 133.671 152.387  1.00 30.00           O
ATOM   2191  CB  THR A 468      74.827 133.526 155.430  1.00  0.00           C
```

ATOM	2192	CG2	THR	A	468	76.244	134.109	155.256	1.00	0.00	C
ATOM	2193	OG1	THR	A	468	74.564	133.506	156.817	1.00	0.00	O
ATOM	2194	H	THR	A	468	72.347	132.886	153.832	1.00	0.00	H
ATOM	2195	HA	THR	A	468	73.448	135.212	155.197	1.00	0.00	H
ATOM	2196	HB	THR	A	468	74.832	132.488	155.108	1.00	0.00	H
ATOM	2197	HG1	THR	A	468	73.673	133.170	156.949	1.00	0.00	H
ATOM	2198	HG21	THR	A	468	76.956	133.607	155.912	1.00	0.00	H
ATOM	2199	HG22	THR	A	468	76.615	133.980	154.240	1.00	0.00	H
ATOM	2200	HG23	THR	A	468	76.267	135.172	155.495	1.00	0.00	H
ATOM	2201	N	VAL	A	469	74.588	135.752	152.993	1.00	30.00	N
ATOM	2202	CA	VAL	A	469	74.952	136.264	151.667	1.00	30.00	C
ATOM	2203	C	VAL	A	469	75.984	135.406	150.897	1.00	30.00	C
ATOM	2204	O	VAL	A	469	75.854	135.277	149.681	1.00	30.00	O
ATOM	2205	CB	VAL	A	469	75.442	137.741	151.762	1.00	0.00	C
ATOM	2206	CG1	VAL	A	469	76.742	137.926	152.573	1.00	0.00	C
ATOM	2207	CG2	VAL	A	469	75.575	138.426	150.389	1.00	0.00	C
ATOM	2208	H	VAL	A	469	74.700	136.404	153.756	1.00	0.00	H
ATOM	2209	HA	VAL	A	469	74.033	136.257	151.078	1.00	0.00	H
ATOM	2210	HB	VAL	A	469	74.663	138.286	152.298	1.00	0.00	H
ATOM	2211	HG11	VAL	A	469	76.963	138.984	152.713	1.00	0.00	H
ATOM	2212	HG12	VAL	A	469	76.664	137.480	153.565	1.00	0.00	H
ATOM	2213	HG13	VAL	A	469	77.603	137.482	152.074	1.00	0.00	H
ATOM	2214	HG21	VAL	A	469	75.784	139.490	150.503	1.00	0.00	H
ATOM	2215	HG22	VAL	A	469	76.383	138.000	149.794	1.00	0.00	H
ATOM	2216	HG23	VAL	A	469	74.653	138.335	149.814	1.00	0.00	H
ATOM	2217	N	GLY	A	470	76.949	134.804	151.613	1.00	30.00	N
ATOM	2218	CA	GLY	A	470	77.963	133.927	151.031	1.00	30.00	C
ATOM	2219	C	GLY	A	470	77.439	132.490	150.896	1.00	30.00	C
ATOM	2220	O	GLY	A	470	77.715	131.847	149.883	1.00	30.00	O
ATOM	2221	H	GLY	A	470	76.988	134.953	152.611	1.00	0.00	H
ATOM	2222	HA2	GLY	A	470	78.295	134.302	150.061	1.00	0.00	H
ATOM	2223	HA3	GLY	A	470	78.836	133.920	151.683	1.00	0.00	H
ATOM	2224	N	ASP	A	471	76.687	131.996	151.898	1.00	30.00	N
ATOM	2225	CA	ASP	A	471	76.179	130.618	151.969	1.00	30.00	C
ATOM	2226	C	ASP	A	471	75.124	130.301	150.895	1.00	30.00	C
ATOM	2227	O	ASP	A	471	75.177	129.219	150.313	1.00	30.00	O
ATOM	2228	CB	ASP	A	471	75.654	130.191	153.360	1.00	30.00	C
ATOM	2229	CG	ASP	A	471	76.589	130.413	154.555	1.00	0.00	C
ATOM	2230	OD1	ASP	A	471	77.746	130.842	154.354	1.00	0.00	O
ATOM	2231	OD2	ASP	A	471	76.122	130.119	155.677	1.00	0.00	O1-
ATOM	2232	H	ASP	A	471	76.507	132.569	152.709	1.00	0.00	H
ATOM	2233	HA	ASP	A	471	77.035	129.973	151.770	1.00	0.00	H
ATOM	2234	HB2	ASP	A	471	74.717	130.713	153.559	1.00	0.00	H
ATOM	2235	HB3	ASP	A	471	75.447	129.125	153.327	1.00	0.00	H
ATOM	2236	N	TYR	A	472	74.210	131.251	150.632	1.00	0.00	N
ATOM	2237	CA	TYR	A	472	73.204	131.149	149.573	1.00	0.00	C
ATOM	2238	C	TYR	A	472	73.815	131.261	148.163	1.00	0.00	C
ATOM	2239	O	TYR	A	472	73.363	130.559	147.257	1.00	0.00	O
ATOM	2240	CB	TYR	A	472	72.097	132.201	149.808	1.00	20.00	C
ATOM	2241	CG	TYR	A	472	71.011	132.233	148.742	1.00	0.00	C
ATOM	2242	CD1	TYR	A	472	70.052	131.200	148.684	1.00	0.00	C
ATOM	2243	CD2	TYR	A	472	70.979	133.269	147.784	1.00	0.00	C
ATOM	2244	CE1	TYR	A	472	69.075	131.196	147.669	1.00	0.00	C
ATOM	2245	CE2	TYR	A	472	70.002	133.265	146.770	1.00	0.00	C
ATOM	2246	CZ	TYR	A	472	69.052	132.228	146.709	1.00	0.00	C
ATOM	2247	OH	TYR	A	472	68.112	132.223	145.721	1.00	0.00	O
ATOM	2248	H	TYR	A	472	74.219	132.113	151.163	1.00	0.00	H
ATOM	2249	HA	TYR	A	472	72.743	130.162	149.651	1.00	0.00	H
ATOM	2250	HB2	TYR	A	472	71.622	132.022	150.773	1.00	0.00	H
ATOM	2251	HB3	TYR	A	472	72.544	133.194	149.876	1.00	0.00	H
ATOM	2252	HD1	TYR	A	472	70.073	130.399	149.409	1.00	0.00	H
ATOM	2253	HD2	TYR	A	472	71.713	134.061	147.814	1.00	0.00	H
ATOM	2254	HE1	TYR	A	472	68.350	130.397	147.628	1.00	0.00	H
ATOM	2255	HE2	TYR	A	472	69.988	134.056	146.034	1.00	0.00	H
ATOM	2256	HH	TYR	A	472	67.522	131.467	145.762	1.00	0.00	H
ATOM	2257	N	PHE	A	473	74.830	132.130	148.008	1.00	0.00	N
ATOM	2258	CA	PHE	A	473	75.510	132.379	146.738	1.00	0.00	C
ATOM	2259	C	PHE	A	473	76.352	131.184	146.251	1.00	0.00	C
ATOM	2260	O	PHE	A	473	76.365	130.928	145.048	1.00	0.00	O
ATOM	2261	CB	PHE	A	473	76.318	133.691	146.834	1.00	20.00	C
ATOM	2262	CG	PHE	A	473	77.018	134.124	145.557	1.00	0.00	C

```
ATOM   2263  CD1 PHE A 473      76.278 134.738 144.525  1.00  0.00           C
ATOM   2264  CD2 PHE A 473      78.365 133.772 145.319  1.00  0.00           C
ATOM   2265  CE1 PHE A 473      76.891 135.044 143.317  1.00  0.00           C
ATOM   2266  CE2 PHE A 473      78.959 134.086 144.104  1.00  0.00           C
ATOM   2267  CZ  PHE A 473      78.226 134.724 143.110  1.00  0.00           C
ATOM   2268  H   PHE A 473      75.152 132.668 148.801  1.00  0.00           H
ATOM   2269  HA  PHE A 473      74.732 132.539 145.989  1.00  0.00           H
ATOM   2270  HB2 PHE A 473      75.638 134.498 147.110  1.00  0.00           H
ATOM   2271  HB3 PHE A 473      77.049 133.627 147.642  1.00  0.00           H
ATOM   2272  HD1 PHE A 473      75.233 134.971 144.670  1.00  0.00           H
ATOM   2273  HD2 PHE A 473      78.934 133.256 146.079  1.00  0.00           H
ATOM   2274  HE1 PHE A 473      76.324 135.524 142.533  1.00  0.00           H
ATOM   2275  HE2 PHE A 473      79.992 133.825 143.927  1.00  0.00           H
ATOM   2276  HZ  PHE A 473      78.695 134.960 142.166  1.00  0.00           H
ATOM   2277  N   ARG A 474      77.002 130.455 147.179  1.00 30.00           N
ATOM   2278  CA  ARG A 474      77.761 129.244 146.853  1.00 30.00           C
ATOM   2279  C   ARG A 474      76.865 128.018 146.586  1.00 30.00           C
ATOM   2280  O   ARG A 474      77.301 127.132 145.857  1.00 30.00           O
ATOM   2281  CB  ARG A 474      78.841 128.957 147.919  1.00 20.00           C
ATOM   2282  CG  ARG A 474      78.344 128.428 149.282  1.00 20.00           C
ATOM   2283  CD  ARG A 474      79.430 127.695 150.082  1.00 20.00           C
ATOM   2284  NE  ARG A 474      79.800 126.425 149.437  1.00 20.00           N
ATOM   2285  CZ  ARG A 474      79.100 125.279 149.474  1.00 20.00           C
ATOM   2286  NH1 ARG A 474      77.991 125.160 150.218  1.00 20.00           N
ATOM   2287  NH2 ARG A 474      79.520 124.238 148.744  1.00 20.00           N1+
ATOM   2288  H   ARG A 474      76.971 130.728 148.152  1.00  0.00           H
ATOM   2289  HA  ARG A 474      78.298 129.436 145.922  1.00  0.00           H
ATOM   2290  HB2 ARG A 474      79.456 129.845 148.077  1.00  0.00           H
ATOM   2291  HB3 ARG A 474      79.514 128.217 147.483  1.00  0.00           H
ATOM   2292  HG2 ARG A 474      77.845 129.192 149.870  1.00  0.00           H
ATOM   2293  HG3 ARG A 474      77.582 127.674 149.094  1.00  0.00           H
ATOM   2294  HD2 ARG A 474      80.339 128.293 150.015  1.00  0.00           H
ATOM   2295  HD3 ARG A 474      79.203 127.608 151.145  1.00  0.00           H
ATOM   2296  HE  ARG A 474      80.627 126.457 148.858  1.00  0.00           H
ATOM   2297 HH11 ARG A 474      77.469 124.296 150.232  1.00  0.00           H
ATOM   2298 HH12 ARG A 474      77.671 125.943 150.770  1.00  0.00           H
ATOM   2299 HH21 ARG A 474      79.004 123.369 148.738  1.00  0.00           H
ATOM   2300 HH22 ARG A 474      80.334 124.325 148.149  1.00  0.00           H
ATOM   2301  N   VAL A 475      75.646 127.985 147.158  1.00  0.00           N
ATOM   2302  CA  VAL A 475      74.630 126.966 146.871  1.00  0.00           C
ATOM   2303  C   VAL A 475      74.040 127.145 145.462  1.00  0.00           C
ATOM   2304  O   VAL A 475      73.874 126.152 144.757  1.00  0.00           O
ATOM   2305  CB  VAL A 475      73.499 126.963 147.943  1.00 20.00           C
ATOM   2306  CG1 VAL A 475      72.158 126.316 147.531  1.00 20.00           C
ATOM   2307  CG2 VAL A 475      74.007 126.290 149.225  1.00 20.00           C
ATOM   2308  H   VAL A 475      75.362 128.737 147.771  1.00  0.00           H
ATOM   2309  HA  VAL A 475      75.121 125.990 146.893  1.00  0.00           H
ATOM   2310  HB  VAL A 475      73.280 128.000 148.200  1.00  0.00           H
ATOM   2311 HG11 VAL A 475      71.471 126.270 148.376  1.00  0.00           H
ATOM   2312 HG12 VAL A 475      71.650 126.885 146.751  1.00  0.00           H
ATOM   2313 HG13 VAL A 475      72.302 125.298 147.168  1.00  0.00           H
ATOM   2314 HG21 VAL A 475      73.288 126.401 150.036  1.00  0.00           H
ATOM   2315 HG22 VAL A 475      74.177 125.223 149.073  1.00  0.00           H
ATOM   2316 HG23 VAL A 475      74.950 126.725 149.553  1.00  0.00           H
ATOM   2317  N   THR A 476      73.805 128.407 145.064  1.00  0.00           N
ATOM   2318  CA  THR A 476      73.432 128.793 143.702  1.00  0.00           C
ATOM   2319  C   THR A 476      74.552 128.477 142.686  1.00  0.00           C
ATOM   2320  O   THR A 476      74.247 128.016 141.587  1.00  0.00           O
ATOM   2321  CB  THR A 476      73.096 130.309 143.629  1.00 20.00           C
ATOM   2322  CG2 THR A 476      72.743 130.850 142.231  1.00  0.00           C
ATOM   2323  OG1 THR A 476      72.005 130.585 144.486  1.00  0.00           O
ATOM   2324  H   THR A 476      73.962 129.165 145.714  1.00  0.00           H
ATOM   2325  HA  THR A 476      72.546 128.221 143.419  1.00  0.00           H
ATOM   2326  HB  THR A 476      73.940 130.885 144.008  1.00  0.00           H
ATOM   2327  HG1 THR A 476      72.283 130.447 145.396  1.00  0.00           H
ATOM   2328 HG21 THR A 476      72.392 131.881 142.290  1.00  0.00           H
ATOM   2329 HG22 THR A 476      73.607 130.849 141.568  1.00  0.00           H
ATOM   2330 HG23 THR A 476      71.956 130.258 141.762  1.00  0.00           H
ATOM   2331  N   GLY A 477      75.819 128.675 143.093  1.00  0.00           N
ATOM   2332  CA  GLY A 477      77.004 128.379 142.292  1.00  0.00           C
ATOM   2333  C   GLY A 477      77.236 126.867 142.139  1.00  0.00           C
```

```
ATOM   2334  O   GLY A 477      77.661 126.438 141.069  1.00  0.00           O
ATOM   2335  H   GLY A 477      75.982 129.070 144.010  1.00  0.00           H
ATOM   2336  HA2 GLY A 477      76.910 128.844 141.310  1.00  0.00           H
ATOM   2337  HA3 GLY A 477      77.876 128.822 142.775  1.00  0.00           H
ATOM   2338  N   GLU A 478      76.931 126.060 143.172  1.00  0.00           N
ATOM   2339  CA  GLU A 478      77.041 124.599 143.144  1.00  0.00           C
ATOM   2340  C   GLU A 478      75.929 123.945 142.302  1.00  0.00           C
ATOM   2341  O   GLU A 478      76.202 122.966 141.612  1.00  0.00           O
ATOM   2342  CB  GLU A 478      77.093 124.052 144.590  1.00 20.00           C
ATOM   2343  CG  GLU A 478      77.306 122.526 144.726  1.00  0.00           C
ATOM   2344  CD  GLU A 478      78.626 122.043 144.114  1.00  0.00           C
ATOM   2345  OE1 GLU A 478      79.675 122.596 144.511  1.00  0.00           O
ATOM   2346  OE2 GLU A 478      78.561 121.136 143.256  1.00  0.00           O1-
ATOM   2347  H   GLU A 478      76.614 126.464 144.043  1.00  0.00           H
ATOM   2348  HA  GLU A 478      77.994 124.362 142.667  1.00  0.00           H
ATOM   2349  HB2 GLU A 478      77.888 124.562 145.135  1.00  0.00           H
ATOM   2350  HB3 GLU A 478      76.171 124.321 145.108  1.00  0.00           H
ATOM   2351  HG2 GLU A 478      77.311 122.254 145.781  1.00  0.00           H
ATOM   2352  HG3 GLU A 478      76.475 121.979 144.280  1.00  0.00           H
ATOM   2353  N   ILE A 479      74.715 124.521 142.325  1.00  0.00           N
ATOM   2354  CA  ILE A 479      73.594 124.129 141.466  1.00  0.00           C
ATOM   2355  C   ILE A 479      73.882 124.385 139.970  1.00  0.00           C
ATOM   2356  O   ILE A 479      73.551 123.532 139.148  1.00  0.00           O
ATOM   2357  CB  ILE A 479      72.270 124.825 141.913  1.00 20.00           C
ATOM   2358  CG1 ILE A 479      71.762 124.197 143.234  1.00  0.00           C
ATOM   2359  CG2 ILE A 479      71.128 124.842 140.870  1.00  0.00           C
ATOM   2360  CD1 ILE A 479      70.767 125.076 144.007  1.00  0.00           C
ATOM   2361  H   ILE A 479      74.552 125.310 142.937  1.00  0.00           H
ATOM   2362  HA  ILE A 479      73.462 123.050 141.581  1.00  0.00           H
ATOM   2363  HB  ILE A 479      72.516 125.867 142.124  1.00  0.00           H
ATOM   2364 HG12 ILE A 479      71.303 123.231 143.023  1.00  0.00           H
ATOM   2365 HG13 ILE A 479      72.600 123.978 143.896  1.00  0.00           H
ATOM   2366 HG21 ILE A 479      70.226 125.299 141.276  1.00  0.00           H
ATOM   2367 HG22 ILE A 479      71.387 125.423 139.984  1.00  0.00           H
ATOM   2368 HG23 ILE A 479      70.871 123.833 140.549  1.00  0.00           H
ATOM   2369 HD11 ILE A 479      70.756 124.808 145.063  1.00  0.00           H
ATOM   2370 HD12 ILE A 479      71.026 126.133 143.941  1.00  0.00           H
ATOM   2371 HD13 ILE A 479      69.753 124.954 143.626  1.00  0.00           H
ATOM   2372  N   LEU A 480      74.554 125.509 139.659  1.00  0.00           N
ATOM   2373  CA  LEU A 480      75.056 125.844 138.323  1.00  0.00           C
ATOM   2374  C   LEU A 480      76.209 124.931 137.862  1.00  0.00           C
ATOM   2375  O   LEU A 480      76.294 124.646 136.667  1.00  0.00           O
ATOM   2376  CB  LEU A 480      75.500 127.325 138.299  1.00 20.00           C
ATOM   2377  CG  LEU A 480      74.334 128.337 138.273  1.00 20.00           C
ATOM   2378  CD1 LEU A 480      74.827 129.762 138.605  1.00 20.00           C
ATOM   2379  CD2 LEU A 480      73.541 128.282 136.950  1.00 20.00           C
ATOM   2380  H   LEU A 480      74.780 126.166 140.394  1.00  0.00           H
ATOM   2381  HA  LEU A 480      74.239 125.706 137.614  1.00  0.00           H
ATOM   2382  HB2 LEU A 480      76.128 127.509 139.171  1.00  0.00           H
ATOM   2383  HB3 LEU A 480      76.139 127.518 137.436  1.00  0.00           H
ATOM   2384  HG  LEU A 480      73.641 128.059 139.067  1.00  0.00           H
ATOM   2385 HD11 LEU A 480      74.200 130.214 139.373  1.00  0.00           H
ATOM   2386 HD12 LEU A 480      75.849 129.768 138.985  1.00  0.00           H
ATOM   2387 HD13 LEU A 480      74.810 130.425 137.741  1.00  0.00           H
ATOM   2388 HD21 LEU A 480      73.392 129.266 136.505  1.00  0.00           H
ATOM   2389 HD22 LEU A 480      74.037 127.666 136.200  1.00  0.00           H
ATOM   2390 HD23 LEU A 480      72.551 127.856 137.114  1.00  0.00           H
ATOM   2391  N   SER A 481      77.053 124.474 138.805  1.00  0.00           N
ATOM   2392  CA  SER A 481      78.172 123.560 138.550  1.00  0.00           C
ATOM   2393  C   SER A 481      77.691 122.152 138.163  1.00  0.00           C
ATOM   2394  O   SER A 481      78.232 121.557 137.230  1.00  0.00           O
ATOM   2395  CB  SER A 481      79.091 123.487 139.780  1.00 20.00           C
ATOM   2396  OG  SER A 481      79.678 124.746 140.011  1.00 20.00           O
ATOM   2397  H   SER A 481      76.923 124.760 139.766  1.00  0.00           H
ATOM   2398  HA  SER A 481      78.758 123.963 137.726  1.00  0.00           H
ATOM   2399  HB2 SER A 481      78.564 123.158 140.673  1.00  0.00           H
ATOM   2400  HB3 SER A 481      79.895 122.771 139.609  1.00  0.00           H
ATOM   2401  HG  SER A 481      79.003 125.344 140.349  1.00  0.00           H
ATOM   2402  N   VAL A 482      76.656 121.672 138.872  1.00  0.00           N
ATOM   2403  CA  VAL A 482      75.976 120.404 138.619  1.00  0.00           C
ATOM   2404  C   VAL A 482      75.147 120.430 137.320  1.00  0.00           C
```

```
ATOM   2405  O   VAL A 482      75.129 119.422 136.618  1.00  0.00           O
ATOM   2406  CB  VAL A 482      75.079 120.011 139.830  1.00 20.00           C
ATOM   2407  CG1 VAL A 482      74.052 118.887 139.567  1.00 20.00           C
ATOM   2408  CG2 VAL A 482      75.955 119.640 141.040  1.00 20.00           C
ATOM   2409  H   VAL A 482      76.291 122.219 139.641  1.00  0.00           H
ATOM   2410  HA  VAL A 482      76.742 119.634 138.497  1.00  0.00           H
ATOM   2411  HB  VAL A 482      74.504 120.892 140.122  1.00  0.00           H
ATOM   2412 HG11 VAL A 482      73.575 118.563 140.492  1.00  0.00           H
ATOM   2413 HG12 VAL A 482      73.253 119.214 138.901  1.00  0.00           H
ATOM   2414 HG13 VAL A 482      74.526 118.015 139.115  1.00  0.00           H
ATOM   2415 HG21 VAL A 482      75.349 119.388 141.911  1.00  0.00           H
ATOM   2416 HG22 VAL A 482      76.594 118.787 140.814  1.00  0.00           H
ATOM   2417 HG23 VAL A 482      76.612 120.456 141.333  1.00  0.00           H
ATOM   2418  N   SER A 483      74.519 121.577 137.003  1.00  0.00           N
ATOM   2419  CA  SER A 483      73.732 121.790 135.784  1.00  0.00           C
ATOM   2420  C   SER A 483      74.571 121.720 134.490  1.00  0.00           C
ATOM   2421  O   SER A 483      74.057 121.249 133.475  1.00  0.00           O
ATOM   2422  CB  SER A 483      72.962 123.122 135.907  1.00 20.00           C
ATOM   2423  OG  SER A 483      72.106 123.353 134.805  1.00 20.00           O
ATOM   2424  H   SER A 483      74.567 122.360 137.642  1.00  0.00           H
ATOM   2425  HA  SER A 483      72.996 120.985 135.735  1.00  0.00           H
ATOM   2426  HB2 SER A 483      72.348 123.120 136.807  1.00  0.00           H
ATOM   2427  HB3 SER A 483      73.653 123.961 135.994  1.00  0.00           H
ATOM   2428  HG  SER A 483      72.642 123.489 134.020  1.00  0.00           H
ATOM   2429  N   GLY A 484      75.845 122.148 134.551  1.00  0.00           N
ATOM   2430  CA  GLY A 484      76.779 122.039 133.431  1.00  0.00           C
ATOM   2431  C   GLY A 484      77.360 120.619 133.324  1.00  0.00           C
ATOM   2432  O   GLY A 484      77.636 120.170 132.214  1.00  0.00           O
ATOM   2433  H   GLY A 484      76.198 122.538 135.414  1.00  0.00           H
ATOM   2434  HA2 GLY A 484      76.287 122.316 132.498  1.00  0.00           H
ATOM   2435  HA3 GLY A 484      77.591 122.748 133.563  1.00  0.00           H
ATOM   2436  N   GLY A 485      77.491 119.892 134.449  1.00  0.00           N
ATOM   2437  CA  GLY A 485      77.910 118.486 134.476  1.00  0.00           C
ATOM   2438  C   GLY A 485      76.794 117.543 133.993  1.00  0.00           C
ATOM   2439  O   GLY A 485      77.088 116.432 133.559  1.00  0.00           O
ATOM   2440  H   GLY A 485      77.245 120.309 135.336  1.00  0.00           H
ATOM   2441  HA2 GLY A 485      78.805 118.344 133.868  1.00  0.00           H
ATOM   2442  HA3 GLY A 485      78.170 118.213 135.495  1.00  0.00           H
ATOM   2443  N   VAL A 486      75.529 117.992 134.030  1.00  0.00           N
ATOM   2444  CA  VAL A 486      74.387 117.343 133.390  1.00  0.00           C
ATOM   2445  C   VAL A 486      74.357 117.651 131.877  1.00  0.00           C
ATOM   2446  O   VAL A 486      74.061 116.749 131.095  1.00  0.00           O
ATOM   2447  CB  VAL A 486      73.055 117.783 134.073  1.00 20.00           C
ATOM   2448  CG1 VAL A 486      71.762 117.536 133.268  1.00 20.00           C
ATOM   2449  CG2 VAL A 486      72.916 117.118 135.454  1.00 20.00           C
ATOM   2450  H   VAL A 486      75.344 118.890 134.455  1.00  0.00           H
ATOM   2451  HA  VAL A 486      74.488 116.262 133.505  1.00  0.00           H
ATOM   2452  HB  VAL A 486      73.111 118.857 134.249  1.00  0.00           H
ATOM   2453 HG11 VAL A 486      70.882 117.806 133.852  1.00  0.00           H
ATOM   2454 HG12 VAL A 486      71.721 118.127 132.353  1.00  0.00           H
ATOM   2455 HG13 VAL A 486      71.667 116.485 133.001  1.00  0.00           H
ATOM   2456 HG21 VAL A 486      72.054 117.507 135.996  1.00  0.00           H
ATOM   2457 HG22 VAL A 486      72.787 116.039 135.358  1.00  0.00           H
ATOM   2458 HG23 VAL A 486      73.793 117.285 136.078  1.00  0.00           H
ATOM   2459  N   TYR A 487      74.702 118.894 131.488  1.00  0.00           N
ATOM   2460  CA  TYR A 487      74.743 119.347 130.095  1.00  0.00           C
ATOM   2461  C   TYR A 487      75.766 118.584 129.233  1.00  0.00           C
ATOM   2462  O   TYR A 487      75.393 118.112 128.161  1.00  0.00           O
ATOM   2463  CB  TYR A 487      74.956 120.875 130.038  1.00 20.00           C
ATOM   2464  CG  TYR A 487      75.012 121.460 128.637  1.00 20.00           C
ATOM   2465  CD1 TYR A 487      73.818 121.656 127.913  1.00 20.00           C
ATOM   2466  CD2 TYR A 487      76.252 121.783 128.044  1.00 20.00           C
ATOM   2467  CE1 TYR A 487      73.862 122.166 126.602  1.00 20.00           C
ATOM   2468  CE2 TYR A 487      76.295 122.291 126.732  1.00 20.00           C
ATOM   2469  CZ  TYR A 487      75.100 122.483 126.010  1.00 20.00           C
ATOM   2470  OH  TYR A 487      75.140 122.973 124.738  1.00 20.00           O
ATOM   2471  H   TYR A 487      74.931 119.586 132.188  1.00  0.00           H
ATOM   2472  HA  TYR A 487      73.756 119.143 129.675  1.00  0.00           H
ATOM   2473  HB2 TYR A 487      74.158 121.378 130.586  1.00  0.00           H
ATOM   2474  HB3 TYR A 487      75.881 121.138 130.549  1.00  0.00           H
ATOM   2475  HD1 TYR A 487      72.866 121.406 128.359  1.00  0.00           H
```

```
ATOM   2476  HD2 TYR A 487      77.173 121.630 128.589  1.00  0.00           H
ATOM   2477  HE1 TYR A 487      72.942 122.309 126.053  1.00  0.00           H
ATOM   2478  HE2 TYR A 487      77.247 122.531 126.281  1.00  0.00           H
ATOM   2479  HH  TYR A 487      74.266 123.104 124.363  1.00  0.00           H
ATOM   2480  N   PHE A 488      77.013 118.451 129.722  1.00  0.00           N
ATOM   2481  CA  PHE A 488      78.086 117.709 129.048  1.00  0.00           C
ATOM   2482  C   PHE A 488      77.871 116.185 128.997  1.00  0.00           C
ATOM   2483  O   PHE A 488      78.450 115.534 128.128  1.00  0.00           O
ATOM   2484  CB  PHE A 488      79.452 118.052 129.682  1.00 20.00           C
ATOM   2485  CG  PHE A 488      79.926 119.474 129.445  1.00 20.00           C
ATOM   2486  CD1 PHE A 488      80.087 119.961 128.131  1.00 20.00           C
ATOM   2487  CD2 PHE A 488      80.229 120.319 130.534  1.00 20.00           C
ATOM   2488  CE1 PHE A 488      80.489 121.273 127.923  1.00 20.00           C
ATOM   2489  CE2 PHE A 488      80.619 121.630 130.305  1.00 20.00           C
ATOM   2490  CZ  PHE A 488      80.741 122.105 129.005  1.00 20.00           C
ATOM   2491  H   PHE A 488      77.251 118.881 130.607  1.00  0.00           H
ATOM   2492  HA  PHE A 488      78.102 118.033 128.006  1.00  0.00           H
ATOM   2493  HB2 PHE A 488      79.403 117.876 130.757  1.00  0.00           H
ATOM   2494  HB3 PHE A 488      80.226 117.386 129.298  1.00  0.00           H
ATOM   2495  HD1 PHE A 488      79.890 119.324 127.281  1.00  0.00           H
ATOM   2496  HD2 PHE A 488      80.142 119.959 131.549  1.00  0.00           H
ATOM   2497  HE1 PHE A 488      80.601 121.648 126.917  1.00  0.00           H
ATOM   2498  HE2 PHE A 488      80.824 122.283 131.140  1.00  0.00           H
ATOM   2499  HZ  PHE A 488      81.038 123.126 128.826  1.00  0.00           H
ATOM   2500  N   PHE A 489      77.033 115.653 129.903  1.00  0.00           N
ATOM   2501  CA  PHE A 489      76.640 114.247 129.943  1.00  0.00           C
ATOM   2502  C   PHE A 489      75.644 113.901 128.829  1.00  0.00           C
ATOM   2503  O   PHE A 489      75.906 112.976 128.061  1.00  0.00           O
ATOM   2504  CB  PHE A 489      76.126 113.907 131.355  1.00 20.00           C
ATOM   2505  CG  PHE A 489      75.675 112.477 131.583  1.00 20.00           C
ATOM   2506  CD1 PHE A 489      76.633 111.458 131.773  1.00 20.00           C
ATOM   2507  CD2 PHE A 489      74.308 112.134 131.508  1.00 20.00           C
ATOM   2508  CE1 PHE A 489      76.219 110.146 131.959  1.00 20.00           C
ATOM   2509  CE2 PHE A 489      73.916 110.816 131.699  1.00 20.00           C
ATOM   2510  CZ  PHE A 489      74.866 109.830 131.935  1.00 20.00           C
ATOM   2511  H   PHE A 489      76.596 116.254 130.586  1.00  0.00           H
ATOM   2512  HA  PHE A 489      77.527 113.639 129.766  1.00  0.00           H
ATOM   2513  HB2 PHE A 489      76.924 114.108 132.067  1.00  0.00           H
ATOM   2514  HB3 PHE A 489      75.314 114.575 131.637  1.00  0.00           H
ATOM   2515  HD1 PHE A 489      77.686 111.697 131.792  1.00  0.00           H
ATOM   2516  HD2 PHE A 489      73.564 112.894 131.317  1.00  0.00           H
ATOM   2517  HE1 PHE A 489      76.952 109.370 132.122  1.00  0.00           H
ATOM   2518  HE2 PHE A 489      72.868 110.557 131.659  1.00  0.00           H
ATOM   2519  HZ  PHE A 489      74.552 108.806 132.079  1.00  0.00           H
ATOM   2520  N   PHE A 490      74.549 114.678 128.737  1.00  0.00           N
ATOM   2521  CA  PHE A 490      73.539 114.532 127.689  1.00  0.00           C
ATOM   2522  C   PHE A 490      74.011 114.958 126.288  1.00  0.00           C
ATOM   2523  O   PHE A 490      73.556 114.354 125.320  1.00  0.00           O
ATOM   2524  CB  PHE A 490      72.221 115.229 128.088  1.00 20.00           C
ATOM   2525  CG  PHE A 490      71.397 114.458 129.106  1.00 20.00           C
ATOM   2526  CD1 PHE A 490      70.767 113.251 128.734  1.00 20.00           C
ATOM   2527  CD2 PHE A 490      71.334 114.875 130.451  1.00 20.00           C
ATOM   2528  CE1 PHE A 490      70.074 112.508 129.681  1.00 20.00           C
ATOM   2529  CE2 PHE A 490      70.636 114.119 131.383  1.00 20.00           C
ATOM   2530  CZ  PHE A 490      70.007 112.941 130.999  1.00 20.00           C
ATOM   2531  H   PHE A 490      74.401 115.420 129.407  1.00  0.00           H
ATOM   2532  HA  PHE A 490      73.325 113.465 127.602  1.00  0.00           H
ATOM   2533  HB2 PHE A 490      72.424 116.231 128.468  1.00  0.00           H
ATOM   2534  HB3 PHE A 490      71.592 115.365 127.207  1.00  0.00           H
ATOM   2535  HD1 PHE A 490      70.826 112.898 127.715  1.00  0.00           H
ATOM   2536  HD2 PHE A 490      71.815 115.788 130.759  1.00  0.00           H
ATOM   2537  HE1 PHE A 490      69.590 111.586 129.393  1.00  0.00           H
ATOM   2538  HE2 PHE A 490      70.583 114.443 132.412  1.00  0.00           H
ATOM   2539  HZ  PHE A 490      69.468 112.357 131.730  1.00  0.00           H
ATOM   2540  N   ARG A 491      74.923 115.945 126.193  1.00  0.00           N
ATOM   2541  CA  ARG A 491      75.539 116.363 124.929  1.00  0.00           C
ATOM   2542  C   ARG A 491      76.588 115.354 124.420  1.00  0.00           C
ATOM   2543  O   ARG A 491      76.696 115.173 123.207  1.00  0.00           O
ATOM   2544  CB  ARG A 491      76.102 117.797 125.049  1.00 20.00           C
ATOM   2545  CG  ARG A 491      76.662 118.354 123.725  1.00 20.00           C
ATOM   2546  CD  ARG A 491      76.962 119.859 123.755  1.00 20.00           C
```

```
ATOM   2547  NE  ARG A 491      77.672 120.271 122.535  1.00 20.00           N
ATOM   2548  CZ  ARG A 491      77.816 121.525 122.071  1.00 20.00           C
ATOM   2549  NH1 ARG A 491      78.499 121.724 120.937  1.00 20.00           N
ATOM   2550  NH2 ARG A 491      77.295 122.579 122.713  1.00 20.00           N1+
ATOM   2551  H   ARG A 491      75.237 116.424 127.027  1.00  0.00           H
ATOM   2552  HA  ARG A 491      74.745 116.407 124.182  1.00  0.00           H
ATOM   2553  HB2 ARG A 491      75.307 118.458 125.398  1.00  0.00           H
ATOM   2554  HB3 ARG A 491      76.883 117.829 125.810  1.00  0.00           H
ATOM   2555  HG2 ARG A 491      77.516 117.790 123.349  1.00  0.00           H
ATOM   2556  HG3 ARG A 491      75.865 118.214 122.992  1.00  0.00           H
ATOM   2557  HD2 ARG A 491      76.002 120.372 123.689  1.00  0.00           H
ATOM   2558  HD3 ARG A 491      77.433 120.192 124.680  1.00  0.00           H
ATOM   2559  HE  ARG A 491      78.134 119.525 122.033  1.00  0.00           H
ATOM   2560 HH11 ARG A 491      78.621 122.657 120.569  1.00  0.00           H
ATOM   2561 HH12 ARG A 491      78.905 120.944 120.439  1.00  0.00           H
ATOM   2562 HH21 ARG A 491      77.412 123.512 122.347  1.00  0.00           H
ATOM   2563 HH22 ARG A 491      76.748 122.448 123.555  1.00  0.00           H
ATOM   2564  N   GLY A 492      77.307 114.690 125.344  1.00  0.00           N
ATOM   2565  CA  GLY A 492      78.273 113.637 125.029  1.00  0.00           C
ATOM   2566  C   GLY A 492      77.553 112.375 124.530  1.00  0.00           C
ATOM   2567  O   GLY A 492      77.985 111.783 123.544  1.00  0.00           O
ATOM   2568  H   GLY A 492      77.172 114.902 126.323  1.00  0.00           H
ATOM   2569  HA2 GLY A 492      78.985 113.987 124.280  1.00  0.00           H
ATOM   2570  HA3 GLY A 492      78.843 113.395 125.925  1.00  0.00           H
ATOM   2571  N   ILE A 493      76.429 112.003 125.171  1.00 30.00           N
ATOM   2572  CA  ILE A 493      75.545 110.906 124.760  1.00 30.00           C
ATOM   2573  C   ILE A 493      74.828 111.178 123.423  1.00 30.00           C
ATOM   2574  O   ILE A 493      74.725 110.259 122.611  1.00 30.00           O
ATOM   2575  CB  ILE A 493      74.514 110.566 125.879  1.00 20.00           C
ATOM   2576  CG1 ILE A 493      75.233 109.840 127.036  1.00 20.00           C
ATOM   2577  CG2 ILE A 493      73.275 109.749 125.445  1.00 20.00           C
ATOM   2578  CD1 ILE A 493      74.471 109.866 128.365  1.00 20.00           C
ATOM   2579  H   ILE A 493      76.135 112.523 125.987  1.00  0.00           H
ATOM   2580  HA  ILE A 493      76.174 110.027 124.604  1.00  0.00           H
ATOM   2581  HB  ILE A 493      74.142 111.515 126.269  1.00  0.00           H
ATOM   2582 HG12 ILE A 493      75.433 108.806 126.752  1.00  0.00           H
ATOM   2583 HG13 ILE A 493      76.211 110.288 127.200  1.00  0.00           H
ATOM   2584 HG21 ILE A 493      72.634 109.517 126.295  1.00  0.00           H
ATOM   2585 HG22 ILE A 493      72.650 110.291 124.736  1.00  0.00           H
ATOM   2586 HG23 ILE A 493      73.570 108.804 124.988  1.00  0.00           H
ATOM   2587 HD11 ILE A 493      75.152 109.655 129.187  1.00  0.00           H
ATOM   2588 HD12 ILE A 493      74.017 110.840 128.553  1.00  0.00           H
ATOM   2589 HD13 ILE A 493      73.684 109.113 128.391  1.00  0.00           H
ATOM   2590  N   GLN A 494      74.394 112.432 123.202  1.00 30.00           N
ATOM   2591  CA  GLN A 494      73.809 112.913 121.947  1.00 30.00           C
ATOM   2592  C   GLN A 494      74.769 112.773 120.755  1.00 30.00           C
ATOM   2593  O   GLN A 494      74.322 112.365 119.686  1.00 30.00           O
ATOM   2594  CB  GLN A 494      73.314 114.364 122.137  1.00 20.00           C
ATOM   2595  CG  GLN A 494      72.773 115.071 120.871  1.00 20.00           C
ATOM   2596  CD  GLN A 494      72.353 116.531 121.085  1.00 20.00           C
ATOM   2597  NE2 GLN A 494      72.267 117.002 122.332  1.00 20.00           N
ATOM   2598  OE1 GLN A 494      72.106 117.239 120.112  1.00 20.00           O
ATOM   2599  H   GLN A 494      74.497 113.124 123.933  1.00  0.00           H
ATOM   2600  HA  GLN A 494      72.939 112.288 121.737  1.00  0.00           H
ATOM   2601  HB2 GLN A 494      72.529 114.355 122.894  1.00  0.00           H
ATOM   2602  HB3 GLN A 494      74.131 114.957 122.542  1.00  0.00           H
ATOM   2603  HG2 GLN A 494      73.527 115.080 120.083  1.00  0.00           H
ATOM   2604  HG3 GLN A 494      71.920 114.519 120.476  1.00  0.00           H
ATOM   2605 HE21 GLN A 494      71.989 117.960 122.489  1.00  0.00           H
ATOM   2606 HE22 GLN A 494      72.468 116.406 123.121  1.00  0.00           H
ATOM   2607  N   TYR A 495      76.061 113.074 120.975  1.00 30.00           N
ATOM   2608  CA  TYR A 495      77.129 112.888 119.995  1.00 30.00           C
ATOM   2609  C   TYR A 495      77.320 111.408 119.612  1.00 30.00           C
ATOM   2610  O   TYR A 495      77.393 111.125 118.419  1.00 30.00           O
ATOM   2611  CB  TYR A 495      78.429 113.545 120.509  1.00 20.00           C
ATOM   2612  CG  TYR A 495      79.629 113.441 119.581  1.00 20.00           C
ATOM   2613  CD1 TYR A 495      79.982 114.525 118.750  1.00 20.00           C
ATOM   2614  CD2 TYR A 495      80.409 112.266 119.561  1.00 20.00           C
ATOM   2615  CE1 TYR A 495      81.117 114.438 117.919  1.00 20.00           C
ATOM   2616  CE2 TYR A 495      81.523 112.169 118.711  1.00 20.00           C
ATOM   2617  CZ  TYR A 495      81.891 113.261 117.904  1.00 20.00           C
```

```
ATOM   2618  OH  TYR A 495      82.997 113.179 117.111  1.00 20.00           O
ATOM   2619  H   TYR A 495      76.346 113.419 121.882  1.00  0.00           H
ATOM   2620  HA  TYR A 495      76.829 113.423 119.091  1.00  0.00           H
ATOM   2621  HB2 TYR A 495      78.245 114.601 120.717  1.00  0.00           H
ATOM   2622  HB3 TYR A 495      78.717 113.111 121.466  1.00  0.00           H
ATOM   2623  HD1 TYR A 495      79.398 115.433 118.761  1.00  0.00           H
ATOM   2624  HD2 TYR A 495      80.154 111.434 120.198  1.00  0.00           H
ATOM   2625  HE1 TYR A 495      81.393 115.278 117.299  1.00  0.00           H
ATOM   2626  HE2 TYR A 495      82.101 111.259 118.700  1.00  0.00           H
ATOM   2627  HH  TYR A 495      83.480 112.359 117.235  1.00  0.00           H
ATOM   2628  N   PHE A 496      77.376 110.513 120.619  1.00 30.00           N
ATOM   2629  CA  PHE A 496      77.578 109.066 120.459  1.00 30.00           C
ATOM   2630  C   PHE A 496      76.406 108.324 119.784  1.00 30.00           C
ATOM   2631  O   PHE A 496      76.625 107.223 119.278  1.00 30.00           O
ATOM   2632  CB  PHE A 496      77.861 108.412 121.833  1.00 20.00           C
ATOM   2633  CG  PHE A 496      79.068 108.906 122.616  1.00 20.00           C
ATOM   2634  CD1 PHE A 496      80.290 109.226 121.981  1.00 20.00           C
ATOM   2635  CD2 PHE A 496      79.024 108.890 124.027  1.00 20.00           C
ATOM   2636  CE1 PHE A 496      81.384 109.626 122.739  1.00 20.00           C
ATOM   2637  CE2 PHE A 496      80.128 109.290 124.767  1.00 20.00           C
ATOM   2638  CZ  PHE A 496      81.298 109.670 124.125  1.00 20.00           C
ATOM   2639  H   PHE A 496      77.310 110.843 121.573  1.00  0.00           H
ATOM   2640  HA  PHE A 496      78.444 108.922 119.811  1.00  0.00           H
ATOM   2641  HB2 PHE A 496      76.981 108.550 122.465  1.00  0.00           H
ATOM   2642  HB3 PHE A 496      77.975 107.333 121.715  1.00  0.00           H
ATOM   2643  HD1 PHE A 496      80.383 109.181 120.907  1.00  0.00           H
ATOM   2644  HD2 PHE A 496      78.123 108.591 124.541  1.00  0.00           H
ATOM   2645  HE1 PHE A 496      82.305 109.901 122.246  1.00  0.00           H
ATOM   2646  HE2 PHE A 496      80.072 109.305 125.844  1.00  0.00           H
ATOM   2647  HZ  PHE A 496      82.146 109.988 124.713  1.00  0.00           H
ATOM   2648  N   LEU A 497      75.203 108.923 119.787  1.00 30.00           N
ATOM   2649  CA  LEU A 497      74.006 108.384 119.141  1.00 30.00           C
ATOM   2650  C   LEU A 497      73.801 108.967 117.732  1.00 30.00           C
ATOM   2651  O   LEU A 497      73.343 108.234 116.856  1.00 30.00           O
ATOM   2652  CB  LEU A 497      72.775 108.667 120.030  1.00 20.00           C
ATOM   2653  CG  LEU A 497      72.717 107.829 121.329  1.00 20.00           C
ATOM   2654  CD1 LEU A 497      71.594 108.328 122.259  1.00 20.00           C
ATOM   2655  CD2 LEU A 497      72.607 106.314 121.058  1.00 20.00           C
ATOM   2656  H   LEU A 497      75.097 109.812 120.255  1.00  0.00           H
ATOM   2657  HA  LEU A 497      74.108 107.304 119.017  1.00  0.00           H
ATOM   2658  HB2 LEU A 497      72.761 109.731 120.276  1.00  0.00           H
ATOM   2659  HB3 LEU A 497      71.859 108.484 119.465  1.00  0.00           H
ATOM   2660  HG  LEU A 497      73.653 107.990 121.864  1.00  0.00           H
ATOM   2661 HD11 LEU A 497      71.928 108.341 123.295  1.00  0.00           H
ATOM   2662 HD12 LEU A 497      71.275 109.342 122.011  1.00  0.00           H
ATOM   2663 HD13 LEU A 497      70.707 107.696 122.211  1.00  0.00           H
ATOM   2664 HD21 LEU A 497      71.862 105.830 121.690  1.00  0.00           H
ATOM   2665 HD22 LEU A 497      72.334 106.101 120.024  1.00  0.00           H
ATOM   2666 HD23 LEU A 497      73.559 105.819 121.252  1.00  0.00           H
ATOM   2667  N   GLN A 498      74.139 110.255 117.536  1.00 30.00           N
ATOM   2668  CA  GLN A 498      74.010 110.986 116.270  1.00 30.00           C
ATOM   2669  C   GLN A 498      74.959 110.452 115.183  1.00 30.00           C
ATOM   2670  O   GLN A 498      74.498 110.091 114.101  1.00 30.00           O
ATOM   2671  CB  GLN A 498      74.189 112.494 116.547  1.00 20.00           C
ATOM   2672  CG  GLN A 498      74.138 113.411 115.304  1.00 20.00           C
ATOM   2673  CD  GLN A 498      74.152 114.913 115.619  1.00 20.00           C
ATOM   2674  NE2 GLN A 498      74.424 115.308 116.867  1.00 20.00           N
ATOM   2675  OE1 GLN A 498      73.921 115.723 114.726  1.00 20.00           O
ATOM   2676  H   GLN A 498      74.498 110.793 118.313  1.00  0.00           H
ATOM   2677  HA  GLN A 498      72.989 110.837 115.912  1.00  0.00           H
ATOM   2678  HB2 GLN A 498      73.400 112.798 117.236  1.00  0.00           H
ATOM   2679  HB3 GLN A 498      75.134 112.651 117.070  1.00  0.00           H
ATOM   2680  HG2 GLN A 498      74.981 113.204 114.644  1.00  0.00           H
ATOM   2681  HG3 GLN A 498      73.234 113.197 114.732  1.00  0.00           H
ATOM   2682 HE21 GLN A 498      74.437 116.292 117.092  1.00  0.00           H
ATOM   2683 HE22 GLN A 498      74.610 114.630 117.591  1.00  0.00           H
ATOM   2684  N   ARG A 499      76.251 110.370 115.528  1.00 30.00           N
ATOM   2685  CA  ARG A 499      77.274 109.623 114.798  1.00 30.00           C
ATOM   2686  C   ARG A 499      77.820 108.549 115.749  1.00 30.00           C
ATOM   2687  O   ARG A 499      77.810 108.752 116.961  1.00 30.00           O
ATOM   2688  CB  ARG A 499      78.376 110.579 114.282  1.00 20.00           C
```

```
ATOM   2689  CG  ARG A 499      79.065 111.477 115.335  1.00 20.00           C
ATOM   2690  CD  ARG A 499      80.298 112.214 114.781  1.00 20.00           C
ATOM   2691  NE  ARG A 499      81.427 111.292 114.576  1.00 20.00           N
ATOM   2692  CZ  ARG A 499      82.673 111.625 114.197  1.00 20.00           C
ATOM   2693  NH1 ARG A 499      83.031 112.898 113.983  1.00 20.00           N
ATOM   2694  NH2 ARG A 499      83.586 110.660 114.040  1.00 20.00           N1+
ATOM   2695  H   ARG A 499      76.529 110.676 116.452  1.00  0.00           H
ATOM   2696  HA  ARG A 499      76.830 109.113 113.941  1.00  0.00           H
ATOM   2697  HB2 ARG A 499      77.943 111.224 113.517  1.00  0.00           H
ATOM   2698  HB3 ARG A 499      79.129 109.981 113.768  1.00  0.00           H
ATOM   2699  HG2 ARG A 499      78.375 112.173 115.813  1.00  0.00           H
ATOM   2700  HG3 ARG A 499      79.422 110.827 116.134  1.00  0.00           H
ATOM   2701  HD2 ARG A 499      80.070 112.590 113.783  1.00  0.00           H
ATOM   2702  HD3 ARG A 499      80.551 113.083 115.387  1.00  0.00           H
ATOM   2703  HE  ARG A 499      81.219 110.314 114.712  1.00  0.00           H
ATOM   2704 HH11 ARG A 499      83.984 113.117 113.725  1.00  0.00           H
ATOM   2705 HH12 ARG A 499      82.369 113.645 114.135  1.00  0.00           H
ATOM   2706 HH21 ARG A 499      84.545 110.909 113.796  1.00  0.00           H
ATOM   2707 HH22 ARG A 499      83.351 109.689 114.181  1.00  0.00           H
ATOM   2708  N   ARG A 500      78.287 107.421 115.196  1.00 30.00           N
ATOM   2709  CA  ARG A 500      78.702 106.254 115.979  1.00 30.00           C
ATOM   2710  C   ARG A 500      80.173 105.876 115.707  1.00 30.00           C
ATOM   2711  O   ARG A 500      80.411 104.848 115.071  1.00 30.00           O
ATOM   2712  CB  ARG A 500      77.725 105.084 115.709  1.00 20.00           C
ATOM   2713  CG  ARG A 500      76.272 105.361 116.147  1.00 20.00           C
ATOM   2714  CD  ARG A 500      75.340 104.153 115.960  1.00 20.00           C
ATOM   2715  NE  ARG A 500      75.642 103.066 116.905  1.00 20.00           N
ATOM   2716  CZ  ARG A 500      75.242 101.788 116.789  1.00 20.00           C
ATOM   2717  NH1 ARG A 500      74.494 101.379 115.754  1.00 20.00           N
ATOM   2718  NH2 ARG A 500      75.598 100.902 117.728  1.00 20.00           N1+
ATOM   2719  H   ARG A 500      78.289 107.313 114.192  1.00  0.00           H
ATOM   2720  HA  ARG A 500      78.637 106.452 117.050  1.00  0.00           H
ATOM   2721  HB2 ARG A 500      77.739 104.834 114.647  1.00  0.00           H
ATOM   2722  HB3 ARG A 500      78.081 104.197 116.236  1.00  0.00           H
ATOM   2723  HG2 ARG A 500      76.312 105.586 117.212  1.00  0.00           H
ATOM   2724  HG3 ARG A 500      75.843 106.244 115.674  1.00  0.00           H
ATOM   2725  HD2 ARG A 500      75.572 103.728 114.983  1.00  0.00           H
ATOM   2726  HD3 ARG A 500      74.286 104.430 115.930  1.00  0.00           H
ATOM   2727  HE  ARG A 500      76.163 103.336 117.727  1.00  0.00           H
ATOM   2728 HH11 ARG A 500      74.199 100.416 115.678  1.00  0.00           H
ATOM   2729 HH12 ARG A 500      74.217 102.039 115.042  1.00  0.00           H
ATOM   2730 HH21 ARG A 500      75.305  99.938 117.659  1.00  0.00           H
ATOM   2731 HH22 ARG A 500      76.160 101.188 118.516  1.00  0.00           H
ATOM   2732  N   PRO A 501      81.147 106.673 116.217  1.00 30.00           N
ATOM   2733  CA  PRO A 501      82.549 106.227 116.292  1.00 30.00           C
ATOM   2734  C   PRO A 501      82.708 105.076 117.304  1.00 30.00           C
ATOM   2735  O   PRO A 501      82.232 105.183 118.434  1.00 30.00           O
ATOM   2736  CB  PRO A 501      83.319 107.493 116.699  1.00 20.00           C
ATOM   2737  CG  PRO A 501      82.320 108.337 117.471  1.00  0.00           C
ATOM   2738  CD  PRO A 501      80.978 107.992 116.836  1.00  0.00           C
ATOM   2739  HA  PRO A 501      82.896 105.897 115.310  1.00  0.00           H
ATOM   2740  HB2 PRO A 501      84.217 107.288 117.281  1.00  0.00           H
ATOM   2741  HB3 PRO A 501      83.633 108.026 115.800  1.00  0.00           H
ATOM   2742  HG2 PRO A 501      82.312 108.028 118.515  1.00  0.00           H
ATOM   2743  HG3 PRO A 501      82.550 109.401 117.442  1.00  0.00           H
ATOM   2744  HD2 PRO A 501      80.195 108.015 117.593  1.00  0.00           H
ATOM   2745  HD3 PRO A 501      80.737 108.713 116.056  1.00  0.00           H
ATOM   2746  N   SER A 502      83.320 103.977 116.846  1.00 30.00           N
ATOM   2747  CA  SER A 502      83.491 102.735 117.597  1.00 30.00           C
ATOM   2748  C   SER A 502      84.637 101.919 116.971  1.00 30.00           C
ATOM   2749  O   SER A 502      85.054 102.212 115.849  1.00 30.00           O
ATOM   2750  CB  SER A 502      82.144 101.967 117.622  1.00 20.00           C
ATOM   2751  OG  SER A 502      82.202 100.799 118.418  1.00  0.00           O
ATOM   2752  H   SER A 502      83.680 103.964 115.902  1.00  0.00           H
ATOM   2753  HA  SER A 502      83.784 102.984 118.619  1.00  0.00           H
ATOM   2754  HB2 SER A 502      81.348 102.597 118.021  1.00  0.00           H
ATOM   2755  HB3 SER A 502      81.844 101.688 116.611  1.00  0.00           H
ATOM   2756  HG  SER A 502      81.345 100.368 118.391  1.00  0.00           H
ATOM   2757  N   LEU A 503      85.120 100.907 117.714  1.00 30.00           N
ATOM   2758  CA  LEU A 503      86.167  99.954 117.319  1.00 30.00           C
ATOM   2759  C   LEU A 503      87.512 100.658 117.053  1.00 30.00           C
```

```
ATOM   2760  O   LEU A 503      88.006 100.651 115.926  1.00 30.00           O
ATOM   2761  CB  LEU A 503      85.702  99.069 116.128  1.00 20.00           C
ATOM   2762  CG  LEU A 503      84.437  98.222 116.400  1.00 20.00           C
ATOM   2763  CD1 LEU A 503      83.939  97.542 115.107  1.00 20.00           C
ATOM   2764  CD2 LEU A 503      84.639  97.213 117.551  1.00 20.00           C
ATOM   2765  H   LEU A 503      84.687 100.725 118.608  1.00  0.00           H
ATOM   2766  HA  LEU A 503      86.334  99.308 118.181  1.00  0.00           H
ATOM   2767  HB2 LEU A 503      85.527  99.700 115.256  1.00  0.00           H
ATOM   2768  HB3 LEU A 503      86.512  98.396 115.840  1.00  0.00           H
ATOM   2769  HG  LEU A 503      83.642  98.903 116.704  1.00  0.00           H
ATOM   2770 HD11 LEU A 503      82.892  97.785 114.921  1.00  0.00           H
ATOM   2771 HD12 LEU A 503      84.501  97.865 114.230  1.00  0.00           H
ATOM   2772 HD13 LEU A 503      84.017  96.455 115.143  1.00  0.00           H
ATOM   2773 HD21 LEU A 503      84.230  96.228 117.327  1.00  0.00           H
ATOM   2774 HD22 LEU A 503      85.693  97.071 117.790  1.00  0.00           H
ATOM   2775 HD23 LEU A 503      84.144  97.562 118.457  1.00  0.00           H
ATOM   2776  N   LYS A 504      88.061 101.269 118.119  1.00 30.00           N
ATOM   2777  CA  LYS A 504      89.340 101.991 118.155  1.00 30.00           C
ATOM   2778  C   LYS A 504      89.322 103.289 117.311  1.00 30.00           C
ATOM   2779  O   LYS A 504      90.324 103.638 116.689  1.00 30.00           O
ATOM   2780  CB  LYS A 504      90.511 101.024 117.831  1.00 20.00           C
ATOM   2781  CG  LYS A 504      91.900 101.493 118.305  1.00  0.00           C
ATOM   2782  CD  LYS A 504      92.989 100.452 118.012  1.00  0.00           C
ATOM   2783  CE  LYS A 504      94.381 100.909 118.467  1.00  0.00           C
ATOM   2784  NZ  LYS A 504      95.401  99.889 118.171  1.00  0.00           N1+
ATOM   2785  H   LYS A 504      87.571 101.220 119.000  1.00  0.00           H
ATOM   2786  HA  LYS A 504      89.460 102.316 119.189  1.00  0.00           H
ATOM   2787  HB2 LYS A 504      90.314 100.074 118.330  1.00  0.00           H
ATOM   2788  HB3 LYS A 504      90.535 100.799 116.764  1.00  0.00           H
ATOM   2789  HG2 LYS A 504      92.177 102.433 117.829  1.00  0.00           H
ATOM   2790  HG3 LYS A 504      91.868 101.694 119.376  1.00  0.00           H
ATOM   2791  HD2 LYS A 504      92.734  99.513 118.505  1.00  0.00           H
ATOM   2792  HD3 LYS A 504      93.006 100.241 116.942  1.00  0.00           H
ATOM   2793  HE2 LYS A 504      94.658 101.838 117.967  1.00  0.00           H
ATOM   2794  HE3 LYS A 504      94.383 101.107 119.540  1.00  0.00           H
ATOM   2795  HZ1 LYS A 504      96.305 100.214 118.482  1.00  0.00           H
ATOM   2796  HZ2 LYS A 504      95.169  99.031 118.652  1.00  0.00           H
ATOM   2797  HZ3 LYS A 504      95.428  99.717 117.176  1.00  0.00           H
ATOM   2798  N   SER A 505      88.178 103.994 117.328  1.00 30.00           N
ATOM   2799  CA  SER A 505      87.998 105.302 116.691  1.00 30.00           C
ATOM   2800  C   SER A 505      87.223 106.294 117.583  1.00 30.00           C
ATOM   2801  O   SER A 505      86.904 107.387 117.120  1.00 30.00           O
ATOM   2802  CB  SER A 505      87.431 105.126 115.265  1.00 20.00           C
ATOM   2803  OG  SER A 505      86.038 104.877 115.245  1.00  0.00           O
ATOM   2804  H   SER A 505      87.375 103.622 117.814  1.00  0.00           H
ATOM   2805  HA  SER A 505      88.982 105.762 116.581  1.00  0.00           H
ATOM   2806  HB2 SER A 505      87.631 106.029 114.691  1.00  0.00           H
ATOM   2807  HB3 SER A 505      87.944 104.318 114.742  1.00  0.00           H
ATOM   2808  HG  SER A 505      85.895 103.946 115.446  1.00  0.00           H
ATOM   2809  N   LEU A 506      86.992 105.918 118.855  1.00 30.00           N
ATOM   2810  CA  LEU A 506      86.514 106.783 119.940  1.00 30.00           C
ATOM   2811  C   LEU A 506      87.600 107.742 120.459  1.00 30.00           C
ATOM   2812  O   LEU A 506      87.251 108.770 121.039  1.00 30.00           O
ATOM   2813  CB  LEU A 506      86.031 105.886 121.104  1.00 20.00           C
ATOM   2814  CG  LEU A 506      84.580 105.394 120.942  1.00 20.00           C
ATOM   2815  CD1 LEU A 506      84.297 104.154 121.818  1.00 20.00           C
ATOM   2816  CD2 LEU A 506      83.573 106.542 121.192  1.00 20.00           C
ATOM   2817  H   LEU A 506      87.280 104.995 119.143  1.00  0.00           H
ATOM   2818  HA  LEU A 506      85.689 107.395 119.571  1.00  0.00           H
ATOM   2819  HB2 LEU A 506      86.712 105.037 121.193  1.00  0.00           H
ATOM   2820  HB3 LEU A 506      86.106 106.406 122.062  1.00  0.00           H
ATOM   2821  HG  LEU A 506      84.467 105.070 119.907  1.00  0.00           H
ATOM   2822 HD11 LEU A 506      83.974 103.315 121.201  1.00  0.00           H
ATOM   2823 HD12 LEU A 506      85.180 103.827 122.367  1.00  0.00           H
ATOM   2824 HD13 LEU A 506      83.516 104.326 122.559  1.00  0.00           H
ATOM   2825 HD21 LEU A 506      83.054 106.807 120.274  1.00  0.00           H
ATOM   2826 HD22 LEU A 506      82.807 106.289 121.925  1.00  0.00           H
ATOM   2827 HD23 LEU A 506      84.062 107.449 121.547  1.00  0.00           H
ATOM   2828  N   PHE A 507      88.873 107.407 120.200  1.00 30.00           N
ATOM   2829  CA  PHE A 507      90.037 108.104 120.747  1.00 30.00           C
ATOM   2830  C   PHE A 507      90.839 108.813 119.641  1.00 30.00           C
```

```
ATOM   2831  O   PHE A 507      91.341 109.905 119.888  1.00 30.00           O
ATOM   2832  CB  PHE A 507      90.944 107.075 121.470  1.00 20.00           C
ATOM   2833  CG  PHE A 507      90.201 105.954 122.185  1.00 20.00           C
ATOM   2834  CD1 PHE A 507      89.504 106.216 123.383  1.00 20.00           C
ATOM   2835  CD2 PHE A 507      90.036 104.701 121.555  1.00 20.00           C
ATOM   2836  CE1 PHE A 507      88.701 105.234 123.947  1.00 20.00           C
ATOM   2837  CE2 PHE A 507      89.235 103.730 122.140  1.00 20.00           C
ATOM   2838  CZ  PHE A 507      88.570 103.996 123.330  1.00 20.00           C
ATOM   2839  H   PHE A 507      89.039 106.557 119.697  1.00  0.00           H
ATOM   2840  HA  PHE A 507      89.718 108.857 121.470  1.00  0.00           H
ATOM   2841  HB2 PHE A 507      91.656 106.621 120.779  1.00  0.00           H
ATOM   2842  HB3 PHE A 507      91.551 107.609 122.203  1.00  0.00           H
ATOM   2843  HD1 PHE A 507      89.578 107.184 123.856  1.00  0.00           H
ATOM   2844  HD2 PHE A 507      90.532 104.490 120.618  1.00  0.00           H
ATOM   2845  HE1 PHE A 507      88.168 105.438 124.865  1.00  0.00           H
ATOM   2846  HE2 PHE A 507      89.121 102.764 121.668  1.00  0.00           H
ATOM   2847  HZ  PHE A 507      87.942 103.238 123.777  1.00  0.00           H
ATOM   2848  N   VAL A 508      91.011 108.167 118.473  1.00 30.00           N
ATOM   2849  CA  VAL A 508      91.838 108.681 117.371  1.00 30.00           C
ATOM   2850  C   VAL A 508      91.177 109.884 116.662  1.00 30.00           C
ATOM   2851  O   VAL A 508      91.807 110.936 116.552  1.00 30.00           O
ATOM   2852  CB  VAL A 508      92.173 107.583 116.321  1.00 20.00           C
ATOM   2853  CG1 VAL A 508      93.197 108.065 115.275  1.00 20.00           C
ATOM   2854  CG2 VAL A 508      92.703 106.295 116.980  1.00 20.00           C
ATOM   2855  H   VAL A 508      90.668 107.224 118.369  1.00  0.00           H
ATOM   2856  HA  VAL A 508      92.779 109.030 117.801  1.00  0.00           H
ATOM   2857  HB  VAL A 508      91.260 107.317 115.786  1.00  0.00           H
ATOM   2858 HG11 VAL A 508      93.388 107.294 114.529  1.00  0.00           H
ATOM   2859 HG12 VAL A 508      92.857 108.952 114.744  1.00  0.00           H
ATOM   2860 HG13 VAL A 508      94.150 108.314 115.744  1.00  0.00           H
ATOM   2861 HG21 VAL A 508      92.977 105.553 116.230  1.00  0.00           H
ATOM   2862 HG22 VAL A 508      93.585 106.496 117.589  1.00  0.00           H
ATOM   2863 HG23 VAL A 508      91.953 105.831 117.622  1.00  0.00           H
ATOM   2864  N   ASP A 509      89.898 109.719 116.286  1.00 30.00           N
ATOM   2865  CA  ASP A 509      88.933 110.808 116.116  1.00 30.00           C
ATOM   2866  C   ASP A 509      88.111 110.864 117.423  1.00 30.00           C
ATOM   2867  O   ASP A 509      88.087 109.884 118.167  1.00 30.00           O
ATOM   2868  CB  ASP A 509      88.026 110.560 114.879  1.00 20.00           C
ATOM   2869  CG  ASP A 509      87.164 111.758 114.443  1.00  0.00           C
ATOM   2870  OD1 ASP A 509      87.471 112.898 114.858  1.00  0.00           O
ATOM   2871  OD2 ASP A 509      86.222 111.520 113.657  1.00  0.00           O1-
ATOM   2872  H   ASP A 509      89.475 108.812 116.418  1.00  0.00           H
ATOM   2873  HA  ASP A 509      89.464 111.755 116.000  1.00  0.00           H
ATOM   2874  HB2 ASP A 509      88.648 110.274 114.030  1.00  0.00           H
ATOM   2875  HB3 ASP A 509      87.361 109.722 115.092  1.00  0.00           H
ATOM   2876  N   SER A 510      87.444 111.999 117.690  1.00 30.00           N
ATOM   2877  CA  SER A 510      86.567 112.214 118.851  1.00 30.00           C
ATOM   2878  C   SER A 510      87.321 112.304 120.203  1.00 30.00           C
ATOM   2879  O   SER A 510      86.698 112.102 121.246  1.00 30.00           O
ATOM   2880  CB  SER A 510      85.408 111.173 118.841  1.00 20.00           C
ATOM   2881  OG  SER A 510      84.411 111.449 119.801  1.00 20.00           O
ATOM   2882  H   SER A 510      87.482 112.751 117.013  1.00  0.00           H
ATOM   2883  HA  SER A 510      86.121 113.198 118.698  1.00  0.00           H
ATOM   2884  HB2 SER A 510      84.933 111.149 117.860  1.00  0.00           H
ATOM   2885  HB3 SER A 510      85.766 110.164 119.038  1.00  0.00           H
ATOM   2886  HG  SER A 510      84.819 111.400 120.671  1.00  0.00           H
ATOM   2887  N   TYR A 511      88.619 112.652 120.196  1.00 30.00           N
ATOM   2888  CA  TYR A 511      89.429 112.811 121.416  1.00 30.00           C
ATOM   2889  C   TYR A 511      88.864 113.883 122.373  1.00 30.00           C
ATOM   2890  O   TYR A 511      88.801 113.681 123.583  1.00 30.00           O
ATOM   2891  CB  TYR A 511      90.889 113.146 121.034  1.00 20.00           C
ATOM   2892  CG  TYR A 511      91.858 113.235 122.203  1.00 20.00           C
ATOM   2893  CD1 TYR A 511      92.437 112.055 122.714  1.00 20.00           C
ATOM   2894  CD2 TYR A 511      92.185 114.482 122.782  1.00 20.00           C
ATOM   2895  CE1 TYR A 511      93.330 112.120 123.800  1.00 20.00           C
ATOM   2896  CE2 TYR A 511      93.075 114.545 123.872  1.00 20.00           C
ATOM   2897  CZ  TYR A 511      93.641 113.361 124.385  1.00 20.00           C
ATOM   2898  OH  TYR A 511      94.489 113.397 125.450  1.00 20.00           O
ATOM   2899  H   TYR A 511      89.104 112.700 119.314  1.00  0.00           H
ATOM   2900  HA  TYR A 511      89.422 111.855 121.944  1.00  0.00           H
ATOM   2901  HB2 TYR A 511      91.273 112.395 120.344  1.00  0.00           H
```

```
ATOM   2902  HB3 TYR A 511      90.929 114.086 120.482  1.00  0.00           H
ATOM   2903  HD1 TYR A 511      92.200 111.098 122.270  1.00  0.00           H
ATOM   2904  HD2 TYR A 511      91.748 115.395 122.404  1.00  0.00           H
ATOM   2905  HE1 TYR A 511      93.772 111.216 124.192  1.00  0.00           H
ATOM   2906  HE2 TYR A 511      93.309 115.503 124.316  1.00  0.00           H
ATOM   2907  HH  TYR A 511      94.593 114.272 125.849  1.00  0.00           H
ATOM   2908  N   SER A 512      88.431 115.010 121.797  1.00 30.00           N
ATOM   2909  CA  SER A 512      87.976 116.175 122.548  1.00 30.00           C
ATOM   2910  C   SER A 512      86.640 115.919 123.282  1.00 30.00           C
ATOM   2911  O   SER A 512      86.448 116.425 124.387  1.00 30.00           O
ATOM   2912  CB  SER A 512      87.888 117.362 121.560  1.00 20.00           C
ATOM   2913  OG  SER A 512      87.910 118.622 122.201  1.00 20.00           O
ATOM   2914  H   SER A 512      88.497 115.097 120.797  1.00  0.00           H
ATOM   2915  HA  SER A 512      88.738 116.396 123.300  1.00  0.00           H
ATOM   2916  HB2 SER A 512      88.735 117.344 120.872  1.00  0.00           H
ATOM   2917  HB3 SER A 512      86.990 117.290 120.944  1.00  0.00           H
ATOM   2918  HG  SER A 512      87.140 118.690 122.779  1.00  0.00           H
ATOM   2919  N   GLU A 513      85.737 115.140 122.665  1.00 30.00           N
ATOM   2920  CA  GLU A 513      84.382 114.940 123.178  1.00 30.00           C
ATOM   2921  C   GLU A 513      84.364 113.970 124.369  1.00 30.00           C
ATOM   2922  O   GLU A 513      83.656 114.219 125.348  1.00 30.00           O
ATOM   2923  CB  GLU A 513      83.450 114.476 122.034  1.00 20.00           C
ATOM   2924  CG  GLU A 513      81.940 114.482 122.376  1.00 20.00           C
ATOM   2925  CD  GLU A 513      81.353 115.871 122.657  1.00 20.00           C
ATOM   2926  OE1 GLU A 513      81.960 116.870 122.214  1.00 20.00           O
ATOM   2927  OE2 GLU A 513      80.289 115.918 123.311  1.00 20.00           O1-
ATOM   2928  H   GLU A 513      85.987 114.651 121.819  1.00  0.00           H
ATOM   2929  HA  GLU A 513      84.027 115.905 123.544  1.00  0.00           H
ATOM   2930  HB2 GLU A 513      83.613 115.095 121.151  1.00  0.00           H
ATOM   2931  HB3 GLU A 513      83.726 113.466 121.729  1.00  0.00           H
ATOM   2932  HG2 GLU A 513      81.733 113.830 123.225  1.00  0.00           H
ATOM   2933  HG3 GLU A 513      81.390 114.064 121.537  1.00  0.00           H
ATOM   2934  N   ILE A 514      85.180 112.904 124.298  1.00  0.00           N
ATOM   2935  CA  ILE A 514      85.290 111.929 125.380  1.00  0.00           C
ATOM   2936  C   ILE A 514      85.817 112.555 126.691  1.00  0.00           C
ATOM   2937  O   ILE A 514      85.308 112.211 127.755  1.00  0.00           O
ATOM   2938  CB  ILE A 514      86.129 110.675 124.995  1.00 20.00           C
ATOM   2939  CG1 ILE A 514      87.587 110.971 124.576  1.00 20.00           C
ATOM   2940  CG2 ILE A 514      85.409 109.846 123.911  1.00 20.00           C
ATOM   2941  CD1 ILE A 514      88.471 109.725 124.439  1.00 20.00           C
ATOM   2942  H   ILE A 514      85.768 112.760 123.486  1.00  0.00           H
ATOM   2943  HA  ILE A 514      84.276 111.585 125.594  1.00  0.00           H
ATOM   2944  HB  ILE A 514      86.178 110.047 125.888  1.00  0.00           H
ATOM   2945 HG12 ILE A 514      88.073 111.651 125.274  1.00  0.00           H
ATOM   2946 HG13 ILE A 514      87.573 111.480 123.615  1.00  0.00           H
ATOM   2947 HG21 ILE A 514      85.927 108.909 123.709  1.00  0.00           H
ATOM   2948 HG22 ILE A 514      84.395 109.589 124.212  1.00  0.00           H
ATOM   2949 HG23 ILE A 514      85.349 110.388 122.966  1.00  0.00           H
ATOM   2950 HD11 ILE A 514      89.526 109.993 124.501  1.00  0.00           H
ATOM   2951 HD12 ILE A 514      88.265 108.994 125.221  1.00  0.00           H
ATOM   2952 HD13 ILE A 514      88.312 109.243 123.475  1.00  0.00           H
ATOM   2953  N   LEU A 515      86.723 113.540 126.596  1.00  0.00           N
ATOM   2954  CA  LEU A 515      87.233 114.290 127.746  1.00  0.00           C
ATOM   2955  C   LEU A 515      86.145 115.130 128.448  1.00  0.00           C
ATOM   2956  O   LEU A 515      86.101 115.144 129.679  1.00  0.00           O
ATOM   2957  CB  LEU A 515      88.426 115.166 127.317  1.00 20.00           C
ATOM   2958  CG  LEU A 515      89.692 114.387 126.893  1.00 20.00           C
ATOM   2959  CD1 LEU A 515      90.726 115.340 126.265  1.00 20.00           C
ATOM   2960  CD2 LEU A 515      90.298 113.580 128.063  1.00 20.00           C
ATOM   2961  H   LEU A 515      87.094 113.792 125.688  1.00  0.00           H
ATOM   2962  HA  LEU A 515      87.578 113.564 128.483  1.00  0.00           H
ATOM   2963  HB2 LEU A 515      88.109 115.816 126.500  1.00  0.00           H
ATOM   2964  HB3 LEU A 515      88.696 115.827 128.139  1.00  0.00           H
ATOM   2965  HG  LEU A 515      89.418 113.672 126.117  1.00  0.00           H
ATOM   2966 HD11 LEU A 515      91.755 115.026 126.440  1.00  0.00           H
ATOM   2967 HD12 LEU A 515      90.590 115.395 125.184  1.00  0.00           H
ATOM   2968 HD13 LEU A 515      90.635 116.351 126.659  1.00  0.00           H
ATOM   2969 HD21 LEU A 515      91.363 113.769 128.200  1.00  0.00           H
ATOM   2970 HD22 LEU A 515      89.820 113.811 129.015  1.00  0.00           H
ATOM   2971 HD23 LEU A 515      90.184 112.509 127.891  1.00  0.00           H
ATOM   2972  N   PHE A 516      85.244 115.769 127.686  1.00  0.00           N
```

```
ATOM   2973  CA  PHE A 516      84.071 116.449 128.255  1.00  0.00           C
ATOM   2974  C   PHE A 516      83.075 115.473 128.911  1.00  0.00           C
ATOM   2975  O   PHE A 516      82.497 115.785 129.952  1.00  0.00           O
ATOM   2976  CB  PHE A 516      83.363 117.344 127.213  1.00 20.00           C
ATOM   2977  CG  PHE A 516      84.036 118.679 126.931  1.00 20.00           C
ATOM   2978  CD1 PHE A 516      84.075 119.677 127.929  1.00 20.00           C
ATOM   2979  CD2 PHE A 516      84.662 118.928 125.691  1.00 20.00           C
ATOM   2980  CE1 PHE A 516      84.698 120.890 127.673  1.00 20.00           C
ATOM   2981  CE2 PHE A 516      85.280 120.151 125.456  1.00 20.00           C
ATOM   2982  CZ  PHE A 516      85.300 121.128 126.444  1.00 20.00           C
ATOM   2983  H   PHE A 516      85.322 115.737 126.677  1.00  0.00           H
ATOM   2984  HA  PHE A 516      84.418 117.099 129.061  1.00  0.00           H
ATOM   2985  HB2 PHE A 516      83.230 116.798 126.277  1.00  0.00           H
ATOM   2986  HB3 PHE A 516      82.354 117.577 127.558  1.00  0.00           H
ATOM   2987  HD1 PHE A 516      83.611 119.513 128.892  1.00  0.00           H
ATOM   2988  HD2 PHE A 516      84.650 118.178 124.913  1.00  0.00           H
ATOM   2989  HE1 PHE A 516      84.716 121.648 128.439  1.00  0.00           H
ATOM   2990  HE2 PHE A 516      85.745 120.345 124.500  1.00  0.00           H
ATOM   2991  HZ  PHE A 516      85.788 122.074 126.257  1.00  0.00           H
ATOM   2992  N   PHE A 517      82.914 114.281 128.326  1.00  0.00           N
ATOM   2993  CA  PHE A 517      82.029 113.242 128.844  1.00  0.00           C
ATOM   2994  C   PHE A 517      82.575 112.545 130.110  1.00  0.00           C
ATOM   2995  O   PHE A 517      81.782 112.218 130.991  1.00  0.00           O
ATOM   2996  CB  PHE A 517      81.655 112.263 127.712  1.00 20.00           C
ATOM   2997  CG  PHE A 517      80.683 111.171 128.119  1.00 20.00           C
ATOM   2998  CD1 PHE A 517      79.332 111.494 128.365  1.00 20.00           C
ATOM   2999  CD2 PHE A 517      81.146 109.872 128.421  1.00 20.00           C
ATOM   3000  CE1 PHE A 517      78.468 110.529 128.858  1.00 20.00           C
ATOM   3001  CE2 PHE A 517      80.258 108.916 128.897  1.00 20.00           C
ATOM   3002  CZ  PHE A 517      78.925 109.243 129.114  1.00 20.00           C
ATOM   3003  H   PHE A 517      83.451 114.047 127.503  1.00  0.00           H
ATOM   3004  HA  PHE A 517      81.100 113.735 129.138  1.00  0.00           H
ATOM   3005  HB2 PHE A 517      81.184 112.828 126.905  1.00  0.00           H
ATOM   3006  HB3 PHE A 517      82.549 111.814 127.281  1.00  0.00           H
ATOM   3007  HD1 PHE A 517      78.971 112.497 128.188  1.00  0.00           H
ATOM   3008  HD2 PHE A 517      82.187 109.619 128.284  1.00  0.00           H
ATOM   3009  HE1 PHE A 517      77.438 110.785 129.056  1.00  0.00           H
ATOM   3010  HE2 PHE A 517      80.610 107.919 129.117  1.00  0.00           H
ATOM   3011  HZ  PHE A 517      78.244 108.498 129.498  1.00  0.00           H
ATOM   3012  N   VAL A 518      83.900 112.368 130.210  1.00  0.00           N
ATOM   3013  CA  VAL A 518      84.565 111.853 131.410  1.00  0.00           C
ATOM   3014  C   VAL A 518      84.503 112.871 132.570  1.00  0.00           C
ATOM   3015  O   VAL A 518      84.185 112.497 133.700  1.00  0.00           O
ATOM   3016  CB  VAL A 518      86.031 111.419 131.122  1.00 20.00           C
ATOM   3017  CG1 VAL A 518      86.884 111.129 132.374  1.00 20.00           C
ATOM   3018  CG2 VAL A 518      86.055 110.168 130.222  1.00 20.00           C
ATOM   3019  H   VAL A 518      84.495 112.565 129.408  1.00  0.00           H
ATOM   3020  HA  VAL A 518      84.027 110.962 131.728  1.00  0.00           H
ATOM   3021  HB  VAL A 518      86.521 112.228 130.576  1.00  0.00           H
ATOM   3022 HG11 VAL A 518      87.864 110.736 132.103  1.00  0.00           H
ATOM   3023 HG12 VAL A 518      87.061 112.027 132.964  1.00  0.00           H
ATOM   3024 HG13 VAL A 518      86.402 110.398 133.025  1.00  0.00           H
ATOM   3025 HG21 VAL A 518      87.069 109.936 129.896  1.00  0.00           H
ATOM   3026 HG22 VAL A 518      85.669 109.296 130.751  1.00  0.00           H
ATOM   3027 HG23 VAL A 518      85.448 110.287 129.327  1.00  0.00           H
ATOM   3028  N   GLN A 519      84.696 114.159 132.255  1.00  0.00           N
ATOM   3029  CA  GLN A 519      84.497 115.280 133.180  1.00  0.00           C
ATOM   3030  C   GLN A 519      83.070 115.304 133.764  1.00  0.00           C
ATOM   3031  O   GLN A 519      82.916 115.522 134.966  1.00  0.00           O
ATOM   3032  CB  GLN A 519      84.823 116.602 132.444  1.00 20.00           C
ATOM   3033  CG  GLN A 519      84.651 117.897 133.275  1.00 20.00           C
ATOM   3034  CD  GLN A 519      84.587 119.174 132.431  1.00 20.00           C
ATOM   3035  NE2 GLN A 519      84.264 120.298 133.073  1.00 20.00           N
ATOM   3036  OE1 GLN A 519      84.830 119.163 131.226  1.00 20.00           O
ATOM   3037  H   GLN A 519      84.889 114.412 131.295  1.00  0.00           H
ATOM   3038  HA  GLN A 519      85.202 115.161 134.005  1.00  0.00           H
ATOM   3039  HB2 GLN A 519      85.847 116.564 132.072  1.00  0.00           H
ATOM   3040  HB3 GLN A 519      84.192 116.661 131.558  1.00  0.00           H
ATOM   3041  HG2 GLN A 519      83.735 117.876 133.863  1.00  0.00           H
ATOM   3042  HG3 GLN A 519      85.473 117.978 133.984  1.00  0.00           H
ATOM   3043 HE21 GLN A 519      84.235 121.172 132.566  1.00  0.00           H
```

```
ATOM   3044 HE22 GLN A 519      84.070 120.290 134.064  1.00  0.00           H
ATOM   3045  N   SER A 520      82.069 115.078 132.895  1.00  0.00           N
ATOM   3046  CA  SER A 520      80.645 115.101 133.224  1.00  0.00           C
ATOM   3047  C   SER A 520      80.224 113.982 134.192  1.00  0.00           C
ATOM   3048  O   SER A 520      79.476 114.263 135.125  1.00  0.00           O
ATOM   3049  CB  SER A 520      79.820 115.125 131.919  1.00 20.00           C
ATOM   3050  OG  SER A 520      79.545 113.850 131.380  1.00 20.00           O
ATOM   3051  H   SER A 520      82.293 114.925 131.921  1.00  0.00           H
ATOM   3052  HA  SER A 520      80.467 116.053 133.729  1.00  0.00           H
ATOM   3053  HB2 SER A 520      80.303 115.735 131.161  1.00  0.00           H
ATOM   3054  HB3 SER A 520      78.863 115.603 132.103  1.00  0.00           H
ATOM   3055  HG  SER A 520      80.374 113.413 131.157  1.00  0.00           H
ATOM   3056  N   LEU A 521      80.736 112.754 133.982  1.00  0.00           N
ATOM   3057  CA  LEU A 521      80.458 111.607 134.847  1.00  0.00           C
ATOM   3058  C   LEU A 521      81.255 111.639 136.164  1.00  0.00           C
ATOM   3059  O   LEU A 521      80.767 111.076 137.139  1.00  0.00           O
ATOM   3060  CB  LEU A 521      80.546 110.283 134.051  1.00 20.00           C
ATOM   3061  CG  LEU A 521      81.945 109.847 133.557  1.00 20.00           C
ATOM   3062  CD1 LEU A 521      82.832 109.222 134.655  1.00 20.00           C
ATOM   3063  CD2 LEU A 521      81.840 108.920 132.329  1.00 20.00           C
ATOM   3064  H   LEU A 521      81.343 112.588 133.191  1.00  0.00           H
ATOM   3065  HA  LEU A 521      79.410 111.686 135.139  1.00  0.00           H
ATOM   3066  HB2 LEU A 521      79.882 110.401 133.193  1.00  0.00           H
ATOM   3067  HB3 LEU A 521      80.106 109.474 134.636  1.00  0.00           H
ATOM   3068  HG  LEU A 521      82.434 110.754 133.215  1.00  0.00           H
ATOM   3069 HD11 LEU A 521      83.292 108.288 134.333  1.00  0.00           H
ATOM   3070 HD12 LEU A 521      83.643 109.898 134.926  1.00  0.00           H
ATOM   3071 HD13 LEU A 521      82.270 109.004 135.563  1.00  0.00           H
ATOM   3072 HD21 LEU A 521      82.684 109.065 131.654  1.00  0.00           H
ATOM   3073 HD22 LEU A 521      81.827 107.867 132.614  1.00  0.00           H
ATOM   3074 HD23 LEU A 521      80.938 109.105 131.747  1.00  0.00           H
ATOM   3075  N   PHE A 522      82.396 112.345 136.212  1.00  0.00           N
ATOM   3076  CA  PHE A 522      83.050 112.700 137.475  1.00  0.00           C
ATOM   3077  C   PHE A 522      82.202 113.667 138.327  1.00  0.00           C
ATOM   3078  O   PHE A 522      82.159 113.513 139.552  1.00  0.00           O
ATOM   3079  CB  PHE A 522      84.469 113.267 137.242  1.00 20.00           C
ATOM   3080  CG  PHE A 522      85.594 112.274 137.017  1.00 20.00           C
ATOM   3081  CD1 PHE A 522      85.828 111.234 137.944  1.00 20.00           C
ATOM   3082  CD2 PHE A 522      86.517 112.476 135.968  1.00 20.00           C
ATOM   3083  CE1 PHE A 522      86.929 110.402 137.790  1.00 20.00           C
ATOM   3084  CE2 PHE A 522      87.611 111.633 135.834  1.00 20.00           C
ATOM   3085  CZ  PHE A 522      87.814 110.598 136.738  1.00 20.00           C
ATOM   3086  H   PHE A 522      82.823 112.681 135.354  1.00  0.00           H
ATOM   3087  HA  PHE A 522      83.137 111.789 138.068  1.00  0.00           H
ATOM   3088  HB2 PHE A 522      84.444 113.966 136.406  1.00  0.00           H
ATOM   3089  HB3 PHE A 522      84.784 113.847 138.107  1.00  0.00           H
ATOM   3090  HD1 PHE A 522      85.171 111.086 138.790  1.00  0.00           H
ATOM   3091  HD2 PHE A 522      86.385 113.286 135.264  1.00  0.00           H
ATOM   3092  HE1 PHE A 522      87.106 109.608 138.501  1.00  0.00           H
ATOM   3093  HE2 PHE A 522      88.315 111.790 135.029  1.00  0.00           H
ATOM   3094  HZ  PHE A 522      88.673 109.951 136.631  1.00  0.00           H
ATOM   3095  N   MET A 523      81.490 114.608 137.679  1.00  0.00           N
ATOM   3096  CA  MET A 523      80.554 115.500 138.372  1.00  0.00           C
ATOM   3097  C   MET A 523      79.317 114.745 138.894  1.00  0.00           C
ATOM   3098  O   MET A 523      78.875 115.026 140.004  1.00  0.00           O
ATOM   3099  CB  MET A 523      80.132 116.678 137.462  1.00 20.00           C
ATOM   3100  CG  MET A 523      79.500 117.856 138.232  1.00 20.00           C
ATOM   3101  SD  MET A 523      80.712 118.956 139.005  1.00 20.00           S
ATOM   3102  CE  MET A 523      79.709 119.687 140.325  1.00 20.00           C
ATOM   3103  H   MET A 523      81.488 114.646 136.669  1.00  0.00           H
ATOM   3104  HA  MET A 523      81.082 115.921 139.230  1.00  0.00           H
ATOM   3105  HB2 MET A 523      81.001 117.046 136.918  1.00  0.00           H
ATOM   3106  HB3 MET A 523      79.435 116.337 136.696  1.00  0.00           H
ATOM   3107  HG2 MET A 523      78.901 118.469 137.562  1.00  0.00           H
ATOM   3108  HG3 MET A 523      78.810 117.499 138.996  1.00  0.00           H
ATOM   3109  HE1 MET A 523      80.254 120.494 140.814  1.00  0.00           H
ATOM   3110  HE2 MET A 523      78.781 120.090 139.927  1.00  0.00           H
ATOM   3111  HE3 MET A 523      79.466 118.935 141.076  1.00  0.00           H
ATOM   3112  N   LEU A 524      78.780 113.804 138.100  1.00  0.00           N
ATOM   3113  CA  LEU A 524      77.569 113.052 138.441  1.00  0.00           C
ATOM   3114  C   LEU A 524      77.809 111.951 139.488  1.00  0.00           C
```

```
ATOM   3115  O   LEU A 524      76.912 111.708 140.292  1.00  0.00           O
ATOM   3116  CB  LEU A 524      76.919 112.493 137.162  1.00 20.00           C
ATOM   3117  CG  LEU A 524      76.346 113.588 136.234  1.00 20.00           C
ATOM   3118  CD1 LEU A 524      76.077 113.023 134.831  1.00 20.00           C
ATOM   3119  CD2 LEU A 524      75.105 114.282 136.835  1.00 20.00           C
ATOM   3120  H   LEU A 524      79.176 113.634 137.185  1.00  0.00           H
ATOM   3121  HA  LEU A 524      76.866 113.752 138.893  1.00  0.00           H
ATOM   3122  HB2 LEU A 524      77.663 111.905 136.624  1.00  0.00           H
ATOM   3123  HB3 LEU A 524      76.120 111.795 137.418  1.00  0.00           H
ATOM   3124  HG  LEU A 524      77.098 114.366 136.107  1.00  0.00           H
ATOM   3125 HD11 LEU A 524      75.211 113.479 134.351  1.00  0.00           H
ATOM   3126 HD12 LEU A 524      76.936 113.208 134.189  1.00  0.00           H
ATOM   3127 HD13 LEU A 524      75.914 111.944 134.848  1.00  0.00           H
ATOM   3128 HD21 LEU A 524      74.227 114.200 136.195  1.00  0.00           H
ATOM   3129 HD22 LEU A 524      74.824 113.861 137.801  1.00  0.00           H
ATOM   3130 HD23 LEU A 524      75.294 115.345 136.989  1.00  0.00           H
ATOM   3131  N   VAL A 525      79.014 111.353 139.515  1.00  0.00           N
ATOM   3132  CA  VAL A 525      79.453 110.456 140.590  1.00  0.00           C
ATOM   3133  C   VAL A 525      79.699 111.223 141.908  1.00  0.00           C
ATOM   3134  O   VAL A 525      79.392 110.681 142.968  1.00  0.00           O
ATOM   3135  CB  VAL A 525      80.711 109.631 140.183  1.00 20.00           C
ATOM   3136  CG1 VAL A 525      81.461 108.933 141.340  1.00 20.00           C
ATOM   3137  CG2 VAL A 525      80.338 108.567 139.133  1.00 20.00           C
ATOM   3138  H   VAL A 525      79.699 111.566 138.802  1.00  0.00           H
ATOM   3139  HA  VAL A 525      78.642 109.749 140.781  1.00  0.00           H
ATOM   3140  HB  VAL A 525      81.418 110.315 139.712  1.00  0.00           H
ATOM   3141 HG11 VAL A 525      82.272 108.312 140.960  1.00  0.00           H
ATOM   3142 HG12 VAL A 525      81.914 109.645 142.028  1.00  0.00           H
ATOM   3143 HG13 VAL A 525      80.793 108.290 141.914  1.00  0.00           H
ATOM   3144 HG21 VAL A 525      81.228 108.073 138.741  1.00  0.00           H
ATOM   3145 HG22 VAL A 525      79.694 107.800 139.563  1.00  0.00           H
ATOM   3146 HG23 VAL A 525      79.805 108.996 138.286  1.00  0.00           H
ATOM   3147  N   SER A 526      80.162 112.486 141.823  1.00  0.00           N
ATOM   3148  CA  SER A 526      80.252 113.401 142.962  1.00  0.00           C
ATOM   3149  C   SER A 526      78.869 113.757 143.544  1.00  0.00           C
ATOM   3150  O   SER A 526      78.721 113.726 144.763  1.00  0.00           O
ATOM   3151  CB  SER A 526      81.060 114.655 142.574  1.00 20.00           C
ATOM   3152  OG  SER A 526      81.147 115.561 143.651  1.00 20.00           O
ATOM   3153  H   SER A 526      80.391 112.873 140.918  1.00  0.00           H
ATOM   3154  HA  SER A 526      80.809 112.885 143.747  1.00  0.00           H
ATOM   3155  HB2 SER A 526      82.066 114.378 142.267  1.00  0.00           H
ATOM   3156  HB3 SER A 526      80.614 115.181 141.735  1.00  0.00           H
ATOM   3157  HG  SER A 526      81.797 115.226 144.278  1.00  0.00           H
ATOM   3158  N   VAL A 527      77.879 114.039 142.678  1.00 30.00           N
ATOM   3159  CA  VAL A 527      76.487 114.303 143.062  1.00 30.00           C
ATOM   3160  C   VAL A 527      75.832 113.106 143.780  1.00 30.00           C
ATOM   3161  O   VAL A 527      75.191 113.310 144.810  1.00 30.00           O
ATOM   3162  CB  VAL A 527      75.631 114.727 141.830  1.00 20.00           C
ATOM   3163  CG1 VAL A 527      74.097 114.645 142.000  1.00  0.00           C
ATOM   3164  CG2 VAL A 527      76.003 116.148 141.386  1.00  0.00           C
ATOM   3165  H   VAL A 527      78.081 114.072 141.688  1.00  0.00           H
ATOM   3166  HA  VAL A 527      76.502 115.135 143.770  1.00  0.00           H
ATOM   3167  HB  VAL A 527      75.888 114.064 141.006  1.00  0.00           H
ATOM   3168 HG11 VAL A 527      73.586 115.060 141.130  1.00  0.00           H
ATOM   3169 HG12 VAL A 527      73.747 113.618 142.104  1.00  0.00           H
ATOM   3170 HG13 VAL A 527      73.767 115.207 142.874  1.00  0.00           H
ATOM   3171 HG21 VAL A 527      75.671 116.883 142.120  1.00  0.00           H
ATOM   3172 HG22 VAL A 527      77.076 116.277 141.264  1.00  0.00           H
ATOM   3173 HG23 VAL A 527      75.536 116.393 140.432  1.00  0.00           H
ATOM   3174  N   VAL A 528      76.057 111.886 143.260  1.00  0.00           N
ATOM   3175  CA  VAL A 528      75.617 110.629 143.869  1.00  0.00           C
ATOM   3176  C   VAL A 528      76.227 110.405 145.268  1.00  0.00           C
ATOM   3177  O   VAL A 528      75.471 110.127 146.196  1.00  0.00           O
ATOM   3178  CB  VAL A 528      75.906 109.412 142.938  1.00 20.00           C
ATOM   3179  CG1 VAL A 528      75.896 108.021 143.612  1.00 20.00           C
ATOM   3180  CG2 VAL A 528      74.926 109.401 141.751  1.00 20.00           C
ATOM   3181  H   VAL A 528      76.587 111.803 142.403  1.00  0.00           H
ATOM   3182  HA  VAL A 528      74.536 110.703 144.000  1.00  0.00           H
ATOM   3183  HB  VAL A 528      76.904 109.545 142.519  1.00  0.00           H
ATOM   3184 HG11 VAL A 528      75.994 107.227 142.871  1.00  0.00           H
ATOM   3185 HG12 VAL A 528      76.723 107.895 144.311  1.00  0.00           H
```

```
ATOM   3186 HG13 VAL A 528      74.965 107.849 144.154  1.00  0.00           H
ATOM   3187 HG21 VAL A 528      75.192 108.632 141.026  1.00  0.00           H
ATOM   3188 HG22 VAL A 528      73.907 109.205 142.085  1.00  0.00           H
ATOM   3189 HG23 VAL A 528      74.916 110.355 141.224  1.00  0.00           H
ATOM   3190  N   LEU A 529      77.553 110.583 145.402  1.00  0.00           N
ATOM   3191  CA  LEU A 529      78.292 110.457 146.663  1.00  0.00           C
ATOM   3192  C   LEU A 529      77.894 111.504 147.722  1.00  0.00           C
ATOM   3193  O   LEU A 529      77.846 111.158 148.902  1.00  0.00           O
ATOM   3194  CB  LEU A 529      79.811 110.518 146.381  1.00 20.00           C
ATOM   3195  CG  LEU A 529      80.401 109.239 145.744  1.00 20.00           C
ATOM   3196  CD1 LEU A 529      81.836 109.479 145.235  1.00 20.00           C
ATOM   3197  CD2 LEU A 529      80.327 108.020 146.684  1.00 20.00           C
ATOM   3198  H   LEU A 529      78.106 110.816 144.587  1.00  0.00           H
ATOM   3199  HA  LEU A 529      78.052 109.480 147.084  1.00  0.00           H
ATOM   3200  HB2 LEU A 529      80.001 111.375 145.734  1.00  0.00           H
ATOM   3201  HB3 LEU A 529      80.358 110.719 147.304  1.00  0.00           H
ATOM   3202  HG  LEU A 529      79.798 109.001 144.868  1.00  0.00           H
ATOM   3203 HD11 LEU A 529      82.016 108.924 144.315  1.00  0.00           H
ATOM   3204 HD12 LEU A 529      82.026 110.529 145.015  1.00  0.00           H
ATOM   3205 HD13 LEU A 529      82.587 109.164 145.960  1.00  0.00           H
ATOM   3206 HD21 LEU A 529      81.282 107.501 146.774  1.00  0.00           H
ATOM   3207 HD22 LEU A 529      80.026 108.307 147.689  1.00  0.00           H
ATOM   3208 HD23 LEU A 529      79.600 107.293 146.320  1.00  0.00           H
ATOM   3209  N   TYR A 530      77.597 112.741 147.287  1.00  0.00           N
ATOM   3210  CA  TYR A 530      77.195 113.865 148.136  1.00  0.00           C
ATOM   3211  C   TYR A 530      75.867 113.612 148.870  1.00  0.00           C
ATOM   3212  O   TYR A 530      75.818 113.778 150.088  1.00  0.00           O
ATOM   3213  CB  TYR A 530      77.162 115.154 147.286  1.00 20.00           C
ATOM   3214  CG  TYR A 530      76.774 116.431 148.011  1.00 20.00           C
ATOM   3215  CD1 TYR A 530      77.707 117.069 148.854  1.00 20.00           C
ATOM   3216  CD2 TYR A 530      75.498 117.004 147.825  1.00 20.00           C
ATOM   3217  CE1 TYR A 530      77.378 118.282 149.486  1.00 20.00           C
ATOM   3218  CE2 TYR A 530      75.165 118.216 148.462  1.00 20.00           C
ATOM   3219  CZ  TYR A 530      76.109 118.861 149.286  1.00 20.00           C
ATOM   3220  OH  TYR A 530      75.798 120.041 149.897  1.00 20.00           O
ATOM   3221  H   TYR A 530      77.675 112.944 146.298  1.00  0.00           H
ATOM   3222  HA  TYR A 530      77.971 113.986 148.895  1.00  0.00           H
ATOM   3223  HB2 TYR A 530      78.147 115.321 146.848  1.00  0.00           H
ATOM   3224  HB3 TYR A 530      76.487 115.020 146.440  1.00  0.00           H
ATOM   3225  HD1 TYR A 530      78.683 116.635 149.009  1.00  0.00           H
ATOM   3226  HD2 TYR A 530      74.775 116.521 147.185  1.00  0.00           H
ATOM   3227  HE1 TYR A 530      78.106 118.766 150.118  1.00  0.00           H
ATOM   3228  HE2 TYR A 530      74.187 118.648 148.309  1.00  0.00           H
ATOM   3229  HH  TYR A 530      74.906 120.342 149.709  1.00  0.00           H
ATOM   3230  N   PHE A 531      74.836 113.180 148.121  1.00  0.00           N
ATOM   3231  CA  PHE A 531      73.519 112.832 148.662  1.00  0.00           C
ATOM   3232  C   PHE A 531      73.455 111.445 149.333  1.00  0.00           C
ATOM   3233  O   PHE A 531      72.560 111.231 150.149  1.00  0.00           O
ATOM   3234  CB  PHE A 531      72.433 113.010 147.580  1.00 20.00           C
ATOM   3235  CG  PHE A 531      72.173 114.458 147.192  1.00 20.00           C
ATOM   3236  CD1 PHE A 531      71.636 115.360 148.134  1.00 20.00           C
ATOM   3237  CD2 PHE A 531      72.527 114.938 145.913  1.00 20.00           C
ATOM   3238  CE1 PHE A 531      71.463 116.697 147.800  1.00 20.00           C
ATOM   3239  CE2 PHE A 531      72.343 116.278 145.596  1.00 20.00           C
ATOM   3240  CZ  PHE A 531      71.817 117.154 146.537  1.00 20.00           C
ATOM   3241  H   PHE A 531      74.955 113.070 147.123  1.00  0.00           H
ATOM   3242  HA  PHE A 531      73.295 113.538 149.462  1.00  0.00           H
ATOM   3243  HB2 PHE A 531      72.699 112.439 146.689  1.00  0.00           H
ATOM   3244  HB3 PHE A 531      71.488 112.592 147.932  1.00  0.00           H
ATOM   3245  HD1 PHE A 531      71.364 115.016 149.121  1.00  0.00           H
ATOM   3246  HD2 PHE A 531      72.932 114.268 145.171  1.00  0.00           H
ATOM   3247  HE1 PHE A 531      71.057 117.385 148.526  1.00  0.00           H
ATOM   3248  HE2 PHE A 531      72.615 116.641 144.616  1.00  0.00           H
ATOM   3249  HZ  PHE A 531      71.682 118.195 146.284  1.00  0.00           H
ATOM   3250  N   SER A 532      74.425 110.557 149.038  1.00 30.00           N
ATOM   3251  CA  SER A 532      74.623 109.263 149.710  1.00 30.00           C
ATOM   3252  C   SER A 532      75.313 109.392 151.091  1.00 30.00           C
ATOM   3253  O   SER A 532      75.553 108.373 151.738  1.00 30.00           O
ATOM   3254  CB  SER A 532      75.390 108.321 148.753  1.00 20.00           C
ATOM   3255  OG  SER A 532      75.511 107.003 149.251  1.00 20.00           O
ATOM   3256  H   SER A 532      75.116 110.799 148.342  1.00  0.00           H
```

```
ATOM   3257  HA  SER A 532      73.640 108.823 149.889  1.00  0.00           H
ATOM   3258  HB2 SER A 532      74.863 108.248 147.802  1.00  0.00           H
ATOM   3259  HB3 SER A 532      76.386 108.709 148.544  1.00  0.00           H
ATOM   3260  HG  SER A 532      75.925 107.045 150.118  1.00  0.00           H
ATOM   3261  N   GLN A 533      75.611 110.632 151.523  1.00 30.00           N
ATOM   3262  CA  GLN A 533      76.224 111.003 152.802  1.00 30.00           C
ATOM   3263  C   GLN A 533      77.687 110.535 152.936  1.00 30.00           C
ATOM   3264  O   GLN A 533      78.129 110.232 154.044  1.00 30.00           O
ATOM   3265  CB  GLN A 533      75.354 110.575 154.012  1.00 20.00           C
ATOM   3266  CG  GLN A 533      73.901 111.080 153.945  1.00 20.00           C
ATOM   3267  CD  GLN A 533      73.132 110.893 155.255  1.00 20.00           C
ATOM   3268  NE2 GLN A 533      72.069 111.677 155.442  1.00 20.00           N
ATOM   3269  OE1 GLN A 533      73.474 110.051 156.082  1.00 20.00           O
ATOM   3270  H   GLN A 533      75.386 111.410 150.919  1.00  0.00           H
ATOM   3271  HA  GLN A 533      76.267 112.091 152.789  1.00  0.00           H
ATOM   3272  HB2 GLN A 533      75.354 109.489 154.116  1.00  0.00           H
ATOM   3273  HB3 GLN A 533      75.814 110.962 154.923  1.00  0.00           H
ATOM   3274  HG2 GLN A 533      73.905 112.137 153.703  1.00  0.00           H
ATOM   3275  HG3 GLN A 533      73.356 110.577 153.146  1.00  0.00           H
ATOM   3276 HE21 GLN A 533      71.522 111.589 156.285  1.00  0.00           H
ATOM   3277 HE22 GLN A 533      71.798 112.352 154.741  1.00  0.00           H
ATOM   3278  N   ARG A 534      78.418 110.504 151.811  1.00 30.00           N
ATOM   3279  CA  ARG A 534      79.844 110.200 151.775  1.00 30.00           C
ATOM   3280  C   ARG A 534      80.634 111.502 151.617  1.00 30.00           C
ATOM   3281  O   ARG A 534      80.343 112.290 150.716  1.00 30.00           O
ATOM   3282  CB  ARG A 534      80.158 109.229 150.615  1.00 20.00           C
ATOM   3283  CG  ARG A 534      79.352 107.913 150.629  1.00 20.00           C
ATOM   3284  CD  ARG A 534      79.668 106.973 151.804  1.00 20.00           C
ATOM   3285  NE  ARG A 534      81.032 106.429 151.722  1.00 20.00           N
ATOM   3286  CZ  ARG A 534      81.605 105.612 152.621  1.00 20.00           C
ATOM   3287  NH1 ARG A 534      80.942 105.192 153.708  1.00 20.00           N
ATOM   3288  NH2 ARG A 534      82.866 105.213 152.421  1.00 20.00           N1+
ATOM   3289  H   ARG A 534      78.001 110.781 150.931  1.00  0.00           H
ATOM   3290  HA  ARG A 534      80.160 109.727 152.706  1.00  0.00           H
ATOM   3291  HB2 ARG A 534      81.224 108.997 150.620  1.00  0.00           H
ATOM   3292  HB3 ARG A 534      79.973 109.730 149.664  1.00  0.00           H
ATOM   3293  HG2 ARG A 534      78.277 108.079 150.543  1.00  0.00           H
ATOM   3294  HG3 ARG A 534      79.628 107.379 149.720  1.00  0.00           H
ATOM   3295  HD2 ARG A 534      79.642 107.540 152.734  1.00  0.00           H
ATOM   3296  HD3 ARG A 534      78.910 106.195 151.902  1.00  0.00           H
ATOM   3297  HE  ARG A 534      81.587 106.705 150.920  1.00  0.00           H
ATOM   3298 HH11 ARG A 534      81.385 104.575 154.373  1.00  0.00           H
ATOM   3299 HH12 ARG A 534      79.990 105.489 153.863  1.00  0.00           H
ATOM   3300 HH21 ARG A 534      83.333 104.596 153.068  1.00  0.00           H
ATOM   3301 HH22 ARG A 534      83.370 105.557 151.605  1.00  0.00           H
ATOM   3302  N   LYS A 535      81.664 111.661 152.466  1.00 30.00           N
ATOM   3303  CA  LYS A 535      82.680 112.715 152.375  1.00 30.00           C
ATOM   3304  C   LYS A 535      83.542 112.589 151.098  1.00 30.00           C
ATOM   3305  O   LYS A 535      84.088 113.589 150.636  1.00 30.00           O
ATOM   3306  CB  LYS A 535      83.528 112.674 153.666  1.00 20.00           C
ATOM   3307  CG  LYS A 535      84.550 113.819 153.819  1.00 20.00           C
ATOM   3308  CD  LYS A 535      85.344 113.778 155.136  1.00 20.00           C
ATOM   3309  CE  LYS A 535      84.508 114.111 156.384  1.00 20.00           C
ATOM   3310  NZ  LYS A 535      85.341 114.129 157.598  1.00 20.00           N1+
ATOM   3311  H   LYS A 535      81.816 110.969 153.185  1.00  0.00           H
ATOM   3312  HA  LYS A 535      82.162 113.676 152.334  1.00  0.00           H
ATOM   3313  HB2 LYS A 535      82.848 112.709 154.516  1.00  0.00           H
ATOM   3314  HB3 LYS A 535      84.046 111.717 153.734  1.00  0.00           H
ATOM   3315  HG2 LYS A 535      85.266 113.784 152.999  1.00  0.00           H
ATOM   3316  HG3 LYS A 535      84.040 114.780 153.730  1.00  0.00           H
ATOM   3317  HD2 LYS A 535      85.795 112.791 155.251  1.00  0.00           H
ATOM   3318  HD3 LYS A 535      86.177 114.478 155.062  1.00  0.00           H
ATOM   3319  HE2 LYS A 535      84.047 115.092 156.273  1.00  0.00           H
ATOM   3320  HE3 LYS A 535      83.705 113.388 156.525  1.00  0.00           H
ATOM   3321  HZ1 LYS A 535      86.066 114.825 157.496  1.00  0.00           H
ATOM   3322  HZ2 LYS A 535      84.769 114.357 158.398  1.00  0.00           H
ATOM   3323  HZ3 LYS A 535      85.764 113.222 157.735  1.00  0.00           H
ATOM   3324  N   GLU A 536      83.596 111.367 150.535  1.00 30.00           N
ATOM   3325  CA  GLU A 536      84.282 110.958 149.308  1.00 30.00           C
ATOM   3326  C   GLU A 536      83.752 111.587 148.004  1.00 30.00           C
ATOM   3327  O   GLU A 536      84.338 111.332 146.951  1.00 30.00           O
```

```
ATOM   3328  CB  GLU A 536      84.267 109.417 149.234  1.00 20.00           C
ATOM   3329  CG  GLU A 536      84.935 108.738 150.450  1.00  0.00           C
ATOM   3330  CD  GLU A 536      84.864 107.213 150.393  1.00  0.00           C
ATOM   3331  OE1 GLU A 536      83.750 106.698 150.148  1.00  0.00           O
ATOM   3332  OE2 GLU A 536      85.912 106.582 150.647  1.00  0.00           O1-
ATOM   3333  H   GLU A 536      83.100 110.620 150.999  1.00  0.00           H
ATOM   3334  HA  GLU A 536      85.312 111.293 149.390  1.00  0.00           H
ATOM   3335  HB2 GLU A 536      83.234 109.076 149.140  1.00  0.00           H
ATOM   3336  HB3 GLU A 536      84.773 109.083 148.327  1.00  0.00           H
ATOM   3337  HG2 GLU A 536      85.978 109.047 150.522  1.00  0.00           H
ATOM   3338  HG3 GLU A 536      84.457 109.044 151.380  1.00  0.00           H
ATOM   3339  N   TYR A 537      82.705 112.429 148.086  1.00 30.00           N
ATOM   3340  CA  TYR A 537      82.207 113.263 146.991  1.00 30.00           C
ATOM   3341  C   TYR A 537      83.282 114.223 146.440  1.00 30.00           C
ATOM   3342  O   TYR A 537      83.316 114.444 145.230  1.00 30.00           O
ATOM   3343  CB  TYR A 537      80.935 114.016 147.457  1.00 20.00           C
ATOM   3344  CG  TYR A 537      81.140 115.372 148.121  1.00 20.00           C
ATOM   3345  CD1 TYR A 537      81.432 115.454 149.498  1.00 20.00           C
ATOM   3346  CD2 TYR A 537      81.097 116.552 147.346  1.00 20.00           C
ATOM   3347  CE1 TYR A 537      81.719 116.700 150.088  1.00 20.00           C
ATOM   3348  CE2 TYR A 537      81.392 117.797 147.933  1.00 20.00           C
ATOM   3349  CZ  TYR A 537      81.716 117.870 149.301  1.00 20.00           C
ATOM   3350  OH  TYR A 537      82.036 119.072 149.862  1.00 20.00           O
ATOM   3351  H   TYR A 537      82.273 112.582 148.987  1.00  0.00           H
ATOM   3352  HA  TYR A 537      81.929 112.596 146.175  1.00  0.00           H
ATOM   3353  HB2 TYR A 537      80.319 113.378 148.092  1.00  0.00           H
ATOM   3354  HB3 TYR A 537      80.330 114.208 146.575  1.00  0.00           H
ATOM   3355  HD1 TYR A 537      81.462 114.560 150.100  1.00  0.00           H
ATOM   3356  HD2 TYR A 537      80.873 116.504 146.290  1.00  0.00           H
ATOM   3357  HE1 TYR A 537      81.958 116.751 151.140  1.00  0.00           H
ATOM   3358  HE2 TYR A 537      81.382 118.692 147.328  1.00  0.00           H
ATOM   3359  HH  TYR A 537      82.190 119.009 150.808  1.00  0.00           H
ATOM   3360  N   VAL A 538      84.130 114.759 147.339  1.00 30.00           N
ATOM   3361  CA  VAL A 538      85.178 115.732 147.036  1.00 30.00           C
ATOM   3362  C   VAL A 538      86.287 115.167 146.130  1.00 30.00           C
ATOM   3363  O   VAL A 538      86.791 115.915 145.299  1.00 30.00           O
ATOM   3364  CB  VAL A 538      85.811 116.315 148.332  1.00 20.00           C
ATOM   3365  CG1 VAL A 538      86.649 115.307 149.143  1.00 20.00           C
ATOM   3366  CG2 VAL A 538      86.657 117.574 148.071  1.00 20.00           C
ATOM   3367  H   VAL A 538      84.028 114.514 148.315  1.00  0.00           H
ATOM   3368  HA  VAL A 538      84.696 116.549 146.498  1.00  0.00           H
ATOM   3369  HB  VAL A 538      84.981 116.621 148.973  1.00  0.00           H
ATOM   3370 HG11 VAL A 538      86.877 115.703 150.132  1.00  0.00           H
ATOM   3371 HG12 VAL A 538      86.122 114.365 149.279  1.00  0.00           H
ATOM   3372 HG13 VAL A 538      87.600 115.082 148.660  1.00  0.00           H
ATOM   3373 HG21 VAL A 538      87.047 117.967 149.008  1.00  0.00           H
ATOM   3374 HG22 VAL A 538      87.518 117.374 147.433  1.00  0.00           H
ATOM   3375 HG23 VAL A 538      86.070 118.363 147.602  1.00  0.00           H
ATOM   3376  N   ALA A 539      86.606 113.867 146.265  1.00  0.00           N
ATOM   3377  CA  ALA A 539      87.574 113.159 145.429  1.00  0.00           C
ATOM   3378  C   ALA A 539      87.159 113.114 143.950  1.00  0.00           C
ATOM   3379  O   ALA A 539      87.986 113.396 143.086  1.00  0.00           O
ATOM   3380  CB  ALA A 539      87.777 111.742 145.983  1.00  0.00           C
ATOM   3381  H   ALA A 539      86.131 113.313 146.963  1.00  0.00           H
ATOM   3382  HA  ALA A 539      88.524 113.692 145.494  1.00  0.00           H
ATOM   3383  HB1 ALA A 539      88.481 111.175 145.373  1.00  0.00           H
ATOM   3384  HB2 ALA A 539      88.170 111.775 146.999  1.00  0.00           H
ATOM   3385  HB3 ALA A 539      86.843 111.180 146.008  1.00  0.00           H
ATOM   3386  N   SER A 540      85.874 112.812 143.700  1.00  0.00           N
ATOM   3387  CA  SER A 540      85.283 112.774 142.365  1.00  0.00           C
ATOM   3388  C   SER A 540      85.051 114.183 141.777  1.00  0.00           C
ATOM   3389  O   SER A 540      85.210 114.353 140.569  1.00  0.00           O
ATOM   3390  CB  SER A 540      83.999 111.924 142.428  1.00 20.00           C
ATOM   3391  OG  SER A 540      83.503 111.637 141.139  1.00  0.00           O
ATOM   3392  H   SER A 540      85.254 112.608 144.471  1.00  0.00           H
ATOM   3393  HA  SER A 540      85.988 112.258 141.708  1.00  0.00           H
ATOM   3394  HB2 SER A 540      84.200 110.972 142.922  1.00  0.00           H
ATOM   3395  HB3 SER A 540      83.226 112.421 143.014  1.00  0.00           H
ATOM   3396  HG  SER A 540      83.178 112.450 140.737  1.00  0.00           H
ATOM   3397  N   MET A 541      84.731 115.172 142.638  1.00  0.00           N
ATOM   3398  CA  MET A 541      84.590 116.590 142.279  1.00  0.00           C
```

```
ATOM   3399  C   MET A 541      85.914 117.206 141.800  1.00  0.00           C
ATOM   3400  O   MET A 541      85.914 117.977 140.846  1.00  0.00           O
ATOM   3401  CB  MET A 541      84.047 117.389 143.486  1.00 20.00           C
ATOM   3402  CG  MET A 541      83.591 118.817 143.136  1.00 20.00           C
ATOM   3403  SD  MET A 541      82.726 119.704 144.463  1.00 20.00           S
ATOM   3404  CE  MET A 541      84.048 119.862 145.692  1.00 20.00           C
ATOM   3405  H   MET A 541      84.610 114.951 143.617  1.00  0.00           H
ATOM   3406  HA  MET A 541      83.865 116.647 141.465  1.00  0.00           H
ATOM   3407  HB2 MET A 541      83.220 116.864 143.951  1.00  0.00           H
ATOM   3408  HB3 MET A 541      84.815 117.443 144.256  1.00  0.00           H
ATOM   3409  HG2 MET A 541      84.439 119.427 142.823  1.00  0.00           H
ATOM   3410  HG3 MET A 541      82.912 118.773 142.284  1.00  0.00           H
ATOM   3411  HE1 MET A 541      83.721 120.499 146.514  1.00  0.00           H
ATOM   3412  HE2 MET A 541      84.304 118.885 146.100  1.00  0.00           H
ATOM   3413  HE3 MET A 541      84.940 120.301 145.246  1.00  0.00           H
ATOM   3414  N   VAL A 542      86.997 116.847 142.496  1.00  0.00           N
ATOM   3415  CA  VAL A 542      88.368 117.260 142.235  1.00  0.00           C
ATOM   3416  C   VAL A 542      88.880 116.772 140.867  1.00  0.00           C
ATOM   3417  O   VAL A 542      89.459 117.569 140.128  1.00  0.00           O
ATOM   3418  CB  VAL A 542      89.257 116.811 143.432  1.00 20.00           C
ATOM   3419  CG1 VAL A 542      90.723 116.431 143.171  1.00 20.00           C
ATOM   3420  CG2 VAL A 542      89.158 117.884 144.533  1.00 20.00           C
ATOM   3421  H   VAL A 542      86.880 116.283 143.331  1.00  0.00           H
ATOM   3422  HA  VAL A 542      88.369 118.351 142.189  1.00  0.00           H
ATOM   3423  HB  VAL A 542      88.825 115.897 143.837  1.00  0.00           H
ATOM   3424 HG11 VAL A 542      91.181 116.069 144.091  1.00  0.00           H
ATOM   3425 HG12 VAL A 542      90.824 115.630 142.439  1.00  0.00           H
ATOM   3426 HG13 VAL A 542      91.308 117.283 142.840  1.00  0.00           H
ATOM   3427 HG21 VAL A 542      89.643 117.582 145.454  1.00  0.00           H
ATOM   3428 HG22 VAL A 542      89.623 118.816 144.212  1.00  0.00           H
ATOM   3429 HG23 VAL A 542      88.123 118.115 144.784  1.00  0.00           H
ATOM   3430  N   PHE A 543      88.563 115.521 140.498  1.00  0.00           N
ATOM   3431  CA  PHE A 543      88.832 114.995 139.157  1.00  0.00           C
ATOM   3432  C   PHE A 543      88.068 115.728 138.039  1.00  0.00           C
ATOM   3433  O   PHE A 543      88.654 115.986 136.987  1.00  0.00           O
ATOM   3434  CB  PHE A 543      88.579 113.476 139.088  1.00 20.00           C
ATOM   3435  CG  PHE A 543      89.601 112.613 139.808  1.00  0.00           C
ATOM   3436  CD1 PHE A 543      89.200 111.733 140.834  1.00  0.00           C
ATOM   3437  CD2 PHE A 543      90.963 112.656 139.438  1.00  0.00           C
ATOM   3438  CE1 PHE A 543      90.141 110.951 141.490  1.00  0.00           C
ATOM   3439  CE2 PHE A 543      91.887 111.858 140.099  1.00  0.00           C
ATOM   3440  CZ  PHE A 543      91.480 111.017 141.127  1.00  0.00           C
ATOM   3441  H   PHE A 543      88.144 114.891 141.171  1.00  0.00           H
ATOM   3442  HA  PHE A 543      89.890 115.171 138.950  1.00  0.00           H
ATOM   3443  HB2 PHE A 543      88.573 113.154 138.046  1.00  0.00           H
ATOM   3444  HB  PHE A 543      87.581 113.253 139.467  1.00  0.00           H
ATOM   3445  HD1 PHE A 543      88.163 111.668 141.125  1.00  0.00           H
ATOM   3446  HD2 PHE A 543      91.296 113.306 138.642  1.00  0.00           H
ATOM   3447  HE1 PHE A 543      89.829 110.291 142.286  1.00  0.00           H
ATOM   3448  HE2 PHE A 543      92.929 111.901 139.819  1.00  0.00           H
ATOM   3449  HZ  PHE A 543      92.208 110.411 141.646  1.00  0.00           H
ATOM   3450  N   SER A 544      86.805 116.104 138.295  1.00  0.00           N
ATOM   3451  CA  SER A 544      86.005 116.911 137.364  1.00  0.00           C
ATOM   3452  C   SER A 544      86.567 118.340 137.215  1.00  0.00           C
ATOM   3453  O   SER A 544      86.563 118.878 136.110  1.00  0.00           O
ATOM   3454  CB  SER A 544      84.531 116.934 137.829  1.00 20.00           C
ATOM   3455  OG  SER A 544      83.698 117.625 136.920  1.00 20.00           O
ATOM   3456  H   SER A 544      86.424 115.980 139.223  1.00  0.00           H
ATOM   3457  HA  SER A 544      86.039 116.432 136.383  1.00  0.00           H
ATOM   3458  HB2 SER A 544      84.142 115.926 137.929  1.00  0.00           H
ATOM   3459  HB3 SER A 544      84.432 117.396 138.809  1.00  0.00           H
ATOM   3460  HG  SER A 544      83.493 117.036 136.186  1.00  0.00           H
ATOM   3461  N   LEU A 545      87.042 118.918 138.331  1.00  0.00           N
ATOM   3462  CA  LEU A 545      87.570 120.277 138.427  1.00  0.00           C
ATOM   3463  C   LEU A 545      88.930 120.422 137.728  1.00  0.00           C
ATOM   3464  O   LEU A 545      89.080 121.339 136.922  1.00  0.00           O
ATOM   3465  CB  LEU A 545      87.576 120.694 139.915  1.00 20.00           C
ATOM   3466  CG  LEU A 545      87.812 122.193 140.200  1.00 20.00           C
ATOM   3467  CD1 LEU A 545      87.144 122.599 141.529  1.00 20.00           C
ATOM   3468  CD2 LEU A 545      89.306 122.574 140.175  1.00 20.00           C
ATOM   3469  H   LEU A 545      86.982 118.412 139.205  1.00  0.00           H
```

```
ATOM   3470  HA  LEU A 545      86.874 120.934 137.905  1.00  0.00           H
ATOM   3471  HB2 LEU A 545      86.585 120.451 140.301  1.00  0.00           H
ATOM   3472  HB3 LEU A 545      88.269 120.080 140.492  1.00  0.00           H
ATOM   3473  HG  LEU A 545      87.314 122.769 139.417  1.00  0.00           H
ATOM   3474 HD11 LEU A 545      87.727 123.323 142.099  1.00  0.00           H
ATOM   3475 HD12 LEU A 545      86.169 123.052 141.342  1.00  0.00           H
ATOM   3476 HD13 LEU A 545      86.979 121.742 142.183  1.00  0.00           H
ATOM   3477 HD21 LEU A 545      89.627 123.070 141.089  1.00  0.00           H
ATOM   3478 HD22 LEU A 545      89.957 121.708 140.054  1.00  0.00           H
ATOM   3479 HD23 LEU A 545      89.514 123.253 139.349  1.00  0.00           H
ATOM   3480  N   ALA A 546      89.851 119.494 138.024  1.00  0.00           N
ATOM   3481  CA  ALA A 546      91.169 119.422 137.405  1.00  0.00           C
ATOM   3482  C   ALA A 546      91.044 119.300 135.881  1.00  0.00           C
ATOM   3483  O   ALA A 546      91.568 120.147 135.154  1.00  0.00           O
ATOM   3484  CB  ALA A 546      91.950 118.235 137.991  1.00 20.00           C
ATOM   3485  H   ALA A 546      89.636 118.792 138.729  1.00  0.00           H
ATOM   3486  HA  ALA A 546      91.700 120.346 137.636  1.00  0.00           H
ATOM   3487  HB1 ALA A 546      91.481 117.278 137.758  1.00  0.00           H
ATOM   3488  HB2 ALA A 546      92.967 118.213 137.601  1.00  0.00           H
ATOM   3489  HB3 ALA A 546      92.027 118.301 139.074  1.00  0.00           H
ATOM   3490  N   MET A 547      90.269 118.294 135.443  1.00  0.00           N
ATOM   3491  CA  MET A 547      89.994 118.006 134.040  1.00  0.00           C
ATOM   3492  C   MET A 547      89.307 119.165 133.298  1.00  0.00           C
ATOM   3493  O   MET A 547      89.658 119.400 132.144  1.00  0.00           O
ATOM   3494  CB  MET A 547      89.212 116.683 133.911  1.00 20.00           C
ATOM   3495  CG  MET A 547      88.903 116.268 132.461  1.00 20.00           C
ATOM   3496  SD  MET A 547      88.255 114.587 132.292  1.00 20.00           S
ATOM   3497  CE  MET A 547      89.797 113.639 132.391  1.00 20.00           C
ATOM   3498  H   MET A 547      89.824 117.685 136.121  1.00  0.00           H
ATOM   3499  HA  MET A 547      90.959 117.847 133.558  1.00  0.00           H
ATOM   3500  HB2 MET A 547      89.766 115.886 134.407  1.00  0.00           H
ATOM   3501  HB3 MET A 547      88.267 116.772 134.449  1.00  0.00           H
ATOM   3502  HG2 MET A 547      88.160 116.942 132.041  1.00  0.00           H
ATOM   3503  HG3 MET A 547      89.789 116.366 131.832  1.00  0.00           H
ATOM   3504  HE1 MET A 547      89.595 112.577 132.260  1.00  0.00           H
ATOM   3505  HE2 MET A 547      90.489 113.953 131.608  1.00  0.00           H
ATOM   3506  HE3 MET A 547      90.278 113.780 133.358  1.00  0.00           H
ATOM   3507  N   GLY A 548      88.396 119.891 133.963  1.00  0.00           N
ATOM   3508  CA  GLY A 548      87.624 120.979 133.368  1.00  0.00           C
ATOM   3509  C   GLY A 548      88.521 122.129 132.892  1.00  0.00           C
ATOM   3510  O   GLY A 548      88.267 122.690 131.826  1.00  0.00           O
ATOM   3511  H   GLY A 548      88.269 119.727 134.953  1.00  0.00           H
ATOM   3512  HA2 GLY A 548      87.055 120.594 132.524  1.00  0.00           H
ATOM   3513  HA3 GLY A 548      86.908 121.346 134.102  1.00  0.00           H
ATOM   3514  N   TRP A 549      89.555 122.469 133.671  1.00  0.00           N
ATOM   3515  CA  TRP A 549      90.532 123.479 133.281  1.00  0.00           C
ATOM   3516  C   TRP A 549      91.442 122.976 132.153  1.00  0.00           C
ATOM   3517  O   TRP A 549      91.710 123.749 131.238  1.00  0.00           O
ATOM   3518  CB  TRP A 549      91.321 123.985 134.493  1.00 20.00           C
ATOM   3519  CG  TRP A 549      90.550 124.860 135.436  1.00 20.00           C
ATOM   3520  CD1 TRP A 549      90.062 124.499 136.643  1.00 20.00           C
ATOM   3521  CD2 TRP A 549      90.130 126.245 135.240  1.00 20.00           C
ATOM   3522  CE2 TRP A 549      89.380 126.659 136.382  1.00 20.00           C
ATOM   3523  CE3 TRP A 549      90.315 127.200 134.214  1.00 20.00           C
ATOM   3524  NE1 TRP A 549      89.401 125.563 137.217  1.00 20.00           N
ATOM   3525  CZ2 TRP A 549      88.821 127.945 136.486  1.00 20.00           C
ATOM   3526  CZ3 TRP A 549      89.783 128.501 134.322  1.00 20.00           C
ATOM   3527  CH2 TRP A 549      89.026 128.871 135.449  1.00 20.00           C
ATOM   3528  H   TRP A 549      89.704 121.984 134.549  1.00  0.00           H
ATOM   3529  HA  TRP A 549      89.998 124.339 132.868  1.00  0.00           H
ATOM   3530  HB2 TRP A 549      91.723 123.132 135.033  1.00  0.00           H
ATOM   3531  HB3 TRP A 549      92.178 124.571 134.157  1.00  0.00           H
ATOM   3532  HD1 TRP A 549      90.204 123.529 137.096  1.00  0.00           H
ATOM   3533  HE1 TRP A 549      89.049 125.524 138.168  1.00  0.00           H
ATOM   3534  HE3 TRP A 549      90.883 126.932 133.335  1.00  0.00           H
ATOM   3535  HZ2 TRP A 549      88.247 128.222 137.355  1.00  0.00           H
ATOM   3536  HZ3 TRP A 549      89.948 129.214 133.526  1.00  0.00           H
ATOM   3537  HH2 TRP A 549      88.613 129.866 135.523  1.00  0.00           H
ATOM   3538  N   THR A 550      91.814 121.685 132.154  1.00  0.00           N
ATOM   3539  CA  THR A 550      92.500 121.076 131.008  1.00  0.00           C
ATOM   3540  C   THR A 550      91.640 121.147 129.722  1.00  0.00           C
```

```
ATOM   3541  O   THR A 550      92.138 121.541 128.669  1.00  0.00           O
ATOM   3542  CB  THR A 550      92.979 119.613 131.279  1.00 20.00           C
ATOM   3543  CG2 THR A 550      93.582 119.369 132.667  1.00 20.00           C
ATOM   3544  OG1 THR A 550      92.072 118.566 130.963  1.00 20.00           O
ATOM   3545  H   THR A 550      91.548 121.079 132.920  1.00  0.00           H
ATOM   3546  HA  THR A 550      93.391 121.676 130.816  1.00  0.00           H
ATOM   3547  HB  THR A 550      93.800 119.450 130.586  1.00  0.00           H
ATOM   3548  HG1 THR A 550      91.185 118.787 131.296  1.00  0.00           H
ATOM   3549 HG21 THR A 550      94.264 118.522 132.650  1.00  0.00           H
ATOM   3550 HG22 THR A 550      94.136 120.230 133.039  1.00  0.00           H
ATOM   3551 HG23 THR A 550      92.817 119.129 133.393  1.00  0.00           H
ATOM   3552  N   ASN A 551      90.336 120.868 129.861  1.00  0.00           N
ATOM   3553  CA  ASN A 551      89.358 120.836 128.772  1.00  0.00           C
ATOM   3554  C   ASN A 551      89.103 122.217 128.154  1.00  0.00           C
ATOM   3555  O   ASN A 551      88.650 122.284 127.014  1.00  0.00           O
ATOM   3556  CB  ASN A 551      88.031 120.193 129.251  1.00 20.00           C
ATOM   3557  CG  ASN A 551      88.081 118.677 129.456  1.00 20.00           C
ATOM   3558  ND2 ASN A 551      86.985 118.076 129.897  1.00 20.00           N
ATOM   3559  OD1 ASN A 551      89.096 118.033 129.215  1.00 20.00           O
ATOM   3560  H   ASN A 551      90.005 120.531 130.761  1.00  0.00           H
ATOM   3561  HA  ASN A 551      89.816 120.199 128.019  1.00  0.00           H
ATOM   3562  HB2 ASN A 551      87.670 120.673 130.158  1.00  0.00           H
ATOM   3563  HB3 ASN A 551      87.265 120.358 128.494  1.00  0.00           H
ATOM   3564 HD21 ASN A 551      86.960 117.068 129.990  1.00  0.00           H
ATOM   3565 HD22 ASN A 551      86.158 118.608 130.180  1.00  0.00           H
ATOM   3566  N   MET A 552      89.437 123.300 128.869  1.00 30.00           N
ATOM   3567  CA  MET A 552      89.316 124.673 128.381  1.00 30.00           C
ATOM   3568  C   MET A 552      90.176 124.962 127.143  1.00 30.00           C
ATOM   3569  O   MET A 552      89.766 125.770 126.309  1.00 30.00           O
ATOM   3570  CB  MET A 552      89.610 125.652 129.533  1.00 20.00           C
ATOM   3571  CG  MET A 552      89.331 127.125 129.196  1.00 20.00           C
ATOM   3572  SD  MET A 552      89.437 128.245 130.613  1.00 20.00           S
ATOM   3573  CE  MET A 552      88.018 127.669 131.579  1.00 20.00           C
ATOM   3574  H   MET A 552      89.850 123.168 129.784  1.00  0.00           H
ATOM   3575  HA  MET A 552      88.281 124.812 128.065  1.00  0.00           H
ATOM   3576  HB2 MET A 552      89.044 125.355 130.414  1.00  0.00           H
ATOM   3577  HB3 MET A 552      90.659 125.566 129.815  1.00  0.00           H
ATOM   3578  HG2 MET A 552      90.048 127.481 128.456  1.00  0.00           H
ATOM   3579  HG3 MET A 552      88.344 127.235 128.745  1.00  0.00           H
ATOM   3580  HE1 MET A 552      87.798 128.385 132.369  1.00  0.00           H
ATOM   3581  HE2 MET A 552      87.131 127.559 130.954  1.00  0.00           H
ATOM   3582  HE3 MET A 552      88.243 126.713 132.048  1.00  0.00           H
ATOM   3583  N   LEU A 553      91.317 124.272 127.000  1.00 30.00           N
ATOM   3584  CA  LEU A 553      92.187 124.445 125.840  1.00 30.00           C
ATOM   3585  C   LEU A 553      91.646 123.781 124.556  1.00 30.00           C
ATOM   3586  O   LEU A 553      92.224 124.036 123.498  1.00 30.00           O
ATOM   3587  CB  LEU A 553      93.660 124.149 126.199  1.00 20.00           C
ATOM   3588  CG  LEU A 553      94.119 122.682 126.102  1.00 20.00           C
ATOM   3589  CD1 LEU A 553      94.617 122.305 124.691  1.00 20.00           C
ATOM   3590  CD2 LEU A 553      95.207 122.410 127.148  1.00 20.00           C
ATOM   3591  H   LEU A 553      91.582 123.568 127.681  1.00  0.00           H
ATOM   3592  HA  LEU A 553      92.179 125.513 125.614  1.00  0.00           H
ATOM   3593  HB2 LEU A 553      93.827 124.519 127.211  1.00  0.00           H
ATOM   3594  HB3 LEU A 553      94.312 124.769 125.580  1.00  0.00           H
ATOM   3595  HG  LEU A 553      93.272 122.039 126.340  1.00  0.00           H
ATOM   3596 HD11 LEU A 553      95.564 121.766 124.710  1.00  0.00           H
ATOM   3597 HD12 LEU A 553      93.893 121.668 124.184  1.00  0.00           H
ATOM   3598 HD13 LEU A 553      94.770 123.179 124.058  1.00  0.00           H
ATOM   3599 HD21 LEU A 553      94.759 122.260 128.130  1.00  0.00           H
ATOM   3600 HD22 LEU A 553      95.778 121.515 126.907  1.00  0.00           H
ATOM   3601 HD23 LEU A 553      95.912 123.238 127.236  1.00  0.00           H
ATOM   3602  N   TYR A 554      90.490 123.084 124.609  1.00 30.00           N
ATOM   3603  CA  TYR A 554      89.673 122.790 123.417  1.00 30.00           C
ATOM   3604  C   TYR A 554      89.433 124.061 122.582  1.00 30.00           C
ATOM   3605  O   TYR A 554      89.627 124.035 121.375  1.00 30.00           O
ATOM   3606  CB  TYR A 554      88.322 122.097 123.762  1.00 20.00           C
ATOM   3607  CG  TYR A 554      87.239 122.229 122.688  1.00 20.00           C
ATOM   3608  CD1 TYR A 554      87.453 121.700 121.396  1.00 20.00           C
ATOM   3609  CD2 TYR A 554      86.087 123.010 122.927  1.00 20.00           C
ATOM   3610  CE1 TYR A 554      86.576 122.019 120.339  1.00 20.00           C
ATOM   3611  CE2 TYR A 554      85.202 123.315 121.874  1.00 20.00           C
```

```
ATOM   3612  CZ  TYR A 554      85.462 122.849 120.571  1.00 20.00           C
ATOM   3613  OH  TYR A 554      84.645 123.221 119.541  1.00 20.00           O
ATOM   3614  H   TYR A 554      90.054 122.905 125.506  1.00  0.00           H
ATOM   3615  HA  TYR A 554      90.251 122.099 122.800  1.00  0.00           H
ATOM   3616  HB2 TYR A 554      88.481 121.046 124.000  1.00  0.00           H
ATOM   3617  HB3 TYR A 554      87.912 122.554 124.661  1.00  0.00           H
ATOM   3618  HD1 TYR A 554      88.331 121.104 121.194  1.00  0.00           H
ATOM   3619  HD2 TYR A 554      85.902 123.431 123.904  1.00  0.00           H
ATOM   3620  HE1 TYR A 554      86.783 121.645 119.348  1.00  0.00           H
ATOM   3621  HE2 TYR A 554      84.345 123.945 122.061  1.00  0.00           H
ATOM   3622  HH  TYR A 554      85.017 123.073 118.661  1.00  0.00           H
ATOM   3623  N   TYR A 555      89.118 125.178 123.243  1.00 30.00           N
ATOM   3624  CA  TYR A 555      88.829 126.437 122.568  1.00 30.00           C
ATOM   3625  C   TYR A 555      90.063 127.121 121.939  1.00 30.00           C
ATOM   3626  O   TYR A 555      89.888 128.162 121.316  1.00 30.00           O
ATOM   3627  CB  TYR A 555      88.056 127.351 123.530  1.00 20.00           C
ATOM   3628  CG  TYR A 555      86.693 126.806 123.939  1.00 20.00           C
ATOM   3629  CD1 TYR A 555      85.582 126.999 123.092  1.00 20.00           C
ATOM   3630  CD2 TYR A 555      86.522 126.117 125.159  1.00 20.00           C
ATOM   3631  CE1 TYR A 555      84.311 126.525 123.468  1.00 20.00           C
ATOM   3632  CE2 TYR A 555      85.253 125.634 125.531  1.00 20.00           C
ATOM   3633  CZ  TYR A 555      84.146 125.842 124.687  1.00 20.00           C
ATOM   3634  OH  TYR A 555      82.912 125.383 125.039  1.00 20.00           O
ATOM   3635  H   TYR A 555      89.046 125.174 124.252  1.00  0.00           H
ATOM   3636  HA  TYR A 555      88.160 126.208 121.735  1.00  0.00           H
ATOM   3637  HB2 TYR A 555      88.659 127.543 124.418  1.00  0.00           H
ATOM   3638  HB3 TYR A 555      87.897 128.327 123.066  1.00  0.00           H
ATOM   3639  HD1 TYR A 555      85.692 127.523 122.155  1.00  0.00           H
ATOM   3640  HD2 TYR A 555      87.359 125.958 125.820  1.00  0.00           H
ATOM   3641  HE1 TYR A 555      83.465 126.684 122.815  1.00  0.00           H
ATOM   3642  HE2 TYR A 555      85.139 125.100 126.463  1.00  0.00           H
ATOM   3643  HH  TYR A 555      82.911 124.877 125.857  1.00  0.00           H
ATOM   3644  N   THR A 556      91.265 126.534 122.069  1.00 30.00           N
ATOM   3645  CA  THR A 556      92.444 126.924 121.291  1.00 30.00           C
ATOM   3646  C   THR A 556      92.586 126.071 120.009  1.00 30.00           C
ATOM   3647  O   THR A 556      93.166 126.544 119.027  1.00 30.00           O
ATOM   3648  CB  THR A 556      93.750 126.802 122.127  1.00 20.00           C
ATOM   3649  CG2 THR A 556      93.700 127.567 123.457  1.00  0.00           C
ATOM   3650  OG1 THR A 556      94.180 125.474 122.380  1.00  0.00           O
ATOM   3651  H   THR A 556      91.333 125.659 122.570  1.00  0.00           H
ATOM   3652  HA  THR A 556      92.350 127.963 120.974  1.00  0.00           H
ATOM   3653  HB  THR A 556      94.547 127.252 121.533  1.00  0.00           H
ATOM   3654  HG1 THR A 556      93.472 124.968 122.815  1.00  0.00           H
ATOM   3655 HG21 THR A 556      94.655 127.506 123.980  1.00  0.00           H
ATOM   3656 HG22 THR A 556      93.487 128.623 123.288  1.00  0.00           H
ATOM   3657 HG23 THR A 556      92.930 127.179 124.124  1.00  0.00           H
ATOM   3658  N   ARG A 557      92.026 124.848 120.010  1.00 30.00           N
ATOM   3659  CA  ARG A 557      91.947 123.965 118.854  1.00 30.00           C
ATOM   3660  C   ARG A 557      90.963 124.606 117.859  1.00 30.00           C
ATOM   3661  O   ARG A 557      89.783 124.731 118.174  1.00 30.00           O
ATOM   3662  CB  ARG A 557      91.499 122.551 119.308  1.00 20.00           C
ATOM   3663  CG  ARG A 557      91.514 121.480 118.203  1.00 20.00           C
ATOM   3664  CD  ARG A 557      90.642 120.245 118.509  1.00 20.00           C
ATOM   3665  NE  ARG A 557      91.258 119.332 119.475  1.00 20.00           N
ATOM   3666  CZ  ARG A 557      92.003 118.254 119.190  1.00 20.00           C
ATOM   3667  NH1 ARG A 557      92.252 117.864 117.936  1.00 20.00           N
ATOM   3668  NH2 ARG A 557      92.504 117.533 120.192  1.00 20.00           N1+
ATOM   3669  H   ARG A 557      91.395 124.603 120.766  1.00  0.00           H
ATOM   3670  HA  ARG A 557      92.937 123.890 118.401  1.00  0.00           H
ATOM   3671  HB2 ARG A 557      92.087 122.208 120.159  1.00  0.00           H
ATOM   3672  HB3 ARG A 557      90.474 122.624 119.663  1.00  0.00           H
ATOM   3673  HG2 ARG A 557      91.201 121.893 117.253  1.00  0.00           H
ATOM   3674  HG3 ARG A 557      92.552 121.174 118.067  1.00  0.00           H
ATOM   3675  HD2 ARG A 557      89.771 120.599 119.061  1.00  0.00           H
ATOM   3676  HD3 ARG A 557      90.219 119.760 117.632  1.00  0.00           H
ATOM   3677  HE  ARG A 557      91.082 119.536 120.454  1.00  0.00           H
ATOM   3678 HH11 ARG A 557      92.823 117.036 117.793  1.00  0.00           H
ATOM   3679 HH12 ARG A 557      91.843 118.334 117.135  1.00  0.00           H
ATOM   3680 HH21 ARG A 557      93.108 116.739 119.972  1.00  0.00           H
ATOM   3681 HH22 ARG A 557      92.288 117.753 121.155  1.00  0.00           H
ATOM   3682  N   GLY A 558      91.468 125.029 116.697  1.00 30.00           N
```

```
ATOM   3683  CA  GLY A 558      90.676 125.779 115.720  1.00 30.00           C
ATOM   3684  C   GLY A 558      91.183 127.224 115.564  1.00 30.00           C
ATOM   3685  O   GLY A 558      90.639 127.949 114.730  1.00 30.00           O
ATOM   3686  H   GLY A 558      92.420 124.811 116.442  1.00  0.00           H
ATOM   3687  HA2 GLY A 558      90.762 125.280 114.754  1.00  0.00           H
ATOM   3688  HA3 GLY A 558      89.613 125.793 115.968  1.00  0.00           H
ATOM   3689  N   PHE A 559      92.236 127.631 116.301  1.00 30.00           N
ATOM   3690  CA  PHE A 559      93.036 128.833 116.038  1.00 30.00           C
ATOM   3691  C   PHE A 559      94.377 128.400 115.423  1.00 30.00           C
ATOM   3692  O   PHE A 559      94.876 127.323 115.753  1.00 30.00           O
ATOM   3693  CB  PHE A 559      93.291 129.613 117.347  1.00 20.00           C
ATOM   3694  CG  PHE A 559      92.072 130.080 118.128  1.00 20.00           C
ATOM   3695  CD1 PHE A 559      90.876 130.470 117.482  1.00 20.00           C
ATOM   3696  CD2 PHE A 559      92.192 130.297 119.517  1.00 20.00           C
ATOM   3697  CE1 PHE A 559      89.812 130.963 118.225  1.00 20.00           C
ATOM   3698  CE2 PHE A 559      91.119 130.795 120.243  1.00 20.00           C
ATOM   3699  CZ  PHE A 559      89.930 131.112 119.601  1.00 20.00           C
ATOM   3700  H   PHE A 559      92.614 127.009 117.003  1.00  0.00           H
ATOM   3701  HA  PHE A 559      92.534 129.487 115.323  1.00  0.00           H
ATOM   3702  HB2 PHE A 559      93.913 129.012 118.012  1.00  0.00           H
ATOM   3703  HB3 PHE A 559      93.880 130.503 117.121  1.00  0.00           H
ATOM   3704  HD1 PHE A 559      90.767 130.376 116.413  1.00  0.00           H
ATOM   3705  HD2 PHE A 559      93.116 130.064 120.025  1.00  0.00           H
ATOM   3706  HE1 PHE A 559      88.889 131.231 117.733  1.00  0.00           H
ATOM   3707  HE2 PHE A 559      91.207 130.928 121.311  1.00  0.00           H
ATOM   3708  HZ  PHE A 559      89.095 131.480 120.176  1.00  0.00           H
ATOM   3709  N   GLN A 560      94.928 129.239 114.529  1.00 30.00           N
ATOM   3710  CA  GLN A 560      96.131 128.944 113.745  1.00 30.00           C
ATOM   3711  C   GLN A 560      97.395 128.705 114.596  1.00 30.00           C
ATOM   3712  O   GLN A 560      98.039 127.669 114.426  1.00 30.00           O
ATOM   3713  CB  GLN A 560      96.325 130.042 112.667  1.00 20.00           C
ATOM   3714  CG  GLN A 560      97.603 129.954 111.799  1.00  0.00           C
ATOM   3715  CD  GLN A 560      97.636 128.740 110.866  1.00  0.00           C
ATOM   3716  NE2 GLN A 560      98.105 127.593 111.362  1.00  0.00           N
ATOM   3717  OE1 GLN A 560      97.257 128.843 109.703  1.00  0.00           O
ATOM   3718  H   GLN A 560      94.460 130.106 114.308  1.00  0.00           H
ATOM   3719  HA  GLN A 560      95.927 128.009 113.219  1.00  0.00           H
ATOM   3720  HB2 GLN A 560      95.456 130.041 112.005  1.00  0.00           H
ATOM   3721  HB3 GLN A 560      96.316 131.017 113.155  1.00  0.00           H
ATOM   3722  HG2 GLN A 560      97.662 130.848 111.177  1.00  0.00           H
ATOM   3723  HG3 GLN A 560      98.505 129.973 112.413  1.00  0.00           H
ATOM   3724 HE21 GLN A 560      98.152 126.770 110.782  1.00  0.00           H
ATOM   3725 HE22 GLN A 560      98.401 127.534 112.328  1.00  0.00           H
ATOM   3726  N   GLN A 561      97.729 129.667 115.473  1.00 30.00           N
ATOM   3727  CA  GLN A 561      98.961 129.645 116.264  1.00 30.00           C
ATOM   3728  C   GLN A 561      98.891 128.729 117.496  1.00 30.00           C
ATOM   3729  O   GLN A 561      99.840 127.984 117.731  1.00 30.00           O
ATOM   3730  CB  GLN A 561      99.358 131.075 116.678  1.00 20.00           C
ATOM   3731  CG  GLN A 561      99.799 131.960 115.494  1.00  0.00           C
ATOM   3732  CD  GLN A 561     100.318 133.337 115.925  1.00  0.00           C
ATOM   3733  NE2 GLN A 561     100.384 134.274 114.979  1.00  0.00           N
ATOM   3734  OE1 GLN A 561     100.677 133.555 117.081  1.00  0.00           O
ATOM   3735  H   GLN A 561      97.147 130.487 115.564  1.00  0.00           H
ATOM   3736  HA  GLN A 561      99.762 129.258 115.631  1.00  0.00           H
ATOM   3737  HB2 GLN A 561      98.535 131.554 117.211  1.00  0.00           H
ATOM   3738  HB3 GLN A 561     100.182 131.008 117.389  1.00  0.00           H
ATOM   3739  HG2 GLN A 561     100.595 131.461 114.939  1.00  0.00           H
ATOM   3740  HG3 GLN A 561      98.968 132.093 114.801  1.00  0.00           H
ATOM   3741 HE21 GLN A 561     100.723 135.198 115.207  1.00  0.00           H
ATOM   3742 HE22 GLN A 561     100.094 134.073 114.033  1.00  0.00           H
ATOM   3743  N   MET A 562      97.794 128.821 118.267  1.00 30.00           N
ATOM   3744  CA  MET A 562      97.638 128.123 119.548  1.00 30.00           C
ATOM   3745  C   MET A 562      96.969 126.740 119.440  1.00 30.00           C
ATOM   3746  O   MET A 562      96.945 126.022 120.441  1.00 30.00           O
ATOM   3747  CB  MET A 562      96.899 129.032 120.552  1.00 20.00           C
ATOM   3748  CG  MET A 562      97.709 130.277 120.947  1.00 20.00           C
ATOM   3749  SD  MET A 562      96.957 131.241 122.284  1.00 20.00           S
ATOM   3750  CE  MET A 562      98.309 132.397 122.623  1.00 20.00           C
ATOM   3751  H   MET A 562      97.050 129.451 118.006  1.00  0.00           H
ATOM   3752  HA  MET A 562      98.627 127.947 119.967  1.00  0.00           H
ATOM   3753  HB2 MET A 562      95.932 129.333 120.143  1.00  0.00           H
```

```
ATOM   3754  HB3 MET A 562      96.680 128.475 121.464  1.00  0.00           H
ATOM   3755  HG2 MET A 562      98.704 129.974 121.273  1.00  0.00           H
ATOM   3756  HG3 MET A 562      97.848 130.931 120.086  1.00  0.00           H
ATOM   3757  HE1 MET A 562      98.025 133.088 123.417  1.00  0.00           H
ATOM   3758  HE2 MET A 562      99.196 131.852 122.942  1.00  0.00           H
ATOM   3759  HE3 MET A 562      98.554 132.974 121.731  1.00  0.00           H
ATOM   3760  N   GLY A 563      96.461 126.365 118.253  1.00 30.00           N
ATOM   3761  CA  GLY A 563      95.778 125.087 118.042  1.00 30.00           C
ATOM   3762  C   GLY A 563      96.743 123.907 117.872  1.00 30.00           C
ATOM   3763  O   GLY A 563      96.305 122.768 118.026  1.00 30.00           O
ATOM   3764  H   GLY A 563      96.509 126.995 117.464  1.00  0.00           H
ATOM   3765  HA2 GLY A 563      95.124 124.873 118.888  1.00  0.00           H
ATOM   3766  HA3 GLY A 563      95.142 125.153 117.160  1.00  0.00           H
ATOM   3767  N   ILE A 564      98.036 124.154 117.587  1.00 30.00           N
ATOM   3768  CA  ILE A 564      99.059 123.111 117.450  1.00 30.00           C
ATOM   3769  C   ILE A 564      99.367 122.388 118.776  1.00 30.00           C
ATOM   3770  O   ILE A 564      99.649 121.192 118.749  1.00 30.00           O
ATOM   3771  CB  ILE A 564     100.395 123.657 116.862  1.00 20.00           C
ATOM   3772  CG1 ILE A 564     101.178 124.645 117.767  1.00  0.00           C
ATOM   3773  CG2 ILE A 564     100.156 124.255 115.468  1.00  0.00           C
ATOM   3774  CD1 ILE A 564     102.555 125.048 117.218  1.00  0.00           C
ATOM   3775  H   ILE A 564      98.347 125.107 117.467  1.00  0.00           H
ATOM   3776  HA  ILE A 564      98.670 122.359 116.759  1.00  0.00           H
ATOM   3777  HB  ILE A 564     101.044 122.791 116.714  1.00  0.00           H
ATOM   3778 HG12 ILE A 564     100.579 125.539 117.925  1.00  0.00           H
ATOM   3779 HG13 ILE A 564     101.352 124.231 118.757  1.00  0.00           H
ATOM   3780 HG21 ILE A 564     101.100 124.506 114.986  1.00  0.00           H
ATOM   3781 HG22 ILE A 564      99.635 123.549 114.820  1.00  0.00           H
ATOM   3782 HG23 ILE A 564      99.561 125.168 115.516  1.00  0.00           H
ATOM   3783 HD11 ILE A 564     103.210 125.378 118.025  1.00  0.00           H
ATOM   3784 HD12 ILE A 564     103.047 124.215 116.715  1.00  0.00           H
ATOM   3785 HD13 ILE A 564     102.469 125.871 116.508  1.00  0.00           H
ATOM   3786  N   TYR A 565      99.270 123.105 119.905  1.00 30.00           N
ATOM   3787  CA  TYR A 565      99.560 122.567 121.234  1.00 30.00           C
ATOM   3788  C   TYR A 565      98.497 121.539 121.663  1.00 30.00           C
ATOM   3789  O   TYR A 565      98.848 120.496 122.216  1.00 30.00           O
ATOM   3790  CB  TYR A 565      99.717 123.709 122.258  1.00 20.00           C
ATOM   3791  CG  TYR A 565     100.768 124.752 121.891  1.00 20.00           C
ATOM   3792  CD1 TYR A 565     102.125 124.572 122.236  1.00 20.00           C
ATOM   3793  CD2 TYR A 565     100.383 125.921 121.206  1.00 20.00           C
ATOM   3794  CE1 TYR A 565     103.079 125.556 121.897  1.00 20.00           C
ATOM   3795  CE2 TYR A 565     101.336 126.890 120.842  1.00 20.00           C
ATOM   3796  CZ  TYR A 565     102.687 126.711 121.188  1.00 20.00           C
ATOM   3797  OH  TYR A 565     103.600 127.662 120.832  1.00 20.00           O
ATOM   3798  H   TYR A 565      99.007 124.079 119.855  1.00  0.00           H
ATOM   3799  HA  TYR A 565     100.520 122.057 121.189  1.00  0.00           H
ATOM   3800  HB2 TYR A 565      98.760 124.214 122.402  1.00  0.00           H
ATOM   3801  HB3 TYR A 565      99.974 123.285 123.230  1.00  0.00           H
ATOM   3802  HD1 TYR A 565     102.441 123.683 122.765  1.00  0.00           H
ATOM   3803  HD2 TYR A 565      99.349 126.071 120.954  1.00  0.00           H
ATOM   3804  HE1 TYR A 565     104.111 125.409 122.176  1.00  0.00           H
ATOM   3805  HE2 TYR A 565     101.032 127.769 120.294  1.00  0.00           H
ATOM   3806  HH  TYR A 565     104.483 127.478 121.168  1.00  0.00           H
ATOM   3807  N   ALA A 566      97.229 121.824 121.323  1.00 30.00           N
ATOM   3808  CA  ALA A 566      96.075 120.966 121.585  1.00 30.00           C
ATOM   3809  C   ALA A 566      96.143 119.621 120.846  1.00 30.00           C
ATOM   3810  O   ALA A 566      95.789 118.595 121.426  1.00 30.00           O
ATOM   3811  CB  ALA A 566      94.789 121.719 121.208  1.00 20.00           C
ATOM   3812  H   ALA A 566      97.042 122.682 120.824  1.00  0.00           H
ATOM   3813  HA  ALA A 566      96.055 120.749 122.654  1.00  0.00           H
ATOM   3814  HB1 ALA A 566      93.906 121.145 121.491  1.00  0.00           H
ATOM   3815  HB2 ALA A 566      94.724 122.683 121.715  1.00  0.00           H
ATOM   3816  HB3 ALA A 566      94.730 121.908 120.136  1.00  0.00           H
ATOM   3817  N   VAL A 567      96.604 119.640 119.589  1.00 30.00           N
ATOM   3818  CA  VAL A 567      96.713 118.424 118.788  1.00 30.00           C
ATOM   3819  C   VAL A 567      97.998 117.635 119.140  1.00 30.00           C
ATOM   3820  O   VAL A 567      97.955 116.405 119.157  1.00 30.00           O
ATOM   3821  CB  VAL A 567      96.675 118.713 117.257  1.00 20.00           C
ATOM   3822  CG1 VAL A 567      96.618 117.428 116.404  1.00 20.00           C
ATOM   3823  CG2 VAL A 567      95.465 119.584 116.867  1.00 20.00           C
ATOM   3824  H   VAL A 567      96.868 120.516 119.158  1.00  0.00           H
```

```
ATOM   3825  HA  VAL A 567      95.864 117.775 119.018  1.00  0.00           H
ATOM   3826  HB  VAL A 567      97.583 119.256 116.986  1.00  0.00           H
ATOM   3827 HG11 VAL A 567      96.634 117.657 115.338  1.00  0.00           H
ATOM   3828 HG12 VAL A 567      97.450 116.758 116.600  1.00  0.00           H
ATOM   3829 HG13 VAL A 567      95.706 116.866 116.607  1.00  0.00           H
ATOM   3830 HG21 VAL A 567      95.455 119.795 115.798  1.00  0.00           H
ATOM   3831 HG22 VAL A 567      94.528 119.087 117.113  1.00  0.00           H
ATOM   3832 HG23 VAL A 567      95.463 120.542 117.380  1.00  0.00           H
ATOM   3833  N   MET A 568      99.119 118.315 119.441  1.00 30.00           N
ATOM   3834  CA  MET A 568     100.368 117.613 119.762  1.00 30.00           C
ATOM   3835  C   MET A 568     100.310 116.870 121.111  1.00 30.00           C
ATOM   3836  O   MET A 568     100.792 115.739 121.162  1.00 30.00           O
ATOM   3837  CB  MET A 568     101.614 118.505 119.551  1.00 20.00           C
ATOM   3838  CG  MET A 568     102.062 119.402 120.712  1.00 20.00           C
ATOM   3839  SD  MET A 568     103.543 120.395 120.345  1.00 20.00           S
ATOM   3840  CE  MET A 568     102.944 121.587 119.121  1.00 20.00           C
ATOM   3841  H   MET A 568      99.141 119.329 119.403  1.00  0.00           H
ATOM   3842  HA  MET A 568     100.467 116.831 119.005  1.00  0.00           H
ATOM   3843  HB2 MET A 568     101.473 119.104 118.653  1.00  0.00           H
ATOM   3844  HB3 MET A 568     102.454 117.845 119.327  1.00  0.00           H
ATOM   3845  HG2 MET A 568     101.249 120.050 121.021  1.00  0.00           H
ATOM   3846  HG3 MET A 568     102.309 118.789 121.579  1.00  0.00           H
ATOM   3847  HE1 MET A 568     103.766 122.217 118.783  1.00  0.00           H
ATOM   3848  HE2 MET A 568     102.519 121.089 118.251  1.00  0.00           H
ATOM   3849  HE3 MET A 568     102.190 122.229 119.568  1.00  0.00           H
ATOM   3850  N   ILE A 569      99.666 117.445 122.146  1.00 30.00           N
ATOM   3851  CA  ILE A 569      99.489 116.750 123.430  1.00 30.00           C
ATOM   3852  C   ILE A 569      98.623 115.474 123.295  1.00 30.00           C
ATOM   3853  O   ILE A 569      98.998 114.439 123.847  1.00 30.00           O
ATOM   3854  CB  ILE A 569      98.940 117.665 124.570  1.00 20.00           C
ATOM   3855  CG1 ILE A 569      99.080 116.991 125.959  1.00 20.00           C
ATOM   3856  CG2 ILE A 569      97.500 118.175 124.356  1.00 20.00           C
ATOM   3857  CD1 ILE A 569      98.825 117.923 127.150  1.00 20.00           C
ATOM   3858  H   ILE A 569      99.270 118.375 122.059  1.00  0.00           H
ATOM   3859  HA  ILE A 569     100.484 116.419 123.735  1.00  0.00           H
ATOM   3860  HB  ILE A 569      99.573 118.552 124.576  1.00  0.00           H
ATOM   3861 HG12 ILE A 569      98.389 116.149 126.034  1.00  0.00           H
ATOM   3862 HG13 ILE A 569     100.077 116.562 126.061  1.00  0.00           H
ATOM   3863 HG21 ILE A 569      97.233 118.932 125.092  1.00  0.00           H
ATOM   3864 HG22 ILE A 569      97.395 118.637 123.379  1.00  0.00           H
ATOM   3865 HG23 ILE A 569      96.761 117.376 124.432  1.00  0.00           H
ATOM   3866 HD11 ILE A 569      99.239 117.502 128.066  1.00  0.00           H
ATOM   3867 HD12 ILE A 569      99.270 118.907 126.998  1.00  0.00           H
ATOM   3868 HD13 ILE A 569      97.756 118.054 127.315  1.00  0.00           H
ATOM   3869  N   GLU A 570      97.558 115.549 122.478  1.00  0.00           N
ATOM   3870  CA  GLU A 570      96.708 114.425 122.081  1.00  0.00           C
ATOM   3871  C   GLU A 570      97.533 113.254 121.515  1.00  0.00           C
ATOM   3872  O   GLU A 570      97.462 112.143 122.040  1.00  0.00           O
ATOM   3873  CB  GLU A 570      95.613 114.937 121.105  1.00 20.00           C
ATOM   3874  CG  GLU A 570      94.871 113.865 120.267  1.00 20.00           C
ATOM   3875  CD  GLU A 570      93.957 114.422 119.176  1.00 20.00           C
ATOM   3876  OE1 GLU A 570      94.035 115.634 118.881  1.00 20.00           O
ATOM   3877  OE2 GLU A 570      93.222 113.600 118.594  1.00 20.00           O1-
ATOM   3878  H   GLU A 570      97.331 116.440 122.061  1.00  0.00           H
ATOM   3879  HA  GLU A 570      96.212 114.051 122.980  1.00  0.00           H
ATOM   3880  HB2 GLU A 570      94.869 115.483 121.687  1.00  0.00           H
ATOM   3881  HB3 GLU A 570      96.038 115.681 120.440  1.00  0.00           H
ATOM   3882  HG2 GLU A 570      95.568 113.213 119.752  1.00  0.00           H
ATOM   3883  HG3 GLU A 570      94.291 113.220 120.925  1.00  0.00           H
ATOM   3884  N   LYS A 571      98.332 113.522 120.471  1.00  0.00           N
ATOM   3885  CA  LYS A 571      99.134 112.493 119.805  1.00  0.00           C
ATOM   3886  C   LYS A 571     100.280 111.947 120.682  1.00  0.00           C
ATOM   3887  O   LYS A 571     100.589 110.756 120.583  1.00  0.00           O
ATOM   3888  CB  LYS A 571      99.621 112.978 118.422  1.00 20.00           C
ATOM   3889  CG  LYS A 571      98.512 113.445 117.446  1.00 20.00           C
ATOM   3890  CD  LYS A 571      97.293 112.512 117.291  1.00 20.00           C
ATOM   3891  CE  LYS A 571      96.180 113.131 116.424  1.00 20.00           C
ATOM   3892  NZ  LYS A 571      94.935 112.343 116.477  1.00 20.00           N1+
ATOM   3893  H   LYS A 571      98.363 114.469 120.106  1.00  0.00           H
ATOM   3894  HA  LYS A 571      98.485 111.635 119.635  1.00  0.00           H
ATOM   3895  HB2 LYS A 571     100.334 113.794 118.556  1.00  0.00           H
```

```
ATOM   3896  HB3 LYS A 571     100.185 112.171 117.952  1.00  0.00           H
ATOM   3897  HG2 LYS A 571      98.158 114.423 117.762  1.00  0.00           H
ATOM   3898  HG3 LYS A 571      98.960 113.611 116.466  1.00  0.00           H
ATOM   3899  HD2 LYS A 571      97.612 111.565 116.854  1.00  0.00           H
ATOM   3900  HD3 LYS A 571      96.874 112.272 118.265  1.00  0.00           H
ATOM   3901  HE2 LYS A 571      95.946 114.141 116.763  1.00  0.00           H
ATOM   3902  HE3 LYS A 571      96.511 113.208 115.388  1.00  0.00           H
ATOM   3903  HZ1 LYS A 571      94.271 112.715 115.815  1.00  0.00           H
ATOM   3904  HZ2 LYS A 571      95.118 111.375 116.257  1.00  0.00           H
ATOM   3905  HZ3 LYS A 571      94.522 112.418 117.399  1.00  0.00           H
ATOM   3906  N   MET A 572     100.842 112.789 121.561  1.00  0.00           N
ATOM   3907  CA  MET A 572     101.828 112.385 122.567  1.00  0.00           C
ATOM   3908  C   MET A 572     101.247 111.453 123.644  1.00  0.00           C
ATOM   3909  O   MET A 572     101.989 110.615 124.151  1.00  0.00           O
ATOM   3910  CB  MET A 572     102.471 113.628 123.208  1.00 20.00           C
ATOM   3911  CG  MET A 572     103.499 114.313 122.295  1.00 20.00           C
ATOM   3912  SD  MET A 572     104.101 115.911 122.901  1.00 20.00           S
ATOM   3913  CE  MET A 572     104.822 115.416 124.487  1.00 20.00           C
ATOM   3914  H   MET A 572     100.523 113.747 121.608  1.00  0.00           H
ATOM   3915  HA  MET A 572     102.611 111.820 122.063  1.00  0.00           H
ATOM   3916  HB2 MET A 572     101.701 114.340 123.510  1.00  0.00           H
ATOM   3917  HB3 MET A 572     102.980 113.334 124.127  1.00  0.00           H
ATOM   3918  HG2 MET A 572     104.359 113.658 122.151  1.00  0.00           H
ATOM   3919  HG3 MET A 572     103.082 114.471 121.301  1.00  0.00           H
ATOM   3920  HE1 MET A 572     105.704 116.010 124.719  1.00  0.00           H
ATOM   3921  HE2 MET A 572     104.088 115.561 125.277  1.00  0.00           H
ATOM   3922  HE3 MET A 572     105.119 114.370 124.471  1.00  0.00           H
ATOM   3923  N   ILE A 573      99.956 111.579 123.958  1.00  0.00           N
ATOM   3924  CA  ILE A 573      99.255 110.630 124.814  1.00  0.00           C
ATOM   3925  C   ILE A 573      98.895 109.362 124.011  1.00  0.00           C
ATOM   3926  O   ILE A 573      99.322 108.265 124.370  1.00  0.00           O
ATOM   3927  CB  ILE A 573      98.004 111.284 125.475  1.00 20.00           C
ATOM   3928  CG1 ILE A 573      98.469 112.337 126.508  1.00  0.00           C
ATOM   3929  CG2 ILE A 573      97.009 110.299 126.127  1.00  0.00           C
ATOM   3930  CD1 ILE A 573      97.416 113.389 126.865  1.00  0.00           C
ATOM   3931  H   ILE A 573      99.411 112.348 123.581  1.00  0.00           H
ATOM   3932  HA  ILE A 573      99.921 110.328 125.622  1.00  0.00           H
ATOM   3933  HB  ILE A 573      97.454 111.817 124.696  1.00  0.00           H
ATOM   3934 HG12 ILE A 573      98.826 111.847 127.414  1.00  0.00           H
ATOM   3935 HG13 ILE A 573      99.322 112.881 126.109  1.00  0.00           H
ATOM   3936 HG21 ILE A 573      96.177 110.829 126.592  1.00  0.00           H
ATOM   3937 HG22 ILE A 573      96.563 109.619 125.402  1.00  0.00           H
ATOM   3938 HG23 ILE A 573      97.492 109.699 126.899  1.00  0.00           H
ATOM   3939 HD11 ILE A 573      97.834 114.153 127.520  1.00  0.00           H
ATOM   3940 HD12 ILE A 573      97.060 113.897 125.968  1.00  0.00           H
ATOM   3941 HD13 ILE A 573      96.556 112.957 127.374  1.00  0.00           H
ATOM   3942  N   LEU A 574      98.152 109.557 122.914  1.00  0.00           N
ATOM   3943  CA  LEU A 574      97.522 108.527 122.087  1.00  0.00           C
ATOM   3944  C   LEU A 574      98.499 107.509 121.464  1.00  0.00           C
ATOM   3945  O   LEU A 574      98.165 106.329 121.379  1.00  0.00           O
ATOM   3946  CB  LEU A 574      96.696 109.263 121.005  1.00 20.00           C
ATOM   3947  CG  LEU A 574      95.854 108.408 120.034  1.00 20.00           C
ATOM   3948  CD1 LEU A 574      94.711 107.656 120.743  1.00 20.00           C
ATOM   3949  CD2 LEU A 574      95.323 109.288 118.890  1.00 20.00           C
ATOM   3950  H   LEU A 574      97.891 110.517 122.689  1.00  0.00           H
ATOM   3951  HA  LEU A 574      96.842 107.969 122.733  1.00  0.00           H
ATOM   3952  HB2 LEU A 574      96.029 109.980 121.488  1.00  0.00           H
ATOM   3953  HB3 LEU A 574      97.393 109.860 120.419  1.00  0.00           H
ATOM   3954  HG  LEU A 574      96.505 107.667 119.569  1.00  0.00           H
ATOM   3955 HD11 LEU A 574      93.753 107.808 120.246  1.00  0.00           H
ATOM   3956 HD12 LEU A 574      94.902 106.582 120.754  1.00  0.00           H
ATOM   3957 HD13 LEU A 574      94.587 107.975 121.779  1.00  0.00           H
ATOM   3958 HD21 LEU A 574      94.400 109.796 119.173  1.00  0.00           H
ATOM   3959 HD22 LEU A 574      96.034 110.064 118.608  1.00  0.00           H
ATOM   3960 HD23 LEU A 574      95.129 108.692 117.998  1.00  0.00           H
ATOM   3961  N   ARG A 575      99.661 107.982 121.000  1.00  0.00           N
ATOM   3962  CA  ARG A 575     100.622 107.200 120.221  1.00  0.00           C
ATOM   3963  C   ARG A 575     101.849 106.859 121.067  1.00  0.00           C
ATOM   3964  O   ARG A 575     102.154 105.678 121.231  1.00  0.00           O
ATOM   3965  CB  ARG A 575     101.025 107.943 118.928  1.00 20.00           C
ATOM   3966  CG  ARG A 575      99.845 108.272 117.999  1.00 20.00           C
```

```
ATOM   3967  CD  ARG A 575     100.299 108.974 116.712  1.00 20.00           C
ATOM   3968  NE  ARG A 575      99.217 109.041 115.720  1.00 20.00           N
ATOM   3969  CZ  ARG A 575      99.234 109.766 114.588  1.00 20.00           C
ATOM   3970  NH1 ARG A 575     100.285 110.534 114.267  1.00 20.00           N
ATOM   3971  NH2 ARG A 575      98.179 109.719 113.764  1.00 20.00           N1+
ATOM   3972  H   ARG A 575      99.863 108.967 121.114  1.00  0.00           H
ATOM   3973  HA  ARG A 575     100.177 106.253 119.911  1.00  0.00           H
ATOM   3974  HB2 ARG A 575     101.574 108.857 119.158  1.00  0.00           H
ATOM   3975  HB3 ARG A 575     101.727 107.318 118.379  1.00  0.00           H
ATOM   3976  HG2 ARG A 575      99.237 107.395 117.776  1.00  0.00           H
ATOM   3977  HG3 ARG A 575      99.199 108.964 118.542  1.00  0.00           H
ATOM   3978  HD2 ARG A 575     100.493 110.016 116.969  1.00  0.00           H
ATOM   3979  HD3 ARG A 575     101.231 108.575 116.310  1.00  0.00           H
ATOM   3980  HE  ARG A 575      98.418 108.451 115.900  1.00  0.00           H
ATOM   3981 HH11 ARG A 575     100.289 111.073 113.412  1.00  0.00           H
ATOM   3982 HH12 ARG A 575     101.085 110.575 114.881  1.00  0.00           H
ATOM   3983 HH21 ARG A 575      98.177 110.248 112.904  1.00  0.00           H
ATOM   3984 HH22 ARG A 575      97.382 109.138 113.983  1.00  0.00           H
ATOM   3985  N   ASP A 576     102.533 107.900 121.568  1.00 30.00           N
ATOM   3986  CA  ASP A 576     103.819 107.787 122.257  1.00 30.00           C
ATOM   3987  C   ASP A 576     103.664 107.172 123.653  1.00 30.00           C
ATOM   3988  O   ASP A 576     104.119 106.049 123.853  1.00 30.00           O
ATOM   3989  CB  ASP A 576     104.633 109.106 122.276  1.00 20.00           C
ATOM   3990  CG  ASP A 576     104.723 109.799 120.911  1.00 20.00           C
ATOM   3991  OD1 ASP A 576     104.809 109.075 119.894  1.00 20.00           O
ATOM   3992  OD2 ASP A 576     104.766 111.047 120.909  1.00 20.00           O1-
ATOM   3993  H   ASP A 576     102.213 108.840 121.385  1.00  0.00           H
ATOM   3994  HA  ASP A 576     104.418 107.078 121.680  1.00  0.00           H
ATOM   3995  HB2 ASP A 576     104.175 109.806 122.974  1.00  0.00           H
ATOM   3996  HB3 ASP A 576     105.647 108.923 122.633  1.00  0.00           H
ATOM   3997  N   LEU A 577     102.991 107.886 124.562  1.00  0.00           N
ATOM   3998  CA  LEU A 577     102.850 107.483 125.960  1.00  0.00           C
ATOM   3999  C   LEU A 577     102.023 106.195 126.140  1.00  0.00           C
ATOM   4000  O   LEU A 577     102.395 105.359 126.960  1.00  0.00           O
ATOM   4001  CB  LEU A 577     102.284 108.673 126.769  1.00 20.00           C
ATOM   4002  CG  LEU A 577     102.470 108.613 128.302  1.00 20.00           C
ATOM   4003  CD1 LEU A 577     103.958 108.544 128.712  1.00 20.00           C
ATOM   4004  CD2 LEU A 577     101.734 109.789 128.985  1.00 20.00           C
ATOM   4005  H   LEU A 577     102.611 108.796 124.312  1.00  0.00           H
ATOM   4006  HA  LEU A 577     103.856 107.260 126.316  1.00  0.00           H
ATOM   4007  HB2 LEU A 577     102.743 109.599 126.424  1.00  0.00           H
ATOM   4008  HB3 LEU A 577     101.222 108.763 126.542  1.00  0.00           H
ATOM   4009  HG  LEU A 577     101.992 107.696 128.654  1.00  0.00           H
ATOM   4010 HD11 LEU A 577     104.234 109.302 129.445  1.00  0.00           H
ATOM   4011 HD12 LEU A 577     104.193 107.575 129.154  1.00  0.00           H
ATOM   4012 HD13 LEU A 577     104.628 108.684 127.866  1.00  0.00           H
ATOM   4013 HD21 LEU A 577     101.077 109.427 129.777  1.00  0.00           H
ATOM   4014 HD22 LEU A 577     102.411 110.514 129.438  1.00  0.00           H
ATOM   4015 HD23 LEU A 577     101.110 110.348 128.286  1.00  0.00           H
ATOM   4016  N   CYS A 578     100.960 106.020 125.341  1.00  0.00           N
ATOM   4017  CA  CYS A 578     100.041 104.876 125.417  1.00  0.00           C
ATOM   4018  C   CYS A 578     100.720 103.535 125.085  1.00  0.00           C
ATOM   4019  O   CYS A 578     100.539 102.583 125.841  1.00  0.00           O
ATOM   4020  CB  CYS A 578      98.767 105.094 124.573  1.00 20.00           C
ATOM   4021  SG  CYS A 578      97.585 103.712 124.654  1.00 20.00           S
ATOM   4022  H   CYS A 578     100.756 106.707 124.629  1.00  0.00           H
ATOM   4023  HA  CYS A 578      99.711 104.816 126.456  1.00  0.00           H
ATOM   4024  HB2 CYS A 578      98.234 105.979 124.915  1.00  0.00           H
ATOM   4025  HB3 CYS A 578      99.041 105.259 123.534  1.00  0.00           H
ATOM   4026  HG  CYS A 578      98.365 102.822 124.033  1.00  0.00           H
ATOM   4027  N   ARG A 579     101.492 103.472 123.986  1.00  0.00           N
ATOM   4028  CA  ARG A 579     102.249 102.266 123.632  1.00  0.00           C
ATOM   4029  C   ARG A 579     103.478 102.060 124.534  1.00  0.00           C
ATOM   4030  O   ARG A 579     103.841 100.912 124.792  1.00  0.00           O
ATOM   4031  CB  ARG A 579     102.657 102.282 122.146  1.00 20.00           C
ATOM   4032  CG  ARG A 579     101.465 102.300 121.170  1.00 20.00           C
ATOM   4033  CD  ARG A 579     101.873 102.014 119.715  1.00 20.00           C
ATOM   4034  NE  ARG A 579     102.668 103.098 119.123  1.00 20.00           N
ATOM   4035  CZ  ARG A 579     102.207 104.086 118.340  1.00 20.00           C
ATOM   4036  NH1 ARG A 579     103.068 105.000 117.875  1.00 20.00           N
ATOM   4037  NH2 ARG A 579     100.911 104.169 118.009  1.00 20.00           N1+
```

```
ATOM   4038  H   ARG A 579     101.617 104.284 123.396  1.00  0.00           H
ATOM   4039  HA  ARG A 579     101.597 101.402 123.776  1.00  0.00           H
ATOM   4040  HB2 ARG A 579     103.323 103.122 121.940  1.00  0.00           H
ATOM   4041  HB3 ARG A 579     103.240 101.379 121.955  1.00  0.00           H
ATOM   4042  HG2 ARG A 579     100.682 101.601 121.465  1.00  0.00           H
ATOM   4043  HG3 ARG A 579     101.015 103.292 121.227  1.00  0.00           H
ATOM   4044  HD2 ARG A 579     102.579 101.184 119.743  1.00  0.00           H
ATOM   4045  HD3 ARG A 579     101.052 101.660 119.091  1.00  0.00           H
ATOM   4046  HE  ARG A 579     103.645 103.110 119.381  1.00  0.00           H
ATOM   4047 HH11 ARG A 579     102.753 105.746 117.273  1.00  0.00           H
ATOM   4048 HH12 ARG A 579     104.045 104.957 118.129  1.00  0.00           H
ATOM   4049 HH21 ARG A 579     100.578 104.905 117.405  1.00  0.00           H
ATOM   4050 HH22 ARG A 579     100.260 103.485 118.366  1.00  0.00           H
ATOM   4051  N   PHE A 580     104.078 103.165 125.006  1.00  0.00           N
ATOM   4052  CA  PHE A 580     105.277 103.151 125.837  1.00  0.00           C
ATOM   4053  C   PHE A 580     105.013 102.770 127.305  1.00  0.00           C
ATOM   4054  O   PHE A 580     105.929 102.257 127.944  1.00  0.00           O
ATOM   4055  CB  PHE A 580     105.995 104.506 125.706  1.00 20.00           C
ATOM   4056  CG  PHE A 580     107.374 104.542 126.322  1.00 20.00           C
ATOM   4057  CD1 PHE A 580     108.455 103.977 125.614  1.00 20.00           C
ATOM   4058  CD2 PHE A 580     107.556 104.958 127.658  1.00 20.00           C
ATOM   4059  CE1 PHE A 580     109.697 103.882 126.217  1.00 20.00           C
ATOM   4060  CE2 PHE A 580     108.808 104.857 128.243  1.00 20.00           C
ATOM   4061  CZ  PHE A 580     109.864 104.319 127.520  1.00 20.00           C
ATOM   4062  H   PHE A 580     103.724 104.077 124.748  1.00  0.00           H
ATOM   4063  HA  PHE A 580     105.946 102.391 125.427  1.00  0.00           H
ATOM   4064  HB2 PHE A 580     106.130 104.734 124.648  1.00  0.00           H
ATOM   4065  HB3 PHE A 580     105.385 105.307 126.124  1.00  0.00           H
ATOM   4066  HD1 PHE A 580     108.318 103.609 124.608  1.00  0.00           H
ATOM   4067  HD2 PHE A 580     106.724 105.345 128.228  1.00  0.00           H
ATOM   4068  HE1 PHE A 580     110.532 103.457 125.681  1.00  0.00           H
ATOM   4069  HE2 PHE A 580     108.960 105.185 129.260  1.00  0.00           H
ATOM   4070  HZ  PHE A 580     110.833 104.236 127.964  1.00  0.00           H
ATOM   4071  N   MET A 581     103.785 102.971 127.819  1.00  0.00           N
ATOM   4072  CA  MET A 581     103.405 102.553 129.176  1.00  0.00           C
ATOM   4073  C   MET A 581     103.154 101.037 129.318  1.00  0.00           C
ATOM   4074  O   MET A 581     102.826 100.609 130.420  1.00  0.00           O
ATOM   4075  CB  MET A 581     102.241 103.406 129.727  1.00 20.00           C
ATOM   4076  CG  MET A 581     100.862 103.156 129.094  1.00 20.00           C
ATOM   4077  SD  MET A 581      99.476 103.918 129.976  1.00 20.00           S
ATOM   4078  CE  MET A 581      99.329 102.776 131.377  1.00 20.00           C
ATOM   4079  H   MET A 581     103.067 103.417 127.263  1.00  0.00           H
ATOM   4080  HA  MET A 581     104.256 102.768 129.825  1.00  0.00           H
ATOM   4081  HB2 MET A 581     102.493 104.462 129.621  1.00  0.00           H
ATOM   4082  HB3 MET A 581     102.174 103.243 130.802  1.00  0.00           H
ATOM   4083  HG2 MET A 581     100.864 103.568 128.092  1.00  0.00           H
ATOM   4084  HG3 MET A 581     100.648 102.093 128.991  1.00  0.00           H
ATOM   4085  HE1 MET A 581      98.486 103.062 132.006  1.00  0.00           H
ATOM   4086  HE2 MET A 581      99.162 101.758 131.022  1.00  0.00           H
ATOM   4087  HE3 MET A 581     100.230 102.788 131.988  1.00  0.00           H
ATOM   4088  N   PHE A 582     103.335 100.255 128.240  1.00  0.00           N
ATOM   4089  CA  PHE A 582     103.455  98.796 128.298  1.00  0.00           C
ATOM   4090  C   PHE A 582     104.933  98.369 128.292  1.00  0.00           C
ATOM   4091  O   PHE A 582     105.265  97.372 128.933  1.00  0.00           O
ATOM   4092  CB  PHE A 582     102.689  98.150 127.128  1.00 20.00           C
ATOM   4093  CG  PHE A 582     101.189  98.370 127.184  1.00 20.00           C
ATOM   4094  CD1 PHE A 582     100.397  97.638 128.095  1.00 20.00           C
ATOM   4095  CD2 PHE A 582     100.597  99.420 126.452  1.00 20.00           C
ATOM   4096  CE1 PHE A 582      99.043  97.920 128.220  1.00 20.00           C
ATOM   4097  CE2 PHE A 582      99.241  99.684 126.589  1.00 20.00           C
ATOM   4098  CZ  PHE A 582      98.467  98.936 127.466  1.00 20.00           C
ATOM   4099  H   PHE A 582     103.542 100.683 127.350  1.00  0.00           H
ATOM   4100  HA  PHE A 582     103.021  98.418 129.225  1.00  0.00           H
ATOM   4101  HB2 PHE A 582     103.069  98.519 126.174  1.00  0.00           H
ATOM   4102  HB3 PHE A 582     102.868  97.074 127.128  1.00  0.00           H
ATOM   4103  HD1 PHE A 582     100.842  96.862 128.701  1.00  0.00           H
ATOM   4104  HD2 PHE A 582     101.199 100.020 125.786  1.00  0.00           H
ATOM   4105  HE1 PHE A 582      98.437  97.354 128.911  1.00  0.00           H
ATOM   4106  HE2 PHE A 582      98.789 100.483 126.019  1.00  0.00           H
ATOM   4107  HZ  PHE A 582      97.414  99.152 127.571  1.00  0.00           H
ATOM   4108  N   VAL A 583     105.791  99.145 127.601  1.00  0.00           N
```

```
ATOM   4109  CA  VAL A 583     107.242  98.962 127.558  1.00  0.00           C
ATOM   4110  C   VAL A 583     107.885  99.277 128.921  1.00  0.00           C
ATOM   4111  O   VAL A 583     108.605  98.429 129.438  1.00  0.00           O
ATOM   4112  CB  VAL A 583     107.914  99.837 126.455  1.00 20.00           C
ATOM   4113  CG1 VAL A 583     109.458  99.792 126.447  1.00 20.00           C
ATOM   4114  CG2 VAL A 583     107.383  99.472 125.057  1.00 20.00           C
ATOM   4115  H   VAL A 583     105.438  99.950 127.104  1.00  0.00           H
ATOM   4116  HA  VAL A 583     107.439  97.912 127.327  1.00  0.00           H
ATOM   4117  HB  VAL A 583     107.641 100.877 126.625  1.00  0.00           H
ATOM   4118 HG11 VAL A 583     109.862 100.343 125.598  1.00  0.00           H
ATOM   4119 HG12 VAL A 583     109.888 100.246 127.340  1.00  0.00           H
ATOM   4120 HG13 VAL A 583     109.824  98.767 126.382  1.00  0.00           H
ATOM   4121 HG21 VAL A 583     107.836 100.098 124.288  1.00  0.00           H
ATOM   4122 HG22 VAL A 583     107.600  98.432 124.811  1.00  0.00           H
ATOM   4123 HG23 VAL A 583     106.304  99.612 124.988  1.00  0.00           H
ATOM   4124  N   TYR A 584     107.586 100.459 129.489  1.00  0.00           N
ATOM   4125  CA  TYR A 584     108.064 100.891 130.804  1.00  0.00           C
ATOM   4126  C   TYR A 584     107.504 100.064 131.975  1.00  0.00           C
ATOM   4127  O   TYR A 584     108.248  99.815 132.922  1.00  0.00           O
ATOM   4128  CB  TYR A 584     107.831 102.413 130.987  1.00 20.00           C
ATOM   4129  CG  TYR A 584     107.952 102.909 132.422  1.00 20.00           C
ATOM   4130  CD1 TYR A 584     109.200 102.863 133.081  1.00 20.00           C
ATOM   4131  CD2 TYR A 584     106.800 103.307 133.135  1.00 20.00           C
ATOM   4132  CE1 TYR A 584     109.285 103.153 134.455  1.00 20.00           C
ATOM   4133  CE2 TYR A 584     106.890 103.615 134.506  1.00 20.00           C
ATOM   4134  CZ  TYR A 584     108.128 103.519 135.169  1.00 20.00           C
ATOM   4135  OH  TYR A 584     108.202 103.772 136.507  1.00 20.00           O
ATOM   4136  H   TYR A 584     106.986 101.108 128.995  1.00  0.00           H
ATOM   4137  HA  TYR A 584     109.142 100.731 130.812  1.00  0.00           H
ATOM   4138  HB2 TYR A 584     108.524 102.969 130.357  1.00  0.00           H
ATOM   4139  HB3 TYR A 584     106.831 102.669 130.631  1.00  0.00           H
ATOM   4140  HD1 TYR A 584     110.086 102.549 132.549  1.00  0.00           H
ATOM   4141  HD2 TYR A 584     105.838 103.338 132.645  1.00  0.00           H
ATOM   4142  HE1 TYR A 584     110.239 103.081 134.958  1.00  0.00           H
ATOM   4143  HE2 TYR A 584     106.003 103.897 135.054  1.00  0.00           H
ATOM   4144  HH  TYR A 584     109.083 103.638 136.863  1.00  0.00           H
ATOM   4145  N   LEU A 585     106.220  99.671 131.913  1.00  0.00           N
ATOM   4146  CA  LEU A 585     105.543  98.986 133.017  1.00  0.00           C
ATOM   4147  C   LEU A 585     106.096  97.578 133.314  1.00  0.00           C
ATOM   4148  O   LEU A 585     105.994  97.147 134.461  1.00  0.00           O
ATOM   4149  CB  LEU A 585     104.019  99.021 132.784  1.00 20.00           C
ATOM   4150  CG  LEU A 585     103.121  98.461 133.914  1.00 20.00           C
ATOM   4151  CD1 LEU A 585     103.358  99.174 135.264  1.00 20.00           C
ATOM   4152  CD2 LEU A 585     101.636  98.503 133.493  1.00 20.00           C
ATOM   4153  H   LEU A 585     105.659  99.896 131.104  1.00  0.00           H
ATOM   4154  HA  LEU A 585     105.743  99.588 133.903  1.00  0.00           H
ATOM   4155  HB2 LEU A 585     103.736 100.063 132.640  1.00  0.00           H
ATOM   4156  HB3 LEU A 585     103.794  98.500 131.852  1.00  0.00           H
ATOM   4157  HG  LEU A 585     103.365  97.406 134.051  1.00  0.00           H
ATOM   4158 HD11 LEU A 585     102.434  99.344 135.816  1.00  0.00           H
ATOM   4159 HD12 LEU A 585     104.004  98.576 135.908  1.00  0.00           H
ATOM   4160 HD13 LEU A 585     103.836 100.145 135.133  1.00  0.00           H
ATOM   4161 HD21 LEU A 585     101.175  97.521 133.603  1.00  0.00           H
ATOM   4162 HD22 LEU A 585     101.051  99.205 134.086  1.00  0.00           H
ATOM   4163 HD23 LEU A 585     101.514  98.799 132.450  1.00  0.00           H
ATOM   4164  N   VAL A 586     106.725  96.918 132.323  1.00  0.00           N
ATOM   4165  CA  VAL A 586     107.419  95.642 132.526  1.00  0.00           C
ATOM   4166  C   VAL A 586     108.773  95.807 133.261  1.00  0.00           C
ATOM   4167  O   VAL A 586     109.160  94.888 133.981  1.00  0.00           O
ATOM   4168  CB  VAL A 586     107.599  94.855 131.189  1.00 20.00           C
ATOM   4169  CG1 VAL A 586     108.906  95.117 130.411  1.00 20.00           C
ATOM   4170  CG2 VAL A 586     107.432  93.342 131.408  1.00 20.00           C
ATOM   4171  H   VAL A 586     106.783  97.324 131.400  1.00  0.00           H
ATOM   4172  HA  VAL A 586     106.786  95.039 133.181  1.00  0.00           H
ATOM   4173  HB  VAL A 586     106.788  95.164 130.526  1.00  0.00           H
ATOM   4174 HG11 VAL A 586     108.855  94.691 129.409  1.00  0.00           H
ATOM   4175 HG12 VAL A 586     109.101  96.180 130.303  1.00  0.00           H
ATOM   4176 HG13 VAL A 586     109.771  94.675 130.905  1.00  0.00           H
ATOM   4177 HG21 VAL A 586     107.519  92.795 130.469  1.00  0.00           H
ATOM   4178 HG22 VAL A 586     108.187  92.951 132.090  1.00  0.00           H
ATOM   4179 HG23 VAL A 586     106.452  93.110 131.828  1.00  0.00           H
```

```
ATOM   4180  N   PHE A 587     109.437  96.973 133.116  1.00  0.00           N
ATOM   4181  CA  PHE A 587     110.611  97.354 133.912  1.00  0.00           C
ATOM   4182  C   PHE A 587     110.230  97.781 135.337  1.00  0.00           C
ATOM   4183  O   PHE A 587     110.911  97.373 136.275  1.00  0.00           O
ATOM   4184  CB  PHE A 587     111.439  98.466 133.232  1.00 20.00           C
ATOM   4185  CG  PHE A 587     112.239  98.025 132.022  1.00 20.00           C
ATOM   4186  CD1 PHE A 587     113.494  97.399 132.180  1.00 20.00           C
ATOM   4187  CD2 PHE A 587     111.673  98.107 130.736  1.00 20.00           C
ATOM   4188  CE1 PHE A 587     114.180  96.937 131.063  1.00 20.00           C
ATOM   4189  CE2 PHE A 587     112.374  97.643 129.632  1.00 20.00           C
ATOM   4190  CZ  PHE A 587     113.626  97.066 129.796  1.00 20.00           C
ATOM   4191  H   PHE A 587     109.060  97.688 132.510  1.00  0.00           H
ATOM   4192  HA  PHE A 587     111.257  96.479 134.009  1.00  0.00           H
ATOM   4193  HB2 PHE A 587     110.795  99.295 132.937  1.00  0.00           H
ATOM   4194  HB3 PHE A 587     112.147  98.889 133.946  1.00  0.00           H
ATOM   4195  HD1 PHE A 587     113.927  97.285 133.163  1.00  0.00           H
ATOM   4196  HD2 PHE A 587     110.703  98.557 130.613  1.00  0.00           H
ATOM   4197  HE1 PHE A 587     115.145  96.468 131.177  1.00  0.00           H
ATOM   4198  HE2 PHE A 587     111.941  97.730 128.646  1.00  0.00           H
ATOM   4199  HZ  PHE A 587     114.167  96.704 128.934  1.00  0.00           H
ATOM   4200  N   LEU A 588     109.150  98.571 135.477  1.00  0.00           N
ATOM   4201  CA  LEU A 588     108.623  99.028 136.765  1.00  0.00           C
ATOM   4202  C   LEU A 588     108.168  97.855 137.643  1.00  0.00           C
ATOM   4203  O   LEU A 588     108.642  97.745 138.771  1.00  0.00           O
ATOM   4204  CB  LEU A 588     107.495 100.062 136.539  1.00 20.00           C
ATOM   4205  CG  LEU A 588     106.831 100.637 137.818  1.00 20.00           C
ATOM   4206  CD1 LEU A 588     107.847 101.304 138.768  1.00 20.00           C
ATOM   4207  CD2 LEU A 588     105.663 101.580 137.457  1.00 20.00           C
ATOM   4208  H   LEU A 588     108.654  98.885 134.653  1.00  0.00           H
ATOM   4209  HA  LEU A 588     109.447  99.525 137.280  1.00  0.00           H
ATOM   4210  HB2 LEU A 588     107.889 100.881 135.940  1.00  0.00           H
ATOM   4211  HB3 LEU A 588     106.719  99.597 135.932  1.00  0.00           H
ATOM   4212  HG  LEU A 588     106.380  99.808 138.366  1.00  0.00           H
ATOM   4213 HD11 LEU A 588     107.490 102.250 139.173  1.00  0.00           H
ATOM   4214 HD12 LEU A 588     108.063 100.655 139.617  1.00  0.00           H
ATOM   4215 HD13 LEU A 588     108.792 101.514 138.270  1.00  0.00           H
ATOM   4216 HD21 LEU A 588     105.838 102.609 137.770  1.00  0.00           H
ATOM   4217 HD22 LEU A 588     105.479 101.607 136.383  1.00  0.00           H
ATOM   4218 HD23 LEU A 588     104.739 101.250 137.933  1.00  0.00           H
ATOM   4219  N   PHE A 589     107.301  96.987 137.094  1.00  0.00           N
ATOM   4220  CA  PHE A 589     106.785  95.797 137.770  1.00  0.00           C
ATOM   4221  C   PHE A 589     107.866  94.718 137.984  1.00  0.00           C
ATOM   4222  O   PHE A 589     107.852  94.061 139.023  1.00  0.00           O
ATOM   4223  CB  PHE A 589     105.558  95.260 137.005  1.00 20.00           C
ATOM   4224  CG  PHE A 589     104.794  94.154 137.713  1.00 20.00           C
ATOM   4225  CD1 PHE A 589     103.903  94.476 138.759  1.00 20.00           C
ATOM   4226  CD2 PHE A 589     105.085  92.798 137.453  1.00 20.00           C
ATOM   4227  CE1 PHE A 589     103.276  93.464 139.473  1.00 20.00           C
ATOM   4228  CE2 PHE A 589     104.449  91.801 138.182  1.00 20.00           C
ATOM   4229  CZ  PHE A 589     103.544  92.133 139.183  1.00 20.00           C
ATOM   4230  H   PHE A 589     106.963  97.144 136.152  1.00  0.00           H
ATOM   4231  HA  PHE A 589     106.428  96.114 138.749  1.00  0.00           H
ATOM   4232  HB2 PHE A 589     104.857  96.080 136.842  1.00  0.00           H
ATOM   4233  HB3 PHE A 589     105.851  94.916 136.012  1.00  0.00           H
ATOM   4234  HD1 PHE A 589     103.705  95.508 139.007  1.00  0.00           H
ATOM   4235  HD2 PHE A 589     105.803  92.531 136.691  1.00  0.00           H
ATOM   4236  HE1 PHE A 589     102.581  93.713 140.260  1.00  0.00           H
ATOM   4237  HE2 PHE A 589     104.666  90.763 137.975  1.00  0.00           H
ATOM   4238  HZ  PHE A 589     103.057  91.351 139.747  1.00  0.00           H
ATOM   4239  N   GLY A 590     108.793  94.574 137.020  1.00  0.00           N
ATOM   4240  CA  GLY A 590     109.869  93.582 137.049  1.00  0.00           C
ATOM   4241  C   GLY A 590     110.883  93.880 138.161  1.00  0.00           C
ATOM   4242  O   GLY A 590     111.198  92.985 138.945  1.00  0.00           O
ATOM   4243  H   GLY A 590     108.741  95.156 136.195  1.00  0.00           H
ATOM   4244  HA2 GLY A 590     109.449  92.583 137.180  1.00  0.00           H
ATOM   4245  HA3 GLY A 590     110.386  93.589 136.089  1.00  0.00           H
ATOM   4246  N   PHE A 591     111.362  95.133 138.251  1.00  0.00           N
ATOM   4247  CA  PHE A 591     112.297  95.570 139.290  1.00  0.00           C
ATOM   4248  C   PHE A 591     111.650  95.811 140.664  1.00  0.00           C
ATOM   4249  O   PHE A 591     112.356  95.664 141.659  1.00  0.00           O
ATOM   4250  CB  PHE A 591     113.112  96.795 138.827  1.00 20.00           C
```

```
ATOM   4251  CG  PHE A 591     114.296  96.487 137.924  1.00 20.00           C
ATOM   4252  CD1 PHE A 591     115.595  96.404 138.472  1.00 20.00           C
ATOM   4253  CD2 PHE A 591     114.110  96.185 136.558  1.00 20.00           C
ATOM   4254  CE1 PHE A 591     116.677  96.111 137.654  1.00 20.00           C
ATOM   4255  CE2 PHE A 591     115.205  95.885 135.759  1.00 20.00           C
ATOM   4256  CZ  PHE A 591     116.483  95.858 136.303  1.00 20.00           C
ATOM   4257  H   PHE A 591     111.073  95.835 137.582  1.00  0.00           H
ATOM   4258  HA  PHE A 591     113.017  94.765 139.446  1.00  0.00           H
ATOM   4259  HB2 PHE A 591     112.463  97.514 138.329  1.00  0.00           H
ATOM   4260  HB3 PHE A 591     113.514  97.320 139.694  1.00  0.00           H
ATOM   4261  HD1 PHE A 591     115.759  96.595 139.523  1.00  0.00           H
ATOM   4262  HD2 PHE A 591     113.124  96.195 136.122  1.00  0.00           H
ATOM   4263  HE1 PHE A 591     117.674  96.081 138.069  1.00  0.00           H
ATOM   4264  HE2 PHE A 591     115.063  95.671 134.710  1.00  0.00           H
ATOM   4265  HZ  PHE A 591     117.331  95.630 135.674  1.00  0.00           H
ATOM   4266  N   SER A 592     110.349  96.156 140.726  1.00  0.00           N
ATOM   4267  CA  SER A 592     109.644  96.354 141.999  1.00  0.00           C
ATOM   4268  C   SER A 592     109.366  95.042 142.750  1.00  0.00           C
ATOM   4269  O   SER A 592     109.596  94.999 143.956  1.00  0.00           O
ATOM   4270  CB  SER A 592     108.381  97.220 141.826  1.00 20.00           C
ATOM   4271  OG  SER A 592     107.382  96.556 141.087  1.00 20.00           O
ATOM   4272  H   SER A 592     109.813  96.287 139.880  1.00  0.00           H
ATOM   4273  HA  SER A 592     110.320  96.931 142.629  1.00  0.00           H
ATOM   4274  HB2 SER A 592     108.622  98.172 141.354  1.00  0.00           H
ATOM   4275  HB3 SER A 592     107.957  97.462 142.801  1.00  0.00           H
ATOM   4276  HG  SER A 592     107.580  96.675 140.153  1.00  0.00           H
ATOM   4277  N   THR A 593     108.945  93.984 142.033  1.00 30.00           N
ATOM   4278  CA  THR A 593     108.808  92.635 142.595  1.00 30.00           C
ATOM   4279  C   THR A 593     110.173  92.004 142.955  1.00 30.00           C
ATOM   4280  O   THR A 593     110.218  91.210 143.895  1.00 30.00           O
ATOM   4281  CB  THR A 593     108.056  91.669 141.641  1.00 20.00           C
ATOM   4282  CG2 THR A 593     106.600  92.085 141.383  1.00 20.00           C
ATOM   4283  OG1 THR A 593     108.720  91.517 140.399  1.00 20.00           O
ATOM   4284  H   THR A 593     108.758  94.085 141.045  1.00  0.00           H
ATOM   4285  HA  THR A 593     108.230  92.715 143.519  1.00  0.00           H
ATOM   4286  HB  THR A 593     108.030  90.678 142.098  1.00  0.00           H
ATOM   4287  HG1 THR A 593     108.554  92.298 139.864  1.00  0.00           H
ATOM   4288 HG21 THR A 593     106.105  91.383 140.712  1.00  0.00           H
ATOM   4289 HG22 THR A 593     106.032  92.101 142.314  1.00  0.00           H
ATOM   4290 HG23 THR A 593     106.523  93.076 140.939  1.00  0.00           H
ATOM   4291  N   ALA A 594     111.261  92.403 142.268  1.00 30.00           N
ATOM   4292  CA  ALA A 594     112.629  92.019 142.617  1.00 30.00           C
ATOM   4293  C   ALA A 594     113.126  92.697 143.907  1.00 30.00           C
ATOM   4294  O   ALA A 594     113.727  92.014 144.732  1.00 30.00           O
ATOM   4295  CB  ALA A 594     113.574  92.312 141.442  1.00 30.00           C
ATOM   4296  H   ALA A 594     111.156  93.044 141.495  1.00  0.00           H
ATOM   4297  HA  ALA A 594     112.640  90.940 142.782  1.00  0.00           H
ATOM   4298  HB1 ALA A 594     114.582  91.951 141.651  1.00  0.00           H
ATOM   4299  HB2 ALA A 594     113.234  91.817 140.531  1.00  0.00           H
ATOM   4300  HB3 ALA A 594     113.647  93.378 141.235  1.00  0.00           H
ATOM   4301  N   VAL A 595     112.851  94.004 144.068  1.00 30.00           N
ATOM   4302  CA  VAL A 595     113.242  94.806 145.233  1.00 30.00           C
ATOM   4303  C   VAL A 595     112.507  94.407 146.529  1.00 30.00           C
ATOM   4304  O   VAL A 595     113.170  94.305 147.559  1.00 30.00           O
ATOM   4305  CB  VAL A 595     113.095  96.336 144.952  1.00 20.00           C
ATOM   4306  CG1 VAL A 595     112.949  97.255 146.187  1.00 20.00           C
ATOM   4307  CG2 VAL A 595     114.268  96.839 144.089  1.00 20.00           C
ATOM   4308  H   VAL A 595     112.363  94.501 143.334  1.00  0.00           H
ATOM   4309  HA  VAL A 595     114.302  94.605 145.407  1.00  0.00           H
ATOM   4310  HB  VAL A 595     112.192  96.474 144.357  1.00  0.00           H
ATOM   4311 HG11 VAL A 595     112.976  98.306 145.900  1.00  0.00           H
ATOM   4312 HG12 VAL A 595     111.999  97.099 146.700  1.00  0.00           H
ATOM   4313 HG13 VAL A 595     113.751  97.089 146.907  1.00  0.00           H
ATOM   4314 HG21 VAL A 595     114.114  97.871 143.775  1.00  0.00           H
ATOM   4315 HG22 VAL A 595     115.208  96.798 144.640  1.00  0.00           H
ATOM   4316 HG23 VAL A 595     114.394  96.239 143.188  1.00  0.00           H
ATOM   4317  N   VAL A 596     111.185  94.156 146.456  1.00 30.00           N
ATOM   4318  CA  VAL A 596     110.357  93.715 147.589  1.00 30.00           C
ATOM   4319  C   VAL A 596     110.851  92.384 148.190  1.00 30.00           C
ATOM   4320  O   VAL A 596     111.076  92.318 149.396  1.00 30.00           O
ATOM   4321  CB  VAL A 596     108.854  93.567 147.191  1.00 20.00           C
```

```
ATOM   4322  CG1 VAL A 596     107.964  92.808 148.204  1.00 20.00           C
ATOM   4323  CG2 VAL A 596     108.209  94.932 146.917  1.00 20.00           C
ATOM   4324  H   VAL A 596     110.704  94.272 145.573  1.00  0.00           H
ATOM   4325  HA  VAL A 596     110.436  94.479 148.366  1.00  0.00           H
ATOM   4326  HB  VAL A 596     108.819  93.012 146.251  1.00  0.00           H
ATOM   4327 HG11 VAL A 596     106.913  92.862 147.919  1.00  0.00           H
ATOM   4328 HG12 VAL A 596     108.214  91.748 148.267  1.00  0.00           H
ATOM   4329 HG13 VAL A 596     108.055  93.233 149.205  1.00  0.00           H
ATOM   4330 HG21 VAL A 596     107.251  94.802 146.418  1.00  0.00           H
ATOM   4331 HG22 VAL A 596     108.027  95.478 147.842  1.00  0.00           H
ATOM   4332 HG23 VAL A 596     108.829  95.561 146.283  1.00  0.00           H
ATOM   4333  N   THR A 597     111.041  91.373 147.326  1.00 30.00           N
ATOM   4334  CA  THR A 597     111.499  90.036 147.712  1.00 30.00           C
ATOM   4335  C   THR A 597     112.995  89.975 148.098  1.00 30.00           C
ATOM   4336  O   THR A 597     113.372  89.032 148.793  1.00 30.00           O
ATOM   4337  CB  THR A 597     111.241  89.002 146.583  1.00 20.00           C
ATOM   4338  CG2 THR A 597     109.753  88.873 146.219  1.00 20.00           C
ATOM   4339  OG1 THR A 597     111.985  89.287 145.411  1.00 20.00           O
ATOM   4340  H   THR A 597     110.848  91.512 146.345  1.00  0.00           H
ATOM   4341  HA  THR A 597     110.927  89.729 148.591  1.00  0.00           H
ATOM   4342  HB  THR A 597     111.575  88.022 146.926  1.00  0.00           H
ATOM   4343  HG1 THR A 597     111.543  89.994 144.931  1.00  0.00           H
ATOM   4344 HG21 THR A 597     109.607  88.146 145.419  1.00  0.00           H
ATOM   4345 HG22 THR A 597     109.175  88.533 147.080  1.00  0.00           H
ATOM   4346 HG23 THR A 597     109.322  89.818 145.893  1.00  0.00           H
ATOM   4347  N   LEU A 598     113.807  90.970 147.690  1.00 30.00           N
ATOM   4348  CA  LEU A 598     115.205  91.091 148.108  1.00 30.00           C
ATOM   4349  C   LEU A 598     115.325  91.645 149.538  1.00 30.00           C
ATOM   4350  O   LEU A 598     116.072  91.072 150.331  1.00 30.00           O
ATOM   4351  CB  LEU A 598     115.999  91.962 147.107  1.00 20.00           C
ATOM   4352  CG  LEU A 598     117.526  91.983 147.361  1.00 20.00           C
ATOM   4353  CD1 LEU A 598     118.180  90.620 147.057  1.00 20.00           C
ATOM   4354  CD2 LEU A 598     118.213  93.141 146.621  1.00 20.00           C
ATOM   4355  H   LEU A 598     113.442  91.710 147.107  1.00  0.00           H
ATOM   4356  HA  LEU A 598     115.643  90.091 148.100  1.00  0.00           H
ATOM   4357  HB2 LEU A 598     115.829  91.607 146.091  1.00  0.00           H
ATOM   4358  HB3 LEU A 598     115.603  92.978 147.138  1.00  0.00           H
ATOM   4359  HG  LEU A 598     117.698  92.191 148.416  1.00  0.00           H
ATOM   4360 HD11 LEU A 598     119.128  90.720 146.532  1.00  0.00           H
ATOM   4361 HD12 LEU A 598     118.383  90.078 147.981  1.00  0.00           H
ATOM   4362 HD13 LEU A 598     117.537  89.984 146.448  1.00  0.00           H
ATOM   4363 HD21 LEU A 598     118.910  93.643 147.288  1.00  0.00           H
ATOM   4364 HD22 LEU A 598     118.782  92.803 145.759  1.00  0.00           H
ATOM   4365 HD23 LEU A 598     117.504  93.891 146.269  1.00  0.00           H
ATOM   4366  N   ILE A 599     114.621  92.757 149.818  1.00 30.00           N
ATOM   4367  CA  ILE A 599     114.710  93.481 151.084  1.00 30.00           C
ATOM   4368  C   ILE A 599     113.295  93.738 151.643  1.00 30.00           C
ATOM   4369  O   ILE A 599     112.518  94.506 151.076  1.00 30.00           O
ATOM   4370  CB  ILE A 599     115.538  94.804 150.963  1.00 20.00           C
ATOM   4371  CG1 ILE A 599     114.936  95.952 150.114  1.00 20.00           C
ATOM   4372  CG2 ILE A 599     116.973  94.524 150.483  1.00 20.00           C
ATOM   4373  CD1 ILE A 599     114.527  97.166 150.951  1.00 20.00           C
ATOM   4374  H   ILE A 599     114.014  93.163 149.117  1.00  0.00           H
ATOM   4375  HA  ILE A 599     115.225  92.861 151.820  1.00  0.00           H
ATOM   4376  HB  ILE A 599     115.645  95.169 151.986  1.00  0.00           H
ATOM   4377 HG12 ILE A 599     114.090  95.614 149.522  1.00  0.00           H
ATOM   4378 HG13 ILE A 599     115.658  96.293 149.370  1.00  0.00           H
ATOM   4379 HG21 ILE A 599     117.620  95.388 150.637  1.00  0.00           H
ATOM   4380 HG22 ILE A 599     117.404  93.684 151.019  1.00  0.00           H
ATOM   4381 HG23 ILE A 599     116.999  94.278 149.422  1.00  0.00           H
ATOM   4382 HD11 ILE A 599     114.323  98.016 150.305  1.00  0.00           H
ATOM   4383 HD12 ILE A 599     113.637  96.962 151.545  1.00  0.00           H
ATOM   4384 HD13 ILE A 599     115.323  97.457 151.634  1.00  0.00           H
ATOM   4385  N   GLU A 600     112.987  93.048 152.753  1.00 30.00           N
ATOM   4386  CA  GLU A 600     111.724  93.150 153.482  1.00 30.00           C
ATOM   4387  C   GLU A 600     111.923  94.086 154.681  1.00 30.00           C
ATOM   4388  O   GLU A 600     112.228  93.632 155.785  1.00 30.00           O
ATOM   4389  CB  GLU A 600     111.257  91.735 153.902  1.00 20.00           C
ATOM   4390  CG  GLU A 600     110.770  90.838 152.740  1.00 20.00           C
ATOM   4391  CD  GLU A 600     109.448  91.266 152.087  1.00 20.00           C
ATOM   4392  OE1 GLU A 600     108.821  92.233 152.577  1.00 20.00           O
```

```
ATOM   4393  OE2 GLU A 600     109.071  90.590 151.104  1.00 20.00           O1-
ATOM   4394  H   GLU A 600     113.670  92.420 153.150  1.00  0.00           H
ATOM   4395  HA  GLU A 600     110.957  93.599 152.849  1.00  0.00           H
ATOM   4396  HB2 GLU A 600     112.075  91.222 154.409  1.00  0.00           H
ATOM   4397  HB3 GLU A 600     110.462  91.812 154.646  1.00  0.00           H
ATOM   4398  HG2 GLU A 600     111.536  90.777 151.966  1.00  0.00           H
ATOM   4399  HG3 GLU A 600     110.633  89.822 153.111  1.00  0.00           H
ATOM   4400  N   ASP A 601     111.773  95.389 154.409  1.00 30.00           N
ATOM   4401  CA  ASP A 601     111.939  96.483 155.364  1.00 30.00           C
ATOM   4402  C   ASP A 601     111.068  97.660 154.880  1.00 30.00           C
ATOM   4403  O   ASP A 601     110.566  97.630 153.753  1.00 30.00           O
ATOM   4404  CB  ASP A 601     113.440  96.870 155.503  1.00 20.00           C
ATOM   4405  CG  ASP A 601     113.816  97.654 156.769  1.00 20.00           C
ATOM   4406  OD1 ASP A 601     112.942  97.839 157.646  1.00 20.00           O
ATOM   4407  OD2 ASP A 601     115.002  98.040 156.848  1.00 20.00           O1-
ATOM   4408  H   ASP A 601     111.525  95.672 153.471  1.00  0.00           H
ATOM   4409  HA  ASP A 601     111.539  96.155 156.325  1.00  0.00           H
ATOM   4410  HB2 ASP A 601     114.046  95.964 155.496  1.00  0.00           H
ATOM   4411  HB3 ASP A 601     113.734  97.466 154.637  1.00  0.00           H
ATOM   4412  N   GLY A 602     110.908  98.682 155.740  1.00 30.00           N
ATOM   4413  CA  GLY A 602     110.155  99.917 155.488  1.00 30.00           C
ATOM   4414  C   GLY A 602     110.609 100.638 154.206  1.00 30.00           C
ATOM   4415  O   GLY A 602     111.750 100.480 153.771  1.00 30.00           O
ATOM   4416  H   GLY A 602     111.394  98.623 156.627  1.00  0.00           H
ATOM   4417  HA2 GLY A 602     109.093  99.677 155.428  1.00  0.00           H
ATOM   4418  HA3 GLY A 602     110.289 100.588 156.337  1.00  0.00           H
ATOM   4419  N   LYS A 603     109.680 101.405 153.604  1.00 30.00           N
ATOM   4420  CA  LYS A 603     109.765 102.090 152.301  1.00 30.00           C
ATOM   4421  C   LYS A 603     109.534 101.148 151.095  1.00 30.00           C
ATOM   4422  O   LYS A 603     109.055 101.619 150.063  1.00 30.00           O
ATOM   4423  CB  LYS A 603     111.066 102.935 152.183  1.00 20.00           C
ATOM   4424  CG  LYS A 603     111.179 103.856 150.957  1.00 20.00           C
ATOM   4425  CD  LYS A 603     112.484 104.670 150.984  1.00 20.00           C
ATOM   4426  CE  LYS A 603     112.625 105.606 149.778  1.00 20.00           C
ATOM   4427  NZ  LYS A 603     113.927 106.292 149.766  1.00 20.00           N1+
ATOM   4428  H   LYS A 603     108.779 101.485 154.053  1.00  0.00           H
ATOM   4429  HA  LYS A 603     108.928 102.789 152.296  1.00  0.00           H
ATOM   4430  HB2 LYS A 603     111.154 103.554 153.077  1.00  0.00           H
ATOM   4431  HB3 LYS A 603     111.943 102.289 152.174  1.00  0.00           H
ATOM   4432  HG2 LYS A 603     111.158 103.261 150.044  1.00  0.00           H
```
[truncated: 963,935 more chars]
